# The Alpha Variant (B.1.1.7) of SARS-CoV-2 Failed to Become Dominant in Mexico

**DOI:** 10.1128/spectrum.02240-21

**Published:** 2022-04-07

**Authors:** Selene Zárate, Blanca Taboada, José Esteban Muñoz-Medina, Pavel Iša, Alejandro Sanchez-Flores, Celia Boukadida, Alfredo Herrera-Estrella, Nelly Selem Mojica, Mauricio Rosales-Rivera, Bruno Gómez-Gil, Angel Gustavo Salas-Lais, Clara Esperanza Santacruz-Tinoco, Héctor Montoya-Fuentes, Julio Elias Alvarado-Yaah, Gloria María Molina-Salinas, Gloria Elena Espinoza-Ayala, José Antonio Enciso-Moreno, Rosa María Gutiérrez-Ríos, Antonio Loza, Joaquín Moreno-Contreras, Rodrigo García-López, Xaira Rivera-Gutierrez, Andreu Comas-García, Rosa María Wong-Chew, Maria-Eugenia Jiménez-Corona, Rosa María del Angel, Joel Armando Vazquez-Perez, Margarita Matías-Florentino, Marissa Pérez-García, Santiago Ávila-Ríos, Hugo G. Castelán-Sánchez, Luis Delaye, León P. Martínez-Castilla, Marina Escalera-Zamudio, Susana López, Carlos F. Arias

**Affiliations:** a Posgrado en Ciencias Genómicas, Universidad Autónoma de la Ciudad de México, Ciudad de México, México; b Departamento de Genética del Desarrollo y Fisiología Molecular, Instituto de Biotecnología, Universidad Nacional Autónoma de México, Cuernavaca, México; c Coordinación de Calidad de Insumos y Laboratorios Especializados, Instituto Mexicano del Seguro Social, Ciudad de México, México; d Unidad Universitaria de Secuenciación Masiva y Bioinformática, Instituto de Biotecnología, Universidad Nacional Autónoma de México, Cuernavaca, México; e Centro de Investigación en Enfermedades Infecciosas, Instituto Nacional de Enfermedades Respiratorias Ismael Cosío Villegas, Ciudad de México, México; f Laboratorio Nacional de Genómica para la Biodiversidad-Unidad de Genómica Avanzada, Centro de Investigación y de Estudios Avanzados del IPN, Irapuato, México; g Centro de Ciencias Matemáticas, Universidad Nacional Autónoma de México, Morelia, México; h Centro de Investigación en Ciencias, Instituto de Investigación en Ciencias Básicas y Aplicadas, Universidad Autónoma del Estado de Morelos, Cuernavaca, México; i Centro de Investigación en Alimentación y Desarrollo AC, Unidad Mazatlám, Mazatlán, México; j Laboratorio Central de Epidemiología, Instituto Mexicano del Seguro Social, Ciudad de México, México; k División de Laboratorios Especializados, Instituto Mexicano del Seguro Social, Ciudad de México, México; l Centro de Investigación Biomedica de Occidente, Instituto Mexicano del Seguro Social, Guadalajara, México; m Unidad de Investigación Médica de Yucatán, Instituto Mexicano del Seguro Social, Mérida, México; n Centro de Investigación Biomédica del Noreste, Instituto Mexicano del Seguro Social, Monterrey, México; o Unidad de Investigación Biomédica de Zacatecas, Instituto Mexicano del Seguro Social, Zacatecas, Zacatecas, México; p Departamento de Microbiología Molecular, Instituto de Biotecnología, Universidad Nacional Autónoma de México, Cuernavaca, México; q Facultad de Medicna y Centro de Investigación en Ciencias de la Salud y Biomedicina, Universidad Autónoma de San Luis Potosí, San Luis Potosí, México; r Facultad de Medicina, Laboratorio de Investigación en Enfermedades Infecciosas, División de Investigación, Universidad Nacional Autónoma de México, Ciudad de México, México; s Departamento de Epidemiología Instituto Nacional de Cardiología Ignacio Chávez, Ciudad de México, México; t Departamento de Infectómica y Patogénesis Molecular, Cinvestav, Ciudad de México, México; u Laboratorio de Biología Molecular de Enfermedades Emergentes y EPOC Instituto Nacional de Enfermedades Respiratorias Ismael Cosío Villegas, Ciudad de México, México; v Programa de Investigadoras e investigadores por México Consejo Nacional de Ciencia y Tecnología, Ciudad de México, México; w Departamento de Ingeniería Genética, Cinvestav Unidad Irapuato, Guanajuato, México; x Departamento de Ciencias Naturales, Universidad Autónoma Metropolitana, Unidad Cuajimalpa, Ciudad de México, México; y Department of Zoology, University of Oxford, Oxford, United Kingdom; Pontificia Universidad Católica de Chile

**Keywords:** Alpha, genomic surveillance, Mexico, SARS-CoV-2

## Abstract

During the coronavirus disease 2019 (COVID-19) pandemic, the emergence and rapid increase of the B.1.1.7 (Alpha) lineage of severe acute respiratory syndrome coronavirus 2 (SARS-CoV-2), first identified in the United Kingdom in September 2020, was well documented in different areas of the world and became a global public health concern because of its increased transmissibility. The B.1.1.7 lineage was first detected in Mexico during December 2020, showing a slow progressive increase in its circulation frequency, which reached its maximum in May 2021 but never became predominant. In this work, we analyzed the patterns of diversity and distribution of this lineage in Mexico using phylogenetic and haplotype network analyses. Despite the reported increase in transmissibility of the B.1.1.7 lineage, in most Mexican states, it did not displace cocirculating lineages, such as B.1.1.519, which dominated the country from February to May 2021. Our results show that the states with the highest prevalence of B.1.1.7 were those at the Mexico-U.S. border. An apparent pattern of dispersion of this lineage from the northern states of Mexico toward the center or the southeast was observed in the largest transmission chains, indicating possible independent introduction events from the United States. However, other entry points cannot be excluded, as shown by multiple introduction events. Local transmission led to a few successful haplotypes with a localized distribution and specific mutations indicating sustained community transmission.

**IMPORTANCE** The emergence and rapid increase of the B.1.1.7 (Alpha) lineage of severe acute respiratory syndrome coronavirus 2 (SARS-CoV-2) throughout the world were due to its increased transmissibility. However, it did not displace cocirculating lineages in most of Mexico, particularly B.1.1.519, which dominated the country from February to May 2021. In this work, we analyzed the distribution of B.1.1.7 in Mexico using phylogenetic and haplotype network analyses. Our results show that the states with the highest prevalence of B.1.1.7 (around 30%) were those at the Mexico-U.S. border, which also exhibited the highest lineage diversity, indicating possible introduction events from the United States. Also, several haplotypes were identified with a localized distribution and specific mutations, indicating that sustained community transmission occurred in the country.

## INTRODUCTION

The second wave of severe acute respiratory syndrome coronavirus 2 (SARS-CoV-2) infections was globally characterized by the emergence of numerous virus lineages displaying specific mutations across their genomes, resulting in their classification as variants of concern (VOCs) and variants of interest (VOIs) by the World Health Organization (WHO). These variants were associated with increased transmissibility or virulence, detrimental changes in epidemiology or clinical disease presentation, or decreased effectiveness of diagnostics, vaccines, or therapeutic methods.

In December 2020, the United Kingdom reported a new SARS-CoV-2 lineage classified as B.1.1.7 by the Pango lineages nomenclature system, and it was designated the Alpha variant by the WHO, the first defined VOC (1). Retrospective analyses showed that this VOC had been circulating as early as September 2020 in the United Kingdom (2). The B.1.1.7 lineage expanded rapidly across the United Kingdom to become predominant during early 2021, spreading to most European countries with similar success. By November 2021, local transmission of this lineage had been reported in 175 countries.

The B.1.1.7 lineage is defined by 18 amino acid changes and three deletions; seven of these changes are in the region of the spike protein (S) (3). Most notably, changes found in B.1.1.7 include the amino acid change N501Y located in the receptor-binding domain of S, which is thought to increase the binding affinity of the virus for its cell receptor angiotensin-converting enzyme 2 (ACE2) (4). N501Y is also a defining amino acid substitution of other VOCs, such as B.1.351 (5), first identified in South Africa, and P.1, first identified in Brazil (6). Moreover, a six-nucleotide deletion in the viral RNA of B.1.1.7 resulted in the loss of amino acids 69 to 70 of the S protein (ΔH69/V70), causing the failure to detect the S gene in a commonly used diagnostic test (7, 8). Additionally, in conjunction with the mutation D614G, ΔH69/V70 deletion might account for enhanced virus infectivity, as shown *in vitro* and supported by the observation that ΔH69/V70 and D614G cooccur in immunocompromised patients, in whom selection of the virus after immunotherapy could have occurred (9, 10). Finally, the P681H change has been shown to increase spike cleavage mediated by the host furin protease, as observed *in vitro*, impacting cell entry and thus viral infectivity (11).

In addition to B.1.1.7, three other VOCs (Beta, Gamma, and Delta) and some VOIs (carrying mutations predicted or known to play a role in the phenotype of the viruses) have been identified worldwide. Tracking these variants and their mutations through genomic surveillance has been critical to detect outbreaks across communities, routes of transmission over time, and, most importantly, to understand their impact on the clinical aspects of the disease. Epidemiological and genomic analyses at a global level have shown that the B.1.1.7 variant displayed a rapid increment in prevalence across many countries, attributable to an increase in its transmissibility estimated to be between 43% and 100% compared to other circulating lineages (1, 8, 12). Moreover, in some countries, the spread of the B.1.1.7 variant has been associated with higher rates of hospitalization and death (13–15).

In the United States, lineage B.1.1.7 was detected by the end of November 2020, and by January 2021, it had spread to 30 states, becoming the dominant variant in March 2021. In Mexico, the B.1.1.7 variant was first detected late in December 2020, and, as opposed to the United States, it did not become the predominant circulating lineage after its introduction. In contrast, in December 2020, lineage B.1.1.519 started to rise, establishing itself as the country’s dominant variant by February 2021 (16). Thus, this work aims to understand the dynamic dispersion of the B.1.1.7 lineage in Mexico using phylogenetic and haplotype network analyses, especially compared with the then-dominant lineage B.1.1.519. Regional differences were found, with higher prevalence in the northern states of the country that share a border with the United States, ranging from 20% to 40%. Moreover, evidence suggests multiple introductions to Mexico followed by some local transmission chains and spread, which resulted in the acquisition of different independent mutations clustered by geographical regions of the country.

## RESULTS

### Demographic and patient information of SARS-CoV-2.

**FIG 1:**
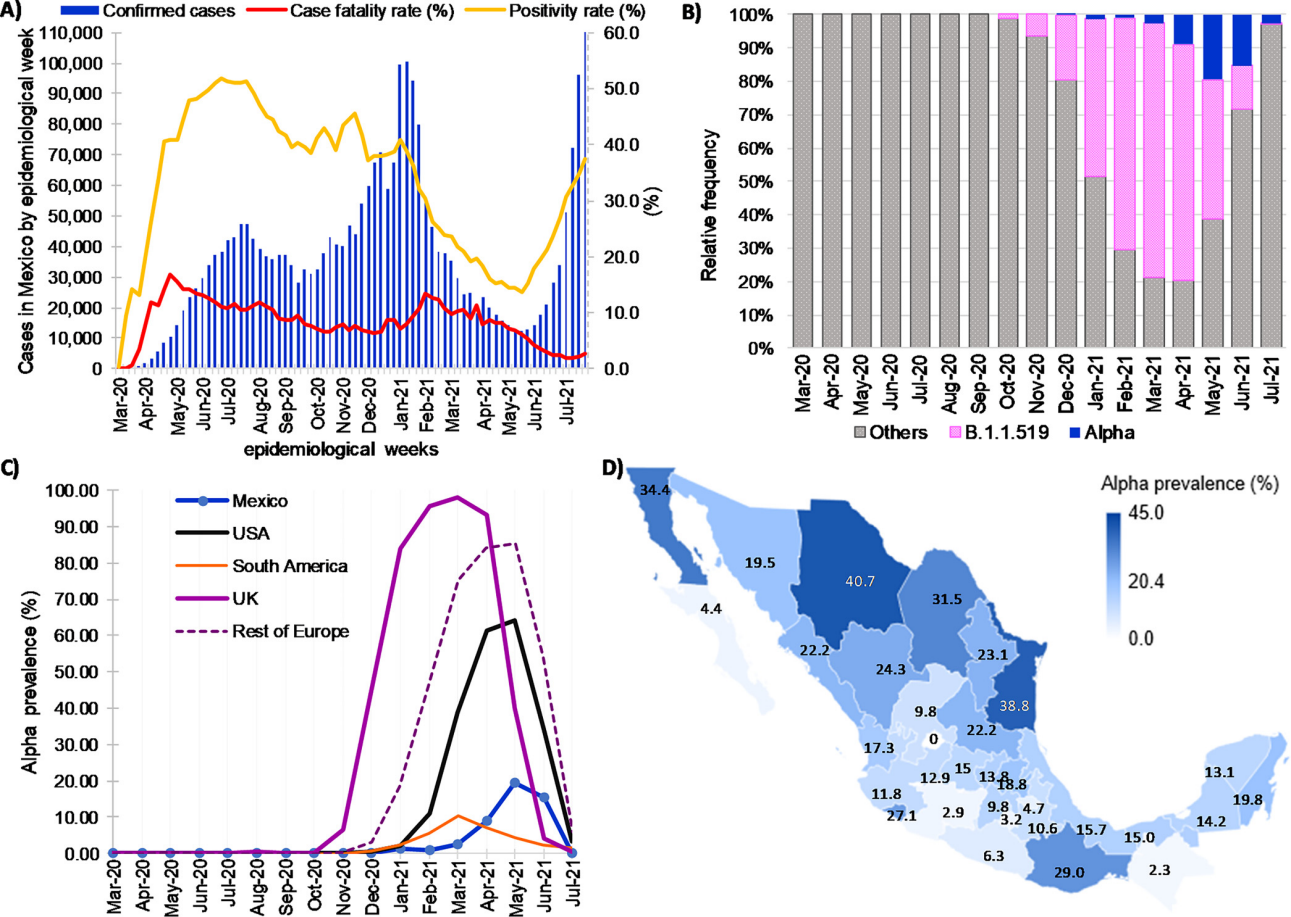
Distribution of SARS-CoV-2 cases and B.1.1.7 variant (Alpha) viruses identified in Mexico between 1 March 2020 and 7 July 2021. (A) Confirmed cases, positivity rate, and case fatality rate (proportion of the number of deaths among confirmed cases) in the country based on confirmation date. (B) Relative frequency of B.1.1.7 and other variants. (C) Prevalence of B.1.1.7 in Mexico and other global places through time estimated from whole-genome sequencing. (D) Prevalence at the state level, considering only sequences from April to June 2021 (months of higher prevalence).

As of the first week of July 2021, the epidemic in Mexico had presented three main waves, and there had been 2,546,017 positive cases of SARS-CoV-2 in Mexico since the first case was detected on February 27, 2020 (Fig. 1A). The first peak was reached at the end of July 2020, having around ∼50,000 new cases in epidemiological week 28 (W28; 40.01 cases per 100,000 inhabitants). The second peak was reached at the beginning of January of 2021, with ∼109,000 new cases in the first epidemiological week (W1) of the year (86.95 cases per 100,000 inhabitants). The third wave peaked by mid-August 2021, with ∼126,000 new cases in W32 (100.47 cases per 100,000 inhabitants).

To determine the genetic diversity and the epidemiological characteristics of the B.1.1.7 lineage in Mexico, 1,620 sequences were considered in the analysis (Tables S1 and S2 in the supplemental material). As shown in Fig. 1B, the B.1.1.7 lineage peaked from April to June 2021; therefore, the demographic characteristics of the patients were studied in this period (Table 1). For comparison, the same period was analyzed for the B.1.1.519 lineage, which was the most prevalent cocirculating lineage in Mexico during this time (Fig. 1B).

**TABLE 1:** Demographic and status information for Mexican patients during April to June 2021

Patient characteristics	All patients	Lineage		*P* value B.1.1.519 and B.1.1.7
		B.1.1.519	B.1.1.7	
Patient age median (IQR)				
April 2021	49 (35–60.75)	49 (35–60)	48 (30–59)	0.3877
May 2021	42 (30–54)	43 (31–54)	43 (30–54)	0.215
June 2021	37 (28–49)	41 (31–52)	39 (29–50)	0.01^*a*^
Total	42 (30–54)	47 (33–60)	41 (30–54)	2.2 × 10^−12^^*a*^
Gender counts (%)				
Female	5,130 (47.9%)	1,913 (47.4 %)	809 (49.8%)	0.132
Male	5,512 (51.5%)	2,101 (52.0%)	806 (49.7 %)	
Unknown	69 (0.6%)	25 (0.6%)	5 (0.4%)	
Patient status counts (%)				
Ambulatory	1,987 (18.5%)	744 (18.4%)	275 (16.4%)	0.017^*b*^
Hospitalized	4,470 (41.7%)	1,716 (42.5%)	739 (45.8%)	
Deceased	220 (2.1%)	88 (2.2%)	24 (1.5%)	
Unknown	4,034 (37.7%)	1,491 (36.9%)	582 (37.9%)	

a Wilcoxon sum of ranks test.

b Chi-square test.

Briefly, the average age of the patients with the B.1.1.7 variant was 42 years old (range of 0 to 93), while it was 47 years old in those infected with B.1.1.519, which was significantly different (Wilcoxon test, *P* = 2.2 × 10^−12^). Since the vaccination campaign for the population older than 60 years of age started in February 2021 and continued with younger groups in subsequent months, we observed an overall decrease in the median age of patients after May, in agreement with the age ranges of vaccination (Fig. S1). The nation-wide longitudinal data on confirmed cases show that major drops in the prevalence of the corresponding age group occurred after the vaccination campaign started for each group. In contrast, the 0 to 17 age group, which remained unvaccinated during this time frame, saw a continuous and linear increase in prevalence (Fig. S1). Although the difference between the median ages of B.1.1.7- and B.1.1.519-infected patients may be in part because of the variant itself, it is difficult to rule out factors such as vaccination, given that the B.1.1.519 prevalence started to decline in May when the older population completed their vaccination scheme, time during which the cases of B.1.1.7 started rising.

The proportion of females and males showed no statistical differences by lineage, and comparing the proportion of ambulatory, hospitalized, and deceased patients between lineages B.1.1.519 and B.1.1.7 resulted in a significant difference (chi-square test, *P* = 0.017). An increase in hospitalized patients was observed for B.1.1.7 during W20 to W24 (16 May to 19 June), corresponding to the prevalence peak of this lineage. However, patient status data correspond to the collection date and do not necessarily reflect the final clinical outcome.

### Geographical and temporal distribution of B.1.1.7.

B.1.1.7 was first identified in Mexico in a single sample collected on 31 December 2020, followed by a low frequency of 1% in January and February 2021 and a slight increase in March (2.5%) and April (8.9%) (Fig. 1C). The highest prevalence of B.1.1.7 was observed in May, reaching 18.8% at the national level but decreasing again in June (15.30%). This trend contrasts with the United Kingdom, the United States, and most of the European countries (Denmark, Finland, Italy, Spain, and Portugal among others), where, in general, after 3 to 5 months of community transmission, B.1.1.7 became the dominant variant, reaching more than 50% in the United States and 97.7% in the United Kingdom (Fig. 1C). Interestingly, as shown in Fig. 1C, the low prevalence of B.1.1.7 in Mexico was also observed in South American countries, such as Brazil, Chile, and Peru.

**FIG 2:**
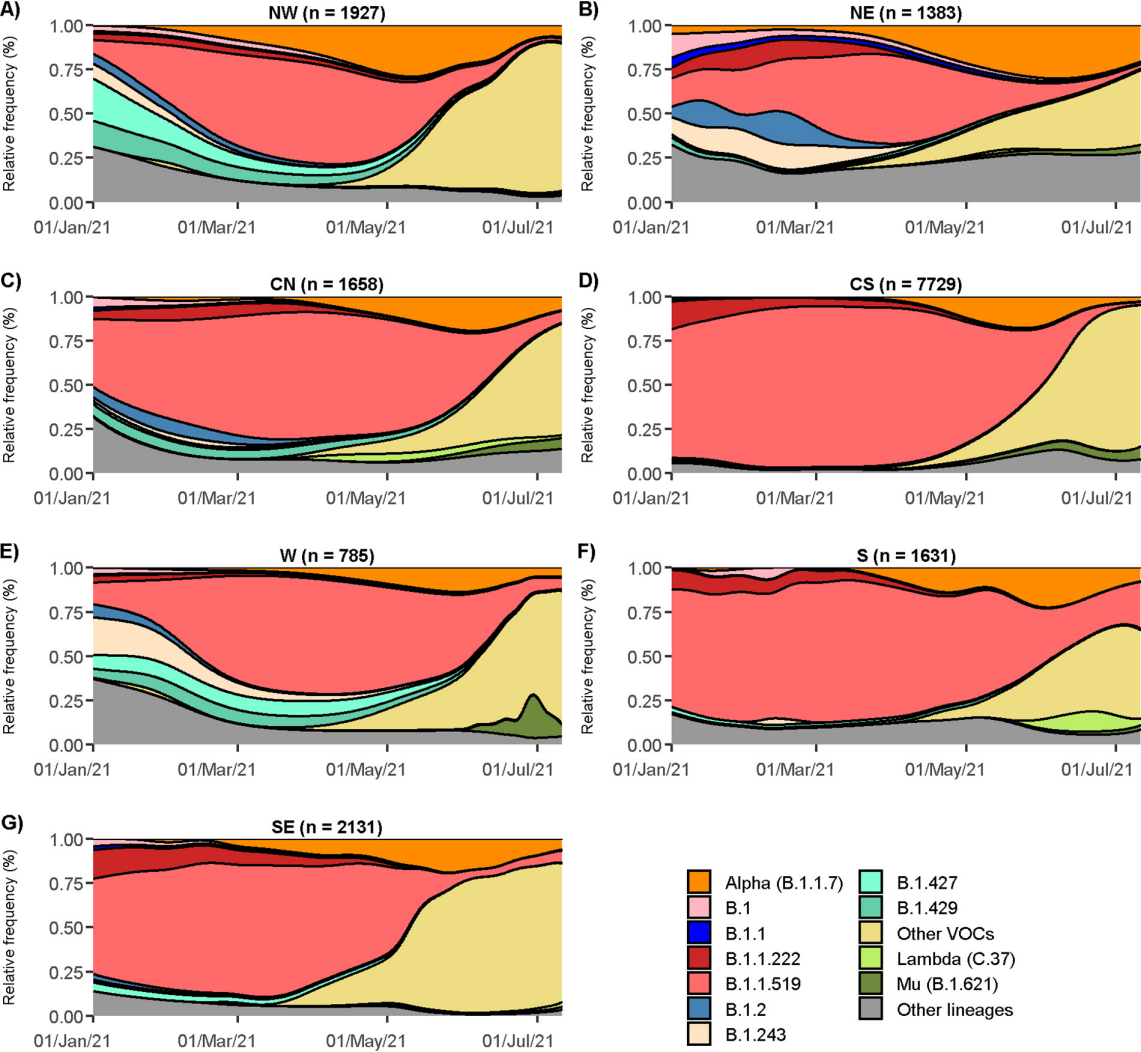
Temporal distribution of the B.1.1.7 variant and other lineages by region, including Northwest (A), Northeast (B), Central North (C), Central South (D), West (E), South (F), and Southeast (G).

The median number of B.1.1.7 sequences per state was 199.5 (interquartile range [IQR] = 66 to 342.5), being identified in 31 of the 32 Mexican states (Fig. 1D). Fig. 2 shows B.1.1.7 variant dispersion and prevalence through time in the seven regions (Fig. 1D) of Mexico defined in this study. Most of the low-prevalence states were in the Central South (CS), Central North (CN), and West (W) regions, while the Northwest (NW; Fig. 2A) and the Northeast (NE; Fig. 2B) regions, especially the states located in the Mexico-U.S. border (Baja California, Chihuahua, Coahuila, and Tamaulipas), had the highest prevalence, reaching its peak (29.5%) in May and June. Moreover, B.1.1.7 was identified as soon as January 2021 in the NE, suggesting that the north of Mexico may have been an entryway for this lineage. Interestingly, in the NW and NE, the B.1.1.519 lineage never achieved the dominant prevalence observed in other regions (Fig. 2C to G). Around June, B.1.1.7 and the other cocirculating lineages, including B.1.1.519, showed a decrease in their prevalence due to the entry of other VOCs in the country, initially Gamma and later Delta. Interestingly, the NE and NW regions exhibited the highest lineage diversity and the lowest prevalence of B.1.1.519 at the time of the introduction of Alpha (Fig. 2). These conditions may have contributed to this variant’s relative success in northern Mexico compared to in the rest of the country.

### Mutations observed in the B.1.1.7 sequences.

The B.1.1.7 lineage is characterized by 18 amino acid changes compared to the reference sequence Wuhan Hu-1 (four inherited from its parental B.1.1 lineage) and three in-frame deletions (open reading frame 1a [ORF1a]:del3676/3678, S:del69/70, and S:del144; Table 2). This large number of genetic changes contrasts with the observation that the SARS-CoV-2 virus accumulates around two nonsynonymous substitutions per month. The lineage-defining amino acid changes and deletions were present in 98.6% (*n* = 1,224) of the Mexican viral genomes (Table 2).

**TABLE 2:** Frequency of additional mutations for the B.1.1.7 lineage observed in sequences of Mexico as well as other abundant mutations identified

Type	Gene^*a*^	Amino acid	Mexican sequences (*n* = 1,241)		Worldwide frequency (%) (*n* = 1,089,354)	Haplotype cluster^*b*^
			No.	Frequency (%)		
Characteristic substitutions	ORF1a	T1001I	1,238	99.70	98.61	C1, C2, C3, C4, C5
		A1708D	1,238	99.70	99.17	C1, C2, C3, C4, C5
		I2230T	1,228	98.90	97.35	C1, C2, C3, C4, C5
	ORF1b	P314L^*b*^	1,241	100.00	99.27	C1, C2, C3, C4, C5
	S	N501Y	1,232	99.27	97.89	C1, C2, C3, C4, C5
		A570D	1,236	99.59	99.50	C1, C2, C3, C4, C5
		D614G^*b*^	1,241	100.00	99.57	C1, C2, C3, C4, C5
		P681H	1,241	99.99	99.28	C1, C2, C3, C4, C5
		T716I	1,239	99.60	99.01	C1, C2, C3, C4, C5
		S982A	1,231	99.19	98.01	C1, C2, C3, C4, C5
		D1118H	1,237	99.68	98.79	C1, C2, C3, C4, C5
	ORF8	Q27stop	1,227	98.88	95.33	C1, C2, C3, C4, C5
		R52I	1,229	99.04	98.68	C1, C2, C3, C4, C5
		Y73C	1,230	99.12	99.14	C1, C2, C3, C4, C5
	N	D3L	1,219	98.23	98.01	C1, C2, C3, C4, C5
		R203K^*b*^	1,234	99.44	98.17	C1, C2, C3, C4, C5
		G204R^*b*^	1,167	94.06	90.94	C1, C2, C3, C4, C5
		S235F	1,236	99.59	98.34	C1, C2, C3, C4, C5
Deletion	ORF1a	del3676/3678	1,233	99.36	96.69	C1, C2, C3, C4, C5
	S	del69/70	1,222	98.48	85.62	C1, C2, C3, C4, C5
		del144	1,223	98.56	94.03	C1, C2, C3, C4, C5
Other prevalent mutations (>5%)	ORF1a	L730F	231	18.60	7.26	C4
		E913D	72	5.80	1.08	C2
		M2259I	381	30.70	5.30	C1, C2
		L3116F	217	17.48	0.29	C4
		Q3966R	63	5.05	5.97	–
	ORF1b	P218L	442	35.42	13.71	C1, C2
		K1383R	105	8.41	0.11	–
		K2557R	66	5.29	20.71	–
	S	S98F	217	17.39	1.73	C4
		D138H	193	15.46	1.03	C4
		L938F	119	9.54	0.09	C5
		K1191N	63	5.05	3.28	–
		E1258D	231	18.51	0.005	C1^*c*^, C2^*c*^, C3^*c*^, C4^*c*^, C5^*c*^
		D1259H	73	5.85	0.002	C1^*c*^, C2^*c*^, C3^*c*^, C4^*c*^, C5^*c*^
	ORF3a	P240S	106	8.49	0.99	C2
	ORF7a	R118G	74	5.93	0.01	C1^*c*^
	ORF8	C61F	255	20.43	2.33	C1
		K68stop	382	30.61	34.39	C1^*c*^, C2^*c*^
	N	N8D	218	17.50	0.41	C4
		G204P	66	5.30	7.46	–

a N, nucleocapsid; S, spike.

b Clusters of the haplotype network where the mutation was detected. An en dash indicates the mutation is not present in any analysed cluster.

c Only some sequences have the amino acid change.

In addition to the lineage-defining changes, 20 nonsynonymous mutations were identified in at least 5% of the Mexican virus genomes (Table 2), with some of them being more prevalent in Mexican isolates than globally. For example, ORF1a:M2259I and ORF1b:P218L changes were found in more than 30% of Mexican sequences, while their worldwide frequencies were 5.3% and 13.7%, respectively. Also, in the spike protein, S:S98F, S:E1258D, and S:D138H mutations were identified in more than 15% of genomes, while globally, they were detected in less than 2%. Finally, 68.7% of the remaining amino acid substitutions were observed within one or two B.1.1.7 sequences.

**FIG 3:**
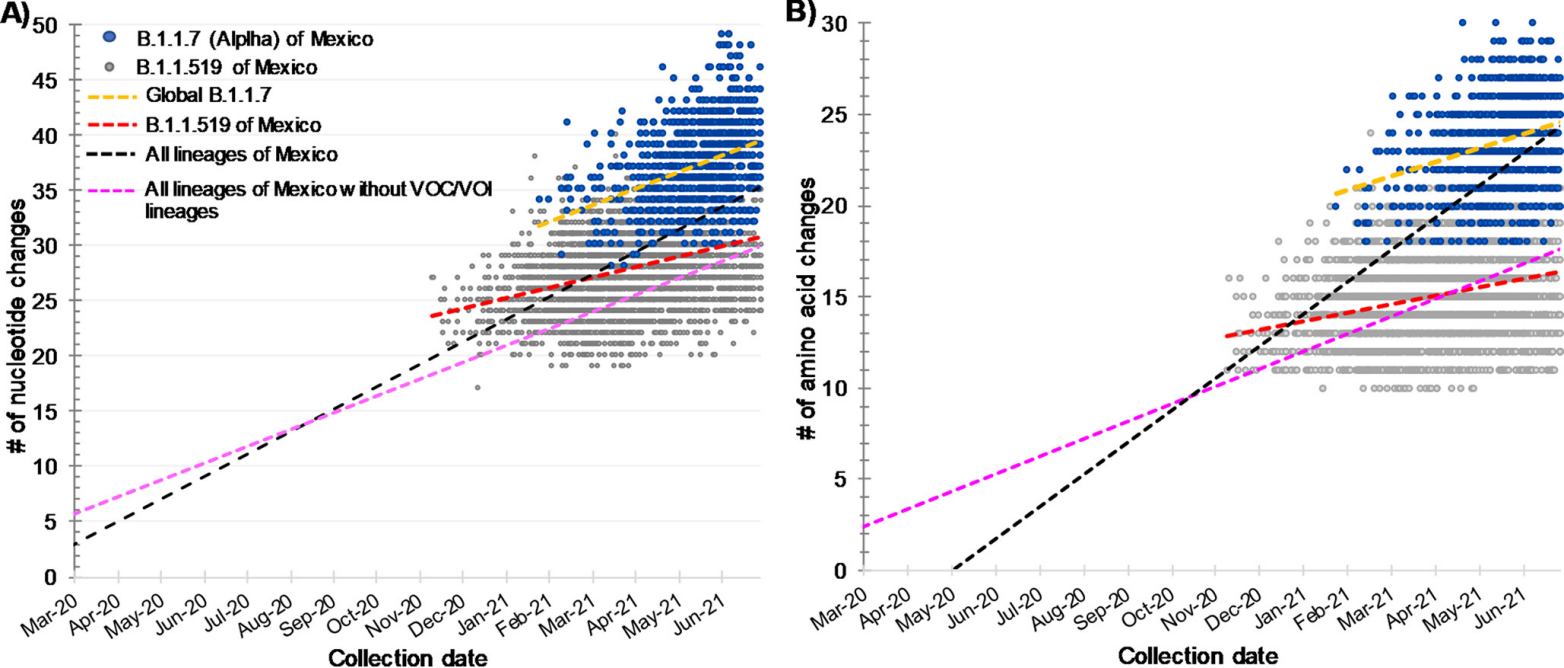
Genomic changes of Mexican B.1.1.7 and B.1.1.519 variants compared to the reference Wuhan-Hu-1 estimated by the Nextclade tool using sample collection date. (A) Nucleotide changes. (B) Amino acid changes.

The temporal comparison between B.1.1.7 and B.1.1.519 variants showed that B.1.1.519 had more nonsynonymous changes (1,565) than B.1.1.7 (1,185) compared to the reference sequence Wuhan-Hu-1, possibly due to the more extended period of circulation of B.1.1.519 in Mexico. Interestingly, in the B.1.1.7 lineage, the amino acid average changes per genome was higher (23.8 ± 2.3) than in B.1.1.519 (15.03 ± 2.2). Furthermore, the B.1.1.7 lineage was more divergent in nucleotide and amino acid changes than B.1.1.519, counting from the root of the phylogeny (Fig. 3A and B), resulting in the acquisition of 14 lineage-specific amino acid changes for B.1.1.7, while B.1.1.519 only obtained seven lineage-specific amino acid changes. Once the B.1.1.7 variant was globally established, its sequences also showed a faster evolution through time compared to the B.1.1.519 lineage (Fig. 3A, yellow linear regression); on average, B.1.1.7 showed 1.6 nucleotides per month compared to 1 for B.1.1.519 (Fig. 3A, red linear regression). This higher nucleotide evolution rate for the B.1.1.7 lineage was also observed in nonsynonymous mutations (Fig. 3B, yellow linear regression), having on average 0.83 amino acid changes per month for B.1.1.7 in contrast to 0.38 for the B.1.1.519 variant (Fig. 3B, red linear regression).

### Phylogenetic and haplotype analysis of B.1.1.7 Mexican sequences.

**FIG 4:**
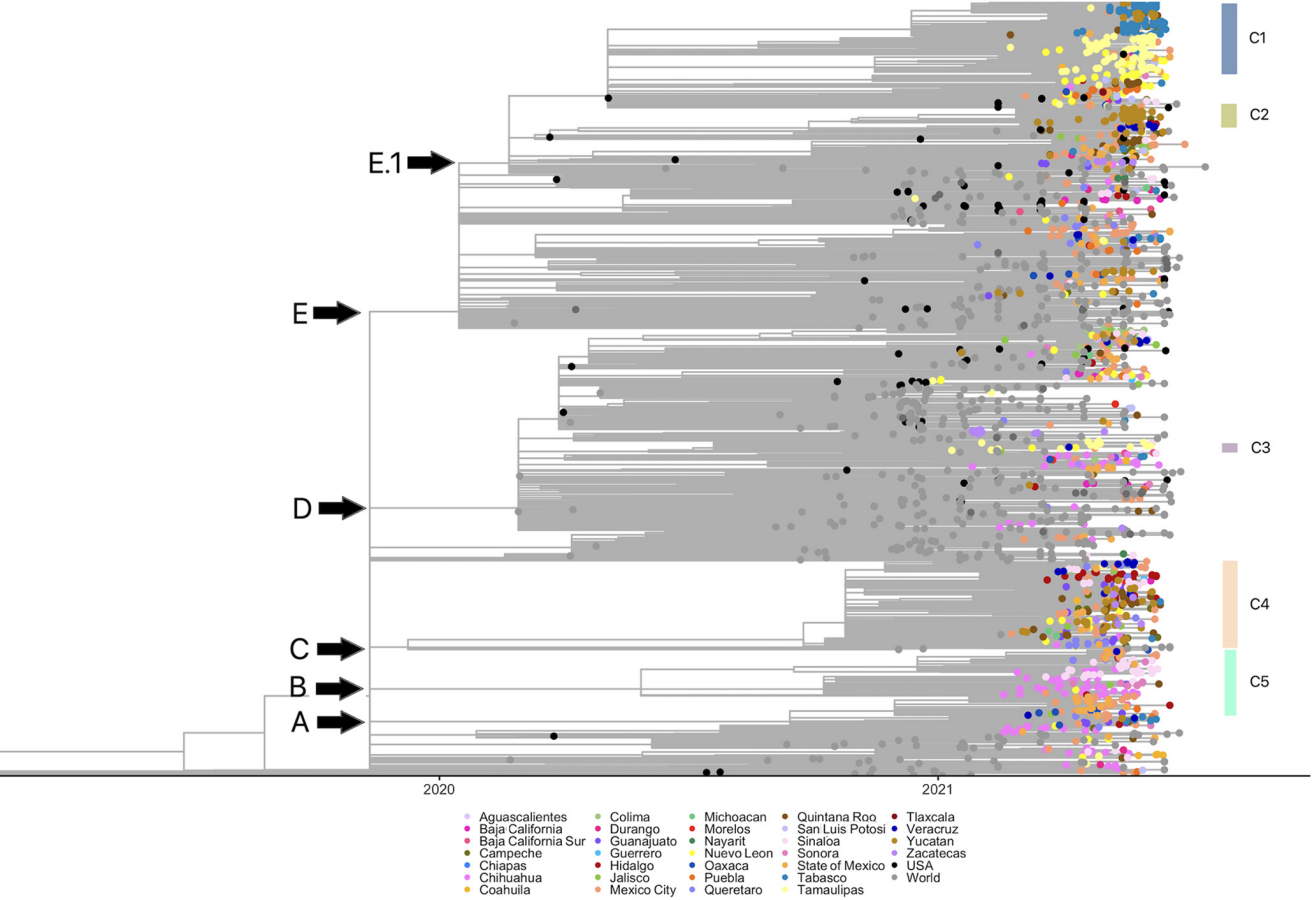
Time-scaled maximum likelihood phylogenetic tree of the B.1.1.7 lineage of Mexican and international sequences. All Mexican isolates are in different colored circles according to sampling location; black circles are from the United States, and gray circles represent other countries. The correspondence of clades with the major Mexican haplotype clusters is indicated.

A time-scaled maximum-likelihood phylogenetic tree was constructed to understand the temporal and spatial evolutionary relationships of Mexican B.1.1.7 sequences with international isolates (Fig. 4). In this figure, multiple international and U.S. sequences can be observed in abundance by October 2020, while Mexican sequences appear later. Five large monophyletic groups are highlighted (Fig. 4, clades A to E), which are polytomies with an internal branch structure. Clades A, B, and C contain mostly Mexican sequences, while clade D is composed mainly of international viruses. In contrast, clade E, the largest one, has a subclade (E.1) with primarily Mexican viruses. The distribution of Mexican sequences throughout the phylogeny suggests that multiple introduction events occurred since they did not form a monophyletic group. Interestingly, many Mexican sequences were singletons (only one observed sequence) or formed small clades, suggesting that many introductions did not lead to community transmission. However, in some cases, these introductions resulted in large local community transmission chains, as observed in the internal subclades (C1 to C5) of clades A, B, C, and E.

**FIG 5:**
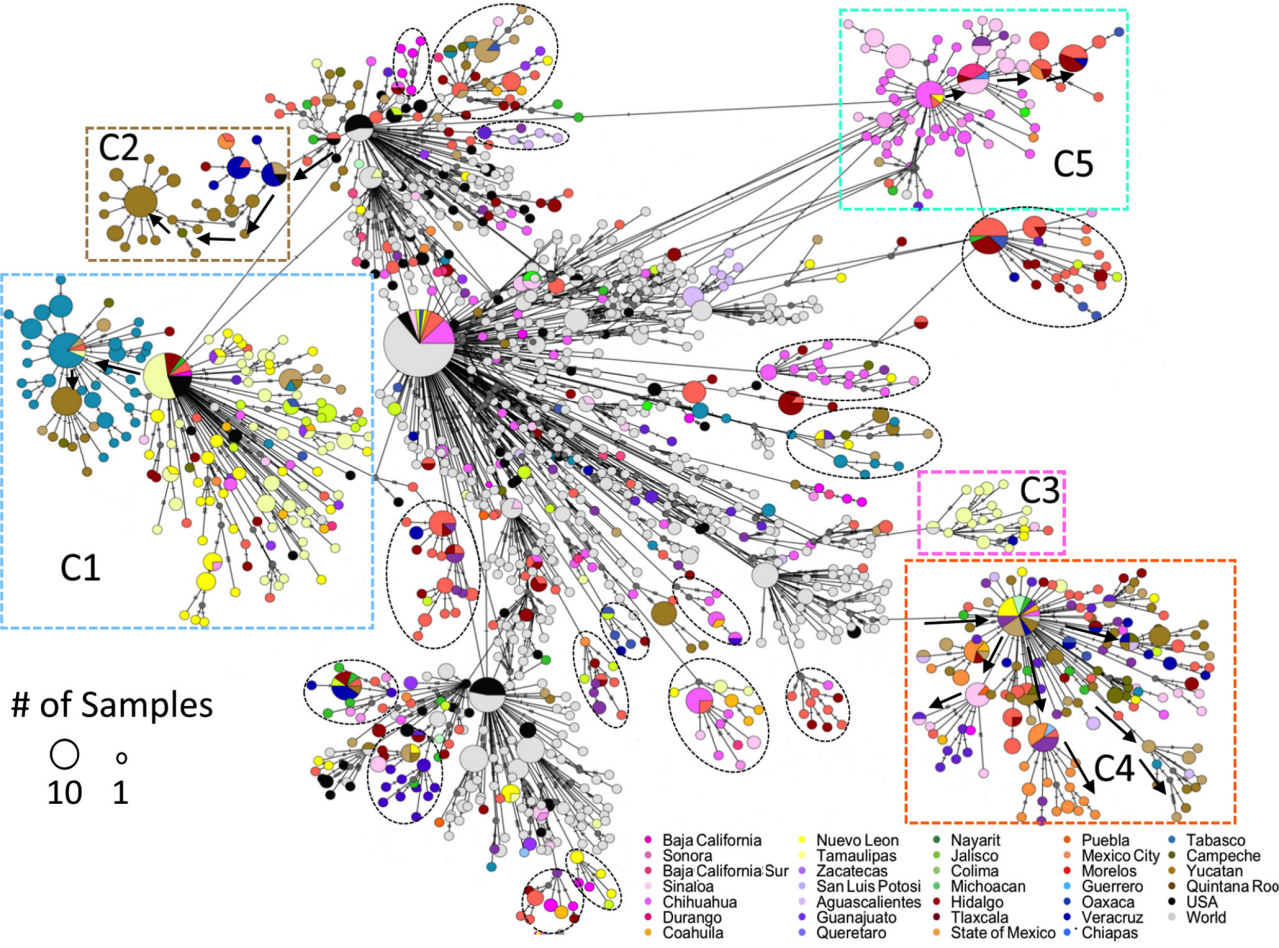
Haplotype network using genome-wide single-nucleotide variations of the B.1.1.7 lineage from Mexican and international sequences. All Mexican isolates are in different colored circles according to sampling location; black circles are from the United States, and gray circles represent other countries. The size of the circles corresponds to the number of samples within the same haplotype (scale is provided). Some Mexican clusters are highlighted with dashed rectangles and circles. In Fig. S2 in the supplemental material, a high-resolution haplotype network is provided, with mutations between sequences represented by the number of dashes in the connecting lines.

A haplotype network was constructed to discern the relationship between sequences at the tips of the tree and to document multiple introductions, local transmission, and spread patterns of B.1.1.7 lineage in Mexico (Fig. 5). The haplotype network shows that some clusters are unique for Mexican virus genomes, in agreement with the phylogeny (Fig. 4), with several of them containing sequences from a single or few Mexican states, sometimes directly deriving from other Mexican haplotype clusters and possibly representing local transmission chains. Moreover, the high-frequency substitutions identified in the B.1.1.7 Mexican sequences were associated with only one or a few clusters (Table 2), suggesting the existence of separate local transmission chains that circulated for several months.

In the center of the network, a large cluster of Mexican and international sequences is located (central nodes in Fig. 5). However, since the Mexican sequences did not form a single subgroup (the majority are singletons), these likely correspond to separate introduction events from the United States (black) or the rest of the world (gray), which did not result in sustained community transmission. Nevertheless, insufficient sampling cannot be ruled out.

Three large clusters of Mexican sequences (multicolored vertices indicate different states) can be observed in the network and are marked as C1, C4, and C5, representing the largest transmission chains in Mexico. In C1, sequences mainly from Tamaulipas (yellow) can be identified. A subgroup of Tabasco sequences (blue) branches out from the initial group, including a smaller subgroup of sequences from Yucatán (brown). These data suggest a flow of viruses in C1 from the Northeast (Tamaulipas) toward the Southeast (Tabasco and Yucatán). In agreement with this observation, the spatiotemporal distribution of C1 sequences (Fig. S3A) shows that the earliest identification and higher prevalence was in the northern state of Tamaulipas, followed by dispersion to other states, especially in the south. Even though many sequences from Tamaulipas and Yucatán were present in C1, other haplotypes were in circulation in those states during the same period, for instance, the sequences in C3 from Tamaulipas (yellow) or in C2 from Yucatán (brown).

C5 contains samples from Chihuahua (dark pink) and Sinaloa (light pink) states in the northwest of the country. A subgroup within C5 shows the presence of sequences from central Mexico (red and orange colors), including Mexico City and Hidalgo. This cluster also suggests a transfer of viruses from the northwest (Chihuahua and Sinaloa, among others) toward the country’s center. Figure S3B corroborates the introduction of C5 in Chihuahua at the Mexican-U.S. border and then its spread to central and western Mexico in parallel to the dispersion observed for C1. In contrast, C2 sequences show a possible introduction of B.1.1.7 in the southeastern state of Quintana Roo, in which the tourism destination of Cancun is located (Fig. S3C). A limited dispersion to neighboring states can then be observed, although it remained at frequencies lower than 5%.

However, C4 shows more diversity in geographical provenance location but with a common origin by a single introduction of an international haplotype (not from the United States). Small subclusters in the periphery of the central node indicate state-specific transmission, but, overall, the circulation pattern seems to be nationwide.

On the other hand, dashed circles indicate other small subgroups, many originating in a single state and potentially representing other local minor transmission chains. The list of sequences comprised in clusters C1 to C5 is reported in Table S4.

Finally, as mentioned before, the observation that the Mexico-U.S. border states showed the highest prevalence of the B.1.1.7 variant and the largest transmission chains (clusters C1 and C5) suggests dispersions of the virus from those states into the country’s interior. To further explore this possibility, an additional phylogenetic analysis (Fig. S4) was done to search for possible introduction events from the United States. The phylogeny shows that many Mexican sequences interspersed within sequences from the United States, forming many Mexican singletons, suggesting numerous introductions without local transmission, at least in northern Mexico. However, some Mexican clades, particularly C1 and C5, were grouped with a sister clade formed by U.S. sequence(s), supporting the idea that the largest national transmission chains entered from the United States.

## DISCUSSION

In Mexico, the prevalence of B.1.1.7 remained at low frequency during the first trimester of 2021, ranging between 1% and 3% and rising to 8.8% in April. Contrary to reports from the United Kingdom, where the circulation of B.1.1.7 peaked in March, in Mexico, the highest prevalence was detected 2 months later, reaching its maximum peak in May (18.8%). Interestingly, B.1.1.7 neither became predominant nor entered an exponential growth phase in Mexico, contrary to what was observed in Europe and the United States (13, 17–19), where it reached 98.0% and 64.1% prevalence, respectively, of all reported sequences. The high prevalence of B.1.1.7 in the United States correlates with the high prevalence observed for the northern states of Mexico, ranging from 20% to 40% and becoming the dominant variant in the region. However, the frequency of this lineage never increased past 20% nationwide. The low relative frequency of B.1.1.7 may be attributed to the expansion of the B.1.1.519 variant, which was dominant in Mexico during the first half of the year, except in the northern states. It has been reported that B.1.1.519 had a secondary attack rate of 2.9 during the surge of the second wave of coronavirus disease 2019 (COVID-19) in Mexico City, while the second most frequent variant (B.1.1.222) in that period had a secondary attack rate of 1.93 (20). These observations suggest that B.1.1.519 has higher fitness than other circulating variants in Mexico and may have limited the spread of B.1.1.7. Furthermore, the introduction to the country of Gamma and Delta in the subsequent months and, in particular, the rapid expansion of the Delta variant from May 2021 may have also contributed to its low prevalence.

The evolution of the B.1.1.7 variant as well as other VOCs was driven by an episodic increase in the evolutionary rate of around 4-fold compared to cocirculating variants (5, 21). This period of rapid evolution led to the acquisition of 14 lineage-specific amino acid substitutions and three deletions in around 14 weeks, being more divergent from the root and its parental lineage than expected according to the estimated mean mutation rate of approximately 24 substitutions per year. Evolutionary jumps have been observed in other VOIs/VOCs, and, interestingly, B.1.1.519 also shows this pattern of discontinuous evolution, having obtained seven key mutations in a short period compared to its parental B.1.1 lineage (16). Moreover, when considering the period following the emergence of B.1.1.7, this variant showed higher nucleotide and amino acid substitution rates (on average 1.6 nucleotides and 0.83 amino acid changes per month) than those observed in lineage B.1.1.519 (1 and 0.38, respectively).

Although limited phenotypic information of lineage B.1.1.519 is available, it shares S:T478K with the Delta variant, which has been more thoroughly characterized. The T478K substitution confers to the S protein a less than 2-fold increase in affinity to ACE2, significantly lower than the 7-fold affinity increase reported for the S:N501Y change (22). T478K also resulted in a 1.5-fold increase in cell entry, as measured in a pseudovirus assay (23). The presence of these substitutions might confer B.1.1.519 some advantage over the lineages circulating in Mexico at the end of 2020 but not necessarily against B.1.1.7, which was able to spread worldwide. Also, this competition took place when overall virus transmission was low, between the second and third waves, which may have given transmission superiority to the B.1.1.519 dominant virus despite the high transmissibility of B.1.1.7; also, as mentioned before, the introduction of other VOCs could have contributed to the B.1.1.7 low prevalence.

Distinct Mexican geographical clusters of the B.1.1.7 variant were identified using phylogenetic and haplotype network approaches. The presence of clusters suggests different local transmission chains, alongside multiple repeated introduction events from other countries that failed to produce sustained community transmission events and do not cluster with other Mexican sequences. A large cluster (C1) with sequences from the northern state of Tamaulipas, with collection dates spanning May to June 2021, showed a possible migration event toward the southeast (Tabasco and Yucatán) of the country, where most sequences were collected in June. Another possible introduction from the northern region can be observed in C5, with sequences from Chihuahua (located at the Mexico-U.S. border) predating those from central Mexico. Together with their phylogenetic relatedness to U.S. sequences, these results support the idea that introduction events that resulted in continued local community transmission occurred in the northern states before spreading south. However, not all B.1.1.7 introductions occurred at the northern border. For instance, C2 sequences appeared first in Quintana Roo in the southeast, but its dispersion was limited, probably due to the circulation of other B.1.17 haplotypes and the dominant B.1.1.519 variant in this region and the introduction of other VOCs. However, given the limitations of sequencing efforts, the necessary subsampling of international data, and the limited diversity of the viruses, the elucidation of the exact origin of most Mexican clades is not feasible.

Recently, some global variants of B.1.1.7 have been designated sublineages (Q.1 to Q.8). The circulation of these sublineages has been local, and none of them have been distributed beyond a few countries. Only three of these have been detected in Mexico, albeit at extremely low frequencies. Q.3, the most sampled sublineage, only reached 2.5% of all Alpha sequences, whereas for Q.1 and Q.8, only one sequence was detected. Some of nonsynonymous mutations, with a prevalence higher than 5% in Mexican sequences, have been reported to be associated with some of the sublineages of B.1.1.7. Interestingly, 10 of the additional prevalent amino acid changes identified abundantly in B.1.17 Mexican sequences were exclusive to one of the five clusters described in this work. Additionally, three mutations were common to two clusters, supporting the idea that most of the prevalent mutations may be associated with events of community transmission chains within Mexico.

In conclusion, in this work, we have established that the circulation of B.1.1.7 in Mexico was temporally and geographically limited, contrary to reports from European countries and the United States. This finding highlights that lineage dynamics is a complex multifactorial phenomenon that is difficult to predict until a more thorough characterization of all variants and a comprehensive analysis of social dynamics is attained. Therefore, to better understand and cope with emerging variants on the global scale that carry mutations of potential biological significance, higher sequencing and surveillance across Mexico are necessary.

## MATERIALS AND METHODS

### Epidemiological analysis of SARS-COV-2 in Mexico.

All demographic data of positive, negative, and deceased cases (age, origin, sex, date of onset of symptoms, date of sampling, and clinical information), by epidemiological week, were provided by the Dirección General de Epidemiología de la Secretaría de Salud (General Epidemiology Department of the Health Ministry) of Mexico. The population size was determined with the projections made by the National Population Council (CONAPO). This information was used to calculate the incidence rate per 100,000 inhabitants. The case fatality rate represents the proportion of the weekly number of deaths among the confirmed number of positive COVID-19 cases in the same period. The positive rate was the weekly proportion of positive COVID-19 cases per processed samples.

### Bioethics and sample collection.

The samples used in this study and their associated metadata are part of the national Public Health response to COVID-19 collected by the Mexican Consortium for Genomic Surveillance (CoViGen-Mex) under the Mexican Official Norm NOM-017-SSA2-2012 (24) for the epidemiological surveillance program. All samples were unlinked from any personal identifiers before the commencement of the study and informed consent was waived. Oropharyngeal or nasopharyngeal swab samples were collected from all 32 states of Mexico in laboratories and hospitals under the scope of the Ministry of Public Health of Mexico (Instituto Mexicano del Seguro Social [IMSS], Centro de Investigación en Alimentación y Desarrollo [CIAD], and Instituto Nacional de Enfermedades Respiratorias [INER]). Around 1,200 samples per month were selected for sequencing. In total, 6,585 positive samples of SARS-CoV-2 were confirmed by real-time reverse transcription PCR (RT-qPCR) collected from 1 December 2020 to 9 July 2021, with a cycle threshold (*C_T_*) value equal to or less than 25. The sample processing and RT-qPCR protocols used are validated by Instituto de Diagnóstico y Referencia Epidemiológicos, Secretaría de Salud, Mexico (InDRE), as approved by the World Health Organization. Briefly, for RT-qPCR, 5 μL of RNA was used in a 25-μL reaction using the Superscript III one-step RT-PCR system (Invitrogen, Darmstadt, Germany). Reverse transcription was done for 10 min at 55°C, followed by PCR for 45 cycles, 95°C for 15 s, and 58°C for 30 s.

### SARS-CoV-2 whole-genome sequencing and genome generation.

The extracted RNA from the 6,585 samples that tested RT-qPCR positive for SARS-CoV-2 were subjected to amplification and next-generation sequencing. From the remanent RNA, total cDNA was synthesized by Superscript III reverse transcriptase (Thermo Fisher, USA) and random hexamers. Next, the POLAR nCoV-2019 amplicon sequencing protocol was used with the V3 primer set (25). Samples were then barcoded using the native barcode kits. Nextera XT sequencing libraries were prepared using the ligation sequencing kit, followed by sequencing on a midoutput kit in the NextSeq500 platform using 2 × 150 cycles of paired-end runs with an insert size of 500 bp. FASTQ reads were generated by the Illumina pipeline at BaseSpace (https://basespace.illumina.com).

Adapters, low-quality bases, dereplication, and off-target reads were removed for each sample using a customized pipeline described previously (26). Then, unique and high-quality reads were mapped with Bowtie2 v2.3.4.3 (27) against the Wuhan-Hu-1 (MN908947) reference genome. Consensus calling was performed with iVar (v1.3.1) (28) using bases scored with a Q value of >20 and a minimum read coverage depth of 20×, bases with lower depth were assigned as N. In total, 6,352 genome sequences comprised at least 90% coverage of the Wuhan-Hu-1 reference genome and were considered useful for genetic diversity and lineage composition analyses.

### Mexican B.1.1.7 genomes data set.

SARS-CoV-2 genome sequences were initially assigned to viral lineages according to the nomenclature proposed by Rambaut et al. (29) using the Pangolin v3.1.7 desktop application. In total, 473 virus genome sequences assigned as B.1.1.7 lineage were deposited in the Global Initiative on Sharing All Influenza Data (GISAID) platform (https://www.gisaid.org/) and GenBank (Table S1 in the supplemental material). To better characterize the genetic and distribution data of the B.1.1.7 lineage in Mexico, we obtained all other Mexican SARS-CoV-2 sequences from GISAID from the same dates as our genomes that were assigned to the B.1.1.7 lineage using the Pangolin database web interface (v3.1.7; https://github.com/cov-lineages/pangolin, accessed on 24 July 2021; Table S1). We obtained 1,141 additional sequences with their metadata, resulting in a total of 1,620 genomes of the B.1.1.7 lineage. To compare and contextualize, we also downloaded all available B.1.1.519 sequences within the same sampling as B.1.1.7. Finally, all other Mexican sequences from that period were used as controls. The metadata (geographical location, gender, age of the patient, and sampling date) of the B.1.1.7 and B.1.1.519 Mexican sequences used in this work are reported in Tables S2 and S3, respectively.

### Sequence alignment.

From the 1,620 genome sequences of B.1.1.7 lineages from Mexico, only those with less than 1% of Ns (undetermined nucleotides) were selected (*n* = 1,241). International B.1.1.7 sequences available in GISAID were subsampled for temporal and spatial analysis using the following strategy: one random sequence per country and month (excluding Mexico) was selected from March to October 2020. From November 2020 to July 2021, no more than 20 sequences per month were selected from Europe, 20 from North America (10 from the United States), 20 from Asia, 10 from Africa, 5 from South America, and 5 from Oceania. In total, 709 international sequences were included in the alignment. The sequences were aligned against the reference sequence from Wuhan (NC_045512.2) using MAFFTv7 (30) (with the parameters –addfragments). The alignment was manually edited to remove untranslated (UTR) regions.

### Regional lineage distribution.

To assess the differences in lineage distribution, we divided the country into seven regions as follows: Northeast (NE; Coahuila, Nuevo León, and Tamaulipas), Northwest (NW; Baja California, Baja California Sur, Chihuahua, Durango, Sonora, and Sinaloa), Central North (CN; Aguascalientes, Guanajuato, Querétaro, San Luis Potosí, and Zacatecas), Central South (CS; Mexico City, Estado de México, Morelos, Hidalgo, Puebla, and Tlaxcala), West (W; Colima, Jalisco, Michoacán, and Nayarit), Southeast (SE; Guerrero, Oaxaca, Chiapas, Veracruz, and Tabasco), and South (S; Campeche, Yucatán, and Quintana Roo). For each region, we built a stack density plot showing lineage circulation through time using the package ggplot2 in R.

### Haplotype network and amino acid changes analysis.

Aligned sequences, considering 1,241 Mexican sequences of high quality along with the other 709 international genomes, were used to generate a haplotype network. The population analysis with reticulate trees (PopArt v1.7) software (31) was used to construct a statistical parsimony, at a 95% confidence level, TCS network (32), which is based on an agglomerative approach, where clusters are progressively combined with one or more connecting edges. Sites with more than 5% of undefined states were masked. Also, to estimate geographical relationships of haplotype groups, the network was colored using the respective state where samples were taken by using a trait in the nexus data.

The Nextclade single nucleotide variant (SNV) calling system was used to identify synonymous/nonsynonymous substitutions in the Mexican sequences (33), enabling us to determine the association of nucleotide SNPs to a particular haplotype or geographical group. Also, Nexclade amino acid mutations annotation was used to compare between variants and to estimate evolutionary rates. For the most prevalent haplotypes, a map series was done showing their distribution through time in the country using the package mxmaps in R.

### Phylogenomic analysis.

The multiple sequence alignment was used to reconstruct a maximum likelihood phylogeny using iqtree v.2.1.1 (34) with the GTR+F+R3 substitution model (35) and the feature LSD2 to scale the resulting phylogeny based on collection date to ensure all child nodes had a later collection date than their parent node (36). ggtree v.3.0.2 (37) and treeio v.1.16.1 (38) packages were used to plot the tree using R. Additionally, to explore further the relationship between B.1.1.7 viruses of Mexico and the United States, a phylogeny was built in Nextstrain using all high-quality sequences from Mexico’s northern states plus 100 sequences per month from the United States and the global subsampling from Nextstrain.

### Statistical analysis.

The statistical analyses and plots were generated with R using the ggplot2 and stats packages available from the CRAN repository. Medians, interquartile ranges (IQRs), and statistical tests to compare groups were calculated and performed in R. Statistically significant differences of the median patient age distribution grouped by lineage were assessed by Wilcoxon rank sum test. In contrast, differences between gender or patient status per lineage were evaluated using the chi-square test.

### Data availability.

The generated sequences of SARS-CoV-2 used in this study have been publicly shared through the Global Initiative on Sharing All Influenza Data (GISAID) repository and have also been deposited in the GenBank NCBI database. Accession numbers are listed in Table S1.
