# Supplementary material for: The Alpha Variant (B.1.1.7) of SARS-CoV-2 Failed to Become Dominant in Mexico
Source: Microbiol Spectr. 2022 Apr 7;10(2):e02240-21. doi: 10.1128/spectrum.02240-21 (PMC9045257; doi:10.1128/spectrum.02240-21)
Supplement: SUPPLEMENTAL FILE 1 — Supplemental material. Download SPECTRUM02240-21_Supp_1_seq8.pdf, PDF file, 7.3 MB [file spectrum02240-21_supp_1_seq8.pdf]

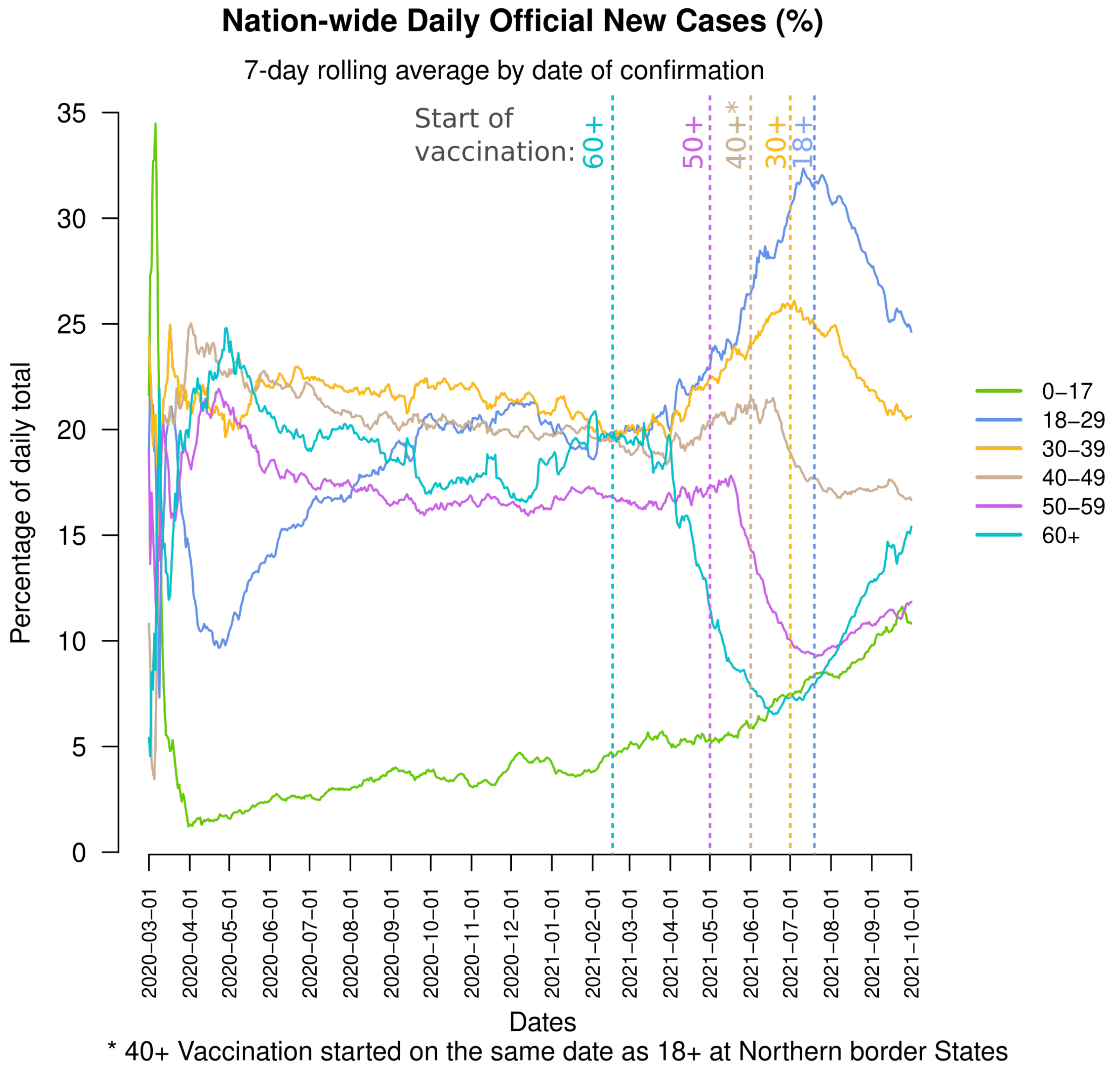

Figure S1. Distribution of daily new cases by group of age. The weekly rolling average by date of confirmation is shown as a proportion of the number of new cases corresponding to each age group. The stating date of the vaccination campaign for each group is shown in dashed vertical lines.

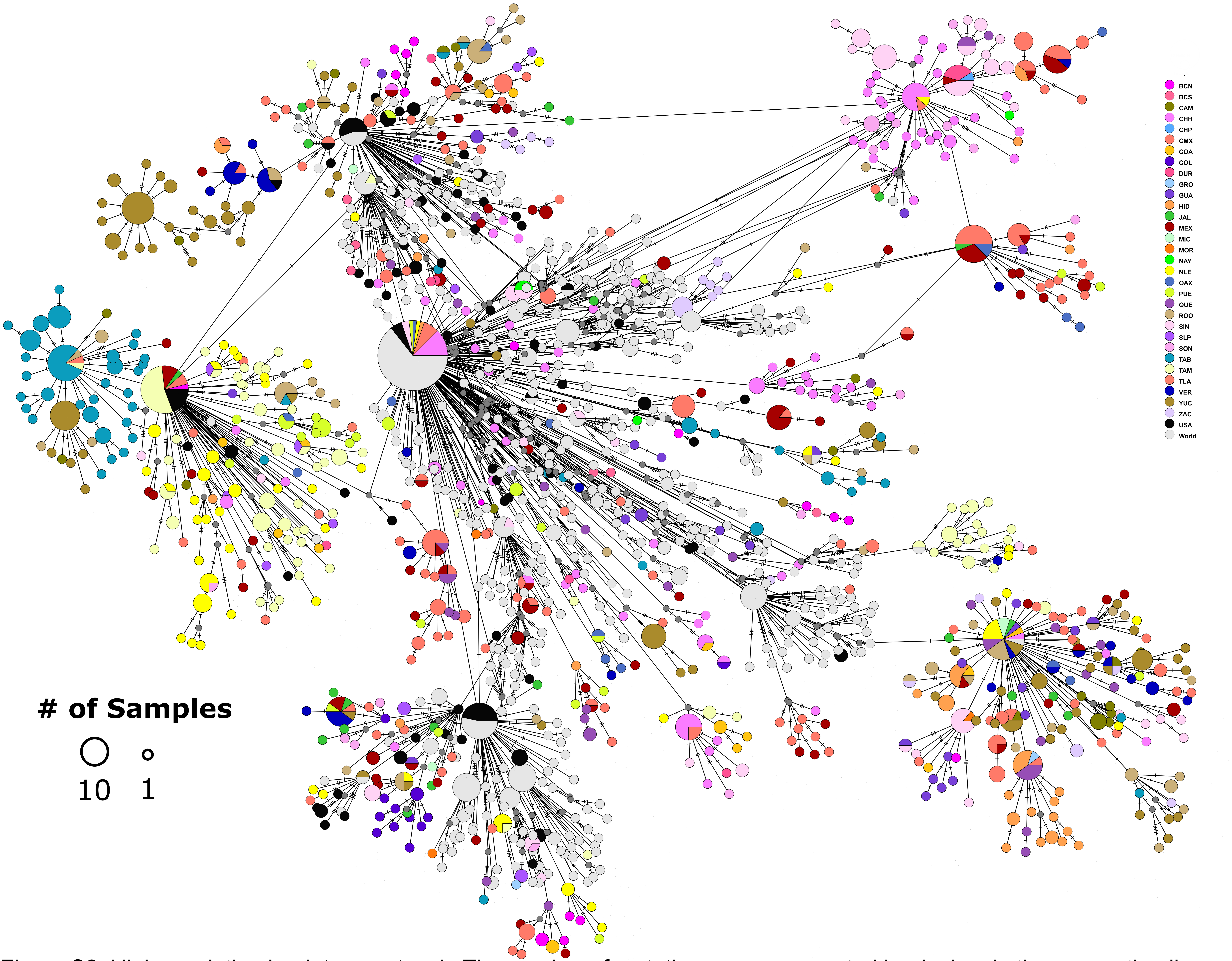

Figure S2. High-resolution haplotype network. The number of mutations are represented by dashes in the connecting lines.

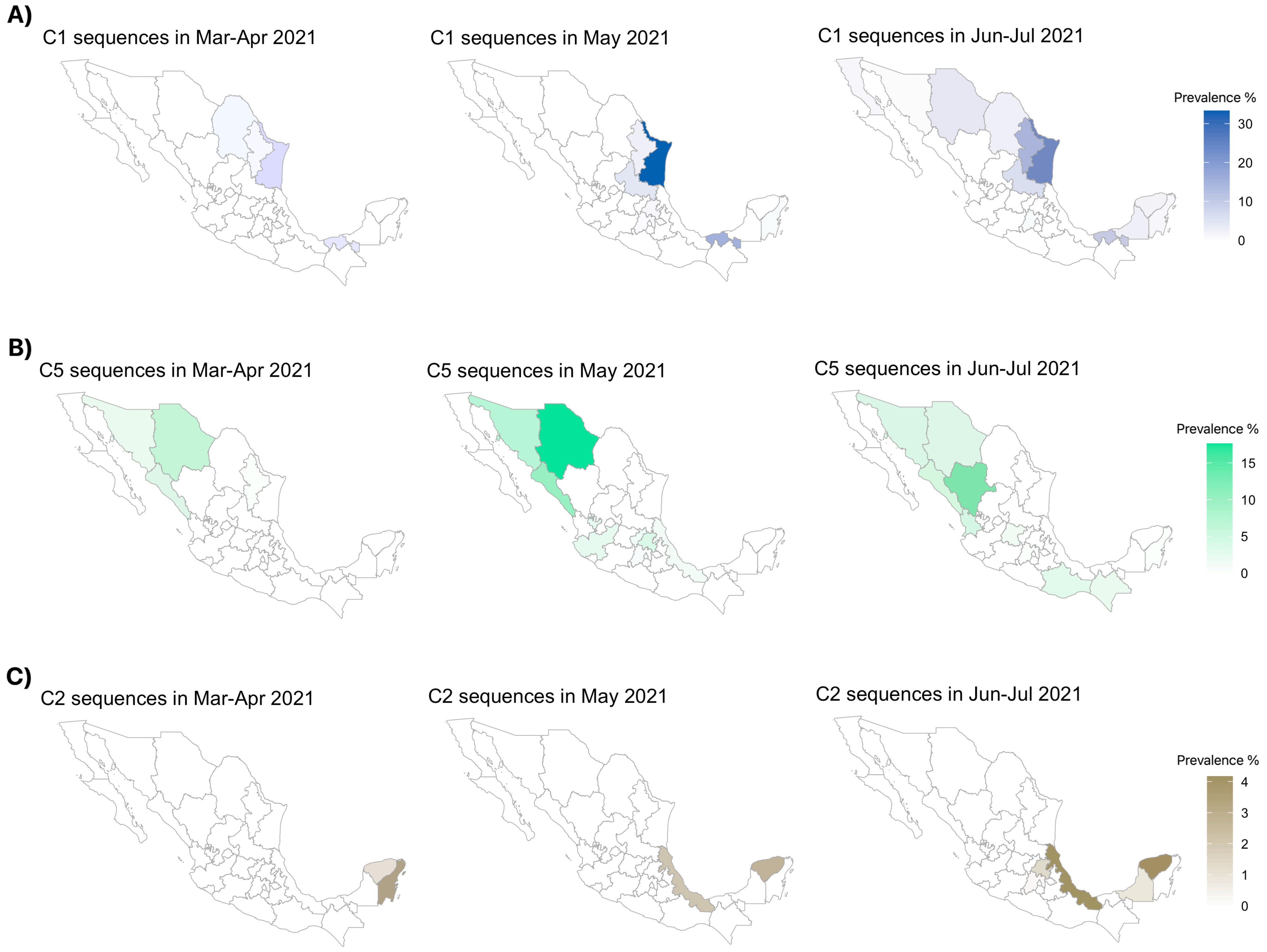

Figure S3 Map series showing the spatiotemporal distribution of B.1.1.7 sequences form different clusters. A) Sequences in C1. B) Sequences in C5. C) Sequences in C2.

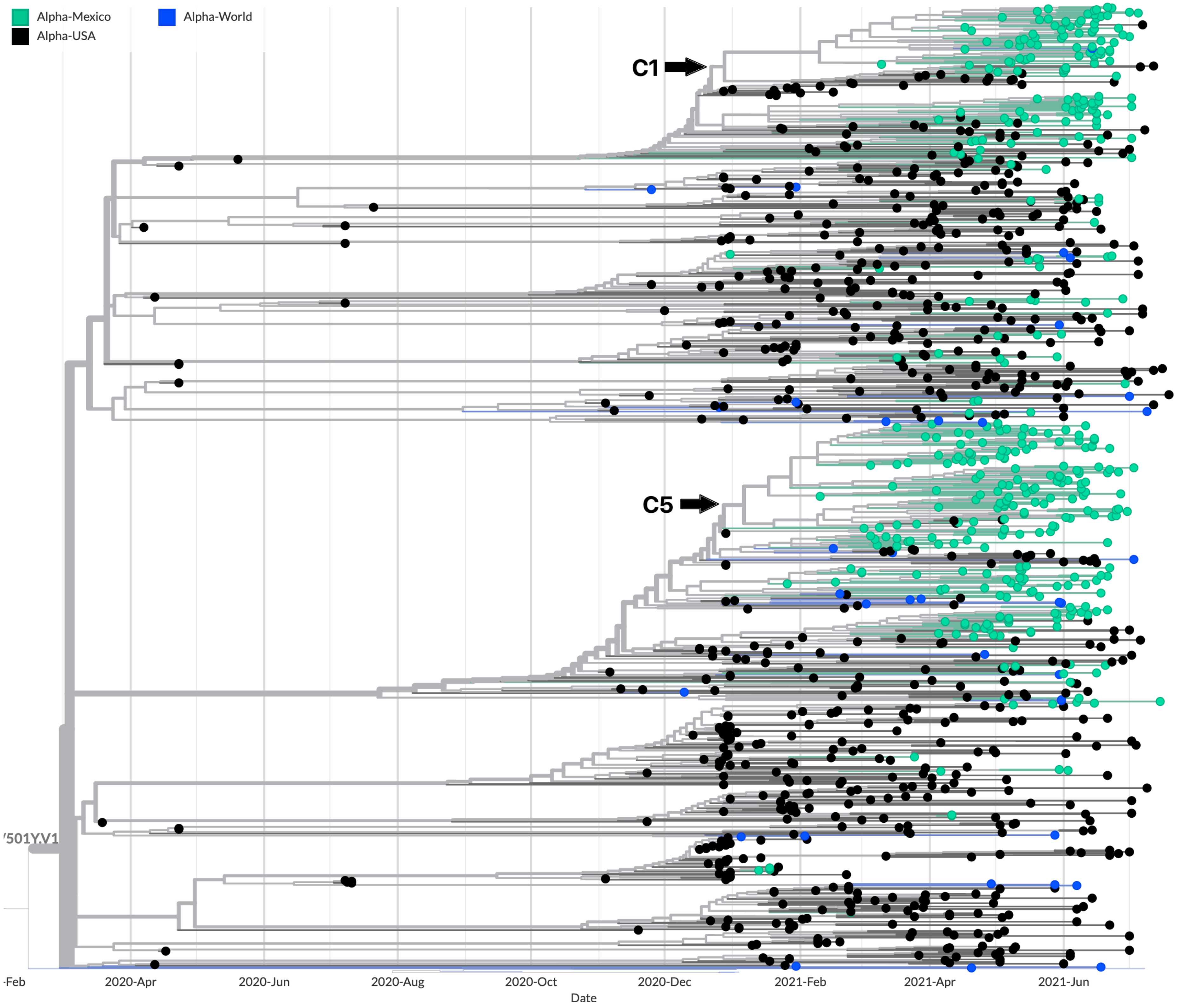

Figure S4. Zoom of a Nextstrain phylogeny depicting the relation between B.1.1.7 sequences sampled in the northern states of Mexico, the USA, and global sequences. The tips are colored by sampling location. The location of clusters C1 and C5 is shown.

Table S1. Description of full-length B.1.1.7 SARS-CoV-2 Mexican genomes generated and used in this study. The 473 genome sequences generated by us are shown in blue.

Virus name	Accession ID	Genbank Id	Nextstrain Clade	Pango lineage (v2.2.1, 21-02-06)	Changes compared to the reference genome of Wohan						
					# of nt	nt substitutions	# of aa	aa changes	# aa Deletions	aa Deletion	Missing bases
hCoV-19/Mexico/BCN-IBT_IMSS_2004/2021	EPI_ISL_2801856	OL461235	B.1.1.7	GRY	31	C241T,C913T,C3037T,C3267T,C5388A,C5986T,T6954C,C14408T,C14676T,C15279T,T16176C,A23063T,C23271A,A23403G,C23604A,C23709T,T24506G,G24914C,G25690T,C26936T,C27972T,G28048T,A28111G,G28280C,A28281T,T28282A,G28396T,G28881A,G28882A,G28883C,C28977T	20	N:D3L,N:R203K,N:G204R,N:S235F,ORF1a:T1001I,ORF1a:A1708D,ORF1a:I2230T,ORF1b:P314L,ORF3a:G100C,ORF8:Q27*,ORF8:R52I,ORF8:Y73C,ORF9b:G38V,S:N501Y,S:A570D,S:D614G,S:P681H,S:T716I,S:S982A,S:D1118H	6	ORF1a:S3675	3
hCoV-19/Mexico/BCN-IBT_IMSS_2033/2021	EPI_ISL_2801878	OL461236	B.1.1.7	GRY	39	C241T,C913T,C2110T,C3037T,C3267T,C5388A,C5986T,T6954C,G7042T,A10195G,C10279T,C14120T,C14408T,C14676T,C15279T,T16176C,C21588T,A23063T,C23271A,A23403G,C23604A,C23709T,T24506G,T24565C,G24914C,C27972T,G28048T,G28075T,A28095T,A28111G,G28280C,A28281T,T28282A,A28877T,G28878C,G28881A,G28882A,G28883C,C28977T	23	N:D3L,N:R203K,N:G204R,N:S235F,ORF1a:T1001I,ORF1a:A1708D,ORF1a:I2230T,ORF1a:M2259I,ORF1b:P218L,ORF1b:P314L,ORF8:Q27*,ORF8:R52I,ORF8:C61F,ORF8:K68*,ORF8:Y73C,S:P9L,S:N501Y,S:A570D,S:D614G,S:P681H,S:T716I,S:S982A,S:D1118H	6	ORF1a:S3675	15
hCoV-19/Mexico/BCN-LANGEBIO_IMSS_1438/2021	EPI_ISL_2942560	OL461237	Q.3	GRY	36	C913T,C3037T,C3267T,G5023A,C5388A,C5986T,T6954C,G10396T,C14408T,C14676T,C15279T,C15960T,T16176C,A17615G,G20962A,C21108T,T23031C,A23063T,C23271A,A23403G,C23604A,C23709T,T24506G,G24914C,G26730C,C27972T,G28048T,A28111G,G28280C,A28281T,T28282A,G28881A,G28882A,G28883C,C28977T,G29440T	24	M:V70L,N:D3L,N:R203K,N:G204R,N:S235F,N:Q389H,ORF1a:T1001I,ORF1a:M1586I,ORF1a:A1708D,ORF1a:I2230T,ORF1b:P314L,ORF1b:K1383R,ORF1b:D2499N,ORF8:Q27*,ORF8:R52I,ORF8:Y73C,S:F490S,S:N501Y,S:A570D,S:D614G,S:P681H,S:T716I,S:S982A,S:D1118H	6	ORF1a:S3675	110
hCoV-19/Mexico/BCN-LANGEBIO_IMSS_1439/2021	EPI_ISL_2942561	OL461238	B.1.1.7	GRY	36	C241T,C913T,C2453T,C3037T,C3267T,A4360G,C5388A,A5570G,C5986T,T6954C,C9611T,C14408T,C14676T,C15279T,T16176C,C21855T,G21974C,A23063T,C23271A,A23403G,C23604A,C23709T,T24506G,G24914C,G25273T,C27972T,G28048T,A28111G,G28280C,A28281T,T28282A,A28295G,G28881A,G28882A,G28883C,C28977T	25	N:D3L,N:N8D,N:R203K,N:G204R,N:S235F,ORF1a:L730F,ORF1a:T1001I,ORF1a:A1708D,ORF1a:M1769V,ORF1a:I2230T,ORF1a:L3116F,ORF1b:P314L,ORF8:Q27*,ORF8:R52I,ORF8:Y73C,S:S98F,S:D138H,S:N501Y,S:A570D,S:D614G,S:P681H,S:T716I,S:S982A,S:D1118H,S:M1237I	6	ORF1a:S3675	0
hCoV-19/Mexico/BCN-LANGEBIO_IMSS_1802/2021	EPI_ISL_2942877	OL461239	B.1.1.7	GRY	39	C241T,C913T,C3037T,C3267T,C5388A,C5986T,T6954C,C7299T,C9391T,C11750T,C14408T,C14676T,T15096C,C15279T,T16176C,A16227C,G17658T,A23063T,C23271A,A23403G,C23604A,C23709T,C23854T,T24506G,G24914C,G25489T,C27389T,G27613A,C27972T,G28048T,A28095T,A28111G,G28280C,A28281T,T28282A,G28881A,G28882A,G28883C,C28977T	24	N:D3L,N:R203K,N:G204R,N:S235F,ORF1a:T1001I,ORF1a:A1708D,ORF1a:I2230T,ORF1a:A2345V,ORF1a:L3829F,ORF1b:P314L,ORF1b:M1397I,ORF3a:A33S,ORF7a:V74I,ORF8:Q27*,ORF8:R52I,ORF8:K68*,ORF8:Y73C,S:N501Y,S:A570D,S:D614G,S:P681H,S:T716I,S:S982A,S:D1118H	6	ORF1a:S3675	1
hCoV-19/Mexico/BCS_IBT_IMSS_1673/2021	EPI_ISL_2681213	OL461240	B.1.1.7	GRY	40	C241T,C913T,C2110T,C2544T,C3037T,C3267T,C5388A,C5986T,T6954C,C11572T,C12809T,C14120T,C14408T,C14676T,C15279T,T16176C,G20247T,C21757T,A23063T,C23271A,A23403G,C23604A,C23709T,T24506G,G24914C,G25565T,C26322A,T26604C,T27940C,C27972T,G28048T,A28095T,A28111G,G28280C,A28281T,T28282A,G28881A,G28882A,G28883C,C28977T	27	E:F26L,M:F28L,N:D3L,N:R203K,N:G204R,N:S235F,ORF1a:T760I,ORF1a:T1001I,ORF1a:A1708D,ORF1a:I2230T,ORF1a:L4182F,ORF1b:P218L,ORF1b:P314L,ORF1b:M2260I,ORF3a:S58I,ORF8:F16S,ORF8:Q27*,ORF8:R52I,ORF8:K68*,ORF8:Y73C,S:N501Y,S:A570D,S:D614G,S:P681H,S:T716I,S:S982A,S:D1118H	6	ORF1a:S3675	60
hCoV-19/Mexico/BCS_INER_IMSS_00663/2021	EPI_ISL_1585658	OL461241	B.1.1.7	GRY	37	C241T,C913T,C2110T,C2544T,C3037T,C3267T,C5388A,C5986T,C6449T,T6954C,C12809T,C14120T,C14408T,C14676T,C15279T,T16176C,A23063T,C23271A,A23403G,C23604A,C23709T,T24506G,G24914C,C26322A,T26604C,T27940C,C27972T,G28048T,A28095T,A28111G,G28280C,A28281T,T28282A,G28881A,G28882A,G28883C,C28977T	26	E:F26L,M:F28L,N:D3L,N:R203K,N:G204R,N:S235F,ORF1a:T760I,ORF1a:T1001I,ORF1a:A1708D,ORF1a:L2062F,ORF1a:I2230T,ORF1a:L4182F,ORF1b:P218L,ORF1b:P314L,ORF8:F16S,ORF8:Q27*,ORF8:R52I,ORF8:K68*,ORF8:Y73C,S:N501Y,S:A570D,S:D614G,S:P681H,S:T716I,S:S982A,S:D1118H	6	ORF1a:S3675	0
hCoV-19/Mexico/BCS-IBT_IMSS_1951/2021	EPI_ISL_2801812	OL461242	B.1.1.7	GRY	36	T137C,C241T,C703T,C913T,C3037T,C3267T,C5388A,C5986T,T6954C,G7798T,C8739T,C14408T,C14676T,C15279T,T16176C,G19677T,A23063T,C23086T,C23271A,A23403G,C23604A,C23709T,T24506G,G24914C,C26833T,C27972T,G28048T,A28111G,G28280C,A28281T,T28282A,G28881A,G28882A,G28883C,C28977T,C29614T	22	M:A104V,N:D3L,N:R203K,N:G204R,N:S235F,ORF1a:T1001I,ORF1a:A1708D,ORF1a:I2230T,ORF1a:K2511N,ORF1a:T2825I,ORF1b:P314L,ORF1b:Q2070H,ORF8:Q27*,ORF8:R52I,ORF8:Y73C,S:N501Y,S:A570D,S:D614G,S:P681H,S:T716I,S:S982A,S:D1118H	6	ORF1a:S3675	0
hCoV-19/Mexico/BCS-IBT_IMSS_1970/2021	EPI_ISL_2801828	OL461243	B.1.1.7	GRY	35	T137C,C241T,C703T,C913T,C3037T,C3267T,C5388A,C5986T,T6954C,G7798T,C14408T,C14676T,C15279T,T16176C,G19677T,A23063T,C23086T,C23271A,A23403G,C23604A,C23709T,T24506G,G24914C,C26833T,C27972T,G28048T,A28111G,G28280C,A28281T,T28282A,G28881A,G28882A,G28883C,C28977T,C29614T	21	M:A104V,N:D3L,N:R203K,N:G204R,N:S235F,ORF1a:T1001I,ORF1a:A1708D,ORF1a:I2230T,ORF1a:K2511N,ORF1b:P314L,ORF1b:Q2070H,ORF8:Q27*,ORF8:R52I,ORF8:Y73C,S:N501Y,S:A570D,S:D614G,S:P681H,S:T716I,S:S982A,S:D1118H	6	ORF1a:S3675	637
hCoV-19/Mexico/CAM_IBT_IMSS_10304-NC/2021	EPI_ISL_2681370	OL461244	B.1.1.7	GRY	37	C222T,C241T,C913T,C3037T,C3267T,C5388A,C5986T,T6954C,C14120T,C14408T,C14676T,C15279T,T16176C,C17762T,A21555G,A23063T,C23271A,A23403G,C23604A,C23709T,T24506G,G24914C,T25180C,C25844T,C26270T,G27589T,C27972T,G28048T,A28095T,A28111G,G28280C,A28281T,T28282A,G28881A,G28882A,G28883C,C28977T	24	E:T9I,N:D3L,N:R203K,N:G204R,N:S235F,ORF1a:T1001I,ORF1a:A1708D,ORF1a:I2230T,ORF1b:P218L,ORF1b:P314L,ORF1b:A1432V,ORF3a:T151I,ORF7a:A66S,ORF8:Q27*,ORF8:R52I,ORF8:K68*,ORF8:Y73C,S:N501Y,S:A570D,S:D614G,S:P681H,S:T716I,S:S982A,S:D1118H	6	ORF1a:S3675	891
hCoV-19/Mexico/CAM_IBT_IMSS_1746/2021	EPI_ISL_2681118	OL461245	B.1.1.7	GRY	38	C241T,C913T,C2453T,C3037T,C3267T,C5388A,C5986T,T6954C,C9611T,C14408T,C14676T,C15279T,G16068T,T16176C,G17562T,C20930T,C21110T,C21855T,G21974C,A23063T,C23271A,A23403G,C23604A,C23709T,T24506G,G24914C,C27972T,G28048T,G28089T,A28111G,G28280C,A28281T,T28282A,A28295G,G28881A,G28882A,G28883C,C28977T	27	N:D3L,N:N8D,N:R203K,N:G204R,N:S235F,ORF1a:L730F,ORF1a:T1001I,ORF1a:A1708D,ORF1a:I2230T,ORF1a:L3116F,ORF1b:P314L,ORF1b:E867D,ORF1b:T2488M,ORF1b:T2548I,ORF8:Q27*,ORF8:R52I,ORF8:G66C,ORF8:Y73C,S:S98F,S:D138H,S:N501Y,S:A570D,S:D614G,S:P681H,S:T716I,S:S982A,S:D1118H	6	ORF1a:S3675	0
hCoV-19/Mexico/CAM_INER_IMSS_1117/2021	EPI_ISL_2490327	OL461246	B.1.1.7	GRY	38	C241T,C913T,C2453T,C3037T,C3267T,C5388A,C5986T,T6954C,C9611T,C12525T,C14408T,C14676T,C15279T,T16176C,A20129C,C20234T,C20930T,C21110T,C21855T,G21974C,A23063T,C23271A,A23403G,C23604A,C23709T,T24506G,G24914C,C27972T,G28048T,A28111G,G28280C,A28281T,T28282A,A28295G,G28881A,G28882A,G28883C,C28977T	28	N:D3L,N:N8D,N:R203K,N:G204R,N:S235F,ORF1a:L730F,ORF1a:T1001I,ORF1a:A1708D,ORF1a:I2230T,ORF1a:L3116F,ORF1a:T4087I,ORF1b:P314L,ORF1b:E2221A,ORF1b:P2256L,ORF1b:T2488M,ORF1b:T2548I,ORF8:Q27*,ORF8:R52I,ORF8:Y73C,S:S98F,S:D138H,S:N501Y,S:A570D,S:D614G,S:P681H,S:T716I,S:S982	6	ORF1a:S3675	0
hCoV-19/Mexico/CAM_INER_IMSS_1141/2021	EPI_ISL_2490328	OL461247	B.1.1.7	GRY	35	C241T,C913T,C2453T,C3037T,C3267T,C5388A,C5986T,T6954C,C9611T,C14408T,C14676T,C15279T,T16176C,C21855T,G21974C,A23063T,C23271A,A23403G,C23604A,C23709T,T24506G,G24914C,C25609T,C27972T,G28048T,A28111G,G28280C,A28281T,T28282A,A28295G,G28881A,G28882A,G28883C,C28940T,C28977T	24	N:D3L,N:N8D,N:R203K,N:G204R,N:S235F,ORF1a:L730F,ORF1a:T1001I,ORF1a:A1708D,ORF1a:I2230T,ORF1a:L3116F,ORF1b:P314L,ORF3a:L73F,ORF8:Q27*,ORF8:R52I,ORF8:Y73C,S:S98F,S:D138H,S:N501Y,S:A570D,S:D614G,S:P681H,S:T716I,S:S982A,S:D1118H	6	ORF1a:S3675	0
hCoV-19/Mexico/CAM_INER_IMSS_1151/2021	EPI_ISL_2490329	OL461248	B.1.1.7	GRY	33	C241T,C913T,C3037T,C3267T,C5388A,C5986T,T6954C,C14408T,C14676T,C15279T,T16176C,C19151T,A23063T,C23123A,C23271A,A23403G,C23604A,C23709T,T24506G,G24914C,C25339T,G26520T,C27972T,G28048T,A28111G,G28280C,A28281T,T28282A,G28881A,G28882A,G28883C,	20	N:D3L,N:R203K,N:G204R,N:S235F,ORF1a:T1001I,ORF1a:A1708D,ORF1a:I2230T,ORF1b:P314L,ORF1b:A1895V,ORF8:Q27*,ORF8:R52I,ORF8:Y73C,S:N501Y,S:P521T,S:A570D,S:D614G,S:P681H,S:T716I,S:S982A,S:D1118H	6	ORF1a:S3675	0
hCoV-19/Mexico/CAM_LANGEBIO_IMSS_0625/2021	EPI_ISL_2402042	OL461249	B.1.1.7	GRY	37	C241T,C913T,C3037T,C3267T,C5388A,C5986T,T6954C,C9693T,C11572T,C14408T,C14676T,C15279T,T16176C,G18651A,T23042C,A23063T,C23271A,A23403G,C23604A,C23709T,T24506G,G24914C,G25218T,C25553T,G25660C,C27615T,C27972T,G28048T,A28095T,A28111G,G28280C,A28281T,T28282A,G28881A,G28882A,G28883C,C28977T	24	N:D3L,N:R203K,N:G204R,N:S235F,ORF1a:T1001I,ORF1a:A1708D,ORF1a:I2230T,ORF1a:A3143V,ORF1b:P314L,ORF3a:A54V,ORF3a:V90L,ORF8:Q27*,ORF8:R52I,ORF8:K68*,ORF8:Y73C,S:S494P,S:N501Y,S:A570D,S:D614G,S:P681H,S:T716I,S:S982A,S:D1118H,S:G1219V	6	ORF1a:S3675	0
hCoV-19/Mexico/CAM_LANGEBIO_IMSS_08144-NC/2021	EPI_ISL_2969870	OL580731	B.1.1.7	GR	33	C241T,C913T,C2453T,C3037T,C3267T,C5388A,C5986T,T6954C,C9611T,G12514T,C14408T,C15279T,T16176C,G21452T,A23063T,C23271A,A23403G,C23604A,C23709T,T24506G,G24914C,C27972T,G28048T,A28111G,G28280C,A28281T,T28282A,A28295G,G28460T,G28881A,G28882A,G28883C,C28977T	24	N:D3L,N:N8D,N:D63Y,N:R203K,N:G204R,N:S235F,ORF1a:L730F,ORF1a:T1001I,ORF1a:A1708D,ORF1a:I2230T,ORF1a:L3116F,ORF1b:P314L,ORF1b:G2662V,ORF8:Q27*,ORF8:R52I,ORF8:Y73C,ORF9b:K59N,S:N501Y,S:A570D,S:D614G,S:P681H,S:T716I,S:S982A,S:D1118H	5	ORF1a:S3675	1602
hCoV-19/Mexico/CAM-IBT_IMSS_1865/2021	EPI_ISL_2801583	OL461250	B.1.1.7	GRY	32	C241T,C913T,C2453T,C3037T,C3267T,C5388A,C5934T,C5986T,T6954C,C9611T,C14408T,C14676T,C15279T,T16176C,A23063T,C23271A,A23403G,C23604A,C23709T,T24506G,G24914C,C27972T,G28048T,A28111G,G28280C,A28281T,T28282A,A28295G,G28881A,G28882A,G28883C,C2	22	N:D3L,N:N8D,N:R203K,N:G204R,N:S235F,ORF1a:L730F,ORF1a:T1001I,ORF1a:A1708D,ORF1a:T1890I,ORF1a:I2230T,ORF1a:L3116F,ORF1b:P314L,ORF8:Q27*,ORF8:R52I,ORF8:Y73C,S:N501Y,S:A570D,S:D614G,S:P681H,S:T716I,S:S982A,	6	ORF1a:S3675	15
hCoV-19/Mexico/CAM-IBT_IMSS_1876/2021	EPI_ISL_2801584	OL461251	B.1.1.7	GRY	37	C241T,C913T,C2453T,C3037T,C3267T,C5388A,C5986T,T6954C,C9611T,C14408T,C14676T,C15279T,G16068T,T16176C,G17562T,C20930T,C21110T,C21855T,A23063T,C23271A,A23403G,C23604A,C23709T,T24506G,G24914C,C27972T,G28048T,G28089T,A28111G,G28280C,A28281T,T28282A,A28295G,G28881A,G28882A,G28883C,C28977T	26	N:D3L,N:N8D,N:R203K,N:G204R,N:S235F,ORF1a:L730F,ORF1a:T1001I,ORF1a:A1708D,ORF1a:I2230T,ORF1a:L3116F,ORF1b:P314L,ORF1b:E867D,ORF1b:T2488M,ORF1b:T2548I,ORF8:Q27*,ORF8:R52I,ORF8:G66C,ORF8:Y73C,S:S98F,S:N501Y,S:A570D,S:D614G,S:P681H,S:T716I,S:S982A,S:D1118H	6	ORF1a:S3675	1

hCoV-19/Mexico/CAM-IBT_IMSS_2098/2021	EPI_ISL_2801645	OL461252	B.1.1.7	GRY	36	C241T,C913T,C2453T,C3037T,C3267T,C5388A,C5986T,C6033T,T6954C,C9611T,C14408T,C14676T,C15279T,T16176C,C21855T,G21974C,A23063T,C23271A,A23403G,C23604A,C23709T,T24506G,G24914C,C25609T,C27972T,G28048T,G28090T,A28111G,G28280C,A28281T,T28282A,A28295G,G28881A,G28882A,G28883C,C28977T	26	N:D3L,N:N8D,N:R203K,N:G204R,N:S235F,ORF1a:L730F,ORF1a:T1001I,ORF1a:A1708D,ORF1a:A1923V,ORF1a:I2230T,ORF1a:L3116F,ORF1b:P314L,ORF3a:L73F,ORF8:Q27*,ORF8:R52I,ORF8:G66V,ORF8:Y73C,S:S98F,S:D138H,S:N501Y,S:A570D,S:D614G,S:P681H,S:T716I,S:S982A,S:D1118H	6	ORF1a:S3675	1
hCoV-19/Mexico/CAM-IBT_IMSS_2102/2021	EPI_ISL_2801649	OL461253	B.1.1.7	GRY	39	C222T,C241T,C913T,C2110T,C3037T,C3267T,C4206T,C5388A,C5986T,T6954C,C7006T,G7042T,C14120T,C14408T,C14676T,C15279T,T16176C,C17762T,T23032G,A23063T,C23271A,A23403G,C23604A,C23709T,T24506G,G24914C,T25180C,C27972T,G28048T,A28095T,A28111G,G28280C,A28281T,T28282A,C28603T,G28881A,G28882A,G28883C,C28977T	24	N:D3L,N:R203K,N:G204R,N:S235F,ORF1a:T1001I,ORF1a:A1314V,ORF1a:A1708D,ORF1a:I2230T,ORF1a:M2259I,ORF1b:P218L,ORF1b:P314L,ORF1b:A1432V,ORF8:Q27*,ORF8:R52I,ORF8:K68*,ORF8:Y73C,S:F490L,S:N501Y,S:A570D,S:D614G,S:P681H,S:T716I,S:S982A,S:D1118H	6	ORF1a:S3675	201
hCoV-19/Mexico/CAM-LANGEBIO_IMSS_0940/2021	EPI_ISL_2671501	OL461254	B.1.1.7	GRY	42	C241T,C913T,C2453T,C3037T,C3267T,C4331T,C5388A,C5986T,T6954C,C9611T,C14408T,C14676T,C15279T,T16176C,G18225T,C20930T,C21110T,C21855T,G21974C,A23063T,C23271A,A23403G,C23604A,C23709T,T24506G,G24914C,G25244T,G25555T,T25706C,C26985T,C27972T,G28048T,A28111G,G28280C,A28281T,T28282A,A28295G,G28690T,G28881A,G28882A,G28883C,C28977T	31	M:H155Y,N:D3L,N:N8D,N:L139F,N:R203K,N:G204R,N:S235F,ORF1a:L730F,ORF1a:T1001I,ORF1a:A1708D,ORF1a:I2230T,ORF1a:L3116F,ORF1b:P314L,ORF1b:M1586I,ORF1b:T2488M,ORF1b:T2548I,ORF3a:V55F,ORF3a:F105S,ORF8:Q27*,ORF8:R52I,ORF8:Y73C,S:S98F,S:D138H,S:N501Y,S:A570D,S:D614G,S:P681H,S:T716I,S:S982A,S:D1118H,S:V1228L	6	ORF1a:S3675	0
hCoV-19/Mexico/CAM-LANGEBIO_IMSS_1190/2021	EPI_ISL_2942351	OL461255	B.1.1.7	GRY	40	C875T,C913T,G942A,C2110T,C3037T,C3267T,C5388A,C5986T,C6016T,T6954C,G7042T,A10195G,C10279T,C14120T,C14408T,C14676T,C15279T,T16176C,C16887T,G17427T,C21682T,A23063T,C23271A,A23403G,C23604A,C23709T,T24506G,G24914C,C27972T,G28048T,G28075T,A28111G,G28280C,A28281T,T28282A,G28881A,G28882A,G28883C,C28896T,C28977T	24	N:D3L,N:R203K,N:G204R,N:A208V,N:S235F,ORF1a:L204F,ORF1a:R226K,ORF1a:T1001I,ORF1a:A1708D,ORF1a:I2230T,ORF1a:M2259I,ORF1b:P218L,ORF1b:P314L,ORF8:Q27*,ORF8:R52I,ORF8:C61F,ORF8:Y73C,S:N501Y,S:A570D,S:D614G,S:P681H,S:T716I,S:S982A,S:D1118H	6	ORF1a:S3675	1
hCoV-19/Mexico/CAM-LANGEBIO_IMSS_1205/2021	EPI_ISL_2942366	OL580732	B.1.1.7	GRY	40	C913T,A1650C,C2453T,C2523T,C3037T,C3267T,C4331T,C5388A,C5986T,T6954C,G8179T,C9611T,C14408T,C14676T,C15279T,T16176C,C20930T,C21077T,C21110T,C21855T,G21974C,A22011G,A23063T,C23271A,A23403G,C23604A,C23709T,T24506G,G24914C,C27972T,G28048T,A28111G,G28280C,A28281T,T28282A,A28295G,G28881A,G28882A,G28883C,C28977T	29	N:D3L,N:N8D,N:R203K,N:G204R,N:S235F,ORF1a:N462T,ORF1a:L730F,ORF1a:T753I,ORF1a:T1001I,ORF1a:A1708D,ORF1a:I2230T,ORF1a:L3116F,ORF1b:P314L,ORF1b:T2488M,ORF1b:T2537I,ORF1b:T2548I,ORF8:Q27*,ORF8:R52I,ORF8:Y73C,S:S98F,S:D138H,S:K150R,S:N501Y,S:A570D,S:D614G,S:P681H,S:T716I,S:S	6	ORF1a:S3675	653
hCoV-19/Mexico/CAM-LANGEBIO_IMSS_1207/2021	EPI_ISL_2942368	OL461256	B.1.1.7	GRY	38	C913T,A1650C,C2453T,C3037T,C3267T,C4331T,C5388A,C5986T,T6954C,G8179T,C9611T,C14408T,C14676T,C15279T,T16176C,C20930T,C21077T,C21110T,C21855T,G21974C,A23063T,C23271A,A23403G,C23604A,C23709T,T24506G,G24914C,C27972T,G28048T,A28111G,G28280C,A28281T,T28282A,A28295G,G28881A,G28882A,G28883C,C28977T	27	N:D3L,N:N8D,N:R203K,N:G204R,N:S235F,ORF1a:N462T,ORF1a:L730F,ORF1a:T1001I,ORF1a:A1708D,ORF1a:I2230T,ORF1a:L3116F,ORF1b:P314L,ORF1b:T2488M,ORF1b:T2537I,ORF1b:T2548I,ORF8:Q27*,ORF8:R52I,ORF8:Y73C,S:S98F,S:D138H,S:N501Y,S:A570D,S:D614G,S:P681H,S:T716I,S:S982A,S:D1118H	6	ORF1a:S3675	815
hCoV-19/Mexico/CAM-LANGEBIO_IMSS_1228/2021	EPI_ISL_2942389	OL461257	B.1.1.7	GRY	45	C241T,C913T,C2110T,G3004T,C3037T,C3267T,C5388A,C5986T,T6954C,G7042T,C7765T,C8078T,C10789T,C14120T,C14408T,C14676T,G14857T,C15279T,G15672C,C15810T,T16176C,G20679T,T22219C,G22567T,A23063T,C23271A,A23403G,C23604A,C23709T,T24506G,G24914C,C25731T,C26110T,C26735T,C27972T,G28048T,A28111G,G28280C,A28281T,T28282A,A28804G,G28881	26	N:D3L,N:R203K,N:G204R,N:S235F,ORF1a:E913D,ORF1a:T1001I,ORF1a:A1708D,ORF1a:I2230T,ORF1a:M2259I,ORF1a:P2605S,ORF1b:P218L,ORF1b:P314L,ORF1b:V464F,ORF1b:E735D,ORF3a:P240S,ORF8:Q27*,ORF8:R52I,ORF8:Y73C,S:L335F,S:N501Y,S:A570D,S:D614G,S:P681H,S:T716I,S:S982A,S:D1118H	6	ORF1a:S3675	11
hCoV-19/Mexico/CAM-LANGEBIO_IMSS_1231/2021	EPI_ISL_2942392	OL461258	B.1.1.7	GRY	41	C241T,C913T,A1650C,C2453T,C2523T,C3037T,C3267T,C4331T,C5388A,C5986T,T6954C,G8179T,C9611T,C14408T,C14676T,C15279T,T16176C,C20930T,C21077T,C21110T,C21855T,G21974C,A22011G,A23063T,C23271A,A23403G,C23604A,C23709T,T24506G,G24914C,C27972T,G28048T,A28111G,G28280C,A28281T,T28282A,A28295G,G28881A,G28882A,G28883C,C28977T	29	N:D3L,N:N8D,N:R203K,N:G204R,N:S235F,ORF1a:N462T,ORF1a:L730F,ORF1a:T753I,ORF1a:T1001I,ORF1a:A1708D,ORF1a:I2230T,ORF1a:L3116F,ORF1b:P314L,ORF1b:T2488M,ORF1b:T2537I,ORF1b:T2548I,ORF8:Q27*,ORF8:R52I,ORF8:Y73C,S:S98F,S:D138H,S:K150R,S:N501Y,S:A570D,S:D614G,S:P681H,S:T716I,S:S	6	ORF1a:S3675	2
hCoV-19/Mexico/CAM-LANGEBIO_IMSS_1548/2021	EPI_ISL_2942659	OL461259	B.1.1.7	GRY	39	C241T,C913T,G1820A,C2453T,C3037T,C3267T,C5388A,C5986T,T6954C,C9611T,C14408T,C14676T,C15279T,G16068T,T16176C,G17562T,C20930T,C21110T,C21855T,G21974C,A23063T,C23271A,A23403G,C23604A,C23709T,T24506G,G24914C,C27972T,G28048T,G28089T,A28111G,G28280C,A28281T,T28282A,A28295G,G28881A,G28882A,G28883C,C28977T	28	N:D3L,N:N8D,N:R203K,N:G204R,N:S235F,ORF1a:G519S,ORF1a:L730F,ORF1a:T1001I,ORF1a:A1708D,ORF1a:I2230T,ORF1a:L3116F,ORF1b:P314L,ORF1b:E867D,ORF1b:T2488M,ORF1b:T2548I,ORF8:Q27*,ORF8:R52I,ORF8:G66C,ORF8:Y73C,S:S98F,S:D138H,S:N501Y,S:A570D,S:D614G,S:P681H,S:T716I,S:S982A,S:D1	6	ORF1a:S3675	0
hCoV-19/Mexico/CAM-LANGEBIO_IMSS_1571/2021	EPI_ISL_2942682	OL461260	B.1.1.7	GRY	39	C241T,C913T,C2110T,C3037T,C3267T,C5388A,C5986T,C6016T,T6954C,G7042T,A10195G,C10279T,C14120T,C14408T,C14676T,C15279T,T16176C,C16887T,G17427T,G22249T,A23063T,C23271A,A23403G,C23604A,C23709T,T24506G,G24914C,C27972T,G28048T,G28075T,A28095T,A28111G,G28280C,A28281T,T28282A,G28881A,G28882A,G28883C,C28977T	23	N:D3L,N:R203K,N:G204R,N:S235F,ORF1a:T1001I,ORF1a:A1708D,ORF1a:I2230T,ORF1a:M2259I,ORF1b:P218L,ORF1b:P314L,ORF8:Q27*,ORF8:R52I,ORF8:C61F,ORF8:K68*,ORF8:Y73C,S:L229F,S:N501Y,S:A570D,S:D614G,S:P681H,S:T716I,S:S982A,S:D1118H	6	ORF1a:S3675	0
hCoV-19/Mexico/CHH_IBT_IMSS_1367/2021	EPI_ISL_2391727	OL461261	B.1.1.7	GRY	31	C241T,C913T,C3037T,C3267T,C5388A,C5986T,C6594T,T6954C,C11750T,C14408T,C14676T,C15279T,T16176C,A23063T,C23271A,A23403G,C23604A,C23709T,T24506G,G24914C,C26270T,C27972T,G28048T,A28111G,G28280C,A28281T,T28282A,G28881A,G28882A,G28883C,C28977T	21	E:T9I,N:D3L,N:R203K,N:G204R,N:S235F,ORF1a:T1001I,ORF1a:A1708D,ORF1a:P2110L,ORF1a:I2230T,ORF1a:L3829F,ORF1b:P314L,ORF8:Q27*,ORF8:R52I,ORF8:Y73C,S:N501Y,S:A570D,S:D614G,S:P681H,S:T716I,S:S982A,S:D1118H	6	ORF1a:S3675	740
hCoV-19/Mexico/CHH_IBT_IMSS_1370/2021	EPI_ISL_2391639	OL461262	B.1.1.7	GRY	29	C241T,C913T,C3037T,C3267T,C5388A,C5986T,T6954C,C14408T,C14676T,C15279T,T16176C,A23063T,C23271A,A23403G,C23604A,C23709T,T24506G,G24914C,C27002T,C27972T,G28048T,A28111G,G28280C,A28281T,T28282A,G28881A,G28882A,G28883C,C28977T	18	N:D3L,N:R203K,N:G204R,N:S235F,ORF1a:T1001I,ORF1a:A1708D,ORF1a:I2230T,ORF1b:P314L,ORF8:Q27*,ORF8:R52I,ORF8:Y73C,S:N501Y,S:A570D,S:D614G,S:P681H,S:T716I,S:S982A,S:D1118H	6	ORF1a:S3675	354
hCoV-19/Mexico/CHH_IBT_IMSS_1423/2021	EPI_ISL_2681357	OL461263	B.1.1.7	GRY	35	C100T,C241T,C913T,C3037T,C3267T,A4552G,C5388A,C5986T,T6954C,G11273A,C14408T,C14649T,C14676T,C15279T,T16176C,C18348T,C22227T,A23063T,C23271A,A23403G,C23604A,C23709T,C24106T,T24506G,G24914C,C27972T,G28048T,A28111G,G28280C,A28281T,T28282A,G28881A,G28882A,G28883C,C28977T	20	N:D3L,N:R203K,N:G204R,N:S235F,ORF1a:T1001I,ORF1a:A1708D,ORF1a:I2230T,ORF1a:V3670I,ORF1b:P314L,ORF8:Q27*,ORF8:R52I,ORF8:Y73C,S:A222V,S:N501Y,S:A570D,S:D614G,S:P681H,S:T716I,S:S982A,S:D1118H	6	ORF1a:S3675	783
hCoV-19/Mexico/CHH_IBT_IMSS_1429/2021	EPI_ISL_2681316	OL461264	B.1.1.7	GRY	33	C241T,C913T,C3037T,C3267T,C5388A,C5986T,T6954C,G13045T,C14408T,C14676T,C15279T,T16176C,C21622T,A23063T,C23271A,A23403G,C23604A,C23709T,C24381T,T24506G,G24914C,C27972T,G28048T,A28111G,T28130C,G28280C,A28281T,T28282A,G28302A,G28881A,G28882A,	21	N:D3L,N:R10Q,N:R203K,N:G204R,N:S235F,ORF1a:T1001I,ORF1a:A1708D,ORF1a:I2230T,ORF1b:P314L,ORF8:Q27*,ORF8:R52I,ORF8:Y73C,ORF9b:E7K,S:N501Y,S:A570D,S:D614G,S:P681H,S:T716I,S:S940F,S:S982A,S:D1118H	6	ORF1a:S3675	483
hCoV-19/Mexico/CHH_IBT_IMSS_1430/2021	EPI_ISL_2681289	OL461265	B.1.1.7	GRY	33	C241T,C913T,C3037T,C3267T,C5388A,C5986T,T6954C,C9298T,C14408T,C14676T,C15279T,T16176C,C19018T,C21618T,A23063T,C23271A,A23403G,C23604A,C23709T,C24374T,T24506G,G24914C,C27972T,G28048T,A28111G,G28280C,A28281T,T28282A,G28881A,G28882A,G28883C,C	22	N:D3L,N:R203K,N:G204R,N:S235F,N:A359S,ORF1a:T1001I,ORF1a:A1708D,ORF1a:I2230T,ORF1b:P314L,ORF1b:P1851S,ORF8:Q27*,ORF8:R52I,ORF8:Y73C,S:T19I,S:N501Y,S:A570D,S:D614G,S:P681H,S:T716I,S:L938F,S:S982A,S:D1118H	6	ORF1a:S3675	291
hCoV-19/Mexico/CHH_IBT_IMSS_1431/2021	EPI_ISL_2681359	OL461266	B.1.1.7	GRY	33	C241T,C913T,C3037T,C3267T,C5388A,C5986T,T6954C,C14408T,C14676T,C15279T,C15656T,T16176C,G20580T,A23063T,C23271A,A23403G,C23604A,C23709T,T24506G,G24914C,T25530C,C26833T,C27732T,C27972T,G28048T,A28111G,G28280C,A28281T,T28282A,G28881A,G28882A,G	20	M:A104V,N:D3L,N:R203K,N:G204R,N:S235F,ORF1a:T1001I,ORF1a:A1708D,ORF1a:I2230T,ORF1b:P314L,ORF1b:T730I,ORF8:Q27*,ORF8:R52I,ORF8:Y73C,S:N501Y,S:A570D,S:D614G,S:P681H,S:T716I,S:S982A,S:D1118H	6	ORF1a:S3675	742
hCoV-19/Mexico/CHH_IBT_IMSS_1432/2021	EPI_ISL_2681137	OL461267	B.1.1.7	GRY	30	C241T,C913T,C3037T,C3267T,A3829G,C5388A,C5986T,T6954C,C14408T,C14676T,C15279T,T16176C,A23063T,C23271A,A23403G,C23604A,C23709T,T24506G,G24914C,C27972T,G28048T,A28111G,G28280C,A28281T,T28282A,G28881A,G28882A,G28883C,C28977T,C29580T	18	N:D3L,N:R203K,N:G204R,N:S235F,ORF1a:T1001I,ORF1a:A1708D,ORF1a:I2230T,ORF1b:P314L,ORF8:Q27*,ORF8:R52I,ORF8:Y73C,S:N501Y,S:A570D,S:D614G,S:P681H,S:T716I,S:S982A,S:D1118H	6	ORF1a:S3675	0
hCoV-19/Mexico/CHH_IBT_IMSS_1434/2021	EPI_ISL_2681103	OL461268	B.1.1.7	GRY	33	C241T,C913T,C3037T,C3267T,A3492G,C5388A,C5986T,T6954C,T12283C,C14408T,C14676T,C15279T,T16176C,C21639A,A23063T,C23271A,A23403G,C23604A,C23709T,C24374T,T24506G,G24914C,C27972T,G28048T,A28111G,G28280C,A28281T,T28282A,C28453T,G28881A,G28882A,G	22	N:D3L,N:R203K,N:G204R,N:S235F,ORF1a:T1001I,ORF1a:N1076S,ORF1a:A1708D,ORF1a:I2230T,ORF1b:P314L,ORF8:Q27*,ORF8:R52I,ORF8:Y73C,ORF9b:A57V,S:P26H,S:N501Y,S:A570D,S:D614G,S:P681H,S:T716I,S:L938F,S:S982A,S:D11	6	ORF1a:S3675	2
hCoV-19/Mexico/CHH_IBT_IMSS_1435/2021	EPI_ISL_2681265	OL461269	B.1.1.7	GRY	31	C241T,C913T,C3037T,C3267T,C5388A,C5986T,C6638T,T6954C,C12559T,C14408T,C14676T,C15279T,T16176C,C21622T,A23063T,C23271A,A23403G,C23604A,C23709T,T24506G,G24914C,C27972T,G28048T,A28111G,G28280C,A28281T,T28282A,G28881A,G28882A,G28883C,C28977T	19	N:D3L,N:R203K,N:G204R,N:S235F,ORF1a:T1001I,ORF1a:A1708D,ORF1a:H2125Y,ORF1a:I2230T,ORF1b:P314L,ORF8:Q27*,ORF8:R52I,ORF8:Y73C,S:N501Y,S:A570D,S:D614G,S:P681H,S:T716I,S:S982A,S:D1118H	6	ORF1a:S3675	293
hCoV-19/Mexico/CHH_IBT_IMSS_1436/2021	EPI_ISL_2681277	OL461270	B.1.1.7	GRY	35	C241T,C913T,C3037T,C3267T,G5164T,C5388A,C5986T,T6954C,C7945T,C14408T,C14676T,C15279T,T16176C,A23063T,C23191T,C23271A,A23403G,C23604A,C23709T,T24506G,G24914C,G25494A,C25710T,C27213T,C27294T,C27972T,G28048T,A28111G,G28280C,A28281T,T28282A,G28881A,G28882A,G28883C,C28977T	19	N:D3L,N:R203K,N:G204R,N:S235F,ORF1a:T1001I,ORF1a:E1633D,ORF1a:A1708D,ORF1a:I2230T,ORF1b:P314L,ORF8:Q27*,ORF8:R52I,ORF8:Y73C,S:N501Y,S:A570D,S:D614G,S:P681H,S:T716I,S:S982A,S:D1118H	6	ORF1a:S3675	402
hCoV-19/Mexico/CHH_IBT_IMSS_1442/2021	EPI_ISL_2681248	OL461271	B.1.1.7	GRY	41	C241T,C913T,C2110T,C3037T,C3267T,G4255T,C5388A,G5578A,C5986T,C6040T,T6954C,C9598T,C12525T,C13426T,C14120T,C14408T,C14676T,C15279T,T16176C,C21575T,G22225T,A23063T,C23271A,A23403G,C23604A,C23709T,T24506G,G24914C,T27462C,T27768G,C27972T,G28048T,A28095T,A28111G,G28280C,A28281T,T28282A,G28881A,G28882A,G28883C,C28977T	25	N:D3L,N:R203K,N:G204R,N:S235F,ORF1a:T1001I,ORF1a:A1708D,ORF1a:M1771I,ORF1a:I2230T,ORF1a:T4087I,ORF1b:P218L,ORF1b:P314L,ORF7a:S98A,ORF7b:S5A,ORF8:Q27*,ORF8:R52I,ORF8:K68*,ORF8:Y73C,S:L5F,S:N501Y,S:A570D,S:D614G,S:P681H,S:T716I,S:S982A,S:D1118H	9	ORF1a:S3675	291

hCoV-19/Mexico/CHH_IBT_IMSS_1443/2021	EPI_ISL_2681219	OL461272	B.1.1.7	GRY	38	A156G,C241T,C913T,C3037T,C3267T,C5388A,C5986T,T6954C,G7042T,C8502T,G9929A,A10195G,C14120T,C14408T,C14676T,C15279T,T16176C,C19011T,A23063T,C23271A,A23403G,C23604A,C23709T,T24506G,G24914C,C27972T,G28048T,G28075T,A28095T,A28111G,G28280C,A28281T,T28282A,T28688C,G28881A,G28882A,G28883C,C28977T	24	N:D3L,N:R203K,N:G204R,N:S235F,ORF1a:T1001I,ORF1a:A1708D,ORF1a:I2230T,ORF1a:M2259I,ORF1a:T2746I,ORF1a:D3222N,ORF1b:P218L,ORF1b:P314L,ORF8:Q27*,ORF8:R52I,ORF8:C61F,ORF8:K68*,ORF8:Y73C,S:N501Y,S:A570D,S:D614G,S:P681H,S:T716I,S:S982A,S:D1118H	6	ORF1a:S367	211
hCoV-19/Mexico/CHH_IBT_IMSS_1444/2021	EPI_ISL_2681104	OL461273	B.1.1.7	GRY	39	A156G,C241T,C913T,C2110T,C3037T,C3267T,C5388A,C5986T,T6954C,G7042T,C8502T,G9929A,A10195G,C14120T,C14408T,C14676T,C15279T,T16176C,C19011T,A23063T,C23271A,A23403G,C23604A,C23709T,T24506G,G24914C,C27972T,G28048T,G28075T,A28095T,A28111G,G28280C,A28281T,T28282A,T28688C,G28881A,G28882A,G28883C,C28977T	24	N:D3L,N:R203K,N:G204R,N:S235F,ORF1a:T1001I,ORF1a:A1708D,ORF1a:I2230T,ORF1a:M2259I,ORF1a:T2746I,ORF1a:D3222N,ORF1b:P218L,ORF1b:P314L,ORF8:Q27*,ORF8:R52I,ORF8:C61F,ORF8:K68*,ORF8:Y73C,S:N501Y,S:A570D,S:D614G,S:P681H,S:T716I,S:S982A,S:D1118H	6	ORF1a:S367	0
hCoV-19/Mexico/CHH_INER_IMSS_00382/2021	EPI_ISL_1585392	OL461274	B.1.1.7	GRY	30	C241T,C913T,C3037T,C3267T,C5388A,C5986T,T6954C,C11750T,C14408T,C14676T,C15279T,T16176C,A23063T,C23271A,A23403G,C23604A,C23709T,T24506G,G24914C,C26270T,C27972T,G28048T,A28111G,G28280C,A28281T,T28282A,G28881A,G28882A,G28883C,C28977T	20	E:T9I,N:D3L,N:R203K,N:G204R,N:S235F,ORF1a:T1001I,ORF1a:A1708D,ORF1a:I2230T,ORF1a:L3829F,ORF1b:P314L,ORF8:Q27*,ORF8:R52I,ORF8:Y73C,S:N501Y,S:A570D,S:D614G,S:P681H,S:T716I,S:S982A,S:D1118H	6	ORF1a:S367	0
hCoV-19/Mexico/CHH_INER_IMSS_00383/2021	EPI_ISL_1585393	OL461275	B.1.1.7	GRY	32	C241T,C913T,G2072T,C3037T,C3267T,C5388A,C5986T,T6954C,C9298T,C14408T,C14676T,C15279T,C15895T,T16176C,A23063T,C23271A,A23403G,C23604A,C23709T,C24374T,T24506G,G24914C,C27972T,G28048T,A28111G,G28280C,A28281T,T28282A,G28881A,G28882A,G28883C,C2	20	N:D3L,N:R203K,N:G204R,N:S235F,ORF1a:G603C,ORF1a:T1001I,ORF1a:A1708D,ORF1a:I2230T,ORF1b:P314L,ORF8:Q27*,ORF8:R52I,ORF8:Y73C,S:N501Y,S:A570D,S:D614G,S:P681H,S:T716I,S:L938F,S:S982A,S:D1118H	6	ORF1a:S367	0
hCoV-19/Mexico/CHH_INER_IMSS_00384/2021	EPI_ISL_1585394	OL461276	B.1.1.7	GRY	32	C241T,C913T,C3037T,C3267T,T4057C,C5388A,C5986T,T6954C,C11750T,A13416G,C14408T,C14676T,C15279T,T16176C,A23063T,C23271A,A23403G,C23604A,C23709T,T24506G,G24914C,C26270T,C27972T,G28048T,A28111G,G28280C,A28281T,T28282A,G28881A,G28882A,G28883C,C	21	E:T9I,N:D3L,N:R203K,N:G204R,N:S235F,ORF1a:T1001I,ORF1a:A1708D,ORF1a:I2230T,ORF1a:L3829F,ORF1a:D4384G,ORF1b:P314L,ORF8:Q27*,ORF8:R52I,ORF8:Y73C,S:N501Y,S:A570D,S:D614G,S:P681H,S:T716I,S:S982A,S:D1118H	6	ORF1a:S367	0
hCoV-19/Mexico/CHH_INER_IMSS_00390/2021	EPI_ISL_1585400	OL461277	B.1.1.7	GRY	30	C241T,C913T,C3037T,C3267T,C5388A,C5986T,A6825G,T6954C,C14408T,C14676T,C15279T,T16176C,A23063T,C23271A,A23403G,C23604A,C23709T,T24506G,G24914C,C27972T,G28048T,A28111G,G28280C,A28281T,T28282A,G28881A,G28882A,G28883C,C28977T,C29774T	19	N:D3L,N:R203K,N:G204R,N:S235F,ORF1a:T1001I,ORF1a:A1708D,ORF1a:N2187S,ORF1a:I2230T,ORF1b:P314L,ORF8:Q27*,ORF8:R52I,ORF8:Y73C,S:N501Y,S:A570D,S:D614G,S:P681H,S:T716I,S:S982A,S:D1118H	6	ORF1a:S367	0
hCoV-19/Mexico/CHH_INER_IMSS_00743/2021	EPI_ISL_2091198	OL461278	B.1.1.7	GRY	29	C241T,C913T,C3037T,C3267T,G5164T,C5388A,C5986T,T6954C,C14408T,C14676T,C15279T,T16176C,A23063T,C23271A,A23403G,C23604A,C23709T,T24506G,G24914C,C27972T,G28048T,A28111G,G28280C,A28281T,T28282A,G28881A,G28882A,G28883C,C28977T	19	N:D3L,N:R203K,N:G204R,N:S235F,ORF1a:T1001I,ORF1a:E1633D,ORF1a:A1708D,ORF1a:I2230T,ORF1b:P314L,ORF8:Q27*,ORF8:R52I,ORF8:Y73C,S:N501Y,S:A570D,S:D614G,S:P681H,S:T716I,S:S982A,S:D1118H	6	ORF1a:S367	0
hCoV-19/Mexico/CHH_INER_IMSS_1042/2021	EPI_ISL_2490312	OL461279	B.1.1.7	GRY	31	C241T,C913T,C3037T,C3096T,C3267T,C5388A,C5986T,T6954C,C9298T,C14408T,C14676T,C15279T,T16176C,A23063T,C23271A,A23403G,C23604A,C23709T,C24374T,T24506G,G24914C,C27972T,G28048T,A28111G,G28280C,A28281T,T28282A,G28881A,G28882A,G28883C,C28977T	20	N:D3L,N:R203K,N:G204R,N:S235F,ORF1a:S944L,ORF1a:T1001I,ORF1a:A1708D,ORF1a:I2230T,ORF1b:P314L,ORF8:Q27*,ORF8:R52I,ORF8:Y73C,S:N501Y,S:A570D,S:D614G,S:P681H,S:T716I,S:L938F,S:S982A,S:D1118H	6	ORF1a:S367	0
hCoV-19/Mexico/CHH_INER_IMSS_1044/2021	EPI_ISL_2490338	OL461280	B.1.1.7	GRY	41	C241T,C913T,C934T,C2110T,C3037T,C3267T,C4206T,C5388A,C5986T,T6954C,A9274G,C9598T,T10913C,C12525T,C13426T,C14120T,C14408T,C14676T,C15279T,T16176C,C21575T,A23063T,C23271A,A23403G,C23604A,C23709T,T24506G,G24914C,T27384C,T27462C,C27972T,G28048T,A28095T,A28111G,G28280C,A28281T,T28282A,G28881A,G28882A,G28883C,C28977T	23	N:D3L,N:R203K,N:G204R,N:S235F,ORF1a:T1001I,ORF1a:A1314V,ORF1a:A1708D,ORF1a:I2230T,ORF1a:T4087I,ORF1b:P218L,ORF1b:P314L,ORF8:Q27*,ORF8:R52I,ORF8:K68*,ORF8:Y73C,S:L5F,S:N501Y,S:A570D,S:D614G,S:P681H,S:T716I,S:S982A,S:D1118H	6	ORF1a:S367	0
hCoV-19/Mexico/CHH_INER_IMSS_1055/2021	EPI_ISL_2490314	OL461281	B.1.1.7	GRY	32	C241T,C913T,C3037T,C3267T,C5388A,C5986T,T6954C,T7098C,C9298T,C14408T,C14676T,C15279T,T16176C,A23063T,C23271A,A23403G,C23604A,C23709T,C24374T,T24506G,G24914C,C27972T,G28048T,A28111G,T28150C,G28280C,A28281T,T28282A,G28881A,G28882A,G28883C,C2	21	N:D3L,N:R203K,N:G204R,N:S235F,ORF1a:T1001I,ORF1a:A1708D,ORF1a:I2230T,ORF1a:I2278T,ORF1b:P314L,ORF8:Q27*,ORF8:R52I,ORF8:Y73C,ORF8:F86S,S:N501Y,S:A570D,S:D614G,S:P681H,S:T716I,S:L938F,S:S982A,S:D1118H	6	ORF1a:S367	0
hCoV-19/Mexico/CHH_INER_IMSS_1060/2021	EPI_ISL_2490317	OL461282	B.1.1.7	GRY	34	C241T,C913T,G2305T,C3037T,C3267T,C5388A,C5986T,T6954C,G8726A,C14408T,C14676T,C14925T,C15279T,T16176C,G19962T,G20709T,A23063T,C23271A,A23403G,C23604A,C23709T,T24506G,G24914C,C27294T,C27972T,G28048T,A28111G,G28280C,A28281T,T28282A,G28881A,G2	21	N:D3L,N:R203K,N:G204R,N:S235F,ORF1a:K680N,ORF1a:T1001I,ORF1a:A1708D,ORF1a:I2230T,ORF1a:A2821T,ORF1b:P314L,ORF1b:M2414I,ORF8:Q27*,ORF8:R52I,ORF8:Y73C,S:N501Y,S:A570D,S:D614G,S:P681H,S:T716I,S:S982A,S:D11	6	ORF1a:S367	0
hCoV-19/Mexico/CHH_INER_IMSS_1061/2021	EPI_ISL_2490318	OL461283	B.1.1.7	GRY	32	C241T,C913T,C2695T,C3037T,C3267T,C5388A,C5986T,T6954C,C14408T,C14649T,C14676T,C15279T,T16176C,C18348T,C22687T,A23063T,C23271A,A23403G,C23604A,C23709T,T24506G,G24914C,C27972T,G28048T,A28111G,G28280C,A28281T,T28282A,G28881A,G28882A,G28883C,C	18	N:D3L,N:R203K,N:G204R,N:S235F,ORF1a:T1001I,ORF1a:A1708D,ORF1a:I2230T,ORF1b:P314L,ORF8:Q27*,ORF8:R52I,ORF8:Y73C,S:N501Y,S:A570D,S:D614G,S:P681H,S:T716I,S:S982A,S:D1118H	6	ORF1a:S367	0
hCoV-19/Mexico/CHH_INER_IMSS_1062/2021	EPI_ISL_2490319	OL461284	B.1.1.7	GRY	32	C241T,G271T,C913T,C3037T,C3267T,C5388A,C5986T,T6954C,C12854T,C14408T,C14676T,C15279T,T16176C,C21639A,A23063T,C23271A,A23403G,C23604A,C23709T,C24374T,T24506G,G24914C,C27972T,G28048T,A28111G,G28280C,A28281T,T28282A,G28881A,G28882A,G28883C,C	22	N:D3L,N:R203K,N:G204R,N:S235F,ORF1a:E2D,ORF1a:T1001I,ORF1a:A1708D,ORF1a:I2230T,ORF1a:P4197S,ORF1b:P314L,ORF8:Q27*,ORF8:R52I,ORF8:Y73C,S:P26H,S:N501Y,S:A570D,S:D614G,S:P681H,S:T716I,S:L938F,S:S982A,S:D111	6	ORF1a:S367	0
hCoV-19/Mexico/CHH_INER_IMSS_1078/2021	EPI_ISL_2490321	OL461285	B.1.1.7	GRY	32	C241T,C913T,C3037T,C3267T,C5388A,C5986T,T6954C,C9298T,C14408T,C14676T,C15279T,T16176C,A23063T,C23271A,A23403G,C23604A,C23709T,C24374T,T24506G,G24914C,G25217T,G27516T,C27972T,G28048T,A28111G,G28280C,A28281T,T28282A,G28881A,G28882A,G28883C,C2	21	N:D3L,N:R203K,N:G204R,N:S235F,ORF1a:T1001I,ORF1a:A1708D,ORF1a:I2230T,ORF1b:P314L,ORF7a:E41D,ORF8:Q27*,ORF8:R52I,ORF8:Y73C,S:N501Y,S:A570D,S:D614G,S:P681H,S:T716I,S:L938F,S:S982A,S:D1118H,S:G1219C	6	ORF1a:S367	0
hCoV-19/Mexico/CHH_INER_IMSS_1082/2021	EPI_ISL_2490322	OL461286	B.1.1.7	GRY	30	C241T,C913T,C3037T,C3267T,C5388A,C5986T,T6954C,C14408T,C14649T,C14676T,C15279T,T16176C,C18348T,A23063T,C23271A,A23403G,C23604A,C23709T,T24506G,G24914C,C27972T,G28048T,A28111G,G28280C,A28281T,T28282A,G28881A,G28882A,G28883C,C28977T	18	N:D3L,N:R203K,N:G204R,N:S235F,ORF1a:T1001I,ORF1a:A1708D,ORF1a:I2230T,ORF1b:P314L,ORF8:Q27*,ORF8:R52I,ORF8:Y73C,S:N501Y,S:A570D,S:D614G,S:P681H,S:T716I,S:S982A,S:D1118H	6	ORF1a:S367	2
hCoV-19/Mexico/CHH_INER_IMSS_1093/2021	EPI_ISL_2490323	OL461287	B.1.1.7	GRY	31	C241T,C913T,C3037T,C3267T,C5051T,G5164T,C5388A,C5986T,T6954C,C14408T,C14676T,C15279T,T16176C,G19525T,A23063T,C23271A,A23403G,C23604A,C23709T,T24506G,G24914C,C27972T,G28048T,A28111G,G28280C,A28281T,T28282A,G28881A,G28882A,G28883C,C28977T	21	N:D3L,N:R203K,N:G204R,N:S235F,ORF1a:T1001I,ORF1a:P1596S,ORF1a:E1633D,ORF1a:A1708D,ORF1a:I2230T,ORF1b:P314L,ORF1b:D2020Y,ORF8:Q27*,ORF8:R52I,ORF8:Y73C,S:N501Y,S:A570D,S:D614G,S:P681H,S:T716I,S:S982A,S:D1	6	ORF1a:S367	0
hCoV-19/Mexico/CHH_INER_IMSS_1094/2021	EPI_ISL_2490324	OL461288	B.1.1.7	GRY	31	C241T,C913T,C3037T,C3267T,C5052T,C5388A,C5986T,T6954C,C11750T,C14408T,C14676T,C15279T,T16176C,A23063T,C23271A,A23403G,C23604A,C23709T,T24506G,G24914C,C26270T,C27972T,G28048T,A28111G,G28280C,A28281T,T28282A,G28881A,G28882A,G28883C,C28977T	21	E:T9I,N:D3L,N:R203K,N:G204R,N:S235F,ORF1a:T1001I,ORF1a:P1596L,ORF1a:A1708D,ORF1a:I2230T,ORF1a:L3829F,ORF1b:P314L,ORF8:Q27*,ORF8:R52I,ORF8:Y73C,S:N501Y,S:A570D,S:D614G,S:P681H,S:T716I,S:S982A,S:D1118H	6	ORF1a:S367	0
hCoV-19/Mexico/CHH_INER_IMSS_1095/2021	EPI_ISL_2490325	OL461289	B.1.1.7	GRY	37	G174T,G210T,C241T,C913T,C2453T,C3037T,C3267T,C5388A,C5986T,T6954C,T9016C,C10252T,C14408T,C14676T,C15279T,T16176C,A23063T,C23271A,A23403G,C23604A,C23709T,T24506G,G24914C,G25126T,G27806T,C27883T,C27972T,G28048T,A28111G,G28280C,A28281T,T28282A,G28881A,G28882A,G28883C,C28977T,C29171T	22	N:D3L,N:R203K,N:G204R,N:S235F,N:H300Y,ORF1a:L730F,ORF1a:T1001I,ORF1a:A1708D,ORF1a:I2230T,ORF1b:P314L,ORF7b:A43V,ORF8:Q27*,ORF8:R52I,ORF8:Y73C,S:N501Y,S:A570D,S:D614G,S:P681H,S:T716I,S:S982A,S:D1118H,S:E1188D	6	ORF1a:S367	0
hCoV-19/Mexico/CHH_INER_IMSS_1103/2021	EPI_ISL_2490326	OL461290	B.1.1.7	GRY	33	C241T,C913T,C3037T,C3267T,A3492G,C5388A,C5986T,T6954C,C14408T,C14676T,C15279T,T16176C,C21639A,A23063T,C23271A,A23403G,C23604A,C23709T,C24374T,T24506G,G24914C,C27972T,G28048T,A28111G,G28198T,G28280C,A28281T,T28282A,C28728T,G28881A,G28882A,G	23	N:D3L,N:A152V,N:R203K,N:G204R,N:S235F,ORF1a:T1001I,ORF1a:N1076S,ORF1a:A1708D,ORF1a:I2230T,ORF1b:P314L,ORF8:Q27*,ORF8:R52I,ORF8:Y73C,ORF8:C102F,S:P26H,S:N501Y,S:A570D,S:D614G,S:P681H,S:T716I,S:L938F,S:S98	6	ORF1a:S367	0
hCoV-19/Mexico/CHH_LANGEBIO_IMSS_0275/2021	EPI_ISL_1661984	OL461291	B.1.1.7	GRY	42	C241T,C913T,C3037T,C3267T,C4345T,C5388A,C5986T,C6145T,C6896T,C6936T,T6954C,C10456T,A12162G,C14408T,C14676T,C15279T,C15720T,A16105G,T16176C,A17615G,A21137G,A23063T,C23271A,A23403G,C23604A,C23709T,C23718T,T24506G,G24914C,G25135T,C25169T,C27972T,G28048T,A28111G,G28280C,A28281T,T28282A,G28881A,G28882A,G28883C,C28977T,G29	26	N:D3L,N:R203K,N:G204R,N:S235F,ORF1a:T1001I,ORF1a:A1708D,ORF1a:S2224F,ORF1a:I2230T,ORF1a:Q3966R,ORF1b:P314L,ORF1b:R880G,ORF1b:K1383R,ORF1b:K2557R,ORF8:Q27*,ORF8:R52I,ORF8:Y73C,S:N501Y,S:A570D,S:D614G,S:P681H,S:T716I,S:T719I,S:S982A,S:D1118H,S:K1191N,S:L1203F	6	ORF1a:S367	27
hCoV-19/Mexico/CHH_LANGEBIO_IMSS_0278/2021	EPI_ISL_1661987	OL461292	B.1.1.7	GRY	30	C913T,C3037T,C3267T,C5388A,C5986T,T6954C,C9298T,C14408T,C14676T,C15279T,T16176C,A23063T,C23271A,A23403G,C23604A,C23709T,C24374T,T24506G,C24912T,G24914C,C27972T,G28048T,A28111G,G28280C,A28281T,T28282A,G28881A,G28882A,G28883C,C28977T	20	N:D3L,N:R203K,N:G204R,N:S235F,ORF1a:T1001I,ORF1a:A1708D,ORF1a:I2230T,ORF1b:P314L,ORF8:Q27*,ORF8:R52I,ORF8:Y73C,S:N501Y,S:A570D,S:D614G,S:P681H,S:T716I,S:L938F,S:S982A,S:T1117I,S:D1118H	6	ORF1a:S367	366
hCoV-19/Mexico/CHH_LANGEBIO_IMSS_0279/2021	EPI_ISL_1661988	OL461293	B.1.1.7	GRY	28	C241T,C913T,C3037T,C3267T,C5388A,C5986T,T6954C,C14408T,C14676T,C15279T,T16176C,A23063T,C23271A,A23403G,C23604A,C23709T,T24506G,G24914C,C27972T,G28048T,A28111G,G28280C,A28281T,T28282A,G28881A,G28882A,G28883C,C28977T	18	N:D3L,N:R203K,N:G204R,N:S235F,ORF1a:T1001I,ORF1a:A1708D,ORF1a:I2230T,ORF1b:P314L,ORF8:Q27*,ORF8:R52I,ORF8:Y73C,S:N501Y,S:A570D,S:D614G,S:P681H,S:T716I,S:S982A,S:D1118H	6	ORF1a:S367	1
hCoV-19/Mexico/CHH_LANGEBIO_IMSS_0515/2021	EPI_ISL_2401942	OL461294	B.1.1.7	GRY	32	C241T,C913T,C3037T,C3267T,C5388A,C5986T,T6954C,C9298T,C14408T,C14676T,C14688T,C15279T,T16176C,G17592T,A23063T,C23271A,A23403G,C23604A,C23709T,C24374T,T24506G,G24914C,C27972T,G28048T,A28111G,G28280C,A28281T,T28282A,G28881A,G28882A,G28883C,	19	N:D3L,N:R203K,N:G204R,N:S235F,ORF1a:T1001I,ORF1a:A1708D,ORF1a:I2230T,ORF1b:P314L,ORF8:Q27*,ORF8:R52I,ORF8:Y73C,S:N501Y,S:A570D,S:D614G,S:P681H,S:T716I,S:L938F,S:S982A,S:D1118H	6	ORF1a:S367	0
hCoV-19/Mexico/CHH_LANGEBIO_IMSS_0530/2021	EPI_ISL_2401957	OL461295	B.1.1.7	GRY	30	C241T,C913T,C3037T,C3267T,C5388A,C5986T,T6954C,C14408T,C14649T,C14676T,C15279T,T16176C,C18348T,A23063T,C23271A,A23403G,C23604A,C23709T,T24506G,G24914C,C27972T,G28048T,A28111G,G28280C,A28281T,T28282A,G28881A,G28882A,G28883C,C28977T	18	N:D3L,N:R203K,N:G204R,N:S235F,ORF1a:T1001I,ORF1a:A1708D,ORF1a:I2230T,ORF1b:P314L,ORF8:Q27*,ORF8:R52I,ORF8:Y73C,S:N501Y,S:A570D,S:D614G,S:P681H,S:T716I,S:S982A,S:D1118H	6	ORF1a:S367	439

hCoV-19/Mexico/CHH_LANGEBIO_IMSS_0559/2021	EPI_ISL_2401981	OL461296	B.1.1.7	GRY	32	C241T,C913T,C3037T,C3267T,G5164T,C5388A,C5986T,T6954C,T9346C,C14408T,C14676T,C15279T,T16176C,A23063T,C23271A,A23403G,C23604A,C23709T,T24506G,G24914C,C25416T,C27635T,C27972T,G28048T,A28111G,G28280C,A28281T,T28282A,G28881A,G28882A,G28883C,C2	20	N:D3L,N:R203K,N:G204R,N:S235F,ORF1a:T1001I,ORF1a:E1633D,ORF1a:A1708D,ORF1a:I2230T,ORF1b:P314L,ORF7a:S81L,ORF8:Q27*,ORF8:R52I,ORF8:Y73C,S:N501Y,S:A570D,S:D614G,S:P681H,S:T716I,S:S982A,S:D1118H	6	ORF1a:S367[illegible]	131
hCoV-19/Mexico/CHH_LANGEBIO_IMSS_0561/2021	EPI_ISL_2401983	OL461297	B.1.1.7	GRY	32	C241T,C913T,T2339A,C3037T,C3267T,C5388A,C5986T,T6954C,C11750T,C14408T,C14676T,C15279T,T16176C,C18647T,A23063T,C23271A,A23403G,C23604A,C23709T,T24506G,G24914C,C27513T,C27972T,G28048T,A28111G,G28280C,A28281T,T28282A,G28881A,G28882A,G28883C,C	21	N:D3L,N:R203K,N:G204R,N:S235F,ORF1a:S692T,ORF1a:T1001I,ORF1a:A1708D,ORF1a:I2230T,ORF1a:L3829F,ORF1b:P314L,ORF1b:P1727L,ORF8:Q27*,ORF8:R52I,ORF8:Y73C,S:N501Y,S:A570D,S:D614G,S:P681H,S:T716I,S:S982A,S:D11	6	ORF1a:S367[illegible]	446
hCoV-19/Mexico/CHH_LANGEBIO_IMSS_0572/2021	EPI_ISL_2401992	OL461298	B.1.1.7	GRY	41	C241T,C913T,C2110T,C2455T,C3037T,C3267T,C4551T,C5388A,C5986T,T6954C,G7042T,A10195G,C10582T,C10834T,C14120T,C14408T,C14676T,C15279T,T16176C,C17804T,A17805T,A23063T,C23271A,A23403G,C23604A,C23709T,T24506G,G24914C,T26171A,C27972T,G28048T,G28075T,A28095T,A28111G,G28280C,A28281T,T28282A,G28881A,G28882A,G28883C,C28977T	25	N:D3L,N:R203K,N:G204R,N:S235F,ORF1a:T1001I,ORF1a:T1429I,ORF1a:A1708D,ORF1a:I2230T,ORF1a:M2259I,ORF1b:P218L,ORF1b:P314L,ORF1b:S1446F,ORF3a:M260K,ORF8:Q27*,ORF8:R52I,ORF8:C61F,ORF8:K68*,ORF8:Y73C,S:N501Y,S:A570D,S:D614G,S:P681H,S:T716I,S:S982A,S:D1118H	6	ORF1a:S367[illegible]	9
hCoV-19/Mexico/CHH_LANGEBIO_IMSS_0580/2021	EPI_ISL_2402000	OL461299	B.1.1.7	GRY	30	C241T,C913T,C3037T,C3267T,G5164T,C5388A,C5986T,T6954C,C14408T,C14676T,C15279T,T16176C,C22987T,A23063T,C23271A,A23403G,C23604A,C23709T,T24506G,G24914C,C27972T,G28048T,A28111G,G28280C,A28281T,T28282A,G28881A,G28882A,G28883C,C28977T	19	N:D3L,N:R203K,N:G204R,N:S235F,ORF1a:T1001I,ORF1a:E1633D,ORF1a:A1708D,ORF1a:I2230T,ORF1b:P314L,ORF8:Q27*,ORF8:R52I,ORF8:Y73C,S:N501Y,S:A570D,S:D614G,S:P681H,S:T716I,S:S982A,S:D1118H	6	ORF1a:S367[illegible]	501
hCoV-19/Mexico/CHH_LANGEBIO_IMSS_0582/2021	EPI_ISL_2402002	OL461300	B.1.1.7	GRY	33	C241T,C913T,T2339A,C3037T,C3267T,C5388A,C5986T,T6954C,C11750T,C14408T,C14676T,C15279T,T16176C,C18647T,A23063T,C23271A,A23403G,C23604A,C23709T,T24506G,G24914C,C27513T,C27972T,G28027T,G28048T,A28111G,G28280C,A28281T,T28282A,G28881A,G28882A,G28883C,C28977T	22	N:D3L,N:R203K,N:G204R,N:S235F,ORF1a:S692T,ORF1a:T1001I,ORF1a:A1708D,ORF1a:I2230T,ORF1a:L3829F,ORF1b:P314L,ORF1b:P1727L,ORF8:Q27*,ORF8:W45L,ORF8:R52I,ORF8:Y73C,S:N501Y,S:A570D,S:D614G,S:P681H,S:T716I,S:S982A,S:D1118H	6	ORF1a:S367[illegible]	0
hCoV-19/Mexico/CHH_LANGEBIO_IMSS_45998-NC/2021	EPI_ISL_2969876	OL461301	B.1.1.7	GRY	29	C913T,C3037T,C3267T,C5388A,C5986T,T6954C,C14408T,C14676T,C15279T,C15656T,T16176C,A23063T,C23271A,A23403G,C23604A,C23709T,T24506G,G24914C,C26833T,C27972T,G28048T,A28111G,G28280C,A28281T,T28282A,G28881A,G28882A,G28883C,C28977T	20	M:A104V,N:D3L,N:R203K,N:G204R,N:S235F,ORF1a:T1001I,ORF1a:A1708D,ORF1a:I2230T,ORF1b:P314L,ORF1b:T730I,ORF8:Q27*,ORF8:R52I,ORF8:Y73C,S:N501Y,S:A570D,S:D614G,S:P681H,S:T716I,S:S982A,S:D1118H	6	ORF1a:S367[illegible]	1294
hCoV-19/Mexico/CHH_LANGEBIO_IMSS_46001-NC/2021	EPI_ISL_2969877	OL461302	B.1.1.7	GRY	28	C913T,C3037T,C3267T,C5388A,C5986T,T6954C,C14408T,C14676T,C15279T,T16176C,A23063T,C23271A,A23403G,C23604A,C23709T,C24374T,T24506G,G24914C,C27972T,G28048T,A28111G,G28280C,A28281T,T28282A,G28881A,G28882A,G28883C,C28977T	18	N:D3L,N:R203K,N:G204R,N:S235F,ORF1a:T1001I,ORF1a:A1708D,ORF1a:I2230T,ORF1b:P314L,ORF8:Q27*,ORF8:R52I,ORF8:Y73C,S:A570D,S:D614G,S:P681H,S:T716I,S:L938F,S:S982A,S:D1118H	6	ORF1a:S367[illegible]	2448
hCoV-19/Mexico/CHH-IBT_IMSS_1799/2021	EPI_ISL_2801678	OL461303	B.1.1.7	GRY	39	A156G,C241T,C913T,C3037T,C3267T,C4084T,C5388A,C5986T,T6954C,G7042T,C8502T,G9929A,A10195G,C14120T,C14408T,C14676T,C15279T,T16176C,C19011T,A23063T,C23271A,A23403G,C23604A,C23709T,T24506G,G24914C,C27972T,G28048T,G28075T,A28095T,A28111G,G28280C,A28281T,T28282A,T28688C,G28881A,G28882A,G28883C,C28977T	24	N:D3L,N:R203K,N:G204R,N:S235F,ORF1a:T1001I,ORF1a:A1708D,ORF1a:I2230T,ORF1a:M2259I,ORF1a:T2746I,ORF1a:D3222N,ORF1b:P218L,ORF1b:P314L,ORF8:Q27*,ORF8:R52I,ORF8:C61F,ORF8:K68*,ORF8:Y73C,S:N501Y,S:A570D,S:D614G,S:P681H,S:T716I,S:S982A,S:D1118H	6	ORF1a:S367[illegible]	696
hCoV-19/Mexico/CHH-IBT_IMSS_1813/2021	EPI_ISL_2801692	OL461304	B.1.1.7	GRY	35	C241T,C913T,G1874T,C3037T,C3267T,C5388A,C5986T,A6084G,T6954C,C9298T,C14408T,C14676T,C15279T,T16176C,C19524T,G22344T,A23063T,C23271A,A23403G,C23604A,C23709T,C24374T,T24506G,G24914C,A24999C,C27972T,G28048T,A28111G,G28280C,A28281T,T28282A,G28881A,G28882A,G28883C,C28977T	23	N:D3L,N:R203K,N:G204R,N:S235F,ORF1a:A537S,ORF1a:T1001I,ORF1a:A1708D,ORF1a:D1940G,ORF1a:I2230T,ORF1b:P314L,ORF8:Q27*,ORF8:R52I,ORF8:Y73C,S:G261V,S:N501Y,S:A570D,S:D614G,S:P681H,S:T716I,S:L938F,S:S982A,S:D1118H,S:D1146A	6	ORF1a:S367[illegible]	0
hCoV-19/Mexico/CHH-INER-IMSS-00220/2021	EPI_ISL_1279266	-	B.1.1.7	GRY	29	C241T,C913T,C3037T,C3267T,G5164T,C5388A,C5986T,T6954C,C14408T,C14676T,C15279T,T16176C,A23063T,C23271A,A23403G,C23604A,C23709T,T24506G,G24914C,C27972T,G28048T,A28111G,G28280C,A28281T,T28282A,G28881A,G28882A,G28883C,C28977T	19	N:D3L,N:R203K,N:G204R,N:S235F,ORF1a:T1001I,ORF1a:E1633D,ORF1a:A1708D,ORF1a:I2230T,ORF1b:P314L,ORF8:Q27*,ORF8:R52I,ORF8:Y73C,S:N501Y,S:A570D,S:D614G,S:P681H,S:T716I,S:S982A,S:D1118H	6	ORF1a:S367[illegible]	0
hCoV-19/Mexico/CHH-LANGEBIO_IMSS_0835/2021	EPI_ISL_2671505	OL461305	B.1.1.7	GRY	30	C913T,C3037T,C3267T,C5388A,C5986T,T6954C,C14408T,C14676T,C15279T,T16176C,C21575T,A23063T,C23271A,A23403G,C23604A,C23709T,C24374T,T24506G,G24914C,C27972T,G28048T,G28079T,A28111G,G28280C,A28281T,T28282A,G28881A,G28882A,G28883C,C28977T	20	N:D3L,N:R203K,N:G204R,N:S235F,ORF1a:T1001I,ORF1a:A1708D,ORF1a:I2230T,ORF1b:P314L,ORF8:Q27*,ORF8:R52I,ORF8:Y73C,S:L5F,S:N501Y,S:A570D,S:D614G,S:P681H,S:T716I,S:L938F,S:S982A,S:D1118H	6	ORF1a:S367[illegible]	221
hCoV-19/Mexico/CHH-LANGEBIO_IMSS_0836/2021	EPI_ISL_2671506	OL461306	B.1.1.7	GRY	32	C241T,C913T,C3037T,C3267T,C5388A,C5986T,T6954C,C9298T,C14408T,C14676T,C15279T,T16176C,A23063T,C23271A,A23403G,C23604A,C23709T,C24374T,T24506G,G24914C,G25217T,G27516T,C27972T,G28048T,A28111G,G28280C,A28281T,T28282A,G28881A,G28882A,G28883C,C2	21	N:D3L,N:R203K,N:G204R,N:S235F,ORF1a:T1001I,ORF1a:A1708D,ORF1a:I2230T,ORF1b:P314L,ORF7a:E41D,ORF8:Q27*,ORF8:R52I,ORF8:Y73C,S:N501Y,S:A570D,S:D614G,S:P681H,S:T716I,S:L938F,S:S982A,S:D1118H,S:G1219C	6	ORF1a:S367[illegible]	5
hCoV-19/Mexico/CHH-LANGEBIO_IMSS_0840/2021	EPI_ISL_2671508	OL461307	B.1.1.7	GRY	29	C913T,C3037T,C3267T,C5388A,C5986T,T6954C,C14408T,C14676T,C15279T,T16176C,A23063T,C23271A,A23403G,C23604A,C23709T,C24374T,T24506G,G24914C,G27827T,C27972T,G28048T,A28111G,G28280C,A28281T,T28282A,G28881A,G28882A,G28883C,C28977T	20	N:D3L,N:R203K,N:G204R,N:S235F,ORF1a:T1001I,ORF1a:A1708D,ORF1a:I2230T,ORF1b:P314L,ORF7b:M24I,ORF8:Q27*,ORF8:R52I,ORF8:Y73C,S:N501Y,S:A570D,S:D614G,S:P681H,S:T716I,S:L938F,S:S982A,S:D1118H	6	ORF1a:S367[illegible]	740
hCoV-19/Mexico/CHH-LANGEBIO_IMSS_0841/2021	EPI_ISL_2671509	OL461308	B.1.1.7	GRY	32	C241T,C913T,G1820A,C3037T,C3267T,C5388A,C5986T,T6954C,C14408T,C14676T,C15279T,T16176C,A23063T,C23271A,A23403G,C23604A,C23709T,C24374T,T24506G,G24914C,C27791T,C27972T,G28048T,A28111G,G28280C,A28281T,T28282A,G28881A,G28882A,G28883C,C28977T,C	21	N:D3L,N:R203K,N:G204R,N:S235F,N:T362I,ORF1a:G519S,ORF1a:T1001I,ORF1a:A1708D,ORF1a:I2230T,ORF1b:P314L,ORF8:Q27*,ORF8:R52I,ORF8:Y73C,S:N501Y,S:A570D,S:D614G,S:P681H,S:T716I,S:L938F,S:S982A,S:D1118H	6	ORF1a:S367[illegible]	0
hCoV-19/Mexico/CHH-LANGEBIO_IMSS_0842/2021	EPI_ISL_2671510	OL461309	B.1.1.7	GRY	34	C913T,C3037T,C3267T,C5052T,C5388A,C5986T,C6541T,T6954C,C11750T,C14408T,C14676T,C15279T,C15952T,T16176C,A23063T,C23271A,A23403G,C23604A,C23709T,T24506G,G24914C,G24985A,C26270T,C27972T,G28048T,A28111G,G28280C,A28281T,T28282A,G28881A,G28882A,G28883C,C28977T,G29861A	21	E:T9I,N:D3L,N:R203K,N:G204R,N:S235F,ORF1a:T1001I,ORF1a:P1596L,ORF1a:A1708D,ORF1a:I2230T,ORF1a:L3829F,ORF1b:P314L,ORF8:Q27*,ORF8:R52I,ORF8:Y73C,S:N501Y,S:A570D,S:D614G,S:P681H,S:T716I,S:S982A,S:D1118H	6	ORF1a:S367[illegible]	2
hCoV-19/Mexico/CHH-LANGEBIO_IMSS_0866/2021	EPI_ISL_2671515	OL461310	B.1.1.7	GRY	31	C913T,G1796A,C2455T,C3037T,C3267T,G5164T,C5388A,C5986T,T6954C,C14408T,C14676T,C15279T,T16176C,A23063T,C23271A,A23403G,C23604A,C23709T,T24506G,G24914C,G25538A,C27972T,G28048T,A28111G,G28280C,A28281T,T28282A,G28881A,G28882A,G28883C,C28977T	21	N:D3L,N:R203K,N:G204R,N:S235F,ORF1a:V511I,ORF1a:T1001I,ORF1a:E1633D,ORF1a:A1708D,ORF1a:I2230T,ORF1b:P314L,ORF3a:G49D,ORF8:Q27*,ORF8:R52I,ORF8:Y73C,S:N501Y,S:A570D,S:D614G,S:P681H,S:T716I,S:S982A,S:D1118	6	ORF1a:S367[illegible]	213
hCoV-19/Mexico/CHH-LANGEBIO_IMSS_0868/2021	EPI_ISL_2671517	OL461311	B.1.1.7	GRY	36	C193T,C913T,C2232T,C3037T,C3267T,C5388A,C5986T,T6954C,T11672A,C12008T,C14408T,C14676T,C15279T,T16176C,C19269T,C22987T,A23063T,C23271A,A23403G,C23604A,C23709T,C24374T,T24506G,C24912T,G24914C,C25614T,C27972T,G28048T,A28111G,G28280C,A28281T,T28282A,G28881A,G28882A,G28883C,C28977T	23	N:D3L,N:R203K,N:G204R,N:S235F,ORF1a:A656V,ORF1a:T1001I,ORF1a:A1708D,ORF1a:I2230T,ORF1a:Y3803N,ORF1a:L3915F,ORF1b:P314L,ORF8:Q27*,ORF8:R52I,ORF8:Y73C,S:N501Y,S:A570D,S:D614G,S:P681H,S:T716I,S:L938F,S:S982A,S:T1117I,S:D1118H	6	ORF1a:S367[illegible]	168
hCoV-19/Mexico/CHH-LANGEBIO_IMSS_0871/2021	EPI_ISL_2671519	OL461312	B.1.1.7	GRY	40	C241T,C913T,C2110T,G2944A,C3037T,C3267T,C4754T,C5388A,C5986T,T6954C,T7984C,A8556G,T9139C,C11750T,C14120T,C14408T,C14676T,C15279T,T16176C,C16293T,C19390T,A23063T,C23271A,A23403G,C23604A,C23709T,T24506G,G24914C,C25578T,C27972T,G28048T,A28111G,G28280C,A28281T,T28282A,G28881A,G28882A,G28883C,C28977T,C29353T	23	N:D3L,N:R203K,N:G204R,N:S235F,ORF1a:T1001I,ORF1a:P1497S,ORF1a:A1708D,ORF1a:I2230T,ORF1a:K2764R,ORF1a:L3829F,ORF1b:P218L,ORF1b:P314L,ORF1b:P1975S,ORF8:Q27*,ORF8:R52I,ORF8:Y73C,S:N501Y,S:A570D,S:D614G,S:P681H,S:T716I,S:S982A,S:D1118H	6	ORF1a:S367[illegible]	59
hCoV-19/Mexico/CHH-LANGEBIO_IMSS_0879/2021	EPI_ISL_2671521	OL461313	B.1.1.7	GRY	39	C913T,C3037T,C3267T,C5388A,G5578A,C5986T,G6734A,T6954C,C9598T,C9857T,C12525T,C13426T,C14120T,C14408T,C14676T,C15279T,T16176C,C17762G,C21575T,A23063T,C23271A,A23403G,C23604A,C23709T,T24506G,G24914C,T27462C,T27768G,C27972T,G28048T,A28095T,A28111G,G28280C,A28281T,T28282A,G28881A,G28882A,G28883C,C28977T	27	N:D3L,N:R203K,N:G204R,N:S235F,ORF1a:T1001I,ORF1a:A1708D,ORF1a:M1771I,ORF1a:V2157I,ORF1a:I2230T,ORF1a:T4087I,ORF1b:P218L,ORF1b:P314L,ORF1b:A1432G,ORF7a:S98A,ORF7b:S5A,ORF8:Q27*,ORF8:R52I,ORF8:K68*,ORF8:Y73C,S:L5F,S:N501Y,S:A570D,S:D614G,S:P681H,S:T716I,S:S982A,S:D1118H	9	ORF1a:S367[illegible]	278
hCoV-19/Mexico/CHH-LANGEBIO_IMSS_0887/2021	EPI_ISL_2671523	OL461314	B.1.1.7	GRY	30	C241T,C913T,C3037T,C3267T,C5388A,C5986T,T6954C,C14408T,C14676T,C15279T,T16176C,C21306T,C21622T,A23063T,C23271A,A23403G,C23604A,C23709T,T24506G,G24914C,C27972T,G28048T,A28111G,G28280C,A28281T,T28282A,G28881A,G28882A,G28883C,C28977T	18	N:D3L,N:R203K,N:G204R,N:S235F,ORF1a:T1001I,ORF1a:A1708D,ORF1a:I2230T,ORF1b:P314L,ORF8:Q27*,ORF8:R52I,ORF8:Y73C,S:N501Y,S:A570D,S:D614G,S:P681H,S:T716I,S:S982A,S:D1118H	6	ORF1a:S367[illegible]	16
hCoV-19/Mexico/CHH-LANGEBIO_IMSS_0889/2021	EPI_ISL_2671525	OL461315	B.1.1.7	GRY	38	C241T,C913T,C2110T,C2338T,C3037T,C3267T,T5278C,C5388A,C5986T,T6954C,G7042T,A8879G,C14120T,C14408T,C14676T,C15279T,T16176C,G21578T,A22525G,A23063T,C23271A,A23403G,C23604A,C23709T,G24415T,T24506G,G24914C,C27972T,G28048T,A28095T,A28111G,G28280C,A28281T,T28282A,G28881A,G28882A,G28883C,C28977T	23	N:D3L,N:R203K,N:G204R,N:S235F,ORF1a:T1001I,ORF1a:A1708D,ORF1a:I2230T,ORF1a:M2259I,ORF1a:T2872A,ORF1b:P218L,ORF1b:P314L,ORF8:Q27*,ORF8:R52I,ORF8:K68*,ORF8:Y73C,S:V6F,S:N501Y,S:A570D,S:D614G,S:P681H,S:T716I,S:S982A,S:D1118H	6	ORF1a:S367[illegible]	0
hCoV-19/Mexico/CHH-LANGEBIO_IMSS_1366/2021	EPI_ISL_2942497	OL461316	B.1.1.7	GRY	37	C241T,C913T,C2110T,C3037T,C3267T,C5388A,C5986T,T6954C,G7042T,A8031G,A10195G,A12555G,C14120T,C14408T,C14676T,C15279T,T16176C,C20544A,A23063T,C23271A,A23403G,C23604A,C23709T,T24506G,G24914C,C27972T,G28048T,G28075T,A28111G,G28280C,A28281T,T28282A,G28881A,G28882A,G28883C,C28977T,G29759C	23	N:D3L,N:R203K,N:G204R,N:S235F,ORF1a:T1001I,ORF1a:A1708D,ORF1a:I2230T,ORF1a:M2259I,ORF1a:K2589R,ORF1a:E4097G,ORF1b:P218L,ORF1b:P314L,ORF8:Q27*,ORF8:R52I,ORF8:C61F,ORF8:Y73C,S:N501Y,S:A570D,S:D614G,S:P681H,S:T716I,S:S982A,S:D1118H	6	ORF1a:S367[illegible]	30

hCoV-19/Mexico/CHH-LANGEBIO_IMSS_1379/2021	EPI_ISL_2942507	OL461317	B.1.1.7	GRY	31	C241T,C913T,C3037T,C3267T,G5164T,C5388A,C5986T,T6954C,G7873T,C14408T,C14676T,C15279T,T16176C,A23063T,C23271A,A23403G,C23604A,C23709T,T24506G,G24914C,C25904T,C27972T,G28048T,A28111G,G28280C,A28281T,T28282A,G28881A,G28882A,G28883C,C28977T	21	N:D3L,N:R203K,N:G204R,N:S235F,ORF1a:T1001I,ORF1a:E1633D,ORF1a:A1708D,ORF1a:I2230T,ORF1a:L2536F,ORF1b:P314L,ORF3a:S171L,ORF8:Q27*,ORF8:R52I,ORF8:Y73C,S:N501Y,S:A570D,S:D614G,S:P681H,S:T716I,S:S982A,S:D11	6 ORF1a:S367	320
hCoV-19/Mexico/CHH-LANGEBIO_IMSS_1681/2021	EPI_ISL_2942772	OL461318	B.1.1.7	GRY	35	C241T,C913T,C1419T,C3037T,C3267T,G5164T,C5388A,C5986T,T6954C,C11962T,C14408T,C14676T,C15279T,T16176C,C21575T,A23063T,C23271A,A23403G,C23604A,C23709T,C24381T,T24506G,G24914C,T25580C,C27972T,G28048T,A28111G,G28280C,A28281T,T28282A,G28881A,G28882A,G28883C,C28977T,C29640T	23	N:D3L,N:R203K,N:G204R,N:S235F,ORF1a:A385V,ORF1a:T1001I,ORF1a:E1633D,ORF1a:A1708D,ORF1a:I2230T,ORF1b:P314L,ORF3a:I63T,ORF8:Q27*,ORF8:R52I,ORF8:Y73C,S:L5F,S:N501Y,S:A570D,S:D614G,S:P681H,S:T716I,S:S940F,S:S982A,S:D1118H	6 ORF1a:S367	43
hCoV-19/Mexico/CMX_IBT_IMSS_11022-NC/2021	EPI_ISL_2681369	OL461319	B.1.1.7	GRY	33	C241T,C913T,C3037T,C3267T,C5388A,C5986T,C6196T,T6954C,C14408T,C14676T,C15279T,T16176C,A23063T,C23271A,A23403G,C23604A,C23709T,C24370T,T24506G,G24914C,G25217T,G26727T,C27972T,G28048T,A28111G,G28280C,A28281T,T28282A,G28881A,G28882A,G28883C,C2	20	M:A69S,N:D3L,N:R203K,N:G204R,N:S235F,ORF1a:T1001I,ORF1a:A1708D,ORF1a:I2230T,ORF1b:P314L,ORF8:Q27*,ORF8:R52I,ORF8:Y73C,S:N501Y,S:A570D,S:D614G,S:P681H,S:T716I,S:S982A,S:D1118H,S:G1219C	6 ORF1a:S367	861
hCoV-19/Mexico/CMX_IBT_IMSS_1133/2021	EPI_ISL_2391710	OL461320	B.1.1.7	GRY	39	C241T,C913T,C3037T,C3267T,C5388A,C5986T,G6647A,T6954C,T9157C,C14408T,C14676T,T15096C,C15279T,T16176C,C17676T,C18183T,C19524T,A21519C,A23063T,C23271A,A23403G,C23604A,C23709T,A24070T,T24506G,G24914C,C25624T,C27972T,G28048T,A28095T,A28111G,G28280C,A28281T,T28282A,G28881A,G28882A,G28883C,G28884C,C28977T	23	N:D3L,N:R203K,N:G204P,N:S235F,ORF1a:T1001I,ORF1a:A1708D,ORF1a:A2128T,ORF1a:I2230T,ORF1b:P314L,ORF1b:R2684S,ORF3a:H78Y,ORF8:Q27*,ORF8:R52I,ORF8:K68*,ORF8:Y73C,S:N501Y,S:A570D,S:D614G,S:P681H,S:T716I,S:Q836H,S:S982A,S:D1118H	6 ORF1a:S367	571
hCoV-19/Mexico/CMX_IBT_IMSS_1134/2021	EPI_ISL_2391660	OL461321	B.1.1.7	GRY	38	C913T,G960A,C3037T,C3267T,C5388A,C5986T,T6954C,G8102A,C14408T,C14676T,T15096C,C15279T,T16176C,C19164T,C20104T,G21893A,A23063T,C23271A,A23403G,C23604A,C23709T,T24506G,G24914C,C25096T,G25437C,G27375A,C27972T,G28048T,A28095T,A28111G,G28280C,A28281T,T28282A,G28881A,G28882A,G28883C,C28977T,T29165C	26	N:D3L,N:R203K,N:G204R,N:S235F,N:Y298H,ORF1a:R232H,ORF1a:T1001I,ORF1a:A1708D,ORF1a:I2230T,ORF1a:V2613I,ORF1b:P314L,ORF1b:L2213F,ORF3a:L15F,ORF6:M58I,ORF8:Q27*,ORF8:R52I,ORF8:K68*,ORF8:Y73C,S:D111N,S:N501Y,S:A570D,S:D614G,S:P681H,S:T716I,S:S982A,S:D1118H	6 ORF1a:S367	317
hCoV-19/Mexico/CMX_IBT_IMSS_1156/2021	EPI_ISL_2391508	OL461322	B.1.1.7	GR	31	C203T,C222T,C241T,C913T,C3037T,C3140T,C3267T,C10029T,C10954T,A12949G,A13237G,C14408T,C15279T,T16176C,T19839C,C23271A,A23403G,C23604A,C23709T,G24914C,G26727T,C27972T,G28048T,A28111G,G28280C,A28281T,T28282A,G28881A,G28882A,G28883C,G29715T	16	M:A69S,N:D3L,N:R203K,N:G204R,ORF1a:P959S,ORF1a:T1001I,ORF1a:T3255I,ORF1b:P314L,ORF8:Q27*,ORF8:R52I,ORF8:Y73C,S:A570D,S:D614G,S:P681H,S:T716I,S:D1118H	0	1
hCoV-19/Mexico/CMX_IBT_IMSS_1263/2021	EPI_ISL_2391617	OL461323	B.1.1.7	GRY	36	C241T,C913T,C3037T,C3267T,C5388A,C5986T,T6954C,G8513A,A12162G,C14408T,C14676T,C15279T,C15720T,T16176C,A17615G,A18172G,A21137G,A23063T,C23271A,A23403G,C23604A,C23709T,T24506G,G24914C,G25135T,C26151T,C27972T,G28048T,A28111G,G28280C,A28281T,T28282A,G28881A,G28882A,G28883C,C28977T	24	N:D3L,N:R203K,N:G204R,N:S235F,ORF1a:T1001I,ORF1a:A1708D,ORF1a:I2230T,ORF1a:V2750I,ORF1a:Q3966R,ORF1b:P314L,ORF1b:K1383R,ORF1b:I1569V,ORF1b:K2557R,ORF8:Q27*,ORF8:R52I,ORF8:Y73C,S:N501Y,S:A570D,S:D614G,S:P681H,S:T716I,S:S982A,S:D1118H,S:K1191N	6	247
hCoV-19/Mexico/CMX_IBT_IMSS_1281/2021	EPI_ISL_2391572	OL461324	B.1.1.7	GRY	39	C241T,C913T,C936T,G1069A,C3037T,C3177T,C3267T,C5388A,C5986T,T6954C,C7735T,C14408T,C14676T,C15279T,A15668T,T16176C,G18538A,C19586T,C20844T,T21471C,A23063T,C23271A,A23403G,C23604A,C23709T,C24374T,T24506G,G24914C,T27384C,C27972T,G28048T,A28111G,G28280C,A28281T,T28282A,G28881A,G28882A,G28883C,C28977T	24	N:D3L,N:R203K,N:G204R,N:S235F,ORF1a:T224I,ORF1a:P971L,ORF1a:T1001I,ORF1a:A1708D,ORF1a:I2230T,ORF1b:P314L,ORF1b:N734I,ORF1b:V1691I,ORF1b:T2040I,ORF8:Q27*,ORF8:R52I,ORF8:Y73C,S:N501Y,S:A570D,S:D614G,S:P681H,S:T716I,S:L938F,S:S982A,S:D1118H	6	22
hCoV-19/Mexico/CMX_IBT_IMSS_1510/2021	EPI_ISL_2681352	OL461325	B.1.1.7	GRY	34	C241T,C913T,C3037T,C3267T,C5388A,C5986T,C6196T,T6954C,C9803T,T11135C,C14408T,C14676T,C15279T,T16176C,A23063T,C23114T,C23271A,A23403G,C23604A,C23709T,G24095T,T24506G,G24914C,C27972T,G28048T,A28111G,G28280C,A28281T,T28282A,G28881A,G28882A,G2	20	N:D3L,N:R203K,N:G204R,N:S235F,ORF1a:T1001I,ORF1a:A1708D,ORF1a:I2230T,ORF1a:F3624L,ORF1b:P314L,ORF8:Q27*,ORF8:R52I,ORF8:Y73C,S:N501Y,S:A570D,S:D614G,S:P681H,S:T716I,S:A845S,S:S982A,S:D1118H	6	767
hCoV-19/Mexico/CMX_IBT_IMSS_1532/2021	EPI_ISL_2681058	OL461326	B.1.1.7	GRY	39	C241T,C823T,C913T,C2110T,G2305T,G3004T,C3037T,C3267T,C5388A,C5986T,T6954C,G7042T,C9803T,C14120T,C14408T,C14676T,C15279T,T16176C,A23063T,C23271A,A23403G,C23604A,C23709T,T24506G,G24914C,A25659G,C26110T,C26894T,C27972T,G28048T,A28095T,A28111G,G28280C,A28281T,T28282A,G28881A,G28882A,G28883C,C28977T	24	N:D3L,N:R203K,N:G204R,N:S235F,ORF1a:K680N,ORF1a:E913D,ORF1a:T1001I,ORF1a:A1708D,ORF1a:I2230T,ORF1a:M2259I,ORF1b:P218L,ORF1b:P314L,ORF3a:P240S,ORF8:Q27*,ORF8:R52I,ORF8:K68*,ORF8:Y73C,S:N501Y,S:A570D,S:D614G,S:P681H,S:T716I,S:S982A,S:D1118H	6	2
hCoV-19/Mexico/CMX_IBT_IMSS_1535/2021	EPI_ISL_2681165	OL461327	B.1.1.7	GRY	42	C241T,C913T,C3037T,C3267T,C5388A,C5944T,C5986T,G6647A,T6954C,C14408T,C14621T,C14676T,T15096C,C15279T,T16176C,C17676T,C18183T,C19524T,A21519C,A23063T,C23271A,A23403G,C23604A,C23709T,A24070T,A24092G,T24506G,G24914C,C25624T,C27737T,C27972T,G28048T,A28095T,A28111G,G28280C,A28281T,T28282A,G28881A,G28882A,G28883C,G28884C,C	26	N:D3L,N:R203K,N:G204P,N:S235F,ORF1a:T1001I,ORF1a:A1708D,ORF1a:A2128T,ORF1a:I2230T,ORF1b:P314L,ORF1b:T385M,ORF1b:R2684S,ORF3a:H78Y,ORF7a:T115I,ORF8:Q27*,ORF8:R52I,ORF8:K68*,ORF8:Y73C,S:N501Y,S:A570D,S:D614G,S:P681H,S:T716I,S:Q836H,S:I844V,S:S982A,S:D1118H	6	0
hCoV-19/Mexico/CMX_IBT_IMSS_1538/2021	EPI_ISL_2681310	OL461328	B.1.1.7	GRY	35	C241T,C913T,C3037T,G3246T,C3267T,T5278C,C5388A,C5986T,T6954C,G7042T,C14120T,C14408T,C14676T,C15279T,T16176C,G21578T,A22525G,A23063T,C23271A,A23403G,C23604A,C23709T,G24415T,G24914C,C27972T,G28048T,A28095T,A28111G,G28280C,A28281T,T28282A,G28881A,G28882A,G28883C,C28977T	22	N:D3L,N:R203K,N:G204R,N:S235F,ORF1a:S994I,ORF1a:T1001I,ORF1a:A1708D,ORF1a:I2230T,ORF1a:M2259I,ORF1b:P218L,ORF1b:P314L,ORF8:Q27*,ORF8:R52I,ORF8:K68*,ORF8:Y73C,S:V6F,S:N501Y,S:A570D,S:D614G,S:P681H,S:T716I,S:D1118H	6	517
hCoV-19/Mexico/CMX_IBT_IMSS_1554/2021	EPI_ISL_2681167	OL461329	B.1.1.7	GRY	43	C241T,C913T,C3037T,C3267T,C5388A,C5944T,C5986T,G6647A,C6936T,T6954C,C14408T,C14621T,C14676T,T15096C,C15279T,T16176C,C17676T,C18183T,C19524T,A21519C,A23063T,C23271A,A23403G,C23604A,C23709T,A24070T,A24092G,T24506G,G24914C,C25624T,C27737T,C27972T,G28048T,A28095T,A28111G,G28280C,A28281T,T28282A,G28881A,G28882A,G28883C,G28	27	N:D3L,N:R203K,N:G204P,N:S235F,ORF1a:T1001I,ORF1a:A1708D,ORF1a:A2128T,ORF1a:S2224F,ORF1a:I2230T,ORF1b:P314L,ORF1b:T385M,ORF1b:R2684S,ORF3a:H78Y,ORF7a:T115I,ORF8:Q27*,ORF8:R52I,ORF8:K68*,ORF8:Y73C,S:N501Y,S:A570D,S:D614G,S:P681H,S:T716I,S:Q836H,S:I844V,S:S982A,S:D1118H	6	0
hCoV-19/Mexico/CMX_IBT_IMSS_1556/2021	EPI_ISL_2681313	OL461330	B.1.1.7	GRY	34	C241T,C913T,C3037T,C3267T,C5388A,C5986T,C6196T,T6954C,C14408T,C14676T,C15279T,T16176C,G19816T,A23063T,C23271A,A23403G,C23604A,C23709T,T24506G,G24914C,G25217T,G26727T,T27285C,C27972T,G28048T,A28111G,G28280C,A28281T,T28282A,G28881A,G28882A,G2	21	M:A69S,N:D3L,N:R203K,N:G204R,N:S235F,ORF1a:T1001I,ORF1a:A1708D,ORF1a:I2230T,ORF1b:P314L,ORF1b:V2117L,ORF8:Q27*,ORF8:R52I,ORF8:Y73C,S:N501Y,S:A570D,S:D614G,S:P681H,S:T716I,S:S982A,S:D1118H,S:G1219C	6	588
hCoV-19/Mexico/CMX_IBT_IMSS_1627/2021	EPI_ISL_2681216	OL461331	B.1.1.7	GRY	36	C241T,C913T,C2937T,C3037T,C3267T,C5388A,C5986T,C6196T,T6954C,A8848G,A11898G,C14408T,C14676T,C15279T,T16176C,T19624C,A23063T,C23271A,A23403G,C23604A,C23709T,T24506G,G24914C,G25217T,C26270T,C27972T,G28048T,A28111G,G28280C,A28281T,T28282A,G28881A,G28882A,G28883C,C28977T,G29715T	22	E:T9I,N:D3L,N:R203K,N:G204R,N:S235F,ORF1a:T891I,ORF1a:T1001I,ORF1a:A1708D,ORF1a:I2230T,ORF1a:Q3878R,ORF1b:P314L,ORF8:Q27*,ORF8:R52I,ORF8:Y73C,S:N501Y,S:A570D,S:D614G,S:P681H,S:T716I,S:S982A,S:D1118H,S:G1219C	6	64
hCoV-19/Mexico/CMX_IBT_IMSS_1636/2021	EPI_ISL_2681195	OL461332	B.1.1.7	GRY	35	C241T,C913T,C3037T,C3267T,C5388A,C5986T,T6954C,C7299T,C11750T,C14408T,C14676T,T15096C,C15279T,T16176C,A23063T,C23271A,A23403G,C23595T,C23604A,C23709T,T24506G,G24914C,G25489T,C27972T,G28048T,A28095T,A28111G,G28280C,A28281T,T28282A,G28881A,G28882A,G28883C,G28913A,C28977T	24	N:D3L,N:R203K,N:G204R,N:G214S,N:S235F,ORF1a:T1001I,ORF1a:A1708D,ORF1a:I2230T,ORF1a:A2345V,ORF1a:L3829F,ORF1b:P314L,ORF3a:A33S,ORF8:Q27*,ORF8:R52I,ORF8:K68*,ORF8:Y73C,S:N501Y,S:A570D,S:D614G,S:T678I,S:P681H,S:T716I,S:S982A,S:D1118H	6	4
hCoV-19/Mexico/CMX_IBT_IMSS_1637/2021	EPI_ISL_2681174	OL461333	B.1.1.7	GRY	34	C241T,C913T,C3037T,C3267T,C5388A,C5986T,T6954C,C7299T,C11750T,C14408T,C14676T,T15096C,C15279T,T16176C,A23063T,C23271A,A23403G,C23604A,C23709T,T24506G,G24914C,G25489T,C27972T,G28048T,A28095T,A28111G,G28280C,A28281T,T28282A,G28881A,G28882A,G28883C,G28913A,C28977T	23	N:D3L,N:R203K,N:G204R,N:G214S,N:S235F,ORF1a:T1001I,ORF1a:A1708D,ORF1a:I2230T,ORF1a:A2345V,ORF1a:L3829F,ORF1b:P314L,ORF3a:A33S,ORF8:Q27*,ORF8:R52I,ORF8:K68*,ORF8:Y73C,S:N501Y,S:A570D,S:D614G,S:P681H,S:T716I,S:S982A,S:D1118H	6	0
hCoV-19/Mexico/CMX_IBT_IMSS_1638/2021	EPI_ISL_2681249	OL461334	B.1.1.7	GRY	34	C241T,C913T,C3037T,C3267T,C5388A,C5986T,T6954C,C7299T,C11750T,C14408T,C14676T,T15096C,C15279T,T16176C,A23063T,C23271A,A23403G,C23604A,C23709T,T24506G,G24914C,G25489T,C27972T,G28048T,A28095T,A28111G,G28280C,A28281T,T28282A,G28881A,G28882A,G28883C,G28913A,C28977T	23	N:D3L,N:R203K,N:G204R,N:G214S,N:S235F,ORF1a:T1001I,ORF1a:A1708D,ORF1a:I2230T,ORF1a:A2345V,ORF1a:L3829F,ORF1b:P314L,ORF3a:A33S,ORF8:Q27*,ORF8:R52I,ORF8:K68*,ORF8:Y73C,S:N501Y,S:A570D,S:D614G,S:P681H,S:T716I,S:S982A,S:D1118H	6	161
hCoV-19/Mexico/CMX_IBT_IMSS_1643/2021	EPI_ISL_2681226	OL461335	B.1.1.7	GRY	34	C241T,C913T,C3037T,C3267T,C5388A,C5986T,T6954C,C7299T,C11750T,C14408T,C14676T,T15096C,C15279T,T16176C,A23063T,C23271A,A23403G,C23604A,C23709T,T24506G,G24914C,G25489T,C27972T,G28048T,A28095T,A28111G,G28280C,A28281T,T28282A,G28881A,G28882A,G28883C,G28913A,C28977T	23	N:D3L,N:R203K,N:G204R,N:G214S,N:S235F,ORF1a:T1001I,ORF1a:A1708D,ORF1a:I2230T,ORF1a:A2345V,ORF1a:L3829F,ORF1b:P314L,ORF3a:A33S,ORF8:Q27*,ORF8:R52I,ORF8:K68*,ORF8:Y73C,S:N501Y,S:A570D,S:D614G,S:P681H,S:T716I,S:S982A,S:D1118H	6	95
hCoV-19/Mexico/CMX_IBT_IMSS_1644/2021	EPI_ISL_2681132	OL461336	B.1.1.7	GRY	34	C241T,C913T,C3037T,C3267T,C5388A,C5986T,T6954C,C7299T,C11750T,C14408T,C14676T,T15096C,C15279T,T16176C,A23063T,C23271A,A23403G,C23604A,C23709T,T24506G,G24914C,G25489T,C27972T,G28048T,A28095T,A28111G,G28280C,A28281T,T28282A,G28881A,G28882A,G28883C,G28913A,C28977T	23	N:D3L,N:R203K,N:G204R,N:G214S,N:S235F,ORF1a:T1001I,ORF1a:A1708D,ORF1a:I2230T,ORF1a:A2345V,ORF1a:L3829F,ORF1b:P314L,ORF3a:A33S,ORF8:Q27*,ORF8:R52I,ORF8:K68*,ORF8:Y73C,S:N501Y,S:A570D,S:D614G,S:P681H,S:T716I,S:S982A,S:D1118H	6	0

hCoV-19/Mexico/CMX_IBT_IMSS_1679/2021	EPI_ISL_2681062	OL461337	Q.3	GRY	39	C241T,C913T,C3037T,C3267T,G5023A,C5031T,C5388A,C5986T,C6568T,T6954C,G8183A,A9529G,G10396T,C14408T,C14676T,C15279T,T16176C,A17615G,G20962A,G22094C,A23063T,C23271A,A23403G,C23604A,C23709T,T24506G,G24914C,G25555T,G26730C,C27972T,G28048T,A28111G,G28280C,A28281T,T28282A,G28881A,G28882A,G28883C,C28977T	26	M:V70L,N:D3L,N:R203K,N:G204R,N:S235F,ORF1a:T1001I,ORF1a:M1586I,ORF1a:T1589I,ORF1a:A1708D,ORF1a:I2230T,ORF1a:G2640R,ORF1b:P314L,ORF1b:K1383R,ORF1b:D2499N,ORF3a:V55F,ORF8:Q27*,ORF8:R52I,ORF8:Y73C,S:D178H,S:N501Y,S:A570D,S:D614G,S:P681H,S:T716I,S:S982A,S:D1118H	6	5
hCoV-19/Mexico/CMX_IBT_IMSS_1686/2021	EPI_ISL_2681066	OL461338	B.1.1.7	GRY	43	C241T,C913T,C3037T,C3267T,C5388A,C5944T,C5986T,G6647A,C6936T,T6954C,C14408T,C14621T,C14676T,T15096C,C15279T,T16176C,C17676T,C18183T,C19524T,A21519C,A23063T,C23271A,A23403G,C23604A,C23709T,A24070T,A24092G,T24506G,G24914C,C25624T,C27737T,C27972T,G28048T,A28095T,A28111G,G28280C,A28281T,T28282A,G28881A,G28882A,G28883C,G28	27	N:D3L,N:R203K,N:G204P,N:S235F,ORF1a:T1001I,ORF1a:A1708D,ORF1a:A2128T,ORF1a:S2224F,ORF1a:I2230T,ORF1b:P314L,ORF1b:T385M,ORF1b:R2684S,ORF3a:H78Y,ORF7a:T115I,ORF8:Q27*,ORF8:R52I,ORF8:K68*,ORF8:Y73C,S:N501Y,S:A570D,S:D614G,S:P681H,S:T716I,S:Q836H,S:I844V,S:S982A,S:D1118H	6	1
hCoV-19/Mexico/CMX_INER_IMSS_00652/2021	EPI_ISL_1585647	OL461339	B.1.1.7	GRY	37	T201A,C241T,C823T,C913T,C2110T,C3037T,C3267T,C5388A,C5986T,T6954C,G7042T,A10195G,C13329T,C14120T,C14408T,C14676T,C15279T,T16176C,A23063T,C23271A,A23403G,C23604A,C23709T,T24506G,G24914C,C27972T,G28048T,G28075T,A28095T,A28111G,G28280C,A28281T,T28282A,G28881A,G28882A,G28883C,C28977T	23	N:D3L,N:R203K,N:G204R,N:S235F,ORF1a:T1001I,ORF1a:A1708D,ORF1a:I2230T,ORF1a:M2259I,ORF1a:T4355I,ORF1b:P218L,ORF1b:P314L,ORF8:Q27*,ORF8:R52I,ORF8:C61F,ORF8:K68*,ORF8:Y73C,S:N501Y,S:A570D,S:D614G,S:P681H,S:T716I,S:S982A,S:D1118H	6	0
hCoV-19/Mexico/CMX_INER_IMSS_00878/2021	EPI_ISL_2091329	OL461340	B.1.1.7	GRY	32	C241T,C913T,C3037T,C3267T,C5388A,C5986T,C6525T,T6954C,C14408T,C14676T,C15279T,T16176C,A17615G,A23063T,C23271A,A23403G,C23604A,C23709T,T24506G,G24914C,C27972T,G28048T,A28111G,G28280C,A28281T,T28282A,C28775T,G28881A,G28882A,G28883C,C28977T,G2	21	N:D3L,N:P168S,N:R203K,N:G204R,N:S235F,ORF1a:T1001I,ORF1a:A1708D,ORF1a:T2087I,ORF1a:I2230T,ORF1b:P314L,ORF1b:K1383R,ORF8:Q27*,ORF8:R52I,ORF8:Y73C,S:N501Y,S:A570D,S:D614G,S:P681H,S:T716I,S:S982A,S:D1118H	9	0
hCoV-19/Mexico/CMX_INER_IMSS_00979/2021	EPI_ISL_2091419	OL461341	B.1.1.7	GRY	33	C241T,C913T,C3037T,C3267T,C5388A,C5986T,C6196T,T6954C,A8848G,C14408T,C14676T,C15279T,T16176C,A23063T,C23271A,A23403G,C23604A,C23709T,T24506G,G24914C,G25217T,G26727T,C27972T,G28048T,A28111G,G28280C,A28281T,T28282A,G28881A,G28882A,G28883C,C2	20	M:A69S,N:D3L,N:R203K,N:G204R,N:S235F,ORF1a:T1001I,ORF1a:A1708D,ORF1a:I2230T,ORF1b:P314L,ORF8:Q27*,ORF8:R52I,ORF8:Y73C,S:N501Y,S:A570D,S:D614G,S:P681H,S:T716I,S:S982A,S:D1118H,S:G1219C	6	0
hCoV-19/Mexico/CMX_INER_IMSS_1254/2021	EPI_ISL_2490349	OL461342	B.1.1.7	GRY	40	C241T,C913T,C2469T,C3037T,C3267T,C5388A,C5986T,T6954C,C7299T,C12924T,C14184T,C14408T,C14676T,T15096C,C15279T,T16176C,A23063T,C23271A,A23403G,C23604A,C23638T,C23709T,T24506G,G24914C,C25584T,G27754T,C27972T,G28048T,A28095T,A28111G,G28280C,A28281T,T28282A,A28877T,G28878C,G28881A,G28882A,G28883C,C28977T,C29722T	23	N:D3L,N:R203K,N:G204R,N:S235F,ORF1a:A735V,ORF1a:T1001I,ORF1a:A1708D,ORF1a:I2230T,ORF1a:A2345V,ORF1a:P4220L,ORF1b:P314L,ORF7a:E121*,ORF8:Q27*,ORF8:R52I,ORF8:K68*,ORF8:Y73C,S:N501Y,S:A570D,S:D614G,S:P681H,S:T716I,S:S982A,S:D1118H	6	0
hCoV-19/Mexico/CMX-IBT_IMSS_15419-NC/2021	EPI_ISL_2801784	OL461343	B.1.1.7	GRY	42	C241T,C913T,C3037T,C3267T,C5388A,C5944T,C5986T,G6647A,T6954C,C14408T,C14621T,C14676T,T15096C,C15279T,T16176C,C17676T,C18183T,C19524T,A21519C,T22385C,A23063T,C23271A,A23403G,C23604A,C23709T,A24070T,T24506G,G24914C,C25624T,C27737T,C27972T,G28048T,A28095T,A28111G,G28280C,A28281T,T28282A,G28881A,G28882A,G28883C,G28884C,C2	26	N:D3L,N:R203K,N:G204P,N:S235F,ORF1a:T1001I,ORF1a:A1708D,ORF1a:A2128T,ORF1a:I2230T,ORF1b:P314L,ORF1b:T385M,ORF1b:R2684S,ORF3a:H78Y,ORF7a:T115I,ORF8:Q27*,ORF8:R52I,ORF8:K68*,ORF8:Y73C,S:F275L,S:N501Y,S:A570D,S:D614G,S:P681H,S:T716I,S:Q836H,S:S982A,S:D1118H	6	848
hCoV-19/Mexico/CMX-IBT_IMSS_1898/2021	EPI_ISL_2801765	OL461344	B.1.1.7	GRY	43	C241T,C913T,C3037T,C3267T,C5388A,C5944T,C5986T,G6647A,C6936T,T6954C,C14408T,C14621T,C14676T,T15096C,C15279T,T16176C,C17676T,C18183T,C19524T,A21519C,A23063T,C23271A,A23403G,C23604A,C23709T,A24070T,A24092G,T24506G,G24914C,C25624T,C27737T,C27972T,G28048T,A28095T,A28111G,G28280C,A28281T,T28282A,G28881A,G28882A,G28883C,G28	27	N:D3L,N:R203K,N:G204P,N:S235F,ORF1a:T1001I,ORF1a:A1708D,ORF1a:A2128T,ORF1a:S2224F,ORF1a:I2230T,ORF1b:P314L,ORF1b:T385M,ORF1b:R2684S,ORF3a:H78Y,ORF7a:T115I,ORF8:Q27*,ORF8:R52I,ORF8:K68*,ORF8:Y73C,S:N501Y,S:A570D,S:D614G,S:P681H,S:T716I,S:Q836H,S:I844V,S:S982A,S:D1118H	6	0
hCoV-19/Mexico/CMX-IBT_IMSS_1917/2021	EPI_ISL_2801781	OL461345	B.1.1.7	GRY	43	C241T,C913T,C3037T,C3267T,C5388A,C5944T,C5986T,G6647A,C6936T,T6954C,C14408T,C14621T,C14676T,T15096C,C15279T,T16176C,C17676T,C18183T,C19524T,A21519C,A23063T,C23271A,A23403G,C23604A,C23709T,A24070T,A24092G,T24506G,G24914C,C25624T,C27737T,C27972T,G28048T,A28095T,A28111G,G28280C,A28281T,T28282A,G28881A,G28882A,G28883C,G28	27	N:D3L,N:R203K,N:G204P,N:S235F,ORF1a:T1001I,ORF1a:A1708D,ORF1a:A2128T,ORF1a:S2224F,ORF1a:I2230T,ORF1b:P314L,ORF1b:T385M,ORF1b:R2684S,ORF3a:H78Y,ORF7a:T115I,ORF8:Q27*,ORF8:R52I,ORF8:K68*,ORF8:Y73C,S:N501Y,S:A570D,S:D614G,S:P681H,S:T716I,S:Q836H,S:I844V,S:S982A,S:D1118H	6	356
hCoV-19/Mexico/CMX-IBT_IMSS_1931/2021	EPI_ISL_2801797	OL461346	Q.3	GRY	40	C241T,C913T,C3037T,C3267T,G5023A,C5031T,C5388A,C5986T,C6568T,T6954C,G8183A,A9529G,G10396T,C14408T,C14676T,C15279T,T16176C,A17615G,G20962A,G22094C,A23063T,C23271A,A23403G,C23604A,C23709T,T24331C,T24506G,G24914C,G25555T,G26730C,C27972T,G28048T,A28111G,G28280C,A28281T,T28282A,G28881A,G28882A,G28883C,C28977T	26	M:V70L,N:D3L,N:R203K,N:G204R,N:S235F,ORF1a:T1001I,ORF1a:M1586I,ORF1a:T1589I,ORF1a:A1708D,ORF1a:I2230T,ORF1a:G2640R,ORF1b:P314L,ORF1b:K1383R,ORF1b:D2499N,ORF3a:V55F,ORF8:Q27*,ORF8:R52I,ORF8:Y73C,S:D178H,S:N501Y,S:A570D,S:D614G,S:P681H,S:T716I,S:S982A,S:D1118H	6	0
hCoV-19/Mexico/CMX-INER-IBT-0189/2021	EPI_ISL_2346447	OL461347	B.1.1.7	GRY	39	G174T,G210T,C241T,C913T,C1420T,C2453T,C3037T,C3267T,C5388A,C5986T,A6546G,T6954C,G7393T,T9016C,C14408T,T14565C,C14676T,C15279T,T16176C,T17803G,A23063T,C23271A,A23403G,C23604A,C23709T,T24506G,G24914C,C27883T,C27972T,G28048T,A28111G,G28280C,A28281T,T28282A,C28610T,G28881A,G28882A,G28883C,C28977T	22	N:D3L,N:R203K,N:G204R,N:S235F,ORF1a:L730F,ORF1a:T1001I,ORF1a:A1708D,ORF1a:D2094G,ORF1a:I2230T,ORF1b:P314L,ORF1b:S1446A,ORF7b:A43V,ORF8:Q27*,ORF8:R52I,ORF8:Y73C,S:N501Y,S:A570D,S:D614G,S:P681H,S:T716I,S:S982A,S:D1118H	6	0
hCoV-19/Mexico/CMX-INER-IBT-0191/2021	EPI_ISL_2346449	OL461348	B.1.1.7	GRY	39	T201A,C241T,C913T,C2110T,A2693T,C3037T,C3267T,C5388A,C5986T,T6954C,G7042T,A10195G,C13329T,C14120T,C14408T,C14676T,C15279T,T16176C,A23063T,C23271A,A23403G,C23604A,C23709T,T24506G,G24914C,C25046G,C25708T,C27972T,G28048T,G28075T,A28095T,A28111G,G28280C,A28281T,T28282A,G28881A,G28882A,G28883C,C28977T	26	N:D3L,N:R203K,N:G204R,N:S235F,ORF1a:N810Y,ORF1a:T1001I,ORF1a:A1708D,ORF1a:I2230T,ORF1a:M2259I,ORF1a:T4355I,ORF1b:P218L,ORF1b:P314L,ORF3a:L106F,ORF8:Q27*,ORF8:R52I,ORF8:C61F,ORF8:K68*,ORF8:Y73C,S:N501Y,S:A570D,S:D614G,S:P681H,S:T716I,S:S982A,S:D1118H,S:P1162A	6	0
hCoV-19/Mexico/CMX-INER-IBT-0192/2021	EPI_ISL_2346450	OL461349	B.1.1.7	GRY	35	C241T,T820C,C913T,C3037T,C3267T,C5388A,C5869T,C5986T,T6954C,C14408T,C14676T,T15096C,C15279T,T16176C,A23063T,C23271A,A23403G,C23604A,C23709T,T24506G,G24914C,G25785T,C27972T,G28048T,A28095T,A28111G,G28280C,A28281T,T28282A,G28881A,G28882A,G28883C,C28977T,C29272T,G29764A	20	N:D3L,N:R203K,N:G204R,N:S235F,ORF1a:T1001I,ORF1a:A1708D,ORF1a:I2230T,ORF1b:P314L,ORF3a:W131C,ORF8:Q27*,ORF8:R52I,ORF8:K68*,ORF8:Y73C,S:N501Y,S:A570D,S:D614G,S:P681H,S:T716I,S:S982A,S:D1118H	6	0
hCoV-19/Mexico/CMX-INER-IBT-0226/2021	EPI_ISL_2490585	OL461350	B.1.1.7	GRY	40	C241T,C292T,C398T,C913T,C3037T,C3267T,C5388A,C5884T,C5986T,T6954C,T9292C,T9293A,C14408T,C14676T,C15279T,T16176C,T16563A,T17343C,A23063T,C23170T,C23271A,A23403G,C23604A,C23709T,T24506G,G24914C,C25626T,C27972T,G28048T,A28111G,G28280C,A28281T,T28282A,A28295C,G28881A,G28882A,G28883C,C28977T,C29200T,C29420T	23	N:D3L,N:N8H,N:R203K,N:G204R,N:S235F,N:P383S,ORF1a:H45Y,ORF1a:T1001I,ORF1a:A1708D,ORF1a:I2230T,ORF1a:Y3010N,ORF1b:P314L,ORF8:Q27*,ORF8:R52I,ORF8:Y73C,ORF9b:K4N,S:N501Y,S:A570D,S:D614G,S:P681H,S:T716I,S:S982A,S:D1118H	6	0
hCoV-19/Mexico/CMX-INER-IBT-0244/2021	EPI_ISL_2648114	OL461351	B.1.1.7	GRY	36	C241T,C913T,C2110T,C3037T,C3267T,C5388A,C5986T,T6736C,T6954C,G7042T,C14120T,C14408T,C14676T,C15279T,T16176C,C17762T,A23063T,C23271A,G23311T,A23403G,C23604A,C23709T,T24506G,G24914C,C27972T,G28048T,A28095T,A28111G,G28280C,A28281T,T28282A,G28881A,G28882A,G28883C,C28977T,C29555T	23	N:D3L,N:R203K,N:G204R,N:S235F,ORF1a:T1001I,ORF1a:A1708D,ORF1a:I2230T,ORF1a:M2259I,ORF1b:P218L,ORF1b:P314L,ORF1b:A1432V,ORF8:Q27*,ORF8:R52I,ORF8:K68*,ORF8:Y73C,S:N501Y,S:A570D,S:E583D,S:D614G,S:P681H,S:T716I,S:S982A,S:D1118H	6	0
hCoV-19/Mexico/CMX-INER-IBT-0249/2021	EPI_ISL_2648118	OL461352	B.1.1.7	GRY	37	C241T,C913T,C3037T,C3267T,A3492G,C4331T,C5388A,C5986T,T6954C,C13423A,C13424A,C14408T,C14676T,C15279T,T16176C,C18555T,C21639A,A23063T,C23271A,A23403G,A23523C,C23604A,C23709T,C24374T,T24506G,G24914C,C27972T,G28048T,A28111G,G28280C,A28281T,T28282A,G28881A,G28882A,G28883C,C28977T,C29732T	23	N:D3L,N:R203K,N:G204R,N:S235F,ORF1a:T1001I,ORF1a:N1076S,ORF1a:A1708D,ORF1a:I2230T,ORF1a:R4387S,ORF1b:P314L,ORF8:Q27*,ORF8:R52I,ORF8:Y73C,S:P26H,S:N501Y,S:A570D,S:D614G,S:E654A,S:P681H,S:T716I,S:L938F,S:S982A,S:D1118H	6	0
hCoV-19/Mexico/CMX-INER-IBT-0250/2021	EPI_ISL_2648119	OL461353	B.1.1.7	GRY	37	C241T,C913T,C1943T,A2015G,C2110T,C3037T,C3267T,C5388A,C5986T,T6954C,T7984C,C14120T,C14408T,C14676T,C15279T,T16176C,C19390T,C22413T,A23063T,C23271A,A23403G,C23604A,C23709T,T24506G,G24914C,C25207T,C27972T,G28048T,A28095T,A28111G,G28280C,A28281T,T28282A,G28881A,G28882A,G28883C,C28977T	24	N:D3L,N:R203K,N:G204R,N:S235F,ORF1a:R560C,ORF1a:M584V,ORF1a:T1001I,ORF1a:A1708D,ORF1a:I2230T,ORF1b:P218L,ORF1b:P314L,ORF1b:P1975S,ORF8:Q27*,ORF8:R52I,ORF8:K68*,ORF8:Y73C,S:T284I,S:N501Y,S:A570D,S:D614G,S:P681H,S:T716I,S:S982A,S:D1118H	6	0
hCoV-19/Mexico/CMX-INER-IBT-0273/2021	EPI_ISL_2777228	OL461354	B.1.1.7	GRY	39	C241T,C913T,C2110T,A2206C,C3037T,C3267T,C5388A,C5986T,T6954C,G7042T,A10195G,C11750T,C14120T,C14408T,C14676T,C15279T,T16176C,G18674A,C20081T,A23063T,C23271A,A23403G,C23604A,C23709T,T24379C,T24506G,G24914C,C27972T,G28048T,G28075T,A28095T,A28111G,G28280C,A28281T,T28282A,G28881A,G28882A,G28883C,C28977T	26	N:D3L,N:R203K,N:G204R,N:S235F,ORF1a:E647D,ORF1a:T1001I,ORF1a:A1708D,ORF1a:I2230T,ORF1a:M2259I,ORF1a:L3829F,ORF1b:P218L,ORF1b:P314L,ORF1b:R1736K,ORF1b:S2205F,ORF8:Q27*,ORF8:R52I,ORF8:C61F,ORF8:K68*,ORF8:Y73C,S:N501Y,S:A570D,S:D614G,S:P681H,S:T716I,S:S982A,S:D1118H	6	0
hCoV-19/Mexico/CMX-LANGEBIO_IMSS_1049/2021	EPI_ISL_2671526	OL461355	B.1.1.7	GRY	38	G101T,C241T,C913T,C2143T,C3037T,C3267T,C5388A,C5986T,T6954C,C10228T,C14408T,C14676T,T15096C,C15279T,T16176C,A23063T,C23271A,A23403G,C23604A,C23709T,G24089A,T24506G,G24914C,G25785T,C27476T,C27972T,G28048T,A28095T,A28111G,G28280C,A28281T,T28282A,A28466G,G28881A,G28882A,G28883C,C28977T,C29642T	23	N:D3L,N:K65E,N:R203K,N:G204R,N:S235F,ORF1a:T1001I,ORF1a:A1708D,ORF1a:I2230T,ORF1b:P314L,ORF3a:W131C,ORF7a:T28I,ORF8:Q27*,ORF8:R52I,ORF8:K68*,ORF8:Y73C,S:N501Y,S:A570D,S:D614G,S:P681H,S:T716I,S:D843N,S:S982A,S:D1118H	6	0
hCoV-19/Mexico/CMX-LANGEBIO_IMSS_1069/2021	EPI_ISL_2671531	OL461356	B.1.1.7	GRY	34	C241T,C913T,C2788T,C3037T,C3267T,C5388A,C5986T,C6196T,T6954C,A8848G,C14408T,C14676T,C15279T,T16176C,A23063T,C23271A,A23403G,C23604A,C23709T,T24506G,G24914C,G25217T,G26727T,C27972T,G28048T,A28111G,G28280C,A28281T,T28282A,G28881A,G28882A,G28	20	M:A69S,N:D3L,N:R203K,N:G204R,N:S235F,ORF1a:T1001I,ORF1a:A1708D,ORF1a:I2230T,ORF1b:P314L,ORF8:Q27*,ORF8:R52I,ORF8:Y73C,S:N501Y,S:A570D,S:D614G,S:P681H,S:T716I,S:S982A,S:D1118H,S:G1219C	6	1

hCoV-19/Mexico/CMX-LANGEBIO_IMSS_1117/2021	EPI_ISL_2671536	OL461357	B.1.1.7	GRY	33	C241T,C913T,C3037T,C3267T,C5388A,C5986T,C6196T,T6954C,A8848G,C14408T,C15279T,T16176C,A23063T,C23271A,A23403G,C23604A,C23709T,C24370T,T24506G,G24914C,G25217T,G26727T,C27972T,G28048T,A28111G,G28280C,A28281T,T28282A,G28881A,G28882A,G28883C,C2	20	M:A69S,N:D3L,N:R203K,N:G204R,N:S235F,ORF1a:T1001I,ORF1a:A1708D,ORF1a:I2230T,ORF1b:P314L,ORF8:Q27*,ORF8:R52I,ORF8:Y73C,S:N501Y,S:A570D,S:D614G,S:P681H,S:T716I,S:S982A,S:D1118H,S:G1219C	6	402
hCoV-19/Mexico/CMX-LANGEBIO_IMSS_1122/2021	EPI_ISL_2671538	OL461358	B.1.1.7	GRY	38	C241T,C913T,C3037T,C3267T,C4832T,C5388A,C5986T,C6196T,T6954C,A8848G,C10078T,C12747T,C14408T,C14676T,C15279T,T16176C,A23063T,C23271A,A23403G,C23604A,C23709T,T24506G,G24914C,G25217T,G26727T,C27972T,G28048T,A28111G,G28280C,A28281T,T28282A,G28541T,G28881A,G28882A,G28883C,C28977T,G29715T,C29750T	23	M:A69S,N:D3L,N:A90S,N:R203K,N:G204R,N:S235F,ORF1a:T1001I,ORF1a:A1708D,ORF1a:I2230T,ORF1a:T4161I,ORF1b:P314L,ORF8:Q27*,ORF8:R52I,ORF8:Y73C,ORF9b:E86D,S:N501Y,S:A570D,S:D614G,S:P681H,S:T716I,S:S982A,S:D1118H,S:G1219C	6	23
hCoV-19/Mexico/CMX-LANGEBIO_IMSS_1461/2021	EPI_ISL_2942580	OL461359	B.1.1.7	GRY	39	C913T,A2317T,C2453T,C3037T,C3267T,C5388A,C5986T,C6433T,T6954C,C9611T,C11750T,C14408T,C14676T,C15279T,T16176C,A18517C,C21008T,C21855T,G21974C,A23063T,C23271A,A23403G,C23604A,C23709T,T24506G,G24914C,C27643T,C27972T,T27977C,G28048T,A28111G,G28280C,A28281T,T28282A,A28295G,G28881A,G28882A,G28883C,C28977T	28	N:D3L,N:N8D,N:R203K,N:G204R,N:S235F,ORF1a:K684N,ORF1a:L730F,ORF1a:T1001I,ORF1a:A1708D,ORF1a:I2230T,ORF1a:L3116F,ORF1a:L3829F,ORF1b:P314L,ORF1b:N1684H,ORF1b:T2514I,ORF7a:P84S,ORF8:Q27*,ORF8:R52I,ORF8:Y73C,S:S98F,S:D138H,S:N501Y,S:A570D,S:D614G,S:P681H,S:T716I,S:S982A,S:D	6	114
hCoV-19/Mexico/CMX-LANGEBIO_IMSS_1463/2021	EPI_ISL_2942582	OL461360	B.1.1.7	GRY	35	C241T,C913T,C3037T,C3267T,C5388A,C5986T,T6954C,C7299T,C11750T,C14408T,C14676T,T15096C,C15279T,T16176C,C16222T,A23063T,C23271A,A23403G,C23604A,C23709T,T24506G,G24914C,G25489T,C27972T,G28048T,A28095T,A28111G,G28280C,A28281T,T28282A,G28881A,G28882A,G28883C,G28913A,C28977T	24	N:D3L,N:R203K,N:G204R,N:G214S,N:S235F,ORF1a:T1001I,ORF1a:A1708D,ORF1a:I2230T,ORF1a:A2345V,ORF1a:L3829F,ORF1b:P314L,ORF1b:H919Y,ORF3a:A33S,ORF8:Q27*,ORF8:R52I,ORF8:K68*,ORF8:Y73C,S:N501Y,S:A570D,S:D614G,S:P681H,S:T716I,S:S982A,S:D1118H	6	0
hCoV-19/Mexico/CMX-LANGEBIO_IMSS_1466/2021	EPI_ISL_2942584	OL461361	B.1.1.7	GRY	43	T78G,C241T,C913T,C3037T,C3267T,C5388A,C5986T,G6647A,T6954C,A10486G,C14408T,C14676T,T15096C,C15279T,T16176C,C17676T,C18183T,C19524T,A21519C,G22217A,A23063T,C23271A,A23403G,C23604A,C23709T,A24070T,T24506G,G24914C,C25624T,G26918T,T27384C,C27406T,C27972T,G28048T,A28111G,G28280C,A28281T,T28282A,G28881A,G28882A,G28883C,G2	24	N:D3L,N:R203K,N:G204P,N:S235F,ORF1a:T1001I,ORF1a:A1708D,ORF1a:A2128T,ORF1a:I2230T,ORF1b:P314L,ORF1b:R2684S,ORF3a:H78Y,ORF7a:L5F,ORF8:Q27*,ORF8:R52I,ORF8:Y73C,S:G219S,S:N501Y,S:A570D,S:D614G,S:P681H,S:T716I,S:Q836H,S:S982A,S:D1118H	6	1
hCoV-19/Mexico/COA_IBT_IMSS_1400/2021	EPI_ISL_2681095	OL461362	B.1.1.7	GRY	37	G219T,C241T,C763T,C913T,C2453T,C3037T,C3267T,C5388A,C5986T,T6954C,C9611T,C14408T,C14676T,C15279T,T16176C,C21855T,G21974C,A23063T,C23271A,A23403G,C23604A,C23709T,T24506G,G24914C,G25273T,C27329T,C27972T,G28048T,A28111G,G28280C,A28281T,T28282A,A28295G,G28881A,G28882A,G28883C,C28977T	25	N:D3L,N:N8D,N:R203K,N:G204R,N:S235F,ORF1a:L730F,ORF1a:T1001I,ORF1a:A1708D,ORF1a:I2230T,ORF1a:L3116F,ORF1b:P314L,ORF6:S43L,ORF8:Q27*,ORF8:R52I,ORF8:Y73C,S:S98F,S:D138H,S:N501Y,S:A570D,S:D614G,S:P681H,S:T716I,S:S982A,S:D1118H,S:M1237I	6	0
hCoV-19/Mexico/COA_INER_IMSS_1085/2021	EPI_ISL_2490340	OL461363	B.1.1.7	GRY	38	C241T,C913T,C2110T,C3037T,C3267T,C5388A,C5986T,T6954C,G7042T,A10195G,C11224T,C13923T,C14120T,C14408T,C14676T,C15279T,T16176C,C20031T,G22131A,A23063T,C23271A,A23403G,C23604A,C23709T,T24506G,G24914C,C27972T,G28048T,G28075T,A28095T,A28111G,G28280C,A28281T,T28282A,G28881A,G28882A,G28883C,C28977T	23	N:D3L,N:R203K,N:G204R,N:S235F,ORF1a:T1001I,ORF1a:A1708D,ORF1a:I2230T,ORF1a:M2259I,ORF1b:P218L,ORF1b:P314L,ORF8:Q27*,ORF8:R52I,ORF8:C61F,ORF8:K68*,ORF8:Y73C,S:R190K,S:N501Y,S:A570D,S:D614G,S:P681H,S:T716I,S:S982A,S:D1118H	6	0
hCoV-19/Mexico/COA_LANGEBIO_IMSS_0535/2021	EPI_ISL_2401962	OL461364	B.1.1.7	GRY	36	C241T,C913T,C2110T,C3037T,C3267T,C5388A,C5986T,T6954C,G7042T,A10195G,C14120T,C14408T,C14676T,C15279T,C15933T,T16176C,A23063T,C23271A,A23403G,C23604A,C23709T,T24506G,G24914C,A27745G,C27972T,G28048T,G28075T,A28095T,A28111G,G28280C,A28281T,T28282A,G28881A,G28882A,G28883C,C28977T	23	N:D3L,N:R203K,N:G204R,N:S235F,ORF1a:T1001I,ORF1a:A1708D,ORF1a:I2230T,ORF1a:M2259I,ORF1b:P218L,ORF1b:P314L,ORF7a:R118G,ORF8:Q27*,ORF8:R52I,ORF8:C61F,ORF8:K68*,ORF8:Y73C,S:N501Y,S:A570D,S:D614G,S:P681H,S:T716I,S:S982A,S:D1118H	6	331
hCoV-19/Mexico/COA_LANGEBIO_IMSS_0575/2021	EPI_ISL_2401995	OL461365	B.1.1.7	GRY	34	C241T,C913T,G2144A,C2453T,C3037T,C3267T,C5388A,C5986T,T6954C,C9611T,C14408T,C14676T,C15279T,T16176C,G18651T,C21855T,A23063T,C23271A,A23403G,C23604A,C23709T,T24506G,G24914C,C27972T,G28048T,A28111G,G28280C,A28281T,T28282A,A28295G,G28881A,G28882A,G28883C,C28977T	24	N:D3L,N:N8D,N:R203K,N:G204R,N:S235F,ORF1a:V627I,ORF1a:L730F,ORF1a:T1001I,ORF1a:A1708D,ORF1a:I2230T,ORF1a:L3116F,ORF1b:P314L,ORF1b:E1728D,ORF8:Q27*,ORF8:R52I,ORF8:Y73C,S:S98F,S:N501Y,S:A570D,S:D614G,S:P681H,S:T716I,S:S982A,S:D1118H	6	1
hCoV-19/Mexico/COA_LANGEBIO_IMSS_0592/2021	EPI_ISL_2402011	OL461366	B.1.1.7	GRY	38	C241T,C913T,C2110T,C3037T,C3267T,C5388A,C5986T,T6954C,G7042T,A10195G,C14120T,C14408T,C14676T,C15279T,C15933T,T16176C,C21597T,A23063T,C23271A,A23403G,C23604A,C23709T,T24506G,G24914C,G25855T,A27745G,C27972T,G28048T,G28075T,A28095T,A28111G,G28280C,A28281T,T28282A,G28881A,G28882A,G28883C,C28977T	25	N:D3L,N:R203K,N:G204R,N:S235F,ORF1a:T1001I,ORF1a:A1708D,ORF1a:I2230T,ORF1a:M2259I,ORF1b:P218L,ORF1b:P314L,ORF3a:D155Y,ORF7a:R118G,ORF8:Q27*,ORF8:R52I,ORF8:C61F,ORF8:K68*,ORF8:Y73C,S:S12F,S:N501Y,S:A570D,S:D614G,S:P681H,S:T716I,S:S982A,S:D1118H	6	34
hCoV-19/Mexico/COA_LANGEBIO_IMSS_0594/2021	EPI_ISL_2402013	OL461367	B.1.1.7	GRY	33	C241T,C913T,C3037T,C3267T,C5388A,C5986T,T6954C,C14408T,C14676T,C15279T,T16176C,A21583G,A23063T,C23271A,A23403G,C23604A,C23709T,T24506G,G24914C,C26833T,T27292C,C27972T,G28048T,C28059A,A28111G,G28280C,A28281T,T28282A,G28881A,G28882A,G28883C	21	M:A104V,N:D3L,N:R203K,N:G204R,N:S235F,ORF1a:T1001I,ORF1a:A1708D,ORF1a:I2230T,ORF1b:P314L,ORF6:Y31H,ORF8:Q27*,ORF8:R52I,ORF8:P56T,ORF8:Y73C,S:N501Y,S:A570D,S:D614G,S:P681H,S:T716I,S:S982A,S:D1118H	6	444
hCoV-19/Mexico/COA_LANGEBIO_IMSS_0595/2021	EPI_ISL_2402014	OL461368	B.1.1.7	GRY	33	C241T,C913T,C2453T,C3037T,C3267T,C5388A,C5986T,T6954C,A9424G,C9611T,C14408T,C14676T,C15279T,T16176C,C21855T,A23063T,C23271A,A23403G,C23604A,C23709T,T24506G,G24914C,C27972T,G28048T,A28111G,G28280C,A28281T,T28282A,A28295G,G28881A,G28882A,G28	22	N:D3L,N:N8D,N:R203K,N:G204R,N:S235F,ORF1a:L730F,ORF1a:T1001I,ORF1a:A1708D,ORF1a:I2230T,ORF1a:L3116F,ORF1b:P314L,ORF8:Q27*,ORF8:R52I,ORF8:Y73C,S:S98F,S:N501Y,S:A570D,S:D614G,S:P681H,S:T716I,S:S982A,S:D111	6	218
hCoV-19/Mexico/COA_LANGEBIO_IMSS_69504-NC/2021	EPI_ISL_2969925	OL461369	B.1.1.7	GRY	35	C913T,C3037T,A3255G,C3267T,C5388A,C5986T,T6001C,G6653T,T6954C,A12555G,C14408T,C14676T,C15279T,T16176C,C18348T,A23063T,C23271A,A23403G,C23604A,C23709T,T24506G,G24914C,G24971A,C27972T,G28048T,A28111G,G28280C,A28281T,T28282A,G28881A,G28882A,G28883C,C28977T,C29632T,G29781T	22	N:D3L,N:R203K,N:G204R,N:S235F,ORF1a:N997S,ORF1a:T1001I,ORF1a:A1708D,ORF1a:V2130F,ORF1a:I2230T,ORF1a:E4097G,ORF1b:P314L,ORF8:Q27*,ORF8:R52I,ORF8:Y73C,S:N501Y,S:A570D,S:D614G,S:P681H,S:T716I,S:S982A,S:D1118H,S:V1137I	6	986
hCoV-19/Mexico/COA-IBT_IMSS_1820/2021	EPI_ISL_2801696	OL461370	B.1.1.7	GRY	36	C241T,C683T,C913T,C3037T,A3255G,C3267T,C5388A,C5986T,T6001C,G6653T,T6954C,A12555G,C14408T,C14676T,C15279T,T16176C,C18348T,A23063T,C23271A,A23403G,C23604A,C23709T,T24506G,G24914C,G24971A,C27972T,G28048T,A28111G,G28280C,A28281T,T28282A,G28881A,G28882A,G28883C,C28977T,C29632T	22	N:D3L,N:R203K,N:G204R,N:S235F,ORF1a:N997S,ORF1a:T1001I,ORF1a:A1708D,ORF1a:V2130F,ORF1a:I2230T,ORF1a:E4097G,ORF1b:P314L,ORF8:Q27*,ORF8:R52I,ORF8:Y73C,S:N501Y,S:A570D,S:D614G,S:P681H,S:T716I,S:S982A,S:D1118H,S:V1137I	6	743
hCoV-19/Mexico/COA-LANGEBIO_IMSS_0874/2021	EPI_ISL_2671546	OL461371	B.1.1.7	GRY	39	T78G,C913T,C3037T,C3267T,C5388A,C5986T,T6954C,G7042T,C7765T,A10195G,C12880T,C14120T,C14408T,C14676T,C15279T,C15933T,T16176C,G22104C,A23063T,C23271A,A23403G,C23604A,C23709T,T24506G,G24914C,A27745G,C27972T,G28048T,G28075T,A28095T,A28111G,G28280C,A28281T,T28282A,G28881A,G28882A,G28883C,C28977T,G29810T	24	N:D3L,N:R203K,N:G204R,N:S235F,ORF1a:T1001I,ORF1a:A1708D,ORF1a:I2230T,ORF1a:M2259I,ORF1b:P218L,ORF1b:P314L,ORF7a:R118G,ORF8:Q27*,ORF8:R52I,ORF8:C61F,ORF8:K68*,ORF8:Y73C,S:G181A,S:N501Y,S:A570D,S:D614G,S:P681H,S:T716I,S:S982A,S:D1118H	6	411
hCoV-19/Mexico/COA-LANGEBIO_IMSS_1345/2021	EPI_ISL_2942482	OL461372	B.1.1.7	GRY	35	C241T,C913T,C3037T,A3255G,C3267T,C5388A,C5986T,T6001C,G6653T,T6954C,A12555G,C14408T,C14676T,C15279T,T16176C,C18348T,A23063T,C23271A,A23403G,C23604A,C23709T,T24506G,G24914C,G24971A,C27972T,G28048T,A28111G,G28280C,A28281T,T28282A,G28881A,G28882A,G28883C,C28977T,C29632T	22	N:D3L,N:R203K,N:G204R,N:S235F,ORF1a:N997S,ORF1a:T1001I,ORF1a:A1708D,ORF1a:V2130F,ORF1a:I2230T,ORF1a:E4097G,ORF1b:P314L,ORF8:Q27*,ORF8:R52I,ORF8:Y73C,S:N501Y,S:A570D,S:D614G,S:P681H,S:T716I,S:S982A,S:D1118H,S:V1137I	6	1
hCoV-19/Mexico/COA-LANGEBIO_IMSS_1377/2021	EPI_ISL_2942505	OL461373	B.1.1.7	GRY	41	C913T,C2110T,C3037T,C3267T,C5388A,C5986T,T6954C,G7042T,C7765T,C10156T,A10195G,C11199T,C12880T,C14120T,C14408T,C14676T,C15279T,C15933T,T16176C,G22104C,A23063T,C23271A,A23403G,C23604A,C23709T,T24506G,G24914C,A27745G,C27972T,G28048T,G28075T,A28095T,A28111G,G28280C,A28281T,T28282A,G28881A,G28882A,G28883C,C28977T,G29810T	25	N:D3L,N:R203K,N:G204R,N:S235F,ORF1a:T1001I,ORF1a:A1708D,ORF1a:I2230T,ORF1a:M2259I,ORF1a:A3645V,ORF1b:P218L,ORF1b:P314L,ORF7a:R118G,ORF8:Q27*,ORF8:R52I,ORF8:C61F,ORF8:K68*,ORF8:Y73C,S:G181A,S:N501Y,S:A570D,S:D614G,S:P681H,S:T716I,S:S982A,S:D1118H	6	266
hCoV-19/Mexico/COA-LANGEBIO_IMSS_1381/2021	EPI_ISL_2942509	OL461374	B.1.1.7	GRY	39	G94T,C241T,C913T,C3037T,A3255G,C3267T,C5388A,C5986T,T6001C,G6653T,T6954C,A12555G,C12921T,C14408T,C14676T,C15279T,T16176C,C18348T,A23063T,C23271A,A23403G,C23604A,C23709T,T24506G,G24914C,G24971A,A25998G,C27972T,G28048T,A28111G,G28280C,A28281T,T28282A,G28727T,G28881A,G28882A,G28883C,C28977T,C29632T	24	N:D3L,N:A152S,N:R203K,N:G204R,N:S235F,ORF1a:N997S,ORF1a:T1001I,ORF1a:A1708D,ORF1a:V2130F,ORF1a:I2230T,ORF1a:E4097G,ORF1a:T4219I,ORF1b:P314L,ORF8:Q27*,ORF8:R52I,ORF8:Y73C,S:N501Y,S:A570D,S:D614G,S:P681H,S:T716I,S:S982A,S:D1118H,S:V1137I	6	0
hCoV-19/Mexico/COA-LANGEBIO_IMSS_1672/2021	EPI_ISL_2942765	OL461375	B.1.1.7	GRY	35	C913T,C3037T,A3255G,C3267T,C5388A,C5986T,T6001C,G6653T,T6954C,A12555G,C14408T,C14676T,C15279T,T16176C,C18348T,C22677T,A23063T,C23271A,A23403G,C23604A,C23709T,T24506G,G24914C,G24971A,C27972T,G28048T,A28111G,G28280C,A28281T,T28282A,G28881A,G28882A,G28883C,C28977T,C29632T	23	N:D3L,N:R203K,N:G204R,N:S235F,ORF1a:N997S,ORF1a:T1001I,ORF1a:A1708D,ORF1a:V2130F,ORF1a:I2230T,ORF1a:E4097G,ORF1b:P314L,ORF8:Q27*,ORF8:R52I,ORF8:Y73C,S:A372V,S:N501Y,S:A570D,S:D614G,S:P681H,S:T716I,S:S982A,S:D1118H,S:V1137I	6	489
hCoV-19/Mexico/COA-LANGEBIO_IMSS_1675/2021	EPI_ISL_2942768	OL461376	B.1.1.7	GRY	36	C241T,C913T,C3037T,A3255G,C3267T,C5388A,C5986T,T6001C,G6653T,T6954C,A12555G,C14408T,C14676T,C15279T,T16176C,C18348T,A23063T,C23271A,A23403G,C23604A,C23709T,T24506G,G24914C,G24971A,A25998G,C27972T,G28048T,A28111G,G28280C,A28281T,T28282A,G28881A,G28882A,G28883C,C28977T,C29632T	22	N:D3L,N:R203K,N:G204R,N:S235F,ORF1a:N997S,ORF1a:T1001I,ORF1a:A1708D,ORF1a:V2130F,ORF1a:I2230T,ORF1a:E4097G,ORF1b:P314L,ORF8:Q27*,ORF8:R52I,ORF8:Y73C,S:N501Y,S:A570D,S:D614G,S:P681H,S:T716I,S:S982A,S:D1118H,S:V1137I	6	49

hCoV-19/Mexico/COA-LANGEBIO_IMSS_1696/2021	EPI_ISL_2942785	OL461377	B.1.1.7	GRY	38	C241T,C913T,C1380T,C3037T,A3255G,C3267T,C5388A,C5986T,T6001C,G6653T,T6954C,A12555G,C14408T,C14676T,C15279T,T16176C,C17745T,C18348T,A23063T,C23271A,A23403G,C23604A,C23709T,T24506G,G24914C,G24971A,C27972T,G28048T,A28111G,G28280C,A28281T,T28282A,G28881A,G28882A,G28883C,C28961T,C28977T,C29632T	24	N:D3L,N:R203K,N:G204R,N:L230F,N:S235F,ORF1a:A372V,ORF1a:N997S,ORF1a:T1001I,ORF1a:A1708D,ORF1a:V2130F,ORF1a:I2230T,ORF1a:E4097G,ORF1b:P314L,ORF8:Q27*,ORF8:R52I,ORF8:Y73C,S:N501Y,S:A570D,S:D614G,S:P681H,S:T716I,S:S982A,S:D1118H,S:V1137I	6	137
hCoV-19/Mexico/COL_LANGEBIO_IMSS_0767/2021	EPI_ISL_2402178	OL461378	B.1.1.7	GRY	39	C203T,C241T,C913T,G1820A,C3037T,C3267T,C5388A,C5986T,T6954C,G9526T,C11747T,A12162G,C14408T,C14676T,C15279T,C15720T,T16176C,A17615G,T19363C,A21137G,A23063T,C23248T,C23271A,A23403G,C23604A,C23709T,T24506G,G24914C,G25135T,C27972T,G28048T,A28111G,G28280C,A28281T,T28282A,G28881A,G28882A,G28883C,C28977T	24	N:D3L,N:R203K,N:G204R,N:S235F,ORF1a:G519S,ORF1a:T1001I,ORF1a:A1708D,ORF1a:I2230T,ORF1a:M3087I,ORF1a:Q3966R,ORF1b:P314L,ORF1b:K1383R,ORF1b:K2557R,ORF8:Q27*,ORF8:R52I,ORF8:Y73C,S:N501Y,S:A570D,S:D614G,S:P681H,S:T716I,S:S982A,S:D1118H,S:K1191N	6	1
hCoV-19/Mexico/COL-IBT_IMSS_1847/2021	EPI_ISL_2801720	OL461379	B.1.1.7	GRY	41	C241T,C673T,C913T,C3037T,C3267T,C5388A,C5986T,T6954C,A12162G,A13169G,C14408T,C14676T,C15279T,C15720T,T16176C,A17615G,C18747T,C20844T,A21137G,A23063T,C23271A,A23403G,C23604A,C23709T,T24506G,G24914C,G25135T,C26110T,G26763T,C27972T,G28048T,A28111G,G28280C,A28281T,T28282A,G28881A,G28882A,G28883C,C28977T,C29095T,G29711T	25	M:A81S,N:D3L,N:R203K,N:G204R,N:S235F,ORF1a:T1001I,ORF1a:A1708D,ORF1a:I2230T,ORF1a:Q3966R,ORF1a:T4302A,ORF1b:P314L,ORF1b:K1383R,ORF1b:K2557R,ORF3a:P240S,ORF8:Q27*,ORF8:R52I,ORF8:Y73C,S:N501Y,S:A570D,S:D614G,S:P681H,S:T716I,S:S982A,S:D1118H,S:K1191N	6	0
hCoV-19/Mexico/COL-IBT_IMSS_1868/2021	EPI_ISL_2801737	OL461380	B.1.1.7	GRY	41	C241T,C673T,C913T,C3037T,C3267T,C5388A,C5986T,T6954C,C11514T,A12162G,C12896T,C14408T,C14676T,C15279T,C15720T,T16176C,A17615G,C18747T,G20683A,C20844T,A21137G,A23063T,C23271A,A23403G,C23604A,C23709T,T24506G,G24914C,G25135T,C26110T,C27972T,G28048T,A28111G,G28280C,A28281T,T28282A,G28881A,G28882A,G28883C,C28977T,C29095T	26	N:D3L,N:R203K,N:G204R,N:S235F,ORF1a:T1001I,ORF1a:A1708D,ORF1a:I2230T,ORF1a:T3750I,ORF1a:Q3966R,ORF1a:P4211S,ORF1b:P314L,ORF1b:K1383R,ORF1b:V2406I,ORF1b:K2557R,ORF3a:P240S,ORF8:Q27*,ORF8:R52I,ORF8:Y73C,S:N501Y,S:A570D,S:D614G,S:P681H,S:T716I,S:S982A,S:D1118H,S:K1191N	6	0
hCoV-19/Mexico/COL-LANGEBIO_IMSS_1011/2021	EPI_ISL_2671550	OL461381	B.1.1.7	GRY	40	C241T,C673T,C913T,C3037T,C3267T,C5388A,C5986T,T6954C,A12162G,C14408T,C14676T,C15279T,C15720T,T16176C,A17615G,C18747T,C20844T,A21137G,A23063T,C23271A,A23403G,C23604A,C23709T,T24506G,G24914C,G25135T,C25624T,C26110T,C27972T,G28048T,A28111G,G28280C,A28281T,T28282A,G28881A,G28882A,G28883C,C28977T,C29095T,G29468C	25	N:D3L,N:R203K,N:G204R,N:S235F,N:D399H,ORF1a:T1001I,ORF1a:A1708D,ORF1a:I2230T,ORF1a:Q3966R,ORF1b:P314L,ORF1b:K1383R,ORF1b:K2557R,ORF3a:H78Y,ORF3a:P240S,ORF8:Q27*,ORF8:R52I,ORF8:Y73C,S:N501Y,S:A570D,S:D614G,S:P681H,S:T716I,S:S982A,S:D1118H,S:K1191N	6	0
hCoV-19/Mexico/COL-LANGEBIO_IMSS_1033/2021	EPI_ISL_2671554	OL461382	B.1.1.7	GRY	39	C241T,C673T,C913T,C3037T,C3267T,C5388A,C5986T,T6954C,A10323G,A12162G,C14408T,C14676T,C15279T,C15720T,T16176C,A17615G,C18747T,C20844T,A21137G,A23063T,C23271A,A23403G,C23604A,C23709T,T24506G,G24914C,G25135T,C26110T,C27972T,G28048T,A28111G,G28280C,A28281T,T28282A,G28881A,G28882A,G28883C,C28977T,C29095T	24	N:D3L,N:R203K,N:G204R,N:S235F,ORF1a:T1001I,ORF1a:A1708D,ORF1a:I2230T,ORF1a:K3353R,ORF1a:Q3966R,ORF1b:P314L,ORF1b:K1383R,ORF1b:K2557R,ORF3a:P240S,ORF8:Q27*,ORF8:R52I,ORF8:Y73C,S:N501Y,S:A570D,S:D614G,S:P681H,S:T716I,S:S982A,S:D1118H,S:K1191N	6	0
hCoV-19/Mexico/COL-LANGEBIO_IMSS_1045/2021	EPI_ISL_2671556	OL461383	B.1.1.7	GRY	41	C241T,C673T,C913T,C3037T,C3267T,C5388A,C5986T,T6954C,A12162G,C14408T,C14676T,C15279T,C15720T,T16176C,A17615G,C18747T,C20844T,A21137G,C22224T,A23063T,C23271A,A23403G,C23604A,C23709T,T24506G,G24914C,G25135T,C26110T,A26128G,C27972T,G28048T,A28111G,G28280C,A28281T,T28282A,G28881A,G28882A,G28883C,C28977T,C29095T,G29254A	25	N:D3L,N:R203K,N:G204R,N:S235F,ORF1a:T1001I,ORF1a:A1708D,ORF1a:I2230T,ORF1a:Q3966R,ORF1b:P314L,ORF1b:K1383R,ORF1b:K2557R,ORF3a:P240S,ORF3a:I246V,ORF8:Q27*,ORF8:R52I,ORF8:Y73C,S:S221L,S:N501Y,S:A570D,S:D614G,S:P681H,S:T716I,S:S982A,S:D1118H,S:K1191N	6	0
hCoV-19/Mexico/COL-LANGEBIO_IMSS_1046/2021	EPI_ISL_2671557	OL461384	B.1.1.7	GRY	39	C241T,C673T,C913T,C3037T,C3267T,C5388A,C5986T,T6954C,A12162G,C12924T,C14408T,C14676T,C15279T,C15720T,T16176C,A17615G,C18747T,C20844T,A21137G,A23063T,C23271A,A23403G,C23604A,C23709T,T24506G,G24914C,G25135T,C26110T,C27972T,G28048T,A28111G,G28280C,A28281T,T28282A,G28881A,G28882A,G28883C,C28977T,C29095T	24	N:D3L,N:R203K,N:G204R,N:S235F,ORF1a:T1001I,ORF1a:A1708D,ORF1a:I2230T,ORF1a:Q3966R,ORF1a:P4220L,ORF1b:P314L,ORF1b:K1383R,ORF1b:K2557R,ORF3a:P240S,ORF8:Q27*,ORF8:R52I,ORF8:Y73C,S:N501Y,S:A570D,S:D614G,S:P681H,S:T716I,S:S982A,S:D1118H,S:K1191N	6	9
hCoV-19/Mexico/COL-LANGEBIO_IMSS_1266/2021	EPI_ISL_2942424	OL461385	B.1.1.7	GRY	39	C673T,C913T,C3037T,C3267T,C5388A,C5986T,T6954C,A12162G,C14408T,C14676T,C15279T,C15720T,T16176C,A17615G,C18747T,G20683A,C20844T,A21137G,A23063T,C23271A,A23403G,C23604A,C23709T,T24506G,G24914C,G25135T,C26110T,C27972T,G28048T,A28111G,G28280C,A28281T,T28282A,G28881A,G28882A,G28883C,C28977T,C29095T,G29395T	25	N:D3L,N:R203K,N:G204R,N:S235F,N:K374N,ORF1a:T1001I,ORF1a:A1708D,ORF1a:I2230T,ORF1a:Q3966R,ORF1b:P314L,ORF1b:K1383R,ORF1b:V2406I,ORF1b:K2557R,ORF3a:P240S,ORF8:Q27*,ORF8:R52I,ORF8:Y73C,S:N501Y,S:A570D,S:D614G,S:P681H,S:T716I,S:S982A,S:D1118H,S:K1191N	6	228
hCoV-19/Mexico/DUR_IBT_IMSS_1409/2021	EPI_ISL_2681086	OL461386	B.1.1.7	GRY	34	C241T,C913T,C3037T,C3267T,C5388A,C5986T,A6459G,T6954C,C9611T,C14408T,C14649T,C14676T,C15279T,T16176C,C18348T,A23063T,C23271A,A23403G,C23604A,C23709T,T24506G,G24914C,C27972T,G28048T,A28111G,G28280C,A28281T,T28282A,G28881A,G28882A,G28883C,C2	20	N:D3L,N:R203K,N:G204R,N:S235F,ORF1a:T1001I,ORF1a:A1708D,ORF1a:N2065S,ORF1a:I2230T,ORF1a:L3116F,ORF1b:P314L,ORF8:Q27*,ORF8:R52I,ORF8:Y73C,S:N501Y,S:A570D,S:D614G,S:P681H,S:T716I,S:S982A,S:D1118H	6	0
hCoV-19/Mexico/DUR_INER_IMSS_1050/2021	EPI_ISL_2490339	OL461387	B.1.1.7	GRY	41	C241T,C913T,C2110T,C3037T,C3267T,C5388A,C5986T,T6954C,T7984C,T9508C,C10747T,C14120T,C14408T,C14676T,C15279T,T16176C,C16323T,C17004T,C17518T,C19390T,A23063T,C23271A,A23403G,C23604A,C23709T,T24506G,G24914C,T25729C,A27642C,C27972T,G28048T,A28095T,A28111G,C28146T,G28280C,A28281T,T28282A,G28881A,G28882A,G28883C,C28977T	24	N:D3L,N:R203K,N:G204R,N:S235F,ORF1a:T1001I,ORF1a:A1708D,ORF1a:I2230T,ORF1b:P218L,ORF1b:P314L,ORF1b:L1351F,ORF1b:P1975S,ORF3a:Y113H,ORF8:Q27*,ORF8:R52I,ORF8:K68*,ORF8:Y73C,ORF8:P85S,S:N501Y,S:A570D,S:D614G,S:P681H,S:T716I,S:S982A,S:D1118H	6	0
hCoV-19/Mexico/DUR_LANGEBIO_IMSS_0551/2021	EPI_ISL_2401974	OL461388	B.1.1.7	GRY	38	C222T,C241T,C913T,C2110T,C3037T,C3267T,C5388A,C5986T,T6954C,G7042T,C8127T,C14120T,C14408T,G14575T,C14676T,C15279T,T16176C,C17762T,C18060T,A23063T,C23271A,A23403G,C23604A,C23709T,T24506G,G24914C,T25180C,C27972T,G28048T,A28095T,A28111G,G28280C,A28281T,T28282A,G28881A,G28882A,G28883C,C28977T	24	N:D3L,N:R203K,N:G204R,N:S235F,ORF1a:T1001I,ORF1a:A1708D,ORF1a:I2230T,ORF1a:M2259I,ORF1a:A2621V,ORF1b:P218L,ORF1b:P314L,ORF1b:A370S,ORF1b:A1432V,ORF8:Q27*,ORF8:R52I,ORF8:K68*,ORF8:Y73C,S:N501Y,S:A570D,S:D614G,S:P681H,S:T716I,S:S982A,S:D1118H	6	12
hCoV-19/Mexico/DUR-LANGEBIO_IMSS_0824/2021	EPI_ISL_2671558	OL461389	B.1.1.7	GRY	33	T78G,C913T,C3037T,C3267T,T4711A,C5388A,C5986T,T6954C,C14408T,C14676T,C15279T,T16176C,C16338T,C21622T,C22120T,A23063T,C23271A,A23403G,C23604A,C23709T,C24381T,T24506G,G24914C,C27972T,G28048T,A28111G,G28280C,A28281T,T28282A,G28881A,G28882A,G2	19	N:D3L,N:R203K,N:G204R,N:S235F,ORF1a:T1001I,ORF1a:A1708D,ORF1a:I2230T,ORF1b:P314L,ORF8:Q27*,ORF8:R52I,ORF8:Y73C,S:N501Y,S:A570D,S:D614G,S:P681H,S:T716I,S:S940F,S:S982A,S:D1118H	6	234
hCoV-19/Mexico/DUR-LANGEBIO_IMSS_0870/2021	EPI_ISL_2671559	OL461390	B.1.1.7	GRY	40	C241T,C913T,C1627T,C2110T,C3037T,C3267T,C5388A,C5986T,T6954C,G7042T,A10195G,C14120T,C14408T,C14676T,C15279T,T16176C,C17762T,A23063T,C23271A,A23403G,C23604A,A23690G,C23709T,T24506G,G24914C,G25785T,T25903G,C27972T,G28048T,G28075T,A28095T,A28111G,G28280C,A28281T,T28282A,G28881A,G28882A,G28883C,C28977T,C29200T	26	N:D3L,N:R203K,N:G204R,N:S235F,ORF1a:T1001I,ORF1a:A1708D,ORF1a:I2230T,ORF1a:M2259I,ORF1b:P218L,ORF1b:P314L,ORF1b:A1432V,ORF3a:W131C,ORF3a:S171A,ORF8:Q27*,ORF8:R52I,ORF8:C61F,ORF8:K68*,ORF8:Y73C,S:N501Y,S:A570D,S:D614G,S:P681H,S:N710D,S:T716I,S:S982A,S:D1118H	6	0
hCoV-19/Mexico/DUR-LANGEBIO_IMSS_1371/2021	EPI_ISL_2942501	OL461391	B.1.1.7	GRY	38	C913T,C2110T,C3037T,C3267T,C5388A,C5986T,T6954C,T7984C,T9508C,C10747T,C14120T,C14408T,C14676T,C15279T,T16176C,C16323T,C17004T,C17518T,C19390T,A23063T,C23271A,A23403G,C23525T,C23604A,C23709T,T24506G,G24914C,C27972T,G28048T,A28111G,C28146T,G28280C,A28281T,T28282A,G28881A,G28882A,G28883C,C28977T	23	N:D3L,N:R203K,N:G204R,N:S235F,ORF1a:T1001I,ORF1a:A1708D,ORF1a:I2230T,ORF1b:P218L,ORF1b:P314L,ORF1b:L1351F,ORF1b:P1975S,ORF8:Q27*,ORF8:R52I,ORF8:Y73C,ORF8:P85S,S:N501Y,S:A570D,S:D614G,S:H655Y,S:P681H,S:T716I,S:S982A,S:D1118H	6	25
hCoV-19/Mexico/GRO-IBT_IMSS_1934/2021	EPI_ISL_2801799	OL461392	B.1.1.7	GRY	41	C241T,C913T,C3037T,C3267T,C5388A,C5944T,C5986T,G6368A,T6954C,C9967T,C13585T,C14408T,C14676T,T15096C,C15279T,T16176C,A19719T,G21901A,C22987T,C22993T,A23063T,C23271A,A23403G,C23604A,C23709T,T24506G,G24914C,G25440T,C25688T,C27972T,G28048T,A28095T,A28111G,G28280C,A28281T,T28282A,G28881A,G28882A,G28883C,G28884C,C28977T	22	N:D3L,N:R203K,N:G204P,N:S235F,ORF1a:T1001I,ORF1a:A1708D,ORF1a:G2035R,ORF1a:I2230T,ORF1b:P314L,ORF3a:K16N,ORF3a:A99V,ORF8:Q27*,ORF8:R52I,ORF8:K68*,ORF8:Y73C,S:N501Y,S:A570D,S:D614G,S:P681H,S:T716I,S:S982A,S:D1118H	6	586
hCoV-19/Mexico/GRO-IBT_IMSS_2023/2021	EPI_ISL_2801870	OL461393	B.1.1.7	GRY	41	C241T,C884T,C913T,C3037T,C3267T,A3934G,C5388A,C5986T,T6954C,A9869G,C11941T,C14408T,C14676T,C15108T,C15279T,T16176C,T16905C,A17615G,C18032T,T20273A,C22498T,A23063T,C23271A,A23403G,C23604A,C23709T,T24506G,G24914C,G26730C,C27143T,C27972T,G28048T,A28111G,G28280C,A28281T,T28282A,C28603T,G28881A,G28882A,G28883C,C28977T	24	M:V70L,N:D3L,N:R203K,N:G204R,N:S235F,ORF1a:R207C,ORF1a:T1001I,ORF1a:A1708D,ORF1a:I2230T,ORF1a:T3202A,ORF1b:P314L,ORF1b:K1383R,ORF1b:T1522I,ORF1b:M2269K,ORF8:Q27*,ORF8:R52I,ORF8:Y73C,S:N501Y,S:A570D,S:D614G,S:P681H,S:T716I,S:S982A,S:D1118H	6	0
hCoV-19/Mexico/GRO-LANGEBIO_IMSS_1391/2021	EPI_ISL_2942518	OL461394	B.1.1.7	GRY	35	C913T,C2453T,C3037T,C3267T,C3784T,C5388A,C5986T,T6954C,A7479G,C9611T,C14408T,C14676T,C15279T,T16176C,G21372T,C21855T,G21974C,A23063T,C23271A,A23403G,C23604A,C23709T,T24506G,G24914C,C27972T,G28048T,A28111G,G28280C,A28281T,T28282A,A28295G,G28881A,G28882A,G28883C,C28977T	25	N:D3L,N:N8D,N:R203K,N:G204R,N:S235F,ORF1a:L730F,ORF1a:T1001I,ORF1a:A1708D,ORF1a:I2230T,ORF1a:N2405S,ORF1a:L3116F,ORF1b:P314L,ORF1b:Q2635H,ORF8:Q27*,ORF8:R52I,ORF8:Y73C,S:S98F,S:D138H,S:N501Y,S:A570D,S:D614G,S:P681H,S:T716I,S:S982A,S:D1118H	6	32
hCoV-19/Mexico/GUA_INER_IMSS_00836/2021	EPI_ISL_2091287	OL461395	B.1.1.7	GRY	36	C241T,C913T,C2110T,C2841T,C3037T,C3267T,C5388A,C5986T,T6954C,G7042T,C14120T,C14408T,C14676T,C15279T,T16176C,T17583G,A23063T,C23271A,A23403G,C23604A,C23709T,C23987T,T24506G,G24914C,C25624T,C27972T,G28048T,A28095T,A28111G,G28280C,A28281T,T28282A,G28881A,G28882A,G28883C,C28977T	24	N:D3L,N:R203K,N:G204R,N:S235F,ORF1a:A859V,ORF1a:T1001I,ORF1a:A1708D,ORF1a:I2230T,ORF1a:M2259I,ORF1b:P218L,ORF1b:P314L,ORF3a:H78Y,ORF8:Q27*,ORF8:R52I,ORF8:K68*,ORF8:Y73C,S:N501Y,S:A570D,S:D614G,S:P681H,S:T716I,S:P809S,S:S982A,S:D1118H	6	0
hCoV-19/Mexico/GUA_INER_IMSS_00867/2021	EPI_ISL_2091318	OL461396	B.1.1.7	GRY	33	C241T,C913T,C2453T,C3037T,C3267T,C5388A,C5986T,T6954C,C9611T,C14408T,C14676T,C15279T,T16176C,C21855T,G21974C,A23063T,C23271A,A23403G,C23604A,C23709T,T24506G,G24914C,C27972T,G28048T,A28111G,G28280C,A28281T,T28282A,A28295G,G28881A,G28882A,G	23	N:D3L,N:N8D,N:R203K,N:G204R,N:S235F,ORF1a:L730F,ORF1a:T1001I,ORF1a:A1708D,ORF1a:I2230T,ORF1a:L3116F,ORF1b:P314L,ORF8:Q27*,ORF8:R52I,ORF8:Y73C,S:S98F,S:D138H,S:N501Y,S:A570D,S:D614G,S:P681H,S:T716I,S:S982	6	0

hCoV-19/Mexico/GUA-LANGEBIO_IMSS_1001/2021	EPI_ISL_2671564	OL461397	B.1.1.7	GRY	34	C241T,C913T,C2453T,C3037T,C3267T,G3692T,C5388A,C5986T,T6954C,C9611T,C14408T,C14676T,C15279T,T16176C,C21855T,G21974C,A23063T,C23271A,A23403G,C23604A,C23709T,T24506G,G24914C,C27972T,G28048T,A28111G,G28280C,A28281T,T28282A,A28295G,G28881A,G28882A,G28883C,C28977T	24	N:D3L,N:N8D,N:R203K,N:G204R,N:S235F,ORF1a:L730F,ORF1a:T1001I,ORF1a:V1143F,ORF1a:A1708D,ORF1a:I2230T,ORF1a:L3116F,ORF1b:P314L,ORF8:Q27*,ORF8:R52I,ORF8:Y73C,S:S98F,S:D138H,S:N501Y,S:A570D,S:D614G,S:P681H,S:T716I,S:S982A,S:D1118H	6	0
hCoV-19/Mexico/GUA-LANGEBIO_IMSS_1003/2021	EPI_ISL_2671565	OL461398	B.1.1.7	GRY	34	C241T,C913T,C2453T,C3037T,C3267T,C3966T,C5388A,C5986T,T6954C,C9611T,C14408T,C14676T,C15279T,T16176C,C21855T,G21974C,A23063T,C23271A,A23403G,C23604A,C23709T,T24506G,G24914C,C27972T,G28048T,A28111G,G28280C,A28281T,T28282A,A28295G,G28881A,G28882A,G28883C,C28977T	24	N:D3L,N:N8D,N:R203K,N:G204R,N:S235F,ORF1a:L730F,ORF1a:T1001I,ORF1a:A1234V,ORF1a:A1708D,ORF1a:I2230T,ORF1a:L3116F,ORF1b:P314L,ORF8:Q27*,ORF8:R52I,ORF8:Y73C,S:S98F,S:D138H,S:N501Y,S:A570D,S:D614G,S:P681H,S:T716I,S:S982A,S:D1118H	6	0
hCoV-19/Mexico/GUA-LANGEBIO_IMSS_1013/2021	EPI_ISL_2671567	OL461399	B.1.1.7	GRY	34	C241T,C913T,C3037T,C3267T,C3653T,A5311C,C5388A,C5986T,C6196T,T6954C,A8848G,C14408T,C14676T,C15279T,T16176C,A23063T,C23271A,A23403G,C23604A,C23709T,T24506G,G24914C,G27754T,C27972T,G28048T,A28111G,G28280C,A28281T,T28282A,G28881A,G28882A,G288	20	N:D3L,N:R203K,N:G204R,N:S235F,ORF1a:T1001I,ORF1a:L1130F,ORF1a:A1708D,ORF1a:I2230T,ORF1b:P314L,ORF7a:E121*,ORF8:Q27*,ORF8:R52I,ORF8:Y73C,S:N501Y,S:A570D,S:D614G,S:P681H,S:T716I,S:S982A,S:D1118H	6	2
hCoV-19/Mexico/HID-LANGEBIO_IMSS_1451/2021	EPI_ISL_2942571	OL461400	B.1.1.7	GRY	37	C241T,C913T,C2453T,C3037T,C3267T,C3784T,C5388A,C5986T,T6954C,A7479G,C9611T,C13860T,G14202A,C14408T,C14676T,C15279T,T16176C,C21855T,G21974C,A23063T,C23271A,A23403G,C23604A,C23709T,T24506G,G24914C,C27972T,G28048T,A28111G,G28280C,A28281T,T28282A,A28295G,G28881A,G28882A,G28883C,C28977T	24	N:D3L,N:N8D,N:R203K,N:G204R,N:S235F,ORF1a:L730F,ORF1a:T1001I,ORF1a:A1708D,ORF1a:I2230T,ORF1a:N2405S,ORF1a:L3116F,ORF1b:P314L,ORF8:Q27*,ORF8:R52I,ORF8:Y73C,S:S98F,S:D138H,S:N501Y,S:A570D,S:D614G,S:P681H,S:T716I,S:S982A,S:D1118H	6	0
hCoV-19/Mexico/JAL_INER_IMSS_00822/2021	EPI_ISL_2091273	OL461401	B.1.1.7	GRY	34	C241T,C913T,C3037T,C3267T,C5388A,C5986T,C6196T,T6954C,A8848G,C14408T,C14676T,C15279T,T16176C,C19524T,A23063T,C23271A,A23403G,C23604A,C23709T,T24506G,G24914C,G25217T,G26727T,C27972T,G28048T,A28111G,G28280C,A28281T,T28282A,G28881A,G28882A,G2	20	M:A69S,N:D3L,N:R203K,N:G204R,N:S235F,ORF1a:T1001I,ORF1a:A1708D,ORF1a:I2230T,ORF1b:P314L,ORF8:Q27*,ORF8:R52I,ORF8:Y73C,S:N501Y,S:A570D,S:D614G,S:P681H,S:T716I,S:S982A,S:D1118H,S:G1219C	6	0
hCoV-19/Mexico/JAL_LANGEBIO_IMSS_0755/2021	EPI_ISL_2402166	OL461402	B.1.1.7	GRY	38	C241T,C878T,C913T,C3037T,C3267T,C3924T,C5388A,C5986T,T6954C,A12162G,C12400T,C14408T,C14676T,C15279T,C15720T,T16176C,A17615G,A21137G,A23063T,C23271A,A23403G,C23604A,C23638T,C23709T,G24095T,T24506G,G24914C,G25135T,C27972T,G28048T,A28111G,G28280C,A28281T,T28282A,G28881A,G28882A,G28883C,C28977T	24	N:D3L,N:R203K,N:G204R,N:S235F,ORF1a:T1001I,ORF1a:P1220L,ORF1a:A1708D,ORF1a:I2230T,ORF1a:Q3966R,ORF1b:P314L,ORF1b:K1383R,ORF1b:K2557R,ORF8:Q27*,ORF8:R52I,ORF8:Y73C,S:N501Y,S:A570D,S:D614G,S:P681H,S:T716I,S:A845S,S:S982A,S:D1118H,S:K1191N	6	0
hCoV-19/Mexico/JAL-IBT_IMSS_1852/2021	EPI_ISL_2801725	OL461403	B.1.1.7	GRY	40	C203T,C241T,C913T,G1820A,C3037T,C3267T,C5388A,G5924A,C5986T,T6954C,G9526T,A12162G,C14408T,C14676T,C15279T,C15720T,T16176C,A17615G,T19363C,A21137G,A23063T,C23248T,C23271A,A23403G,C23604A,C23709T,T24506G,G24914C,G25135T,C27972T,G28048T,A28111G,G28280C,A28281T,T28282A,A28855T,G28881A,G28882A,G28883C,C28977T	25	N:D3L,N:R203K,N:G204R,N:S235F,ORF1a:G519S,ORF1a:T1001I,ORF1a:A1708D,ORF1a:V1887I,ORF1a:I2230T,ORF1a:M3087I,ORF1a:Q3966R,ORF1b:P314L,ORF1b:K1383R,ORF1b:K2557R,ORF8:Q27*,ORF8:R52I,ORF8:Y73C,S:N501Y,S:A570D,S:D614G,S:P681H,S:T716I,S:S982A,S:D1118H,S:K1191N	6	0
hCoV-19/Mexico/JAL-IBT_IMSS_1881/2021	EPI_ISL_2801747	OL461404	B.1.1.7	GRY	35	C241T,C913T,C3037T,C3267T,C5388A,C5986T,C6449T,T6954C,C14408T,C14676T,C15279T,T16176C,G17280T,A18992G,A23063T,C23271A,A23403G,C23604A,C23709T,T24506G,G24914C,C25553T,C27689T,C27972T,G28048T,A28111G,G28280C,A28281T,T28282A,C28310T,G28881A,G28882A,G28883C,C28977T,G29179T	23	N:D3L,N:P13S,N:R203K,N:G204R,N:S235F,ORF1a:T1001I,ORF1a:A1708D,ORF1a:L2062F,ORF1a:I2230T,ORF1b:P314L,ORF1b:K1842R,ORF3a:A54V,ORF7a:P99L,ORF8:Q27*,ORF8:R52I,ORF8:Y73C,S:N501Y,S:A570D,S:D614G,S:P681H,S:T716I,S:S982A,S:D1118H	6	482
hCoV-19/Mexico/JAL-LANGEBIO_IMSS_1009/2021	EPI_ISL_2671572	OL461405	B.1.1.7	GRY	41	C203T,C241T,C913T,G1820A,C3037T,C3267T,C5310T,C5388A,C5986T,T6954C,C7843T,G9526T,G11042T,A12162G,C14408T,C14676T,C15279T,C15720T,T16176C,A17615G,T19363C,A21137G,A23063T,C23248T,C23271A,A23403G,C23604A,C23709T,T24506G,G24914C,G25135T,C27972T,G28048T,A28111G,G28280C,A28281T,T28282A,G28881A,G28882A,G28883C,C28977T	26	N:D3L,N:R203K,N:G204R,N:S235F,ORF1a:G519S,ORF1a:T1001I,ORF1a:T1682I,ORF1a:A1708D,ORF1a:I2230T,ORF1a:M3087I,ORF1a:V3593F,ORF1a:Q3966R,ORF1b:P314L,ORF1b:K1383R,ORF1b:K2557R,ORF8:Q27*,ORF8:R52I,ORF8:Y73C,S:N501Y,S:A570D,S:D614G,S:P681H,S:T716I,S:S982A,S:D1118H,S:K1191N	6	1
hCoV-19/Mexico/JAL-LANGEBIO_IMSS_1015/2021	EPI_ISL_2671573	OL461406	B.1.1.7	GRY	40	C203T,C241T,C913T,G1820A,C3037T,C3267T,T4579A,C5388A,C5986T,T6954C,G9526T,A12162G,C14408T,C14676T,C15279T,C15720T,T16176C,A17615G,T19363C,A21137G,A23063T,C23248T,C23271A,A23403G,C23604A,C23709T,T24506G,G24914C,G25135T,T27053C,C27972T,G28048T,A28111G,G28280C,A28281T,T28282A,G28881A,G28882A,G28883C,C28977T	24	N:D3L,N:R203K,N:G204R,N:S235F,ORF1a:G519S,ORF1a:T1001I,ORF1a:A1708D,ORF1a:I2230T,ORF1a:M3087I,ORF1a:Q3966R,ORF1b:P314L,ORF1b:K1383R,ORF1b:K2557R,ORF8:Q27*,ORF8:R52I,ORF8:Y73C,S:N501Y,S:A570D,S:D614G,S:P681H,S:T716I,S:S982A,S:D1118H,S:K1191N	6	0
hCoV-19/Mexico/JAL-LANGEBIO_IMSS_1024/2021	EPI_ISL_2671577	OL461407	B.1.1.7	GRY	37	C241T,C913T,C2453T,C3037T,C3267T,G3871T,C5388A,C5986T,T6954C,C9611T,C14408T,C14676T,C15279T,T16176C,C21691T,C21855T,G21974C,A23063T,C23271A,A23403G,C23604A,C23709T,T24506G,A24874G,G24914C,C27643T,C27972T,G28048T,A28111G,G28280C,A28281T,T28282A,A28295G,G28881A,G28882A,G28883C,C28977T	25	N:D3L,N:N8D,N:R203K,N:G204R,N:S235F,ORF1a:L730F,ORF1a:T1001I,ORF1a:K1202N,ORF1a:A1708D,ORF1a:I2230T,ORF1a:L3116F,ORF1b:P314L,ORF7a:P84S,ORF8:Q27*,ORF8:R52I,ORF8:Y73C,S:S98F,S:D138H,S:N501Y,S:A570D,S:D614G,S:P681H,S:T716I,S:S982A,S:D1118H	6	5
hCoV-19/Mexico/JAL-LANGEBIO_IMSS_1032/2021	EPI_ISL_2671581	OL461408	B.1.1.7	GRY	38	C241T,C913T,C2110T,C3037T,C3267T,C5388A,C5986T,T6954C,G7042T,T9379C,A10195G,C13517T,C14120T,C14408T,C14676T,C15279T,T16176C,C21036T,G21523A,A23063T,C23271A,A23403G,C23604A,C23709T,T24506G,G24914C,C27972T,G28048T,G28075T,A28095T,A28111G,G28280C,A28281T,T28282A,G28881A,G28882A,G28883C,C28977T	24	N:D3L,N:R203K,N:G204R,N:S235F,ORF1a:T1001I,ORF1a:A1708D,ORF1a:I2230T,ORF1a:M2259I,ORF1b:T17I,ORF1b:P218L,ORF1b:P314L,ORF1b:V2686I,ORF8:Q27*,ORF8:R52I,ORF8:C61F,ORF8:K68*,ORF8:Y73C,S:N501Y,S:A570D,S:D614G,S:P681H,S:T716I,S:S982A,S:D1118H	6	6
hCoV-19/Mexico/JAL-LANGEBIO_IMSS_1247/2021	EPI_ISL_2942408	OL461409	B.1.1.7	GRY	35	C241T,C913T,C3037T,C3267T,G5150T,C5388A,C5986T,T6954C,C11750T,C14408T,C14676T,C15279T,T16176C,A23063T,C23086T,C23271A,A23403G,C23604A,C23709T,G24173T,T24506G,G24914C,C26833T,C26873T,C27972T,G28048T,A28111G,G28280C,A28281T,T28282A,G28881A,G28882A,G28883C,C28977T,C29614T	22	M:A104V,N:D3L,N:R203K,N:G204R,N:S235F,ORF1a:T1001I,ORF1a:V1629F,ORF1a:A1708D,ORF1a:I2230T,ORF1a:L3829F,ORF1b:P314L,ORF8:Q27*,ORF8:R52I,ORF8:Y73C,S:N501Y,S:A570D,S:D614G,S:P681H,S:T716I,S:A871S,S:S982A,S:D1118H	6	9
hCoV-19/Mexico/JAL-LANGEBIO_IMSS_1257/2021	EPI_ISL_2942416	OL461410	B.1.1.7	GRY	43	C913T,C2110T,C3037T,C3267T,C5388A,C5986T,T6954C,G7042T,C8389T,G10105T,C12115T,C13517T,C14120T,C14408T,C14676T,C15279T,T16176C,G16900T,C17762T,G19414T,C21077T,A23063T,C23271A,G23311T,A23403G,C23604A,C23709T,T24506G,G24914C,T25180C,C25665T,G25785T,G26416T,C27972T,G28048T,A28111G,G28280C,A28281T,T28282A,G28881A,G28882A,G28883C,C28977T	29	E:V58F,N:D3L,N:R203K,N:G204R,N:S235F,ORF1a:T1001I,ORF1a:A1708D,ORF1a:I2230T,ORF1a:M2259I,ORF1a:M3280I,ORF1b:T17I,ORF1b:P218L,ORF1b:P314L,ORF1b:G1145C,ORF1b:A1432V,ORF1b:V1983L,ORF1b:T2537I,ORF3a:W131C,ORF8:Q27*,ORF8:R52I,ORF8:Y73C,S:N501Y,S:A570D,S:E583D,S:D614G,S:P681H,S:T716I,S:S982A,S:D1118H	6	40
hCoV-19/Mexico/JAL-LANGEBIO_IMSS_1281/2021	EPI_ISL_2942436	OL461411	B.1.1.7	GRY	44	C913T,C2110T,C3037T,C3267T,C5388A,C5986T,T6954C,G7042T,C8389T,C12115T,C13517T,C14120T,C14408T,C14676T,C15279T,T16176C,G16900T,C17762T,C18788T,C21077T,A23063T,C23271A,A23403G,C23604A,C23709T,C24210T,T24506G,G24914C,T25180C,C25546T,C25665T,G25785T,G26416T,C27972T,G28048T,A28111G,G28280C,A28281T,T28282A,G28881A,G28882A,G28883C,C28977T,C29769T	29	E:V58F,N:D3L,N:R203K,N:G204R,N:S235F,ORF1a:T1001I,ORF1a:A1708D,ORF1a:I2230T,ORF1a:M2259I,ORF1b:T17I,ORF1b:P218L,ORF1b:P314L,ORF1b:G1145C,ORF1b:A1432V,ORF1b:T1774I,ORF1b:T2537I,ORF3a:L52F,ORF3a:W131C,ORF8:Q27*,ORF8:R52I,ORF8:Y73C,S:N501Y,S:A570D,S:D614G,S:P681H,S:T716I,S:T883I,S:S982A,S:D1118H	6	4
hCoV-19/Mexico/JAL-LANGEBIO_IMSS_1317/2021	EPI_ISL_2942464	OL461412	B.1.1.7	GRY	44	C203T,C241T,C913T,G1820A,C3037T,G3251T,C3267T,C5388A,C5986T,C6525T,T6954C,G9526T,A12162G,C14408T,C14676T,C15279T,C15720T,T16176C,A17615G,T19363C,C19602T,A21137G,A23063T,C23248T,C23271A,A23403G,C23604A,C23709T,T24506G,G24914C,G25135T,C27972T,G28048T,A28111G,G28280C,A28281T,T28282A,A28666G,G28881A,G28882A,G28883C,C2897	27	N:D3L,N:I131M,N:R203K,N:G204R,N:S235F,ORF1a:G519S,ORF1a:D996Y,ORF1a:T1001I,ORF1a:A1708D,ORF1a:T2087I,ORF1a:I2230T,ORF1a:M3087I,ORF1a:Q3966R,ORF1b:P314L,ORF1b:K1383R,ORF1b:K2557R,ORF8:Q27*,ORF8:R52I,ORF8:Y73C,S:N501Y,S:A570D,S:D614G,S:P681H,S:T716I,S:S982A,S:D1118H,S:	6	15
hCoV-19/Mexico/JAL-LANGEBIO_IMSS_1621/2021	EPI_ISL_2942725	OL461413	B.1.1.7	GRY	43	C241T,C913T,G1820A,T2220C,C3037T,G3251T,C3267T,C5388A,C5986T,C6525T,T6954C,G9526T,A12162G,C14408T,C14676T,C15279T,C15720T,T16176C,A17615G,T19363C,C19602T,A21137G,A23063T,C23248T,C23271A,A23403G,C23604A,C23709T,T24506G,G24914C,G25135T,G26951T,C27972T,G28048T,A28111G,G28280C,A28281T,T28282A,A28666G,G28881A,G28882A,G28883C,C28977T	28	N:D3L,N:I131M,N:R203K,N:G204R,N:S235F,ORF1a:G519S,ORF1a:I652T,ORF1a:D996Y,ORF1a:T1001I,ORF1a:A1708D,ORF1a:T2087I,ORF1a:I2230T,ORF1a:M3087I,ORF1a:Q3966R,ORF1b:P314L,ORF1b:K1383R,ORF1b:K2557R,ORF8:Q27*,ORF8:R52I,ORF8:Y73C,S:N501Y,S:A570D,S:D614G,S:P681H,S:T716I,S:S982A,S:D1118H,S:K1191N	6	485
hCoV-19/Mexico/MEX_IBT_IMSS_1135/2021	EPI_ISL_2391531	OL461414	B.1.1.7	GRY	35	C241T,C913T,C2143T,C3037T,C3267T,C5388A,C5986T,T6954C,C14408T,C14676T,T15096C,C15279T,T16176C,A23063T,C23271A,A23403G,C23604A,C23709T,G24089A,T24506G,G24914C,G25785T,C27476T,C27972T,G28048T,A28095T,A28111G,G28280C,A28281T,T28282A,G28881A,G28882A,G28883C,C28977T,C29642T	22	N:D3L,N:R203K,N:G204R,N:S235F,ORF1a:T1001I,ORF1a:A1708D,ORF1a:I2230T,ORF1b:P314L,ORF3a:W131C,ORF7a:T28I,ORF8:Q27*,ORF8:R52I,ORF8:K68*,ORF8:Y73C,S:N501Y,S:A570D,S:D614G,S:P681H,S:T716I,S:D843N,S:S982A,S:D1118H	6	0
hCoV-19/Mexico/MEX_IBT_IMSS_1249/2021	EPI_ISL_2391647	OL461415	B.1.1.7	GRY	35	C241T,C913T,C3037T,C3267T,C5388A,C5986T,C6196T,T6954C,A8848G,C14408T,C14676T,C15279T,T16176C,T19624C,T21570G,A23063T,C23271A,A23403G,C23604A,C23709T,G24173A,T24506G,G24914C,G25217T,C27972T,G28048T,A28111G,G28280C,A28281T,T28282A,G28881A,G28882A,G28883C,C28977T,G29715T	21	N:D3L,N:R203K,N:G204R,N:S235F,ORF1a:T1001I,ORF1a:A1708D,ORF1a:I2230T,ORF1b:P314L,ORF8:Q27*,ORF8:R52I,ORF8:Y73C,S:V3G,S:N501Y,S:A570D,S:D614G,S:P681H,S:T716I,S:A871T,S:S982A,S:D1118H,S:G1219C	6	254

hCoV-19/Mexico/MEX_IBT_IMSS_1512/2021	EPI_ISL_2681069	OL461416	B.1.1.7	GRY	39	C241T,C913T,G1957A,C2189T,A2276G,G2527A,C3037T,A3094T,C3267T,G4136T,C5388A,C5986T,T6954C,C11704T,C14408T,C14676T,C15279T,T16176C,G19542T,G22841A,A23063T,C23271A,A23403G,C23604A,C23709T,T24506G,G24914C,G25954T,C27972T,G28048T,A28111G,G28280C,A28281T,T28282A,C28744T,G28881A,G28882A,G28883C,C28977T	24	N:D3L,N:R203K,N:G204R,N:S235F,ORF1a:L642F,ORF1a:I671V,ORF1a:T1001I,ORF1a:V1291F,ORF1a:A1708D,ORF1a:I2230T,ORF1b:P314L,ORF1b:M2025I,ORF3a:G188C,ORF8:Q27*,ORF8:R52I,ORF8:Y73C,S:D427N,S:N501Y,S:A570D,S:D614G,S:P681H,S:T716I,S:S982A,S:D1118H	6	1
hCoV-19/Mexico/MEX_IBT_IMSS_1513/2021	EPI_ISL_2681215	OL461417	B.1.1.7	GRY	39	C241T,C913T,G1957A,C2189T,A2276G,G2527A,C3037T,A3094T,C3267T,G4136T,C5388A,C5986T,T6954C,C11704T,C14408T,C14676T,C15279T,T16176C,G19542T,G22841A,A23063T,C23271A,A23403G,C23604A,C23709T,T24506G,G24914C,G25954T,C27972T,G28048T,A28111G,G28280C,A28281T,T28282A,C28744T,G28881A,G28882A,G28883C,C28977T	24	N:D3L,N:R203K,N:G204R,N:S235F,ORF1a:L642F,ORF1a:I671V,ORF1a:T1001I,ORF1a:V1291F,ORF1a:A1708D,ORF1a:I2230T,ORF1b:P314L,ORF1b:M2025I,ORF3a:G188C,ORF8:Q27*,ORF8:R52I,ORF8:Y73C,S:D427N,S:N501Y,S:A570D,S:D614G,S:P681H,S:T716I,S:S982A,S:D1118H	6	55
hCoV-19/Mexico/MEX_IBT_IMSS_1571/2021	EPI_ISL_2681111	OL461418	B.1.1.7	GRY	41	C241T,C913T,G1069A,C1609T,C3037T,T3049C,C3267T,C5388A,G5558A,C5986T,T6954C,C8655T,A12162G,G14294A,C14408T,C14676T,C15279T,C15720T,T16176C,A17615G,C18329T,A21137G,A23063T,C23271A,A23403G,C23604A,C23709T,T24506G,G24914C,G25135T,C27972T,G28048T,A28111G,G28280C,A28281T,T28282A,G28881A,G28882A,G28883C,G28916A,C28977T	27	N:D3L,N:R203K,N:G204R,N:G215S,N:S235F,ORF1a:T1001I,ORF1a:A1708D,ORF1a:V1765I,ORF1a:I2230T,ORF1a:S2797F,ORF1a:Q3966R,ORF1b:R276H,ORF1b:P314L,ORF1b:K1383R,ORF1b:T1621I,ORF1b:K2557R,ORF8:Q27*,ORF8:R52I,ORF8:Y73C,S:N501Y,S:A570D,S:D614G,S:P681H,S:T716I,S:S982A,S:D1118H,S:	6	0
hCoV-19/Mexico/MEX_IBT_IMSS_1667/2021	EPI_ISL_2681238	OL461419	B.1.1.7	GRY	39	C203T,C241T,C913T,G1820A,C3037T,C3267T,C5388A,G5924A,C5986T,T6954C,G9526T,A12162G,C14408T,C14676T,C15279T,C15720T,T16176C,A17615G,T19363C,A21137G,A23063T,C23248T,C23271A,A23403G,C23604A,C23709T,T24506G,G24914C,G25135T,C27972T,G28048T,A28111G,G28280C,A28281T,T28282A,G28881A,G28882A,G28883C,C28977T	25	N:D3L,N:R203K,N:G204R,N:S235F,ORF1a:G519S,ORF1a:T1001I,ORF1a:A1708D,ORF1a:V1887I,ORF1a:I2230T,ORF1a:M3087I,ORF1a:Q3966R,ORF1b:P314L,ORF1b:K1383R,ORF1b:K2557R,ORF8:Q27*,ORF8:R52I,ORF8:Y73C,S:N501Y,S:A570D,S:D614G,S:P681H,S:T716I,S:S982A,S:D1118H,S:K1191N	6	128
hCoV-19/Mexico/MEX_INER_IMSS_00690/2021	EPI_ISL_2091146	OL461420	B.1.1.7	GRY	31	C241T,C913T,C3037T,C3267T,C5388A,C5986T,T6954C,C11750T,A12982G,C14408T,C14676T,C15279T,T16176C,A23063T,C23271A,A23403G,C23604A,C23709T,T24506G,G24914C,C26270T,C27972T,G28048T,A28111G,G28280C,A28281T,T28282A,G28881A,G28882A,G28883C,C28977T	20	E:T9I,N:D3L,N:R203K,N:G204R,N:S235F,ORF1a:T1001I,ORF1a:A1708D,ORF1a:I2230T,ORF1a:L3829F,ORF1b:P314L,ORF8:Q27*,ORF8:R52I,ORF8:Y73C,S:N501Y,S:A570D,S:D614G,S:P681H,S:T716I,S:S982A,S:D1118H	6	0
hCoV-19/Mexico/MEX_LANGEBIO_IMSS_20813-NC/2021	EPI_ISL_2969962	OL461421	B.1.1.7	GRY	35	C241T,C913T,C3037T,C3267T,C5388A,C5986T,T6954C,C10228T,C14408T,C14676T,T15096C,C15279T,T16176C,A23063T,C23271A,A23403G,C23604A,C23709T,T24506G,G24914C,G25785T,C27476T,C27972T,G28048T,A28095T,A28111G,G28280C,A28281T,T28282A,A28466G,G28881A,G28882A,G28883C,C28977T,C29642T	22	N:D3L,N:K65E,N:R203K,N:G204R,N:S235F,ORF1a:T1001I,ORF1a:A1708D,ORF1a:I2230T,ORF1b:P314L,ORF3a:W131C,ORF7a:T28I,ORF8:Q27*,ORF8:R52I,ORF8:K68*,ORF8:Y73C,S:N501Y,S:A570D,S:D614G,S:P681H,S:T716I,S:S982A,S:D1118H	6	996
hCoV-19/Mexico/MEX-IBT_IMSS_14917-NC/2021	EPI_ISL_2801753	OL461422	B.1.1.7	GRY	36	C241T,C913T,C3037T,C3267T,C5388A,C5986T,T6954C,G7042T,G11083T,C14120T,C14408T,C14676T,C15279T,T16176C,G21578T,A22525G,A23063T,C23271A,A23403G,C23604A,C23709T,G24415T,G24914C,G25906T,C26058T,C27972T,G28048T,A28095T,A28111G,G28280C,A28281T,T28282A,G28881A,G28882A,G28883C,C28977T	23	N:D3L,N:R203K,N:G204R,N:S235F,ORF1a:T1001I,ORF1a:A1708D,ORF1a:I2230T,ORF1a:M2259I,ORF1a:L3606F,ORF1b:P218L,ORF1b:P314L,ORF3a:G172C,ORF8:Q27*,ORF8:R52I,ORF8:K68*,ORF8:Y73C,S:V6F,S:N501Y,S:A570D,S:D614G,S:P681H,S:T716I,S:D1118H	6	879
hCoV-19/Mexico/MEX-LANGEBIO_IMSS_1085/2021	EPI_ISL_2671594	OL461423	B.1.1.7	GRY	38	G101T,C241T,C913T,C2143T,C3037T,C3267T,C5388A,C5986T,T6954C,C10228T,C14408T,C14676T,T15096C,C15279T,T16176C,A23063T,C23271A,A23403G,C23604A,C23709T,G24089A,T24506G,G24914C,G25785T,C27476T,C27972T,G28048T,A28095T,A28111G,G28280C,A28281T,T28282A,A28466G,G28881A,G28882A,G28883C,C28977T,C29642T	23	N:D3L,N:K65E,N:R203K,N:G204R,N:S235F,ORF1a:T1001I,ORF1a:A1708D,ORF1a:I2230T,ORF1b:P314L,ORF3a:W131C,ORF7a:T28I,ORF8:Q27*,ORF8:R52I,ORF8:K68*,ORF8:Y73C,S:N501Y,S:A570D,S:D614G,S:P681H,S:T716I,S:D843N,S:S982A,S:D1118H	6	0
hCoV-19/Mexico/MEX-LANGEBIO_IMSS_1114/2021	EPI_ISL_2671601	OL461424	B.1.1.7	GRY	37	C241T,C913T,C2110T,C2299T,C3037T,C3267T,C5388A,C5986T,T6954C,T9379C,A10195G,C11750T,C14120T,C14408T,C14676T,C15279T,T16176C,C21036T,A23063T,C23271A,A23403G,C23604A,C23709T,T24506G,G24914C,C27972T,G28048T,G28075T,A28095T,A28111G,G28280C,A28281T,T28282A,G28881A,G28882A,G28883C,C28977T	22	N:D3L,N:R203K,N:G204R,N:S235F,ORF1a:T1001I,ORF1a:A1708D,ORF1a:I2230T,ORF1a:L3829F,ORF1b:P218L,ORF1b:P314L,ORF8:Q27*,ORF8:R52I,ORF8:C61F,ORF8:K68*,ORF8:Y73C,S:N501Y,S:A570D,S:D614G,S:P681H,S:T716I,S:S982A,S:D1118H	6	1
hCoV-19/Mexico/MEX-LANGEBIO_IMSS_1121/2021	EPI_ISL_2671605	OL461425	B.1.1.7	GRY	32	C913T,C3037T,C3267T,A3492G,C5388A,C5986T,T6954C,C14408T,C14676T,C15279T,T16176C,C18555T,C21639A,A23063T,C23271A,A23403G,C23604A,C23709T,C24374T,T24506G,G24914C,C27972T,G28048T,A28111G,G28280C,A28281T,T28282A,G28881A,G28882A,G28883C,C28977	21	N:D3L,N:R203K,N:G204R,N:S235F,ORF1a:T1001I,ORF1a:N1076S,ORF1a:A1708D,ORF1a:I2230T,ORF1b:P314L,ORF8:Q27*,ORF8:R52I,ORF8:Y73C,S:P26H,S:N501Y,S:A570D,S:D614G,S:P681H,S:T716I,S:L938F,S:S982A,S:D1118H	6	1
hCoV-19/Mexico/MEX-LANGEBIO_IMSS_1474/2021	EPI_ISL_2942592	OL461426	B.1.1.7	GRY	41	C241T,C913T,T1177C,T1537G,C2110T,G2528A,C3037T,C3267T,C4296T,C5142T,C5388A,C5986T,T6954C,G7042T,C10789T,C14120T,C14408T,C14676T,C15279T,T16176C,C20470A,A23063T,C23271A,A23403G,C23604A,C23709T,T24506G,G24914C,C25452T,G25471T,C27972T,G28048T,A28111G,G28280C,A28281T,T28282A,G28881A,G28882A,G28883C,C28977T,C29167T	25	N:D3L,N:R203K,N:G204R,N:S235F,ORF1a:E755K,ORF1a:T1001I,ORF1a:T1344I,ORF1a:T1626I,ORF1a:A1708D,ORF1a:I2230T,ORF1a:M2259I,ORF1b:P218L,ORF1b:P314L,ORF1b:Q2335K,ORF3a:D27Y,ORF8:Q27*,ORF8:R52I,ORF8:Y73C,S:N501Y,S:A570D,S:D614G,S:P681H,S:T716I,S:S982A,S:D1118H	6	1
hCoV-19/Mexico/MEX-LANGEBIO_IMSS_1475/2021	EPI_ISL_2942593	OL461427	B.1.1.7	GRY	43	C241T,C913T,T1177C,T1537G,C2110T,G2528A,C3037T,C3267T,C4296T,C5142T,C5388A,C5986T,T6954C,G7042T,C10789T,C14120T,C14408T,C14676T,C15279T,T16176C,C20470A,A23063T,C23271A,A23403G,C23604A,C23709T,T24506G,G24914C,C25452T,G25471T,C27972T,G28048T,A28111G,A28220G,G28280C,A28281T,T28282A,C28801T,G28881A,G28882A,G28883C,C28977T	25	N:D3L,N:R203K,N:G204R,N:S235F,ORF1a:E755K,ORF1a:T1001I,ORF1a:T1344I,ORF1a:T1626I,ORF1a:A1708D,ORF1a:I2230T,ORF1a:M2259I,ORF1b:P218L,ORF1b:P314L,ORF1b:Q2335K,ORF3a:D27Y,ORF8:Q27*,ORF8:R52I,ORF8:Y73C,S:N501Y,S:A570D,S:D614G,S:P681H,S:T716I,S:S982A,S:D1118H	6	0
hCoV-19/Mexico/MEX-LANGEBIO_IMSS_1476/2021	EPI_ISL_2942594	OL461428	B.1.1.7	GRY	42	C241T,C913T,T1177C,T1537G,C2110T,G2528A,C3037T,C3267T,C4296T,C5142T,C5388A,C5986T,T6954C,G7042T,C10789T,C14120T,C14408T,C14676T,C15279T,T16176C,C20470A,A23063T,C23271A,A23403G,C23604A,C23709T,T24506G,G24914C,C25452T,G25471T,C27972T,G28048T,A28111G,G28280C,A28281T,T28282A,C28801T,G28881A,G28882A,G28883C,C28977T,C29167T	25	N:D3L,N:R203K,N:G204R,N:S235F,ORF1a:E755K,ORF1a:T1001I,ORF1a:T1344I,ORF1a:T1626I,ORF1a:A1708D,ORF1a:I2230T,ORF1a:M2259I,ORF1b:P218L,ORF1b:P314L,ORF1b:Q2335K,ORF3a:D27Y,ORF8:Q27*,ORF8:R52I,ORF8:Y73C,S:N501Y,S:A570D,S:D614G,S:P681H,S:T716I,S:S982A,S:D1118H	6	8
hCoV-19/Mexico/MEX-LANGEBIO_IMSS_1479/2021	EPI_ISL_2942596	OL461429	B.1.1.7	G	34	C913T,C2110T,G3004T,C3037T,C3267T,C5388A,C5986T,T6954C,G7042T,C9996T,C10525T,C10789T,C14120T,C14408T,C14676T,C15279T,T16176C,A23063T,C23271A,A23403G,G23593T,C23604A,C23709T,T24506G,G24914C,C25844T,C26110T,C26735T,C27389T,C27972T,G28048T,A28111G,A28804G,C28932T	22	N:A220V,ORF1a:E913D,ORF1a:T1001I,ORF1a:A1708D,ORF1a:I2230T,ORF1a:M2259I,ORF1a:S3244L,ORF1b:P218L,ORF1b:P314L,ORF3a:T151I,ORF3a:P240S,ORF8:Q27*,ORF8:R52I,ORF8:Y73C,S:N501Y,S:A570D,S:D614G,S:Q677H,S:P681H,S:T716I,S:S982A,S:D1118H	6	2
hCoV-19/Mexico/MEX-LANGEBIO_IMSS_1576/2021	EPI_ISL_2942687	OL461430	B.1.1.7	GRY	39	T154C,C241T,C913T,C3037T,C3267T,C5388A,C5944T,C5986T,T6954C,C7564T,C9967T,C13585T,C14408T,C14676T,T15096C,C15279T,T16176C,G21901A,C22987T,A23063T,C23271A,A23403G,C23604A,C23709T,T24506G,G24914C,C27972T,G28048T,A28095T,A28111G,G28280C,A28281T,T28282A,G28881A,G28882A,G28883C,G28884C,C28926T,C28977T	20	N:D3L,N:R203K,N:G204P,N:A218V,N:S235F,ORF1a:T1001I,ORF1a:A1708D,ORF1a:I2230T,ORF1b:P314L,ORF8:Q27*,ORF8:R52I,ORF8:K68*,ORF8:Y73C,S:N501Y,S:A570D,S:D614G,S:P681H,S:T716I,S:S982A,S:D1118H	6	0
hCoV-19/Mexico/MIC_INER_IMSS_1184/2021	EPI_ISL_2490332	OL461431	B.1.1.7	GRY	39	C241T,C878T,C913T,C3037T,C3267T,C5388A,C5986T,T6954C,A11991G,A12162G,C12400T,C14408T,C14676T,C15279T,C15720T,T16176C,A17615G,A21137G,T22660C,A23063T,C23271A,A23403G,C23604A,C23709T,G24095T,T24506G,G24914C,G25135T,C27972T,G28048T,A28111G,G28280C,A28281T,T28282A,G28881A,G28882A,G28883C,C28961T,C28977T	25	N:D3L,N:R203K,N:G204R,N:L230F,N:S235F,ORF1a:T1001I,ORF1a:A1708D,ORF1a:I2230T,ORF1a:E3909G,ORF1a:Q3966R,ORF1b:P314L,ORF1b:K1383R,ORF1b:K2557R,ORF8:Q27*,ORF8:R52I,ORF8:Y73C,S:N501Y,S:A570D,S:D614G,S:P681H,S:T716I,S:A845S,S:S982A,S:D1118H,S:K1191N	6	1
hCoV-19/Mexico/MOR_IBT_IMSS_1570/2021	EPI_ISL_2681243	OL461432	B.1.1.7	GRY	38	C241T,G599A,C913T,C3037T,C3267T,C5388A,C5986T,T6954C,C8956T,C14408T,C14676T,C15279T,T16176C,A17615G,A19079G,C19854T,A23063T,C23271A,T23323C,A23403G,C23604A,C23709T,C23896T,T24506G,G24914C,G25660T,G25687A,T27483C,C27972T,G28048T,A28111G,G28280C,A28281T,T28282A,G28881A,G28882A,G28883C,C28977T	23	N:D3L,N:R203K,N:G204R,N:S235F,ORF1a:G112S,ORF1a:T1001I,ORF1a:A1708D,ORF1a:I2230T,ORF1b:P314L,ORF1b:K1383R,ORF1b:E1871G,ORF3a:V90F,ORF3a:A99T,ORF8:Q27*,ORF8:R52I,ORF8:Y73C,S:N501Y,S:A570D,S:D614G,S:P681H,S:T716I,S:S982A,S:D1118H	6	253
hCoV-19/Mexico/MOR_LANGEBIO_IMSS_91231-NC/2021	EPI_ISL_2969986	OL461433	B.1.1.7	GRY	33	C913T,T1171C,C3037T,C3267T,C5388A,C5986T,T6954C,C14408T,C14676T,C15279T,T16176C,A17615G,C18032T,A21979G,A23063T,C23271A,A23403G,C23604A,C23709T,T24506G,G24914C,G26730C,C27972T,G28048T,A28111G,G28280C,A28281T,T28282A,C28603T,G28881A,G28882	21	M:V70L,N:D3L,N:R203K,N:G204R,N:S235F,ORF1a:T1001I,ORF1a:A1708D,ORF1a:I2230T,ORF1b:P314L,ORF1b:K1383R,ORF1b:T1522I,ORF8:Q27*,ORF8:R52I,ORF8:Y73C,S:N501Y,S:A570D,S:D614G,S:P681H,S:T716I,S:S982A,S:D1118H	6	979
hCoV-19/Mexico/NAY-IBT_IMSS_1850/2021	EPI_ISL_2801723	OL461434	B.1.1.7	GR	45	C241T,C622T,C913T,C3037T,C3267T,C5388A,C5986T,C6706T,T6954C,C8950T,C14408T,C14676T,C15279T,T16176C,C16323T,C16750T,G17122A,C19524T,C21234T,C21575T,A21993T,A22834G,A23063T,C23271A,A23403G,C23525T,C23604A,C23709T,T24506G,G24914T,G25526T,G25690A,C25844T,C27577T,C27972T,G28048T,A28111G,G28280C,A28281T,T28282A,G28881A,G2888	27	N:D3L,N:R203K,N:G204R,N:S235F,ORF1a:T1001I,ORF1a:A1708D,ORF1a:I2230T,ORF1b:P314L,ORF1b:P1095S,ORF1b:A1219T,ORF3a:W45L,ORF3a:G100S,ORF3a:T151I,ORF7a:Q62*,ORF8:Q27*,ORF8:R52I,ORF8:Y73C,S:L5F,S:Y144F,S:N501Y,S:A570D,S:D614G,S:H655Y,S:P681H,S:T716I,S:S982A,S:D1118Y	6	0
hCoV-19/Mexico/NAY-IBT_IMSS_1851/2021	EPI_ISL_2801724	OL461435	B.1.1.7	GRY	36	C241T,C913T,C2110T,C3037T,C3267T,C5175T,C5388A,C5986T,T6954C,T7984C,C14120T,C14408T,C14676T,C15279T,T16176C,C19390T,T19866C,G21123T,A23063T,C23271A,A23403G,C23604A,C23709T,T24506G,G24914C,C27972T,G28048T,A28095T,A28111G,G28280C,A28281T,T28282A,G28881A,G28882A,G28883C,C28977T	22	N:D3L,N:R203K,N:G204R,N:S235F,ORF1a:T1001I,ORF1a:T1637I,ORF1a:A1708D,ORF1a:I2230T,ORF1b:P218L,ORF1b:P314L,ORF1b:P1975S,ORF8:Q27*,ORF8:R52I,ORF8:K68*,ORF8:Y73C,S:N501Y,S:A570D,S:D614G,S:P681H,S:T716I,S:S982A,S:D1118H	6	0

hCoV-19/Mexico/NAY-LANGEBIO_IMSS_1041/2021	EPI_ISL_2671618	OL461436	B.1.1.7	GRY	36	C241T,C913T,C2110T,C3037T,C3267T,C5175T,C5388A,C5986T,T6954C,T7984C,C14120T,C14408T,C14676T,C15279T,T16176C,C19390T,T19866C,G21123T,A23063T,C23271A,A23403G,C23604A,C23709T,T24506G,G24914C,C27972T,G28048T,A28095T,A28111G,G28280C,A28281T,T28282A,G28881A,G28882A,G28883C,C28977T	22	N:D3L,N:R203K,N:G204R,N:S235F,ORF1a:T1001I,ORF1a:T1637I,ORF1a:A1708D,ORF1a:I2230T,ORF1b:P218L,ORF1b:P314L,ORF1b:P1975S,ORF8:Q27*,ORF8:R52I,ORF8:K68*,ORF8:Y73C,S:N501Y,S:A570D,S:D614G,S:P681H,S:T716I,S:S982A,S:D1118H	6	0
hCoV-19/Mexico/NLE_IBT_IMSS_1404/2021	EPI_ISL_2681127	OL461437	B.1.1.7	GRY	39	C186T,C241T,C913T,C2110T,C3037T,C3177T,C3267T,C5388A,C5986T,T6954C,G7042T,G9929A,A10195G,C14120T,C14408T,C14676T,C15279T,T16176C,A23063T,C23271A,A23403G,C23604A,C23709T,T24506G,G24914C,C27972T,G28048T,G28075T,A28095T,A28111G,G28280C,A28281T,T28282A,T28688C,G28881A,G28882A,G28883C,C28977T,C29095T	24	N:D3L,N:R203K,N:G204R,N:S235F,ORF1a:P971L,ORF1a:T1001I,ORF1a:A1708D,ORF1a:I2230T,ORF1a:M2259I,ORF1a:D3222N,ORF1b:P218L,ORF1b:P314L,ORF8:Q27*,ORF8:R52I,ORF8:C61F,ORF8:K68*,ORF8:Y73C,S:N501Y,S:A570D,S:D614G,S:P681H,S:T716I,S:S982A,S:D1118H	6	1
hCoV-19/Mexico/NLE_IBT_IMSS_1411/2021	EPI_ISL_2681303	OL461438	B.1.1.7	GRY	40	C241T,C913T,C3037T,C3177T,C3267T,C5388A,C5986T,T6954C,G7042T,G9929A,A10195G,G11521T,C14120T,C14408T,C14676T,C15279T,T16176C,T17088A,C21614T,G22225A,A23063T,C23271A,A23403G,C23604A,C23709T,T24506G,G24914C,C27972T,G28048T,G28075T,A28095T,A28111G,G28280C,A28281T,T28282A,T28688C,G28881A,G28882A,G28883C,C28977T	26	N:D3L,N:R203K,N:G204R,N:S235F,ORF1a:P971L,ORF1a:T1001I,ORF1a:A1708D,ORF1a:I2230T,ORF1a:M2259I,ORF1a:D3222N,ORF1a:M3752I,ORF1b:P218L,ORF1b:P314L,ORF8:Q27*,ORF8:R52I,ORF8:C61F,ORF8:K68*,ORF8:Y73C,S:L18F,S:N501Y,S:A570D,S:D614G,S:P681H,S:T716I,S:S982A,S:D1118H	6	443
hCoV-19/Mexico/NLE_IBT_IMSS_1415/2021	EPI_ISL_2681197	OL461439	B.1.1.7	GRY	42	C241T,C913T,C2110T,C3037T,C3267T,C5388A,C5986T,C6813T,T6954C,G7042T,A8658G,A10195G,C10834T,G10870T,C14120T,C14408T,C14676T,C15279T,C15933T,T16176C,C16869T,A23063T,C23271A,A23403G,C23604A,C23709T,T24506G,G24914C,A27745G,C27972T,G28048T,G28075T,A28095T,A28111G,G28280C,A28281T,T28282A,G28881A,G28882A,G28883C,C28977T,G294	25	N:D3L,N:R203K,N:G204R,N:S235F,ORF1a:T1001I,ORF1a:A1708D,ORF1a:T2183I,ORF1a:I2230T,ORF1a:M2259I,ORF1a:K2798R,ORF1b:P218L,ORF1b:P314L,ORF7a:R118G,ORF8:Q27*,ORF8:R52I,ORF8:C61F,ORF8:K68*,ORF8:Y73C,S:N501Y,S:A570D,S:D614G,S:P681H,S:T716I,S:S982A,S:D1118H	7	127
hCoV-19/Mexico/NLE_IBT_IMSS_1418/2021	EPI_ISL_2681096	OL461440	B.1.1.7	GRY	38	C241T,C913T,C2110T,C3037T,C3267T,C5388A,C5986T,T6954C,G7042T,G9929A,A10195G,C14120T,C14408T,C14676T,C15279T,T16176C,A23063T,C23271A,A23403G,C23604A,C23709T,T24506G,G24914C,C26060T,C27972T,G27987T,G28048T,G28075T,A28095T,A28111G,G28280C,A28281T,T28282A,T28688C,G28881A,G28882A,G28883C,C28977T	25	N:D3L,N:R203K,N:G204R,N:S235F,ORF1a:T1001I,ORF1a:A1708D,ORF1a:I2230T,ORF1a:M2259I,ORF1a:D3222N,ORF1b:P218L,ORF1b:P314L,ORF3a:T223I,ORF8:Q27*,ORF8:V32L,ORF8:R52I,ORF8:C61F,ORF8:K68*,ORF8:Y73C,S:N501Y,S:A570D,S:D614G,S:P681H,S:T716I,S:S982A,S:D1118H	6	0
hCoV-19/Mexico/NLE_IBT_IMSS_1426/2021	EPI_ISL_2681102	OL461441	B.1.1.7	GRY	38	C241T,C913T,C2110T,C3037T,C3267T,G4904T,C5388A,C5986T,T6954C,G7042T,A10195G,C10977T,C14120T,C14408T,C14676T,C15279T,C15933T,T16176C,A23063T,C23271A,A23403G,C23604A,C23709T,T24506G,G24914C,A27745G,C27972T,G28048T,G28075T,A28095T,A28111G,G28280C,A28281T,T28282A,G28881A,G28882A,G28883C,C28977T	25	N:D3L,N:R203K,N:G204R,N:S235F,ORF1a:T1001I,ORF1a:D1547Y,ORF1a:A1708D,ORF1a:I2230T,ORF1a:M2259I,ORF1a:A3571V,ORF1b:P218L,ORF1b:P314L,ORF7a:R118G,ORF8:Q27*,ORF8:R52I,ORF8:C61F,ORF8:K68*,ORF8:Y73C,S:N501Y,S:A570D,S:D614G,S:P681H,S:T716I,S:S982A,S:D1118H	6	0
hCoV-19/Mexico/NLE_IBT_IMSS_1427/2021	EPI_ISL_2681308	OL461442	B.1.1.7	GRY	39	C241T,C913T,C1288T,C1869T,C3037T,C3267T,C5388A,C5784T,C5986T,T6954C,A7196G,A10195G,G10379A,C14120T,C14408T,C14676T,C15279T,T16176C,A23063T,C23271A,A23403G,C23604A,C23709T,T24506G,G24914C,C25672T,C27972T,G28048T,G28075T,A28095T,A28111G,G28280C,A28281T,T28282A,T28582C,G28881A,G28882A,G28883C,C28977T	26	N:D3L,N:R203K,N:G204R,N:S235F,ORF1a:A535V,ORF1a:T1001I,ORF1a:A1708D,ORF1a:T1840I,ORF1a:I2230T,ORF1a:I2311V,ORF1a:G3372R,ORF1b:P218L,ORF1b:P314L,ORF3a:L94F,ORF8:Q27*,ORF8:R52I,ORF8:C61F,ORF8:K68*,ORF8:Y73C,S:N501Y,S:A570D,S:D614G,S:P681H,S:T716I,S:S982A,S:D1118H	6	378
hCoV-19/Mexico/NLE_IBT_IMSS_1749/2021	EPI_ISL_2681358	OL461443	B.1.1.7	GR	38	C241T,C913T,C3037T,C3267T,C5388A,C5986T,T6954C,T7984C,T9508C,C10747T,C12462T,C14120T,C14408T,C14676T,C15279T,T16176C,C16323T,C17004T,C17518T,C19390T,A23063T,C23271A,A23403G,C23604A,C23709T,T24506G,G24914C,C27972T,G28048T,A28111G,C28146T,G28280C,A28281T,T28282A,G28881A,G28882A,G28883C,C28977T	23	N:D3L,N:R203K,N:G204R,N:S235F,ORF1a:T1001I,ORF1a:A1708D,ORF1a:I2230T,ORF1a:T4066I,ORF1b:P218L,ORF1b:P314L,ORF1b:L1351F,ORF1b:P1975S,ORF8:Q27*,ORF8:R52I,ORF8:Y73C,ORF8:P85S,S:N501Y,S:A570D,S:D614G,S:P681H,S:T716I,S:S982A,S:D1118H	4	763
hCoV-19/Mexico/NLE_INER_IMSS_00735/2021	EPI_ISL_2091191	OL461444	B.1.1.7	GRY	34	C241T,C913T,C2453T,C3037T,C3267T,C5388A,C5986T,T6954C,T9445C,C9611T,C14408T,C14676T,C15279T,T16176C,C21855T,G21974C,A23063T,C23271A,A23403G,C23604A,C23709T,T24506G,G24914C,C27972T,G28048T,A28111G,G28280C,A28281T,T28282A,A28295G,G28881A,G28	23	N:D3L,N:N8D,N:R203K,N:G204R,N:S235F,ORF1a:L730F,ORF1a:T1001I,ORF1a:A1708D,ORF1a:I2230T,ORF1a:L3116F,ORF1b:P314L,ORF8:Q27*,ORF8:R52I,ORF8:Y73C,S:S98F,S:D138H,S:N501Y,S:A570D,S:D614G,S:P681H,S:T716I,S:S982	6	0
hCoV-19/Mexico/NLE_INER_IMSS_00746/2021	EPI_ISL_2091201	OL461445	B.1.1.7	GRY	38	C241T,C913T,C2110T,G2659A,C3037T,C3267T,C5388A,C5986T,T6954C,G7042T,A10195G,C14120T,C14408T,C14676T,C15279T,T16176C,A17460G,C18261T,G19872T,A23063T,C23271A,A23403G,C23604A,C23709T,T24506G,G24914C,C27972T,G28048T,G28075T,A28095T,A28111G,G28280C,A28281T,T28282A,G28881A,G28882A,G28883C,C28977T	22	N:D3L,N:R203K,N:G204R,N:S235F,ORF1a:T1001I,ORF1a:A1708D,ORF1a:I2230T,ORF1a:M2259I,ORF1b:P218L,ORF1b:P314L,ORF8:Q27*,ORF8:R52I,ORF8:C61F,ORF8:K68*,ORF8:Y73C,S:N501Y,S:A570D,S:D614G,S:P681H,S:T716I,S:S982A,S:D1118H	6	0
hCoV-19/Mexico/NLE_INER_IMSS_00753/2021	EPI_ISL_2091207	OL461446	B.1.1.7	GRY	37	C241T,G520T,C913T,C3037T,C3267T,C3486T,T4057C,C5388A,C5986T,T6954C,C11750T,G12179T,C14408T,C14676T,C15279T,T16176C,C20325T,A23063T,C23271A,A23403G,C23604A,C23709T,T24506G,G24914C,C26270T,C26892T,C27972T,G28048T,G28089T,A28111G,G28280C,A28281T,T28282A,G28881A,G28882A,G28883C,C28977T	25	E:T9I,M:L124F,N:D3L,N:R203K,N:G204R,N:S235F,ORF1a:M85I,ORF1a:T1001I,ORF1a:A1074V,ORF1a:A1708D,ORF1a:I2230T,ORF1a:L3829F,ORF1a:D3972Y,ORF1b:P314L,ORF8:Q27*,ORF8:R52I,ORF8:G66C,ORF8:Y73C,S:N501Y,S:A570D,S:D614G,S:P681H,S:T716I,S:S982A,S:D1118H	6	0
hCoV-19/Mexico/NLE_INER_IMSS_00765/2021	EPI_ISL_2091219	OL461447	B.1.1.7	GRY	41	T201A,C241T,C913T,C1076T,C2110T,C3037T,C3267T,C5388A,C5986T,C6388T,T6954C,G7042T,A10195G,C11224T,G11335A,C14120T,C14408T,C14676T,A14856G,C15279T,T16176C,A23063T,C23271A,A23403G,C23604A,C23709T,T24506G,G24914C,C27972T,G28048T,G28075T,A28095T,A28111G,G28280C,A28281T,T28282A,G28881A,G28882A,G28883C,C28977T,G29751T	23	N:D3L,N:R203K,N:G204R,N:S235F,ORF1a:P271S,ORF1a:T1001I,ORF1a:A1708D,ORF1a:I2230T,ORF1a:M2259I,ORF1b:P218L,ORF1b:P314L,ORF8:Q27*,ORF8:R52I,ORF8:C61F,ORF8:K68*,ORF8:Y73C,S:N501Y,S:A570D,S:D614G,S:P681H,S:T716I,S:S982A,S:D1118H	6	0
hCoV-19/Mexico/NLE_INER_IMSS_00768/2021	EPI_ISL_2091222	OL461448	B.1.1.7	GRY	35	C241T,C913T,C2453T,C3037T,C3267T,C5388A,C5986T,T6954C,T9445C,C9611T,C14408T,C14676T,C15279T,T16176C,G21578A,C21855T,G21974C,A23063T,C23271A,A23403G,C23604A,C23709T,T24506G,G24914C,C27972T,G28048T,A28111G,G28280C,A28281T,T28282A,A28295G,G28881A,G28882A,G28883C,C28977T	24	N:D3L,N:N8D,N:R203K,N:G204R,N:S235F,ORF1a:L730F,ORF1a:T1001I,ORF1a:A1708D,ORF1a:I2230T,ORF1a:L3116F,ORF1b:P314L,ORF8:Q27*,ORF8:R52I,ORF8:Y73C,S:V6I,S:S98F,S:D138H,S:N501Y,S:A570D,S:D614G,S:P681H,S:T716I,S:S982A,S:D1118H	6	0
hCoV-19/Mexico/NLE_INER_IMSS_1018/2021	EPI_ISL_2490337	OL461449	B.1.1.7	GRY	38	C241T,C913T,C2110T,C3037T,C3177T,C3267T,C5388A,C5986T,T6954C,G7042T,G9929A,A10195G,C13378T,C14120T,C14408T,C14676T,C15279T,T16176C,A23063T,C23271A,A23403G,C23604A,C23709T,T24506G,G24914C,C27972T,G28048T,G28075T,A28095T,A28111G,G28280C,A28281T,T28282A,T28688C,G28881A,G28882A,G28883C,C28977T	24	N:D3L,N:R203K,N:G204R,N:S235F,ORF1a:P971L,ORF1a:T1001I,ORF1a:A1708D,ORF1a:I2230T,ORF1a:M2259I,ORF1a:D3222N,ORF1b:P218L,ORF1b:P314L,ORF8:Q27*,ORF8:R52I,ORF8:C61F,ORF8:K68*,ORF8:Y73C,S:N501Y,S:A570D,S:D614G,S:P681H,S:T716I,S:S982A,S:D1118H	6	0
hCoV-19/Mexico/NLE_INER_IMSS_1027/2021	EPI_ISL_2835916	OL461450	B.1.1.7	GRY	38	C241T,C913T,C2110T,C2536T,G2776A,C3037T,C3267T,C5388A,C5986T,T6954C,C11565T,C14120T,C14408T,C14676T,C15279T,C15933T,T16176C,C18555T,G22014T,A23063T,C23271A,A23403G,C23604A,C23709T,T24506G,G24914C,C27972T,G28048T,A28095T,A28111G,G28280C,A28281T,T28282A,C28313T,G28881A,G28882A,G28883C,C28977T	23	N:D3L,N:R14C,N:R203K,N:G204R,N:S235F,ORF1a:T1001I,ORF1a:A1708D,ORF1a:I2230T,ORF1a:P3767L,ORF1b:P218L,ORF1b:P314L,ORF8:Q27*,ORF8:R52I,ORF8:K68*,ORF8:Y73C,S:S151I,S:N501Y,S:A570D,S:D614G,S:P681H,S:T716I,S:S982A,S:D1118H	7	0
hCoV-19/Mexico/NLE_INER_IMSS_1049/2021	EPI_ISL_2490313	OL461451	B.1.1.7	GRY	35	C241T,C913T,C1912T,C3037T,C3096T,C3177T,C3267T,C5388A,C5986T,T6954C,C8266T,C10252T,C14408T,C14676T,C15279T,T16176C,A23063T,C23271A,A23403G,C23604A,C23709T,T24506G,G24914C,G24933T,C27879T,C27972T,G28048T,A28111G,G28280C,A28281T,T28282A,G28881A,G28882A,G28883C,C28977T	22	N:D3L,N:R203K,N:G204R,N:S235F,ORF1a:S944L,ORF1a:P971L,ORF1a:T1001I,ORF1a:A1708D,ORF1a:I2230T,ORF1b:P314L,ORF7b:H42Y,ORF8:Q27*,ORF8:R52I,ORF8:Y73C,S:N501Y,S:A570D,S:D614G,S:P681H,S:T716I,S:S982A,S:D1118H,S:G1124V	6	0
hCoV-19/Mexico/NLE_INER_IMSS_1056/2021	EPI_ISL_2490315	OL461452	B.1.1.7	GRY	34	C241T,C913T,C2453T,C3037T,C3267T,C5388A,C5986T,T6954C,T9445C,C9611T,C14408T,C14676T,C15279T,T16176C,C21855T,G21974C,A23063T,C23271A,A23403G,C23604A,C23709T,T24506G,G24914C,C27972T,G28048T,A28111G,G28280C,A28281T,T28282A,A28295G,G28881A,G28	23	N:D3L,N:N8D,N:R203K,N:G204R,N:S235F,ORF1a:L730F,ORF1a:T1001I,ORF1a:A1708D,ORF1a:I2230T,ORF1a:L3116F,ORF1b:P314L,ORF8:Q27*,ORF8:R52I,ORF8:Y73C,S:S98F,S:D138H,S:N501Y,S:A570D,S:D614G,S:P681H,S:T716I,S:S982	6	0
hCoV-19/Mexico/NLE_INER_IMSS_1058/2021	EPI_ISL_2490316	OL461453	B.1.1.7	GRY	34	C241T,C913T,C2453T,C3037T,C3267T,C5388A,C5986T,T6954C,C9611T,C14408T,C14676T,C15279T,T16176C,C21855T,G21974C,A23063T,C23271A,A23403G,C23604A,C23709T,T24506G,G24914C,T25959C,C27972T,G28048T,A28111G,G28280C,A28281T,T28282A,A28295G,G28881A,G2	23	N:D3L,N:N8D,N:R203K,N:G204R,N:S235F,ORF1a:L730F,ORF1a:T1001I,ORF1a:A1708D,ORF1a:I2230T,ORF1a:L3116F,ORF1b:P314L,ORF8:Q27*,ORF8:R52I,ORF8:Y73C,S:S98F,S:D138H,S:N501Y,S:A570D,S:D614G,S:P681H,S:T716I,S:S982	6	0
hCoV-19/Mexico/NLE_INER_IMSS_1073/2021	EPI_ISL_2490320	OL461454	B.1.1.7	GRY	40	C241T,C913T,G2281A,C3037T,C3267T,C5388A,C5986T,T6954C,A12162G,C13665T,C14408T,C14676T,C15279T,C15720T,T16176C,A17615G,C18747T,C20844T,A21137G,A23063T,C23271A,A23403G,C23604A,C23709T,T24506G,G24914C,G25135T,C26110T,G26828T,C27972T,G28048T,A28111G,G28280C,A28281T,T28282A,G28881A,G28882A,G28883C,C28977T,C29095T	23	N:D3L,N:R203K,N:G204R,N:S235F,ORF1a:T1001I,ORF1a:A1708D,ORF1a:I2230T,ORF1a:Q3966R,ORF1b:P314L,ORF1b:K1383R,ORF1b:K2557R,ORF3a:P240S,ORF8:Q27*,ORF8:R52I,ORF8:Y73C,S:N501Y,S:A570D,S:D614G,S:P681H,S:T716I,S:S982A,S:D1118H,S:K1191N	6	1
hCoV-19/Mexico/NLE_INER_IMSS_1091/2021	EPI_ISL_2490341	OL461455	B.1.1.7	GRY	40	C241T,C913T,C2110T,C3037T,C3177T,C3267T,C5388A,C5986T,T6954C,G7042T,G9929A,A10195G,G11851T,C12651T,C14120T,C14408T,C14676T,C15279T,T16176C,C20628G,A23063T,C23271A,A23403G,C23604A,C23709T,T24506G,G24914C,C27972T,G28048T,G28075T,A28095T,A28111G,G28280C,A28281T,T28282A,T28688C,G28881A,G28882A,G28883C,C28977T	26	N:D3L,N:R203K,N:G204R,N:S235F,ORF1a:P971L,ORF1a:T1001I,ORF1a:A1708D,ORF1a:I2230T,ORF1a:M2259I,ORF1a:D3222N,ORF1a:M3862I,ORF1a:T4129I,ORF1b:P218L,ORF1b:P314L,ORF8:Q27*,ORF8:R52I,ORF8:C61F,ORF8:K68*,ORF8:Y73C,S:N501Y,S:A570D,S:D614G,S:P681H,S:T716I,S:S982A,S:D1118H	6	0

hCoV-19/Mexico/NLE_INER_IMSS_1102/2021	EPI_ISL_2490342	OL461456	B.1.1.7	GRY	42	C241T,T907C,C913T,C1473T,C2110T,C3037T,C3267T,C5388A,C5986T,T6954C,G7042T,A10195G,C14120T,C14408T,C14676T,C15279T,C15933T,T16176C,C21597T,A23063T,C23271A,A23403G,C23604A,C23709T,T24506G,G24914C,G25471T,G25855T,G26774T,A27745G,C27972T,G28048T,G28075T,A28095T,A28111G,G28280C,A28281T,T28282A,G28881A,G28882A,G28883C,C2897	28	M:M84I,N:D3L,N:R203K,N:G204R,N:S235F,ORF1a:T403I,ORF1a:T1001I,ORF1a:A1708D,ORF1a:I2230T,ORF1a:M2259I,ORF1b:P218L,ORF1b:P314L,ORF3a:D27Y,ORF3a:D155Y,ORF7a:R118G,ORF8:Q27*,ORF8:R52I,ORF8:C61F,ORF8:K68*,ORF8:Y73C,S:S12F,S:N501Y,S:A570D,S:D614G,S:P681H,S:T716I,S:S982A,S:D11	6	0
hCoV-19/Mexico/NLE_LANGEBIO_IMSS_0504/2021	EPI_ISL_2401933	OL461457	B.1.1.7	GRY	38	C241T,C913T,C2110T,C3037T,C3267T,C5388A,C5986T,T6954C,G7042T,C7834T,A10195G,A10323G,C14120T,C14408T,C14676T,C15279T,T16176C,C16833T,C22347T,A23063T,C23271A,A23403G,C23604A,C23709T,T24506G,G24914C,C27972T,G28048T,G28075T,A28095T,A28111G,G28280C,A28281T,T28282A,G28881A,G28882A,G28883C,C28977T	24	N:D3L,N:R203K,N:G204R,N:S235F,ORF1a:T1001I,ORF1a:A1708D,ORF1a:I2230T,ORF1a:M2259I,ORF1a:K3353R,ORF1b:P218L,ORF1b:P314L,ORF8:Q27*,ORF8:R52I,ORF8:C61F,ORF8:K68*,ORF8:Y73C,S:A262V,S:N501Y,S:A570D,S:D614G,S:P681H,S:T716I,S:S982A,S:D1118H	6	292
hCoV-19/Mexico/NLE_LANGEBIO_IMSS_0508/2021	EPI_ISL_2401937	OL461458	B.1.1.7	GRY	38	G204T,C241T,C913T,C3037T,C3267T,C5388A,C5986T,T6954C,A8547G,C9693T,C11572T,C14408T,C14676T,C15279T,T16176C,T21709C,T23042C,A23063T,C23271A,A23403G,C23604A,C23709T,T24506G,G24914C,G25218T,C25553T,C27615T,C27972T,G28048T,A28095T,A28111G,G28280C,A28281T,T28282A,G28881A,G28882A,G28883C,C28977T	24	N:D3L,N:R203K,N:G204R,N:S235F,ORF1a:T1001I,ORF1a:A1708D,ORF1a:I2230T,ORF1a:K2761R,ORF1a:A3143V,ORF1b:P314L,ORF3a:A54V,ORF8:Q27*,ORF8:R52I,ORF8:K68*,ORF8:Y73C,S:S494P,S:N501Y,S:A570D,S:D614G,S:P681H,S:T716I,S:S982A,S:D1118H,S:G1219V	6	15
hCoV-19/Mexico/NLE_LANGEBIO_IMSS_0520/2021	EPI_ISL_2401947	OL461459	B.1.1.7	GRY	38	C241T,C913T,C2110T,C2388T,C3037T,C3267T,C5388A,C5986T,T6954C,G7042T,C7834T,A10195G,C14120T,C14408T,C14676T,C15279T,T16176C,C16833T,C22347T,A23063T,C23271A,A23403G,C23604A,C23709T,T24506G,G24914C,C27972T,G28048T,G28075T,A28095T,A28111G,G28280C,A28281T,T28282A,G28881A,G28882A,G28883C,C28977T	24	N:D3L,N:R203K,N:G204R,N:S235F,ORF1a:T708I,ORF1a:T1001I,ORF1a:A1708D,ORF1a:I2230T,ORF1a:M2259I,ORF1b:P218L,ORF1b:P314L,ORF8:Q27*,ORF8:R52I,ORF8:C61F,ORF8:K68*,ORF8:Y73C,S:A262V,S:N501Y,S:A570D,S:D614G,S:P681H,S:T716I,S:S982A,S:D1118H	6	19
hCoV-19/Mexico/NLE_LANGEBIO_IMSS_0538/2021	EPI_ISL_2401965	OL461460	B.1.1.7	GRY	42	C241T,C913T,G1727A,C2110T,C3037T,C3267T,C4832T,C5388A,C5986T,A6378G,T6954C,G7042T,A10195G,C14120T,C14408T,C14676T,A14791G,C15279T,T16176C,C21762T,A23063T,C23271A,A23403G,C23604A,C23709T,C24237T,T24506G,G24816C,G24914C,C27972T,G28048T,G28075T,A28095T,A28111G,G28280C,A28281T,T28282A,G28881A,G28882A,G28883C,C28977T,C2951	28	N:D3L,N:R203K,N:G204R,N:S235F,ORF1a:V488M,ORF1a:T1001I,ORF1a:A1708D,ORF1a:N2038S,ORF1a:I2230T,ORF1a:M2259I,ORF1b:P218L,ORF1b:P314L,ORF1b:S442G,ORF8:Q27*,ORF8:R52I,ORF8:C61F,ORF8:K68*,ORF8:Y73C,S:A67V,S:N501Y,S:A570D,S:D614G,S:P681H,S:T716I,S:A892V,S:S982A,S:G1085A,S:D1	6	0
hCoV-19/Mexico/NLE_LANGEBIO_IMSS_0554/2021	EPI_ISL_2401977	OL461461	B.1.1.7	GRY	37	C241T,C292T,C913T,C1060T,C3037T,C3267T,C4543T,C5388A,C5986T,T6954C,G12769A,C14408T,C14676T,C15279T,T16176C,T16563A,A23063T,C23170T,C23271A,A23403G,C23604A,C23709T,T24506G,G24914C,C25626T,C27972T,G28048T,A28111G,G28280C,A28281T,T28282A,A28295C,C28728T,G28881A,G28882A,G28883C,C28977T	21	N:D3L,N:N8H,N:A152V,N:R203K,N:G204R,N:S235F,ORF1a:T1001I,ORF1a:A1708D,ORF1a:I2230T,ORF1b:P314L,ORF8:Q27*,ORF8:R52I,ORF8:Y73C,ORF9b:K4N,S:N501Y,S:A570D,S:D614G,S:P681H,S:T716I,S:S982A,S:D1118H	6	3
hCoV-19/Mexico/NLE_LANGEBIO_IMSS_0555/2021	EPI_ISL_2401978	OL461462	B.1.1.7	GRY	35	C241T,C913T,C2453T,C3037T,C3267T,C5388A,C5986T,T6954C,T9445C,C9611T,C14408T,C14676T,C15279T,T16176C,C21855T,A23063T,C23271A,A23403G,C23604A,C23709T,T24506G,G24914C,C27649A,C27972T,G28048T,A28111G,G28280C,A28281T,T28282A,A28295G,G28881A,G28882A,G28883C,G28922T,C28977T	24	N:D3L,N:N8D,N:R203K,N:G204R,N:A217S,N:S235F,ORF1a:L730F,ORF1a:T1001I,ORF1a:A1708D,ORF1a:I2230T,ORF1a:L3116F,ORF1b:P314L,ORF7a:L86M,ORF8:Q27*,ORF8:R52I,ORF8:Y73C,S:S98F,S:N501Y,S:A570D,S:D614G,S:P681H,S:T716I,S:S982A,S:D1118H	6	0
hCoV-19/Mexico/NLE_LANGEBIO_IMSS_0565/2021	EPI_ISL_2401987	OL461463	B.1.1.7	GRY	31	C241T,C913T,C3037T,C3267T,C5388A,C5986T,T6954C,C9298T,C13584T,C14408T,C14676T,C15279T,T16176C,A23063T,C23271A,A23403G,C23604A,C23709T,C24374T,T24506G,G24914C,C27972T,G28048T,A28111G,G28280C,A28281T,T28282A,G28881A,G28882A,G28883C,C28977T	19	N:D3L,N:R203K,N:G204R,N:S235F,ORF1a:T1001I,ORF1a:A1708D,ORF1a:I2230T,ORF1b:P314L,ORF8:Q27*,ORF8:R52I,ORF8:Y73C,S:N501Y,S:A570D,S:D614G,S:P681H,S:T716I,S:L938F,S:S982A,S:D1118H	6	51
hCoV-19/Mexico/NLE_LANGEBIO_IMSS_0568/2021	EPI_ISL_2401990	OL461464	B.1.1.7	GRY	37	C241T,C913T,G1820A,C2110T,C3037T,C3267T,C5388A,C5986T,T6954C,G7042T,G9929A,A10195G,C14120T,C14408T,C14676T,C15279T,T16176C,A23063T,C23271A,A23403G,C23604A,C23709T,T24506G,G24914C,C27972T,G28048T,G28075T,A28095T,A28111G,G28280C,A28281T,T28282A,T28688C,G28881A,G28882A,G28883C,C28977T	24	N:D3L,N:R203K,N:G204R,N:S235F,ORF1a:G519S,ORF1a:T1001I,ORF1a:A1708D,ORF1a:I2230T,ORF1a:M2259I,ORF1a:D3222N,ORF1b:P218L,ORF1b:P314L,ORF8:Q27*,ORF8:R52I,ORF8:C61F,ORF8:K68*,ORF8:Y73C,S:N501Y,S:A570D,S:D614G,S:P681H,S:T716I,S:S982A,S:D1118H	6	6
hCoV-19/Mexico/NLE_LANGEBIO_IMSS_0586/2021	EPI_ISL_2402006	OL461465	B.1.1.7	GRY	31	C241T,C913T,C3037T,C3267T,C5388A,C5986T,T6954C,C9693T,C14408T,C14676T,C15279T,T16176C,C18348T,A20860G,A23063T,C23271A,A23403G,C23604A,C23709T,T24506G,G24914C,C27972T,G28048T,A28111G,G28280C,A28281T,T28282A,G28881A,G28882A,G28883C,C28977T	20	N:D3L,N:R203K,N:G204R,N:S235F,ORF1a:T1001I,ORF1a:A1708D,ORF1a:I2230T,ORF1a:A3143V,ORF1b:P314L,ORF1b:I2465V,ORF8:Q27*,ORF8:R52I,ORF8:Y73C,S:N501Y,S:A570D,S:D614G,S:P681H,S:T716I,S:S982A,S:D1118H	6	0
hCoV-19/Mexico/NLE_LANGEBIO_IMSS_0587/2021	EPI_ISL_2402007	OL461466	B.1.1.7	GRY	30	C241T,C913T,C3037T,C3267T,C5388A,C5986T,T6954C,C14408T,C14649T,C14676T,C15279T,T16176C,C18348T,A23063T,C23271A,A23403G,C23604A,C23709T,T24506G,G24914C,C27972T,G28048T,A28111G,G28280C,A28281T,T28282A,G28881A,G28882A,G28883C,C28977T	18	N:D3L,N:R203K,N:G204R,N:S235F,ORF1a:T1001I,ORF1a:A1708D,ORF1a:I2230T,ORF1b:P314L,ORF8:Q27*,ORF8:R52I,ORF8:Y73C,S:N501Y,S:A570D,S:D614G,S:P681H,S:T716I,S:S982A,S:D1118H	6	505
hCoV-19/Mexico/NLE_LANGEBIO_IMSS_61186-NC/2021	EPI_ISL_2970006	OL461467	B.1.1.7	GRY	39	C913T,C2388T,C3037T,C3267T,C5388A,C5986T,T6954C,C7834T,A10195G,C14120T,C14408T,C14676T,C15279T,T16176C,C16833T,A20540C,C21727T,C22347T,A23063T,C23271A,A23403G,C23604A,C23709T,T24506G,G24914C,C27972T,G28048T,G28075T,A28095T,A28111G,G28280C,A28281T,T28282A,G28851T,G28881A,G28882A,G28883C,C28977T,G28980T	26	N:D3L,N:S193I,N:R203K,N:G204R,N:S235F,N:G236V,ORF1a:T708I,ORF1a:T1001I,ORF1a:A1708D,ORF1a:I2230T,ORF1b:P218L,ORF1b:P314L,ORF1b:K2358T,ORF8:Q27*,ORF8:R52I,ORF8:C61F,ORF8:K68*,ORF8:Y73C,S:A262V,S:N501Y,S:A570D,S:D614G,S:P681H,S:T716I,S:S982A,S:D1118H	6	1157
hCoV-19/Mexico/NLE-IBT_IMSS_1764/2021	EPI_ISL_2801661	OL461468	B.1.1.7	GRY	39	C241T,C913T,C3037T,C3267T,C4276T,C5388A,C5986T,T6954C,C7945T,A10195G,C10834T,A13187G,C14120T,C14408T,A14613G,C14676T,C15279T,T16176C,A23063T,C23271A,A23403G,C23604A,C23709T,T24506G,G24914C,C27972T,G28048T,G28075T,A28095T,A28111G,C28236T,G28280C,A28281T,T28282A,G28881A,G28882A,G28883C,C28977T,C29642T	23	N:D3L,N:R203K,N:G204R,N:S235F,ORF1a:T1001I,ORF1a:A1708D,ORF1a:I2230T,ORF1a:I4308V,ORF1b:P218L,ORF1b:P314L,ORF8:Q27*,ORF8:R52I,ORF8:C61F,ORF8:K68*,ORF8:Y73C,ORF8:R115C,S:N501Y,S:A570D,S:D614G,S:P681H,S:T716I,S:S982A,S:D1118H	6	824
hCoV-19/Mexico/NLE-IBT_IMSS_1768/2021	EPI_ISL_2801663	OL461469	B.1.1.7	G	35	C241T,C913T,C3037T,C3177T,C3267T,C4890T,C5388A,C5986T,T6954C,G9929A,A10195G,C12741T,C14120T,C14408T,C14676T,C15279T,T16176C,C20628G,A23063T,C23271A,A23403G,C23604A,C23709T,T24506G,G24914C,C27972T,C28000A,G28048T,G28075T,A28095T,A28111G,G28280C,T28282A,T28688C,C28977T	24	N:D3Q,N:S235F,ORF1a:P971L,ORF1a:T1001I,ORF1a:T1542I,ORF1a:A1708D,ORF1a:I2230T,ORF1a:D3222N,ORF1a:T4159I,ORF1b:P218L,ORF1b:P314L,ORF8:Q27*,ORF8:P36Q,ORF8:R52I,ORF8:C61F,ORF8:K68*,ORF8:Y73C,S:N501Y,S:A570D,S:D614G,S:P681H,S:T716I,S:S982A,S:D1118H	4	672
hCoV-19/Mexico/NLE-IBT_IMSS_1772/2021	EPI_ISL_2801666	OL461470	B.1.1.7	GRY	32	C241T,C913T,C1912T,C3037T,C3177T,C3267T,C5388A,C5986T,T6954C,C10252T,C14408T,C14676T,C15279T,T16176C,A23063T,C23271A,A23403G,C23604A,C23709T,T24506G,G24914C,C27879T,C27972T,G28048T,A28111G,G28280C,A28281T,T28282A,G28881A,G28882A,G28883C,C2	20	N:D3L,N:R203K,N:G204R,N:S235F,ORF1a:P971L,ORF1a:T1001I,ORF1a:A1708D,ORF1a:I2230T,ORF1b:P314L,ORF7b:H42Y,ORF8:Q27*,ORF8:R52I,ORF8:Y73C,S:N501Y,S:A570D,S:D614G,S:P681H,S:T716I,S:S982A,S:D1118H	6	705
hCoV-19/Mexico/NLE-IBT_IMSS_1774/2021	EPI_ISL_2801667	OL461471	B.1.1.7	GRY	39	C241T,C913T,C2110T,C3037T,C3267T,G4904T,C5388A,C5986T,T6954C,G7042T,A10195G,C14120T,C14408T,C14676T,C15279T,C15933T,T16176C,A23063T,C23271A,A23403G,C23604A,C23709T,T24506G,G24914C,G27364C,G27561T,A27745G,C27972T,G28048T,G28075T,A28095T,A28111G,G28280C,A28281T,T28282A,G28881A,G28882A,G28883C,C28977T	25	N:D3L,N:R203K,N:G204R,N:S235F,ORF1a:T1001I,ORF1a:D1547Y,ORF1a:A1708D,ORF1a:I2230T,ORF1a:M2259I,ORF1b:P218L,ORF1b:P314L,ORF6:E55Q,ORF7a:R118G,ORF8:Q27*,ORF8:R52I,ORF8:C61F,ORF8:K68*,ORF8:Y73C,S:N501Y,S:A570D,S:D614G,S:P681H,S:T716I,S:S982A,S:D1118H	6	95
hCoV-19/Mexico/NLE-IBT_IMSS_1779/2021	EPI_ISL_2801668	OL461472	B.1.1.7	GRY	42	C241T,C913T,C2110T,C2143T,C2675T,C3037T,C3267T,C5144T,C5388A,C5986T,C6539T,T6954C,G7042T,C9246T,A10195G,C10834T,C14120T,C14408T,C14676T,C15279T,T16176C,A23063T,C23271A,A23403G,C23604A,C23709T,T24506G,G24914C,G25650T,C27972T,G28048T,G28075T,A28095T,A28111G,G28280C,A28281T,T28282A,G28881A,G28882A,G28883C,C28977T,C29642T	26	N:D3L,N:R203K,N:G204R,N:S235F,ORF1a:P804S,ORF1a:T1001I,ORF1a:A1708D,ORF1a:H2092Y,ORF1a:I2230T,ORF1a:M2259I,ORF1a:A2994V,ORF1b:P218L,ORF1b:P314L,ORF3a:L86F,ORF8:Q27*,ORF8:R52I,ORF8:C61F,ORF8:K68*,ORF8:Y73C,S:N501Y,S:A570D,S:D614G,S:P681H,S:T716I,S:S982A,S:D1118H	6	0
hCoV-19/Mexico/NLE-IBT_IMSS_1786/2021	EPI_ISL_2801670	OL461473	B.1.1.7	GRY	40	C241T,C913T,C2110T,C3037T,C3267T,G4904T,C5388A,C5986T,T6954C,G7042T,C10116T,A10195G,C10977T,C14120T,C14408T,C14676T,C15279T,C15933T,T16176C,A23063T,C23271A,A23403G,C23604A,C23709T,T24506G,G24914C,G27598A,A27745G,C27972T,G28048T,G28075T,A28095T,A28111G,G28280C,A28281T,T28282A,G28881A,G28882A,G28883C,C28977T	27	N:D3L,N:R203K,N:G204R,N:S235F,ORF1a:T1001I,ORF1a:D1547Y,ORF1a:A1708D,ORF1a:I2230T,ORF1a:M2259I,ORF1a:T3284I,ORF1a:A3571V,ORF1b:P218L,ORF1b:P314L,ORF7a:D69N,ORF7a:R118G,ORF8:Q27*,ORF8:R52I,ORF8:C61F,ORF8:K68*,ORF8:Y73C,S:N501Y,S:A570D,S:D614G,S:P681H,S:T716I,S:S982A,S:	6	287
hCoV-19/Mexico/NLE-IBT_IMSS_1796/2021	EPI_ISL_2801675	OL461474	B.1.1.7	GRY	42	C241T,C913T,C2110T,C3037T,C3177T,C3267T,C4890T,C5388A,C5986T,T6954C,G7042T,G9929A,A10195G,C11050T,C12741T,C14120T,C14408T,C14676T,C15279T,T16176C,C20628G,A23063T,C23271A,A23403G,C23604A,C23709T,T24506G,G24914C,C27972T,C28000A,G28048T,G28075T,A28095T,A28111G,G28280C,A28281T,T28282A,T28688C,G28881A,G28882A,G28883C,C2897	27	N:D3L,N:R203K,N:G204R,N:S235F,ORF1a:P971L,ORF1a:T1001I,ORF1a:T1542I,ORF1a:A1708D,ORF1a:I2230T,ORF1a:M2259I,ORF1a:D3222N,ORF1a:T4159I,ORF1b:P218L,ORF1b:P314L,ORF8:Q27*,ORF8:P36Q,ORF8:R52I,ORF8:C61F,ORF8:K68*,ORF8:Y73C,S:N501Y,S:A570D,S:D614G,S:P681H,S:T716I,S:S982A,S:D1	6	1
hCoV-19/Mexico/NLE-IBT_IMSS_1797/2021	EPI_ISL_2801676	OL461475	B.1.1.7	GRY	41	C241T,C913T,C3037T,C3177T,C3267T,C4890T,C5388A,C5986T,T6954C,G7042T,G9929A,A10195G,C12741T,C14120T,C14408T,C14676T,C15279T,T16176C,C20628G,A23063T,C23271A,C23347T,A23403G,C23604A,C23709T,T24506G,G24914C,C27972T,C28000A,G28048T,G28075T,A28095T,A28111G,G28280C,A28281T,T28282A,T28688C,G28881A,G28882A,G28883C,C28977T	27	N:D3L,N:R203K,N:G204R,N:S235F,ORF1a:P971L,ORF1a:T1001I,ORF1a:T1542I,ORF1a:A1708D,ORF1a:I2230T,ORF1a:M2259I,ORF1a:D3222N,ORF1a:T4159I,ORF1b:P218L,ORF1b:P314L,ORF8:Q27*,ORF8:P36Q,ORF8:R52I,ORF8:C61F,ORF8:K68*,ORF8:Y73C,S:N501Y,S:A570D,S:D614G,S:P681H,S:T716I,S:S982A,S:D1	6	811

hCoV-19/Mexico/NLE-IBT_IMSS_1800/2021	EPI_ISL_2801679	OL461476	B.1.1.7	GRY	40	C241T,C913T,C3037T,C3177T,C3267T,C4890T,C5388A,C5986T,T6954C,G9929A,A10195G,C11050T,C12741T,C14120T,C14408T,C14676T,C15279T,T16176C,C20628G,A23063T,C23271A,A23403G,C23604A,C23709T,T24506G,G24914C,C27972T,C28000A,G28048T,G28075T,A28095T,A28111G,G28280C,A28281T,T28282A,T28688C,G28881A,G28882A,G28883C,C28977T	26	N:D3L,N:R203K,N:G204R,N:S235F,ORF1a:P971L,ORF1a:T1001I,ORF1a:T1542I,ORF1a:A1708D,ORF1a:I2230T,ORF1a:D3222N,ORF1a:T4159I,ORF1b:P218L,ORF1b:P314L,ORF8:Q27*,ORF8:P36Q,ORF8:R52I,ORF8:C61F,ORF8:K68*,ORF8:Y73C,S:N501Y,S:A570D,S:D614G,S:P681H,S:T716I,S:S982A,S:D1118H	6	764
hCoV-19/Mexico/NLE-IBT_IMSS_1809/2021	EPI_ISL_2801685	OL461477	B.1.1.7	GRY	41	C241T,C913T,C2110T,C3037T,C3177T,C3267T,C4890T,C5388A,C5986T,T6954C,G7042T,G9929A,A10195G,C12741T,C14120T,C14408T,C14676T,C15279T,T16176C,C20628G,A23063T,C23271A,A23403G,C23604A,C23709T,T24506G,G24914C,C27972T,C28000A,G28048T,G28075T,A28095T,A28111G,G28280C,A28281T,T28282A,T28688C,G28881A,G28882A,G28883C,C28977T	27	N:D3L,N:R203K,N:G204R,N:S235F,ORF1a:P971L,ORF1a:T1001I,ORF1a:T1542I,ORF1a:A1708D,ORF1a:I2230T,ORF1a:M2259I,ORF1a:D3222N,ORF1a:T4159I,ORF1b:P218L,ORF1b:P314L,ORF8:Q27*,ORF8:P36Q,ORF8:R52I,ORF8:C61F,ORF8:K68*,ORF8:Y73C,S:N501Y,S:A570D,S:D614G,S:P681H,S:T716I,S:S982A,S:D1	6	1
hCoV-19/Mexico/NLE-IBT_IMSS_1810/2021	EPI_ISL_2801686	OL461478	B.1.1.7	GRY	40	C241T,C913T,C3037T,C3267T,C5388A,C5986T,T6954C,G7042T,A10195G,C10834T,G10870T,C14120T,C14408T,C14676T,C15279T,C15933T,T16176C,C16869T,C17547T,A23063T,C23271A,A23403G,C23604A,C23709T,T24506G,G24914C,A27745G,C27972T,G28048T,G28075T,A28095T,A28111G,G28280C,A28281T,T28282A,G28881A,G28882A,G28883C,C28977T,G29422C	23	N:D3L,N:R203K,N:G204R,N:S235F,ORF1a:T1001I,ORF1a:A1708D,ORF1a:I2230T,ORF1a:M2259I,ORF1b:P218L,ORF1b:P314L,ORF7a:R118G,ORF8:Q27*,ORF8:R52I,ORF8:C61F,ORF8:K68*,ORF8:Y73C,S:N501Y,S:A570D,S:D614G,S:P681H,S:T716I,S:S982A,S:D1118H	6	706
hCoV-19/Mexico/NLE-IBT_IMSS_66028-NC/2021	EPI_ISL_2801687	OL461479	B.1.1.7	GRY	37	C913T,C2453T,C3037T,C3267T,C5388A,C5986T,T6954C,T9445C,C9611T,C14408T,C14676T,C15279T,T16176C,A18470G,C18568T,G18762T,C21855T,G21974C,A23063T,C23271A,A23403G,C23604A,C23709T,T24506G,G24914C,G25534T,C27972T,G28048T,A28111G,G28280C,A28281T,T28282A,A28295G,G28881A,G28882A,G28883C,C28977T	27	N:D3L,N:N8D,N:R203K,N:G204R,N:S235F,ORF1a:L730F,ORF1a:T1001I,ORF1a:A1708D,ORF1a:I2230T,ORF1a:L3116F,ORF1b:P314L,ORF1b:D1668G,ORF1b:L1701F,ORF1b:M1765I,ORF3a:V48F,ORF8:Q27*,ORF8:R52I,ORF8:Y73C,S:S98F,S:D138H,S:N501Y,S:A570D,S:D614G,S:P681H,S:T716I,S:S982A,S:D1118H	6	1305
hCoV-19/Mexico/NLE-LANGEBIO_IMSS_0829/2021	EPI_ISL_2671625	OL461480	B.1.1.7	GRY	37	C241T,C913T,C3037T,C3267T,C5388A,C5986T,T6954C,C9693T,C11572T,C14408T,C14676T,C15279T,T16176C,T21709C,T23042C,A23063T,C23271A,A23403G,C23604A,C23709T,T24506G,G24914C,G25218T,C25553T,C27615T,C27689T,C27972T,G28048T,A28095T,A28111G,G28280C,A28281T,T28282A,G28881A,G28882A,G28883C,C28977T	24	N:D3L,N:R203K,N:G204R,N:S235F,ORF1a:T1001I,ORF1a:A1708D,ORF1a:I2230T,ORF1a:A3143V,ORF1b:P314L,ORF3a:A54V,ORF7a:P99L,ORF8:Q27*,ORF8:R52I,ORF8:K68*,ORF8:Y73C,S:S494P,S:N501Y,S:A570D,S:D614G,S:P681H,S:T716I,S:S982A,S:D1118H,S:G1219V	6	3
hCoV-19/Mexico/NLE-LANGEBIO_IMSS_0833/2021	EPI_ISL_2671626	OL580733	B.1.1.7	GRY	34	C913T,C3037T,C3267T,G4904T,C5388A,C5986T,T6954C,A10195G,C14120T,C14408T,C14676T,C15279T,C15933T,T16176C,A23063T,C23271A,A23403G,C23604A,C23709T,T24506G,G24914C,G27364C,A27745G,C27972T,G28048T,G28075T,A28111G,G28280C,A28281T,T28282A,G28881A,G28882A,G28883C,C28977T	23	N:D3L,N:R203K,N:G204R,N:S235F,ORF1a:T1001I,ORF1a:D1547Y,ORF1a:A1708D,ORF1a:I2230T,ORF1b:P218L,ORF1b:P314L,ORF6:E55Q,ORF7a:R118G,ORF8:Q27*,ORF8:R52I,ORF8:C61F,ORF8:Y73C,S:N501Y,S:A570D,S:D614G,S:P681H,S:T716I,S:S982A,S:D1118H	6	482
hCoV-19/Mexico/NLE-LANGEBIO_IMSS_0851/2021	EPI_ISL_2671631	OL461481	B.1.1.7	GRY	42	C241T,C913T,C2110T,C3037T,C3177T,C3267T,C4890T,C5388A,C5986T,T6954C,G7042T,G9929A,A10195G,C11050T,C12741T,C14120T,C14408T,C14676T,C15279T,T16176C,C20628G,A23063T,C23271A,A23403G,C23604A,C23709T,T24506G,G24914C,C27972T,C28000A,G28048T,G28075T,A28095T,A28111G,G28280C,A28281T,T28282A,T28688C,G28881A,G28882A,G28883C,C2897	27	N:D3L,N:R203K,N:G204R,N:S235F,ORF1a:P971L,ORF1a:T1001I,ORF1a:T1542I,ORF1a:A1708D,ORF1a:I2230T,ORF1a:M2259I,ORF1a:D3222N,ORF1a:T4159I,ORF1b:P218L,ORF1b:P314L,ORF8:Q27*,ORF8:P36Q,ORF8:R52I,ORF8:C61F,ORF8:K68*,ORF8:Y73C,S:N501Y,S:A570D,S:D614G,S:P681H,S:T716I,S:S982A,S:D1	6	0
hCoV-19/Mexico/NLE-LANGEBIO_IMSS_0857/2021	EPI_ISL_2671633	OL461482	B.1.1.7	GRY	32	C823T,C913T,C1912T,C3037T,C3177T,C3267T,C5388A,C5986T,T6954C,C10252T,C14408T,C14676T,C15279T,T16176C,A23063T,C23271A,A23403G,C23604A,C23709T,T24506G,G24914C,C27879T,C27972T,G28048T,A28111G,G28280C,A28281T,T28282A,G28881A,G28882A,G28883C,C2	20	N:D3L,N:R203K,N:G204R,N:S235F,ORF1a:P971L,ORF1a:T1001I,ORF1a:A1708D,ORF1a:I2230T,ORF1b:P314L,ORF7b:H42Y,ORF8:Q27*,ORF8:R52I,ORF8:Y73C,S:N501Y,S:A570D,S:D614G,S:P681H,S:T716I,S:S982A,S:D1118H	6	147
hCoV-19/Mexico/NLE-LANGEBIO_IMSS_1355/2021	EPI_ISL_2942486	OL461483	B.1.1.7	GRY	42	C241T,C913T,C2110T,C3037T,C3177T,C3267T,C4890T,C5388A,C5986T,T6954C,G7042T,G9929A,A10195G,C11050T,C12741T,C14120T,C14408T,C14676T,C15279T,T16176C,C20628G,A23063T,C23271A,A23403G,C23604A,C23709T,T24506G,G24914C,C27972T,C28000A,G28048T,G28075T,A28095T,A28111G,G28280C,A28281T,T28282A,T28688C,G28881A,G28882A,G28883C,C2897	27	N:D3L,N:R203K,N:G204R,N:S235F,ORF1a:P971L,ORF1a:T1001I,ORF1a:T1542I,ORF1a:A1708D,ORF1a:I2230T,ORF1a:M2259I,ORF1a:D3222N,ORF1a:T4159I,ORF1b:P218L,ORF1b:P314L,ORF8:Q27*,ORF8:P36Q,ORF8:R52I,ORF8:C61F,ORF8:K68*,ORF8:Y73C,S:N501Y,S:A570D,S:D614G,S:P681H,S:T716I,S:S982A,S:D1	6	96
hCoV-19/Mexico/NLE-LANGEBIO_IMSS_1357/2021	EPI_ISL_2942488	OL461484	B.1.1.7	GRY	37	C913T,C2388T,C3037T,C3267T,C5388A,C5986T,T6954C,G7042T,C7834T,A10195G,C14120T,C14408T,C14676T,C15279T,T16176C,C16833T,C18508T,C22347T,A23063T,C23271A,A23403G,C23604A,C23709T,T24506G,G24914C,C27972T,G28048T,G28075T,A28111G,G28280C,A28281T,T28282A,G28851T,G28881A,G28882A,G28883C,C28977T	25	N:D3L,N:S193I,N:R203K,N:G204R,N:S235F,ORF1a:T708I,ORF1a:T1001I,ORF1a:A1708D,ORF1a:I2230T,ORF1a:M2259I,ORF1b:P218L,ORF1b:P314L,ORF1b:L1681F,ORF8:Q27*,ORF8:R52I,ORF8:C61F,ORF8:Y73C,S:A262V,S:N501Y,S:A570D,S:D614G,S:P681H,S:T716I,S:S982A,S:D1118H	6	605
hCoV-19/Mexico/NLE-LANGEBIO_IMSS_1365/2021	EPI_ISL_2942496	OL461485	Q.3	GRY	39	G149T,C241T,C913T,C1190T,C3037T,C3267T,C4582T,G5023A,C5388A,C5986T,T6954C,G10396T,C12651T,C14408T,C14676T,C15279T,T16176C,A17615G,G20962A,C21108T,A23063T,C23271A,A23403G,C23604A,C23709T,T24506G,G24914C,G26730C,C27972T,G28048T,A28111G,G28280C,A28281T,T28282A,G28881A,G28882A,G28883C,C28977T,G29440T	25	M:V70L,N:D3L,N:R203K,N:G204R,N:S235F,N:Q389H,ORF1a:P309S,ORF1a:T1001I,ORF1a:M1586I,ORF1a:A1708D,ORF1a:I2230T,ORF1a:T4129I,ORF1b:P314L,ORF1b:K1383R,ORF1b:D2499N,ORF8:Q27*,ORF8:R52I,ORF8:Y73C,S:N501Y,S:A570D,S:D614G,S:P681H,S:T716I,S:S982A,S:D1118H	6	84
hCoV-19/Mexico/NLE-LANGEBIO_IMSS_1660/2021	EPI_ISL_2942757	OL461486	B.1.1.7	GRY	44	C241T,C913T,C2110T,C2143T,C2675T,C3037T,C3267T,C5144T,C5388A,C5986T,C6016T,A6403T,C6539T,T6954C,G7042T,C9246T,A10195G,C10834T,C14120T,C14408T,C14676T,C15279T,T16176C,A23063T,C23271A,A23403G,C23604A,C23709T,T24506G,G24914C,G25650T,C27972T,G28048T,G28075T,A28095T,A28111G,G28280C,A28281T,T28282A,G28881A,G28882A,G28883C,C2	26	N:D3L,N:R203K,N:G204R,N:S235F,ORF1a:P804S,ORF1a:T1001I,ORF1a:A1708D,ORF1a:H2092Y,ORF1a:I2230T,ORF1a:M2259I,ORF1a:A2994V,ORF1b:P218L,ORF1b:P314L,ORF3a:L86F,ORF8:Q27*,ORF8:R52I,ORF8:C61F,ORF8:K68*,ORF8:Y73C,S:N501Y,S:A570D,S:D614G,S:P681H,S:T716I,S:S982A,S:D1118H	6	0
hCoV-19/Mexico/NLE-LANGEBIO_IMSS_1666/2021	EPI_ISL_2942762	OL461487	B.1.1.7	GRY	44	C241T,C913T,C2110T,C3037T,C3267T,C5388A,C5986T,C6813T,T6954C,G7042T,A7479G,A10195G,C10834T,G10870T,C14120T,C14408T,C14676T,C15279T,C15933T,T16176C,C16869T,C17547T,G22218A,A23063T,C23271A,A23403G,C23604A,C23709T,T24506G,G24914C,A27745G,C27972T,G28048T,G28075T,A28095T,A28111G,G28280C,A28281T,T28282A,G28881A,G28882A,G288	26	N:D3L,N:R203K,N:G204R,N:S235F,ORF1a:T1001I,ORF1a:A1708D,ORF1a:T2183I,ORF1a:I2230T,ORF1a:M2259I,ORF1a:N2405S,ORF1b:P218L,ORF1b:P314L,ORF7a:R118G,ORF8:Q27*,ORF8:R52I,ORF8:C61F,ORF8:K68*,ORF8:Y73C,S:G219D,S:N501Y,S:A570D,S:D614G,S:P681H,S:T716I,S:S982A,S:D1118H	6	0
hCoV-19/Mexico/NLE-LANGEBIO_IMSS_1680/2021	EPI_ISL_2942771	OL461488	B.1.1.7	GRY	40	C241T,T288G,C913T,C3037T,C3267T,C5388A,C5986T,T6954C,A10195G,C10834T,G10870T,C14120T,C14408T,C14676T,C15279T,C15933T,T16176C,C16869T,C17547T,A23063T,C23271A,A23403G,C23604A,C23709T,T24506G,G24914C,A27745G,C27972T,G28048T,G28075T,A28095T,A28111G,G28280C,A28281T,T28282A,G28881A,G28882A,G28883C,C28977T,G29422C	23	N:D3L,N:R203K,N:G204R,N:S235F,ORF1a:F8C,ORF1a:T1001I,ORF1a:A1708D,ORF1a:I2230T,ORF1b:P218L,ORF1b:P314L,ORF7a:R118G,ORF8:Q27*,ORF8:R52I,ORF8:C61F,ORF8:K68*,ORF8:Y73C,S:N501Y,S:A570D,S:D614G,S:P681H,S:T716I,S:S982A,S:D1118H	6	39
hCoV-19/Mexico/NLE-LANGEBIO_IMSS_1688/2021	EPI_ISL_2942778	OL461489	B.1.1.7	GRY	40	C241T,C913T,C2110T,C3037T,C3177T,C3267T,C4890T,C5388A,C5986T,T6954C,G7042T,G9929A,A10195G,C12741T,C14120T,C14408T,C14676T,C15279T,T16176C,G17721T,C20628G,A23063T,C23271A,A23403G,C23604A,C23709T,T24506G,G24914C,T27308A,C27972T,G28048T,A28111G,G28280C,A28281T,T28282A,A28295G,G28881A,G28882A,G28883C,C28977T	26	N:D3L,N:N8D,N:R203K,N:G204R,N:S235F,ORF1a:P971L,ORF1a:T1001I,ORF1a:T1542I,ORF1a:A1708D,ORF1a:I2230T,ORF1a:M2259I,ORF1a:D3222N,ORF1a:T4159I,ORF1b:P218L,ORF1b:P314L,ORF6:I36K,ORF8:Q27*,ORF8:R52I,ORF8:Y73C,S:N501Y,S:A570D,S:D614G,S:P681H,S:T716I,S:S982A,S:D1118H	6	100
hCoV-19/Mexico/OAX_INER_IMSS_1120/2021	EPI_ISL_2490345	OL461490	B.1.1.7	GRY	39	C241T,C913T,C3037T,C3267T,C5388A,C5944T,C5986T,G6368A,T6954C,C9967T,C13585T,C14408T,C14676T,T15096C,C15279T,T16176C,G21901A,C22987T,C22993T,A23063T,C23271A,A23403G,C23604A,C23709T,T24506G,G24914C,C25688T,C27972T,G28048T,A28095T,A28111G,G28280C,A28281T,T28282A,G28881A,G28882A,G28883C,G28884C,C28977T	21	N:D3L,N:R203K,N:G204P,N:S235F,ORF1a:T1001I,ORF1a:A1708D,ORF1a:G2035R,ORF1a:I2230T,ORF1b:P314L,ORF3a:A99V,ORF8:Q27*,ORF8:R52I,ORF8:K68*,ORF8:Y73C,S:N501Y,S:A570D,S:D614G,S:P681H,S:T716I,S:S982A,S:D1118H	6	0
hCoV-19/Mexico/OAX_LANGEBIO_IMSS_0779/2021	EPI_ISL_2402190	OL461491	B.1.1.7	GRY	36	T201A,C241T,C913T,C2110T,C3037T,C3267T,C5388A,C5986T,T6954C,G7042T,A10195G,C14120T,C14408T,C14676T,C15279T,T16176C,A23063T,C23271A,A23403G,C23604A,C23709T,T24506G,G24914C,T25612G,C27972T,G28048T,G28075T,A28095T,A28111G,G28280C,A28281T,T28282A,G28881A,G28882A,G28883C,C28977T	23	N:D3L,N:R203K,N:G204R,N:S235F,ORF1a:T1001I,ORF1a:A1708D,ORF1a:I2230T,ORF1a:M2259I,ORF1b:P218L,ORF1b:P314L,ORF3a:S74A,ORF8:Q27*,ORF8:R52I,ORF8:C61F,ORF8:K68*,ORF8:Y73C,S:N501Y,S:A570D,S:D614G,S:P681H,S:T716I,S:S982A,S:D1118H	6	249
hCoV-19/Mexico/OAX_LANGEBIO_IMSS_0812/2021	EPI_ISL_2402222	OL461492	B.1.1.7	GRY	37	C241T,C913T,C3037T,C3267T,C5388A,C5944T,C5986T,T6954C,C9967T,C13585T,C14408T,C14676T,T15096C,C15279T,T16176C,A17344G,G18412T,C22987T,A23063T,C23271A,A23403G,C23604A,C23709T,T24506G,G24914C,C27972T,G28048T,A28095T,A28111G,G28280C,A28281T,T28282A,G28881A,G28882A,G28883C,G28884C,C28977T	21	N:D3L,N:R203K,N:G204P,N:S235F,ORF1a:T1001I,ORF1a:A1708D,ORF1a:I2230T,ORF1b:P314L,ORF1b:I1293V,ORF1b:V1649F,ORF8:Q27*,ORF8:R52I,ORF8:K68*,ORF8:Y73C,S:N501Y,S:A570D,S:D614G,S:P681H,S:T716I,S:S982A,S:D1118H	6	801
hCoV-19/Mexico/OAX-IBT_IMSS_1929/2021	EPI_ISL_2801795	OL461493	B.1.1.7	GRY	39	C222T,C241T,C913T,C2110T,C3037T,C3267T,C4423T,C5388A,C5986T,C6681T,T6954C,G7042T,A10359G,C14120T,C14408T,C14676T,C15279T,T16176C,C17762T,A23063T,C23271A,A23403G,C23604A,G23608T,C23709T,T24506G,G24914C,T25180C,C27972T,G28048T,A28095T,A28111G,G28280C,A28281T,T28282A,G28881A,G28882A,G28883C,C28977T	24	N:D3L,N:R203K,N:G204R,N:S235F,ORF1a:T1001I,ORF1a:A1708D,ORF1a:A2139V,ORF1a:I2230T,ORF1a:M2259I,ORF1a:K3365R,ORF1b:P218L,ORF1b:P314L,ORF1b:A1432V,ORF8:Q27*,ORF8:R52I,ORF8:K68*,ORF8:Y73C,S:N501Y,S:A570D,S:D614G,S:P681H,S:T716I,S:S982A,S:D1118H	6	0

hCoV-19/Mexico/OAX-IBT_IMSS_1980/2021	EPI_ISL_2801837	OL461494	B.1.1.7	GRY	38	C241T,C913T,C2453T,C3037T,C3267T,C5388A,C5986T,C6433T,C6573T,T6954C,C9611T,C14408T,C14676T,C15279T,T16176C,C21008T,C21855T,G21974C,A23063T,C23271A,A23403G,C23604A,C23709T,T24506G,G24914C,C27643T,C27972T,G28048T,A28111G,G28280C,A28281T,T28282A,A28295G,G28713A,G28881A,G28882A,G28883C,C28977T	27	N:D3L,N:N8D,N:G147D,N:R203K,N:G204R,N:S235F,ORF1a:L730F,ORF1a:T1001I,ORF1a:A1708D,ORF1a:S2103F,ORF1a:I2230T,ORF1a:L3116F,ORF1b:P314L,ORF1b:T2514I,ORF7a:P84S,ORF8:Q27*,ORF8:R52I,ORF8:Y73C,S:S98F,S:D138H,S:N501Y,S:A570D,S:D614G,S:P681H,S:T716I,S:S982A,S:D1118H	6	1
hCoV-19/Mexico/OAX-IBT_IMSS_1981/2021	EPI_ISL_2801838	OL461495	B.1.1.7	GRY	37	C241T,C913T,C2453T,C3037T,C3267T,C5388A,C5986T,C6433T,C6573T,T6954C,C9611T,C14408T,C14676T,C15279T,T16176C,C21008T,C21855T,G21974C,A23063T,C23271A,A23403G,C23604A,C23709T,T24506G,G24914C,C27643T,C27972T,G28048T,A28111G,G28280C,A28281T,T28282A,A28295G,G28881A,G28882A,G28883C,C28977T	26	N:D3L,N:N8D,N:R203K,N:G204R,N:S235F,ORF1a:L730F,ORF1a:T1001I,ORF1a:A1708D,ORF1a:S2103F,ORF1a:I2230T,ORF1a:L3116F,ORF1b:P314L,ORF1b:T2514I,ORF7a:P84S,ORF8:Q27*,ORF8:R52I,ORF8:Y73C,S:S98F,S:D138H,S:N501Y,S:A570D,S:D614G,S:P681H,S:T716I,S:S982A,S:D1118H	6	0
hCoV-19/Mexico/OAX-LANGEBIO_IMSS_1126/2021	EPI_ISL_2671639	OL461496	B.1.1.7	GRY	38	C241T,C913T,C2110T,C3037T,C3267T,C5388A,C5986T,T6954C,G7042T,A10195G,T10816C,C14120T,C14408T,C14676T,C15279T,T16176C,A23063T,C23271A,A23403G,C23604A,C23709T,C23854T,T24506G,G24914C,C25002T,C27513T,C27972T,G28048T,G28075T,A28095T,A28111G,G28280C,A28281T,T28282A,G28881A,G28882A,G28883C,C28977T	23	N:D3L,N:R203K,N:G204R,N:S235F,ORF1a:T1001I,ORF1a:A1708D,ORF1a:I2230T,ORF1a:M2259I,ORF1b:P218L,ORF1b:P314L,ORF8:Q27*,ORF8:R52I,ORF8:C61F,ORF8:K68*,ORF8:Y73C,S:N501Y,S:A570D,S:D614G,S:P681H,S:T716I,S:S982A,S:D1118H,S:S1147L	6	21
hCoV-19/Mexico/PUE_IBT_IMSS_1252/2021	EPI_ISL_2391628	OL461497	B.1.1.7	GRY	36	C241T,C913T,C3037T,C3267T,C5388A,C5986T,T6954C,G7042T,A10195G,C14120T,C14408T,C14676T,C15279T,T16176C,A23063T,C23271A,A23403G,C23604A,C23709T,T24506G,G24914C,T25612G,C27434T,C27972T,G28048T,G28075T,A28095T,A28111G,C28132T,G28280C,A28281T,T28282A,G28881A,G28882A,G28883C,C28977T	25	N:D3L,N:R203K,N:G204R,N:S235F,ORF1a:T1001I,ORF1a:A1708D,ORF1a:I2230T,ORF1a:M2259I,ORF1b:P218L,ORF1b:P314L,ORF3a:S74A,ORF7a:T14I,ORF8:Q27*,ORF8:R52I,ORF8:C61F,ORF8:K68*,ORF8:Y73C,ORF8:T80I,S:N501Y,S:A570D,S:D614G,S:P681H,S:T716I,S:S982A,S:D1118H	6	427
hCoV-19/Mexico/PUE_IBT_IMSS_1254/2021	EPI_ISL_2391580	OL461498	B.1.1.7	GRY	37	T201A,C241T,C913T,C2110T,C3037T,C3267T,C5388A,C5986T,T6954C,G7042T,C9994T,A10195G,C13329T,C14120T,C14408T,C14676T,C15279T,T16176C,A23063T,C23271A,A23403G,C23604A,C23709T,T24506G,G24914C,C27972T,G28048T,G28075T,A28095T,A28111G,G28280C,A28281T,T28282A,G28881A,G28882A,G28883C,C28977T	23	N:D3L,N:R203K,N:G204R,N:S235F,ORF1a:T1001I,ORF1a:A1708D,ORF1a:I2230T,ORF1a:M2259I,ORF1a:T4355I,ORF1b:P218L,ORF1b:P314L,ORF8:Q27*,ORF8:R52I,ORF8:C61F,ORF8:K68*,ORF8:Y73C,S:N501Y,S:A570D,S:D614G,S:P681H,S:T716I,S:S982A,S:D1118H	6	0
hCoV-19/Mexico/PUE_IBT_IMSS_1522/2021	EPI_ISL_2681162	OL461499	B.1.1.7	GRY	38	C241T,C913T,C2449T,C3037T,A3196T,C3267T,C5388A,C5944T,C5986T,C6402T,T6954C,C9166T,A10323G,C14408T,C14676T,T15096C,C15279T,T16176C,A23063T,C23271A,A23403G,C23604A,C23709T,T24506G,G24914C,C27972T,G28048T,A28095T,A28111G,G28280C,A28281T,T28282A,G28881A,G28882A,G28883C,G28884C,C28977T,T29710C	22	N:D3L,N:R203K,N:G204P,N:S235F,ORF1a:E977D,ORF1a:T1001I,ORF1a:A1708D,ORF1a:P2046L,ORF1a:I2230T,ORF1a:K3353R,ORF1b:P314L,ORF8:Q27*,ORF8:R52I,ORF8:K68*,ORF8:Y73C,S:N501Y,S:A570D,S:D614G,S:P681H,S:T716I,S:S982A,S:D1118H	6	0
hCoV-19/Mexico/PUE_IBT_IMSS_1523/2021	EPI_ISL_2681191	OL461500	B.1.1.7	GRY	37	T201A,C241T,C913T,C2110T,C3037T,C3267T,C5388A,C5986T,T6954C,G7042T,A10195G,C14120T,C14408T,C14676T,C15279T,T16176C,A23063T,C23271A,A23403G,C23604A,C23709T,T24506G,G24914C,T25612G,C27972T,G28048T,G28075T,A28095T,A28111G,G28280C,A28281T,T28282A,G28881A,G28882A,G28883C,G28904T,C28977T	24	N:D3L,N:R203K,N:G204R,N:A211S,N:S235F,ORF1a:T1001I,ORF1a:A1708D,ORF1a:I2230T,ORF1a:M2259I,ORF1b:P218L,ORF1b:P314L,ORF3a:S74A,ORF8:Q27*,ORF8:R52I,ORF8:C61F,ORF8:K68*,ORF8:Y73C,S:N501Y,S:A570D,S:D614G,S:P681H,S:T716I,S:S982A,S:D1118H	6	0
hCoV-19/Mexico/PUE_IBT_IMSS_1549/2021	EPI_ISL_2681347	OL461501	B.1.1.7	GRY	39	C241T,T754C,C913T,C3037T,C3267T,C5388A,C5986T,T6954C,C7299T,A13768T,C14408T,C14676T,T15096C,C15279T,T16176C,C19862T,A23063T,C23271A,A23403G,C23604A,C23709T,T24506G,G24914C,C26606T,G27281T,C27972T,G28048T,A28111G,C28153T,G28280C,A28281T,T28282A,A28877T,G28878C,G28881A,G28882A,G28883C,C28977T,A29491G	23	N:D3L,N:R203K,N:G204R,N:S235F,ORF1a:T1001I,ORF1a:A1708D,ORF1a:I2230T,ORF1a:A2345V,ORF1b:M101L,ORF1b:P314L,ORF1b:A2132V,ORF6:W27L,ORF8:Q27*,ORF8:R52I,ORF8:Y73C,ORF8:T87I,S:N501Y,S:A570D,S:D614G,S:P681H,S:T716I,S:S982A,S:D1118H	6	704
hCoV-19/Mexico/PUE_IBT_IMSS_1676/2021	EPI_ISL_2681149	OL461502	B.1.1.7	GRY	39	C203T,C241T,C913T,G1820A,C3037T,C3267T,C5388A,G5924A,C5986T,T6954C,G9526T,A12162G,C14408T,C14676T,C15279T,C15720T,T16176C,A17615G,T19363C,A21137G,A23063T,C23248T,C23271A,A23403G,C23604A,C23709T,T24506G,G24914C,G25135T,C27972T,G28048T,A28111G,G28280C,A28281T,T28282A,G28881A,G28882A,G28883C,C28977T	25	N:D3L,N:R203K,N:G204R,N:S235F,ORF1a:G519S,ORF1a:T1001I,ORF1a:A1708D,ORF1a:V1887I,ORF1a:I2230T,ORF1a:M3087I,ORF1a:Q3966R,ORF1b:P314L,ORF1b:K1383R,ORF1b:K2557R,ORF8:Q27*,ORF8:R52I,ORF8:Y73C,S:N501Y,S:A570D,S:D614G,S:P681H,S:T716I,S:S982A,S:D1118H,S:K1191N	6	0
hCoV-19/Mexico/PUE_INER_IMSS_1277/2021	EPI_ISL_2490350	OL461503	B.1.1.7	GRY	37	T201A,C241T,C913T,C2110T,C3037T,C3267T,C5388A,C5986T,T6954C,G7042T,A10195G,C13329T,C14120T,C14408T,C14676T,C15279T,T16176C,A23063T,C23271A,A23403G,C23604A,C23709T,T24506G,G24914C,T25612G,C27972T,G28048T,G28075T,A28095T,A28111G,G28280C,A28281T,T28282A,G28881A,G28882A,G28883C,C28977T	24	N:D3L,N:R203K,N:G204R,N:S235F,ORF1a:T1001I,ORF1a:A1708D,ORF1a:I2230T,ORF1a:M2259I,ORF1a:T4355I,ORF1b:P218L,ORF1b:P314L,ORF3a:S74A,ORF8:Q27*,ORF8:R52I,ORF8:C61F,ORF8:K68*,ORF8:Y73C,S:N501Y,S:A570D,S:D614G,S:P681H,S:T716I,S:S982A,S:D1118H	6	0
hCoV-19/Mexico/PUE_INER_IMSS_1306/2021	EPI_ISL_2490351	OL461504	B.1.1.7	GRY	39	T201A,C241T,C913T,C2110T,C3037T,C3041T,C3267T,C5388A,C5986T,T6954C,G7042T,C9994T,A10195G,C13329T,C14120T,C14408T,C14676T,C15279T,T16176C,A23063T,C23271A,A23403G,C23604A,C23709T,T24506G,G24914C,T25612G,C27972T,G28048T,G28075T,A28095T,A28111G,G28280C,A28281T,T28282A,G28881A,G28882A,G28883C,C28977T	25	N:D3L,N:R203K,N:G204R,N:S235F,ORF1a:P926S,ORF1a:T1001I,ORF1a:A1708D,ORF1a:I2230T,ORF1a:M2259I,ORF1a:T4355I,ORF1b:P218L,ORF1b:P314L,ORF3a:S74A,ORF8:Q27*,ORF8:R52I,ORF8:C61F,ORF8:K68*,ORF8:Y73C,S:N501Y,S:A570D,S:D614G,S:P681H,S:T716I,S:S982A,S:D1118H	6	0
hCoV-19/Mexico/PUE_INER_IMSS_1322/2021	EPI_ISL_2490352	OL461505	B.1.1.7	GRY	38	C241T,C913T,C3037T,C3267T,C5388A,C5986T,T6954C,C7299T,A13768T,C14408T,C14676T,T15096C,C15279T,T16176C,C19862T,A23063T,C23271A,A23403G,C23604A,C23709T,T24506G,G24914C,C26606T,G27281T,C27972T,G28048T,A28095T,A28111G,G28280C,A28281T,T28282A,A28877T,G28878C,G28881A,G28882A,G28883C,C28977T,A29491G	23	N:D3L,N:R203K,N:G204R,N:S235F,ORF1a:T1001I,ORF1a:A1708D,ORF1a:I2230T,ORF1a:A2345V,ORF1b:M101L,ORF1b:P314L,ORF1b:A2132V,ORF6:W27L,ORF8:Q27*,ORF8:R52I,ORF8:K68*,ORF8:Y73C,S:N501Y,S:A570D,S:D614G,S:P681H,S:T716I,S:S982A,S:D1118H	6	0
hCoV-19/Mexico/PUE_INER_IMSS_1323/2021	EPI_ISL_2490353	OL461506	B.1.1.7	GRY	39	C241T,C913T,C3037T,C3267T,C5388A,C5944T,C5986T,T6954C,C9870T,C9967T,C11591T,C13585T,C14408T,C14676T,T15096C,C15279T,T16176C,G18412T,C19983T,C22987T,A23063T,C23271A,A23403G,C23604A,C23709T,T24506G,G24914C,C27972T,G28048T,A28095T,A28111G,G28280C,A28281T,T28282A,G28881A,G28882A,G28883C,G28884C,C28977T	22	N:D3L,N:R203K,N:G204P,N:S235F,ORF1a:T1001I,ORF1a:A1708D,ORF1a:I2230T,ORF1a:T3202M,ORF1a:L3776F,ORF1b:P314L,ORF1b:V1649F,ORF8:Q27*,ORF8:R52I,ORF8:K68*,ORF8:Y73C,S:N501Y,S:A570D,S:D614G,S:P681H,S:T716I,S:S982A,S:D1118H	6	0
hCoV-19/Mexico/PUE_LANGEBIO_IMSS_0438/2021	EPI_ISL_1662170	OL461507	B.1.1.7	GRY	41	C241T,C913T,C3037T,C3267T,C5388A,C5944T,C5986T,G6647A,T6954C,C14408T,C14676T,T15096C,C15279T,T16176C,C17676T,C18183T,C19524T,A21141G,A21519C,G22217A,A23063T,C23271A,A23403G,C23604A,C23709T,A24070T,T24506G,G24914C,C25624T,C27972T,G28048T,A28095T,A28111G,G28280C,A28281T,T28282A,G28881A,G28882A,G28883C,G28884C,C28977T	24	N:D3L,N:R203K,N:G204P,N:S235F,ORF1a:T1001I,ORF1a:A1708D,ORF1a:A2128T,ORF1a:I2230T,ORF1b:P314L,ORF1b:R2684S,ORF3a:H78Y,ORF8:Q27*,ORF8:R52I,ORF8:K68*,ORF8:Y73C,S:G219S,S:N501Y,S:A570D,S:D614G,S:P681H,S:T716I,S:Q836H,S:S982A,S:D1118H	6	0
hCoV-19/Mexico/PUE_LANGEBIO_IMSS_0610/2021	EPI_ISL_2402028	OL461508	B.1.1.7	GRY	38	C241T,C913T,C2110T,G2782T,C3037T,C3267T,C4456T,C5388A,C5986T,T6954C,T9931C,C14120T,C14408T,C14676T,C15279T,T16176C,C16733T,C19390T,T22020C,A23063T,C23271A,A23403G,C23604A,C23709T,T24506G,C24904T,G24914C,C27972T,G28048T,A28095T,A28111G,G28280C,A28281T,T28282A,G28881A,G28882A,G28883C,C28977T	23	N:D3L,N:R203K,N:G204R,N:S235F,ORF1a:T1001I,ORF1a:A1708D,ORF1a:I2230T,ORF1b:P218L,ORF1b:P314L,ORF1b:S1089L,ORF1b:P1975S,ORF8:Q27*,ORF8:R52I,ORF8:K68*,ORF8:Y73C,S:M153T,S:N501Y,S:A570D,S:D614G,S:P681H,S:T716I,S:S982A,S:D1118H	6	177
hCoV-19/Mexico/PUE-IBT_IMSS_1889/2021	EPI_ISL_2801758	OL461509	B.1.1.7	GRY	39	T201A,C241T,C913T,C2110T,C3037T,C3267T,C3777T,C5388A,C5986T,T6954C,G7042T,C9532T,A10195G,C13329T,C14120T,C14408T,C14676T,C15279T,T16176C,A23063T,C23271A,A23403G,C23604A,C23709T,T24506G,G24914C,T25612G,C27972T,G28048T,G28075T,A28095T,A28111G,G28280C,A28281T,T28282A,G28881A,G28882A,G28883C,C28977T	25	N:D3L,N:R203K,N:G204R,N:S235F,ORF1a:T1001I,ORF1a:T1171I,ORF1a:A1708D,ORF1a:I2230T,ORF1a:M2259I,ORF1a:T4355I,ORF1b:P218L,ORF1b:P314L,ORF3a:S74A,ORF8:Q27*,ORF8:R52I,ORF8:C61F,ORF8:K68*,ORF8:Y73C,S:N501Y,S:A570D,S:D614G,S:P681H,S:T716I,S:S982A,S:D1118H	6	0
hCoV-19/Mexico/PUE-IBT_IMSS_1911/2021	EPI_ISL_2801776	OL461510	Q.3	GRY	38	G149T,C241T,C913T,C3037T,C3267T,G5023A,C5388A,C5986T,T6954C,G10396T,C12513T,C14408T,C14676T,C15279T,T16176C,A17615G,G20962A,C21108T,A23063T,C23271A,A23403G,C23604A,C23709T,T24506G,G24914C,G26730C,C27972T,G28048T,A28111G,G28280C,A28281T,T28282A,C28603T,G28881A,G28882A,G28883C,C28977T,G29440T	24	M:V70L,N:D3L,N:R203K,N:G204R,N:S235F,N:Q389H,ORF1a:T1001I,ORF1a:M1586I,ORF1a:A1708D,ORF1a:I2230T,ORF1a:T4083M,ORF1b:P314L,ORF1b:K1383R,ORF1b:D2499N,ORF8:Q27*,ORF8:R52I,ORF8:Y73C,S:N501Y,S:A570D,S:D614G,S:P681H,S:T716I,S:S982A,S:D1118H	6	171
hCoV-19/Mexico/PUE-IBT_IMSS_1913/2021	EPI_ISL_2801778	OL461511	B.1.1.7	GRY	36	T201A,C241T,C913T,C3037T,C3267T,C5388A,C5986T,T6954C,G7042T,A10195G,C14120T,C14408T,C14676T,C15279T,T16176C,C19536T,A23063T,C23271A,A23403G,C23604A,C23709T,T24506G,G24914C,T25612G,C27972T,G28048T,G28075T,A28095T,A28111G,G28280C,A28281T,T28282A,G28881A,G28882A,G28883C,C28977T	23	N:D3L,N:R203K,N:G204R,N:S235F,ORF1a:T1001I,ORF1a:A1708D,ORF1a:I2230T,ORF1a:M2259I,ORF1b:P218L,ORF1b:P314L,ORF3a:S74A,ORF8:Q27*,ORF8:R52I,ORF8:C61F,ORF8:K68*,ORF8:Y73C,S:N501Y,S:A570D,S:D614G,S:P681H,S:T716I,S:S982A,S:D1118H	6	590
hCoV-19/Mexico/PUE-IBT_IMSS_1944/2021	EPI_ISL_2801806	OL461512	B.1.1.7	GRY	38	T201A,C241T,C913T,C2110T,C3037T,C3267T,C5388A,C5986T,T6954C,G7042T,A10195G,C14120T,C14408T,C14676T,C15279T,T16176C,C19536T,A22194C,A23063T,C23271A,A23403G,C23604A,C23709T,T24506G,G24914C,T25612G,C27972T,G28048T,G28075T,A28095T,A28111G,G28280C,A28281T,T28282A,G28881A,G28882A,G28883C,C28977T	24	N:D3L,N:R203K,N:G204R,N:S235F,ORF1a:T1001I,ORF1a:A1708D,ORF1a:I2230T,ORF1a:M2259I,ORF1b:P218L,ORF1b:P314L,ORF3a:S74A,ORF8:Q27*,ORF8:R52I,ORF8:C61F,ORF8:K68*,ORF8:Y73C,S:N211T,S:N501Y,S:A570D,S:D614G,S:P681H,S:T716I,S:S982A,S:D1118H	6	10

hCoV-19/Mexico/PUE-IBT_IMSS_2022/2021	EPI_ISL_2801869	OL461513	B.1.1.7	GRY	41	T201A,C241T,C913T,C2110T,C3037T,C3267T,T3905C,C5388A,C5986T,T6954C,G7042T,A10195G,C13329T,C14120T,C14408T,C14676T,C15279T,T16176C,A18473G,G19723T,C21575T,A23063T,C23271A,A23403G,C23604A,C23709T,T24506G,G24914C,T25612G,C27972T,G28048T,G28075T,A28095T,A28111G,G28280C,A28281T,T28282A,G28881A,G28882A,G28883C,C28977T	28	N:D3L,N:R203K,N:G204R,N:S235F,ORF1a:T1001I,ORF1a:F1214L,ORF1a:A1708D,ORF1a:I2230T,ORF1a:M2259I,ORF1a:T4355I,ORF1b:P218L,ORF1b:P314L,ORF1b:Q1669R,ORF1b:V2086F,ORF3a:S74A,ORF8:Q27*,ORF8:R52I,ORF8:C61F,ORF8:K68*,ORF8:Y73C,S:L5F,S:N501Y,S:A570D,S:D614G,S:P681H,S:T716I,S:S9	6	5
hCoV-19/Mexico/PUE-LANGEBIO_IMSS_1091/2021	EPI_ISL_2671642	OL461514	B.1.1.7	GRY	38	C241T,C913T,C3037T,C3267T,C5388A,C5986T,T6954C,A10323G,C11704T,A12162G,C14408T,C14676T,C15279T,C15720T,T16176C,T17009C,A17187G,A17615G,G21063A,A21137G,A23063T,C23271A,A23403G,C23604A,C23709T,T24506G,G24914C,G25135T,C27972T,G28048T,A28111G,G28280C,A28281T,T28282A,G28881A,G28882A,G28883C,C28977T	24	N:D3L,N:R203K,N:G204R,N:S235F,ORF1a:T1001I,ORF1a:A1708D,ORF1a:I2230T,ORF1a:K3353R,ORF1a:Q3966R,ORF1b:P314L,ORF1b:I1181T,ORF1b:K1383R,ORF1b:K2557R,ORF8:Q27*,ORF8:R52I,ORF8:Y73C,S:N501Y,S:A570D,S:D614G,S:P681H,S:T716I,S:S982A,S:D1118H,S:K1191N	6	2
hCoV-19/Mexico/PUE-LANGEBIO_IMSS_1093/2021	EPI_ISL_2671644	OL461515	B.1.1.7	GRY	38	T201A,C241T,C913T,C1545T,C2110T,C3037T,C3267T,C5388A,C5986T,T6954C,G7042T,A10195G,C14120T,C14408T,C14676T,C15279T,T16176C,C22444T,A23063T,C23271A,A23403G,C23604A,C23709T,T24506G,G24914C,T25612G,C27972T,G28048T,G28075T,A28095T,A28111G,G28280C,A28281T,T28282A,G28881A,G28882A,G28883C,C28977T	24	N:D3L,N:R203K,N:G204R,N:S235F,ORF1a:A427V,ORF1a:T1001I,ORF1a:A1708D,ORF1a:I2230T,ORF1a:M2259I,ORF1b:P218L,ORF1b:P314L,ORF3a:S74A,ORF8:Q27*,ORF8:R52I,ORF8:C61F,ORF8:K68*,ORF8:Y73C,S:N501Y,S:A570D,S:D614G,S:P681H,S:T716I,S:S982A,S:D1118H	6	1
hCoV-19/Mexico/PUE-LANGEBIO_IMSS_1153/2021	EPI_ISL_2671652	OL461516	B.1.1.7	GRY	38	C241T,C913T,C2449T,C3037T,A3196T,C3267T,C5388A,C5944T,C5986T,C6402T,T6954C,C9166T,A10323G,C14408T,C14676T,T15096C,C15279T,T16176C,A23063T,C23271A,A23403G,C23604A,C23709T,T24506G,G24914C,C27972T,G28048T,A28095T,A28111G,G28280C,A28281T,T28282A,G28881A,G28882A,G28883C,G28884C,C28977T,T29710C	22	N:D3L,N:R203K,N:G204P,N:S235F,ORF1a:E977D,ORF1a:T1001I,ORF1a:A1708D,ORF1a:P2046L,ORF1a:I2230T,ORF1a:K3353R,ORF1b:P314L,ORF8:Q27*,ORF8:R52I,ORF8:K68*,ORF8:Y73C,S:N501Y,S:A570D,S:D614G,S:P681H,S:T716I,S:S982A,S:D1118H	6	0
hCoV-19/Mexico/PUE-LANGEBIO_IMSS_1387/2021	EPI_ISL_2942515	OL461517	B.1.1.7	GRY	37	C913T,A2317T,C2453T,C3037T,C3267T,C5388A,C5986T,C6433T,T6954C,C9611T,C11750T,C14408T,C14676T,C15279T,T16176C,C21008T,C21855T,G21974C,A23063T,C23271A,A23403G,C23604A,C23709T,T24506G,G24914C,C27643T,C27972T,G28048T,A28111G,G28280C,A28281T,T28282A,A28295G,G28881A,G28882A,G28883C,C28977T	27	N:D3L,N:N8D,N:R203K,N:G204R,N:S235F,ORF1a:K684N,ORF1a:L730F,ORF1a:T1001I,ORF1a:A1708D,ORF1a:I2230T,ORF1a:L3116F,ORF1a:L3829F,ORF1b:P314L,ORF1b:T2514I,ORF7a:P84S,ORF8:Q27*,ORF8:R52I,ORF8:Y73C,S:S98F,S:D138H,S:N501Y,S:A570D,S:D614G,S:P681H,S:T716I,S:S982A,S:D1118H	6	459
hCoV-19/Mexico/PUE-LANGEBIO_IMSS_1853/2021	EPI_ISL_2942920	OL461518	B.1.1.7	GRY	38	C913T,C2449T,C3037T,C3267T,C5388A,C5944T,C5986T,C6402T,G6669T,T6954C,C9166T,A10323G,A12594G,C14408T,C14676T,T15096C,C15279T,T16176C,A23063T,C23271A,A23403G,C23604A,C23709T,T24506G,G24914C,C27972T,G28048T,A28095T,A28111G,G28280C,A28281T,T28282A,G28881A,G28882A,G28883C,G28884C,C28977T,T29710C	23	N:D3L,N:R203K,N:G204P,N:S235F,ORF1a:T1001I,ORF1a:A1708D,ORF1a:P2046L,ORF1a:W2135L,ORF1a:I2230T,ORF1a:K3353R,ORF1a:Q4110R,ORF1b:P314L,ORF8:Q27*,ORF8:R52I,ORF8:K68*,ORF8:Y73C,S:N501Y,S:A570D,S:D614G,S:P681H,S:T716I,S:S982A,S:D1118H	6	553
hCoV-19/Mexico/QUE_LANGEBIO_IMSS_0516/2021	EPI_ISL_2401943	OL461519	B.1.1.7	GRY	37	C241T,C913T,C2110T,C3037T,C3267T,C5388A,C5986T,T6954C,A9274G,C9598T,C12525T,C13426T,C14120T,C14408T,C14676T,C15279T,T16176C,C21575T,A23063T,C23271A,A23403G,C23604A,C23709T,T24506G,G24914C,T27462C,C27972T,G28048T,A28095T,A28111G,G28280C,A28281T,T28282A,G28881A,G28882A,G28883C,C28977T	22	N:D3L,N:R203K,N:G204R,N:S235F,ORF1a:T1001I,ORF1a:A1708D,ORF1a:I2230T,ORF1a:T4087I,ORF1b:P218L,ORF1b:P314L,ORF8:Q27*,ORF8:R52I,ORF8:K68*,ORF8:Y73C,S:L5F,S:N501Y,S:A570D,S:D614G,S:P681H,S:T716I,S:S982A,S:D1118H	6	5
hCoV-19/Mexico/QUE_LANGEBIO_IMSS_0810/2021	EPI_ISL_2402220	OL461520	B.1.1.7	GRY	38	C241T,C913T,C1450T,C3037T,C3267T,C5388A,C5944T,C5986T,T6954C,C9967T,C13585T,C14408T,C14676T,T15096C,C15279T,T16176C,A17344G,G18412T,C22987T,A23063T,C23271A,A23403G,C23604A,C23709T,T24506G,G24914C,C27972T,G28048T,A28095T,A28111G,G28280C,A28281T,T28282A,G28881A,G28882A,G28883C,G28884C,C28977T	21	N:D3L,N:R203K,N:G204P,N:S235F,ORF1a:T1001I,ORF1a:A1708D,ORF1a:I2230T,ORF1b:P314L,ORF1b:I1293V,ORF1b:V1649F,ORF8:Q27*,ORF8:R52I,ORF8:K68*,ORF8:Y73C,S:N501Y,S:A570D,S:D614G,S:P681H,S:T716I,S:S982A,S:D1118H	6	23
hCoV-19/Mexico/QUE_LANGEBIO_IMSS_0811/2021	EPI_ISL_2402221	OL461521	B.1.1.7	GRY	37	C241T,C913T,C3037T,C3267T,C5388A,C5944T,C5986T,T6954C,C9967T,C13585T,C14408T,C14676T,T15096C,C15279T,T16176C,A17344G,G18412T,C22987T,A23063T,C23271A,A23403G,C23604A,C23709T,T24506G,G24914C,C27972T,G28048T,A28095T,A28111G,G28280C,A28281T,T28282A,G28881A,G28882A,G28883C,G28884C,C28977T	21	N:D3L,N:R203K,N:G204P,N:S235F,ORF1a:T1001I,ORF1a:A1708D,ORF1a:I2230T,ORF1b:P314L,ORF1b:I1293V,ORF1b:V1649F,ORF8:Q27*,ORF8:R52I,ORF8:K68*,ORF8:Y73C,S:N501Y,S:A570D,S:D614G,S:P681H,S:T716I,S:S982A,S:D1118H	6	331
hCoV-19/Mexico/QUE-IBT_IMSS_1993/2021	EPI_ISL_2801848	OL461522	B.1.1.7	GRY	41	C241T,C913T,C3037T,C3267T,C4540T,C5388A,C5944T,C5986T,T6954C,C8950T,C9967T,C13585T,C14408T,C14676T,T15096C,C15279T,T16176C,G18412T,C19374T,T22565C,C22987T,A23063T,C23271A,A23403G,C23604A,C23709T,T24506G,G24914C,G27033T,C27972T,G28048T,A28095T,A28111G,G28280C,A28281T,T28282A,G28881A,G28882A,G28883C,G28884C,C28977T	21	M:A171S,N:D3L,N:R203K,N:G204P,N:S235F,ORF1a:T1001I,ORF1a:A1708D,ORF1a:I2230T,ORF1b:P314L,ORF1b:V1649F,ORF8:Q27*,ORF8:R52I,ORF8:K68*,ORF8:Y73C,S:N501Y,S:A570D,S:D614G,S:P681H,S:T716I,S:S982A,S:D1118H	6	1
hCoV-19/Mexico/QUE-IBT_IMSS_1994/2021	EPI_ISL_2801849	OL461523	B.1.1.7	GRY	40	C241T,C913T,C3037T,C3267T,C4540T,C5388A,C5944T,C5986T,T6954C,C8950T,C9967T,C13585T,C14408T,C14676T,T15096C,C15279T,T16176C,G18412T,C19374T,T22565C,C22987T,A23063T,C23271A,A23403G,C23604A,C23709T,T24506G,G24914C,C27972T,G28048T,A28095T,A28111G,G28280C,A28281T,T28282A,G28881A,G28882A,G28883C,G28884C,C28977T	20	N:D3L,N:R203K,N:G204P,N:S235F,ORF1a:T1001I,ORF1a:A1708D,ORF1a:I2230T,ORF1b:P314L,ORF1b:V1649F,ORF8:Q27*,ORF8:R52I,ORF8:K68*,ORF8:Y73C,S:N501Y,S:A570D,S:D614G,S:P681H,S:T716I,S:S982A,S:D1118H	6	9
hCoV-19/Mexico/ROO_IBT_IMSS_1716/2021	EPI_ISL_2681136	OL461524	B.1.1.7	GRY	34	C241T,C913T,C2453T,C3037T,C3267T,C5388A,C5986T,T6954C,C9611T,C9893T,C14408T,C14676T,C15279T,T16176C,C21855T,G21974C,A23063T,C23271A,A23403G,C23604A,C23709T,T24506G,G24914C,C27972T,G28048T,A28111G,G28280C,A28281T,T28282A,A28295G,G28881A,G28882A,G28883C,C28977T	24	N:D3L,N:N8D,N:R203K,N:G204R,N:S235F,ORF1a:L730F,ORF1a:T1001I,ORF1a:A1708D,ORF1a:I2230T,ORF1a:L3116F,ORF1a:L3210F,ORF1b:P314L,ORF8:Q27*,ORF8:R52I,ORF8:Y73C,S:S98F,S:D138H,S:N501Y,S:A570D,S:D614G,S:P681H,S:T716I,S:S982A,S:D1118H	6	0
hCoV-19/Mexico/ROO_IBT_IMSS_1729/2021	EPI_ISL_2681233	OL461525	B.1.1.7	GRY	37	C241T,A686C,C913T,C3037T,C3267T,C5388A,C5986T,T6954C,A10195G,C10279T,C14120T,C14408T,C14676T,C15279T,T16176C,C16887T,G17427T,A23063T,C23271A,A23403G,C23604A,C23709T,T24506G,G24914C,C27972T,C28000T,G28048T,G28075T,A28095T,A28111G,G28280C,A28281T,T28282A,G28881A,G28882A,G28883C,C28977T	23	N:D3L,N:R203K,N:G204R,N:S235F,ORF1a:K141Q,ORF1a:T1001I,ORF1a:A1708D,ORF1a:I2230T,ORF1b:P218L,ORF1b:P314L,ORF8:Q27*,ORF8:P36L,ORF8:R52I,ORF8:C61F,ORF8:K68*,ORF8:Y73C,S:N501Y,S:A570D,S:D614G,S:P681H,S:T716I,S:S982A,S:D1118H	6	197
hCoV-19/Mexico/ROO_IBT_IMSS_1732/2021	EPI_ISL_2681050	OL461526	B.1.1.7	GRY	42	C241T,C913T,C2110T,C2453T,C3037T,C3267T,C5388A,C5986T,T6954C,G7042T,C9611T,C14120T,C14408T,C14676T,G14857T,C15279T,T16176C,C21855T,G21974C,T22219C,A23063T,C23271A,A23403G,C23604A,C23709T,T24506G,G24914C,C25609T,C25731T,G26506T,C27002T,C27972T,G28048T,A28111G,G28280C,A28281T,T28282A,A28804G,G28881A,G28882A,G28883C,C28	26	N:D3L,N:R203K,N:G204R,N:S235F,ORF1a:L730F,ORF1a:T1001I,ORF1a:A1708D,ORF1a:I2230T,ORF1a:M2259I,ORF1a:L3116F,ORF1b:P218L,ORF1b:P314L,ORF1b:V464F,ORF3a:L73F,ORF8:Q27*,ORF8:R52I,ORF8:Y73C,S:S98F,S:D138H,S:N501Y,S:A570D,S:D614G,S:P681H,S:T716I,S:S982A,S:D1118H	6	0
hCoV-19/Mexico/ROO_IBT_IMSS_1733/2021	EPI_ISL_2681133	OL461527	B.1.1	GR	38	C241T,C913T,C3037T,C3267T,G4207T,A6613G,T6954C,C9893T,C13860T,C14408T,C14676T,C14932T,C15279T,T16176C,G16959T,C18131T,T18429C,G21974T,G23012A,A23063T,C23271A,A23403G,C23525T,T24506G,T26149C,A28111G,G28280C,A28281T,T28282A,A28295G,C28512G,C28603T,G28881A,G28882A,G28883C,C28977T,T29834A,T29858A	21	N:D3L,N:N8D,N:P80R,N:R203K,N:G204R,N:S235F,ORF1a:T1001I,ORF1a:I2230T,ORF1a:L3210F,ORF1b:P314L,ORF1b:T1555I,ORF3a:S253P,ORF8:Y73C,ORF9b:Q77E,S:D138Y,S:E484K,S:N501Y,S:A570D,S:D614G,S:H655Y,S:S982A	3	0
hCoV-19/Mexico/ROO_IBT_IMSS_1745/2021	EPI_ISL_2681176	OL461528	B.1.1.7	GRY	39	C222T,C241T,C913T,C2110T,C3037T,C3267T,C4423T,C5388A,C5986T,C6681T,T6954C,G7042T,A10359G,C14120T,C14408T,C14676T,C15279T,T16176C,C17762T,A23063T,C23271A,A23403G,C23604A,G23608T,C23709T,T24506G,G24914C,T25180C,C27972T,G28048T,A28095T,A28111G,G28280C,A28281T,T28282A,G28881A,G28882A,G28883C,C28977T	24	N:D3L,N:R203K,N:G204R,N:S235F,ORF1a:T1001I,ORF1a:A1708D,ORF1a:A2139V,ORF1a:I2230T,ORF1a:M2259I,ORF1a:K3365R,ORF1b:P218L,ORF1b:P314L,ORF1b:A1432V,ORF8:Q27*,ORF8:R52I,ORF8:K68*,ORF8:Y73C,S:N501Y,S:A570D,S:D614G,S:P681H,S:T716I,S:S982A,S:D1118H	6	46
hCoV-19/Mexico/ROO_INER_IMSS_00420/2021	EPI_ISL_1585428	OL461529	B.1.1.7	GRY	36	G204T,C241T,C913T,C3037T,C3267T,C5388A,C5986T,T6954C,C9693T,C11572T,C14408T,C14676T,C15279T,T16176C,T23042C,A23063T,C23271A,A23403G,C23604A,C23709T,T24506G,G24914C,G25218T,C25553T,C27615T,C27972T,G28048T,A28095T,A28111G,G28280C,A28281T,T28282A,G28881A,G28882A,G28883C,C28977T	23	N:D3L,N:R203K,N:G204R,N:S235F,ORF1a:T1001I,ORF1a:A1708D,ORF1a:I2230T,ORF1a:A3143V,ORF1b:P314L,ORF3a:A54V,ORF8:Q27*,ORF8:R52I,ORF8:K68*,ORF8:Y73C,S:S494P,S:N501Y,S:A570D,S:D614G,S:P681H,S:T716I,S:S982A,S:D1118H,S:G1219V	6	0
hCoV-19/Mexico/ROO_INER_IMSS_00436/2021	EPI_ISL_1585444	OL461530	B.1.1.7	GRY	37	C241T,C913T,C2110T,C3037T,C3267T,C5388A,C5986T,T6954C,G7042T,A10195G,C10279T,C14120T,C14408T,C14676T,C15279T,T16176C,C16887T,G17427T,A23063T,C23271A,A23403G,C23604A,C23709T,T24506G,G24914C,C27972T,G28048T,G28075T,A28095T,A28111G,G28280C,A28281T,T28282A,G28881A,G28882A,G28883C,C28977T	22	N:D3L,N:R203K,N:G204R,N:S235F,ORF1a:T1001I,ORF1a:A1708D,ORF1a:I2230T,ORF1a:M2259I,ORF1b:P218L,ORF1b:P314L,ORF8:Q27*,ORF8:R52I,ORF8:C61F,ORF8:K68*,ORF8:Y73C,S:N501Y,S:A570D,S:D614G,S:P681H,S:T716I,S:S982A,S:D1118H	6	0
hCoV-19/Mexico/ROO_INER_IMSS_00771/2021	EPI_ISL_2091225	OL461531	B.1.1.7	GRY	33	C241T,C913T,C2453T,C3037T,C3267T,C5388A,C5986T,T6954C,C9611T,C14408T,C14676T,C15279T,T16176C,C21855T,G21974C,A23063T,C23271A,A23403G,C23604A,C23709T,T24506G,G24914C,C27972T,G28048T,A28111G,G28280C,A28281T,T28282A,A28295G,G28881A,G28882A,G	23	N:D3L,N:N8D,N:R203K,N:G204R,N:S235F,ORF1a:L730F,ORF1a:T1001I,ORF1a:A1708D,ORF1a:I2230T,ORF1a:L3116F,ORF1b:P314L,ORF8:Q27*,ORF8:R52I,ORF8:Y73C,S:S98F,S:D138H,S:N501Y,S:A570D,S:D614G,S:P681H,S:T716I,S:S982	6	0

hCoV-19/Mexico/ROO_INER_IMSS_00790/2021	EPI_ISL_2091243	OL461532	B.1.1.7	GRY	33	C241T,C913T,C2453T,C3037T,C3267T,C5388A,C5986T,T6954C,C9611T,C14408T,C14676T,C15279T,T16176C,C21855T,G21974C,A23063T,C23271A,A23403G,C23604A,C23709T,T24506G,G24914C,C27972T,G28048T,A28111G,G28280C,A28281T,T28282A,A28295G,G28881A,G28882A,G	23	N:D3L,N:N8D,N:R203K,N:G204R,N:S235F,ORF1a:L730F,ORF1a:T1001I,ORF1a:A1708D,ORF1a:I2230T,ORF1a:L3116F,ORF1b:P314L,ORF8:Q27*,ORF8:R52I,ORF8:Y73C,S:S98F,S:D138H,S:N501Y,S:A570D,S:D614G,S:P681H,S:T716I,S:S982	6	0
hCoV-19/Mexico/ROO_INER_IMSS_00791/2021	EPI_ISL_2091244	OL461533	B.1.1.7	GRY	38	C241T,C913T,C2110T,G3004T,C3037T,C3267T,C5388A,C5986T,T6954C,G7042T,C10789T,C14120T,C14408T,C14676T,C15279T,T16176C,G17019T,A23063T,C23271A,A23403G,C23604A,C23709T,T24506G,G24914C,C26110T,C26735T,C27972T,G28048T,A28095T,A28111G,G28280C,A28281T,T28282A,A28804G,G28881A,G28882A,G28883C,C28977T	24	N:D3L,N:R203K,N:G204R,N:S235F,ORF1a:E913D,ORF1a:T1001I,ORF1a:A1708D,ORF1a:I2230T,ORF1a:M2259I,ORF1b:P218L,ORF1b:P314L,ORF1b:E1184D,ORF3a:P240S,ORF8:Q27*,ORF8:R52I,ORF8:K68*,ORF8:Y73C,S:N501Y,S:A570D,S:D614G,S:P681H,S:T716I,S:S982A,S:D1118H	6	0
hCoV-19/Mexico/ROO_INER_IMSS_00792/2021	EPI_ISL_2091245	OL461534	B.1.1.7	GRY	35	C222T,C241T,C913T,C2110T,C3037T,C3267T,C5388A,C5986T,T6954C,G7042T,C14120T,C14408T,C14676T,C15279T,T16176C,C17762T,A23063T,C23271A,A23403G,C23604A,C23709T,T24506G,G24914C,T25180C,C27972T,G28048T,A28095T,A28111G,G28280C,A28281T,T28282A,G28881A,G28882A,G28883C,C28977T	22	N:D3L,N:R203K,N:G204R,N:S235F,ORF1a:T1001I,ORF1a:A1708D,ORF1a:I2230T,ORF1a:M2259I,ORF1b:P218L,ORF1b:P314L,ORF1b:A1432V,ORF8:Q27*,ORF8:R52I,ORF8:K68*,ORF8:Y73C,S:N501Y,S:A570D,S:D614G,S:P681H,S:T716I,S:S982A,S:D1118H	6	0
hCoV-19/Mexico/ROO_INER_IMSS_1130/2021	EPI_ISL_2490311	OL461535	B.1.1.7	GRY	38	C241T,C466T,C913T,A2270G,C3037T,C3267T,C5388A,C5884T,C5986T,T6954C,C14408T,G14635A,C14676T,C15279T,T16176C,A21027G,C21219T,G22361T,A23063T,C23271A,A23403G,C23604A,C23709T,T24506G,G24914C,G25996A,C26305T,C27879T,C27972T,G28048T,A28111G,G28280C,A28281T,T28282A,G28881A,G28882A,G28883C,C28977T	24	E:L21F,N:D3L,N:R203K,N:G204R,N:S235F,ORF1a:K669E,ORF1a:T1001I,ORF1a:A1708D,ORF1a:I2230T,ORF1b:P314L,ORF1b:A390T,ORF3a:V202I,ORF7b:H42Y,ORF8:Q27*,ORF8:R52I,ORF8:Y73C,S:V267L,S:N501Y,S:A570D,S:D614G,S:P681H,S:T716I,S:S982A,S:D1118H	7	0
hCoV-19/Mexico/ROO_INER_IMSS_1152/2021	EPI_ISL_2490330	OL461536	B.1.1.7	GRY	34	C241T,C913T,C2453T,C3037T,C3267T,C5388A,C5986T,T6954C,C9611T,C14408T,C14676T,C15279T,T16176C,C21855T,G21974C,A23063T,C23271A,A23403G,C23604A,C23709T,T24506G,G24914C,C25886T,C27972T,G28048T,A28111G,G28280C,A28281T,T28282A,A28295G,G28881A,G28882A,G28883C,C28977T	24	N:D3L,N:N8D,N:R203K,N:G204R,N:S235F,ORF1a:L730F,ORF1a:T1001I,ORF1a:A1708D,ORF1a:I2230T,ORF1a:L3116F,ORF1b:P314L,ORF3a:S165F,ORF8:Q27*,ORF8:R52I,ORF8:Y73C,S:S98F,S:D138H,S:N501Y,S:A570D,S:D614G,S:P681H,S:T716I,S:S982A,S:D1118H	6	1
hCoV-19/Mexico/ROO_INER_IMSS_1162/2021	EPI_ISL_2490347	OL461537	B.1.1.7	GRY	36	C222T,C241T,C913T,C2110T,C3037T,C3267T,C5388A,C5986T,T6954C,G7042T,C14120T,C14408T,C14676T,C15279T,T16176C,C17762T,C18452T,A23063T,C23271A,A23403G,C23604A,C23709T,T24506G,G24914C,T25180C,C27972T,G28048T,A28095T,A28111G,G28280C,A28281T,T28282A,G28881A,G28882A,G28883C,C28977T	23	N:D3L,N:R203K,N:G204R,N:S235F,ORF1a:T1001I,ORF1a:A1708D,ORF1a:I2230T,ORF1a:M2259I,ORF1b:P218L,ORF1b:P314L,ORF1b:A1432V,ORF1b:A1662V,ORF8:Q27*,ORF8:R52I,ORF8:K68*,ORF8:Y73C,S:N501Y,S:A570D,S:D614G,S:P681H,S:T716I,S:S982A,S:D1118H	6	0
hCoV-19/Mexico/ROO_LANGEBIO_IMSS_07605-NC/2021	EPI_ISL_2970025	OL461538	B.1.1.7	GRY	31	C913T,C3037T,C3267T,C5388A,C5986T,T6954C,C14120T,C14408T,C14676T,C15279T,T16176C,C17762T,A23063T,C23271A,A23403G,C23604A,C23709T,T24506G,G24914C,T25180C,C27972T,G28048T,A28095T,A28111G,G28280C,A28281T,T28282A,G28881A,G28882A,G28883C,C2897	21	N:D3L,N:R203K,N:G204R,N:S235F,ORF1a:T1001I,ORF1a:A1708D,ORF1a:I2230T,ORF1b:P218L,ORF1b:P314L,ORF1b:A1432V,ORF8:Q27*,ORF8:R52I,ORF8:K68*,ORF8:Y73C,S:N501Y,S:A570D,S:D614G,S:P681H,S:T716I,S:S982A,S:D1118H	6	1078
hCoV-19/Mexico/ROO-IBT_IMSS_1756/2021	EPI_ISL_2801655	OL461539	B.1.1.7	GRY	39	C241T,C913T,G2281A,C3037T,C3267T,C5388A,C5986T,T6954C,A12162G,C14408T,C14676T,C15279T,C15720T,T16176C,A17615G,C18747T,C20844T,A21137G,A23063T,C23271A,A23403G,C23604A,C23709T,T24506G,G24914C,G25135T,C26110T,G26828T,C27972T,G28048T,A28111G,G28280C,A28281T,T28282A,G28881A,G28882A,G28883C,C28977T,C29095T	23	N:D3L,N:R203K,N:G204R,N:S235F,ORF1a:T1001I,ORF1a:A1708D,ORF1a:I2230T,ORF1a:Q3966R,ORF1b:P314L,ORF1b:K1383R,ORF1b:K2557R,ORF3a:P240S,ORF8:Q27*,ORF8:R52I,ORF8:Y73C,S:N501Y,S:A570D,S:D614G,S:P681H,S:T716I,S:S982A,S:D1118H,S:K1191N	6	0
hCoV-19/Mexico/ROO-IBT_IMSS_1812/2021	EPI_ISL_2801577	OL461540	B.1.1.7	GRY	38	C222T,C241T,C913T,C3037T,C3267T,C4423T,C5388A,C5986T,C6681T,T6954C,G7042T,A10359G,C14120T,C14408T,C14676T,C15279T,T16176C,C17762T,A23063T,C23271A,A23403G,C23604A,G23608T,C23709T,T24506G,G24914C,T25180C,C27972T,G28048T,A28095T,A28111G,G28280C,A28281T,T28282A,G28881A,G28882A,G28883C,C28977T	24	N:D3L,N:R203K,N:G204R,N:S235F,ORF1a:T1001I,ORF1a:A1708D,ORF1a:A2139V,ORF1a:I2230T,ORF1a:M2259I,ORF1a:K3365R,ORF1b:P218L,ORF1b:P314L,ORF1b:A1432V,ORF8:Q27*,ORF8:R52I,ORF8:K68*,ORF8:Y73C,S:N501Y,S:A570D,S:D614G,S:P681H,S:T716I,S:S982A,S:D1118H	6	606
hCoV-19/Mexico/ROO-IBT_IMSS_1825/2021	EPI_ISL_2801579	OL461541	B.1.1.7	GRY	40	C222T,C241T,C913T,C3037T,C3267T,C4423T,C5388A,C5986T,C6681T,T6954C,G7042T,A10359G,C14120T,C14408T,C14676T,C15279T,T16176C,G16207T,C17762T,G18301A,A23063T,C23271A,A23403G,C23604A,G23608T,C23709T,T24506G,G24914C,T25180C,C27972T,G28048T,A28095T,A28111G,G28280C,A28281T,T28282A,G28881A,G28882A,G28883C,C28977T	26	N:D3L,N:R203K,N:G204R,N:S235F,ORF1a:T1001I,ORF1a:A1708D,ORF1a:A2139V,ORF1a:I2230T,ORF1a:M2259I,ORF1a:K3365R,ORF1b:P218L,ORF1b:P314L,ORF1b:A914S,ORF1b:A1432V,ORF1b:G1612S,ORF8:Q27*,ORF8:R52I,ORF8:K68*,ORF8:Y73C,S:N501Y,S:A570D,S:D614G,S:P681H,S:T716I,S:S982A,S:D1118H	6	188
hCoV-19/Mexico/ROO-IBT_IMSS_1947/2021	EPI_ISL_2801592	OL461542	B.1.1.7	GRY	41	C241T,C913T,C1513T,C3037T,C3267T,C3583T,C5388A,C5944T,C5986T,T6954C,G11138T,G11401T,C12469T,C14408T,C14676T,T15096C,C15279T,T16176C,C17491T,C19862T,A23063T,C23271A,A23403G,C23506T,C23604A,C23709T,T24506G,G24914C,C27972T,G28048T,A28095T,A28111G,G28280C,A28281T,T28282A,G28881A,G28882A,G28883C,G28884C,C28977T,C29546A	23	N:D3L,N:R203K,N:G204P,N:S235F,ORF1a:T1001I,ORF1a:A1708D,ORF1a:I2230T,ORF1a:A3625S,ORF1a:M3712I,ORF1b:P314L,ORF1b:P1342S,ORF1b:A2132V,ORF8:Q27*,ORF8:R52I,ORF8:K68*,ORF8:Y73C,S:N501Y,S:A570D,S:D614G,S:P681H,S:T716I,S:S982A,S:D1118H	6	0
hCoV-19/Mexico/ROO-IBT_IMSS_1969/2021	EPI_ISL_2801594	OL461543	Q.3	GRY	41	C241T,C913T,C1150T,C3037T,C3267T,G5023A,C5388A,C5986T,C6568T,T6954C,G8183A,A9529G,G10396T,C14391T,C14408T,C14676T,C15279T,T16176C,G16336A,A17615G,G20962A,T21147C,G22094C,A23063T,C23271A,A23403G,C23604A,C23709T,T24506G,G24914C,G26730C,C27972T,G28048T,A28111G,G28280C,A28281T,T28282A,G28881A,G28882A,G28883C,C28977T	25	M:V70L,N:D3L,N:R203K,N:G204R,N:S235F,ORF1a:T1001I,ORF1a:M1586I,ORF1a:A1708D,ORF1a:I2230T,ORF1a:G2640R,ORF1b:P314L,ORF1b:V957I,ORF1b:K1383R,ORF1b:D2499N,ORF8:Q27*,ORF8:R52I,ORF8:Y73C,S:D178H,S:N501Y,S:A570D,S:D614G,S:P681H,S:T716I,S:S982A,S:D1118H	6	669
hCoV-19/Mexico/ROO-IBT_IMSS_2068/2021	EPI_ISL_2801619	OL461544	B.1.1.7	GRY	42	C241T,C913T,C2110T,C2143T,C3037T,G3047T,C3267T,C3429T,C5388A,C5986T,C6730T,C7945T,A10195G,C10834T,C14120T,C14408T,C14676T,C15279T,T16176C,T20445C,A23063T,C23271A,A23403G,C23604A,C23709T,T24506G,G24914C,C27972T,G28048T,G28075T,A28095T,A28111G,C28236T,G28280C,A28281T,T28282A,G28881A,G28882A,G28883C,C28896G,C28977T,C296	24	N:D3L,N:R203K,N:G204R,N:A208G,N:S235F,ORF1a:D928Y,ORF1a:T1001I,ORF1a:T1055I,ORF1a:A1708D,ORF1b:P218L,ORF1b:P314L,ORF8:Q27*,ORF8:R52I,ORF8:C61F,ORF8:K68*,ORF8:Y73C,ORF8:R115C,S:N501Y,S:A570D,S:D614G,S:P681H,S:T716I,S:S982A,S:D1118H	6	64
hCoV-19/Mexico/ROO-IBT_IMSS_2093/2021	EPI_ISL_2801641	OL461545	B.1.1.7	GRY	40	C241T,C913T,C1441T,C2110T,C3037T,C3267T,C5388A,C5986T,C6690T,T6954C,G7042T,C8748T,A10159C,C14120T,C14408T,C14676T,C15279T,T16176C,C21597G,A22023G,A23044G,A23063T,C23271A,A23403G,C23604A,C23709T,G24415T,G24914C,C27972T,G28048T,A28095T,A28111G,G28280C,A28281T,T28282A,G28881A,G28882A,G28883C,C28977T,C29743T	24	N:D3L,N:R203K,N:G204R,N:S235F,ORF1a:T1001I,ORF1a:A1708D,ORF1a:A2142V,ORF1a:I2230T,ORF1a:M2259I,ORF1a:A2828V,ORF1b:P218L,ORF1b:P314L,ORF8:Q27*,ORF8:R52I,ORF8:K68*,ORF8:Y73C,S:S12C,S:E154G,S:N501Y,S:A570D,S:D614G,S:P681H,S:T716I,S:D1118H	6	173
hCoV-19/Mexico/ROO-IBT_IMSS_2106/2021	EPI_ISL_2801653	OL461546	B.1.1.7	GRY	39	C241T,T597C,C913T,C1471T,G2144A,C2453T,C3037T,C3267T,C5388A,C5986T,T6954C,C9286T,C9611T,A12547G,C14408T,C14676T,C15279T,T16176C,G18651T,C21855T,G21974C,A23063T,C23271A,A23403G,C23604A,C23709T,T24506G,G24914C,C27972T,G28048T,A28111G,G28280C,A28281T,T28282A,A28295G,G28881A,G28882A,G28883C,C28977T	26	N:D3L,N:N8D,N:R203K,N:G204R,N:S235F,ORF1a:V111A,ORF1a:V627I,ORF1a:L730F,ORF1a:T1001I,ORF1a:A1708D,ORF1a:I2230T,ORF1a:L3116F,ORF1b:P314L,ORF1b:E1728D,ORF8:Q27*,ORF8:R52I,ORF8:Y73C,S:S98F,S:D138H,S:N501Y,S:A570D,S:D614G,S:P681H,S:T716I,S:S982A,S:D1118H	6	0
hCoV-19/Mexico/ROO-LANGEBIO_IMSS_0900/2021	EPI_ISL_2671655	OL461547	B.1.1.7	GRY	36	C241T,C913T,C2453T,C3037T,C3267T,C5388A,C5986T,T6954C,C9611T,C14408T,C14676T,C15279T,A15860G,T16176C,C19488T,G20002T,C21855T,G21974C,A23063T,C23271A,A23403G,C23604A,C23709T,T24506G,G24914C,C27972T,G28048T,A28111G,G28280C,A28281T,T28282A,A28295G,G28881A,G28882A,G28883C,C28977T	25	N:D3L,N:N8D,N:R203K,N:G204R,N:S235F,ORF1a:L730F,ORF1a:T1001I,ORF1a:A1708D,ORF1a:I2230T,ORF1a:L3116F,ORF1b:P314L,ORF1b:K798R,ORF1b:D2179Y,ORF8:Q27*,ORF8:R52I,ORF8:Y73C,S:S98F,S:D138H,S:N501Y,S:A570D,S:D614G,S:P681H,S:T716I,S:S982A,S:D1118H	6	1
hCoV-19/Mexico/ROO-LANGEBIO_IMSS_0907/2021	EPI_ISL_2671660	OL461548	B.1.1.7	GRY	36	C222T,C241T,C913T,C2110T,C3037T,C3267T,C5388A,C5986T,T6954C,G7042T,C14120T,C14408T,C14676T,C15279T,T16176C,C17762T,A23063T,C23271A,A23403G,C23604A,C23709T,T24506G,G24914C,T25180C,G25641T,C27972T,G28048T,A28095T,A28111G,G28280C,A28281T,T28282A,G28881A,G28882A,G28883C,C28977T	23	N:D3L,N:R203K,N:G204R,N:S235F,ORF1a:T1001I,ORF1a:A1708D,ORF1a:I2230T,ORF1a:M2259I,ORF1b:P218L,ORF1b:P314L,ORF1b:A1432V,ORF3a:L83F,ORF8:Q27*,ORF8:R52I,ORF8:K68*,ORF8:Y73C,S:N501Y,S:A570D,S:D614G,S:P681H,S:T716I,S:S982A,S:D1118H	6	0
hCoV-19/Mexico/ROO-LANGEBIO_IMSS_0926/2021	EPI_ISL_2671671	OL461549	B.1.1.7	GRY	36	C241T,C913T,C2453T,C3037T,C3267T,A4452G,C5388A,C5986T,T6954C,C9611T,C14408T,C14676T,C15279T,T16176C,G21451A,C21855T,G21974C,A23063T,C23271A,A23403G,C23604A,C23709T,T24506G,G24914C,C25886T,C27972T,G28048T,A28111G,G28280C,A28281T,T28282A,A28295G,G28881A,G28882A,G28883C,C28977T	26	N:D3L,N:N8D,N:R203K,N:G204R,N:S235F,ORF1a:L730F,ORF1a:T1001I,ORF1a:K1396R,ORF1a:A1708D,ORF1a:I2230T,ORF1a:L3116F,ORF1b:P314L,ORF1b:G2662S,ORF3a:S165F,ORF8:Q27*,ORF8:R52I,ORF8:Y73C,S:S98F,S:D138H,S:N501Y,S:A570D,S:D614G,S:P681H,S:T716I,S:S982A,S:D1118H	6	2
hCoV-19/Mexico/ROO-LANGEBIO_IMSS_0928/2021	EPI_ISL_2671673	OL461550	B.1.1.7	GRY	35	C241T,C913T,C2453T,C3037T,C3267T,C3796T,C5388A,C5986T,T6954C,C9611T,C14408T,C14676T,C15279T,T16176C,C21855T,G21974C,A23063T,C23271A,A23403G,C23604A,C23709T,T24506G,G24914C,C25886T,C27972T,G28048T,A28111G,G28280C,A28281T,T28282A,A28295G,G28881A,G28882A,G28883C,C28977T	24	N:D3L,N:N8D,N:R203K,N:G204R,N:S235F,ORF1a:L730F,ORF1a:T1001I,ORF1a:A1708D,ORF1a:I2230T,ORF1a:L3116F,ORF1b:P314L,ORF3a:S165F,ORF8:Q27*,ORF8:R52I,ORF8:Y73C,S:S98F,S:D138H,S:N501Y,S:A570D,S:D614G,S:P681H,S:T716I,S:S982A,S:D1118H	6	4

hCoV-19/Mexico/ROO-LANGEBIO_IMSS_0937/2021	EPI_ISL_2671680	OL461551	B.1.1.7	GRY	34	C241T,C913T,C2453T,C3037T,C3267T,C5388A,C5986T,T6954C,C9611T,C9893T,C14408T,C14676T,C15279T,T16176C,C21855T,G21974C,A23063T,C23271A,A23403G,C23604A,C23709T,T24506G,G24914C,C27972T,G28048T,A28111G,G28280C,A28281T,T28282A,A28295G,G28881A,G28882A,G28883C,C28977T	24	N:D3L,N:N8D,N:R203K,N:G204R,N:S235F,ORF1a:L730F,ORF1a:T1001I,ORF1a:A1708D,ORF1a:I2230T,ORF1a:L3116F,ORF1a:L3210F,ORF1b:P314L,ORF8:Q27*,ORF8:R52I,ORF8:Y73C,S:S98F,S:D138H,S:N501Y,S:A570D,S:D614G,S:P681H,S:T716I,S:S982A,S:D1118H	6	8
hCoV-19/Mexico/ROO-LANGEBIO_IMSS_0939/2021	EPI_ISL_2671682	OL461552	B.1.1.7	GRY	36	C913T,C2110T,C3037T,C3267T,C4423T,C5388A,C5986T,C6681T,T6954C,A10359G,C14120T,C14408T,C14676T,C15279T,T16176C,C17762T,A23063T,C23271A,A23403G,C23604A,G23608T,C23709T,T24506G,G24914C,T25180C,C27972T,G28048T,A28095T,A28111G,G28280C,A28281T,T28282A,G28881A,G28882A,G28883C,C28977T	23	N:D3L,N:R203K,N:G204R,N:S235F,ORF1a:T1001I,ORF1a:A1708D,ORF1a:A2139V,ORF1a:I2230T,ORF1a:K3365R,ORF1b:P218L,ORF1b:P314L,ORF1b:A1432V,ORF8:Q27*,ORF8:R52I,ORF8:K68*,ORF8:Y73C,S:N501Y,S:A570D,S:D614G,S:P681H,S:T716I,S:S982A,S:D1118H	6	422
hCoV-19/Mexico/ROO-LANGEBIO_IMSS_0960/2021	EPI_ISL_2671690	OL461553	B.1.1.7	GRY	40	C241T,C913T,G2281A,C3037T,C3267T,C5388A,C5986T,T6954C,A12162G,C14408T,C14676T,C15279T,C15720T,T16176C,A17615G,C18747T,C20844T,A21137G,A23063T,C23271A,A23403G,C23604A,C23709T,T24506G,G24914C,G25135T,C26110T,C26521T,G26828T,C27972T,G28048T,A28111G,G28280C,A28281T,T28282A,G28881A,G28882A,G28883C,C28977T,C29095T	23	N:D3L,N:R203K,N:G204R,N:S235F,ORF1a:T1001I,ORF1a:A1708D,ORF1a:I2230T,ORF1a:Q3966R,ORF1b:P314L,ORF1b:K1383R,ORF1b:K2557R,ORF3a:P240S,ORF8:Q27*,ORF8:R52I,ORF8:Y73C,S:N501Y,S:A570D,S:D614G,S:P681H,S:T716I,S:S982A,S:D1118H,S:K1191N	6	2
hCoV-19/Mexico/ROO-LANGEBIO_IMSS_0972/2021	EPI_ISL_2671696	OL461554	B.1.1.7	GRY	42	C241T,C913T,C2110T,C2143T,C3037T,G3047T,C3267T,C5388A,C5986T,C6730T,T6954C,C7945T,A10195G,C10834T,C14120T,C14408T,C14676T,C15279T,T16176C,T20445C,A23063T,C23271A,A23403G,C23604A,C23709T,T24506G,G24914C,C27972T,G28048T,G28075T,A28095T,A28111G,C28236T,G28280C,A28281T,T28282A,G28881A,G28882A,G28883C,C28896G,C28977T,C296	24	N:D3L,N:R203K,N:G204R,N:A208G,N:S235F,ORF1a:D928Y,ORF1a:T1001I,ORF1a:A1708D,ORF1a:I2230T,ORF1b:P218L,ORF1b:P314L,ORF8:Q27*,ORF8:R52I,ORF8:C61F,ORF8:K68*,ORF8:Y73C,ORF8:R115C,S:N501Y,S:A570D,S:D614G,S:P681H,S:T716I,S:S982A,S:D1118H	6	1
hCoV-19/Mexico/ROO-LANGEBIO_IMSS_1237/2021	EPI_ISL_2942398	OL461555	B.1.1.7	GRY	39	C241T,C841T,C913T,C3037T,C3267T,A3946G,C5388A,C5986T,T6954C,C8320T,C11758T,C12068T,C12400T,C14408T,C14676T,C15279T,T16176C,T20733C,C21812T,A23063T,C23271A,A23403G,C23604A,C23709T,T24506G,G24914C,C25511T,A27107G,C27972T,G28048T,A28111G,G28280C,A28281T,T28282A,G28881A,G28882A,G28883C,C28977T,C29732T	19	N:D3L,N:R203K,N:G204R,N:S235F,ORF1a:T1001I,ORF1a:A1708D,ORF1a:I2230T,ORF1b:P314L,ORF3a:S40L,ORF8:Q27*,ORF8:R52I,ORF8:Y73C,S:N501Y,S:A570D,S:D614G,S:P681H,S:T716I,S:S982A,S:D1118H	6	0
hCoV-19/Mexico/ROO-LANGEBIO_IMSS_1545/2021	EPI_ISL_2942656	OL461556	B.1.1.7	GRY	39	C241T,C913T,C2110T,C3037T,C3267T,C5388A,C5986T,T6954C,G7042T,C7788T,A10195G,C10279T,C14120T,C14408T,C14676T,C15279T,T16176C,C16887T,G17427T,A23063T,C23271A,A23403G,C23604A,C23709T,T24506G,G24914C,A27943G,C27972T,G28048T,G28075T,A28095T,A28111G,G28280C,A28281T,T28282A,G28881A,G28882A,G28883C,C28977T	24	N:D3L,N:R203K,N:G204R,N:S235F,ORF1a:T1001I,ORF1a:A1708D,ORF1a:I2230T,ORF1a:M2259I,ORF1a:A2508V,ORF1b:P218L,ORF1b:P314L,ORF8:H17R,ORF8:Q27*,ORF8:R52I,ORF8:C61F,ORF8:K68*,ORF8:Y73C,S:N501Y,S:A570D,S:D614G,S:P681H,S:T716I,S:S982A,S:D1118H	6	0
hCoV-19/Mexico/ROO-LANGEBIO_IMSS_1578/2021	EPI_ISL_2942689	OL461557	B.1.1.7	GRY	37	C241T,C913T,C3037T,C3040T,C3267T,T3537C,C5388A,C5986T,C6813T,T6954C,C9298T,T9508C,C14408T,C14676T,C15279T,T16176C,A23063T,C23271A,A23403G,C23604A,C23709T,C23758T,C24374T,T24506G,G24914C,C27972T,G28048T,A28111G,G28280C,A28281T,T28282A,G28881A,G28882A,G28883C,C28977T,C29555T,C29762T	21	N:D3L,N:R203K,N:G204R,N:S235F,ORF1a:T1001I,ORF1a:I1091T,ORF1a:A1708D,ORF1a:T2183I,ORF1a:I2230T,ORF1b:P314L,ORF8:Q27*,ORF8:R52I,ORF8:Y73C,S:N501Y,S:A570D,S:D614G,S:P681H,S:T716I,S:L938F,S:S982A,S:D1118H	6	0
hCoV-19/Mexico/ROO-LANGEBIO_IMSS_1590/2021	EPI_ISL_2942701	OL461558	B.1.1.7	GRY	36	C241T,C913T,C2453T,C3037T,C3267T,C5388A,C5986T,T6954C,C9611T,C9893T,C10582T,C14408T,C14676T,C15279T,T16176C,C21855T,G21974C,A23063T,C23271A,A23403G,C23604A,C23709T,T24506G,G24914C,C25350T,C27972T,G28048T,A28111G,G28280C,A28281T,T28282A,A28295G,G28881A,G28882A,G28883C,C28977T	25	N:D3L,N:N8D,N:R203K,N:G204R,N:S235F,ORF1a:L730F,ORF1a:T1001I,ORF1a:A1708D,ORF1a:I2230T,ORF1a:L3116F,ORF1a:L3210F,ORF1b:P314L,ORF8:Q27*,ORF8:R52I,ORF8:Y73C,S:S98F,S:D138H,S:N501Y,S:A570D,S:D614G,S:P681H,S:T716I,S:S982A,S:D1118H,S:P1263L	6	0
hCoV-19/Mexico/ROO-LANGEBIO_IMSS_1595/2021	EPI_ISL_2942704	OL461559	B.1.1.7	GRY	36	C241T,C823T,C913T,C1758T,C2110T,G3004T,C3037T,C3267T,C5388A,C5986T,T6954C,G7042T,C9559T,C14120T,C14408T,C14676T,C15279T,T16176C,A23063T,C23271A,A23403G,C23604A,C23709T,T24506G,G24914C,C26110T,C27972T,G28048T,A28111G,G28280C,A28281T,T28282A,G28881A,G28882A,G28883C,C28977T	23	N:D3L,N:R203K,N:G204R,N:S235F,ORF1a:A498V,ORF1a:E913D,ORF1a:T1001I,ORF1a:A1708D,ORF1a:I2230T,ORF1a:M2259I,ORF1b:P218L,ORF1b:P314L,ORF3a:P240S,ORF8:Q27*,ORF8:R52I,ORF8:Y73C,S:N501Y,S:A570D,S:D614G,S:P681H,S:T716I,S:S982A,S:D1118H	6	0
hCoV-19/Mexico/SIN_CIAD-CLN_D0035/2021	EPI_ISL_2680920	OL461560	B.1.1.7	GR	33	C241T,C292T,C913T,C3037T,C3267T,A3492G,C5388A,C5986T,T6954C,C14408T,C14676T,C15279T,T16176C,C21639A,A23063T,C23271A,A23403G,C23604A,C23709T,C24374T,T24506G,G24914C,A25164T,C27972T,G28048T,A28111G,G28280C,A28281T,T28282A,G28881A,G28882A,G28	22	N:D3L,N:R203K,N:G204R,N:S235F,ORF1a:T1001I,ORF1a:N1076S,ORF1a:A1708D,ORF1a:I2230T,ORF1b:P314L,ORF8:Q27*,ORF8:R52I,ORF8:Y73C,S:P26H,S:N501Y,S:A570D,S:D614G,S:P681H,S:T716I,S:L938F,S:S982A,S:D1118H,S:Q1201	3	33
hCoV-19/Mexico/SIN_CIAD-CLN_D0511/2021	EPI_ISL_2927967	OL461561	B.1.1.7	GRY	35	T2C,C241T,C379T,C913T,G1306T,C3037T,C3267T,A3492G,C5388A,C5986T,G6901A,T6954C,C14408T,C14676T,C15279T,T16176C,C19263T,C21639A,G22801T,A23063T,C23271A,A23403G,C23604A,C23709T,C24374T,T24506G,G24914C,A25998G,C27972T,G28048T,A28111G,G28881A,G	21	N:R203K,N:G204R,N:S235F,ORF1a:E347D,ORF1a:T1001I,ORF1a:N1076S,ORF1a:A1708D,ORF1a:I2230T,ORF1b:P314L,ORF8:Q27*,ORF8:R52I,ORF8:Y73C,S:P26H,S:N501Y,S:A570D,S:D614G,S:P681H,S:T716I,S:L938F,S:S982A,S:D1118H	6	27
hCoV-19/Mexico/SIN_CIAD-CLN_D0557/2021	EPI_ISL_2927968	OL461562	B.1.1.7	GRY	38	C241T,C913T,C2453T,C3037T,C3267T,T5152C,C5388A,C5986T,T6954C,C9611T,C14408T,C14676T,C15279T,T16176C,C21855T,G21974C,A23063T,C23271A,A23403G,C23604A,C23709T,T24506G,G24914C,G25273T,T25711C,C26882T,C27972T,G27996A,G28048T,A28111G,G28280C,A28281T,T28282A,A28295G,G28881A,G28882A,G28883C,C28977T	26	N:D3L,N:N8D,N:R203K,N:G204R,N:S235F,ORF1a:L730F,ORF1a:T1001I,ORF1a:A1708D,ORF1a:I2230T,ORF1a:L3116F,ORF1b:P314L,ORF3a:Y107H,ORF8:Q27*,ORF8:D35N,ORF8:R52I,ORF8:Y73C,S:S98F,S:D138H,S:N501Y,S:A570D,S:D614G,S:P681H,S:T716I,S:S982A,S:D1118H,S:M1237I	6	46
hCoV-19/Mexico/SIN_CIAD-CLN_D1476/2021	EPI_ISL_2680923	OL580734	B.1.1.7	GR	33	C241T,C913T,C3037T,C3267T,A3492G,C5388A,C5986T,T6954C,G8017T,C11750T,C14408T,C14676T,C15279T,T16176C,C21639A,A23063T,C23271A,A23403G,C23604A,C23709T,C24374T,T24506G,G24914C,C27972T,G28048T,A28111G,G28280C,A28281T,T28282A,G28881A,G28882A,G2	22	N:D3L,N:R203K,N:G204R,N:S235F,ORF1a:T1001I,ORF1a:N1076S,ORF1a:A1708D,ORF1a:I2230T,ORF1a:L3829F,ORF1b:P314L,ORF8:Q27*,ORF8:R52I,ORF8:Y73C,S:P26H,S:N501Y,S:A570D,S:D614G,S:P681H,S:T716I,S:L938F,S:S982A,S:D	3	19
hCoV-19/Mexico/SIN_CIAD-CLN_D1477/2021	EPI_ISL_2680924	OL461563	B.1.1.7	GR	33	C241T,C913T,C3037T,C3267T,A3492G,C5388A,C5986T,T6954C,G8017T,C11750T,C14408T,C14676T,C15279T,T16176C,C21639A,A23063T,C23271A,A23403G,C23604A,C23709T,C24374T,T24506G,G24914C,C27972T,G28048T,A28111G,G28280C,A28281T,T28282A,G28881A,G28882A,G2	22	N:D3L,N:R203K,N:G204R,N:S235F,ORF1a:T1001I,ORF1a:N1076S,ORF1a:A1708D,ORF1a:I2230T,ORF1a:L3829F,ORF1b:P314L,ORF8:Q27*,ORF8:R52I,ORF8:Y73C,S:P26H,S:N501Y,S:A570D,S:D614G,S:P681H,S:T716I,S:L938F,S:S982A,S:D	3	17
hCoV-19/Mexico/SIN_CIAD-CLN_D1511/2021	EPI_ISL_2680927	OL461564	B.1.1.7	GR	34	C241T,C913T,C3037T,C3267T,A3492G,C5388A,C5986T,T6954C,G8017T,C11750T,C14408T,C14676T,C15279T,T16176C,C21639A,C22747T,A23063T,C23271A,A23403G,C23604A,C23709T,C24374T,T24506G,G24914C,C27972T,G28048T,A28111G,G28280C,A28281T,T28282A,G28881A,G28	22	N:D3L,N:R203K,N:G204R,N:S235F,ORF1a:T1001I,ORF1a:N1076S,ORF1a:A1708D,ORF1a:I2230T,ORF1a:L3829F,ORF1b:P314L,ORF8:Q27*,ORF8:R52I,ORF8:Y73C,S:P26H,S:N501Y,S:A570D,S:D614G,S:P681H,S:T716I,S:L938F,S:S982A,S:D	3	15
hCoV-19/Mexico/SIN_CIAD-CLN_F0851/2021	EPI_ISL_2533936	OL461565	Q.3	GR	36	C241T,C913T,C3037T,C3267T,G5023A,C5388A,C5986T,T6954C,A9085G,G10396T,C14408T,C14676T,C15279T,T16176C,A17615G,G20962A,T21318C,A23063T,C23271A,A23403G,C23604A,C23709T,T24506G,G24914C,G26730C,C26826T,C27972T,G28048T,A28111G,G28280C,A28281T,T28282A,G28881A,G28882A,G28883C,C28977T	22	M:V70L,N:D3L,N:R203K,N:G204R,N:S235F,ORF1a:T1001I,ORF1a:M1586I,ORF1a:A1708D,ORF1a:I2230T,ORF1b:P314L,ORF1b:K1383R,ORF1b:D2499N,ORF8:Q27*,ORF8:R52I,ORF8:Y73C,S:N501Y,S:A570D,S:D614G,S:P681H,S:T716I,S:S982A,S:D1118H	3	9
hCoV-19/Mexico/SIN_CIAD-CLN_F0854/2021	EPI_ISL_2680928	OL461566	Q.3	GRY	36	C241T,C913T,C3037T,C3267T,G5023A,C5388A,C5986T,T6954C,A9085G,G10396T,C14408T,C14676T,C15279T,T16176C,A17615G,G20962A,G22104T,A23063T,C23271A,A23403G,C23604A,C23709T,T24506G,G24914C,G26730C,C26826T,C27972T,G28048T,A28111G,G28280C,A28281T,T28282A,G28881A,G28882A,G28883C,C28977T	23	M:V70L,N:D3L,N:R203K,N:G204R,N:S235F,ORF1a:T1001I,ORF1a:M1586I,ORF1a:A1708D,ORF1a:I2230T,ORF1b:P314L,ORF1b:K1383R,ORF1b:D2499N,ORF8:Q27*,ORF8:R52I,ORF8:Y73C,S:G181V,S:N501Y,S:A570D,S:D614G,S:P681H,S:T716I,S:S982A,S:D1118H	6	4
hCoV-19/Mexico/SIN_CIAD-CLN_S0600/2021	EPI_ISL_2927971	OL461567	B.1.1.7	GR	33	C241T,C913T,C3037T,C3267T,A3492G,C5388A,C5986T,T6954C,C14408T,C14676T,C15279T,T16176C,C17010T,C21639A,C22326T,A23063T,C23271A,A23403G,C23604A,C23709T,C24374T,T24506G,G24914C,C27972T,G28048T,A28111G,G28280C,A28281T,T28282A,G28881A,G28882A,G	22	N:D3L,N:R203K,N:G204R,N:S235F,ORF1a:T1001I,ORF1a:N1076S,ORF1a:A1708D,ORF1a:I2230T,ORF1b:P314L,ORF8:Q27*,ORF8:R52I,ORF8:Y73C,S:P26H,S:S255F,S:N501Y,S:A570D,S:D614G,S:P681H,S:T716I,S:L938F,S:S982A,S:D1118H	3	11
hCoV-19/Mexico/SIN_CIAD-MZT_HJ1711/2021	EPI_ISL_2533917	OL461568	B.1.1.7	GRY	32	C241T,C913T,C3037T,C3267T,T3532C,C5388A,C5986T,T6954C,C14408T,C14649T,C14676T,C15279T,T16176C,C18348T,A23063T,C23271A,A23403G,C23604A,C23709T,T24506G,G24914C,T25228A,C27972T,G28048T,A28111G,G28280C,A28281T,T28282A,G28881A,G28882A,G28883C,C	18	N:D3L,N:R203K,N:G204R,N:S235F,ORF1a:T1001I,ORF1a:A1708D,ORF1a:I2230T,ORF1b:P314L,ORF8:Q27*,ORF8:R52I,ORF8:Y73C,S:N501Y,S:A570D,S:D614G,S:P681H,S:T716I,S:S982A,S:D1118H	6	0
hCoV-19/Mexico/SIN_CIAD-MZT_HJ1739/2021	EPI_ISL_2680932	OL461569	B.1.1.7	GR	34	C241T,C913T,C2453T,C3037T,C3267T,C5388A,T6954C,C9611T,C14408T,C14676T,C15279T,T16176C,C21855T,G21974C,A23063T,C23271A,A23403G,C23604A,C23709T,T24506G,G24914C,G25273T,G25483A,C27972T,G28048T,A28111G,G28280C,A28281T,T28282A,A28295G,G28881A,G28882A,G28883C,C28977T	25	N:D3L,N:N8D,N:R203K,N:G204R,N:S235F,ORF1a:L730F,ORF1a:T1001I,ORF1a:A1708D,ORF1a:I2230T,ORF1a:L3116F,ORF1b:P314L,ORF3a:A31T,ORF8:Q27*,ORF8:R52I,ORF8:Y73C,S:S98F,S:D138H,S:N501Y,S:A570D,S:D614G,S:P681H,S:T716I,S:S982A,S:D1118H,S:M1237I	1	362
hCoV-19/Mexico/SIN_CIAD-MZT_HJ1742/2021	EPI_ISL_2680933	OL461570	B.1.1.7	GRY	38	C241T,C913T,C2453T,C3037T,C3267T,T5152C,C5388A,G6554T,T6954C,C9611T,C14408T,C14676T,C15279T,T16176C,C21855T,G21974C,A23063T,C23271A,A23403G,C23604A,C23709T,T24506G,G24914C,G25273T,T25711C,C26882T,C27972T,G27996A,G28048T,A28111G,G28280C,A28281T,T28282A,A28295G,G28881A,G28882A,G28883C,C28977T	27	N:D3L,N:N8D,N:R203K,N:G204R,N:S235F,ORF1a:L730F,ORF1a:T1001I,ORF1a:A1708D,ORF1a:A2097S,ORF1a:I2230T,ORF1a:L3116F,ORF1b:P314L,ORF3a:Y107H,ORF8:Q27*,ORF8:D35N,ORF8:R52I,ORF8:Y73C,S:S98F,S:D138H,S:N501Y,S:A570D,S:D614G,S:P681H,S:T716I,S:S982A,S:D1118H,S:M1237I	6	305

hCoV-19/Mexico/SIN_CIAD-MZT_HJ1755/2021	EPI_ISL_2680936	OL461571	B.1.1.7	GR	38	C241T,C913T,C2453T,C3037T,C3267T,C3646T,C5388A,G5516T,C5986T,T6954C,C9611T,C14408T,C14676T,C15279T,T16176C,C18421T,C21855T,G21974C,A23063T,C23271A,A23403G,C23604A,C23709T,T24506G,G24914C,G25273T,C25831T,C27972T,G28048T,A28111G,G28280C,A28281T,T28282A,A28295G,G28881A,G28882A,G28883C,C28977T	27	N:D3L,N:N8D,N:R203K,N:G204R,N:S235F,ORF1a:L730F,ORF1a:T1001I,ORF1a:A1708D,ORF1a:V1751L,ORF1a:I2230T,ORF1a:L3116F,ORF1b:P314L,ORF1b:P1652S,ORF3a:L147F,ORF8:Q27*,ORF8:R52I,ORF8:Y73C,S:S98F,S:D138H,S:N501Y,S:A570D,S:D614G,S:P681H,S:T716I,S:S982A,S:D1118H,S:M1237I	3	15
hCoV-19/Mexico/SIN_CIAD-MZT_HJ1757/2021	EPI_ISL_2894554	OL461572	P.1.1	GR	39	C241T,C913T,C2749T,C3037T,C5175T,C5388A,A6319G,A6613G,C7279T,T7984C,C12778T,C12784T,C13860T,C13887T,C14408T,C14676T,C15279T,T16176C,G17259T,C19390T,T19866C,C20104T,C21614T,C21621A,C21638T,A23063T,A23403G,C23604A,C24642T,G25088T,G28048T,A28095T,A28111G,G28280C,A28281T,T28282A,G28881A,G28882A,G28883C	20	N:D3L,N:R203K,N:G204R,ORF1a:T1637I,ORF1a:A1708D,ORF1b:P314L,ORF1b:E1264D,ORF1b:P1975S,ORF1b:L2213F,ORF8:R52I,ORF8:K68*,ORF8:Y73C,S:L18F,S:T20N,S:P26S,S:N501Y,S:D614G,S:P681H,S:T1027I,S:V1176F	4	0
hCoV-19/Mexico/SIN_CIAD-MZT_HJ1776/2021	EPI_ISL_2894557	OL461573	B.1.1.7	GRY	42	C241T,C913T,G2281A,C3037T,C3267T,C5388A,C5986T,G6094T,T6954C,A12162G,C13665T,C14408T,C14676T,C15279T,C15720T,T16176C,A17615G,C18747T,C20844T,A21137G,A23063T,C23271A,A23403G,C23604A,C23709T,T24506G,G24914C,G25135T,C26110T,G26828T,C27972T,G27987T,G28048T,A28111G,G28280C,A28281T,T28282A,G28881A,G28882A,G28883C,C28977T,C	25	N:D3L,N:R203K,N:G204R,N:S235F,ORF1a:T1001I,ORF1a:A1708D,ORF1a:Q1943H,ORF1a:I2230T,ORF1a:Q3966R,ORF1b:P314L,ORF1b:K1383R,ORF1b:K2557R,ORF3a:P240S,ORF8:Q27*,ORF8:V32L,ORF8:R52I,ORF8:Y73C,S:N501Y,S:A570D,S:D614G,S:P681H,S:T716I,S:S982A,S:D1118H,S:K1191N	7	1
hCoV-19/Mexico/SIN_CIAD-MZT_HJ1782/2021	EPI_ISL_2894558	OL461574	B.1.1.7	GRY	35	C241T,C913T,C2453T,C3037T,C3267T,C5388A,C5986T,T6954C,C9611T,C14408T,C14676T,C15279T,T16176C,C21855T,G21974C,A22005G,A23063T,C23271A,A23403G,C23604A,C23709T,T24506G,G24914C,G25273T,C27972T,G28048T,A28111G,G28280C,A28281T,T28282A,A28295G,G28881A,G28882A,G28883C,C28977T	25	N:D3L,N:N8D,N:R203K,N:G204R,N:S235F,ORF1a:L730F,ORF1a:T1001I,ORF1a:A1708D,ORF1a:I2230T,ORF1a:L3116F,ORF1b:P314L,ORF8:Q27*,ORF8:R52I,ORF8:Y73C,S:S98F,S:D138H,S:N148S,S:N501Y,S:A570D,S:D614G,S:P681H,S:T716I,S:S982A,S:D1118H,S:M1237I	3	0
hCoV-19/Mexico/SIN_CIAD-MZT_HJ1922/2021	EPI_ISL_2927976	OL461575	B.1.1.7	GR	36	C241T,C913T,C2110T,C3037T,C3267T,C5175T,C5388A,C5986T,T6954C,T7984C,C14120T,C14408T,C14676T,C15279T,T16176C,C19017T,C19390T,T19866C,G21123T,A23063T,C23271A,A23403G,C23604A,C23709T,T24506G,G24914C,C27972T,G28048T,A28111G,G28280C,A28281T,T28282A,G28881A,G28882A,G28883C,C28977T	21	N:D3L,N:R203K,N:G204R,N:S235F,ORF1a:T1001I,ORF1a:T1637I,ORF1a:A1708D,ORF1a:I2230T,ORF1b:P218L,ORF1b:P314L,ORF1b:P1975S,ORF8:Q27*,ORF8:R52I,ORF8:Y73C,S:N501Y,S:A570D,S:D614G,S:P681H,S:T716I,S:S982A,S:D1118H	5	13
hCoV-19/Mexico/SIN_CIAD-MZT_ID2430/2021	EPI_ISL_2533913	OL461576	B.1.1.7	GR	45	C241T,C913T,C3037T,C3267T,C5388A,C5986T,G6421T,T6954C,T7204C,C9298T,T11288A,C11289A,T11290G,T11296G,C14408T,C14676T,C15279T,T16176C,A17153G,G18880T,C19554T,C21762G,T21763G,A21764T,A21766C,G22094A,A23063T,C23271A,A23403G,C23604A,C23709T,C24374T,T24506G,G24914C,C27972T,G28048T,A28111G,G28280C,A28281T,T28282A,G28774T,G28	27	N:D3L,N:L167F,N:R203K,N:G204R,N:S235F,ORF1a:T1001I,ORF1a:A1708D,ORF1a:I2230T,ORF1a:S3675K,ORF1a:F3677L,ORF1b:P314L,ORF1b:Y1229C,ORF1b:A1805S,ORF8:Q27*,ORF8:R52I,ORF8:Y73C,S:I68F,S:V70I,S:D178N,S:N501Y,S:A570D,S:D614G,S:P681H,S:T716I,S:L938F,S:S982A,S:D1118H	1	0
hCoV-19/Mexico/SIN_CIAD-MZT_ID2531/2021	EPI_ISL_2533918	OL461577	B.1.1.7	GR	41	C241T,C913T,G2281A,C3037T,C3267T,C5388A,C5986T,T6954C,A12162G,C13665T,C14408T,C14676T,C15279T,C15720T,T16176C,A17615G,C18747T,C20844T,A21137G,A23063T,C23271A,A23403G,C23604A,C23709T,T24506G,G24914C,G25135T,C26110T,G26828T,C27972T,G27987T,G28048T,A28111G,G28280C,A28281T,T28282A,G28881A,G28882A,G28883C,C28977T,C29095T	24	N:D3L,N:R203K,N:G204R,N:S235F,ORF1a:T1001I,ORF1a:A1708D,ORF1a:I2230T,ORF1a:Q3966R,ORF1b:P314L,ORF1b:K1383R,ORF1b:K2557R,ORF3a:P240S,ORF8:Q27*,ORF8:V32L,ORF8:R52I,ORF8:Y73C,S:N501Y,S:A570D,S:D614G,S:P681H,S:T716I,S:S982A,S:D1118H,S:K1191N	3	11
hCoV-19/Mexico/SIN_CIAD-MZT_ID2728/2021	EPI_ISL_2927972	OL461578	B.1.1.7	GRY	35	C241T,C913T,C2110T,C3037T,C3267T,C5175T,C5388A,C5986T,T6954C,T7984C,C14120T,C14408T,C14676T,C15279T,T16176C,C19390T,T19866C,G21123T,A23063T,C23271A,A23403G,C23604A,C23709T,T24506G,G24914C,C27972T,G28048T,A28111G,G28280C,A28281T,T28282A,G28881A,G28882A,G28883C,C28977T	21	N:D3L,N:R203K,N:G204R,N:S235F,ORF1a:T1001I,ORF1a:T1637I,ORF1a:A1708D,ORF1a:I2230T,ORF1b:P218L,ORF1b:P314L,ORF1b:P1975S,ORF8:Q27*,ORF8:R52I,ORF8:Y73C,S:N501Y,S:A570D,S:D614G,S:P681H,S:T716I,S:S982A,S:D1118H	6	0
hCoV-19/Mexico/SIN_CIAD-MZT_S6810/2021	EPI_ISL_2533914	OL461579	B.1.1.7	GR	38	C16G,T18G,C241T,C913T,C2110T,C3037T,C3267T,C5388A,C5986T,T6954C,T7984C,C14120T,C14408T,C14676T,C15279T,T16176C,C19390T,G21123T,A23063T,C23271A,A23403G,C23604A,C23709T,T24506G,G24914C,C26894A,C26895A,C27972T,G28048T,A28095T,A28111G,G28280C,A28281T,T28282A,G28881A,G28882A,G28883C,C28977T	22	M:H125N,N:D3L,N:R203K,N:G204R,N:S235F,ORF1a:T1001I,ORF1a:A1708D,ORF1a:I2230T,ORF1b:P218L,ORF1b:P314L,ORF1b:P1975S,ORF8:Q27*,ORF8:R52I,ORF8:K68*,ORF8:Y73C,S:N501Y,S:A570D,S:D614G,S:P681H,S:T716I,S:S982A,S:D1118H	1	0
hCoV-19/Mexico/SIN_CIAD-MZT_S6840/2021	EPI_ISL_2533915	OL461580	B.1.1.7	GR	36	C241T,C913T,C2110T,C3037T,C3267T,C5388A,C5986T,T6954C,T7984C,C14120T,C14408T,C14676T,C15279T,T16176C,C19390T,G21123T,A23063T,C23271A,A23403G,C23604A,C23709T,T24506G,G24914C,C26894A,C26895A,C27972T,G28048T,A28095T,A28111G,G28280C,A28281T,T28282A,G28881A,G28882A,G28883C,C28977T	22	M:H125N,N:D3L,N:R203K,N:G204R,N:S235F,ORF1a:T1001I,ORF1a:A1708D,ORF1a:I2230T,ORF1b:P218L,ORF1b:P314L,ORF1b:P1975S,ORF8:Q27*,ORF8:R52I,ORF8:K68*,ORF8:Y73C,S:N501Y,S:A570D,S:D614G,S:P681H,S:T716I,S:S982A,S:D1118H	3	14
hCoV-19/Mexico/SIN_CIAD-MZT_S6867/2021	EPI_ISL_2533916	OL461581	B.1.1.7	GRY	36	C241T,C913T,C2110T,C3037T,C3267T,C5388A,C5986T,T6954C,T7984C,C14120T,C14408T,C14676T,C15279T,T16176C,C19390T,G21123T,A23063T,C23271A,A23403G,C23604A,C23709T,T24506G,G24914C,C26894A,C26895A,C27972T,G28048T,A28095T,A28111G,G28280C,A28281T,T28282A,G28881A,G28882A,G28883C,C28977T	22	M:H125N,N:D3L,N:R203K,N:G204R,N:S235F,ORF1a:T1001I,ORF1a:A1708D,ORF1a:I2230T,ORF1b:P218L,ORF1b:P314L,ORF1b:P1975S,ORF8:Q27*,ORF8:R52I,ORF8:K68*,ORF8:Y73C,S:N501Y,S:A570D,S:D614G,S:P681H,S:T716I,S:S982A,S:D1118H	6	0
hCoV-19/Mexico/SIN_CIAD-MZT_S6921/2021	EPI_ISL_2533919	OL461582	B.1.1.7	GRY	35	C241T,C913T,C2453T,C3037T,C3267T,C5388A,C5986T,T6954C,C9611T,C14408T,C14676T,C15279T,T16176C,C21855T,G21974C,A22005G,A23063T,C23271A,A23403G,C23604A,C23709T,T24506G,G24914C,G25273T,C27972T,G28048T,A28111G,G28280C,A28281T,T28282A,A28295G,G28881A,G28882A,G28883C,C28977T	25	N:D3L,N:N8D,N:R203K,N:G204R,N:S235F,ORF1a:L730F,ORF1a:T1001I,ORF1a:A1708D,ORF1a:I2230T,ORF1a:L3116F,ORF1b:P314L,ORF8:Q27*,ORF8:R52I,ORF8:Y73C,S:S98F,S:D138H,S:N148S,S:N501Y,S:A570D,S:D614G,S:P681H,S:T716I,S:S982A,S:D1118H,S:M1237I	6	0
hCoV-19/Mexico/SIN_CIAD-MZT_S7016/2021	EPI_ISL_2680938	OL461583	B.1.1.7	GRY	36	C241T,C913T,C2453T,C3037T,C3267T,C5388A,A5570G,C5986T,T6954C,C9611T,C11235T,C14408T,C14676T,C15279T,T16176C,C21855T,G21974C,A23063T,C23271A,A23403G,C23604A,C23709T,T24506G,G24914C,G25273T,C27972T,G28048T,A28111G,G28280C,A28281T,T28282A,A28295G,G28881A,G28882A,G28883C,C28977T	26	N:D3L,N:N8D,N:R203K,N:G204R,N:S235F,ORF1a:L730F,ORF1a:T1001I,ORF1a:A1708D,ORF1a:M1769V,ORF1a:I2230T,ORF1a:L3116F,ORF1a:A3657V,ORF1b:P314L,ORF8:Q27*,ORF8:R52I,ORF8:Y73C,S:S98F,S:D138H,S:N501Y,S:A570D,S:D614G,S:P681H,S:T716I,S:S982A,S:D1118H,S:M1237I	6	2
hCoV-19/Mexico/SIN_CIAD-MZT_S7028/2021	EPI_ISL_2680941	OL461584	B.1.1.222	GR	32	C241T,C913T,C3037T,C3140T,C5388A,G7067A,C7296T,G8017T,C10029T,T10330G,C11750T,C12789T,C14408T,C14676T,C15279T,T16176C,C21306T,A23063T,C23271A,A23403G,C23604A,A23756G,C27972T,G28048T,A28111G,G28280C,A28281T,T28282A,G28881A,G28882A,G28883C,C28977T	21	N:D3L,N:R203K,N:G204R,N:S235F,ORF1a:P959S,ORF1a:A1708D,ORF1a:E2268K,ORF1a:A2344V,ORF1a:T3255I,ORF1a:D3355E,ORF1a:L3829F,ORF1a:T4175I,ORF1b:P314L,ORF8:Q27*,ORF8:R52I,ORF8:Y73C,S:N501Y,S:A570D,S:D614G,S:P681H,S:T732A	1	1498
hCoV-19/Mexico/SIN_CIAD-MZT_S7158/2021	EPI_ISL_2927974	OL461585	B.1.1.7	GRY	38	C241T,C913T,G1245A,C3037T,C3267T,C5388A,C5986T,G6421T,T6954C,T7204C,C9298T,C14408T,C14676T,C15279T,T16176C,A17153G,G18880T,G22094A,A23063T,C23271A,A23403G,C23604A,C23664T,C23709T,C24374T,T24506G,G24914C,C27972T,G28048T,A28111G,G28280C,A28281T,T28282A,G28774T,G28881A,G28882A,G28883C,C28977T	25	N:D3L,N:L167F,N:R203K,N:G204R,N:S235F,ORF1a:G327D,ORF1a:T1001I,ORF1a:A1708D,ORF1a:I2230T,ORF1b:P314L,ORF1b:Y1229C,ORF1b:A1805S,ORF8:Q27*,ORF8:R52I,ORF8:Y73C,S:D178N,S:N501Y,S:A570D,S:D614G,S:P681H,S:A701V,S:T716I,S:L938F,S:S982A,S:D1118H	6	317
hCoV-19/Mexico/SIN_INER_IMSS_1180/2021	EPI_ISL_2490331	OL461586	B.1.1.7	GRY	32	C241T,C913T,C3037T,C3267T,A3492G,C5388A,C5986T,T6954C,C14408T,C14676T,C15279T,T16176C,C21639A,A23063T,C23271A,A23403G,C23604A,C23709T,C24374T,T24506G,G24914C,A25164T,C27972T,G28048T,A28111G,G28280C,A28281T,T28282A,G28881A,G28882A,G28883C,C	22	N:D3L,N:R203K,N:G204R,N:S235F,ORF1a:T1001I,ORF1a:N1076S,ORF1a:A1708D,ORF1a:I2230T,ORF1b:P314L,ORF8:Q27*,ORF8:R52I,ORF8:Y73C,S:P26H,S:N501Y,S:A570D,S:D614G,S:P681H,S:T716I,S:L938F,S:S982A,S:D1118H,S:Q1201	6	1
hCoV-19/Mexico/SIN-CIAD-CLN_D0174/2021	EPI_ISL_2987687	OL461587	B.1.1.7	GR	36	C241T,C913T,C3037T,C3267T,A3492G,C5388A,C5986T,G6901A,T6954C,C14408T,C14676T,A14862G,C15279T,T16176C,C21639A,G22801T,A23063T,C23271A,A23403G,C23604A,C23709T,C24374T,T24506G,G24914C,A25998G,C27972T,G28027T,G28048T,A28111G,G28280C,A28281T,T28282A,G28881A,G28882A,G28883C,C28977T	22	N:D3L,N:R203K,N:G204R,N:S235F,ORF1a:T1001I,ORF1a:N1076S,ORF1a:A1708D,ORF1a:I2230T,ORF1b:P314L,ORF8:Q27*,ORF8:W45L,ORF8:R52I,ORF8:Y73C,S:P26H,S:N501Y,S:A570D,S:D614G,S:P681H,S:T716I,S:L938F,S:S982A,S:D1118H	3	7
hCoV-19/Mexico/SIN-CIAD-CLN_F0865/2021	EPI_ISL_2987694	OL461588	B.1.1.7	GRY	42	C241T,C913T,C2110T,A2522G,C3037T,C3267T,A3647G,C5388A,C5986T,T6304G,C6317T,T6954C,G7042T,A7323G,A10195G,T11233A,T11686G,C14120T,C14408T,C14676T,C15279T,T16176C,A23063T,C23271A,A23403G,C23604A,C23709T,T24506G,G24914C,C27972T,G28048T,G28075T,G28085T,A28095T,A28111G,G28280C,A28281T,T28282A,G28881A,G28882A,G28883C,C28977	27	N:D3L,N:R203K,N:G204R,N:S235F,ORF1a:T753A,ORF1a:T1001I,ORF1a:I1128V,ORF1a:A1708D,ORF1a:P2018S,ORF1a:I2230T,ORF1a:M2259I,ORF1a:Y2353C,ORF1b:P218L,ORF1b:P314L,ORF8:Q27*,ORF8:R52I,ORF8:C61F,ORF8:E64D,ORF8:K68*,ORF8:Y73C,S:N501Y,S:A570D,S:D614G,S:P681H,S:T716I,S:S982A,S:D1	6	0
hCoV-19/Mexico/SIN-CIAD-MZT_HJ1803/2021	EPI_ISL_2987696	OL461589	B.1.1.7	GRY	40	C241T,C913T,G2281A,C3037T,C3267T,C5388A,T6954C,A12162G,C13665T,C14408T,C14676T,C15279T,C15720T,T16176C,A17615G,C18747T,C20844T,A21137G,A23063T,C23271A,A23403G,C23604A,C23709T,T24506G,G24914C,G25135T,C26110T,G26828T,C27972T,G27987T,G28048T,A28111G,G28280C,A28281T,T28282A,G28881A,G28882A,G28883C,C28977T,C29095T	24	N:D3L,N:R203K,N:G204R,N:S235F,ORF1a:T1001I,ORF1a:A1708D,ORF1a:I2230T,ORF1a:Q3966R,ORF1b:P314L,ORF1b:K1383R,ORF1b:K2557R,ORF3a:P240S,ORF8:Q27*,ORF8:V32L,ORF8:R52I,ORF8:Y73C,S:N501Y,S:A570D,S:D614G,S:P681H,S:T716I,S:S982A,S:D1118H,S:K1191N	6	96

hCoV-19/Mexico/SIN-CIAD-MZT_S7079/2021	EPI_ISL_2987713	OL461590	B.1.1.7	GRY	38	C241T,C913T,C3037T,C3267T,C5388A,C5526T,C5986T,G6421T,T6954C,T7204C,C9298T,C14408T,C14676T,C15279T,T16176C,A17153G,G18880T,C19554T,G22094A,A23063T,C23271A,A23403G,C23604A,C23709T,C24374T,T24506G,G24914C,C27972T,G28048T,A28111G,G28280C,A28281T,T28282A,G28774T,G28881A,G28882A,G28883C,C28977T	24	N:D3L,N:L167F,N:R203K,N:G204R,N:S235F,ORF1a:T1001I,ORF1a:A1708D,ORF1a:T1754I,ORF1a:I2230T,ORF1b:P314L,ORF1b:Y1229C,ORF1b:A1805S,ORF8:Q27*,ORF8:R52I,ORF8:Y73C,S:D178N,S:N501Y,S:A570D,S:D614G,S:P681H,S:T716I,S:L938F,S:S982A,S:D1118H	6	0
hCoV-19/Mexico/SIN-CIAD-MZT_S7103/2021	EPI_ISL_2987717	OL461591	B.1.1.7	GRY	39	C241T,C913T,C3037T,C3267T,C5388A,C5526T,C5986T,G6421T,T6954C,C7195T,T7204C,C9298T,C14408T,C14676T,C15279T,T16176C,A17153G,G18880T,C19554T,G22094A,A23063T,C23271A,A23403G,C23604A,C23709T,C24374T,T24506G,G24914C,C27972T,G28048T,A28111G,G28280C,A28281T,T28282A,G28774T,G28881A,G28882A,G28883C,C28977T	24	N:D3L,N:L167F,N:R203K,N:G204R,N:S235F,ORF1a:T1001I,ORF1a:A1708D,ORF1a:T1754I,ORF1a:I2230T,ORF1b:P314L,ORF1b:Y1229C,ORF1b:A1805S,ORF8:Q27*,ORF8:R52I,ORF8:Y73C,S:D178N,S:N501Y,S:A570D,S:D614G,S:P681H,S:T716I,S:L938F,S:S982A,S:D1118H	6	17
hCoV-19/Mexico/SIN-CIAD-MZT_S7104/2021	EPI_ISL_2987718	OL461592	B.1.1.7	GRY	39	C241T,C913T,C3037T,C3267T,C5388A,C5526T,C5986T,G6421T,T6954C,C7195T,T7204C,C9298T,C14408T,C14676T,C15279T,T16176C,A17153G,G18880T,C19554T,G22094A,A23063T,C23271A,A23403G,C23604A,C23709T,C24374T,T24506G,G24914C,C27972T,G28048T,A28111G,G28280C,A28281T,T28282A,G28774T,G28881A,G28882A,G28883C,C28977T	24	N:D3L,N:L167F,N:R203K,N:G204R,N:S235F,ORF1a:T1001I,ORF1a:A1708D,ORF1a:T1754I,ORF1a:I2230T,ORF1b:P314L,ORF1b:Y1229C,ORF1b:A1805S,ORF8:Q27*,ORF8:R52I,ORF8:Y73C,S:D178N,S:N501Y,S:A570D,S:D614G,S:P681H,S:T716I,S:L938F,S:S982A,S:D1118H	6	0
hCoV-19/Mexico/SIN-CIAD-MZT_S7130/2021	EPI_ISL_2987721	OL461593	B.1.1.7	GRY	38	C241T,C913T,C1288T,C2110T,C3037T,C3267T,C5388A,C5869T,C5986T,T6954C,C6997T,G7042T,A10195G,C14120T,C14408T,C14676T,C15279T,T16176C,A23063T,C23271A,A23403G,C23604A,C23709T,C24418T,T24506G,G24914C,C27972T,G28048T,G28075T,A28095T,A28111G,G28280C,A28281T,T28282A,G28881A,G28882A,G28883C,C28977T	22	N:D3L,N:R203K,N:G204R,N:S235F,ORF1a:T1001I,ORF1a:A1708D,ORF1a:I2230T,ORF1a:M2259I,ORF1b:P218L,ORF1b:P314L,ORF8:Q27*,ORF8:R52I,ORF8:C61F,ORF8:K68*,ORF8:Y73C,S:N501Y,S:A570D,S:D614G,S:P681H,S:T716I,S:S982A,S:D1118H	6	0
hCoV-19/Mexico/SIN-IBT_IMSS_1832/2021	EPI_ISL_2801707	OL461594	B.1.1.7	GRY	36	C241T,C913T,C3037T,C3267T,A3492G,C5388A,C5986T,G6901A,T6954C,C14408T,C14676T,A14862G,C15279T,T16176C,C21639A,G22801T,A23063T,C23271A,A23403G,C23604A,C23709T,C24374T,T24506G,G24914C,A25998G,C27972T,G28027T,G28048T,A28111G,G28280C,A28281T,T28282A,G28881A,G28882A,G28883C,C28977T	22	N:D3L,N:R203K,N:G204R,N:S235F,ORF1a:T1001I,ORF1a:N1076S,ORF1a:A1708D,ORF1a:I2230T,ORF1b:P314L,ORF8:Q27*,ORF8:W45L,ORF8:R52I,ORF8:Y73C,S:P26H,S:N501Y,S:A570D,S:D614G,S:P681H,S:T716I,S:L938F,S:S982A,S:D1118H	6	0
hCoV-19/Mexico/SIN-IBT_IMSS_1871/2021	EPI_ISL_2801739	OL461595	B.1.1.7	GRY	35	C241T,C706T,C913T,C3037T,C3267T,A3492G,C5388A,C5986T,T6954C,C14408T,C14676T,C15279T,T16176C,C16362T,C21639A,C21846T,A23063T,C23271A,A23403G,C23604A,C23709T,C24374T,T24506G,G24914C,A25164T,C27972T,G28048T,A28111G,G28280C,A28281T,T28282A,G28881A,G28882A,G28883C,C28977T	23	N:D3L,N:R203K,N:G204R,N:S235F,ORF1a:T1001I,ORF1a:N1076S,ORF1a:A1708D,ORF1a:I2230T,ORF1b:P314L,ORF8:Q27*,ORF8:R52I,ORF8:Y73C,S:P26H,S:T95I,S:N501Y,S:A570D,S:D614G,S:P681H,S:T716I,S:L938F,S:S982A,S:D1118H,S:Q1201L	6	0
hCoV-19/Mexico/SIN-IBT_IMSS_1879/2021	EPI_ISL_2801745	OL461596	B.1.1.7	GRY	31	C241T,C913T,C3037T,C3267T,A3492G,C5388A,C5986T,T6954C,C14408T,C14676T,C15279T,T16176C,C21639A,A23063T,C23271A,A23403G,C23604A,C23709T,C24374T,T24506G,G24914C,C27972T,G28048T,A28111G,G28280C,A28281T,T28282A,G28881A,G28882A,G28883C,C28977T	21	N:D3L,N:R203K,N:G204R,N:S235F,ORF1a:T1001I,ORF1a:N1076S,ORF1a:A1708D,ORF1a:I2230T,ORF1b:P314L,ORF8:Q27*,ORF8:R52I,ORF8:Y73C,S:P26H,S:N501Y,S:A570D,S:D614G,S:P681H,S:T716I,S:L938F,S:S982A,S:D1118H	6	0
hCoV-19/Mexico/SIN-LANGEBIO_IMSS_1269/2021	EPI_ISL_2942427	OL461597	B.1.1.7	GRY	32	C241T,C913T,C3037T,C3267T,C5388A,C5986T,T6954C,C14408T,C14676T,C15279T,T16176C,G19677T,A23063T,C23086T,C23271A,A23403G,C23604A,C23709T,T24506G,G24914C,C26833T,C27972T,G28048T,A28111G,G28280C,A28281T,T28282A,G28881A,G28882A,G28883C,C28977T,	20	M:A104V,N:D3L,N:R203K,N:G204R,N:S235F,ORF1a:T1001I,ORF1a:A1708D,ORF1a:I2230T,ORF1b:P314L,ORF1b:Q2070H,ORF8:Q27*,ORF8:R52I,ORF8:Y73C,S:N501Y,S:A570D,S:D614G,S:P681H,S:T716I,S:S982A,S:D1118H	6	0
hCoV-19/Mexico/SIN-LANGEBIO_IMSS_1277/2021	EPI_ISL_2942433	OL461598	B.1.1.7	GRY	40	C222T,C241T,C913T,C2110T,C3037T,C3267T,C4423T,C5388A,C5986T,C6681T,T6954C,G7042T,A10359G,C14120T,C14408T,C14676T,C15279T,T16176C,C17762T,A23063T,C23271A,A23403G,C23604A,G23608T,C23709T,T24506G,G24914C,T25180C,T27328C,C27972T,G28048T,A28095T,A28111G,G28280C,A28281T,T28282A,G28881A,G28882A,G28883C,C28977T	25	N:D3L,N:R203K,N:G204R,N:S235F,ORF1a:T1001I,ORF1a:A1708D,ORF1a:A2139V,ORF1a:I2230T,ORF1a:M2259I,ORF1a:K3365R,ORF1b:P218L,ORF1b:P314L,ORF1b:A1432V,ORF6:S43P,ORF8:Q27*,ORF8:R52I,ORF8:K68*,ORF8:Y73C,S:N501Y,S:A570D,S:D614G,S:P681H,S:T716I,S:S982A,S:D1118H	6	16
hCoV-19/Mexico/SIN-LANGEBIO_IMSS_1291/2021	EPI_ISL_2942444	OL461599	B.1.1.7	GRY	40	C241T,C913T,C3037T,C3267T,C5388A,C5526T,C5986T,G6421T,T6954C,T7204C,C9298T,A13357G,C14408T,C14676T,C15279T,T16176C,A17153G,G18880T,C19554T,G22094A,A23063T,C23271A,A23403G,C23604A,C23709T,C24374T,T24506G,G24914C,C27972T,C28006T,G28048T,A28111G,G28280C,A28281T,T28282A,G28774T,G28881A,G28882A,G28883C,C28977T	25	N:D3L,N:L167F,N:R203K,N:G204R,N:S235F,ORF1a:T1001I,ORF1a:A1708D,ORF1a:T1754I,ORF1a:I2230T,ORF1b:P314L,ORF1b:Y1229C,ORF1b:A1805S,ORF8:Q27*,ORF8:P38L,ORF8:R52I,ORF8:Y73C,S:D178N,S:N501Y,S:A570D,S:D614G,S:P681H,S:T716I,S:L938F,S:S982A,S:D1118H	6	1
hCoV-19/Mexico/SIN-LANGEBIO_IMSS_1293/2021	EPI_ISL_2942445	OL461600	B.1.1.7	GRY	39	C241T,C292T,C913T,C2156T,T2446A,C3037T,C3267T,C5388A,C5986T,T6954C,A8010T,C14408T,C14676T,C15279T,T16176C,T16563A,C16580T,G19101T,A23063T,C23170T,C23271A,A23403G,C23604A,C23709T,T24506G,G24914C,C25626T,G26959A,C27972T,G28048T,A28111G,G28280C,A28281T,T28282A,A28295C,G28881A,G28882A,G28883C,C28977T	25	M:R146H,N:D3L,N:N8H,N:R203K,N:G204R,N:S235F,ORF1a:L631F,ORF1a:T1001I,ORF1a:A1708D,ORF1a:I2230T,ORF1a:D2582V,ORF1b:P314L,ORF1b:T1038I,ORF1b:Q1878H,ORF8:Q27*,ORF8:R52I,ORF8:Y73C,ORF9b:K4N,S:N501Y,S:A570D,S:D614G,S:P681H,S:T716I,S:S982A,S:D1118H	6	16
hCoV-19/Mexico/SIN-LANGEBIO_IMSS_1305/2021	EPI_ISL_2942455	OL461601	B.1.1.7	GRY	35	C241T,C706T,C913T,C3037T,C3267T,A3492G,C5388A,C5986T,T6954C,C14408T,C14676T,C15279T,T16176C,C16362T,C21639A,C21846T,A23063T,C23271A,A23403G,C23604A,C23709T,C24374T,T24506G,G24914C,A25164T,C27972T,G28048T,A28111G,G28280C,A28281T,T28282A,G28881A,G28882A,G28883C,C28977T	23	N:D3L,N:R203K,N:G204R,N:S235F,ORF1a:T1001I,ORF1a:N1076S,ORF1a:A1708D,ORF1a:I2230T,ORF1b:P314L,ORF8:Q27*,ORF8:R52I,ORF8:Y73C,S:P26H,S:T95I,S:N501Y,S:A570D,S:D614G,S:P681H,S:T716I,S:L938F,S:S982A,S:D1118H,S:Q1201L	6	14
hCoV-19/Mexico/SLP_IBT_IMSS_1474/2021	EPI_ISL_2681212	OL461602	B.1.1.7	GRY	41	C241T,C913T,C2110T,C2299T,C3037T,C3267T,C4582T,C5388A,C5986T,T6954C,G7042T,T9379C,A10195G,C11750T,C14120T,C14408T,C14676T,C15279T,T16176C,G20718T,C21036T,A23063T,C23271A,A23403G,C23604A,C23709T,T24506G,G24914C,G26172A,C27972T,G28048T,G28075T,A28095T,A28111G,G28280C,A28281T,T28282A,G28881A,G28882A,G28883C,C28977T	25	N:D3L,N:R203K,N:G204R,N:S235F,ORF1a:T1001I,ORF1a:A1708D,ORF1a:I2230T,ORF1a:M2259I,ORF1a:L3829F,ORF1b:P218L,ORF1b:P314L,ORF1b:M2417I,ORF3a:M260I,ORF8:Q27*,ORF8:R52I,ORF8:C61F,ORF8:K68*,ORF8:Y73C,S:N501Y,S:A570D,S:D614G,S:P681H,S:T716I,S:S982A,S:D1118H	6	83
hCoV-19/Mexico/SLP-IBT_IMSS_1823/2021	EPI_ISL_2801699	OL461603	B.1.1.7	GR	40	C241T,G626A,C913T,C3037T,C3267T,C5388A,C5986T,C6636T,T6954C,G7042T,C10138T,A10195G,C14120T,C14408T,C14676T,C15279T,T16176C,C16260T,C20031T,A23063T,C23271A,A23403G,C23604A,C23709T,T24506G,G24914C,T25640G,C27286T,C27972T,G28048T,G28075T,A28095T,A28111G,G28280C,A28281T,T28282A,G28881A,G28882A,G28883C,C28977T	26	N:D3L,N:R203K,N:G204R,N:S235F,ORF1a:V121I,ORF1a:T1001I,ORF1a:A1708D,ORF1a:T2124I,ORF1a:I2230T,ORF1a:M2259I,ORF1b:P218L,ORF1b:P314L,ORF3a:L83W,ORF6:L29F,ORF8:Q27*,ORF8:R52I,ORF8:C61F,ORF8:K68*,ORF8:Y73C,S:N501Y,S:A570D,S:D614G,S:P681H,S:T716I,S:S982A,S:D1118H	6	664
hCoV-19/Mexico/SLP-IBT_IMSS_1824/2021	EPI_ISL_2801700	OL461604	B.1.1.7	GR	39	C241T,G626A,C913T,C2110T,C3037T,C3267T,C5388A,C5986T,C6636T,T6954C,G7042T,C10138T,A10195G,C14120T,C14408T,C14676T,C15279T,T16176C,A23063T,C23271A,A23403G,C23604A,C23709T,T24506G,G24914C,T25640G,C27286T,C27972T,G28048T,G28075T,A28095T,A28111G,G28280C,A28281T,T28282A,G28881A,G28882A,G28883C,C28977T	26	N:D3L,N:R203K,N:G204R,N:S235F,ORF1a:V121I,ORF1a:T1001I,ORF1a:A1708D,ORF1a:T2124I,ORF1a:I2230T,ORF1a:M2259I,ORF1b:P218L,ORF1b:P314L,ORF3a:L83W,ORF6:L29F,ORF8:Q27*,ORF8:R52I,ORF8:C61F,ORF8:K68*,ORF8:Y73C,S:N501Y,S:A570D,S:D614G,S:P681H,S:T716I,S:S982A,S:D1118H	6	0
hCoV-19/Mexico/SLP-IBT_IMSS_1830/2021	EPI_ISL_2801705	OL461605	B.1.1.7	GRY	45	C241T,C913T,C2110T,C3037T,C3267T,G4300T,C4900T,C5388A,C5986T,G6576T,T6954C,T7984C,C10747T,C14120T,C14408T,C14676T,C15279T,T16176C,C18120T,C19390T,A23063T,C23271A,T23323C,A23403G,C23604A,C23709T,T24506G,G24914C,C26176T,C26313T,C27737T,C27972T,G28048T,A28095T,A28111G,G28280C,A28281T,T28282A,G28817T,A28877T,G28878C,G28881	25	N:D3L,N:A182S,N:R203K,N:G204R,N:S235F,ORF1a:T1001I,ORF1a:A1708D,ORF1a:S2104I,ORF1a:I2230T,ORF1b:P218L,ORF1b:P314L,ORF1b:P1975S,ORF3a:P262S,ORF7a:T115I,ORF8:Q27*,ORF8:R52I,ORF8:K68*,ORF8:Y73C,S:N501Y,S:A570D,S:D614G,S:P681H,S:T716I,S:S982A,S:D1118H	6	0
hCoV-19/Mexico/SLP-LANGEBIO_IMSS_0974/2021	EPI_ISL_2671700	OL461606	B.1.1.7	GRY	40	C241T,C906T,C913T,C1889T,C2147A,C3037T,C3267T,A5111G,C5388A,C5986T,T6954C,A12162G,C14408T,C14676T,C15279T,C15720T,G16122T,T16176C,A17615G,G20578T,A21137G,A23063T,C23271A,A23403G,C23604A,C23709T,T24506G,G24914C,G25135T,C26110T,C27972T,G28048T,A28111G,G28280C,A28281T,T28282A,G28881A,G28882A,G28883C,C28977T	29	N:D3L,N:R203K,N:G204R,N:S235F,ORF1a:T214I,ORF1a:R542C,ORF1a:L628I,ORF1a:T1001I,ORF1a:K1616E,ORF1a:A1708D,ORF1a:I2230T,ORF1a:Q3966R,ORF1b:P314L,ORF1b:E885D,ORF1b:K1383R,ORF1b:V2371L,ORF1b:K2557R,ORF3a:P240S,ORF8:Q27*,ORF8:R52I,ORF8:Y73C,S:N501Y,S:A570D,S:D614G,S:P681H,S:T716I,S:S982A,S:D1118H,S:K1191N	6	91
hCoV-19/Mexico/SLP-LANGEBIO_IMSS_1326/2021	EPI_ISL_2942471	OL461607	B.1.1.291	GR	31	C241T,C913T,C3037T,C3267T,C5388A,A10195G,A10323G,C13197T,C14120T,C14408T,C15279T,C15933T,T16176C,G21974T,G23012A,A23063T,C23271A,A23403G,C23604A,C23709T,G24914C,C27972T,G28048T,A28111G,G28280C,A28281T,T28282A,G28881A,G28882A,G28883C,C295	21	N:D3L,N:R203K,N:G204R,N:S416L,ORF1a:T1001I,ORF1a:A1708D,ORF1a:K3353R,ORF1a:T4311I,ORF1b:P218L,ORF1b:P314L,ORF8:Q27*,ORF8:R52I,ORF8:Y73C,S:D138Y,S:E484K,S:N501Y,S:A570D,S:D614G,S:P681H,S:T716I,S:D1118H	3	0
hCoV-19/Mexico/SLP-LANGEBIO_IMSS_1327/2021	EPI_ISL_2942472	OL461608	B.1.1.7	GRY	43	C222T,C241T,C913T,C2110T,A2895G,C3037T,C3267T,C3743T,C5388A,C5986T,T6954C,G7042T,C12733T,C14120T,C14408T,C14676T,A15046G,C15279T,T16176C,C17762T,C21846T,G22630T,A23063T,C23271A,A23403G,C23604A,C23709T,T24506G,G24914C,T25180C,G25311T,C27741T,C27972T,G28048T,A28111G,G28280C,A28281T,T28282A,G28881A,G28882A,G28883C,C28977	27	N:D3L,N:R203K,N:G204R,N:S235F,ORF1a:D877G,ORF1a:T1001I,ORF1a:H1160Y,ORF1a:A1708D,ORF1a:I2230T,ORF1a:M2259I,ORF1b:P218L,ORF1b:P314L,ORF1b:I527V,ORF1b:A1432V,ORF8:Q27*,ORF8:R52I,ORF8:Y73C,S:T95I,S:K356N,S:N501Y,S:A570D,S:D614G,S:P681H,S:T716I,S:S982A,S:D1118H,S:C1250F	6	0

hCoV-19/Mexico/SLP-LANGEBIO_IMSS_1333/2021	EPI_ISL_2942476	OL461609	B.1.1.7	GRY	36	C241T,C913T,C2110T,C3037T,C3267T,G4904T,C5388A,T6954C,G7042T,A10195G,C10977T,C14120T,C14408T,C14676T,C15279T,C15933T,T16176C,A23063T,C23271A,A23403G,C23604A,C23709T,T24506G,G24914C,A27745G,C27972T,G28048T,G28075T,A28111G,G28280C,A28281T,T28282A,G28881A,G28882A,G28883C,C28977T	24	N:D3L,N:R203K,N:G204R,N:S235F,ORF1a:T1001I,ORF1a:D1547Y,ORF1a:A1708D,ORF1a:I2230T,ORF1a:M2259I,ORF1a:A3571V,ORF1b:P218L,ORF1b:P314L,ORF7a:R118G,ORF8:Q27*,ORF8:R52I,ORF8:C61F,ORF8:Y73C,S:N501Y,S:A570D,S:D614G,S:P681H,S:T716I,S:S982A,S:D1118H	6	92
hCoV-19/Mexico/SLP-LANGEBIO_IMSS_1334/2021	EPI_ISL_2942477	OL461610	B.1.1.7	GRY	36	C913T,C3037T,C3267T,C5388A,C6120T,T6954C,G7042T,A10195G,C14120T,C14408T,C15279T,C15933T,T16176C,C17550T,C21597T,A23063T,C23271A,A23403G,C23604A,C23709T,T24506G,G24914C,G25855T,A27745G,G27762T,C27972T,G28048T,G28075T,A28111G,G28280C,A28281T,T28282A,G28881A,G28882A,G28883C,C28977T	26	N:D3L,N:R203K,N:G204R,N:S235F,ORF1a:T1001I,ORF1a:A1708D,ORF1a:S1952L,ORF1a:I2230T,ORF1a:M2259I,ORF1b:P218L,ORF1b:P314L,ORF3a:D155Y,ORF7a:R118G,ORF7b:E3*,ORF8:Q27*,ORF8:R52I,ORF8:C61F,ORF8:Y73C,S:S12F,S:N501Y,S:A570D,S:D614G,S:P681H,S:T716I,S:S982A,S:D1118H	6	635
hCoV-19/Mexico/SON_CIAD-HMO_16010/2021	EPI_ISL_2927979	OL461611	B.1.1.7	GRY	33	C241T,C913T,C3037T,C3267T,C5388A,C5986T,C6525T,T6954C,C9298T,C14408T,C14676T,C15279T,T16176C,A23063T,C23271A,A23403G,C23604A,C23709T,C24374T,T24506G,G24914C,T26486C,C27972T,G28048T,A28111G,G28280C,A28281T,T28282A,G28881A,G28882A,G28883C,C2	20	N:D3L,N:R203K,N:G204R,N:S235F,ORF1a:T1001I,ORF1a:A1708D,ORF1a:T2087I,ORF1a:I2230T,ORF1b:P314L,ORF8:Q27*,ORF8:R52I,ORF8:Y73C,S:N501Y,S:A570D,S:D614G,S:P681H,S:T716I,S:L938F,S:S982A,S:D1118H	6	52
hCoV-19/Mexico/SON_CIAD-HMO_16011/2021	EPI_ISL_2927980	OL461612	B.1.1.7	GRY	33	C241T,C913T,C3037T,C3267T,C5388A,C5986T,C6525T,T6954C,C9298T,C14408T,C14676T,C15279T,T16176C,A23063T,C23271A,A23403G,C23604A,C23709T,C24374T,T24506G,G24914C,T26486C,C27972T,G28048T,A28111G,G28280C,A28281T,T28282A,G28881A,G28882A,G28883C,C2	20	N:D3L,N:R203K,N:G204R,N:S235F,ORF1a:T1001I,ORF1a:A1708D,ORF1a:T2087I,ORF1a:I2230T,ORF1b:P314L,ORF8:Q27*,ORF8:R52I,ORF8:Y73C,S:N501Y,S:A570D,S:D614G,S:P681H,S:T716I,S:L938F,S:S982A,S:D1118H	6	41
hCoV-19/Mexico/SON_CIAD-HMO_2555/2021	EPI_ISL_2894566	OL461613	B.1.1.7	GRY	34	C241T,C913T,C3037T,C3267T,C4099T,C5005T,C5388A,C5986T,T6954C,A8244G,C9298T,C14408T,C14676T,C15279T,T16176C,A20075T,A23063T,C23271A,A23403G,C23604A,C23709T,C24374T,T24506G,G24914C,C27972T,G28048T,A28111G,G28280C,A28281T,T28282A,G28881A,G2888	21	N:D3L,N:R203K,N:G204R,N:S235F,ORF1a:T1001I,ORF1a:A1708D,ORF1a:I2230T,ORF1a:Q2660R,ORF1b:P314L,ORF1b:Q2203L,ORF8:Q27*,ORF8:R52I,ORF8:Y73C,S:N501Y,S:A570D,S:D614G,S:P681H,S:T716I,S:L938F,S:S982A,S:D1118H	3	20
hCoV-19/Mexico/SON_CIAD-HMO_2613/2021	EPI_ISL_2894569	OL461614	B.1.1.7	GRY	30	C241T,C913T,C3037T,C3267T,C4099T,C5005T,C5388A,T6954C,C9298T,C14408T,C14676T,A20075T,A23063T,C23271A,A23403G,C23604A,C23709T,C24374T,T24506G,G24914C,G28048T,A28111G,G28280C,A28281T,T28282A,G28881A,G28882A,G28883C,C28977T,A29818G	19	N:D3L,N:R203K,N:G204R,N:S235F,ORF1a:T1001I,ORF1a:A1708D,ORF1a:I2230T,ORF1b:P314L,ORF1b:Q2203L,ORF8:R52I,ORF8:Y73C,S:N501Y,S:A570D,S:D614G,S:P681H,S:T716I,S:L938F,S:S982A,S:D1118H	3	116
hCoV-19/Mexico/SON-IBT_IMSS_1840/2021	EPI_ISL_2801715	OL461615	B.1.1.7	GRY	35	C241T,C913T,C3037T,C3267T,C5388A,C5986T,C6525T,T6954C,C9298T,A10807G,G13385T,C14408T,C14676T,C15279T,T16176C,A23063T,C23271A,A23403G,C23604A,C23709T,C24374T,T24506G,G24914C,T26486C,C27972T,G28048T,A28111G,G28280C,A28281T,T28282A,G28881A,G28882A,G28883C,C28977T,C29144T	21	N:D3L,N:R203K,N:G204R,N:S235F,ORF1a:T1001I,ORF1a:A1708D,ORF1a:T2087I,ORF1a:I2230T,ORF1a:G4374C,ORF1b:P314L,ORF8:Q27*,ORF8:R52I,ORF8:Y73C,S:N501Y,S:A570D,S:D614G,S:P681H,S:T716I,S:L938F,S:S982A,S:D1118H	6	707
hCoV-19/Mexico/SON-LANGEBIO_IMSS_1288/2021	EPI_ISL_2942442	OL461616	B.1.1.7	GRY	41	C21T,C241T,C913T,C3037T,C3267T,C3486T,T4057C,C5388A,C5986T,T6954C,C11750T,C14408T,C14676T,C15279T,C15386T,T16176C,G16917T,C18828T,A23063T,C23271A,A23403G,C23604A,C23709T,T24436C,T24506G,G24914C,C26270T,G26746T,C26822T,C27476T,C27972T,G28048T,A28111G,G28280C,A28281T,T28282A,C28290T,G28881A,G28882A,G28883C,C28977T	26	E:T9I,M:W75L,N:D3L,N:P6L,N:R203K,N:G204R,N:S235F,ORF1a:T1001I,ORF1a:A1074V,ORF1a:A1708D,ORF1a:I2230T,ORF1a:L3829F,ORF1b:P314L,ORF1b:S640L,ORF7a:T28I,ORF8:Q27*,ORF8:R52I,ORF8:Y73C,ORF9b:P3S,S:N501Y,S:A570D,S:D614G,S:P681H,S:T716I,S:S982A,S:D1118H	6	0
hCoV-19/Mexico/SON-LANGEBIO_IMSS_1289/2021	EPI_ISL_2942443	OL461617	B.1.1.7	GRY	32	C913T,C3037T,C3267T,C5388A,C5986T,T6954C,C9451T,C14408T,C14676T,C15279T,T16176C,T16905C,A23063T,C23271A,A23403G,C23604A,C23709T,T24506G,G24914C,G25690T,C27849T,C27972T,G28048T,A28111G,G28280C,A28281T,T28282A,G28396T,G28881A,G28882A,G28883C	21	N:D3L,N:R203K,N:G204R,N:S235F,ORF1a:T1001I,ORF1a:A1708D,ORF1a:I2230T,ORF1b:P314L,ORF3a:G100C,ORF7b:L32F,ORF8:Q27*,ORF8:R52I,ORF8:Y73C,ORF9b:G38V,S:N501Y,S:A570D,S:D614G,S:P681H,S:T716I,S:S982A,S:D1118H	6	147
hCoV-19/Mexico/TAB_IBT_IMSS_10132-NC/2021	EPI_ISL_2681374	OL461618	B.1.1.7	GRY	39	C241T,C913T,C3037T,C3267T,C5388A,C5986T,T6954C,G7042T,A10195G,C10279T,C14120T,C14408T,C14676T,C15279T,T16176C,C16887T,G17427T,A20540C,T20542G,A23063T,C23271A,A23403G,C23604A,C23709T,T24506G,G24914C,G25052T,C27972T,G28048T,G28075T,A28095T,A28111G,G28280C,A28281T,T28282A,G28881A,G28882A,G28883C,C28977T	25	N:D3L,N:R203K,N:G204R,N:S235F,ORF1a:T1001I,ORF1a:A1708D,ORF1a:I2230T,ORF1a:M2259I,ORF1b:P218L,ORF1b:P314L,ORF1b:K2358T,ORF1b:S2359A,ORF8:Q27*,ORF8:R52I,ORF8:C61F,ORF8:K68*,ORF8:Y73C,S:N501Y,S:A570D,S:D614G,S:P681H,S:T716I,S:S982A,S:D1118H,S:V1164F	6	1132
hCoV-19/Mexico/TAB_IBT_IMSS_1351/2021	EPI_ISL_2391728	OL461619	B.1.1.7	GRY	32	C241T,C913T,C2453T,C3037T,C3267T,C5388A,C5986T,T6954C,C9611T,C14408T,C14676T,C15279T,T16176C,C21855T,A23063T,C23271A,A23403G,C23604A,C23709T,T24506G,G24914C,C27972T,G28048T,A28111G,G28280C,A28281T,T28282A,A28295G,G28881A,G28882A,G28883C,C	22	N:D3L,N:N8D,N:R203K,N:G204R,N:S235F,ORF1a:L730F,ORF1a:T1001I,ORF1a:A1708D,ORF1a:I2230T,ORF1a:L3116F,ORF1b:P314L,ORF8:Q27*,ORF8:R52I,ORF8:Y73C,S:S98F,S:N501Y,S:A570D,S:D614G,S:P681H,S:T716I,S:S982A,S:D111	6	706
hCoV-19/Mexico/TAB_INER_IMSS_1105/2021	EPI_ISL_2490343	OL461620	B.1.1.7	GRY	37	C241T,C913T,C2110T,C3037T,C3267T,C5388A,C5986T,T6954C,G7042T,A10195G,C10279T,C14120T,C14408T,C14676T,C15279T,T16176C,C16887T,G17427T,A23063T,C23271A,A23403G,C23604A,C23709T,T24506G,G24914C,C27972T,G28048T,G28075T,A28095T,A28111G,G28280C,A28281T,T28282A,G28881A,G28882A,G28883C,C28977T	22	N:D3L,N:R203K,N:G204R,N:S235F,ORF1a:T1001I,ORF1a:A1708D,ORF1a:I2230T,ORF1a:M2259I,ORF1b:P218L,ORF1b:P314L,ORF8:Q27*,ORF8:R52I,ORF8:C61F,ORF8:K68*,ORF8:Y73C,S:N501Y,S:A570D,S:D614G,S:P681H,S:T716I,S:S982A,S:D1118H	6	0
hCoV-19/Mexico/TAB_INER_IMSS_1124/2021	EPI_ISL_2490346	OL461621	B.1.1.7	GRY	40	C241T,C865T,C913T,C2110T,C3037T,C3267T,C5388A,C5986T,T6954C,G7042T,A10195G,G10201T,C10279T,C14120T,C14408T,C14676T,C15279T,T16176C,C16887T,G17427T,G21724A,A23063T,C23271A,A23403G,C23604A,C23709T,T24506G,G24914C,C27972T,G28048T,G28075T,A28095T,A28111G,G28280C,A28281T,T28282A,G28881A,G28882A,G28883C,C28977T	23	N:D3L,N:R203K,N:G204R,N:S235F,ORF1a:T1001I,ORF1a:A1708D,ORF1a:I2230T,ORF1a:M2259I,ORF1a:M3312I,ORF1b:P218L,ORF1b:P314L,ORF8:Q27*,ORF8:R52I,ORF8:C61F,ORF8:K68*,ORF8:Y73C,S:N501Y,S:A570D,S:D614G,S:P681H,S:T716I,S:S982A,S:D1118H	6	0
hCoV-19/Mexico/TAB_LANGEBIO_IMSS_0647/2021	EPI_ISL_2402064	OL461622	B.1.1.7	GRY	38	C222T,C241T,C913T,C2110T,C3037T,C3267T,C5388A,C5986T,T6954C,G7042T,C14120T,C14408T,C14676T,C15279T,T16176C,C17762T,A23063T,C23271A,A23403G,C23604A,C23709T,T24506G,G24914C,T25180C,C25844T,C26270T,G27589T,C27972T,G28048T,A28095T,A28111G,G28280C,A28281T,T28282A,G28881A,G28882A,G28883C,C28977T	25	E:T9I,N:D3L,N:R203K,N:G204R,N:S235F,ORF1a:T1001I,ORF1a:A1708D,ORF1a:I2230T,ORF1a:M2259I,ORF1b:P218L,ORF1b:P314L,ORF1b:A1432V,ORF3a:T151I,ORF7a:A66S,ORF8:Q27*,ORF8:R52I,ORF8:K68*,ORF8:Y73C,S:N501Y,S:A570D,S:D614G,S:P681H,S:T716I,S:S982A,S:D1118H	6	164
hCoV-19/Mexico/TAB_LANGEBIO_IMSS_09609-NC/2021	EPI_ISL_2970045	OL461623	B.1.1.7	GR	37	C913T,C3037T,C3267T,G5012T,C5388A,C5986T,T6954C,C9693T,C11572T,C14408T,C14676T,C15279T,T16176C,G18651A,T23042C,A23063T,C23271A,A23403G,C23604A,C23709T,T24506G,G24914C,G25218T,C25553T,G25660C,C27615T,C27972T,G28048T,A28095T,A28111G,G28280C,A28281T,T28282A,G28881A,G28882A,G28883C,C28977T	25	N:D3L,N:R203K,N:G204R,N:S235F,ORF1a:T1001I,ORF1a:V1583F,ORF1a:A1708D,ORF1a:I2230T,ORF1a:A3143V,ORF1b:P314L,ORF3a:A54V,ORF3a:V90L,ORF8:Q27*,ORF8:R52I,ORF8:K68*,ORF8:Y73C,S:S494P,S:N501Y,S:A570D,S:D614G,S:P681H,S:T716I,S:S982A,S:D1118H,S:G1219V	5	1011
hCoV-19/Mexico/TAB-LANGEBIO_IMSS_0911/2021	EPI_ISL_2671705	OL461624	B.1.1.7	GRY	37	C913T,C2110T,C3037T,C3267T,C4113T,C5388A,C5986T,T6954C,C9430T,A10195G,C10279T,C14120T,C14408T,C14676T,C15279T,T16176C,C16887T,T16944C,G17427T,A23063T,C23271A,A23403G,C23604A,C23709T,T24506G,G24914C,C27972T,G28048T,G28075T,A28111G,G28280C,A28281T,T28282A,G28881A,G28882A,G28883C,C28977T	21	N:D3L,N:R203K,N:G204R,N:S235F,ORF1a:T1001I,ORF1a:A1283V,ORF1a:A1708D,ORF1a:I2230T,ORF1b:P218L,ORF1b:P314L,ORF8:Q27*,ORF8:R52I,ORF8:C61F,ORF8:Y73C,S:N501Y,S:A570D,S:D614G,S:P681H,S:T716I,S:S982A,S:D1118H	6	385
hCoV-19/Mexico/TAB-LANGEBIO_IMSS_1519/2021	EPI_ISL_2942632	OL461625	B.1.1.7	GRY	39	C241T,C913T,C2110T,C3037T,C3267T,C5388A,C5986T,T6954C,G7042T,A10195G,C10279T,C14120T,C14408T,C14676T,C15279T,T16176C,C16887T,G17427T,G20467A,A23063T,C23271A,A23403G,C23604A,C23709T,T24506G,G24914C,C27972T,G28048T,G28075T,A28095T,A28111G,G28280C,A28281T,T28282A,G28881A,G28882A,G28883C,C28977T,C29774T	23	N:D3L,N:R203K,N:G204R,N:S235F,ORF1a:T1001I,ORF1a:A1708D,ORF1a:I2230T,ORF1a:M2259I,ORF1b:P218L,ORF1b:P314L,ORF1b:A2334T,ORF8:Q27*,ORF8:R52I,ORF8:C61F,ORF8:K68*,ORF8:Y73C,S:N501Y,S:A570D,S:D614G,S:P681H,S:T716I,S:S982A,S:D1118H	6	0
hCoV-19/Mexico/TAB-LANGEBIO_IMSS_1539/2021	EPI_ISL_2942650	OL461626	B.1.1.7	GRY	38	C241T,C913T,C2110T,C3037T,C3267T,G4504T,C5388A,C5986T,T6954C,A10195G,C10279T,C14120T,C14408T,C14676T,C15279T,T16176C,C16887T,G17427T,A23063T,C23271A,A23403G,C23604A,C23709T,T24506G,G24914C,C27972T,G28048T,G28075T,A28095T,A28111G,G28280C,A28281T,T28282A,G28881A,G28882A,G28883C,C28977T,C29149T	22	N:D3L,N:R203K,N:G204R,N:S235F,ORF1a:T1001I,ORF1a:E1413D,ORF1a:A1708D,ORF1a:I2230T,ORF1b:P218L,ORF1b:P314L,ORF8:Q27*,ORF8:R52I,ORF8:C61F,ORF8:K68*,ORF8:Y73C,S:N501Y,S:A570D,S:D614G,S:P681H,S:T716I,S:S982A,S:D1118H	6	1
hCoV-19/Mexico/TAM_IBT_IMSS_1373/2021	EPI_ISL_2391732	OL461627	B.1.1.7	GRY	38	C241T,C913T,C3037T,C3267T,C5388A,C5986T,T6954C,G7042T,A10195G,C12534T,C14120T,C14408T,C14676T,C15279T,C15933T,T16176C,A23063T,C23271A,A23403G,C23604A,C23709T,T24506G,G24914C,G25855T,C27649T,A27745G,C27972T,G28048T,G28075T,A28095T,A28111G,G28280C,A28281T,T28282A,G28881A,G28882A,G28883C,C28977T	25	N:D3L,N:R203K,N:G204R,N:S235F,ORF1a:T1001I,ORF1a:A1708D,ORF1a:I2230T,ORF1a:M2259I,ORF1a:T4090I,ORF1b:P218L,ORF1b:P314L,ORF3a:D155Y,ORF7a:R118G,ORF8:Q27*,ORF8:R52I,ORF8:C61F,ORF8:K68*,ORF8:Y73C,S:N501Y,S:A570D,S:D614G,S:P681H,S:T716I,S:S982A,S:D1118H	6	695
hCoV-19/Mexico/TAM_LANGEBIO_IMSS_0049/2021	EPI_ISL_1351540	OL461628	B.1.1.7	GRY	36	C241T,C292T,G872T,C913T,C3037T,C3267T,C4226T,C5388A,C5628T,C5986T,T6954C,C11750T,C14408T,C14676T,C15279T,T16176C,A23063T,C23170T,C23271A,A23403G,C23604A,C23709T,T24506G,G24914C,C25626T,C27972T,G28048T,A28111G,G28280C,A28281T,T28282A,A28295C,G28881A,G28882A,G28883C,C28977T	24	N:D3L,N:N8H,N:R203K,N:G204R,N:S235F,ORF1a:D203Y,ORF1a:T1001I,ORF1a:P1321S,ORF1a:A1708D,ORF1a:T1788M,ORF1a:I2230T,ORF1a:L3829F,ORF1b:P314L,ORF8:Q27*,ORF8:R52I,ORF8:Y73C,ORF9b:K4N,S:N501Y,S:A570D,S:D614G,S:P681H,S:T716I,S:S982A,S:D1118H	6	9

hCoV-19/Mexico/TAM_LANGEBIO_IMSS_38905-NC/2021	EPI_ISL_2970048	OL461629	B.1.1.7	GR	33	C913T,C3037T,C3267T,C5388A,C5986T,T6954C,T12645C,C14408T,C14676T,G14707A,C15279T,T16176C,T16563A,C23170T,C23271A,A23403G,C23604A,C23709T,T24506G,G24914C,C25626T,C27972T,G28048T,A28111G,G28280C,A28281T,T28282A,A28295C,G28395T,G28881A,G28882A,G28883C,C28977T	23	N:D3L,N:N8H,N:R41L,N:R203K,N:G204R,N:S235F,ORF1a:T1001I,ORF1a:A1708D,ORF1a:I2230T,ORF1a:I4127T,ORF1b:P314L,ORF1b:A414T,ORF8:Q27*,ORF8:R52I,ORF8:Y73C,ORF9b:K4N,ORF9b:G38C,S:A570D,S:D614G,S:P681H,S:T716I,S:S982A,S:D1118H	6	999
hCoV-19/Mexico/TAM_LANGEBIO_IMSS_67638-NC/2021	EPI_ISL_2970051	OL461630	B.1.1.7	GRY	31	C913T,C3037T,C3267T,C3543T,C5388A,T6954C,G7042T,A10195G,C10341T,C14120T,C14408T,C14676T,C15126T,C15279T,T16176C,A23063T,C23271A,A23403G,C23604A,C23709T,T24506G,G24914C,C26645T,C27972T,G28048T,G28075T,C28603T,G28881A,G28882A,G28883C,C28977T	21	N:R203K,N:G204R,N:S235F,ORF1a:T1001I,ORF1a:T1093I,ORF1a:A1708D,ORF1a:I2230T,ORF1a:M2259I,ORF1a:P3359L,ORF1b:P218L,ORF1b:P314L,ORF8:Q27*,ORF8:R52I,ORF8:C61F,S:N501Y,S:A570D,S:D614G,S:P681H,S:T716I,S:S982	6	1126
hCoV-19/Mexico/TAM-IBT_IMSS_1792/2021	EPI_ISL_2801673	OL461631	B.1.1.7	GRY	42	C241T,C913T,C2110T,C3037T,C3267T,C5388A,C5986T,T6954C,G7042T,A10195G,C10834T,G10870T,C14120T,C14408T,C14676T,C15279T,C15933T,T16176C,C16869T,A23063T,C23271A,A23403G,C23604A,C23709T,T24506G,G24914C,G26951T,A27745G,C27972T,C28045T,G28048T,G28075T,A28095T,A28111G,G28280C,A28281T,T28282A,G28881A,G28882A,G28883C,C28977T,G29	24	N:D3L,N:R203K,N:G204R,N:S235F,ORF1a:T1001I,ORF1a:A1708D,ORF1a:I2230T,ORF1a:M2259I,ORF1b:P218L,ORF1b:P314L,ORF7a:R118G,ORF8:Q27*,ORF8:A51V,ORF8:R52I,ORF8:C61F,ORF8:K68*,ORF8:Y73C,S:N501Y,S:A570D,S:D614G,S:P681H,S:T716I,S:S982A,S:D1118H	6	0
hCoV-19/Mexico/TAM-LANGEBIO_IMSS_0849/2021	EPI_ISL_2671717	OL461632	B.1.1.7	GRY	40	C241T,C913T,C2110T,C3037T,C3267T,C5388A,C5986T,T6954C,G7042T,A10195G,C10834T,G10870T,C14120T,C14408T,C14676T,C15279T,C15933T,T16176C,C16869T,A23063T,C23271A,A23403G,C23604A,C23709T,T24506G,G24914C,A27745G,C27972T,G28048T,G28075T,A28095T,A28111G,G28280C,A28281T,T28282A,G28881A,G28882A,G28883C,C28977T,G29422C	23	N:D3L,N:R203K,N:G204R,N:S235F,ORF1a:T1001I,ORF1a:A1708D,ORF1a:I2230T,ORF1a:M2259I,ORF1b:P218L,ORF1b:P314L,ORF7a:R118G,ORF8:Q27*,ORF8:R52I,ORF8:C61F,ORF8:K68*,ORF8:Y73C,S:N501Y,S:A570D,S:D614G,S:P681H,S:T716I,S:S982A,S:D1118H	6	1
hCoV-19/Mexico/TAM-LANGEBIO_IMSS_0854/2021	EPI_ISL_2671718	OL461633	B.1.1.7	GRY	40	C913T,C2110T,C3037T,C3267T,C5388A,C5986T,T6954C,G7042T,A10195G,C10834T,G10870T,C14120T,C14408T,C14676T,C15279T,C15933T,T16176C,C16869T,C21691T,A23063T,C23271A,A23403G,C23604A,C23709T,T24506G,G24914C,A27745G,C27972T,G28048T,G28075T,A28095T,A28111G,G28280C,A28281T,T28282A,G28881A,G28882A,G28883C,C28977T,G29422C	23	N:D3L,N:R203K,N:G204R,N:S235F,ORF1a:T1001I,ORF1a:A1708D,ORF1a:I2230T,ORF1a:M2259I,ORF1b:P218L,ORF1b:P314L,ORF7a:R118G,ORF8:Q27*,ORF8:R52I,ORF8:C61F,ORF8:K68*,ORF8:Y73C,S:N501Y,S:A570D,S:D614G,S:P681H,S:T716I,S:S982A,S:D1118H	6	31
hCoV-19/Mexico/TAM-LANGEBIO_IMSS_0884/2021	EPI_ISL_2671722	OL461634	B.1.1.7	GRY	38	C241T,C913T,C2110T,C3037T,C3267T,C5388A,C5986T,T6954C,G7042T,A10195G,C14120T,C14408T,C14676T,C15279T,C15933T,T16176C,C22450T,A23063T,C23271A,A23403G,C23604A,C23709T,T24506G,G24620T,G24914C,A27745G,C27972T,G28048T,G28075T,A28095T,A28111G,G28280C,A28281T,T28282A,G28881A,G28882A,G28883C,C28977T	24	N:D3L,N:R203K,N:G204R,N:S235F,ORF1a:T1001I,ORF1a:A1708D,ORF1a:I2230T,ORF1a:M2259I,ORF1b:P218L,ORF1b:P314L,ORF7a:R118G,ORF8:Q27*,ORF8:R52I,ORF8:C61F,ORF8:K68*,ORF8:Y73C,S:N501Y,S:A570D,S:D614G,S:P681H,S:T716I,S:S982A,S:A1020S,S:D1118H	7	0
hCoV-19/Mexico/TAM-LANGEBIO_IMSS_1368/2021	EPI_ISL_2942498	OL461635	B.1.1.7	GRY	39	C110T,C241T,C913T,C2110T,C3037T,C3267T,C3543T,C5388A,C5986T,T6954C,G7042T,A10195G,C10341T,C14120T,C14408T,C14676T,C15126T,C15279T,T16176C,A23063T,C23271A,A23403G,C23604A,C23709T,T24506G,G24914C,C26645T,C27972T,G28048T,G28075T,A28111G,G28280C,A28281T,T28282A,C28603T,G28881A,G28882A,G28883C,C28977T	23	N:D3L,N:R203K,N:G204R,N:S235F,ORF1a:T1001I,ORF1a:T1093I,ORF1a:A1708D,ORF1a:I2230T,ORF1a:M2259I,ORF1a:P3359L,ORF1b:P218L,ORF1b:P314L,ORF8:Q27*,ORF8:R52I,ORF8:C61F,ORF8:Y73C,S:N501Y,S:A570D,S:D614G,S:P681H,S:T716I,S:S982A,S:D1118H	6	1
hCoV-19/Mexico/TAM-LANGEBIO_IMSS_1375/2021	EPI_ISL_2942504	OL461636	B.1.1.7	GRY	33	C913T,C3037T,C3267T,C5388A,C5986T,T6954C,C14408T,C14676T,C15279T,T16176C,T16563A,T22016C,T22861C,A23063T,C23170T,C23271A,A23403G,C23604A,C23709T,T24506G,G24914C,C25626T,C27972T,G28048T,A28111G,G28280C,A28281T,T28282A,A28295C,G28881A,G28882	21	N:D3L,N:N8H,N:R203K,N:G204R,N:S235F,ORF1a:T1001I,ORF1a:A1708D,ORF1a:I2230T,ORF1b:P314L,ORF8:Q27*,ORF8:R52I,ORF8:Y73C,ORF9b:K4N,S:W152R,S:N501Y,S:A570D,S:D614G,S:P681H,S:T716I,S:S982A,S:D1118H	6	221
hCoV-19/Mexico/TAM-LANGEBIO_IMSS_1671/2021	EPI_ISL_2942764	OL461637	B.1.1.7	GRY	37	C241T,C913T,C3037T,C3267T,C5388A,C5986T,T6954C,G7042T,A10195G,C14120T,C14408T,C14676T,C15279T,C15933T,T16176C,A23063T,C23271A,A23403G,C23604A,C23709T,T24506G,G24914C,C26110T,A27745G,C27972T,G28048T,G28075T,A28095T,A28111G,G28280C,A28281T,T28282A,G28487A,G28881A,G28882A,G28883C,C28977T	25	N:D3L,N:V72I,N:R203K,N:G204R,N:S235F,ORF1a:T1001I,ORF1a:A1708D,ORF1a:I2230T,ORF1a:M2259I,ORF1b:P218L,ORF1b:P314L,ORF3a:P240S,ORF7a:R118G,ORF8:Q27*,ORF8:R52I,ORF8:C61F,ORF8:K68*,ORF8:Y73C,S:N501Y,S:A570D,S:D614G,S:P681H,S:T716I,S:S982A,S:D1118H	6	64
hCoV-19/Mexico/TLA-LANGEBIO_IMSS_1065/2021	EPI_ISL_2671723	OL461638	B.1.1.7	GRY	38	T201A,C241T,C913T,C2110T,C3037T,C3267T,C3777T,C5388A,C5986T,T6954C,G7042T,A10195G,C14120T,C14408T,C14676T,C15279T,T16176C,A23063T,C23271A,A23403G,C23604A,C23709T,T24506G,G24914C,T25612G,C27972T,G28048T,G28075T,A28095T,A28111G,G28280C,A28281T,T28282A,C28651T,G28881A,G28882A,G28883C,C28977T	24	N:D3L,N:R203K,N:G204R,N:S235F,ORF1a:T1001I,ORF1a:T1171I,ORF1a:A1708D,ORF1a:I2230T,ORF1a:M2259I,ORF1b:P218L,ORF1b:P314L,ORF3a:S74A,ORF8:Q27*,ORF8:R52I,ORF8:C61F,ORF8:K68*,ORF8:Y73C,S:N501Y,S:A570D,S:D614G,S:P681H,S:T716I,S:S982A,S:D1118H	6	76
hCoV-19/Mexico/VER_IBT_IMSS_1010/2021	EPI_ISL_1811518	OL461639	B.1.1.7	GRY	37	C241T,C913T,C3037T,C3267T,C5388A,C5986T,T6954C,C7299T,A13768T,C14408T,C14676T,T15096C,C15279T,T16176C,A23063T,C23271A,A23403G,C23604A,C23709T,T24506G,G24914C,G25340T,C26606T,G27506T,C27972T,G28048T,A28095T,A28111G,G28280C,A28281T,T28282A,A28877T,G28878C,G28881A,G28882A,G28883C,C28977T	23	N:D3L,N:R203K,N:G204R,N:S235F,ORF1a:T1001I,ORF1a:A1708D,ORF1a:I2230T,ORF1a:A2345V,ORF1b:M101L,ORF1b:P314L,ORF7a:G38V,ORF8:Q27*,ORF8:R52I,ORF8:K68*,ORF8:Y73C,S:N501Y,S:A570D,S:D614G,S:P681H,S:T716I,S:S982A,S:D1118H,S:D1260Y	6	252
hCoV-19/Mexico/VER_IBT_IMSS_1146/2021	EPI_ISL_2391570	OL461640	B.1.1.7	GRY	38	C241T,C292T,C913T,C1060T,C3037T,C3267T,C5388A,C5986T,T6954C,C14408T,C14676T,C15279T,T16176C,T16563A,A23063T,C23170T,C23271A,A23403G,C23604A,C23709T,T24506G,G24914C,C25096T,C25626T,C26131T,C27972T,G28048T,A28111G,G28280C,A28281T,T28282A,A28295C,C28728T,G28881A,G28882A,G28883C,C28977T,T29377C	22	N:D3L,N:N8H,N:A152V,N:R203K,N:G204R,N:S235F,ORF1a:T1001I,ORF1a:A1708D,ORF1a:I2230T,ORF1b:P314L,ORF3a:H247Y,ORF8:Q27*,ORF8:R52I,ORF8:Y73C,ORF9b:K4N,S:N501Y,S:A570D,S:D614G,S:P681H,S:T716I,S:S982A,S:D1118H	6	0
hCoV-19/Mexico/VER_IBT_IMSS_1214/2021	EPI_ISL_2391651	OL461641	B.1.1.7	GRY	34	C241T,C913T,C2453T,C3037T,C3267T,C5388A,C5986T,T6954C,C9611T,C14408T,C14676T,C15279T,T16176C,C21855T,A23063T,C23271A,A23403G,C23604A,C23709T,T24506G,G24914C,C27643T,C27972T,G28048T,A28111G,G28280C,A28281T,T28282A,A28295G,G28881A,G28882A,G28883C,C28977T,G29692T	23	N:D3L,N:N8D,N:R203K,N:G204R,N:S235F,ORF1a:L730F,ORF1a:T1001I,ORF1a:A1708D,ORF1a:I2230T,ORF1a:L3116F,ORF1b:P314L,ORF7a:P84S,ORF8:Q27*,ORF8:R52I,ORF8:Y73C,S:S98F,S:N501Y,S:A570D,S:D614G,S:P681H,S:T716I,S:S982A,S:D1118H	6	332
hCoV-19/Mexico/VER_IBT_IMSS_1326/2021	EPI_ISL_2391668	OL461642	B.1.1.7	GRY	35	C241T,C913T,C3037T,C3267T,C5388A,C5986T,T6954C,C14408T,C14676T,T15096C,C15279T,T16176C,A23063T,C23271A,A23403G,C23604A,C23709T,G24089A,T24506G,G24914C,G25785T,C27476T,C27972T,G28048T,A28095T,A28111G,G28280C,A28281T,T28282A,G28881A,G28882A,G28883C,C28977T,C29642T,T29760C	22	N:D3L,N:R203K,N:G204R,N:S235F,ORF1a:T1001I,ORF1a:A1708D,ORF1a:I2230T,ORF1b:P314L,ORF3a:W131C,ORF7a:T28I,ORF8:Q27*,ORF8:R52I,ORF8:K68*,ORF8:Y73C,S:N501Y,S:A570D,S:D614G,S:P681H,S:T716I,S:D843N,S:S982A,S:D1118H	6	429
hCoV-19/Mexico/VER_IBT_IMSS_1330/2021	EPI_ISL_2391554	OL461643	B.1.1.7	GRY	43	C241T,C913T,C3037T,C3267T,C5388A,C5944T,C5986T,G6647A,T6954C,C14408T,C14676T,T15096C,C15279T,T16176C,C17676T,C18183T,C19524T,A21519C,G22104T,A23063T,C23271A,A23403G,G23429T,C23604A,C23709T,A24070T,A24092G,T24506G,G24914C,C25624T,C27737T,C27972T,G28048T,A28095T,A28111G,G28280C,A28281T,T28282A,G28881A,G28882A,G28883C,G	27	N:D3L,N:R203K,N:G204P,N:S235F,ORF1a:T1001I,ORF1a:A1708D,ORF1a:A2128T,ORF1a:I2230T,ORF1b:P314L,ORF1b:R2684S,ORF3a:H78Y,ORF7a:T115I,ORF8:Q27*,ORF8:R52I,ORF8:K68*,ORF8:Y73C,S:G181V,S:N501Y,S:A570D,S:D614G,S:A623S,S:P681H,S:T716I,S:Q836H,S:I844V,S:S982A,S:D1118H	6	0
hCoV-19/Mexico/VER_IBT_IMSS_1331/2021	EPI_ISL_2391555	OL461644	B.1.1.7	GRY	42	C241T,C913T,C3037T,C3267T,C5388A,C5944T,C5986T,G6647A,T6954C,C14408T,C14676T,T15096C,C15279T,T16176C,C17676T,C18183T,C19524T,A21519C,A23063T,C23271A,A23403G,G23429T,C23604A,C23709T,A24070T,A24092G,T24506G,G24914C,C25624T,C27737T,C27972T,G28048T,A28095T,A28111G,G28280C,A28281T,T28282A,G28881A,G28882A,G28883C,G28884C,C	26	N:D3L,N:R203K,N:G204P,N:S235F,ORF1a:T1001I,ORF1a:A1708D,ORF1a:A2128T,ORF1a:I2230T,ORF1b:P314L,ORF1b:R2684S,ORF3a:H78Y,ORF7a:T115I,ORF8:Q27*,ORF8:R52I,ORF8:K68*,ORF8:Y73C,S:N501Y,S:A570D,S:D614G,S:A623S,S:P681H,S:T716I,S:Q836H,S:I844V,S:S982A,S:D1118H	6	0
hCoV-19/Mexico/VER_IBT_IMSS_1333/2021	EPI_ISL_2391694	OL461645	B.1.1.7	GRY	34	C241T,C913T,C2453T,C3037T,C3267T,C5388A,C5986T,T6954C,C9611T,C14408T,C14676T,C15279T,T16176C,G18651T,C21855T,G21974C,A23063T,C23271A,A23403G,C23604A,C23709T,T24506G,G24914C,C27972T,G28048T,A28111G,G28280C,A28281T,T28282A,A28295G,G28881A,G28882A,G28883C,C28977T	24	N:D3L,N:N8D,N:R203K,N:G204R,N:S235F,ORF1a:L730F,ORF1a:T1001I,ORF1a:A1708D,ORF1a:I2230T,ORF1a:L3116F,ORF1b:P314L,ORF1b:E1728D,ORF8:Q27*,ORF8:R52I,ORF8:Y73C,S:S98F,S:D138H,S:N501Y,S:A570D,S:D614G,S:P681H,S:T716I,S:S982A,S:D1118H	6	564
hCoV-19/Mexico/VER_IBT_IMSS_1650/2021	EPI_ISL_2681350	OL461646	B.1.1.7	GRY	38	C241T,C913T,C1594T,A2317T,C2453T,C3037T,C3267T,C5388A,C5986T,C6433T,T6954C,C9611T,C14408T,C14676T,C15279T,T16176C,C21008T,C21855T,G21974C,A23063T,C23271A,A23403G,C23604A,C23709T,T24506G,G24914C,C27643T,C27972T,G28048T,A28111G,G28280C,A28281T,T28282A,A28295G,G28881A,G28882A,G28883C,C28977T	26	N:D3L,N:N8D,N:R203K,N:G204R,N:S235F,ORF1a:K684N,ORF1a:L730F,ORF1a:T1001I,ORF1a:A1708D,ORF1a:I2230T,ORF1a:L3116F,ORF1b:P314L,ORF1b:T2514I,ORF7a:P84S,ORF8:Q27*,ORF8:R52I,ORF8:Y73C,S:S98F,S:D138H,S:N501Y,S:A570D,S:D614G,S:P681H,S:T716I,S:S982A,S:D1118H	6	705
hCoV-19/Mexico/VER_IBT_IMSS_1663/2021	EPI_ISL_2681147	OL461647	B.1.1.7	GRY	37	C241T,C913T,C2453T,C3037T,C3267T,C3695T,C5388A,C5986T,T6954C,C9611T,C9693T,C14408T,C14676T,C15279T,T16176C,C21008T,C21855T,G21974C,A23063T,C23271A,A23403G,C23604A,C23709T,T24506G,G24914C,C27643T,C27972T,G28048T,A28111G,G28280C,A28281T,T28282A,A28295G,G28881A,G28882A,G28883C,C28977T	26	N:D3L,N:N8D,N:R203K,N:G204R,N:S235F,ORF1a:L730F,ORF1a:T1001I,ORF1a:A1708D,ORF1a:I2230T,ORF1a:L3116F,ORF1a:A3143V,ORF1b:P314L,ORF1b:T2514I,ORF7a:P84S,ORF8:Q27*,ORF8:R52I,ORF8:Y73C,S:S98F,S:D138H,S:N501Y,S:A570D,S:D614G,S:P681H,S:T716I,S:S982A,S:D1118H	6	0

hCoV-19/Mexico/VER_IBT_IMSS_1664/2021	EPI_ISL_2681180	OL461648	B.1.1.7	GRY	40	C241T,C913T,C2110T,G3004T,C3037T,C3267T,C5388A,C5986T,T6954C,G7042T,C7609T,C10525T,C10789T,C14120T,C14408T,C14676T,C15279T,T16176C,A23063T,C23271A,A23403G,G23593T,C23604A,C23709T,T24506G,G24914C,C26110T,C26735T,C27972T,G28048T,A28095T,A28111G,G28280C,A28281T,T28282A,A28804G,G28881A,G28882A,G28883C,C28977T	24	N:D3L,N:R203K,N:G204R,N:S235F,ORF1a:E913D,ORF1a:T1001I,ORF1a:A1708D,ORF1a:I2230T,ORF1a:M2259I,ORF1b:P218L,ORF1b:P314L,ORF3a:P240S,ORF8:Q27*,ORF8:R52I,ORF8:K68*,ORF8:Y73C,S:N501Y,S:A570D,S:D614G,S:Q677H,S:P681H,S:T716I,S:S982A,S:D1118H	6	0
hCoV-19/Mexico/VER_INER_IMSS_00966/2021	EPI_ISL_2091407	OL461649	B.1.1.7	GRY	34	C241T,C913T,C2453T,C3037T,C3267T,C5388A,C5986T,T6954C,C9611T,C14408T,C14676T,C15279T,T16176C,C21855T,G21974C,A23063T,C23271A,A23403G,C23604A,C23709T,T24506G,G24914C,C27643T,C27972T,G28048T,A28111G,G28280C,A28281T,T28282A,A28295G,G28881A,G28882A,G28883C,C28977T	24	N:D3L,N:N8D,N:R203K,N:G204R,N:S235F,ORF1a:L730F,ORF1a:T1001I,ORF1a:A1708D,ORF1a:I2230T,ORF1a:L3116F,ORF1b:P314L,ORF7a:P84S,ORF8:Q27*,ORF8:R52I,ORF8:Y73C,S:S98F,S:D138H,S:N501Y,S:A570D,S:D614G,S:P681H,S:T716I,S:S982A,S:D1118H	6	0
hCoV-19/Mexico/VER_LANGEBIO_IMSS_0428/2021	EPI_ISL_1662210	OL461650	B.1.1.7	GRY	34	C241T,C913T,C1572T,C3037T,C3267T,C5388A,C5986T,C6196T,T6954C,A8848G,C14408T,C14676T,C15279T,T16176C,A23063T,C23271A,A23403G,C23604A,C23709T,T24506G,G24914C,G25217T,G26727T,C27972T,G28048T,A28111G,G28280C,A28281T,T28282A,G28881A,G28882A,G28	21	M:A69S,N:D3L,N:R203K,N:G204R,N:S235F,ORF1a:T436I,ORF1a:T1001I,ORF1a:A1708D,ORF1a:I2230T,ORF1b:P314L,ORF8:Q27*,ORF8:R52I,ORF8:Y73C,S:N501Y,S:A570D,S:D614G,S:P681H,S:T716I,S:S982A,S:D1118H,S:G1219C	6	0
hCoV-19/Mexico/VER_LANGEBIO_IMSS_11722-NC/2021	EPI_ISL_2970055	OL461651	B.1.1.7	GRY	39	C241T,C913T,G1730A,C2453T,C3037T,C3267T,C5388A,C5986T,T6954C,G7675T,C9611T,C14408T,C14676T,C15279T,T16176C,C18570T,C21855T,G21974C,A23063T,C23271A,A23403G,C23604A,C23709T,T24506G,G24914C,C25609T,A27377C,C27972T,G28048T,A28111G,G28280C,A28281T,T28282A,A28295G,G28881A,G28882A,G28883C,C28977T,G29041T	27	N:D3L,N:N8D,N:R203K,N:G204R,N:S235F,N:K256N,ORF1a:E489K,ORF1a:L730F,ORF1a:T1001I,ORF1a:A1708D,ORF1a:I2230T,ORF1a:L3116F,ORF1b:P314L,ORF3a:L73F,ORF6:E59A,ORF8:Q27*,ORF8:R52I,ORF8:Y73C,S:S98F,S:D138H,S:N501Y,S:A570D,S:D614G,S:P681H,S:T716I,S:S982A,S:D1118H	6	1047
hCoV-19/Mexico/VER-IBT_IMSS_1892/2021	EPI_ISL_2801760	OL461652	B.1.1.7	GRY	36	C241T,C913T,C3037T,C3267T,G4904T,C5388A,C5986T,T6954C,G7042T,A10195G,C14120T,C14408T,C14676T,C15279T,C15933T,T16176C,A23063T,C23271A,A23403G,C23604A,C23709T,T24506G,G24914C,A27745G,C27972T,G28048T,G28075T,A28095T,A28111G,G28280C,A28281T,T28282A,G28881A,G28882A,G28883C,C28977T	24	N:D3L,N:R203K,N:G204R,N:S235F,ORF1a:T1001I,ORF1a:D1547Y,ORF1a:A1708D,ORF1a:I2230T,ORF1a:M2259I,ORF1b:P218L,ORF1b:P314L,ORF7a:R118G,ORF8:Q27*,ORF8:R52I,ORF8:C61F,ORF8:K68*,ORF8:Y73C,S:N501Y,S:A570D,S:D614G,S:P681H,S:T716I,S:S982A,S:D1118H	6	789
hCoV-19/Mexico/VER-IBT_IMSS_1923/2021	EPI_ISL_2801788	OL461653	B.1.1.7	GRY	42	C203T,C241T,C913T,G1820A,C3037T,C3267T,G5023T,C5388A,G5924A,C5986T,T6954C,G9526T,A12162G,C14408T,C14676T,C15279T,C15720T,T16176C,A17615G,T19363C,A21137G,A23063T,C23248T,C23271A,A23403G,C23604A,C23709T,T24506G,C24865T,G24914C,G25135T,G27670T,C27972T,G28048T,A28111G,G28280C,A28281T,T28282A,G28881A,G28882A,G28883C,C2897	27	N:D3L,N:R203K,N:G204R,N:S235F,ORF1a:G519S,ORF1a:T1001I,ORF1a:M1586I,ORF1a:A1708D,ORF1a:V1887I,ORF1a:I2230T,ORF1a:M3087I,ORF1a:Q3966R,ORF1b:P314L,ORF1b:K1383R,ORF1b:K2557R,ORF7a:V93F,ORF8:Q27*,ORF8:R52I,ORF8:Y73C,S:N501Y,S:A570D,S:D614G,S:P681H,S:T716I,S:S982A,S:D1118	6	716
hCoV-19/Mexico/VER-IBT_IMSS_1983/2021	EPI_ISL_2801841	OL461654	B.1.1.7	GRY	38	C241T,C913T,A2317T,C2453T,C3037T,C3267T,C5388A,C5986T,C6433T,T6954C,C9611T,C11750T,C14408T,C14676T,C15279T,T16176C,C21008T,C21855T,G21974C,A23063T,C23271A,A23403G,C23604A,C23709T,T24506G,G24914C,C27643T,C27972T,G28048T,A28111G,G28280C,A28281T,T28282A,A28295G,G28881A,G28882A,G28883C,C28977T	27	N:D3L,N:N8D,N:R203K,N:G204R,N:S235F,ORF1a:K684N,ORF1a:L730F,ORF1a:T1001I,ORF1a:A1708D,ORF1a:I2230T,ORF1a:L3116F,ORF1a:L3829F,ORF1b:P314L,ORF1b:T2514I,ORF7a:P84S,ORF8:Q27*,ORF8:R52I,ORF8:Y73C,S:S98F,S:D138H,S:N501Y,S:A570D,S:D614G,S:P681H,S:T716I,S:S982A,S:D1118H	6	0
hCoV-19/Mexico/VER-IBT_IMSS_1990/2021	EPI_ISL_2801847	OL461655	B.1.1.7	GRY	40	C241T,C913T,A2317T,C2453T,C3037T,C3267T,C5388A,C5986T,C6433T,T6954C,C8386T,C9611T,C11235T,C11750T,C14408T,C14676T,C15279T,T16176C,C21008T,C21855T,G21974C,A23063T,C23271A,A23403G,C23604A,C23709T,T24506G,G24914C,C27643T,C27972T,G28048T,A28111G,G28280C,A28281T,T28282A,A28295G,G28881A,G28882A,G28883C,C28977T	28	N:D3L,N:N8D,N:R203K,N:G204R,N:S235F,ORF1a:K684N,ORF1a:L730F,ORF1a:T1001I,ORF1a:A1708D,ORF1a:I2230T,ORF1a:L3116F,ORF1a:A3657V,ORF1a:L3829F,ORF1b:P314L,ORF1b:T2514I,ORF7a:P84S,ORF8:Q27*,ORF8:R52I,ORF8:Y73C,S:S98F,S:D138H,S:N501Y,S:A570D,S:D614G,S:P681H,S:T716I,S:S982A,S:D	6	0
hCoV-19/Mexico/VER-IBT_IMSS_1998/2021	EPI_ISL_2801851	OL461656	B.1.1.7	GRY	36	C241T,C913T,C2453T,C3037T,C3267T,C5388A,C5986T,T6954C,C9611T,C14408T,C14676T,C15279T,T16176C,G17797A,C21855T,G21974C,A23063T,C23271A,A23403G,C23604A,C23709T,T24506G,G24914C,C25609T,G27516T,C27972T,G28048T,A28111G,G28280C,A28281T,T28282A,A28295G,G28881A,G28882A,G28883C,C28977T	26	N:D3L,N:N8D,N:R203K,N:G204R,N:S235F,ORF1a:L730F,ORF1a:T1001I,ORF1a:A1708D,ORF1a:I2230T,ORF1a:L3116F,ORF1b:P314L,ORF1b:V1444I,ORF3a:L73F,ORF7a:E41D,ORF8:Q27*,ORF8:R52I,ORF8:Y73C,S:S98F,S:D138H,S:N501Y,S:A570D,S:D614G,S:P681H,S:T716I,S:S982A,S:D1118H	6	101
hCoV-19/Mexico/VER-IBT_IMSS_2043/2021	EPI_ISL_2801887	OL461657	B.1.1.7	GRY	41	T154C,C241T,C913T,C3037T,C3267T,C5388A,C5944T,C5986T,T6954C,C7564T,C9967T,C13585T,C14408T,C14676T,T15096C,C15279T,T16176C,A18184G,C21614T,G21901A,C22987T,A23063T,C23271A,A23403G,C23604A,C23709T,T24506G,G24914C,C25509A,C27972T,G28048T,A28095T,A28111G,G28280C,A28281T,T28282A,G28881A,G28882A,G28883C,G28884C,C28977T	21	N:D3L,N:R203K,N:G204P,N:S235F,ORF1a:T1001I,ORF1a:A1708D,ORF1a:I2230T,ORF1b:P314L,ORF1b:M1573V,ORF8:Q27*,ORF8:R52I,ORF8:K68*,ORF8:Y73C,S:L18F,S:N501Y,S:A570D,S:D614G,S:P681H,S:T716I,S:S982A,S:D1118H	6	0
hCoV-19/Mexico/VER-LANGEBIO_IMSS_1103/2021	EPI_ISL_2671737	OL461658	B.1.1.7	GRY	34	C913T,C2453T,C3037T,C3267T,C5388A,C5986T,T6954C,C9611T,C14408T,C14676T,C15279T,T16176C,C21855T,G21974C,A23063T,C23271A,A23403G,C23604A,C23709T,T24506G,G24914C,C25609T,C27972T,G28048T,A28111G,G28280C,A28281T,T28282A,A28295G,G28881A,G28882A,G28883C,C28977T,G29081T	25	N:D3L,N:N8D,N:R203K,N:G204R,N:S235F,N:V270L,ORF1a:L730F,ORF1a:T1001I,ORF1a:A1708D,ORF1a:I2230T,ORF1a:L3116F,ORF1b:P314L,ORF3a:L73F,ORF8:Q27*,ORF8:R52I,ORF8:Y73C,S:S98F,S:D138H,S:N501Y,S:A570D,S:D614G,S:P681H,S:T716I,S:S982A,S:D1118H	6	1
hCoV-19/Mexico/VER-LANGEBIO_IMSS_1105/2021	EPI_ISL_2671739	OL461659	B.1.1.7	GRY	37	C241T,C913T,C2453T,C3037T,C3267T,C5388A,C5986T,C6433T,C6573T,T6954C,C9611T,C14408T,C14676T,C15279T,T16176C,C21008T,C21855T,G21974C,A23063T,C23271A,A23403G,C23604A,C23709T,T24506G,G24914C,C27643T,C27972T,G28048T,A28111G,G28280C,A28281T,T28282A,A28295G,G28881A,G28882A,G28883C,C28977T	26	N:D3L,N:N8D,N:R203K,N:G204R,N:S235F,ORF1a:L730F,ORF1a:T1001I,ORF1a:A1708D,ORF1a:S2103F,ORF1a:I2230T,ORF1a:L3116F,ORF1b:P314L,ORF1b:T2514I,ORF7a:P84S,ORF8:Q27*,ORF8:R52I,ORF8:Y73C,S:S98F,S:D138H,S:N501Y,S:A570D,S:D614G,S:P681H,S:T716I,S:S982A,S:D1118H	6	6
hCoV-19/Mexico/VER-LANGEBIO_IMSS_1134/2021	EPI_ISL_2671744	OL461660	B.1.1.7	GRY	40	C203T,C241T,C913T,G1820A,C3037T,C3267T,C5388A,G5924A,C5986T,T6954C,G9526T,A12162G,C14408T,C14676T,C15279T,C15720T,T16176C,A17615G,T19363C,A21137G,A23063T,T23197C,C23248T,C23271A,A23403G,C23604A,C23709T,T24506G,G24914C,G25135T,C27972T,G28048T,A28111G,G28280C,A28281T,T28282A,G28881A,G28882A,G28883C,C28977T	25	N:D3L,N:R203K,N:G204R,N:S235F,ORF1a:G519S,ORF1a:T1001I,ORF1a:A1708D,ORF1a:V1887I,ORF1a:I2230T,ORF1a:M3087I,ORF1a:Q3966R,ORF1b:P314L,ORF1b:K1383R,ORF1b:K2557R,ORF8:Q27*,ORF8:R52I,ORF8:Y73C,S:N501Y,S:A570D,S:D614G,S:P681H,S:T716I,S:S982A,S:D1118H,S:K1191N	6	0
hCoV-19/Mexico/VER-LANGEBIO_IMSS_1424/2021	EPI_ISL_2942548	OL461661	B.1.1.7	GRY	39	C241T,C913T,G1820A,C3037T,C3267T,G5023T,C5388A,G5924A,C5986T,T6954C,G9526T,A12162G,C14408T,C14676T,C15279T,C15720T,T16176C,A17615G,T19363C,A21137G,A23063T,C23248T,C23271A,A23403G,C23604A,C23709T,T24506G,G24914C,G25135T,C27972T,G28048T,A28111G,G28280C,A28281T,T28282A,G28881A,G28882A,G28883C,C28977T	26	N:D3L,N:R203K,N:G204R,N:S235F,ORF1a:G519S,ORF1a:T1001I,ORF1a:M1586I,ORF1a:A1708D,ORF1a:V1887I,ORF1a:I2230T,ORF1a:M3087I,ORF1a:Q3966R,ORF1b:P314L,ORF1b:K1383R,ORF1b:K2557R,ORF8:Q27*,ORF8:R52I,ORF8:Y73C,S:N501Y,S:A570D,S:D614G,S:P681H,S:T716I,S:S982A,S:D1118H,S:K1191N	6	224
hCoV-19/Mexico/VER-LANGEBIO_IMSS_1426/2021	EPI_ISL_2942550	OL461662	B.1.1.7	GRY	42	C241T,C913T,C2110T,G3004T,C3037T,C3267T,C5388A,C5986T,T6954C,G7042T,C9996T,C10525T,C10789T,C14120T,C14408T,C14676T,C15279T,T16176C,A23063T,C23271A,A23403G,G23593T,C23604A,C23709T,T24506G,G24914C,C25844T,C26110T,C26735T,C27389T,C27972T,G28048T,A28095T,A28111G,G28280C,A28281T,T28282A,A28804G,G28881A,G28882A,G28883C,C289	26	N:D3L,N:R203K,N:G204R,N:S235F,ORF1a:E913D,ORF1a:T1001I,ORF1a:A1708D,ORF1a:I2230T,ORF1a:M2259I,ORF1a:S3244L,ORF1b:P218L,ORF1b:P314L,ORF3a:T151I,ORF3a:P240S,ORF8:Q27*,ORF8:R52I,ORF8:K68*,ORF8:Y73C,S:N501Y,S:A570D,S:D614G,S:Q677H,S:P681H,S:T716I,S:S982A,S:D1118H	6	0
hCoV-19/Mexico/VER-LANGEBIO_IMSS_1428/2021	EPI_ISL_2942551	OL461663	B.1.1.7	GRY	35	C913T,C1613T,C2453T,C3037T,C3267T,C5388A,T6954C,C9611T,C9893T,C14408T,C14676T,T15195C,C15279T,T16176C,C21855T,G21974C,A23063T,C23271A,A23403G,C23604A,C23709T,T24506G,G24914C,G25455T,C27972T,G28048T,A28111G,G28280C,A28281T,T28282A,A28295G,G28881A,G28882A,G28883C,C28977T	26	N:D3L,N:N8D,N:R203K,N:G204R,N:S235F,ORF1a:L450F,ORF1a:L730F,ORF1a:T1001I,ORF1a:A1708D,ORF1a:I2230T,ORF1a:L3116F,ORF1a:L3210F,ORF1b:P314L,ORF3a:K21N,ORF8:Q27*,ORF8:R52I,ORF8:Y73C,S:S98F,S:D138H,S:N501Y,S:A570D,S:D614G,S:P681H,S:T716I,S:S982A,S:D1118H	6	772
hCoV-19/Mexico/VER-LANGEBIO_IMSS_1750/2021	EPI_ISL_2942831	OL461664	B.1.1.7	GRY	41	C241T,C913T,C2110T,G3004T,C3037T,C3267T,C5388A,C5986T,T6954C,G7042T,C9996T,C10525T,C10789T,C14120T,C14408T,C14676T,C15279T,T16176C,A23063T,C23271A,A23403G,G23593T,C23604A,C23709T,T24506G,G24914C,C25844T,C26110T,C26735T,C27972T,G28048T,A28111G,G28280C,A28281T,T28282A,G28739T,A28804G,G28881A,G28882A,G28883C,C28977T	26	N:D3L,N:A156S,N:R203K,N:G204R,N:S235F,ORF1a:E913D,ORF1a:T1001I,ORF1a:A1708D,ORF1a:I2230T,ORF1a:M2259I,ORF1a:S3244L,ORF1b:P218L,ORF1b:P314L,ORF3a:T151I,ORF3a:P240S,ORF8:Q27*,ORF8:R52I,ORF8:Y73C,S:N501Y,S:A570D,S:D614G,S:Q677H,S:P681H,S:T716I,S:S982A,S:D1118H	6	0
hCoV-19/Mexico/YUC_IBT_IMSS_10287-NC/2021	EPI_ISL_2681375	OL461665	B.1.1.7	GRY	44	C241T,C913T,G3004T,C3037T,C3267T,C5388A,C5986T,T6954C,C7765T,C10789T,C14120T,C14408T,C14676T,G14857T,C15279T,G15672C,C15810T,T16176C,G20679T,T22219C,G22567T,A23063T,C23271A,A23403G,C23604A,C23709T,T24506G,G24914C,C25731T,C26110T,C26735T,C27002T,C27972T,G28048T,A28095T,A28111G,G28280C,A28281T,T28282A,A28804G,G28881A,G2	25	N:D3L,N:R203K,N:G204R,N:S235F,ORF1a:E913D,ORF1a:T1001I,ORF1a:A1708D,ORF1a:I2230T,ORF1b:P218L,ORF1b:P314L,ORF1b:V464F,ORF1b:E735D,ORF3a:P240S,ORF8:Q27*,ORF8:R52I,ORF8:K68*,ORF8:Y73C,S:L335F,S:N501Y,S:A570D,S:D614G,S:P681H,S:T716I,S:S982A,S:D1118H	6	1291
hCoV-19/Mexico/YUC_IBT_IMSS_1346/2021	EPI_ISL_2391720	OL461666	B.1.1.7	GRY	40	C241T,C913T,C3037T,C3267T,C5388A,C5986T,T6954C,C7604T,C9693T,C11572T,C11824A,C14408T,C14676T,T14910C,C15279T,T16176C,G18651A,T23042C,A23063T,C23271A,A23403G,C23604A,C23709T,T24506G,G24914C,G25218T,C25553T,G25660C,C27615T,C27972T,G28048T,A28095T,A28111G,G28280C,A28281T,T28282A,G28881A,G28882A,G28883C,C28977T	24	N:D3L,N:R203K,N:G204R,N:S235F,ORF1a:T1001I,ORF1a:A1708D,ORF1a:I2230T,ORF1a:A3143V,ORF1b:P314L,ORF3a:A54V,ORF3a:V90L,ORF8:Q27*,ORF8:R52I,ORF8:K68*,ORF8:Y73C,S:S494P,S:N501Y,S:A570D,S:D614G,S:P681H,S:T716I,S:S982A,S:D1118H,S:G1219V	6	655

hCoV-19/Mexico/YUC_IBT_IMSS_1727/2021	EPI_ISL_2681108	OL461667	B.1.1.7	GRY	42	C241T,C913T,G3004T,C3037T,C3267T,C5388A,C5986T,T6954C,C7765T,C8078T,C10789T,C11704T,C14408T,C14676T,C15279T,G15672C,C15810T,T16176C,G20679T,G22567T,A23063T,C23271A,A23403G,C23604A,C23709T,T24506G,G24914C,C26110T,C26735T,C27972T,G28048T,A28095T,A28111G,G28280C,A28281T,T28282A,A28295G,G28881A,G28882A,G28883C,G28960T,C2	26	N:D3L,N:N8D,N:R203K,N:G204R,N:Q229H,N:S235F,ORF1a:E913D,ORF1a:T1001I,ORF1a:A1708D,ORF1a:I2230T,ORF1a:P2605S,ORF1b:P314L,ORF1b:E735D,ORF3a:P240S,ORF8:Q27*,ORF8:R52I,ORF8:K68*,ORF8:Y73C,S:L335F,S:N501Y,S:A570D,S:D614G,S:P681H,S:T716I,S:S982A,S:D1118H	6	0
hCoV-19/Mexico/YUC_IBT_IMSS_1748/2021	EPI_ISL_2681328	OL461668	B.1.1.7	GRY	40	C241T,C913T,C2453T,C3037T,C3267T,C5388A,C5986T,A6575T,T6954C,C7528T,C9611T,C14408T,C14676T,C15279T,T16176C,C20930T,C21855T,G21974C,A23063T,C23271A,A23403G,C23604A,C23709T,T24506G,C24745T,G24914C,C26907T,C27972T,G28048T,A28111G,G28280C,A28281T,T28282A,A28295G,G28881A,G28882A,G28883C,C28977T,C29415T,C29743T	26	N:D3L,N:N8D,N:R203K,N:G204R,N:S235F,N:A381V,ORF1a:L730F,ORF1a:T1001I,ORF1a:A1708D,ORF1a:S2104C,ORF1a:I2230T,ORF1a:L3116F,ORF1b:P314L,ORF1b:T2488M,ORF8:Q27*,ORF8:R52I,ORF8:Y73C,S:S98F,S:D138H,S:N501Y,S:A570D,S:D614G,S:P681H,S:T716I,S:S982A,S:D1118H	6	603
hCoV-19/Mexico/YUC_INER_IMSS_00782/2021	EPI_ISL_2091236	OL461669	B.1.1.7	GRY	36	C222T,C241T,C913T,C2110T,C3037T,C3267T,G4201T,C5388A,C5986T,T6954C,G7042T,C14120T,C14408T,C14676T,C15279T,T16176C,C17762T,A23063T,C23271A,A23403G,C23604A,C23709T,T24506G,G24914C,T25180C,C27972T,G28048T,A28095T,A28111G,G28280C,A28281T,T28282A,G28881A,G28882A,G28883C,C28977T	23	N:D3L,N:R203K,N:G204R,N:S235F,ORF1a:T1001I,ORF1a:M1312I,ORF1a:A1708D,ORF1a:I2230T,ORF1a:M2259I,ORF1b:P218L,ORF1b:P314L,ORF1b:A1432V,ORF8:Q27*,ORF8:R52I,ORF8:K68*,ORF8:Y73C,S:N501Y,S:A570D,S:D614G,S:P681H,S:T716I,S:S982A,S:D1118H	6	0
hCoV-19/Mexico/YUC_INER_IMSS_1108/2021	EPI_ISL_2490344	OL461670	B.1.1.7	GRY	45	C241T,C913T,C2110T,G3004T,C3037T,C3267T,C5388A,C5986T,T6954C,G7042T,C7765T,C10789T,C14120T,C14408T,C14676T,G14857T,C15279T,G15672C,C15810T,T16176C,G20679T,T22219C,G22567T,A23063T,C23271A,A23403G,C23604A,C23709T,T24506G,G24914C,C25731T,C26110T,C26735T,C27972T,G28048T,A28095T,A28111G,G28280C,A28281T,T28282A,A28804G,G288	26	N:D3L,N:R203K,N:G204R,N:S235F,ORF1a:E913D,ORF1a:T1001I,ORF1a:A1708D,ORF1a:I2230T,ORF1a:M2259I,ORF1b:P218L,ORF1b:P314L,ORF1b:V464F,ORF1b:E735D,ORF3a:P240S,ORF8:Q27*,ORF8:R52I,ORF8:K68*,ORF8:Y73C,S:L335F,S:N501Y,S:A570D,S:D614G,S:P681H,S:T716I,S:S982A,S:D1118H	6	0
hCoV-19/Mexico/YUC_LANGEBIO_IMSS_0596/2021	EPI_ISL_2402015	OL461671	B.1.1.7	GRY	39	C241T,C913T,C1076T,C3037T,C3267T,C5388A,C5986T,C6388T,T6954C,G7042T,A10195G,C11224T,G11335A,C14120T,C14408T,C14676T,A14856G,C15279T,T16176C,A23063T,C23271A,A23403G,C23604A,C23709T,T24506G,G24914C,C27972T,G28048T,G28075T,A28095T,A28111G,G28280C,A28281T,T28282A,G28881A,G28882A,G28883C,C28977T,G29751T	23	N:D3L,N:R203K,N:G204R,N:S235F,ORF1a:P271S,ORF1a:T1001I,ORF1a:A1708D,ORF1a:I2230T,ORF1a:M2259I,ORF1b:P218L,ORF1b:P314L,ORF8:Q27*,ORF8:R52I,ORF8:C61F,ORF8:K68*,ORF8:Y73C,S:N501Y,S:A570D,S:D614G,S:P681H,S:T716I,S:S982A,S:D1118H	6	354
hCoV-19/Mexico/YUC-IBT_IMSS_1845/2021	EPI_ISL_2801564	OL461672	B.1.1.7	GRY	35	C241T,C254T,C913T,C2453T,C3037T,C3267T,C5388A,C5986T,T6954C,C9611T,C14408T,C14676T,C15279T,T16176C,T18099C,C21855T,A23063T,C23271A,A23403G,C23604A,C23709T,T24506G,G24914C,C25609T,C27972T,G28048T,A28111G,G28280C,A28281T,T28282A,A28295G,G28881A,G28882A,G28883C,C28977T	23	N:D3L,N:N8D,N:R203K,N:G204R,N:S235F,ORF1a:L730F,ORF1a:T1001I,ORF1a:A1708D,ORF1a:I2230T,ORF1a:L3116F,ORF1b:P314L,ORF3a:L73F,ORF8:Q27*,ORF8:R52I,ORF8:Y73C,S:S98F,S:N501Y,S:A570D,S:D614G,S:P681H,S:T716I,S:S982A,S:D1118H	6	741
hCoV-19/Mexico/YUC-IBT_IMSS_2008/2021	EPI_ISL_2801598	OL461673	B.1.1.7	GRY	42	C241T,C913T,C2110T,C3037T,C3267T,C5388A,C5986T,C6701T,T6954C,G7042T,C9962T,C11750T,C14120T,C14408T,C14676T,C15279T,T16176C,G16416A,C17678T,G19656T,C21721T,A23063T,C23271A,A23403G,C23604A,C23709T,T24506G,G24914C,G26065T,C27972T,G28048T,C28054T,A28095T,A28111G,G28280C,A28281T,T28282A,C28367T,G28881A,G28882A,G28883C,C28977T	29	N:D3L,N:R32C,N:R203K,N:G204R,N:S235F,ORF1a:T1001I,ORF1a:A1708D,ORF1a:L2146F,ORF1a:I2230T,ORF1a:M2259I,ORF1a:H3233Y,ORF1a:L3829F,ORF1b:P218L,ORF1b:P314L,ORF1b:T1404M,ORF1b:K2063N,ORF3a:V225F,ORF8:Q27*,ORF8:R52I,ORF8:S54L,ORF8:K68*,ORF8:Y73C,S:N501Y,S:A570D,S:D614G,S:P681H,S:T716I,S:S982A,S:D1118H	6	0
hCoV-19/Mexico/YUC-IBT_IMSS_2027/2021	EPI_ISL_2801600	OL461674	B.1.1.7	GRY	42	C241T,C913T,C2110T,G3004T,C3037T,C3267T,C4099T,C5388A,C5986T,T6954C,G7042T,C10789T,C14120T,C14408T,C14676T,G14857T,C15279T,T16176C,T18144C,A19838C,G22567T,A23063T,C23271A,A23403G,C23604A,C23709T,T24506G,G24914C,C26110T,C26735T,C27972T,G28048T,A28095T,A28111G,G28280C,A28281T,T28282A,A28804G,G28881A,G28882A,G28883C,C28	26	N:D3L,N:R203K,N:G204R,N:S235F,ORF1a:E913D,ORF1a:T1001I,ORF1a:A1708D,ORF1a:I2230T,ORF1a:M2259I,ORF1b:P218L,ORF1b:P314L,ORF1b:V464F,ORF1b:N2124T,ORF3a:P240S,ORF8:Q27*,ORF8:R52I,ORF8:K68*,ORF8:Y73C,S:L335F,S:N501Y,S:A570D,S:D614G,S:P681H,S:T716I,S:S982A,S:D1118H	6	20
hCoV-19/Mexico/YUC-IBT_IMSS_2048/2021	EPI_ISL_2801601	OL461675	B.1.1.7	GRY	45	C241T,C913T,G3004T,C3037T,C3176T,C3267T,C5388A,C5986T,T6954C,C7765T,C10789T,C14120T,C14408T,C14676T,G14857T,C15279T,G15672C,C15810T,T16176C,G20679T,T22219C,G22567T,A23063T,C23271A,A23403G,C23604A,C23709T,T24506G,G24914C,C25731T,C26110T,C26735T,C27002T,C27972T,G28048T,A28095T,A28111G,G28280C,A28281T,T28282A,A28804G,G28	26	N:D3L,N:R203K,N:G204R,N:S235F,ORF1a:E913D,ORF1a:P971S,ORF1a:T1001I,ORF1a:A1708D,ORF1a:I2230T,ORF1b:P218L,ORF1b:P314L,ORF1b:V464F,ORF1b:E735D,ORF3a:P240S,ORF8:Q27*,ORF8:R52I,ORF8:K68*,ORF8:Y73C,S:L335F,S:N501Y,S:A570D,S:D614G,S:P681H,S:T716I,S:S982A,S:D1118H	6	637
hCoV-19/Mexico/YUC-IBT_IMSS_2049/2021	EPI_ISL_2801602	OL461676	B.1.1.7	GRY	38	C241T,C913T,C3037T,C3045T,C3267T,C5388A,C5986T,T6954C,G7042T,A10195G,C10279T,C14120T,C14408T,C14676T,C15279T,T16176C,C16887T,G17427T,A23063T,C23271A,A23403G,C23604A,C23709T,T24506G,G24914C,A27943G,C27972T,G28048T,G28075T,A28095T,A28111G,G28280C,A28281T,T28282A,G28881A,G28882A,G28883C,C28977T	24	N:D3L,N:R203K,N:G204R,N:S235F,ORF1a:P927L,ORF1a:T1001I,ORF1a:A1708D,ORF1a:I2230T,ORF1a:M2259I,ORF1b:P218L,ORF1b:P314L,ORF8:H17R,ORF8:Q27*,ORF8:R52I,ORF8:C61F,ORF8:K68*,ORF8:Y73C,S:N501Y,S:A570D,S:D614G,S:P681H,S:T716I,S:S982A,S:D1118H	6	705
hCoV-19/Mexico/YUC-LANGEBIO_IMSS_0942/2021	EPI_ISL_2671750	OL461677	B.1.1.7	GRY	40	C913T,C2469T,C3037T,C3267T,T4966C,C5388A,C5974T,C5986T,T6954C,C7299T,C7945T,C12099T,C14184T,C14408T,C14676T,T15096C,C15279T,T16176C,A23063T,C23271A,A23403G,C23604A,C23709T,G24140C,T24506G,G24914C,C27972T,G28048T,A28095T,A28111G,G28280C,A28281T,T28282A,A28877T,G28878C,G28881A,G28882A,G28883C,C28977T,C29722T	23	N:D3L,N:R203K,N:G204R,N:S235F,ORF1a:A735V,ORF1a:T1001I,ORF1a:A1708D,ORF1a:I2230T,ORF1a:A2345V,ORF1a:A3945V,ORF1b:P314L,ORF8:Q27*,ORF8:R52I,ORF8:K68*,ORF8:Y73C,S:N501Y,S:A570D,S:D614G,S:P681H,S:T716I,S:V860L,S:S982A,S:D1118H	6	189
hCoV-19/Mexico/YUC-LANGEBIO_IMSS_0964/2021	EPI_ISL_2671754	OL461678	B.1.1.7	GRY	42	C241T,C913T,C2110T,C2143T,C3037T,G3047T,C3267T,C5388A,C5986T,G6446T,C6730T,T6954C,C7945T,A10195G,C10834T,C14120T,C14408T,C14676T,C15279T,T16176C,T20445C,A23063T,C23271A,A23403G,C23604A,C23709T,T24506G,G24914C,C27972T,G28048T,G28075T,A28111G,C28236T,G28280C,A28281T,T28282A,G28881A,G28882A,G28883C,C28896G,C28977T,C29642	24	N:D3L,N:R203K,N:G204R,N:A208G,N:S235F,ORF1a:D928Y,ORF1a:T1001I,ORF1a:A1708D,ORF1a:V2061F,ORF1a:I2230T,ORF1b:P218L,ORF1b:P314L,ORF8:Q27*,ORF8:R52I,ORF8:C61F,ORF8:Y73C,ORF8:R115C,S:N501Y,S:A570D,S:D614G,S:P681H,S:T716I,S:S982A,S:D1118H	6	0
hCoV-19/Mexico/YUC-LANGEBIO_IMSS_1240/2021	EPI_ISL_2942401	OL580735	B.1.1.7	GRY	37	C913T,C2110T,C3037T,C3267T,C5388A,C5986T,T6954C,G7042T,A10195G,C10279T,C14120T,C14408T,C14676T,C15279T,T16176C,C16887T,G17427T,G22319A,A23063T,C23271A,A23403G,C23604A,C23709T,T24506G,G24914C,A27943G,C27972T,G28048T,G28075T,A28111G,G28280C,A28281T,T28282A,G28881A,G28882A,G28883C,C28977T	23	N:D3L,N:R203K,N:G204R,N:S235F,ORF1a:T1001I,ORF1a:A1708D,ORF1a:I2230T,ORF1a:M2259I,ORF1b:P218L,ORF1b:P314L,ORF8:H17R,ORF8:Q27*,ORF8:R52I,ORF8:C61F,ORF8:Y73C,S:D253N,S:N501Y,S:A570D,S:D614G,S:P681H,S:T716I,S:S982A,S:D1118H	6	417
hCoV-19/Mexico/YUC-LANGEBIO_IMSS_1245/2021	EPI_ISL_2942406	OL461679	B.1.1.7	GRY	37	C241T,C913T,C2110T,C3037T,C3267T,C5388A,C5986T,T6954C,G7042T,A10195G,C10279T,C14120T,C14408T,C14676T,C15279T,T16176C,C16887T,G17427T,A23063T,C23271A,A23403G,C23604A,C23709T,T24506G,G24914C,A27943G,C27972T,G28048T,G28075T,A28111G,G28280C,A28281T,T28282A,G28881A,G28882A,G28883C,C28977T	22	N:D3L,N:R203K,N:G204R,N:S235F,ORF1a:T1001I,ORF1a:A1708D,ORF1a:I2230T,ORF1a:M2259I,ORF1b:P218L,ORF1b:P314L,ORF8:H17R,ORF8:Q27*,ORF8:R52I,ORF8:C61F,ORF8:Y73C,S:N501Y,S:A570D,S:D614G,S:P681H,S:T716I,S:S982A,S:D1118H	6	16
hCoV-19/Mexico/YUC-LANGEBIO_IMSS_1562/2021	EPI_ISL_2942673	OL461680	B.1.1.7	GRY	46	C241T,C913T,C2110T,G3004T,C3037T,C3267T,C5388A,C5986T,T6954C,G7042T,C7765T,C10789T,C14120T,C14408T,C14676T,G14857T,C15279T,G15672C,C15810T,T16176C,G20679T,T22219C,G22567T,A23063T,C23271A,A23403G,C23604A,C23709T,T24506G,G24914C,C25731T,C26110T,C26735T,C27002T,C27972T,G28048T,A28095T,A28111G,G28280C,A28281T,T28282A,A28804G,G28881A,G28882A,G28883C,C28977T	26	N:D3L,N:R203K,N:G204R,N:S235F,ORF1a:E913D,ORF1a:T1001I,ORF1a:A1708D,ORF1a:I2230T,ORF1a:M2259I,ORF1b:P218L,ORF1b:P314L,ORF1b:V464F,ORF1b:E735D,ORF3a:P240S,ORF8:Q27*,ORF8:R52I,ORF8:K68*,ORF8:Y73C,S:L335F,S:N501Y,S:A570D,S:D614G,S:P681H,S:T716I,S:S982A,S:D1118H	6	12
hCoV-19/Mexico/YUC-LANGEBIO_IMSS_1586/2021	EPI_ISL_2942697	OL461681	B.1.1.7	GRY	41	C241T,C823T,C913T,C1758T,C2110T,G3004T,C3037T,C3267T,C5388A,C5986T,T6954C,G7042T,C9559T,C14120T,C14408T,C14676T,C15279T,T16176C,A23063T,C23271A,A23403G,C23604A,C23709T,T24506G,G24914C,G25599T,C26110T,G27382C,A27383T,T27384C,C27972T,G28048T,A28095T,A28111G,G28280C,A28281T,T28282A,G28881A,G28882A,G28883C,C28977T	26	N:D3L,N:R203K,N:G204R,N:S235F,ORF1a:A498V,ORF1a:E913D,ORF1a:T1001I,ORF1a:A1708D,ORF1a:I2230T,ORF1a:M2259I,ORF1b:P218L,ORF1b:P314L,ORF3a:W69C,ORF3a:P240S,ORF6:D61L,ORF8:Q27*,ORF8:R52I,ORF8:K68*,ORF8:Y73C,S:N501Y,S:A570D,S:D614G,S:P681H,S:T716I,S:S982A,S:D1118H	6	0
hCoV-19/Mexico/ZAC_IBT_IMSS_1462/2021	EPI_ISL_2681120	OL461682	B.1.1.7	GRY	35	C241T,C913T,C2453T,C3037T,C3267T,T5338C,C5388A,C5986T,T6954C,C9611T,C14408T,C14676T,C15279T,T16176C,C21855T,G21974C,A23063T,C23271A,A23403G,C23604A,C23709T,T24506G,G24914C,C25609T,C27972T,G28048T,A28111G,G28280C,A28281T,T28282A,A28295G,G28881A,G28882A,G28883C,C28977T	24	N:D3L,N:N8D,N:R203K,N:G204R,N:S235F,ORF1a:L730F,ORF1a:T1001I,ORF1a:A1708D,ORF1a:I2230T,ORF1a:L3116F,ORF1b:P314L,ORF3a:L73F,ORF8:Q27*,ORF8:R52I,ORF8:Y73C,S:S98F,S:D138H,S:N501Y,S:A570D,S:D614G,S:P681H,S:T716I,S:S982A,S:D1118H	6	0
hCoV-19/Mexico/ZAC_IBT_IMSS_1470/2021	EPI_ISL_2681121	OL461683	B.1.1.7	GRY	37	C241T,C913T,C2453T,C3037T,C3267T,C5388A,C5986T,T6954C,C9611T,C9893T,C10029T,C10714T,C14408T,C14676T,C15279T,T16176C,C21855T,G21974C,A23063T,C23271A,A23403G,C23604A,C23709T,T24506G,G24914C,C27972T,G28048T,A28111G,G28280C,A28281T,T28282A,A28295G,G28881A,G28882A,G28883C,G28960T,C28977T	26	N:D3L,N:N8D,N:R203K,N:G204R,N:Q229H,N:S235F,ORF1a:L730F,ORF1a:T1001I,ORF1a:A1708D,ORF1a:I2230T,ORF1a:L3116F,ORF1a:L3210F,ORF1a:T3255I,ORF1b:P314L,ORF8:Q27*,ORF8:R52I,ORF8:Y73C,S:S98F,S:D138H,S:N501Y,S:A570D,S:D614G,S:P681H,S:T716I,S:S982A,S:D1118H	6	0

hCoV-19/Mexico/ZAC_IBT_IMSS_1472/2021	EPI_ISL_2681092	OL461684	B.1.1.7	GRY	38	C241T,C913T,C2453T,C3037T,C3267T,C5388A,C5986T,T6954C,C9611T,C14408T,C14676T,C15279T,T16176C,C18115T,C21855T,G21974C,T22469C,A23063T,C23271A,A23403G,C23604A,C23709T,T24506G,G24914C,C27389T,C27972T,G28048T,A28111G,G28280C,A28281T,T28282A,A28295G,C28348T,G28881A,G28882A,G28883C,C28977T,C29098T	25	N:D3L,N:N8D,N:R203K,N:G204R,N:S235F,ORF1a:L730F,ORF1a:T1001I,ORF1a:A1708D,ORF1a:I2230T,ORF1a:L3116F,ORF1b:P314L,ORF1b:H1550Y,ORF8:Q27*,ORF8:R52I,ORF8:Y73C,ORF9b:A22V,S:S98F,S:D138H,S:N501Y,S:A570D,S:D614G,S:P681H,S:T716I,S:S982A,S:D1118H	6	0
hCoV-19/Mexico/ZAC_IBT_IMSS_1480/2021	EPI_ISL_2681142	OL461685	B.1.1.7	GRY	37	C241T,C913T,C2110T,C3037T,C3267T,C5388A,C5986T,T6954C,G7042T,C12141A,C12754T,C14120T,C14408T,C14676T,C15279T,T16176C,T17583G,A23063T,C23271A,A23403G,C23604A,C23709T,T24506G,G24914C,C25624T,C27972T,G28048T,A28095T,A28111G,G28280C,A28281T,T28282A,C28531T,G28881A,G28882A,G28883C,C28977T	24	N:D3L,N:R203K,N:G204R,N:S235F,ORF1a:T1001I,ORF1a:A1708D,ORF1a:I2230T,ORF1a:M2259I,ORF1a:T3959N,ORF1b:P218L,ORF1b:P314L,ORF3a:H78Y,ORF8:Q27*,ORF8:R52I,ORF8:K68*,ORF8:Y73C,ORF9b:T83I,S:N501Y,S:A570D,S:D614G,S:P681H,S:T716I,S:S982A,S:D1118H	6	0
hCoV-19/Mexico/ZAC_IBT_IMSS_1483/2021	EPI_ISL_2681190	OL461686	B.1.1.7	GRY	35	C241T,C913T,C1912T,C3037T,C3267T,A4855G,C5388A,C5986T,T6954C,T9167C,C11344T,T11485C,C14408T,C14676T,C15279T,T16176C,C22498T,A23063T,C23271A,A23403G,C23604A,C23709T,T24506G,G24914C,C26013T,C27972T,G28048T,A28111G,G28280C,A28281T,T28282A,G28881A,G28882A,G28883C,C28977T	19	N:D3L,N:R203K,N:G204R,N:S235F,ORF1a:T1001I,ORF1a:A1708D,ORF1a:I2230T,ORF1a:Y2968H,ORF1b:P314L,ORF8:Q27*,ORF8:R52I,ORF8:Y73C,S:N501Y,S:A570D,S:D614G,S:P681H,S:T716I,S:S982A,S:D1118H	6	132
hCoV-19/Mexico/ZAC_INER_IMSS_1210/2021	EPI_ISL_2835939	OL461687	B.1.1.7	GRY	36	C241T,C913T,C3037T,C3267T,C5388A,C5986T,G6362A,T6954C,C12970T,C13176T,C14408T,C14676T,C15279T,T16176C,G17427T,C17977T,A23063T,C23271A,A23403G,C23604A,C23709T,C24023T,C24054T,T24506G,G24914C,C27972T,G28048T,A28111G,G28209T,G28280C,A28281T,T28282A,G28881A,G28882A,G28883C,C28977T	23	N:D3L,N:R203K,N:G204R,N:S235F,ORF1a:T1001I,ORF1a:A1708D,ORF1a:A2033T,ORF1a:I2230T,ORF1a:T4304I,ORF1b:P314L,ORF1b:L1504F,ORF8:Q27*,ORF8:R52I,ORF8:Y73C,ORF8:E106*,S:N501Y,S:A570D,S:D614G,S:P681H,S:T716I,S:A831V,S:S982A,S:D1118H	6	0
hCoV-19/Mexico/ZAC_INER_IMSS_1217/2021	EPI_ISL_2835941	OL461688	B.1.1.7	GRY	35	C241T,C913T,C3037T,C3267T,C5388A,C5986T,G6362A,T6954C,C12970T,C13176T,C14408T,C14676T,C15279T,T16176C,C17977T,A23063T,C23271A,A23403G,C23604A,C23709T,C24023T,C24054T,T24506G,G24914C,C27972T,G28048T,A28111G,G28209T,G28280C,A28281T,T28282A,G28881A,G28882A,G28883C,C28977T	23	N:D3L,N:R203K,N:G204R,N:S235F,ORF1a:T1001I,ORF1a:A1708D,ORF1a:A2033T,ORF1a:I2230T,ORF1a:T4304I,ORF1b:P314L,ORF1b:L1504F,ORF8:Q27*,ORF8:R52I,ORF8:Y73C,ORF8:E106*,S:N501Y,S:A570D,S:D614G,S:P681H,S:T716I,S:A831V,S:S982A,S:D1118H	6	0
hCoV-19/Mexico/ZAC_INER_IMSS_1225/2021	EPI_ISL_2835942	OL461689	B.1.1.7	GRY	36	C241T,C913T,C3037T,C3267T,C5388A,C5986T,G6362A,T6954C,C12970T,C13176T,C14408T,C14676T,C15279T,T16176C,G17427T,C17977T,A23063T,C23271A,A23403G,C23604A,C23709T,C24023T,C24054T,T24506G,G24914C,C27972T,G28048T,A28111G,G28209T,G28280C,A28281T,T28282A,G28881A,G28882A,G28883C,C28977T	23	N:D3L,N:R203K,N:G204R,N:S235F,ORF1a:T1001I,ORF1a:A1708D,ORF1a:A2033T,ORF1a:I2230T,ORF1a:T4304I,ORF1b:P314L,ORF1b:L1504F,ORF8:Q27*,ORF8:R52I,ORF8:Y73C,ORF8:E106*,S:N501Y,S:A570D,S:D614G,S:P681H,S:T716I,S:A831V,S:S982A,S:D1118H	6	0
hCoV-19/Mexico/ZAC_INER_IMSS_1230/2021	EPI_ISL_2490333	OL461690	B.1.1.7	GRY	39	C241T,C913T,C2453T,C3037T,C3267T,G5023T,C5388A,C5986T,T6954C,C8836T,T9445C,C9611T,C14408T,C14676T,C15279T,T16176C,C16293T,G20102T,C21855T,G21974C,A23063T,C23271A,A23403G,C23604A,C23709T,T24506G,G24914C,G25785T,C27972T,G28048T,A28111G,G28280C,A28281T,T28282A,A28295G,G28881A,G28882A,G28883C,C28977T	26	N:D3L,N:N8D,N:R203K,N:G204R,N:S235F,ORF1a:L730F,ORF1a:T1001I,ORF1a:M1586I,ORF1a:A1708D,ORF1a:I2230T,ORF1a:L3116F,ORF1b:P314L,ORF1b:S2212I,ORF3a:W131C,ORF8:Q27*,ORF8:R52I,ORF8:Y73C,S:S98F,S:D138H,S:N501Y,S:A570D,S:D614G,S:P681H,S:T716I,S:S982A,S:D1118H	6	0
hCoV-19/Mexico/ZAC_INER_IMSS_1235/2021	EPI_ISL_2835943	OL461691	B.1.1.7	GRY	35	C241T,C913T,C3037T,C3267T,C5388A,C5986T,G6362A,T6954C,C12970T,C13176T,C14408T,C14676T,C15279T,T16176C,C17977T,A23063T,C23271A,A23403G,C23604A,C23709T,C24023T,C24054T,T24506G,G24914C,C27972T,G28048T,A28111G,G28209T,G28280C,A28281T,T28282A,G28881A,G28882A,G28883C,C28977T	23	N:D3L,N:R203K,N:G204R,N:S235F,ORF1a:T1001I,ORF1a:A1708D,ORF1a:A2033T,ORF1a:I2230T,ORF1a:T4304I,ORF1b:P314L,ORF1b:L1504F,ORF8:Q27*,ORF8:R52I,ORF8:Y73C,ORF8:E106*,S:N501Y,S:A570D,S:D614G,S:P681H,S:T716I,S:A831V,S:S982A,S:D1118H	6	0
hCoV-19/Mexico/ZAC_INER_IMSS_1237/2021	EPI_ISL_2490348	OL461692	B.1.1.7	GRY	38	C241T,C913T,C2110T,C2841T,C3037T,C3267T,C5388A,C5986T,T6954C,G7042T,C12141A,C14120T,C14408T,C14676T,C15279T,T16176C,T17583G,A23063T,C23271A,A23403G,C23604A,C23709T,T24506G,G24914C,C25624T,C27972T,G28048T,A28095T,A28111G,G28280C,A28281T,T28282A,C28531T,G28881A,G28882A,G28883C,C28977T,G29254T	25	N:D3L,N:R203K,N:G204R,N:S235F,ORF1a:A859V,ORF1a:T1001I,ORF1a:A1708D,ORF1a:I2230T,ORF1a:M2259I,ORF1a:T3959N,ORF1b:P218L,ORF1b:P314L,ORF3a:H78Y,ORF8:Q27*,ORF8:R52I,ORF8:K68*,ORF8:Y73C,ORF9b:T83I,S:N501Y,S:A570D,S:D614G,S:P681H,S:T716I,S:S982A,S:D1118H	6	0
hCoV-19/Mexico/ZAC_INER_IMSS_1244/2021	EPI_ISL_2835945	OL461693	B.1.1.7	GRY	36	C241T,C913T,C3037T,C3267T,C5388A,C5986T,G6362A,T6954C,A10323G,C12970T,C13176T,C14408T,C14676T,C15279T,T16176C,C17977T,A23063T,C23271A,A23403G,C23604A,C23709T,C24023T,C24054T,T24506G,G24914C,C27972T,G28048T,A28111G,G28209T,G28280C,A28281T,T28282A,G28881A,G28882A,G28883C,C28977T	24	N:D3L,N:R203K,N:G204R,N:S235F,ORF1a:T1001I,ORF1a:A1708D,ORF1a:A2033T,ORF1a:I2230T,ORF1a:K3353R,ORF1a:T4304I,ORF1b:P314L,ORF1b:L1504F,ORF8:Q27*,ORF8:R52I,ORF8:Y73C,ORF8:E106*,S:N501Y,S:A570D,S:D614G,S:P681H,S:T716I,S:A831V,S:S982A,S:D1118H	6	0
hCoV-19/Mexico/ZAC_LANGEBIO_IMSS_0301/2021	EPI_ISL_1662226	OL461694	B.1.1.7	GRY	37	C241T,C913T,A2410G,C3037T,C3267T,C3777T,C4084T,C5388A,C5986T,G6362A,T6954C,C12970T,C13176T,C14408T,C14676T,C15279T,T16176C,A21367T,A23063T,C23271A,A23403G,C23604A,C23709T,C24023T,C24054T,T24506G,G24914C,C27972T,G28048T,A28111G,G28280C,A28281T,T28282A,G28881A,G28882A,G28883C,C28977T	23	N:D3L,N:R203K,N:G204R,N:S235F,ORF1a:T1001I,ORF1a:T1171I,ORF1a:A1708D,ORF1a:A2033T,ORF1a:I2230T,ORF1a:T4304I,ORF1b:P314L,ORF1b:I2634F,ORF8:Q27*,ORF8:R52I,ORF8:Y73C,S:N501Y,S:A570D,S:D614G,S:P681H,S:T716I,S:A831V,S:S982A,S:D1118H	6	1
hCoV-19/Mexico/ZAC_LANGEBIO_IMSS_0305/2021	EPI_ISL_1662230	OL461695	B.1.1.7	GRY	37	C241T,A773G,C913T,C3037T,C3267T,C3777T,C4084T,C5388A,C5986T,G6362A,T6954C,C12970T,C13176T,C14408T,C14676T,C15279T,T16176C,G22627A,A23063T,C23271A,A23403G,C23604A,C23709T,C24023T,C24054T,T24506G,G24914C,C27972T,G28048T,A28111G,G28280C,A28281T,T28282A,G28881A,G28882A,G28883C,C28977T	23	N:D3L,N:R203K,N:G204R,N:S235F,ORF1a:T170A,ORF1a:T1001I,ORF1a:T1171I,ORF1a:A1708D,ORF1a:A2033T,ORF1a:I2230T,ORF1a:T4304I,ORF1b:P314L,ORF8:Q27*,ORF8:R52I,ORF8:Y73C,S:N501Y,S:A570D,S:D614G,S:P681H,S:T716I,S:A831V,S:S982A,S:D1118H	6	1
hCoV-19/Mexico/ZAC_LANGEBIO_IMSS_0704/2021	EPI_ISL_2402118	OL461696	B.1.1.7	GRY	31	C241T,C913T,C3037T,C3267T,G5164T,C5388A,C5986T,T6954C,G14055T,C14408T,C14676T,C15279T,T16176C,A23063T,C23271A,A23403G,C23604A,C23709T,T24506G,G24914C,T27384C,C27972T,G28048T,A28111G,G28280C,A28281T,T28282A,G28881A,G28882A,G28883C,C28977T	19	N:D3L,N:R203K,N:G204R,N:S235F,ORF1a:T1001I,ORF1a:E1633D,ORF1a:A1708D,ORF1a:I2230T,ORF1b:P314L,ORF8:Q27*,ORF8:R52I,ORF8:Y73C,S:N501Y,S:A570D,S:D614G,S:P681H,S:T716I,S:S982A,S:D1118H	6	0
hCoV-19/Mexico/ZAC-IBT_IMSS_11154-NC/2021	EPI_ISL_2801703	OL461697	B.1.1.7	GRY	35	C241T,C913T,C3037T,C3267T,C5388A,C5986T,T6954C,C12141A,C12754T,C14120T,C14408T,C14676T,C15279T,T16176C,T17583G,A23063T,C23271A,A23403G,C23604A,C23709T,T24506G,G24914C,C25624T,C27972T,G28048T,A28095T,A28111G,G28280C,A28281T,T28282A,C28531T,G28881A,G28882A,G28883C,C28977T	23	N:D3L,N:R203K,N:G204R,N:S235F,ORF1a:T1001I,ORF1a:A1708D,ORF1a:I2230T,ORF1a:T3959N,ORF1b:P218L,ORF1b:P314L,ORF3a:H78Y,ORF8:Q27*,ORF8:R52I,ORF8:K68*,ORF8:Y73C,ORF9b:T83I,S:N501Y,S:A570D,S:D614G,S:P681H,S:T716I,S:S982A,S:D1118H	6	953
hCoV-19/Mexico/ZAC-IBT_IMSS_1821/2021	EPI_ISL_2801697	OL461698	B.1.1.7	GRY	37	C222T,C241T,C913T,C3037T,C3267T,C5388A,C5986T,T6954C,G7042T,C8127T,C14120T,C14408T,C14676T,C15279T,T16176C,C17762T,A23063T,C23271A,A23403G,C23604A,C23709T,T24506G,G24914C,T25180C,C25782T,G26109T,C27972T,G28048T,A28095T,A28111G,G28280C,A28281T,T28282A,G28881A,G28882A,G28883C,C28977T	24	N:D3L,N:R203K,N:G204R,N:S235F,ORF1a:T1001I,ORF1a:A1708D,ORF1a:I2230T,ORF1a:M2259I,ORF1a:A2621V,ORF1b:P218L,ORF1b:P314L,ORF1b:A1432V,ORF3a:E239D,ORF8:Q27*,ORF8:R52I,ORF8:K68*,ORF8:Y73C,S:N501Y,S:A570D,S:D614G,S:P681H,S:T716I,S:S982A,S:D1118H	6	479
hCoV-19/Mexico/ZAC-IBT_IMSS_1826/2021	EPI_ISL_2801701	OL461699	B.1.1.7	GRY	38	C241T,T597C,C913T,C1471T,G2144A,C2453T,C3037T,C3267T,C5388A,C5986T,T6954C,C9286T,C9611T,A12547G,C14408T,C14676T,C15279T,T16176C,G18651T,C21855T,A23063T,C23271A,A23403G,C23604A,C23709T,T24506G,G24914C,C27972T,G28048T,A28111G,G28280C,A28281T,T28282A,A28295G,G28881A,G28882A,G28883C,C28977T	25	N:D3L,N:N8D,N:R203K,N:G204R,N:S235F,ORF1a:V111A,ORF1a:V627I,ORF1a:L730F,ORF1a:T1001I,ORF1a:A1708D,ORF1a:I2230T,ORF1a:L3116F,ORF1b:P314L,ORF1b:E1728D,ORF8:Q27*,ORF8:R52I,ORF8:Y73C,S:S98F,S:N501Y,S:A570D,S:D614G,S:P681H,S:T716I,S:S982A,S:D1118H	6	0
hCoV-19/Mexico/ZAC-IBT_IMSS_1827/2021	EPI_ISL_2801702	OL461700	B.1.1.7	GRY	40	C241T,C913T,C2110T,C3037T,C3267T,C5388A,C5986T,T6954C,G7042T,C12141A,C14120T,G14181A,C14408T,C14676T,C15279T,T16176C,T17583G,G17658T,G22103T,G22104T,A23063T,C23271A,A23403G,C23604A,C23709T,T24506G,G24914C,C25624T,C27972T,G28048T,A28095T,A28111G,G28280C,A28281T,T28282A,C28531T,G28881A,G28882A,G28883C,C28977T	26	N:D3L,N:R203K,N:G204R,N:S235F,ORF1a:T1001I,ORF1a:A1708D,ORF1a:I2230T,ORF1a:M2259I,ORF1a:T3959N,ORF1b:P218L,ORF1b:P314L,ORF1b:M1397I,ORF3a:H78Y,ORF8:Q27*,ORF8:R52I,ORF8:K68*,ORF8:Y73C,ORF9b:T83I,S:G181L,S:N501Y,S:A570D,S:D614G,S:P681H,S:T716I,S:S982A,S:D1118H	6	0
hCoV-19/Mexico/ZAC-IBT_IMSS_1831/2021	EPI_ISL_2801706	OL461701	B.1.1.7	GRY	37	C241T,C913T,C2453T,C3037T,C3267T,C5388A,C5986T,T6954C,C9611T,C9893T,C10029T,C10714T,C14408T,C14676T,C15279T,T16176C,C21855T,G21974C,A23063T,C23271A,A23403G,C23604A,C23709T,T24506G,G24914C,C27972T,G28048T,A28111G,G28280C,A28281T,T28282A,A28295G,G28881A,G28882A,G28883C,G28960T,C28977T	26	N:D3L,N:N8D,N:R203K,N:G204R,N:Q229H,N:S235F,ORF1a:L730F,ORF1a:T1001I,ORF1a:A1708D,ORF1a:I2230T,ORF1a:L3116F,ORF1a:L3210F,ORF1a:T3255I,ORF1b:P314L,ORF8:Q27*,ORF8:R52I,ORF8:Y73C,S:S98F,S:D138H,S:N501Y,S:A570D,S:D614G,S:P681H,S:T716I,S:S982A,S:D1118H	6	662
hCoV-19/Mexico/ZAC-LANGEBIO_IMSS_0977/2021	EPI_ISL_2671760	OL461702	B.1.1.7	GRY	37	C913T,C2110T,C3037T,C3267T,C3294A,C5388A,C5986T,T6954C,C12141A,C14120T,C14408T,C14676T,C15279T,T16176C,T17583G,A23063T,C23271A,A23403G,C23604A,C23709T,T24506G,G24914C,C25624T,C27972T,G28048T,A28095T,A28111G,G28280C,A28281T,T28282A,C28531T,G28881A,G28882A,G28883C,C28977T,C29007G,G29254T	25	N:D3L,N:R203K,N:G204R,N:S235F,N:T245S,ORF1a:T1001I,ORF1a:P1010H,ORF1a:A1708D,ORF1a:I2230T,ORF1a:T3959N,ORF1b:P218L,ORF1b:P314L,ORF3a:H78Y,ORF8:Q27*,ORF8:R52I,ORF8:K68*,ORF8:Y73C,ORF9b:T83I,S:N501Y,S:A570D,S:D614G,S:P681H,S:T716I,S:S982A,S:D1118H	6	28

hCoV-19/Mexico/ZAC-INER-IMSS-00023/2021	EPI_ISL_1287761	-	B.1.1.7	GRY	35	C241T,C913T,C3037T,C3267T,C5388A,C5986T,G6362A,T6954C,C12970T,C13176T,C14408T,C14676T,C15279T,T16176C,C17977T,A20013G,A23063T,C23271A,A23403G,C23604A,C23709T,C24023T,C24054T,T24506G,G24914C,C27972T,G28048T,A28111G,G28280C,A28281T,T28282A,G28881A,G28882A,G28883C,C28977T	22	N:D3L,N:R203K,N:G204R,N:S235F,ORF1a:T1001I,ORF1a:A1708D,ORF1a:A2033T,ORF1a:I2230T,ORF1a:T4304I,ORF1b:P314L,ORF1b:L1504F,ORF8:Q27*,ORF8:R52I,ORF8:Y73C,S:N501Y,S:A570D,S:D614G,S:P681H,S:T716I,S:A831V,S:S982A,S:D1118H	6	0
hCoV-19/Mexico/ZAC-INER-IMSS-00026/2021	EPI_ISL_1279265	-	B.1.1.7	GRY	35	C241T,C913T,C3037T,C3267T,C5388A,C5986T,G6362A,T6954C,C12970T,C13176T,C14408T,C14676T,C15279T,T16176C,T17214C,C17977T,A23063T,C23271A,A23403G,C23604A,C23709T,C24023T,C24054T,T24506G,G24914C,C27972T,G28048T,A28111G,G28280C,A28281T,T28282A,G28881A,G28882A,G28883C,C28977T	22	N:D3L,N:R203K,N:G204R,N:S235F,ORF1a:T1001I,ORF1a:A1708D,ORF1a:A2033T,ORF1a:I2230T,ORF1a:T4304I,ORF1b:P314L,ORF1b:L1504F,ORF8:Q27*,ORF8:R52I,ORF8:Y73C,S:N501Y,S:A570D,S:D614G,S:P681H,S:T716I,S:A831V,S:S982A,S:D1118H	6	0
hCoV-19/Mexico/ZAC-INER-IMSS-00050/2021	EPI_ISL_1287762	-	B.1.1.7	GRY	35	C241T,C913T,C3037T,C3267T,C5388A,C5986T,G6362A,T6954C,C12970T,C13176T,C14408T,C14676T,C15279T,T16176C,C17977T,A20013G,A23063T,C23271A,A23403G,C23604A,C23709T,C24023T,C24054T,T24506G,G24914C,C27972T,G28048T,A28111G,G28280C,A28281T,T28282A,G28881A,G28882A,G28883C,C28977T	22	N:D3L,N:R203K,N:G204R,N:S235F,ORF1a:T1001I,ORF1a:A1708D,ORF1a:A2033T,ORF1a:I2230T,ORF1a:T4304I,ORF1b:P314L,ORF1b:L1504F,ORF8:Q27*,ORF8:R52I,ORF8:Y73C,S:N501Y,S:A570D,S:D614G,S:P681H,S:T716I,S:A831V,S:S982A,S:D1118H	6	0
hCoV-19/Mexico/ZAC-INER-IMSS-00059/2021	EPI_ISL_1287763	-	B.1.1.7	GRY	36	C241T,T356C,C913T,C3037T,C3267T,C5388A,C5986T,G6362A,T6954C,C12970T,C13176T,C14408T,C14676T,C15279T,T16176C,C17977T,A20013G,A23063T,C23271A,A23403G,C23604A,C23709T,C24023T,C24054T,T24506G,G24914C,C27972T,G28048T,A28111G,G28280C,A28281T,T28282A,G28881A,G28882A,G28883C,C28977T	23	N:D3L,N:R203K,N:G204R,N:S235F,ORF1a:F31L,ORF1a:T1001I,ORF1a:A1708D,ORF1a:A2033T,ORF1a:I2230T,ORF1a:T4304I,ORF1b:P314L,ORF1b:L1504F,ORF8:Q27*,ORF8:R52I,ORF8:Y73C,S:N501Y,S:A570D,S:D614G,S:P681H,S:T716I,S:A831V,S:S982A,S:D1118H	6	0
hCoV-19/Mexico/ZAC-INER-IMSS-00078/2021	EPI_ISL_1287764	-	B.1.1.7	GRY	35	C241T,C913T,C3037T,C3267T,G4960T,C5140A,C6027T,A8413G,C12970T,C13176T,C14408T,C14676T,C15279T,T16176C,C17977T,A20013G,A23063T,C23271A,A23403G,C23604A,C23709T,C24023T,C24054T,T24506G,G24914C,C27972T,G28048T,A28111G,G28280C,A28281T,T28282A,G28881A,G28882A,G28883C,C28977T	21	N:D3L,N:R203K,N:G204R,N:S235F,ORF1a:T1001I,ORF1a:D1625E,ORF1a:P1921L,ORF1a:T4304I,ORF1b:P314L,ORF1b:L1504F,ORF8:Q27*,ORF8:R52I,ORF8:Y73C,S:N501Y,S:A570D,S:D614G,S:P681H,S:T716I,S:A831V,S:S982A,S:D1118H	6	0
hCoV-19/Mexico/ZAC-INER-IMSS-00079/2021	EPI_ISL_1287765	-	B.1.1.7	GRY	35	C241T,C913T,C3037T,C3267T,C5388A,C5986T,G6362A,T6954C,C12970T,C13176T,C14408T,C14676T,C15279T,T16176C,C17977T,A20013G,A23063T,C23271A,A23403G,C23604A,C23709T,C24023T,C24054T,T24506G,G24914C,C27972T,G28048T,A28111G,G28280C,A28281T,T28282A,G28881A,G28882A,G28883C,C28977T	22	N:D3L,N:R203K,N:G204R,N:S235F,ORF1a:T1001I,ORF1a:A1708D,ORF1a:A2033T,ORF1a:I2230T,ORF1a:T4304I,ORF1b:P314L,ORF1b:L1504F,ORF8:Q27*,ORF8:R52I,ORF8:Y73C,S:N501Y,S:A570D,S:D614G,S:P681H,S:T716I,S:A831V,S:S982A,S:D1118H	6	0
hCoV-19/Mexico/ZAC-INER-IMSS-00086/2021	EPI_ISL_1287767	-	B.1.1.7	GRY	34	C241T,C913T,C3037T,C3267T,C5388A,C5986T,G6362A,T6954C,C12970T,C13176T,C14408T,C14676T,C15279T,T16176C,C17977T,A23063T,C23271A,A23403G,C23604A,C23709T,C24023T,C24054T,T24506G,G24914C,C27972T,G28048T,A28111G,G28280C,A28281T,T28282A,G28881A,G2	22	N:D3L,N:R203K,N:G204R,N:S235F,ORF1a:T1001I,ORF1a:A1708D,ORF1a:A2033T,ORF1a:I2230T,ORF1a:T4304I,ORF1b:P314L,ORF1b:L1504F,ORF8:Q27*,ORF8:R52I,ORF8:Y73C,S:N501Y,S:A570D,S:D614G,S:P681H,S:T716I,S:A831V,S:S98	6	0
hCoV-19/Mexico/BCN-CICESE-M1409/2021	EPI_ISL_2455241	-	Q.3	GRY	39	C241T,C913T,C3037T,C3267T,G5023A,C5388A,G5437T,C5986T,C6568T,T6954C,A9529G,G10396T,C14408T,C14676T,C15081T,C15279T,T16176C,A17615G,G20962A,G22094C,A23063T,C23271A,A23403G,C23604A,C23709T,T24506G,G24914C,C26110T,G26730C,C27972T,G28048T,A28111G,G28280C,A28281T,T28282A,G28881A,G28882A,G28883C,C28977T	25	M:V70L,N:D3L,N:R203K,N:G204R,N:S235F,ORF1a:T1001I,ORF1a:M1586I,ORF1a:A1708D,ORF1a:E1724D,ORF1a:I2230T,ORF1b:P314L,ORF1b:K1383R,ORF1b:D2499N,ORF3a:P240S,ORF8:Q27*,ORF8:R52I,ORF8:Y73C,S:D178H,S:N501Y,S:A570D,S:D614G,S:P681H,S:T716I,S:S982A,S:D1118H	6	0
hCoV-19/Mexico/BCN-CICESE-M1411/2021	EPI_ISL_2455242	-	Q.3	GRY	38	C241T,C913T,C3037T,C3267T,G5023A,C5388A,C5986T,C6568T,T6954C,A9529G,G10396T,C14408T,C14676T,C15081T,C15279T,T16176C,A17615G,G20962A,G22094C,A23063T,C23271A,A23403G,C23604A,C23709T,T24506G,G24914C,C26110T,G26730C,C27972T,G28048T,A28111G,G28280C,A28281T,T28282A,G28881A,G28882A,G28883C,C28977T	24	M:V70L,N:D3L,N:R203K,N:G204R,N:S235F,ORF1a:T1001I,ORF1a:M1586I,ORF1a:A1708D,ORF1a:I2230T,ORF1b:P314L,ORF1b:K1383R,ORF1b:D2499N,ORF3a:P240S,ORF8:Q27*,ORF8:R52I,ORF8:Y73C,S:D178H,S:N501Y,S:A570D,S:D614G,S:P681H,S:T716I,S:S982A,S:D1118H	6	15
hCoV-19/Mexico/BCN-InDRE_FB13738_S1741/2021	EPI_ISL_2101880	-	B.1.1.7	GRY	43	C241T,C835T,C913T,C2110T,G2272T,G2782T,C3037T,C3267T,C4113T,C4456T,C5388A,C5986T,T6954C,C8266T,G11312A,C14120T,C14408T,C14676T,C15279T,T16176C,C16733T,C19390T,G22094T,A23063T,C23271A,A23403G,C23604A,C23709T,T24506G,C24904T,G24914C,A25336C,C27972T,G28048T,A28111G,G28280C,A28281T,T28282A,G28881A,G28882A,G28883C,C28932T,C	27	N:D3L,N:R203K,N:G204R,N:A220V,N:S235F,ORF1a:K669N,ORF1a:T1001I,ORF1a:A1283V,ORF1a:A1708D,ORF1a:I2230T,ORF1a:V3683I,ORF1b:P218L,ORF1b:P314L,ORF1b:S1089L,ORF1b:P1975S,ORF8:Q27*,ORF8:R52I,ORF8:Y73C,S:D178Y,S:N501Y,S:A570D,S:D614G,S:P681H,S:T716I,S:S982A,S:D1118H,S:E1258D	6	0
hCoV-19/Mexico/BCN-InDRE_FB16371_S2477/2021	EPI_ISL_2455998	-	B.1.1.7	GRY	44	C241T,C835T,C913T,C2110T,G2272T,G2782T,C3037T,C3267T,C4113T,C4456T,C5388A,C5806T,C5986T,T6954C,G6986A,G11312A,C14120T,C14408T,C14676T,C15279T,T16176C,C16608T,C16733T,C19390T,C19586T,G22094T,A23063T,C23271A,A23403G,C23604A,C23709T,T24506G,C24904T,G24914C,C27972T,G28048T,A28111G,G28280C,A28281T,T28282A,G28881A,G28882A,G2	27	N:D3L,N:R203K,N:G204R,N:S235F,ORF1a:K669N,ORF1a:T1001I,ORF1a:A1283V,ORF1a:A1708D,ORF1a:I2230T,ORF1a:G2241S,ORF1a:V3683I,ORF1b:P218L,ORF1b:P314L,ORF1b:S1089L,ORF1b:P1975S,ORF1b:T2040I,ORF8:Q27*,ORF8:R52I,ORF8:Y73C,S:D178Y,S:N501Y,S:A570D,S:D614G,S:P681H,S:T716I,S:S982A	6	0
hCoV-19/Mexico/BCN-InDRE_FB16375_S2478/2021	EPI_ISL_2455999	-	B.1.1.7	GRY	41	C241T,C835T,C913T,C2110T,G2272T,G2782T,C3037T,C3267T,C4113T,C4456T,C5388A,C5986T,T6954C,G11312A,C14120T,C14408T,C14676T,C15279T,T16176C,C16733T,C19390T,G22094T,A23063T,C23271A,A23403G,C23604A,C23709T,T24506G,C24904T,G24914C,A25336C,C27972T,G28048T,A28111G,G28280C,A28281T,T28282A,G28881A,G28882A,G28883C,C28977T	26	N:D3L,N:R203K,N:G204R,N:S235F,ORF1a:K669N,ORF1a:T1001I,ORF1a:A1283V,ORF1a:A1708D,ORF1a:I2230T,ORF1a:V3683I,ORF1b:P218L,ORF1b:P314L,ORF1b:S1089L,ORF1b:P1975S,ORF8:Q27*,ORF8:R52I,ORF8:Y73C,S:D178Y,S:N501Y,S:A570D,S:D614G,S:P681H,S:T716I,S:S982A,S:D1118H,S:E1258D	6	0
hCoV-19/Mexico/BCN-InDRE_FB16376_S2479/2021	EPI_ISL_2456000	-	Q.3	GRY	40	C241T,C913T,C3037T,C3267T,G5023A,C5388A,C5986T,C6568T,T6954C,A9529G,G10396T,C14408T,C14676T,C15279T,T16176C,A17615G,C18131T,G18984T,G20962A,G22094C,A23063T,C23271A,A23403G,C23604A,C23709T,C24378T,T24506G,G24914C,A25336C,G26730C,C27972T,G28048T,A28111G,G28280C,A28281T,T28282A,G28881A,G28882A,G28883C,C28977T	27	M:V70L,N:D3L,N:R203K,N:G204R,N:S235F,ORF1a:T1001I,ORF1a:M1586I,ORF1a:A1708D,ORF1a:I2230T,ORF1b:P314L,ORF1b:K1383R,ORF1b:T1555I,ORF1b:M1839I,ORF1b:D2499N,ORF8:Q27*,ORF8:R52I,ORF8:Y73C,S:D178H,S:N501Y,S:A570D,S:D614G,S:P681H,S:T716I,S:S939F,S:S982A,S:D1118H,S:E1258D	6	0
hCoV-19/Mexico/BCN-InDRE_FB17815_S4114/2021	EPI_ISL_2920685	-	B.1.1.7	GRY	45	C241T,C835T,C913T,C2110T,G2272T,G2782T,C3037T,C3267T,C4113T,C4456T,C5388A,C5986T,T6954C,G11312A,G12661A,T13180C,C14120T,C14408T,C14676T,C15279T,T16176C,C16733T,C19390T,G20383A,G22094T,A23063T,C23271A,A23403G,C23604A,C23709T,T24506G,C24904T,G24914C,C26882T,C27972T,G28048T,A28111G,G28280C,A28281T,T28282A,G28881A,G28882A,	26	N:D3L,N:R203K,N:G204R,N:S235F,ORF1a:K669N,ORF1a:T1001I,ORF1a:A1283V,ORF1a:A1708D,ORF1a:I2230T,ORF1a:V3683I,ORF1b:P218L,ORF1b:P314L,ORF1b:S1089L,ORF1b:P1975S,ORF1b:A2306T,ORF8:Q27*,ORF8:R52I,ORF8:Y73C,S:D178Y,S:N501Y,S:A570D,S:D614G,S:P681H,S:T716I,S:S982A,S:D1118H	6	0
hCoV-19/Mexico/BCN-InDRE_FB18286_S3966/2021	EPI_ISL_2858980	-	B.1.1.7	GRY	42	C241T,C841T,C913T,C3037T,C3267T,A4501C,C5388A,C5986T,A6366G,T6954C,C14408T,C14676T,T15096C,C15279T,T16176C,T19541C,C22993A,A23063T,C23271A,A23403G,C23604A,C23709T,T24506G,C24789T,G24914C,T25027C,A25336C,G25855T,G26428C,C27418T,C27972T,G28048T,A28111G,G28280C,A28281T,T28282A,C28606T,G28881A,G28882A,G28883C,G28884C,C28	26	E:V62L,N:D3L,N:R203K,N:G204P,N:S235F,ORF1a:T1001I,ORF1a:Q1412H,ORF1a:A1708D,ORF1a:Q2034R,ORF1a:I2230T,ORF1b:P314L,ORF1b:M2025T,ORF3a:D155Y,ORF8:Q27*,ORF8:R52I,ORF8:Y73C,S:S477R,S:N501Y,S:A570D,S:D614G,S:P681H,S:T716I,S:S982A,S:T1076I,S:D1118H,S:E1258D	6	0
hCoV-19/Mexico/BCN-InDRE_FB19191_S4500/2021	EPI_ISL_3033354	-	B.1.1.7	GRY	44	C241T,C835T,C913T,A1558G,C2110T,G2272T,G2782T,C3037T,C3267T,C4113T,C4456T,C5388A,C5806T,C5986T,T6954C,G11312A,C14120T,C14408T,C14676T,C15279T,T16176C,C16608T,C16733T,C19390T,G22094T,A23063T,C23271A,A23403G,C23604A,C23709T,T24506G,C24904T,G24914C,C27972T,G28048T,A28095T,A28111G,G28280C,A28281T,T28282A,G28881A,G28882A,G	27	N:D3L,N:R203K,N:G204R,N:S235F,ORF1a:I431M,ORF1a:K669N,ORF1a:T1001I,ORF1a:A1283V,ORF1a:A1708D,ORF1a:I2230T,ORF1a:V3683I,ORF1b:P218L,ORF1b:P314L,ORF1b:S1089L,ORF1b:P1975S,ORF8:Q27*,ORF8:R52I,ORF8:K68*,ORF8:Y73C,S:D178Y,S:N501Y,S:A570D,S:D614G,S:P681H,S:T716I,S:S982A,S:D1	6	0
hCoV-19/Mexico/BCS-InDRE_FB17119_S3086/2021	EPI_ISL_2663353	-	B.1.1.7	GRY	30	C241T,C913T,C3037T,C3267T,C5388A,C5986T,T6954C,C14408T,C14676T,C15279T,T16176C,G22427T,A23063T,C23271A,A23403G,C23604A,C23709T,T24506G,G24914C,G25690T,C27972T,G28048T,A28111G,G28262A,A28352G,G28396T,G28881A,G28882A,G28883C,C28977T	21	N:N27D,N:R203K,N:G204R,N:S235F,ORF1a:T1001I,ORF1a:A1708D,ORF1a:I2230T,ORF1b:P314L,ORF3a:G100C,ORF8:Q27*,ORF8:R52I,ORF8:Y73C,ORF9b:G38V,S:V289L,S:N501Y,S:A570D,S:D614G,S:P681H,S:T716I,S:S982A,S:D1118H	6	0
hCoV-19/Mexico/BCS-InDRE_FB19249_S4501/2021	EPI_ISL_3033355	-	B.1.1.7	GRY	38	C241T,C913T,C2110T,C3037T,C3267T,C5388A,C5986T,T6954C,G7745A,T11522G,C14120T,C14408T,C14676T,C15279T,T16176C,C19390T,C21855T,A23063T,C23271A,A23403G,C23604A,C23709T,T24506G,G24914C,G25993C,C27972T,G28048T,A28095T,A28111G,G28280C,A28281T,T28282A,G28881A,G28882A,G28883C,C28977T,G29260T,G29540T	25	N:D3L,N:R203K,N:G204R,N:S235F,ORF1a:T1001I,ORF1a:A1708D,ORF1a:I2230T,ORF1a:V2494I,ORF1a:F3753V,ORF1b:P218L,ORF1b:P314L,ORF1b:P1975S,ORF3a:V201L,ORF8:Q27*,ORF8:R52I,ORF8:K68*,ORF8:Y73C,S:S98F,S:N501Y,S:A570D,S:D614G,S:P681H,S:T716I,S:S982A,S:D1118H	6	0
hCoV-19/Mexico/BCS-InDRE_FB19255_S4502/2021	EPI_ISL_3033356	-	B.1.1.7	GRY	37	T137C,C241T,C703T,C913T,C3037T,C3267T,C5388A,C5986T,T6954C,G7798T,C8739T,C14408T,C14676T,C15279T,T16176C,T17589C,G19677T,A23063T,C23086T,C23271A,A23403G,C23604A,C23709T,T24506G,G24914C,C26833T,C27972T,G28048T,A28111G,G28280C,A28281T,T28282A,G28881A,G28882A,G28883C,C28977T,C29614T	22	M:A104V,N:D3L,N:R203K,N:G204R,N:S235F,ORF1a:T1001I,ORF1a:A1708D,ORF1a:I2230T,ORF1a:K2511N,ORF1a:T2825I,ORF1b:P314L,ORF1b:Q2070H,ORF8:Q27*,ORF8:R52I,ORF8:Y73C,S:N501Y,S:A570D,S:D614G,S:P681H,S:T716I,S:S982A,S:D1118H	6	0

hCoV-19/Mexico/CAM-InDRE_FB14881_S2388/2021	EPI_ISL_2455913	-	B.1.1.7	GRY	36	C241T,C913T,C1063T,C3037T,C3267T,C5388A,C5986T,G6922A,T6954C,C11750T,T12313C,C13115T,C14408T,C14676T,C15279T,T16176C,A23063T,C23271A,A23403G,C23604A,C23709T,T24506G,G24914C,A25336C,C26270T,C27972T,G28048T,C28087T,A28111G,G28280C,A28281T,T28282A,G28881A,G28882A,G28883C,C28977T	22	E:T9I,N:D3L,N:R203K,N:G204R,N:S235F,ORF1a:T1001I,ORF1a:A1708D,ORF1a:I2230T,ORF1a:L3829F,ORF1b:P314L,ORF8:Q27*,ORF8:R52I,ORF8:A65V,ORF8:Y73C,S:N501Y,S:A570D,S:D614G,S:P681H,S:T716I,S:S982A,S:D1118H,S:E1258D	6	49
hCoV-19/Mexico/CAM-InDRE_FB14886_S2047/2021	EPI_ISL_2283689	-	B.1.1.7	GRY	36	C241T,C913T,C1960T,C2453T,C3037T,C3267T,C5388A,C5986T,T6954C,C9611T,C14408T,C14676T,C15279T,T16176C,C20930T,C21110T,C21855T,G21974C,A23063T,C23271A,A23403G,C23604A,C23709T,T24506G,G24914C,C27972T,G28048T,A28111G,G28280C,A28281T,T28282A,A28295G,G28881A,G28882A,G28883C,C28977T	25	N:D3L,N:N8D,N:R203K,N:G204R,N:S235F,ORF1a:L730F,ORF1a:T1001I,ORF1a:A1708D,ORF1a:I2230T,ORF1a:L3116F,ORF1b:P314L,ORF1b:T2488M,ORF1b:T2548I,ORF8:Q27*,ORF8:R52I,ORF8:Y73C,S:S98F,S:D138H,S:N501Y,S:A570D,S:D614G,S:P681H,S:T716I,S:S982A,S:D1118H	6	0
hCoV-19/Mexico/CAM-InDRE_FB17371_S3924/2021	EPI_ISL_2858938	-	B.1.1.7	GRY	31	C241T,C2453T,C3037T,C3267T,C5388A,C5986T,C6033T,T6954C,C9611T,C14408T,C14676T,C15279T,T16176C,C17573T,C21855T,G21974C,A23063T,C23271A,A23403G,C23604A,C23709T,T24506G,C25609T,C27972T,G28048T,A28111G,A28295G,G28881A,G28882A,G28883C,C28977T	24	N:N8D,N:R203K,N:G204R,N:S235F,ORF1a:L730F,ORF1a:T1001I,ORF1a:A1708D,ORF1a:A1923V,ORF1a:I2230T,ORF1a:L3116F,ORF1b:P314L,ORF1b:A1369V,ORF3a:L73F,ORF8:Q27*,ORF8:R52I,ORF8:Y73C,S:S98F,S:D138H,S:N501Y,S:A570D,S:D614G,S:P681H,S:T716I,S:S982A	6	714
hCoV-19/Mexico/CAM-InDRE_FB17391_S3486/2021	EPI_ISL_2779001	-	B.1.1.7	GRY	40	C222T,C241T,C913T,C2110T,C3037T,C3267T,C5388A,C5986T,T6954C,G7042T,C14120T,C14408T,C14676T,C15279T,T16176C,C17762T,A21555G,A23063T,C23271A,A23403G,C23604A,C23709T,T24506G,G24914C,T25180C,A25336C,G25337C,C25844T,C26270T,G27589T,C27972T,G28048T,A28111G,G28280C,A28281T,T28282A,G28881A,G28882A,G28883C,C28977T	26	E:T9I,N:D3L,N:R203K,N:G204R,N:S235F,ORF1a:T1001I,ORF1a:A1708D,ORF1a:I2230T,ORF1a:M2259I,ORF1b:P218L,ORF1b:P314L,ORF1b:A1432V,ORF3a:T151I,ORF7a:A66S,ORF8:Q27*,ORF8:R52I,ORF8:Y73C,S:N501Y,S:A570D,S:D614G,S:P681H,S:T716I,S:S982A,S:D1118H,S:E1258D,S:D1259H	6	0
hCoV-19/Mexico/CAM-InDRE_FB18927_S4478/2021	EPI_ISL_3033332	-	B.1.1.7	GRY	39	C241T,C913T,C2110T,C3037T,C3267T,C5388A,C5986T,T6954C,G7042T,T9504C,A10195G,C10279T,C14120T,C14408T,C14676T,C15279T,T16176C,C16887T,G17427T,A23063T,C23271A,A23403G,C23604A,C23709T,T24506G,G24914C,A27943G,C27972T,G28048T,G28075T,A28095T,A28111G,G28280C,A28281T,T28282A,G28881A,G28882A,G28883C,C28977T	24	N:D3L,N:R203K,N:G204R,N:S235F,ORF1a:T1001I,ORF1a:A1708D,ORF1a:I2230T,ORF1a:M2259I,ORF1a:F3080S,ORF1b:P218L,ORF1b:P314L,ORF8:H17R,ORF8:Q27*,ORF8:R52I,ORF8:C61F,ORF8:K68*,ORF8:Y73C,S:N501Y,S:A570D,S:D614G,S:P681H,S:T716I,S:S982A,S:D1118H	6	0
hCoV-19/Mexico/CAM-InDRE_FB18929_S4479/2021	EPI_ISL_3033333	-	B.1.1.7	GRY	43	C241T,C913T,C2110T,C2189T,C2862T,C3037T,C3267T,C5388A,C5986T,T6954C,G7042T,A10195G,C14120T,C14408T,C14676T,C14805T,C15279T,C15714T,T16176C,T16542C,C18788T,A23063T,C23271A,A23403G,C23604A,C23709T,T24506G,G24914C,C26058T,G26450T,C27972T,G28048T,G28075T,A28095T,A28111G,G28280C,A28281T,T28282A,G28881A,G28882A,G28883C,C2897	27	E:R69I,N:D3L,N:R203K,N:G204R,N:S235F,N:T334I,ORF1a:L642F,ORF1a:T866I,ORF1a:T1001I,ORF1a:A1708D,ORF1a:I2230T,ORF1a:M2259I,ORF1b:P218L,ORF1b:P314L,ORF1b:T1774I,ORF8:Q27*,ORF8:R52I,ORF8:C61F,ORF8:K68*,ORF8:Y73C,S:N501Y,S:A570D,S:D614G,S:P681H,S:T716I,S:S982A,S:D1118H	6	115
hCoV-19/Mexico/CAM-InDRE_FB18939_S4480/2021	EPI_ISL_3033334	-	B.1.1.7	GRY	39	C241T,C823T,C913T,C1758T,C2110T,G3004T,C3037T,C3267T,C3874T,C5388A,C5986T,T6954C,G7042T,C8964T,C9559T,C14120T,C14408T,C14676T,C15279T,T16176C,A23063T,C23271A,A23403G,C23604A,C23709T,T24506G,G24914C,C26110T,C27972T,G28048T,A28095T,A28111G,G28280C,A28281T,T28282A,G28881A,G28882A,G28883C,C28977T	25	N:D3L,N:R203K,N:G204R,N:S235F,ORF1a:A498V,ORF1a:E913D,ORF1a:T1001I,ORF1a:A1708D,ORF1a:I2230T,ORF1a:M2259I,ORF1a:S2900L,ORF1b:P218L,ORF1b:P314L,ORF3a:P240S,ORF8:Q27*,ORF8:R52I,ORF8:K68*,ORF8:Y73C,S:N501Y,S:A570D,S:D614G,S:P681H,S:T716I,S:S982A,S:D1118H	6	0
hCoV-19/Mexico/CHH-InDRE_F11326_S902/2021	EPI_ISL_1423512	-	B.1.1.7	GRY	30	C241T,C913T,T1147C,C3037T,C3267T,C5388A,C5986T,T6954C,A11260G,C14408T,C14676T,C15279T,T16176C,A23063T,C23271A,A23403G,C23604A,C23709T,T24506G,G24914C,C27972T,G28048T,A28111G,G28280C,A28281T,T28282A,G28881A,G28882A,G28883C,C28977T	18	N:D3L,N:R203K,N:G204R,N:S235F,ORF1a:T1001I,ORF1a:A1708D,ORF1a:I2230T,ORF1b:P314L,ORF8:Q27*,ORF8:R52I,ORF8:Y73C,S:N501Y,S:A570D,S:D614G,S:P681H,S:T716I,S:S982A,S:D1118H	6	0
hCoV-19/Mexico/CHH-InDRE_F11642_S896/2021	EPI_ISL_1423506	-	B.1.1.7	GR	29	C241T,C913T,C3037T,C3267T,C5388A,C5986T,T6954C,C9298T,C14408T,C14676T,C15279T,T16176C,C23271A,A23403G,C23604A,C23709T,C24374T,T24506G,G24914C,C27972T,G28048T,A28111G,G28280C,A28281T,T28282A,G28881A,G28882A,G28883C,C28977T	18	N:D3L,N:R203K,N:G204R,N:S235F,ORF1a:T1001I,ORF1a:A1708D,ORF1a:I2230T,ORF1b:P314L,ORF8:Q27*,ORF8:R52I,ORF8:Y73C,S:A570D,S:D614G,S:P681H,S:T716I,S:L938F,S:S982A,S:D1118H	6	747
hCoV-19/Mexico/CHH-InDRE_F11646_S897/2021	EPI_ISL_1423507	-	B.1.1.7	GRY	31	C241T,C913T,C3037T,C3267T,C5388A,C5986T,T6954C,C11750T,G13201T,C14408T,C14676T,C15279T,T16176C,A23063T,C23271A,A23403G,C23604A,C23709T,T24506G,G24914C,C26270T,C27972T,G28048T,A28111G,G28280C,A28281T,T28282A,G28881A,G28882A,G28883C,C28977T	20	E:T9I,N:D3L,N:R203K,N:G204R,N:S235F,ORF1a:T1001I,ORF1a:A1708D,ORF1a:I2230T,ORF1a:L3829F,ORF1b:P314L,ORF8:Q27*,ORF8:R52I,ORF8:Y73C,S:N501Y,S:A570D,S:D614G,S:P681H,S:T716I,S:S982A,S:D1118H	6	68
hCoV-19/Mexico/CHH-InDRE_F11654_S898/2021	EPI_ISL_1423508	-	B.1.1.7	GR	31	C241T,C913T,C3037T,C3267T,C5388A,C5986T,T6954C,C11750T,C14408T,C14676T,C15279T,T16176C,C20629T,C23271A,A23403G,C23604A,C23709T,T24506G,G24914C,C26270T,C27972T,G28048T,A28111G,G28280C,A28281T,T28282A,G28881A,G28882A,G28883C,C28977T,C29520T	21	E:T9I,N:D3L,N:R203K,N:G204R,N:S235F,N:S416L,ORF1a:T1001I,ORF1a:A1708D,ORF1a:I2230T,ORF1a:L3829F,ORF1b:P314L,ORF1b:H2388Y,ORF8:Q27*,ORF8:R52I,ORF8:Y73C,S:A570D,S:D614G,S:P681H,S:T716I,S:S982A,S:D1118H	6	1140
hCoV-19/Mexico/CHH-InDRE_F11656_S899/2021	EPI_ISL_1423509	-	B.1.1.7	GR	28	C241T,C913T,C3037T,C3267T,C5388A,C5986T,T6954C,C14408T,C14676T,C15279T,T16176C,C23271A,A23403G,C23604A,C23709T,C24130T,T24506G,G24914C,C27972T,G28048T,A28111G,G28280C,A28281T,T28282A,G28881A,G28882A,G28883C,C28977T	17	N:D3L,N:R203K,N:G204R,N:S235F,ORF1a:T1001I,ORF1a:A1708D,ORF1a:I2230T,ORF1b:P314L,ORF8:Q27*,ORF8:R52I,ORF8:Y73C,S:A570D,S:D614G,S:P681H,S:T716I,S:S982A,S:D1118H	6	765
hCoV-19/Mexico/CHH-InDRE_F12155_S1187/2021	EPI_ISL_1626794	-	B.1.1.7	GRY	31	C241T,C913T,C3037T,C3267T,C5388A,C5986T,T6954C,C11750T,C14408T,C14676T,C15279T,T16176C,A23063T,C23271A,A23403G,C23604A,C23709T,T24506G,G24914C,C26270T,C27972T,G28048T,A28111G,G28280C,A28281T,T28282A,G28881A,G28882A,G28883C,C28977T,G29810T	20	E:T9I,N:D3L,N:R203K,N:G204R,N:S235F,ORF1a:T1001I,ORF1a:A1708D,ORF1a:I2230T,ORF1a:L3829F,ORF1b:P314L,ORF8:Q27*,ORF8:R52I,ORF8:Y73C,S:N501Y,S:A570D,S:D614G,S:P681H,S:T716I,S:S982A,S:D1118H	6	0
hCoV-19/Mexico/CHH-InDRE_F12156_S1188/2021	EPI_ISL_1626795	-	B.1.1.7	GRY	31	C241T,G271T,C913T,C3037T,C3267T,C5388A,C5986T,T6954C,C14408T,C14676T,C15279T,T16176C,C21639A,A23063T,C23271A,A23403G,C23604A,C23709T,C24374T,T24506G,G24914C,C27972T,G28048T,A28111G,G28280C,A28281T,T28282A,G28881A,G28882A,G28883C,C28977T	21	N:D3L,N:R203K,N:G204R,N:S235F,ORF1a:E2D,ORF1a:T1001I,ORF1a:A1708D,ORF1a:I2230T,ORF1b:P314L,ORF8:Q27*,ORF8:R52I,ORF8:Y73C,S:P26H,S:N501Y,S:A570D,S:D614G,S:P681H,S:T716I,S:L938F,S:S982A,S:D1118H	6	55
hCoV-19/Mexico/CHH-InDRE_F12158_S1189/2021	EPI_ISL_1626796	-	B.1.1.7	GRY	28	C241T,C913T,C3037T,C3267T,C5388A,C5986T,T6954C,C14408T,C14676T,C15279T,T16176C,A23063T,C23271A,A23403G,C23604A,C23709T,T24506G,G24914C,C27972T,G28048T,A28111G,G28280C,A28281T,T28282A,G28881A,G28882A,G28883C,C28977T	18	N:D3L,N:R203K,N:G204R,N:S235F,ORF1a:T1001I,ORF1a:A1708D,ORF1a:I2230T,ORF1b:P314L,ORF8:Q27*,ORF8:R52I,ORF8:Y73C,S:N501Y,S:A570D,S:D614G,S:P681H,S:T716I,S:S982A,S:D1118H	6	0
hCoV-19/Mexico/CHH-InDRE_F12160_S1190/2021	EPI_ISL_1626797	-	B.1.1.7	GRY	32	C241T,C913T,C3037T,C3267T,C5388A,C5986T,T6954C,C7420T,C11750T,G13201T,C14408T,C14676T,C15279T,T16176C,A23063T,C23271A,A23403G,C23604A,C23709T,T24506G,G24914C,C26270T,C27972T,G28048T,A28111G,G28280C,A28281T,T28282A,G28881A,G28882A,G28883C,C	20	E:T9I,N:D3L,N:R203K,N:G204R,N:S235F,ORF1a:T1001I,ORF1a:A1708D,ORF1a:I2230T,ORF1a:L3829F,ORF1b:P314L,ORF8:Q27*,ORF8:R52I,ORF8:Y73C,S:N501Y,S:A570D,S:D614G,S:P681H,S:T716I,S:S982A,S:D1118H	6	0
hCoV-19/Mexico/CHH-InDRE_F12161_S1191/2021	EPI_ISL_1626798	-	B.1.1.7	GRY	30	C241T,C913T,C3037T,C3267T,C5388A,C5986T,T6954C,C9298T,C14408T,C14676T,C15279T,T16176C,A23063T,C23271A,A23403G,C23604A,C23709T,C24374T,T24506G,G24914C,C27972T,G28048T,A28111G,G28280C,A28281T,T28282A,G28881A,G28882A,G28883C,C28977T	19	N:D3L,N:R203K,N:G204R,N:S235F,ORF1a:T1001I,ORF1a:A1708D,ORF1a:I2230T,ORF1b:P314L,ORF8:Q27*,ORF8:R52I,ORF8:Y73C,S:N501Y,S:A570D,S:D614G,S:P681H,S:T716I,S:L938F,S:S982A,S:D1118H	6	0
hCoV-19/Mexico/CHH-InDRE_F12165_S1192/2021	EPI_ISL_1626799	-	B.1.1.7	GRY	32	C241T,C913T,C3037T,C3267T,C5388A,C5986T,T6954C,C8139T,C9298T,C14408T,C14676T,C15279T,T16176C,A23063T,C23271A,A23403G,C23604A,C23709T,A24123G,C24374T,T24506G,G24914C,C27972T,G28048T,A28111G,G28280C,A28281T,T28282A,G28881A,G28882A,G28883C,C	21	N:D3L,N:R203K,N:G204R,N:S235F,ORF1a:T1001I,ORF1a:A1708D,ORF1a:I2230T,ORF1a:S2625F,ORF1b:P314L,ORF8:Q27*,ORF8:R52I,ORF8:Y73C,S:N501Y,S:A570D,S:D614G,S:P681H,S:T716I,S:K854R,S:L938F,S:S982A,S:D1118H	6	0
hCoV-19/Mexico/CHH-InDRE_F12168_S1193/2021	EPI_ISL_1626800	-	B.1.1.7	GRY	34	C241T,C913T,C3037T,C3267T,C5388A,C5986T,T6954C,C11750T,T13389C,C13665T,A13712G,C14408T,C14676T,C15279T,T16176C,A23063T,C23271A,A23403G,C23604A,C23709T,T24506G,G24914C,C25611G,C26270T,C27972T,G28048T,A28111G,G28280C,A28281T,T28282A,G28881A,G	22	E:T9I,N:D3L,N:R203K,N:G204R,N:S235F,ORF1a:T1001I,ORF1a:A1708D,ORF1a:I2230T,ORF1a:L3829F,ORF1a:M4375T,ORF1b:K82R,ORF1b:P314L,ORF8:Q27*,ORF8:R52I,ORF8:Y73C,S:N501Y,S:A570D,S:D614G,S:P681H,S:T716I,S:S982A,S:	6	7
hCoV-19/Mexico/CHH-InDRE_F12169_S1194/2021	EPI_ISL_1626801	-	B.1.1.7	GRY	34	C241T,C913T,C3037T,C3267T,C5388A,C5986T,T6954C,C11750T,C13665T,C14408T,C14676T,C15279T,T16176C,T20478C,A23063T,C23271A,A23403G,C23604A,C23709T,T24506G,G24914C,C25611G,C26110T,C26270T,C27972T,G28048T,A28111G,G28280C,A28281T,T28282A,G28881A,G28882A,G28883C,C28977T	21	E:T9I,N:D3L,N:R203K,N:G204R,N:S235F,ORF1a:T1001I,ORF1a:A1708D,ORF1a:I2230T,ORF1a:L3829F,ORF1b:P314L,ORF3a:P240S,ORF8:Q27*,ORF8:R52I,ORF8:Y73C,S:N501Y,S:A570D,S:D614G,S:P681H,S:T716I,S:S982A,S:D1118H	6	86
hCoV-19/Mexico/CHH-InDRE_F12356_S1196/2021	EPI_ISL_1626803	-	B.1.1.7	GRY	34	C241T,C913T,C3037T,C3267T,C3486T,T4057C,C5388A,C5986T,T6954C,C11750T,C14408T,C14676T,C15279T,T16176C,A23063T,C23271A,A23403G,C23604A,C23709T,T24506G,G24914C,A25336C,G25337C,C26270T,C27972T,G28048T,A28111G,G28280C,A28281T,T28282A,G28881A,G28882A,G28883C,C28977T	23	E:T9I,N:D3L,N:R203K,N:G204R,N:S235F,ORF1a:T1001I,ORF1a:A1074V,ORF1a:A1708D,ORF1a:I2230T,ORF1a:L3829F,ORF1b:P314L,ORF8:Q27*,ORF8:R52I,ORF8:Y73C,S:N501Y,S:A570D,S:D614G,S:P681H,S:T716I,S:S982A,S:D1118H,S:E1258D,S:D1259H	6	0
hCoV-19/Mexico/CHH-InDRE_F12359_S1197/2021	EPI_ISL_1626804	-	B.1.1.7	GRY	35	C241T,C913T,C3037T,C3267T,C5388A,C5986T,T6954C,C9298T,C14408T,C14676T,C15279T,T16176C,G18803T,A23063T,C23271A,A23403G,C23604A,C23709T,C24374T,T24506G,G24914C,G25334C,A25336C,G25337C,C27972T,G28048T,A28111G,G28280C,A28281T,T28282A,G28774T,G28881A,G28882A,G28883C,C28977T	23	N:D3L,N:L167F,N:R203K,N:G204R,N:S235F,ORF1a:T1001I,ORF1a:A1708D,ORF1a:I2230T,ORF1b:P314L,ORF1b:S1779I,ORF8:Q27*,ORF8:R52I,ORF8:Y73C,S:N501Y,S:A570D,S:D614G,S:P681H,S:T716I,S:L938F,S:S982A,S:D1118H,S:E1258H,S:D1259H	6	0

hCoV-19/Mexico/CHH-InDRE_F12366_S1198/2021	EPI_ISL_1626805	-	B.1.1.7	GRY	37	C241T,C913T,C3037T,C3267T,C3486T,T4057C,C5388A,C5986T,T6954C,C11750T,C12970T,C14408T,C14676T,C15279T,T16176C,T20007C,A23063T,C23271A,A23403G,C23604A,C23709T,T24506G,G24914C,A25336C,G25337C,C26270T,C27972T,G28048T,A28111G,G28280C,A28281T,T28282A,C28290T,G28881A,G28882A,G28883C,C28977T	25	E:T9I,N:D3L,N:P6L,N:R203K,N:G204R,N:S235F,ORF1a:T1001I,ORF1a:A1074V,ORF1a:A1708D,ORF1a:I2230T,ORF1a:L3829F,ORF1b:P314L,ORF8:Q27*,ORF8:R52I,ORF8:Y73C,ORF9b:P3S,S:N501Y,S:A570D,S:D614G,S:P681H,S:T716I,S:S982A,S:D1118H,S:E1258D,S:D1259H	6	0
hCoV-19/Mexico/CHH-InDRE_FB12868_S1488/2021	EPI_ISL_1805466	-	B.1.1.7	GRY	32	C241T,C913T,C3037T,C3267T,C5388A,C5986T,T6954C,C9298T,C14120T,C14408T,C14676T,C15279T,T16176C,A23063T,C23271A,A23403G,C23604A,C23709T,C24374T,T24506G,G24914C,C27972T,G28048T,G28079T,A28111G,G28280C,A28281T,T28282A,G28881A,G28882A,G28883C,	20	N:D3L,N:R203K,N:G204R,N:S235F,ORF1a:T1001I,ORF1a:A1708D,ORF1a:I2230T,ORF1b:P218L,ORF1b:P314L,ORF8:Q27*,ORF8:R52I,ORF8:Y73C,S:N501Y,S:A570D,S:D614G,S:P681H,S:T716I,S:L938F,S:S982A,S:D1118H	6	0
hCoV-19/Mexico/CHH-InDRE_FB12871_S1489/2021	EPI_ISL_1805467	-	B.1.1.7	GRY	33	C241T,G789A,C913T,C3037T,C3267T,C5388A,C5986T,T6954C,C11750T,G13201T,C14408T,C14676T,C15279T,T16176C,A23063T,C23271A,A23403G,C23604A,C23709T,T24506G,G24914C,C26270T,C27972T,G28048T,A28111G,A28210T,G28280C,A28281T,T28282A,G28881A,G28882A,G2	22	E:T9I,N:D3L,N:R203K,N:G204R,N:S235F,ORF1a:R175H,ORF1a:T1001I,ORF1a:A1708D,ORF1a:I2230T,ORF1a:L3829F,ORF1b:P314L,ORF8:Q27*,ORF8:R52I,ORF8:Y73C,ORF8:E106V,S:N501Y,S:A570D,S:D614G,S:P681H,S:T716I,S:S982A,S:D	6	0
hCoV-19/Mexico/CHH-InDRE_FB12877_S1490/2021	EPI_ISL_1805468	-	B.1.1.7	GRY	32	C241T,C913T,C2462T,C3037T,C3267T,C5388A,C5986T,T6954C,C9298T,C14408T,C14676T,C15279T,T16176C,A23063T,C23271A,A23403G,C23604A,C23709T,C24374T,T24506G,G24914C,C27972T,G28048T,T28094C,A28111G,G28280C,A28281T,T28282A,G28881A,G28882A,G28883C,C2	19	N:D3L,N:R203K,N:G204R,N:S235F,ORF1a:T1001I,ORF1a:A1708D,ORF1a:I2230T,ORF1b:P314L,ORF8:Q27*,ORF8:R52I,ORF8:Y73C,S:N501Y,S:A570D,S:D614G,S:P681H,S:T716I,S:L938F,S:S982A,S:D1118H	6	0
hCoV-19/Mexico/CHH-InDRE_FB12878_S1491/2021	EPI_ISL_1805469	-	B.1.1.7	GRY	29	C241T,C913T,C3037T,C3267T,C5388A,C5986T,C9298T,C14408T,C14676T,C15279T,T16176C,A23063T,C23271A,A23403G,C23604A,C23709T,C24374T,T24506G,G24914C,C27972T,G28048T,A28111G,G28280C,A28281T,T28282A,G28881A,G28882A,G28883C,C28977T	18	N:D3L,N:R203K,N:G204R,N:S235F,ORF1a:T1001I,ORF1a:A1708D,ORF1b:P314L,ORF8:Q27*,ORF8:R52I,ORF8:Y73C,S:N501Y,S:A570D,S:D614G,S:P681H,S:T716I,S:L938F,S:S982A,S:D1118H	6	228
hCoV-19/Mexico/CHH-InDRE_FB13242_S1496/2021	EPI_ISL_1805474	-	B.1.1.7	GRY	30	C241T,C913T,C3037T,C3267T,C5388A,C5986T,T6954C,C14408T,C14676T,C15279T,T16176C,A17153G,A23063T,C23271A,A23403G,C23604A,C23709T,C24374T,T24506G,G24914C,C27972T,G28048T,A28111G,G28280C,A28281T,T28282A,G28881A,G28882A,G28883C,C28977T	20	N:D3L,N:R203K,N:G204R,N:S235F,ORF1a:T1001I,ORF1a:A1708D,ORF1a:I2230T,ORF1b:P314L,ORF1b:Y1229C,ORF8:Q27*,ORF8:R52I,ORF8:Y73C,S:N501Y,S:A570D,S:D614G,S:P681H,S:T716I,S:L938F,S:S982A,S:D1118H	6	1647
hCoV-19/Mexico/CHH-InDRE_FB13243_S1497/2021	EPI_ISL_1805475	-	B.1.1.7	GRY	32	T78G,C241T,C913T,C3037T,C3267T,C5388A,C5986T,T6954C,C14408T,C14649T,C14676T,C15279T,T16176C,C18348T,A23063T,C23271A,A23403G,C23604A,C23709T,T24506G,G24914C,A25336C,C27972T,G28048T,A28111G,G28280C,A28281T,T28282A,G28881A,G28882A,G28883C,C2	19	N:D3L,N:R203K,N:G204R,N:S235F,ORF1a:T1001I,ORF1a:A1708D,ORF1a:I2230T,ORF1b:P314L,ORF8:Q27*,ORF8:R52I,ORF8:Y73C,S:N501Y,S:A570D,S:D614G,S:P681H,S:T716I,S:S982A,S:D1118H,S:E1258D	6	749
hCoV-19/Mexico/CHH-InDRE_FB13246_S1498/2021	EPI_ISL_1805476	-	B.1.1.7	GRY	33	C241T,C913T,C3037T,C3267T,G5164T,C5388A,C5986T,T6954C,C8626T,C14408T,C14676T,C15279T,T16176C,A23063T,C23271A,A23403G,C23604A,C23709T,T24506G,G24914C,A25336C,G25337C,C25714T,C27972T,G28048T,A28111G,G28280C,A28281T,T28282A,G28881A,G28882A,G2	22	N:D3L,N:R203K,N:G204R,N:S235F,ORF1a:T1001I,ORF1a:E1633D,ORF1a:A1708D,ORF1a:I2230T,ORF1b:P314L,ORF3a:L108F,ORF8:Q27*,ORF8:R52I,ORF8:Y73C,S:N501Y,S:A570D,S:D614G,S:P681H,S:T716I,S:S982A,S:D1118H,S:E1258D,	6	0
hCoV-19/Mexico/CHH-InDRE_FB13247_S1499/2021	EPI_ISL_1805477	-	B.1.1.7	GRY	33	C203T,C241T,C913T,C3037T,C3267T,C5388A,C5986T,T6954C,T9699C,C14408T,C14676T,C15279T,T16176C,C19029T,A23063T,C23271A,A23403G,C23604A,C23709T,T24506G,G24914C,A25336C,G25337C,C27972T,G28048T,A28111G,G28280C,A28281T,T28282A,G28881A,G28882A,G2	21	N:D3L,N:R203K,N:G204R,N:S235F,ORF1a:T1001I,ORF1a:A1708D,ORF1a:I2230T,ORF1a:I3145T,ORF1b:P314L,ORF8:Q27*,ORF8:R52I,ORF8:Y73C,S:N501Y,S:A570D,S:D614G,S:P681H,S:T716I,S:S982A,S:D1118H,S:E1258D,S:D1259H	6	0
hCoV-19/Mexico/CHH-InDRE_FB13252_S1500/2021	EPI_ISL_1805478	-	B.1.1.7	GR	28	C913T,C3037T,C3267T,C5388A,C5986T,T6954C,C14408T,C14676T,C15279T,T16176C,C23271A,A23403G,C23604A,C23709T,C24374T,T24506G,G24914C,A25336C,C27972T,G28048T,A28111G,G28280C,A28281T,T28282A,G28881A,G28882A,G28883C,C28977T	19	N:D3L,N:R203K,N:G204R,N:S235F,ORF1a:T1001I,ORF1a:A1708D,ORF1a:I2230T,ORF1b:P314L,ORF8:Q27*,ORF8:R52I,ORF8:Y73C,S:A570D,S:D614G,S:P681H,S:T716I,S:L938F,S:S982A,S:D1118H,S:E1258D	6	3116
hCoV-19/Mexico/CHH-InDRE_FB13253_S1501/2021	EPI_ISL_1805479	-	B.1.1.7	GRY	35	T78G,C241T,C913T,G2819A,C3037T,A3084G,C3267T,C5388A,C5986T,T6954C,C9298T,C14408T,C14676T,C15279T,T16176C,A23063T,C23271A,A23403G,C23604A,C23709T,C24374T,T24506G,G24914C,A25336C,G25337C,C27972T,G28048T,A28111G,G28280C,A28281T,T28282A,G28881A,G28882A,G28883C,C28977T	23	N:D3L,N:R203K,N:G204R,N:S235F,ORF1a:V852I,ORF1a:E940G,ORF1a:T1001I,ORF1a:A1708D,ORF1a:I2230T,ORF1b:P314L,ORF8:Q27*,ORF8:R52I,ORF8:Y73C,S:N501Y,S:A570D,S:D614G,S:P681H,S:T716I,S:L938F,S:S982A,S:D1118H,S:E1258D,S:D1259H	6	0
hCoV-19/Mexico/CHH-InDRE_FB13662_S1732/2021	EPI_ISL_2101871	-	B.1.1.7	GRY	35	C241T,C913T,C2453T,C3037T,C3267T,C3695T,C5388A,C5986T,T6954C,C7720T,C9611T,C14408T,C14676T,C15279T,T16176C,C21855T,A23063T,C23271A,A23403G,C23604A,C23709T,T24506G,G24914C,A25336C,C27972T,G28048T,A28111G,G28280C,A28281T,T28282A,A28295G,G28881A,G28882A,G28883C,C28977T	23	N:D3L,N:N8D,N:R203K,N:G204R,N:S235F,ORF1a:L730F,ORF1a:T1001I,ORF1a:A1708D,ORF1a:I2230T,ORF1a:L3116F,ORF1b:P314L,ORF8:Q27*,ORF8:R52I,ORF8:Y73C,S:S98F,S:N501Y,S:A570D,S:D614G,S:P681H,S:T716I,S:S982A,S:D1118H,S:E1258D	6	0
hCoV-19/Mexico/CHH-InDRE_FB13663_S1733/2021	EPI_ISL_2101872	-	B.1.1.7	GRY	32	C241T,C913T,C3037T,C3267T,C5388A,C5986T,T6954C,C9298T,C14408T,C14676T,C15279T,T16176C,A23063T,C23271A,A23403G,C23604A,C23709T,C24374T,T24506G,G24914C,A25336C,C27972T,G28048T,A28111G,G28280C,A28281T,T28282A,G28314A,G28881A,G28882A,G28883C,C	22	N:D3L,N:R14H,N:R203K,N:G204R,N:S235F,ORF1a:T1001I,ORF1a:A1708D,ORF1a:I2230T,ORF1b:P314L,ORF8:Q27*,ORF8:R52I,ORF8:Y73C,ORF9b:A11T,S:N501Y,S:A570D,S:D614G,S:P681H,S:T716I,S:L938F,S:S982A,S:D1118H,S:E1258D	6	0
hCoV-19/Mexico/CHH-InDRE_FB13668_S1734/2021	EPI_ISL_2101873	-	B.1.1.7	GRY	30	C241T,C913T,C3037T,C3267T,C5388A,C5986T,T6954C,C14408T,C14676T,C15279T,T16176C,A23063T,C23271A,A23403G,C23604A,C23709T,T24506G,G24914C,A25336C,C26833T,C27972T,G28048T,A28111G,G28280C,A28281T,T28282A,G28881A,G28882A,G28883C,C28977T	20	M:A104V,N:D3L,N:R203K,N:G204R,N:S235F,ORF1a:T1001I,ORF1a:A1708D,ORF1a:I2230T,ORF1b:P314L,ORF8:Q27*,ORF8:R52I,ORF8:Y73C,S:N501Y,S:A570D,S:D614G,S:P681H,S:T716I,S:S982A,S:D1118H,S:E1258D	6	0
hCoV-19/Mexico/CHH-InDRE_FB13671_S1735/2021	EPI_ISL_2101874	-	B.1.1.7	GRY	31	C241T,C913T,C3037T,C3267T,C5388A,C5986T,T6954C,C9298T,C14408T,C14676T,C15279T,T16176C,A23063T,C23271A,A23403G,C23604A,C23709T,C24374T,T24506G,G24914C,A25336C,C27972T,G28048T,A28111G,G28280C,A28281T,T28282A,G28881A,G28882A,G28883C,C28977T	20	N:D3L,N:R203K,N:G204R,N:S235F,ORF1a:T1001I,ORF1a:A1708D,ORF1a:I2230T,ORF1b:P314L,ORF8:Q27*,ORF8:R52I,ORF8:Y73C,S:N501Y,S:A570D,S:D614G,S:P681H,S:T716I,S:L938F,S:S982A,S:D1118H,S:E1258D	6	0
hCoV-19/Mexico/CHH-InDRE_FB13672_S1736/2021	EPI_ISL_2101875	-	B.1.1.7	GRY	32	C241T,C913T,C3037T,C3267T,C5388A,C5986T,T6954C,C9298T,C14408T,C14676T,C15279T,T16176C,G17523T,A23063T,C23271A,A23403G,C23604A,C23709T,C24374T,T24506G,G24914C,C27972T,G28048T,A28111G,G28280C,A28281T,T28282A,G28881A,G28882A,G28883C,C28977T,G	21	N:D3L,N:R203K,N:G204R,N:S235F,N:E253D,ORF1a:T1001I,ORF1a:A1708D,ORF1a:I2230T,ORF1b:P314L,ORF1b:M1352I,ORF8:Q27*,ORF8:R52I,ORF8:Y73C,S:N501Y,S:A570D,S:D614G,S:P681H,S:T716I,S:L938F,S:S982A,S:D1118H	6	0
hCoV-19/Mexico/CHH-InDRE_FB13678_S1751/2021	EPI_ISL_2101890	-	B.1.1.7	GRY	34	C241T,C913T,C3037T,C3267T,T3537C,C5388A,C5986T,T6954C,C9298T,C14408T,C14676T,C15279T,T16176C,G20683T,A23063T,C23271A,A23403G,C23604A,C23709T,C24374T,T24506G,G24914C,A25336C,C27972T,G28048T,A28111G,G28280C,A28281T,T28282A,G28881A,G28882A,G28883C,C28977T,C29367T	23	N:D3L,N:R203K,N:G204R,N:S235F,N:P365L,ORF1a:T1001I,ORF1a:I1091T,ORF1a:A1708D,ORF1a:I2230T,ORF1b:P314L,ORF1b:V2406F,ORF8:Q27*,ORF8:R52I,ORF8:Y73C,S:N501Y,S:A570D,S:D614G,S:P681H,S:T716I,S:L938F,S:S982A,S:D1118H,S:E1258D	6	0
hCoV-19/Mexico/CHH-InDRE_FB13679_S1737/2021	EPI_ISL_2101876	-	B.1.1.7	GRY	34	C241T,C913T,C3037T,C3267T,C5388A,C5986T,T6954C,T7202C,C11750T,C14408T,C14676T,C15279T,T16176C,A23063T,C23271A,A23403G,C23604A,C23709T,T24506G,G24914C,A25336C,C26270T,C27972T,G28048T,A28111G,G28280C,A28281T,T28282A,G28857C,G28881A,G28882A,G28883C,C28977T,G29260T	23	E:T9I,N:D3L,N:R195T,N:R203K,N:G204R,N:S235F,ORF1a:T1001I,ORF1a:A1708D,ORF1a:I2230T,ORF1a:S2313P,ORF1a:L3829F,ORF1b:P314L,ORF8:Q27*,ORF8:R52I,ORF8:Y73C,S:N501Y,S:A570D,S:D614G,S:P681H,S:T716I,S:S982A,S:D1118H,S:E1258D	6	165
hCoV-19/Mexico/CHH-InDRE_FB13688_S1738/2021	EPI_ISL_2101877	-	B.1.1.7	GRY	31	C241T,C913T,C3037T,C3267T,C5388A,C5986T,T6954C,C14408T,C14676T,C15279T,T16176C,G18677T,A23063T,C23271A,A23403G,C23604A,C23709T,T24506G,G24914C,A25336C,C27002T,C27972T,G28048T,A28111G,G28280C,A28281T,T28282A,G28881A,G28882A,G28883C,C28977T	20	N:D3L,N:R203K,N:G204R,N:S235F,ORF1a:T1001I,ORF1a:A1708D,ORF1a:I2230T,ORF1b:P314L,ORF1b:R1737L,ORF8:Q27*,ORF8:R52I,ORF8:Y73C,S:N501Y,S:A570D,S:D614G,S:P681H,S:T716I,S:S982A,S:D1118H,S:E1258D	6	253
hCoV-19/Mexico/CHH-InDRE_FB13702_S1739/2021	EPI_ISL_2101878	-	B.1.1.7	GRY	34	C98T,C241T,C913T,C3037T,C3267T,C5388A,C5986T,C6525T,T6954C,C9298T,C14408T,C14676T,C15279T,T16176C,A23063T,C23271A,A23403G,C23604A,C23709T,C24374T,T24506G,G24914C,A25336C,G25395T,C27972T,G28048T,A28111G,G28280C,A28281T,T28282A,G28881A,G28882	22	N:D3L,N:R203K,N:G204R,N:S235F,ORF1a:T1001I,ORF1a:A1708D,ORF1a:T2087I,ORF1a:I2230T,ORF1b:P314L,ORF3a:M1I,ORF8:Q27*,ORF8:R52I,ORF8:Y73C,S:N501Y,S:A570D,S:D614G,S:P681H,S:T716I,S:L938F,S:S982A,S:D1118H,S:E12	6	0
hCoV-19/Mexico/CHH-InDRE_FB13814_S1742/2021	EPI_ISL_2101881	-	B.1.1.7	GRY	33	C241T,C398T,C913T,C3037T,C3267T,C5388A,C5986T,T6954C,G8470A,C14408T,C14676T,C15279T,T16176C,C21622T,A23063T,C23271A,A23403G,C23604A,C23709T,C24381T,T24506G,G24914C,A25336C,C27972T,G28048T,A28111G,G28280C,A28281T,T28282A,G28881A,G28882A,G28	21	N:D3L,N:R203K,N:G204R,N:S235F,ORF1a:H45Y,ORF1a:T1001I,ORF1a:A1708D,ORF1a:I2230T,ORF1b:P314L,ORF8:Q27*,ORF8:R52I,ORF8:Y73C,S:N501Y,S:A570D,S:D614G,S:P681H,S:T716I,S:S940F,S:S982A,S:D1118H,S:E1258D	6	0
hCoV-19/Mexico/CHH-InDRE_FB13819_S1743/2021	EPI_ISL_2101882	-	B.1.1.7	GRY	38	C241T,C913T,C1457T,C2453T,C3037T,C3267T,C5388A,C5986T,T6954C,C9611T,A12159C,C14408T,C14676T,C15279T,T16176C,G20580A,C21855T,A23063T,C23271A,A23403G,C23604A,C23709T,T24506G,G24914C,A25336C,C27972T,G28048T,A28111G,G28280C,A28281T,T28282A,A28295G,C28606T,C28687T,G28881A,G28882A,G28883C,C28977T	25	N:D3L,N:N8D,N:R203K,N:G204R,N:S235F,ORF1a:R398C,ORF1a:L730F,ORF1a:T1001I,ORF1a:A1708D,ORF1a:I2230T,ORF1a:L3116F,ORF1a:E3965A,ORF1b:P314L,ORF8:Q27*,ORF8:R52I,ORF8:Y73C,S:S98F,S:N501Y,S:A570D,S:D614G,S:P681H,S:T716I,S:S982A,S:D1118H,S:E1258D	6	0
hCoV-19/Mexico/CHH-InDRE_FB13820_S1744/2021	EPI_ISL_2101883	-	B.1.1.7	GRY	33	C241T,C913T,C3037T,C3267T,C5388A,C5986T,T6954C,C14408T,C14676T,C15279T,C15656T,T16176C,A23063T,C23271A,A23403G,C23604A,C23709T,T24506G,G24914C,A25336C,C26833T,C27732T,C27972T,G28048T,A28111G,G28280C,A28281T,T28282A,G28881A,G28882A,G28883C,C	22	M:A104V,N:D3L,N:R203K,N:G204R,N:A211V,N:S235F,ORF1a:T1001I,ORF1a:A1708D,ORF1a:I2230T,ORF1b:P314L,ORF1b:T730I,ORF8:Q27*,ORF8:R52I,ORF8:Y73C,S:N501Y,S:A570D,S:D614G,S:P681H,S:T716I,S:S982A,S:D1118H,S:E1258	6	7

hCoV-19/Mexico/CHH-InDRE_FB13822_S1745/2021	EPI_ISL_2101884	-	B.1.1.7	GRY	36	C96T,C241T,C913T,C3037T,C3267T,C5388A,C5986T,T6954C,C9298T,C14408T,C14676T,C15279T,T16176C,G17338A,G18803T,C22450T,A23063T,C23271A,A23403G,C23604A,C23709T,C24374T,T24506G,G24914C,A25336C,C27972T,G28048T,A28111G,G28280C,A28281T,T28282A,G28774T,G28881A,G28882A,G28883C,C28977T	23	N:D3L,N:L167F,N:R203K,N:G204R,N:S235F,ORF1a:T1001I,ORF1a:A1708D,ORF1a:I2230T,ORF1b:P314L,ORF1b:A1291T,ORF1b:S1779I,ORF8:Q27*,ORF8:R52I,ORF8:Y73C,S:N501Y,S:A570D,S:D614G,S:P681H,S:T716I,S:L938F,S:S982A,S:D1118H,S:E1258D	6	0
hCoV-19/Mexico/CHH-InDRE_FB13825_S1746/2021	EPI_ISL_2101885	-	B.1.1.7	GRY	31	C241T,C913T,C3037T,C5388A,C5986T,T6954C,C9298T,C14408T,C14676T,C15279T,T15504C,T16176C,A23063T,C23271A,A23403G,C23604A,C23635T,C23709T,C24374T,T24506G,G24914C,C27972T,G28048T,A28111G,G28280C,A28281T,T28282A,G28881A,G28882A,G28883C,C28977T	18	N:D3L,N:R203K,N:G204R,N:S235F,ORF1a:A1708D,ORF1a:I2230T,ORF1b:P314L,ORF8:Q27*,ORF8:R52I,ORF8:Y73C,S:N501Y,S:A570D,S:D614G,S:P681H,S:T716I,S:L938F,S:S982A,S:D1118H	6	1374
hCoV-19/Mexico/CHH-InDRE_FB13827_S1747/2021	EPI_ISL_2101886	-	B.1.1.7	GRY	31	C241T,C913T,C3037T,C3267T,C5388A,C5986T,T6954C,C14408T,C14649T,C14676T,C15279T,T16176C,C18348T,G21600T,A23063T,C23271A,A23403G,C23604A,C23709T,T24506G,G24914C,C27972T,G28048T,A28111G,G28280C,A28281T,T28282A,G28881A,G28882A,G28883C,C28977T	19	N:D3L,N:R203K,N:G204R,N:S235F,ORF1a:T1001I,ORF1a:A1708D,ORF1a:I2230T,ORF1b:P314L,ORF8:Q27*,ORF8:R52I,ORF8:Y73C,S:S13I,S:N501Y,S:A570D,S:D614G,S:P681H,S:T716I,S:S982A,S:D1118H	6	303
hCoV-19/Mexico/CHH-InDRE_FB13832_S1748/2021	EPI_ISL_2101887	-	B.1.1.7	GRY	34	C241T,C913T,C2143T,C3037T,C3267T,C5388A,C5986T,T6954C,C14408T,C14676T,T15096C,C15279T,T16176C,A23063T,C23271A,A23403G,C23604A,C23709T,G24089A,T24506G,G24914C,A25336C,G25785T,C27972T,G28048T,A28111G,G28280C,A28281T,T28282A,G28881A,G28882A,	21	N:D3L,N:R203K,N:G204R,N:S235F,ORF1a:T1001I,ORF1a:A1708D,ORF1a:I2230T,ORF1b:P314L,ORF3a:W131C,ORF8:Q27*,ORF8:R52I,ORF8:Y73C,S:N501Y,S:A570D,S:D614G,S:P681H,S:T716I,S:D843N,S:S982A,S:D1118H,S:E1258D	6	0
hCoV-19/Mexico/CHH-InDRE_FB13835_S1749/2021	EPI_ISL_2101888	-	B.1.1.7	GRY	31	C241T,C913T,C3037T,C3267T,C5388A,C5986T,T6954C,C14408T,C14649T,C14676T,C15279T,T16176C,C18348T,G21600T,A23063T,C23271A,A23403G,C23604A,C23709T,T24506G,G24914C,C27972T,G28048T,A28111G,G28280C,A28281T,T28282A,G28881A,G28882A,G28883C,C28977T	19	N:D3L,N:R203K,N:G204R,N:S235F,ORF1a:T1001I,ORF1a:A1708D,ORF1a:I2230T,ORF1b:P314L,ORF8:Q27*,ORF8:R52I,ORF8:Y73C,S:S13I,S:N501Y,S:A570D,S:D614G,S:P681H,S:T716I,S:S982A,S:D1118H	6	0
hCoV-19/Mexico/CHH-InDRE_FB13840_S1750/2021	EPI_ISL_2101889	-	B.1.1.7	GRY	34	C241T,C913T,C2143T,C3037T,C3267T,C5388A,C5986T,T6954C,C14408T,C14676T,T15096C,C15279T,T16176C,A23063T,C23271A,A23403G,C23604A,C23709T,G24089A,T24506G,G24914C,A25336C,G25785T,C27972T,G28048T,A28111G,G28280C,A28281T,T28282A,G28881A,G28882A,	21	N:D3L,N:R203K,N:G204R,N:S235F,ORF1a:T1001I,ORF1a:A1708D,ORF1a:I2230T,ORF1b:P314L,ORF3a:W131C,ORF8:Q27*,ORF8:R52I,ORF8:Y73C,S:N501Y,S:A570D,S:D614G,S:P681H,S:T716I,S:D843N,S:S982A,S:D1118H,S:E1258D	6	334
hCoV-19/Mexico/CHH-InDRE_FB14222_S1764/2021	EPI_ISL_2101903	-	B.1.1.7	GRY	33	C241T,C913T,C3037T,C3267T,G5164T,C5388A,C5986T,T6954C,C12885T,C14408T,C14676T,C15279T,T16176C,A23063T,C23191T,C23271A,A23403G,C23604A,C23709T,T24506G,G24914C,A25336C,C25710T,C27972T,G28048T,A28111G,G28280C,A28281T,T28282A,G28881A,G28882A,G	21	N:D3L,N:R203K,N:G204R,N:S235F,ORF1a:T1001I,ORF1a:E1633D,ORF1a:A1708D,ORF1a:I2230T,ORF1a:T4207I,ORF1b:P314L,ORF8:Q27*,ORF8:R52I,ORF8:Y73C,S:N501Y,S:A570D,S:D614G,S:P681H,S:T716I,S:S982A,S:D1118H,S:E1258D	6	75
hCoV-19/Mexico/CHH-InDRE_FB14223_S1765/2021	EPI_ISL_2101904	-	B.1.1.7	GRY	34	C241T,C913T,C1190T,C3037T,C3267T,C5388A,C5986T,T6954C,A8884G,C9298T,C14408T,C14676T,C15279T,T16176C,A23063T,C23271A,A23403G,C23604A,C23643T,C23709T,C24374T,T24506G,G24914C,A25336C,C27972T,G28048T,A28111G,G28280C,A28281T,T28282A,G28881A,G28882A,G28883C,C28977T	23	N:D3L,N:R203K,N:G204R,N:S235F,ORF1a:P309S,ORF1a:T1001I,ORF1a:A1708D,ORF1a:I2230T,ORF1a:I2873M,ORF1b:P314L,ORF8:Q27*,ORF8:R52I,ORF8:Y73C,S:N501Y,S:A570D,S:D614G,S:P681H,S:A694V,S:T716I,S:L938F,S:S982A,S:D1118H,S:E1258D	6	0
hCoV-19/Mexico/CHH-InDRE_FB14877_S2086/2021	EPI_ISL_2283725	-	B.1.1.7	GRY	36	C241T,C913T,C2094T,C3037T,C3267T,T4057C,G4300T,C5388A,C5986T,T6954C,C11750T,A13416G,C14408T,C14676T,C15141T,C15279T,T16176C,A23063T,C23271A,A23403G,C23604A,C23709T,T24506G,G24914C,A25336C,C26270T,C27972T,G28048T,A28111G,G28280C,A28281T,T28282A,G28881A,G28882A,G28883C,C28977T	23	E:T9I,N:D3L,N:R203K,N:G204R,N:S235F,ORF1a:S610L,ORF1a:T1001I,ORF1a:A1708D,ORF1a:I2230T,ORF1a:L3829F,ORF1a:D4384G,ORF1b:P314L,ORF8:Q27*,ORF8:R52I,ORF8:Y73C,S:N501Y,S:A570D,S:D614G,S:P681H,S:T716I,S:S982A,S:D1118H,S:E1258D	6	0
hCoV-19/Mexico/CHH-InDRE_FB14878_S2389/2021	EPI_ISL_2455914	-	B.1.1.7	GRY	35	C241T,C913T,C3037T,C3267T,C5388A,C5986T,T6954C,C12747T,C14408T,C14676T,C15279T,T16176C,A19302T,A23063T,C23086T,C23271A,A23403G,C23604A,C23709T,T24506G,G24914C,A25336C,C26833T,C26873T,C27972T,G28048T,A28111G,G28280C,A28281T,T28282A,G28881A,G28882A,G28883C,C28977T,C29614T	21	M:A104V,N:D3L,N:R203K,N:G204R,N:S235F,ORF1a:T1001I,ORF1a:A1708D,ORF1a:I2230T,ORF1a:T4161I,ORF1b:P314L,ORF8:Q27*,ORF8:R52I,ORF8:Y73C,S:N501Y,S:A570D,S:D614G,S:P681H,S:T716I,S:S982A,S:D1118H,S:E1258D	6	0
hCoV-19/Mexico/CHH-InDRE_FB14879_S2392/2021	EPI_ISL_2455917	-	B.1.1.7	GRY	34	C241T,G271T,C913T,C3037T,C3267T,C5388A,C5986T,T6954C,C12854T,C14408T,C14676T,C15279T,T16176C,C21639A,A23063T,C23271A,A23403G,C23604A,C23709T,C24374T,T24506G,G24914C,C25275T,A25336C,C27972T,G28048T,A28111G,G28280C,A28281T,T28282A,G28881A,G28882A,G28883C,C28977T	24	N:D3L,N:R203K,N:G204R,N:S235F,ORF1a:E2D,ORF1a:T1001I,ORF1a:A1708D,ORF1a:I2230T,ORF1a:P4197S,ORF1b:P314L,ORF8:Q27*,ORF8:R52I,ORF8:Y73C,S:P26H,S:N501Y,S:A570D,S:D614G,S:P681H,S:T716I,S:L938F,S:S982A,S:D1118H,S:T1238I,S:E1258D	6	84
hCoV-19/Mexico/CHH-InDRE_FB14880_S2401/2021	EPI_ISL_2455926	-	B.1.1.7	GRY	32	C241T,C913T,C3037T,C3267T,C5388A,C5986T,T6954C,C14408T,C14649T,C14676T,C15279T,T16176C,C18348T,A23063T,C23271A,A23403G,C23604A,C23709T,T24506G,G24914C,T25228A,A25336C,C27972T,G28048T,A28111G,G28280C,A28281T,T28282A,G28881A,G28882A,G28883C,	19	N:D3L,N:R203K,N:G204R,N:S235F,ORF1a:T1001I,ORF1a:A1708D,ORF1a:I2230T,ORF1b:P314L,ORF8:Q27*,ORF8:R52I,ORF8:Y73C,S:N501Y,S:A570D,S:D614G,S:P681H,S:T716I,S:S982A,S:D1118H,S:E1258D	6	0
hCoV-19/Mexico/CHH-InDRE_FB14901_S2402/2021	EPI_ISL_2455927	-	B.1.1.7	GRY	33	C241T,C913T,C3037T,C3267T,A4420G,C5388A,C5986T,T6954C,C9298T,C14408T,C14676T,C15279T,T16176C,A23063T,C23271A,A23403G,C23604A,C23635T,C23709T,C24374T,T24506G,G24914C,A25336C,C27972T,G28048T,A28111G,G28280C,A28281T,T28282A,G28881A,G28882A,G2	20	N:D3L,N:R203K,N:G204R,N:S235F,ORF1a:T1001I,ORF1a:A1708D,ORF1a:I2230T,ORF1b:P314L,ORF8:Q27*,ORF8:R52I,ORF8:Y73C,S:N501Y,S:A570D,S:D614G,S:P681H,S:T716I,S:L938F,S:S982A,S:D1118H,S:E1258D	6	255
hCoV-19/Mexico/CHH-InDRE_FB14906_S2403/2021	EPI_ISL_2455928	-	B.1.1.7	GRY	33	C241T,C913T,C3037T,C3267T,C5388A,C5986T,A6459G,T6954C,C14408T,C14649T,C14676T,C15279T,T16176C,C18348T,A23063T,C23271A,A23403G,C23604A,C23709T,T24506G,G24914C,A25336C,C27972T,G28048T,A28111G,G28280C,A28281T,T28282A,G28881A,G28882A,G28883C,	20	N:D3L,N:R203K,N:G204R,N:S235F,ORF1a:T1001I,ORF1a:A1708D,ORF1a:N2065S,ORF1a:I2230T,ORF1b:P314L,ORF8:Q27*,ORF8:R52I,ORF8:Y73C,S:N501Y,S:A570D,S:D614G,S:P681H,S:T716I,S:S982A,S:D1118H,S:E1258D	6	0
hCoV-19/Mexico/CHH-InDRE_FB14908_S2404/2021	EPI_ISL_2484164	-	B.1.1.7	GRY	39	C241T,G529T,C913T,C2110T,C3037T,C3267T,C3811T,C5388A,C5986T,T6954C,C7086T,C12116T,C14120T,C14408T,C14676T,C15279T,T16176C,A17039G,A23063T,C23271A,A23403G,C23557T,C23604A,C23709T,T24506G,G24914C,A25336C,A25946C,C27972T,G28048T,A28111G,G28280C,A28281T,T28282A,G28881A,G28882A,G28883C,C28977T,C29445T	24	N:D3L,N:R203K,N:G204R,N:S235F,N:T391I,ORF1a:T1001I,ORF1a:A1708D,ORF1a:I2230T,ORF1a:T2274I,ORF1a:L3951F,ORF1b:P218L,ORF1b:P314L,ORF1b:N1191S,ORF3a:Q185P,ORF8:Q27*,ORF8:R52I,S:N501Y,S:A570D,S:D614G,S:P681H,S:T716I,S:S982A,S:D1118H,S:E1258D	7	0
hCoV-19/Mexico/CHH-InDRE_FB14909_S2405/2021	EPI_ISL_2455929	-	B.1.1.7	GRY	33	C241T,C913T,C3037T,C3177T,C3267T,C5388A,C5986T,T6954C,C9298T,C14408T,C14676T,C15279T,T16176C,A23063T,C23271A,A23403G,C23604A,C23709T,C24374T,T24506G,G24914C,A25336C,C27389T,C27972T,G28048T,A28111G,G28280C,A28281T,T28282A,G28881A,G28882A,G2	21	N:D3L,N:R203K,N:G204R,N:S235F,ORF1a:P971L,ORF1a:T1001I,ORF1a:A1708D,ORF1a:I2230T,ORF1b:P314L,ORF8:Q27*,ORF8:R52I,ORF8:Y73C,S:N501Y,S:A570D,S:D614G,S:P681H,S:T716I,S:L938F,S:S982A,S:D1118H,S:E1258D	6	47
hCoV-19/Mexico/CHH-InDRE_FB14913_S2390/2021	EPI_ISL_2455915	-	B.1.1.7	GRY	32	C241T,C913T,C3037T,C3267T,C5388A,C5986T,T6954C,C14408T,C14649T,C14676T,C15279T,T16176C,C18348T,A23063T,C23114T,C23271A,A23403G,C23604A,C23709T,T24506G,G24914C,A25336C,C27972T,G28048T,A28111G,G28280C,A28281T,T28282A,G28881A,G28882A,G28883C,	19	N:D3L,N:R203K,N:G204R,N:S235F,ORF1a:T1001I,ORF1a:A1708D,ORF1a:I2230T,ORF1b:P314L,ORF8:Q27*,ORF8:R52I,ORF8:Y73C,S:N501Y,S:A570D,S:D614G,S:P681H,S:T716I,S:S982A,S:D1118H,S:E1258D	6	0
hCoV-19/Mexico/CHH-InDRE_FB14915_S2049/2021	EPI_ISL_2283691	-	B.1.1.7	GRY	31	C241T,C913T,C3037T,C3267T,C5388A,C5986T,T6954C,C9298T,C14408T,C14676T,C15279T,T16176C,G21004T,A23063T,C23271A,A23403G,C23604A,C23709T,C24374T,T24506G,G24914C,C27972T,G28048T,A28111G,G28280C,A28281T,T28282A,G28881A,G28882A,G28883C,C28977T	20	N:D3L,N:R203K,N:G204R,N:S235F,ORF1a:T1001I,ORF1a:A1708D,ORF1a:I2230T,ORF1b:P314L,ORF1b:A2513S,ORF8:Q27*,ORF8:R52I,ORF8:Y73C,S:N501Y,S:A570D,S:D614G,S:P681H,S:T716I,S:L938F,S:S982A,S:D1118H	6	8
hCoV-19/Mexico/CHH-InDRE_FB14918_S2050/2021	EPI_ISL_2283692	-	B.1.1.7	GRY	32	C241T,C913T,C3037T,C3267T,C5388A,C5986T,T6954C,C14408T,C14676T,C15279T,T16176C,C18501T,C21622T,A23063T,C23271A,A23403G,C23533T,C23604A,C23709T,C24381T,T24506G,G24914C,C27972T,G28048T,A28111G,G28280C,A28281T,T28282A,G28881A,G28882A,G28883C,	19	N:D3L,N:R203K,N:G204R,N:S235F,ORF1a:T1001I,ORF1a:A1708D,ORF1a:I2230T,ORF1b:P314L,ORF8:Q27*,ORF8:R52I,ORF8:Y73C,S:N501Y,S:A570D,S:D614G,S:P681H,S:T716I,S:S940F,S:S982A,S:D1118H	6	0
hCoV-19/Mexico/CHH-InDRE_FB15893_S2394/2021	EPI_ISL_2455919	-	B.1.1.7	GRY	35	C241T,C913T,C3037T,C3267T,G5164T,C5388A,C5986T,T6954C,C7945T,C14408T,C14676T,C15279T,T16176C,A23063T,C23191T,C23271A,A23403G,C23604A,C23709T,T24506G,G24914C,A25336C,G25494A,C25710T,C27294T,C27972T,G28048T,A28111G,G28280C,A28281T,T28282A,G28881A,G28882A,G28883C,C28977T	20	N:D3L,N:R203K,N:G204R,N:S235F,ORF1a:T1001I,ORF1a:E1633D,ORF1a:A1708D,ORF1a:I2230T,ORF1b:P314L,ORF8:Q27*,ORF8:R52I,ORF8:Y73C,S:N501Y,S:A570D,S:D614G,S:P681H,S:T716I,S:S982A,S:D1118H,S:E1258D	6	0
hCoV-19/Mexico/CHH-InDRE_FB15895_S2395/2021	EPI_ISL_2455920	-	B.1.1.7	GRY	37	C241T,C913T,C3037T,C3267T,G4300T,C5388A,C5986T,C6525T,C6720T,T6954C,C9298T,T12118C,C14408T,C14676T,C15279T,T16176C,T21236A,A23063T,C23271A,A23403G,C23604A,C23709T,C24374T,T24506G,G24914C,A25336C,A26069G,C27972T,G28048T,A28111G,G28280C,A28281T,T28282A,G28881A,G28882A,G28883C,C28977T	24	N:D3L,N:R203K,N:G204R,N:S235F,ORF1a:T1001I,ORF1a:A1708D,ORF1a:T2087I,ORF1a:T2152I,ORF1a:I2230T,ORF1b:P314L,ORF1b:F2590Y,ORF3a:E226G,ORF8:Q27*,ORF8:R52I,ORF8:Y73C,S:N501Y,S:A570D,S:D614G,S:P681H,S:T716I,S:L938F,S:S982A,S:D1118H,S:E1258D	6	280
hCoV-19/Mexico/CHH-InDRE_FB16251_S2733/2021	EPI_ISL_2533759	-	B.1.1.7	GRY	32	C140G,C241T,C913T,C3037T,C3267T,C5388A,C5986T,T6954C,C9298T,C14408T,C14676T,C15279T,T16176C,G17523T,A23063T,C23271A,A23403G,C23604A,C23709T,C24374T,T24506G,G24914C,C27972T,G28048T,A28111G,G28280C,A28281T,T28282A,G28881A,G28882A,G28883C,C2	20	N:D3L,N:R203K,N:G204R,N:S235F,ORF1a:T1001I,ORF1a:A1708D,ORF1a:I2230T,ORF1b:P314L,ORF1b:M1352I,ORF8:Q27*,ORF8:R52I,ORF8:Y73C,S:N501Y,S:A570D,S:D614G,S:P681H,S:T716I,S:L938F,S:S982A,S:D1118H	6	0

hCoV-19/Mexico/CHH-InDRE_FB16252_S2734/2021	EPI_ISL_2533760	-	B.1.1.7	GRY	35	C241T,C913T,C3037T,C3267T,C5388A,C5812T,C5986T,T6954C,C9298T,C14408T,C14676T,C15279T,T16176C,A23063T,C23271A,A23403G,G23593T,C23604A,C23709T,C24374T,T24506G,G24914C,G27827T,C27972T,G28048T,A28111G,G28209T,G28280C,A28281T,T28282A,G28881A,G28882A,G28883C,C28977T,C29750T	22	N:D3L,N:R203K,N:G204R,N:S235F,ORF1a:T1001I,ORF1a:A1708D,ORF1a:I2230T,ORF1b:P314L,ORF7b:M24I,ORF8:Q27*,ORF8:R52I,ORF8:Y73C,ORF8:E106*,S:N501Y,S:A570D,S:D614G,S:Q677H,S:P681H,S:T716I,S:L938F,S:S982A,S:D1118H	6	0
hCoV-19/Mexico/CHH-InDRE_FB16254_S2735/2021	EPI_ISL_2533761	-	B.1.1.7	GRY	31	C241T,C913T,C3037T,C3267T,G5164T,C5388A,C5986T,T6954C,C14408T,G14653T,C14676T,C15279T,T16176C,A23063T,C23271A,A23403G,C23604A,C23709T,T24506G,G24914C,G25906A,C27972T,G28048T,A28111G,G28280C,A28281T,T28282A,G28881A,G28882A,G28883C,C28977T	21	N:D3L,N:R203K,N:G204R,N:S235F,ORF1a:T1001I,ORF1a:E1633D,ORF1a:A1708D,ORF1a:I2230T,ORF1b:P314L,ORF1b:V396F,ORF3a:G172S,ORF8:Q27*,ORF8:R52I,ORF8:Y73C,S:N501Y,S:A570D,S:D614G,S:P681H,S:T716I,S:S982A,S:D111	6	0
hCoV-19/Mexico/CHH-InDRE_FB16255_S2736/2021	EPI_ISL_2533762	-	B.1.1.7	GRY	33	C241T,C292T,C913T,C3037T,C3267T,C5388A,C5986T,T6954C,C13424T,C14408T,C14649T,C14676T,C15279T,T16176C,C18348T,C21789T,A23063T,C23271A,A23403G,C23604A,C23709T,T24506G,G24914C,C27972T,G28048T,A28111G,G28280C,A28281T,T28282A,G28881A,G28882A,G2	20	N:D3L,N:R203K,N:G204R,N:S235F,ORF1a:T1001I,ORF1a:A1708D,ORF1a:I2230T,ORF1a:R4387C,ORF1b:P314L,ORF8:Q27*,ORF8:R52I,ORF8:Y73C,S:T76I,S:N501Y,S:A570D,S:D614G,S:P681H,S:T716I,S:S982A,S:D1118H	6	0
hCoV-19/Mexico/CHH-InDRE_FB16972_S3127/2021	EPI_ISL_2663394	-	B.1.1.7	GRY	33	C241T,C913T,C3037T,C3267T,C5388A,C5986T,T6954C,C9298T,C14408T,C14676T,C15279T,T16176C,T22246C,A23063T,C23271A,A23403G,C23604A,C23709T,C24374T,T24506G,G24914C,C27513T,C27972T,G28048T,A28111G,G28280C,A28281T,T28282A,A28874G,G28881A,G28882A,G	20	N:D3L,N:S201G,N:R203K,N:G204R,N:S235F,ORF1a:T1001I,ORF1a:A1708D,ORF1a:I2230T,ORF1b:P314L,ORF8:Q27*,ORF8:R52I,ORF8:Y73C,S:N501Y,S:A570D,S:D614G,S:P681H,S:T716I,S:L938F,S:S982A,S:D1118H	6	0
hCoV-19/Mexico/CHH-InDRE_FB16976_S3128/2021	EPI_ISL_2663395	-	B.1.1.7	GRY	30	C241T,C913T,C3037T,C3267T,A3829G,C5388A,C5986T,T6954C,C14408T,C14676T,C15279T,T16176C,A23063T,C23271A,A23403G,C23604A,C23709T,T24506G,G24914C,C27972T,G28048T,A28111G,G28280C,A28281T,T28282A,G28881A,G28882A,G28883C,C28977T,C29580T	18	N:D3L,N:R203K,N:G204R,N:S235F,ORF1a:T1001I,ORF1a:A1708D,ORF1a:I2230T,ORF1b:P314L,ORF8:Q27*,ORF8:R52I,ORF8:Y73C,S:N501Y,S:A570D,S:D614G,S:P681H,S:T716I,S:S982A,S:D1118H	6	0
hCoV-19/Mexico/CHH-InDRE_FB16978_S3129/2021	EPI_ISL_2663396	-	B.1.1.7	GRY	33	C241T,C913T,C2453T,C3037T,C3267T,C5388A,C5986T,T6954C,C9611T,C14408T,C14676T,C15279T,T16176C,C21855T,G21974C,A23063T,C23271A,A23403G,C23604A,C23709T,T24506G,G24914C,C27972T,G28048T,A28111G,G28280C,A28281T,T28282A,A28295G,G28881A,G28882A,G	23	N:D3L,N:N8D,N:R203K,N:G204R,N:S235F,ORF1a:L730F,ORF1a:T1001I,ORF1a:A1708D,ORF1a:I2230T,ORF1a:L3116F,ORF1b:P314L,ORF8:Q27*,ORF8:R52I,ORF8:Y73C,S:S98F,S:D138H,S:N501Y,S:A570D,S:D614G,S:P681H,S:T716I,S:S982	6	0
hCoV-19/Mexico/CHH-InDRE_FB16984_S3130/2021	EPI_ISL_2663397	-	B.1.1.7	GRY	36	C241T,C913T,T1453C,C3037T,C3267T,C5388A,C5986T,T6954C,C14408T,C14676T,C15279T,T16176C,C21575T,A23063T,C23086T,C23271A,A23403G,C23604A,C23709T,G24357T,T24506G,C24904T,G24914C,C26833T,C26873T,C27972T,G28048T,A28111G,G28280C,A28281T,T28282A,G28881A,G28882A,G28883C,C28977T,C29614T	21	M:A104V,N:D3L,N:R203K,N:G204R,N:S235F,ORF1a:T1001I,ORF1a:A1708D,ORF1a:I2230T,ORF1b:P314L,ORF8:Q27*,ORF8:R52I,ORF8:Y73C,S:L5F,S:N501Y,S:A570D,S:D614G,S:P681H,S:T716I,S:G932V,S:S982A,S:D1118H	6	0
hCoV-19/Mexico/CHH-InDRE_FB16985_S3131/2021	EPI_ISL_2663398	-	B.1.1.7	GRY	33	G102A,C241T,G343T,C913T,C3037T,C3267T,C5388A,C5986T,T6954C,C14408T,C14649T,C14676T,C15279T,T16176C,C18348T,A23063T,C23271A,A23403G,C23604A,C23709T,T24506G,G24914C,G25537A,C27972T,G28048T,A28111G,G28280C,A28281T,T28282A,G28881A,G28882A,G288	19	N:D3L,N:R203K,N:G204R,N:S235F,ORF1a:T1001I,ORF1a:A1708D,ORF1a:I2230T,ORF1b:P314L,ORF3a:G49S,ORF8:Q27*,ORF8:R52I,ORF8:Y73C,S:N501Y,S:A570D,S:D614G,S:P681H,S:T716I,S:S982A,S:D1118H	6	0
hCoV-19/Mexico/CHH-InDRE_FB16989_S3940/2021	EPI_ISL_2858954	-	B.1.1.7	GRY	36	C241T,C913T,C3037T,C3267T,C5388A,C5986T,T6954C,C9532T,C10036T,C11750T,C14408T,C14676T,C15279T,T16176C,T21655A,C21736T,A23063T,C23271A,A23403G,C23604A,C23709T,T24506G,G24914C,A25336C,G25337C,C26270T,C27972T,G28048T,A28111G,G28280C,A28281T,T28282A,G28881A,G28882A,G28883C,C28977T	22	E:T9I,N:D3L,N:R203K,N:G204R,N:S235F,ORF1a:T1001I,ORF1a:A1708D,ORF1a:I2230T,ORF1a:L3829F,ORF1b:P314L,ORF8:Q27*,ORF8:R52I,ORF8:Y73C,S:N501Y,S:A570D,S:D614G,S:P681H,S:T716I,S:S982A,S:D1118H,S:E1258D,S:D1259H	6	358
hCoV-19/Mexico/CHH-InDRE_FB16992_S3941/2021	EPI_ISL_2858955	-	B.1.1.7	GR	32	C241T,C913T,C3037T,C3267T,C5388A,C5986T,T6954C,C14408T,C14676T,C15279T,T16176C,C21622T,C23271A,A23403G,C23604A,C23709T,C24381T,T24506G,G24914C,G25290T,A25336C,C27972T,G28048T,A28111G,G28280C,A28281T,T28282A,G28881A,G28882A,G28883C,C28977T,	21	N:D3L,N:R203K,N:G204R,N:S235F,N:D348Y,ORF1a:T1001I,ORF1a:A1708D,ORF1a:I2230T,ORF1b:P314L,ORF8:Q27*,ORF8:R52I,ORF8:Y73C,S:A570D,S:D614G,S:P681H,S:T716I,S:S940F,S:S982A,S:D1118H,S:C1243F,S:E1258D	6	978
hCoV-19/Mexico/CHH-InDRE_FB16993_S3942/2021	EPI_ISL_2858956	-	B.1.1.7	GRY	36	C241T,C913T,C3037T,C3267T,G5164T,C5388A,C5986T,T6954C,C7945T,C14408T,C14676T,C15279T,T16176C,A23063T,C23191T,C23271A,A23403G,C23604A,C23709T,T24506G,G24914C,A25336C,G25337C,G25494A,C25710T,C27294T,C27972T,G28048T,A28111G,G28280C,A28281T,T28282A,G28881A,G28882A,G28883C,C28977T	21	N:D3L,N:R203K,N:G204R,N:S235F,ORF1a:T1001I,ORF1a:E1633D,ORF1a:A1708D,ORF1a:I2230T,ORF1b:P314L,ORF8:Q27*,ORF8:R52I,ORF8:Y73C,S:N501Y,S:A570D,S:D614G,S:P681H,S:T716I,S:S982A,S:D1118H,S:E1258D,S:D1259H	6	0
hCoV-19/Mexico/CHH-InDRE_FB17409_S3925/2021	EPI_ISL_2858939	-	B.1.1.7	GRY	36	C241T,C913T,A2866G,C3037T,C3267T,A4503C,G5164T,C5388A,C5986T,T6954C,C14183T,C14408T,C14676T,C15279T,C15720T,T16176C,A23063T,C23271A,A23403G,C23604A,C23709T,T24506G,G24914C,C25549T,T27384C,C27972T,G28048T,A28111G,G28280C,A28281T,T28282A,G28881A,G28882A,G28883C,C28977T,C29741T	22	N:D3L,N:R203K,N:G204R,N:S235F,ORF1a:T1001I,ORF1a:E1413A,ORF1a:E1633D,ORF1a:A1708D,ORF1a:I2230T,ORF1b:T239I,ORF1b:P314L,ORF3a:L53F,ORF8:Q27*,ORF8:R52I,ORF8:Y73C,S:N501Y,S:A570D,S:D614G,S:P681H,S:T716I,S:S982A,S:D1118H	6	0
hCoV-19/Mexico/CHH-InDRE_FB17410_S3926/2021	EPI_ISL_2858940	-	B.1.1.7	GRY	32	C241T,C913T,C3037T,C3267T,C5388A,C5986T,T6954C,C9298T,C14408T,C14605T,C14676T,C15279T,T16176C,G18907T,A23063T,C23271A,A23403G,C23604A,C23709T,C24374T,T24506G,G24914C,C27972T,G28048T,A28111G,G28280C,A28281T,T28282A,G28881A,G28882A,G28883C,	20	N:D3L,N:R203K,N:G204R,N:S235F,ORF1a:T1001I,ORF1a:A1708D,ORF1a:I2230T,ORF1b:P314L,ORF1b:V1814F,ORF8:Q27*,ORF8:R52I,ORF8:Y73C,S:N501Y,S:A570D,S:D614G,S:P681H,S:T716I,S:L938F,S:S982A,S:D1118H	6	0
hCoV-19/Mexico/CHH-InDRE_FB18221_S4131/2021	EPI_ISL_2920702	-	B.1.1.7	GRY	33	C241T,C913T,C3037T,C3267T,G5164T,C5388A,C5850T,C5986T,T6954C,C10790T,C14408T,C14676T,C15279T,T16176C,C17883T,A23063T,C23271A,A23403G,C23604A,C23709T,T24506G,G24914C,C27972T,G28048T,A28111G,G28280C,A28281T,T28282A,G28881A,G28882A,G28883C,C2	21	N:D3L,N:R203K,N:G204R,N:S235F,ORF1a:T1001I,ORF1a:E1633D,ORF1a:A1708D,ORF1a:P1862L,ORF1a:I2230T,ORF1a:H3509Y,ORF1b:P314L,ORF8:Q27*,ORF8:R52I,ORF8:Y73C,S:N501Y,S:A570D,S:D614G,S:P681H,S:T716I,S:S982A,S:D1	6	0
hCoV-19/Mexico/CHH-InDRE_FB18222_S4132/2021	EPI_ISL_2920703	-	B.1.1.7	GRY	33	C241T,C913T,C3037T,C3267T,G5164T,C5388A,C5986T,C6573T,T6954C,C14408T,G14653T,C14676T,C15279T,T16176C,A23063T,C23271A,A23403G,C23604A,C23709T,T24506G,G24914C,G25906A,C27603T,C27972T,G28048T,A28111G,G28280C,A28281T,T28282A,G28881A,G28882A,G28883C,C28977T	22	N:D3L,N:R203K,N:G204R,N:S235F,ORF1a:T1001I,ORF1a:E1633D,ORF1a:A1708D,ORF1a:S2103F,ORF1a:I2230T,ORF1b:P314L,ORF1b:V396F,ORF3a:G172S,ORF8:Q27*,ORF8:R52I,ORF8:Y73C,S:N501Y,S:A570D,S:D614G,S:P681H,S:T716I,S:S982A,S:D1118H	6	0
hCoV-19/Mexico/CHH-InDRE_FB18224_S4133/2021	EPI_ISL_2920704	-	Q.8	G	25	C241T,C282T,C913T,C3037T,C5388A,C5986T,C6843T,T6954C,C14408T,C14676T,C15279T,T16176C,C17738T,A23063T,A23403G,C23604A,C23709T,G24914C,G26152T,C27972T,G28048T,A28111G,T28322G,G28881T,C28977T	18	N:F17V,N:R203M,N:S235F,ORF1a:P6L,ORF1a:A1708D,ORF1a:S2193F,ORF1a:I2230T,ORF1b:P314L,ORF1b:T1424I,ORF3a:G254*,ORF8:Q27*,ORF8:R52I,ORF8:Y73C,S:N501Y,S:D614G,S:P681H,S:T716I,S:D1118H	6	56
hCoV-19/Mexico/CHH-InDRE_FB18225_S3965/2021	EPI_ISL_2858979	-	B.1.1.7	GRY	36	C241T,C913T,C3037T,C3267T,G5164T,C5388A,C5986T,T6954C,C7945T,C14408T,C14676T,C15279T,T16176C,A23063T,C23191T,C23271A,A23403G,C23604A,C23709T,T24506G,G24914C,A25336C,G25494A,C25710T,C27213T,C27294T,C27972T,G28048T,A28111G,G28280C,A28281T,T28282A,G28881A,G28882A,G28883C,C28977T	20	N:D3L,N:R203K,N:G204R,N:S235F,ORF1a:T1001I,ORF1a:E1633D,ORF1a:A1708D,ORF1a:I2230T,ORF1b:P314L,ORF8:Q27*,ORF8:R52I,ORF8:Y73C,S:N501Y,S:A570D,S:D614G,S:P681H,S:T716I,S:S982A,S:D1118H,S:E1258D	6	0
hCoV-19/Mexico/CHH-INMEGEN-InDRE-04-04-126/2021	EPI_ISL_1628624	-	B.1.1.7	GRY	31	C241T,C913T,C3037T,C3267T,C5388A,C5986T,T6954C,C14408T,C14649T,C14676T,C15279T,T16176C,C18348T,A23063T,C23114T,C23271A,A23403G,C23604A,C23709T,T24506G,G24914C,C27972T,G28048T,A28111G,G28280C,A28281T,T28282A,G28881A,G28882A,G28883C,C28977T	18	N:D3L,N:R203K,N:G204R,N:S235F,ORF1a:T1001I,ORF1a:A1708D,ORF1a:I2230T,ORF1b:P314L,ORF8:Q27*,ORF8:R52I,ORF8:Y73C,S:N501Y,S:A570D,S:D614G,S:P681H,S:T716I,S:S982A,S:D1118H	6	7
hCoV-19/Mexico/CHP-InDRE_FB17387_S3482/2021	EPI_ISL_2778997	-	B.1.1.7	GRY	34	C241T,C245T,C913T,C3037T,C3267T,A3492G,C5388A,C5986T,T6954C,C14408T,C14676T,C15279T,T16176C,C21639A,A23063T,C23271A,A23403G,C23604A,C23709T,C24374T,T24506G,G24914C,A25336C,G25337C,C27972T,G28048T,A28111G,G28280C,A28281T,T28282A,G28881A,G2	23	N:D3L,N:R203K,N:G204R,N:S235F,ORF1a:T1001I,ORF1a:N1076S,ORF1a:A1708D,ORF1a:I2230T,ORF1b:P314L,ORF8:Q27*,ORF8:R52I,ORF8:Y73C,S:P26H,S:N501Y,S:A570D,S:D614G,S:P681H,S:T716I,S:L938F,S:S982A,S:D1118H,S:E1258	6	0
hCoV-19/Mexico/CMX-InDRE_F12295_S1200/2021	EPI_ISL_1626807	-	B.1.1.7	GRY	35	G174T,G210T,C241T,C913T,C2453T,C3037T,C3267T,C5388A,C5986T,T6954C,A8588G,T9016C,C14408T,C14676T,C15279T,T16176C,A23063T,C23271A,A23403G,C23604A,C23709T,T24506G,G24914C,A25336C,C27883T,C27972T,G28048T,A28111G,G28280C,A28281T,T28282A,G28881A,G28882A,G28883C,C28977T	22	N:D3L,N:R203K,N:G204R,N:S235F,ORF1a:L730F,ORF1a:T1001I,ORF1a:A1708D,ORF1a:I2230T,ORF1a:K2775E,ORF1b:P314L,ORF7b:A43V,ORF8:Q27*,ORF8:R52I,ORF8:Y73C,S:N501Y,S:A570D,S:D614G,S:P681H,S:T716I,S:S982A,S:D1118H,S:E1258D	6	0
hCoV-19/Mexico/CMX-InDRE_F34543_S900/2021	EPI_ISL_1423510	-	B.1.1.7	GRY	37	C241T,C913T,C2453T,C3037T,C3267T,G3483T,C5388A,C5986T,T6954C,C9611T,C14408T,C14676T,G14874T,C15279T,T16176C,C19524T,C21855T,G21974C,A23063T,C23271A,A23403G,C23604A,C23709T,T24506G,G24914C,G27461T,C27972T,G28048T,A28111G,G28280C,A28281T,T28282A,A28295G,G28881A,G28882A,G28883C,C28977T	26	N:D3L,N:N8D,N:R203K,N:G204R,N:S235F,ORF1a:L730F,ORF1a:T1001I,ORF1a:G1073V,ORF1a:A1708D,ORF1a:I2230T,ORF1a:L3116F,ORF1b:P314L,ORF1b:K469N,ORF7a:C23F,ORF8:Q27*,ORF8:R52I,ORF8:Y73C,S:S98F,S:D138H,S:N501Y,S:A570D,S:D614G,S:P681H,S:T716I,S:S982A,S:D1118H	6	0
hCoV-19/Mexico/CMX-InDRE_FB13260_S1502/2021	EPI_ISL_1805480	-	B.1.1.7	GRY	40	T78G,C241T,C913T,C3037T,C3267T,C5388A,C5986T,G6647A,T6954C,C14408T,C14676T,T15096C,C15279T,T16176C,C17676T,C18183T,C19524T,A21519C,G22217A,A23063T,C23271A,A23403G,C23604A,C23709T,A24070T,T24506G,G24914C,A25336C,C25624T,C27972T,G28048T,A28111G,G28280C,A28281T,T28282A,G28881A,G28882A,G28883C,G28884C,C28977T	24	N:D3L,N:R203K,N:G204P,N:S235F,ORF1a:T1001I,ORF1a:A1708D,ORF1a:A2128T,ORF1a:I2230T,ORF1b:P314L,ORF1b:R2684S,ORF3a:H78Y,ORF8:Q27*,ORF8:R52I,ORF8:Y73C,S:G219S,S:N501Y,S:A570D,S:D614G,S:P681H,S:T716I,S:Q836H,S:S982A,S:D1118H,S:E1258D	6	0

hCoV-19/Mexico/CMX-InDRE_FB16175_S2435/2021	EPI_ISL_2455959	-	B.1.1.7	GRY	42	C241T,C913T,C3037T,C3267T,C3966T,C4206T,C5388A,C5986T,T6954C,A12162G,C14408T,C14676T,C15279T,G15594T,C15720T,T16176C,A17615G,G18498T,A21137G,A23063T,G23270T,C23271A,A23403G,C23604A,C23709T,T24506G,G24914C,G25135T,C26110T,C27236T,C27972T,G28048T,G28079T,A28111G,G28280C,A28281T,T28282A,G28881A,G28882A,G28883C,C28977T,G29227T	28	N:D3L,N:R203K,N:G204R,N:S235F,ORF1a:T1001I,ORF1a:A1234V,ORF1a:A1314V,ORF1a:A1708D,ORF1a:I2230T,ORF1a:Q3966R,ORF1b:P314L,ORF1b:K709N,ORF1b:K1383R,ORF1b:M1677I,ORF1b:K2557R,ORF3a:P240S,ORF6:A12V,ORF8:Q27*,ORF8:R52I,ORF8:Y73C,S:N501Y,S:A570Y,S:D614G,S:P681H,S:T716I,S:S982A,S:D1118H,S:K1191N	6	0
hCoV-19/Mexico/CMX-InDRE_FB16521_S3061/2021	EPI_ISL_2663328	-	B.1.1.7	GRY	36	C241T,C913T,C2110T,C3037T,C3267T,C5388A,C5986T,T6954C,G7042T,A10195G,C10279T,C14120T,C14408T,C14676T,C15279T,T16176C,C21588T,A23063T,C23271A,A23403G,C23604A,C23709T,T24506G,T24565C,G24914C,C27972T,G28048T,G28075T,A28111G,G28280C,A28281T,T28282A,G28881A,G28882A,G28883C,C28977T	22	N:D3L,N:R203K,N:G204R,N:S235F,ORF1a:T1001I,ORF1a:A1708D,ORF1a:I2230T,ORF1a:M2259I,ORF1b:P218L,ORF1b:P314L,ORF8:Q27*,ORF8:R52I,ORF8:C61F,ORF8:Y73C,S:P9L,S:N501Y,S:A570D,S:D614G,S:P681H,S:T716I,S:S982A,S:D1118H	6	75
hCoV-19/Mexico/CMX-InDRE_FB16522_S3062/2021	EPI_ISL_2663329	-	B.1.1.7	GRY	37	C241T,C913T,C2110T,C3037T,C3267T,G3482A,G3728T,C5388A,C5986T,T6954C,T7984C,C14120T,C14408T,C14676T,C15279T,T16176C,C19390T,G21600T,A23063T,C23271A,A23403G,C23604A,C23709T,T24506G,G24914C,A25336C,G25337C,C27972T,G28048T,A28111G,G28280C,A28281T,T28282A,G28881A,G28882A,G28883C,C28977T	25	N:D3L,N:R203K,N:G204R,N:S235F,ORF1a:T1001I,ORF1a:G1073R,ORF1a:G1155C,ORF1a:A1708D,ORF1a:I2230T,ORF1b:P218L,ORF1b:P314L,ORF1b:P1975S,ORF8:Q27*,ORF8:R52I,ORF8:Y73C,S:S13I,S:N501Y,S:A570D,S:D614G,S:P681H,S:T716I,S:S982A,S:D1118H,S:E1258D,S:D1259H	6	0
hCoV-19/Mexico/CMX-InDRE_FB16523_S3063/2021	EPI_ISL_2663330	-	B.1.1.7	GRY	37	C241T,C913T,C2110T,C3037T,C3267T,G3482A,G3728T,C5388A,C5986T,T6954C,T7984C,C14120T,C14408T,C14676T,C15279T,T16176C,C19390T,G21600T,A23063T,C23271A,A23403G,C23604A,C23709T,T24506G,G24914C,A25336C,G25337C,C27972T,G28048T,A28111G,G28280C,A28281T,T28282A,G28881A,G28882A,G28883C,C28977T	25	N:D3L,N:R203K,N:G204R,N:S235F,ORF1a:T1001I,ORF1a:G1073R,ORF1a:G1155C,ORF1a:A1708D,ORF1a:I2230T,ORF1b:P218L,ORF1b:P314L,ORF1b:P1975S,ORF8:Q27*,ORF8:R52I,ORF8:Y73C,S:S13I,S:N501Y,S:A570D,S:D614G,S:P681H,S:T716I,S:S982A,S:D1118H,S:E1258D,S:D1259H	6	0
hCoV-19/Mexico/CMX-InDRE_FB16969_S3126/2021	EPI_ISL_2663393	-	B.1.1.7	GRY	38	C241T,C913T,C3037T,C3267T,C4206T,C5388A,C5986T,T6954C,G7272A,C10525T,C11572T,C12933T,C14408T,C14676T,C15279T,T16176C,A21466G,C21843T,T22441A,A23063T,C23271A,A23403G,C23604A,C23709T,T24506G,G24914C,C27972T,G28048T,A28111G,G28280C,A28281T,T28282A,G28881A,G28882A,G28883C,C28977T,C29272T,C29784T	23	N:D3L,N:R203K,N:G204R,N:S235F,ORF1a:T1001I,ORF1a:A1314V,ORF1a:A1708D,ORF1a:I2230T,ORF1a:R2336K,ORF1a:P4223L,ORF1b:P314L,ORF1b:M2667V,ORF8:Q27*,ORF8:R52I,ORF8:Y73C,S:S94F,S:N501Y,S:A570D,S:D614G,S:P681H,S:T716I,S:S982A,S:D1118H	6	0
hCoV-19/Mexico/CMX-InDRE_FB17954_S3934/2021	EPI_ISL_2858948	-	B.1.1.7	GRY	42	C241T,C913T,C3037T,C3267T,C5388A,C5986T,G6647A,C6936T,T6954C,C14408T,C14621T,C14676T,T15096C,C15279T,T16176C,C17676T,C18183T,C19524T,A21519C,A23063T,C23271A,A23403G,C23604A,C23709T,A24070T,A24092G,T24506G,G24914C,A25020G,C25624T,C27737T,C27972T,G28048T,A28111G,G28280C,A28281T,T28282A,G28881A,G28882A,G28883C,G28884C,C	27	N:D3L,N:R203K,N:G204P,N:S235F,ORF1a:T1001I,ORF1a:A1708D,ORF1a:A2128T,ORF1a:S2224F,ORF1a:I2230T,ORF1b:P314L,ORF1b:T385M,ORF1b:R2684S,ORF3a:H78Y,ORF7a:T115I,ORF8:Q27*,ORF8:R52I,ORF8:Y73C,S:N501Y,S:A570D,S:D614G,S:P681H,S:T716I,S:Q836H,S:I844V,S:S982A,S:D1118H,S:D1153G	6	142
hCoV-19/Mexico/CMX-InDRE_FB17958_S3935/2021	EPI_ISL_2858949	-	B.1.1.7	GRY	37	C241T,C823T,C913T,C2110T,G2305T,G3004T,C3037T,C3267T,C5388A,C5986T,T6954C,G7042T,C9803T,C14120T,C14408T,C14676T,C15279T,T16176C,A23063T,C23271A,A23403G,C23604A,C23709T,T24506G,G24914C,A25659G,C26110T,C27972T,G28048T,A28111G,G28280C,A28281T,T28282A,G28881A,G28882A,G28883C,C28977T	23	N:D3L,N:R203K,N:G204R,N:S235F,ORF1a:K680N,ORF1a:E913D,ORF1a:T1001I,ORF1a:A1708D,ORF1a:I2230T,ORF1a:M2259I,ORF1b:P218L,ORF1b:P314L,ORF3a:P240S,ORF8:Q27*,ORF8:R52I,ORF8:Y73C,S:N501Y,S:A570D,S:D614G,S:P681H,S:T716I,S:S982A,S:D1118H	6	0
hCoV-19/Mexico/CMX-InDRE_FB17960_S3936/2021	EPI_ISL_2858950	-	B.1.1.7	GRY	42	C241T,C913T,C3037T,C3267T,C5388A,C5986T,G6647A,T6954C,A10486G,C14408T,C14676T,T15096C,C15279T,T16176C,C17676T,C18183T,C19524T,A21519C,G22217A,A23063T,C23271A,A23403G,C23604A,C23709T,A24070T,T24506G,G24914C,A25336C,C25624T,G26918T,T27384C,C27972T,G28048T,A28111G,G28280C,A28281T,T28282A,G28881A,G28882A,G28883C,G28884C,	24	N:D3L,N:R203K,N:G204P,N:S235F,ORF1a:T1001I,ORF1a:A1708D,ORF1a:A2128T,ORF1a:I2230T,ORF1b:P314L,ORF1b:R2684S,ORF3a:H78Y,ORF8:Q27*,ORF8:R52I,ORF8:Y73C,S:G219S,S:N501Y,S:A570D,S:D614G,S:P681H,S:T716I,S:Q836H,S:S982A,S:D1118H,S:E1258D	6	0
hCoV-19/Mexico/CMX-InDRE_FB17970_S3937/2021	EPI_ISL_2858951	-	B.1.1.7	GRY	39	C241T,C913T,C3037T,C3267T,C5388A,C5986T,G6647A,C6936T,T6954C,C14408T,C14621T,C14676T,C15279T,T16176C,C18183T,A21519C,A23063T,C23271A,A23403G,C23604A,C23709T,A24070T,A24092G,T24506G,G24914C,A25336C,C25624T,C27737T,C27972T,G28048T,A28111G,G28280C,A28281T,T28282A,G28881A,G28882A,G28883C,G28884C,C28977T	27	N:D3L,N:R203K,N:G204P,N:S235F,ORF1a:T1001I,ORF1a:A1708D,ORF1a:A2128T,ORF1a:S2224F,ORF1a:I2230T,ORF1b:P314L,ORF1b:T385M,ORF1b:R2684S,ORF3a:H78Y,ORF7a:T115I,ORF8:Q27*,ORF8:R52I,ORF8:Y73C,S:N501Y,S:A570D,S:D614G,S:P681H,S:T716I,S:Q836H,S:I844V,S:S982A,S:D1118H,S:E1258D	6	0
hCoV-19/Mexico/CMX-InDRE_FB19386_S4507/2021	EPI_ISL_3033361	-	B.1.1.7	GRY	39	C241T,C913T,T1234C,G1598A,C2453T,C3037T,C3267T,C5388A,C5986T,T6954C,C9611T,C14408T,C14676T,C15279T,T16176C,C21008T,C21855T,G21974C,A23063T,C23271A,A23403G,C23604A,C23709T,T24506G,G24914C,C27643T,C27972T,G28048T,A28111G,T28149A,A28247G,G28280C,A28281T,T28282A,A28295G,G28881A,G28882A,G28883C,C28977T	27	N:D3L,N:N8D,N:R203K,N:G204R,N:S235F,ORF1a:G445S,ORF1a:L730F,ORF1a:T1001I,ORF1a:A1708D,ORF1a:I2230T,ORF1a:L3116F,ORF1b:P314L,ORF1b:T2514I,ORF7a:P84S,ORF8:Q27*,ORF8:R52I,ORF8:Y73C,ORF8:F86I,S:S98F,S:D138H,S:N501Y,S:A570D,S:D614G,S:P681H,S:T716I,S:S982A,S:D1118H	6	0
hCoV-19/Mexico/CMX-InDRE_FB19393_S4508/2021	EPI_ISL_3046076	-	B.1.1.7	GRY	39	C241T,C913T,C2453T,C3037T,C3267T,A3481T,C5388A,C5986T,T6954C,C9611T,C10543T,C14408T,C14676T,C15279T,T16176C,T19305C,C21008T,C21855T,G21974C,A23063T,C23271A,A23403G,C23604A,C23709T,T24506G,G24914C,G25437T,C27643T,C27972T,G28048T,A28111G,G28280C,A28281T,T28282A,A28295G,G28881A,G28882A,G28883C,C28977T	26	N:D3L,N:N8D,N:R203K,N:G204R,N:S235F,ORF1a:L730F,ORF1a:T1001I,ORF1a:A1708D,ORF1a:I2230T,ORF1a:L3116F,ORF1b:P314L,ORF1b:T2514I,ORF3a:L15F,ORF7a:P84S,ORF8:Q27*,ORF8:R52I,ORF8:Y73C,S:S98F,S:D138H,S:N501Y,S:A570D,S:D614G,S:P681H,S:T716I,S:S982A,S:D1118H	6	0
hCoV-19/Mexico/CMX-InDRE_FB19400_S4509/2021	EPI_ISL_3033362	-	B.1.1.7	GRY	39	C241T,C913T,A2317T,C2453T,C3037T,C3267T,C5388A,C5986T,C6433T,T6954C,C9611T,C11750T,C14408T,C14676T,C15279T,T16176C,C21008T,C21855T,G21974C,A23063T,C23271A,A23403G,C23604A,C23709T,T24506G,G24914C,C27643T,C27972T,T27977C,G28048T,A28111G,G28280C,A28281T,T28282A,A28295G,G28881A,G28882A,G28883C,C28977T	27	N:D3L,N:N8D,N:R203K,N:G204R,N:S235F,ORF1a:K684N,ORF1a:L730F,ORF1a:T1001I,ORF1a:A1708D,ORF1a:I2230T,ORF1a:L3116F,ORF1a:L3829F,ORF1b:P314L,ORF1b:T2514I,ORF7a:P84S,ORF8:Q27*,ORF8:R52I,ORF8:Y73C,S:S98F,S:D138H,S:N501Y,S:A570D,S:D614G,S:P681H,S:T716I,S:S982A,S:D1118H	6	0
hCoV-19/Mexico/CMX-InDRE_FD45086_S1508/2021	EPI_ISL_1805486	-	B.1.1.7	GRY	40	T78G,C241T,C913T,T1107C,C2110T,C3037T,C3267T,C5388A,C5986T,G6166A,T6954C,T7984C,C8247T,C14120T,C14408T,C14676T,C15279T,T16176C,C19032T,C19390T,A23063T,C23271A,A23403G,C23604A,C23709T,T24506G,G24914C,A25336C,G25337C,C26882T,C27972T,G28048T,A28111G,G28280C,A28281T,T28282A,G28881A,G28882A,G28883C,C28977T	24	N:D3L,N:R203K,N:G204R,N:S235F,ORF1a:I281T,ORF1a:T1001I,ORF1a:A1708D,ORF1a:I2230T,ORF1a:S2661F,ORF1b:P218L,ORF1b:P314L,ORF1b:P1975S,ORF8:Q27*,ORF8:R52I,ORF8:Y73C,S:N501Y,S:A570D,S:D614G,S:P681H,S:T716I,S:S982A,S:D1118H,S:E1258D,S:D1259H	6	22
hCoV-19/Mexico/CMX-InDRE_FD49735_S1767/2021	EPI_ISL_2101906	-	B.1.1.7	GRY	34	C241T,C913T,C3037T,C3267T,C5388A,C5986T,C6196T,T6954C,A8848G,C14408T,C14676T,C15279T,T16176C,T19624C,A23063T,C23271A,A23403G,C23604A,C23709T,T24506G,G24914C,G25217T,A25336C,C27972T,G28048T,A28111G,G28280C,A28281T,T28282A,G28881A,G28882A,G2	20	N:D3L,N:R203K,N:G204R,N:S235F,ORF1a:T1001I,ORF1a:A1708D,ORF1a:I2230T,ORF1b:P314L,ORF8:Q27*,ORF8:R52I,ORF8:Y73C,S:N501Y,S:A570D,S:D614G,S:P681H,S:T716I,S:S982A,S:D1118H,S:G1219C,S:E1258D	6	0
hCoV-19/Mexico/CMX-InDRE_FD54664_S1768/2021	EPI_ISL_2101907	-	B.1.1.7	GRY	34	C241T,C913T,C1943T,C2110T,C3037T,C3267T,C5388A,C5986T,T6954C,T7984C,C14120T,C14408T,C14676T,C15279T,T16176C,C19390T,A23063T,C23271A,A23403G,C23604A,C23709T,T24506G,G24914C,C25207T,C27972T,G28048T,A28111G,G28280C,A28281T,T28282A,G28881A,G28	21	N:D3L,N:R203K,N:G204R,N:S235F,ORF1a:R560C,ORF1a:T1001I,ORF1a:A1708D,ORF1a:I2230T,ORF1b:P218L,ORF1b:P314L,ORF1b:P1975S,ORF8:Q27*,ORF8:R52I,ORF8:Y73C,S:N501Y,S:A570D,S:D614G,S:P681H,S:T716I,S:S982A,S:D111	6	0
hCoV-19/Mexico/CMX-InDRE_FD54888_S1769/2021	EPI_ISL_2101908	-	B.1.1.7	GRY	40	C241T,C913T,C3037T,C3267T,C5388A,C5986T,C6555T,G6647A,T6954C,C14408T,C14676T,T15096C,C15279T,T16176C,C17676T,C18183T,C19524T,A21519C,G22217A,A23063T,C23271A,A23403G,C23604A,C23709T,A24070T,T24506G,G24914C,A25336C,C25624T,C27972T,G28048T,A28111G,G28280C,A28281T,T28282A,G28881A,G28882A,G28883C,G28884C,C28977T	25	N:D3L,N:R203K,N:G204P,N:S235F,ORF1a:T1001I,ORF1a:A1708D,ORF1a:A2097V,ORF1a:A2128T,ORF1a:I2230T,ORF1b:P314L,ORF1b:R2684S,ORF3a:H78Y,ORF8:Q27*,ORF8:R52I,ORF8:Y73C,S:G219S,S:N501Y,S:A570D,S:D614G,S:P681H,S:T716I,S:Q836H,S:S982A,S:D1118H,S:E1258D	6	18
hCoV-19/Mexico/CMX-InDRE_FD55350_S1770/2021	EPI_ISL_2101909	-	B.1.1.7	GRY	36	C241T,C913T,C2143T,C2453T,C3037T,C3267T,C5388A,C5986T,T6954C,C14408T,C14676T,T15096C,C15279T,T16176C,A23063T,C23271A,A23403G,C23604A,C23709T,G24089A,T24506G,G24914C,A25336C,G25785T,C27476T,C27972T,G28048T,A28111G,G28280C,A28281T,T28282A,G28881A,G28882A,G28883C,C28977T,C29642T	23	N:D3L,N:R203K,N:G204R,N:S235F,ORF1a:L730F,ORF1a:T1001I,ORF1a:A1708D,ORF1a:I2230T,ORF1b:P314L,ORF3a:W131C,ORF7a:T28I,ORF8:Q27*,ORF8:R52I,ORF8:Y73C,S:N501Y,S:A570D,S:D614G,S:P681H,S:T716I,S:D843N,S:S982A,S:D1118H,S:E1258D	6	0
hCoV-19/Mexico/CMX-InDRE_FD57542_S1771/2021	EPI_ISL_2101910	-	B.1.1.7	GRY	39	C241T,C913T,C3037T,C3267T,C5388A,C5986T,G6647A,T6954C,T9157C,C14408T,C14676T,T15096C,C15279T,T16176C,C17676T,C18183T,C19524T,A21519C,A23063T,C23271A,A23403G,C23604A,C23709T,A24070T,T24506G,G24914C,A25336C,C25624T,C27972T,G28048T,A28111G,G28280C,A28281T,T28282A,G28881A,G28882A,G28883C,G28884C,C28977T	23	N:D3L,N:R203K,N:G204P,N:S235F,ORF1a:T1001I,ORF1a:A1708D,ORF1a:A2128T,ORF1a:I2230T,ORF1b:P314L,ORF1b:R2684S,ORF3a:H78Y,ORF8:Q27*,ORF8:R52I,ORF8:Y73C,S:N501Y,S:A570D,S:D614G,S:P681H,S:T716I,S:Q836H,S:S982A,S:D1118H,S:E1258D	6	311
hCoV-19/Mexico/CMX-InDRE_FD58317_S1772/2021	EPI_ISL_2101911	-	B.1.1.7	GRY	38	C241T,C913T,C3037T,C3267T,C5388A,C5986T,G6647A,T6954C,C14408T,C14676T,T15096C,C15279T,T16176C,C17676T,C18183T,C19524T,A21519C,A23063T,C23271A,A23403G,C23604A,C23709T,A24070T,T24506G,G24914C,A25336C,C25624T,C27972T,G28048T,A28111G,G28280C,A28281T,T28282A,G28881A,G28882A,G28883C,G28884C,C28977T	23	N:D3L,N:R203K,N:G204P,N:S235F,ORF1a:T1001I,ORF1a:A1708D,ORF1a:A2128T,ORF1a:I2230T,ORF1b:P314L,ORF1b:R2684S,ORF3a:H78Y,ORF8:Q27*,ORF8:R52I,ORF8:Y73C,S:N501Y,S:A570D,S:D614G,S:P681H,S:T716I,S:Q836H,S:S982A,S:D1118H,S:E1258D	6	81

hCoV-19/Mexico/CMX-InDRE_FD58467_S1773/2021	EPI_ISL_2101912	-	B.1.1.7	GRY	36	C241T,C583T,C913T,C2453T,C3037T,C3267T,C5388A,C5986T,T6954C,G12892A,C13647T,C14408T,C14676T,C15279T,T16176C,C18167T,A23063T,C23271A,A23403G,C23604A,C23709T,T24506G,G24914C,A25336C,A26105G,C27972T,G28048T,A28111G,G28280C,A28281T,T28282A,G28881A,G28882A,G28883C,C28977T,C29550T	22	N:D3L,N:R203K,N:G204R,N:S235F,ORF1a:L730F,ORF1a:T1001I,ORF1a:A1708D,ORF1a:I2230T,ORF1b:P314L,ORF1b:P1567L,ORF3a:D238G,ORF8:Q27*,ORF8:R52I,ORF8:Y73C,S:N501Y,S:A570D,S:D614G,S:P681H,S:T716I,S:S982A,S:D1118H,S:E1258D	6	13
hCoV-19/Mexico/CMX-InDRE_FD63274_S2065/2021	EPI_ISL_2283705	-	B.1.1.7	GRY	39	C203T,C241T,C913T,G1820A,C3037T,C3267T,C5388A,C5986T,T6954C,G9526T,A12162G,C14408T,C14676T,C15279T,C15720T,T16176C,A17615G,G18301A,T19363C,A21137G,A23063T,C23248T,C23271A,A23403G,C23604A,C23709T,T24506G,G24914C,G25135T,C27972T,G28048T,A28111G,G28280C,A28281T,T28282A,G28881A,G28882A,G28883C,C28977T	25	N:D3L,N:R203K,N:G204R,N:S235F,ORF1a:G519S,ORF1a:T1001I,ORF1a:A1708D,ORF1a:I2230T,ORF1a:M3087I,ORF1a:Q3966R,ORF1b:P314L,ORF1b:K1383R,ORF1b:G1612S,ORF1b:K2557R,ORF8:Q27*,ORF8:R52I,ORF8:Y73C,S:N501Y,S:A570D,S:D614G,S:P681H,S:T716I,S:S982A,S:D1118H,S:K1191N	6	0
hCoV-19/Mexico/CMX-InDRE_FD63747_S2064/2021	EPI_ISL_2283704	-	B.1.1.7	GRY	35	C241T,C913T,C2453T,C3037T,C3267T,C4794T,C5388A,C5986T,T6954C,C9611T,C14408T,C14676T,C15279T,T16176C,C21855T,G21974C,A23063T,C23271A,A23403G,C23604A,C23709T,G24348T,T24506G,G24914C,C27972T,G28048T,A28111G,G28280C,A28281T,T28282A,A28295G,G28881A,G28882A,G28883C,C28977T	25	N:D3L,N:N8D,N:R203K,N:G204R,N:S235F,ORF1a:L730F,ORF1a:T1001I,ORF1a:S1510F,ORF1a:A1708D,ORF1a:I2230T,ORF1a:L3116F,ORF1b:P314L,ORF8:Q27*,ORF8:R52I,ORF8:Y73C,S:S98F,S:D138H,S:N501Y,S:A570D,S:D614G,S:P681H,S:T716I,S:S929I,S:S982A,S:D1118H	6	0
hCoV-19/Mexico/CMX-InDRE_FD63907_S2067/2021	EPI_ISL_2283707	-	B.1.1.7	GRY	34	C241T,C913T,C3037T,C3267T,C5388A,C5986T,C6196T,T6954C,A8848G,C14408T,C14676T,C15279T,T16176C,A23063T,C23271A,A23403G,C23604A,C23709T,T24506G,G24914C,G25217T,G26727T,C27972T,G28048T,A28111G,G28280C,A28281T,T28282A,G28881A,G28882A,G28883C,C2	20	M:A69S,N:D3L,N:R203K,N:G204R,N:S235F,ORF1a:T1001I,ORF1a:A1708D,ORF1a:I2230T,ORF1b:P314L,ORF8:Q27*,ORF8:R52I,ORF8:Y73C,S:N501Y,S:A570D,S:D614G,S:P681H,S:T716I,S:S982A,S:D1118H,S:G1219C	6	0
hCoV-19/Mexico/CMX-InDRE_FD64062_S2068/2021	EPI_ISL_2283708	-	B.1.1.7	GRY	36	C241T,C913T,T2486C,C3037T,C3267T,C5388A,C5986T,C6196T,T6954C,A8848G,C14408T,C14676T,C15279T,T16176C,A23063T,C23271A,T23332C,A23403G,C23604A,C23709T,T24506G,G24914C,G25217T,G25538A,G26727T,C27972T,G28048T,A28111G,G28280C,A28281T,T28282A,G28881A,G28882A,G28883C,C28977T,G29715T	22	M:A69S,N:D3L,N:R203K,N:G204R,N:S235F,ORF1a:F741L,ORF1a:T1001I,ORF1a:A1708D,ORF1a:I2230T,ORF1b:P314L,ORF3a:G49D,ORF8:Q27*,ORF8:R52I,ORF8:Y73C,S:N501Y,S:A570D,S:D614G,S:P681H,S:T716I,S:S982A,S:D1118H,S:G1219C	6	0
hCoV-19/Mexico/CMX-InDRE_FD64177_S2066/2021	EPI_ISL_2283706	-	B.1.1.7	GRY	39	C241T,C913T,G1299A,C2110T,C2388T,C3037T,C3267T,C3743T,C5388A,C5986T,T6954C,G7042T,A10195G,C14120T,C14408T,C14676T,C15279T,T16176C,A23063T,C23271A,A23403G,C23604A,C23709T,T24506G,G24914C,T25789A,C26201T,C27972T,G28048T,G28075T,A28095T,A28111G,G28280C,A28281T,T28282A,G28881A,G28882A,G28883C,C28977T	27	N:D3L,N:R203K,N:G204R,N:S235F,ORF1a:G345D,ORF1a:T708I,ORF1a:T1001I,ORF1a:H1160Y,ORF1a:A1708D,ORF1a:I2230T,ORF1a:M2259I,ORF1b:P218L,ORF1b:P314L,ORF3a:C133S,ORF3a:T270I,ORF8:Q27*,ORF8:R52I,ORF8:C61F,ORF8:K68*,ORF8:Y73C,S:N501Y,S:A570D,S:D614G,S:P681H,S:T716I,S:S982A,S:D1	6	0
hCoV-19/Mexico/CMX-InDRE_FD64253_S2063/2021	EPI_ISL_2295589	-	B.1.1.7	G	35	C241T,C913T,C3037T,C3267T,C5388A,C5986T,G6647A,T6954C,C14408T,C14676T,T15096C,C15279T,T16176C,C17676T,C18183T,C19524T,A21519C,A23063T,C23271A,A23403G,C23604A,C23709T,A24070T,A24092G,T24506G,G24914C,C25624T,C27737T,C27972T,G28048T,A28111G,G28280C,A28281T,T28282A,C28977T	21	N:D3L,ORF1a:T1001I,ORF1a:A1708D,ORF1a:A2128T,ORF1a:I2230T,ORF1b:P314L,ORF1b:R2684S,ORF3a:H78Y,ORF7a:T115I,ORF8:Q27*,ORF8:R52I,ORF8:Y73C,S:N501Y,S:A570D,S:D614G,S:P681H,S:T716I,S:Q836H,S:I844V,S:S982A,S:D1118H	7	16
hCoV-19/Mexico/CMX-InDRE_FD64705_S2062/2021	EPI_ISL_2295588	-	B.1.1.7	G	32	C241T,C913T,C3037T,C3267T,C5388A,C5944T,C5986T,T6954C,C9967T,C14408T,C14676T,T15096C,C15279T,T16176C,A17344G,G18412T,G21204T,C22987T,A23063T,C23271A,A23403G,C23604A,C23709T,T24506G,G24914C,C27972T,G28048T,A28111G,G28280C,A28281T,T28282A,C28	18	N:D3L,ORF1a:T1001I,ORF1a:A1708D,ORF1a:I2230T,ORF1b:P314L,ORF1b:I1293V,ORF1b:V1649F,ORF1b:K2579N,ORF8:Q27*,ORF8:R52I,ORF8:Y73C,S:N501Y,S:A570D,S:D614G,S:P681H,S:T716I,S:S982A,S:D1118H	7	21
hCoV-19/Mexico/CMX-InDRE_FD69482_S2781/2021	EPI_ISL_2533807	-	B.1.1.7	GRY	40	C203T,C241T,C913T,G1820A,C3037T,C3267T,C5388A,G5924A,C5986T,T6954C,G9526T,A12162G,C14408T,C14676T,C15279T,C15720T,T16176C,A17615G,T19363C,A21137G,A23063T,C23248T,C23271A,A23403G,C23604A,C23709T,T24506G,G24914C,G25135T,A25336C,C27972T,G28048T,A28111G,G28280C,A28281T,T28282A,G28881A,G28882A,G28883C,C28977T	26	N:D3L,N:R203K,N:G204R,N:S235F,ORF1a:G519S,ORF1a:T1001I,ORF1a:A1708D,ORF1a:V1887I,ORF1a:I2230T,ORF1a:M3087I,ORF1a:Q3966R,ORF1b:P314L,ORF1b:K1383R,ORF1b:K2557R,ORF8:Q27*,ORF8:R52I,ORF8:Y73C,S:N501Y,S:A570D,S:D614G,S:P681H,S:T716I,S:S982A,S:D1118H,S:K1191N,S:E1258D	6	0
hCoV-19/Mexico/CMX-InDRE_FD69743_S3075/2021	EPI_ISL_2663342	-	B.1.1.7	GRY	36	T78G,C241T,C913T,C3037T,C3267T,C5388A,C5986T,C6196T,T6954C,A8848G,C11074T,C14408T,C14676T,C15279T,T16176C,A23063T,C23271A,A23403G,C23604A,C23709T,T24506G,G24914C,G25217T,A25336C,G26727T,C27972T,G28048T,A28111G,G28280C,A28281T,T28282A,G28881A,G28882A,G28883C,C28977T,G29715T	21	M:A69S,N:D3L,N:R203K,N:G204R,N:S235F,ORF1a:T1001I,ORF1a:A1708D,ORF1a:I2230T,ORF1b:P314L,ORF8:Q27*,ORF8:R52I,ORF8:Y73C,S:N501Y,S:A570D,S:D614G,S:P681H,S:T716I,S:S982A,S:D1118H,S:G1219C,S:E1258D	6	81
hCoV-19/Mexico/CMX-InDRE_FD71018_S2782/2021	EPI_ISL_2533808	-	B.1.1.7	GRY	41	G174T,G210T,C241T,C913T,C1420T,C2453T,C3037T,C3267T,C4475T,C5388A,C5986T,T6954C,C8956T,T9016C,C14408T,T14565C,C14676T,C15279T,T16176C,T17803G,A23063T,C23271A,A23403G,C23604A,C23709T,C24378T,T24506G,G24914C,C27883T,C27972T,G28048T,A28111G,G28280C,A28281T,T28282A,C28312T,G28881A,G28882A,G28883C,C28977T,C29073T	25	N:D3L,N:R203K,N:G204R,N:S235F,N:A267V,ORF1a:L730F,ORF1a:T1001I,ORF1a:R1404C,ORF1a:A1708D,ORF1a:I2230T,ORF1b:P314L,ORF1b:S1446A,ORF7b:A43V,ORF8:Q27*,ORF8:R52I,ORF8:Y73C,ORF9b:P10L,S:N501Y,S:A570D,S:D614G,S:P681H,S:T716I,S:S939F,S:S982A,S:D1118H	15	0
hCoV-19/Mexico/CMX-InDRE_FD71149_S2784/2021	EPI_ISL_2533810	-	B.1.1.7	GRY	32	C241T,C913T,C3037T,C3267T,C5388A,C5986T,C6196T,T6954C,A8848G,C14408T,C14676T,C15279T,T16176C,A23063T,C23114T,C23271A,A23403G,C23604A,C23709T,T24506G,G24914C,C27972T,G28048T,A28111G,G28280C,A28281T,T28282A,G28881A,G28882A,G28883C,C28977T,G	18	N:D3L,N:R203K,N:G204R,N:S235F,ORF1a:T1001I,ORF1a:A1708D,ORF1a:I2230T,ORF1b:P314L,ORF8:Q27*,ORF8:R52I,ORF8:Y73C,S:N501Y,S:A570D,S:D614G,S:P681H,S:T716I,S:S982A,S:D1118H	6	0
hCoV-19/Mexico/CMX-InDRE_FD71474_S2785/2021	EPI_ISL_2533811	-	B.1.1.7	GRY	35	C241T,C913T,C3037T,C3267T,C5388A,C5986T,C6196T,T6954C,A8848G,C14408T,C14676T,C15279T,T16176C,G19816T,A23063T,C23271A,A23403G,C23604A,C23709T,T24506G,G24914C,G25217T,G26727T,T27285C,C27972T,G28048T,A28111G,G28280C,A28281T,T28282A,G28881A,G28882A,G28883C,C28977T,G29715T	21	M:A69S,N:D3L,N:R203K,N:G204R,N:S235F,ORF1a:T1001I,ORF1a:A1708D,ORF1a:I2230T,ORF1b:P314L,ORF1b:V2117L,ORF8:Q27*,ORF8:R52I,ORF8:Y73C,S:N501Y,S:A570D,S:D614G,S:P681H,S:T716I,S:S982A,S:D1118H,S:G1219C	6	0
hCoV-19/Mexico/CMX-InDRE_FD71481_S3076/2021	EPI_ISL_2663343	-	B.1.1.7	GRY	34	T78G,C241T,C913T,C3037T,C3267T,C5388A,C5986T,T6954C,C14408T,C14676T,C15279T,T16176C,A23063T,C23271A,A23403G,C23604A,C23709T,T24506G,G24914C,G25217T,A25336C,G26727T,C27972T,G28048T,A28111G,G28280C,A28281T,T28282A,G28881A,G28882A,G28883C,C28	21	M:A69S,N:D3L,N:R203K,N:G204R,N:S235F,ORF1a:T1001I,ORF1a:A1708D,ORF1a:I2230T,ORF1b:P314L,ORF8:Q27*,ORF8:R52I,ORF8:Y73C,S:N501Y,S:A570D,S:D614G,S:P681H,S:T716I,S:S982A,S:D1118H,S:G1219C,S:E1258D	6	770
hCoV-19/Mexico/CMX-InDRE_FD71675_S3077/2021	EPI_ISL_2663344	-	B.1.1.7	GRY	35	T78G,C913T,G1598A,C2453T,C3037T,C3267T,C5388A,C5986T,T6954C,C9611T,C14408T,C14676T,C15279T,T16176C,C21008T,C21855T,G21974C,A23063T,C23271A,A23403G,C23604A,C23709T,T24506G,G24914C,G28048T,A28111G,T28149A,G28280C,A28281T,T28282A,A28295G,G28881A,G28882A,G28883C,C28977T	25	N:D3L,N:N8D,N:R203K,N:G204R,N:S235F,ORF1a:G445S,ORF1a:L730F,ORF1a:T1001I,ORF1a:A1708D,ORF1a:I2230T,ORF1a:L3116F,ORF1b:P314L,ORF1b:T2514I,ORF8:R52I,ORF8:Y73C,ORF8:F86I,S:S98F,S:D138H,S:N501Y,S:A570D,S:D614G,S:P681H,S:T716I,S:S982A,S:D1118H	6	488
hCoV-19/Mexico/CMX-InDRE_FD72271_S3091/2021	EPI_ISL_2663358	-	B.1.1.7	GRY	36	C241T,C913T,C2110T,C3037T,C3267T,C5388A,C5986T,T6954C,G7042T,A10195G,C10279T,C14120T,C14408T,C14676T,C15279T,T16176C,C16887T,G17427T,A23063T,C23271A,A23403G,C23604A,C23709T,T24506G,G24914C,C27972T,G28048T,G28075T,A28111G,G28280C,A28281T,T28282A,G28881A,G28882A,G28883C,C28977T	21	N:D3L,N:R203K,N:G204R,N:S235F,ORF1a:T1001I,ORF1a:A1708D,ORF1a:I2230T,ORF1a:M2259I,ORF1b:P218L,ORF1b:P314L,ORF8:Q27*,ORF8:R52I,ORF8:C61F,ORF8:Y73C,S:N501Y,S:A570D,S:D614G,S:P681H,S:T716I,S:S982A,S:D1118H	6	52
hCoV-19/Mexico/CMX-InDRE_FD72328_S2786/2021	EPI_ISL_2533812	-	B.1.1.7	GRY	41	C241T,C913T,C3037T,C3267T,C5388A,C5986T,G6647A,T6954C,C14408T,C14621T,C14676T,T15096C,C15279T,T16176C,C17676T,C18183T,A18336G,C19524T,A21519C,A23063T,C23271A,A23403G,C23604A,C23709T,A24070T,A24092G,T24506G,G24914C,C25624T,C27737T,C27972T,G28048T,A28111G,G28280C,A28281T,T28282A,G28881A,G28882A,G28883C,G28884C,C28977T	25	N:D3L,N:R203K,N:G204P,N:S235F,ORF1a:T1001I,ORF1a:A1708D,ORF1a:A2128T,ORF1a:I2230T,ORF1b:P314L,ORF1b:T385M,ORF1b:R2684S,ORF3a:H78Y,ORF7a:T115I,ORF8:Q27*,ORF8:R52I,ORF8:Y73C,S:N501Y,S:A570D,S:D614G,S:P681H,S:T716I,S:Q836H,S:I844V,S:S982A,S:D1118H	6	0
hCoV-19/Mexico/CMX-InDRE_FD72355_S2787/2021	EPI_ISL_2533813	-	B.1.1.7	GRY	36	C241T,C913T,C3037T,C3267T,C5388A,C5986T,C6196T,T6954C,A8848G,C14408T,C14676T,C15279T,T16176C,G19816T,A23063T,C23271A,A23403G,C23604A,C23709T,T24506G,G24914C,G25217T,A25336C,G26727T,T27285C,C27972T,G28048T,A28111G,G28280C,A28281T,T28282A,G28881A,G28882A,G28883C,C28977T,G29715T	22	M:A69S,N:D3L,N:R203K,N:G204R,N:S235F,ORF1a:T1001I,ORF1a:A1708D,ORF1a:I2230T,ORF1b:P314L,ORF1b:V2117L,ORF8:Q27*,ORF8:R52I,ORF8:Y73C,S:N501Y,S:A570D,S:D614G,S:P681H,S:T716I,S:S982A,S:D1118H,S:G1219C,S:E1258D	6	0
hCoV-19/Mexico/CMX-InDRE_FD72360_S2788/2021	EPI_ISL_2533814	-	B.1.1.7	GRY	36	C241T,C913T,C3037T,C3267T,C5388A,C5986T,C6196T,T6954C,A8848G,C14408T,C14676T,C15279T,T16176C,G19816T,A23063T,C23271A,A23403G,C23604A,C23709T,T24506G,G24914C,G25217T,A25336C,G26727T,T27285C,C27972T,G28048T,A28111G,G28280C,A28281T,T28282A,G28881A,G28882A,G28883C,C28977T,G29715T	22	M:A69S,N:D3L,N:R203K,N:G204R,N:S235F,ORF1a:T1001I,ORF1a:A1708D,ORF1a:I2230T,ORF1b:P314L,ORF1b:V2117L,ORF8:Q27*,ORF8:R52I,ORF8:Y73C,S:N501Y,S:A570D,S:D614G,S:P681H,S:T716I,S:S982A,S:D1118H,S:G1219C,S:E1258D	6	0
hCoV-19/Mexico/CMX-InDRE_FD72362_S2789/2021	EPI_ISL_2533815	-	B.1.1.7	GRY	39	C241T,G388T,C913T,T1066A,C3037T,C3267T,C4900T,C5388A,C5986T,T6954C,A12162G,C14408T,C14676T,C15279T,C15720T,T16176C,A17615G,A21137G,G21850T,A23063T,C23271A,A23403G,C23604A,C23709T,T24506G,G24914C,G25135T,C27739T,C27972T,G28048T,A28111G,G28280C,A28281T,T28282A,A28777G,G28881A,G28882A,G28883C,C28977T	26	N:D3L,N:R203K,N:G204R,N:S235F,ORF1a:E41D,ORF1a:N267K,ORF1a:T1001I,ORF1a:A1708D,ORF1a:I2230T,ORF1a:Q3966R,ORF1b:P314L,ORF1b:K1383R,ORF1b:K2557R,ORF7a:L116F,ORF8:Q27*,ORF8:R52I,ORF8:Y73C,S:E96D,S:N501Y,S:A570D,S:D614G,S:P681H,S:T716I,S:S982A,S:D1118H,S:K1191N	6	0

hCoV-19/Mexico/CMX-InDRE_FD77651_S4136/2021	EPI_ISL_2920707	-	B.1.1.7	GRY	38	C241T,C913T,C3037T,C3267T,A3492G,C5388A,C5986T,T6954C,C14408T,C14676T,C14724T,C15279T,T16176C,C16616T,C17550T,C18555T,G18883A,C21639A,A23063T,C23271A,A23403G,C23604A,C23709T,C24374T,T24506G,G24914C,C27972T,G28048T,A28111G,G28280C,A28281T,T28282A,G28881A,G28882A,G28883C,C28977T,C29732T,G29751T	23	N:D3L,N:R203K,N:G204R,N:S235F,ORF1a:T1001I,ORF1a:N1076S,ORF1a:A1708D,ORF1a:I2230T,ORF1b:P314L,ORF1b:T1050I,ORF1b:V1806I,ORF8:Q27*,ORF8:R52I,ORF8:Y73C,S:P26H,S:N501Y,S:A570D,S:D614G,S:P681H,S:T716I,S:L938F,S:S982A,S:D1118H	6	0
hCoV-19/Mexico/CMX-InDRE_FD77860_S4137/2021	EPI_ISL_2920708	-	B.1.1.7	GRY	36	C241T,C913T,C3037T,C3267T,A3492G,C5388A,C5986T,T6954C,C13105T,C14408T,C14676T,C14724T,C15279T,T16176C,C18555T,C21639A,A23063T,C23271A,A23403G,C23604A,C23709T,C24374T,T24506G,G24914C,C27972T,G28048T,A28111G,G28280C,A28281T,T28282A,G28881A,G28882A,G28883C,C28977T,C29732T,G29751T	21	N:D3L,N:R203K,N:G204R,N:S235F,ORF1a:T1001I,ORF1a:N1076S,ORF1a:A1708D,ORF1a:I2230T,ORF1b:P314L,ORF8:Q27*,ORF8:R52I,ORF8:Y73C,S:P26H,S:N501Y,S:A570D,S:D614G,S:P681H,S:T716I,S:L938F,S:S982A,S:D1118H	6	0
hCoV-19/Mexico/CMX-InDRE_FD77861_S4138/2021	EPI_ISL_2920709	-	B.1.1.7	GRY	36	C241T,C913T,C3037T,C3267T,A3492G,C5388A,C5986T,T6954C,C13105T,C14408T,C14676T,C14724T,C15279T,T16176C,C18555T,C21639A,A23063T,C23271A,A23403G,C23604A,C23709T,C24374T,T24506G,G24914C,C27972T,G28048T,A28111G,G28280C,A28281T,T28282A,G28881A,G28882A,G28883C,C28977T,C29732T,G29751T	21	N:D3L,N:R203K,N:G204R,N:S235F,ORF1a:T1001I,ORF1a:N1076S,ORF1a:A1708D,ORF1a:I2230T,ORF1b:P314L,ORF8:Q27*,ORF8:R52I,ORF8:Y73C,S:P26H,S:N501Y,S:A570D,S:D614G,S:P681H,S:T716I,S:L938F,S:S982A,S:D1118H	6	0
hCoV-19/Mexico/CMX-InDRE_FD77862_S4139/2021	EPI_ISL_2920710	-	B.1.1.7	GRY	36	C241T,C913T,C3037T,C3267T,A3492G,C5388A,C5986T,T6954C,C13105T,C14408T,C14676T,C14724T,C15279T,T16176C,C18555T,C21639A,A23063T,C23271A,A23403G,C23604A,C23709T,C24374T,T24506G,G24914C,C27972T,G28048T,A28111G,G28280C,A28281T,T28282A,G28881A,G28882A,G28883C,C28977T,C29732T,G29751T	21	N:D3L,N:R203K,N:G204R,N:S235F,ORF1a:T1001I,ORF1a:N1076S,ORF1a:A1708D,ORF1a:I2230T,ORF1b:P314L,ORF8:Q27*,ORF8:R52I,ORF8:Y73C,S:P26H,S:N501Y,S:A570D,S:D614G,S:P681H,S:T716I,S:L938F,S:S982A,S:D1118H	6	0
hCoV-19/Mexico/CMX-InDRE_FD77863_S4140/2021	EPI_ISL_2920711	-	B.1.1.7	GRY	36	C241T,C913T,C3037T,C3267T,A3492G,C5388A,C5986T,T6954C,C13105T,C14408T,C14676T,C14724T,C15279T,T16176C,C18555T,C21639A,A23063T,C23271A,A23403G,C23604A,C23709T,C24374T,T24506G,G24914C,C27972T,G28048T,A28111G,G28280C,A28281T,T28282A,G28881A,G28882A,G28883C,C28977T,C29732T,G29751T	21	N:D3L,N:R203K,N:G204R,N:S235F,ORF1a:T1001I,ORF1a:N1076S,ORF1a:A1708D,ORF1a:I2230T,ORF1b:P314L,ORF8:Q27*,ORF8:R52I,ORF8:Y73C,S:P26H,S:N501Y,S:A570D,S:D614G,S:P681H,S:T716I,S:L938F,S:S982A,S:D1118H	6	0
hCoV-19/Mexico/CMX-InDRE_FD77882_S4141/2021	EPI_ISL_2920712	-	B.1.1.7	GRY	37	C222T,C241T,C913T,C2110T,C3037T,C3267T,C5388A,C5986T,T6954C,G7042T,C14120T,C14408T,C14676T,C15279T,T16176C,C17762T,A23063T,C23271A,A23403G,C23604A,C23709T,T24506G,G24914C,T25180C,G25641T,C25672T,C27972T,G28048T,A28095T,A28111G,G28280C,A28281T,T28282A,G28881A,G28882A,G28883C,C28977T	24	N:D3L,N:R203K,N:G204R,N:S235F,ORF1a:T1001I,ORF1a:A1708D,ORF1a:I2230T,ORF1a:M2259I,ORF1b:P218L,ORF1b:P314L,ORF1b:A1432V,ORF3a:L83F,ORF3a:L94F,ORF8:Q27*,ORF8:R52I,ORF8:K68*,ORF8:Y73C,S:N501Y,S:A570D,S:D614G,S:P681H,S:T716I,S:S982A,S:D1118H	6	0
hCoV-19/Mexico/CMX-INER-INMEGEN-00136/2021	EPI_ISL_1824482	-	B.1.1.7	GRY	31	C241T,C913T,C3037T,C3267T,C5388A,C5986T,T6954C,C14408T,C14649T,C14676T,C15279T,T16176C,C18348T,A23063T,C23114T,C23271A,A23403G,C23604A,C23709T,T24506G,G24914C,C27972T,G28048T,A28111G,G28280C,A28281T,T28282A,G28881A,G28882A,G28883C,C28977T	18	N:D3L,N:R203K,N:G204R,N:S235F,ORF1a:T1001I,ORF1a:A1708D,ORF1a:I2230T,ORF1b:P314L,ORF8:Q27*,ORF8:R52I,ORF8:Y73C,S:N501Y,S:A570D,S:D614G,S:P681H,S:T716I,S:S982A,S:D1118H	6	0
hCoV-19/Mexico/CMX-INER-INMEGEN-00149/2021	EPI_ISL_1824495	-	B.1.1.7	GRY	41	C241T,C913T,C3037T,C3267T,C5388A,C5944T,C5986T,G6647A,T6954C,G8021T,C14408T,C14676T,T15096C,C15279T,T16176C,C17676T,C18183T,C19524T,A21519C,A23063T,C23271A,A23403G,C23604A,C23709T,A24070T,T24506G,G24914C,C25624T,C27972T,G28048T,A28095T,A28111G,G28280C,A28281T,T28282A,G28881A,G28882A,G28883C,G28884C,A28949C,C28977T	24	N:D3L,N:R203K,N:G204P,N:S235F,ORF1a:T1001I,ORF1a:A1708D,ORF1a:A2128T,ORF1a:I2230T,ORF1a:V2586F,ORF1b:P314L,ORF1b:R2684S,ORF3a:H78Y,ORF8:Q27*,ORF8:R52I,ORF8:K68*,ORF8:Y73C,S:N501Y,S:A570D,S:D614G,S:P681H,S:T716I,S:Q836H,S:S982A,S:D1118H	6	0
hCoV-19/Mexico/CMX-INER-INMEGEN-00159/2021	EPI_ISL_2160651	-	B.1.1.7	GRY	34	C241T,C913T,C2453T,C3037T,C3267T,C5388A,C5986T,T6954C,C9611T,C14408T,C14676T,C15279T,T16176C,C21855T,G21974C,A23063T,C23271A,A23403G,A23503G,C23604A,C23709T,T24506G,G24914C,C27972T,G28048T,A28111G,G28280C,A28281T,T28282A,A28295G,G28881A,G	23	N:D3L,N:N8D,N:R203K,N:G204R,N:S235F,ORF1a:L730F,ORF1a:T1001I,ORF1a:A1708D,ORF1a:I2230T,ORF1a:L3116F,ORF1b:P314L,ORF8:Q27*,ORF8:R52I,ORF8:Y73C,S:S98F,S:D138H,S:N501Y,S:A570D,S:D614G,S:P681H,S:T716I,S:S982	6	0
hCoV-19/Mexico/CMX-INER-INMEGEN-00164/2021	EPI_ISL_2160659	-	B.1.1.7	GRY	34	C241T,C913T,C2453T,C3037T,C3267T,C5388A,C5986T,T6954C,C9611T,C14408T,C14676T,C15279T,T16176C,C21855T,G21974C,A23063T,C23271A,A23403G,A23503G,C23604A,C23709T,T24506G,G24914C,C27972T,G28048T,A28111G,G28280C,A28281T,T28282A,A28295G,G28881A,G	23	N:D3L,N:N8D,N:R203K,N:G204R,N:S235F,ORF1a:L730F,ORF1a:T1001I,ORF1a:A1708D,ORF1a:I2230T,ORF1a:L3116F,ORF1b:P314L,ORF8:Q27*,ORF8:R52I,ORF8:Y73C,S:S98F,S:D138H,S:N501Y,S:A570D,S:D614G,S:P681H,S:T716I,S:S982	6	0
hCoV-19/Mexico/CMX-INER-INMEGEN-00167/2021	EPI_ISL_2160665	-	B.1.1.7	GRY	35	C222T,C241T,C913T,C2110T,C3037T,C3267T,C5388A,C5986T,T6954C,G7042T,C14120T,C14408T,C14676T,C15279T,T16176C,C17762T,A23063T,C23271A,A23403G,C23604A,C23709T,T24506G,G24914C,T25180C,C27972T,G28048T,A28095T,A28111G,G28280C,A28281T,T28282A,G28881A,G28882A,G28883C,C28977T	22	N:D3L,N:R203K,N:G204R,N:S235F,ORF1a:T1001I,ORF1a:A1708D,ORF1a:I2230T,ORF1a:M2259I,ORF1b:P218L,ORF1b:P314L,ORF1b:A1432V,ORF8:Q27*,ORF8:R52I,ORF8:K68*,ORF8:Y73C,S:N501Y,S:A570D,S:D614G,S:P681H,S:T716I,S:S982A,S:D1118H	6	0
hCoV-19/Mexico/CMX-INER-INMEGEN-00203/2021	EPI_ISL_2341315	-	B.1.1.7	GRY	41	C241T,C913T,C3037T,C3267T,C5388A,C5944T,C5986T,G6647A,T6954C,G8021T,C14408T,C14676T,T15096C,C15279T,T16176C,C17676T,C18183T,C19524T,A21519C,A23063T,C23271A,A23403G,C23604A,C23709T,A24070T,T24506G,G24914C,C25624T,C27972T,G28048T,A28095T,A28111G,G28280C,A28281T,T28282A,G28881A,G28882A,G28883C,G28884C,A28949C,C28977T	24	N:D3L,N:R203K,N:G204P,N:S235F,ORF1a:T1001I,ORF1a:A1708D,ORF1a:A2128T,ORF1a:I2230T,ORF1a:V2586F,ORF1b:P314L,ORF1b:R2684S,ORF3a:H78Y,ORF8:Q27*,ORF8:R52I,ORF8:K68*,ORF8:Y73C,S:N501Y,S:A570D,S:D614G,S:P681H,S:T716I,S:Q836H,S:S982A,S:D1118H	6	0
hCoV-19/Mexico/CMX-INER-INMEGEN-00227/2021	EPI_ISL_2646128	-	B.1.1.7	GRY	36	C241T,C913T,C2453T,C3037T,C3267T,C5388A,C5986T,T6954C,C9611T,C9893T,C14408T,C14676T,C15279T,T16176C,C19017T,C21855T,G21974C,A23063T,C23271A,A23403G,C23533T,C23604A,C23709T,T24506G,G24914C,C27972T,G28048T,A28111G,G28280C,A28281T,T28282A,A28295G,G28881A,G28882A,G28883C,C28977T	24	N:D3L,N:N8D,N:R203K,N:G204R,N:S235F,ORF1a:L730F,ORF1a:T1001I,ORF1a:A1708D,ORF1a:I2230T,ORF1a:L3116F,ORF1a:L3210F,ORF1b:P314L,ORF8:Q27*,ORF8:R52I,ORF8:Y73C,S:S98F,S:D138H,S:N501Y,S:A570D,S:D614G,S:P681H,S:T716I,S:S982A,S:D1118H	6	0
hCoV-19/Mexico/CMX-INMEGEN-04-01-327/2021	EPI_ISL_1585917	-	B.1.1.7	GRY	31	C241T,C913T,C3037T,C3267T,C5388A,C5986T,T6954C,C14408T,C14676T,C15279T,T16176C,C21622T,A23063T,C23271A,A23403G,C23604A,C23709T,G24399T,T24506G,G24914C,G27676C,C27972T,G28048T,A28111G,G28280C,A28281T,T28282A,G28881A,G28882A,G28883C,C28977T	20	N:D3L,N:R203K,N:G204R,N:S235F,ORF1a:T1001I,ORF1a:A1708D,ORF1a:I2230T,ORF1b:P314L,ORF7a:E95Q,ORF8:Q27*,ORF8:R52I,ORF8:Y73C,S:N501Y,S:A570D,S:D614G,S:P681H,S:T716I,S:G946V,S:S982A,S:D1118H	6	1329
hCoV-19/Mexico/CMX-INMEGEN-04-04-55/2021	EPI_ISL_1626614	-	B.1.1.7	GRY	31	C241T,C913T,C3037T,C3267T,C5388A,C5986T,T6954C,C14408T,C14649T,C14676T,C15279T,T16176C,C18348T,A23063T,C23114T,C23271A,A23403G,C23604A,C23709T,T24506G,G24914C,C27972T,G28048T,A28111G,G28280C,A28281T,T28282A,G28881A,G28882A,G28883C,C28977T	18	N:D3L,N:R203K,N:G204R,N:S235F,ORF1a:T1001I,ORF1a:A1708D,ORF1a:I2230T,ORF1b:P314L,ORF8:Q27*,ORF8:R52I,ORF8:Y73C,S:N501Y,S:A570D,S:D614G,S:P681H,S:T716I,S:S982A,S:D1118H	6	6
hCoV-19/Mexico/CMX-INMEGEN-04-05-47/2021	EPI_ISL_1711297	-	B.1.1.7	GR	36	C241T,C913T,T1822A,C3037T,C3267T,C5388A,C5986T,T6043C,T6954C,A8533G,G11083T,G11417A,C11750T,C14408T,C14676T,C15279T,T16176C,G21255T,C23271A,A23403G,C23604A,C23709T,T24506G,G24914C,T25533C,C27972T,G28048T,A28095T,A28111G,G28280C,A28281T,T28282A,G28881A,G28882A,G28883C,C28977T	21	N:D3L,N:R203K,N:G204R,N:S235F,ORF1a:T1001I,ORF1a:A1708D,ORF1a:I2230T,ORF1a:L3606F,ORF1a:V3718I,ORF1a:L3829F,ORF1b:P314L,ORF8:Q27*,ORF8:R52I,ORF8:K68*,ORF8:Y73C,S:A570D,S:D614G,S:P681H,S:T716I,S:S982A,S:D1118H	6	312
hCoV-19/Mexico/CMX-INMEGEN-04-05-49/2021	EPI_ISL_1711301	-	B.1.1.7	GRY	35	C241T,C913T,C2143T,C3037T,C3267T,C5388A,C5986T,T6954C,C14408T,C14676T,T15096C,C15279T,T16176C,A23063T,C23271A,A23403G,C23604A,C23709T,G24089A,T24506G,G24914C,G25785T,C27476T,C27972T,G28048T,A28095T,A28111G,G28280C,A28281T,T28282A,G28881A,G28882A,G28883C,C28977T,C29642T	22	N:D3L,N:R203K,N:G204R,N:S235F,ORF1a:T1001I,ORF1a:A1708D,ORF1a:I2230T,ORF1b:P314L,ORF3a:W131C,ORF7a:T28I,ORF8:Q27*,ORF8:R52I,ORF8:K68*,ORF8:Y73C,S:N501Y,S:A570D,S:D614G,S:P681H,S:T716I,S:D843N,S:S982A,S:D1118H	6	10
hCoV-19/Mexico/CMX-INMEGEN-04-05-6/2021	EPI_ISL_1711300	-	B.1.1.7	GRY	35	C222T,C241T,C913T,C2110T,C3037T,C3267T,C5388A,C5986T,T6954C,G7042T,C14120T,C14408T,C14676T,C15279T,T16176C,C17762T,A23063T,C23271A,A23403G,C23604A,C23709T,T24506G,G24914C,T25180C,C27972T,G28048T,A28095T,A28111G,G28280C,A28281T,T28282A,G28881A,G28882A,G28883C,C28977T	22	N:D3L,N:R203K,N:G204R,N:S235F,ORF1a:T1001I,ORF1a:A1708D,ORF1a:I2230T,ORF1a:M2259I,ORF1b:P218L,ORF1b:P314L,ORF1b:A1432V,ORF8:Q27*,ORF8:R52I,ORF8:K68*,ORF8:Y73C,S:N501Y,S:A570D,S:D614G,S:P681H,S:T716I,S:S982A,S:D1118H	6	20
hCoV-19/Mexico/CMX-INMEGEN-04-05-63/2021	EPI_ISL_1711298	-	B.1.1.7	GR	36	G174T,G210T,C241T,C913T,C1420T,C2453T,C3037T,C3267T,C5388A,C5986T,T6954C,T9016C,C9661T,C14408T,T14565C,C14676T,C15279T,T16176C,T17803G,C23271A,A23403G,C23604A,C23709T,T24506G,G24914C,C27883T,C27972T,G28048T,A28111G,G28280C,A28281T,T28282A,G28881A,G28882A,G28883C,C28977T	20	N:D3L,N:R203K,N:G204R,N:S235F,ORF1a:L730F,ORF1a:T1001I,ORF1a:A1708D,ORF1a:I2230T,ORF1b:P314L,ORF1b:S1446A,ORF7b:A43V,ORF8:Q27*,ORF8:R52I,ORF8:Y73C,S:A570D,S:D614G,S:P681H,S:T716I,S:S982A,S:D1118H	6	122
hCoV-19/Mexico/CMX-INMEGEN-04-05-71/2021	EPI_ISL_1711302	-	B.1.1.7	GRY	36	C241T,C913T,C2453T,C3037T,C3267T,C5388A,C5986T,C6471T,T6954C,C9611T,C14408T,C14676T,C15279T,T16176C,C21855T,G21974C,A23063T,C23271A,A23403G,A23503G,C23604A,C23709T,T24506G,G24914C,C27577T,C27972T,G28048T,A28111G,G28280C,A28281T,T28282A,A28295G,G28881A,G28882A,G28883C,C28977T	25	N:D3L,N:N8D,N:R203K,N:G204R,N:S235F,ORF1a:L730F,ORF1a:T1001I,ORF1a:A1708D,ORF1a:T2069I,ORF1a:I2230T,ORF1a:L3116F,ORF1b:P314L,ORF7a:Q62*,ORF8:Q27*,ORF8:R52I,ORF8:Y73C,S:S98F,S:D138H,S:N501Y,S:A570D,S:D614G,S:P681H,S:T716I,S:S982A,S:D1118H	6	0

hCoV-19/Mexico/CMX-INMEGEN-04-05-72/2021	EPI_ISL_1711299	-	B.1.1.7	GRY	35	C241T,C913T,C2453T,C3037T,C3267T,C5388A,C5986T,T6954C,C9611T,C14408T,C14676T,C15279T,T16176C,C21855T,G21974C,A23063T,C23271A,A23403G,A23503G,C23604A,C23709T,T24506G,G24914C,G25440T,C27972T,G28048T,A28111G,G28280C,A28281T,T28282A,A28295G,G28881A,G28882A,G28883C,C28977T	24	N:D3L,N:N8D,N:R203K,N:G204R,N:S235F,ORF1a:L730F,ORF1a:T1001I,ORF1a:A1708D,ORF1a:I2230T,ORF1a:L3116F,ORF1b:P314L,ORF3a:K16N,ORF8:Q27*,ORF8:R52I,ORF8:Y73C,S:S98F,S:D138H,S:N501Y,S:A570D,S:D614G,S:P681H,S:T716I,S:S982A,S:D1118H	6	0
hCoV-19/Mexico/CMX-INMEGEN-04-06-38/2021	EPI_ISL_1807344	-	B.1.1.7	GR	41	C241T,C913T,C3037T,C3267T,C5388A,C5944T,C5986T,G6647A,T6954C,G8021T,C14408T,C14676T,T15096C,C15279T,T16176C,C17676T,C18183T,C19524T,G21255T,A21519C,C23271A,A23403G,C23604A,C23709T,A24070T,T24506G,G24914C,C25624T,C27972T,G28048T,A28095T,A28111G,G28280C,A28281T,T28282A,G28881A,G28882A,G28883C,G28884C,A28949C,C28977T	23	N:D3L,N:R203K,N:G204P,N:S235F,ORF1a:T1001I,ORF1a:A1708D,ORF1a:A2128T,ORF1a:I2230T,ORF1a:V2586F,ORF1b:P314L,ORF1b:R2684S,ORF3a:H78Y,ORF8:Q27*,ORF8:R52I,ORF8:K68*,ORF8:Y73C,S:A570D,S:D614G,S:P681H,S:T716I,S:Q836H,S:S982A,S:D1118H	6	774
hCoV-19/Mexico/CMX-INMEGEN-04-06-73/2021	EPI_ISL_1807363	-	B.1.1.7	GRY	35	C241T,C913T,C2453T,C3037T,C3267T,C5388A,C5986T,T6954C,C9611T,C14408T,C14676T,C15279T,T16176C,C21855T,G21974C,A23063T,C23271A,A23403G,A23503G,C23604A,C23709T,T24506G,G24914C,G25440T,C27972T,G28048T,A28111G,G28280C,A28281T,T28282A,A28295G,G28881A,G28882A,G28883C,C28977T	24	N:D3L,N:N8D,N:R203K,N:G204R,N:S235F,ORF1a:L730F,ORF1a:T1001I,ORF1a:A1708D,ORF1a:I2230T,ORF1a:L3116F,ORF1b:P314L,ORF3a:K16N,ORF8:Q27*,ORF8:R52I,ORF8:Y73C,S:S98F,S:D138H,S:N501Y,S:A570D,S:D614G,S:P681H,S:T716I,S:S982A,S:D1118H	6	29
hCoV-19/Mexico/CMX-INMEGEN-04-07-195/2021	EPI_ISL_2080627	-	B.1.1.7	G	26	C829T,C913T,C3037T,C3267T,C5184A,C5388A,C5986T,T6954C,G7761T,C14408T,C14676T,C15279T,T16176C,C23271A,A23403G,C23604A,C23709T,T24506G,G24914C,G27281A,C27972T,G28048T,A28111G,G28280C,A28281T,T28282A	17	N:D3L,ORF1a:T1001I,ORF1a:P1640H,ORF1a:A1708D,ORF1a:I2230T,ORF1a:G2499V,ORF1b:P314L,ORF6:W27*,ORF8:Q27*,ORF8:R52I,ORF8:Y73C,S:A570D,S:D614G,S:P681H,S:T716I,S:S982A,S:D1118H	6	6705
hCoV-19/Mexico/CMX-INMEGEN-04-07-230/2021	EPI_ISL_2080647	-	B.1.1.7	GR	33	C241T,C913T,C2453T,C3037T,C3267T,C5388A,C5986T,T6954C,C9611T,C14408T,C14676T,C15279T,T16176C,C21855T,G21974C,C23271A,A23403G,C23604A,C23709T,T24506G,G24914C,G25440T,C27972T,G28048T,A28111G,G28280C,A28281T,T28282A,A28295G,G28881A,G28882A,G28883C,C28977T	23	N:D3L,N:N8D,N:R203K,N:G204R,N:S235F,ORF1a:L730F,ORF1a:T1001I,ORF1a:A1708D,ORF1a:I2230T,ORF1a:L3116F,ORF1b:P314L,ORF3a:K16N,ORF8:Q27*,ORF8:R52I,ORF8:Y73C,S:S98F,S:D138H,S:A570D,S:D614G,S:P681H,S:T716I,S:S982A,S:D1118H	6	397
hCoV-19/Mexico/CMX-INMEGEN-04-07-243/2021	EPI_ISL_2080658	-	B.1.1.7	GR	34	C913T,C2788T,C3037T,C3267T,C5388A,C5986T,C6196T,T6954C,C14408T,C14676T,C15279T,T16176C,C23271A,A23403G,C23604A,C23709T,T24506G,G24914C,G25217T,A25601T,G26727T,C27923T,C27972T,G28048T,A28111G,G28280C,A28281T,T28282A,G28436T,G28881A,G28882A,G28883C,C28977T,G29715T	21	M:A69S,N:D3L,N:A55S,N:R203K,N:G204R,N:S235F,ORF1a:T1001I,ORF1a:A1708D,ORF1a:I2230T,ORF1b:P314L,ORF3a:Q70L,ORF8:Q27*,ORF8:R52I,ORF8:Y73C,S:A570D,S:D614G,S:P681H,S:T716I,S:S982A,S:D1118H,S:G1219C	6	2913
hCoV-19/Mexico/CMX-INMEGEN-04-07-292/2021	EPI_ISL_2080689	-	B.1.1.7	GR	35	C241T,C913T,C3037T,C3267T,C4900T,C5388A,C5986T,T6954C,A12162G,C13994T,C14408T,C14676T,C15279T,C15720T,T16176C,T17499C,A17615G,A21137G,C23271A,A23403G,C23604A,C23709T,T24506G,G24914C,G25135T,C27972T,G28048T,A28111G,G28280C,A28281T,T28282A,G28881A,G28882A,G28883C,C28977T	22	N:D3L,N:R203K,N:G204R,N:S235F,ORF1a:T1001I,ORF1a:A1708D,ORF1a:I2230T,ORF1a:Q3966R,ORF1b:A176V,ORF1b:P314L,ORF1b:K1383R,ORF1b:K2557R,ORF8:Q27*,ORF8:R52I,ORF8:Y73C,S:A570D,S:D614G,S:P681H,S:T716I,S:S982A,S:D1118H,S:K1191N	6	1731
hCoV-19/Mexico/CMX-INMEGEN-05-01-108/2021	EPI_ISL_2230864	-	B.1.1.7	GRY	33	C241T,C913T,C3037T,C3267T,C5388A,C5986T,C6196T,T6954C,A8848G,C14408T,C14676T,C15279T,T16176C,T19624C,C22987T,A23063T,C23271A,A23403G,C23604A,C23709T,T24506G,G24914C,C27972T,G28048T,A28111G,G28280C,A28281T,T28282A,G28881A,G28882A,G28883C,C2	18	N:D3L,N:R203K,N:G204R,N:S235F,ORF1a:T1001I,ORF1a:A1708D,ORF1a:I2230T,ORF1b:P314L,ORF8:Q27*,ORF8:R52I,ORF8:Y73C,S:N501Y,S:A570D,S:D614G,S:P681H,S:T716I,S:S982A,S:D1118H	6	0
hCoV-19/Mexico/CMX-INMEGEN-05-01-177/2021	EPI_ISL_2230880	-	B.1.1.7	GRY	38	C241T,C913T,C3037T,C3267T,C5388A,C5944T,C5986T,T6954C,C9967T,C13585T,C14408T,C14676T,T15096C,C15279T,T16176C,A17344G,G18412T,C22987T,A23063T,C23271A,A23403G,C23604A,C23644A,C23709T,T24506G,G24914C,C27972T,G28048T,A28095T,A28111G,G28280C,A28281T,T28282A,G28881A,G28882A,G28883C,G28884C,C28977T	21	N:D3L,N:R203K,N:G204P,N:S235F,ORF1a:T1001I,ORF1a:A1708D,ORF1a:I2230T,ORF1b:P314L,ORF1b:I1293V,ORF1b:V1649F,ORF8:Q27*,ORF8:R52I,ORF8:K68*,ORF8:Y73C,S:N501Y,S:A570D,S:D614G,S:P681H,S:T716I,S:S982A,S:D1118H	6	0
hCoV-19/Mexico/CMX-INMEGEN-05-01-19/2021	EPI_ISL_2230820	-	B.1.1.7	GR	34	C913T,C2110T,G2945T,C3037T,C3267T,A3390G,C5388A,C5986T,T6954C,T7984C,C14120T,C14408T,C14676T,C15279T,T16176C,C19390T,C22033T,C23271A,A23403G,C23604A,C23709T,T24506G,G24914C,C27972T,G28048T,A28095T,A28111G,G28280C,A28281T,T28282A,G28881A,G28882A,G28883C,C28977T	22	N:D3L,N:R203K,N:G204R,N:S235F,ORF1a:G894C,ORF1a:T1001I,ORF1a:N1042S,ORF1a:A1708D,ORF1a:I2230T,ORF1b:P218L,ORF1b:P314L,ORF1b:P1975S,ORF8:Q27*,ORF8:R52I,ORF8:K68*,ORF8:Y73C,S:A570D,S:D614G,S:P681H,S:T716I,S:S982A,S:D1118H	6	818
hCoV-19/Mexico/CMX-INMEGEN-05-01-192/2021	EPI_ISL_2105723	-	B.1.1.7	GR	32	G174C,C241T,C913T,C3037T,C3267T,C5388A,C5986T,C6196T,C6726T,T6954C,C11750T,C14408T,C14676T,C15279T,T16176C,C23271A,A23403G,C23604A,C23709T,T24506G,G24914C,C27972T,G28048T,A28111G,G28280C,A28281T,T28282A,G28881A,G28882A,G28883C,C28977T,C295	20	N:D3L,N:R203K,N:G204R,N:S235F,N:T417I,ORF1a:T1001I,ORF1a:A1708D,ORF1a:T2154I,ORF1a:I2230T,ORF1a:L3829F,ORF1b:P314L,ORF8:Q27*,ORF8:R52I,ORF8:Y73C,S:A570D,S:D614G,S:P681H,S:T716I,S:S982A,S:D1118H	6	829
hCoV-19/Mexico/CMX-INMEGEN-05-01-221/2021	EPI_ISL_2105741	-	B.1.1.7	GRY	35	C241T,C913T,C2453T,C3037T,C3267T,C3784T,C5388A,C5986T,T6954C,A7479G,C9611T,C14408T,C14676T,C15279T,T16176C,C21855T,G21974C,A23063T,C23271A,A23403G,C23604A,C23709T,T24506G,G24914C,C27972T,G28048T,A28111G,G28280C,A28281T,T28282A,A28295G,G28881A,G28882A,G28883C,C28977T	24	N:D3L,N:N8D,N:R203K,N:G204R,N:S235F,ORF1a:L730F,ORF1a:T1001I,ORF1a:A1708D,ORF1a:I2230T,ORF1a:N2405S,ORF1a:L3116F,ORF1b:P314L,ORF8:Q27*,ORF8:R52I,ORF8:Y73C,S:S98F,S:D138H,S:N501Y,S:A570D,S:D614G,S:P681H,S:T716I,S:S982A,S:D1118H	6	0
hCoV-19/Mexico/CMX-INMEGEN-05-01-226/2021	EPI_ISL_2105746	-	B.1.1.7	GR	36	C241T,C913T,C3037T,C3267T,C4900T,C5388A,C5986T,T6954C,A12162G,C13994T,C14408T,C14676T,C15279T,C15371T,C15720T,T16176C,T17499C,A17615G,A21137G,C23271A,A23403G,C23604A,C23709T,T24506G,G24914C,G25135T,C27972T,G28048T,A28111G,G28280C,A28281T,T28282A,G28881A,G28882A,G28883C,C28977T	23	N:D3L,N:R203K,N:G204R,N:S235F,ORF1a:T1001I,ORF1a:A1708D,ORF1a:I2230T,ORF1a:Q3966R,ORF1b:A176V,ORF1b:P314L,ORF1b:T635M,ORF1b:K1383R,ORF1b:K2557R,ORF8:Q27*,ORF8:R52I,ORF8:Y73C,S:A570D,S:D614G,S:P681H,S:T716I,S:S982A,S:D1118H,S:K1191N	6	960
hCoV-19/Mexico/CMX-INMEGEN-05-01-228/2021	EPI_ISL_2105748	-	B.1.1.7	GRY	36	C241T,C913T,C3037T,C3267T,C5388A,C5944T,C5986T,T6954C,G11562T,G12223T,C14408T,C14676T,T15096C,C15279T,T16176C,A23063T,C23271A,A23403G,C23604A,C23709T,G24348C,T24506G,G24914C,C26642T,C27972T,G28048T,A28095T,A28111G,G28280C,A28281T,T28282A,G28881A,G28882A,G28883C,G28884C,C28977T	21	N:D3L,N:R203K,N:G204P,N:S235F,ORF1a:T1001I,ORF1a:A1708D,ORF1a:I2230T,ORF1a:C3766F,ORF1b:P314L,ORF8:Q27*,ORF8:R52I,ORF8:K68*,ORF8:Y73C,S:N501Y,S:A570D,S:D614G,S:P681H,S:T716I,S:S929T,S:S982A,S:D1118H	6	258
hCoV-19/Mexico/CMX-INMEGEN-05-01-229/2021	EPI_ISL_2105749	-	B.1.1.7	GRY	33	C241T,C913T,C3037T,C3267T,C5388A,C5986T,C6196T,T6954C,A8848G,C14408T,C14676T,C15279T,T16176C,A23063T,C23271A,A23403G,C23604A,C23709T,T24506G,G24914C,G25217T,G26727T,C27972T,G28048T,A28111G,G28280C,A28281T,T28282A,G28881A,G28882A,G28883C,C2	20	M:A69S,N:D3L,N:R203K,N:G204R,N:S235F,ORF1a:T1001I,ORF1a:A1708D,ORF1a:I2230T,ORF1b:P314L,ORF8:Q27*,ORF8:R52I,ORF8:Y73C,S:N501Y,S:A570D,S:D614G,S:P681H,S:T716I,S:S982A,S:D1118H,S:G1219C	6	0
hCoV-19/Mexico/CMX-INMEGEN-05-01-260/2021	EPI_ISL_2105773	-	B.1.1.7	GRY	37	G174T,G210T,C241T,C913T,C2453T,C3037T,C3267T,A4547G,C5388A,C5986T,T6954C,A8588G,T9016C,C14408T,C14676T,C15279T,T16176C,C18828T,A23063T,C23271A,A23403G,C23604A,C23709T,T24506G,G24914C,C25469T,C27883T,C27972T,G28048T,A28111G,G28280C,A28281T,T28282A,G28881A,G28882A,G28883C,C28977T	23	N:D3L,N:R203K,N:G204R,N:S235F,ORF1a:L730F,ORF1a:T1001I,ORF1a:K1428E,ORF1a:A1708D,ORF1a:I2230T,ORF1a:K2775E,ORF1b:P314L,ORF3a:S26L,ORF7b:A43V,ORF8:Q27*,ORF8:R52I,ORF8:Y73C,S:N501Y,S:A570D,S:D614G,S:P681H,S:T716I,S:S982A,S:D1118H	6	0
hCoV-19/Mexico/CMX-INMEGEN-05-01-262/2021	EPI_ISL_2105775	-	B.1.1.7	GRY	37	G174T,G210T,C241T,C913T,C2453T,C3037T,C3267T,C5388A,C5986T,T6954C,A8588G,T9016C,C14408T,C14676T,C15279T,T16176C,C18828T,G22104T,A23063T,C23271A,A23403G,C23604A,C23709T,T24506G,G24914C,C25469T,C27883T,C27972T,G28048T,A28111G,G28280C,A28281T,T28282A,G28881A,G28882A,G28883C,C28977T	23	N:D3L,N:R203K,N:G204R,N:S235F,ORF1a:L730F,ORF1a:T1001I,ORF1a:A1708D,ORF1a:I2230T,ORF1a:K2775E,ORF1b:P314L,ORF3a:S26L,ORF7b:A43V,ORF8:Q27*,ORF8:R52I,ORF8:Y73C,S:G181V,S:N501Y,S:A570D,S:D614G,S:P681H,S:T716I,S:S982A,S:D1118H	6	0
hCoV-19/Mexico/CMX-INMEGEN-05-01-272/2021	EPI_ISL_2105784	-	B.1.1.7	GR	39	C241T,C913T,C1513T,C3037T,C3267T,C3583T,C5388A,C5944T,C5986T,T6954C,G11138T,G11401T,C12469T,C14408T,C14676T,T15096C,C15279T,T16176C,C17491T,C19862T,C23271A,A23403G,C23604A,C23709T,T24506G,G24914C,C27972T,G28048T,A28095T,A28111G,G28280C,A28281T,T28282A,G28881A,G28882A,G28883C,G28884C,C28977T,C29546A	22	N:D3L,N:R203K,N:G204P,N:S235F,ORF1a:T1001I,ORF1a:A1708D,ORF1a:I2230T,ORF1a:A3625S,ORF1a:M3712I,ORF1b:P314L,ORF1b:P1342S,ORF1b:A2132V,ORF8:Q27*,ORF8:R52I,ORF8:K68*,ORF8:Y73C,S:A570D,S:D614G,S:P681H,S:T716I,S:S982A,S:D1118H	6	1628
hCoV-19/Mexico/CMX-INMEGEN-05-01-301/2021	EPI_ISL_2105810	-	B.1.1.7	GRY	34	C241T,C913T,C3037T,C3267T,C5388A,C5986T,C6196T,T6954C,A8848G,G11083T,C14408T,C14676T,C15279T,T16176C,C21638G,A23063T,C23271A,A23403G,C23604A,C23709T,T24506G,G24914C,C27641T,C27972T,G28048T,A28111G,G28280C,A28281T,T28282A,G28881A,G28882A,G2	21	N:D3L,N:R203K,N:G204R,N:S235F,ORF1a:T1001I,ORF1a:A1708D,ORF1a:I2230T,ORF1a:L3606F,ORF1b:P314L,ORF7a:S83L,ORF8:Q27*,ORF8:R52I,ORF8:Y73C,S:P26A,S:N501Y,S:A570D,S:D614G,S:P681H,S:T716I,S:S982A,S:D1118H	6	0
hCoV-19/Mexico/CMX-INMEGEN-05-01-316/2021	EPI_ISL_2105824	-	B.1.1.7	G	23	C913T,C3037T,C5388A,C14408T,C14676T,C15279T,T16176C,C23271A,A23403G,C23604A,C23709T,T24506G,G24914C,G25217T,G26727T,C27972T,G28048T,A28111G,G28280C,A28281T,T28282A,G29715T,G29825T	14	M:A69S,N:D3L,ORF1a:A1708D,ORF1b:P314L,ORF8:Q27*,ORF8:R52I,ORF8:Y73C,S:A570D,S:D614G,S:P681H,S:T716I,S:S982A,S:D1118H,S:G1219C	6	8375

hCoV-19/Mexico/CMX-INMEGEN-05-01-319/2021	EPI_ISL_2105826	-	B.1.1.7	GR	36	C241T,C913T,C2110T,C3037T,C3267T,C5388A,C5986T,T6954C,G7042T,A10195G,C10279T,C14120T,C14408T,C14676T,C15279T,T16176C,C21588T,C23271A,A23403G,C23604A,C23709T,T24506G,T24565C,G24914C,C27972T,G28048T,G28075T,A28095T,A28111G,G28280C,A28281T,T28282A,G28881A,G28882A,G28883C,C28977T	22	N:D3L,N:R203K,N:G204R,N:S235F,ORF1a:T1001I,ORF1a:A1708D,ORF1a:I2230T,ORF1a:M2259I,ORF1b:P218L,ORF1b:P314L,ORF8:Q27*,ORF8:R52I,ORF8:C61F,ORF8:K68*,ORF8:Y73C,S:P9L,S:A570D,S:D614G,S:P681H,S:T716I,S:S982A,S:D1118H	6	431
hCoV-19/Mexico/CMX-INMEGEN-05-01-42/2021	EPI_ISL_2230892	-	B.1.1.7	GR	32	C913T,C2453T,C3037T,C3267T,C5388A,C5986T,T6954C,C9611T,C14408T,C14676T,C15279T,T16176C,C21855T,G21974C,C23271A,A23403G,C23604A,C23709T,T24506G,G24914C,G25440T,C27972T,G28048T,A28111G,G28280C,A28281T,T28282A,A28295G,G28881A,G28882A,G28883C,C28977T	23	N:D3L,N:N8D,N:R203K,N:G204R,N:S235F,ORF1a:L730F,ORF1a:T1001I,ORF1a:A1708D,ORF1a:I2230T,ORF1a:L3116F,ORF1b:P314L,ORF3a:K16N,ORF8:Q27*,ORF8:R52I,ORF8:Y73C,S:S98F,S:D138H,S:A570D,S:D614G,S:P681H,S:T716I,S:S982A,S:D1118H	6	1629
hCoV-19/Mexico/CMX-INMEGEN-05-01-74/2021	EPI_ISL_2277643	-	B.1.1.7	G	30	C913T,C1612T,C2453T,C3037T,C3267T,C5388A,C5986T,C8964T,C9611T,C14408T,C14676T,C15279T,T16176C,C21855T,G21974C,C23271A,A23403G,C23604A,C23709T,G24914C,T27113C,G27115T,C27972T,G28048T,A28111G,G28280C,A28281T,T28282A,A28295G,C28977T	20	M:R198L,N:D3L,N:N8D,N:S235F,ORF1a:L730F,ORF1a:T1001I,ORF1a:A1708D,ORF1a:S2900L,ORF1a:L3116F,ORF1b:P314L,ORF8:Q27*,ORF8:R52I,ORF8:Y73C,S:S98F,S:D138H,S:A570D,S:D614G,S:P681H,S:T716I,S:D1118H	6	8795
hCoV-19/Mexico/CMX-INMEGEN-05-01-87/2021	EPI_ISL_2277644	-	B.1.1.7	G	29	C913T,G2945T,C3037T,C3267T,C5388A,C5986T,T6954C,T7984C,C8146T,C14120T,C14408T,C14676T,C15279T,T16176C,T16476G,C19390T,C22033T,C23271A,A23403G,C23604A,C23709T,T24506G,G24914C,C27972T,G28048T,A28111G,G28280C,A28281T,T28282A	18	N:D3L,ORF1a:G894C,ORF1a:T1001I,ORF1a:A1708D,ORF1a:I2230T,ORF1b:P218L,ORF1b:P314L,ORF1b:S1003R,ORF1b:P1975S,ORF8:Q27*,ORF8:R52I,ORF8:Y73C,S:A570D,S:D614G,S:P681H,S:T716I,S:S982A,S:D1118H	6	5914
hCoV-19/Mexico/CMX-INMEGEN-05-01-98/2021	EPI_ISL_2230863	-	B.1.1.7	GRY	32	C241T,C913T,C3037T,C3267T,C5388A,C5986T,C6196T,T6954C,A8848G,C14408T,C14676T,C15279T,T16176C,T19624C,A23063T,C23271A,A23403G,C23604A,C23709T,T24506G,G24914C,C27972T,G28048T,A28111G,G28280C,A28281T,T28282A,G28881A,G28882A,G28883C,C28977T,G	18	N:D3L,N:R203K,N:G204R,N:S235F,ORF1a:T1001I,ORF1a:A1708D,ORF1a:I2230T,ORF1b:P314L,ORF8:Q27*,ORF8:R52I,ORF8:Y73C,S:N501Y,S:A570D,S:D614G,S:P681H,S:T716I,S:S982A,S:D1118H	6	0
hCoV-19/Mexico/CMX-INMEGEN-05-02-101/2021	EPI_ISL_2230870	-	B.1.1.7	GRY	35	C241T,C913T,C2453T,C3037T,C3267T,C5388A,C5986T,T6954C,C9611T,C14408T,C14676T,C15279T,T16176C,C21855T,G21974C,A23063T,C23271A,A23403G,A23503G,C23604A,C23709T,T24506G,G24914C,G25440T,C27972T,G28048T,A28111G,G28280C,A28281T,T28282A,A28295G,G28881A,G28882A,G28883C,C28977T	24	N:D3L,N:N8D,N:R203K,N:G204R,N:S235F,ORF1a:L730F,ORF1a:T1001I,ORF1a:A1708D,ORF1a:I2230T,ORF1a:L3116F,ORF1b:P314L,ORF3a:K16N,ORF8:Q27*,ORF8:R52I,ORF8:Y73C,S:S98F,S:D138H,S:N501Y,S:A570D,S:D614G,S:P681H,S:T716I,S:S982A,S:D1118H	6	0
hCoV-19/Mexico/CMX-INMEGEN-05-02-107/2021	EPI_ISL_2881754	-	B.1.1.7	G	31	C823T,C913T,C2110T,G3004T,C3037T,C3267T,C5388A,C5986T,T6954C,G7042T,C14120T,C14408T,C14676T,C14928T,C15279T,T16176C,C23271A,A23403G,C23604A,C23709T,T24506G,G24914C,C26110T,C27972T,G28048T,A28095T,A28111G,G28280C,A28281T,T28282A,C28977T	20	N:D3L,N:S235F,ORF1a:E913D,ORF1a:T1001I,ORF1a:A1708D,ORF1a:I2230T,ORF1a:M2259I,ORF1b:P218L,ORF1b:P314L,ORF3a:P240S,ORF8:Q27*,ORF8:R52I,ORF8:K68*,ORF8:Y73C,S:A570D,S:D614G,S:P681H,S:T716I,S:S982A,S:D1118H	6	2158
hCoV-19/Mexico/CMX-INMEGEN-05-02-108/2021	EPI_ISL_2230830	-	B.1.1.7	GR	32	C913T,C3037T,C3267T,C5388A,C5986T,C6196T,T6954C,A8848G,C14408T,C14676T,C15279T,T16176C,C23271A,T23332C,A23403G,C23604A,C23709T,T24506G,G24914C,G25217T,G26727T,C27972T,G28048T,A28111G,G28280C,A28281T,T28282A,G28881A,G28882A,G28883C,C28977T,	19	M:A69S,N:D3L,N:R203K,N:G204R,N:S235F,ORF1a:T1001I,ORF1a:A1708D,ORF1a:I2230T,ORF1b:P314L,ORF8:Q27*,ORF8:R52I,ORF8:Y73C,S:A570D,S:D614G,S:P681H,S:T716I,S:S982A,S:D1118H,S:G1219C	6	609
hCoV-19/Mexico/CMX-INMEGEN-05-02-109/2021	EPI_ISL_2230872	-	B.1.1.7	GRY	34	C241T,C913T,G2890A,C3037T,C3267T,C5388A,C5986T,C6196T,T6954C,A8848G,C9073T,C14408T,C14676T,C15279T,T16176C,T19624C,A23063T,C23271A,A23403G,C23604A,C23709T,T24506G,G24914C,C27972T,G28048T,A28111G,G28280C,A28281T,T28282A,G28881A,G28882A,G288	18	N:D3L,N:R203K,N:G204R,N:S235F,ORF1a:T1001I,ORF1a:A1708D,ORF1a:I2230T,ORF1b:P314L,ORF8:Q27*,ORF8:R52I,ORF8:Y73C,S:N501Y,S:A570D,S:D614G,S:P681H,S:T716I,S:S982A,S:D1118H	6	0
hCoV-19/Mexico/CMX-INMEGEN-05-02-122/2021	EPI_ISL_2230874	-	B.1.1.7	GRY	34	C241T,C913T,C3037T,C3267T,C5388A,C5986T,C6196T,T6954C,A8848G,C14408T,C14676T,C15279T,T16176C,T19624C,A23063T,C23271A,A23403G,C23604A,C23709T,T24506G,G24914C,G25217T,C27972T,G28027T,G28048T,A28111G,G28280C,A28281T,T28282A,G28881A,G28882A,G2	20	N:D3L,N:R203K,N:G204R,N:S235F,ORF1a:T1001I,ORF1a:A1708D,ORF1a:I2230T,ORF1b:P314L,ORF8:Q27*,ORF8:W45L,ORF8:R52I,ORF8:Y73C,S:N501Y,S:A570D,S:D614G,S:P681H,S:T716I,S:S982A,S:D1118H,S:G1219C	6	28
hCoV-19/Mexico/CMX-INMEGEN-05-02-123/2021	EPI_ISL_2230875	-	B.1.1.7	GRY	38	G174T,G210T,C241T,C913T,C2453T,C3037T,C3267T,C5388A,C5986T,C6487T,T6954C,T9016C,G11083T,C11950T,C14408T,C14676T,C15279T,T16176C,C17676T,A23063T,C23271A,A23403G,C23604A,C23709T,T24506G,G24914C,C27883T,C27972T,G28048T,A28111G,G28280C,A28281T,T28282A,G28628T,G28881A,G28882A,G28883C,C28977T	21	N:D3L,N:A119S,N:R203K,N:G204R,N:S235F,ORF1a:L730F,ORF1a:T1001I,ORF1a:A1708D,ORF1a:I2230T,ORF1a:L3606F,ORF1b:P314L,ORF7b:A43V,ORF8:Q27*,ORF8:R52I,ORF8:Y73C,S:A570D,S:D614G,S:P681H,S:T716I,S:S982A,S:D1118H	6	239
hCoV-19/Mexico/CMX-INMEGEN-05-02-159/2021	EPI_ISL_2230882	-	B.1.1.7	G	37	C241T,C913T,C2110T,G2782T,C3037T,C3267T,C4456T,C5388A,C5986T,T6954C,A12850G,C14120T,C14408T,C14676T,C15279T,T16176C,C16733T,C19390T,G20578T,A23063T,C23271A,A23403G,C23604A,C23683T,C23709T,T24506G,C24904T,G24914C,C27972T,G28048T,A28095T,A28111G,G28280C,A28281T,T28282A,A28467G,C28977T	23	N:D3L,N:K65R,N:S235F,ORF1a:T1001I,ORF1a:A1708D,ORF1a:I2230T,ORF1b:P218L,ORF1b:P314L,ORF1b:S1089L,ORF1b:P1975S,ORF1b:V2371L,ORF8:Q27*,ORF8:R52I,ORF8:K68*,ORF8:Y73C,ORF9b:N62D,S:N501Y,S:A570D,S:D614G,S:P681H,S:T716I,S:S982A,S:D1118H	6	7
hCoV-19/Mexico/CMX-INMEGEN-05-02-172/2021	EPI_ISL_2230884	-	B.1.1.7	GRY	39	C241T,C913T,C2110T,G3004T,C3037T,C3267T,C5388A,C5986T,T6954C,G7042T,C10728T,A10729G,A10730T,C14120T,C14408T,C14676T,C15279T,T16176C,C19602T,A23063T,C23271A,A23403G,C23604A,C23709T,T24506G,G24914C,C27689T,C27972T,G28001T,G28048T,A28095T,A28111G,G28280C,A28281T,T28282A,G28881A,G28882A,G28883C,C28977T	25	N:D3L,N:R203K,N:G204R,N:S235F,ORF1a:E913D,ORF1a:T1001I,ORF1a:A1708D,ORF1a:I2230T,ORF1a:M2259I,ORF1a:T3488M,ORF1a:T3489S,ORF1b:P218L,ORF1b:P314L,ORF7a:P99L,ORF8:Q27*,ORF8:R52I,ORF8:K68*,ORF8:Y73C,S:N501Y,S:A570D,S:D614G,S:P681H,S:T716I,S:S982A,S:D1118H	6	266
hCoV-19/Mexico/CMX-INMEGEN-05-02-29/2021	EPI_ISL_2230868	-	B.1.1.7	GRY	33	C241T,C913T,C3037T,C3267T,C5388A,C5986T,C6196T,T6954C,A8848G,C14408T,C14676T,C15279T,T16176C,T19624C,A23063T,C23271A,A23403G,C23604A,C23709T,T24506G,G24914C,G25217T,C27972T,G28048T,A28111G,G28280C,A28281T,T28282A,G28881A,G28882A,G28883C,C2	19	N:D3L,N:R203K,N:G204R,N:S235F,ORF1a:T1001I,ORF1a:A1708D,ORF1a:I2230T,ORF1b:P314L,ORF8:Q27*,ORF8:R52I,ORF8:Y73C,S:N501Y,S:A570D,S:D614G,S:P681H,S:T716I,S:S982A,S:D1118H,S:G1219C	6	161
hCoV-19/Mexico/CMX-INMEGEN-05-02-35/2021	EPI_ISL_2230869	-	B.1.1.7	GRY	39	C241T,C913T,C2453T,C3037T,C3093T,C3267T,T4159C,C5388A,C5986T,T6954C,G8653T,C9611T,C9907T,C14408T,C14676T,C15279T,T16176C,C21855T,G21974C,A23063T,C23271A,A23403G,C23604A,C23709T,T24506G,G24914C,C25609T,G27758T,C27972T,G28048T,A28111G,G28280C,A28281T,T28282A,A28295G,G28881A,G28882A,G28883C,C28977T	28	N:D3L,N:N8D,N:R203K,N:G204R,N:S235F,ORF1a:L730F,ORF1a:P943L,ORF1a:T1001I,ORF1a:A1708D,ORF1a:I2230T,ORF1a:M2796I,ORF1a:L3116F,ORF1b:P314L,ORF3a:L73F,ORF7a:*122L,ORF7b:M1I,ORF8:Q27*,ORF8:R52I,ORF8:Y73C,S:S98F,S:D138H,S:N501Y,S:A570D,S:D614G,S:P681H,S:T716I,S:S982A,S:D1118	6	117
hCoV-19/Mexico/CMX-INMEGEN-05-03-104/2021	EPI_ISL_2319256	-	B.1.1.7	GRY	43	C241T,C913T,C3037T,C3267T,C5388A,C5944T,C5986T,G6647A,T6954C,C12003T,C14408T,C14676T,T15096C,C15279T,T16176C,C17502T,C17676T,C18183T,C19524T,A21519C,A23063T,C23271A,A23403G,C23604A,C23709T,A24070T,A24092G,T24506G,G24914C,C25624T,C27737T,C27972T,G28048T,A28095T,A28111G,G28280C,A28281T,T28282A,G28881A,G28882A,G28883C,G2	26	N:D3L,N:R203K,N:G204P,N:S235F,ORF1a:T1001I,ORF1a:A1708D,ORF1a:A2128T,ORF1a:I2230T,ORF1a:S3913L,ORF1b:P314L,ORF1b:R2684S,ORF3a:H78Y,ORF7a:T115I,ORF8:Q27*,ORF8:R52I,ORF8:K68*,ORF8:Y73C,S:N501Y,S:A570D,S:D614G,S:P681H,S:T716I,S:Q836H,S:I844V,S:S982A,S:D1118H	6	1
hCoV-19/Mexico/CMX-INMEGEN-05-03-3/2021	EPI_ISL_2319294	-	B.1.1.7	GRY	38	C241T,C913T,C3037T,C3267T,C5388A,C5944T,C5986T,T6954C,C9967T,C10868T,C13585T,C14408T,C14676T,T15096C,C15279T,T16176C,A17344G,G18412T,C22987T,A23063T,C23271A,A23403G,C23604A,C23709T,T24506G,G24914C,C27972T,G28048T,A28095T,A28111G,G28280C,A28281T,T28282A,G28881A,G28882A,G28883C,G28884C,C28977T	21	N:D3L,N:R203K,N:G204P,N:S235F,ORF1a:T1001I,ORF1a:A1708D,ORF1a:I2230T,ORF1b:P314L,ORF1b:I1293V,ORF1b:V1649F,ORF8:Q27*,ORF8:R52I,ORF8:K68*,ORF8:Y73C,S:N501Y,S:A570D,S:D614G,S:P681H,S:T716I,S:S982A,S:D1118H	6	32
hCoV-19/Mexico/CMX-INMEGEN-05-03-342/2021	EPI_ISL_2319403	-	B.1.1.7	GRY	38	C241T,G529T,C575A,C913T,C1519T,C3037T,C3267T,C5388A,C5986T,T6954C,C14408T,C14676T,C15279T,T16176C,C16468T,A17615G,C20235G,C21575T,A22011C,A23063T,C23271A,A23403G,C23604A,C23709T,T24506G,G24914C,C26735T,G27461T,C27972T,G28048T,A28111G,G28280C,A28281T,T28282A,G28881A,G28882A,G28883C,C28977T	24	N:D3L,N:R203K,N:G204R,N:S235F,ORF1a:L104I,ORF1a:T1001I,ORF1a:A1708D,ORF1a:I2230T,ORF1b:P314L,ORF1b:P1001S,ORF1b:K1383R,ORF7a:C23F,ORF8:Q27*,ORF8:R52I,ORF8:Y73C,S:L5F,S:K150T,S:N501Y,S:A570D,S:D614G,S:P681H,S:T716I,S:S982A,S:D1118H	6	673
hCoV-19/Mexico/CMX-INMEGEN-05-03-350/2021	EPI_ISL_2319405	-	B.1.1.7	G	30	G529T,C575A,C913T,C3037T,C3267T,T5260A,C5388A,C5986T,C14408T,C14676T,C15279T,T16176C,C16468T,A17615G,A22011C,C23271A,A23403G,C23604A,C23709T,T24506G,G24914C,C26735T,G27461T,C27972T,G28048T,A28111G,G28280C,A28281T,T28282A,C28977T	19	N:D3L,N:S235F,ORF1a:L104I,ORF1a:T1001I,ORF1a:A1708D,ORF1b:P314L,ORF1b:P1001S,ORF1b:K1383R,ORF7a:C23F,ORF8:Q27*,ORF8:R52I,ORF8:Y73C,S:K150T,S:A570D,S:D614G,S:P681H,S:T716I,S:S982A,S:D1118H	6	2294
hCoV-19/Mexico/CMX-INMEGEN-05-03-351/2021	EPI_ISL_2319406	-	B.1.1.7	GR	33	C913T,C2788T,C3037T,C3267T,T5260A,C5388A,C5986T,C6196T,A8848G,C11812A,C14408T,C14676T,C15279T,T16176C,C23271A,A23403G,C23604A,C23709T,T24506G,G24914C,G25217T,G26727T,C27972T,G28048T,A28111G,G28280C,A28281T,T28282A,G28881A,G28882A,G28883C,C	18	M:A69S,N:D3L,N:R203K,N:G204R,N:S235F,ORF1a:T1001I,ORF1a:A1708D,ORF1b:P314L,ORF8:Q27*,ORF8:R52I,ORF8:Y73C,S:A570D,S:D614G,S:P681H,S:T716I,S:S982A,S:D1118H,S:G1219C	6	1874
hCoV-19/Mexico/CMX-INMEGEN-05-03-51/2021	EPI_ISL_2319210	-	B.1.1.7	G	32	C913T,C3037T,C3267T,C5388A,C5986T,T6954C,T8569C,A13768T,C14408T,C14676T,T15096C,C15279T,T16176C,C19862T,C23271A,A23403G,C23604A,C23709T,T24506G,G24914C,G25534T,C26606T,G27281T,C27972T,G28048T,A28095T,A28111G,G28280C,A28281T,T28282A,C28977T,	20	N:D3L,N:S235F,ORF1a:T1001I,ORF1a:A1708D,ORF1a:I2230T,ORF1b:M101L,ORF1b:P314L,ORF1b:A2132V,ORF3a:V48F,ORF6:W27L,ORF8:Q27*,ORF8:R52I,ORF8:K68*,ORF8:Y73C,S:A570D,S:D614G,S:P681H,S:T716I,S:S982A,S:D1118H	6	1412
hCoV-19/Mexico/CMX-INMEGEN-05-03-53/2021	EPI_ISL_2319212	-	B.1.1.7	O	33	C913T,C3037T,C3267T,C5388A,C5986T,C6196T,T6954C,A8848G,C12114T,C14408T,C14676T,C15279T,T16176C,T19624C,C23271A,A23403G,C23604A,C23709T,G24173A,T24506G,G24914C,G25217T,C27972T,G28048T,A28111G,G28280C,A28281T,T28282A,G28881A,G28882A,G28883C,	20	N:D3L,N:R203K,N:G204R,N:S235F,ORF1a:T1001I,ORF1a:A1708D,ORF1a:I2230T,ORF1a:S3950F,ORF1b:P314L,ORF8:Q27*,ORF8:R52I,ORF8:Y73C,S:A570D,S:D614G,S:P681H,S:T716I,S:A871T,S:S982A,S:D1118H,S:G1219C	6	1465

hCoV-19/Mexico/CMX-INMEGEN-05-03-54/2021	EPI_ISL_2319213	-	B.1.1.7	GR	35	C913T,C2110T,C3037T,C3267T,C5388A,C5986T,T6954C,G7042T,A10195G,C10279T,C14120T,C14408T,C14676T,C15279T,T16176C,C16887T,G17427T,C23271A,A23403G,C23604A,C23709T,T24506G,G24914C,C27972T,G28048T,G28075T,A28095T,A28111G,G28280C,A28281T,T28282A,G28881A,G28882A,G28883C,C28977T	21	N:D3L,N:R203K,N:G204R,N:S235F,ORF1a:T1001I,ORF1a:A1708D,ORF1a:I2230T,ORF1a:M2259I,ORF1b:P218L,ORF1b:P314L,ORF8:Q27*,ORF8:R52I,ORF8:C61F,ORF8:K68*,ORF8:Y73C,S:A570D,S:D614G,S:P681H,S:T716I,S:S982A,S:D1118H	6	628
hCoV-19/Mexico/CMX-INMEGEN-05-03-61/2021	EPI_ISL_2881761	-	B.1.1.7	G	35	C913T,C3037T,C3267T,C5388A,C5986T,T6954C,C9521T,C10036T,C10116T,C14408T,C14676T,C15279T,T16176C,A17615G,A19079G,C19854T,C21008T,T21579C,C23271A,A23403G,C23604A,C23709T,T24506G,G24914C,C26305T,C26326T,T26972C,C27972T,G28048T,A28111G,T28224C,G28280C,A28281T,T28282A,C28977T	23	E:L21F,N:D3L,N:S235F,ORF1a:T1001I,ORF1a:A1708D,ORF1a:I2230T,ORF1a:L3086F,ORF1a:T3284I,ORF1b:P314L,ORF1b:K1383R,ORF1b:E1871G,ORF1b:T2514I,ORF8:Q27*,ORF8:R52I,ORF8:Y73C,ORF8:Y111H,S:V6A,S:A570D,S:D614G,S:P681H,S:T716I,S:S982A,S:D1118H	6	3321
hCoV-19/Mexico/CMX-INMEGEN-05-03-64/2021	EPI_ISL_2319221	-	B.1.1.7	GRY	33	C913T,C3037T,C3267T,C5388A,C5986T,C6196T,T6954C,A8848G,C9803T,T11135C,C14408T,C14676T,C15279T,T16176C,A23063T,C23271A,A23403G,C23604A,C23709T,G24095T,T24506G,G24914C,C27972T,G28048T,A28111G,G28280C,A28281T,T28282A,G28881A,G28882A,G28883C,	20	N:D3L,N:R203K,N:G204R,N:S235F,ORF1a:T1001I,ORF1a:A1708D,ORF1a:I2230T,ORF1a:F3624L,ORF1b:P314L,ORF8:Q27*,ORF8:R52I,ORF8:Y73C,S:N501Y,S:A570D,S:D614G,S:P681H,S:T716I,S:A845S,S:S982A,S:D1118H	6	347
hCoV-19/Mexico/CMX-INMEGEN-05-03-68/2021	EPI_ISL_2319225	-	B.1.1.7	GRY	34	C241T,C913T,C3037T,C3267T,C5388A,C5986T,C6255T,T6954C,G10157T,C14408T,C14676T,C15279T,T16176C,C18162T,G22093C,A23063T,C23271A,A23403G,G23420A,C23604A,C23709T,T24506G,G24914C,G26520T,C27972T,G28048T,A28111G,G28280C,A28281T,T28282A,G28881A,	22	N:D3L,N:R203K,N:G204R,N:S235F,ORF1a:T1001I,ORF1a:A1708D,ORF1a:A1997V,ORF1a:I2230T,ORF1a:V3298L,ORF1b:P314L,ORF8:Q27*,ORF8:R52I,ORF8:Y73C,S:M177I,S:N501Y,S:A570D,S:D614G,S:V620I,S:P681H,S:T716I,S:S982A,S:D	6	88
hCoV-19/Mexico/CMX-INMEGEN-05-03-8/2021	EPI_ISL_2319177	-	B.1.1.7	GRY	40	C241T,C913T,C2110T,C3037T,C3267T,C4093T,C5388A,C5986T,T6954C,G7042T,C11514T,T13521C,C14120T,C14408T,C14676T,C15279T,T16176C,C16293T,C17762T,C18877T,A23063T,C23271A,A23403G,C23604A,C23709T,T24506G,G24914C,G26020T,T26987C,C27972T,G28048T,A28095T,A28111G,G28280C,A28281T,T28282A,G28881A,G28882A,G28883C,C28977T	24	N:D3L,N:R203K,N:G204R,N:S235F,ORF1a:T1001I,ORF1a:A1708D,ORF1a:I2230T,ORF1a:M2259I,ORF1a:T3750I,ORF1b:P218L,ORF1b:P314L,ORF1b:A1432V,ORF3a:D210Y,ORF8:Q27*,ORF8:R52I,ORF8:K68*,ORF8:Y73C,S:N501Y,S:A570D,S:D614G,S:P681H,S:T716I,S:S982A,S:D1118H	6	0
hCoV-19/Mexico/CMX-INMEGEN-05-04-15/2021	EPI_ISL_2349893	-	B.1.1.7	GRY	36	C241T,C913T,C3037T,C3267T,C5388A,C5986T,C6196T,T6954C,A8848G,G10870T,G11083T,C14408T,C14676T,C15279T,T16176C,T19624C,A23063T,C23271A,A23403G,C23604A,C23709T,G24173A,T24506G,G24914C,G25217T,C27972T,G28048T,A28111G,G28280C,A28281T,T28282A,G28881A,G28882A,G28883C,C28977T,G29715T	21	N:D3L,N:R203K,N:G204R,N:S235F,ORF1a:T1001I,ORF1a:A1708D,ORF1a:I2230T,ORF1a:L3606F,ORF1b:P314L,ORF8:Q27*,ORF8:R52I,ORF8:Y73C,S:N501Y,S:A570D,S:D614G,S:P681H,S:T716I,S:A871T,S:S982A,S:D1118H,S:G1219C	6	54
hCoV-19/Mexico/CMX-INMEGEN-05-04-170/2021	EPI_ISL_2350004	-	B.1.1.7	GRY	34	C241T,C913T,C3037T,C3267T,C5388A,C5986T,T6954C,C7299T,C11750T,C14408T,C14676T,T15096C,C15279T,T16176C,A23063T,C23271A,A23403G,C23604A,C23709T,T24506G,G24914C,G25489T,C27972T,G28048T,A28095T,A28111G,G28280C,A28281T,T28282A,G28881A,G28882A,G28883C,G28913A,C28977T	23	N:D3L,N:R203K,N:G204R,N:G214S,N:S235F,ORF1a:T1001I,ORF1a:A1708D,ORF1a:I2230T,ORF1a:A2345V,ORF1a:L3829F,ORF1b:P314L,ORF3a:A33S,ORF8:Q27*,ORF8:R52I,ORF8:K68*,ORF8:Y73C,S:N501Y,S:A570D,S:D614G,S:P681H,S:T716I,S:S982A,S:D1118H	6	584
hCoV-19/Mexico/CMX-INMEGEN-05-04-172/2021	EPI_ISL_2350006	-	B.1.1.7	GRY	36	C241T,C913T,C3037T,C3267T,C5388A,C5986T,C6196T,A6350G,T6954C,A8848G,C14230T,C14408T,C14676T,C15279T,T16176C,A23063T,C23271A,A23403G,C23604A,C23709T,T24506G,G24914C,G25217T,G26727T,C27881T,C27972T,G28048T,A28111G,G28280C,A28281T,T28282A,G28881A,G28882A,G28883C,C28977T,G29715T	22	M:A69S,N:D3L,N:R203K,N:G204R,N:S235F,ORF1a:T1001I,ORF1a:A1708D,ORF1a:K2029E,ORF1a:I2230T,ORF1b:P255S,ORF1b:P314L,ORF8:Q27*,ORF8:R52I,ORF8:Y73C,S:N501Y,S:A570D,S:D614G,S:P681H,S:T716I,S:S982A,S:D1118H,S:G1219C	6	310
hCoV-19/Mexico/CMX-INMEGEN-05-04-177/2021	EPI_ISL_2350009	-	B.1.1.7	GRY	42	C241T,C913T,C1513T,C2627T,C3037T,C3267T,C3583T,C5388A,C5944T,C5986T,C6312T,T6954C,G11138T,G11401T,C12469T,C14408T,C14676T,T15096C,C15279T,T16176C,C17491T,C19862T,A23063T,C23271A,A23403G,C23604A,C23709T,T24506G,G24914C,C27972T,G28048T,A28095T,A28111G,G28280C,A28281T,T28282A,G28881A,G28882A,G28883C,G28884C,C28977T,C2954	25	N:D3L,N:R203K,N:G204P,N:S235F,ORF1a:L788F,ORF1a:T1001I,ORF1a:A1708D,ORF1a:T2016I,ORF1a:I2230T,ORF1a:A3625S,ORF1a:M3712I,ORF1b:P314L,ORF1b:P1342S,ORF1b:A2132V,ORF8:Q27*,ORF8:R52I,ORF8:K68*,ORF8:Y73C,S:N501Y,S:A570D,S:D614G,S:P681H,S:T716I,S:S982A,S:D1118H	6	170
hCoV-19/Mexico/CMX-INMEGEN-05-04-179/2021	EPI_ISL_2350010	-	B.1.1.7	GRY	37	C241T,C913T,C2453T,C3037T,C3267T,C5388A,C5986T,T6954C,C9611T,C11344T,C14408T,C14676T,C15279T,T16176C,C21855T,G21974C,A23063T,C23271A,A23403G,C23604A,C23709T,T24506G,G24914C,G26774T,G27754T,C27972T,T27984C,G28048T,A28111G,G28280C,A28281T,T28282A,A28295G,G28881A,G28882A,G28883C,C28977T	26	M:M84I,N:D3L,N:N8D,N:R203K,N:G204R,N:S235F,ORF1a:L730F,ORF1a:T1001I,ORF1a:A1708D,ORF1a:I2230T,ORF1a:L3116F,ORF1b:P314L,ORF7a:E121*,ORF8:Q27*,ORF8:Y31H,ORF8:R52I,ORF8:Y73C,S:S98F,S:D138H,S:N501Y,S:A570D,S:D614G,S:P681H,S:T716I,S:S982A,S:D1118H	6	341
hCoV-19/Mexico/CMX-INMEGEN-05-04-18/2021	EPI_ISL_2349896	-	B.1.1.7	GRY	34	C241T,C913T,C2453T,C3037T,C3267T,C5388A,C5986T,T6954C,C8064T,C9611T,C14408T,C14676T,C15279T,T16176C,G20670A,G21468T,C21855T,G21974C,A23063T,C23271A,A23403G,C23604A,C23709T,T24506G,G24914C,C27972T,G28048T,A28111G,T28282A,A28295G,G28881A,G28882A,G28883C,C28977T	24	N:N8D,N:R203K,N:G204R,N:S235F,ORF1a:L730F,ORF1a:T1001I,ORF1a:A1708D,ORF1a:I2230T,ORF1a:S2600L,ORF1a:L3116F,ORF1b:P314L,ORF1b:M2667I,ORF8:Q27*,ORF8:R52I,ORF8:Y73C,S:S98F,S:D138H,S:N501Y,S:A570D,S:D614G,S:P681H,S:T716I,S:S982A,S:D1118H	6	32
hCoV-19/Mexico/CMX-INMEGEN-05-04-215/2021	EPI_ISL_2350035	-	B.1.1.7	GR	37	C241T,C823T,C913T,C2110T,G2305T,G3004T,C3037T,C3267T,C5388A,C5986T,C6813T,T6954C,G7042T,C9803T,C14120T,C14408T,C14676T,C15279T,T16176C,C23271A,A23403G,C23604A,C23709T,T24506G,G24914C,C26110T,C27972T,G28048T,A28095T,A28111G,G28280C,A28281T,T28282A,G28881A,G28882A,G28883C,C28977T	24	N:D3L,N:R203K,N:G204R,N:S235F,ORF1a:K680N,ORF1a:E913D,ORF1a:T1001I,ORF1a:A1708D,ORF1a:T2183I,ORF1a:I2230T,ORF1a:M2259I,ORF1b:P218L,ORF1b:P314L,ORF3a:P240S,ORF8:Q27*,ORF8:R52I,ORF8:K68*,ORF8:Y73C,S:A570D,S:D614G,S:P681H,S:T716I,S:S982A,S:D1118H	6	272
hCoV-19/Mexico/CMX-INMEGEN-05-04-217/2021	EPI_ISL_2350036	-	B.1.1.7	GR	40	C241T,C913T,G2281A,C3037T,C3267T,C5388A,C5986T,T6954C,A12162G,C13665T,C14408T,C14676T,C15279T,C15720T,T16176C,A17615G,C18747T,C20844T,A21137G,C23271A,A23403G,C23604A,C23709T,T24506G,G24914C,G25135T,C26110T,C26521T,G26828T,C27972T,G28048T,A28111G,G28280C,A28281T,T28282A,G28881A,G28882A,G28883C,C28977T,C29095T	22	N:D3L,N:R203K,N:G204R,N:S235F,ORF1a:T1001I,ORF1a:A1708D,ORF1a:I2230T,ORF1a:Q3966R,ORF1b:P314L,ORF1b:K1383R,ORF1b:K2557R,ORF3a:P240S,ORF8:Q27*,ORF8:R52I,ORF8:Y73C,S:A570D,S:D614G,S:P681H,S:T716I,S:S982A,S:D1118H,S:K1191N	6	538
hCoV-19/Mexico/CMX-INMEGEN-05-04-234/2021	EPI_ISL_2350048	-	B.1.1.7	GRY	33	C241T,C913T,C3037T,C3267T,A3492G,C5388A,C5986T,T6954C,C14408T,C14676T,C15279T,T16176C,C18555T,C21639A,A23063T,C23271A,A23403G,C23604A,C23709T,C24374T,T24506G,G24914C,C27972T,G28048T,A28111G,G28280C,A28281T,T28282A,G28881A,G28882A,G28883C,	21	N:D3L,N:R203K,N:G204R,N:S235F,ORF1a:T1001I,ORF1a:N1076S,ORF1a:A1708D,ORF1a:I2230T,ORF1b:P314L,ORF8:Q27*,ORF8:R52I,ORF8:Y73C,S:P26H,S:N501Y,S:A570D,S:D614G,S:P681H,S:T716I,S:L938F,S:S982A,S:D1118H	6	90
hCoV-19/Mexico/CMX-INMEGEN-05-04-300/2021	EPI_ISL_2350092	-	B.1.1.7	G	26	C913T,C3037T,C3267T,C5388A,C5986T,C6196T,T6954C,A11983G,C14408T,C14676T,C15279T,T16176C,C23271A,A23403G,C23604A,C23709T,T24506G,G24914C,C27972T,G28048T,A28111G,G28280C,A28281T,T28282A,C28977T,G29715T	15	N:D3L,N:S235F,ORF1a:T1001I,ORF1a:A1708D,ORF1a:I2230T,ORF1b:P314L,ORF8:Q27*,ORF8:R52I,ORF8:Y73C,S:A570D,S:D614G,S:P681H,S:T716I,S:S982A,S:D1118H	6	3122
hCoV-19/Mexico/CMX-INMEGEN-05-04-321/2021	EPI_ISL_2350105	-	B.1.1.7	GR	41	C913T,G2281A,C3037T,C3267T,C5284T,C5986T,T6954C,T7605C,A12162G,C13665T,C14408T,C14676T,C15279T,C15720T,T16176C,A17615G,C18747T,C20178T,C20844T,A21137G,C23271A,A23403G,C23604A,C23709T,T24506G,G24914C,G25135T,C26110T,C26521T,G26828T,C27972T,G28048T,A28111G,G28280C,A28281T,T28282A,G28881A,G28882A,G28883C,C28977T,C29095T	22	N:D3L,N:R203K,N:G204R,N:S235F,ORF1a:T1001I,ORF1a:I2230T,ORF1a:L2447P,ORF1a:Q3966R,ORF1b:P314L,ORF1b:K1383R,ORF1b:K2557R,ORF3a:P240S,ORF8:Q27*,ORF8:R52I,ORF8:Y73C,S:A570D,S:D614G,S:P681H,S:T716I,S:S982A,S:D1118H,S:K1191N	6	2598
hCoV-19/Mexico/CMX-INMEGEN-05-04-364/2021	EPI_ISL_2350131	-	B.1.1.7	GRY	33	C241T,C913T,C3037T,C3267T,A3492G,C5388A,C5986T,T6954C,C14408T,C14676T,C15279T,T16176C,C18555T,C21639A,A23063T,C23271A,A23403G,C23604A,C23709T,C24374T,T24506G,G24914C,C27972T,G28048T,A28111G,G28280C,A28281T,T28282A,G28881A,G28882A,G28883C,	21	N:D3L,N:R203K,N:G204R,N:S235F,ORF1a:T1001I,ORF1a:N1076S,ORF1a:A1708D,ORF1a:I2230T,ORF1b:P314L,ORF8:Q27*,ORF8:R52I,ORF8:Y73C,S:P26H,S:N501Y,S:A570D,S:D614G,S:P681H,S:T716I,S:L938F,S:S982A,S:D1118H	6	24
hCoV-19/Mexico/CMX-INMEGEN-05-04-40/2021	EPI_ISL_2349916	-	B.1.1.7	GRY	32	C241T,C913T,C2453T,C3037T,C3267T,C5388A,C5986T,T6954C,C9611T,C14408T,C14676T,C15037T,C15279T,T16176C,C21855T,G21974C,A23063T,C23271A,A23403G,C23604A,C23709T,T24506G,G24914C,C27972T,G28048T,A28111G,T28282A,A28295G,G28881A,G28882A,G28883C,C28977T	23	N:N8D,N:R203K,N:G204R,N:S235F,ORF1a:L730F,ORF1a:T1001I,ORF1a:A1708D,ORF1a:I2230T,ORF1a:L3116F,ORF1b:P314L,ORF1b:R524C,ORF8:Q27*,ORF8:R52I,ORF8:Y73C,S:S98F,S:D138H,S:N501Y,S:A570D,S:D614G,S:P681H,S:T716I,S:S982A,S:D1118H	6	274
hCoV-19/Mexico/CMX-INMEGEN-05-04-64/2021	EPI_ISL_2349930	-	B.1.1.7	GR	32	C241T,C913T,C3037T,C3267T,A3492G,C5388A,C5986T,T6954C,C14408T,C14676T,C15279T,T16176C,C18555T,C21639A,C23271A,A23403G,C23604A,C23709T,C24374T,T24506G,G24914C,C27972T,G28048T,A28111G,G28280C,A28281T,T28282A,G28881A,G28882A,G28883C,C28977T,C	20	N:D3L,N:R203K,N:G204R,N:S235F,ORF1a:T1001I,ORF1a:N1076S,ORF1a:A1708D,ORF1a:I2230T,ORF1b:P314L,ORF8:Q27*,ORF8:R52I,ORF8:Y73C,S:P26H,S:A570D,S:D614G,S:P681H,S:T716I,S:L938F,S:S982A,S:D1118H	6	1537
hCoV-19/Mexico/CMX-INMEGEN-05-04-75/2021	EPI_ISL_2349939	-	B.1.1.7	G	30	C241T,C913T,C3037T,C3267T,C5388A,A12162G,C14408T,C14676T,C15279T,C15720T,T16176C,A17615G,C18747T,C20844T,A21137G,C23271A,A23403G,T24506G,G24914C,G25135T,C26521T,G26828T,C27972T,G28048T,A28111G,G28280C,A28281T,T28282A,C28977T,C29095T	16	N:D3L,N:S235F,ORF1a:T1001I,ORF1a:A1708D,ORF1a:Q3966R,ORF1b:P314L,ORF1b:K1383R,ORF1b:K2557R,ORF8:Q27*,ORF8:R52I,ORF8:Y73C,S:A570D,S:D614G,S:S982A,S:D1118H,S:K1191N	6	8667
hCoV-19/Mexico/CMX-INMEGEN-05-04-78/2021	EPI_ISL_2349941	-	B.1.1.7	GRY	40	C222T,C241T,C913T,C2110T,C3037T,C3267T,C5388A,C5986T,T6954C,G7042T,C8389T,C12115T,C13517T,C14120T,C14408T,C14676T,C15279T,T16176C,G16900T,C17762T,A23063T,C23271A,A23403G,C23604A,C23709T,T24506G,G24914C,T25180C,G26416T,C27972T,G28048T,A28095T,A28111G,G28280C,A28281T,T28282A,G28881A,G28882A,G28883C,C28977T	25	E:V58F,N:D3L,N:R203K,N:G204R,N:S235F,ORF1a:T1001I,ORF1a:A1708D,ORF1a:I2230T,ORF1a:M2259I,ORF1b:T17I,ORF1b:P218L,ORF1b:P314L,ORF1b:G1145C,ORF1b:A1432V,ORF8:Q27*,ORF8:R52I,ORF8:K68*,ORF8:Y73C,S:N501Y,S:A570D,S:D614G,S:P681H,S:T716I,S:S982A,S:D1118H	6	495

hCoV-19/Mexico/CMX-INMEGEN-05-04-80/2021	EPI_ISL_2349943	-	B.1.1.7	GRY	35	C241T,C913T,C2453T,C3037T,C3267T,C5388A,C5986T,T6954C,C9166T,C9611T,C14408T,C14676T,C15279T,T16176C,C21855T,G21974C,A23063T,C23271A,A23403G,C23604A,C23709T,C24198T,T24506G,G24914C,C27643T,C27972T,G28048T,A28111G,T28282A,A28295G,C28728T,G28881A,G28882A,G28883C,C28977T	25	N:N8D,N:A152V,N:R203K,N:G204R,N:S235F,ORF1a:L730F,ORF1a:T1001I,ORF1a:A1708D,ORF1a:I2230T,ORF1a:L3116F,ORF1b:P314L,ORF7a:P84S,ORF8:Q27*,ORF8:R52I,ORF8:Y73C,S:S98F,S:D138H,S:N501Y,S:A570D,S:D614G,S:P681H,S:T716I,S:A879V,S:S982A,S:D1118H	6	394
hCoV-19/Mexico/CMX-INMEGEN-05-05-111/2021	EPI_ISL_2881801	-	B.1.1.7	GR	32	C241T,C913T,C3037T,C3267T,C5388A,C5986T,C6196T,T6954C,A8848G,C14408T,C14676T,C15279T,T16176C,T19624C,C23271A,A23403G,C23604A,C23709T,T24506G,G24914C,G25217T,C27972T,G28048T,A28111G,G28280C,A28281T,T28282A,G28881A,G28882A,G28883C,C28977T,G	18	N:D3L,N:R203K,N:G204R,N:S235F,ORF1a:T1001I,ORF1a:A1708D,ORF1a:I2230T,ORF1b:P314L,ORF8:Q27*,ORF8:R52I,ORF8:Y73C,S:A570D,S:D614G,S:P681H,S:T716I,S:S982A,S:D1118H,S:G1219C	6	626
hCoV-19/Mexico/CMX-INMEGEN-05-05-130/2021	EPI_ISL_2444478	-	B.1.1.7	GRY	35	C241T,C913T,C3037T,C3267T,C5388A,C5986T,C6196T,T6954C,A8848G,T11135C,C14408T,C14676T,C15279T,T16176C,A23063T,C23114T,C23271A,A23403G,C23604A,C23709T,G24095T,T24506G,G24914C,A26203G,C27972T,G28048T,A28111G,G28280C,A28281T,T28282A,G28881A,G28882A,G28883C,C28977T,G29715T	21	N:D3L,N:R203K,N:G204R,N:S235F,ORF1a:T1001I,ORF1a:A1708D,ORF1a:I2230T,ORF1a:F3624L,ORF1b:P314L,ORF3a:T271A,ORF8:Q27*,ORF8:R52I,ORF8:Y73C,S:N501Y,S:A570D,S:D614G,S:P681H,S:T716I,S:A845S,S:S982A,S:D1118H	6	2
hCoV-19/Mexico/CMX-INMEGEN-05-05-15/2021	EPI_ISL_2444407	-	B.1.1.7	GRY	34	C241T,C913T,C3037T,C3267T,A3492G,C4331T,C5388A,C5986T,T6954C,C14408T,C14676T,C15279T,T16176C,C18555T,C21639A,A23063T,C23271A,A23403G,C23604A,C23709T,C24374T,T24506G,G24914C,C27972T,G28048T,A28111G,G28280C,A28281T,T28282A,G28881A,G28882A,G2	21	N:D3L,N:R203K,N:G204R,N:S235F,ORF1a:T1001I,ORF1a:N1076S,ORF1a:A1708D,ORF1a:I2230T,ORF1b:P314L,ORF8:Q27*,ORF8:R52I,ORF8:Y73C,S:P26H,S:N501Y,S:A570D,S:D614G,S:P681H,S:T716I,S:L938F,S:S982A,S:D1118H	6	0
hCoV-19/Mexico/CMX-INMEGEN-05-05-155/2021	EPI_ISL_2444493	-	B.1.1.7	GRY	36	C241T,T899C,C913T,C3037T,C3267T,C5388A,C5986T,T6954C,T8815C,G12070T,C14408T,C14676T,T15096C,C15279T,T16176C,T16476C,G18907T,C19290T,A23063T,C23271A,A23403G,C23604A,C23709T,T24506G,G24914C,C27972T,G28048T,A28095T,A28111G,G28280C,A28281T,T28282A,G28881A,G28882A,G28883C,C28977T	21	N:D3L,N:R203K,N:G204R,N:S235F,ORF1a:S212P,ORF1a:T1001I,ORF1a:A1708D,ORF1a:I2230T,ORF1b:P314L,ORF1b:V1814F,ORF8:Q27*,ORF8:R52I,ORF8:K68*,ORF8:Y73C,S:N501Y,S:A570D,S:D614G,S:P681H,S:T716I,S:S982A,S:D1118H	6	6
hCoV-19/Mexico/CMX-INMEGEN-05-05-165/2021	EPI_ISL_2881805	-	B.1.1.7	GRY	38	C241T,C913T,G1957A,A2276G,G2527A,C3037T,A3094T,C3267T,G4136T,C5388A,C5986T,T6954C,C11704T,C14408T,C14676T,C15279T,T16176C,G19542T,G22841A,A23063T,C23271A,A23403G,C23604A,C23709T,T24506G,G24914C,G25954T,C27972T,G28048T,A28111G,G28280C,A28281T,T28282A,C28744T,G28881A,G28882A,G28883C,C28977T	23	N:D3L,N:R203K,N:G204R,N:S235F,ORF1a:I671V,ORF1a:T1001I,ORF1a:V1291F,ORF1a:A1708D,ORF1a:I2230T,ORF1b:P314L,ORF1b:M2025I,ORF3a:G188C,ORF8:Q27*,ORF8:R52I,ORF8:Y73C,S:D427N,S:N501Y,S:A570D,S:D614G,S:P681H,S:T716I,S:S982A,S:D1118H	6	216
hCoV-19/Mexico/CMX-INMEGEN-05-05-167/2021	EPI_ISL_2444501	-	B.1.1.7	GRY	34	C241T,C913T,C2110T,C3037T,C3267T,T5278C,C5388A,C5986T,T6954C,G7042T,A8879G,C14120T,C14408T,C14676T,C15279T,T16176C,A23063T,C23271A,A23403G,C23604A,C23709T,G24415T,G24914C,C27972T,G28048T,A28095T,A28111G,G28280C,A28281T,T28282A,G28881A,G2888	21	N:D3L,N:R203K,N:G204R,N:S235F,ORF1a:T1001I,ORF1a:A1708D,ORF1a:I2230T,ORF1a:M2259I,ORF1a:T2872A,ORF1b:P218L,ORF1b:P314L,ORF8:Q27*,ORF8:R52I,ORF8:K68*,ORF8:Y73C,S:N501Y,S:A570D,S:D614G,S:P681H,S:T716I,S:D	6	169
hCoV-19/Mexico/CMX-INMEGEN-05-05-182/2021	EPI_ISL_2444508	-	B.1.1.7	GRY	35	C241T,C913T,C2110T,C3037T,C3267T,T5278C,C5388A,C5986T,T6954C,G7042T,A8879G,C14120T,C14408T,C14676T,C15279T,T16176C,A23063T,C23271A,A23403G,C23604A,C23709T,G24415T,T24506G,G24914C,C27972T,G28048T,A28095T,A28111G,G28280C,A28281T,T28282A,G28881A,G28882A,G28883C,C28977T	22	N:D3L,N:R203K,N:G204R,N:S235F,ORF1a:T1001I,ORF1a:A1708D,ORF1a:I2230T,ORF1a:M2259I,ORF1a:T2872A,ORF1b:P218L,ORF1b:P314L,ORF8:Q27*,ORF8:R52I,ORF8:K68*,ORF8:Y73C,S:N501Y,S:A570D,S:D614G,S:P681H,S:T716I,S:S982A,S:D1118H	6	16
hCoV-19/Mexico/CMX-INMEGEN-05-05-19/2021	EPI_ISL_2444411	-	B.1.1.7	GR	32	C913T,C2044T,C3037T,C3267T,A3492G,C5388A,C5986T,T6954C,C14408T,C14676T,C15279T,T16176C,C18555T,C21639A,C23271A,A23403G,C23604A,C23709T,C24374T,T24506G,G24914C,C27972T,G28048T,A28111G,G28280C,A28281T,T28282A,G28881A,G28882A,G28883C,C28977T,	20	N:D3L,N:R203K,N:G204R,N:S235F,ORF1a:T1001I,ORF1a:N1076S,ORF1a:A1708D,ORF1a:I2230T,ORF1b:P314L,ORF8:Q27*,ORF8:R52I,ORF8:Y73C,S:P26H,S:A570D,S:D614G,S:P681H,S:T716I,S:L938F,S:S982A,S:D1118H	6	1468
hCoV-19/Mexico/CMX-INMEGEN-05-05-206/2021	EPI_ISL_2550753	-	B.1.1.7	GR	39	C203T,C241T,C913T,G1820A,C3037T,G3251T,C3267T,C5388A,C5986T,C6525T,T6954C,G9526T,A12162G,C14408T,C14676T,C15279T,C15720T,T16176C,A17615G,T19363C,A21137G,C23248T,C23271A,A23403G,C23604A,C23709T,T24506G,G24914C,G25135T,C27972T,G28048T,A28111G,G28280C,A28281T,T28282A,G28881A,G28882A,G28883C,C28977T	25	N:D3L,N:R203K,N:G204R,N:S235F,ORF1a:G519S,ORF1a:D996Y,ORF1a:T1001I,ORF1a:A1708D,ORF1a:T2087I,ORF1a:I2230T,ORF1a:M3087I,ORF1a:Q3966R,ORF1b:P314L,ORF1b:K1383R,ORF1b:K2557R,ORF8:Q27*,ORF8:R52I,ORF8:Y73C,S:A570D,S:D614G,S:P681H,S:T716I,S:S982A,S:D1118H,S:K1191N	6	1010
hCoV-19/Mexico/CMX-INMEGEN-05-05-21/2021	EPI_ISL_2444412	-	B.1.1.7	GRY	35	C241T,C913T,C3037T,C3267T,C5388A,C5986T,C6196T,T6954C,A8848G,C14408T,C14676T,C15279T,T16176C,G19816T,A23063T,C23271A,A23403G,C23604A,C23709T,T24506G,G24914C,G25217T,G26727T,T27285C,C27972T,G28048T,A28111G,G28280C,A28281T,T28282A,G28881A,G28882A,G28883C,C28977T,G29715T	21	M:A69S,N:D3L,N:R203K,N:G204R,N:S235F,ORF1a:T1001I,ORF1a:A1708D,ORF1a:I2230T,ORF1b:P314L,ORF1b:V2117L,ORF8:Q27*,ORF8:R52I,ORF8:Y73C,S:N501Y,S:A570D,S:D614G,S:P681H,S:T716I,S:S982A,S:D1118H,S:G1219C	6	0
hCoV-19/Mexico/CMX-INMEGEN-05-05-232/2021	EPI_ISL_2444538	-	B.1.1.7	GRY	34	C241T,C913T,C3037T,C3267T,A3492G,C4331T,C5388A,C5986T,T6954C,C14408T,C14676T,C15279T,T16176C,C18555T,C21639A,A23063T,C23271A,A23403G,C23604A,C23709T,C24374T,T24506G,G24914C,C27972T,G28048T,A28111G,G28280C,A28281T,T28282A,G28881A,G28882A,G2	21	N:D3L,N:R203K,N:G204R,N:S235F,ORF1a:T1001I,ORF1a:N1076S,ORF1a:A1708D,ORF1a:I2230T,ORF1b:P314L,ORF8:Q27*,ORF8:R52I,ORF8:Y73C,S:P26H,S:N501Y,S:A570D,S:D614G,S:P681H,S:T716I,S:L938F,S:S982A,S:D1118H	6	126
hCoV-19/Mexico/CMX-INMEGEN-05-05-239/2021	EPI_ISL_2444542	-	B.1.1.7	GR	36	C241T,C913T,C2453T,C3037T,C3267T,C5388A,C5986T,A6575T,T6954C,A7432G,C9611T,C14408T,C14676T,C15279T,T16176C,G19999T,C20930T,C21855T,G21974C,C23271A,A23403G,C23604A,C23709T,T24506G,G24914C,C27972T,G28048T,A28111G,G28280C,A28281T,T28282A,A28295G,G28881A,G28882A,G28883C,C28977T	25	N:D3L,N:N8D,N:R203K,N:G204R,N:S235F,ORF1a:L730F,ORF1a:T1001I,ORF1a:A1708D,ORF1a:S2104C,ORF1a:I2230T,ORF1a:L3116F,ORF1b:P314L,ORF1b:V2178F,ORF1b:T2488M,ORF8:Q27*,ORF8:R52I,ORF8:Y73C,S:S98F,S:D138H,S:A570D,S:D614G,S:P681H,S:T716I,S:S982A,S:D1118H	6	482
hCoV-19/Mexico/CMX-INMEGEN-05-05-280/2021	EPI_ISL_2444559	-	B.1.1.7	GRY	37	C241T,T899C,C913T,C3037T,C3267T,C5388A,C5986T,T6954C,T8815C,A10159G,G12070T,C14408T,C14676T,T15096C,C15279T,T16176C,T16476C,G18907T,C19290T,A23063T,C23271A,A23403G,C23604A,C23709T,T24506G,G24914C,C27972T,G28048T,A28095T,A28111G,G28280C,A28281T,T28282A,G28881A,G28882A,G28883C,C28977T	21	N:D3L,N:R203K,N:G204R,N:S235F,ORF1a:S212P,ORF1a:T1001I,ORF1a:A1708D,ORF1a:I2230T,ORF1b:P314L,ORF1b:V1814F,ORF8:Q27*,ORF8:R52I,ORF8:K68*,ORF8:Y73C,S:N501Y,S:A570D,S:D614G,S:P681H,S:T716I,S:S982A,S:D1118H	6	381
hCoV-19/Mexico/CMX-INMEGEN-05-05-29/2021	EPI_ISL_2444417	-	B.1.1.7	GRY	41	A223T,C241T,C913T,C2110T,C3037T,C3267T,G4504A,C5388A,C5986T,C6706T,T6954C,T9127C,C11103T,C12809T,C14120T,C14408T,C14599T,C14676T,C15279T,T16176C,A20412G,A23063T,C23271A,A23403G,C23604A,C23709T,T24506G,G24914C,C25797T,A26924G,C27972T,G28048T,A28095T,A28111G,G28280C,A28281T,T28282A,G28881A,G28882A,G28883C,C28977T	22	N:D3L,N:R203K,N:G204R,N:S235F,ORF1a:T1001I,ORF1a:A1708D,ORF1a:I2230T,ORF1a:P3613L,ORF1a:L4182F,ORF1b:P218L,ORF1b:P314L,ORF8:Q27*,ORF8:R52I,ORF8:K68*,ORF8:Y73C,S:N501Y,S:A570D,S:D614G,S:P681H,S:T716I,S:S982A,S:D1118H	6	0
hCoV-19/Mexico/CMX-INMEGEN-05-05-92/2021	EPI_ISL_2444459	-	B.1.1.7	GR	38	C913T,C2110T,C3037T,C3267T,G4504A,C5388A,C5986T,C6706T,T6954C,T9127C,C11103T,C12809T,C14120T,C14408T,C14599T,C14676T,C15279T,T16176C,A20412G,C23271A,A23403G,C23604A,C23709T,T24506G,G24914C,C25797T,A26924G,C27972T,G28048T,A28095T,A28111G,G28280C,A28281T,T28282A,G28881A,G28882A,G28883C,C28977T	21	N:D3L,N:R203K,N:G204R,N:S235F,ORF1a:T1001I,ORF1a:A1708D,ORF1a:I2230T,ORF1a:P3613L,ORF1a:L4182F,ORF1b:P218L,ORF1b:P314L,ORF8:Q27*,ORF8:R52I,ORF8:K68*,ORF8:Y73C,S:A570D,S:D614G,S:P681H,S:T716I,S:S982A,S:D1118H	6	1421
hCoV-19/Mexico/CMX-INMEGEN-05-05-97/2021	EPI_ISL_2444461	-	B.1.1.7	GR	32	C241T,C913T,C3037T,C3267T,A3492G,C5388A,C5986T,T6954C,C14408T,C14676T,C15279T,T16176C,C18555T,C21639A,C23271A,A23403G,C23604A,C23709T,C24374T,T24506G,G24914C,C27972T,G28048T,A28111G,G28280C,A28281T,T28282A,G28881A,G28882A,G28883C,C28977T,C	20	N:D3L,N:R203K,N:G204R,N:S235F,ORF1a:T1001I,ORF1a:N1076S,ORF1a:A1708D,ORF1a:I2230T,ORF1b:P314L,ORF8:Q27*,ORF8:R52I,ORF8:Y73C,S:P26H,S:A570D,S:D614G,S:P681H,S:T716I,S:L938F,S:S982A,S:D1118H	6	331
hCoV-19/Mexico/CMX-INMEGEN-05-06-172/2021	EPI_ISL_2603665	-	B.1.1.7	GRY	35	C241T,C913T,C3037T,C3267T,C5388A,C5986T,C6196T,T6954C,A8848G,C14408T,C14676T,C15279T,T16176C,G19816T,A23063T,C23271A,A23403G,C23604A,C23709T,T24506G,G24914C,G25217T,G26727T,T27285C,C27972T,G28048T,A28111G,G28280C,A28281T,T28282A,G28881A,G28882A,G28883C,C28977T,G29715T	21	M:A69S,N:D3L,N:R203K,N:G204R,N:S235F,ORF1a:T1001I,ORF1a:A1708D,ORF1a:I2230T,ORF1b:P314L,ORF1b:V2117L,ORF8:Q27*,ORF8:R52I,ORF8:Y73C,S:N501Y,S:A570D,S:D614G,S:P681H,S:T716I,S:S982A,S:D1118H,S:G1219C	6	84
hCoV-19/Mexico/CMX-INMEGEN-05-06-184/2021	EPI_ISL_2603675	-	B.1.1.7	GRY	38	C241T,T400C,C913T,A916G,C3037T,C3267T,G3892T,C5388A,C5986T,T6954C,C8986T,C10341T,C12970T,C14408T,C14676T,C15279T,T16176C,G22745A,A23063T,C23271A,A23403G,C23604A,C23709T,T24506G,G24914C,G25244T,C26527T,G27505T,C27972T,G28048T,A28111G,G28280C,A28281T,T28282A,G28881A,G28882A,G28883C,C28977T	24	M:A2V,N:D3L,N:R203K,N:G204R,N:S235F,ORF1a:T1001I,ORF1a:E1209D,ORF1a:A1708D,ORF1a:I2230T,ORF1a:P3359L,ORF1b:P314L,ORF7a:G38*,ORF8:Q27*,ORF8:R52I,ORF8:Y73C,S:V395I,S:N501Y,S:A570D,S:D614G,S:P681H,S:T716I,S:S982A,S:D1118H,S:V1228L	6	497
hCoV-19/Mexico/CMX-INMEGEN-05-06-185/2021	EPI_ISL_2603676	-	B.1.1.7	GRY	36	C241T,C913T,C2523T,C3037T,C3267T,C5388A,C5986T,C6196T,T6954C,A8848G,C14408T,C14676T,C15279T,T16176C,G19816T,A23063T,C23271A,A23403G,C23604A,C23709T,T24506G,G24914C,G25217T,G26727T,T27285C,C27972T,G28048T,A28111G,G28280C,A28281T,T28282A,G28881A,G28882A,G28883C,C28977T,G29715T	22	M:A69S,N:D3L,N:R203K,N:G204R,N:S235F,ORF1a:T753I,ORF1a:T1001I,ORF1a:A1708D,ORF1a:I2230T,ORF1b:P314L,ORF1b:V2117L,ORF8:Q27*,ORF8:R52I,ORF8:Y73C,S:N501Y,S:A570D,S:D614G,S:P681H,S:T716I,S:S982A,S:D1118H,S:G1219C	6	179

hCoV-19/Mexico/CMX-INMEGEN-05-06-187/2021	EPI_ISL_2603677	-	B.1.1.7	GRY	36	C241T,C913T,C2061T,C3037T,C3267T,C5388A,C5986T,T6954C,C12115T,A12162G,C14408T,C14676T,C15279T,T16176C,A17615G,G20275T,G22346T,A23063T,C23271A,A23403G,C23604A,C23709T,A24322G,T24506G,G24914C,C27855A,C27972T,G28048T,A28111G,G28280C,A28281T,T28282A,G28881A,G28882A,G28883C,C28977T	24	N:D3L,N:R203K,N:G204R,N:S235F,ORF1a:A599V,ORF1a:T1001I,ORF1a:A1708D,ORF1a:I2230T,ORF1a:Q3966R,ORF1b:P314L,ORF1b:K1383R,ORF1b:D2270Y,ORF7b:L34M,ORF8:Q27*,ORF8:R52I,ORF8:Y73C,S:A262S,S:N501Y,S:A570D,S:D614G,S:P681H,S:T716I,S:S982A,S:D1118H	6	88
hCoV-19/Mexico/CMX-INMEGEN-05-06-200/2021	EPI_ISL_2603689	-	B.1.1.7	GRY	37	C222T,C241T,C913T,C2110T,C3037T,C3267T,C5388A,C5986T,T6954C,G7042T,C14120T,C14408T,C14676T,C15279T,T16176C,C17762T,A23063T,C23271A,A23403G,C23604A,C23709T,T24506G,G24914C,T25180C,G25641T,C25672T,C27972T,G28048T,A28095T,A28111G,G28280C,A28281T,T28282A,G28881A,G28882A,G28883C,C28977T	24	N:D3L,N:R203K,N:G204R,N:S235F,ORF1a:T1001I,ORF1a:A1708D,ORF1a:I2230T,ORF1a:M2259I,ORF1b:P218L,ORF1b:P314L,ORF1b:A1432V,ORF3a:L83F,ORF3a:L94F,ORF8:Q27*,ORF8:R52I,ORF8:K68*,ORF8:Y73C,S:N501Y,S:A570D,S:D614G,S:P681H,S:T716I,S:S982A,S:D1118H	6	143
hCoV-19/Mexico/CMX-INMEGEN-05-06-202/2021	EPI_ISL_2603691	-	B.1.1.7	GRY	35	C241T,C913T,C2453T,C3037T,C3267T,C5388A,C5934T,C5986T,T6954C,C9611T,C14408T,C14676T,C15279T,T16176C,C20946T,C21855T,G21974C,A23063T,C23271A,A23403G,C23604A,C23709T,T24506G,G24914C,C27972T,G28048T,A28111G,G28280C,A28281T,T28282A,A28295G,G28881A,G28882A,G28883C,C28977T	24	N:D3L,N:N8D,N:R203K,N:G204R,N:S235F,ORF1a:L730F,ORF1a:T1001I,ORF1a:A1708D,ORF1a:T1890I,ORF1a:I2230T,ORF1a:L3116F,ORF1b:P314L,ORF8:Q27*,ORF8:R52I,ORF8:Y73C,S:S98F,S:D138H,S:N501Y,S:A570D,S:D614G,S:P681H,S:T716I,S:S982A,S:D1118H	6	54
hCoV-19/Mexico/CMX-INMEGEN-05-06-22/2021	EPI_ISL_2603611	-	B.1.1.7	GR	34	C241T,C913T,C3037T,C3267T,C5388A,C5986T,C6196T,T6954C,A8848G,C14408T,C14676T,C15279T,T16176C,G19816T,C23271A,A23403G,C23604A,C23709T,T24506G,G24914C,G25217T,G26727T,T27285C,C27972T,G28048T,A28111G,G28280C,A28281T,T28282A,G28881A,G28882A,G2	20	M:A69S,N:D3L,N:R203K,N:G204R,N:S235F,ORF1a:T1001I,ORF1a:A1708D,ORF1a:I2230T,ORF1b:P314L,ORF1b:V2117L,ORF8:Q27*,ORF8:R52I,ORF8:Y73C,S:A570D,S:D614G,S:P681H,S:T716I,S:S982A,S:D1118H,S:G1219C	6	574
hCoV-19/Mexico/CMX-INMEGEN-05-06-267/2021	EPI_ISL_2603737	-	B.1.1.7	GRY	31	C241T,C913T,C3037T,C3267T,A3492G,C5388A,C5986T,T6954C,C14408T,C14676T,C15279T,T16176C,C21639A,A23063T,C23271A,A23403G,C23604A,C23709T,C24374T,G24770T,G24914C,C27972T,G28048T,A28111G,G28280C,A28281T,T28282A,G28881A,G28882A,G28883C,C28977T	21	N:D3L,N:R203K,N:G204R,N:S235F,ORF1a:T1001I,ORF1a:N1076S,ORF1a:A1708D,ORF1a:I2230T,ORF1b:P314L,ORF8:Q27*,ORF8:R52I,ORF8:Y73C,S:P26H,S:N501Y,S:A570D,S:D614G,S:P681H,S:T716I,S:L938F,S:A1070S,S:D1118H	6	311
hCoV-19/Mexico/CMX-INMEGEN-05-06-273/2021	EPI_ISL_2603742	-	B.1.1.7	GRY	35	C241T,C913T,C2453T,C3037T,C3267T,C5388A,C5934T,C5986T,T6954C,C9611T,C14408T,C14676T,C15279T,T16176C,C20946T,C21855T,G21974C,A23063T,C23271A,A23403G,C23604A,C23709T,T24506G,G24914C,C27972T,G28048T,A28111G,G28280C,A28281T,T28282A,A28295G,G28881A,G28882A,G28883C,C28977T	24	N:D3L,N:N8D,N:R203K,N:G204R,N:S235F,ORF1a:L730F,ORF1a:T1001I,ORF1a:A1708D,ORF1a:T1890I,ORF1a:I2230T,ORF1a:L3116F,ORF1b:P314L,ORF8:Q27*,ORF8:R52I,ORF8:Y73C,S:S98F,S:D138H,S:N501Y,S:A570D,S:D614G,S:P681H,S:T716I,S:S982A,S:D1118H	6	0
hCoV-19/Mexico/CMX-INMEGEN-05-06-279/2021	EPI_ISL_2603748	-	B.1.1.7	GRY	38	C222T,C241T,C913T,C2110T,C3037T,C3267T,C5388A,C5986T,T6954C,G7042T,C14120T,C14408T,C14676T,C15279T,T16176C,C17762T,A23063T,C23271A,C23277T,A23403G,C23604A,C23709T,T24506G,G24914C,T25180C,G25641T,C25672T,C27972T,G28048T,A28095T,A28111G,G28280C,A28281T,T28282A,G28881A,G28882A,G28883C,C28977T	25	N:D3L,N:R203K,N:G204R,N:S235F,ORF1a:T1001I,ORF1a:A1708D,ORF1a:I2230T,ORF1a:M2259I,ORF1b:P218L,ORF1b:P314L,ORF1b:A1432V,ORF3a:L83F,ORF3a:L94F,ORF8:Q27*,ORF8:R52I,ORF8:K68*,ORF8:Y73C,S:N501Y,S:A570D,S:T572I,S:D614G,S:P681H,S:T716I,S:S982A,S:D1118H	6	0
hCoV-19/Mexico/CMX-INMEGEN-05-06-28/2021	EPI_ISL_2603615	-	B.1.1.7	GR	35	C241T,C913T,C1201T,C3037T,C3267T,C5388A,C5986T,C6196T,T6954C,A8848G,C14408T,C14676T,C15279T,T16176C,G19816T,C23271A,A23403G,C23604A,C23709T,T24506G,G24914C,G25217T,G26727T,T27285C,C27972T,G28048T,A28111G,G28280C,A28281T,T28282A,G28881A,G28882A,G28883C,C28977T,G29715T	20	M:A69S,N:D3L,N:R203K,N:G204R,N:S235F,ORF1a:T1001I,ORF1a:A1708D,ORF1a:I2230T,ORF1b:P314L,ORF1b:V2117L,ORF8:Q27*,ORF8:R52I,ORF8:Y73C,S:A570D,S:D614G,S:P681H,S:T716I,S:S982A,S:D1118H,S:G1219C	6	679
hCoV-19/Mexico/CMX-INMEGEN-05-06-280/2021	EPI_ISL_2603749	-	B.1.1.7	GRY	41	C241T,C913T,C2110T,G3004T,C3037T,C3267T,C5388A,C5986T,T6954C,G7042T,C9996T,C10525T,C10789T,C14120T,C14408T,C14676T,C15279T,T16176C,A23063T,C23271A,A23403G,G23593T,C23604A,C23709T,T24506G,G24914C,C25844T,C26110T,C26735T,C27972T,G28048T,A28095T,A28111G,G28280C,A28281T,T28282A,A28804G,G28881A,G28882A,G28883C,C28977T	26	N:D3L,N:R203K,N:G204R,N:S235F,ORF1a:E913D,ORF1a:T1001I,ORF1a:A1708D,ORF1a:I2230T,ORF1a:M2259I,ORF1a:S3244L,ORF1b:P218L,ORF1b:P314L,ORF3a:T151I,ORF3a:P240S,ORF8:Q27*,ORF8:R52I,ORF8:K68*,ORF8:Y73C,S:N501Y,S:A570D,S:D614G,S:Q677H,S:P681H,S:T716I,S:S982A,S:D1118H	6	224
hCoV-19/Mexico/CMX-INMEGEN-05-06-281/2021	EPI_ISL_2603750	-	B.1.1.7	GRY	39	C241T,C913T,C3037T,C3267T,C3695T,C4683T,C5388A,C5986T,T6954C,C7749T,A12162G,C14408T,C14676T,C15279T,C15720T,T16176C,C16985T,A17615G,A21137G,A23063T,C23271A,A23403G,C23604A,C23709T,T24506G,G24914C,G24959T,G25135T,C26110T,C27972T,G28048T,A28111G,G28280C,A28281T,T28282A,G28881A,G28882A,G28883C,C28977T	27	N:D3L,N:R203K,N:G204R,N:S235F,ORF1a:T1001I,ORF1a:A1473V,ORF1a:A1708D,ORF1a:I2230T,ORF1a:T2495I,ORF1a:Q3966R,ORF1b:P314L,ORF1b:T1173I,ORF1b:K1383R,ORF1b:K2557R,ORF3a:P240S,ORF8:Q27*,ORF8:R52I,ORF8:Y73C,S:N501Y,S:A570D,S:D614G,S:P681H,S:T716I,S:S982A,S:D1118H,S:V1133F,S	6	0
hCoV-19/Mexico/CMX-INMEGEN-05-06-286/2021	EPI_ISL_2603754	-	B.1.1.7	GRY	35	C241T,C913T,C2453T,C3037T,C3267T,A3559G,C5388A,A5570G,C5986T,T6954C,C9611T,C14408T,C14676T,C15279T,T16176C,C21855T,G21974C,A23063T,C23271A,A23403G,C23604A,C23709T,T24506G,G24914C,G25273T,C27972T,G28048T,A28111G,T28282A,C28292A,A28295G,G28881A,G28882A,G28883C,C28977T	25	N:Q7K,N:N8D,N:R203K,N:G204R,N:S235F,ORF1a:L730F,ORF1a:T1001I,ORF1a:A1708D,ORF1a:M1769V,ORF1a:I2230T,ORF1a:L3116F,ORF1b:P314L,ORF8:Q27*,ORF8:R52I,ORF8:Y73C,S:S98F,S:D138H,S:N501Y,S:A570D,S:D614G,S:P681H,S:T716I,S:S982A,S:D1118H,S:M1237I	6	194
hCoV-19/Mexico/CMX-INMEGEN-05-06-296/2021	EPI_ISL_2603760	-	B.1.1.7	GRY	34	C241T,C913T,C3037T,C3267T,A3492G,C4331T,C5388A,C5986T,T6954C,C14408T,C14676T,C15279T,T16176C,C18555T,C21639A,A23063T,C23271A,A23403G,C23604A,C23709T,C24374T,T24506G,G24914C,C27972T,G28048T,A28111G,G28280C,A28281T,T28282A,G28881A,G28882A,G2	21	N:D3L,N:R203K,N:G204R,N:S235F,ORF1a:T1001I,ORF1a:N1076S,ORF1a:A1708D,ORF1a:I2230T,ORF1b:P314L,ORF8:Q27*,ORF8:R52I,ORF8:Y73C,S:P26H,S:N501Y,S:A570D,S:D614G,S:P681H,S:T716I,S:L938F,S:S982A,S:D1118H	6	0
hCoV-19/Mexico/CMX-INMEGEN-05-06-298/2021	EPI_ISL_2603762	-	B.1.1.7	GRY	37	C241T,C823T,C913T,C2110T,G3004T,C3037T,C3267T,C5388A,C5986T,T6954C,G7042T,C14120T,C14408T,C14676T,C15279T,T16176C,G18186T,T22501C,A23063T,C23271A,A23403G,C23604A,C23709T,T24506G,G24914C,C26110T,C27972T,G28048T,A28095T,A28111G,G28280C,A28281T,T28282A,G28881A,G28882A,G28883C,C28977T	24	N:D3L,N:R203K,N:G204R,N:S235F,ORF1a:E913D,ORF1a:T1001I,ORF1a:A1708D,ORF1a:I2230T,ORF1a:M2259I,ORF1b:P218L,ORF1b:P314L,ORF1b:M1573I,ORF3a:P240S,ORF8:Q27*,ORF8:R52I,ORF8:K68*,ORF8:Y73C,S:N501Y,S:A570D,S:D614G,S:P681H,S:T716I,S:S982A,S:D1118H	6	0
hCoV-19/Mexico/CMX-INMEGEN-05-06-332/2021	EPI_ISL_2603786	-	Q.3	GR	38	C241T,C913T,C3037T,C3267T,G5023A,C5388A,C5986T,C6568T,T6954C,A9529G,G10396T,C14408T,C14676T,C15279T,T16176C,A17615G,C18131T,G18984T,G20962A,G22094C,C23271A,A23403G,C23604A,C23709T,C24378T,T24506G,G24914C,G26730C,C27972T,G28048T,A28111G,G28280C,A28281T,T28282A,G28881A,G28882A,G28883C,C28977T	25	M:V70L,N:D3L,N:R203K,N:G204R,N:S235F,ORF1a:T1001I,ORF1a:M1586I,ORF1a:A1708D,ORF1a:I2230T,ORF1b:P314L,ORF1b:K1383R,ORF1b:T1555I,ORF1b:M1839I,ORF1b:D2499N,ORF8:Q27*,ORF8:R52I,ORF8:Y73C,S:D178H,S:A570D,S:D614G,S:P681H,S:T716I,S:S939F,S:S982A,S:D1118H	6	999
hCoV-19/Mexico/CMX-INMEGEN-05-06-342/2021	EPI_ISL_2603793	-	B.1.1.7	GRY	37	C241T,C913T,G1820A,C2574T,C3037T,C3267T,A3492G,C5388A,C5986T,T6954C,T9642C,C11674T,C14408T,C14676T,C15279T,T16176C,C18555T,C21639A,A23063T,C23271A,A23403G,C23604A,C23709T,C24374T,T24506G,G24914C,C27972T,G28048T,A28111G,G28280C,A28281T,T28282A,G28881A,G28882A,G28883C,C28977T,C29732T	24	N:D3L,N:R203K,N:G204R,N:S235F,ORF1a:G519S,ORF1a:T770I,ORF1a:T1001I,ORF1a:N1076S,ORF1a:A1708D,ORF1a:I2230T,ORF1a:I3126T,ORF1b:P314L,ORF8:Q27*,ORF8:R52I,ORF8:Y73C,S:P26H,S:N501Y,S:A570D,S:D614G,S:P681H,S:T716I,S:L938F,S:S982A,S:D1118H	6	0
hCoV-19/Mexico/CMX-INMEGEN-05-06-369/2021	EPI_ISL_2603813	-	B.1.1.7	GRY	38	C241T,C913T,C2110T,C3037T,C3267T,G4904T,C5388A,C5986T,T6954C,G7042T,C9166T,A10195G,C14120T,C14408T,C14676T,C15279T,C15933T,T16176C,A23063T,C23271A,A23403G,C23604A,C23709T,T24506G,G24914C,A27745G,C27972T,G28048T,G28075T,A28095T,A28111G,G28280C,A28281T,T28282A,G28881A,G28882A,G28883C,C28977T	24	N:D3L,N:R203K,N:G204R,N:S235F,ORF1a:T1001I,ORF1a:D1547Y,ORF1a:A1708D,ORF1a:I2230T,ORF1a:M2259I,ORF1b:P218L,ORF1b:P314L,ORF7a:R118G,ORF8:Q27*,ORF8:R52I,ORF8:C61F,ORF8:K68*,ORF8:Y73C,S:N501Y,S:A570D,S:D614G,S:P681H,S:T716I,S:S982A,S:D1118H	6	0
hCoV-19/Mexico/CMX-INMEGEN-05-06-40/2021	EPI_ISL_2603625	-	B.1.1.7	GRY	37	C241T,C913T,G1598A,C2453T,C3037T,C3267T,C5388A,C5986T,T6954C,C9611T,C14408T,C14676T,C15279T,T16176C,C21008T,C21855T,G21974C,A23063T,C23271A,A23403G,C23604A,C23709T,T24506G,G24914C,C27643T,C27972T,G28048T,A28111G,T28149A,G28280C,A28281T,T28282A,A28295G,G28881A,G28882A,G28883C,C28977T	27	N:D3L,N:N8D,N:R203K,N:G204R,N:S235F,ORF1a:G445S,ORF1a:L730F,ORF1a:T1001I,ORF1a:A1708D,ORF1a:I2230T,ORF1a:L3116F,ORF1b:P314L,ORF1b:T2514I,ORF7a:P84S,ORF8:Q27*,ORF8:R52I,ORF8:Y73C,ORF8:F86I,S:S98F,S:D138H,S:N501Y,S:A570D,S:D614G,S:P681H,S:T716I,S:S982A,S:D1118H	6	53
hCoV-19/Mexico/CMX-INMEGEN-05-06-47/2021	EPI_ISL_2603630	-	B.1.1.7	G	33	C241T,T754C,C913T,C3037T,C3267T,C5388A,C5986T,T6954C,C7299T,A13768T,C14408T,C14676T,T15096C,C15279T,T16176C,C19862T,C23271A,A23403G,C23604A,C23709T,T24506G,G24914C,C26606T,G27281T,C27972T,G28048T,A28111G,C28153T,G28280C,A28281T,T28282A,C289	20	N:D3L,N:S235F,ORF1a:T1001I,ORF1a:A1708D,ORF1a:I2230T,ORF1a:A2345V,ORF1b:M101L,ORF1b:P314L,ORF1b:A2132V,ORF6:W27L,ORF8:Q27*,ORF8:R52I,ORF8:Y73C,ORF8:T87I,S:A570D,S:D614G,S:P681H,S:T716I,S:S982A,S:D1118H	6	1111
hCoV-19/Mexico/CMX-INMEGEN-05-06-55/2021	EPI_ISL_2603634	-	B.1.1.7	GRY	39	C241T,C913T,C2453T,C3037T,C3093T,C3267T,T4159C,C5388A,C5986T,T6954C,G8653T,C9611T,C9907T,C14408T,C14676T,C15279T,T16176C,C21855T,G21974C,A23063T,C23271A,A23403G,C23604A,C23709T,T24506G,G24914C,C25609T,G27758T,C27972T,G28048T,A28111G,G28280C,A28281T,T28282A,A28295G,G28881A,G28882A,G28883C,C28977T	28	N:D3L,N:N8D,N:R203K,N:G204R,N:S235F,ORF1a:L730F,ORF1a:P943L,ORF1a:T1001I,ORF1a:A1708D,ORF1a:I2230T,ORF1a:M2796I,ORF1a:L3116F,ORF1b:P314L,ORF3a:L73F,ORF7a:*122L,ORF7b:M1I,ORF8:Q27*,ORF8:R52I,ORF8:Y73C,S:S98F,S:D138H,S:N501Y,S:A570D,S:D614G,S:P681H,S:T716I,S:S982A,S:D1118	6	0
hCoV-19/Mexico/CMX-INMEGEN-05-06-61/2021	EPI_ISL_2603636	-	B.1.1.7	GR	35	C241T,A259G,C913T,C3037T,C3267T,C5388A,C5986T,C6196T,T6954C,A8848G,C14408T,C14676T,C15279T,T16176C,G19816T,C23271A,A23403G,C23604A,C23709T,T24506G,G24914C,G25217T,G26727T,T27285C,C27972T,G28048T,A28111G,G28280C,A28281T,T28282A,G28881A,G28882A,G28883C,C28977T,G29715T	20	M:A69S,N:D3L,N:R203K,N:G204R,N:S235F,ORF1a:T1001I,ORF1a:A1708D,ORF1a:I2230T,ORF1b:P314L,ORF1b:V2117L,ORF8:Q27*,ORF8:R52I,ORF8:Y73C,S:A570D,S:D614G,S:P681H,S:T716I,S:S982A,S:D1118H,S:G1219C	6	1563

hCoV-19/Mexico/CMX-INMEGEN-05-06-66/2021	EPI_ISL_2603641	-	B.1.1.7	GR	41	C241T,C913T,C3037T,C3267T,C5388A,C5986T,G6647A,T6954C,A10486G,C14408T,C14676T,T15096C,C15279T,T16176C,C17676T,C18183T,C19524T,A21519C,G22217A,C23271A,A23403G,C23604A,C23709T,A24070T,T24506G,G24914C,C25624T,G26918T,T27384C,C27972T,G28048T,A28095T,A28111G,G28280C,A28281T,T28282A,G28881A,G28882A,G28883C,G28884C,C28977T	23	N:D3L,N:R203K,N:G204P,N:S235F,ORF1a:T1001I,ORF1a:A1708D,ORF1a:A2128T,ORF1a:I2230T,ORF1b:P314L,ORF1b:R2684S,ORF3a:H78Y,ORF8:Q27*,ORF8:R52I,ORF8:K68*,ORF8:Y73C,S:G219S,S:A570D,S:D614G,S:P681H,S:T716I,S:Q836H,S:S982A,S:D1118H	6	1184
hCoV-19/Mexico/CMX-INMEGEN-05-06-72/2021	EPI_ISL_2603646	-	B.1.1.7	GR	41	C241T,C913T,C3037T,C3267T,C5388A,C5944T,C5986T,G6647A,T6954C,C14408T,C14621T,C14676T,T15096C,C15279T,T16176C,C17676T,C18183T,C19524T,A21519C,C23271A,A23403G,C23604A,C23709T,A24070T,A24092G,T24506G,G24914C,C25624T,C27737T,C27972T,G28048T,A28095T,A28111G,G28280C,A28281T,T28282A,G28881A,G28882A,G28883C,G28884C,C28977T	25	N:D3L,N:R203K,N:G204P,N:S235F,ORF1a:T1001I,ORF1a:A1708D,ORF1a:A2128T,ORF1a:I2230T,ORF1b:P314L,ORF1b:T385M,ORF1b:R2684S,ORF3a:H78Y,ORF7a:T115I,ORF8:Q27*,ORF8:R52I,ORF8:K68*,ORF8:Y73C,S:A570D,S:D614G,S:P681H,S:T716I,S:Q836H,S:I844V,S:S982A,S:D1118H	6	377
hCoV-19/Mexico/CMX-INMEGEN-05-06-73/2021	EPI_ISL_2603647	-	B.1.1.7	GR	41	C241T,C913T,C3037T,C3267T,C5388A,C5944T,C5986T,G6647A,T6954C,C14408T,C14621T,C14676T,T15096C,C15279T,T16176C,C17676T,C18183T,C19524T,A21519C,C23271A,A23403G,C23604A,C23709T,A24070T,A24092G,T24506G,G24914C,C25624T,C27737T,C27972T,G28048T,A28095T,A28111G,G28280C,A28281T,T28282A,G28881A,G28882A,G28883C,G28884C,C28977T	25	N:D3L,N:R203K,N:G204P,N:S235F,ORF1a:T1001I,ORF1a:A1708D,ORF1a:A2128T,ORF1a:I2230T,ORF1b:P314L,ORF1b:T385M,ORF1b:R2684S,ORF3a:H78Y,ORF7a:T115I,ORF8:Q27*,ORF8:R52I,ORF8:K68*,ORF8:Y73C,S:A570D,S:D614G,S:P681H,S:T716I,S:Q836H,S:I844V,S:S982A,S:D1118H	6	355
hCoV-19/Mexico/CMX-INMEGEN-05-06-76/2021	EPI_ISL_2603650	-	B.1.1.7	GR	36	C241T,C913T,C2453T,C3037T,C3267T,C5388A,C5986T,A6575T,T6954C,A7432G,C9611T,C14408T,C14676T,C15279T,T16176C,G19999T,C20930T,C21855T,G21974C,C23271A,A23403G,C23604A,C23709T,T24506G,G24914C,C27972T,G28048T,A28111G,G28280C,A28281T,T28282A,A28295G,G28881A,G28882A,G28883C,C28977T	25	N:D3L,N:N8D,N:R203K,N:G204R,N:S235F,ORF1a:L730F,ORF1a:T1001I,ORF1a:A1708D,ORF1a:S2104C,ORF1a:I2230T,ORF1a:L3116F,ORF1b:P314L,ORF1b:V2178F,ORF1b:T2488M,ORF8:Q27*,ORF8:R52I,ORF8:Y73C,S:S98F,S:D138H,S:A570D,S:D614G,S:P681H,S:T716I,S:S982A,S:D1118H	6	227
hCoV-19/Mexico/CMX-INMEGEN-05-06-77/2021	EPI_ISL_2603651	-	B.1.1.7	GRY	37	C241T,C913T,C2453T,C3037T,C3267T,C5388A,C5986T,A6575T,T6954C,A7432G,C9611T,C14408T,C14676T,C15279T,T16176C,G19999T,C20930T,C21855T,G21974C,A23063T,C23271A,A23403G,C23604A,C23709T,T24506G,G24914C,C27972T,G28048T,A28111G,G28280C,A28281T,T28282A,A28295G,G28881A,G28882A,G28883C,C28977T	26	N:D3L,N:N8D,N:R203K,N:G204R,N:S235F,ORF1a:L730F,ORF1a:T1001I,ORF1a:A1708D,ORF1a:S2104C,ORF1a:I2230T,ORF1a:L3116F,ORF1b:P314L,ORF1b:V2178F,ORF1b:T2488M,ORF8:Q27*,ORF8:R52I,ORF8:Y73C,S:S98F,S:D138H,S:N501Y,S:A570D,S:D614G,S:P681H,S:T716I,S:S982A,S:D1118H	6	0
hCoV-19/Mexico/CMX-INMEGEN-05-06-78/2021	EPI_ISL_2603652	-	B.1.1.7	GRY	36	C241T,C913T,C2453T,C3037T,C3267T,C5388A,C5986T,T6954C,C7165T,C9611T,C14408T,C14676T,C15279T,T16176C,C21855T,G21974C,A23063T,C23271A,A23403G,C23604A,C23709T,T24506G,G24914C,G27358T,C27972T,G28048T,A28111G,G28280C,A28281T,T28282A,A28295G,G28881A,G28882A,G28883C,C28977T,C29774T	24	N:D3L,N:N8D,N:R203K,N:G204R,N:S235F,ORF1a:L730F,ORF1a:T1001I,ORF1a:A1708D,ORF1a:I2230T,ORF1a:L3116F,ORF1b:P314L,ORF6:D53Y,ORF8:Q27*,ORF8:R52I,ORF8:Y73C,S:S98F,S:D138H,S:N501Y,S:A570D,S:D614G,S:P681H,S:T716I,S:S982A,S:D1118H	6	0
hCoV-19/Mexico/CMX-INMEGEN-05-06-79/2021	EPI_ISL_2603653	-	B.1.1.7	GRY	43	C241T,C913T,C3037T,C3267T,C5388A,C5944T,C5986T,G6647A,T6954C,C14408T,C14621T,C14676T,T15096C,C15279T,T16176C,C17676T,C18183T,C19524T,C21058T,A21519C,A23063T,C23271A,A23403G,C23604A,C23709T,A24070T,A24092G,T24506G,G24914C,C25624T,C27737T,C27972T,G28048T,A28095T,A28111G,G28280C,A28281T,T28282A,G28881A,G28882A,G28883C,G2	27	N:D3L,N:R203K,N:G204P,N:S235F,ORF1a:T1001I,ORF1a:A1708D,ORF1a:A2128T,ORF1a:I2230T,ORF1b:P314L,ORF1b:T385M,ORF1b:P2531S,ORF1b:R2684S,ORF3a:H78Y,ORF7a:T115I,ORF8:Q27*,ORF8:R52I,ORF8:K68*,ORF8:Y73C,S:N501Y,S:A570D,S:D614G,S:P681H,S:T716I,S:Q836H,S:I844V,S:S982A,S:D1118H	6	168
hCoV-19/Mexico/CMX-INMEGEN-05-06-8/2021	EPI_ISL_2603600	-	B.1.1.7	GRY	41	C222T,C241T,C913T,C2110T,C3037T,C3267T,C4202T,C5388A,C5986T,T6954C,G7042T,G9805T,C10632T,C13517T,C14120T,C14408T,C14676T,C15279T,T16176C,G16900T,C17762T,A23063T,C23271A,A23403G,C23604A,C23709T,T24506G,G24914C,T25180C,G26416T,C27972T,G28048T,A28095T,A28111G,G28280C,A28281T,T28282A,G28881A,G28882A,G28883C,C28977T	26	E:V58F,N:D3L,N:R203K,N:G204R,N:S235F,ORF1a:T1001I,ORF1a:A1708D,ORF1a:I2230T,ORF1a:M2259I,ORF1a:A3456V,ORF1b:T17I,ORF1b:P218L,ORF1b:P314L,ORF1b:G1145C,ORF1b:A1432V,ORF8:Q27*,ORF8:R52I,ORF8:K68*,ORF8:Y73C,S:N501Y,S:A570D,S:D614G,S:P681H,S:T716I,S:S982A,S:D1118H	6	0
hCoV-19/Mexico/CMX-INMEGEN-05-06-84/2021	EPI_ISL_2603655	-	B.1.1.7	GR	36	C241T,C913T,G1820A,C3037T,C3267T,C5388A,C5986T,T6954C,C9967T,C12455T,C13585T,C14408T,C14676T,T15096C,C15279T,T16176C,A17344G,G18412T,C23271A,A23403G,C23604A,C23709T,T24506G,G24914C,C27972T,G28048T,A28095T,A28111G,G28280C,A28281T,T28282A,G28881A,G28882A,G28883C,G28884C,C28977T	22	N:D3L,N:R203K,N:G204P,N:S235F,ORF1a:G519S,ORF1a:T1001I,ORF1a:A1708D,ORF1a:I2230T,ORF1a:L4064F,ORF1b:P314L,ORF1b:I1293V,ORF1b:V1649F,ORF8:Q27*,ORF8:R52I,ORF8:K68*,ORF8:Y73C,S:A570D,S:D614G,S:P681H,S:T716I,S:S982A,S:D1118H	6	456
hCoV-19/Mexico/CMX-INMEGEN-05-06-93/2021	EPI_ISL_2603658	-	B.1.1.7	GR	34	C241T,C913T,C3037T,C3267T,C5388A,C5986T,T6954C,G9802T,C9967T,C14408T,C14676T,T15096C,C15279T,T16176C,A17344G,G18412T,C23271A,A23403G,C23604A,C23709T,T24506G,G24914C,C27972T,G28048T,A28095T,A28111G,G28280C,A28281T,T28282A,G28881A,G28882A,G2	20	N:D3L,N:R203K,N:G204P,N:S235F,ORF1a:T1001I,ORF1a:A1708D,ORF1a:I2230T,ORF1b:P314L,ORF1b:I1293V,ORF1b:V1649F,ORF8:Q27*,ORF8:R52I,ORF8:K68*,ORF8:Y73C,S:A570D,S:D614G,S:P681H,S:T716I,S:S982A,S:D1118H	6	593
hCoV-19/Mexico/CMX-INMEGEN-06-01-132/2021	EPI_ISL_2616957	-	B.1.1.7	GRY	34	C241T,C913T,C1471T,C3037T,C3267T,C5388A,C5730T,C5986T,T6954C,C14408T,C14676T,C15279T,T16176C,G21604T,A23063T,C23271A,A23403G,C23604A,C23709T,T24506G,G24914C,T25025C,G25785C,G26520T,G27670T,C27807T,A28111G,G28280C,A28281T,T28282A,G28881A,G	21	N:D3L,N:R203K,N:G204R,N:S235F,ORF1a:T1001I,ORF1a:A1708D,ORF1a:T1822I,ORF1a:I2230T,ORF1b:P314L,ORF3a:W131C,ORF7a:V93F,ORF8:Y73C,S:Q14H,S:N501Y,S:A570D,S:D614G,S:P681H,S:T716I,S:S982A,S:D1118H,S:Y1155H	6	347
hCoV-19/Mexico/CMX-INMEGEN-06-01-23/2021	EPI_ISL_2616862	-	B.1.1.7	GRY	38	C241T,C913T,C3037T,C3267T,C4206T,C5388A,C5986T,T6954C,G7272A,C9214T,C11572T,C12933T,C14408T,C14676T,C15279T,T16176C,A21466G,C21642T,C21843T,A23063T,C23271A,A23403G,C23604A,C23709T,T24506G,G24914C,C27972T,G28048T,A28111G,G28280C,A28281T,T28282A,G28881A,G28882A,G28883C,C28977T,C29272T,C29784T	24	N:D3L,N:R203K,N:G204R,N:S235F,ORF1a:T1001I,ORF1a:A1314V,ORF1a:A1708D,ORF1a:I2230T,ORF1a:R2336K,ORF1a:P4223L,ORF1b:P314L,ORF1b:M2667V,ORF8:Q27*,ORF8:R52I,ORF8:Y73C,S:A27V,S:S94F,S:N501Y,S:A570D,S:D614G,S:P681H,S:T716I,S:S982A,S:D1118H	6	792
hCoV-19/Mexico/CMX-INMEGEN-06-01-30/2021	EPI_ISL_2616927	-	B.1.1.7	GRY	37	C241T,C913T,C3037T,C3267T,C4206T,C5388A,C5986T,T6954C,G7272A,C11572T,C12933T,C14408T,C14676T,C15279T,T16176C,A21466G,C21843T,T22441A,A23063T,C23271A,A23403G,C23604A,C23709T,T24506G,G24914C,C27972T,G28048T,A28111G,G28280C,A28281T,T28282A,G28881A,G28882A,G28883C,C28977T,C29272T,C29784T	23	N:D3L,N:R203K,N:G204R,N:S235F,ORF1a:T1001I,ORF1a:A1314V,ORF1a:A1708D,ORF1a:I2230T,ORF1a:R2336K,ORF1a:P4223L,ORF1b:P314L,ORF1b:M2667V,ORF8:Q27*,ORF8:R52I,ORF8:Y73C,S:S94F,S:N501Y,S:A570D,S:D614G,S:P681H,S:T716I,S:S982A,S:D1118H	6	485
hCoV-19/Mexico/CMX-INMEGEN-06-01-40/2021	EPI_ISL_2616914	-	B.1.1.7	GRY	39	C241T,C913T,A2317T,C2453T,C3037T,C3267T,C5388A,C5986T,C6433T,T6954C,C9611T,C14408T,C14676T,C15279T,T16176C,C19172T,C21008T,C21855T,G21974C,A23063T,C23271A,A23403G,C23604A,C23709T,T24506G,G24914C,C27643T,C27972T,G28048T,A28111G,G28280C,A28281T,T28282A,A28295G,G28881A,G28882A,G28883C,C28977T,T29655C	27	N:D3L,N:N8D,N:R203K,N:G204R,N:S235F,ORF1a:K684N,ORF1a:L730F,ORF1a:T1001I,ORF1a:A1708D,ORF1a:I2230T,ORF1a:L3116F,ORF1b:P314L,ORF1b:T1902I,ORF1b:T2514I,ORF7a:P84S,ORF8:Q27*,ORF8:R52I,ORF8:Y73C,S:S98F,S:D138H,S:N501Y,S:A570D,S:D614G,S:P681H,S:T716I,S:S982A,S:D1118H	6	573
hCoV-19/Mexico/CMX-INMEGEN-06-01-58/2021	EPI_ISL_2616976	-	B.1.1.7	GR	33	C823T,C913T,C2110T,G2305T,G3004T,C3037T,C3267T,C5388A,T6954C,G7042T,C9803T,C14120T,C14408T,C14676T,C15279T,T16176C,C23271A,A23403G,C23604A,C23709T,T24506G,G24914C,C26110T,C27972T,G28048T,A28111G,G28280C,A28281T,T28282A,G28881A,G28882A,G28883C,C28977T	22	N:D3L,N:R203K,N:G204R,N:S235F,ORF1a:K680N,ORF1a:E913D,ORF1a:T1001I,ORF1a:A1708D,ORF1a:I2230T,ORF1a:M2259I,ORF1b:P218L,ORF1b:P314L,ORF3a:P240S,ORF8:Q27*,ORF8:R52I,ORF8:Y73C,S:A570D,S:D614G,S:P681H,S:T716I,S:S982A,S:D1118H	6	2833
hCoV-19/Mexico/CMX-INMEGEN-06-01-62/2021	EPI_ISL_2616899	-	B.1.1.7	GRY	40	C241T,C913T,C2710T,C3037T,C3267T,C5388A,C5986T,G6647A,T6954C,C14408T,C14621T,C14676T,T15096C,C15279T,T16176C,C17676T,C18183T,A21519C,A23063T,C23271A,A23403G,C23604A,C23709T,A24070T,A24092G,T24506G,G24914C,C25624T,C27737T,C27972T,G28048T,A28111G,G28280C,A28281T,T28282A,G28881A,G28882A,G28883C,G28884C,C28977T	25	N:D3L,N:R203K,N:G204P,N:S235F,ORF1a:T1001I,ORF1a:A1708D,ORF1a:A2128T,ORF1a:I2230T,ORF1b:P314L,ORF1b:T385M,ORF1b:R2684S,ORF3a:H78Y,ORF7a:T115I,ORF8:Q27*,ORF8:R52I,ORF8:Y73C,S:N501Y,S:A570D,S:D614G,S:P681H,S:T716I,S:Q836H,S:I844V,S:S982A,S:D1118H	6	629
hCoV-19/Mexico/CMX-INMEGEN-06-01-73/2021	EPI_ISL_2616940	-	B.1.1.7	GRY	35	C241T,C913T,C1471T,C3037T,C3267T,C5388A,C5730T,C5986T,T6954C,C14408T,C14676T,C15279T,T16176C,C21057T,G21604T,A23063T,C23271A,A23403G,C23604A,C23709T,T24506G,G24914C,T25025C,G25785C,G26520T,G27670T,C27807T,A28111G,G28280C,A28281T,T28282A,G28881A,G28882A,G28883C,C28977T	21	N:D3L,N:R203K,N:G204R,N:S235F,ORF1a:T1001I,ORF1a:A1708D,ORF1a:T1822I,ORF1a:I2230T,ORF1b:P314L,ORF3a:W131C,ORF7a:V93F,ORF8:Y73C,S:Q14H,S:N501Y,S:A570D,S:D614G,S:P681H,S:T716I,S:S982A,S:D1118H,S:Y1155H	6	408
hCoV-19/Mexico/CMX-INMEGEN-06-01-80/2021	EPI_ISL_2616996	-	B.1.1.7	G	38	C913T,C3037T,C3267T,T4909C,C5388A,C5986T,T6954C,C7299T,T8971C,C9443T,C11620T,T12601A,A13768T,C14408T,C14676T,T15096C,C15279T,T16176C,C19862T,C23271A,A23403G,C23604A,C23709T,T24506G,G24914C,G25354T,C26606T,G27281T,C27972T,G28048T,A28111G,G28280C,A28281T,T28282A,C28977T,G29392T,A29491G,C29769T	22	N:D3L,N:S235F,N:K373N,ORF1a:T1001I,ORF1a:A1708D,ORF1a:I2230T,ORF1a:A2345V,ORF1a:L3060F,ORF1a:S4112R,ORF1b:M101L,ORF1b:P314L,ORF1b:A2132V,ORF6:W27L,ORF8:Q27*,ORF8:R52I,ORF8:Y73C,S:A570D,S:D614G,S:P681H,S:T716I,S:S982A,S:D1118H	6	1686
hCoV-19/Mexico/CMX-INMEGEN-06-02-190/2021	EPI_ISL_2692703	-	B.1.1.7	GRY	38	C241T,C913T,C3037T,C3267T,C4206T,C5388A,C5986T,T6954C,G7272A,C11572T,C12933T,C14408T,C14676T,C15279T,T16176C,A21466G,C21843T,T22441A,A23063T,C23271A,A23403G,C23604A,C23709T,T24506G,G24914C,G26501T,C27972T,G28048T,A28111G,G28280C,A28281T,T28282A,G28881A,G28882A,G28883C,C28977T,C29272T,C29784T	23	N:D3L,N:R203K,N:G204R,N:S235F,ORF1a:T1001I,ORF1a:A1314V,ORF1a:A1708D,ORF1a:I2230T,ORF1a:R2336K,ORF1a:P4223L,ORF1b:P314L,ORF1b:M2667V,ORF8:Q27*,ORF8:R52I,ORF8:Y73C,S:S94F,S:N501Y,S:A570D,S:D614G,S:P681H,S:T716I,S:S982A,S:D1118H	6	3

hCoV-19/Mexico/CMX-INMEGEN-06-02-203/2021	EPI_ISL_2692715	-	B.1.1.7	GRY	43	C241T,C913T,C2110T,C2453T,G3004T,C3037T,C3267T,C5388A,C5986T,T6954C,G7042T,C9996T,C10525T,C10789T,C14120T,C14408T,C14676T,C15279T,T16176C,A23063T,C23271A,A23403G,G23593T,C23604A,C23709T,T24506G,G24914C,C25844T,C26110T,C26735T,C27389T,C27972T,G28048T,A28095T,A28111G,G28280C,A28281T,T28282A,A28804G,G28881A,G28882A,G28883	27	N:D3L,N:R203K,N:G204R,N:S235F,ORF1a:L730F,ORF1a:E913D,ORF1a:T1001I,ORF1a:A1708D,ORF1a:I2230T,ORF1a:M2259I,ORF1a:S3244L,ORF1b:P218L,ORF1b:P314L,ORF3a:T151I,ORF3a:P240S,ORF8:Q27*,ORF8:R52I,ORF8:K68*,ORF8:Y73C,S:N501Y,S:A570D,S:D614G,S:Q677H,S:P681H,S:T716I,S:S982A,S:D11	6	0
hCoV-19/Mexico/CMX-INMEGEN-06-02-228/2021	EPI_ISL_2692739	-	Q.3	GRY	40	C241T,C913T,C1480T,C3037T,C3267T,G5023A,C5388A,C5986T,C6568T,T6954C,G8183A,A9529G,C9967T,G10396T,C14408T,C14676T,C15279T,T16176C,A17615G,G20962A,G22094C,A23063T,C23271A,A23403G,C23604A,C23709T,T24506G,G24914C,G26730C,C27972T,G28048T,A28111G,G28280C,A28281T,T28282A,C28453T,G28881A,G28882A,G28883C,C28977T	25	M:V70L,N:D3L,N:R203K,N:G204R,N:S235F,ORF1a:T1001I,ORF1a:M1586I,ORF1a:A1708D,ORF1a:I2230T,ORF1a:G2640R,ORF1b:P314L,ORF1b:K1383R,ORF1b:D2499N,ORF8:Q27*,ORF8:R52I,ORF8:Y73C,ORF9b:A57V,S:D178H,S:N501Y,S:A570D,S:D614G,S:P681H,S:T716I,S:S982A,S:D1118H	6	572
hCoV-19/Mexico/CMX-INMEGEN-06-02-246/2021	EPI_ISL_2692754	-	Q.3	GRY	40	C241T,C913T,C3037T,C3267T,G5023A,C5031T,C5388A,C5986T,C6568T,T6954C,G8183A,A9529G,G10396T,G11365T,C14408T,C14676T,C15279T,T16176C,A17615G,G20962A,G22094C,A23063T,C23271A,A23403G,C23604A,C23709T,T24506G,G24914C,G25555T,G26730C,C27972T,G28048T,A28111G,G28280C,A28281T,T28282A,G28881A,G28882A,G28883C,C28977T	26	M:V70L,N:D3L,N:R203K,N:G204R,N:S235F,ORF1a:T1001I,ORF1a:M1586I,ORF1a:T1589I,ORF1a:A1708D,ORF1a:I2230T,ORF1a:G2640R,ORF1b:P314L,ORF1b:K1383R,ORF1b:D2499N,ORF3a:V55F,ORF8:Q27*,ORF8:R52I,ORF8:Y73C,S:D178H,S:N501Y,S:A570D,S:D614G,S:P681H,S:T716I,S:S982A,S:D1118H	6	221
hCoV-19/Mexico/CMX-INMEGEN-06-02-304/2021	EPI_ISL_2692795	-	B.1.1.7	GRY	34	C241T,C913T,C3037T,C3267T,A3492G,C4331T,C5388A,C5986T,T6954C,C14408T,C14676T,C15279T,T16176C,C18555T,C21639A,A23063T,C23271A,A23403G,C23604A,C23709T,C24374T,T24506G,G24914C,C27972T,G28048T,A28111G,G28280C,A28281T,T28282A,G28881A,G28882A,G2	21	N:D3L,N:R203K,N:G204R,N:S235F,ORF1a:T1001I,ORF1a:N1076S,ORF1a:A1708D,ORF1a:I2230T,ORF1b:P314L,ORF8:Q27*,ORF8:R52I,ORF8:Y73C,S:P26H,S:N501Y,S:A570D,S:D614G,S:P681H,S:T716I,S:L938F,S:S982A,S:D1118H	6	39
hCoV-19/Mexico/CMX-INMEGEN-06-02-305/2021	EPI_ISL_2692796	-	B.1.1.7	GR	35	C241T,G521T,C913T,C3037T,C3267T,A3492G,C5388A,C5986T,T6954C,C12114T,C14408T,C14676T,C15279T,T16176C,A18147G,C18555T,C21639A,C23271A,A23403G,C23604A,C23709T,C24374T,T24506G,G24914C,C27972T,G28048T,A28111G,G28280C,A28281T,T28282A,G28881A,G28882A,G28883C,C28977T,C29732T	22	N:D3L,N:R203K,N:G204R,N:S235F,ORF1a:V86F,ORF1a:T1001I,ORF1a:N1076S,ORF1a:A1708D,ORF1a:I2230T,ORF1a:S3950F,ORF1b:P314L,ORF8:Q27*,ORF8:R52I,ORF8:Y73C,S:P26H,S:A570D,S:D614G,S:P681H,S:T716I,S:L938F,S:S982A,S:D1118H	6	305
hCoV-19/Mexico/CMX-INMEGEN-06-02-308/2021	EPI_ISL_2692798	-	B.1.1.7	GRY	37	C241T,C913T,C3037T,C3267T,A3492G,C4331T,C5388A,C5986T,T6954C,C13423A,C13424A,C14408T,C14676T,C15279T,T16176C,C18555T,C21639A,A23063T,C23271A,A23403G,A23523C,C23604A,C23709T,C24374T,T24506G,G24914C,C27972T,G28048T,A28111G,G28280C,A28281T,T28282A,G28881A,G28882A,G28883C,C28977T,C29732T	23	N:D3L,N:R203K,N:G204R,N:S235F,ORF1a:T1001I,ORF1a:N1076S,ORF1a:A1708D,ORF1a:I2230T,ORF1a:R4387S,ORF1b:P314L,ORF8:Q27*,ORF8:R52I,ORF8:Y73C,S:P26H,S:N501Y,S:A570D,S:D614G,S:E654A,S:P681H,S:T716I,S:L938F,S:S982A,S:D1118H	6	0
hCoV-19/Mexico/CMX-INMEGEN-06-03-138/2021	EPI_ISL_2810128	-	B.1.1.7	GRY	35	C241T,C913T,C3037T,C3267T,C5388A,C5986T,C6196T,T6954C,A8848G,C14408T,C14676T,C15279T,T16176C,T19624C,A23063T,C23271A,A23403G,C23604A,C23709T,G24173A,T24506G,G24914C,G25217T,C25844T,C27972T,G28048T,A28111G,G28280C,A28281T,T28282A,G28881A,G28882A,G28883C,C28977T,G29715T	21	N:D3L,N:R203K,N:G204R,N:S235F,ORF1a:T1001I,ORF1a:A1708D,ORF1a:I2230T,ORF1b:P314L,ORF3a:T151I,ORF8:Q27*,ORF8:R52I,ORF8:Y73C,S:N501Y,S:A570D,S:D614G,S:P681H,S:T716I,S:A871T,S:S982A,S:D1118H,S:G1219C	6	13
hCoV-19/Mexico/CMX-INMEGEN-06-03-147/2021	EPI_ISL_2810137	-	Q.3	GRY	39	C241T,C913T,C3037T,C3267T,G5023A,C5031T,C5388A,C5986T,C6568T,T6954C,G8183A,A9529G,G10396T,C14408T,C14676T,C15279T,T16176C,A17615G,G20962A,G22094C,A23063T,C23271A,A23403G,C23604A,C23709T,T24506G,G24914C,G25555T,G26730C,C27972T,G28048T,A28111G,G28280C,A28281T,T28282A,G28881A,G28882A,G28883C,C28977T	26	M:V70L,N:D3L,N:R203K,N:G204R,N:S235F,ORF1a:T1001I,ORF1a:M1586I,ORF1a:T1589I,ORF1a:A1708D,ORF1a:I2230T,ORF1a:G2640R,ORF1b:P314L,ORF1b:K1383R,ORF1b:D2499N,ORF3a:V55F,ORF8:Q27*,ORF8:R52I,ORF8:Y73C,S:D178H,S:N501Y,S:A570D,S:D614G,S:P681H,S:T716I,S:S982A,S:D1118H	6	37
hCoV-19/Mexico/CMX-INMEGEN-06-03-150/2021	EPI_ISL_2810140	-	B.1.1.7	GRY	36	C241T,G521T,C913T,C3037T,C3267T,A3492G,C5388A,C5986T,T6954C,C12114T,C14408T,C14676T,C15279T,T16176C,A18147G,C18555T,C21639A,A23063T,C23271A,A23403G,C23604A,C23709T,C24374T,T24506G,G24914C,C27972T,G28048T,A28111G,G28280C,A28281T,T28282A,G28881A,G28882A,G28883C,C28977T,C29732T	23	N:D3L,N:R203K,N:G204R,N:S235F,ORF1a:V86F,ORF1a:T1001I,ORF1a:N1076S,ORF1a:A1708D,ORF1a:I2230T,ORF1a:S3950F,ORF1b:P314L,ORF8:Q27*,ORF8:R52I,ORF8:Y73C,S:P26H,S:N501Y,S:A570D,S:D614G,S:P681H,S:T716I,S:L938F,S:S982A,S:D1118H	6	708
hCoV-19/Mexico/CMX-INMEGEN-06-03-155/2021	EPI_ISL_2810143	-	Q.3	GRY	39	C241T,C913T,C3037T,C3267T,G5023A,C5031T,C5388A,C5986T,C6568T,T6954C,G8183A,A9529G,G10396T,C14408T,C14676T,C15279T,T16176C,A17615G,G20962A,G22094C,A23063T,C23271A,A23403G,C23604A,C23709T,T24506G,G24914C,G25555T,G26730C,C27972T,G28048T,A28111G,G28280C,A28281T,T28282A,G28881A,G28882A,G28883C,C28977T	26	M:V70L,N:D3L,N:R203K,N:G204R,N:S235F,ORF1a:T1001I,ORF1a:M1586I,ORF1a:T1589I,ORF1a:A1708D,ORF1a:I2230T,ORF1a:G2640R,ORF1b:P314L,ORF1b:K1383R,ORF1b:D2499N,ORF3a:V55F,ORF8:Q27*,ORF8:R52I,ORF8:Y73C,S:D178H,S:N501Y,S:A570D,S:D614G,S:P681H,S:T716I,S:S982A,S:D1118H	6	68
hCoV-19/Mexico/CMX-INMEGEN-06-03-163/2021	EPI_ISL_2810149	-	B.1.1.7	GRY	40	C241T,C913T,A2317T,C2453T,C3037T,C3267T,C5388A,C5986T,C6433T,T6954C,C9611T,G11273T,C11750T,C14408T,C14676T,C15279T,T16176C,C21008T,C21855T,G21974C,A23063T,C23271A,A23403G,C23604A,C23709T,T24506G,G24914C,C25710T,C27643T,C27972T,G28048T,A28111G,G28280C,A28281T,T28282A,A28295G,G28881A,G28882A,G28883C,C28977T	28	N:D3L,N:N8D,N:R203K,N:G204R,N:S235F,ORF1a:K684N,ORF1a:L730F,ORF1a:T1001I,ORF1a:A1708D,ORF1a:I2230T,ORF1a:L3116F,ORF1a:V3670F,ORF1a:L3829F,ORF1b:P314L,ORF1b:T2514I,ORF7a:P84S,ORF8:Q27*,ORF8:R52I,ORF8:Y73C,S:S98F,S:D138H,S:N501Y,S:A570D,S:D614G,S:P681H,S:T716I,S:S982A,S:D	6	530
hCoV-19/Mexico/CMX-INMEGEN-06-03-189/2021	EPI_ISL_2842783	-	B.1.1.7	GR	31	C241T,C913T,C3037T,C3267T,A3492G,C5388A,C5986T,T6954C,C14408T,C14676T,C15279T,T16176C,C21639A,C23271A,A23403G,C23604A,C23709T,C24374T,T24506G,G24914C,A25164T,C27972T,G28048T,A28111G,G28280C,A28281T,T28282A,G28881A,G28882A,G28883C,C28977T	21	N:D3L,N:R203K,N:G204R,N:S235F,ORF1a:T1001I,ORF1a:N1076S,ORF1a:A1708D,ORF1a:I2230T,ORF1b:P314L,ORF8:Q27*,ORF8:R52I,ORF8:Y73C,S:P26H,S:A570D,S:D614G,S:P681H,S:T716I,S:L938F,S:S982A,S:D1118H,S:Q1201L	6	650
hCoV-19/Mexico/CMX-INMEGEN-06-03-212/2021	EPI_ISL_2842785	-	B.1.1.7	GRY	42	C241T,C913T,C3037T,C3267T,C5388A,C5944T,C5986T,G6647A,T6954C,C14408T,C14621T,C14676T,T15096C,C15279T,T16176C,C17676T,C18183T,C19524T,A21519C,A23063T,C23271A,A23403G,C23604A,C23709T,A24070T,A24092G,T24506G,G24914C,C25624T,C27737T,C27972T,G28048T,A28095T,A28111G,G28280C,A28281T,T28282A,G28881A,G28882A,G28883C,G28884C,C	26	N:D3L,N:R203K,N:G204P,N:S235F,ORF1a:T1001I,ORF1a:A1708D,ORF1a:A2128T,ORF1a:I2230T,ORF1b:P314L,ORF1b:T385M,ORF1b:R2684S,ORF3a:H78Y,ORF7a:T115I,ORF8:Q27*,ORF8:R52I,ORF8:K68*,ORF8:Y73C,S:N501Y,S:A570D,S:D614G,S:P681H,S:T716I,S:Q836H,S:I844V,S:S982A,S:D1118H	6	49
hCoV-19/Mexico/CMX-INMEGEN-06-03-228/2021	EPI_ISL_2842803	-	B.1.1.7	GRY	37	C241T,C913T,C3037T,C3267T,C4206T,C5388A,C5986T,T6954C,G7272A,C11572T,C12933T,C14408T,C14676T,C15279T,T16176C,A21466G,C21843T,T22441A,A23063T,C23271A,A23403G,C23604A,C23709T,T24506G,G24914C,C27972T,G28048T,A28111G,G28280C,A28281T,T28282A,G28881A,G28882A,G28883C,C28977T,C29272T,C29784T	23	N:D3L,N:R203K,N:G204R,N:S235F,ORF1a:T1001I,ORF1a:A1314V,ORF1a:A1708D,ORF1a:I2230T,ORF1a:R2336K,ORF1a:P4223L,ORF1b:P314L,ORF1b:M2667V,ORF8:Q27*,ORF8:R52I,ORF8:Y73C,S:S94F,S:N501Y,S:A570D,S:D614G,S:P681H,S:T716I,S:S982A,S:D1118H	6	19
hCoV-19/Mexico/CMX-INMEGEN-06-03-284/2021	EPI_ISL_2810246	-	B.1.1.7	GRY	36	C241T,C913T,C2110T,G3004T,C3037T,C3267T,C5388A,C5986T,T6954C,G7042T,C14120T,C14408T,C14676T,C15279T,T16176C,G16900A,C17474T,A23063T,C23271A,A23403G,C23604A,C23709T,T24506G,G24914C,C26110T,C27972T,G28048T,A28095T,A28111G,G28280C,A28281T,T28282A,G28881A,G28882A,G28883C,C28977T	25	N:D3L,N:R203K,N:G204R,N:S235F,ORF1a:E913D,ORF1a:T1001I,ORF1a:A1708D,ORF1a:I2230T,ORF1a:M2259I,ORF1b:P218L,ORF1b:P314L,ORF1b:G1145S,ORF1b:T1336I,ORF3a:P240S,ORF8:Q27*,ORF8:R52I,ORF8:K68*,ORF8:Y73C,S:N501Y,S:A570D,S:D614G,S:P681H,S:T716I,S:S982A,S:D1118H	9	0
hCoV-19/Mexico/CMX-INMEGEN-06-03-319/2021	EPI_ISL_2810277	-	B.1.1.7	GRY	36	C241T,C913T,C3037T,C3267T,C5388A,C5986T,C6196T,T6954C,A8848G,C14408T,C14676T,C15279T,T16176C,T16461C,T19624C,A23063T,C23271A,A23403G,C23604A,C23709T,G24173A,T24506G,G24914C,G25217T,C25844T,C27972T,G28048T,A28111G,G28280C,A28281T,T28282A,G28881A,G28882A,G28883C,C28977T,G29715T	21	N:D3L,N:R203K,N:G204R,N:S235F,ORF1a:T1001I,ORF1a:A1708D,ORF1a:I2230T,ORF1b:P314L,ORF3a:T151I,ORF8:Q27*,ORF8:R52I,ORF8:Y73C,S:N501Y,S:A570D,S:D614G,S:P681H,S:T716I,S:A871T,S:S982A,S:D1118H,S:G1219C	6	2
hCoV-19/Mexico/CMX-INMEGEN-06-03-32/2021	EPI_ISL_2810050	-	B.1.1.7	GRY	35	C241T,C913T,C2453T,C3037T,C3267T,C5388A,C5986T,T6954C,C7165T,C9611T,C14408T,C14676T,C15279T,T16176C,G18498T,C21855T,G21974C,A23063T,C23271A,A23403G,C23604A,C23709T,T24506G,G24914C,C27972T,G28048T,A28111G,G28280C,A28281T,T28282A,A28295G,G28881A,G28882A,G28883C,C28977T	24	N:D3L,N:N8D,N:R203K,N:G204R,N:S235F,ORF1a:L730F,ORF1a:T1001I,ORF1a:A1708D,ORF1a:I2230T,ORF1a:L3116F,ORF1b:P314L,ORF1b:M1677I,ORF8:Q27*,ORF8:R52I,ORF8:Y73C,S:S98F,S:D138H,S:N501Y,S:A570D,S:D614G,S:P681H,S:T716I,S:S982A,S:D1118H	6	0
hCoV-19/Mexico/CMX-INMEGEN-06-03-33/2021	EPI_ISL_2810051	-	B.1.1.7	GR	37	C241T,C913T,G1322T,C3037T,C3267T,C5388A,C5986T,C6196T,T6954C,A8848G,C14408T,C14676T,C15279T,T16176C,G18169T,T18573C,G21724T,C23271A,A23403G,C23604A,C23709T,T24506G,G24914C,G25217T,G26727T,C27972T,G28048T,A28111G,G28280C,A28281T,T28282A,G28881A,G28882A,G28883C,C28977T,C29686T,G29715T	22	M:A69S,N:D3L,N:R203K,N:G204R,N:S235F,ORF1a:G353C,ORF1a:T1001I,ORF1a:A1708D,ORF1a:I2230T,ORF1b:P314L,ORF1b:G1568C,ORF8:Q27*,ORF8:R52I,ORF8:Y73C,S:L54F,S:A570D,S:D614G,S:P681H,S:T716I,S:S982A,S:D1118H,S:G1219C	6	1544
hCoV-19/Mexico/CMX-INMEGEN-06-03-38/2021	EPI_ISL_2810055	-	B.1.1.7	G	32	C241T,C913T,C3037T,C3267T,C5388A,C5986T,T6954C,C10277T,C14408T,C14676T,C15279T,C16173T,T16176C,A16534G,C18486T,A21012C,C21855T,C22264T,A23063T,C23271A,A23403G,C23604A,C23709T,C24034T,T24506G,G24914C,T28246C,G28280C,A28281T,T28282A,C28977T,C2	17	N:D3L,N:S235F,N:A336V,ORF1a:T1001I,ORF1a:A1708D,ORF1a:I2230T,ORF1a:L3338F,ORF1b:P314L,ORF1b:S1023G,S:S98F,S:N501Y,S:A570D,S:D614G,S:P681H,S:T716I,S:S982A,S:D1118H	6	399
hCoV-19/Mexico/CMX-INMEGEN-06-03-39/2021	EPI_ISL_2810056	-	B.1.1.7	GR	34	C241T,C913T,C3037T,C3267T,C5388A,C5986T,C6196T,C6813T,T6954C,A8848G,T12730A,C14408T,C14676T,C15279T,T16176C,T16521C,C23271A,A23403G,C23604A,C23709T,T24506G,G24914C,G27852T,C27972T,G28048T,A28111G,G28280C,A28281T,T28282A,G28881A,G28882A,G28	19	N:D3L,N:R203K,N:G204R,N:S235F,ORF1a:T1001I,ORF1a:A1708D,ORF1a:T2183I,ORF1a:I2230T,ORF1b:P314L,ORF7b:E33*,ORF8:Q27*,ORF8:R52I,ORF8:Y73C,S:A570D,S:D614G,S:P681H,S:T716I,S:S982A,S:D1118H	6	546

hCoV-19/Mexico/CMX-INMEGEN-06-03-41/2021	EPI_ISL_2810058	-	B.1.1.7	GRY	38	C241T,C913T,G1322T,C3037T,C3267T,C5388A,C5986T,C6196T,T6954C,A8848G,C14408T,C14676T,C15279T,T16176C,G18169T,T18573C,G21724T,A23063T,C23271A,A23403G,C23604A,C23709T,T24506G,G24914C,G25217T,G26727T,C27972T,G28048T,A28111G,G28280C,A28281T,T28282A,G28881A,G28882A,G28883C,C28977T,C29686T,G29715T	23	M:A69S,N:D3L,N:R203K,N:G204R,N:S235F,ORF1a:G353C,ORF1a:T1001I,ORF1a:A1708D,ORF1a:I2230T,ORF1b:P314L,ORF1b:G1568C,ORF8:Q27*,ORF8:R52I,ORF8:Y73C,S:L54F,S:N501Y,S:A570D,S:D614G,S:P681H,S:T716I,S:S982A,S:D1118H,S:G1219C	6	217
hCoV-19/Mexico/CMX-INMEGEN-06-03-65/2021	EPI_ISL_2810074	-	B.1.1.7	GRY	37	C241T,C913T,C2453T,C3037T,C3267T,C5388A,C5986T,T6954C,C9611T,C14408T,C14676T,C15279T,T16176C,C19343T,G21451A,C21855T,G21974C,A23063T,C23271A,A23403G,C23604A,C23709T,C23934T,T24506G,G24914C,C25886T,C27972T,G28048T,A28111G,G28280C,A28281T,T28282A,A28295G,G28881A,G28882A,G28883C,C28977T	27	N:D3L,N:N8D,N:R203K,N:G204R,N:S235F,ORF1a:L730F,ORF1a:T1001I,ORF1a:A1708D,ORF1a:I2230T,ORF1a:L3116F,ORF1b:P314L,ORF1b:A1959V,ORF1b:G2662S,ORF3a:S165F,ORF8:Q27*,ORF8:R52I,ORF8:Y73C,S:S98F,S:D138H,S:N501Y,S:A570D,S:D614G,S:P681H,S:T716I,S:T791I,S:S982A,S:D1118H	6	203
hCoV-19/Mexico/CMX-INMEGEN-06-04-107/2021	EPI_ISL_2894237	-	B.1.1.7	GRY	45	C241T,C913T,T1177C,T1537G,C2110T,G2528A,C3037T,C3267T,C4296T,C5142T,C5388A,C5986T,T6954C,G7042T,C10789T,C14120T,C14408T,C14676T,C15279T,T16176C,C17375T,A19950G,C20470A,A23063T,C23271A,A23403G,C23604A,C23709T,G23749A,T24506G,G24914C,C25452T,G25471T,C27972T,G28048T,A28095T,A28111G,G28280C,A28281T,T28282A,G28881A,G28882A,	27	N:D3L,N:R203K,N:G204R,N:S235F,ORF1a:E755K,ORF1a:T1001I,ORF1a:T1344I,ORF1a:T1626I,ORF1a:A1708D,ORF1a:I2230T,ORF1a:M2259I,ORF1b:P218L,ORF1b:P314L,ORF1b:T1303I,ORF1b:Q2335K,ORF3a:D27Y,ORF8:Q27*,ORF8:R52I,ORF8:K68*,ORF8:Y73C,S:N501Y,S:A570D,S:D614G,S:P681H,S:T716I,S:S982A,S:	6	0
hCoV-19/Mexico/CMX-INMEGEN-06-04-191/2021	EPI_ISL_2894261	-	B.1.1.7	GRY	37	C222T,C241T,C913T,C2110T,C3037T,C3267T,C5388A,C5986T,T6954C,G7042T,C14120T,C14408T,C14676T,C15279T,T16176C,C17762T,A23063T,C23271A,A23403G,C23604A,C23709T,T24506G,G24914C,T25180C,G25641T,C25672T,C27972T,G28048T,A28095T,A28111G,G28280C,A28281T,T28282A,G28881A,G28882A,G28883C,C28977T	24	N:D3L,N:R203K,N:G204R,N:S235F,ORF1a:T1001I,ORF1a:A1708D,ORF1a:I2230T,ORF1a:M2259I,ORF1b:P218L,ORF1b:P314L,ORF1b:A1432V,ORF3a:L83F,ORF3a:L94F,ORF8:Q27*,ORF8:R52I,ORF8:K68*,ORF8:Y73C,S:N501Y,S:A570D,S:D614G,S:P681H,S:T716I,S:S982A,S:D1118H	6	0
hCoV-19/Mexico/CMX-INMEGEN-06-04-208/2021	EPI_ISL_2894273	-	B.1.1.7	GRY	41	C241T,G376T,C913T,C3037T,C3267T,C5388A,C5944T,C5986T,T6954C,C9967T,C10868T,C13585T,C14408T,C14676T,T15096C,C15279T,T16176C,A17344G,G18412T,C22987T,A23063T,C23271A,A23403G,C23604A,C23709T,T24506G,G24914C,T25435C,C27972T,G28048T,A28095T,A28111G,G28280C,A28281T,T28282A,G28881A,G28882A,G28883C,G28884C,C28977T,A28984G	22	N:D3L,N:R203K,N:G204P,N:S235F,ORF1a:E37D,ORF1a:T1001I,ORF1a:A1708D,ORF1a:I2230T,ORF1b:P314L,ORF1b:I1293V,ORF1b:V1649F,ORF8:Q27*,ORF8:R52I,ORF8:K68*,ORF8:Y73C,S:N501Y,S:A570D,S:D614G,S:P681H,S:T716I,S:S982A,S:D1118H	6	0
hCoV-19/Mexico/CMX-INMEGEN-06-04-209/2021	EPI_ISL_2894274	-	B.1.1.7	GR	33	C241T,C913T,C2453T,C3037T,C3267T,C5388A,C5986T,T6954C,C9611T,C9893T,C14408T,C14676T,C15279T,T16176C,C20823T,C21855T,G21974C,C23271A,A23403G,C23604A,C23709T,T24506G,G24914C,C25509T,C27972T,G28048T,A28111G,T28282A,A28295G,G28881A,G28882A,G28	22	N:N8D,N:R203K,N:G204R,N:S235F,ORF1a:L730F,ORF1a:T1001I,ORF1a:A1708D,ORF1a:I2230T,ORF1a:L3116F,ORF1a:L3210F,ORF1b:P314L,ORF8:Q27*,ORF8:R52I,ORF8:Y73C,S:S98F,S:D138H,S:A570D,S:D614G,S:P681H,S:T716I,S:S982	6	2056
hCoV-19/Mexico/CMX-INMEGEN-06-04-64/2021	EPI_ISL_2894208	-	B.1.1.7	GR	32	C241T,C913T,C3037T,C3267T,C5388A,C5986T,C6196T,T6954C,A8848G,T11135C,C14408T,C14676T,C15279T,T16176C,C23271A,A23403G,C23604A,C23709T,G24095T,T24506G,G24914C,C27972T,G28048T,A28111G,G28280C,A28281T,T28282A,G28881A,G28882A,G28883C,C28977T,G	19	N:D3L,N:R203K,N:G204R,N:S235F,ORF1a:T1001I,ORF1a:A1708D,ORF1a:I2230T,ORF1a:F3624L,ORF1b:P314L,ORF8:Q27*,ORF8:R52I,ORF8:Y73C,S:A570D,S:D614G,S:P681H,S:T716I,S:A845S,S:S982A,S:D1118H	6	948
hCoV-19/Mexico/CMX-INMEGEN-06-04-66/2021	EPI_ISL_2894210	-	B.1.1.7	GRY	35	C241T,C913T,C3037T,C3267T,C5388A,C5986T,C6196T,T6954C,A8848G,A11983G,T13417C,C14408T,C14676T,C15279T,T16176C,A23063T,C23271A,A23403G,C23604A,C23709T,T24506G,G24914C,C27002T,C27434T,C27972T,G28048T,A28111G,G28280C,A28281T,T28282A,G28881A,G28882A,G28883C,C28977T,G29715T	19	N:D3L,N:R203K,N:G204R,N:S235F,ORF1a:T1001I,ORF1a:A1708D,ORF1a:I2230T,ORF1b:P314L,ORF7a:T14I,ORF8:Q27*,ORF8:R52I,ORF8:Y73C,S:N501Y,S:A570D,S:D614G,S:P681H,S:T716I,S:S982A,S:D1118H	6	274
hCoV-19/Mexico/CMX-INMEGEN-16-21/2021	EPI_ISL_2978376	-	B.1.1.7	GRY	36	C241T,C913T,C2110T,C3037T,C3267T,C5388A,C5986T,T6954C,G7042T,A10195G,C14120T,C14408T,C14676T,C15279T,C15933T,T16176C,C22450T,A23063T,C23271A,A23403G,C23604A,C23709T,T24506G,G24914C,A27745G,C27972T,G28048T,G28075T,A28111G,G28280C,A28281T,T28282A,G28881A,G28882A,G28883C,C28977T	22	N:D3L,N:R203K,N:G204R,N:S235F,ORF1a:T1001I,ORF1a:A1708D,ORF1a:I2230T,ORF1a:M2259I,ORF1b:P218L,ORF1b:P314L,ORF7a:R118G,ORF8:Q27*,ORF8:R52I,ORF8:C61F,ORF8:Y73C,S:N501Y,S:A570D,S:D614G,S:P681H,S:T716I,S:S982A,S:D1118H	6	238
hCoV-19/Mexico/CMX-INMEGEN-16-75/2021	EPI_ISL_2978423	-	B.1.1.7	GRY	43	C110T,C241T,C913T,C2110T,C3037T,C3267T,C3543T,C4900T,C5388A,C5986T,T6954C,G7042T,A10195G,C10341T,C14120T,C14408T,C14676T,C15108T,C15279T,T16176C,C16376T,C22792T,A23063T,C23271A,A23403G,C23604A,C23709T,T24506G,G24914C,C26645T,C27804T,C27972T,G28048T,G28075T,A28111G,G28280C,A28281T,T28282A,C28603T,G28881A,G28882A,G28883	24	N:D3L,N:R203K,N:G204R,N:S235F,ORF1a:T1001I,ORF1a:T1093I,ORF1a:A1708D,ORF1a:I2230T,ORF1a:M2259I,ORF1a:P3359L,ORF1b:P218L,ORF1b:P314L,ORF1b:P970L,ORF8:Q27*,ORF8:R52I,ORF8:C61F,ORF8:Y73C,S:N501Y,S:A570D,S:D614G,S:P681H,S:T716I,S:S982A,S:D1118H	6	19
hCoV-19/Mexico/CMX-INMEGEN-InDRE-04-04-99/2021	EPI_ISL_1628598	-	B.1.1.7	GRY	30	C241T,C913T,C3037T,C3267T,C5388A,C5986T,T6954C,C9298T,C14408T,C14676T,C15279T,T16176C,A23063T,C23271A,A23403G,C23604A,C23709T,C24374T,T24506G,G24914C,C27972T,G28048T,A28111G,G28280C,A28281T,T28282A,G28881A,G28882A,G28883C,C28977T	19	N:D3L,N:R203K,N:G204R,N:S235F,ORF1a:T1001I,ORF1a:A1708D,ORF1a:I2230T,ORF1b:P314L,ORF8:Q27*,ORF8:R52I,ORF8:Y73C,S:N501Y,S:A570D,S:D614G,S:P681H,S:T716I,S:L938F,S:S982A,S:D1118H	6	7
hCoV-19/Mexico/CMX-INMEGEN-InDRE-04-06-165/2021	EPI_ISL_1810898	-	B.1.1.7	GRY	41	C241T,C913T,C3037T,C3267T,C5388A,C5944T,C5986T,G6647A,T6954C,C14408T,C14676T,T15096C,C15279T,T16176C,C17676T,C18183T,C19524T,A21519C,G22217A,A23063T,C23271A,A23403G,C23604A,C23709T,A24070T,T24506G,G24914C,C25624T,T27322C,C27972T,G28048T,A28095T,A28111G,G28280C,A28281T,T28282A,G28881A,G28882A,G28883C,G28884C,C28977T	25	N:D3L,N:R203K,N:G204P,N:S235F,ORF1a:T1001I,ORF1a:A1708D,ORF1a:A2128T,ORF1a:I2230T,ORF1b:P314L,ORF1b:R2684S,ORF3a:H78Y,ORF6:S41P,ORF8:Q27*,ORF8:R52I,ORF8:K68*,ORF8:Y73C,S:G219S,S:N501Y,S:A570D,S:D614G,S:P681H,S:T716I,S:Q836H,S:S982A,S:D1118H	6	0
hCoV-19/Mexico/CMX-INMEGEN-InDRE-04-06-166/2021	EPI_ISL_1810899	-	B.1.1.7	GRY	40	C241T,C913T,C3037T,C3267T,C5388A,C5944T,C5986T,G6647A,T6954C,C14408T,C14676T,T15096C,C15279T,T16176C,C17676T,C18183T,C19524T,A21519C,G22217A,A23063T,C23271A,A23403G,C23604A,C23709T,A24070T,T24506G,G24914C,C25624T,C27972T,G28048T,A28095T,A28111G,G28280C,A28281T,T28282A,G28881A,G28882A,G28883C,G28884C,C28977T	24	N:D3L,N:R203K,N:G204P,N:S235F,ORF1a:T1001I,ORF1a:A1708D,ORF1a:A2128T,ORF1a:I2230T,ORF1b:P314L,ORF1b:R2684S,ORF3a:H78Y,ORF8:Q27*,ORF8:R52I,ORF8:K68*,ORF8:Y73C,S:G219S,S:N501Y,S:A570D,S:D614G,S:P681H,S:T716I,S:Q836H,S:S982A,S:D1118H	6	0
hCoV-19/Mexico/CMX-INMEGEN-InDRE-04-06-175/2021	EPI_ISL_1810908	-	B.1.1.7	GRY	40	C241T,C913T,C3037T,C3267T,C5388A,C5944T,C5986T,G6647A,T6954C,C14408T,C14676T,T15096C,C15279T,T16176C,C17676T,C18183T,C19524T,A21519C,G22217A,A23063T,C23271A,A23403G,C23604A,C23709T,A24070T,T24506G,G24914C,C25624T,C27972T,G28048T,A28095T,A28111G,G28280C,A28281T,T28282A,G28881A,G28882A,G28883C,G28884C,C28977T	24	N:D3L,N:R203K,N:G204P,N:S235F,ORF1a:T1001I,ORF1a:A1708D,ORF1a:A2128T,ORF1a:I2230T,ORF1b:P314L,ORF1b:R2684S,ORF3a:H78Y,ORF8:Q27*,ORF8:R52I,ORF8:K68*,ORF8:Y73C,S:G219S,S:N501Y,S:A570D,S:D614G,S:P681H,S:T716I,S:Q836H,S:S982A,S:D1118H	6	19
hCoV-19/Mexico/CMX-INMEGEN-InDRE-04-06-182/2021	EPI_ISL_1810915	-	B.1.1.7	GR	39	C241T,C913T,G1299A,C2110T,C2388T,C3037T,C3267T,C5388A,C5986T,T6954C,G7042T,A8976G,A10195G,C14120T,C14408T,C14676T,C15279T,T16176C,C23271A,A23403G,C23604A,C23709T,T24506G,G24914C,T25789A,C26201T,C27972T,G28048T,G28075T,A28095T,A28111G,G28280C,A28281T,T28282A,G28881A,G28882A,G28883C,C28977T,C29218T	26	N:D3L,N:R203K,N:G204R,N:S235F,ORF1a:G345D,ORF1a:T708I,ORF1a:T1001I,ORF1a:A1708D,ORF1a:I2230T,ORF1a:M2259I,ORF1a:E2904G,ORF1b:P218L,ORF1b:P314L,ORF3a:C133S,ORF3a:T270I,ORF8:Q27*,ORF8:R52I,ORF8:C61F,ORF8:K68*,ORF8:Y73C,S:A570D,S:D614G,S:P681H,S:T716I,S:S982A,S:D1118H	6	1576
hCoV-19/Mexico/CMX-INMEGEN-INDRE-05-03-195/2021	EPI_ISL_2319317	-	B.1.1.7	GR	30	C241T,C913T,C3037T,C3177T,C3267T,C3593A,C5388A,C5986T,C6936T,T6954C,C7420T,C14408T,C14676T,C15279T,A15668T,T16176C,C20844T,C23271A,A23403G,C23604A,C23709T,C24374T,T24506G,G24914C,G27463T,G28881A,G28882A,G28883C,C28977T,C29580T	19	N:R203K,N:G204R,N:S235F,ORF1a:P971L,ORF1a:T1001I,ORF1a:L1110I,ORF1a:A1708D,ORF1a:S2224F,ORF1a:I2230T,ORF1b:P314L,ORF1b:N734I,ORF7a:V24F,S:A570D,S:D614G,S:P681H,S:T716I,S:L938F,S:S982A,S:D1118H	6	1121
hCoV-19/Mexico/CMX-INMEGEN-INDRE-05-03-209/2021	EPI_ISL_2319330	-	B.1.1.7	G	28	C241T,C913T,C3037T,C3177T,C3267T,C3593A,C5388A,C5986T,C6936T,T6954C,C7420T,T11618G,C14408T,C14676T,C15279T,A15668T,T16176C,C20844T,C23271A,A23403G,C23604A,C23709T,C24374T,T24506G,G24914C,G27463T,G28167A,C28977T	19	N:S235F,ORF1a:P971L,ORF1a:T1001I,ORF1a:L1110I,ORF1a:A1708D,ORF1a:S2224F,ORF1a:I2230T,ORF1a:F3785V,ORF1b:P314L,ORF1b:N734I,ORF7a:V24F,ORF8:E92K,S:A570D,S:D614G,S:P681H,S:T716I,S:L938F,S:S982A,S:D1118H	6	1637
hCoV-19/Mexico/CMX-INMEGEN-INDRE-05-03-211/2021	EPI_ISL_2319331	-	B.1.1.7	GR	37	C222T,C241T,C913T,C2110T,C3037T,C3267T,C5388A,C5986T,T6954C,G7042T,C10189T,C14120T,C14408T,C14676T,C15279T,T16176C,C17762T,C22624T,C23271A,A23403G,C23604A,C23709T,T24506G,G24914C,T25180C,G26062T,C27972T,G28048T,A28095T,A28111G,G28280C,A28281T,T28282A,G28881A,G28882A,G28883C,C28977T	22	N:D3L,N:R203K,N:G204R,N:S235F,ORF1a:T1001I,ORF1a:A1708D,ORF1a:I2230T,ORF1a:M2259I,ORF1b:P218L,ORF1b:P314L,ORF1b:A1432V,ORF3a:G224C,ORF8:Q27*,ORF8:R52I,ORF8:K68*,ORF8:Y73C,S:A570D,S:D614G,S:P681H,S:T716I,S:S982A,S:D1118H	6	1147
hCoV-19/Mexico/CMX-INMEGEN-INDRE-05-03-212/2021	EPI_ISL_2319332	-	B.1.1.7	GRY	34	C241T,C913T,C3037T,C3267T,C5388A,C5986T,C6196T,T6954C,A8848G,C14408T,C14676T,C15279T,T16176C,T19624C,A23063T,C23271A,A23403G,C23604A,C23709T,T24506G,G24914C,G25217T,C25916T,C27972T,G28048T,A28111G,G28280C,A28281T,T28282A,G28881A,G28882A,G2	20	N:D3L,N:R203K,N:G204R,N:S235F,ORF1a:T1001I,ORF1a:A1708D,ORF1a:I2230T,ORF1b:P314L,ORF3a:T175I,ORF8:Q27*,ORF8:R52I,ORF8:Y73C,S:N501Y,S:A570D,S:D614G,S:P681H,S:T716I,S:S982A,S:D1118H,S:G1219C	6	0
hCoV-19/Mexico/CMX-INMEGEN-INDRE-05-03-233/2021	EPI_ISL_2319347	-	B.1.1.7	GRY	44	C241T,C913T,C3037T,C3267T,C5388A,C5944T,C5986T,G6647A,T6954C,C9996T,A10486G,C14408T,C14676T,T15096C,C15279T,T16176C,C17676T,C18183T,C19524T,A21519C,G22217A,A23063T,C23271A,A23403G,C23604A,C23709T,A24070T,T24506G,G24914C,C25624T,G26918T,T27384C,C27972T,G28048T,A28095T,A28111G,G28280C,A28281T,T28282A,G28881A,G28882A,G28	25	N:D3L,N:R203K,N:G204P,N:S235F,ORF1a:T1001I,ORF1a:A1708D,ORF1a:A2128T,ORF1a:I2230T,ORF1a:S3244L,ORF1b:P314L,ORF1b:R2684S,ORF3a:H78Y,ORF8:Q27*,ORF8:R52I,ORF8:K68*,ORF8:Y73C,S:G219S,S:N501Y,S:A570D,S:D614G,S:P681H,S:T716I,S:Q836H,S:S982A,S:D1118H	6	0

hCoV-19/Mexico/CMX-INMEGEN-INDRE-05-03-249/2021	EPI_ISL_2319359	-	B.1.1.7	GRY	34	C241T,C913T,C2453T,C3037T,C3267T,C5388A,C5986T,A6575T,T6954C,C9611T,C14408T,C14676T,C15279T,T16176C,C20930T,C21855T,G21974C,A23063T,C23271A,A23403G,C23604A,C23709T,T23877C,T24506G,G24914C,C27972T,G28048T,A28111G,T28282A,A28295G,G28881A,G28882A,G28883C,C28977T	25	N:N8D,N:R203K,N:G204R,N:S235F,ORF1a:L730F,ORF1a:T1001I,ORF1a:A1708D,ORF1a:S2104C,ORF1a:I2230T,ORF1a:L3116F,ORF1b:P314L,ORF1b:T2488M,ORF8:Q27*,ORF8:R52I,ORF8:Y73C,S:S98F,S:D138H,S:N501Y,S:A570D,S:D614G,S:P681H,S:T716I,S:V772A,S:S982A,S:D1118H	6	9
hCoV-19/Mexico/CMX-INMEGEN-INDRE-05-03-253/2021	EPI_ISL_2319363	-	B.1.1.7	G	32	C241T,C913T,C3037T,C3267T,C5388A,C5986T,T6954C,C7299T,A13768T,C14408T,C14676T,T15096C,C15279T,T16176C,C19862T,C23271A,A23403G,C23604A,C23709T,T24506G,G24914C,C26606T,G27281T,C27972T,G28048T,A28095T,A28111G,G28280C,A28281T,T28282A,C28977T,A29	20	N:D3L,N:S235F,ORF1a:T1001I,ORF1a:A1708D,ORF1a:I2230T,ORF1a:A2345V,ORF1b:M101L,ORF1b:P314L,ORF1b:A2132V,ORF6:W27L,ORF8:Q27*,ORF8:R52I,ORF8:K68*,ORF8:Y73C,S:A570D,S:D614G,S:P681H,S:T716I,S:S982A,S:D1118	6	970
hCoV-19/Mexico/CMX-INMEGEN-INDRE-05-03-271/2021	EPI_ISL_2319371	-	B.1.1.7	GRY	37	C241T,C878T,C913T,C3037T,C3267T,C5388A,C5986T,T6954C,A12162G,C12400T,C14408T,C14676T,C15279T,C15720T,T16176C,A17615G,A21137G,A23063T,C23271A,A23403G,C23604A,C23709T,G24095T,T24506G,G24914C,G25135T,G26998T,C27972T,G28048T,A28111G,G28280C,A28281T,T28282A,G28881A,G28882A,G28883C,C28977T	24	M:C159F,N:D3L,N:R203K,N:G204R,N:S235F,ORF1a:T1001I,ORF1a:A1708D,ORF1a:I2230T,ORF1a:Q3966R,ORF1b:P314L,ORF1b:K1383R,ORF1b:K2557R,ORF8:Q27*,ORF8:R52I,ORF8:Y73C,S:N501Y,S:A570D,S:D614G,S:P681H,S:T716I,S:A845S,S:S982A,S:D1118H,S:K1191N	6	0
hCoV-19/Mexico/CMX-INMEGEN-INDRE-05-03-275/2021	EPI_ISL_2319375	-	B.1.1.7	G	32	C241T,C913T,C2110T,C3037T,C3267T,C5388A,C5986T,T6954C,G7042T,C7834T,A10195G,C14120T,C14408T,C14676T,C15279T,T16176C,C16833T,C23271A,A23403G,C23604A,C23709T,T24506G,G24914C,C27972T,G28048T,G28075T,A28095T,A28111G,G28280C,A28281T,T28282A,C289	19	N:D3L,N:S235F,ORF1a:T1001I,ORF1a:A1708D,ORF1a:I2230T,ORF1a:M2259I,ORF1b:P218L,ORF1b:P314L,ORF8:Q27*,ORF8:R52I,ORF8:C61F,ORF8:K68*,ORF8:Y73C,S:A570D,S:D614G,S:P681H,S:T716I,S:S982A,S:D1118H	6	2273
hCoV-19/Mexico/COA-InDRE_FB13031_S1493/2021	EPI_ISL_1805471	-	B.1.1.7	G	30	C241T,C913T,C2453T,C3037T,C3267T,C5388A,C5986T,T6954C,C9611T,C14408T,C14676T,C15279T,T16176C,C21855T,G21974C,A23063T,C23271A,A23403G,C23604A,C23709T,T24506G,G24914C,C27972T,G28048T,A28111G,G28280C,A28281T,T28282A,A28295G,C28977T	21	N:D3L,N:N8D,N:S235F,ORF1a:L730F,ORF1a:T1001I,ORF1a:A1708D,ORF1a:I2230T,ORF1a:L3116F,ORF1b:P314L,ORF8:Q27*,ORF8:R52I,ORF8:Y73C,S:S98F,S:D138H,S:N501Y,S:A570D,S:D614G,S:P681H,S:T716I,S:S982A,S:D1118H	6	890
hCoV-19/Mexico/COA-InDRE_FB14127_S1762/2021	EPI_ISL_2101901	-	B.1.1.7	GRY	37	C241T,C913T,C2110T,G2534A,C3037T,C3267T,C5388A,C5986T,T6954C,G7042T,A10195G,C11020T,C14120T,C14408T,C14676T,C15279T,T16176C,A23063T,C23271A,A23403G,C23604A,C23709T,T24506G,G24914C,C26936T,C27972T,G28048T,G28075T,A28095T,A28111G,G28280C,A28281T,T28282A,G28881A,G28882A,G28883C,C28977T	23	N:D3L,N:R203K,N:G204R,N:S235F,ORF1a:V757I,ORF1a:T1001I,ORF1a:A1708D,ORF1a:I2230T,ORF1a:M2259I,ORF1b:P218L,ORF1b:P314L,ORF8:Q27*,ORF8:R52I,ORF8:C61F,ORF8:K68*,ORF8:Y73C,S:N501Y,S:A570D,S:D614G,S:P681H,S:T716I,S:S982A,S:D1118H	6	274
hCoV-19/Mexico/COA-InDRE_FB16336_S2737/2021	EPI_ISL_2533763	-	Q.3	GRY	38	C241T,C913T,C3037T,C3267T,G5023A,C5388A,C5986T,C6568T,C6848T,T6954C,A9529G,G10396T,C14408T,C14676T,C15279T,T16176C,A17615G,G20962A,G22094C,A23063T,C23271A,A23403G,C23604A,C23709T,T24506G,G24914C,C25433T,G26730C,C27972T,G28048T,A28111G,G28280C,A28281T,T28282A,G28881A,G28882A,G28883C,C28977T	25	M:V70L,N:D3L,N:R203K,N:G204R,N:S235F,ORF1a:T1001I,ORF1a:M1586I,ORF1a:A1708D,ORF1a:P2195S,ORF1a:I2230T,ORF1b:P314L,ORF1b:K1383R,ORF1b:D2499N,ORF3a:T14I,ORF8:Q27*,ORF8:R52I,ORF8:Y73C,S:D178H,S:N501Y,S:A570D,S:D614G,S:P681H,S:T716I,S:S982A,S:D1118H	6	0
hCoV-19/Mexico/COA-InDRE_FB16772_S2777/2021	EPI_ISL_2533803	-	Q.3	GRY	38	C241T,C913T,C3037T,C3267T,G5023A,C5388A,C5986T,C6568T,C6848T,T6954C,A9529G,G10396T,C14408T,C14676T,C15279T,T16176C,A17615G,G20962A,G22094C,A23063T,C23271A,A23403G,C23604A,C23709T,T24506G,G24914C,C25433T,G26730C,C27972T,G28048T,A28111G,G28280C,A28281T,T28282A,G28881A,G28882A,G28883C,C28977T	25	M:V70L,N:D3L,N:R203K,N:G204R,N:S235F,ORF1a:T1001I,ORF1a:M1586I,ORF1a:A1708D,ORF1a:P2195S,ORF1a:I2230T,ORF1b:P314L,ORF1b:K1383R,ORF1b:D2499N,ORF3a:T14I,ORF8:Q27*,ORF8:R52I,ORF8:Y73C,S:D178H,S:N501Y,S:A570D,S:D614G,S:P681H,S:T716I,S:S982A,S:D1118H	6	0
hCoV-19/Mexico/COA-InDRE_FB17411_S3519/2021	EPI_ISL_2779034	-	B.1.1.7	GRY	34	C241T,C913T,C3037T,C3267T,G5164T,C5388A,C5986T,T6954C,C7945T,C14408T,C14676T,C15279T,T16176C,A23063T,C23191T,C23271A,A23403G,C23604A,C23709T,T24506G,G24914C,G25494A,C25710T,C27294T,C27972T,G28048T,A28111G,G28280C,A28281T,T28282A,G28881A,G2	19	N:D3L,N:R203K,N:G204R,N:S235F,ORF1a:T1001I,ORF1a:E1633D,ORF1a:A1708D,ORF1a:I2230T,ORF1b:P314L,ORF8:Q27*,ORF8:R52I,ORF8:Y73C,S:N501Y,S:A570D,S:D614G,S:P681H,S:T716I,S:S982A,S:D1118H	6	0
hCoV-19/Mexico/COA-InDRE_FB17711_S4112/2021	EPI_ISL_2920683	-	B.1.1.7	GRY	36	C222T,C241T,C913T,C2110T,C3037T,C3267T,C5388A,C5986T,T6954C,G7042T,C10189T,C14120T,C14408T,C14676T,C15279T,T16176C,C17762T,C18348T,A23063T,C23271A,A23403G,C23604A,C23709T,T24506G,G24914C,T25180C,C27972T,G28048T,A28111G,G28280C,A28281T,T28282A,G28881A,G28882A,G28883C,C28977T	21	N:D3L,N:R203K,N:G204R,N:S235F,ORF1a:T1001I,ORF1a:A1708D,ORF1a:I2230T,ORF1a:M2259I,ORF1b:P218L,ORF1b:P314L,ORF1b:A1432V,ORF8:Q27*,ORF8:R52I,ORF8:Y73C,S:N501Y,S:A570D,S:D614G,S:P681H,S:T716I,S:S982A,S:D1118H	6	0
hCoV-19/Mexico/COA-InDRE_FB18231_S4134/2021	EPI_ISL_2920705	-	B.1.1.7	GRY	38	C222T,C241T,C913T,C2110T,C3037T,C3267T,C5388A,C5986T,T6954C,G7042T,C10189T,C14120T,C14408T,C14676T,C15279T,T16176C,C17762T,C18348T,A23063T,C23271A,T23284C,A23403G,C23604A,C23709T,T24506G,G24914C,T25180C,C27972T,G28048T,A28095T,A28111G,G28280C,A28281T,T28282A,G28881A,G28882A,G28883C,C28977T	22	N:D3L,N:R203K,N:G204R,N:S235F,ORF1a:T1001I,ORF1a:A1708D,ORF1a:I2230T,ORF1a:M2259I,ORF1b:P218L,ORF1b:P314L,ORF1b:A1432V,ORF8:Q27*,ORF8:R52I,ORF8:K68*,ORF8:Y73C,S:N501Y,S:A570D,S:D614G,S:P681H,S:T716I,S:S982A,S:D1118H	6	0
hCoV-19/Mexico/COL-InDRE_FB15039_S2055/2021	EPI_ISL_2283697	-	B.1.1.7	GRY	41	C241T,C673T,C913T,C3037T,C3267T,C5388A,C5986T,T6954C,A12162G,C13665T,C14408T,C14676T,C15279T,C15720T,T16176C,A17615G,C18747T,C20844T,A21137G,A23063T,C23271A,A23403G,C23604A,C23709T,T24506G,G24914C,G25135T,C26110T,C27972T,G28048T,A28111G,G28280C,A28281T,T28282A,G28881A,G28882A,G28883C,C28977T,C29095T,G29862T,A29866C	23	N:D3L,N:R203K,N:G204R,N:S235F,ORF1a:T1001I,ORF1a:A1708D,ORF1a:I2230T,ORF1a:Q3966R,ORF1b:P314L,ORF1b:K1383R,ORF1b:K2557R,ORF3a:P240S,ORF8:Q27*,ORF8:R52I,ORF8:Y73C,S:N501Y,S:A570D,S:D614G,S:P681H,S:T716I,S:S982A,S:D1118H,S:K1191N	6	0
hCoV-19/Mexico/COL-InDRE_FB16092_S2459/2021	EPI_ISL_2455980	-	B.1.1.7	GRY	42	C241T,C673T,C913T,C3037T,C3267T,C5388A,C5986T,T6954C,G9190T,A12162G,C13665T,C14408T,C14676T,C15279T,C15720T,T16176C,A17615G,C18747T,C20844T,A21137G,A23063T,C23271A,A23403G,C23604A,C23709T,T24506G,G24914C,G25135T,A25336C,C25578T,C26110T,C27972T,G28048T,A28111G,G28280C,A28281T,T28282A,G28881A,G28882A,G28883C,C28977T,C29	24	N:D3L,N:R203K,N:G204R,N:S235F,ORF1a:T1001I,ORF1a:A1708D,ORF1a:I2230T,ORF1a:Q3966R,ORF1b:P314L,ORF1b:K1383R,ORF1b:K2557R,ORF3a:P240S,ORF8:Q27*,ORF8:R52I,ORF8:Y73C,S:N501Y,S:A570D,S:D614G,S:P681H,S:T716I,S:S982A,S:D1118H,S:K1191N,S:E1258D	6	0
hCoV-19/Mexico/COL-InDRE_FB16093_S3078/2021	EPI_ISL_2663345	-	B.1.1.7	GRY	35	C241T,C913T,C3037T,C3267T,G5164T,C5388A,C5986T,T6954C,C7945T,C14408T,C14676T,C15279T,T16176C,A23063T,C23191T,C23271A,A23403G,C23604A,C23709T,T24506G,G24914C,G25494A,C25710T,C27213T,C27294T,C27972T,G28048T,A28111G,G28280C,A28281T,T28282A,G28881A,G28882A,G28883C,C28977T	19	N:D3L,N:R203K,N:G204R,N:S235F,ORF1a:T1001I,ORF1a:E1633D,ORF1a:A1708D,ORF1a:I2230T,ORF1b:P314L,ORF8:Q27*,ORF8:R52I,ORF8:Y73C,S:N501Y,S:A570D,S:D614G,S:P681H,S:T716I,S:S982A,S:D1118H	6	0
hCoV-19/Mexico/COL-InDRE_FB16402_S2480/2021	EPI_ISL_2456001	-	B.1.1.7	GRY	42	C241T,C673T,C913T,C1093T,C3037T,C3267T,C5388A,C5986T,T6954C,A12162G,C13665T,C14408T,C14676T,C15279T,C15720T,T16176C,A17615G,G17623T,C18747T,C20844T,A21137G,A23063T,C23271A,A23403G,C23604A,C23709T,T24506G,G24914C,G25135T,C25578T,C26110T,C27972T,G28048T,A28111G,G28280C,A28281T,T28282A,G28881A,G28882A,G28883C,C28977T,C29	24	N:D3L,N:R203K,N:G204R,N:S235F,ORF1a:T1001I,ORF1a:A1708D,ORF1a:I2230T,ORF1a:Q3966R,ORF1b:P314L,ORF1b:K1383R,ORF1b:A1386S,ORF1b:K2557R,ORF3a:P240S,ORF8:Q27*,ORF8:R52I,ORF8:Y73C,S:N501Y,S:A570D,S:D614G,S:P681H,S:T716I,S:S982A,S:D1118H,S:K1191N	6	0
hCoV-19/Mexico/COL-InDRE_FB17828_S4115/2021	EPI_ISL_2920686	-	B.1.1.7	GRY	43	C241T,C913T,G1836A,G2281A,C3037T,C3267T,C5388A,C5986T,T6954C,A12162G,C14408T,C14676T,C15279T,C15720T,T16176C,A17615G,C18698T,C18747T,C20844T,A21137G,A23063T,C23271A,A23403G,C23604A,C23709T,T24506G,G24914C,G25135T,A25336C,G25337C,C26110T,G26828T,C27972T,G28048T,A28111G,G28280C,A28281T,T28282A,G28881A,G28882A,G28883C,	27	N:D3L,N:R203K,N:G204R,N:S235F,ORF1a:G524D,ORF1a:T1001I,ORF1a:A1708D,ORF1a:I2230T,ORF1a:Q3966R,ORF1b:P314L,ORF1b:K1383R,ORF1b:A1744V,ORF1b:K2557R,ORF3a:P240S,ORF8:Q27*,ORF8:R52I,ORF8:Y73C,S:N501Y,S:A570D,S:D614G,S:P681H,S:T716I,S:S982A,S:D1118H,S:K1191N,S:E1258D,S:D12	6	0
hCoV-19/Mexico/DUR-InDRE_FB17381_S3478/2021	EPI_ISL_2778993	-	B.1.1.7	GRY	34	C241T,C245T,C913T,C3037T,C3267T,A3492G,C5388A,C5986T,T6954C,C14408T,C14676T,C15279T,T16176C,C21639A,A23063T,C23271A,A23403G,C23604A,C23709T,C24374T,T24506G,G24914C,A25336C,G25337C,C27972T,G28048T,A28111G,G28280C,A28281T,T28282A,G28881A,G2	23	N:D3L,N:R203K,N:G204R,N:S235F,ORF1a:T1001I,ORF1a:N1076S,ORF1a:A1708D,ORF1a:I2230T,ORF1b:P314L,ORF8:Q27*,ORF8:R52I,ORF8:Y73C,S:P26H,S:N501Y,S:A570D,S:D614G,S:P681H,S:T716I,S:L938F,S:S982A,S:D1118H,S:E1258	6	0
hCoV-19/Mexico/DUR-InDRE_FB17382_S3479/2021	EPI_ISL_2778994	-	B.1.1.7	GRY	34	C241T,C245T,C913T,C3037T,C3267T,A3492G,C5388A,C5986T,T6954C,C14408T,C14676T,C15279T,T16176C,C21639A,A23063T,C23271A,A23403G,C23604A,C23709T,C24374T,T24506G,G24914C,A25336C,G25337C,C27972T,G28048T,A28111G,G28280C,A28281T,T28282A,G28881A,G2	23	N:D3L,N:R203K,N:G204R,N:S235F,ORF1a:T1001I,ORF1a:N1076S,ORF1a:A1708D,ORF1a:I2230T,ORF1b:P314L,ORF8:Q27*,ORF8:R52I,ORF8:Y73C,S:P26H,S:N501Y,S:A570D,S:D614G,S:P681H,S:T716I,S:L938F,S:S982A,S:D1118H,S:E1258	6	0
hCoV-19/Mexico/DUR-InDRE_FB17386_S3481/2021	EPI_ISL_2778996	-	B.1.1.7	GRY	34	C241T,C245T,C913T,C3037T,C3267T,A3492G,C5388A,C5986T,T6954C,C14408T,C14676T,C15279T,T16176C,C21639A,A23063T,C23271A,A23403G,C23604A,C23709T,C24374T,T24506G,G24914C,A25336C,G25337C,C27972T,G28048T,A28111G,G28280C,A28281T,T28282A,G28881A,G2	23	N:D3L,N:R203K,N:G204R,N:S235F,ORF1a:T1001I,ORF1a:N1076S,ORF1a:A1708D,ORF1a:I2230T,ORF1b:P314L,ORF8:Q27*,ORF8:R52I,ORF8:Y73C,S:P26H,S:N501Y,S:A570D,S:D614G,S:P681H,S:T716I,S:L938F,S:S982A,S:D1118H,S:E1258	6	0
hCoV-19/Mexico/DUR-InDRE_FB17390_S3485/2021	EPI_ISL_2779000	-	B.1.1.7	GRY	34	C241T,C245T,C913T,C3037T,C3267T,A3492G,C5388A,C5986T,T6954C,C14408T,C14676T,C15279T,T16176C,C21639A,A23063T,C23271A,A23403G,C23604A,C23709T,C24374T,T24506G,G24914C,A25336C,G25337C,C27972T,G28048T,A28111G,G28280C,A28281T,T28282A,G28881A,G2	23	N:D3L,N:R203K,N:G204R,N:S235F,ORF1a:T1001I,ORF1a:N1076S,ORF1a:A1708D,ORF1a:I2230T,ORF1b:P314L,ORF8:Q27*,ORF8:R52I,ORF8:Y73C,S:P26H,S:N501Y,S:A570D,S:D614G,S:P681H,S:T716I,S:L938F,S:S982A,S:D1118H,S:E1258	6	0
hCoV-19/Mexico/GRO-InDRE_F48G_S568/2021	EPI_ISL_1334381	-	B.1.1.7	GRY	32	C241T,C913T,C3037T,C3267T,C5388A,C5986T,T6954C,C14408T,C14676T,C15279T,T16176C,A17615G,C18032T,A23063T,C23271A,A23403G,C23604A,C23709T,T24506G,G24914C,G26730C,C27972T,G28048T,A28111G,G28280C,A28281T,T28282A,C28603T,G28881A,G28882A,G28883C,	21	M:V70L,N:D3L,N:R203K,N:G204R,N:S235F,ORF1a:T1001I,ORF1a:A1708D,ORF1a:I2230T,ORF1b:P314L,ORF1b:K1383R,ORF1b:T1522I,ORF8:Q27*,ORF8:R52I,ORF8:Y73C,S:N501Y,S:A570D,S:D614G,S:P681H,S:T716I,S:S982A,S:D1118H	6	706

hCoV-19/Mexico/GUA-InDRE_F10833_S574/2021	EPI_ISL_1334386	-	B.1.1.7	GRY	35	C241T,C913T,C3037T,C3267T,C5388A,C5986T,T6954C,C9693T,C14408T,C14676T,C15279T,T16176C,C22716A,T22717C,T23042C,A23063T,C23271A,A23403G,C23604A,C23709T,T24506G,G24914C,G25218T,C25553T,C27615T,C27972T,G28048T,A28111G,G28280C,A28281T,T28282A,G28881A,G28882A,G28883C,C28977T	23	N:D3L,N:R203K,N:G204R,N:S235F,ORF1a:T1001I,ORF1a:A1708D,ORF1a:I2230T,ORF1a:A3143V,ORF1b:P314L,ORF3a:A54V,ORF8:Q27*,ORF8:R52I,ORF8:Y73C,S:T385N,S:S494P,S:N501Y,S:A570D,S:D614G,S:P681H,S:T716I,S:S982A,S:D1118H,S:G1219V	6	147
hCoV-19/Mexico/GUA-InDRE_F12716_S1202/2021	EPI_ISL_1626809	-	B.1.1.7	GRY	36	C241T,C913T,C2110T,C2841T,C3037T,C3267T,C5388A,C5986T,T6954C,G7042T,C14120T,C14408T,C14676T,C15279T,T16176C,T17583G,A23063T,C23271A,A23403G,C23604A,C23709T,T24506G,G24914C,A25336C,G25337C,C25624T,C27972T,G28048T,A28111G,G28280C,A28281T,T28282A,G28881A,G28882A,G28883C,C28977T	24	N:D3L,N:R203K,N:G204R,N:S235F,ORF1a:A859V,ORF1a:T1001I,ORF1a:A1708D,ORF1a:I2230T,ORF1a:M2259I,ORF1b:P218L,ORF1b:P314L,ORF3a:H78Y,ORF8:Q27*,ORF8:R52I,ORF8:Y73C,S:N501Y,S:A570D,S:D614G,S:P681H,S:T716I,S:S982A,S:D1118H,S:E1258D,S:D1259H	6	0
hCoV-19/Mexico/GUA-InDRE_FB13877_S2078/2021	EPI_ISL_2283718	-	B.1.1.7	GRY	34	C241T,C913T,C2453T,C3037T,C3267T,C5388A,C5986T,T6954C,C9611T,C14408T,C14676T,C15279T,T16176C,C21855T,G21974C,A23063T,C23271A,A23403G,C23604A,C23709T,T24506G,G24914C,C27972T,G28048T,A28111G,G28280C,A28281T,T28282A,A28295G,C28737T,G28881A,G28882A,G28883C,C28977T	24	N:D3L,N:N8D,N:A155V,N:R203K,N:G204R,N:S235F,ORF1a:L730F,ORF1a:T1001I,ORF1a:A1708D,ORF1a:I2230T,ORF1a:L3116F,ORF1b:P314L,ORF8:Q27*,ORF8:R52I,ORF8:Y73C,S:S98F,S:D138H,S:N501Y,S:A570D,S:D614G,S:P681H,S:T716I,S:S982A,S:D1118H	6	411
hCoV-19/Mexico/GUA-InDRE_FB13881_S1754/2021	EPI_ISL_2101893	-	B.1.1.7	GRY	35	C241T,G376T,C913T,C2453T,C3037T,C3267T,C5388A,C5986T,T6954C,C9611T,C14408T,C14676T,C15279T,T16176C,C21855T,G21974C,A23063T,C23271A,A23403G,C23604A,C23709T,T24506G,G24914C,G25273T,C27972T,G28048T,A28111G,G28280C,A28281T,T28282A,A28295G,G28881A,G28882A,G28883C,C28977T	25	N:D3L,N:N8D,N:R203K,N:G204R,N:S235F,ORF1a:E37D,ORF1a:L730F,ORF1a:T1001I,ORF1a:A1708D,ORF1a:I2230T,ORF1a:L3116F,ORF1b:P314L,ORF8:Q27*,ORF8:R52I,ORF8:Y73C,S:S98F,S:D138H,S:N501Y,S:A570D,S:D614G,S:P681H,S:T716I,S:S982A,S:D1118H,S:M1237I	6	0
hCoV-19/Mexico/GUA-InDRE_FB13904_S2077/2021	EPI_ISL_2283717	-	B.1.1.7	GRY	34	C241T,C913T,C2453T,G2494T,C3037T,C3267T,C5388A,C5986T,T6954C,C9611T,C14408T,C14676T,C15279T,T16176C,C21855T,G21974C,A23063T,C23271A,A23403G,C23604A,C23709T,T24506G,G24914C,C27972T,G28048T,A28111G,G28280C,A28281T,T28282A,A28295G,G28881A,G28882A,G28883C,C28977T	24	N:D3L,N:N8D,N:R203K,N:G204R,N:S235F,ORF1a:L730F,ORF1a:E743D,ORF1a:T1001I,ORF1a:A1708D,ORF1a:I2230T,ORF1a:L3116F,ORF1b:P314L,ORF8:Q27*,ORF8:R52I,ORF8:Y73C,S:S98F,S:D138H,S:N501Y,S:A570D,S:D614G,S:P681H,S:T716I,S:S982A,S:D1118H	6	0
hCoV-19/Mexico/GUA-InDRE_FB13907_S1755/2021	EPI_ISL_2101894	-	B.1.1.7	GRY	34	C241T,C913T,C2453T,C3037T,C3267T,C5388A,C5986T,T6954C,C9611T,C14408T,C14676T,C15279T,T16176C,C21855T,G21974C,A23063T,C23271A,A23403G,C23604A,C23709T,T24506G,G24914C,C25886T,C27972T,G28048T,A28111G,G28280C,A28281T,T28282A,A28295G,G28881A,G28882A,G28883C,C28977T	24	N:D3L,N:N8D,N:R203K,N:G204R,N:S235F,ORF1a:L730F,ORF1a:T1001I,ORF1a:A1708D,ORF1a:I2230T,ORF1a:L3116F,ORF1b:P314L,ORF3a:S165F,ORF8:Q27*,ORF8:R52I,ORF8:Y73C,S:S98F,S:D138H,S:N501Y,S:A570D,S:D614G,S:P681H,S:T716I,S:S982A,S:D1118H	6	0
hCoV-19/Mexico/GUA-InDRE_FB14636_S2081/2021	EPI_ISL_2295590	-	B.1.1.7	GRY	40	C241T,C913T,C3037T,C3267T,C5388A,C5986T,T6954C,G7936T,G11634T,A11847G,A12162G,C14408T,C14676T,C15279T,C15720T,T16176C,A17615G,A21137G,C21618T,A23063T,C23271A,A23403G,C23604A,C23709T,T24506G,G24914C,G25135T,A25336C,C25613T,C26110T,C27972T,G28048T,A28111G,G28280C,A28281T,T28282A,G28881A,G28882A,G28883C,C28977T	28	N:D3L,N:R203K,N:G204R,N:S235F,ORF1a:T1001I,ORF1a:A1708D,ORF1a:I2230T,ORF1a:C3790F,ORF1a:K3861R,ORF1a:Q3966R,ORF1b:P314L,ORF1b:K1383R,ORF1b:K2557R,ORF3a:S74F,ORF3a:P240S,ORF8:Q27*,ORF8:R52I,ORF8:Y73C,S:T19I,S:N501Y,S:A570D,S:D614G,S:P681H,S:T716I,S:S982A,S:D1118H,S:K119	12	0
hCoV-19/Mexico/GUA-InDRE_FB14800_S2082/2021	EPI_ISL_2283721	-	B.1.1.7	GRY	34	C241T,C913T,C3037T,C3267T,C3653T,A5311C,C5388A,C5986T,C6196T,T6954C,A8848G,C14408T,C14676T,C15279T,T16176C,A23063T,C23271A,A23403G,C23604A,C23709T,T24506G,G24914C,G27754T,C27972T,G28048T,A28111G,G28280C,A28281T,T28282A,G28881A,G28882A,G288	20	N:D3L,N:R203K,N:G204R,N:S235F,ORF1a:T1001I,ORF1a:L1130F,ORF1a:A1708D,ORF1a:I2230T,ORF1b:P314L,ORF7a:E121*,ORF8:Q27*,ORF8:R52I,ORF8:Y73C,S:N501Y,S:A570D,S:D614G,S:P681H,S:T716I,S:S982A,S:D1118H	6	0
hCoV-19/Mexico/GUA-InDRE_FB14824_S2083/2021	EPI_ISL_2283722	-	B.1.1.7	GRY	36	C241T,C913T,C1441T,C2110T,C2676T,C3037T,C3267T,G3620A,C5388A,C5986T,T6954C,G7042T,T10264A,C14120T,C14408T,C14676T,C15279T,T16176C,T21534C,C21750T,A23063T,C23271A,A23403G,C23604A,C23709T,G24415T,G24914C,C27697T,C27737T,C27972T,G28048T,A28111G,G28881A,G28882A,G28883C,C28977T	23	N:R203K,N:G204R,N:S235F,ORF1a:P804L,ORF1a:T1001I,ORF1a:G1119S,ORF1a:A1708D,ORF1a:I2230T,ORF1a:M2259I,ORF1b:P218L,ORF1b:P314L,ORF7a:L102F,ORF7a:T115I,ORF8:Q27*,ORF8:R52I,ORF8:Y73C,S:T63I,S:N501Y,S:A570D,S:D614G,S:P681H,S:T716I,S:D1118H	6	0
hCoV-19/Mexico/GUA-InDRE_FB14826_S2470/2021	EPI_ISL_2455991	-	B.1.1.7	GRY	36	C241T,C913T,C3037T,C3267T,C4115T,C5388A,C5986T,C6196T,T6954C,A8848G,C14408T,C14676T,C15279T,T16176C,C19154T,A23063T,C23271A,A23403G,C23604A,C23709T,T24506G,G24914C,A25336C,G27754T,C27972T,G28048T,A28111G,G28280C,A28281T,T28282A,G28881A,G28882A,G28883C,C28977T,G29294A,G29715T	23	N:D3L,N:R203K,N:G204R,N:S235F,N:D341N,ORF1a:T1001I,ORF1a:P1284S,ORF1a:A1708D,ORF1a:I2230T,ORF1b:P314L,ORF1b:T1896I,ORF7a:E121*,ORF8:Q27*,ORF8:R52I,ORF8:Y73C,S:N501Y,S:A570D,S:D614G,S:P681H,S:T716I,S:S982A,S:D1118H,S:E1258D	6	0
hCoV-19/Mexico/GUA-InDRE_FB14832_S3059/2021	EPI_ISL_2663326	-	B.1.1.7	GRY	35	T78G,C241T,C913T,C2453T,C3037T,C3267T,C5388A,C5986T,T6954C,C9611T,C14408T,C14676T,C15279T,T16176C,G17572T,C21855T,G21974C,A23063T,C23271A,A23403G,C23604A,C23709T,T24506G,G24914C,C27972T,G28048T,A28111G,G28280C,A28281T,T28282A,A28295G,G28881A,G28882A,G28883C,C28977T	24	N:D3L,N:N8D,N:R203K,N:G204R,N:S235F,ORF1a:L730F,ORF1a:T1001I,ORF1a:A1708D,ORF1a:I2230T,ORF1a:L3116F,ORF1b:P314L,ORF1b:A1369S,ORF8:Q27*,ORF8:R52I,ORF8:Y73C,S:S98F,S:D138H,S:N501Y,S:A570D,S:D614G,S:P681H,S:T716I,S:S982A,S:D1118H	6	1064
hCoV-19/Mexico/GUA-InDRE_FB14839_S2469/2021	EPI_ISL_2455990	-	B.1.1.7	GRY	38	C241T,C913T,C3037T,C3267T,C4115T,C5388A,C5986T,C6196T,T6372C,T6954C,A8848G,C14408T,C14676T,C15279T,T16176C,T19965G,G22927T,A23063T,C23271A,A23403G,C23604A,C23709T,T24506G,G24914C,A25336C,G27754T,C27972T,G28048T,A28111G,G28280C,A28281T,T28282A,G28881A,G28882A,G28883C,C28977T,G29294A,G29715T	25	N:D3L,N:R203K,N:G204R,N:S235F,N:D341N,ORF1a:T1001I,ORF1a:P1284S,ORF1a:A1708D,ORF1a:M2036T,ORF1a:I2230T,ORF1b:P314L,ORF1b:I2166M,ORF7a:E121*,ORF8:Q27*,ORF8:R52I,ORF8:Y73C,S:L455F,S:N501Y,S:A570D,S:D614G,S:P681H,S:T716I,S:S982A,S:D1118H,S:E1258D	6	0
hCoV-19/Mexico/GUA-InDRE_FB16209_S2465/2021	EPI_ISL_2455986	-	B.1.1.7	GRY	42	C241T,C913T,C2334T,C3037T,C3267T,C5388A,C5986T,T6954C,A12162G,C13665T,C14408T,C14676T,C15279T,C15720T,T16176C,A17615G,C18747T,C20320T,C20404T,A21137G,A23063T,C23271A,A23403G,C23604A,C23709T,T24506G,G24914C,G25135T,G25218T,A25336C,C26110T,C27972T,G28048T,A28111G,G28280C,A28281T,T28282A,G28881A,G28882A,G28883C,C28977T,C29095T	28	N:D3L,N:R203K,N:G204R,N:S235F,ORF1a:A690V,ORF1a:T1001I,ORF1a:A1708D,ORF1a:I2230T,ORF1a:Q3966R,ORF1b:P314L,ORF1b:K1383R,ORF1b:H2285Y,ORF1b:P2313S,ORF1b:K2557R,ORF3a:P240S,ORF8:Q27*,ORF8:R52I,ORF8:Y73C,S:N501Y,S:A570D,S:D614G,S:P681H,S:T716I,S:S982A,S:D1118H,S:K1191N,S:G1219V,S:E1258D	6	0
hCoV-19/Mexico/GUA-InDRE_FB16216_S2466/2021	EPI_ISL_2455987	-	B.1.1.7	GRY	40	C241T,G376T,C913T,C2453T,C3037T,C3267T,C3688T,C5388A,C5770T,C5986T,T6954C,C7604T,C9611T,C14408T,C14676T,C15279T,T16176C,C20233T,C21855T,G21974C,A23063T,C23271A,A23403G,C23604A,C23709T,T24506G,G24914C,G25273T,A25336C,C27972T,G28048T,A28111G,G28280C,A28281T,T28282A,A28295G,G28881A,G28882A,G28883C,C28977T	27	N:D3L,N:N8D,N:R203K,N:G204R,N:S235F,ORF1a:E37D,ORF1a:L730F,ORF1a:T1001I,ORF1a:A1708D,ORF1a:I2230T,ORF1a:L3116F,ORF1b:P314L,ORF1b:P2256S,ORF8:Q27*,ORF8:R52I,ORF8:Y73C,S:S98F,S:D138H,S:N501Y,S:A570D,S:D614G,S:P681H,S:T716I,S:S982A,S:D1118H,S:M1237I,S:E1258D	6	54
hCoV-19/Mexico/GUA-InDRE_FB16219_S2467/2021	EPI_ISL_2455988	-	B.1.1.7	GRY	36	C241T,C913T,C2453T,C3037T,C3267T,C5388A,C5986T,T6954C,C9611T,C14408T,C14676T,C15279T,T16176C,C16989T,C21855T,G21974C,A23063T,C23271A,A23403G,C23604A,C23709T,T24506G,G24914C,G25135T,A25336C,C27972T,G28048T,A28111G,G28280C,A28281T,T28282A,A28295G,G28881A,G28882A,G28883C,C28977T	25	N:D3L,N:N8D,N:R203K,N:G204R,N:S235F,ORF1a:L730F,ORF1a:T1001I,ORF1a:A1708D,ORF1a:I2230T,ORF1a:L3116F,ORF1b:P314L,ORF8:Q27*,ORF8:R52I,ORF8:Y73C,S:S98F,S:D138H,S:N501Y,S:A570D,S:D614G,S:P681H,S:T716I,S:S982A,S:D1118H,S:K1191N,S:E1258D	6	0
hCoV-19/Mexico/GUA-InDRE_FB16459_S3475/2021	EPI_ISL_2778990	-	B.1.1.7	GRY	38	C241T,C913T,C2453T,C3037T,C3267T,C5388A,C5986T,T6954C,C9611T,C14408T,C14676T,C15279T,T16176C,G17572T,G19518T,C21855T,G21974C,A23063T,C23271A,A23403G,C23604A,C23709T,T24506G,G24914C,C25046T,A25336C,G26056T,C27972T,G28048T,A28111G,G28280C,A28281T,T28282A,A28295G,G28881A,G28882A,G28883C,C28977T	28	N:D3L,N:N8D,N:R203K,N:G204R,N:S235F,ORF1a:L730F,ORF1a:T1001I,ORF1a:A1708D,ORF1a:I2230T,ORF1a:L3116F,ORF1b:P314L,ORF1b:A1369S,ORF1b:L2017F,ORF3a:D222Y,ORF8:Q27*,ORF8:R52I,ORF8:Y73C,S:S98F,S:D138H,S:N501Y,S:A570D,S:D614G,S:P681H,S:T716I,S:S982A,S:D1118H,S:P1162S,S:E1258D	6	0
hCoV-19/Mexico/GUA-InDRE_FB16460_S3779/2021	EPI_ISL_2779281	-	B.1.1.7	GRY	40	C241T,G376T,C913T,C2453T,C3037T,C3267T,C5388A,C5770T,C5986T,T6954C,C7604T,C9611T,C14408T,C14676T,C15279T,T16176C,C20233T,C21855T,G21974C,A23063T,C23271A,A23403G,C23604A,C23709T,T24506G,G24914C,G25273T,A25336C,G25337C,C27972T,G28048T,A28111G,G28280C,A28281T,T28282A,A28295G,G28881A,G28882A,G28883C,C28977T	28	N:D3L,N:N8D,N:R203K,N:G204R,N:S235F,ORF1a:E37D,ORF1a:L730F,ORF1a:T1001I,ORF1a:A1708D,ORF1a:I2230T,ORF1a:L3116F,ORF1b:P314L,ORF1b:P2256S,ORF8:Q27*,ORF8:R52I,ORF8:Y73C,S:S98F,S:D138H,S:N501Y,S:A570D,S:D614G,S:P681H,S:T716I,S:S982A,S:D1118H,S:M1237I,S:E1258D,S:D1259H	6	71
hCoV-19/Mexico/GUA-InDRE_FB16463_S3476/2021	EPI_ISL_2778991	-	B.1.1.7	GRY	39	C241T,G376T,C913T,C2094T,C2453T,C3037T,C3267T,C5388A,C5986T,G6419T,C6472A,T6954C,C9611T,C14408T,C14676T,C15279T,T16176C,C21855T,A23063T,C23271A,A23403G,C23604A,C23709T,T24506G,G24914C,G25273T,A25336C,G25337C,C27972T,G28048T,A28111G,G28280C,A28281T,T28282A,A28295G,G28881A,G28882A,G28883C,C28977T	28	N:D3L,N:N8D,N:R203K,N:G204R,N:S235F,ORF1a:E37D,ORF1a:S610L,ORF1a:L730F,ORF1a:T1001I,ORF1a:A1708D,ORF1a:V2052L,ORF1a:I2230T,ORF1a:L3116F,ORF1b:P314L,ORF8:Q27*,ORF8:R52I,ORF8:Y73C,S:S98F,S:N501Y,S:A570D,S:D614G,S:P681H,S:T716I,S:S982A,S:D1118H,S:M1237I,S:E1258D,S:D1259H	6	0
hCoV-19/Mexico/GUA-InDRE_FB16465_S3477/2021	EPI_ISL_2778992	-	B.1.1.7	GRY	33	C241T,C913T,C3037T,C3267T,C5388A,C5986T,C6196T,T6954C,A8848G,C13176T,C14408T,C14676T,C15279T,T16176C,A23063T,C23271A,A23403G,C23604A,C23709T,T24506G,G24914C,G27754T,C27972T,G28048T,A28111G,G28280C,A28281T,T28282A,G28881A,G28882A,G28883C,C2	20	N:D3L,N:R203K,N:G204R,N:S235F,ORF1a:T1001I,ORF1a:A1708D,ORF1a:I2230T,ORF1a:T4304I,ORF1b:P314L,ORF7a:E121*,ORF8:Q27*,ORF8:R52I,ORF8:Y73C,S:N501Y,S:A570D,S:D614G,S:P681H,S:T716I,S:S982A,S:D1118H	6	671

hCoV-19/Mexico/GUA-InDRE_FB16466_S3504/2021	EPI_ISL_2779019	-	B.1.1.7	GRY	35	C241T,C913T,C3037T,C3267T,C5388A,C5986T,T6954C,A8848G,C13176T,C14408T,C14676T,C15279T,T16176C,A23063T,C23271A,A23403G,C23604A,C23709T,T24506G,G24914C,A25336C,G25337C,G27754T,C27972T,G28048T,A28111G,G28280C,A28281T,T28282A,G28881A,G28882A,G28883C,C28977T,C29296T,G29715T	22	N:D3L,N:R203K,N:G204R,N:S235F,ORF1a:T1001I,ORF1a:A1708D,ORF1a:I2230T,ORF1a:T4304I,ORF1b:P314L,ORF7a:E121*,ORF8:Q27*,ORF8:R52I,ORF8:Y73C,S:N501Y,S:A570D,S:D614G,S:P681H,S:T716I,S:S982A,S:D1118H,S:E1258D,S:D1259H	6	63
hCoV-19/Mexico/GUA-InDRE_FB16891_S3116/2021	EPI_ISL_2663383	-	B.1.1.7	GRY	36	C241T,C913T,G2272T,C3037T,C3267T,C5388A,C5986T,C6196T,A6350G,T6954C,A8848G,C14408T,C14676T,C15279T,T16176C,A23063T,C23271A,A23403G,C23604A,C23709T,T24506G,G24914C,G25217T,G26727T,T27570C,C27972T,G28048T,A28111G,G28280C,A28281T,T28282A,G28881A,G28882A,G28883C,C28977T,G29715T	22	M:A69S,N:D3L,N:R203K,N:G204R,N:S235F,ORF1a:K669N,ORF1a:T1001I,ORF1a:A1708D,ORF1a:K2029E,ORF1a:I2230T,ORF1b:P314L,ORF8:Q27*,ORF8:R52I,ORF8:Y73C,S:N501Y,S:A570D,S:D614G,S:P681H,S:T716I,S:S982A,S:D1118H,S:G1219C	6	0
hCoV-19/Mexico/GUA-InDRE_FB16894_S3117/2021	EPI_ISL_2663384	-	B.1.1.7	GRY	39	C241T,C913T,C2110T,C3037T,C3267T,C5388A,C5986T,T6954C,G7042T,A10195G,C14120T,C14408T,C14676T,C15279T,T16176C,G18379A,C21575T,A23063T,C23271A,A23403G,C23604A,C23709T,T24506G,G24914C,G25987A,G27604A,T27645C,C27972T,G28048T,G28075T,A28095T,A28111G,G28280C,A28281T,T28282A,G28881A,G28882A,G28883C,C28977T	26	N:D3L,N:R203K,N:G204R,N:S235F,ORF1a:T1001I,ORF1a:A1708D,ORF1a:I2230T,ORF1a:M2259I,ORF1b:P218L,ORF1b:P314L,ORF1b:G1638S,ORF3a:D199N,ORF7a:V71I,ORF8:Q27*,ORF8:R52I,ORF8:C61F,ORF8:K68*,ORF8:Y73C,S:L5F,S:N501Y,S:A570D,S:D614G,S:P681H,S:T716I,S:S982A,S:D1118H	6	0
hCoV-19/Mexico/GUA-InDRE_FB16899_S3118/2021	EPI_ISL_2663385	-	B.1.1.7	GRY	37	C241T,G376T,C913T,A1260T,C2094T,C2453T,C3037T,C3267T,C5388A,C5986T,T6954C,C9611T,C14408T,C14676T,C15279T,T16176C,C21855T,G21974C,A23063T,C23271A,A23403G,C23604A,C23709T,T24506G,G24914C,G25273T,C27972T,G28048T,A28111G,G28280C,A28281T,T28282A,A28295G,G28881A,G28882A,G28883C,C28977T	27	N:D3L,N:N8D,N:R203K,N:G204R,N:S235F,ORF1a:E37D,ORF1a:Q332L,ORF1a:S610L,ORF1a:L730F,ORF1a:T1001I,ORF1a:A1708D,ORF1a:I2230T,ORF1a:L3116F,ORF1b:P314L,ORF8:Q27*,ORF8:R52I,ORF8:Y73C,S:S98F,S:D138H,S:N501Y,S:A570D,S:D614G,S:P681H,S:T716I,S:S982A,S:D1118H,S:M1237I	6	0
hCoV-19/Mexico/GUA-InDRE_FB16904_S3119/2021	EPI_ISL_2663386	-	B.1.1.7	GRY	36	C241T,C913T,C3037T,C3267T,C4878T,C5388A,C5986T,T6954C,G11083T,C14408T,C14676T,C15279T,T16176C,G19075A,C20436T,A23063T,C23271A,A23403G,C23604A,C23709T,T24506G,G24914C,C27005T,C27972T,G28048T,G28077A,A28111G,G28280C,A28281T,T28282A,G28881A,G28882A,G28883C,C28896T,C28977T,C29095T	23	N:D3L,N:R203K,N:G204R,N:A208V,N:S235F,ORF1a:T1001I,ORF1a:T1538I,ORF1a:A1708D,ORF1a:I2230T,ORF1a:L3606F,ORF1b:P314L,ORF1b:V1870I,ORF8:Q27*,ORF8:R52I,ORF8:V62M,ORF8:Y73C,S:N501Y,S:A570D,S:D614G,S:P681H,S:T716I,S:S982A,S:D1118H	6	87
hCoV-19/Mexico/GUA-InDRE_FB16908_S3120/2021	EPI_ISL_2663387	-	B.1.1.7	GRY	34	C241T,C913T,C3037T,C3267T,T3537C,C5388A,C5986T,T6954C,C9298T,C14408T,C14676T,C15279T,T16176C,A23063T,C23271A,T23371A,A23403G,C23604A,C23709T,C23758T,C24374T,T24506G,G24914C,C27972T,G28048T,A28111G,G28280C,A28281T,T28282A,G28881A,G28882A,G2	21	N:D3L,N:R203K,N:G204R,N:S235F,ORF1a:T1001I,ORF1a:I1091T,ORF1a:A1708D,ORF1a:I2230T,ORF1b:P314L,ORF8:Q27*,ORF8:R52I,ORF8:Y73C,S:N501Y,S:A570D,S:N603K,S:D614G,S:P681H,S:T716I,S:L938F,S:S982A,S:D1118H	6	0
hCoV-19/Mexico/GUA-InDRE_FB16911_S3121/2021	EPI_ISL_2663388	-	B.1.1.7	GRY	36	C241T,C913T,C2003T,C3037T,C3267T,C5388A,C5986T,C6196T,T6954C,A8848G,C13176T,C14408T,C14676T,C15279T,T16176C,C21736T,A23063T,C23271A,A23403G,C23604A,C23709T,T24506G,G24914C,G26062T,G27754T,C27972T,G28048T,A28111G,G28280C,A28281T,T28282A,G28881A,G28882A,G28883C,C28977T,G29715T	22	N:D3L,N:R203K,N:G204R,N:S235F,ORF1a:L580F,ORF1a:T1001I,ORF1a:A1708D,ORF1a:I2230T,ORF1a:T4304I,ORF1b:P314L,ORF3a:G224C,ORF7a:E121*,ORF8:Q27*,ORF8:R52I,ORF8:Y73C,S:N501Y,S:A570D,S:D614G,S:P681H,S:T716I,S:S982A,S:D1118H	6	0
hCoV-19/Mexico/GUA-InDRE_FB17325_S3532/2021	EPI_ISL_2779047	-	B.1.1.7	GRY	40	C241T,C913T,C2453T,C3037T,C3267T,T5152C,C5388A,C5986T,T6954C,C9611T,C14408T,C14676T,C15279T,T16176C,C21855T,G21974C,A23063T,C23271A,A23403G,C23604A,C23709T,T24506G,G24914C,G25273T,A25336C,T25711C,C26882T,C27972T,G27996A,G28048T,A28111G,G28280C,A28281T,T28282A,A28295G,G28881A,G28882A,G28883C,C28977T,C29750T	27	N:D3L,N:N8D,N:R203K,N:G204R,N:S235F,ORF1a:L730F,ORF1a:T1001I,ORF1a:A1708D,ORF1a:I2230T,ORF1a:L3116F,ORF1b:P314L,ORF3a:Y107H,ORF8:Q27*,ORF8:D35N,ORF8:R52I,ORF8:Y73C,S:S98F,S:D138H,S:N501Y,S:A570D,S:D614G,S:P681H,S:T716I,S:S982A,S:D1118H,S:M1237I,S:E1258D	6	0
hCoV-19/Mexico/GUA-InDRE_FB17346_S3533/2021	EPI_ISL_2779048	-	B.1.1.7	GRY	33	C241T,C913T,C2110T,C3037T,C3267T,C5388A,C5986T,T6954C,G7745A,C14120T,C14408T,C14676T,C15279T,T16176C,C21855T,A23063T,C23271A,A23403G,C23604A,C23709T,T24506G,G24914C,C27972T,G28048T,A28111G,G28280C,A28281T,T28282A,G28881A,G28882A,G28883C,C2	21	N:D3L,N:R203K,N:G204R,N:S235F,ORF1a:T1001I,ORF1a:A1708D,ORF1a:I2230T,ORF1a:V2494I,ORF1b:P218L,ORF1b:P314L,ORF8:Q27*,ORF8:R52I,ORF8:Y73C,S:S98F,S:N501Y,S:A570D,S:D614G,S:P681H,S:T716I,S:S982A,S:D1118H	6	351
hCoV-19/Mexico/HID-InDRE_F12574_S1201/2021	EPI_ISL_1626808	-	B.1.1.7	GRY	37	C241T,C913T,C3037T,C3267T,C5388A,C5986T,T6954C,C7765T,G8179T,A11983G,C14408T,C14676T,T15096C,C15279T,C15960T,T16176C,A23063T,C23271A,A23403G,C23604A,C23709T,T24506G,G24914C,A25336C,C27972T,G28048T,A28111G,G28280C,A28281T,T28282A,G28287A,C28724T,G28881A,G28882A,G28883C,G28884C,C28977T	22	N:D3L,N:G5E,N:P151S,N:R203K,N:G204P,N:S235F,ORF1a:T1001I,ORF1a:A1708D,ORF1a:I2230T,ORF1b:P314L,ORF8:Q27*,ORF8:R52I,ORF8:Y73C,ORF9b:D2N,S:N501Y,S:A570D,S:D614G,S:P681H,S:T716I,S:S982A,S:D1118H,S:E1258D	6	0
hCoV-19/Mexico/HID-InDRE_FB13039_S1505/2021	EPI_ISL_1805483	-	B.1.1.7	GRY	35	C241T,C913T,C2453T,C3037T,C3267T,C5388A,C5986T,T6954C,A7479G,C9611T,C14408T,C14676T,C15279T,T16176C,C21855T,G21974C,A23063T,C23271A,A23403G,C23604A,C23709T,T24506G,G24914C,A25336C,C27972T,G28048T,A28111G,G28280C,A28281T,T28282A,A28295G,G28881A,G28882A,G28883C,C28977T	25	N:D3L,N:N8D,N:R203K,N:G204R,N:S235F,ORF1a:L730F,ORF1a:T1001I,ORF1a:A1708D,ORF1a:I2230T,ORF1a:N2405S,ORF1a:L3116F,ORF1b:P314L,ORF8:Q27*,ORF8:R52I,ORF8:Y73C,S:S98F,S:D138H,S:N501Y,S:A570D,S:D614G,S:P681H,S:T716I,S:S982A,S:D1118H,S:E1258D	6	0
hCoV-19/Mexico/HID-InDRE_FB13996_S2048/2021	EPI_ISL_2283690	-	B.1.1.7	GRY	36	C241T,C913T,C2453T,C3037T,C3267T,C3315T,C3784T,C5388A,C5986T,T6954C,A7479G,C9611T,C14408T,C14676T,C15279T,T16176C,C21855T,G21974C,A23063T,C23271A,A23403G,C23604A,C23709T,T24506G,G24914C,C27972T,G28048T,A28111G,G28280C,A28281T,T28282A,A28295G,G28881A,G28882A,G28883C,C28977T	25	N:D3L,N:N8D,N:R203K,N:G204R,N:S235F,ORF1a:L730F,ORF1a:T1001I,ORF1a:T1017I,ORF1a:A1708D,ORF1a:I2230T,ORF1a:N2405S,ORF1a:L3116F,ORF1b:P314L,ORF8:Q27*,ORF8:R52I,ORF8:Y73C,S:S98F,S:D138H,S:N501Y,S:A570D,S:D614G,S:P681H,S:T716I,S:S982A,S:D1118H	6	3
hCoV-19/Mexico/HID-InDRE_FB14798_S2046/2021	EPI_ISL_2283688	-	B.1.1.7	GRY	31	C241T,C913T,C3037T,C3267T,C3411T,C5388A,C5986T,T6954C,T11408C,C14408T,C14676T,C15279T,T16176C,A23063T,C23271A,A23403G,C23604A,C23709T,C24374T,T24506G,G24914C,C27972T,G28048T,A28111G,G28280C,A28281T,T28282A,G28881A,G28882A,G28883C,C28977T	20	N:D3L,N:R203K,N:G204R,N:S235F,ORF1a:T1001I,ORF1a:A1049V,ORF1a:A1708D,ORF1a:I2230T,ORF1b:P314L,ORF8:Q27*,ORF8:R52I,ORF8:Y73C,S:N501Y,S:A570D,S:D614G,S:P681H,S:T716I,S:L938F,S:S982A,S:D1118H	6	0
hCoV-19/Mexico/HID-InDRE_FB17207_S3093/2021	EPI_ISL_2663360	-	B.1.1.7	GRY	35	C241T,C913T,C2453T,C3037T,C3267T,C3784T,C5388A,C5986T,T6954C,A7479G,C9611T,C14408T,C14676T,C15279T,T16176C,C21855T,G21974C,A23063T,C23271A,A23403G,C23604A,C23709T,T24506G,G24914C,C27972T,G28048T,A28111G,G28280C,A28281T,T28282A,A28295G,G28881A,G28882A,G28883C,C28977T	24	N:D3L,N:N8D,N:R203K,N:G204R,N:S235F,ORF1a:L730F,ORF1a:T1001I,ORF1a:A1708D,ORF1a:I2230T,ORF1a:N2405S,ORF1a:L3116F,ORF1b:P314L,ORF8:Q27*,ORF8:R52I,ORF8:Y73C,S:S98F,S:D138H,S:N501Y,S:A570D,S:D614G,S:P681H,S:T716I,S:S982A,S:D1118H	6	0
hCoV-19/Mexico/HID-InDRE_FB17212_S3094/2021	EPI_ISL_2663361	-	B.1.1.7	GRY	35	C241T,C913T,G2144A,C2453T,C3037T,C3267T,C5388A,C5986T,T6954C,C9611T,C14408T,C14676T,C15279T,T16176C,G18651T,C21855T,G21974C,A23063T,C23271A,A23403G,C23604A,C23709T,T24506G,G24914C,C27972T,G28048T,A28111G,G28280C,A28281T,T28282A,A28295G,G28881A,G28882A,G28883C,C28977T	25	N:D3L,N:N8D,N:R203K,N:G204R,N:S235F,ORF1a:V627I,ORF1a:L730F,ORF1a:T1001I,ORF1a:A1708D,ORF1a:I2230T,ORF1a:L3116F,ORF1b:P314L,ORF1b:E1728D,ORF8:Q27*,ORF8:R52I,ORF8:Y73C,S:S98F,S:D138H,S:N501Y,S:A570D,S:D614G,S:P681H,S:T716I,S:S982A,S:D1118H	6	0
hCoV-19/Mexico/HID-InDRE_FB17213_S3095/2021	EPI_ISL_2663362	-	B.1.1.7	GRY	36	C241T,C913T,G2144A,C2453T,C3037T,C3267T,A5139G,C5388A,C5986T,T6954C,C9611T,C14408T,C14676T,C15279T,T16176C,G18651T,C21855T,G21974C,A23063T,C23271A,A23403G,C23604A,C23709T,T24506G,G24914C,C27972T,G28048T,A28111G,G28280C,A28281T,T28282A,A28295G,G28881A,G28882A,G28883C,C28977T	26	N:D3L,N:N8D,N:R203K,N:G204R,N:S235F,ORF1a:V627I,ORF1a:L730F,ORF1a:T1001I,ORF1a:D1625G,ORF1a:A1708D,ORF1a:I2230T,ORF1a:L3116F,ORF1b:P314L,ORF1b:E1728D,ORF8:Q27*,ORF8:R52I,ORF8:Y73C,S:S98F,S:D138H,S:N501Y,S:A570D,S:D614G,S:P681H,S:T716I,S:S982A,S:D1118H	6	0
hCoV-19/Mexico/HID-InDRE_FB17214_S3096/2021	EPI_ISL_2663363	-	B.1.1.7	GRY	35	C241T,C913T,C2453T,C3037T,C3267T,C3784T,C5388A,C5986T,T6954C,A7479G,C9611T,C14408T,C14676T,C15279T,T16176C,C21855T,G21974C,A23063T,C23271A,A23403G,C23604A,C23709T,T24506G,G24914C,C27972T,G28048T,A28111G,G28280C,A28281T,T28282A,A28295G,G28881A,G28882A,G28883C,C28977T	24	N:D3L,N:N8D,N:R203K,N:G204R,N:S235F,ORF1a:L730F,ORF1a:T1001I,ORF1a:A1708D,ORF1a:I2230T,ORF1a:N2405S,ORF1a:L3116F,ORF1b:P314L,ORF8:Q27*,ORF8:R52I,ORF8:Y73C,S:S98F,S:D138H,S:N501Y,S:A570D,S:D614G,S:P681H,S:T716I,S:S982A,S:D1118H	6	0
hCoV-19/Mexico/HID-InDRE_FB17221_S3097/2021	EPI_ISL_2663364	-	B.1.1.7	GRY	37	C241T,C913T,C2110T,C3037T,C3267T,C5388A,C5986T,G6166A,T6954C,T7984C,C8917T,A9377G,C14120T,C14408T,C14676T,C15279T,T16176C,C18744T,C19390T,A23063T,C23271A,A23403G,C23604A,C23709T,T24506G,G24914C,C27972T,G28048T,A28095T,A28111G,G28280C,A28281T,T28282A,G28881A,G28882A,G28883C,C28977T	22	N:D3L,N:R203K,N:G204R,N:S235F,ORF1a:T1001I,ORF1a:A1708D,ORF1a:I2230T,ORF1a:I3038V,ORF1b:P218L,ORF1b:P314L,ORF1b:P1975S,ORF8:Q27*,ORF8:R52I,ORF8:K68*,ORF8:Y73C,S:N501Y,S:A570D,S:D614G,S:P681H,S:T716I,S:S982A,S:D1118H	6	0
hCoV-19/Mexico/HID-InDRE_FB17223_S3098/2021	EPI_ISL_2663365	-	B.1.1.7	GRY	33	C241T,C913T,C3037T,C3267T,A3492G,C5388A,C5986T,T6954C,C14408T,C14676T,C15279T,T16176C,C18555T,C21639A,A23063T,C23271A,A23403G,C23604A,C23709T,C24374T,T24506G,G24914C,C27972T,G28048T,A28111G,G28280C,A28281T,T28282A,G28881A,G28882A,G28883C,	21	N:D3L,N:R203K,N:G204R,N:S235F,ORF1a:T1001I,ORF1a:N1076S,ORF1a:A1708D,ORF1a:I2230T,ORF1b:P314L,ORF8:Q27*,ORF8:R52I,ORF8:Y73C,S:P26H,S:N501Y,S:A570D,S:D614G,S:P681H,S:T716I,S:L938F,S:S982A,S:D1118H	6	0
hCoV-19/Mexico/HID-InDRE_FB17224_S3099/2021	EPI_ISL_2663366	-	B.1.1.7	GRY	31	C241T,C913T,C3037T,C3267T,C5388A,C5986T,C6196T,T6954C,A8848G,C14408T,C14676T,C15279T,T16176C,A23063T,C23271A,A23403G,C23604A,C23709T,T24506G,G24914C,C27972T,G28048T,A28111G,G28280C,A28281T,T28282A,G28881A,G28882A,G28883C,C28977T,G29715T	18	N:D3L,N:R203K,N:G204R,N:S235F,ORF1a:T1001I,ORF1a:A1708D,ORF1a:I2230T,ORF1b:P314L,ORF8:Q27*,ORF8:R52I,ORF8:Y73C,S:N501Y,S:A570D,S:D614G,S:P681H,S:T716I,S:S982A,S:D1118H	6	0

hCoV-19/Mexico/HID-InDRE_FB17225_S3100/2021	EPI_ISL_2663367	-	B.1.1.7	GRY	37	C241T,C913T,C2453T,C3037T,C3267T,C3784T,C5388A,C5986T,T6954C,A7479G,C9611T,C12439T,C14408T,C14676T,C15279T,T16176C,C21855T,G21974C,A23063T,C23271A,A23403G,C23604A,C23709T,T24506G,G24914C,C27213T,C27972T,G28048T,A28111G,G28280C,A28281T,T28282A,A28295G,G28881A,G28882A,G28883C,C28977T	24	N:D3L,N:N8D,N:R203K,N:G204R,N:S235F,ORF1a:L730F,ORF1a:T1001I,ORF1a:A1708D,ORF1a:I2230T,ORF1a:N2405S,ORF1a:L3116F,ORF1b:P314L,ORF8:Q27*,ORF8:R52I,ORF8:Y73C,S:S98F,S:D138H,S:N501Y,S:A570D,S:D614G,S:P681H,S:T716I,S:S982A,S:D1118H	6	0
hCoV-19/Mexico/HID-InDRE_FB17226_S3101/2021	EPI_ISL_2663368	-	B.1.1.7	GRY	37	C241T,C913T,C2453T,C3037T,C3267T,C3784T,C5388A,C5986T,T6954C,A7479G,C9611T,C12439T,C14408T,C14676T,C15279T,T16176C,C21855T,G21974C,A23063T,C23271A,A23403G,C23604A,C23709T,T24506G,G24914C,C27213T,C27972T,G28048T,A28111G,G28280C,A28281T,T28282A,A28295G,G28881A,G28882A,G28883C,C28977T	24	N:D3L,N:N8D,N:R203K,N:G204R,N:S235F,ORF1a:L730F,ORF1a:T1001I,ORF1a:A1708D,ORF1a:I2230T,ORF1a:N2405S,ORF1a:L3116F,ORF1b:P314L,ORF8:Q27*,ORF8:R52I,ORF8:Y73C,S:S98F,S:D138H,S:N501Y,S:A570D,S:D614G,S:P681H,S:T716I,S:S982A,S:D1118H	6	0
hCoV-19/Mexico/HID-InDRE_FB17234_S3102/2021	EPI_ISL_2663369	-	B.1.1.7	GRY	35	C241T,C913T,G2144A,C2453T,C3037T,C3267T,C5388A,C5986T,T6954C,C9611T,C14408T,C14676T,C15279T,T16176C,G18651T,C21855T,G21974C,A23063T,C23271A,A23403G,C23604A,C23709T,T24506G,G24914C,C27972T,G28048T,A28111G,G28280C,A28281T,T28282A,A28295G,G28881A,G28882A,G28883C,C28977T	25	N:D3L,N:N8D,N:R203K,N:G204R,N:S235F,ORF1a:V627I,ORF1a:L730F,ORF1a:T1001I,ORF1a:A1708D,ORF1a:I2230T,ORF1a:L3116F,ORF1b:P314L,ORF1b:E1728D,ORF8:Q27*,ORF8:R52I,ORF8:Y73C,S:S98F,S:D138H,S:N501Y,S:A570D,S:D614G,S:P681H,S:T716I,S:S982A,S:D1118H	6	0
hCoV-19/Mexico/HID-InDRE_FB17235_S3103/2021	EPI_ISL_2663370	-	B.1.1.7	GRY	35	C241T,C913T,G2144A,C2453T,C3037T,C3267T,C5388A,C5986T,T6954C,C9611T,C14408T,C14676T,C15279T,T16176C,G18651T,C21855T,G21974C,A23063T,C23271A,A23403G,C23604A,C23709T,T24506G,G24914C,C27972T,G28048T,A28111G,G28280C,A28281T,T28282A,A28295G,G28881A,G28882A,G28883C,C28977T	25	N:D3L,N:N8D,N:R203K,N:G204R,N:S235F,ORF1a:V627I,ORF1a:L730F,ORF1a:T1001I,ORF1a:A1708D,ORF1a:I2230T,ORF1a:L3116F,ORF1b:P314L,ORF1b:E1728D,ORF8:Q27*,ORF8:R52I,ORF8:Y73C,S:S98F,S:D138H,S:N501Y,S:A570D,S:D614G,S:P681H,S:T716I,S:S982A,S:D1118H	6	0
hCoV-19/Mexico/HID-InDRE_FB17239_S3104/2021	EPI_ISL_2663371	-	B.1.1.7	GRY	37	C241T,C913T,C2453T,C3037T,C3267T,C3784T,C5388A,C5986T,T6954C,A7479G,C7528T,C9611T,C14408T,C14676T,C15279T,T16176C,C21855T,G21974C,A23063T,C23271A,A23403G,C23604A,C23709T,G24173T,T24506G,G24914C,C27972T,G28048T,A28111G,G28280C,A28281T,T28282A,A28295G,G28881A,G28882A,G28883C,C28977T	25	N:D3L,N:N8D,N:R203K,N:G204R,N:S235F,ORF1a:L730F,ORF1a:T1001I,ORF1a:A1708D,ORF1a:I2230T,ORF1a:N2405S,ORF1a:L3116F,ORF1b:P314L,ORF8:Q27*,ORF8:R52I,ORF8:Y73C,S:S98F,S:D138H,S:N501Y,S:A570D,S:D614G,S:P681H,S:T716I,S:A871S,S:S982A,S:D1118H	6	0
hCoV-19/Mexico/HID-InDRE_FB17241_S3105/2021	EPI_ISL_2663372	-	B.1.1.7	GRY	38	C241T,C913T,G2144A,C2453T,C3037T,C3267T,A5139G,C5388A,C5986T,T6954C,C9611T,C14408T,C14676T,C15279T,T16176C,G18651T,C21855T,G21974C,G22471T,A23063T,C23271A,A23403G,C23604A,C23709T,T24506G,G24914C,C25810T,C27972T,G28048T,A28111G,G28280C,A28281T,T28282A,A28295G,G28881A,G28882A,G28883C,C28977T	28	N:D3L,N:N8D,N:R203K,N:G204R,N:S235F,ORF1a:V627I,ORF1a:L730F,ORF1a:T1001I,ORF1a:D1625G,ORF1a:A1708D,ORF1a:I2230T,ORF1a:L3116F,ORF1b:P314L,ORF1b:E1728D,ORF3a:L140F,ORF8:Q27*,ORF8:R52I,ORF8:Y73C,S:S98F,S:D138H,S:L303F,S:N501Y,S:A570D,S:D614G,S:P681H,S:T716I,S:S982A,S:D1118	6	0
hCoV-19/Mexico/HID-InDRE_FB17242_S3106/2021	EPI_ISL_2663373	-	B.1.1.7	GRY	38	C241T,C913T,G2144A,C2453T,C3037T,C3267T,A5139G,C5388A,C5986T,T6954C,C9611T,C14408T,C14676T,C15279T,T16176C,G18651T,C21855T,G21974C,G22471T,A23063T,C23271A,A23403G,C23604A,C23709T,T24506G,G24914C,C25810T,C27972T,G28048T,A28111G,G28280C,A28281T,T28282A,A28295G,G28881A,G28882A,G28883C,C28977T	28	N:D3L,N:N8D,N:R203K,N:G204R,N:S235F,ORF1a:V627I,ORF1a:L730F,ORF1a:T1001I,ORF1a:D1625G,ORF1a:A1708D,ORF1a:I2230T,ORF1a:L3116F,ORF1b:P314L,ORF1b:E1728D,ORF3a:L140F,ORF8:Q27*,ORF8:R52I,ORF8:Y73C,S:S98F,S:D138H,S:L303F,S:N501Y,S:A570D,S:D614G,S:P681H,S:T716I,S:S982A,S:D1118	6	0
hCoV-19/Mexico/HID-InDRE_FB17245_S3107/2021	EPI_ISL_2663374	-	B.1.1.7	GRY	38	C241T,C913T,G2144A,C2453T,C3037T,C3267T,A5139G,C5388A,C5986T,T6954C,C9611T,C14408T,C14676T,C15279T,T16176C,G18651T,C21855T,G21974C,G22471T,A23063T,C23271A,A23403G,C23604A,C23709T,T24506G,G24914C,C25810T,C27972T,G28048T,A28111G,G28280C,A28281T,T28282A,A28295G,G28881A,G28882A,G28883C,C28977T	28	N:D3L,N:N8D,N:R203K,N:G204R,N:S235F,ORF1a:V627I,ORF1a:L730F,ORF1a:T1001I,ORF1a:D1625G,ORF1a:A1708D,ORF1a:I2230T,ORF1a:L3116F,ORF1b:P314L,ORF1b:E1728D,ORF3a:L140F,ORF8:Q27*,ORF8:R52I,ORF8:Y73C,S:S98F,S:D138H,S:L303F,S:N501Y,S:A570D,S:D614G,S:P681H,S:T716I,S:S982A,S:D1118	6	0
hCoV-19/Mexico/HID-InDRE_FB17246_S3108/2021	EPI_ISL_2663375	-	B.1.1.7	GRY	40	C241T,C913T,C2453T,C3037T,C3267T,C3738T,C3784T,G4300T,C5388A,C5986T,T6954C,A7479G,C9611T,C12439T,C14408T,C14676T,C15279T,T16176C,C17850T,C21855T,G21974C,A23063T,C23271A,A23403G,C23604A,C23709T,T24506G,G24914C,C27213T,C27972T,G28048T,A28111G,G28280C,A28281T,T28282A,A28295G,G28881A,G28882A,G28883C,C28977T	25	N:D3L,N:N8D,N:R203K,N:G204R,N:S235F,ORF1a:L730F,ORF1a:T1001I,ORF1a:P1158L,ORF1a:A1708D,ORF1a:I2230T,ORF1a:N2405S,ORF1a:L3116F,ORF1b:P314L,ORF8:Q27*,ORF8:R52I,ORF8:Y73C,S:S98F,S:D138H,S:N501Y,S:A570D,S:D614G,S:P681H,S:T716I,S:S982A,S:D1118H	6	0
hCoV-19/Mexico/HID-InDRE_FB17887_S3933/2021	EPI_ISL_2858947	-	B.1.1.7	GR	35	C241T,C913T,C2453T,C3037T,C3267T,G4207T,C5388A,C5986T,T6954C,A7479G,C9611T,G14202A,C14408T,C14676T,C15279T,T16176C,C21855T,G21974C,C23271A,A23403G,C23604A,C23709T,T24506G,G24914C,C27972T,G28048T,A28111G,G28280C,A28281T,T28282A,A28295G,G28881A,G28882A,G28883C,C28977T	23	N:D3L,N:N8D,N:R203K,N:G204R,N:S235F,ORF1a:L730F,ORF1a:T1001I,ORF1a:A1708D,ORF1a:I2230T,ORF1a:N2405S,ORF1a:L3116F,ORF1b:P314L,ORF8:Q27*,ORF8:R52I,ORF8:Y73C,S:S98F,S:D138H,S:A570D,S:D614G,S:P681H,S:T716I,S:S982A,S:D1118H	6	324
hCoV-19/Mexico/HID-InDRE_FB19125_S4493/2021	EPI_ISL_3033347	-	B.1.1.7	GRY	43	C241T,C913T,C2110T,C2453T,G3004T,C3037T,C3267T,C5388A,C5986T,T6954C,G7042T,C9996T,C10525T,C10789T,C14120T,C14408T,C14676T,C15279T,T16176C,A23063T,C23271A,A23403G,G23593T,C23604A,C23709T,T24506G,G24914C,C25844T,C26110T,C26735T,C27389T,C27972T,G28048T,A28095T,A28111G,G28280C,A28281T,T28282A,A28804G,G28881A,G28882A,G28883	27	N:D3L,N:R203K,N:G204R,N:S235F,ORF1a:L730F,ORF1a:E913D,ORF1a:T1001I,ORF1a:A1708D,ORF1a:I2230T,ORF1a:M2259I,ORF1a:S3244L,ORF1b:P218L,ORF1b:P314L,ORF3a:T151I,ORF3a:P240S,ORF8:Q27*,ORF8:R52I,ORF8:K68*,ORF8:Y73C,S:N501Y,S:A570D,S:D614G,S:Q677H,S:P681H,S:T716I,S:S982A,S:D11	6	0
hCoV-19/Mexico/HID-InDRE_FB19127_S4494/2021	EPI_ISL_3033348	-	B.1.1.7	GRY	37	C241T,C913T,C2453T,C3037T,C3267T,C3784T,C5388A,C5986T,T6954C,A7479G,C9611T,T9787C,G14202A,C14408T,C14676T,C15279T,T16176C,C21855T,G21974C,A23063T,C23271A,A23403G,C23604A,C23709T,T24506G,G24914C,C27972T,G28048T,A28111G,G28280C,A28281T,T28282A,A28295G,G28881A,G28882A,G28883C,C28977T	24	N:D3L,N:N8D,N:R203K,N:G204R,N:S235F,ORF1a:L730F,ORF1a:T1001I,ORF1a:A1708D,ORF1a:I2230T,ORF1a:N2405S,ORF1a:L3116F,ORF1b:P314L,ORF8:Q27*,ORF8:R52I,ORF8:Y73C,S:S98F,S:D138H,S:N501Y,S:A570D,S:D614G,S:P681H,S:T716I,S:S982A,S:D1118H	6	0
hCoV-19/Mexico/HID-InDRE_FB19128_S4495/2021	EPI_ISL_3033349	-	B.1.1.7	GRY	37	C241T,C913T,C2453T,C3037T,C3267T,C3784T,C5388A,C5986T,T6954C,A7479G,C9611T,T9787C,G14202A,C14408T,C14676T,C15279T,T16176C,C21855T,G21974C,A23063T,C23271A,A23403G,C23604A,C23709T,T24506G,G24914C,C27972T,G28048T,A28111G,G28280C,A28281T,T28282A,A28295G,G28881A,G28882A,G28883C,C28977T	24	N:D3L,N:N8D,N:R203K,N:G204R,N:S235F,ORF1a:L730F,ORF1a:T1001I,ORF1a:A1708D,ORF1a:I2230T,ORF1a:N2405S,ORF1a:L3116F,ORF1b:P314L,ORF8:Q27*,ORF8:R52I,ORF8:Y73C,S:S98F,S:D138H,S:N501Y,S:A570D,S:D614G,S:P681H,S:T716I,S:S982A,S:D1118H	6	0
hCoV-19/Mexico/HID-InDRE_FB19136_S4496/2021	EPI_ISL_3033350	-	B.1.1.7	GRY	39	C241T,C913T,G2144A,C2453T,C3037T,C3267T,C5388A,C5986T,T6954C,A8203G,C9611T,C11651T,C14408T,C14676T,C15279T,T16176C,G18651T,G20397T,C21855T,G21974C,A23063T,C23271A,A23403G,C23604A,C23709T,T24506G,G24914C,C27972T,G28048T,A28111G,G28280C,A28281T,T28282A,A28295G,G28881A,G28882A,G28883C,C28977T,G29751T	27	N:D3L,N:N8D,N:R203K,N:G204R,N:S235F,ORF1a:V627I,ORF1a:L730F,ORF1a:T1001I,ORF1a:A1708D,ORF1a:I2230T,ORF1a:L3116F,ORF1a:L3796F,ORF1b:P314L,ORF1b:E1728D,ORF1b:K2310N,ORF8:Q27*,ORF8:R52I,ORF8:Y73C,S:S98F,S:D138H,S:N501Y,S:A570D,S:D614G,S:P681H,S:T716I,S:S982A,S:D1118H	6	0
hCoV-19/Mexico/HID-InDRE_FB19137_S4497/2021	EPI_ISL_3033351	-	B.1.1.7	GRY	37	C241T,C913T,C2453T,C3037T,C3267T,C3784T,C5388A,C5986T,T6954C,A7479G,C9611T,C14408T,C14676T,C15279T,T16176C,C21789T,C21855T,G21974C,A23063T,C23271A,A23403G,C23604A,C23709T,T24506G,G24914C,C27737T,C27972T,G28048T,A28111G,G28280C,A28281T,T28282A,A28295G,G28881A,G28882A,G28883C,C28977T	26	N:D3L,N:N8D,N:R203K,N:G204R,N:S235F,ORF1a:L730F,ORF1a:T1001I,ORF1a:A1708D,ORF1a:I2230T,ORF1a:N2405S,ORF1a:L3116F,ORF1b:P314L,ORF7a:T115I,ORF8:Q27*,ORF8:R52I,ORF8:Y73C,S:T76I,S:S98F,S:D138H,S:N501Y,S:A570D,S:D614G,S:P681H,S:T716I,S:S982A,S:D1118H	6	0
hCoV-19/Mexico/HID-InDRE_FB19139_S4498/2021	EPI_ISL_3033352	-	B.1.1.7	GRY	43	C241T,C913T,C2110T,C2453T,G3004T,C3037T,C3267T,C5388A,C5986T,T6954C,G7042T,C9996T,C10525T,C10789T,C14120T,C14408T,C14676T,C15279T,T16176C,A23063T,C23271A,A23403G,G23593T,C23604A,C23709T,T24506G,G24914C,C25844T,C26110T,C26735T,C27389T,C27972T,G28048T,A28095T,A28111G,G28280C,A28281T,T28282A,A28804G,G28881A,G28882A,G28883	27	N:D3L,N:R203K,N:G204R,N:S235F,ORF1a:L730F,ORF1a:E913D,ORF1a:T1001I,ORF1a:A1708D,ORF1a:I2230T,ORF1a:M2259I,ORF1a:S3244L,ORF1b:P218L,ORF1b:P314L,ORF3a:T151I,ORF3a:P240S,ORF8:Q27*,ORF8:R52I,ORF8:K68*,ORF8:Y73C,S:N501Y,S:A570D,S:D614G,S:Q677H,S:P681H,S:T716I,S:S982A,S:D11	6	0
hCoV-19/Mexico/HID-INMEGEN-05-02-116/2021	EPI_ISL_2230873	-	B.1.1.7	GRY	35	C241T,C913T,C2453T,C3037T,C3267T,C3784T,C5388A,C5986T,T6954C,A7479G,C9611T,C14408T,C14676T,C15279T,T16176C,C21855T,G21974C,A23063T,C23271A,A23403G,C23604A,C23709T,T24506G,G24914C,C27972T,G28048T,A28111G,G28280C,A28281T,T28282A,A28295G,G28881A,G28882A,G28883C,C28977T	24	N:D3L,N:N8D,N:R203K,N:G204R,N:S235F,ORF1a:L730F,ORF1a:T1001I,ORF1a:A1708D,ORF1a:I2230T,ORF1a:N2405S,ORF1a:L3116F,ORF1b:P314L,ORF8:Q27*,ORF8:R52I,ORF8:Y73C,S:S98F,S:D138H,S:N501Y,S:A570D,S:D614G,S:P681H,S:T716I,S:S982A,S:D1118H	6	0
hCoV-19/Mexico/HID-INMEGEN-05-02-154/2021	EPI_ISL_2230876	-	B.1.1.7	GRY	37	C241T,C913T,C2453T,C3037T,C3267T,C3784T,C3924T,C5388A,C5986T,T6954C,A7479G,C9611T,C14408T,C14676T,C15279T,T16176C,C21855T,G21974C,A23063T,C23271A,A23403G,C23604A,C23709T,T24506G,G24914C,T25496C,C27972T,G28048T,A28111G,G28280C,A28281T,T28282A,A28295G,G28881A,G28882A,G28883C,C28977T	26	N:D3L,N:N8D,N:R203K,N:G204R,N:S235F,ORF1a:L730F,ORF1a:T1001I,ORF1a:P1220L,ORF1a:A1708D,ORF1a:I2230T,ORF1a:N2405S,ORF1a:L3116F,ORF1b:P314L,ORF3a:I35T,ORF8:Q27*,ORF8:R52I,ORF8:Y73C,S:S98F,S:D138H,S:N501Y,S:A570D,S:D614G,S:P681H,S:T716I,S:S982A,S:D1118H	6	3
hCoV-19/Mexico/HID-INMEGEN-05-02-171/2021	EPI_ISL_2230878	-	B.1.1.7	GRY	40	C241T,C913T,C2453T,C3037T,C3267T,C3784T,C3924T,C5388A,C5986T,T6954C,A7479G,C9611T,C14408T,C14676T,C15279T,T16176C,C21855T,C21897T,G21974C,A23063T,C23271A,A23403G,C23604A,C23709T,T24506G,G24914C,T25496C,C26549T,A26563G,C27972T,G28048T,A28111G,G28280C,A28281T,T28282A,A28295G,G28881A,G28882A,G28883C,C28977T	28	M:K14R,N:D3L,N:N8D,N:R203K,N:G204R,N:S235F,ORF1a:L730F,ORF1a:T1001I,ORF1a:P1220L,ORF1a:A1708D,ORF1a:I2230T,ORF1a:N2405S,ORF1a:L3116F,ORF1b:P314L,ORF3a:I35T,ORF8:Q27*,ORF8:R52I,ORF8:Y73C,S:S98F,S:S112L,S:D138H,S:N501Y,S:A570D,S:D614G,S:P681H,S:T716I,S:S982A,S:D1118H	6	2

hCoV-19/Mexico/HID-INMEGEN-05-02-179/2021	EPI_ISL_2230885	-	B.1.1.7	GRY	37	C241T,C913T,G1400A,C2110T,C3037T,C3267T,C5388A,C5986T,T6954C,G7042T,G9929A,A10195G,C14120T,C14408T,C14676T,C15279T,T16176C,A23063T,C23271A,A23403G,C23604A,C23709T,T24506G,G24914C,C27972T,G28048T,G28075T,A28095T,A28111G,G28280C,A28281T,T28282A,T28688C,G28881A,G28882A,G28883C,C28977T	24	N:D3L,N:R203K,N:G204R,N:S235F,ORF1a:G379R,ORF1a:T1001I,ORF1a:A1708D,ORF1a:I2230T,ORF1a:M2259I,ORF1a:D3222N,ORF1b:P218L,ORF1b:P314L,ORF8:Q27*,ORF8:R52I,ORF8:C61F,ORF8:K68*,ORF8:Y73C,S:N501Y,S:A570D,S:D614G,S:P681H,S:T716I,S:S982A,S:D1118H	6	0
hCoV-19/Mexico/HID-INMEGEN-05-03-156/2021	EPI_ISL_2319289	-	B.1.1.7	GRY	39	C241T,C913T,C3037T,C3267T,C3704T,C5388A,C5986T,T6954C,G6986A,A12162G,C14408T,C14676T,C15279T,C15720T,T16176C,A17615G,A21137G,T22020C,A23063T,C23271A,A23403G,C23604A,C23709T,T24506G,C24904A,G24914C,G25135T,A27003G,C27972T,G28048T,A28111G,G28280C,A28281T,T28282A,G28881A,G28882A,G28883C,C28977T,C29280T	27	M:I161V,N:D3L,N:R203K,N:G204R,N:S235F,N:A336V,ORF1a:T1001I,ORF1a:P1147S,ORF1a:A1708D,ORF1a:I2230T,ORF1a:G2241S,ORF1a:Q3966R,ORF1b:P314L,ORF1b:K1383R,ORF1b:K2557R,ORF8:Q27*,ORF8:R52I,ORF8:Y73C,S:M153T,S:N501Y,S:A570D,S:D614G,S:P681H,S:T716I,S:S982A,S:D1118H,S:K1191N	6	94
hCoV-19/Mexico/HID-INMEGEN-05-03-157/2021	EPI_ISL_2319290	-	B.1.1.7	GR	37	C913T,C3037T,C3267T,C3704T,C5388A,C5986T,T6954C,G6986A,A12162G,C14408T,C14676T,C15279T,C15720T,T16176C,A17615G,A21137G,T22020C,C23271A,A23403G,C23604A,C23709T,T24506G,C24904A,G24914C,G25135T,A27003G,C27972T,G28048T,A28111G,G28280C,A28281T,T28282A,G28881A,G28882A,G28883C,C28977T,C29280T	26	M:I161V,N:D3L,N:R203K,N:G204R,N:S235F,N:A336V,ORF1a:T1001I,ORF1a:P1147S,ORF1a:A1708D,ORF1a:I2230T,ORF1a:G2241S,ORF1a:Q3966R,ORF1b:P314L,ORF1b:K1383R,ORF1b:K2557R,ORF8:Q27*,ORF8:R52I,ORF8:Y73C,S:M153T,S:A570D,S:D614G,S:P681H,S:T716I,S:S982A,S:D1118H,S:K1191N	4	2993
hCoV-19/Mexico/HID-INMEGEN-05-03-160/2021	EPI_ISL_2319293	-	B.1.1.7	GR	36	C241T,C913T,C2644T,C3037T,C3267T,C5388A,C5986T,C6196T,C6730T,A8848G,A11477C,C14408T,C14676T,C15279T,T16176C,C23271A,A23403G,C23604A,C23709T,T24506G,G24914C,G25217T,G25489C,C26464T,G26727T,C27972T,G28048T,A28111G,G28280C,A28281T,T28282A,G28881A,G28882A,G28883C,C28977T,G29715T	20	M:A69S,N:D3L,N:R203K,N:G204R,N:S235F,ORF1a:T1001I,ORF1a:A1708D,ORF1a:I3738L,ORF1b:P314L,ORF3a:A33P,ORF8:Q27*,ORF8:R52I,ORF8:Y73C,S:A570D,S:D614G,S:P681H,S:T716I,S:S982A,S:D1118H,S:G1219C	6	1328
hCoV-19/Mexico/HID-INMEGEN-05-03-29/2021	EPI_ISL_2319191	-	B.1.1.7	GRY	38	C241T,C913T,C3037T,C3267T,C5388A,C5944T,C5986T,C6026T,T6954C,C9967T,C13585T,C14408T,C14676T,T15096C,C15279T,T16176C,G18412T,C19983T,C22987T,A23063T,C23271A,A23403G,C23604A,C23709T,T24506G,G24914C,C27972T,G28048T,A28095T,A28111G,G28280C,A28281T,T28282A,G28881A,G28882A,G28883C,G28884C,C28977T	21	N:D3L,N:R203K,N:G204P,N:S235F,ORF1a:T1001I,ORF1a:A1708D,ORF1a:P1921S,ORF1a:I2230T,ORF1b:P314L,ORF1b:V1649F,ORF8:Q27*,ORF8:R52I,ORF8:K68*,ORF8:Y73C,S:N501Y,S:A570D,S:D614G,S:P681H,S:T716I,S:S982A,S:D1118H	6	80
hCoV-19/Mexico/HID-INMEGEN-05-03-32/2021	EPI_ISL_2319193	-	B.1.1.7	G	26	C913T,C3037T,C3267T,C5388A,C5986T,T6954C,C9967T,C14408T,C14676T,C15279T,T16176C,G18412T,C23271A,A23403G,C23604A,C23709T,T24506G,G24914C,C27972T,G28048T,A28095T,A28111G,G28280C,A28281T,T28282A,C28977T	17	N:D3L,N:S235F,ORF1a:T1001I,ORF1a:A1708D,ORF1a:I2230T,ORF1b:P314L,ORF1b:V1649F,ORF8:Q27*,ORF8:R52I,ORF8:K68*,ORF8:Y73C,S:A570D,S:D614G,S:P681H,S:T716I,S:S982A,S:D1118H	6	2595
hCoV-19/Mexico/HID-INMEGEN-05-04-25/2021	EPI_ISL_2349903	-	B.1.1.7	GRY	38	C241T,C913T,C2453T,C3037T,C3267T,C3784T,C3924T,C5388A,C5986T,T6954C,A7479G,C9611T,C14408T,C14676T,C15279T,T16176C,C21855T,G21974C,A23063T,C23271A,A23403G,C23604A,C23709T,T24506G,G24914C,T25496C,C27972T,C28011T,G28048T,A28111G,G28280C,A28281T,T28282A,A28295G,G28881A,G28882A,G28883C,C28977T	27	N:D3L,N:N8D,N:R203K,N:G204R,N:S235F,ORF1a:L730F,ORF1a:T1001I,ORF1a:P1220L,ORF1a:A1708D,ORF1a:I2230T,ORF1a:N2405S,ORF1a:L3116F,ORF1b:P314L,ORF3a:I35T,ORF8:Q27*,ORF8:H40Y,ORF8:R52I,ORF8:Y73C,S:S98F,S:D138H,S:N501Y,S:A570D,S:D614G,S:P681H,S:T716I,S:S982A,S:D1118H	6	235
hCoV-19/Mexico/HID-INMEGEN-05-04-37/2021	EPI_ISL_2349913	-	B.1.1.7	GRY	37	C241T,C913T,C2453T,C3037T,C3267T,C3784T,C3924T,C5388A,C5986T,T6954C,A7479G,C9611T,C14408T,C14676T,C15279T,T16176C,C21855T,G21974C,A23063T,C23271A,A23403G,C23604A,C23709T,T24506G,G24914C,T25496C,C27972T,C28011T,G28048T,A28111G,T28282A,A28295G,G28739T,G28881A,G28882A,G28883C,C28977T	27	N:N8D,N:A156S,N:R203K,N:G204R,N:S235F,ORF1a:L730F,ORF1a:T1001I,ORF1a:P1220L,ORF1a:A1708D,ORF1a:I2230T,ORF1a:N2405S,ORF1a:L3116F,ORF1b:P314L,ORF3a:I35T,ORF8:Q27*,ORF8:H40Y,ORF8:R52I,ORF8:Y73C,S:S98F,S:D138H,S:N501Y,S:A570D,S:D614G,S:P681H,S:T716I,S:S982A,S:D1118H	6	3
hCoV-19/Mexico/HID-INMEGEN-05-05-141/2021	EPI_ISL_2444484	-	B.1.1.7	GRY	36	C241T,C913T,C2453T,C3037T,C3267T,C3784T,C5388A,C5986T,T6954C,A7479G,C9611T,C14408T,C14676T,C15279T,T16176C,C18959T,C21855T,G21974C,A23063T,C23271A,A23403G,C23604A,C23709T,T24506G,G24914C,C27972T,G28048T,A28111G,G28280C,A28281T,T28282A,A28295G,G28881A,G28882A,G28883C,C28977T	25	N:D3L,N:N8D,N:R203K,N:G204R,N:S235F,ORF1a:L730F,ORF1a:T1001I,ORF1a:A1708D,ORF1a:I2230T,ORF1a:N2405S,ORF1a:L3116F,ORF1b:P314L,ORF1b:A1831V,ORF8:Q27*,ORF8:R52I,ORF8:Y73C,S:S98F,S:D138H,S:N501Y,S:A570D,S:D614G,S:P681H,S:T716I,S:S982A,S:D1118H	6	0
hCoV-19/Mexico/HID-INMEGEN-05-05-71/2021	EPI_ISL_2444449	-	B.1.1.7	G	30	C241T,C913T,C3037T,C3267T,C5388A,C5986T,T6954C,A13768T,C14408T,C14676T,T15096C,C15279T,T16176C,C23271A,A23403G,C23520T,C23604A,C23709T,T24506G,G24914C,C26606T,C27972T,G28048T,A28095T,A28111G,G28280C,A28281T,T28282A,G28300T,C28977T	20	N:D3L,N:Q9H,N:S235F,ORF1a:T1001I,ORF1a:A1708D,ORF1a:I2230T,ORF1b:M101L,ORF1b:P314L,ORF8:Q27*,ORF8:R52I,ORF8:K68*,ORF8:Y73C,ORF9b:S6I,S:A570D,S:D614G,S:A653V,S:P681H,S:T716I,S:S982A,S:D1118H	6	1237
hCoV-19/Mexico/HID-INMEGEN-05-06-371/2021	EPI_ISL_2603814	-	B.1.1.7	GRY	34	C241T,C913T,C3037T,C3267T,A3492G,C5388A,C5986T,T6954C,C14408T,C14676T,C15279T,T16176C,C18555T,C21639A,A23063T,C23271A,A23403G,C23604A,C23709T,C24374T,T24506G,C24616T,G24914C,C27972T,G28048T,A28111G,G28280C,A28281T,T28282A,G28881A,G28882A,	21	N:D3L,N:R203K,N:G204R,N:S235F,ORF1a:T1001I,ORF1a:N1076S,ORF1a:A1708D,ORF1a:I2230T,ORF1b:P314L,ORF8:Q27*,ORF8:R52I,ORF8:Y73C,S:P26H,S:N501Y,S:A570D,S:D614G,S:P681H,S:T716I,S:L938F,S:S982A,S:D1118H	6	0
hCoV-19/Mexico/HID-INMEGEN-06-03-153/2021	EPI_ISL_2928491	-	B.1.1.7	GRY	36	C241T,C913T,C2110T,C3037T,C3267T,G4300A,C5388A,C5986T,T6954C,T7984C,C10450T,C14120T,C14408T,C14676T,C15279T,T16176C,C19390T,A23063T,C23271A,A23403G,C23604A,C23709T,T24506G,G24914C,A27237G,C27972T,G28048T,A28095T,A28111G,G28280C,A28281T,T28282A,G28881A,G28882A,G28883C,C28977T	22	N:D3L,N:R203K,N:G204R,N:S235F,ORF1a:T1001I,ORF1a:A1708D,ORF1a:I2230T,ORF1b:P218L,ORF1b:P314L,ORF1b:P1975S,ORF8:Q27*,ORF8:R52I,ORF8:K68*,ORF8:Y73C,ORF8:F120L,S:N501Y,S:A570D,S:D614G,S:P681H,S:T716I,S:S982A,S:D1118H	6	0
hCoV-19/Mexico/HID-INMEGEN-06-03-173/2021	EPI_ISL_2928492	-	B.1.1.7	GRY	35	C241T,C913T,C2110T,C3037T,C3267T,G4300A,C5388A,C5986T,T6954C,T7984C,C10450T,C14120T,C14408T,C14676T,C15279T,T16176C,A23063T,C23271A,A23403G,C23604A,C23709T,T24506G,G24914C,A27237G,C27972T,G28048T,A28095T,A28111G,G28280C,A28281T,T28282A,G28881A,G28882A,G28883C,C28977T	21	N:D3L,N:R203K,N:G204R,N:S235F,ORF1a:T1001I,ORF1a:A1708D,ORF1a:I2230T,ORF1b:P218L,ORF1b:P314L,ORF8:Q27*,ORF8:R52I,ORF8:K68*,ORF8:Y73C,ORF8:F120L,S:N501Y,S:A570D,S:D614G,S:P681H,S:T716I,S:S982A,S:D1118H	6	389
hCoV-19/Mexico/HID-INMEGEN-06-03-186/2021	EPI_ISL_2810168	-	B.1.1.7	GRY	38	G91T,C241T,C913T,C2453T,C3037T,C3267T,C3784T,C5388A,C5986T,T6954C,A7479G,C9611T,C12439T,C14408T,C14676T,C15279T,T16176C,C21855T,G21974C,A23063T,C23271A,A23403G,C23604A,C23709T,T24506G,G24914C,C27972T,G28048T,A28111G,G28280C,A28281T,T28282A,A28295G,G28881A,G28882A,G28883C,C28977T,G29447T	25	N:D3L,N:N8D,N:R203K,N:G204R,N:S235F,N:V392L,ORF1a:L730F,ORF1a:T1001I,ORF1a:A1708D,ORF1a:I2230T,ORF1a:N2405S,ORF1a:L3116F,ORF1b:P314L,ORF8:Q27*,ORF8:R52I,ORF8:Y73C,S:S98F,S:D138H,S:N501Y,S:A570D,S:D614G,S:P681H,S:T716I,S:S982A,S:D1118H	6	1169
hCoV-19/Mexico/JAL-InDRE_F11689_S895/2021	EPI_ISL_1423505	-	B.1.1.7	GRY	35	C241T,C913T,C3037T,C3267T,C5388A,C5986T,T6954C,A12162G,C14408T,C14676T,C15279T,C15720T,T16176C,A17615G,A21137G,A23063T,C23271A,A23403G,C23604A,C23709T,T24506G,G24914C,G25135T,C26110T,C27972T,G28048T,A28111G,G28280C,A28281T,T28282A,G28628C,G28881A,G28882A,G28883C,C28977T	24	N:D3L,N:A119P,N:R203K,N:G204R,N:S235F,ORF1a:T1001I,ORF1a:A1708D,ORF1a:I2230T,ORF1a:Q3966R,ORF1b:P314L,ORF1b:K1383R,ORF1b:K2557R,ORF3a:P240S,ORF8:Q27*,ORF8:R52I,ORF8:Y73C,S:N501Y,S:A570D,S:D614G,S:P681H,S:T716I,S:S982A,S:D1118H,S:K1191N	6	0
hCoV-19/Mexico/JAL-InDRE_FB13392_S1763/2021	EPI_ISL_2101902	-	B.1.1.7	GRY	36	C241T,C823T,C913T,C2110T,G3004T,C3037T,C3267T,C5388A,C5986T,T6954C,G7042T,C14120T,C14408T,C14676T,C15279T,T16176C,A23063T,C23271A,A23403G,C23604A,C23709T,T24506G,G24914C,T25475C,C26110T,C27972T,G28048T,A28095T,A28111G,G28280C,A28281T,T28282A,G28881A,G28882A,G28883C,C28977T	24	N:D3L,N:R203K,N:G204R,N:S235F,ORF1a:E913D,ORF1a:T1001I,ORF1a:A1708D,ORF1a:I2230T,ORF1a:M2259I,ORF1b:P218L,ORF1b:P314L,ORF3a:F28S,ORF3a:P240S,ORF8:Q27*,ORF8:R52I,ORF8:K68*,ORF8:Y73C,S:N501Y,S:A570D,S:D614G,S:P681H,S:T716I,S:S982A,S:D1118H	6	0
hCoV-19/Mexico/JAL-InDRE_FB13396_S1730/2021	EPI_ISL_2101869	-	B.1.1.7	GRY	36	C241T,C913T,G1738T,C3037T,C3267T,G4255A,C5388A,C5986T,T6954C,G11507A,C14408T,C14676T,C15279T,T16176C,A23063T,C23271A,A23403G,C23604A,C23709T,T24506G,G24914C,A25336C,C25702T,C27972T,G28048T,A28111G,G28280C,A28281T,T28282A,G28541T,G28881A,G28882A,G28883C,G28908T,C28928T,C28977T	25	N:D3L,N:A90S,N:R203K,N:G204R,N:G212V,N:L219F,N:S235F,ORF1a:T1001I,ORF1a:A1708D,ORF1a:I2230T,ORF1a:V3748I,ORF1b:P314L,ORF3a:P104S,ORF8:Q27*,ORF8:R52I,ORF8:Y73C,ORF9b:E86D,S:N501Y,S:A570D,S:D614G,S:P681H,S:T716I,S:S982A,S:D1118H,S:E1258D	6	176
hCoV-19/Mexico/JAL-InDRE_FB13967_S1756/2021	EPI_ISL_2101895	-	B.1.1.7	GRY	34	C241T,C913T,C2453T,C3037T,C3267T,C5388A,C5986T,T6954C,C9611T,C14408T,C14676T,C15279T,T16176C,C21855T,G21974C,A23063T,C23271A,A23403G,C23604A,C23709T,T24506G,G24914C,C27643T,C27972T,G28048T,A28111G,G28280C,A28281T,T28282A,A28295G,G28881A,G28882A,G28883C,C28977T	24	N:D3L,N:N8D,N:R203K,N:G204R,N:S235F,ORF1a:L730F,ORF1a:T1001I,ORF1a:A1708D,ORF1a:I2230T,ORF1a:L3116F,ORF1b:P314L,ORF7a:P84S,ORF8:Q27*,ORF8:R52I,ORF8:Y73C,S:S98F,S:D138H,S:N501Y,S:A570D,S:D614G,S:P681H,S:T716I,S:S982A,S:D1118H	6	0
hCoV-19/Mexico/JAL-InDRE_FB13972_S1752/2021	EPI_ISL_2101891	-	B.1.1.7	GRY	35	C241T,C913T,C2453T,C3037T,C3267T,C5388A,C5986T,T6954C,C9611T,C14408T,C14676T,C15279T,T16176C,C21855T,G21974C,A23063T,C23271A,A23403G,C23604A,C23709T,T24506G,C24748T,G24914C,C25516T,C27972T,G28048T,A28111G,G28280C,A28281T,T28282A,A28295G,G28881A,G28882A,G28883C,C28977T	24	N:D3L,N:N8D,N:R203K,N:G204R,N:S235F,ORF1a:L730F,ORF1a:T1001I,ORF1a:A1708D,ORF1a:I2230T,ORF1a:L3116F,ORF1b:P314L,ORF3a:P42S,ORF8:Q27*,ORF8:R52I,ORF8:Y73C,S:S98F,S:D138H,S:N501Y,S:A570D,S:D614G,S:P681H,S:T716I,S:S982A,S:D1118H	6	0
hCoV-19/Mexico/JAL-InDRE_FB14113_S1761/2021	EPI_ISL_2101900	-	B.1.1.7	GRY	35	C241T,C913T,C2453T,C3037T,C3267T,C5388A,C5986T,C6310T,T6954C,C9611T,C14408T,C14676T,C15279T,T16176C,C21855T,G21974C,A23063T,C23271A,A23403G,C23604A,C23709T,T24506G,G24914C,C27643T,C27972T,G28048T,A28111G,G28280C,A28281T,T28282A,A28295G,G28881A,G28882A,G28883C,C28977T	24	N:D3L,N:N8D,N:R203K,N:G204R,N:S235F,ORF1a:L730F,ORF1a:T1001I,ORF1a:A1708D,ORF1a:I2230T,ORF1a:L3116F,ORF1b:P314L,ORF7a:P84S,ORF8:Q27*,ORF8:R52I,ORF8:Y73C,S:S98F,S:D138H,S:N501Y,S:A570D,S:D614G,S:P681H,S:T716I,S:S982A,S:D1118H	6	0

hCoV-19/Mexico/JAL-InDRE_FB14278_S1766/2021	EPI_ISL_2101905	-	B.1.1.7	GRY	33	C241T,C913T,C3037T,C3267T,A3492G,C5388A,C5986T,T6954C,C14408T,C14676T,C15279T,T16176C,C21639A,G22021T,A23063T,C23271A,A23403G,C23604A,C23709T,C24374T,T24506G,G24914C,A25336C,C27972T,G28048T,A28111G,G28280C,A28281T,T28282A,G28881A,G28882A,	23	N:D3L,N:R203K,N:G204R,N:S235F,ORF1a:T1001I,ORF1a:N1076S,ORF1a:A1708D,ORF1a:I2230T,ORF1b:P314L,ORF8:Q27*,ORF8:R52I,ORF8:Y73C,S:P26H,S:M153I,S:N501Y,S:A570D,S:D614G,S:P681H,S:T716I,S:L938F,S:S982A,S:D1118	15	0
hCoV-19/Mexico/JAL-InDRE_FB14563_S2044/2021	EPI_ISL_2283686	-	B.1.1.7	GRY	41	C241T,C913T,C2110T,G3004T,C3037T,C3267T,G3728T,C5388A,C5986T,T6954C,G7042T,C9893T,C11824T,C14120T,C14408T,C14676T,C15279T,T16176C,A23063T,C23271A,A23403G,C23604A,C23709T,T24506G,G24914C,T25518C,G25644T,G27948T,C27972T,T28014G,G28048T,C28093T,A28095T,A28111G,G28280C,A28281T,T28282A,G28881A,G28882A,G28883C,C28977T	27	N:D3L,N:R203K,N:G204R,N:S235F,ORF1a:E913D,ORF1a:T1001I,ORF1a:G1155C,ORF1a:A1708D,ORF1a:I2230T,ORF1a:M2259I,ORF1a:L3210F,ORF1b:P218L,ORF1b:P314L,ORF8:E19*,ORF8:Q27*,ORF8:F41V,ORF8:R52I,ORF8:S67F,ORF8:K68*,ORF8:Y73C,S:N501Y,S:A570D,S:D614G,S:P681H,S:T716I,S:S982A,S:D111	6	0
hCoV-19/Mexico/JAL-InDRE_FB16243_S2434/2021	EPI_ISL_2455958	-	B.1.1.7	GRY	43	C241T,C673T,C913T,C3037T,C3267T,C5388A,C5986T,T6661C,T6954C,C8318A,A12162G,C13665T,C14408T,C14676T,C15279T,C15720T,T16176C,A17615G,C18747T,C20844T,A21137G,A23063T,C23271A,A23403G,C23604A,C23709T,T24506G,G24914C,G25135T,A25336C,G25606T,C26110T,C27972T,G28048T,A28111G,G28280C,A28281T,T28282A,G28881A,G28882A,G28883C,C289	26	N:D3L,N:R203K,N:G204R,N:S235F,ORF1a:T1001I,ORF1a:A1708D,ORF1a:I2230T,ORF1a:P2685T,ORF1a:Q3966R,ORF1b:P314L,ORF1b:K1383R,ORF1b:K2557R,ORF3a:A72S,ORF3a:P240S,ORF8:Q27*,ORF8:R52I,ORF8:Y73C,S:N501Y,S:A570D,S:D614G,S:P681H,S:T716I,S:S982A,S:D1118H,S:K1191N,S:E1258D	6	56
hCoV-19/Mexico/JAL-InDRE_FB16511_S2741/2021	EPI_ISL_2533767	-	B.1.1.7	GRY	34	C241T,C913T,C2453T,C3037T,C3267T,C5388A,C5986T,T6954C,C9611T,C14408T,C14676T,C15279T,T16176C,C21855T,G21974C,A23063T,C23271A,A23403G,C23604A,C23709T,T24506G,G24914C,C25516T,C27972T,G28048T,A28111G,G28280C,A28281T,T28282A,A28295G,G28881A,G28882A,G28883C,C28977T	24	N:D3L,N:N8D,N:R203K,N:G204R,N:S235F,ORF1a:L730F,ORF1a:T1001I,ORF1a:A1708D,ORF1a:I2230T,ORF1a:L3116F,ORF1b:P314L,ORF3a:P42S,ORF8:Q27*,ORF8:R52I,ORF8:Y73C,S:S98F,S:D138H,S:N501Y,S:A570D,S:D614G,S:P681H,S:T716I,S:S982A,S:D1118H	6	6
hCoV-19/Mexico/JAL-InDRE_FB16739_S2776/2021	EPI_ISL_2533802	-	B.1.1.7	GRY	36	C241T,C913T,C3037T,C3267T,T3537C,C5388A,C5986T,T6954C,C9298T,C14408T,C14676T,C15279T,T16176C,A23063T,C23271A,A23403G,C23604A,C23709T,C23758T,C24374T,T24506G,G24914C,C27972T,G28048T,A28111G,G28280C,A28281T,T28282A,G28881A,G28882A,G28883C,G28904T,C28977T,G29348T,C29367T,C29762T	23	N:D3L,N:R203K,N:G204R,N:A211S,N:S235F,N:A359S,N:P365L,ORF1a:T1001I,ORF1a:I1091T,ORF1a:A1708D,ORF1a:I2230T,ORF1b:P314L,ORF8:Q27*,ORF8:R52I,ORF8:Y73C,S:N501Y,S:A570D,S:D614G,S:P681H,S:T716I,S:L938F,S:S982A,S:D1118H	6	0
hCoV-19/Mexico/JAL-InDRE_FB18117_S3958/2021	EPI_ISL_2858972	-	B.1.1.7	GRY	40	C241T,C292T,C913T,C3037T,C3267T,C5388A,C5986T,T6954C,T9418C,C12357T,C14408T,C14676T,C15279T,T16176C,A17615G,A23063T,C23271A,A23403G,C23604A,C23709T,T24506G,G24914C,G25177A,G25409C,G26730C,C27389T,C27635T,C27972T,G28048T,A28111G,G28280C,A28281T,T28282A,C28453T,G28881A,G28882A,G28883C,C28977T,C29353T,G29757T	24	M:V70L,N:D3L,N:R203K,N:G204R,N:S235F,ORF1a:T1001I,ORF1a:A1708D,ORF1a:I2230T,ORF1a:T4031I,ORF1b:P314L,ORF1b:K1383R,ORF3a:R6T,ORF7a:S81L,ORF8:Q27*,ORF8:R52I,ORF8:Y73C,ORF9b:A57V,S:N501Y,S:A570D,S:D614G,S:P681H,S:T716I,S:S982A,S:D1118H	6	0
hCoV-19/Mexico/MEX-InDRE_FB17389_S3484/2021	EPI_ISL_2778999	-	B.1.1.7	GRY	34	C241T,C245T,C913T,C3037T,C3267T,A3492G,C5388A,C5986T,T6954C,C14408T,C14676T,C15279T,T16176C,C21639A,A23063T,C23271A,A23403G,C23604A,C23709T,C24374T,T24506G,G24914C,A25336C,G25337C,C27972T,G28048T,A28111G,G28280C,A28281T,T28282A,G28881A,G2	23	N:D3L,N:R203K,N:G204R,N:S235F,ORF1a:T1001I,ORF1a:N1076S,ORF1a:A1708D,ORF1a:I2230T,ORF1b:P314L,ORF8:Q27*,ORF8:R52I,ORF8:Y73C,S:P26H,S:N501Y,S:A570D,S:D614G,S:P681H,S:T716I,S:L938F,S:S982A,S:D1118H,S:E1258	6	0
hCoV-19/Mexico/MEX-InDRE_FD68596_S2471/2021	EPI_ISL_2455992	-	B.1.1.7	GRY	36	C241T,C913T,C2453T,C3037T,C3267T,C5388A,C5986T,T6954C,C9611T,A10761G,A12358T,C14408T,C14676T,C15279T,T16176C,C21855T,A23063T,C23271A,A23403G,C23604A,C23709T,T24506G,G24914C,A25336C,C25886T,C27972T,G28048T,A28111G,G28280C,A28281T,T28282A,A28295G,G28881A,G28882A,G28883C,C28977T	25	N:D3L,N:N8D,N:R203K,N:G204R,N:S235F,ORF1a:L730F,ORF1a:T1001I,ORF1a:A1708D,ORF1a:I2230T,ORF1a:L3116F,ORF1a:K3499R,ORF1b:P314L,ORF3a:S165F,ORF8:Q27*,ORF8:R52I,ORF8:Y73C,S:S98F,S:N501Y,S:A570D,S:D614G,S:P681H,S:T716I,S:S982A,S:D1118H,S:E1258D	6	24
hCoV-19/Mexico/MEX-InDRE_FD71099_S2783/2021	EPI_ISL_2533809	-	B.1.1.7	GRY	36	C241T,C913T,G2144A,C2453T,C3037T,C3267T,C5388A,C5986T,T6954C,C9611T,C14408T,C14676T,C15279T,T16176C,G18651T,C21855T,G21974C,A23063T,C23271A,A23403G,C23604A,C23709T,T24506G,G24914C,C27972T,G28048T,A28111G,G28280C,A28281T,T28282A,A28295G,G28881A,G28882A,G28883C,C28977T,G29705T	25	N:D3L,N:N8D,N:R203K,N:G204R,N:S235F,ORF1a:V627I,ORF1a:L730F,ORF1a:T1001I,ORF1a:A1708D,ORF1a:I2230T,ORF1a:L3116F,ORF1b:P314L,ORF1b:E1728D,ORF8:Q27*,ORF8:R52I,ORF8:Y73C,S:S98F,S:D138H,S:N501Y,S:A570D,S:D614G,S:P681H,S:T716I,S:S982A,S:D1118H	6	0
hCoV-19/Mexico/MEX-INMEGEN-05-01-227/2021	EPI_ISL_2105747	-	B.1.1.7	G	37	C241T,C913T,C999T,C3037T,C3267T,C5388A,C5986T,T6954C,C7299T,C12576T,A13768T,C14408T,C14676T,T15096C,C15279T,T16176C,C19862T,C21077T,A23063T,C23271A,A23403G,C23604A,C23709T,T24506G,G24914C,C26606T,G27281T,C27972T,G28048T,A28095T,A28111G,G28280C,A28281T,T28282A,A28877T,C28977T,A29491G	24	N:D3L,N:S235F,ORF1a:S245F,ORF1a:T1001I,ORF1a:A1708D,ORF1a:I2230T,ORF1a:A2345V,ORF1a:A4104V,ORF1b:M101L,ORF1b:P314L,ORF1b:A2132V,ORF1b:T2537I,ORF6:W27L,ORF8:Q27*,ORF8:R52I,ORF8:K68*,ORF8:Y73C,S:N501Y,S:A570D,S:D614G,S:P681H,S:T716I,S:S982A,S:D1118H	6	6
hCoV-19/Mexico/MEX-INMEGEN-05-01-240/2021	EPI_ISL_2105759	-	B.1.1.7	GRY	34	C241T,C913T,G2890A,C3037T,C3267T,C5388A,C5986T,C6196T,T6954C,A8848G,C9073T,C14408T,C14676T,C15279T,T16176C,T19624C,A23063T,C23271A,A23403G,C23604A,C23709T,T24506G,G24914C,C27972T,G28048T,A28111G,G28280C,A28281T,T28282A,G28881A,G28882A,G288	18	N:D3L,N:R203K,N:G204R,N:S235F,ORF1a:T1001I,ORF1a:A1708D,ORF1a:I2230T,ORF1b:P314L,ORF8:Q27*,ORF8:R52I,ORF8:Y73C,S:N501Y,S:A570D,S:D614G,S:P681H,S:T716I,S:S982A,S:D1118H	6	0
hCoV-19/Mexico/MEX-INMEGEN-05-01-256/2021	EPI_ISL_2178757	-	B.1.1.7	G	37	C241T,C913T,C3037T,C3267T,C5388A,C5986T,G6647A,T6954C,C14408T,C14676T,T15096C,C15279T,T16176C,C17676T,C18183T,C19032T,C19524T,A21519C,C23271A,A23403G,C23604A,C23709T,A24070T,A24092G,T24506G,G24914C,C25624T,C27737T,C27972T,G28048T,A28095T,A28111G,G28280C,A28281T,T28282A,C28977T,G29543T	21	N:D3L,ORF1a:T1001I,ORF1a:A1708D,ORF1a:A2128T,ORF1a:I2230T,ORF1b:P314L,ORF1b:R2684S,ORF3a:H78Y,ORF7a:T115I,ORF8:Q27*,ORF8:R52I,ORF8:K68*,ORF8:Y73C,S:A570D,S:D614G,S:P681H,S:T716I,S:Q836H,S:I844V,S:S982A,S:D1118H	7	1233
hCoV-19/Mexico/MEX-INMEGEN-05-01-266/2021	EPI_ISL_2105778	-	B.1.1.7	GRY	34	C241T,C913T,C2453T,C3037T,C3267T,C5388A,C5986T,T6954C,C9611T,C14408T,C14676T,C15279T,T16176C,C21855T,G21974C,A23063T,C23271A,A23403G,A23503G,C23604A,C23709T,T24506G,G24914C,C27972T,G28048T,A28111G,G28280C,A28281T,T28282A,A28295G,G28881A,G	23	N:D3L,N:N8D,N:R203K,N:G204R,N:S235F,ORF1a:L730F,ORF1a:T1001I,ORF1a:A1708D,ORF1a:I2230T,ORF1a:L3116F,ORF1b:P314L,ORF8:Q27*,ORF8:R52I,ORF8:Y73C,S:S98F,S:D138H,S:N501Y,S:A570D,S:D614G,S:P681H,S:T716I,S:S982	6	0
hCoV-19/Mexico/MEX-INMEGEN-05-01-278/2021	EPI_ISL_2105790	-	B.1.1.7	GRY	33	C241T,C913T,C3037T,C3267T,C5388A,C5986T,C6196T,T6954C,A8848G,C14408T,C14676T,C15279T,T16176C,T19624C,A23063T,C23271A,A23403G,C23604A,C23709T,T24506G,G24914C,G25217T,C27972T,G28048T,A28111G,G28280C,A28281T,T28282A,G28881A,G28882A,G28883C,C2	19	N:D3L,N:R203K,N:G204R,N:S235F,ORF1a:T1001I,ORF1a:A1708D,ORF1a:I2230T,ORF1b:P314L,ORF8:Q27*,ORF8:R52I,ORF8:Y73C,S:N501Y,S:A570D,S:D614G,S:P681H,S:T716I,S:S982A,S:D1118H,S:G1219C	6	0
hCoV-19/Mexico/MEX-INMEGEN-05-01-283/2021	EPI_ISL_2105795	-	B.1.1.7	GRY	39	C241T,C913T,G960A,C2040T,C3037T,C3267T,C5388A,C5986T,T6954C,G8102A,C14408T,C14676T,T15096C,C15279T,T16176C,C19164T,C20104T,G21893A,A23063T,C23271A,A23403G,C23604A,C23709T,T24506G,G24914C,C25096T,G25437C,G27375A,C27972T,G28048T,A28095T,A28111G,G28280C,A28281T,T28282A,G28881A,G28882A,G28883C,C28977T	26	N:D3L,N:R203K,N:G204R,N:S235F,ORF1a:R232H,ORF1a:T592I,ORF1a:T1001I,ORF1a:A1708D,ORF1a:I2230T,ORF1a:V2613I,ORF1b:P314L,ORF1b:L2213F,ORF3a:L15F,ORF6:M58I,ORF8:Q27*,ORF8:R52I,ORF8:K68*,ORF8:Y73C,S:D111N,S:N501Y,S:A570D,S:D614G,S:P681H,S:T716I,S:S982A,S:D1118H	6	66
hCoV-19/Mexico/MEX-INMEGEN-05-01-309/2021	EPI_ISL_2105817	-	B.1.1.7	GRY	41	C241T,T856C,C913T,C2110T,C3037T,C3267T,G4255A,C5388A,C5986T,C6388T,T6954C,C11750T,C12525T,C14120T,C14408T,C14676T,C15279T,C15952T,T16176C,C19186T,A23063T,G23120T,C23271A,A23403G,C23604A,C23709T,T24506G,G24914C,G25126A,T27462C,C27972T,G28048T,A28095T,A28111G,G28280C,A28281T,T28282A,G28881A,G28882A,G28883C,C28977T	23	N:D3L,N:R203K,N:G204R,N:S235F,ORF1a:T1001I,ORF1a:A1708D,ORF1a:I2230T,ORF1a:L3829F,ORF1a:T4087I,ORF1b:P218L,ORF1b:P314L,ORF8:Q27*,ORF8:R52I,ORF8:K68*,ORF8:Y73C,S:N501Y,S:A520S,S:A570D,S:D614G,S:P681H,S:T716I,S:S982A,S:D1118H	6	0
hCoV-19/Mexico/MEX-INMEGEN-05-01-314/2021	EPI_ISL_2105822	-	B.1.1.7	GRY	35	C241T,C913T,C2453T,C3037T,G3259T,C3267T,C5388A,C5986T,T6954C,C9611T,G13921T,C14408T,C14676T,C15279T,T16176C,C21855T,G21974C,A23063T,C23271A,A23403G,C23604A,C23709T,T24506G,G24914C,C27972T,G28048T,A28111G,G28280C,A28281T,T28282A,A28295G,G28881A,G28882A,G28883C,C28977T	25	N:D3L,N:N8D,N:R203K,N:G204R,N:S235F,ORF1a:L730F,ORF1a:Q998H,ORF1a:T1001I,ORF1a:A1708D,ORF1a:I2230T,ORF1a:L3116F,ORF1b:D152Y,ORF1b:P314L,ORF8:Q27*,ORF8:R52I,ORF8:Y73C,S:S98F,S:D138H,S:N501Y,S:A570D,S:D614G,S:P681H,S:T716I,S:S982A,S:D1118H	6	0
hCoV-19/Mexico/MEX-INMEGEN-05-01-326/2021	EPI_ISL_2105832	-	B.1.1.7	GRY	43	C203T,C241T,C913T,G1820A,G2588A,C3037T,C3267T,C4456T,C5388A,C5986T,T6954C,A12162G,C14408T,C14676T,C15279T,C15720T,T16176C,C16338T,A17615G,A21137G,C22858T,A23063T,C23248T,C23271A,A23403G,C23604A,G23608T,C23709T,T24506G,G24914C,G25135T,C27972T,G28001A,G28048T,A28111G,G28280C,A28281T,T28282A,G28632T,G28881A,G28882A,G28	25	N:D3L,N:G120V,N:R203K,N:G204R,N:S235F,ORF1a:G519S,ORF1a:E775K,ORF1a:T1001I,ORF1a:A1708D,ORF1a:I2230T,ORF1a:Q3966R,ORF1b:P314L,ORF1b:K1383R,ORF1b:K2557R,ORF8:Q27*,ORF8:R52I,ORF8:Y73C,S:N501Y,S:A570D,S:D614G,S:P681H,S:T716I,S:S982A,S:D1118H,S:K1191N	6	0
hCoV-19/Mexico/MEX-INMEGEN-05-01-345/2021	EPI_ISL_2105850	-	B.1.1.7	GRY	42	C241T,C913T,C3037T,C3267T,C5388A,C5944T,C5986T,G6647A,T6954C,C14408T,C14676T,T15096C,C15279T,T16176C,C17676T,C18183T,C19524T,C21234T,A21519C,G22217A,A23063T,C23271A,A23403G,C23604A,C23709T,A24070T,T24506G,G24914C,C25624T,C27972T,G28048T,A28095T,A28111G,G28280C,A28281T,T28282A,G28436T,G28881A,G28882A,G28883C,G28884C,C	25	N:D3L,N:A55S,N:R203K,N:G204P,N:S235F,ORF1a:T1001I,ORF1a:A1708D,ORF1a:A2128T,ORF1a:I2230T,ORF1b:P314L,ORF1b:R2684S,ORF3a:H78Y,ORF8:Q27*,ORF8:R52I,ORF8:K68*,ORF8:Y73C,S:G219S,S:N501Y,S:A570D,S:D614G,S:P681H,S:T716I,S:Q836H,S:S982A,S:D1118H	6	0
hCoV-19/Mexico/MEX-INMEGEN-05-01-349/2021	EPI_ISL_2105853	-	B.1.1.7	GRY	40	C222T,C241T,C913T,C2110T,C3037T,C3267T,C5388A,C5934T,C5986T,T6954C,G7042T,C13517T,C14120T,C14408T,C14676T,C14697T,C15279T,T16176C,G16900T,C17762T,A23063T,C23271A,A23403G,C23604A,C23709T,T24506G,G24914C,T25180C,G26416T,C27972T,G28048T,A28095T,A28111G,G28280C,A28281T,T28282A,G28881A,G28882A,G28883C,C28977T	26	E:V58F,N:D3L,N:R203K,N:G204R,N:S235F,ORF1a:T1001I,ORF1a:A1708D,ORF1a:T1890I,ORF1a:I2230T,ORF1a:M2259I,ORF1b:T17I,ORF1b:P218L,ORF1b:P314L,ORF1b:G1145C,ORF1b:A1432V,ORF8:Q27*,ORF8:R52I,ORF8:K68*,ORF8:Y73C,S:N501Y,S:A570D,S:D614G,S:P681H,S:T716I,S:S982A,S:D1118H	6	0

hCoV-19/Mexico/MEX-INMEGEN-05-01-366/2021	EPI_ISL_2105867	-	B.1.1.7	GR	40	C241T,C913T,C2110T,C2918T,C3037T,C3267T,C5388A,C5986T,T6954C,G7042T,T9519C,A10195G,C10279T,C11514T,C14120T,C14408T,C14676T,C15279T,T16176C,C16887T,G17427T,C20313T,C23271A,A23403G,C23604A,C23709T,T24506G,G24914C,C27972T,G28048T,G28075T,A28095T,A28111G,G28280C,A28281T,T28282A,G28881A,G28882A,G28883C,C28977T	24	N:D3L,N:R203K,N:G204R,N:S235F,ORF1a:P885S,ORF1a:T1001I,ORF1a:A1708D,ORF1a:I2230T,ORF1a:M2259I,ORF1a:F3085S,ORF1a:T3750I,ORF1b:P218L,ORF1b:P314L,ORF8:Q27*,ORF8:R52I,ORF8:C61F,ORF8:K68*,ORF8:Y73C,S:A570D,S:D614G,S:P681H,S:T716I,S:S982A,S:D1118H	6	550
hCoV-19/Mexico/MEX-INMEGEN-05-02-1/2021	EPI_ISL_2230865	-	B.1.1.7	GRY	42	G174T,G210T,C241T,C913T,C1420T,C2453T,C3037T,C3267T,C5388A,C5986T,C6445T,T6954C,T9016C,C9661T,C10125T,G13514A,C14408T,T14565C,C14676T,C15279T,T16176C,T17803G,G21777T,A23063T,C23271A,A23403G,C23604A,C23709T,T24506G,G24914C,C27883T,C27972T,G28048T,A28111G,G28280C,A28281T,T28282A,G28881A,G28882A,G28883C,C28977T,G29212T	24	N:D3L,N:R203K,N:G204R,N:S235F,ORF1a:L730F,ORF1a:T1001I,ORF1a:A1708D,ORF1a:I2230T,ORF1a:T3287I,ORF1b:G16D,ORF1b:P314L,ORF1b:S1446A,ORF7b:A43V,ORF8:Q27*,ORF8:R52I,ORF8:Y73C,S:G72V,S:N501Y,S:A570D,S:D614G,S:P681H,S:T716I,S:S982A,S:D1118H	6	0
hCoV-19/Mexico/MEX-INMEGEN-05-02-104/2021	EPI_ISL_2230871	-	B.1.1.7	GRY	36	C241T,C913T,C3037T,C3267T,C5388A,C5884T,C5986T,C6196T,T6954C,A8848G,C14408T,C14676T,C15279T,T16176C,C19017T,A23063T,C23271A,A23403G,C23604A,C23709T,T24506G,G24914C,G25217T,G26727T,C27972T,G28048T,A28111G,G28280C,A28281T,T28282A,G28881A,G28882A,G28883C,C28977T,G29260T,G29715T	20	M:A69S,N:D3L,N:R203K,N:G204R,N:S235F,ORF1a:T1001I,ORF1a:A1708D,ORF1a:I2230T,ORF1b:P314L,ORF8:Q27*,ORF8:R52I,ORF8:Y73C,S:N501Y,S:A570D,S:D614G,S:P681H,S:T716I,S:S982A,S:D1118H,S:G1219C	6	24
hCoV-19/Mexico/MEX-INMEGEN-05-02-135/2021	EPI_ISL_2230832	-	B.1.1.7	GRY	34	C241T,C913T,C3037T,C3267T,G4261A,C4423T,C5025T,C5388A,C5986T,T6954C,C14408T,C14676T,T15096C,C15279T,T16176C,G21604T,A23063T,C23271A,A23403G,C23604A,C23709T,T24506G,G24914C,C27972T,G28048T,A28095T,A28111G,G28280C,A28281T,T28282A,G28881A,G28	22	N:D3L,N:R203K,N:G204R,N:S235F,ORF1a:T1001I,ORF1a:S1587L,ORF1a:A1708D,ORF1a:I2230T,ORF1b:P314L,ORF7a:F63H,ORF8:Q27*,ORF8:R52I,ORF8:K68*,ORF8:Y73C,S:Q14H,S:N501Y,S:A570D,S:D614G,S:P681H,S:T716I,S:S982A,S:D1	7	573
hCoV-19/Mexico/MEX-INMEGEN-05-02-162/2021	EPI_ISL_2230883	-	B.1.1.7	GRY	37	C241T,C913T,C3037T,C3267T,C5388A,C5944T,C5986T,T6954C,C9967T,C13585T,C14408T,C14676T,T15096C,C15279T,T16176C,A17344G,G18412T,C22987T,A23063T,C23271A,A23403G,C23604A,C23709T,T24506G,G24914C,C27972T,G28048T,A28095T,A28111G,G28280C,A28281T,T28282A,G28881A,G28882A,G28883C,G28884C,C28977T	21	N:D3L,N:R203K,N:G204P,N:S235F,ORF1a:T1001I,ORF1a:A1708D,ORF1a:I2230T,ORF1b:P314L,ORF1b:I1293V,ORF1b:V1649F,ORF8:Q27*,ORF8:R52I,ORF8:K68*,ORF8:Y73C,S:N501Y,S:A570D,S:D614G,S:P681H,S:T716I,S:S982A,S:D1118H	6	0
hCoV-19/Mexico/MEX-INMEGEN-05-02-166/2021	EPI_ISL_2230877	-	B.1.1.7	GRY	36	C241T,C913T,C2453T,C3037T,C3267T,C5388A,C5986T,T6954C,C9611T,C14408T,C14676T,C15279T,T16176C,C21855T,C21952T,G21974C,A23063T,C23271A,A23403G,C23604A,C23709T,T24506G,G24914C,C25886T,C27972T,G28048T,A28111G,G28280C,A28281T,T28282A,A28295G,G28436T,G28881A,G28882A,G28883C,C28977T	25	N:D3L,N:N8D,N:A55S,N:R203K,N:G204R,N:S235F,ORF1a:L730F,ORF1a:T1001I,ORF1a:A1708D,ORF1a:I2230T,ORF1a:L3116F,ORF1b:P314L,ORF3a:S165F,ORF8:Q27*,ORF8:R52I,ORF8:Y73C,S:S98F,S:D138H,S:N501Y,S:A570D,S:D614G,S:P681H,S:T716I,S:S982A,S:D1118H	6	14
hCoV-19/Mexico/MEX-INMEGEN-05-02-168/2021	EPI_ISL_2230806	-	B.1.1.7	GR	36	C241T,C913T,C2143T,C3037T,C3267T,C5388A,C5986T,T6954C,C14408T,C14676T,T15096C,C15279T,T16176C,C23271A,A23403G,C23604A,C23709T,G24089A,T24506G,G24914C,G25266T,G25785T,G26416T,C27476T,C27972T,G28048T,A28095T,A28111G,G28280C,A28281T,T28282A,G28881A,G28882A,G28883C,C28977T,C29642T	23	E:V58F,N:D3L,N:R203K,N:G204R,N:S235F,ORF1a:T1001I,ORF1a:A1708D,ORF1a:I2230T,ORF1b:P314L,ORF3a:W131C,ORF7a:T28I,ORF8:Q27*,ORF8:R52I,ORF8:K68*,ORF8:Y73C,S:A570D,S:D614G,S:P681H,S:T716I,S:D843N,S:S982A,S:D1118H,S:C1235F	6	1299
hCoV-19/Mexico/MEX-INMEGEN-05-02-175/2021	EPI_ISL_2230825	-	B.1.1.7	GR	37	C241T,C913T,C2453T,C3037T,C3267T,C3784T,C3924T,C5388A,C5986T,T6954C,A7479G,C9611T,C14408T,C14676T,C15279T,T16176C,C21855T,G21974C,C23271A,A23403G,C23604A,C23709T,T24506G,G24914C,T25496C,C27972T,C28011T,G28048T,A28111G,G28280C,A28281T,T28282A,A28295G,G28881A,G28882A,G28883C,C28977T	26	N:D3L,N:N8D,N:R203K,N:G204R,N:S235F,ORF1a:L730F,ORF1a:T1001I,ORF1a:P1220L,ORF1a:A1708D,ORF1a:I2230T,ORF1a:N2405S,ORF1a:L3116F,ORF1b:P314L,ORF3a:I35T,ORF8:Q27*,ORF8:H40Y,ORF8:R52I,ORF8:Y73C,S:S98F,S:D138H,S:A570D,S:D614G,S:P681H,S:T716I,S:S982A,S:D1118H	6	722
hCoV-19/Mexico/MEX-INMEGEN-05-02-187/2021	EPI_ISL_2230879	-	B.1.1.7	GRY	39	G174T,G210T,C241T,C913T,C1420T,C2453T,C3037T,C3267T,C5388A,C5986T,T6954C,T9016C,C9661T,C14408T,T14565C,C14676T,C15279T,T16176C,T17803G,G20931T,A23063T,C23271A,A23403G,C23604A,C23709T,T24506G,G24914C,C27883T,C27972T,G28048T,A28111G,G28280C,A28281T,T28282A,G28727T,G28881A,G28882A,G28883C,C28977T	22	N:D3L,N:A152S,N:R203K,N:G204R,N:S235F,ORF1a:L730F,ORF1a:T1001I,ORF1a:A1708D,ORF1a:I2230T,ORF1b:P314L,ORF1b:S1446A,ORF7b:A43V,ORF8:Q27*,ORF8:R52I,ORF8:Y73C,S:N501Y,S:A570D,S:D614G,S:P681H,S:T716I,S:S982A,S:D1118H	6	1
hCoV-19/Mexico/MEX-INMEGEN-05-02-2/2021	EPI_ISL_2230866	-	B.1.1.7	GRY	36	C241T,C913T,C3037T,C3267T,C5388A,C5986T,C6196T,T6954C,A8848G,C14408T,C14676T,C15279T,T16176C,C18568T,G19656T,A23063T,C23271A,A23403G,C23604A,C23709T,T24506G,G24914C,G25217T,G26428T,G26727T,C27972T,G28048T,A28111G,G28280C,A28281T,T28282A,G28881A,G28882A,G28883C,C28977T,G29715T	23	E:V62F,M:A69S,N:D3L,N:R203K,N:G204R,N:S235F,ORF1a:T1001I,ORF1a:A1708D,ORF1a:I2230T,ORF1b:P314L,ORF1b:L1701F,ORF1b:K2063N,ORF8:Q27*,ORF8:R52I,ORF8:Y73C,S:N501Y,S:A570D,S:D614G,S:P681H,S:T716I,S:S982A,S:D1118H,S:G1219C	6	0
hCoV-19/Mexico/MEX-INMEGEN-05-02-23/2021	EPI_ISL_2230841	-	B.1.1.7	GRY	33	C241T,C913T,C3037T,C3267T,C5388A,C5986T,C6196T,T6954C,A8848G,C14408T,C14676T,C15279T,T16176C,A23063T,C23271A,A23403G,C23604A,C23709T,T24506G,G24914C,G25217T,G26727T,C27972T,G28048T,A28111G,G28280C,A28281T,T28282A,G28881A,G28882A,G28883C,C2	20	M:A69S,N:D3L,N:R203K,N:G204R,N:S235F,ORF1a:T1001I,ORF1a:A1708D,ORF1a:I2230T,ORF1b:P314L,ORF8:Q27*,ORF8:R52I,ORF8:Y73C,S:N501Y,S:A570D,S:D614G,S:P681H,S:T716I,S:S982A,S:D1118H,S:G1219C	6	521
hCoV-19/Mexico/MEX-INMEGEN-05-02-24/2021	EPI_ISL_2230867	-	B.1.1.7	GRY	35	C241T,C913T,T1993C,C2453T,C3037T,C3267T,C5388A,C5986T,T6954C,T8050C,C9611T,C14408T,C14676T,C15279T,T16176C,C21855T,G21974C,A23063T,C23271A,A23403G,C23604A,C23709T,T24506G,G24914C,C27972T,G28048T,A28111G,G28280C,A28281T,T28282A,A28295G,G28881A,G28882A,G28883C,C28977T	23	N:D3L,N:N8D,N:R203K,N:G204R,N:S235F,ORF1a:L730F,ORF1a:T1001I,ORF1a:A1708D,ORF1a:I2230T,ORF1a:L3116F,ORF1b:P314L,ORF8:Q27*,ORF8:R52I,ORF8:Y73C,S:S98F,S:D138H,S:N501Y,S:A570D,S:D614G,S:P681H,S:T716I,S:S982A,S:D1118H	6	38
hCoV-19/Mexico/MEX-INMEGEN-05-02-47/2021	EPI_ISL_2230881	-	B.1.1.7	GRY	39	C241T,C913T,G960A,C2040T,C3037T,C3267T,C5388A,C5986T,T6954C,G8102A,C14408T,C14676T,T15096C,C15279T,T16176C,C19164T,C20104T,G21893A,A23063T,C23271A,A23403G,C23604A,C23709T,T24506G,G24914C,C25096T,G25437C,G27375A,C27972T,G28048T,A28095T,A28111G,G28280C,A28281T,T28282A,G28881A,G28882A,G28883C,C28977T	26	N:D3L,N:R203K,N:G204R,N:S235F,ORF1a:R232H,ORF1a:T592I,ORF1a:T1001I,ORF1a:A1708D,ORF1a:I2230T,ORF1a:V2613I,ORF1b:P314L,ORF1b:L2213F,ORF3a:L15F,ORF6:M58I,ORF8:Q27*,ORF8:R52I,ORF8:K68*,ORF8:Y73C,S:D111N,S:N501Y,S:A570D,S:D614G,S:P681H,S:T716I,S:S982A,S:D1118H	6	0
hCoV-19/Mexico/MEX-INMEGEN-05-03-103/2021	EPI_ISL_2319255	-	Q.3	GRY	41	C203T,C241T,C355T,C913T,C3037T,C3267T,C5183T,C5388A,C5986T,T6954C,T8053C,C9924T,C14408T,C14676T,C15279T,C15933T,T16176C,A17615G,C18452T,G20962A,A21137G,A23063T,C23271A,A23403G,C23604A,C23709T,T24506G,G24914C,G25785T,G26730C,C26768T,C27972T,G28048T,A28111G,G28280C,A28281T,T28282A,G28881A,G28882A,G28883C,C28977T	26	M:V70L,N:D3L,N:R203K,N:G204R,N:S235F,ORF1a:T1001I,ORF1a:P1640S,ORF1a:A1708D,ORF1a:I2230T,ORF1a:A3220V,ORF1b:P314L,ORF1b:K1383R,ORF1b:A1662V,ORF1b:D2499N,ORF1b:K2557R,ORF3a:W131C,ORF8:Q27*,ORF8:R52I,ORF8:Y73C,S:N501Y,S:A570D,S:D614G,S:P681H,S:T716I,S:S982A,S:D1118	6	0
hCoV-19/Mexico/MEX-INMEGEN-05-03-118/2021	EPI_ISL_2319264	-	B.1.1.7	GRY	35	C241T,C913T,C3037T,C3267T,C5388A,C5986T,C6196T,T6954C,A8848G,C14408T,C14676T,C15279T,T16176C,A23063T,C23271A,T23332C,A23403G,C23604A,C23709T,T24506G,G24914C,G25217T,G25538A,G26727T,C27972T,G28048T,A28111G,G28280C,A28281T,T28282A,G28881A,G28882A,G28883C,C28977T,G29506T	22	M:A69S,N:D3L,N:R203K,N:G204R,N:S235F,N:M411I,ORF1a:T1001I,ORF1a:A1708D,ORF1a:I2230T,ORF1b:P314L,ORF3a:G49D,ORF8:Q27*,ORF8:R52I,ORF8:Y73C,S:N501Y,S:A570D,S:D614G,S:P681H,S:T716I,S:S982A,S:D1118H,S:G1219C	6	144
hCoV-19/Mexico/MEX-INMEGEN-05-03-127/2021	EPI_ISL_2319273	-	B.1.1.7	GRY	42	C241T,C913T,C2110T,C3037T,C3267T,A5181C,C5388A,C5986T,T6954C,C9565T,C12789T,C13297T,C14120T,C14408T,C14676T,C15279T,T16176C,G17427A,T17428C,C19390T,G22541A,A23063T,A23128T,C23271A,A23403G,C23604A,C23709T,T24506G,G24914C,G26690T,C27972T,G28048T,A28095T,A28111G,G28280C,A28281T,T28282A,G28827C,G28881A,G28882A,G28883C,C2	26	N:D3L,N:R185P,N:R203K,N:G204R,N:S235F,ORF1a:T1001I,ORF1a:D1639A,ORF1a:A1708D,ORF1a:I2230T,ORF1a:T4175I,ORF1b:P218L,ORF1b:P314L,ORF1b:Y1321H,ORF1b:P1975S,ORF8:Q27*,ORF8:R52I,ORF8:K68*,ORF8:Y73C,S:V327I,S:N501Y,S:A570D,S:D614G,S:P681H,S:T716I,S:S982A,S:D1118H	6	0
hCoV-19/Mexico/MEX-INMEGEN-05-03-15/2021	EPI_ISL_2319183	-	B.1.1.7	GR	35	C241T,C913T,C2110T,C3037T,C3267T,C5388A,C5986T,T6954C,G7042T,A10195G,C14120T,C14408T,C14676T,C15279T,T16176C,C23271A,A23403G,C23604A,A23690G,C23709T,T24506G,G24914C,C27972T,G28048T,G28075T,A28095T,A28111G,G28280C,A28281T,T28282A,G28881A,G28882A,G28883C,C28977T,C29200T	22	N:D3L,N:R203K,N:G204R,N:S235F,ORF1a:T1001I,ORF1a:A1708D,ORF1a:I2230T,ORF1a:M2259I,ORF1b:P218L,ORF1b:P314L,ORF8:Q27*,ORF8:R52I,ORF8:C61F,ORF8:K68*,ORF8:Y73C,S:A570D,S:D614G,S:P681H,S:N710D,S:T716I,S:S982A,S:D1118H	6	574
hCoV-19/Mexico/MEX-INMEGEN-05-03-153/2021	EPI_ISL_2341033	-	B.1.1.7	G	31	C913T,C3037T,C3267T,C5388A,C5986T,G6647A,T6954C,C14408T,C14676T,C15279T,T16176C,C17676T,C18183T,A21519C,C23271A,A23403G,C23604A,C23709T,A24070T,T24506G,G24914C,C25624T,G26849T,C27972T,G28048T,A28095T,A28111G,G28280C,A28281T,T28282A,C28977T	20	M:M109I,N:D3L,ORF1a:T1001I,ORF1a:A1708D,ORF1a:A2128T,ORF1a:I2230T,ORF1b:P314L,ORF1b:R2684S,ORF3a:H78Y,ORF8:Q27*,ORF8:R52I,ORF8:K68*,ORF8:Y73C,S:A570D,S:D614G,S:P681H,S:T716I,S:Q836H,S:S982A,S:D1118H	7	2754
hCoV-19/Mexico/MEX-INMEGEN-05-03-167/2021	EPI_ISL_2319298	-	B.1.1.7	GRY	34	C241T,C913T,C3037T,C3177T,C3267T,C3593A,C5388A,C5986T,C6936T,T6954C,C7420T,C14408T,C14676T,C15279T,A15668T,T16176C,C20844T,A23063T,C23271A,A23403G,C23604A,C23709T,C24374T,T24506G,G24914C,G27463T,A28254C,G28280C,A28281T,T28282A,G28881A,G2888	21	N:D3L,N:R203K,N:G204R,N:S235F,ORF1a:P971L,ORF1a:T1001I,ORF1a:L1110I,ORF1a:A1708D,ORF1a:S2224F,ORF1a:I2230T,ORF1b:P314L,ORF1b:N734I,ORF7a:V24F,S:N501Y,S:A570D,S:D614G,S:P681H,S:T716I,S:L938F,S:S982A,S:D111	6	1485
hCoV-19/Mexico/MEX-INMEGEN-05-03-287/2021	EPI_ISL_2319383	-	B.1.1.7	GR	35	C241T,C913T,G2144A,C2453T,C3037T,C3267T,C5388A,C5986T,T6954C,C9611T,C10252T,C14408T,C14676T,C15279T,T16176C,G18651T,C21855T,G21974C,C23271A,A23403G,C23604A,C23709T,T24506G,G24914C,C27972T,G28048T,A28111G,G28280C,A28281T,T28282A,A28295G,G28881A,G28882A,G28883C,C28977T	24	N:D3L,N:N8D,N:R203K,N:G204R,N:S235F,ORF1a:V627I,ORF1a:L730F,ORF1a:T1001I,ORF1a:A1708D,ORF1a:I2230T,ORF1a:L3116F,ORF1b:P314L,ORF1b:E1728D,ORF8:Q27*,ORF8:R52I,ORF8:Y73C,S:S98F,S:D138H,S:A570D,S:D614G,S:P681H,S:T716I,S:S982A,S:D1118H	6	979

hCoV-19/Mexico/MEX-INMEGEN-05-03-292/2021	EPI_ISL_2319385	-	B.1.1.7	GR	35	C913T,C2110T,C3037T,C3267T,C5388A,C5986T,T6954C,G7042T,A10195G,C10279T,C14120T,C14408T,C14676T,C15279T,T16176C,C16887T,G17427T,C23271A,A23403G,C23604A,C23709T,T24506G,G24914C,C27972T,G28048T,G28075T,A28095T,A28111G,G28280C,A28281T,T28282A,G28881A,G28882A,G28883C,C28977T	21	N:D3L,N:R203K,N:G204R,N:S235F,ORF1a:T1001I,ORF1a:A1708D,ORF1a:I2230T,ORF1a:M2259I,ORF1b:P218L,ORF1b:P314L,ORF8:Q27*,ORF8:R52I,ORF8:C61F,ORF8:K68*,ORF8:Y73C,S:A570D,S:D614G,S:P681H,S:T716I,S:S982A,S:D1118H	6	1588
hCoV-19/Mexico/MEX-INMEGEN-05-03-300/2021	EPI_ISL_2881779	-	B.1.1.7	G	27	C913T,G2144A,C2453T,C3037T,C3267T,C5388A,C5986T,C9611T,C10252T,C14408T,C14676T,C15279T,T16176C,G18651T,C21855T,G21974C,C23271A,A23403G,C23604A,C23709T,T24506G,G24914C,C27972T,G28048T,A28111G,A28295G,C28977T	20	N:N8D,N:S235F,ORF1a:V627I,ORF1a:L730F,ORF1a:T1001I,ORF1a:A1708D,ORF1a:L3116F,ORF1b:P314L,ORF1b:E1728D,ORF8:Q27*,ORF8:R52I,ORF8:Y73C,S:S98F,S:D138H,S:A570D,S:D614G,S:P681H,S:T716I,S:S982A,S:D1118H	6	4959
hCoV-19/Mexico/MEX-INMEGEN-05-03-328/2021	EPI_ISL_2319396	-	B.1.1.7	GR	31	C913T,C3037T,C3267T,C5388A,C5986T,C6196T,T6954C,A8848G,C14408T,C14676T,C15279T,T16176C,T19624C,C23271A,A23403G,C23604A,C23709T,T24506G,G24914C,G25217T,C27972T,G28048T,A28111G,G28280C,A28281T,T28282A,G28881A,G28882A,G28883C,C28977T,G29715T	18	N:D3L,N:R203K,N:G204R,N:S235F,ORF1a:T1001I,ORF1a:A1708D,ORF1a:I2230T,ORF1b:P314L,ORF8:Q27*,ORF8:R52I,ORF8:Y73C,S:A570D,S:D614G,S:P681H,S:T716I,S:S982A,S:D1118H,S:G1219C	6	1247
hCoV-19/Mexico/MEX-INMEGEN-05-03-332/2021	EPI_ISL_2319400	-	B.1.1.7	GRY	38	C241T,C913T,G960A,C2040T,C3037T,C3267T,C5388A,C5986T,G8102A,C14408T,C14676T,T15096C,C15279T,T16176C,C19164T,C20104T,G21893A,A23063T,C23271A,A23403G,C23604A,C23709T,T24506G,G24914C,C25096T,G25437C,G27375A,C27972T,G28048T,A28095T,A28111G,G28280C,A28281T,T28282A,G28881A,G28882A,G28883C,C28977T	25	N:D3L,N:R203K,N:G204R,N:S235F,ORF1a:R232H,ORF1a:T592I,ORF1a:T1001I,ORF1a:A1708D,ORF1a:V2613I,ORF1b:P314L,ORF1b:L2213F,ORF3a:L15F,ORF6:M58I,ORF8:Q27*,ORF8:R52I,ORF8:K68*,ORF8:Y73C,S:D111N,S:N501Y,S:A570D,S:D614G,S:P681H,S:T716I,S:S982A,S:D1118H	6	909
hCoV-19/Mexico/MEX-INMEGEN-05-03-340/2021	EPI_ISL_2319402	-	B.1.1.7	GR	28	C913T,C3037T,C3177T,C3267T,C3593A,C5388A,C5986T,C6936T,T6954C,C7420T,C14408T,C14676T,C15279T,A15668T,T16176C,C20844T,C23271A,A23403G,C23604A,C23709T,C24374T,T24506G,G24914C,G27463T,G28881A,G28882A,G28883C,C28977T	19	N:R203K,N:G204R,N:S235F,ORF1a:P971L,ORF1a:T1001I,ORF1a:L1110I,ORF1a:A1708D,ORF1a:S2224F,ORF1a:I2230T,ORF1b:P314L,ORF1b:N734I,ORF7a:V24F,S:A570D,S:D614G,S:P681H,S:T716I,S:L938F,S:S982A,S:D1118H	6	2204
hCoV-19/Mexico/MEX-INMEGEN-05-03-347/2021	EPI_ISL_2341034	-	B.1.1.7	GR	32	C913T,A1643T,T2785C,C3037T,C3267T,T5260A,C5388A,C5986T,C6128T,G10907A,C14408T,C14676T,C15279T,T16176C,C23271A,A23403G,C23604A,C23709T,T24506G,G24914C,C27972T,G28048T,A28095T,A28111G,G28280C,A28281T,T28282A,G28881A,G28882A,G28883C,C28977T,G	21	N:D3L,N:R203K,N:G204R,N:S235F,ORF1a:N460Y,ORF1a:T1001I,ORF1a:A1708D,ORF1a:L1955F,ORF1a:A3548T,ORF1b:P314L,ORF8:Q27*,ORF8:R52I,ORF8:K68*,ORF8:Y73C,ORF8:F120L,S:A570D,S:D614G,S:P681H,S:T716I,S:S982A,S:D1	6	2165
hCoV-19/Mexico/MEX-INMEGEN-05-03-360/2021	EPI_ISL_2319411	-	B.1.1.7	GR	30	C241T,C913T,C2453T,C3037T,C3267T,C5388A,C5986T,T6954C,C9611T,C14408T,C14676T,C15279T,T16176C,C21855T,G21974C,C23271A,A23403G,C23604A,C23709T,T24506G,G24914C,C27972T,G28048T,A28111G,T28282A,A28295G,G28881A,G28882A,G28883C,C28977T	21	N:N8D,N:R203K,N:G204R,N:S235F,ORF1a:L730F,ORF1a:T1001I,ORF1a:A1708D,ORF1a:I2230T,ORF1a:L3116F,ORF1b:P314L,ORF8:Q27*,ORF8:R52I,ORF8:Y73C,S:S98F,S:D138H,S:A570D,S:D614G,S:P681H,S:T716I,S:S982A,S:D1118H	6	1288
hCoV-19/Mexico/MEX-INMEGEN-05-03-368/2021	EPI_ISL_2319412	-	B.1.1.7	GRY	36	C913T,C3037T,C3267T,C5388A,C5944T,C5986T,T6954C,C9967T,C10868T,C13585T,C14408T,C14676T,T15096C,C15279T,T16176C,A17344G,G18412T,A23063T,C23271A,A23403G,C23604A,C23709T,T24506G,G24914C,C27972T,G28048T,A28095T,A28111G,G28280C,A28281T,T28282A,G28881A,G28882A,G28883C,G28884C,C28977T	21	N:D3L,N:R203K,N:G204P,N:S235F,ORF1a:T1001I,ORF1a:A1708D,ORF1a:I2230T,ORF1b:P314L,ORF1b:I1293V,ORF1b:V1649F,ORF8:Q27*,ORF8:R52I,ORF8:K68*,ORF8:Y73C,S:N501Y,S:A570D,S:D614G,S:P681H,S:T716I,S:S982A,S:D1118H	6	1251
hCoV-19/Mexico/MEX-INMEGEN-05-03-4/2021	EPI_ISL_2319174	-	B.1.1.7	GR	40	C241T,C913T,C2110T,C2918T,C3037T,C3267T,C5388A,C5986T,T6954C,G7042T,T9519C,A10195G,C10279T,C11514T,C14120T,C14408T,C14676T,C15279T,T16176C,C16887T,G17427T,C20313T,C23271A,A23403G,C23604A,C23709T,T24506G,G24914C,C27972T,G28048T,G28075T,A28095T,A28111G,G28280C,A28281T,T28282A,G28881A,G28882A,G28883C,C28977T	24	N:D3L,N:R203K,N:G204R,N:S235F,ORF1a:P885S,ORF1a:T1001I,ORF1a:A1708D,ORF1a:I2230T,ORF1a:M2259I,ORF1a:F3085S,ORF1a:T3750I,ORF1b:P218L,ORF1b:P314L,ORF8:Q27*,ORF8:R52I,ORF8:C61F,ORF8:K68*,ORF8:Y73C,S:A570D,S:D614G,S:P681H,S:T716I,S:S982A,S:D1118H	6	237
hCoV-19/Mexico/MEX-INMEGEN-05-03-43/2021	EPI_ISL_2319202	-	B.1.1.7	GR	36	T201A,C241T,C913T,C2110T,C3037T,C3267T,C5388A,C5986T,T6954C,G7042T,A10195G,C13329T,C14120T,C14408T,C14676T,C15279T,T16176C,C23271A,A23403G,C23604A,C23709T,T24506G,G24914C,T25612G,C27972T,G28048T,G28075T,A28095T,A28111G,G28280C,A28281T,T28282A,G28881A,G28882A,G28883C,C28977T	23	N:D3L,N:R203K,N:G204R,N:S235F,ORF1a:T1001I,ORF1a:A1708D,ORF1a:I2230T,ORF1a:M2259I,ORF1a:T4355I,ORF1b:P218L,ORF1b:P314L,ORF3a:S74A,ORF8:Q27*,ORF8:R52I,ORF8:C61F,ORF8:K68*,ORF8:Y73C,S:A570D,S:D614G,S:P681H,S:T716I,S:S982A,S:D1118H	6	490
hCoV-19/Mexico/MEX-INMEGEN-05-03-46/2021	EPI_ISL_2319205	-	B.1.1.7	GRY	39	C241T,C913T,G1957A,A2276G,G2527A,C3037T,A3094T,C3267T,G4136T,C5388A,C5986T,T6954C,C11704T,C14408T,C14676T,C15279T,T16176C,G19542T,A21866G,G22841A,A23063T,C23271A,A23403G,C23604A,C23709T,T24506G,G24914C,G25954T,C27972T,G28048T,A28111G,G28280C,A28281T,T28282A,C28744T,G28881A,G28882A,G28883C,C28977T	24	N:D3L,N:R203K,N:G204R,N:S235F,ORF1a:I671V,ORF1a:T1001I,ORF1a:V1291F,ORF1a:A1708D,ORF1a:I2230T,ORF1b:P314L,ORF1b:M2025I,ORF3a:G188C,ORF8:Q27*,ORF8:R52I,ORF8:Y73C,S:R102G,S:D427N,S:N501Y,S:A570D,S:D614G,S:P681H,S:T716I,S:S982A,S:D1118H	6	196
hCoV-19/Mexico/MEX-INMEGEN-05-03-5/2021	EPI_ISL_2319175	-	B.1.1.7	GRY	33	C241T,C913T,C3037T,C3267T,C5388A,C5986T,C6196T,T6954C,A8848G,C14408T,C14676T,C15279T,T16176C,T19624C,A23063T,C23271A,A23403G,C23604A,C23709T,T24506G,G24914C,G25217T,C27972T,G28048T,A28111G,G28280C,A28281T,T28282A,G28881A,G28882A,G28883C,C2	19	N:D3L,N:R203K,N:G204R,N:S235F,ORF1a:T1001I,ORF1a:A1708D,ORF1a:I2230T,ORF1b:P314L,ORF8:Q27*,ORF8:R52I,ORF8:Y73C,S:N501Y,S:A570D,S:D614G,S:P681H,S:T716I,S:S982A,S:D1118H,S:G1219C	6	0
hCoV-19/Mexico/MEX-INMEGEN-05-03-69/2021	EPI_ISL_2319226	-	B.1.1.7	GRY	36	C241T,C913T,C3037T,C3267T,C5388A,C5986T,C6196T,T6954C,A8848G,C14408T,C14676T,C15279T,T16176C,A23063T,C23271A,T23332C,A23403G,C23604A,C23709T,T24506G,G24914C,G25217T,G25538A,G26727T,C27972T,G28048T,A28111G,G28280C,A28281T,T28282A,G28881A,G28882A,G28883C,C28977T,G29506T,G29715T	22	M:A69S,N:D3L,N:R203K,N:G204R,N:S235F,N:M411I,ORF1a:T1001I,ORF1a:A1708D,ORF1a:I2230T,ORF1b:P314L,ORF3a:G49D,ORF8:Q27*,ORF8:R52I,ORF8:Y73C,S:N501Y,S:A570D,S:D614G,S:P681H,S:T716I,S:S982A,S:D1118H,S:G1219C	6	0
hCoV-19/Mexico/MEX-INMEGEN-05-03-72/2021	EPI_ISL_2319229	-	B.1.1.7	GRY	38	C222T,C241T,C913T,C2110T,C3037T,C3267T,C4754T,C5388A,C5986T,T6954C,G7042T,C14120T,C14408T,C14676T,C15279T,T16176C,C17762T,C18452T,C19263T,A23063T,C23271A,A23403G,C23604A,C23709T,T24506G,G24914C,T25180C,C27972T,G28048T,A28095T,A28111G,G28280C,A28281T,T28282A,G28881A,G28882A,G28883C,C28977T	24	N:D3L,N:R203K,N:G204R,N:S235F,ORF1a:T1001I,ORF1a:P1497S,ORF1a:A1708D,ORF1a:I2230T,ORF1a:M2259I,ORF1b:P218L,ORF1b:P314L,ORF1b:A1432V,ORF1b:A1662V,ORF8:Q27*,ORF8:R52I,ORF8:K68*,ORF8:Y73C,S:N501Y,S:A570D,S:D614G,S:P681H,S:T716I,S:S982A,S:D1118H	6	11
hCoV-19/Mexico/MEX-INMEGEN-05-03-73/2021	EPI_ISL_2319230	-	B.1.1.7	GRY	34	C241T,C913T,C2788T,C3037T,C3267T,C5388A,C5986T,C6196T,T6954C,A8848G,C14408T,C14676T,C15279T,T16176C,A23063T,C23271A,A23403G,C23604A,C23709T,T24506G,G24914C,G25217T,G26727T,C27972T,G28048T,A28111G,G28280C,A28281T,T28282A,G28881A,G28882A,G28	20	M:A69S,N:D3L,N:R203K,N:G204R,N:S235F,ORF1a:T1001I,ORF1a:A1708D,ORF1a:I2230T,ORF1b:P314L,ORF8:Q27*,ORF8:R52I,ORF8:Y73C,S:N501Y,S:A570D,S:D614G,S:P681H,S:T716I,S:S982A,S:D1118H,S:G1219C	6	0
hCoV-19/Mexico/MEX-INMEGEN-05-03-91/2021	EPI_ISL_2319245	-	B.1.1.7	GRY	37	C241T,C913T,G1957A,A2276G,G2527A,C3037T,A3094T,C3267T,G4136T,C5388A,C5986T,T6954C,C14408T,C14676T,C15279T,T16176C,G19542T,G22841A,A23063T,C23271A,A23403G,C23604A,C23709T,T24506G,G24914C,G25954T,C27972T,G28048T,A28111G,G28280C,A28281T,T28282A,C28744T,G28881A,G28882A,G28883C,C28977T	23	N:D3L,N:R203K,N:G204R,N:S235F,ORF1a:I671V,ORF1a:T1001I,ORF1a:V1291F,ORF1a:A1708D,ORF1a:I2230T,ORF1b:P314L,ORF1b:M2025I,ORF3a:G188C,ORF8:Q27*,ORF8:R52I,ORF8:Y73C,S:D427N,S:N501Y,S:A570D,S:D614G,S:P681H,S:T716I,S:S982A,S:D1118H	6	249
hCoV-19/Mexico/MEX-INMEGEN-05-04-100/2021	EPI_ISL_2349957	-	Q.3	GRY	41	C203T,C241T,C355T,C913T,C3037T,C3267T,C5183T,C5388A,C5986T,T6954C,T8053C,C9924T,C14408T,C14676T,C15279T,C15933T,T16176C,A17615G,C18452T,G20962A,A21137G,A23063T,C23271A,A23403G,C23604A,C23709T,T24506G,G24914C,G25785T,G26730C,C26768T,C27972T,G28048T,A28111G,G28280C,A28281T,T28282A,G28881A,G28882A,G28883C,C28977T	26	M:V70L,N:D3L,N:R203K,N:G204R,N:S235F,ORF1a:T1001I,ORF1a:P1640S,ORF1a:A1708D,ORF1a:I2230T,ORF1a:A3220V,ORF1b:P314L,ORF1b:K1383R,ORF1b:A1662V,ORF1b:D2499N,ORF1b:K2557R,ORF3a:W131C,ORF8:Q27*,ORF8:R52I,ORF8:Y73C,S:N501Y,S:A570D,S:D614G,S:P681H,S:T716I,S:S982A,S:D1118	6	0
hCoV-19/Mexico/MEX-INMEGEN-05-04-103/2021	EPI_ISL_2349960	-	B.1.1.7	GR	32	C913T,C2453T,C3037T,C3267T,C5388A,C5986T,T6954C,C9611T,C14408T,C14676T,C15279T,T16176C,C21855T,G21974C,C23271A,A23403G,C23604A,C23709T,T24506G,G24914C,G27358T,C27972T,G28048T,A28111G,G28280C,A28281T,T28282A,A28295G,G28881A,G28882A,G28883C,C28977T	23	N:D3L,N:N8D,N:R203K,N:G204R,N:S235F,ORF1a:L730F,ORF1a:T1001I,ORF1a:A1708D,ORF1a:I2230T,ORF1a:L3116F,ORF1b:P314L,ORF6:D53Y,ORF8:Q27*,ORF8:R52I,ORF8:Y73C,S:S98F,S:D138H,S:A570D,S:D614G,S:P681H,S:T716I,S:S982A,S:D1118H	6	1878
hCoV-19/Mexico/MEX-INMEGEN-05-04-105/2021	EPI_ISL_2349961	-	B.1.1.7	GR	35	C913T,C2110T,C3037T,C3267T,C5388A,C5986T,T6954C,G7042T,C14120T,C14408T,C14676T,C15279T,T16176C,C17762T,C23271A,T23296C,A23403G,C23604A,C23709T,T24295C,T24506G,G24914C,A25564G,C27686T,C27972T,G28048T,A28095T,A28111G,G28280C,A28281T,T28282A,G28881A,G28882A,G28883C,C28977T	23	N:D3L,N:R203K,N:G204R,N:S235F,ORF1a:T1001I,ORF1a:A1708D,ORF1a:I2230T,ORF1a:M2259I,ORF1b:P218L,ORF1b:P314L,ORF1b:A1432V,ORF3a:S58G,ORF7a:S98F,ORF8:Q27*,ORF8:R52I,ORF8:K68*,ORF8:Y73C,S:A570D,S:D614G,S:P681H,S:T716I,S:S982A,S:D1118H	6	916
hCoV-19/Mexico/MEX-INMEGEN-05-04-110/2021	EPI_ISL_2349965	-	B.1.1.7	GRY	35	C913T,C3037T,C3267T,G4510A,C5388A,C5986T,C6196T,T6954C,A8848G,C9803T,T11135C,C14408T,C14676T,C15279T,T16176C,A23063T,C23114T,C23271A,A23403G,C23604A,C23709T,G24095T,T24506G,G24914C,C27972T,G28048T,A28111G,G28280C,A28281T,T28282A,G28881A,G28882A,G28883C,C28977T,G29715T	20	N:D3L,N:R203K,N:G204R,N:S235F,ORF1a:T1001I,ORF1a:A1708D,ORF1a:I2230T,ORF1a:F3624L,ORF1b:P314L,ORF8:Q27*,ORF8:R52I,ORF8:Y73C,S:N501Y,S:A570D,S:D614G,S:P681H,S:T716I,S:A845S,S:S982A,S:D1118H	6	219
hCoV-19/Mexico/MEX-INMEGEN-05-04-112/2021	EPI_ISL_2349966	-	B.1.1.7	GR	35	C913T,G1820A,C3037T,C3267T,C5388A,G5924A,C5986T,T6954C,G9526T,A12162G,C14408T,C14676T,C15279T,C15720T,T16176C,A17615G,A21137G,C23248T,C23271A,A23403G,C23604A,C23709T,T24506G,G24914C,G25135T,C27972T,G28048T,A28111G,G28280C,A28281T,T28282A,G28881A,G28882A,G28883C,C28977T	24	N:D3L,N:R203K,N:G204R,N:S235F,ORF1a:G519S,ORF1a:T1001I,ORF1a:A1708D,ORF1a:V1887I,ORF1a:I2230T,ORF1a:M3087I,ORF1a:Q3966R,ORF1b:P314L,ORF1b:K1383R,ORF1b:K2557R,ORF8:Q27*,ORF8:R52I,ORF8:Y73C,S:A570D,S:D614G,S:P681H,S:T716I,S:S982A,S:D1118H,S:K1191N	6	1484

hCoV-19/Mexico/MEX-INMEGEN-05-04-113/2021	EPI_ISL_2349967	-	B.1.1.7	GRY	38	C241T,C913T,C3037T,C3267T,C5388A,C5944T,C5986T,T6954C,C9967T,C10868T,C13585T,C14408T,C14676T,T15096C,C15279T,T16176C,A17344G,G18412T,C22987T,A23063T,C23271A,A23403G,C23604A,C23709T,T24506G,G24914C,C27972T,G28048T,A28095T,A28111G,G28280C,A28281T,T28282A,G28881A,G28882A,G28883C,G28884C,C28977T	21	N:D3L,N:R203K,N:G204P,N:S235F,ORF1a:T1001I,ORF1a:A1708D,ORF1a:I2230T,ORF1b:P314L,ORF1b:I1293V,ORF1b:V1649F,ORF8:Q27*,ORF8:R52I,ORF8:K68*,ORF8:Y73C,S:N501Y,S:A570D,S:D614G,S:P681H,S:T716I,S:S982A,S:D1118H	6	0
hCoV-19/Mexico/MEX-INMEGEN-05-04-133/2021	EPI_ISL_2349979	-	B.1.1.7	GRY	34	C241T,C913T,C3037T,C3267T,C5388A,C5986T,C6196T,T6954C,A8848G,T9622C,C14408T,C14676T,C15279T,T16176C,A23063T,C23271A,A23403G,C23604A,C23709T,T24506G,G24914C,G25217T,G26727T,C27972T,G28048T,A28111G,G28280C,A28281T,T28282A,G28881A,G28882A,G28	20	M:A69S,N:D3L,N:R203K,N:G204R,N:S235F,ORF1a:T1001I,ORF1a:A1708D,ORF1a:I2230T,ORF1b:P314L,ORF8:Q27*,ORF8:R52I,ORF8:Y73C,S:N501Y,S:A570D,S:D614G,S:P681H,S:T716I,S:S982A,S:D1118H,S:G1219C	6	724
hCoV-19/Mexico/MEX-INMEGEN-05-04-156/2021	EPI_ISL_2443076	-	B.1.1.7	GRY	36	C241T,C913T,A1643T,T2785C,C3037T,C3267T,C5388A,A5538T,C5986T,C6128T,T6954C,T7763G,G10907A,C14408T,C14676T,C15279T,T16176C,A23063T,C23271A,A23403G,C23604A,C23709T,T24506G,G24914C,C27972T,G28048T,A28095T,A28111G,G28280C,A28281T,T28282A,G28881A,G28882A,G28883C,C28977T,G29179T	25	N:D3L,N:R203K,N:G204R,N:S235F,ORF1a:N460Y,ORF1a:T1001I,ORF1a:A1708D,ORF1a:Q1758L,ORF1a:L1955F,ORF1a:I2230T,ORF1a:S2500A,ORF1a:A3548T,ORF1b:P314L,ORF8:Q27*,ORF8:R52I,ORF8:K68*,ORF8:Y73C,ORF8:F120L,S:N501Y,S:A570D,S:D614G,S:P681H,S:T716I,S:S982A,S:D1118H	6	421
hCoV-19/Mexico/MEX-INMEGEN-05-04-165/2021	EPI_ISL_2350000	-	B.1.1.7	GRY	34	C241T,C913T,G1598A,C2453T,C3037T,C3267T,C5388A,C5986T,T6954C,C9611T,G11335T,C14408T,C14676T,C15279T,T16176C,C21008T,C21855T,G21974C,A23063T,C23271A,A23403G,C23604A,C23709T,T24506G,G24914C,C27643T,A28111G,T28149A,T28282A,A28295G,G28881A,G28882A,G28883C,C28977T	24	N:N8D,N:R203K,N:G204R,N:S235F,ORF1a:G445S,ORF1a:L730F,ORF1a:T1001I,ORF1a:A1708D,ORF1a:I2230T,ORF1a:L3116F,ORF1b:P314L,ORF1b:T2514I,ORF7a:P84S,ORF8:Y73C,ORF8:F86I,S:S98F,S:D138H,S:N501Y,S:A570D,S:D614G,S:P681H,S:T716I,S:S982A,S:D1118H	6	613
hCoV-19/Mexico/MEX-INMEGEN-05-04-189/2021	EPI_ISL_2350017	-	B.1.1.7	G	25	C913T,C3037T,C3267T,C5986T,T6954C,G7042T,C14120T,C14408T,C14676T,C15279T,T16176C,C17762T,C23271A,A23403G,C23604A,C23709T,T24506G,G24914C,T25180C,C27972T,G28048T,A28111G,G28280C,A28281T,T28282A	16	N:D3L,ORF1a:T1001I,ORF1a:I2230T,ORF1a:M2259I,ORF1b:P218L,ORF1b:P314L,ORF1b:A1432V,ORF8:Q27*,ORF8:R52I,ORF8:Y73C,S:A570D,S:D614G,S:P681H,S:T716I,S:S982A,S:D1118H	6	6692
hCoV-19/Mexico/MEX-INMEGEN-05-04-197/2021	EPI_ISL_2350022	-	B.1.1.7	GR	37	C241T,C913T,C2110T,C3037T,C3267T,C5388A,C5986T,T6954C,G7042T,C14120T,C14408T,C14676T,C15279T,T16176C,C17762T,C23271A,T23296C,A23403G,C23604A,C23709T,T24295C,T24506G,G24914C,A25564G,C26208T,C27686T,C27972T,G28048T,A28095T,A28111G,G28280C,A28281T,T28282A,G28881A,G28882A,G28883C,C28977T	23	N:D3L,N:R203K,N:G204R,N:S235F,ORF1a:T1001I,ORF1a:A1708D,ORF1a:I2230T,ORF1a:M2259I,ORF1b:P218L,ORF1b:P314L,ORF1b:A1432V,ORF3a:S58G,ORF7a:S98F,ORF8:Q27*,ORF8:R52I,ORF8:K68*,ORF8:Y73C,S:A570D,S:D614G,S:P681H,S:T716I,S:S982A,S:D1118H	6	211
hCoV-19/Mexico/MEX-INMEGEN-05-04-200/2021	EPI_ISL_2350025	-	B.1.1.7	GRY	32	C241T,C913T,C3037T,C3177T,C3267T,C3593A,C5388A,C5986T,C6936T,T6954C,C7420T,C14408T,C14676T,C15279T,A15668T,T16176C,C20844T,A23063T,C23271A,A23403G,C23604A,C23709T,C24374T,T24506G,G24914C,G27463T,A28254C,T28282A,G28881A,G28882A,G28883C,C2897	20	N:R203K,N:G204R,N:S235F,ORF1a:P971L,ORF1a:T1001I,ORF1a:L1110I,ORF1a:A1708D,ORF1a:S2224F,ORF1a:I2230T,ORF1b:P314L,ORF1b:N734I,ORF7a:V24F,S:N501Y,S:A570D,S:D614G,S:P681H,S:T716I,S:L938F,S:S982A,S:D1118H	7	780
hCoV-19/Mexico/MEX-INMEGEN-05-04-204/2021	EPI_ISL_2350027	-	B.1.1.7	GRY	36	C222T,C241T,C913T,C2110T,C3037T,C3267T,C5388A,C5986T,T6954C,G7042T,C14120T,C14408T,C14676T,C15279T,T16176C,C17762T,A23063T,C23271A,A23403G,C23604A,C23709T,T24506G,G24914C,T25180C,C27972T,G28048T,A28095T,A28111G,G28280C,A28281T,T28282A,C28873T,G28881A,G28882A,G28883C,C28977T	22	N:D3L,N:R203K,N:G204R,N:S235F,ORF1a:T1001I,ORF1a:A1708D,ORF1a:I2230T,ORF1a:M2259I,ORF1b:P218L,ORF1b:P314L,ORF1b:A1432V,ORF8:Q27*,ORF8:R52I,ORF8:K68*,ORF8:Y73C,S:N501Y,S:A570D,S:D614G,S:P681H,S:T716I,S:S982A,S:D1118H	6	154
hCoV-19/Mexico/MEX-INMEGEN-05-04-21/2021	EPI_ISL_2349899	-	B.1.1.7	GRY	38	G174T,G210T,C241T,C913T,C1420T,C2453T,C3037T,C3267T,C5388A,C5986T,T6954C,T9016C,C9661T,C10125T,C14408T,T14565C,C14676T,C15279T,T16176C,T17803G,A23063T,C23271A,A23403G,C23604A,C23709T,T24506G,G24914C,C27883T,C27972T,G28048T,A28111G,G28280C,A28281T,T28282A,G28881A,G28882A,G28883C,C28977T	22	N:D3L,N:R203K,N:G204R,N:S235F,ORF1a:L730F,ORF1a:T1001I,ORF1a:A1708D,ORF1a:I2230T,ORF1a:T3287I,ORF1b:P314L,ORF1b:S1446A,ORF7b:A43V,ORF8:Q27*,ORF8:R52I,ORF8:Y73C,S:N501Y,S:A570D,S:D614G,S:P681H,S:T716I,S:S982A,S:D1118H	6	892
hCoV-19/Mexico/MEX-INMEGEN-05-04-214/2021	EPI_ISL_2350034	-	B.1.1.7	G	23	C913T,G1598A,C2453T,C3037T,C3267T,C5388A,C9611T,C14408T,C14676T,C15279T,T16176C,C21008T,C21855T,G21974C,C23271A,A23403G,C23604A,C23709T,T24506G,G24914C,A28111G,T28149A,A28295G	18	N:N8D,ORF1a:G445S,ORF1a:L730F,ORF1a:T1001I,ORF1a:A1708D,ORF1a:L3116F,ORF1b:P314L,ORF1b:T2514I,ORF8:Y73C,ORF8:F86I,S:S98F,S:D138H,S:A570D,S:D614G,S:P681H,S:T716I,S:S982A,S:D1118H	6	7759
hCoV-19/Mexico/MEX-INMEGEN-05-04-229/2021	EPI_ISL_2350045	-	B.1.1.7	G	29	C913T,C2788T,C3037T,C3267T,C5388A,C5986T,C6196T,T6954C,C14408T,C14676T,C15279T,T16176C,C23271A,A23403G,C23604A,C23709T,T24506G,G24914C,G25217T,G26727T,C27972T,G28048T,A28111G,G28280C,A28281T,T28282A,G28881A,C28977T,G29715T	17	M:A69S,N:D3L,N:S235F,ORF1a:T1001I,ORF1a:A1708D,ORF1a:I2230T,ORF1b:P314L,ORF8:Q27*,ORF8:R52I,ORF8:Y73C,S:A570D,S:D614G,S:P681H,S:T716I,S:S982A,S:D1118H,S:G1219C	6	2919
hCoV-19/Mexico/MEX-INMEGEN-05-04-235/2021	EPI_ISL_2350049	-	B.1.1.7	GR	33	C854T,C913T,C3037T,C3177T,C3267T,C3593A,C5388A,C5986T,C6936T,T6954C,C7420T,C7728T,C14408T,C14676T,C15279T,A15668T,T16176C,C20844T,C23271A,A23403G,C23604A,C23709T,C24374T,T24506G,G24914C,G27463T,G28280C,A28281T,T28282A,G28881A,G28882A,G28883C,C28977T	22	N:D3L,N:R203K,N:G204R,N:S235F,ORF1a:P197S,ORF1a:P971L,ORF1a:T1001I,ORF1a:L1110I,ORF1a:A1708D,ORF1a:S2224F,ORF1a:I2230T,ORF1a:S2488F,ORF1b:P314L,ORF1b:N734I,ORF7a:V24F,S:A570D,S:D614G,S:P681H,S:T716I,S:L938F,S:S982A,S:D1118H	6	2091
hCoV-19/Mexico/MEX-INMEGEN-05-04-245/2021	EPI_ISL_2350057	-	B.1.1.7	GRY	34	C241T,C913T,C2788T,C3037T,C3267T,C5388A,C5986T,C6196T,T6954C,A8848G,C14408T,C14676T,C15279T,T16176C,A23063T,C23271A,A23403G,C23604A,C23709T,T24506G,G24914C,G25217T,G26727T,C27972T,G28048T,A28111G,G28280C,A28281T,T28282A,G28881A,G28882A,G28	20	M:A69S,N:D3L,N:R203K,N:G204R,N:S235F,ORF1a:T1001I,ORF1a:A1708D,ORF1a:I2230T,ORF1b:P314L,ORF8:Q27*,ORF8:R52I,ORF8:Y73C,S:N501Y,S:A570D,S:D614G,S:P681H,S:T716I,S:S982A,S:D1118H,S:G1219C	6	20
hCoV-19/Mexico/MEX-INMEGEN-05-04-248/2021	EPI_ISL_2350059	-	B.1.1.7	GR	30	C913T,C3037T,C3267T,C5388A,C5884T,C14408T,C14676T,C15279T,T16176C,C19017T,C23271A,A23403G,C23604A,C23709T,T24506G,G24914C,G25217T,G26727T,C27972T,G28048T,A28111G,G28280C,A28281T,T28282A,G28881A,G28882A,G28883C,C28977T,G29260T,G29715T	18	M:A69S,N:D3L,N:R203K,N:G204R,N:S235F,ORF1a:T1001I,ORF1a:A1708D,ORF1b:P314L,ORF8:Q27*,ORF8:R52I,ORF8:Y73C,S:A570D,S:D614G,S:P681H,S:T716I,S:S982A,S:D1118H,S:G1219C	6	5050
hCoV-19/Mexico/MEX-INMEGEN-05-04-253/2021	EPI_ISL_2350062	-	B.1.1.7	GRY	34	C241T,C913T,C2788T,C3037T,C3267T,C5388A,C5986T,C6196T,T6954C,A8848G,C14408T,C14676T,C15279T,T16176C,A23063T,C23271A,A23403G,C23604A,C23709T,T24506G,G24914C,G25217T,G26727T,C27972T,G28048T,A28111G,G28280C,A28281T,T28282A,G28881A,G28882A,G28	20	M:A69S,N:D3L,N:R203K,N:G204R,N:S235F,ORF1a:T1001I,ORF1a:A1708D,ORF1a:I2230T,ORF1b:P314L,ORF8:Q27*,ORF8:R52I,ORF8:Y73C,S:N501Y,S:A570D,S:D614G,S:P681H,S:T716I,S:S982A,S:D1118H,S:G1219C	6	67
hCoV-19/Mexico/MEX-INMEGEN-05-04-258/2021	EPI_ISL_2350065	-	B.1.1.7	GR	35	C241T,C854T,C913T,C3037T,C3177T,C3267T,C3593A,C5388A,C5986T,C6936T,T6954C,C7420T,C7728T,C14408T,C14676T,C15279T,A15668T,T16176C,C20844T,C23271A,A23403G,C23604A,C23709T,C24374T,T24506G,G24914C,G27463T,A28254C,G28280C,A28281T,T28282A,G28881A,G28882A,G28883C,C28977T	22	N:D3L,N:R203K,N:G204R,N:S235F,ORF1a:P197S,ORF1a:P971L,ORF1a:T1001I,ORF1a:L1110I,ORF1a:A1708D,ORF1a:S2224F,ORF1a:I2230T,ORF1a:S2488F,ORF1b:P314L,ORF1b:N734I,ORF7a:V24F,S:A570D,S:D614G,S:P681H,S:T716I,S:L938F,S:S982A,S:D1118H	6	1057
hCoV-19/Mexico/MEX-INMEGEN-05-04-262/2021	EPI_ISL_2350068	-	B.1.1.7	GR	34	C241T,C913T,C3037T,C3267T,C5388A,C5986T,C6196T,T6954C,A8848G,C14408T,C14676T,C15279T,T16176C,G19816T,C23271A,A23403G,C23604A,C23709T,T24506G,G24914C,G25217T,G26727T,T27285C,C27972T,G28048T,A28111G,G28280C,A28281T,T28282A,G28881A,G28882A,G2	20	M:A69S,N:D3L,N:R203K,N:G204R,N:S235F,ORF1a:T1001I,ORF1a:A1708D,ORF1a:I2230T,ORF1b:P314L,ORF1b:V2117L,ORF8:Q27*,ORF8:R52I,ORF8:Y73C,S:A570D,S:D614G,S:P681H,S:T716I,S:S982A,S:D1118H,S:G1219C	6	535
hCoV-19/Mexico/MEX-INMEGEN-05-04-283/2021	EPI_ISL_2350081	-	B.1.1.7	GR	34	C241T,C913T,C3037T,C3267T,C5388A,C5986T,C6196T,T6954C,C14408T,C14676T,C15279T,T16176C,C18568T,G19656T,C23271A,A23403G,C23604A,C23709T,T24506G,G24914C,G25217T,G26428T,G26727T,C27972T,G28048T,A28111G,G28280C,A28281T,T28282A,G28881A,G28882A,G28883C,C28977T,G29715T	22	E:V62F,M:A69S,N:D3L,N:R203K,N:G204R,N:S235F,ORF1a:T1001I,ORF1a:A1708D,ORF1a:I2230T,ORF1b:P314L,ORF1b:L1701F,ORF1b:K2063N,ORF8:Q27*,ORF8:R52I,ORF8:Y73C,S:A570D,S:D614G,S:P681H,S:T716I,S:S982A,S:D1118H,S:G1219C	6	1423
hCoV-19/Mexico/MEX-INMEGEN-05-04-285/2021	EPI_ISL_2350082	-	B.1.1.7	GRY	39	C241T,C913T,G1957A,C2189T,A2276G,G2527A,C3037T,A3094T,C3267T,G4136T,C5388A,C5986T,T6954C,C11704T,C14408T,C14676T,C15279T,T16176C,G19542T,G22841A,A23063T,C23271A,A23403G,C23604A,C23709T,T24506G,G24914C,G25954T,C27972T,G28048T,A28111G,G28280C,A28281T,T28282A,C28744T,G28881A,G28882A,G28883C,C28977T	24	N:D3L,N:R203K,N:G204R,N:S235F,ORF1a:L642F,ORF1a:I671V,ORF1a:T1001I,ORF1a:V1291F,ORF1a:A1708D,ORF1a:I2230T,ORF1b:P314L,ORF1b:M2025I,ORF3a:G188C,ORF8:Q27*,ORF8:R52I,ORF8:Y73C,S:D427N,S:N501Y,S:A570D,S:D614G,S:P681H,S:T716I,S:S982A,S:D1118H	6	17
hCoV-19/Mexico/MEX-INMEGEN-05-04-292/2021	EPI_ISL_2350087	-	Q.3	GR	36	C241T,C913T,C3037T,C3267T,G5023A,C5388A,C5986T,C6568T,T6954C,G8183A,A9529G,G10396T,C14408T,C14676T,C15279T,T16176C,A17615G,G20962A,G22094C,C23271A,A23403G,C23604A,C23709T,T24506G,G24914C,G26730C,C27972T,G28048T,A28111G,G28280C,A28281T,T28282A,G28881A,G28882A,G28883C,C28977T	23	M:V70L,N:D3L,N:R203K,N:G204R,N:S235F,ORF1a:T1001I,ORF1a:M1586I,ORF1a:A1708D,ORF1a:I2230T,ORF1a:G2640R,ORF1b:P314L,ORF1b:K1383R,ORF1b:D2499N,ORF8:Q27*,ORF8:R52I,ORF8:Y73C,S:D178H,S:A570D,S:D614G,S:P681H,S:T716I,S:S982A,S:D1118H	6	378
hCoV-19/Mexico/MEX-INMEGEN-05-04-296/2021	EPI_ISL_2350090	-	B.1.1.7	GR	31	C913T,C3037T,C3267T,C5388A,C5986T,C6196T,T6954C,C14408T,C14676T,C15279T,T16176C,T19624C,C23271A,A23403G,C23604A,C23709T,T24506G,G24914C,G25217T,C25916T,C27972T,G28048T,A28111G,G28280C,A28281T,T28282A,G28881A,G28882A,G28883C,C28977T,G29715T	19	N:D3L,N:R203K,N:G204R,N:S235F,ORF1a:T1001I,ORF1a:A1708D,ORF1a:I2230T,ORF1b:P314L,ORF3a:T175I,ORF8:Q27*,ORF8:R52I,ORF8:Y73C,S:A570D,S:D614G,S:P681H,S:T716I,S:S982A,S:D1118H,S:G1219C	6	2856

hCoV-19/Mexico/MEX-INMEGEN-05-04-337/2021	EPI_ISL_2350115	-	B.1.1.7	GRY	43	T201A,C241T,C913T,C2110T,C2675T,A2693T,C3037T,C3267T,C5388A,C5986T,T6954C,G7042T,A10195G,C13329T,C14120T,C14408T,C14676T,C15279T,T16176C,C21811T,T22679C,A23063T,C23271A,A23403G,C23604A,C23709T,T24506G,G24914C,C25046G,C25708T,C26270T,C27972T,G28048T,G28075T,A28095T,A28111G,G28280C,A28281T,T28282A,G28881A,G28882A,G28883	29	E:T9I,N:D3L,N:R203K,N:G204R,N:S235F,ORF1a:P804S,ORF1a:N810Y,ORF1a:T1001I,ORF1a:A1708D,ORF1a:I2230T,ORF1a:M2259I,ORF1a:T4355I,ORF1b:P218L,ORF1b:P314L,ORF3a:L106F,ORF8:Q27*,ORF8:R52I,ORF8:C61F,ORF8:K68*,ORF8:Y73C,S:S373P,S:N501Y,S:A570D,S:D614G,S:P681H,S:T716I,S:S982A,S:D1	6	0
hCoV-19/Mexico/MEX-INMEGEN-05-04-341/2021	EPI_ISL_2350118	-	B.1.1.7	GR	39	C913T,G2281A,C3037T,C3267T,C5388A,C5986T,T6954C,A12162G,C13665T,C14408T,C14676T,C15279T,C15720T,T16176C,A17615G,C18747T,C20844T,A21137G,C23271A,A23403G,C23604A,C23709T,T24506G,G24914C,G25135T,C26110T,C26521T,G26828T,C27972T,G28048T,A28111G,G28280C,A28281T,T28282A,G28881A,G28882A,G28883C,C28977T,C29095T	22	N:D3L,N:R203K,N:G204R,N:S235F,ORF1a:T1001I,ORF1a:A1708D,ORF1a:I2230T,ORF1a:Q3966R,ORF1b:P314L,ORF1b:K1383R,ORF1b:K2557R,ORF3a:P240S,ORF8:Q27*,ORF8:R52I,ORF8:Y73C,S:A570D,S:D614G,S:P681H,S:T716I,S:S982A,S:D1118H,S:K1191N	6	1930
hCoV-19/Mexico/MEX-INMEGEN-05-04-355/2021	EPI_ISL_2350123	-	B.1.1.7	GRY	38	C222T,C241T,C913T,C2110T,C3037T,C3267T,C5388A,C5986T,T6954C,G7042T,C14120T,C14408T,C14676T,C15279T,T16176C,C17762T,C23029T,A23063T,C23271A,A23403G,C23604A,C23709T,G24197T,T24506G,G24914C,T25180C,C27972T,G28048T,A28095T,A28111G,G28280C,A28281T,T28282A,G28881A,G28882A,G28883C,C28977T,C29420T	24	N:D3L,N:R203K,N:G204R,N:S235F,N:P383S,ORF1a:T1001I,ORF1a:A1708D,ORF1a:I2230T,ORF1a:M2259I,ORF1b:P218L,ORF1b:P314L,ORF1b:A1432V,ORF8:Q27*,ORF8:R52I,ORF8:K68*,ORF8:Y73C,S:N501Y,S:A570D,S:D614G,S:P681H,S:T716I,S:A879S,S:S982A,S:D1118H	6	8
hCoV-19/Mexico/MEX-INMEGEN-05-04-357/2021	EPI_ISL_2350124	-	B.1.1.7	GRY	36	C241T,C913T,C3037T,C3267T,C5388A,C5986T,C6196T,T6954C,C7765T,A8848G,C14408T,C14676T,C15279T,T16176C,C22425T,C22792T,A23063T,C23271A,A23403G,C23604A,C23709T,T24506G,G24914C,G25217T,G26727T,C27972T,G28048T,A28111G,G28280C,A28281T,T28282A,G28881A,G28882A,G28883C,C28977T,G29715T	21	M:A69S,N:D3L,N:R203K,N:G204R,N:S235F,ORF1a:T1001I,ORF1a:A1708D,ORF1a:I2230T,ORF1b:P314L,ORF8:Q27*,ORF8:R52I,ORF8:Y73C,S:A288V,S:N501Y,S:A570D,S:D614G,S:P681H,S:T716I,S:S982A,S:D1118H,S:G1219C	6	399
hCoV-19/Mexico/MEX-INMEGEN-05-04-36/2021	EPI_ISL_2349912	-	B.1.1.7	GRY	34	C241T,C913T,C2453T,C3037T,C3267T,C5388A,C5986T,T6954C,C9166T,C9611T,C14408T,C14676T,C15279T,T16176C,C21855T,G21974C,A23063T,C23271A,A23403G,C23604A,C23709T,T24506G,G24914C,C27643T,C27972T,G28048T,A28111G,T28282A,A28295G,C28728T,G28881A,G28882A,G28883C,C28977T	24	N:N8D,N:A152V,N:R203K,N:G204R,N:S235F,ORF1a:L730F,ORF1a:T1001I,ORF1a:A1708D,ORF1a:I2230T,ORF1a:L3116F,ORF1b:P314L,ORF7a:P84S,ORF8:Q27*,ORF8:R52I,ORF8:Y73C,S:S98F,S:D138H,S:N501Y,S:A570D,S:D614G,S:P681H,S:T716I,S:S982A,S:D1118H	6	3
hCoV-19/Mexico/MEX-INMEGEN-05-04-373/2021	EPI_ISL_2350138	-	B.1.1.7	GR	37	C913T,G960A,C2040T,C3037T,C3267T,C5388A,C5986T,T6954C,G8102A,C14408T,C14676T,T15096C,C15279T,T16176C,C19164T,C20104T,G21893A,C23271A,A23403G,C23604A,C23709T,T24506G,G24914C,C25096T,G25437C,G27375A,C27972T,G28048T,A28095T,A28111G,G28280C,A28281T,T28282A,G28881A,G28882A,G28883C,C28977T	25	N:D3L,N:R203K,N:G204R,N:S235F,ORF1a:R232H,ORF1a:T592I,ORF1a:T1001I,ORF1a:A1708D,ORF1a:I2230T,ORF1a:V2613I,ORF1b:P314L,ORF1b:L2213F,ORF3a:L15F,ORF6:M58I,ORF8:Q27*,ORF8:R52I,ORF8:K68*,ORF8:Y73C,S:D111N,S:A570D,S:D614G,S:P681H,S:T716I,S:S982A,S:D1118H	6	2179
hCoV-19/Mexico/MEX-INMEGEN-05-04-53/2021	EPI_ISL_2349922	-	B.1.1.7	G	41	T201A,C241T,C913T,C2110T,C2675T,A2693T,C3037T,C3267T,C5388A,C5986T,T6954C,G7042T,A10195G,C13329T,C14120T,C14408T,C14676T,C15279T,T16176C,C21811T,A23063T,C23271A,A23403G,C23604A,C23709T,T24506G,G24914C,C25046G,C25708T,C26270T,C27972T,G28048T,G28075T,A28095T,A28111G,A28254C,G28280C,A28281T,T28282A,C28977T,C29708T	27	E:T9I,N:D3L,N:S235F,ORF1a:P804S,ORF1a:N810Y,ORF1a:T1001I,ORF1a:A1708D,ORF1a:I2230T,ORF1a:M2259I,ORF1a:T4355I,ORF1b:P218L,ORF1b:P314L,ORF3a:L106F,ORF8:Q27*,ORF8:R52I,ORF8:C61F,ORF8:K68*,ORF8:Y73C,ORF8:I121L,S:N501Y,S:A570D,S:D614G,S:P681H,S:T716I,S:S982A,S:D1118H,S:P1162A	6	748
hCoV-19/Mexico/MEX-INMEGEN-05-04-61/2021	EPI_ISL_2349927	-	B.1.1.7	G	27	C241T,C913T,C3037T,C3267T,T3537C,C5388A,C5986T,T6954C,C14408T,C14676T,C15279T,T16176C,G20683T,C23271A,A23403G,C23604A,C23709T,C24374T,T24506G,G24914C,C27972T,G28048T,A28111G,G28280C,A28281T,T28282A,C29367T	18	N:D3L,N:P365L,ORF1a:T1001I,ORF1a:I1091T,ORF1a:A1708D,ORF1a:I2230T,ORF1b:P314L,ORF1b:V2406F,ORF8:Q27*,ORF8:R52I,ORF8:Y73C,S:A570D,S:D614G,S:P681H,S:T716I,S:L938F,S:S982A,S:D1118H	6	3932
hCoV-19/Mexico/MEX-INMEGEN-05-04-69/2021	EPI_ISL_2349934	-	B.1.1.7	GRY	40	C241T,C913T,C2334T,C3037T,C3267T,C5388A,C5986T,T6954C,A12162G,C13665T,C14408T,C14676T,C15279T,C15720T,T16176C,A17615G,C18747T,C20404T,A21137G,A23063T,C23271A,A23403G,C23604A,C23709T,T24506G,G24914C,G25135T,G25218T,C26110T,C27972T,G28048T,A28111G,G28280C,A28281T,T28282A,G28881A,G28882A,G28883C,C28977T,C29095T	26	N:D3L,N:R203K,N:G204R,N:S235F,ORF1a:A690V,ORF1a:T1001I,ORF1a:A1708D,ORF1a:I2230T,ORF1a:Q3966R,ORF1b:P314L,ORF1b:K1383R,ORF1b:P2313S,ORF1b:K2557R,ORF3a:P240S,ORF8:Q27*,ORF8:R52I,ORF8:Y73C,S:N501Y,S:A570D,S:D614G,S:P681H,S:T716I,S:S982A,S:D1118H,S:K1191N,S:G1219V	6	91
hCoV-19/Mexico/MEX-INMEGEN-05-04-82/2021	EPI_ISL_2349945	-	B.1.1.7	GRY	36	C241T,C913T,C2110T,C2338T,C3037T,C3267T,T5278C,C5388A,C5986T,T6954C,G7042T,A8879G,C14120T,C14408T,C14676T,C15279T,T16176C,G21578T,A23063T,C23271A,A23403G,C23604A,C23709T,G24415T,G24914C,C27972T,G28048T,A28095T,A28111G,G28280C,A28281T,T28282A,G28881A,G28882A,G28883C,C28977T	22	N:D3L,N:R203K,N:G204R,N:S235F,ORF1a:T1001I,ORF1a:A1708D,ORF1a:I2230T,ORF1a:M2259I,ORF1a:T2872A,ORF1b:P218L,ORF1b:P314L,ORF8:Q27*,ORF8:R52I,ORF8:K68*,ORF8:Y73C,S:V6F,S:N501Y,S:A570D,S:D614G,S:P681H,S:T716I,S:D1118H	6	165
hCoV-19/Mexico/MEX-INMEGEN-05-04-85/2021	EPI_ISL_2349947	-	B.1.1.7	GRY	36	C241T,C913T,C2453T,C3037T,C3267T,C5388A,C5986T,T6954C,C9166T,C9611T,C14408T,C14676T,C15279T,T16176C,C21855T,G21974C,A23063T,C23271A,A23403G,C23604A,C23709T,T24506G,G24914C,C27643T,C27972T,G28048T,A28111G,G28280C,A28281T,T28282A,A28295G,C28728T,G28881A,G28882A,G28883C,C28977T	25	N:D3L,N:N8D,N:A152V,N:R203K,N:G204R,N:S235F,ORF1a:L730F,ORF1a:T1001I,ORF1a:A1708D,ORF1a:I2230T,ORF1a:L3116F,ORF1b:P314L,ORF7a:P84S,ORF8:Q27*,ORF8:R52I,ORF8:Y73C,S:S98F,S:D138H,S:N501Y,S:A570D,S:D614G,S:P681H,S:T716I,S:S982A,S:D1118H	6	122
hCoV-19/Mexico/MEX-INMEGEN-05-04-94/2021	EPI_ISL_2349952	-	B.1.1.7	GRY	40	C241T,C913T,C3037T,C3267T,C5388A,C5944T,C5986T,G6647A,T6954C,C14408T,C14676T,T15096C,C15279T,T16176C,C17676T,C18183T,C19524T,A21519C,A23063T,C23271A,A23403G,C23604A,C23709T,A24070T,T24506G,G24914C,C25624T,G26849T,C27972T,G28048T,A28095T,A28111G,G28280C,A28281T,T28282A,G28881A,G28882A,G28883C,G28884C,C28977T	24	M:M109I,N:D3L,N:R203K,N:G204P,N:S235F,ORF1a:T1001I,ORF1a:A1708D,ORF1a:A2128T,ORF1a:I2230T,ORF1b:P314L,ORF1b:R2684S,ORF3a:H78Y,ORF8:Q27*,ORF8:R52I,ORF8:K68*,ORF8:Y73C,S:N501Y,S:A570D,S:D614G,S:P681H,S:T716I,S:Q836H,S:S982A,S:D1118H	6	94
hCoV-19/Mexico/MEX-INMEGEN-05-04-95/2021	EPI_ISL_2349953	-	B.1.1.7	GRY	40	C241T,C913T,G1957A,A2276G,G2527A,C3037T,A3094T,C3267T,G4136T,C5388A,C5986T,T6954C,G12514T,C14408T,C14676T,C15279T,T16176C,C18788T,G19542T,G22841A,A23063T,C23271A,A23403G,C23604A,C23709T,T24506G,G24914C,G25954T,C27972T,G28048T,A28111G,G28280C,A28281T,T28282A,C28744T,G28881A,G28882A,G28883C,C28977T,G29134T	24	N:D3L,N:R203K,N:G204R,N:S235F,ORF1a:I671V,ORF1a:T1001I,ORF1a:V1291F,ORF1a:A1708D,ORF1a:I2230T,ORF1b:P314L,ORF1b:T1774I,ORF1b:M2025I,ORF3a:G188C,ORF8:Q27*,ORF8:R52I,ORF8:Y73C,S:D427N,S:N501Y,S:A570D,S:D614G,S:P681H,S:T716I,S:S982A,S:D1118H	6	5
hCoV-19/Mexico/MEX-INMEGEN-05-05-102/2021	EPI_ISL_2444462	-	B.1.1.7	GR	34	C241T,C913T,C3037T,C3267T,C4331T,C5388A,C5986T,T6954C,C14408T,C14676T,T15096C,C15279T,T16176C,C19164T,C23271A,A23403G,C23604A,C23709T,T24280C,T24506G,G24914C,C25096T,G27459T,C27972T,G28048T,A28095T,A28111G,G28280C,A28281T,T28282A,G28881A,G	19	N:D3L,N:R203K,N:G204R,N:S235F,ORF1a:T1001I,ORF1a:A1708D,ORF1a:I2230T,ORF1b:P314L,ORF7a:E22D,ORF8:Q27*,ORF8:R52I,ORF8:K68*,ORF8:Y73C,S:A570D,S:D614G,S:P681H,S:T716I,S:S982A,S:D1118H	6	408
hCoV-19/Mexico/MEX-INMEGEN-05-05-112/2021	EPI_ISL_2444469	-	B.1.1.7	GR	36	C241T,C913T,G1085T,C3037T,C3267T,C5388A,C5986T,T6954C,T12862C,A14125G,C14408T,C14676T,C15279T,T16176C,A17615G,G19414T,G21839T,G22801T,C23271A,A23403G,C23604A,C23709T,T24506G,G24914C,G25440T,A26973G,C27972T,G28048T,A28111G,G28280C,A28281T,T28282A,G28881A,G28882A,G28883C,C28977T	24	M:I151V,N:D3L,N:R203K,N:G204R,N:S235F,ORF1a:V274L,ORF1a:T1001I,ORF1a:A1708D,ORF1a:I2230T,ORF1b:S220G,ORF1b:P314L,ORF1b:K1383R,ORF1b:V1983L,ORF3a:K16N,ORF8:Q27*,ORF8:R52I,ORF8:Y73C,S:A93S,S:A570D,S:D614G,S:P681H,S:T716I,S:S982A,S:D1118H	6	254
hCoV-19/Mexico/MEX-INMEGEN-05-05-113/2021	EPI_ISL_2444470	-	B.1.1.7	GRY	34	C241T,C913T,C1473T,A1631G,T2201A,C3037T,C3267T,A3933C,C5388A,C5986T,T6954C,C11750T,C14408T,C14676T,C15279T,T16176C,A23063T,C23271A,A23403G,C23604A,C23709T,T24506G,G24914C,C26058T,C27972T,G28048T,A28111G,G28280C,A28281T,T28282A,G28881A,G28882A,G28883C,C28977T	23	N:D3L,N:R203K,N:G204R,N:S235F,ORF1a:T403I,ORF1a:K456E,ORF1a:W646R,ORF1a:T1001I,ORF1a:E1223A,ORF1a:A1708D,ORF1a:I2230T,ORF1a:L3829F,ORF1b:P314L,ORF8:Q27*,ORF8:R52I,ORF8:Y73C,S:N501Y,S:A570D,S:D614G,S:P681H,S:T716I,S:S982A,S:D1118H	6	341
hCoV-19/Mexico/MEX-INMEGEN-05-05-136/2021	EPI_ISL_2550747	-	Q.3	GRY	39	G152A,C241T,C913T,C3037T,C3267T,G5023A,C5388A,C5986T,C6568T,T6954C,G8183A,A9529G,C9541T,G10396T,C14408T,C14676T,C15279T,T16176C,A17615G,G20962A,G22094C,A23063T,C23271A,A23403G,C23604A,C23709T,T24506G,G24914C,G26730C,C27972T,G28048T,A28111G,G28280C,A28281T,T28282A,G28881A,G28882A,G28883C,C28977T	24	M:V70L,N:D3L,N:R203K,N:G204R,N:S235F,ORF1a:T1001I,ORF1a:M1586I,ORF1a:A1708D,ORF1a:I2230T,ORF1a:G2640R,ORF1b:P314L,ORF1b:K1383R,ORF1b:D2499N,ORF8:Q27*,ORF8:R52I,ORF8:Y73C,S:D178H,S:N501Y,S:A570D,S:D614G,S:P681H,S:T716I,S:S982A,S:D1118H	6	0
hCoV-19/Mexico/MEX-INMEGEN-05-05-148/2021	EPI_ISL_2444489	-	B.1.1.7	GR	33	C241T,C913T,C3037T,C3267T,C5388A,C5986T,C6196T,T6954C,A8848G,C14408T,C14676T,C15279T,T16176C,C23271A,A23403G,C23604A,C23709T,T24506G,G24914C,G25217T,G26727T,C27972T,G28048T,A28111G,G28280C,A28281T,T28282A,G28621A,G28881A,G28882A,G28883C,C	19	M:A69S,N:D3L,N:R203K,N:G204R,N:S235F,ORF1a:T1001I,ORF1a:A1708D,ORF1a:I2230T,ORF1b:P314L,ORF8:Q27*,ORF8:R52I,ORF8:Y73C,S:A570D,S:D614G,S:P681H,S:T716I,S:S982A,S:D1118H,S:G1219C	6	986
hCoV-19/Mexico/MEX-INMEGEN-05-05-149/2021	EPI_ISL_2881804	-	B.1.1.7	GR	39	C241T,C539T,C913T,C3037T,C3267T,C4655T,C5388A,C5986T,T6954C,C7749T,A9869G,C11941T,C14408T,C14676T,C15108T,C15279T,T16176C,T16905C,A17615G,C18032T,C23271A,A23403G,C23604A,C23709T,T24506G,G24914C,G26730C,C27143T,C27972T,G28048T,A28111G,G28280C,A28281T,T28282A,C28603T,G28881A,G28882A,G28883C,C28977T	24	M:V70L,N:D3L,N:R203K,N:G204R,N:S235F,ORF1a:L92F,ORF1a:T1001I,ORF1a:R1464W,ORF1a:A1708D,ORF1a:I2230T,ORF1a:T2495I,ORF1a:T3202A,ORF1b:P314L,ORF1b:K1383R,ORF1b:T1522I,ORF8:Q27*,ORF8:R52I,ORF8:Y73C,S:A570D,S:D614G,S:P681H,S:T716I,S:S982A,S:D1118H	6	768
hCoV-19/Mexico/MEX-INMEGEN-05-05-153/2021	EPI_ISL_2444492	-	B.1.1.7	GRY	34	C241T,C913T,C1473T,A1631G,T2201A,C3037T,C3267T,A3933C,C5388A,C5986T,T6954C,C11750T,C14408T,C14676T,C15279T,T16176C,A23063T,C23271A,A23403G,C23604A,C23709T,T24506G,G24914C,C26058T,C27972T,G28048T,A28111G,G28280C,A28281T,T28282A,G28881A,G28882A,G28883C,C28977T	23	N:D3L,N:R203K,N:G204R,N:S235F,ORF1a:T403I,ORF1a:K456E,ORF1a:W646R,ORF1a:T1001I,ORF1a:E1223A,ORF1a:A1708D,ORF1a:I2230T,ORF1a:L3829F,ORF1b:P314L,ORF8:Q27*,ORF8:R52I,ORF8:Y73C,S:N501Y,S:A570D,S:D614G,S:P681H,S:T716I,S:S982A,S:D1118H	6	20

hCoV-19/Mexico/MEX-INMEGEN-05-05-156/2021	EPI_ISL_2444494	-	B.1.1.7	GR	41	C241T,C913T,T1177C,T1537G,C2110T,G2528A,C3037T,C3267T,C4296T,C5142T,C5388A,C5986T,T6954C,G7042T,C10789T,C14120T,C14408T,C14676T,C15279T,T16176C,C20470A,C23271A,A23403G,C23604A,C23709T,T24506G,G24914C,C25452T,G25471T,C27972T,G28048T,A28095T,A28111G,G28280C,A28281T,T28282A,G28881A,G28882A,G28883C,C28977T,C29167T	25	N:D3L,N:R203K,N:G204R,N:S235F,ORF1a:E755K,ORF1a:T1001I,ORF1a:T1344I,ORF1a:T1626I,ORF1a:A1708D,ORF1a:I2230T,ORF1a:M2259I,ORF1b:P218L,ORF1b:P314L,ORF1b:Q2335K,ORF3a:D27Y,ORF8:Q27*,ORF8:R52I,ORF8:K68*,ORF8:Y73C,S:A570D,S:D614G,S:P681H,S:T716I,S:S982A,S:D1118H	6	509
hCoV-19/Mexico/MEX-INMEGEN-05-05-161/2021	EPI_ISL_2444497	-	B.1.1.7	G	33	C913T,C2110T,C3037T,C3267T,C3393T,C5388A,C5986T,T6954C,G7042T,C7834T,A10195G,C14120T,C14408T,C14676T,C15279T,T16176C,C16833T,C19152A,C23271A,A23403G,C23604A,C23709T,T24506G,G24914C,C27972T,G28048T,G28075T,A28095T,A28111G,G28280C,A28281T,T28	20	N:D3L,N:S235F,ORF1a:T1001I,ORF1a:A1043V,ORF1a:A1708D,ORF1a:I2230T,ORF1a:M2259I,ORF1b:P218L,ORF1b:P314L,ORF8:Q27*,ORF8:R52I,ORF8:C61F,ORF8:K68*,ORF8:Y73C,S:A570D,S:D614G,S:P681H,S:T716I,S:S982A,S:D1118H	8	2555
hCoV-19/Mexico/MEX-INMEGEN-05-05-166/2021	EPI_ISL_2444500	-	B.1.1.7	GR	32	C241T,C913T,C3037T,C3267T,C5388A,C5986T,C6196T,T6954C,A8848G,C14408T,C14676T,C15279T,T16176C,C23271A,A23403G,C23604A,C23709T,T24506G,G24914C,G25217T,G26727T,C27972T,G28048T,A28111G,G28280C,A28281T,T28282A,G28881A,G28882A,G28883C,C28977T,G	19	M:A69S,N:D3L,N:R203K,N:G204R,N:S235F,ORF1a:T1001I,ORF1a:A1708D,ORF1a:I2230T,ORF1b:P314L,ORF8:Q27*,ORF8:R52I,ORF8:Y73C,S:A570D,S:D614G,S:P681H,S:T716I,S:S982A,S:D1118H,S:G1219C	6	379
hCoV-19/Mexico/MEX-INMEGEN-05-05-169/2021	EPI_ISL_2444502	-	B.1.1.7	GRY	40	C241T,C913T,C2110T,C3037T,C3267T,C3393T,G3728T,C5388A,C5986T,T6954C,G7042T,C7834T,A10195G,C14120T,C14408T,C14676T,C15279T,T16176C,C16833T,C19152A,C22347T,A23063T,C23271A,A23403G,C23604A,C23709T,T24506G,G24914C,C27972T,G28048T,G28075T,A28095T,A28111G,G28280C,A28281T,T28282A,G28881A,G28882A,G28883C,C28977T	25	N:D3L,N:R203K,N:G204R,N:S235F,ORF1a:T1001I,ORF1a:A1043V,ORF1a:G1155C,ORF1a:A1708D,ORF1a:I2230T,ORF1a:M2259I,ORF1b:P218L,ORF1b:P314L,ORF8:Q27*,ORF8:R52I,ORF8:C61F,ORF8:K68*,ORF8:Y73C,S:A262V,S:N501Y,S:A570D,S:D614G,S:P681H,S:T716I,S:S982A,S:D1118H	8	157
hCoV-19/Mexico/MEX-INMEGEN-05-05-200/2021	EPI_ISL_2550752	-	B.1.1.7	GRY	34	C241T,C913T,C3037T,C3267T,A3492G,C4331T,C5388A,C5986T,T6954C,C14408T,C14676T,C15279T,T16176C,C18555T,C21639A,A23063T,C23271A,A23403G,C23604A,C23709T,C24374T,T24506G,G24914C,C27972T,G28048T,A28111G,G28280C,A28281T,T28282A,G28881A,G28882A,G2	21	N:D3L,N:R203K,N:G204R,N:S235F,ORF1a:T1001I,ORF1a:N1076S,ORF1a:A1708D,ORF1a:I2230T,ORF1b:P314L,ORF8:Q27*,ORF8:R52I,ORF8:Y73C,S:P26H,S:N501Y,S:A570D,S:D614G,S:P681H,S:T716I,S:L938F,S:S982A,S:D1118H	6	76
hCoV-19/Mexico/MEX-INMEGEN-05-05-204/2021	EPI_ISL_2444521	-	B.1.1.7	G	30	C241T,C913T,C3037T,C3267T,C5388A,C5986T,T6954C,A13768T,C14408T,C14676T,T15096C,C15279T,T16176C,C23271A,A23403G,C23520T,C23604A,C23709T,T24506G,G24914C,C26606T,C27972T,G28048T,A28095T,A28111G,G28280C,A28281T,T28282A,G28300T,C28977T	20	N:D3L,N:Q9H,N:S235F,ORF1a:T1001I,ORF1a:A1708D,ORF1a:I2230T,ORF1b:M101L,ORF1b:P314L,ORF8:Q27*,ORF8:R52I,ORF8:K68*,ORF8:Y73C,ORF9b:S6I,S:A570D,S:D614G,S:A653V,S:P681H,S:T716I,S:S982A,S:D1118H	6	1503
hCoV-19/Mexico/MEX-INMEGEN-05-05-205/2021	EPI_ISL_2444522	-	B.1.1.7	GRY	40	C222T,C241T,C913T,C2110T,C3037T,C3267T,C5388A,C5986T,T6954C,G7042T,C8389T,C12115T,C13517T,C14120T,C14408T,C14676T,C15279T,T16176C,G16900T,C17762T,A23063T,C23271A,A23403G,C23604A,C23709T,T24506G,G24914C,T25180C,G26416T,C27972T,G28048T,A28095T,A28111G,G28280C,A28281T,T28282A,G28881A,G28882A,G28883C,C28977T	25	E:V58F,N:D3L,N:R203K,N:G204R,N:S235F,ORF1a:T1001I,ORF1a:A1708D,ORF1a:I2230T,ORF1a:M2259I,ORF1b:T17I,ORF1b:P218L,ORF1b:P314L,ORF1b:G1145C,ORF1b:A1432V,ORF8:Q27*,ORF8:R52I,ORF8:K68*,ORF8:Y73C,S:N501Y,S:A570D,S:D614G,S:P681H,S:T716I,S:S982A,S:D1118H	6	0
hCoV-19/Mexico/MEX-INMEGEN-05-05-212/2021	EPI_ISL_2444525	-	B.1.1.7	GRY	40	C203T,C241T,C913T,G1820A,C3037T,C3267T,G5023T,C5388A,G5924A,C5986T,T6954C,G9526T,A12162G,C14408T,C14676T,C15279T,C15720T,T16176C,A17615G,T19363C,A21137G,A23063T,C23248T,C23271A,A23403G,C23604A,C23709T,T24506G,G24914C,G25135T,C27972T,G28048T,A28111G,G28280C,A28281T,T28282A,G28881A,G28882A,G28883C,C28977T	26	N:D3L,N:R203K,N:G204R,N:S235F,ORF1a:G519S,ORF1a:T1001I,ORF1a:M1586I,ORF1a:A1708D,ORF1a:V1887I,ORF1a:I2230T,ORF1a:M3087I,ORF1a:Q3966R,ORF1b:P314L,ORF1b:K1383R,ORF1b:K2557R,ORF8:Q27*,ORF8:R52I,ORF8:Y73C,S:N501Y,S:A570D,S:D614G,S:P681H,S:T716I,S:S982A,S:D1118H,S:K1191N	6	54
hCoV-19/Mexico/MEX-INMEGEN-05-05-224/2021	EPI_ISL_2444534	-	Q.3	GRY	40	C241T,C913T,G2229A,C3037T,C3267T,G5023A,C5388A,C5986T,C6568T,T6954C,A9529G,G10396T,C14408T,C14676T,C15279T,T16176C,A17615G,C18131T,G18984T,G20962A,G22094C,A23063T,C23271A,A23403G,C23604A,C23709T,C24378T,T24506G,G24914C,G26730C,C27972T,G28048T,A28111G,G28280C,A28281T,T28282A,G28881A,G28882A,G28883C,C28977T	27	M:V70L,N:D3L,N:R203K,N:G204R,N:S235F,ORF1a:C655Y,ORF1a:T1001I,ORF1a:M1586I,ORF1a:A1708D,ORF1a:I2230T,ORF1b:P314L,ORF1b:K1383R,ORF1b:T1555I,ORF1b:M1839I,ORF1b:D2499N,ORF8:Q27*,ORF8:R52I,ORF8:Y73C,S:D178H,S:N501Y,S:A570D,S:D614G,S:P681H,S:T716I,S:S939F,S:S982A,S:D1118H	6	169
hCoV-19/Mexico/MEX-INMEGEN-05-05-229/2021	EPI_ISL_2550754	-	B.1.1.7	GRY	42	C241T,C913T,C1426T,G2281A,C3037T,C3267T,C5388A,C5986T,T6954C,A12162G,C13665T,C14408T,C14676T,C15279T,C15720T,T16176C,A17615G,C18747T,C20844T,A21137G,A23063T,C23271A,A23403G,C23604A,C23709T,T24506G,G24914C,G25135T,C26110T,C26521T,G26828T,C27972T,G28048T,A28111G,G28280C,A28281T,T28282A,G28881A,G28882A,G28883C,C28977T,C2	23	N:D3L,N:R203K,N:G204R,N:S235F,ORF1a:T1001I,ORF1a:A1708D,ORF1a:I2230T,ORF1a:Q3966R,ORF1b:P314L,ORF1b:K1383R,ORF1b:K2557R,ORF3a:P240S,ORF8:Q27*,ORF8:R52I,ORF8:Y73C,S:N501Y,S:A570D,S:D614G,S:P681H,S:T716I,S:S982A,S:D1118H,S:K1191N	6	0
hCoV-19/Mexico/MEX-INMEGEN-05-05-247/2021	EPI_ISL_2444546	-	B.1.1.7	GR	43	C241T,C913T,C3037T,C3267T,C4206T,C4900T,C5388A,C5986T,T6954C,C6982T,C11941T,A12162G,C13824T,C14230T,C14408T,C14676T,C15279T,C15720T,T16176C,A17615G,A21137G,C23271A,A23403G,C23604A,C23709T,G24047T,T24506G,G24914C,G25135T,G25302T,G27014T,C27972T,G28048T,A28111G,G28280C,A28281T,T28282A,C28791T,G28881A,G28882A,G28883C,C28	26	N:D3L,N:A173V,N:R203K,N:G204R,N:S235F,ORF1a:T1001I,ORF1a:A1314V,ORF1a:A1708D,ORF1a:I2230T,ORF1a:Q3966R,ORF1b:P255S,ORF1b:P314L,ORF1b:K1383R,ORF1b:K2557R,ORF8:Q27*,ORF8:R52I,ORF8:Y73C,S:A570D,S:D614G,S:P681H,S:T716I,S:A829S,S:S982A,S:D1118H,S:K1191N,S:C1247F	6	764
hCoV-19/Mexico/MEX-INMEGEN-05-05-279/2021	EPI_ISL_2444558	-	B.1.1.7	GR	42	T201A,C241T,C913T,C2110T,C2675T,A2693T,C3037T,C3267T,C5388A,C5986T,T6954C,G7042T,A10195G,C13329T,C14120T,C14408T,C14676T,C15279T,T16176C,C21811T,T22679C,C23271A,A23403G,C23604A,C23709T,T24506G,G24914C,C25046G,C25708T,C26270T,C27972T,G28048T,G28075T,A28095T,A28111G,G28280C,A28281T,T28282A,G28881A,G28882A,G28883C,C28977	28	E:T9I,N:D3L,N:R203K,N:G204R,N:S235F,ORF1a:P804S,ORF1a:N810Y,ORF1a:T1001I,ORF1a:A1708D,ORF1a:I2230T,ORF1a:M2259I,ORF1a:T4355I,ORF1b:P218L,ORF1b:P314L,ORF3a:L106F,ORF8:Q27*,ORF8:R52I,ORF8:C61F,ORF8:K68*,ORF8:Y73C,S:S373P,S:A570D,S:D614G,S:P681H,S:T716I,S:S982A,S:D1118H,S:P	6	895
hCoV-19/Mexico/MEX-INMEGEN-05-05-3/2021	EPI_ISL_2444399	-	B.1.1.7	GRY	34	C241T,C913T,C3037T,C3267T,A3492G,C4331T,C5388A,C5986T,T6954C,C14408T,C14676T,C15279T,T16176C,C18555T,C21639A,A23063T,C23271A,A23403G,C23604A,C23709T,C24374T,T24506G,G24914C,C27972T,G28048T,A28111G,G28280C,A28281T,T28282A,G28881A,G28882A,G2	21	N:D3L,N:R203K,N:G204R,N:S235F,ORF1a:T1001I,ORF1a:N1076S,ORF1a:A1708D,ORF1a:I2230T,ORF1b:P314L,ORF8:Q27*,ORF8:R52I,ORF8:Y73C,S:P26H,S:N501Y,S:A570D,S:D614G,S:P681H,S:T716I,S:L938F,S:S982A,S:D1118H	6	0
hCoV-19/Mexico/MEX-INMEGEN-05-05-30/2021	EPI_ISL_2444418	-	B.1.1.7	GRY	41	C241T,A435G,C913T,G1957A,C2189T,A2276G,G2527A,C3037T,A3094T,C3267T,G4136T,C5388A,C5986T,T6954C,C11704T,C11750T,C14408T,C14676T,C15279T,T16176C,G19542T,G22841A,A23063T,C23271A,A23403G,C23604A,C23709T,T24506G,G24914C,G25954T,C27972T,G28048T,A28111G,G28280C,A28281T,T28282A,C28744T,G28881A,G28882A,G28883C,C28977T	26	N:D3L,N:R203K,N:G204R,N:S235F,ORF1a:E57G,ORF1a:L642F,ORF1a:I671V,ORF1a:T1001I,ORF1a:V1291F,ORF1a:A1708D,ORF1a:I2230T,ORF1a:L3829F,ORF1b:P314L,ORF1b:M2025I,ORF3a:G188C,ORF8:Q27*,ORF8:R52I,ORF8:Y73C,S:D427N,S:N501Y,S:A570D,S:D614G,S:P681H,S:T716I,S:S982A,S:D1118H	6	5
hCoV-19/Mexico/MEX-INMEGEN-05-05-34/2021	EPI_ISL_2444421	-	B.1.1.7	G	35	C241T,C913T,C3037T,C3267T,C5388A,C5986T,T6954C,C7299T,A13768T,C14408T,C14676T,T15096C,C15279T,T16176C,T16728C,A18262G,A23063T,C23271A,A23403G,C23520T,C23604A,C23709T,T24506G,G24914C,C26606T,C27972T,G28048T,A28095T,A28111G,G28280C,A28281T,T28282A,G28300T,A28877T,C28977T	23	N:D3L,N:Q9H,N:S235F,ORF1a:T1001I,ORF1a:A1708D,ORF1a:I2230T,ORF1a:A2345V,ORF1b:M101L,ORF1b:P314L,ORF1b:T1599A,ORF8:Q27*,ORF8:R52I,ORF8:K68*,ORF8:Y73C,ORF9b:S6I,S:N501Y,S:A570D,S:D614G,S:A653V,S:P681H,S:T716I,S:S982A,S:D1118H	6	6
hCoV-19/Mexico/MEX-INMEGEN-05-05-39/2021	EPI_ISL_2444424	-	B.1.1.7	GRY	42	C241T,C913T,G1069A,C1609T,C3037T,T3049C,C3267T,C5388A,G5558A,C5986T,T6954C,C8655T,A12162G,G14294A,C14408T,C14676T,C15279T,C15720T,T16176C,A17615G,C18329T,A21137G,C22636T,A23063T,C23271A,A23403G,C23604A,C23709T,T24506G,G24914C,G25135T,C27972T,G28048T,A28111G,G28280C,A28281T,T28282A,G28881A,G28882A,G28883C,G28916A,C28	27	N:D3L,N:R203K,N:G204R,N:G215S,N:S235F,ORF1a:T1001I,ORF1a:A1708D,ORF1a:V1765I,ORF1a:I2230T,ORF1a:S2797F,ORF1a:Q3966R,ORF1b:R276H,ORF1b:P314L,ORF1b:K1383R,ORF1b:T1621I,ORF1b:K2557R,ORF8:Q27*,ORF8:R52I,ORF8:Y73C,S:N501Y,S:A570D,S:D614G,S:P681H,S:T716I,S:S982A,S:D1118H,S:	6	32
hCoV-19/Mexico/MEX-INMEGEN-05-05-40/2021	EPI_ISL_2444425	-	B.1.1.7	GRY	36	C241T,C913T,C2937T,C3037T,C3267T,C5388A,C5986T,C6196T,T6954C,A8848G,A11898G,C14408T,C14676T,C15279T,T16176C,T19624C,A23063T,C23271A,A23403G,C23604A,C23709T,T24506G,G24914C,G25217T,C26270T,C27972T,G28048T,A28111G,G28280C,A28281T,T28282A,G28881A,G28882A,G28883C,C28977T,G29715T	22	E:T9I,N:D3L,N:R203K,N:G204R,N:S235F,ORF1a:T891I,ORF1a:T1001I,ORF1a:A1708D,ORF1a:I2230T,ORF1a:Q3878R,ORF1b:P314L,ORF8:Q27*,ORF8:R52I,ORF8:Y73C,S:N501Y,S:A570D,S:D614G,S:P681H,S:T716I,S:S982A,S:D1118H,S:G1219C	6	79
hCoV-19/Mexico/MEX-INMEGEN-05-05-42/2021	EPI_ISL_2444426	-	B.1.1.7	GRY	39	C241T,C913T,G1487T,C2110T,C3037T,C3267T,C3393T,C5388A,C5986T,T6954C,G7042T,C7834T,A10195G,C14120T,C14408T,C14676T,C15279T,T16176C,C16833T,C19152A,A23063T,C23271A,A23403G,C23604A,C23709T,T24506G,G24914C,C27972T,G28048T,G28075T,A28095T,A28111G,G28280C,A28281T,T28282A,G28881A,G28882A,G28883C,C28977T	24	N:D3L,N:R203K,N:G204R,N:S235F,ORF1a:G408C,ORF1a:T1001I,ORF1a:A1043V,ORF1a:A1708D,ORF1a:I2230T,ORF1a:M2259I,ORF1b:P218L,ORF1b:P314L,ORF8:Q27*,ORF8:R52I,ORF8:C61F,ORF8:K68*,ORF8:Y73C,S:N501Y,S:A570D,S:D614G,S:P681H,S:T716I,S:S982A,S:D1118H	6	184
hCoV-19/Mexico/MEX-INMEGEN-05-05-46/2021	EPI_ISL_2444428	-	B.1.1.7	GRY	38	C241T,C815T,C913T,C3037T,C3267T,C5388A,C5986T,T6954C,G8513A,C9491T,A12162G,C13051T,C14408T,C14676T,C15279T,C15720T,T16176C,A17615G,A21137G,A23063T,C23271A,A23403G,C23604A,C23709T,T24506G,G24914C,G25135T,C27903T,C27972T,G28048T,A28111G,G28280C,A28281T,T28282A,G28881A,G28882A,G28883C,C28977T	26	N:D3L,N:R203K,N:G204R,N:S235F,ORF1a:R184C,ORF1a:T1001I,ORF1a:A1708D,ORF1a:I2230T,ORF1a:V2750I,ORF1a:H3076Y,ORF1a:Q3966R,ORF1b:P314L,ORF1b:K1383R,ORF1b:K2557R,ORF8:L4F,ORF8:Q27*,ORF8:R52I,ORF8:Y73C,S:N501Y,S:A570D,S:D614G,S:P681H,S:T716I,S:S982A,S:D1118H,S:K1191N	6	156
hCoV-19/Mexico/MEX-INMEGEN-05-05-47/2021	EPI_ISL_2444429	-	B.1.1.7	GRY	37	G174T,G210T,C241T,C913T,C2453T,C3037T,C3267T,C5388A,C5986T,T6954C,A8588G,T9016C,C14408T,C14676T,C15279T,T16176C,G21867T,A23063T,C23271A,A23403G,C23604A,C23709T,T24506G,G24914C,C25916T,C27883T,C27972T,G28048T,A28111G,G28280C,A28281T,T28282A,C28453T,G28881A,G28882A,G28883C,C28977T	24	N:D3L,N:R203K,N:G204R,N:S235F,ORF1a:L730F,ORF1a:T1001I,ORF1a:A1708D,ORF1a:I2230T,ORF1a:K2775E,ORF1b:P314L,ORF3a:T175I,ORF7b:A43V,ORF8:Q27*,ORF8:R52I,ORF8:Y73C,ORF9b:A57V,S:R102I,S:N501Y,S:A570D,S:D614G,S:P681H,S:T716I,S:S982A,S:D1118H	6	56

hCoV-19/Mexico/MEX-INMEGEN-05-05-50/2021	EPI_ISL_2444432	-	B.1.1.7	GRY	35	C241T,C913T,C3037T,C3267T,C5388A,C5986T,C6196T,T6954C,A8848G,C14408T,C14676T,C15279T,T16176C,G19816T,A23063T,C23271A,A23403G,C23604A,C23709T,T24506G,G24914C,G25217T,G26727T,T27285C,C27972T,G28048T,A28111G,G28280C,A28281T,T28282A,G28881A,G28882A,G28883C,C28977T,G29715T	21	M:A69S,N:D3L,N:R203K,N:G204R,N:S235F,ORF1a:T1001I,ORF1a:A1708D,ORF1a:I2230T,ORF1b:P314L,ORF1b:V2117L,ORF8:Q27*,ORF8:R52I,ORF8:Y73C,S:N501Y,S:A570D,S:D614G,S:P681H,S:T716I,S:S982A,S:D1118H,S:G1219C	6	20
hCoV-19/Mexico/MEX-INMEGEN-05-05-58/2021	EPI_ISL_2444438	-	B.1.1.7	GRY	44	C203T,C241T,C913T,G1820A,G2588A,C3037T,C3267T,C4456T,C5388A,C5986T,T6954C,A12162G,C14408T,C14676T,C15279T,C15720T,T16176C,C16338T,A17615G,A21137G,C22858T,A23063T,C23248T,C23271A,A23403G,C23604A,G23608T,C23709T,T24506G,A24568T,G24914C,G25135T,C27972T,G28001A,G28048T,A28111G,G28280C,A28281T,T28282A,G28881A,G28882A,G28	26	N:D3L,N:R203K,N:G204R,N:S235F,N:A308S,ORF1a:G519S,ORF1a:E775K,ORF1a:T1001I,ORF1a:A1708D,ORF1a:I2230T,ORF1a:Q3966R,ORF1b:P314L,ORF1b:K1383R,ORF1b:K2557R,ORF8:Q27*,ORF8:R52I,ORF8:Y73C,S:N501Y,S:A570D,S:D614G,S:P681H,S:T716I,S:S982A,S:Q1002H,S:D1118H,S:K1191N	6	381
hCoV-19/Mexico/MEX-INMEGEN-05-05-72/2021	EPI_ISL_2444450	-	B.1.1.7	G	32	C241T,C913T,C2110T,C3037T,C3267T,C5388A,C5986T,T6954C,G7042T,A10195G,C14120T,C14408T,C14676T,C15279T,T16176C,C23271A,A23403G,C23604A,A23690G,C23709T,T24506G,G24914C,C27972T,G28048T,G28075T,A28095T,A28111G,G28280C,A28281T,T28282A,C28977T,C2	20	N:D3L,N:S235F,ORF1a:T1001I,ORF1a:A1708D,ORF1a:I2230T,ORF1a:M2259I,ORF1b:P218L,ORF1b:P314L,ORF8:Q27*,ORF8:R52I,ORF8:C61F,ORF8:K68*,ORF8:Y73C,S:A570D,S:D614G,S:P681H,S:N710D,S:T716I,S:S982A,S:D1118H	6	1539
hCoV-19/Mexico/MEX-INMEGEN-05-05-96/2021	EPI_ISL_2550741	-	B.1.1.7	GR	40	C241T,C913T,C1722T,G1957A,A2276G,G2527A,C3037T,A3094T,C3267T,G4136T,C5388A,C5986T,T6954C,C10681T,C12357T,C14408T,C14599T,C14676T,C15279T,G15372T,T16176C,G19542T,G22841A,C23271A,A23403G,C23604A,C23709T,T24506G,G24914C,G25954T,C27972T,G28048T,A28111G,G28280C,A28281T,T28282A,G28881A,G28882A,G28883C,C28977T	24	N:D3L,N:R203K,N:G204R,N:S235F,ORF1a:A486V,ORF1a:I671V,ORF1a:T1001I,ORF1a:V1291F,ORF1a:A1708D,ORF1a:I2230T,ORF1a:T4031I,ORF1b:P314L,ORF1b:M2025I,ORF3a:G188C,ORF8:Q27*,ORF8:R52I,ORF8:Y73C,S:D427N,S:A570D,S:D614G,S:P681H,S:T716I,S:S982A,S:D1118H	6	470
hCoV-19/Mexico/MEX-INMEGEN-05-06-193/2021	EPI_ISL_2603682	-	B.1.1.7	GR	38	C241T,C913T,C2110T,C3037T,C3267T,C3623T,T5278C,C5388A,C5986T,T6954C,G7042T,A8879G,G11083T,C14120T,C14408T,C14676T,C15279T,T16176C,G21578T,C23271A,A23403G,C23604A,C23709T,G24415T,G24914C,G25906T,C26058T,C27972T,G28048T,A28095T,A28111G,G28280C,A28281T,T28282A,G28881A,G28882A,G28883C,C28977T	24	N:D3L,N:R203K,N:G204R,N:S235F,ORF1a:T1001I,ORF1a:P1120S,ORF1a:A1708D,ORF1a:I2230T,ORF1a:M2259I,ORF1a:T2872A,ORF1a:L3606F,ORF1b:P218L,ORF1b:P314L,ORF3a:G172C,ORF8:Q27*,ORF8:R52I,ORF8:K68*,ORF8:Y73C,S:V6F,S:A570D,S:D614G,S:P681H,S:T716I,S:D1118H	6	603
hCoV-19/Mexico/MEX-INMEGEN-05-06-218/2021	EPI_ISL_2881838	-	B.1.1.7	G	31	C241T,C913T,C3037T,C3267T,C5388A,C5986T,C6196T,T6954C,A8848G,C14408T,C14676T,C15279T,T16176C,G19816T,C23271A,A23403G,C23604A,C23709T,T24506G,G24914C,G25217T,G26727T,T27285C,C27972T,G28048T,A28111G,G28280C,A28281T,T28282A,C28977T,G29715T	18	M:A69S,N:D3L,N:S235F,ORF1a:T1001I,ORF1a:A1708D,ORF1a:I2230T,ORF1b:P314L,ORF1b:V2117L,ORF8:Q27*,ORF8:R52I,ORF8:Y73C,S:A570D,S:D614G,S:P681H,S:T716I,S:S982A,S:D1118H,S:G1219C	6	1908
hCoV-19/Mexico/MEX-INMEGEN-05-06-234/2021	EPI_ISL_2603715	-	B.1.1.7	GRY	42	C241T,C539A,C913T,C3037T,C3267T,G4160T,C5388A,C5986T,T6954C,C7299T,C11074T,C11750T,C14408T,C14676T,T15096C,C15279T,T16176C,C17977T,C18252T,T21820C,A23063T,C23271A,A23403G,C23604A,C23709T,T24506G,G24914C,G25489T,T26149C,C27972T,G28048T,G28077C,A28095T,A28111G,G28280C,A28281T,T28282A,C28807T,G28881A,G28882A,G28883C,C2897	27	N:D3L,N:R203K,N:G204R,N:S235F,ORF1a:L92I,ORF1a:T1001I,ORF1a:V1299L,ORF1a:A1708D,ORF1a:I2230T,ORF1a:A2345V,ORF1a:L3829F,ORF1b:P314L,ORF1b:L1504F,ORF3a:A33S,ORF3a:S253P,ORF8:Q27*,ORF8:R52I,ORF8:V62L,ORF8:K68*,ORF8:Y73C,S:N501Y,S:A570D,S:D614G,S:P681H,S:T716I,S:S982A,S:D111	6	15
hCoV-19/Mexico/MEX-INMEGEN-05-06-235/2021	EPI_ISL_2603716	-	B.1.1.7	GRY	34	C241T,C913T,C2061T,C3037T,C3267T,C5388A,C5986T,T6954C,C12115T,A12162G,C14408T,C14676T,C15279T,T16176C,A17615G,G20275T,A23063T,C23271A,A23403G,C23604A,C23709T,T24506G,G24914C,C27855A,C27972T,G28048T,A28111G,G28280C,A28281T,T28282A,G28881A,G28882A,G28883C,C28977T	23	N:D3L,N:R203K,N:G204R,N:S235F,ORF1a:A599V,ORF1a:T1001I,ORF1a:A1708D,ORF1a:I2230T,ORF1a:Q3966R,ORF1b:P314L,ORF1b:K1383R,ORF1b:D2270Y,ORF7b:L34M,ORF8:Q27*,ORF8:R52I,ORF8:Y73C,S:N501Y,S:A570D,S:D614G,S:P681H,S:T716I,S:S982A,S:D1118H	6	339
hCoV-19/Mexico/MEX-INMEGEN-05-06-250/2021	EPI_ISL_2603723	-	B.1.1.7	GR	34	C241T,C913T,C3037T,C3267T,C5388A,C5986T,C6196T,T6954C,A8848G,C14408T,C14676T,C15279T,T16176C,G19816T,C23271A,A23403G,C23604A,C23709T,T24506G,G24914C,G25217T,G26727T,T27285C,C27972T,G28048T,A28111G,G28280C,A28281T,T28282A,G28881A,G28882A,G2	20	M:A69S,N:D3L,N:R203K,N:G204R,N:S235F,ORF1a:T1001I,ORF1a:A1708D,ORF1a:I2230T,ORF1b:P314L,ORF1b:V2117L,ORF8:Q27*,ORF8:R52I,ORF8:Y73C,S:A570D,S:D614G,S:P681H,S:T716I,S:S982A,S:D1118H,S:G1219C	6	1247
hCoV-19/Mexico/MEX-INMEGEN-05-06-310/2021	EPI_ISL_2603772	-	B.1.1.7	GRY	34	C241T,C913T,C3037T,C3267T,A3492G,C4331T,C5388A,C5986T,T6954C,C14408T,C14676T,C15279T,T16176C,C18555T,C21639A,A23063T,C23271A,A23403G,C23604A,C23709T,C24374T,T24506G,G24914C,C27972T,G28048T,A28111G,G28280C,A28281T,T28282A,G28881A,G28882A,G2	21	N:D3L,N:R203K,N:G204R,N:S235F,ORF1a:T1001I,ORF1a:N1076S,ORF1a:A1708D,ORF1a:I2230T,ORF1b:P314L,ORF8:Q27*,ORF8:R52I,ORF8:Y73C,S:P26H,S:N501Y,S:A570D,S:D614G,S:P681H,S:T716I,S:L938F,S:S982A,S:D1118H	6	0
hCoV-19/Mexico/MEX-INMEGEN-05-06-336/2021	EPI_ISL_2603787	-	B.1.1.7	GRY	34	C241T,C913T,C3037T,C3267T,A3492G,C4331T,C5388A,C5986T,T6954C,C14408T,C14676T,C15279T,T16176C,C18555T,C21639A,A23063T,C23271A,A23403G,C23604A,C23709T,C24374T,T24506G,G24914C,C27972T,G28048T,A28111G,G28280C,A28281T,T28282A,G28881A,G28882A,G2	21	N:D3L,N:R203K,N:G204R,N:S235F,ORF1a:T1001I,ORF1a:N1076S,ORF1a:A1708D,ORF1a:I2230T,ORF1b:P314L,ORF8:Q27*,ORF8:R52I,ORF8:Y73C,S:P26H,S:N501Y,S:A570D,S:D614G,S:P681H,S:T716I,S:L938F,S:S982A,S:D1118H	6	0
hCoV-19/Mexico/MEX-INMEGEN-05-06-340/2021	EPI_ISL_2603791	-	B.1.1.7	GRY	35	C241T,C913T,C2453T,C3037T,C3267T,C3695T,C5388A,C5986T,T6954C,C9611T,C9693T,C14408T,C14676T,C15279T,T16176C,C21008T,C21855T,G21974C,A23063T,C23271A,A23403G,C23604A,C23709T,T24506G,G24914C,C27643T,C27972T,G28048T,A28111G,T28282A,A28295G,G28881A,G28882A,G28883C,C28977T	25	N:N8D,N:R203K,N:G204R,N:S235F,ORF1a:L730F,ORF1a:T1001I,ORF1a:A1708D,ORF1a:I2230T,ORF1a:L3116F,ORF1a:A3143V,ORF1b:P314L,ORF1b:T2514I,ORF7a:P84S,ORF8:Q27*,ORF8:R52I,ORF8:Y73C,S:S98F,S:D138H,S:N501Y,S:A570D,S:D614G,S:P681H,S:T716I,S:S982A,S:D1118H	6	3
hCoV-19/Mexico/MEX-INMEGEN-05-06-345/2021	EPI_ISL_2603796	-	B.1.1.7	GRY	42	C241T,C913T,G1264A,C2110T,C3037T,C3267T,C5388A,C5986T,T6954C,T7984C,T9508C,C10747T,A12964G,C14120T,C14408T,C14676T,C15279T,T16176C,C16323T,C16575T,C17004T,C17518T,C19390T,A23063T,C23271A,A23403G,C23604A,C23709T,T24506G,G24914C,C27972T,G28048T,A28095T,A28111G,C28146T,G28280C,A28281T,T28282A,G28881A,G28882A,G28883C,C2897	23	N:D3L,N:R203K,N:G204R,N:S235F,ORF1a:T1001I,ORF1a:A1708D,ORF1a:I2230T,ORF1b:P218L,ORF1b:P314L,ORF1b:L1351F,ORF1b:P1975S,ORF8:Q27*,ORF8:R52I,ORF8:K68*,ORF8:Y73C,ORF8:P85S,S:N501Y,S:A570D,S:D614G,S:P681H,S:T716I,S:S982A,S:D1118H	6	262
hCoV-19/Mexico/MEX-INMEGEN-05-06-356/2021	EPI_ISL_2603803	-	B.1.1.7	GRY	42	C241T,C913T,G1264A,C2110T,C3037T,C3267T,C5388A,C5986T,T6954C,T7984C,T9508C,C10747T,A12964G,C14120T,C14408T,C14676T,C15279T,T16176C,C16323T,C16575T,C17004T,C17518T,C19390T,A23063T,C23271A,A23403G,C23604A,C23709T,T24506G,G24914C,C27972T,G28048T,A28095T,A28111G,C28146T,G28280C,A28281T,T28282A,G28881A,G28882A,G28883C,C2897	23	N:D3L,N:R203K,N:G204R,N:S235F,ORF1a:T1001I,ORF1a:A1708D,ORF1a:I2230T,ORF1b:P218L,ORF1b:P314L,ORF1b:L1351F,ORF1b:P1975S,ORF8:Q27*,ORF8:R52I,ORF8:K68*,ORF8:Y73C,ORF8:P85S,S:N501Y,S:A570D,S:D614G,S:P681H,S:T716I,S:S982A,S:D1118H	6	16
hCoV-19/Mexico/MEX-INMEGEN-05-06-357/2021	EPI_ISL_2603804	-	B.1.1.7	G	38	C241T,C913T,T1177C,T1537G,C2110T,G2528A,C3037T,C3267T,C4296T,C5142T,C5388A,C5986T,T6954C,G7042T,C10789T,C14120T,C14408T,C14676T,C15279T,T16176C,C20470A,C23271A,A23403G,C23604A,C23709T,T24506G,G24914C,C25452T,G25471T,C27972T,G28048T,A28095T,A28111G,G28280C,A28281T,T28282A,C28977T,C29167T	23	N:D3L,N:S235F,ORF1a:E755K,ORF1a:T1001I,ORF1a:T1344I,ORF1a:T1626I,ORF1a:A1708D,ORF1a:I2230T,ORF1a:M2259I,ORF1b:P218L,ORF1b:P314L,ORF1b:Q2335K,ORF3a:D27Y,ORF8:Q27*,ORF8:R52I,ORF8:K68*,ORF8:Y73C,S:A570D,S:D614G,S:P681H,S:T716I,S:S982A,S:D1118H	6	937
hCoV-19/Mexico/MEX-INMEGEN-06-01-10/2021	EPI_ISL_2616828	-	B.1.1.7	GRY	31	C241T,C913T,C3037T,C3267T,C5388A,C5986T,C6196T,T6954C,A8848G,C14408T,C14676T,C15279T,T16176C,G20580T,A23063T,C23271A,A23403G,C23604A,C23709T,T24506G,G24914C,G25217T,G26727T,G28280C,A28281T,T28282A,G28881A,G28882A,G28883C,C28977T,G29715T	17	M:A69S,N:D3L,N:R203K,N:G204R,N:S235F,ORF1a:T1001I,ORF1a:A1708D,ORF1a:I2230T,ORF1b:P314L,S:N501Y,S:A570D,S:D614G,S:P681H,S:T716I,S:S982A,S:D1118H,S:G1219C	6	1058
hCoV-19/Mexico/MEX-INMEGEN-06-01-117/2021	EPI_ISL_2616849	-	B.1.1.7	GR	42	C241T,C913T,T1177C,T1537G,C2110T,G2528A,C3037T,C3267T,C4296T,C5142T,C5388A,C5986T,T6954C,G7042T,C10789T,C14120T,C14408T,C14676T,C15279T,T16176C,A19950G,C20470A,C23271A,A23403G,C23604A,C23709T,G23749A,T24506G,G24914C,C25452T,G25471T,C27972T,G28048T,A28111G,G28280C,A28281T,T28282A,G28881A,G28882A,G28883C,C28977T,C29167T	24	N:D3L,N:R203K,N:G204R,N:S235F,ORF1a:E755K,ORF1a:T1001I,ORF1a:T1344I,ORF1a:T1626I,ORF1a:A1708D,ORF1a:I2230T,ORF1a:M2259I,ORF1b:P218L,ORF1b:P314L,ORF1b:Q2335K,ORF3a:D27Y,ORF8:Q27*,ORF8:R52I,ORF8:Y73C,S:A570D,S:D614G,S:P681H,S:T716I,S:S982A,S:D1118H	6	882
hCoV-19/Mexico/MEX-INMEGEN-06-01-148/2021	EPI_ISL_2616949	-	B.1.1.7	G	40	C241T,C913T,C1625T,C3037T,C3267T,T4909C,C5388A,C5986T,T6954C,C7299T,T8971C,C11620T,A13768T,C14408T,C14676T,T15096C,C15279T,T16176C,C19862T,A23063T,C23271A,A23403G,C23604A,C23709T,T24506G,G24914C,G25354T,C26606T,G27281T,G27827T,C27972T,G28048T,A28111G,G28280C,A28281T,T28282A,A28877T,C28977T,A29491G,C29769T	22	N:D3L,N:S235F,ORF1a:L454F,ORF1a:T1001I,ORF1a:A1708D,ORF1a:I2230T,ORF1a:A2345V,ORF1b:M101L,ORF1b:P314L,ORF1b:A2132V,ORF6:W27L,ORF7b:M24I,ORF8:Q27*,ORF8:R52I,ORF8:Y73C,S:N501Y,S:A570D,S:D614G,S:P681H,S:T716I,S:S982A,S:D1118H	6	366
hCoV-19/Mexico/MEX-INMEGEN-06-01-154/2021	EPI_ISL_2616888	-	B.1.1.7	GR	37	C241T,C913T,C3037T,C3267T,C5388A,C5986T,T6954C,C7299T,C11074T,C11750T,C14408T,C14676T,T15096C,C15279T,T16176C,T21820C,C23271A,A23403G,C23604A,C23709T,T24506G,G24914C,G25489T,T26149C,C27972T,G28048T,G28077C,A28095T,A28111G,G28280C,A28281T,T28282A,C28807T,G28881A,G28882A,G28883C,C28977T	23	N:D3L,N:R203K,N:G204R,N:S235F,ORF1a:T1001I,ORF1a:A1708D,ORF1a:I2230T,ORF1a:A2345V,ORF1a:L3829F,ORF1b:P314L,ORF3a:A33S,ORF3a:S253P,ORF8:Q27*,ORF8:R52I,ORF8:V62L,ORF8:K68*,ORF8:Y73C,S:A570D,S:D614G,S:P681H,S:T716I,S:S982A,S:D1118H	6	680
hCoV-19/Mexico/MEX-INMEGEN-06-01-25/2021	EPI_ISL_2616824	-	B.1.1.7	GR	37	C203T,C241T,C913T,G1820A,C3037T,C3267T,C5388A,G5924A,C5986T,T6954C,G9526T,A12162G,C14408T,C14676T,C15279T,C15720T,T16176C,A17615G,A21137G,C23248T,C23271A,A23403G,C23604A,C23709T,T24506G,G24914C,G25135T,C27972T,G28048T,A28111G,G28280C,A28281T,T28282A,G28881A,G28882A,G28883C,C28977T	24	N:D3L,N:R203K,N:G204R,N:S235F,ORF1a:G519S,ORF1a:T1001I,ORF1a:A1708D,ORF1a:V1887I,ORF1a:I2230T,ORF1a:M3087I,ORF1a:Q3966R,ORF1b:P314L,ORF1b:K1383R,ORF1b:K2557R,ORF8:Q27*,ORF8:R52I,ORF8:Y73C,S:A570D,S:D614G,S:P681H,S:T716I,S:S982A,S:D1118H,S:K1191N	6	1066

hCoV-19/Mexico/MEX-INMEGEN-06-01-34/2021	EPI_ISL_2616978	-	B.1.1.7	GR	41	C241T,C913T,G1069A,C1609T,C3037T,T3049C,C3267T,C5388A,G5558A,C5986T,T6954C,A12162G,G14294A,C14408T,C14676T,C15279T,C15450T,C15720T,T16176C,A17615G,C18329T,A21137G,C22636T,C23271A,A23403G,C23604A,C23709T,T24506G,G24914C,G25135T,C27972T,G28048T,A28111G,G28280C,A28281T,T28282A,G28881A,G28882A,G28883C,G28916A,C28977T	25	N:D3L,N:R203K,N:G204R,N:G215S,N:S235F,ORF1a:T1001I,ORF1a:A1708D,ORF1a:V1765I,ORF1a:I2230T,ORF1a:Q3966R,ORF1b:R276H,ORF1b:P314L,ORF1b:K1383R,ORF1b:T1621I,ORF1b:K2557R,ORF8:Q27*,ORF8:R52I,ORF8:Y73C,S:A570D,S:D614G,S:P681H,S:T716I,S:S982A,S:D1118H,S:K1191N	6	2595
hCoV-19/Mexico/MEX-INMEGEN-06-01-77/2021	EPI_ISL_2616998	-	B.1.1.7	GR	41	C241T,C913T,G2281A,C3037T,A3061G,C3267T,C5388A,C5986T,T6954C,A12162G,C13665T,C14408T,C14676T,C15279T,C15720T,T16176C,A17615G,C18747T,C20844T,A21137G,C23271A,A23403G,C23604A,C23709T,T24506G,G24914C,G25135T,C26110T,C26521T,G26828T,C27972T,G28048T,A28111G,G28280C,A28281T,T28282A,G28881A,G28882A,G28883C,C28977T,C29095T	22	N:D3L,N:R203K,N:G204R,N:S235F,ORF1a:T1001I,ORF1a:A1708D,ORF1a:I2230T,ORF1a:Q3966R,ORF1b:P314L,ORF1b:K1383R,ORF1b:K2557R,ORF3a:P240S,ORF8:Q27*,ORF8:R52I,ORF8:Y73C,S:A570D,S:D614G,S:P681H,S:T716I,S:S982A,S:D1118H,S:K1191N	6	1663
hCoV-19/Mexico/MEX-INMEGEN-06-01-9/2021	EPI_ISL_2616850	-	B.1.1.7	G	26	C241T,C913T,C3037T,C3267T,C5388A,C5986T,C6402T,T6954C,A8848G,C14408T,C14676T,C15279T,T16176C,G20580T,C23271A,A23403G,C23604A,C23709T,T24506G,G24914C,G25217T,G26727T,C27874T,G28881T,C28977T,G29715T	16	M:A69S,N:R203M,N:S235F,ORF1a:T1001I,ORF1a:A1708D,ORF1a:P2046L,ORF1a:I2230T,ORF1b:P314L,ORF7b:T40I,S:A570D,S:D614G,S:P681H,S:T716I,S:S982A,S:D1118H,S:G1219C	8	882
hCoV-19/Mexico/MEX-INMEGEN-06-02-194/2021	EPI_ISL_2778956	-	B.1.1.7	GR	37	C241T,C913T,C2453T,C3037T,C3267T,A3481T,C5388A,C5986T,T6954C,C9611T,C10543T,C14408T,C14676T,C15279T,T16176C,C21008T,C21855T,G21974C,C23271A,A23403G,C23604A,C23709T,T24506G,G24914C,G25437T,C27643T,C27972T,G28048T,A28111G,G28280C,A28281T,T28282A,A28295G,G28881A,G28882A,G28883C,C28977T	25	N:D3L,N:N8D,N:R203K,N:G204R,N:S235F,ORF1a:L730F,ORF1a:T1001I,ORF1a:A1708D,ORF1a:I2230T,ORF1a:L3116F,ORF1b:P314L,ORF1b:T2514I,ORF3a:L15F,ORF7a:P84S,ORF8:Q27*,ORF8:R52I,ORF8:Y73C,S:S98F,S:D138H,S:A570D,S:D614G,S:P681H,S:T716I,S:S982A,S:D1118H	6	1653
hCoV-19/Mexico/MEX-INMEGEN-06-02-240/2021	EPI_ISL_2692750	-	B.1.1.7	GRY	40	C203T,C241T,C913T,G1820A,C3037T,C3267T,G5023T,C5388A,G5924A,C5986T,T6954C,G9526T,A12162G,C14408T,C14676T,C15279T,C15720T,C15738T,T16176C,A17615G,A21137G,A23063T,C23248T,C23271A,A23403G,C23604A,C23709T,T24506G,G24914C,G25135T,C27972T,G28048T,A28111G,G28280C,A28281T,T28282A,G28881A,G28882A,G28883C,C28977T	26	N:D3L,N:R203K,N:G204R,N:S235F,ORF1a:G519S,ORF1a:T1001I,ORF1a:M1586I,ORF1a:A1708D,ORF1a:V1887I,ORF1a:I2230T,ORF1a:M3087I,ORF1a:Q3966R,ORF1b:P314L,ORF1b:K1383R,ORF1b:K2557R,ORF8:Q27*,ORF8:R52I,ORF8:Y73C,S:N501Y,S:A570D,S:D614G,S:P681H,S:T716I,S:S982A,S:D1118H,S:K1191N	6	413
hCoV-19/Mexico/MEX-INMEGEN-06-02-293/2021	EPI_ISL_2692787	-	B.1.1.7	GR	39	A223T,C241T,C913T,C2110T,C3037T,C3267T,G4504A,C5388A,C5986T,C6706T,T6954C,T9127C,C11103T,C12809T,C14120T,C14408T,C14599T,C14676T,C15279T,T16176C,A20412G,C23271A,A23403G,C23604A,C23709T,T24506G,G24914C,C25797T,A26924G,C27972T,G28048T,A28111G,G28280C,A28281T,T28282A,G28881A,G28882A,G28883C,C28977T	20	N:D3L,N:R203K,N:G204R,N:S235F,ORF1a:T1001I,ORF1a:A1708D,ORF1a:I2230T,ORF1a:P3613L,ORF1a:L4182F,ORF1b:P218L,ORF1b:P314L,ORF8:Q27*,ORF8:R52I,ORF8:Y73C,S:A570D,S:D614G,S:P681H,S:T716I,S:S982A,S:D1118H	6	370
hCoV-19/Mexico/MEX-INMEGEN-06-03-129/2021	EPI_ISL_2810121	-	B.1.1.7	GRY	36	C241T,C913T,C3037T,C3267T,C5388A,C5986T,C6196T,C6285T,T6954C,A8848G,C14408T,C14676T,C15279T,T16176C,C16393T,A23063T,C23271A,A23403G,C23604A,C23709T,C24370T,T24506G,G24914C,G25217T,G26727T,C27972T,G28048T,A28111G,G28280C,A28281T,T28282A,G28881A,G28882A,G28883C,C28977T,G29715T	22	M:A69S,N:D3L,N:R203K,N:G204R,N:S235F,ORF1a:T1001I,ORF1a:A1708D,ORF1a:T2007I,ORF1a:I2230T,ORF1b:P314L,ORF1b:P976S,ORF8:Q27*,ORF8:R52I,ORF8:Y73C,S:N501Y,S:A570D,S:D614G,S:P681H,S:T716I,S:S982A,S:D1118H,S:G1219C	6	892
hCoV-19/Mexico/MEX-INMEGEN-06-03-135/2021	EPI_ISL_2810126	-	B.1.1.7	GRY	36	C241T,C913T,C1101T,C2788T,C3037T,C3267T,C5388A,C5986T,C6196T,T6954C,A8848G,C14408T,C14676T,C15279T,T16176C,C16877T,A23063T,C23271A,A23403G,C23604A,C23709T,T24506G,G24914C,G25217T,G26727T,C27972T,G28048T,A28111G,G28280C,A28281T,T28282A,G28881A,G28882A,G28883C,C28977T,G29715T	22	M:A69S,N:D3L,N:R203K,N:G204R,N:S235F,ORF1a:S279F,ORF1a:T1001I,ORF1a:A1708D,ORF1a:I2230T,ORF1b:P314L,ORF1b:T1137I,ORF8:Q27*,ORF8:R52I,ORF8:Y73C,S:N501Y,S:A570D,S:D614G,S:P681H,S:T716I,S:S982A,S:D1118H,S:G1219C	6	0
hCoV-19/Mexico/MEX-INMEGEN-06-03-136/2021	EPI_ISL_2810127	-	B.1.1.7	GRY	42	C241T,C913T,G2281A,C3037T,A3061G,C3267T,C5388A,C5986T,T6954C,A12162G,C13665T,C14408T,C14676T,C15279T,C15720T,T16176C,A17615G,C18747T,C20844T,A21137G,A23063T,C23271A,A23403G,C23604A,C23709T,T24506G,G24914C,G25135T,C26110T,C26521T,G26828T,C27972T,G28048T,A28111G,G28280C,A28281T,T28282A,G28881A,G28882A,G28883C,C28977T,C	23	N:D3L,N:R203K,N:G204R,N:S235F,ORF1a:T1001I,ORF1a:A1708D,ORF1a:I2230T,ORF1a:Q3966R,ORF1b:P314L,ORF1b:K1383R,ORF1b:K2557R,ORF3a:P240S,ORF8:Q27*,ORF8:R52I,ORF8:Y73C,S:N501Y,S:A570D,S:D614G,S:P681H,S:T716I,S:S982A,S:D1118H,S:K1191N	6	126
hCoV-19/Mexico/MEX-INMEGEN-06-03-159/2021	EPI_ISL_2810146	-	B.1.1.7	GRY	42	C241T,C913T,G2281A,C3037T,A3061G,C3267T,C5388A,C5986T,T6954C,A12162G,C13665T,C14408T,C14676T,C15279T,C15720T,T16176C,A17615G,C18747T,C20844T,A21137G,A23063T,C23271A,A23403G,C23604A,C23709T,T24506G,G24914C,G25135T,C26110T,C26521T,G26828T,C27972T,G28048T,A28111G,G28280C,A28281T,T28282A,G28881A,G28882A,G28883C,C28977T,C	23	N:D3L,N:R203K,N:G204R,N:S235F,ORF1a:T1001I,ORF1a:A1708D,ORF1a:I2230T,ORF1a:Q3966R,ORF1b:P314L,ORF1b:K1383R,ORF1b:K2557R,ORF3a:P240S,ORF8:Q27*,ORF8:R52I,ORF8:Y73C,S:N501Y,S:A570D,S:D614G,S:P681H,S:T716I,S:S982A,S:D1118H,S:K1191N	6	1258
hCoV-19/Mexico/MEX-INMEGEN-06-03-160/2021	EPI_ISL_2810147	-	B.1.1.7	GRY	37	C241T,C913T,C2110T,C3037T,C3267T,C5388A,C5986T,T6954C,G7042T,A10195G,C14120T,C14408T,C14676T,C15279T,C15933T,T16176C,C22450T,A23063T,C23271A,A23403G,C23604A,C23709T,T24506G,G24914C,A27745G,C27972T,G28048T,G28075T,A28095T,A28111G,G28280C,A28281T,T28282A,G28881A,G28882A,G28883C,C28977T	23	N:D3L,N:R203K,N:G204R,N:S235F,ORF1a:T1001I,ORF1a:A1708D,ORF1a:I2230T,ORF1a:M2259I,ORF1b:P218L,ORF1b:P314L,ORF7a:R118G,ORF8:Q27*,ORF8:R52I,ORF8:C61F,ORF8:K68*,ORF8:Y73C,S:N501Y,S:A570D,S:D614G,S:P681H,S:T716I,S:S982A,S:D1118H	6	16
hCoV-19/Mexico/MEX-INMEGEN-06-03-185/2021	EPI_ISL_2810167	-	B.1.1.7	GRY	40	C241T,C913T,C2453T,C3037T,C3093T,C3267T,T4159C,C5388A,C5986T,T6954C,G8653T,C9611T,C9907T,C14408T,C14676T,C15279T,T16176C,C21077T,C21855T,G21974C,A23063T,C23271A,A23403G,C23604A,C23709T,T24506G,G24914C,C25609T,G27758T,C27972T,G28048T,A28111G,G28280C,A28281T,T28282A,A28295G,G28881A,G28882A,G28883C,C28977T	29	N:D3L,N:N8D,N:R203K,N:G204R,N:S235F,ORF1a:L730F,ORF1a:P943L,ORF1a:T1001I,ORF1a:A1708D,ORF1a:I2230T,ORF1a:M2796I,ORF1a:L3116F,ORF1b:P314L,ORF1b:T2537I,ORF3a:L73F,ORF7a:*122L,ORF7b:M1I,ORF8:Q27*,ORF8:R52I,ORF8:Y73C,S:S98F,S:D138H,S:N501Y,S:A570D,S:D614G,S:P681H,S:T716I,S:S	6	774
hCoV-19/Mexico/MEX-INMEGEN-06-03-193/2021	EPI_ISL_2810174	-	B.1.1.7	GR	39	G101T,C241T,C913T,C2143T,C3037T,C3267T,C5388A,C5986T,T6954C,C10228T,C14408T,C14676T,T15096C,C15279T,T16176C,C23271A,A23403G,C23604A,C23709T,T24506G,G24914C,G25785T,C27476T,C27494T,G27948T,C27972T,G28048T,A28095T,A28111G,G28280C,A28281T,T28282A,A28466G,G28881A,G28882A,G28883C,C28977T,C29167T,C29642T	23	N:D3L,N:K65E,N:R203K,N:G204R,N:S235F,ORF1a:T1001I,ORF1a:A1708D,ORF1a:I2230T,ORF1b:P314L,ORF3a:W131C,ORF7a:T28I,ORF7a:P34L,ORF8:E19*,ORF8:Q27*,ORF8:R52I,ORF8:K68*,ORF8:Y73C,S:A570D,S:D614G,S:P681H,S:T716I,S:S982A,S:D1118H	6	1796
hCoV-19/Mexico/MEX-INMEGEN-06-03-62/2021	EPI_ISL_2810072	-	B.1.1.7	GRY	37	C241T,C913T,C2110T,C3037T,C3267T,C5388A,C5986T,T6954C,G7042T,A10195G,C14120T,C14408T,C14676T,C15279T,C15933T,T16176C,C22450T,A23063T,C23271A,A23403G,C23604A,C23709T,T24506G,G24914C,A27745G,C27972T,G28048T,G28075T,A28095T,A28111G,G28280C,A28281T,T28282A,G28881A,G28882A,G28883C,C28977T	23	N:D3L,N:R203K,N:G204R,N:S235F,ORF1a:T1001I,ORF1a:A1708D,ORF1a:I2230T,ORF1a:M2259I,ORF1b:P218L,ORF1b:P314L,ORF7a:R118G,ORF8:Q27*,ORF8:R52I,ORF8:C61F,ORF8:K68*,ORF8:Y73C,S:N501Y,S:A570D,S:D614G,S:P681H,S:T716I,S:S982A,S:D1118H	6	2
hCoV-19/Mexico/MEX-INMEGEN-06-03-69/2021	EPI_ISL_2810078	-	B.1.1.7	GRY	32	C241T,C913T,C1912T,C3037T,C3177T,C3267T,C5388A,T5479A,C5986T,T6954C,G7067A,C10252T,C14408T,C14676T,C15279T,T16176C,A23063T,C23271A,A23403G,C23604A,C23709T,T24506G,G24914C,G25217T,C27879T,C27972T,G28048T,A28111G,G28881A,G28882A,G28883C,C28977T	22	N:R203K,N:G204R,N:S235F,ORF1a:P971L,ORF1a:T1001I,ORF1a:A1708D,ORF1a:H1738Q,ORF1a:I2230T,ORF1a:E2268K,ORF1b:P314L,ORF7b:H42Y,ORF8:Q27*,ORF8:R52I,ORF8:Y73C,S:N501Y,S:A570D,S:D614G,S:P681H,S:T716I,S:S982A,S:D1118H,S:G1219C	6	21
hCoV-19/Mexico/MEX-INMEGEN-06-04-375/2021	EPI_ISL_2894414	-	B.1.1.7	GRY	36	C241T,C913T,C2110T,C3037T,C3267T,C5388A,C5986T,T6954C,G7042T,A10195G,C14120T,C14408T,C14676T,C15279T,C15933T,T16176C,A23063T,C23271A,A23403G,C23604A,C23709T,T24506G,G24914C,A27745G,C27972T,G28048T,G28075T,A28095T,A28111G,G28280C,A28281T,T28282A,G28881A,G28882A,G28883C,C28977T	23	N:D3L,N:R203K,N:G204R,N:S235F,ORF1a:T1001I,ORF1a:A1708D,ORF1a:I2230T,ORF1a:M2259I,ORF1b:P218L,ORF1b:P314L,ORF7a:R118G,ORF8:Q27*,ORF8:R52I,ORF8:C61F,ORF8:K68*,ORF8:Y73C,S:N501Y,S:A570D,S:D614G,S:P681H,S:T716I,S:S982A,S:D1118H	6	170
hCoV-19/Mexico/MEX-INMEGEN-16-260/2021	EPI_ISL_2978574	-	B.1.1.7	GRY	43	C241T,C913T,C3037T,C3267T,C5388A,C5944T,C5986T,G6647A,C6936T,T6954C,C14408T,C14621T,C14676T,T15096C,C15279T,T16176C,C17676T,C18183T,C19524T,A21519C,A23063T,C23271A,A23403G,C23604A,C23709T,A24070T,A24092G,T24506G,G24914C,C25624T,C27737T,C27972T,G28048T,A28095T,A28111G,G28280C,A28281T,T28282A,G28881A,G28882A,G28883C,G28	27	N:D3L,N:R203K,N:G204P,N:S235F,ORF1a:T1001I,ORF1a:A1708D,ORF1a:A2128T,ORF1a:S2224F,ORF1a:I2230T,ORF1b:P314L,ORF1b:T385M,ORF1b:R2684S,ORF3a:H78Y,ORF7a:T115I,ORF8:Q27*,ORF8:R52I,ORF8:K68*,ORF8:Y73C,S:N501Y,S:A570D,S:D614G,S:P681H,S:T716I,S:Q836H,S:I844V,S:S982A,S:D1118H	6	48
hCoV-19/Mexico/MEX-INMEGEN-16-262/2021	EPI_ISL_2978576	-	B.1.1.7	GRY	43	C241T,C913T,C3037T,C3267T,G4184A,C5388A,C5944T,C5986T,G6647A,C6936T,T6954C,C14408T,C14621T,C14676T,T15096C,C15279T,T16176C,C17676T,C18183T,A21519C,A23063T,C23271A,A23403G,C23604A,C23709T,A24070T,A24092G,T24506G,G24914C,C25624T,C27737T,C27972T,G28048T,A28095T,A28111G,G28280C,A28281T,T28282A,G28881A,G28882A,G28883C,G288	28	N:D3L,N:R203K,N:G204P,N:S235F,ORF1a:T1001I,ORF1a:G1307S,ORF1a:A1708D,ORF1a:A2128T,ORF1a:S2224F,ORF1a:I2230T,ORF1b:P314L,ORF1b:T385M,ORF1b:R2684S,ORF3a:H78Y,ORF7a:T115I,ORF8:Q27*,ORF8:R52I,ORF8:K68*,ORF8:Y73C,S:N501Y,S:A570D,S:D614G,S:P681H,S:T716I,S:Q836H,S:I844V,S:S98	6	208
hCoV-19/Mexico/MEX-INMEGEN-16-263/2021	EPI_ISL_2978577	-	B.1.1.7	GRY	39	C241T,C913T,C2110T,C3037T,A3075T,C3267T,C5388A,C5986T,T6954C,G7042T,A10195G,C14120T,C14408T,C14676T,C15279T,C15933T,T16176C,C18814T,C22450T,A23063T,C23271A,A23403G,C23604A,C23709T,T24506G,G24914C,A27745G,C27972T,G28048T,G28075T,A28095T,A28111G,G28280C,A28281T,T28282A,G28881A,G28882A,G28883C,C28977T	24	N:D3L,N:R203K,N:G204R,N:S235F,ORF1a:E937V,ORF1a:T1001I,ORF1a:A1708D,ORF1a:I2230T,ORF1a:M2259I,ORF1b:P218L,ORF1b:P314L,ORF7a:R118G,ORF8:Q27*,ORF8:R52I,ORF8:C61F,ORF8:K68*,ORF8:Y73C,S:N501Y,S:A570D,S:D614G,S:P681H,S:T716I,S:S982A,S:D1118H	6	205

hCoV-19/Mexico/MEX-INMEGEN-16-99/2021	EPI_ISL_2978445	-	B.1.1.7	GRY	41	G101T,C241T,C913T,C2143T,C2388T,C3037T,C3267T,C5388A,C5986T,T6954C,C10228T,C14408T,C14676T,T15096C,C15279T,T16176C,C22414T,C22995T,A23063T,C23271A,A23403G,C23604A,C23709T,G24089A,T24506G,G24914C,G25785T,C27476T,C27972T,G28048T,A28095T,A28111G,G28280C,A28281T,T28282A,A28466G,G28881A,G28882A,G28883C,C28977T,C29642T	25	N:D3L,N:K65E,N:R203K,N:G204R,N:S235F,ORF1a:T708I,ORF1a:T1001I,ORF1a:A1708D,ORF1a:I2230T,ORF1b:P314L,ORF3a:W131C,ORF7a:T28I,ORF8:Q27*,ORF8:R52I,ORF8:K68*,ORF8:Y73C,S:T478I,S:N501Y,S:A570D,S:D614G,S:P681H,S:T716I,S:D843N,S:S982A,S:D1118H	6	0
hCoV-19/Mexico/MIC-InDRE_FB14989_S2053/2021	EPI_ISL_2283695	-	B.1.1.7	GRY	33	C241T,C913T,C2453T,C3037T,C3267T,C5388A,C5986T,T6954C,C9611T,C14408T,C14676T,C15279T,T16176C,C21855T,G21974C,A23063T,C23271A,A23403G,C23604A,C23709T,T24506G,G24914C,C27972T,G28048T,A28111G,G28280C,A28281T,T28282A,A28295G,G28881A,G28882A,G	23	N:D3L,N:N8D,N:R203K,N:G204R,N:S235F,ORF1a:L730F,ORF1a:T1001I,ORF1a:A1708D,ORF1a:I2230T,ORF1a:L3116F,ORF1b:P314L,ORF8:Q27*,ORF8:R52I,ORF8:Y73C,S:S98F,S:D138H,S:N501Y,S:A570D,S:D614G,S:P681H,S:T716I,S:S982	6	158
hCoV-19/Mexico/MIC-InDRE_FB14998_S2054/2021	EPI_ISL_2283696	-	B.1.1.7	GRY	33	C241T,C913T,C2453T,C3037T,C3267T,C5388A,C5986T,T6954C,C9611T,C14408T,C14676T,C15279T,T16176C,C21855T,G21974C,A23063T,C23271A,A23403G,C23604A,C23709T,T24506G,G24914C,C27972T,G28048T,A28111G,G28280C,A28281T,T28282A,A28295G,G28881A,G28882A,G	23	N:D3L,N:N8D,N:R203K,N:G204R,N:S235F,ORF1a:L730F,ORF1a:T1001I,ORF1a:A1708D,ORF1a:I2230T,ORF1a:L3116F,ORF1b:P314L,ORF8:Q27*,ORF8:R52I,ORF8:Y73C,S:S98F,S:D138H,S:N501Y,S:A570D,S:D614G,S:P681H,S:T716I,S:S982	6	0
hCoV-19/Mexico/MIC-InDRE_FB18021_S3946/2021	EPI_ISL_2858960	-	B.1.1.7	GRY	33	C241T,C913T,C2110T,C3037T,C3267T,C5388A,C5986T,T6954C,G7745A,C14120T,C14408T,C14676T,C15279T,T16176C,C21855T,A23063T,C23271A,A23403G,C23604A,C23709T,T24506G,G24914C,A25336C,C27972T,G28048T,A28111G,G28280C,A28281T,T28282A,G28881A,G28882A,G2	22	N:D3L,N:R203K,N:G204R,N:S235F,ORF1a:T1001I,ORF1a:A1708D,ORF1a:I2230T,ORF1a:V2494I,ORF1b:P218L,ORF1b:P314L,ORF8:Q27*,ORF8:R52I,ORF8:Y73C,S:S98F,S:N501Y,S:A570D,S:D614G,S:P681H,S:T716I,S:S982A,S:D1118H,S:E12	6	138
hCoV-19/Mexico/MOR-InDRE_FB13639_S1731/2021	EPI_ISL_2101870	-	B.1.1.7	GRY	36	C241T,C912T,C913T,C3037T,C3267T,C5388A,C5986T,T6954C,C7732T,C14408T,C14676T,C15279T,T16176C,A17799G,C18568T,A23063T,C23271A,A23403G,A23541T,C23604A,C23709T,T24506G,G24914C,A25336C,C27972T,G28048T,A28111G,G28198T,G28280C,A28281T,T28282A,C28728T,G28881A,G28882A,G28883C,C28977T	24	N:D3L,N:A152V,N:R203K,N:G204R,N:S235F,ORF1a:S216F,ORF1a:T1001I,ORF1a:A1708D,ORF1a:I2230T,ORF1b:P314L,ORF1b:L1701F,ORF8:Q27*,ORF8:R52I,ORF8:Y73C,ORF8:C102F,S:N501Y,S:A570D,S:D614G,S:Y660F,S:P681H,S:T716I,S:S982A,S:D1118H,S:E1258D	6	54
hCoV-19/Mexico/MOR-InDRE_FB16889_S3115/2021	EPI_ISL_2663382	-	B.1.1.7	GRY	35	C241T,C913T,C2453T,C3037T,C3267T,C5388A,A5570G,C5986T,T6954C,C9611T,C14408T,C14676T,C15279T,T16176C,C21855T,G21974C,A23063T,C23271A,A23403G,C23604A,C23709T,T24506G,G24914C,G25273T,C27972T,G28048T,A28111G,G28280C,A28281T,T28282A,A28295G,G28881A,G28882A,G28883C,C28977T	25	N:D3L,N:N8D,N:R203K,N:G204R,N:S235F,ORF1a:L730F,ORF1a:T1001I,ORF1a:A1708D,ORF1a:M1769V,ORF1a:I2230T,ORF1a:L3116F,ORF1b:P314L,ORF8:Q27*,ORF8:R52I,ORF8:Y73C,S:S98F,S:D138H,S:N501Y,S:A570D,S:D614G,S:P681H,S:T716I,S:S982A,S:D1118H,S:M1237I	6	0
hCoV-19/Mexico/NAY-InDRE_FB17385_S3480/2021	EPI_ISL_2778995	-	B.1.1.7	GRY	35	C241T,C245T,C913T,C3037T,C3267T,A3492G,C5388A,C5986T,T6954C,C14408T,C14676T,C15279T,T16176C,G20580T,C21639A,A23063T,C23271A,A23403G,C23604A,C23709T,C24374T,T24506G,G24914C,A25336C,G25337C,C27972T,G28048T,A28111G,G28280C,A28281T,T28282A,G28881A,G28882A,G28883C,C28977T	23	N:D3L,N:R203K,N:G204R,N:S235F,ORF1a:T1001I,ORF1a:N1076S,ORF1a:A1708D,ORF1a:I2230T,ORF1b:P314L,ORF8:Q27*,ORF8:R52I,ORF8:Y73C,S:P26H,S:N501Y,S:A570D,S:D614G,S:P681H,S:T716I,S:L938F,S:S982A,S:D1118H,S:E1258D,S:D1259H	6	0
hCoV-19/Mexico/NLE-InDRE_237/2021	EPI_ISL_961467	-	B.1.1.7	GRY	32	C241T,C913T,C3037T,C3267T,C5388A,C5986T,T6954C,C14408T,C14676T,C15279T,T16176C,A17615G,C18032T,A23063T,C23271A,A23403G,C23604A,C23709T,T24506G,G24914C,G26730C,C27972T,G28048T,A28111G,G28280C,A28281T,T28282A,C28603T,G28881A,G28882A,G28883C,	21	M:V70L,N:D3L,N:R203K,N:G204R,N:S235F,ORF1a:T1001I,ORF1a:A1708D,ORF1a:I2230T,ORF1b:P314L,ORF1b:K1383R,ORF1b:T1522I,ORF8:Q27*,ORF8:R52I,ORF8:Y73C,S:N501Y,S:A570D,S:D614G,S:P681H,S:T716I,S:S982A,S:D1118H	6	0
hCoV-19/Mexico/NLE-InDRE_238/2021	EPI_ISL_961468	-	B.1.1.7	GRY	32	C241T,C913T,C3037T,C3267T,C5388A,C5986T,T6954C,C14408T,C14676T,C15279T,T16176C,A17615G,C18032T,A23063T,C23271A,A23403G,C23604A,C23709T,T24506G,G24914C,G26730C,C27972T,G28048T,A28111G,G28280C,A28281T,T28282A,C28603T,G28881A,G28882A,G28883C,	21	M:V70L,N:D3L,N:R203K,N:G204R,N:S235F,ORF1a:T1001I,ORF1a:A1708D,ORF1a:I2230T,ORF1b:P314L,ORF1b:K1383R,ORF1b:T1522I,ORF8:Q27*,ORF8:R52I,ORF8:Y73C,S:N501Y,S:A570D,S:D614G,S:P681H,S:T716I,S:S982A,S:D1118H	6	0
hCoV-19/Mexico/NLE-InDRE_240/2021	EPI_ISL_961469	-	B.1.1.7	GRY	32	C241T,C913T,C3037T,C3267T,C5388A,C5986T,T6954C,C14408T,C14676T,C15279T,T16176C,A17615G,C18032T,A23063T,C23271A,A23403G,C23604A,C23709T,T24506G,G24914C,G26730C,C27972T,G28048T,A28111G,G28280C,A28281T,T28282A,C28603T,G28881A,G28882A,G28883C,	21	M:V70L,N:D3L,N:R203K,N:G204R,N:S235F,ORF1a:T1001I,ORF1a:A1708D,ORF1a:I2230T,ORF1b:P314L,ORF1b:K1383R,ORF1b:T1522I,ORF8:Q27*,ORF8:R52I,ORF8:Y73C,S:N501Y,S:A570D,S:D614G,S:P681H,S:T716I,S:S982A,S:D1118H	6	0
hCoV-19/Mexico/NLE-InDRE_F11681_S1199/2021	EPI_ISL_1626806	-	B.1.1.7	GRY	36	C241T,C913T,C2110T,C3037T,C3267T,C5388A,C5986T,G6166A,T6954C,T7984C,C14120T,C14408T,C14676T,C15279T,T16176C,C19390T,G22218A,A23063T,C23271A,A23403G,C23604A,C23709T,T24506G,G24914C,A25336C,G25337C,C27972T,G28048T,A28111G,G28280C,A28281T,T28282A,G28881A,G28882A,G28883C,C28977T	23	N:D3L,N:R203K,N:G204R,N:S235F,ORF1a:T1001I,ORF1a:A1708D,ORF1a:I2230T,ORF1b:P218L,ORF1b:P314L,ORF1b:P1975S,ORF8:Q27*,ORF8:R52I,ORF8:Y73C,S:G219D,S:N501Y,S:A570D,S:D614G,S:P681H,S:T716I,S:S982A,S:D1118H,S:E1258D,S:D1259H	6	59
hCoV-19/Mexico/NLE-InDRE_FB13119_S1506/2021	EPI_ISL_1805484	-	B.1.1.7	GRY	38	C241T,C292T,C913T,C1060T,C3037T,C3267T,C5388A,C5986T,T6954C,C14408T,C14676T,C15279T,T16176C,T16563A,A23063T,C23170T,C23271A,A23403G,C23604A,C23709T,T24506G,G24914C,A25336C,G25337C,C25413T,C25626T,C27972T,G28048T,A28111G,G28280C,A28281T,T28282A,A28295C,C28728T,G28881A,G28882A,G28883C,C28977T	23	N:D3L,N:N8H,N:A152V,N:R203K,N:G204R,N:S235F,ORF1a:T1001I,ORF1a:A1708D,ORF1a:I2230T,ORF1b:P314L,ORF8:Q27*,ORF8:R52I,ORF8:Y73C,ORF9b:K4N,S:N501Y,S:A570D,S:D614G,S:P681H,S:T716I,S:S982A,S:D1118H,S:E1258D,S:D1259H	6	0
hCoV-19/Mexico/NLE-InDRE_FB13201_S1495/2021	EPI_ISL_1805473	-	B.1.1.7	GRY	39	T78G,C241T,C913T,G1542A,C2110T,C3037T,C3267T,C5388A,C5986T,T6954C,G7042T,A10195G,C14120T,C14408T,C14676T,C15279T,T16176C,A23063T,C23271A,A23403G,C23604A,C23709T,C24130T,T24506G,G24914C,A25336C,G25337C,C27972T,G28048T,G28075T,A28111G,G28280C,A28281T,T28282A,G28881A,G28882A,G28883C,C28977T,G28985T	25	N:D3L,N:R203K,N:G204R,N:S235F,N:G238C,ORF1a:R426H,ORF1a:T1001I,ORF1a:A1708D,ORF1a:I2230T,ORF1a:M2259I,ORF1b:P218L,ORF1b:P314L,ORF8:Q27*,ORF8:R52I,ORF8:C61F,ORF8:Y73C,S:N501Y,S:A570D,S:D614G,S:P681H,S:T716I,S:S982A,S:D1118H,S:E1258D,S:D1259H	6	25
hCoV-19/Mexico/NLE-InDRE_FB14845_S2084/2021	EPI_ISL_2283723	-	B.1.1.7	GRY	35	C241T,C913T,C2110T,C3037T,C3267T,G4288T,C5388A,C5986T,T6954C,C14120T,C14408T,C14676T,C15279T,T16176C,C16887T,T17310C,A23063T,C23271A,A23403G,C23604A,C23709T,T24506G,G24914C,A25315G,C27972T,G28048T,A28111G,G28280C,A28281T,T28282A,G28881A,G28882A,G28883C,C28977T,C29769T	20	N:D3L,N:R203K,N:G204R,N:S235F,ORF1a:T1001I,ORF1a:E1341D,ORF1a:A1708D,ORF1a:I2230T,ORF1b:P218L,ORF1b:P314L,ORF8:Q27*,ORF8:R52I,ORF8:Y73C,S:N501Y,S:A570D,S:D614G,S:P681H,S:T716I,S:S982A,S:D1118H	6	0
hCoV-19/Mexico/NLE-InDRE_FB14848_S2085/2021	EPI_ISL_2283724	-	B.1.1.7	GRY	37	C241T,C875T,C913T,C2453T,C3037T,C3267T,C5388A,C5986T,C6026T,T6954C,T9445C,C9611T,C14408T,C14676T,C15279T,T16176C,C21855T,G21974C,A23063T,C23271A,A23403G,C23604A,C23709T,T24506G,G24914C,A25336C,C27972T,G28048T,A28111G,G28280C,A28281T,T28282A,A28295G,G28881A,G28882A,G28883C,C28977T	26	N:D3L,N:N8D,N:R203K,N:G204R,N:S235F,ORF1a:L204F,ORF1a:L730F,ORF1a:T1001I,ORF1a:A1708D,ORF1a:P1921S,ORF1a:I2230T,ORF1a:L3116F,ORF1b:P314L,ORF8:Q27*,ORF8:R52I,ORF8:Y73C,S:S98F,S:D138H,S:N501Y,S:A570D,S:D614G,S:P681H,S:T716I,S:S982A,S:D1118H,S:E1258D	6	0
hCoV-19/Mexico/NLE-InDRE_FB16177_S2461/2021	EPI_ISL_2455982	-	B.1.1.7	GRY	42	C222T,C241T,C913T,C2110T,C3037T,C3267T,C3743T,C5388A,C5986T,G6816A,T6954C,G7042T,C14120T,C14408T,C14676T,A15046G,C15279T,A15702T,T16176C,C17762T,C21846T,A23063T,C23271A,A23403G,C23604A,C23709T,T24506G,G24914C,T25180C,G25311T,A25336C,C27741T,C27972T,G28048T,A28111G,G28280C,A28281T,T28282A,G28881A,G28882A,G28883C,C28977	27	N:D3L,N:R203K,N:G204R,N:S235F,ORF1a:T1001I,ORF1a:H1160Y,ORF1a:A1708D,ORF1a:R2184K,ORF1a:I2230T,ORF1a:M2259I,ORF1b:P218L,ORF1b:P314L,ORF1b:I527V,ORF1b:A1432V,ORF8:Q27*,ORF8:R52I,ORF8:Y73C,S:T95I,S:N501Y,S:A570D,S:D614G,S:P681H,S:T716I,S:S982A,S:D1118H,S:C1250F,S:E1258D	6	0
hCoV-19/Mexico/NLE-InDRE_FB16180_S2462/2021	EPI_ISL_2455983	-	B.1.1.7	GRY	35	C241T,C913T,C3037T,C3267T,C5388A,C5986T,T6954C,C9967T,C13585T,C14408T,C14676T,T15096C,C15279T,T16176C,G21901A,C22987T,A23063T,C23271A,A23403G,C23604A,C23709T,T24506G,G24914C,A25336C,C27972T,G28048T,A28111G,G28280C,A28281T,T28282A,G28881A,G28882A,G28883C,G28884C,C28977T	19	N:D3L,N:R203K,N:G204P,N:S235F,ORF1a:T1001I,ORF1a:A1708D,ORF1a:I2230T,ORF1b:P314L,ORF8:Q27*,ORF8:R52I,ORF8:Y73C,S:N501Y,S:A570D,S:D614G,S:P681H,S:T716I,S:S982A,S:D1118H,S:E1258D	6	0
hCoV-19/Mexico/NLE-InDRE_FB16187_S2463/2021	EPI_ISL_2455984	-	Q.3	GRY	37	G149T,C241T,C913T,C3037T,C3267T,G5023A,C5388A,C5986T,T6954C,G10396T,C14408T,C14676T,C15279T,T16176C,A17615G,G20962A,C21108T,A23063T,C23271A,A23403G,C23604A,C23709T,T24506G,G24914C,A25336C,G26730C,C27972T,G28048T,A28111G,G28280C,A28281T,T28282A,G28881A,G28882A,G28883C,C28977T,G29440T	24	M:V70L,N:D3L,N:R203K,N:G204R,N:S235F,N:Q389H,ORF1a:T1001I,ORF1a:M1586I,ORF1a:A1708D,ORF1a:I2230T,ORF1b:P314L,ORF1b:K1383R,ORF1b:D2499N,ORF8:Q27*,ORF8:R52I,ORF8:Y73C,S:N501Y,S:A570D,S:D614G,S:P681H,S:T716I,S:S982A,S:D1118H,S:E1258D	6	0
hCoV-19/Mexico/NLE-InDRE_FB16191_S2464/2021	EPI_ISL_2455985	-	B.1.1.7	GRY	37	C241T,C913T,C2110T,C3037T,C3267T,C5388A,C5986T,T6954C,G7042T,A10195G,C12049A,C14120T,C14408T,C14676T,C15279T,T16176C,C21110T,A23063T,C23271A,A23403G,C23604A,C23709T,T24506G,G24914C,A25336C,G26062T,C27972T,G28048T,G28075T,A28111G,G28280C,A28281T,T28282A,G28881A,G28882A,G28883C,C28977T	25	N:D3L,N:R203K,N:G204R,N:S235F,ORF1a:T1001I,ORF1a:A1708D,ORF1a:I2230T,ORF1a:M2259I,ORF1a:N3928K,ORF1b:P218L,ORF1b:P314L,ORF1b:T2548I,ORF3a:G224C,ORF8:Q27*,ORF8:R52I,ORF8:C61F,ORF8:Y73C,S:N501Y,S:A570D,S:D614G,S:P681H,S:T716I,S:S982A,S:D1118H,S:E1258D	6	8
hCoV-19/Mexico/NLE-InDRE_FB16492_S3060/2021	EPI_ISL_2663327	-	Q.3	GRY	41	T78G,G149T,C241T,C913T,C3037T,C3267T,G5023A,C5388A,C5986T,T6954C,A10323G,G10396T,C14408T,C14676T,C15279T,A15581G,T16176C,A17615G,G20962A,C21108T,A23063T,C23271A,A23403G,C23604A,C23709T,T24506G,G24914C,A25336C,G25337C,G26730C,C27972T,G28048T,A28111G,G28280C,A28281T,T28282A,G28881A,G28882A,G28883C,C28977T,G29440T	27	M:V70L,N:D3L,N:R203K,N:G204R,N:S235F,N:Q389H,ORF1a:T1001I,ORF1a:M1586I,ORF1a:A1708D,ORF1a:I2230T,ORF1a:K3353R,ORF1b:P314L,ORF1b:K705R,ORF1b:K1383R,ORF1b:D2499N,ORF8:Q27*,ORF8:R52I,ORF8:Y73C,S:N501Y,S:A570D,S:D614G,S:P681H,S:T716I,S:S982A,S:D1118H,S:E1258D,S:D1259H	6	0

hCoV-19/Mexico/NLE-InDRE_FB16950_S3122/2021	EPI_ISL_2663389	-	B.1.1.7	GRY	38	C241T,C913T,C2453T,C3037T,C3267T,C5388A,C5986T,T6954C,C6990T,G8179T,C9611T,C9808T,C14408T,C14676T,C15279T,T16176C,C21855T,G21974C,A23063T,C23271A,A23403G,C23604A,C23709T,T24506G,G24914C,C25886T,A26528G,C27972T,G28048T,A28111G,G28280C,A28281T,T28282A,A28295G,G28881A,G28882A,G28883C,C28977T	25	N:D3L,N:N8D,N:R203K,N:G204R,N:S235F,ORF1a:L730F,ORF1a:T1001I,ORF1a:A1708D,ORF1a:I2230T,ORF1a:S2242F,ORF1a:L3116F,ORF1b:P314L,ORF3a:S165F,ORF8:Q27*,ORF8:R52I,ORF8:Y73C,S:S98F,S:D138H,S:N501Y,S:A570D,S:D614G,S:P681H,S:T716I,S:S982A,S:D1118H	6	0
hCoV-19/Mexico/NLE-InDRE_FB16953_S3123/2021	EPI_ISL_2663390	-	B.1.1.7	GRY	39	C241T,C913T,C1513T,C2110T,C3037T,C3267T,C5388A,C5986T,C6500T,T6954C,G7042T,C7834T,A10195G,C14120T,C14408T,C14676T,C15279T,T16176C,C16833T,C22347T,A23063T,C23271A,A23403G,C23604A,C23709T,T24506G,G24914C,C27972T,G28048T,G28075T,A28095T,A28111G,G28280C,A28281T,T28282A,G28881A,G28882A,G28883C,C28977T	24	N:D3L,N:R203K,N:G204R,N:S235F,ORF1a:T1001I,ORF1a:A1708D,ORF1a:P2079S,ORF1a:I2230T,ORF1a:M2259I,ORF1b:P218L,ORF1b:P314L,ORF8:Q27*,ORF8:R52I,ORF8:C61F,ORF8:K68*,ORF8:Y73C,S:A262V,S:N501Y,S:A570D,S:D614G,S:P681H,S:T716I,S:S982A,S:D1118H	6	83
hCoV-19/Mexico/NLE-InDRE_FB16961_S3124/2021	EPI_ISL_2663391	-	B.1.1.7	GRY	40	G204T,C241T,C913T,C2110T,C3037T,C3267T,G4904T,C5388A,C5986T,A6324T,T6954C,G7042T,A10195G,C10977T,C14120T,C14408T,C14676T,C15279T,C15933T,T16176C,A23063T,C23271A,A23403G,C23604A,C23709T,T24506G,G24914C,A27745G,C27972T,G28048T,G28075T,A28095T,A28111G,G28280C,A28281T,T28282A,G28881A,G28882A,G28883C,C28977T	26	N:D3L,N:R203K,N:G204R,N:S235F,ORF1a:T1001I,ORF1a:D1547Y,ORF1a:A1708D,ORF1a:E2020V,ORF1a:I2230T,ORF1a:M2259I,ORF1a:A3571V,ORF1b:P218L,ORF1b:P314L,ORF7a:R118G,ORF8:Q27*,ORF8:R52I,ORF8:C61F,ORF8:K68*,ORF8:Y73C,S:N501Y,S:A570D,S:D614G,S:P681H,S:T716I,S:S982A,S:D1118H	6	0
hCoV-19/Mexico/NLE-InDRE_FB16964_S3125/2021	EPI_ISL_2663392	-	B.1.1.7	GRY	41	C241T,C913T,C2110T,C3037T,C3177T,C3267T,C4890T,C5388A,C5986T,T6954C,G7042T,G9929A,A10195G,C12741T,C14120T,C14408T,C14676T,C15279T,T16176C,C20628G,A23063T,C23271A,A23403G,C23604A,C23709T,T24506G,G24914C,C27972T,C28000A,G28048T,G28075T,A28095T,A28111G,G28280C,A28281T,T28282A,T28688C,G28881A,G28882A,G28883C,C28977T	27	N:D3L,N:R203K,N:G204R,N:S235F,ORF1a:P971L,ORF1a:T1001I,ORF1a:T1542I,ORF1a:A1708D,ORF1a:I2230T,ORF1a:M2259I,ORF1a:D3222N,ORF1a:T4159I,ORF1b:P218L,ORF1b:P314L,ORF8:Q27*,ORF8:P36Q,ORF8:R52I,ORF8:C61F,ORF8:K68*,ORF8:Y73C,S:N501Y,S:A570D,S:D614G,S:P681H,S:T716I,S:S982A,S:D1	6	0
hCoV-19/Mexico/NLE-InDRE_FB17360_S3921/2021	EPI_ISL_2858935	-	Q.3	GRY	37	G149T,C241T,C913T,C3037T,C3267T,G5023A,C5388A,C5986T,T6954C,G10396T,C14408T,C14676T,C15279T,T16176C,A17615G,G20962A,C21108T,A23063T,C23271A,A23403G,C23604A,C23709T,C24502A,T24506G,G24914C,G26730C,C27972T,G28048T,A28111G,G28280C,A28281T,T28282A,G28881A,G28882A,G28883C,C28977T,G29440T	23	M:V70L,N:D3L,N:R203K,N:G204R,N:S235F,N:Q389H,ORF1a:T1001I,ORF1a:M1586I,ORF1a:A1708D,ORF1a:I2230T,ORF1b:P314L,ORF1b:K1383R,ORF1b:D2499N,ORF8:Q27*,ORF8:R52I,ORF8:Y73C,S:N501Y,S:A570D,S:D614G,S:P681H,S:T716I,S:S982A,S:D1118H	6	0
hCoV-19/Mexico/NLE-InDRE_FB17366_S3922/2021	EPI_ISL_2858936	-	B.1.1.7	GRY	40	C241T,C913T,C2110T,C3037T,C3177T,C3267T,C4890T,C5388A,C5986T,T6954C,G7042T,G9929A,A10195G,C12741T,C14120T,C14408T,C14676T,C15279T,T16176C,C20628G,A23063T,C23271A,A23403G,C23604A,C23709T,T24506G,G24914C,C27972T,C28000A,G28048T,G28075T,A28111G,G28280C,A28281T,T28282A,T28688C,G28881A,G28882A,G28883C,C28977T	26	N:D3L,N:R203K,N:G204R,N:S235F,ORF1a:P971L,ORF1a:T1001I,ORF1a:T1542I,ORF1a:A1708D,ORF1a:I2230T,ORF1a:M2259I,ORF1a:D3222N,ORF1a:T4159I,ORF1b:P218L,ORF1b:P314L,ORF8:Q27*,ORF8:P36Q,ORF8:R52I,ORF8:C61F,ORF8:Y73C,S:N501Y,S:A570D,S:D614G,S:P681H,S:T716I,S:S982A,S:D1118H	6	0
hCoV-19/Mexico/NLE-InDRE_FB17368_S3923/2021	EPI_ISL_2858937	-	B.1.1.7	GRY	41	C241T,C913T,C2110T,C3037T,C3177T,C3267T,C4890T,C5388A,C5986T,T6954C,G7042T,G9929A,A10195G,C11050T,C12741T,C14120T,C14408T,C14676T,C15279T,T16176C,C20628G,A23063T,C23271A,A23403G,C23604A,C23709T,T24506G,G24914C,C27972T,C28000A,G28048T,G28075T,A28111G,G28280C,A28281T,T28282A,T28688C,G28881A,G28882A,G28883C,C28977T	26	N:D3L,N:R203K,N:G204R,N:S235F,ORF1a:P971L,ORF1a:T1001I,ORF1a:T1542I,ORF1a:A1708D,ORF1a:I2230T,ORF1a:M2259I,ORF1a:D3222N,ORF1a:T4159I,ORF1b:P218L,ORF1b:P314L,ORF8:Q27*,ORF8:P36Q,ORF8:R52I,ORF8:C61F,ORF8:Y73C,S:N501Y,S:A570D,S:D614G,S:P681H,S:T716I,S:S982A,S:D1118H	6	0
hCoV-19/Mexico/NLE-InDRE_FB18082_S4116/2021	EPI_ISL_2920687	-	B.1.1.7	GRY	42	C241T,C913T,C1346T,C2110T,C3037T,C3177T,C3267T,C4890T,C5388A,C5986T,T6954C,G7042T,G9929A,A10195G,C11050T,C12741T,C14120T,C14408T,C14676T,C15279T,T16176C,C20628G,A23063T,C23271A,A23403G,C23604A,C23709T,T24506G,G24914C,A25336C,C27972T,C28000A,G28048T,G28075T,A28111G,G28280C,A28281T,T28282A,G28881A,G28882A,G28883C,C28977T	28	N:D3L,N:R203K,N:G204R,N:S235F,ORF1a:P361S,ORF1a:P971L,ORF1a:T1001I,ORF1a:T1542I,ORF1a:A1708D,ORF1a:I2230T,ORF1a:M2259I,ORF1a:D3222N,ORF1a:T4159I,ORF1b:P218L,ORF1b:P314L,ORF8:Q27*,ORF8:P36Q,ORF8:R52I,ORF8:C61F,ORF8:Y73C,S:N501Y,S:A570D,S:D614G,S:P681H,S:T716I,S:S982A,S:D1118H,S:E1258D	6	0
hCoV-19/Mexico/NLE-InDRE_FB18085_S4117/2021	EPI_ISL_2920688	-	B.1.1.7	GRY	44	C241T,C913T,C2110T,C3037T,C3267T,C5388A,C5986T,T6954C,G7042T,C7420T,G10105T,A10195G,C13860T,C14120T,C14408T,C14676T,C15279T,C15933T,T16176C,C20480T,A23063T,T23152A,C23271A,A23403G,C23604A,C23709T,C23757T,T24506G,G24914C,A25336C,A27745G,C27972T,G28048T,G28075T,A28111G,G28280C,A28281T,T28282A,C28512A,G28881A,G28882A,G28	29	N:D3L,N:P80Q,N:R203K,N:G204R,N:S235F,N:A267S,ORF1a:T1001I,ORF1a:A1708D,ORF1a:I2230T,ORF1a:M2259I,ORF1a:M3280I,ORF1b:P218L,ORF1b:P314L,ORF1b:S2338L,ORF7a:R118G,ORF8:Q27*,ORF8:R52I,ORF8:C61F,ORF8:Y73C,ORF9b:Q77K,S:N501Y,S:A570D,S:D614G,S:P681H,S:T716I,S:T732I,S:S982A,S:D	6	0
hCoV-19/Mexico/NLE-InDRE_FB18090_S4118/2021	EPI_ISL_2920689	-	B.1.1.7	GRY	46	C241T,C913T,C2110T,C3037T,C3177T,C3267T,C4890T,C5388A,C5986T,T6954C,G7042T,G9929A,A10195G,C11050T,C12741T,C14120T,C14408T,C14676T,C14724T,C15279T,T16176C,C19011T,C20628G,A23063T,C23271A,A23403G,C23604A,C23709T,T24506G,G24914C,A25336C,G25337C,C25710T,C26228T,C27972T,C28000A,G28048T,G28075T,A28111G,G28280C,A28281T,T28282A,G28881A,G28882A,G28883C,C28977T	28	N:D3L,N:R203K,N:G204R,N:S235F,ORF1a:P971L,ORF1a:T1001I,ORF1a:T1542I,ORF1a:A1708D,ORF1a:I2230T,ORF1a:M2259I,ORF1a:D3222N,ORF1a:T4159I,ORF1b:P218L,ORF1b:P314L,ORF8:Q27*,ORF8:P36Q,ORF8:R52I,ORF8:C61F,ORF8:Y73C,S:N501Y,S:A570D,S:D614G,S:P681H,S:T716I,S:S982A,S:D1118H,S:E1258D,S:D1259H	6	0
hCoV-19/Mexico/NLE-InDRE_FB18093_S4119/2021	EPI_ISL_2920690	-	B.1.1.7	GRY	44	C241T,C913T,C2110T,C3037T,C3177T,C3267T,C4890T,C5388A,C5986T,T6954C,G7042T,G9929A,A10195G,C11050T,C12741T,C14120T,C14408T,C14676T,C15279T,T16176C,C20628G,G22344T,A23063T,C23271A,A23403G,C23601T,C23604A,C23709T,T24506G,G24914C,C27972T,C28000A,G28048T,G28075T,A28111G,G28280C,A28281T,T28282A,T28688C,G28881A,G28882A,G2888	28	N:D3L,N:R203K,N:G204R,N:S235F,ORF1a:P971L,ORF1a:T1001I,ORF1a:T1542I,ORF1a:A1708D,ORF1a:I2230T,ORF1a:M2259I,ORF1a:D3222N,ORF1a:T4159I,ORF1b:P218L,ORF1b:P314L,ORF8:Q27*,ORF8:P36Q,ORF8:R52I,ORF8:C61F,ORF8:Y73C,S:G261V,S:N501Y,S:A570D,S:D614G,S:S680F,S:P681H,S:T716I,S:S982	6	0
hCoV-19/Mexico/NLE-InDRE_FB18094_S4120/2021	EPI_ISL_2920691	-	B.1.1.7	GRY	43	C241T,C913T,C2110T,C2143T,C2675T,C3037T,C3267T,C5144T,C5388A,C5986T,A6403T,T6954C,G7042T,C9246T,A10195G,C10834T,C14120T,C14408T,C14676T,C15279T,T16176C,A23063T,C23271A,A23403G,C23604A,C23709T,T24506G,G24914C,A25336C,G25337C,G25650T,C27972T,G28048T,G28075T,A28111G,G28280C,A28281T,T28282A,G28881A,G28882A,G28883C,C28977	26	N:D3L,N:R203K,N:G204R,N:S235F,ORF1a:P804S,ORF1a:T1001I,ORF1a:A1708D,ORF1a:I2230T,ORF1a:M2259I,ORF1a:A2994V,ORF1b:P218L,ORF1b:P314L,ORF3a:L86F,ORF8:Q27*,ORF8:R52I,ORF8:C61F,ORF8:Y73C,S:N501Y,S:A570D,S:D614G,S:P681H,S:T716I,S:S982A,S:D1118H,S:E1258D,S:D1259H	6	0
hCoV-19/Mexico/NLE-InDRE_FB18095_S4121/2021	EPI_ISL_2920692	-	B.1.1.7	GRY	42	C241T,C913T,C2110T,C2143T,C2675T,C3037T,C3267T,C5144T,C5388A,C5986T,A6403T,T6954C,G7042T,C9246T,A10195G,C10834T,C14120T,C14408T,C14676T,C15279T,T16176C,A23063T,C23271A,A23403G,C23604A,C23709T,T24506G,G24914C,A25336C,G25650T,C27972T,G28048T,G28075T,A28111G,G28280C,A28281T,T28282A,G28881A,G28882A,G28883C,C28977T,C29642T	25	N:D3L,N:R203K,N:G204R,N:S235F,ORF1a:P804S,ORF1a:T1001I,ORF1a:A1708D,ORF1a:I2230T,ORF1a:M2259I,ORF1a:A2994V,ORF1b:P218L,ORF1b:P314L,ORF3a:L86F,ORF8:Q27*,ORF8:R52I,ORF8:C61F,ORF8:Y73C,S:N501Y,S:A570D,S:D614G,S:P681H,S:T716I,S:S982A,S:D1118H,S:E1258D	6	0
hCoV-19/Mexico/NLE-InDRE_FB18317_S3969/2021	EPI_ISL_2858983	-	B.1.1.7	GRY	42	C241T,C913T,C2110T,C3037T,C3177T,C3267T,C5388A,C5986T,T6954C,G7042T,A9750G,G9929A,A10195G,C14120T,C14408T,C14676T,C15279T,T16176C,T17088A,C21614T,A23063T,C23271A,C23277T,A23403G,C23604A,C23709T,T24506G,G24914C,A25336C,C27972T,G28048T,G28075T,A28111G,G28280C,A28281T,T28282A,C28310A,T28688C,G28881A,G28882A,G28883C,C2897	29	N:D3L,N:P13T,N:R203K,N:G204R,N:S235F,ORF1a:P971L,ORF1a:T1001I,ORF1a:A1708D,ORF1a:I2230T,ORF1a:M2259I,ORF1a:K3162R,ORF1a:D3222N,ORF1b:P218L,ORF1b:P314L,ORF8:Q27*,ORF8:R52I,ORF8:C61F,ORF8:Y73C,ORF9b:H9Q,S:L18F,S:N501Y,S:A570D,S:T572I,S:D614G,S:P681H,S:T716I,S:S982A,S:D111	6	0
hCoV-19/Mexico/NLE-LESPNL-20210407010/2021	EPI_ISL_1911302	-	B.1.1.7	O	32	C292T,C913T,C1060T,C3037T,C3267T,C5986T,T6954C,C14408T,C14676T,T16176C,T16563A,A23063T,C23170T,C23271A,A23403G,C23604A,C23709T,T24506G,G24914C,C25413T,C25626T,C27972T,G28048T,A28111G,G28280C,A28281T,T28282A,A28295C,C28728T,G28881A,G28882A,G	19	N:D3L,N:N8H,N:A152V,N:R203K,N:G204R,ORF1a:T1001I,ORF1a:I2230T,ORF1b:P314L,ORF8:Q27*,ORF8:R52I,ORF8:Y73C,ORF9b:K4N,S:N501Y,S:A570D,S:D614G,S:P681H,S:T716I,S:S982A,S:D1118H	3	940
hCoV-19/Mexico/NLE-LESPNL-20210512-417/2021	EPI_ISL_2102612	-	B.1.576	O	19	C527T,C2110T,C3267T,G4288T,C5986T,T6954C,C14408T,C14676T,C15279T,T16176C,T17310C,C23271A,A23403G,C23604A,G24914C,A25315G,C27972T,A28095T,A28111G	11	ORF1a:T1001I,ORF1a:E1341D,ORF1a:I2230T,ORF1b:P314L,ORF8:Q27*,ORF8:K68*,ORF8:Y73C,S:A570D,S:D614G,S:P681H,S:D1118H	1	2643
hCoV-19/Mexico/NLE-LESPNL-20210512-431/2021	EPI_ISL_2102626	-	B.1.1	O	21	C913T,C3037T,C5986T,T6954C,C14408T,C15279T,T16176C,A17153G,C19554T,A23063T,C23271A,A23403G,C23604A,C24374T,G24914C,C27972T,G28774T,G28881A,G28882A,G28883C,C28	14	N:L167F,N:R203K,N:G204R,N:S235F,ORF1a:I2230T,ORF1b:P314L,ORF1b:Y1229C,ORF8:Q27*,S:N501Y,S:A570D,S:D614G,S:P681H,S:L938F,S:D1118H	6	1327
hCoV-19/Mexico/NLE-LESPNL-20210512-433/2021	EPI_ISL_2102628	-	B.1.1.7	O	22	C3037T,C3267T,C5986T,T6954C,C9611T,C14408T,C14676T,T16176C,C23271A,C23604A,C23709T,G24914C,C27972T,G28048T,A28111G,G28280C,A28281T,T28282A,A28295G,G28881A,G28	15	N:D3L,N:N8D,N:R203K,N:G204R,ORF1a:T1001I,ORF1a:I2230T,ORF1a:L3116F,ORF1b:P314L,ORF8:Q27*,ORF8:R52I,ORF8:Y73C,S:A570D,S:P681H,S:T716I,S:D	0	1728
hCoV-19/Mexico/NLE-LESPNL-20210512-435/2021	EPI_ISL_2102630	-	B.1.1.7	G	25	C241T,C913T,C3037T,C3267T,T6954C,C8318T,C11497T,C11750T,C14408T,C14676T,T15096C,T16176C,G16975T,C23271A,A23403G,C23604A,C23709T,T24506G,C27972T,A28095T,A28111G,G28280C,A28281T,T28282A,C28977T	16	N:D3L,N:S235F,ORF1a:T1001I,ORF1a:I2230T,ORF1a:P2685S,ORF1a:L3829F,ORF1b:P314L,ORF1b:V1170F,ORF8:Q27*,ORF8:K68*,ORF8:Y73C,S:A570D,S:D614G,S:P681H,S:T716I,S:S982A	3	1835
hCoV-19/Mexico/NLE-LESPNL-20210512-439/2021	EPI_ISL_2102633	-	B.1.1.7	O	25	C3037T,C3267T,T6954C,T9445C,C9611T,C14408T,C14676T,T16176C,C21855T,C23271A,A23403G,C23604A,C23709T,T24506G,G24914C,C27972T,A28111G,G28280C,A28281T,T28282A,A28295G,G28881A,G28882A,G28883C,C28977T	18	N:D3L,N:N8D,N:R203K,N:G204R,N:S235F,ORF1a:T1001I,ORF1a:I2230T,ORF1a:L3116F,ORF1b:P314L,ORF8:Q27*,ORF8:Y73C,S:S98F,S:A570D,S:D614G,S:P681H,S:T716I,S:S982A,S:D1118H	0	1899
hCoV-19/Mexico/NLE-LESPNL-20210512-453/2021	EPI_ISL_2102645	-	B.1.1.7	O	27	C222T,C241T,C3037T,C3267T,C5986T,C14408T,C15279T,T16176C,G20274T,A23063T,C23271A,C23604A,C23709T,T24506G,G24914C,T25180C,C27972T,G28048T,A28095T,A28111G,G28280C,A28281T,T28282A,G28881A,G28882A,G28883C,C28977T	17	N:D3L,N:R203K,N:G204R,N:S235F,ORF1a:T1001I,ORF1b:P314L,ORF1b:M2269I,ORF8:Q27*,ORF8:R52I,ORF8:K68*,ORF8:Y73C,S:N501Y,S:A570D,S:P681H,S:T716I,S:S982A,S:D1118H	3	2145

hCoV-19/Mexico/NLE-LESPNL-20210512-454/2021	EPI_ISL_2102646	-	B.1.576	O	23	C222T,C241T,C913T,C2110T,C3267T,C8127T,C14408T,C14676T,C15279T,T16176C,C17762T,G20274T,A23063T,C23271A,A23403G,C23604A,G24914C,C27972T,A28095T,A28111G,G28280C,A	14	N:D3L,ORF1a:T1001I,ORF1a:A2621V,ORF1b:P314L,ORF1b:A1432V,ORF1b:M2269I,ORF8:Q27*,ORF8:K68*,ORF8:Y73C,S:N501Y,S:A570D,S:D614G,S:P681H,S	1	2406
hCoV-19/Mexico/NLE-LESPNL-20210512-455/2021	EPI_ISL_2102647	-	B.1.1	O	27	C292T,C2156T,C3037T,C3267T,T6954C,C14408T,C14676T,C15279T,T16176C,T16563A,C16580T,G19101T,C23170T,C23271A,C23604A,G24914C,C25626T,C27972T,A28111G,G28280C,A28281T,T28282A,A28295C,G28881A,G28882A,G28883C,C28977T	17	N:D3L,N:N8H,N:R203K,N:G204R,N:S235F,ORF1a:L631F,ORF1a:T1001I,ORF1a:I2230T,ORF1b:P314L,ORF1b:T1038I,ORF1b:Q1878H,ORF8:Q27*,ORF8:Y73C,ORF9b:K4N,S:A570D,S:P681H,S:D1118H	5	1547
hCoV-19/Mexico/NLE-LESPNL-20210512-458/2021	EPI_ISL_2102650	-	B.1.1.7	O	21	C913T,C3037T,C3267T,C5986T,T6954C,G8222A,T9445C,C14676T,C15279T,T16176C,A23063T,C23271A,C23709T,G24914C,C27972T,A28111G,A28295G,G28881A,G28882A,G28883C,C28977T	13	N:N8D,N:R203K,N:G204R,N:S235F,ORF1a:T1001I,ORF1a:I2230T,ORF1a:E2653K,ORF8:Q27*,ORF8:Y73C,S:N501Y,S:A570D,S:T716I,S:D1118H	3	2509
hCoV-19/Mexico/NLE-LESPNL-20210512-460/2021	EPI_ISL_2102652	-	B.1.1	O	24	C913T,C1174T,C3037T,C3267T,T6954C,A8848G,A11477C,C14408T,C14676T,C15279T,T16176C,C23271A,C23604A,T24506G,G25489C,G26727T,C27972T,A28111G,G28280C,T28282A,G28881A,G28882A,G28883C,C28977T	15	M:A69S,N:D3Q,N:R203K,N:G204R,N:S235F,ORF1a:T1001I,ORF1a:I2230T,ORF1a:I3738L,ORF1b:P314L,ORF3a:A33P,ORF8:Q27*,ORF8:Y73C,S:A570D,S:P681H,S:S982A	1	2841
hCoV-19/Mexico/NLE-UANL-067/2021	EPI_ISL_1789696	-	B.1.1.7	GRY	39	C241T,C913T,C3037T,C3267T,C4900T,C5388A,C5986T,T6954C,A12162G,C14053T,C14408T,C14676T,C15279T,C15720T,T16176C,A17615G,A21137G,C22444T,A23063T,C23271A,A23403G,C23604A,C23709T,T24506G,G24914C,G25135T,C27972T,G28048T,A28111G,G28280C,A28281T,T28282A,C28603T,G28881A,G28882A,G28883C,A28967G,C28977T,G29711T	23	N:D3L,N:R203K,N:G204R,N:S232G,N:S235F,ORF1a:T1001I,ORF1a:A1708D,ORF1a:I2230T,ORF1a:Q3966R,ORF1b:P314L,ORF1b:K1383R,ORF1b:K2557R,ORF8:Q27*,ORF8:R52I,ORF8:Y73C,S:N501Y,S:A570D,S:D614G,S:P681H,S:T716I,S:S982A,S:D1118H,S:K1191N	6	0
hCoV-19/Mexico/NLE-UANL-068/2021	EPI_ISL_1789697	-	B.1.1.7	GRY	38	C241T,C913T,C3037T,C3267T,C4900T,C5388A,C5986T,T6954C,A12162G,C14053T,C14408T,C14676T,C15279T,C15720T,T16176C,A17615G,A21137G,C22444T,A23063T,C23271A,A23403G,C23604A,C23709T,T24506G,G24914C,G25135T,C27972T,G28048T,A28111G,G28280C,A28281T,T28282A,G28881A,G28882A,G28883C,A28967G,C28977T,G29711T	23	N:D3L,N:R203K,N:G204R,N:S232G,N:S235F,ORF1a:T1001I,ORF1a:A1708D,ORF1a:I2230T,ORF1a:Q3966R,ORF1b:P314L,ORF1b:K1383R,ORF1b:K2557R,ORF8:Q27*,ORF8:R52I,ORF8:Y73C,S:N501Y,S:A570D,S:D614G,S:P681H,S:T716I,S:S982A,S:D1118H,S:K1191N	6	0
hCoV-19/Mexico/NLE-UANL-075/2021	EPI_ISL_1789699	-	B.1.1.7	GRY	36	C241T,C913T,C2110T,C3037T,C3267T,C5388A,C5986T,T6954C,G7042T,G9929A,A10195G,C14120T,C14408T,C14676T,C15279T,T16176C,A23063T,C23271A,A23403G,C23604A,C23709T,T24506G,G24914C,C27972T,G28048T,G28075T,A28095T,A28111G,G28280C,A28281T,T28282A,T28688C,G28881A,G28882A,G28883C,C28977T	23	N:D3L,N:R203K,N:G204R,N:S235F,ORF1a:T1001I,ORF1a:A1708D,ORF1a:I2230T,ORF1a:M2259I,ORF1a:D3222N,ORF1b:P218L,ORF1b:P314L,ORF8:Q27*,ORF8:R52I,ORF8:C61F,ORF8:K68*,ORF8:Y73C,S:N501Y,S:A570D,S:D614G,S:P681H,S:T716I,S:S982A,S:D1118H	6	0
hCoV-19/Mexico/OAX-InDRE_FB13216_S1507/2021	EPI_ISL_1805485	-	B.1.1.7	GRY	35	C241T,C913T,C3037T,C3267T,C5388A,C5986T,T6954C,A8848G,C13458T,C14408T,C14676T,C15279T,T16176C,A23063T,C23271A,A23403G,C23604A,C23709T,T24506G,G24914C,G25217T,A25336C,G25337C,G26727T,C27972T,G28048T,A28111G,G28280C,A28281T,T28282A,G28881A,G28882A,G28883C,C28977T,G29715T	23	M:A69S,N:D3L,N:R203K,N:G204R,N:S235F,ORF1a:T1001I,ORF1a:A1708D,ORF1a:I2230T,ORF1a:S4398L,ORF1b:P314L,ORF8:Q27*,ORF8:R52I,ORF8:Y73C,S:N501Y,S:A570D,S:D614G,S:P681H,S:T716I,S:S982A,S:D1118H,S:G1219C,S:E1258D,S:D1259H	6	0
hCoV-19/Mexico/OAX-InDRE_FB13300_S1504/2021	EPI_ISL_1805482	-	B.1.1.7	GRY	34	T78G,C241T,C913T,C3037T,C3267T,C5388A,C5986T,T6954C,A8848G,C14408T,C14676T,C15279T,T16176C,T22293C,A23063T,C23271A,A23403G,C23604A,C23709T,T24506G,G24914C,A25336C,C27641T,C27972T,G28048T,A28111G,G28280C,A28281T,T28282A,G28881A,G28882A,G288	21	N:D3L,N:R203K,N:G204R,N:S235F,ORF1a:T1001I,ORF1a:A1708D,ORF1a:I2230T,ORF1b:P314L,ORF7a:S83L,ORF8:Q27*,ORF8:R52I,ORF8:Y73C,S:L244S,S:N501Y,S:A570D,S:D614G,S:P681H,S:T716I,S:S982A,S:D1118H,S:E1258D	6	139
hCoV-19/Mexico/OAX-InDRE_FB14523_S2037/2021	EPI_ISL_2283681	-	B.1.1.7	GRY	35	C241T,C913T,C3037T,C3267T,C5388A,C5986T,T6954C,C7299T,A13768T,C14408T,C14676T,T15096C,C15279T,T16176C,A23063T,C23271A,A23403G,C23604A,C23709T,T24506G,G24914C,C26606T,C27972T,G28048T,A28095T,A28111G,G28280C,A28281T,T28282A,A28877T,G28878C,G28881A,G28882A,G28883C,C28977T	21	N:D3L,N:R203K,N:G204R,N:S235F,ORF1a:T1001I,ORF1a:A1708D,ORF1a:I2230T,ORF1a:A2345V,ORF1b:M101L,ORF1b:P314L,ORF8:Q27*,ORF8:R52I,ORF8:K68*,ORF8:Y73C,S:N501Y,S:A570D,S:D614G,S:P681H,S:T716I,S:S982A,S:D1118H	6	0
hCoV-19/Mexico/OAX-InDRE_FB14526_S2038/2021	EPI_ISL_2283682	-	B.1.1.7	GRY	33	C241T,C913T,C3037T,C3267T,C5388A,C5986T,C6196T,T6954C,A8848G,C14408T,C14676T,C15279T,T16176C,A23063T,C23271A,A23403G,C23604A,C23709T,T24506G,G24914C,G25217T,G26727T,C27972T,G28048T,A28111G,G28280C,A28281T,T28282A,G28881A,G28882A,G28883C,C2	20	M:A69S,N:D3L,N:R203K,N:G204R,N:S235F,ORF1a:T1001I,ORF1a:A1708D,ORF1a:I2230T,ORF1b:P314L,ORF8:Q27*,ORF8:R52I,ORF8:Y73C,S:N501Y,S:A570D,S:D614G,S:P681H,S:T716I,S:S982A,S:D1118H,S:G1219C	6	4
hCoV-19/Mexico/OAX-InDRE_FB14528_S2039/2021	EPI_ISL_2283683	-	B.1.1.7	GRY	33	C241T,C913T,C3037T,C3267T,C5388A,C5986T,C6196T,T6954C,A8848G,C14408T,C14676T,C15279T,T16176C,A23063T,C23271A,A23403G,C23604A,C23709T,T24506G,G24914C,G25217T,G26727T,C27972T,G28048T,A28111G,G28280C,A28281T,T28282A,G28881A,G28882A,G28883C,C2	20	M:A69S,N:D3L,N:R203K,N:G204R,N:S235F,ORF1a:T1001I,ORF1a:A1708D,ORF1a:I2230T,ORF1b:P314L,ORF8:Q27*,ORF8:R52I,ORF8:Y73C,S:N501Y,S:A570D,S:D614G,S:P681H,S:T716I,S:S982A,S:D1118H,S:G1219C	6	0
hCoV-19/Mexico/OAX-InDRE_FB14529_S2040/2021	EPI_ISL_2283684	-	B.1.1.7	GRY	30	C241T,C913T,C3037T,C3267T,C5388A,C5986T,T6954C,A8848G,C14408T,C14676T,C15279T,T16176C,A23063T,C23271A,A23403G,C23604A,C23709T,T24506G,G24914C,C27972T,G28048T,A28111G,G28280C,A28281T,T28282A,G28881A,G28882A,G28883C,C28977T,G29715T	18	N:D3L,N:R203K,N:G204R,N:S235F,ORF1a:T1001I,ORF1a:A1708D,ORF1a:I2230T,ORF1b:P314L,ORF8:Q27*,ORF8:R52I,ORF8:Y73C,S:N501Y,S:A570D,S:D614G,S:P681H,S:T716I,S:S982A,S:D1118H	6	180
hCoV-19/Mexico/OAX-InDRE_FB14532_S2042/2021	EPI_ISL_2283685	-	B.1.1.7	GRY	40	C241T,C913T,C3037T,C3267T,C5388A,C5986T,T6954C,C7299T,A13768T,C14408T,C14676T,T15096C,C15279T,T16176C,C19862T,A23063T,C23271A,A23403G,C23604A,C23709T,T24506G,G24914C,G25244T,C26606T,G27281T,C27972T,G28048T,A28095T,A28111G,G28280C,A28281T,T28282A,A28877T,G28878C,G28881A,G28882A,G28883C,C28977T,G29392C,A29491G	25	N:D3L,N:R203K,N:G204R,N:S235F,N:K373N,ORF1a:T1001I,ORF1a:A1708D,ORF1a:I2230T,ORF1a:A2345V,ORF1b:M101L,ORF1b:P314L,ORF1b:A2132V,ORF6:W27L,ORF8:Q27*,ORF8:R52I,ORF8:K68*,ORF8:Y73C,S:N501Y,S:A570D,S:D614G,S:P681H,S:T716I,S:S982A,S:D1118H,S:V1228L	6	10
hCoV-19/Mexico/OAX-InDRE_FB14970_S2052/2021	EPI_ISL_2283694	-	B.1.1.7	GRY	39	C241T,C913T,C3037T,C3267T,C5388A,G5924T,C5986T,C6196T,T6954C,A8848G,C14408T,C14676T,C15279T,T16176C,C18568T,G19656T,C19862T,G21255C,A23063T,C23271A,A23403G,C23604A,C23709T,T24506G,G24914C,G25217T,G26428T,G26727T,C27972T,G28048T,A28111G,G28280C,A28281T,T28282A,G28881A,G28882A,G28883C,C28977T,G29715T	25	E:V62F,M:A69S,N:D3L,N:R203K,N:G204R,N:S235F,ORF1a:T1001I,ORF1a:A1708D,ORF1a:V1887F,ORF1a:I2230T,ORF1b:P314L,ORF1b:L1701F,ORF1b:K2063N,ORF1b:A2132V,ORF8:Q27*,ORF8:R52I,ORF8:Y73C,S:N501Y,S:A570D,S:D614G,S:P681H,S:T716I,S:S982A,S:D1118H,S:G1219C	6	6
hCoV-19/Mexico/OAX-InDRE_FB15918_S2397/2021	EPI_ISL_2455922	-	B.1.1.7	GRY	42	C241T,C913T,C1059T,C3037T,C3267T,C5388A,G5924T,C5986T,C6196T,T6954C,A8848G,C14408T,C14676T,C15279T,T16176C,C18568T,G19656T,C19862T,C21008T,G21255C,A23063T,C23271A,A23403G,C23604A,C23709T,T24506G,G24914C,G25217T,A25336C,G26428T,G26727T,C27972T,G28048T,A28111G,G28280C,A28281T,T28282A,G28881A,G28882A,G28883C,C28977T,G29	28	E:V62F,M:A69S,N:D3L,N:R203K,N:G204R,N:S235F,ORF1a:T265I,ORF1a:T1001I,ORF1a:A1708D,ORF1a:V1887F,ORF1a:I2230T,ORF1b:P314L,ORF1b:L1701F,ORF1b:K2063N,ORF1b:A2132V,ORF1b:T2514I,ORF8:Q27*,ORF8:R52I,ORF8:Y73C,S:N501Y,S:A570D,S:D614G,S:P681H,S:T716I,S:S982A,S:D1118H,S:G1219C,S:	6	0
hCoV-19/Mexico/OAX-InDRE_FB17118_S3085/2021	EPI_ISL_2663352	-	B.1.1.7	GRY	36	C241T,C913T,C3037T,C3267T,C5388A,C5986T,T6954C,C7299T,A13768T,C14408T,C14676T,T15096C,C15279T,T16176C,C19862T,A23063T,C23271A,A23403G,C23604A,C23709T,T24506G,G24914C,C26606T,G27281T,C27972T,G28048T,A28095T,A28111G,G28280C,A28281T,T28282A,G28881A,G28882A,G28883C,C28977T,A29491G	23	N:D3L,N:R203K,N:G204R,N:S235F,ORF1a:T1001I,ORF1a:A1708D,ORF1a:I2230T,ORF1a:A2345V,ORF1b:M101L,ORF1b:P314L,ORF1b:A2132V,ORF6:W27L,ORF8:Q27*,ORF8:R52I,ORF8:K68*,ORF8:Y73C,S:N501Y,S:A570D,S:D614G,S:P681H,S:T716I,S:S982A,S:D1118H	6	0
hCoV-19/Mexico/OAX-InDRE_FB18623_S4153/2021	EPI_ISL_2920724	-	B.1.1.7	GRY	40	C241T,C913T,C3037T,C3267T,A3492G,C4331T,C5388A,C5986T,T6954C,C10789T,C13423A,C13424A,C14408T,C14676T,C15279T,T16176C,C18555T,C21639A,A23063T,C23271A,A23403G,A23523C,C23604A,C23709T,C24374T,T24506G,G24914C,A25336C,G25337C,C27972T,G28048T,A28111G,G28280C,A28281T,T28282A,G28881A,G28882A,G28883C,C28977T,C29732T	25	N:D3L,N:R203K,N:G204R,N:S235F,ORF1a:T1001I,ORF1a:N1076S,ORF1a:A1708D,ORF1a:I2230T,ORF1a:R4387S,ORF1b:P314L,ORF8:Q27*,ORF8:R52I,ORF8:Y73C,S:P26H,S:N501Y,S:A570D,S:D614G,S:E654A,S:P681H,S:T716I,S:L938F,S:S982A,S:D1118H,S:E1258D,S:D1259H	6	0
hCoV-19/Mexico/PUE-InDRE_FB16502_S2472/2021	EPI_ISL_2455993	-	B.1.1.7	GRY	36	T201A,C241T,C913T,C2110T,C3037T,C3267T,C5388A,C5986T,T6954C,G7042T,A10195G,C13329T,C14120T,C14408T,C14676T,C15279T,T16176C,T22266C,A23063T,C23271A,A23403G,C23604A,C23709T,T24506G,G24914C,C27972T,G28048T,G28075T,A28111G,G28280C,A28281T,T28282A,G28881A,G28882A,G28883C,C28977T	23	N:D3L,N:R203K,N:G204R,N:S235F,ORF1a:T1001I,ORF1a:A1708D,ORF1a:I2230T,ORF1a:M2259I,ORF1a:T4355I,ORF1b:P218L,ORF1b:P314L,ORF8:Q27*,ORF8:R52I,ORF8:C61F,ORF8:Y73C,S:I235T,S:N501Y,S:A570D,S:D614G,S:P681H,S:T716I,S:S982A,S:D1118H	6	0
hCoV-19/Mexico/PUE-InDRE_FB16507_S2473/2021	EPI_ISL_2455994	-	B.1.1.7	GRY	38	T201A,C241T,C913T,C2110T,C3037T,C3267T,C5388A,C5986T,T6954C,G7042T,A10195G,C13329T,C14120T,C14408T,C14676T,C15279T,T16176C,C19944T,A23063T,C23271A,A23403G,T23443C,C23604A,C23709T,T24506G,G24914C,T25612G,C27972T,G28048T,G28075T,A28111G,G28280C,A28281T,T28282A,G28881A,G28882A,G28883C,C28977T	23	N:D3L,N:R203K,N:G204R,N:S235F,ORF1a:T1001I,ORF1a:A1708D,ORF1a:I2230T,ORF1a:M2259I,ORF1a:T4355I,ORF1b:P218L,ORF1b:P314L,ORF3a:S74A,ORF8:Q27*,ORF8:R52I,ORF8:C61F,ORF8:Y73C,S:N501Y,S:A570D,S:D614G,S:P681H,S:T716I,S:S982A,S:D1118H	6	46
hCoV-19/Mexico/PUE-InDRE_FB17005_S3132/2021	EPI_ISL_2663399	-	B.1.1.7	GRY	37	C241T,C913T,C3037T,C3267T,C5388A,C5944T,C5986T,T6954C,C9967T,C13585T,C14408T,C14676T,T15096C,C15279T,T16176C,G18412T,C22987T,A23063T,C23271A,A23403G,C23604A,C23709T,T24506G,G24914C,C27972T,G28048T,A28095T,A28111G,G28280C,A28281T,T28282A,G28881A,G28882A,G28883C,G28884C,C28977T,G29162T	21	N:D3L,N:R203K,N:G204P,N:S235F,N:D297Y,ORF1a:T1001I,ORF1a:A1708D,ORF1a:I2230T,ORF1b:P314L,ORF1b:V1649F,ORF8:Q27*,ORF8:R52I,ORF8:K68*,ORF8:Y73C,S:N501Y,S:A570D,S:D614G,S:P681H,S:T716I,S:S982A,S:D1118H	6	0
hCoV-19/Mexico/PUE-InDRE_FB17749_S3520/2021	EPI_ISL_2779035	-	B.1.1.7	GRY	25	C203T,C241T,C913T,C3037T,C5388A,T6954C,A8848G,C14408T,A23063T,T23332C,A23403G,C23604A,C23709T,G24914C,G25217T,G25538A,G26727T,C27972T,G28048T,A28111G,G28881A,G28882A,G28883C,C28977T,G29715T	17	M:A69S,N:R203K,N:G204R,N:S235F,ORF1a:A1708D,ORF1a:I2230T,ORF1b:P314L,ORF3a:G49D,ORF8:Q27*,ORF8:R52I,ORF8:Y73C,S:N501Y,S:D614G,S:P681H,S:T716I,S:D1118H,S:G1219C	3	0

hCoV-19/Mexico/PUE-InDRE_FB17750_S3521/2021	EPI_ISL_2779036	-	B.1.1.7	GRY	39	C241T,C913T,C2449T,C3037T,C3267T,C5388A,C5944T,C5986T,C6402T,T6954C,C9166T,A10323G,C14408T,C14676T,T15096C,C15279T,T16176C,A23063T,C23271A,A23403G,C23604A,C23709T,T24506G,G24914C,C27972T,G28048T,T28053C,A28095T,A28111G,G28280C,A28281T,T28282A,G28881A,G28882A,G28883C,G28884C,C28977T,T29710C,G29759T	22	N:D3L,N:R203K,N:G204P,N:S235F,ORF1a:T1001I,ORF1a:A1708D,ORF1a:P2046L,ORF1a:I2230T,ORF1a:K3353R,ORF1b:P314L,ORF8:Q27*,ORF8:R52I,ORF8:S54P,ORF8:K68*,ORF8:Y73C,S:N501Y,S:A570D,S:D614G,S:P681H,S:T716I,S:S982A,S:D1118H	6	0
hCoV-19/Mexico/PUE-InDRE_FB17754_S3522/2021	EPI_ISL_2779037	-	B.1.1.7	GRY	35	C241T,C913T,C3037T,C3267T,C4331T,C5388A,C5986T,T6954C,C14408T,C14676T,T15096C,C15279T,T16176C,C19164T,A23063T,C23271A,A23403G,C23604A,C23709T,T24280C,T24506G,G24914C,C25096T,G27459T,C27972T,G28048T,A28095T,A28111G,G28280C,A28281T,T28282A,G28881A,G28882A,G28883C,C28977T	20	N:D3L,N:R203K,N:G204R,N:S235F,ORF1a:T1001I,ORF1a:A1708D,ORF1a:I2230T,ORF1b:P314L,ORF7a:E22D,ORF8:Q27*,ORF8:R52I,ORF8:K68*,ORF8:Y73C,S:N501Y,S:A570D,S:D614G,S:P681H,S:T716I,S:S982A,S:D1118H	6	0
hCoV-19/Mexico/PUE-InDRE_FB17755_S3523/2021	EPI_ISL_2779038	-	B.1.1.7	GRY	38	T201A,C241T,C913T,C2110T,C3037T,C3267T,C5388A,C5986T,T6954C,G7042T,A10195G,C13329T,C14120T,C14408T,C14676T,C15279T,T16176C,A23063T,C23271A,A23403G,C23604A,C23709T,T24506G,G24914C,T25612G,C27972T,G28048T,G28075T,A28095T,A28111G,G28280C,A28281T,T28282A,G28881A,G28882A,G28883C,G28904T,C28977T	25	N:D3L,N:R203K,N:G204R,N:A211S,N:S235F,ORF1a:T1001I,ORF1a:A1708D,ORF1a:I2230T,ORF1a:M2259I,ORF1a:T4355I,ORF1b:P218L,ORF1b:P314L,ORF3a:S74A,ORF8:Q27*,ORF8:R52I,ORF8:C61F,ORF8:K68*,ORF8:Y73C,S:N501Y,S:A570D,S:D614G,S:P681H,S:T716I,S:S982A,S:D1118H	6	0
hCoV-19/Mexico/PUE-InDRE_FB17756_S3524/2021	EPI_ISL_2779039	-	B.1.1.7	GRY	39	T201A,C241T,C913T,C2110T,C3037T,C3267T,C5388A,C5986T,T6954C,G7042T,A10195G,C13329T,C14120T,C14408T,C14676T,C15279T,T16176C,A23063T,C23271A,A23403G,C23604A,C23709T,T24506G,G24914C,T25612G,C27972T,G28048T,G28075T,A28095T,A28111G,G28280C,A28281T,T28282A,G28881A,G28882A,G28883C,G28904T,C28977T,G29543T	25	N:D3L,N:R203K,N:G204R,N:A211S,N:S235F,ORF1a:T1001I,ORF1a:A1708D,ORF1a:I2230T,ORF1a:M2259I,ORF1a:T4355I,ORF1b:P218L,ORF1b:P314L,ORF3a:S74A,ORF8:Q27*,ORF8:R52I,ORF8:C61F,ORF8:K68*,ORF8:Y73C,S:N501Y,S:A570D,S:D614G,S:P681H,S:T716I,S:S982A,S:D1118H	6	0
hCoV-19/Mexico/PUE-InDRE_FB17764_S3525/2021	EPI_ISL_2779040	-	B.1.1.7	GRY	36	C241T,C392T,C913T,C3037T,C3267T,C5388A,C5986T,C6196T,T6954C,A8848G,C14408T,C14676T,C15279T,T16176C,A23063T,C23271A,T23332C,A23403G,C23604A,C23709T,T24506G,G24914C,G25217T,G25538A,G26727T,C27972T,G28048T,A28111G,G28280C,A28281T,T28282A,G28881A,G28882A,G28883C,C28977T,G29715T	22	M:A69S,N:D3L,N:R203K,N:G204R,N:S235F,ORF1a:R43C,ORF1a:T1001I,ORF1a:A1708D,ORF1a:I2230T,ORF1b:P314L,ORF3a:G49D,ORF8:Q27*,ORF8:R52I,ORF8:Y73C,S:N501Y,S:A570D,S:D614G,S:P681H,S:T716I,S:S982A,S:D1118H,S:G1219C	6	0
hCoV-19/Mexico/PUE-InDRE_FB17766_S3526/2021	EPI_ISL_2779041	-	B.1.1.7	GRY	38	T201A,C241T,C913T,C2110T,C3037T,C3267T,C5388A,C5986T,T6954C,G7042T,A10195G,C13329T,C14120T,C14408T,C14676T,C15279T,T16176C,C19536T,A23063T,C23271A,A23403G,C23604A,C23709T,T24506G,G24914C,T25612G,C27972T,G28048T,G28075T,A28095T,A28111G,G28280C,A28281T,T28282A,G28881A,G28882A,G28883C,C28977T	24	N:D3L,N:R203K,N:G204R,N:S235F,ORF1a:T1001I,ORF1a:A1708D,ORF1a:I2230T,ORF1a:M2259I,ORF1a:T4355I,ORF1b:P218L,ORF1b:P314L,ORF3a:S74A,ORF8:Q27*,ORF8:R52I,ORF8:C61F,ORF8:K68*,ORF8:Y73C,S:N501Y,S:A570D,S:D614G,S:P681H,S:T716I,S:S982A,S:D1118H	6	0
hCoV-19/Mexico/PUE-InDRE_FB17767_S3527/2021	EPI_ISL_2779042	-	B.1.1.7	GRY	38	T201A,C241T,C913T,C2110T,C3037T,C3267T,C5388A,C5986T,T6954C,G7042T,A10195G,C13329T,C14120T,C14408T,C14676T,C15279T,T16176C,A23063T,C23271A,A23403G,C23604A,C23709T,T24506G,G24914C,T25612G,C27972T,G28048T,G28075T,A28095T,A28111G,G28280C,A28281T,T28282A,G28881A,G28882A,G28883C,G28904T,C28977T	25	N:D3L,N:R203K,N:G204R,N:A211S,N:S235F,ORF1a:T1001I,ORF1a:A1708D,ORF1a:I2230T,ORF1a:M2259I,ORF1a:T4355I,ORF1b:P218L,ORF1b:P314L,ORF3a:S74A,ORF8:Q27*,ORF8:R52I,ORF8:C61F,ORF8:K68*,ORF8:Y73C,S:N501Y,S:A570D,S:D614G,S:P681H,S:T716I,S:S982A,S:D1118H	6	0
hCoV-19/Mexico/PUE-InDRE_FB19607_S4512/2021	EPI_ISL_3033365	-	B.1.1.7	G	32	C241T,C913T,C3037T,C3267T,C5388A,C5526T,C5986T,C6402T,T6954C,A8848G,C10138T,C12357T,C14408T,C14676T,C15279T,T16176C,C21618G,C21727T,A23063T,C23114T,C23271A,A23403G,C23604A,C23709T,T24506G,G24914C,C27972T,G28048T,A28111G,G28881T,C28977T,G297	20	N:R203M,N:S235F,ORF1a:T1001I,ORF1a:A1708D,ORF1a:T1754I,ORF1a:P2046L,ORF1a:I2230T,ORF1a:T4031I,ORF1b:P314L,ORF8:Q27*,ORF8:R52I,ORF8:Y73C,S:T19R,S:N501Y,S:A570D,S:D614G,S:P681H,S:T716I,S:S982A,S:D1118H	6	0
hCoV-19/Mexico/PUE-InDRE_FB19622_S4513/2021	EPI_ISL_3033366	-	B.1.1.7	GRY	35	C241T,C913T,C2453T,C3037T,C3267T,C5388A,C5986T,T6954C,C9611T,C14408T,C14676T,C15279T,T16176C,C16616T,C21855T,G21974C,A23063T,C23271A,A23403G,C23604A,C23709T,T24506G,G24914C,C25609T,C27972T,G28048T,A28111G,G28280C,A28281T,T28282A,A28295G,G28881A,G28882A,G28883C,C28977T	25	N:D3L,N:N8D,N:R203K,N:G204R,N:S235F,ORF1a:L730F,ORF1a:T1001I,ORF1a:A1708D,ORF1a:I2230T,ORF1a:L3116F,ORF1b:P314L,ORF1b:T1050I,ORF3a:L73F,ORF8:Q27*,ORF8:R52I,ORF8:Y73C,S:S98F,S:D138H,S:N501Y,S:A570D,S:D614G,S:P681H,S:T716I,S:S982A,S:D1118H	6	0
hCoV-19/Mexico/PUE-InDRE_FB19629_S4514/2021	EPI_ISL_3033367	-	B.1.1.7	GRY	33	C241T,C913T,C3037T,C3267T,C5388A,C5526T,C5986T,C6196T,T6954C,A8848G,C14408T,C14676T,C15279T,T16176C,A23063T,C23114T,C23271A,A23403G,C23604A,C23709T,T24506G,G24914C,C27972T,G28048T,A28111G,G28280C,A28281T,T28282A,G28881A,G28882A,G28883C,C28	19	N:D3L,N:R203K,N:G204R,N:S235F,ORF1a:T1001I,ORF1a:A1708D,ORF1a:T1754I,ORF1a:I2230T,ORF1b:P314L,ORF8:Q27*,ORF8:R52I,ORF8:Y73C,S:N501Y,S:A570D,S:D614G,S:P681H,S:T716I,S:S982A,S:D1118H	6	0
hCoV-19/Mexico/PUE-InDRE_FB19630_S4515/2021	EPI_ISL_3033368	-	B.1.1.7	GRY	36	C241T,C913T,C3037T,C3267T,C5388A,C5526T,C5986T,C6196T,T6954C,A8848G,C10138T,C12357T,C14408T,C14676T,C15279T,T16176C,C21727T,A23063T,C23114T,C23271A,A23403G,C23604A,C23709T,T24506G,G24914C,C27972T,G28048T,A28111G,G28280C,A28281T,T28282A,G28881A,G28882A,G28883C,C28977T,G29715T	20	N:D3L,N:R203K,N:G204R,N:S235F,ORF1a:T1001I,ORF1a:A1708D,ORF1a:T1754I,ORF1a:I2230T,ORF1a:T4031I,ORF1b:P314L,ORF8:Q27*,ORF8:R52I,ORF8:Y73C,S:N501Y,S:A570D,S:D614G,S:P681H,S:T716I,S:S982A,S:D1118H	6	0
hCoV-19/Mexico/QUE-InDRE_F11371_S903/2021	EPI_ISL_1423513	-	B.1.1.7	GRY	40	C241T,C913T,C3037T,C3267T,A3337C,C5388A,C5986T,T6954C,G7393T,G8525A,G8600T,C14408T,C14676T,C15279T,T16176C,T17007C,A17615G,G22484T,A23063T,C23271A,A23403G,C23604A,C23709T,C24026T,C24378T,T24506G,G24914C,C25587T,C25624T,C27972T,G28048T,A28111G,G28280C,A28281T,T28282A,G28881A,G28882A,G28883C,C28977T,G29648T	26	N:D3L,N:R203K,N:G204R,N:S235F,ORF1a:T1001I,ORF1a:E1024D,ORF1a:A1708D,ORF1a:I2230T,ORF1a:V2754I,ORF1a:V2779L,ORF1b:P314L,ORF1b:K1383R,ORF3a:H78Y,ORF8:Q27*,ORF8:R52I,ORF8:Y73C,S:V308L,S:N501Y,S:A570D,S:D614G,S:P681H,S:T716I,S:L822F,S:S939F,S:S982A,S:D1118H	6	0
hCoV-19/Mexico/QUE-InDRE_FB12849_S1487/2021	EPI_ISL_1805465	-	B.1.1.7	GRY	32	C241T,C913T,C3037T,C3267T,C5388A,C5986T,C6026T,T6954C,C11750T,C14408T,C14676T,C15279T,T16176C,C18647T,A23063T,C23271A,A23403G,C23604A,C23709T,T24506G,G24914C,C27972T,G28048T,A28111G,G28280C,A28281T,T28282A,G28881A,G28882A,G28883C,C28977T,C	22	N:D3L,N:R203K,N:G204R,N:S235F,N:T393I,ORF1a:T1001I,ORF1a:A1708D,ORF1a:P1921S,ORF1a:I2230T,ORF1a:L3829F,ORF1b:P314L,ORF1b:P1727L,ORF8:Q27*,ORF8:R52I,ORF8:Y73C,S:N501Y,S:A570D,S:D614G,S:P681H,S:T716I,S:S9	6	0
hCoV-19/Mexico/QUE-InDRE_FB14548_S2468/2021	EPI_ISL_2455989	-	B.1.1.7	GRY	38	C241T,C913T,C3037T,C3267T,C5388A,C5986T,G6647A,T6954C,C14408T,C14676T,T15096C,C15279T,T16176C,C17676T,C18183T,C19524T,A21519C,A23063T,C23271A,A23403G,C23604A,C23709T,A24070T,T24506G,G24914C,C25624T,C27972T,G28048T,A28111G,G28280C,A28281T,T28282A,G28881A,G28882A,G28883C,G28884C,C28977T,G29742T	22	N:D3L,N:R203K,N:G204P,N:S235F,ORF1a:T1001I,ORF1a:A1708D,ORF1a:A2128T,ORF1a:I2230T,ORF1b:P314L,ORF1b:R2684S,ORF3a:H78Y,ORF8:Q27*,ORF8:R52I,ORF8:Y73C,S:N501Y,S:A570D,S:D614G,S:P681H,S:T716I,S:Q836H,S:S982A,S:D1118H	6	11
hCoV-19/Mexico/QUE-InDRE_FB14550_S2080/2021	EPI_ISL_2283720	-	B.1.1.7	GRY	38	C241T,C913T,C3037T,C3267T,C5388A,C5986T,G6647A,T6954C,C14408T,C14676T,T15096C,C15279T,T16176C,C17676T,C18183T,C19524T,A21519C,A23063T,C23271A,A23403G,C23604A,C23709T,A24070T,T24506G,G24914C,C25624T,C27972T,G28048T,A28111G,G28280C,A28281T,T28282A,G28881A,G28882A,G28883C,G28884C,C28977T,G29742T	22	N:D3L,N:R203K,N:G204P,N:S235F,ORF1a:T1001I,ORF1a:A1708D,ORF1a:A2128T,ORF1a:I2230T,ORF1b:P314L,ORF1b:R2684S,ORF3a:H78Y,ORF8:Q27*,ORF8:R52I,ORF8:Y73C,S:N501Y,S:A570D,S:D614G,S:P681H,S:T716I,S:Q836H,S:S982A,S:D1118H	6	0
hCoV-19/Mexico/QUE-InDRE_FB15517_S2371/2021	EPI_ISL_2455896	-	Q.3	GRY	39	C241T,C913T,C3037T,C3267T,G5023A,C5388A,C5986T,C6568T,T6954C,A9529G,G10396T,C14408T,C14676T,C15279T,T16176C,A17615G,T19301C,A19302T,G20962A,G22094C,A23063T,C23271A,A23403G,C23604A,C23709T,T24506G,G24914C,A25336C,G26730C,C27972T,G28048T,A28111G,G28280C,A28281T,T28282A,G28881A,G28882A,G28883C,C28977T	25	M:V70L,N:D3L,N:R203K,N:G204R,N:S235F,ORF1a:T1001I,ORF1a:M1586I,ORF1a:A1708D,ORF1a:I2230T,ORF1b:P314L,ORF1b:K1383R,ORF1b:V1945A,ORF1b:D2499N,ORF8:Q27*,ORF8:R52I,ORF8:Y73C,S:D178H,S:N501Y,S:A570D,S:D614G,S:P681H,S:T716I,S:S982A,S:D1118H,S:E1258D	6	27
hCoV-19/Mexico/QUE-InDRE_FB15562_S2372/2021	EPI_ISL_2455897	-	B.1.1.7	GRY	33	C241T,C913T,C2453T,C3037T,C3267T,C5388A,C5986T,T6954C,C9611T,C14408T,C14676T,C15279T,T16176C,C21855T,A23063T,C23271A,A23403G,C23604A,C23709T,T24506G,G24914C,A25336C,C27972T,G28048T,A28111G,G28280C,A28281T,T28282A,A28295G,G28881A,G28882A,G	23	N:D3L,N:N8D,N:R203K,N:G204R,N:S235F,ORF1a:L730F,ORF1a:T1001I,ORF1a:A1708D,ORF1a:I2230T,ORF1a:L3116F,ORF1b:P314L,ORF8:Q27*,ORF8:R52I,ORF8:Y73C,S:S98F,S:N501Y,S:A570D,S:D614G,S:P681H,S:T716I,S:S982A,S:D111	6	150
hCoV-19/Mexico/QUE-InDRE_FB15604_S2373/2021	EPI_ISL_2455898	-	B.1.1.7	GRY	35	C241T,C913T,C3037T,C3267T,A3492G,C5388A,C5986T,T6954C,C14408T,C14676T,C15279T,T16176C,C21639A,A23063T,C23271A,A23403G,C23604A,C23709T,C24374T,T24506G,G24914C,A25164T,A25336C,C26801T,C27972T,G28048T,A28111G,G28280C,A28281T,T28282A,G28881A,G28882A,G28883C,C28977T,C29754T	23	N:D3L,N:R203K,N:G204R,N:S235F,ORF1a:T1001I,ORF1a:N1076S,ORF1a:A1708D,ORF1a:I2230T,ORF1b:P314L,ORF8:Q27*,ORF8:R52I,ORF8:Y73C,S:P26H,S:N501Y,S:A570D,S:D614G,S:P681H,S:T716I,S:L938F,S:S982A,S:D1118H,S:Q1201L,S:E1258D	6	10
hCoV-19/Mexico/QUE-InDRE_FB15641_S2374/2021	EPI_ISL_2455899	-	B.1.1.7	GRY	37	C241T,C913T,C3037T,C3267T,C5388A,C5986T,C6196T,T6954C,A8848G,C9803T,T11135C,C14408T,C14676T,C15279T,T16176C,A23063T,C23114T,C23271A,A23403G,C23604A,C23709T,G24095T,T24506G,G24914C,A25336C,C27972T,G28048T,A28111G,G28280C,A28281T,T28282A,G28881A,G28882A,G28883C,C28977T,G29715T,C29754T	21	N:D3L,N:R203K,N:G204R,N:S235F,ORF1a:T1001I,ORF1a:A1708D,ORF1a:I2230T,ORF1a:F3624L,ORF1b:P314L,ORF8:Q27*,ORF8:R52I,ORF8:Y73C,S:N501Y,S:A570D,S:D614G,S:P681H,S:T716I,S:A845S,S:S982A,S:D1118H,S:E1258D	6	0
hCoV-19/Mexico/QUE-InDRE_FB15653_S2375/2021	EPI_ISL_2455900	-	B.1.1.7	GRY	41	C241T,C913T,C2102T,C3037T,C3267T,C5388A,C5986T,G6647A,T6954C,C12789T,C14408T,C14676T,T15096C,C15279T,T16176C,C17676T,C18183T,C19524T,A21519C,A23063T,C23271A,A23403G,C23604A,C23709T,A24070T,T24506G,G24914C,A25336C,C25624T,C27972T,G28048T,A28111G,G28280C,A28281T,T28282A,G28881A,G28882A,G28883C,G28884C,C28977T,G29742T	24	N:D3L,N:R203K,N:G204P,N:S235F,ORF1a:T1001I,ORF1a:A1708D,ORF1a:A2128T,ORF1a:I2230T,ORF1a:T4175I,ORF1b:P314L,ORF1b:R2684S,ORF3a:H78Y,ORF8:Q27*,ORF8:R52I,ORF8:Y73C,S:N501Y,S:A570D,S:D614G,S:P681H,S:T716I,S:Q836H,S:S982A,S:D1118H,S:E1258D	6	0

hCoV-19/Mexico/QUE-InDRE_FB15659_S2376/2021	EPI_ISL_2455901	-	B.1.1.7	GRY	34	C241T,C913T,C2453T,C3037T,C3267T,C5388A,C5986T,T6954C,C7165T,C9611T,C14408T,C14676T,C15279T,T16176C,C21855T,A23063T,C23271A,A23403G,C23604A,C23709T,T24506G,G24914C,A25336C,C27972T,G28048T,A28111G,G28280C,A28281T,T28282A,A28295G,G28881A,G28	23	N:D3L,N:N8D,N:R203K,N:G204R,N:S235F,ORF1a:L730F,ORF1a:T1001I,ORF1a:A1708D,ORF1a:I2230T,ORF1a:L3116F,ORF1b:P314L,ORF8:Q27*,ORF8:R52I,ORF8:Y73C,S:S98F,S:N501Y,S:A570D,S:D614G,S:P681H,S:T716I,S:S982A,S:D111	6	0
hCoV-19/Mexico/QUE-InDRE_FB15667_S2377/2021	EPI_ISL_2455902	-	B.1.1.7	GRY	38	C241T,C913T,C2453T,C3037T,C3267T,C5388A,C5986T,T6954C,C9611T,C9679T,C14408T,C14676T,C15279T,C15981T,T16176C,C17304T,C21855T,G21974C,A23063T,C23271A,A23403G,C23604A,C23709T,T24506G,G24914C,A25336C,G27754T,C27972T,G28048T,A28111G,G28280C,A28281T,T28282A,A28295G,G28881A,G28882A,G28883C,C28977T	25	N:D3L,N:N8D,N:R203K,N:G204R,N:S235F,ORF1a:L730F,ORF1a:T1001I,ORF1a:A1708D,ORF1a:I2230T,ORF1a:L3116F,ORF1b:P314L,ORF7a:E121*,ORF8:Q27*,ORF8:R52I,ORF8:Y73C,S:S98F,S:D138H,S:N501Y,S:A570D,S:D614G,S:P681H,S:T716I,S:S982A,S:D1118H,S:E1258D	6	26
hCoV-19/Mexico/QUE-InDRE_FB15699_S2378/2021	EPI_ISL_2455903	-	B.1.1.7	GRY	37	C241T,C913T,C2453T,C3037T,C3267T,C5388A,C5986T,T6954C,C9611T,C9679T,C14408T,C14676T,C15279T,C15981T,T16176C,C17304T,C21855T,A23063T,C23271A,A23403G,C23604A,C23709T,T24506G,G24914C,A25336C,G27754T,C27972T,G28048T,A28111G,G28280C,A28281T,T28282A,A28295G,G28881A,G28882A,G28883C,C28977T	24	N:D3L,N:N8D,N:R203K,N:G204R,N:S235F,ORF1a:L730F,ORF1a:T1001I,ORF1a:A1708D,ORF1a:I2230T,ORF1a:L3116F,ORF1b:P314L,ORF7a:E121*,ORF8:Q27*,ORF8:R52I,ORF8:Y73C,S:S98F,S:N501Y,S:A570D,S:D614G,S:P681H,S:T716I,S:S982A,S:D1118H,S:E1258D	6	453
hCoV-19/Mexico/QUE-InDRE_FB15700_S2379/2021	EPI_ISL_2455904	-	B.1.1.7	GRY	39	C241T,C913T,C3037T,C3267T,C5388A,C5986T,G6647A,T6954C,C14408T,C14676T,T15096C,C15279T,T16176C,C17676T,C18183T,C19524T,A21519C,A23063T,C23271A,A23403G,C23604A,C23709T,A24070T,T24506G,G24914C,A25336C,C25624T,C27972T,G28048T,A28111G,G28280C,A28281T,T28282A,G28881A,G28882A,G28883C,G28884C,C28977T,G29742T	23	N:D3L,N:R203K,N:G204P,N:S235F,ORF1a:T1001I,ORF1a:A1708D,ORF1a:A2128T,ORF1a:I2230T,ORF1b:P314L,ORF1b:R2684S,ORF3a:H78Y,ORF8:Q27*,ORF8:R52I,ORF8:Y73C,S:N501Y,S:A570D,S:D614G,S:P681H,S:T716I,S:Q836H,S:S982A,S:D1118H,S:E1258D	6	303
hCoV-19/Mexico/QUE-InDRE_FB15725_S2380/2021	EPI_ISL_2455905	-	B.1.1.7	GRY	38	C241T,C913T,C2110T,C3037T,C3267T,C5388A,C5986T,A6081G,T6954C,T7984C,A9048G,C14120T,C14408T,C14676T,C15279T,T16176C,A19302T,C20629T,A23063T,C23271A,A23403G,C23601T,C23604A,G23678T,C23709T,T24506G,G24914C,A25336C,C27972T,G28048T,A28111G,G28280C,A28281T,T28282A,G28881A,G28882A,G28883C,C28977T	25	N:D3L,N:R203K,N:G204R,N:S235F,ORF1a:T1001I,ORF1a:A1708D,ORF1a:D1939G,ORF1a:I2230T,ORF1a:K2928R,ORF1b:P218L,ORF1b:P314L,ORF1b:H2388Y,ORF8:Q27*,ORF8:R52I,ORF8:Y73C,S:N501Y,S:A570D,S:D614G,S:S680F,S:P681H,S:A706S,S:T716I,S:S982A,S:D1118H,S:E1258D	6	113
hCoV-19/Mexico/QUE-InDRE_FB15757_S2381/2021	EPI_ISL_2455906	-	B.1.1.7	GRY	36	C241T,C913T,C2453T,C3037T,C3267T,T3745C,C5388A,C5986T,T6954C,C9611T,C14408T,C14676T,C15279T,T16176C,C21855T,G21974C,A23063T,C23271A,A23403G,C23604A,C23709T,T24506G,G24914C,A25336C,G26153T,C27972T,G28048T,A28111G,G28280C,A28281T,T28282A,A28295G,G28881A,G28882A,G28883C,C28977T	25	N:D3L,N:N8D,N:R203K,N:G204R,N:S235F,ORF1a:L730F,ORF1a:T1001I,ORF1a:A1708D,ORF1a:I2230T,ORF1a:L3116F,ORF1b:P314L,ORF3a:G254V,ORF8:Q27*,ORF8:R52I,ORF8:Y73C,S:S98F,S:D138H,S:N501Y,S:A570D,S:D614G,S:P681H,S:T716I,S:S982A,S:D1118H,S:E1258D	6	537
hCoV-19/Mexico/QUE-InDRE_FB15774_S2382/2021	EPI_ISL_2455907	-	B.1.1.7	GRY	36	C241T,C913T,C2453T,C3037T,C3267T,C5388A,C5986T,T6954C,C9611T,C14408T,C14676T,C15279T,T16176C,C21008T,C21855T,A23063T,C23271A,A23403G,C23604A,C23709T,T24506G,G24914C,A25271G,A25336C,C27643T,C27972T,G28048T,A28111G,G28280C,A28281T,T28282A,A28295G,G28881A,G28882A,G28883C,C28977T	26	N:D3L,N:N8D,N:R203K,N:G204R,N:S235F,ORF1a:L730F,ORF1a:T1001I,ORF1a:A1708D,ORF1a:I2230T,ORF1a:L3116F,ORF1b:P314L,ORF1b:T2514I,ORF7a:P84S,ORF8:Q27*,ORF8:R52I,ORF8:Y73C,S:S98F,S:N501Y,S:A570D,S:D614G,S:P681H,S:T716I,S:S982A,S:D1118H,S:M1237V,S:E1258D	6	21
hCoV-19/Mexico/QUE-InDRE_FB15777_S2383/2021	EPI_ISL_2455908	-	B.1.1.7	GRY	39	C241T,C913T,C2110T,C2841T,C3037T,C3267T,C5388A,C5986T,T6954C,G7042T,T10123C,C14120T,C14408T,C14676T,C15279T,T16176C,T17583G,A23063T,C23271A,G23401T,A23403G,C23604A,C23709T,T24506G,G24914C,A25336C,C25624T,G25690T,C27972T,G28048T,A28111G,G28280C,A28281T,T28282A,C28846T,G28881A,G28882A,G28883C,C28977T	25	N:D3L,N:R203K,N:G204R,N:S235F,ORF1a:A859V,ORF1a:T1001I,ORF1a:A1708D,ORF1a:I2230T,ORF1a:M2259I,ORF1b:P218L,ORF1b:P314L,ORF3a:H78Y,ORF3a:G100C,ORF8:Q27*,ORF8:R52I,ORF8:Y73C,S:N501Y,S:A570D,S:Q613H,S:D614G,S:P681H,S:T716I,S:S982A,S:D1118H,S:E1258D	6	0
hCoV-19/Mexico/QUE-InDRE_FB15789_S2384/2021	EPI_ISL_2455909	-	B.1.1.7	GRY	34	C241T,C913T,C2453T,C3037T,C3267T,C5388A,C5986T,T6954C,A7479G,C9611T,C14408T,C14676T,C15279T,T16176C,C21855T,A23063T,C23271A,A23403G,C23604A,C23709T,T24506G,G24914C,A25336C,C27972T,G28048T,A28111G,G28280C,A28281T,T28282A,A28295G,G28881A,G28882A,G28883C,C28977T	24	N:D3L,N:N8D,N:R203K,N:G204R,N:S235F,ORF1a:L730F,ORF1a:T1001I,ORF1a:A1708D,ORF1a:I2230T,ORF1a:N2405S,ORF1a:L3116F,ORF1b:P314L,ORF8:Q27*,ORF8:R52I,ORF8:Y73C,S:S98F,S:N501Y,S:A570D,S:D614G,S:P681H,S:T716I,S:S982A,S:D1118H,S:E1258D	6	0
hCoV-19/Mexico/QUE-InDRE_FB15790_S2385/2021	EPI_ISL_2455910	-	B.1.1.7	GRY	34	C241T,C913T,C2453T,C3037T,C3267T,C5388A,C5986T,T6954C,A7479G,C9611T,C14408T,C14676T,C15279T,T16176C,C21855T,A23063T,C23271A,A23403G,C23604A,C23709T,T24506G,G24914C,A25336C,C27972T,G28048T,A28111G,G28280C,A28281T,T28282A,A28295G,G28881A,G28882A,G28883C,C28977T	24	N:D3L,N:N8D,N:R203K,N:G204R,N:S235F,ORF1a:L730F,ORF1a:T1001I,ORF1a:A1708D,ORF1a:I2230T,ORF1a:N2405S,ORF1a:L3116F,ORF1b:P314L,ORF8:Q27*,ORF8:R52I,ORF8:Y73C,S:S98F,S:N501Y,S:A570D,S:D614G,S:P681H,S:T716I,S:S982A,S:D1118H,S:E1258D	6	0
hCoV-19/Mexico/QUE-InDRE_FB15807_S2386/2021	EPI_ISL_2455911	-	B.1.1.7	GRY	36	C241T,C913T,C1191T,C2453T,C3037T,C3267T,C5388A,C5986T,T6954C,A7479G,C9611T,C11339T,C14408T,C14676T,C15279T,T16176C,C21855T,A23063T,C23271A,A23403G,C23604A,C23709T,T24506G,G24914C,A25336C,C27972T,G28048T,A28111G,G28280C,A28281T,T28282A,A28295G,G28881A,G28882A,G28883C,C28977T	25	N:D3L,N:N8D,N:R203K,N:G204R,N:S235F,ORF1a:P309L,ORF1a:L730F,ORF1a:T1001I,ORF1a:A1708D,ORF1a:I2230T,ORF1a:N2405S,ORF1a:L3116F,ORF1b:P314L,ORF8:Q27*,ORF8:R52I,ORF8:Y73C,S:S98F,S:N501Y,S:A570D,S:D614G,S:P681H,S:T716I,S:S982A,S:D1118H,S:E1258D	6	63
hCoV-19/Mexico/QUE-InDRE_FB15808_S2387/2021	EPI_ISL_2455912	-	B.1.1.7	GRY	37	C241T,C913T,C2141A,C3037T,C3267T,C3486T,T4057C,C4782T,C5388A,C5986T,T6954C,G8017T,C11750T,C14408T,C14676T,C15279T,T16176C,C16887T,A23063T,C23271A,A23403G,C23604A,C23709T,T24506G,G24914C,A25336C,C26270T,C27972T,G28048T,A28111G,G28280C,A28281T,T28282A,G28881A,G28882A,G28883C,C28977T	24	E:T9I,N:D3L,N:R203K,N:G204R,N:S235F,ORF1a:P626T,ORF1a:T1001I,ORF1a:A1074V,ORF1a:S1506L,ORF1a:A1708D,ORF1a:I2230T,ORF1a:L3829F,ORF1b:P314L,ORF8:Q27*,ORF8:R52I,ORF8:Y73C,S:N501Y,S:A570D,S:D614G,S:P681H,S:T716I,S:S982A,S:D1118H,S:E1258D	6	54
hCoV-19/Mexico/QUE-InDRE_FB16146_S2439/2021	EPI_ISL_2455963	-	B.1.1.7	GRY	35	C241T,C913T,C2453T,C3037T,C3267T,C5388A,C5986T,T6954C,C7165T,C9611T,C14408T,C14676T,C15279T,T16176C,C21855T,A23063T,C23271A,A23403G,C23604A,C23709T,T24506G,G24914C,A25336C,G27842A,C27972T,G28048T,A28111G,G28280C,A28281T,T28282A,A28295G,G28881A,G28882A,G28883C,C28977T	24	N:D3L,N:N8D,N:R203K,N:G204R,N:S235F,ORF1a:L730F,ORF1a:T1001I,ORF1a:A1708D,ORF1a:I2230T,ORF1a:L3116F,ORF1b:P314L,ORF7b:W29*,ORF8:Q27*,ORF8:R52I,ORF8:Y73C,S:S98F,S:N501Y,S:A570D,S:D614G,S:P681H,S:T716I,S:S982A,S:D1118H,S:E1258D	6	378
hCoV-19/Mexico/QUE-InDRE_FB16154_S2438/2021	EPI_ISL_2455962	-	B.1.1.7	GRY	35	C241T,C913T,C1191T,C2453T,C3037T,C3267T,C5388A,C5986T,T6954C,A7479G,C9611T,C14408T,C14676T,C15279T,T16176C,C21855T,A23063T,C23271A,A23403G,C23604A,C23709T,T24506G,G24914C,A25336C,C27972T,G28048T,A28111G,G28280C,A28281T,T28282A,A28295G,G28881A,G28882A,G28883C,C28977T	25	N:D3L,N:N8D,N:R203K,N:G204R,N:S235F,ORF1a:P309L,ORF1a:L730F,ORF1a:T1001I,ORF1a:A1708D,ORF1a:I2230T,ORF1a:N2405S,ORF1a:L3116F,ORF1b:P314L,ORF8:Q27*,ORF8:R52I,ORF8:Y73C,S:S98F,S:N501Y,S:A570D,S:D614G,S:P681H,S:T716I,S:S982A,S:D1118H,S:E1258D	6	37
hCoV-19/Mexico/QUE-InDRE_FB16156_S2437/2021	EPI_ISL_2455961	-	B.1.1.7	GRY	34	C241T,C913T,C3037T,C3267T,A3492G,C5388A,C5986T,T6954C,C14408T,C14676T,C15279T,T16176C,C20148T,C21639A,A23063T,C23271A,A23403G,C23604A,C23709T,C24374T,T24506G,G24914C,A25164T,A25336C,C27972T,G28048T,A28111G,G28280C,A28281T,T28282A,G28881A,G	23	N:D3L,N:R203K,N:G204R,N:S235F,ORF1a:T1001I,ORF1a:N1076S,ORF1a:A1708D,ORF1a:I2230T,ORF1b:P314L,ORF8:Q27*,ORF8:R52I,ORF8:Y73C,S:P26H,S:N501Y,S:A570D,S:D614G,S:P681H,S:T716I,S:L938F,S:S982A,S:D1118H,S:Q1201	6	0
hCoV-19/Mexico/QUE-InDRE_FB16157_S2436/2021	EPI_ISL_2455960	-	B.1.1.7	GRY	36	C241T,C784T,C913T,C2710T,C3037T,C3267T,A3492G,C5388A,C5986T,T6954C,C14408T,C14676T,C15279T,T16176C,C20148T,C21639A,A23063T,C23271A,A23403G,C23604A,C23709T,C24374T,T24506G,G24914C,A25164T,A25336C,C27972T,G28048T,A28111G,G28280C,A28281T,T28282A,G28881A,G28882A,G28883C,C28977T	23	N:D3L,N:R203K,N:G204R,N:S235F,ORF1a:T1001I,ORF1a:N1076S,ORF1a:A1708D,ORF1a:I2230T,ORF1b:P314L,ORF8:Q27*,ORF8:R52I,ORF8:Y73C,S:P26H,S:N501Y,S:A570D,S:D614G,S:P681H,S:T716I,S:L938F,S:S982A,S:D1118H,S:Q1201L,S:E1258D	6	18
hCoV-19/Mexico/QUE-InDRE_FB16164_S2460/2021	EPI_ISL_2455981	-	B.1.1.7	GRY	35	C241T,C913T,C2453T,C3037T,C3267T,C5388A,C5986T,T6954C,A7479G,C9611T,C14408T,C14676T,C15279T,T16176C,C21855T,G21974C,A23063T,C23271A,A23403G,C23604A,C23709T,T24506G,G24914C,A25336C,C27972T,G28048T,A28111G,G28280C,A28281T,T28282A,A28295G,G28881A,G28882A,G28883C,C28977T	25	N:D3L,N:N8D,N:R203K,N:G204R,N:S235F,ORF1a:L730F,ORF1a:T1001I,ORF1a:A1708D,ORF1a:I2230T,ORF1a:N2405S,ORF1a:L3116F,ORF1b:P314L,ORF8:Q27*,ORF8:R52I,ORF8:Y73C,S:S98F,S:D138H,S:N501Y,S:A570D,S:D614G,S:P681H,S:T716I,S:S982A,S:D1118H,S:E1258D	6	0
hCoV-19/Mexico/QUE-InDRE_FB16431_S2481/2021	EPI_ISL_2456002	-	B.1.1.7	GRY	40	C241T,C913T,G1264A,C2110T,C3037T,C3267T,C5388A,C5986T,T6954C,T7984C,T9508C,A12964G,C14120T,C14408T,C14676T,C15279T,T16176C,C16323T,C16575T,C17004T,C17518T,C19390T,A23063T,C23271A,A23403G,C23604A,C23709T,T24506G,G24914C,C27972T,G28048T,A28111G,C28146T,G28280C,A28281T,T28282A,G28881A,G28882A,G28883C,C28977T	22	N:D3L,N:R203K,N:G204R,N:S235F,ORF1a:T1001I,ORF1a:A1708D,ORF1a:I2230T,ORF1b:P218L,ORF1b:P314L,ORF1b:L1351F,ORF1b:P1975S,ORF8:Q27*,ORF8:R52I,ORF8:Y73C,ORF8:P85S,S:N501Y,S:A570D,S:D614G,S:P681H,S:T716I,S:S982A,S:D1118H	6	0
hCoV-19/Mexico/QUE-InDRE_FB16434_S2738/2021	EPI_ISL_2533764	-	B.1.1.7	GRY	41	C241T,C913T,C3037T,C3267T,C5388A,C5986T,G6647A,T6954C,C14408T,C14621T,C14676T,T15096C,C15279T,T16176C,C17676T,C18183T,C19524T,A21519C,A23063T,C23271A,A23403G,C23604A,C23709T,A24070T,A24092G,T24506G,G24914C,A25336C,C25624T,C27737T,C27972T,G28048T,A28111G,G28280C,A28281T,T28282A,G28881A,G28882A,G28883C,G28884C,C28977T	26	N:D3L,N:R203K,N:G204P,N:S235F,ORF1a:T1001I,ORF1a:A1708D,ORF1a:A2128T,ORF1a:I2230T,ORF1b:P314L,ORF1b:T385M,ORF1b:R2684S,ORF3a:H78Y,ORF7a:T115I,ORF8:Q27*,ORF8:R52I,ORF8:Y73C,S:N501Y,S:A570D,S:D614G,S:P681H,S:T716I,S:Q836H,S:I844V,S:S982A,S:D1118H,S:E1258D	6	0

hCoV-19/Mexico/QUE-InDRE_FB16436_S2739/2021	EPI_ISL_2533765	-	B.1.1.7	GRY	43	C241T,T345C,C913T,C2453T,C3037T,C3267T,C4890T,C5388A,C5986T,T6954C,C7165T,C9611T,C10341T,C12459T,C14408T,C14676T,C15279T,T16176C,C21597T,C21855T,G21974C,C22716A,T22717C,A23063T,C23271A,A23403G,C23604A,C23709T,T24506G,G24914C,C25413T,C27972T,G28048T,A28111G,G28280C,A28281T,T28282A,A28295G,G28881A,G28882A,G28883C,C2897	29	N:D3L,N:N8D,N:R203K,N:G204R,N:S235F,ORF1a:L27P,ORF1a:L730F,ORF1a:T1001I,ORF1a:T1542I,ORF1a:A1708D,ORF1a:I2230T,ORF1a:L3116F,ORF1a:P3359L,ORF1a:T4065I,ORF1b:P314L,ORF8:Q27*,ORF8:R52I,ORF8:Y73C,S:S12F,S:S98F,S:D138H,S:T385N,S:N501Y,S:A570D,S:D614G,S:P681H,S:T716I,S:S982A,S:D	6	0
hCoV-19/Mexico/QUE-InDRE_FB16437_S2740/2021	EPI_ISL_2533766	-	B.1.1.7	GRY	36	C241T,C913T,C2453T,C3037T,C3267T,C5388A,C5986T,T6954C,C7165T,C9611T,C14408T,C14676T,C15279T,T16176C,G17478A,C21855T,G21974C,A23063T,C23271A,A23403G,C23604A,C23709T,T24506G,G24914C,G25494T,C27972T,G28048T,A28111G,G28280C,A28281T,T28282A,A28295G,G28881A,G28882A,G28883C,C28977T	23	N:D3L,N:N8D,N:R203K,N:G204R,N:S235F,ORF1a:L730F,ORF1a:T1001I,ORF1a:A1708D,ORF1a:I2230T,ORF1a:L3116F,ORF1b:P314L,ORF8:Q27*,ORF8:R52I,ORF8:Y73C,S:S98F,S:D138H,S:N501Y,S:A570D,S:D614G,S:P681H,S:T716I,S:S982A,S:D1118H	6	0
hCoV-19/Mexico/QUE-InDRE_FB16868_S3113/2021	EPI_ISL_2663380	-	B.1.1.7	GRY	38	C241T,C753T,C913T,C2453T,C3037T,C3267T,C3695T,C5388A,C5986T,T6954C,C9611T,C14408T,C14676T,C15279T,T16176C,C21008T,C21855T,G21974C,A23063T,C23271A,A23403G,C23604A,C23709T,T24506G,G24914C,C27643T,C27972T,G28048T,A28111G,G28280C,A28281T,T28282A,A28295G,C28831T,G28881A,G28882A,G28883C,C28977T	26	N:D3L,N:N8D,N:R203K,N:G204R,N:S235F,ORF1a:T163I,ORF1a:L730F,ORF1a:T1001I,ORF1a:A1708D,ORF1a:I2230T,ORF1a:L3116F,ORF1b:P314L,ORF1b:T2514I,ORF7a:P84S,ORF8:Q27*,ORF8:R52I,ORF8:Y73C,S:S98F,S:D138H,S:N501Y,S:A570D,S:D614G,S:P681H,S:T716I,S:S982A,S:D1118H	6	0
hCoV-19/Mexico/QUE-InDRE_FB16876_S3114/2021	EPI_ISL_2663381	-	B.1.1.7	GRY	36	C241T,C913T,C2453T,C3037T,C3267T,C3784T,C5388A,C5986T,G6352T,T6954C,A7479G,C9611T,C14408T,C14676T,C15279T,T16176C,C21855T,G21974C,A23063T,C23271A,A23403G,C23604A,C23709T,T24506G,G24914C,C27972T,G28048T,A28111G,G28280C,A28281T,T28282A,A28295G,G28881A,G28882A,G28883C,C28977T	25	N:D3L,N:N8D,N:R203K,N:G204R,N:S235F,ORF1a:L730F,ORF1a:T1001I,ORF1a:A1708D,ORF1a:K2029N,ORF1a:I2230T,ORF1a:N2405S,ORF1a:L3116F,ORF1b:P314L,ORF8:Q27*,ORF8:R52I,ORF8:Y73C,S:S98F,S:D138H,S:N501Y,S:A570D,S:D614G,S:P681H,S:T716I,S:S982A,S:D1118H	6	0
hCoV-19/Mexico/QUE-InDRE_FB16879_S3064/2021	EPI_ISL_2709686	-	B.1.1.7	G	40	C241T,C913T,C1513T,C2627T,C3037T,C3267T,C3583T,C5388A,C5986T,C6312T,T6954C,G11138T,G11401T,C12469T,C14408T,C14676T,T15096C,C15279T,T16176C,C17491T,C19862T,A21404G,A23063T,C23271A,A23403G,C23604A,C23709T,C24382T,T24506G,G24914C,A25336C,G25337C,C27972T,G28048T,A28111G,G28280C,A28281T,T28282A,C28977T,C29546A	24	N:D3L,ORF1a:L788F,ORF1a:T1001I,ORF1a:A1708D,ORF1a:T2016I,ORF1a:I2230T,ORF1a:A3625S,ORF1a:M3712I,ORF1b:P314L,ORF1b:P1342S,ORF1b:A2132V,ORF1b:K2646R,ORF8:Q27*,ORF8:R52I,ORF8:Y73C,S:N501Y,S:A570D,S:D614G,S:P681H,S:T716I,S:S982A,S:D1118H,S:E1258D,S:D1259H	7	37
hCoV-19/Mexico/QUE-InDRE_FB16881_S3065/2021	EPI_ISL_2663331	-	B.1.1.7	GRY	43	C241T,C913T,C1513T,C2627T,C3037T,C3267T,C3583T,C5388A,C5986T,C6312T,T6954C,A7531G,C10279T,G11138T,G11401T,C14408T,C14676T,T15096C,C15279T,T16176C,C17491T,C19862T,A23063T,C23271A,A23403G,C23604A,C23709T,T24506G,G24914C,A25336C,G25337C,C27972T,G28048T,A28111G,G28280C,A28281T,T28282A,G28881A,G28882A,G28883C,G28884C,C289	26	N:D3L,N:R203K,N:G204P,N:S235F,ORF1a:L788F,ORF1a:T1001I,ORF1a:A1708D,ORF1a:T2016I,ORF1a:I2230T,ORF1a:A3625S,ORF1a:M3712I,ORF1b:P314L,ORF1b:P1342S,ORF1b:A2132V,ORF8:Q27*,ORF8:R52I,ORF8:Y73C,S:N501Y,S:A570D,S:D614G,S:P681H,S:T716I,S:S982A,S:D1118H,S:E1258D,S:D1259H	6	0
hCoV-19/Mexico/QUE-InDRE_FB16884_S3066/2021	EPI_ISL_2663332	-	B.1.1.7	GRY	43	C241T,C913T,C1513T,C2627T,C3037T,C3267T,C3583T,C5388A,C5986T,C6312T,T6954C,G11138T,G11401T,C12469T,C14408T,C14676T,T15096C,C15279T,T16176C,C17491T,C19862T,A21404G,A23063T,C23271A,A23403G,C23604A,C23709T,C24382T,T24506G,G24914C,A25336C,C27972T,G28048T,A28111G,G28280C,A28281T,T28282A,A28877T,G28878C,G28881A,G28882A,G28	25	N:D3L,N:R203K,N:G204R,ORF1a:L788F,ORF1a:T1001I,ORF1a:A1708D,ORF1a:T2016I,ORF1a:I2230T,ORF1a:A3625S,ORF1a:M3712I,ORF1b:P314L,ORF1b:P1342S,ORF1b:A2132V,ORF1b:K2646R,ORF8:Q27*,ORF8:R52I,ORF8:Y73C,S:N501Y,S:A570D,S:D614G,S:P681H,S:T716I,S:S982A,S:D1118H,S:E1258D	6	541
hCoV-19/Mexico/QUE-InDRE_FB17253_S3534/2021	EPI_ISL_2779049	-	B.1.1.7	GRY	44	C241T,C913T,C1513T,C2627T,C3037T,C3267T,C3583T,C5388A,C5944T,C5986T,C6312T,T6954C,G11138T,G11401T,C12469T,C14408T,C14676T,T15096C,C15279T,T16176C,C17491T,C19862T,A21404G,A23063T,C23271A,A23403G,C23604A,C23709T,C24382T,T24506G,G24914C,C27972T,G28048T,A28095T,A28111G,G28280C,A28281T,T28282A,G28881A,G28882A,G28883C,G288	26	N:D3L,N:R203K,N:G204P,N:S235F,ORF1a:L788F,ORF1a:T1001I,ORF1a:A1708D,ORF1a:T2016I,ORF1a:I2230T,ORF1a:A3625S,ORF1a:M3712I,ORF1b:P314L,ORF1b:P1342S,ORF1b:A2132V,ORF1b:K2646R,ORF8:Q27*,ORF8:R52I,ORF8:K68*,ORF8:Y73C,S:N501Y,S:A570D,S:D614G,S:P681H,S:T716I,S:S982A,S:D1118H	6	402
hCoV-19/Mexico/QUE-InDRE_FB17258_S3109/2021	EPI_ISL_2663376	-	B.1.1.7	GRY	41	C241T,C913T,C3037T,C3267T,C4540T,C5388A,C5944T,C5986T,T6954C,C8950T,C9967T,C13585T,C14408T,C14676T,T15096C,C15279T,T16176C,G18412T,C19374T,T22565C,C22987T,A23063T,C23271A,A23403G,C23604A,C23709T,T24506G,G24914C,G27033T,C27972T,G28048T,A28095T,A28111G,G28280C,A28281T,T28282A,G28881A,G28882A,G28883C,G28884C,C28977T	21	M:A171S,N:D3L,N:R203K,N:G204P,N:S235F,ORF1a:T1001I,ORF1a:A1708D,ORF1a:I2230T,ORF1b:P314L,ORF1b:V1649F,ORF8:Q27*,ORF8:R52I,ORF8:K68*,ORF8:Y73C,S:N501Y,S:A570D,S:D614G,S:P681H,S:T716I,S:S982A,S:D1118H	6	0
hCoV-19/Mexico/QUE-InDRE_FB17259_S3110/2021	EPI_ISL_2663377	-	B.1.1.7	GRY	40	C241T,T345C,C913T,C2453T,C3037T,C3267T,C5388A,C5986T,T6954C,C7165T,C9611T,C12459T,C14408T,C14676T,C15279T,T16176C,C21855T,G21974C,C22716A,T22717C,A23063T,C23271A,A23403G,C23604A,C23709T,T24506G,G24914C,C25413T,G26849T,C27972T,G28048T,A28111G,G28280C,A28281T,T28282A,A28295G,G28881A,G28882A,G28883C,C28977T	27	M:M109I,N:D3L,N:N8D,N:R203K,N:G204R,N:S235F,ORF1a:L27P,ORF1a:L730F,ORF1a:T1001I,ORF1a:A1708D,ORF1a:I2230T,ORF1a:L3116F,ORF1a:T4065I,ORF1b:P314L,ORF8:Q27*,ORF8:R52I,ORF8:Y73C,S:S98F,S:D138H,S:T385N,S:N501Y,S:A570D,S:D614G,S:P681H,S:T716I,S:S982A,S:D1118H	6	0
hCoV-19/Mexico/QUE-InDRE_FB18359_S4142/2021	EPI_ISL_2920713	-	B.1.1.7	GRY	43	C241T,C913T,C3037T,C3267T,C5388A,C5944T,C5986T,C6401T,G6647A,T6954C,C14408T,C14676T,T15096C,C15279T,T16176C,C17676T,C18183T,C19524T,A21519C,A23063T,C23271A,A23403G,C23604A,C23709T,A24070T,T24506G,G24914C,G25595T,C25624T,G27506T,C27972T,G28048T,A28095T,A28111G,G28280C,A28281T,T28282A,G28881A,G28882A,G28883C,G28884C,C2	26	N:D3L,N:R203K,N:G204P,N:S235F,ORF1a:T1001I,ORF1a:A1708D,ORF1a:P2046S,ORF1a:A2128T,ORF1a:I2230T,ORF1b:P314L,ORF1b:R2684S,ORF3a:R68I,ORF3a:H78Y,ORF7a:G38V,ORF8:Q27*,ORF8:R52I,ORF8:K68*,ORF8:Y73C,S:N501Y,S:A570D,S:D614G,S:P681H,S:T716I,S:Q836H,S:S982A,S:D1118H	6	0
hCoV-19/Mexico/ROO-InDRE_FB12917_S1492/2021	EPI_ISL_1805470	-	B.1.1.7	GRY	37	C241T,C913T,C2110T,G3004T,C3037T,C3267T,C5388A,C5986T,T6954C,G7042T,C10789T,C14120T,C14408T,C14676T,C15279T,T16176C,A23063T,C23271A,A23403G,C23604A,C23709T,T24506G,G24914C,C26110T,C26735T,C27972T,G28048T,A28095T,A28111G,G28280C,A28281T,T28282A,A28804G,G28881A,G28882A,G28883C,C28977T	23	N:D3L,N:R203K,N:G204R,N:S235F,ORF1a:E913D,ORF1a:T1001I,ORF1a:A1708D,ORF1a:I2230T,ORF1a:M2259I,ORF1b:P218L,ORF1b:P314L,ORF3a:P240S,ORF8:Q27*,ORF8:R52I,ORF8:K68*,ORF8:Y73C,S:N501Y,S:A570D,S:D614G,S:P681H,S:T716I,S:S982A,S:D1118H	6	0
hCoV-19/Mexico/ROO-InDRE_FB13273_S1503/2021	EPI_ISL_1805481	-	B.1.1.7	GRY	35	T78G,C241T,C913T,C2453T,C3037T,C3267T,C5388A,C5986T,T6954C,C9611T,C14408T,C14676T,C15279T,T16176C,G17019T,C21855T,G21974C,A23063T,C23271A,A23403G,C23604A,C23709T,T24506G,G24914C,C27972T,G28048T,A28111G,G28280C,A28281T,T28282A,A28295G,G28881A,G28882A,G28883C,C28977T	24	N:D3L,N:N8D,N:R203K,N:G204R,N:S235F,ORF1a:L730F,ORF1a:T1001I,ORF1a:A1708D,ORF1a:I2230T,ORF1a:L3116F,ORF1b:P314L,ORF1b:E1184D,ORF8:Q27*,ORF8:R52I,ORF8:Y73C,S:S98F,S:D138H,S:N501Y,S:A570D,S:D614G,S:P681H,S:T716I,S:S982A,S:D1118H	6	854
hCoV-19/Mexico/ROO-InDRE_FB13727_S1740/2021	EPI_ISL_2101879	-	B.1.1.7	GRY	37	C241T,C913T,G2144A,C2453T,C3037T,C3267T,C5388A,C5986T,T6954C,C9611T,C14408T,C14676T,C15279T,T16176C,G18651T,C21855T,G21974C,A23063T,C23271A,A23403G,C23604A,C23709T,T24506G,G24914C,A25336C,C27972T,G28048T,A28111G,G28280C,A28281T,T28282A,A28295G,G28881A,G28882A,G28883C,C28977T,G29745T	26	N:D3L,N:N8D,N:R203K,N:G204R,N:S235F,ORF1a:V627I,ORF1a:L730F,ORF1a:T1001I,ORF1a:A1708D,ORF1a:I2230T,ORF1a:L3116F,ORF1b:P314L,ORF1b:E1728D,ORF8:Q27*,ORF8:R52I,ORF8:Y73C,S:S98F,S:D138H,S:N501Y,S:A570D,S:D614G,S:P681H,S:T716I,S:S982A,S:D1118H,S:E1258D	6	82
hCoV-19/Mexico/ROO-InDRE_FB14100_S1759/2021	EPI_ISL_2101898	-	B.1.1.7	GRY	33	C241T,C913T,C2453T,C3037T,C3267T,C5388A,C5986T,T6954C,C9611T,C14408T,C14676T,C15279T,T16176C,C21855T,G21974C,A23063T,C23271A,A23403G,C23604A,C23709T,T24506G,G24914C,C27972T,G28048T,A28111G,G28280C,A28281T,T28282A,A28295G,G28881A,G28882A,G	23	N:D3L,N:N8D,N:R203K,N:G204R,N:S235F,ORF1a:L730F,ORF1a:T1001I,ORF1a:A1708D,ORF1a:I2230T,ORF1a:L3116F,ORF1b:P314L,ORF8:Q27*,ORF8:R52I,ORF8:Y73C,S:S98F,S:D138H,S:N501Y,S:A570D,S:D614G,S:P681H,S:T716I,S:S982	6	0
hCoV-19/Mexico/ROO-InDRE_FB14101_S1760/2021	EPI_ISL_2101899	-	B.1.1.7	GRY	33	C241T,C913T,C2453T,C3037T,C3267T,C5388A,C5986T,T6954C,C9611T,C14408T,C14676T,C15279T,T16176C,C21855T,G21974C,A23063T,C23271A,A23403G,C23604A,C23709T,T24506G,G24914C,C27972T,G28048T,A28111G,G28280C,A28281T,T28282A,A28295G,G28881A,G28882A,G	23	N:D3L,N:N8D,N:R203K,N:G204R,N:S235F,ORF1a:L730F,ORF1a:T1001I,ORF1a:A1708D,ORF1a:I2230T,ORF1a:L3116F,ORF1b:P314L,ORF8:Q27*,ORF8:R52I,ORF8:Y73C,S:S98F,S:D138H,S:N501Y,S:A570D,S:D614G,S:P681H,S:T716I,S:S982	6	0
hCoV-19/Mexico/ROO-InDRE_FB16369_S2476/2021	EPI_ISL_2455997	-	B.1.1.7	GRY	44	C241T,C913T,C3037T,C3267T,C3966T,C4206T,C5388A,C5986T,T6954C,A12162G,C14408T,C14676T,C15279T,G15594T,C15720T,T16176C,A17615G,G18498T,A21137G,G22801T,A23063T,C23271A,A23403G,C23604A,C23709T,T24506G,G24914C,G25135T,G25352T,C26110T,A26530G,C27236T,C27972T,G28048T,G28079T,A28111G,G28280C,A28281T,T28282A,G28881A,G28882A,G28883C,C28977T,G29227T	30	M:D3G,N:D3L,N:R203K,N:G204R,N:S235F,ORF1a:T1001I,ORF1a:A1234V,ORF1a:A1314V,ORF1a:A1708D,ORF1a:I2230T,ORF1a:Q3966R,ORF1b:P314L,ORF1b:K709N,ORF1b:K1383R,ORF1b:M1677I,ORF1b:K2557R,ORF3a:P240S,ORF6:A12V,ORF8:Q27*,ORF8:R52I,ORF8:Y73C,S:N501Y,S:A570D,S:D614G,S:P681H,S:T716I,S:S982A,S:D1118H,S:K1191N,S:V1264L	6	14
hCoV-19/Mexico/ROO-InDRE_FB16793_S2778/2021	EPI_ISL_2533804	-	B.1.1.7	GRY	39	C241T,C913T,C3037T,C3267T,C4752T,C5388A,C5986T,T6954C,C9693T,C11572T,C14408T,C14676T,C15279T,T16176C,G18651A,C18788T,T23042C,A23063T,C23271A,A23403G,C23604A,C23709T,T24506G,G24914C,G25218T,C25553T,G25684A,C27615T,C27972T,G28048T,A28095T,A28111G,G28280C,A28281T,T28282A,G28881A,G28882A,G28883C,C28977T	26	N:D3L,N:R203K,N:G204R,N:S235F,ORF1a:T1001I,ORF1a:T1496I,ORF1a:A1708D,ORF1a:I2230T,ORF1a:A3143V,ORF1b:P314L,ORF1b:T1774I,ORF3a:A54V,ORF3a:A98T,ORF8:Q27*,ORF8:R52I,ORF8:K68*,ORF8:Y73C,S:S494P,S:N501Y,S:A570D,S:D614G,S:P681H,S:T716I,S:S982A,S:D1118H,S:G1219V	6	0
hCoV-19/Mexico/ROO-InDRE_FB16795_S2779/2021	EPI_ISL_2533805	-	B.1.1.7	GRY	36	C241T,C673T,C913T,C2453T,C3037T,C3267T,C5388A,C5986T,T6954C,C9611T,C9893T,C14408T,C14676T,C15279T,T16176C,G17027T,C21855T,G21974C,A23063T,C23271A,A23403G,C23604A,C23709T,T24506G,G24914C,C27972T,G28048T,A28111G,G28280C,A28281T,T28282A,A28295G,G28881A,G28882A,G28883C,C28977T	25	N:D3L,N:N8D,N:R203K,N:G204R,N:S235F,ORF1a:L730F,ORF1a:T1001I,ORF1a:A1708D,ORF1a:I2230T,ORF1a:L3116F,ORF1a:L3210F,ORF1b:P314L,ORF1b:S1187I,ORF8:Q27*,ORF8:R52I,ORF8:Y73C,S:S98F,S:D138H,S:N501Y,S:A570D,S:D614G,S:P681H,S:T716I,S:S982A,S:D1118H	6	0

hCoV-19/Mexico/ROO-InDRE_FB17190_S3089/2021	EPI_ISL_2663356	-	B.1.1.7	GRY	39	C222T,C241T,C913T,C2110T,C3037T,C3267T,C4423T,C5388A,C5986T,C6681T,T6954C,G7042T,A10359G,C14120T,C14408T,C14676T,C15279T,T16176C,C17762T,A23063T,C23271A,A23403G,C23604A,G23608T,C23709T,T24506G,G24914C,T25180C,C27972T,G28048T,A28095T,A28111G,G28280C,A28281T,T28282A,G28881A,G28882A,G28883C,C28977T	24	N:D3L,N:R203K,N:G204R,N:S235F,ORF1a:T1001I,ORF1a:A1708D,ORF1a:A2139V,ORF1a:I2230T,ORF1a:M2259I,ORF1a:K3365R,ORF1b:P218L,ORF1b:P314L,ORF1b:A1432V,ORF8:Q27*,ORF8:R52I,ORF8:K68*,ORF8:Y73C,S:N501Y,S:A570D,S:D614G,S:P681H,S:T716I,S:S982A,S:D1118H	6	0
hCoV-19/Mexico/ROO-InDRE_FB17192_S3090/2021	EPI_ISL_2663357	-	B.1.1.7	GRY	34	C241T,T287A,C913T,G2144A,C2453T,C3037T,C3267T,C4534T,C5388A,C5986T,T6954C,C9611T,C14408T,C14676T,C15279T,T16176C,G18651T,C21855T,G21974C,A23063T,C23271A,A23403G,C23604A,C23709T,T24506G,G24914C,C27972T,G28048T,A28111G,A28295G,G28881A,G28882A,G28883C,C28977T	25	N:N8D,N:R203K,N:G204R,N:S235F,ORF1a:F8I,ORF1a:V627I,ORF1a:L730F,ORF1a:T1001I,ORF1a:A1708D,ORF1a:I2230T,ORF1a:L3116F,ORF1b:P314L,ORF1b:E1728D,ORF8:Q27*,ORF8:R52I,ORF8:Y73C,S:S98F,S:D138H,S:N501Y,S:A570D,S:D614G,S:P681H,S:T716I,S:S982A,S:D1118H	6	0
hCoV-19/Mexico/ROO-InDRE_FB19165_S4499/2021	EPI_ISL_3033353	-	B.1.1.7	GRY	39	C241T,T287A,C913T,G2144A,C2453T,C3037T,C3267T,C4534T,C5388A,C5986T,T6954C,C9611T,C14408T,C14676T,C15279T,T16176C,G18651T,C21812T,C21855T,G21974C,A23063T,C23271A,A23403G,C23604A,C23709T,T24506G,G24914C,A26206G,C27972T,G28048T,A28111G,G28280C,A28281T,T28282A,A28295G,G28881A,G28882A,G28883C,C28977T	27	N:D3L,N:N8D,N:R203K,N:G204R,N:S235F,ORF1a:F8I,ORF1a:V627I,ORF1a:L730F,ORF1a:T1001I,ORF1a:A1708D,ORF1a:I2230T,ORF1a:L3116F,ORF1b:P314L,ORF1b:E1728D,ORF3a:S272G,ORF8:Q27*,ORF8:R52I,ORF8:Y73C,S:S98F,S:D138H,S:N501Y,S:A570D,S:D614G,S:P681H,S:T716I,S:S982A,S:D1118H	6	0
hCoV-19/Mexico/ROO-INMEGEN-05-06-205/2021	EPI_ISL_2603694	-	B.1.1.7	GR	38	C241T,T597C,C913T,C1471T,G2144A,C2453T,C3037T,C3267T,C5388A,C5986T,T6954C,C9286T,C9611T,A12547G,C14408T,C14676T,C15279T,T16176C,G18651T,C21855T,G21974C,C23271A,A23403G,C23604A,C23709T,T24506G,G24914C,C27972T,G28048T,A28111G,G28280C,A28281T,T28282A,A28295G,G28881A,G28882A,G28883C,C28977T	25	N:D3L,N:N8D,N:R203K,N:G204R,N:S235F,ORF1a:V111A,ORF1a:V627I,ORF1a:L730F,ORF1a:T1001I,ORF1a:A1708D,ORF1a:I2230T,ORF1a:L3116F,ORF1b:P314L,ORF1b:E1728D,ORF8:Q27*,ORF8:R52I,ORF8:Y73C,S:S98F,S:D138H,S:A570D,S:D614G,S:P681H,S:T716I,S:S982A,S:D1118H	6	329
hCoV-19/Mexico/ROO-INMEGEN-05-06-212/2021	EPI_ISL_2603700	-	B.1.1.7	GR	37	C241T,C823T,C913T,C1758T,C2110T,G3004T,C3037T,C3267T,C5388A,C5986T,T6954C,G7042T,C9559T,C14120T,C14408T,C14676T,C15279T,T16176C,C23271A,A23403G,C23604A,C23709T,C24208T,T24506G,G24914C,C26110T,C27972T,G28048T,A28095T,A28111G,G28280C,A28281T,T28282A,G28881A,G28882A,G28883C,C28977T	23	N:D3L,N:R203K,N:G204R,N:S235F,ORF1a:A498V,ORF1a:E913D,ORF1a:T1001I,ORF1a:A1708D,ORF1a:I2230T,ORF1a:M2259I,ORF1b:P218L,ORF1b:P314L,ORF3a:P240S,ORF8:Q27*,ORF8:R52I,ORF8:K68*,ORF8:Y73C,S:A570D,S:D614G,S:P681H,S:T716I,S:S982A,S:D1118H	6	1569
hCoV-19/Mexico/ROO-INMEGEN-05-06-268/2021	EPI_ISL_2603738	-	B.1.1.7	GR	40	C241T,C913T,C2110T,G3004T,C3037T,C3267T,C5388A,C5986T,T6954C,G7042T,C7072T,C10525T,C10789T,C14120T,C14408T,C14676T,C15279T,T16176C,C23271A,A23403G,G23593T,C23604A,C23709T,T24506G,G24914C,C26110T,C26735T,G27281T,C27972T,G28048T,A28095T,A28111G,G28280C,A28281T,T28282A,A28804G,G28881A,G28882A,G28883C,C28977T	24	N:D3L,N:R203K,N:G204R,N:S235F,ORF1a:E913D,ORF1a:T1001I,ORF1a:A1708D,ORF1a:I2230T,ORF1a:M2259I,ORF1b:P218L,ORF1b:P314L,ORF3a:P240S,ORF6:W27L,ORF8:Q27*,ORF8:R52I,ORF8:K68*,ORF8:Y73C,S:A570D,S:D614G,S:Q677H,S:P681H,S:T716I,S:S982A,S:D1118H	6	413
hCoV-19/Mexico/ROO-INMEGEN-06-01-197/2021	EPI_ISL_2616800	-	B.1.1.7	GR	40	C222T,C241T,C913T,C2110T,C3037T,C3267T,C5388A,C5986T,T6954C,G7042T,C8389T,C9979T,G11029T,C12115T,C14120T,C14408T,C14676T,C15279T,T16176C,G16900T,C17762T,C23271A,A23403G,C23604A,C23709T,T24506G,G24914C,T25180C,G26416T,G27664T,C27972T,G28048T,A28111G,G28280C,A28281T,T28282A,G28881A,G28882A,G28883C,C28977T	24	E:V58F,N:D3L,N:R203K,N:G204R,N:S235F,ORF1a:T1001I,ORF1a:A1708D,ORF1a:I2230T,ORF1a:M2259I,ORF1a:L3588F,ORF1b:P218L,ORF1b:P314L,ORF1b:G1145C,ORF1b:A1432V,ORF7a:E91*,ORF8:Q27*,ORF8:R52I,ORF8:Y73C,S:A570D,S:D614G,S:P681H,S:T716I,S:S982A,S:D1118H	6	1234
hCoV-19/Mexico/ROO-INMEGEN-06-01-205/2021	EPI_ISL_2616938	-	B.1.1.7	GRY	39	T99C,C241T,C913T,C3037T,C3267T,C5284T,C5388A,C5986T,T6954C,A9238G,C10369T,C14408T,C14676T,C15279T,T16176C,C17004T,A17615G,G20056A,A23063T,C23271A,A23403G,C23604A,C23709T,T24506G,G24914C,C25413T,G26062T,G26065T,C27972T,G28048T,A28111G,G28280C,A28281T,T28282A,G28881A,G28882A,G28883C,C28977T,G29777T	22	N:D3L,N:R203K,N:G204R,N:S235F,ORF1a:T1001I,ORF1a:A1708D,ORF1a:I2230T,ORF1b:P314L,ORF1b:K1383R,ORF1b:G2197S,ORF3a:G224C,ORF3a:V225F,ORF8:Q27*,ORF8:R52I,ORF8:Y73C,S:N501Y,S:A570D,S:D614G,S:P681H,S:T716I,S:S982A,S:D1118H	6	410
hCoV-19/Mexico/ROO-INMEGEN-06-01-212/2021	EPI_ISL_2616851	-	B.1.1.7	GRY	40	C222T,C241T,C913T,C2110T,C3037T,C3267T,C4423T,C5388A,C5986T,C6681T,T6954C,G7042T,A10359G,C14120T,C14408T,C14676T,C15279T,C16173T,T16176C,C17762T,A23063T,C23271A,A23403G,C23604A,G23608T,C23709T,T24506G,G24914C,T25180C,C27972T,G28048T,A28095T,A28111G,G28280C,A28281T,T28282A,G28881A,G28882A,G28883C,C28977T	24	N:D3L,N:R203K,N:G204R,N:S235F,ORF1a:T1001I,ORF1a:A1708D,ORF1a:A2139V,ORF1a:I2230T,ORF1a:M2259I,ORF1a:K3365R,ORF1b:P218L,ORF1b:P314L,ORF1b:A1432V,ORF8:Q27*,ORF8:R52I,ORF8:K68*,ORF8:Y73C,S:N501Y,S:A570D,S:D614G,S:P681H,S:T716I,S:S982A,S:D1118H	6	873
hCoV-19/Mexico/ROO-INMEGEN-06-01-220/2021	EPI_ISL_2616979	-	Q.3	GR	38	C241T,C913T,C3037T,G3179A,C3267T,G5023A,C5388A,C5986T,C6568T,T6954C,G8183A,A9529G,G10396T,C14408T,C14676T,C15279T,T16176C,A17615G,G17721T,G20962A,G22094C,C23271A,A23403G,C23604A,C23709T,T24506G,G24914C,G26730C,C27972T,G28048T,A28111G,G28280C,A28281T,T28282A,G28881A,G28882A,G28883C,C28977T	24	M:V70L,N:D3L,N:R203K,N:G204R,N:S235F,ORF1a:E972K,ORF1a:T1001I,ORF1a:M1586I,ORF1a:A1708D,ORF1a:I2230T,ORF1a:G2640R,ORF1b:P314L,ORF1b:K1383R,ORF1b:D2499N,ORF8:Q27*,ORF8:R52I,ORF8:Y73C,S:D178H,S:A570D,S:D614G,S:P681H,S:T716I,S:S982A,S:D1118H	6	2592
hCoV-19/Mexico/ROO-INMEGEN-06-01-224/2021	EPI_ISL_2616981	-	B.1.1.7	GR	32	C241T,C913T,C3037T,C3267T,A3350G,C5388A,T6954C,G14202A,C14408T,C14676T,C15279T,T16176C,C17002T,A17615G,G22081A,C23271A,A23403G,C23604A,C23709T,T24506G,G24914C,G27467C,C27972T,G28048T,A28111G,G28280C,A28281T,T28282A,G28881A,G28882A,G28883	21	N:D3L,N:R203K,N:G204R,N:S235F,ORF1a:T1001I,ORF1a:S1029G,ORF1a:A1708D,ORF1a:I2230T,ORF1b:P314L,ORF1b:L1179F,ORF1b:K1383R,ORF7a:R25T,ORF8:Q27*,ORF8:R52I,ORF8:Y73C,S:A570D,S:D614G,S:P681H,S:T716I,S:S982A,S	6	2367
hCoV-19/Mexico/ROO-INMEGEN-06-01-233/2021	EPI_ISL_2616887	-	B.1.1.7	GRY	39	C241T,C913T,C3037T,C3267T,G3431T,C4752T,C5388A,C5986T,T6954C,C9693T,C11572T,C14408T,C14676T,C15279T,T16176C,G18651A,C18788T,T23042C,A23063T,C23271A,A23403G,C23604A,C23709T,T24506G,G24914C,G25218T,C25553T,G25660C,C27615T,C27972T,G28048T,A28111G,G28280C,A28281T,T28282A,G28881A,G28882A,G28883C,C28977T	26	N:D3L,N:R203K,N:G204R,N:S235F,ORF1a:T1001I,ORF1a:V1056L,ORF1a:T1496I,ORF1a:A1708D,ORF1a:I2230T,ORF1a:A3143V,ORF1b:P314L,ORF1b:T1774I,ORF3a:A54V,ORF3a:V90L,ORF8:Q27*,ORF8:R52I,ORF8:Y73C,S:S494P,S:N501Y,S:A570D,S:D614G,S:P681H,S:T716I,S:S982A,S:D1118H,S:G1219V	6	684
hCoV-19/Mexico/ROO-INMEGEN-06-01-241/2021	EPI_ISL_2616867	-	B.1.1.7	GRY	40	C241T,C913T,C2110T,C2143T,C3037T,G3047T,C3267T,C5388A,C5986T,C6730T,C7945T,A10195G,C10834T,C14120T,C14408T,C14676T,C15279T,T16176C,T20445C,A23063T,C23271A,A23403G,C23604A,C23709T,T24506G,G24914C,C27972T,G28048T,G28075T,A28111G,C28236T,G28280C,A28281T,T28282A,G28881A,G28882A,G28883C,C28896G,C28977T,C29642T	22	N:D3L,N:R203K,N:G204R,N:A208G,N:S235F,ORF1a:D928Y,ORF1a:T1001I,ORF1a:A1708D,ORF1b:P218L,ORF1b:P314L,ORF8:Q27*,ORF8:R52I,ORF8:C61F,ORF8:Y73C,ORF8:R115C,S:N501Y,S:A570D,S:D614G,S:P681H,S:T716I,S:S982A,S:D1118H	6	756
hCoV-19/Mexico/ROO-INMEGEN-06-01-253/2021	EPI_ISL_2616898	-	B.1.1.7	GRY	40	C241T,C913T,G2281A,C3037T,C3267T,C5388A,C5986T,T6954C,A12162G,C13665T,C14408T,C14676T,C15279T,C15720T,T16176C,A17615G,C18747T,C20844T,A21137G,A23063T,C23271A,A23403G,C23604A,C23709T,T24506G,G24914C,G25135T,C26110T,G26828T,C27972T,G28048T,A28111G,G28280C,A28281T,T28282A,G28881A,G28882A,G28883C,C28977T,C29095T	23	N:D3L,N:R203K,N:G204R,N:S235F,ORF1a:T1001I,ORF1a:A1708D,ORF1a:I2230T,ORF1a:Q3966R,ORF1b:P314L,ORF1b:K1383R,ORF1b:K2557R,ORF3a:P240S,ORF8:Q27*,ORF8:R52I,ORF8:Y73C,S:N501Y,S:A570D,S:D614G,S:P681H,S:T716I,S:S982A,S:D1118H,S:K1191N	6	631
hCoV-19/Mexico/ROO-INMEGEN-06-01-268/2021	EPI_ISL_2616994	-	B.1.1.7	GR	40	C222T,C241T,C913T,C2110T,C3037T,C3267T,C5388A,C5986T,T6954C,G7042T,C8389T,C9979T,G11029T,C12115T,C14120T,C14408T,C14676T,C15279T,T16176C,G16900T,C17762T,C23271A,A23403G,C23604A,C23709T,T24506G,G24914C,T25180C,G26416T,G27664T,C27972T,G28048T,A28111G,G28280C,A28281T,T28282A,G28881A,G28882A,G28883C,C28977T	24	E:V58F,N:D3L,N:R203K,N:G204R,N:S235F,ORF1a:T1001I,ORF1a:A1708D,ORF1a:I2230T,ORF1a:M2259I,ORF1a:L3588F,ORF1b:P218L,ORF1b:P314L,ORF1b:G1145C,ORF1b:A1432V,ORF7a:E91*,ORF8:Q27*,ORF8:R52I,ORF8:Y73C,S:A570D,S:D614G,S:P681H,S:T716I,S:S982A,S:D1118H	6	1720
hCoV-19/Mexico/ROO-INMEGEN-06-01-301/2021	EPI_ISL_2616969	-	B.1.1.7	GRY	40	C222T,C241T,C913T,C2110T,C3037T,C3267T,C4423T,C5388A,C5986T,C6681T,T6954C,G7042T,A10359G,C14120T,C14408T,C14676T,C15279T,T16176C,C17762T,C21622T,A23063T,C23271A,A23403G,C23604A,G23608T,C23709T,T24506G,G24914C,T25180C,C27972T,G28048T,A28095T,A28111G,G28280C,A28281T,T28282A,G28881A,G28882A,G28883C,C28977T	24	N:D3L,N:R203K,N:G204R,N:S235F,ORF1a:T1001I,ORF1a:A1708D,ORF1a:A2139V,ORF1a:I2230T,ORF1a:M2259I,ORF1a:K3365R,ORF1b:P218L,ORF1b:P314L,ORF1b:A1432V,ORF8:Q27*,ORF8:R52I,ORF8:K68*,ORF8:Y73C,S:N501Y,S:A570D,S:D614G,S:P681H,S:T716I,S:S982A,S:D1118H	6	103
hCoV-19/Mexico/ROO-INMEGEN-06-01-309/2021	EPI_ISL_2616841	-	B.1.1.7	GR	39	C222T,C241T,C913T,C2110T,C3037T,C3267T,C4423T,C5388A,C5986T,C6681T,T6954C,G7042T,A10359G,C14120T,C14408T,C14676T,C15279T,T16176C,C17762T,C21622T,C23271A,A23403G,C23604A,G23608T,C23709T,T24506G,G24914C,T25180C,C27972T,G28048T,A28095T,A28111G,G28280C,A28281T,T28282A,G28881A,G28882A,G28883C,C28977T	23	N:D3L,N:R203K,N:G204R,N:S235F,ORF1a:T1001I,ORF1a:A1708D,ORF1a:A2139V,ORF1a:I2230T,ORF1a:M2259I,ORF1a:K3365R,ORF1b:P218L,ORF1b:P314L,ORF1b:A1432V,ORF8:Q27*,ORF8:R52I,ORF8:K68*,ORF8:Y73C,S:A570D,S:D614G,S:P681H,S:T716I,S:S982A,S:D1118H	6	941
hCoV-19/Mexico/ROO-INMEGEN-06-01-322/2021	EPI_ISL_2616965	-	B.1.1.7	GRY	34	C241T,C913T,C2453T,C3037T,C3267T,C5388A,C5934T,C5986T,T6954C,C9611T,C14408T,C14676T,C15279T,T15663C,T16176C,G20352A,C20946T,C21855T,G21974C,A23063T,C23271A,A23403G,C23604A,C23709T,T24506G,G24914C,C27972T,G28048T,A28111G,A28295G,G28881A,G28882A,G28883C,C28977T	23	N:N8D,N:R203K,N:G204R,N:S235F,ORF1a:L730F,ORF1a:T1001I,ORF1a:A1708D,ORF1a:T1890I,ORF1a:I2230T,ORF1a:L3116F,ORF1b:P314L,ORF8:Q27*,ORF8:R52I,ORF8:Y73C,S:S98F,S:D138H,S:N501Y,S:A570D,S:D614G,S:P681H,S:T716I,S:S982A,S:D1118H	6	4
hCoV-19/Mexico/ROO-INMEGEN-06-01-342/2021	EPI_ISL_2616963	-	B.1.1.7	GRY	40	C222T,C241T,C913T,C2110T,C3037T,C3267T,C4423T,C5388A,C5986T,C6681T,T6954C,G7042T,A10359G,C14120T,C14408T,C14676T,C15279T,T16176C,C17762T,A23063T,C23271A,A23403G,C23604A,G23608T,C23709T,T24506G,G24914C,T25180C,C27972T,G28048T,A28095T,A28111G,G28280C,A28281T,T28282A,G28881A,G28882A,G28883C,C28977T,C29733T	24	N:D3L,N:R203K,N:G204R,N:S235F,ORF1a:T1001I,ORF1a:A1708D,ORF1a:A2139V,ORF1a:I2230T,ORF1a:M2259I,ORF1a:K3365R,ORF1b:P218L,ORF1b:P314L,ORF1b:A1432V,ORF8:Q27*,ORF8:R52I,ORF8:K68*,ORF8:Y73C,S:N501Y,S:A570D,S:D614G,S:P681H,S:T716I,S:S982A,S:D1118H	6	302

hCoV-19/Mexico/ROO-INMEGEN-06-01-353/2021	EPI_ISL_2616971	-	B.1.1.7	GRY	43	C241T,C913T,C2110T,C2143T,C3037T,G3047T,C3267T,C5388A,C5986T,C6730T,C7945T,A10195G,C10834T,C14120T,C14408T,C14676T,C15279T,T16176C,T20445C,A23063T,C23271A,A23403G,C23604A,C23709T,T24506G,G24914C,T27384C,C27972T,G28048T,G28075T,A28095T,A28111G,C28236T,G28280C,A28281T,T28282A,G28307A,G28881A,G28882A,G28883C,C28896G,C28	25	N:D3L,N:A12T,N:R203K,N:G204R,N:A208G,N:S235F,ORF1a:D928Y,ORF1a:T1001I,ORF1a:A1708D,ORF1b:P218L,ORF1b:P314L,ORF8:Q27*,ORF8:R52I,ORF8:C61F,ORF8:K68*,ORF8:Y73C,ORF8:R115C,ORF9b:M8I,S:N501Y,S:A570D,S:D614G,S:P681H,S:T716I,S:S982A,S:D1118H	6	176
hCoV-19/Mexico/ROO-INMEGEN-06-02-104/2021	EPI_ISL_2692631	-	B.1.1.7	GRY	36	C241T,C913T,C2453T,C3037T,C3177T,C3267T,C5388A,C5986T,T6954C,C9611T,C9893T,C14408T,C14676T,C15279T,T16176C,C21855T,G21974C,A23063T,C23271A,A23403G,C23604A,C23709T,T24506G,G24914C,C27972T,G28048T,A28111G,G28280C,A28281T,T28282A,A28295G,C28775T,G28881A,G28882A,G28883C,C28977T	26	N:D3L,N:N8D,N:P168S,N:R203K,N:G204R,N:S235F,ORF1a:L730F,ORF1a:P971L,ORF1a:T1001I,ORF1a:A1708D,ORF1a:I2230T,ORF1a:L3116F,ORF1a:L3210F,ORF1b:P314L,ORF8:Q27*,ORF8:R52I,ORF8:Y73C,S:S98F,S:D138H,S:N501Y,S:A570D,S:D614G,S:P681H,S:T716I,S:S982A,S:D1118H	6	0
hCoV-19/Mexico/ROO-INMEGEN-06-02-106/2021	EPI_ISL_2692633	-	B.1.1.7	GRY	40	C222T,C241T,C913T,C2110T,C3037T,C3267T,C4423T,C5388A,C5986T,C6681T,T6954C,G7042T,C9693T,A10359G,C14120T,C14408T,C14676T,C15279T,T16176C,C17762T,A23063T,C23271A,A23403G,C23604A,G23608T,C23709T,T24506G,G24914C,T25180C,C27972T,G28048T,A28095T,A28111G,G28280C,A28281T,T28282A,G28881A,G28882A,G28883C,C28977T	25	N:D3L,N:R203K,N:G204R,N:S235F,ORF1a:T1001I,ORF1a:A1708D,ORF1a:A2139V,ORF1a:I2230T,ORF1a:M2259I,ORF1a:A3143V,ORF1a:K3365R,ORF1b:P218L,ORF1b:P314L,ORF1b:A1432V,ORF8:Q27*,ORF8:R52I,ORF8:K68*,ORF8:Y73C,S:N501Y,S:A570D,S:D614G,S:P681H,S:T716I,S:S982A,S:D1118H	6	0
hCoV-19/Mexico/ROO-INMEGEN-06-02-130/2021	EPI_ISL_2692651	-	B.1.1.7	GRY	34	C241T,C913T,C1314T,C1912T,C3037T,C3177T,C3267T,C5388A,C5986T,T6954C,C10252T,C14408T,C14676T,C15279T,T15795C,T16176C,A23063T,C23271A,A23403G,C23604A,C23709T,T24506G,G24914C,C27879T,C27972T,G28048T,A28111G,G28280C,A28281T,T28282A,G28881A,G28	21	N:D3L,N:R203K,N:G204R,N:S235F,ORF1a:T350I,ORF1a:P971L,ORF1a:T1001I,ORF1a:A1708D,ORF1a:I2230T,ORF1b:P314L,ORF7b:H42Y,ORF8:Q27*,ORF8:R52I,ORF8:Y73C,S:N501Y,S:A570D,S:D614G,S:P681H,S:T716I,S:S982A,S:D1118H	6	2
hCoV-19/Mexico/ROO-INMEGEN-06-02-146/2021	EPI_ISL_2692665	-	B.1.1.7	GRY	39	C222T,C241T,C913T,C2110T,C3037T,C3267T,C4423T,C5388A,C5986T,C6681T,T6954C,G7042T,A10359G,C14120T,C14408T,C14676T,C15279T,T16176C,C17762T,A23063T,C23271A,A23403G,C23604A,G23608T,C23709T,T24506G,G24914C,T25180C,C27972T,G28048T,A28095T,A28111G,G28280C,A28281T,T28282A,G28881A,G28882A,G28883C,C28977T	24	N:D3L,N:R203K,N:G204R,N:S235F,ORF1a:T1001I,ORF1a:A1708D,ORF1a:A2139V,ORF1a:I2230T,ORF1a:M2259I,ORF1a:K3365R,ORF1b:P218L,ORF1b:P314L,ORF1b:A1432V,ORF8:Q27*,ORF8:R52I,ORF8:K68*,ORF8:Y73C,S:N501Y,S:A570D,S:D614G,S:P681H,S:T716I,S:S982A,S:D1118H	6	0
hCoV-19/Mexico/ROO-INMEGEN-06-02-183/2021	EPI_ISL_2692696	-	B.1.1.7	GRY	40	C241T,C673T,C913T,C3037T,C3267T,C5388A,C5986T,T6954C,A10323G,A12162G,C13665T,C14408T,C14676T,C15279T,C15720T,T16176C,A17615G,C18747T,C20844T,A21137G,A23063T,C23271A,A23403G,C23604A,C23709T,T24506G,G24914C,G25135T,C26110T,C27972T,G28048T,A28111G,G28280C,A28281T,T28282A,G28881A,G28882A,G28883C,C28977T,C29095T	24	N:D3L,N:R203K,N:G204R,N:S235F,ORF1a:T1001I,ORF1a:A1708D,ORF1a:I2230T,ORF1a:K3353R,ORF1a:Q3966R,ORF1b:P314L,ORF1b:K1383R,ORF1b:K2557R,ORF3a:P240S,ORF8:Q27*,ORF8:R52I,ORF8:Y73C,S:N501Y,S:A570D,S:D614G,S:P681H,S:T716I,S:S982A,S:D1118H,S:K1191N	6	411
hCoV-19/Mexico/ROO-INMEGEN-06-02-320/2021	EPI_ISL_2692807	-	B.1.1.7	GRY	37	C222T,C241T,C913T,C2110T,C3037T,C3267T,C4206T,C5388A,C5986T,T6954C,C7006T,G7042T,C9165T,C14120T,C14408T,C14676T,C15279T,T16176C,C17762T,A23063T,C23271A,A23403G,C23604A,C23709T,T24506G,G24914C,T25180C,C27972T,G28048T,A28111G,G28280C,A28281T,T28282A,G28881A,G28882A,G28883C,C28977T	23	N:D3L,N:R203K,N:G204R,N:S235F,ORF1a:T1001I,ORF1a:A1314V,ORF1a:A1708D,ORF1a:I2230T,ORF1a:M2259I,ORF1a:T2967I,ORF1b:P218L,ORF1b:P314L,ORF1b:A1432V,ORF8:Q27*,ORF8:R52I,ORF8:Y73C,S:N501Y,S:A570D,S:D614G,S:P681H,S:T716I,S:S982A,S:D1118H	6	58
hCoV-19/Mexico/ROO-INMEGEN-06-02-40/2021	EPI_ISL_2692575	-	B.1.1.7	GRY	33	C241T,C913T,C3037T,C3267T,C5388A,C5986T,C6196T,C6268T,T6954C,A8848G,C14408T,C14676T,C15279T,T16176C,A23063T,C23271A,A23403G,C23604A,C23709T,T24506G,G24914C,C27131T,C27972T,G28048T,A28111G,G28280C,A28281T,T28282A,G28881A,G28882A,G28883C,C28	18	N:D3L,N:R203K,N:G204R,N:S235F,ORF1a:T1001I,ORF1a:A1708D,ORF1a:I2230T,ORF1b:P314L,ORF8:Q27*,ORF8:R52I,ORF8:Y73C,S:N501Y,S:A570D,S:D614G,S:P681H,S:T716I,S:S982A,S:D1118H	6	21
hCoV-19/Mexico/ROO-INMEGEN-06-02-58/2021	EPI_ISL_2692591	-	B.1.1.7	GR	35	C241T,C913T,C2453T,C3037T,C3267T,C5388A,C5986T,T6954C,C9611T,C9893T,C14408T,C14676T,C15279T,T16176C,A17342G,C21855T,G21974C,C23271A,A23403G,C23604A,C23709T,T24506G,G24914C,G25906T,C27972T,G28048T,A28111G,G28280C,A28281T,T28282A,A28295G,G28881A,G28882A,G28883C,C28977T	25	N:D3L,N:N8D,N:R203K,N:G204R,N:S235F,ORF1a:L730F,ORF1a:T1001I,ORF1a:A1708D,ORF1a:I2230T,ORF1a:L3116F,ORF1a:L3210F,ORF1b:P314L,ORF1b:D1292G,ORF3a:G172C,ORF8:Q27*,ORF8:R52I,ORF8:Y73C,S:S98F,S:D138H,S:A570D,S:D614G,S:P681H,S:T716I,S:S982A,S:D1118H	6	2033
hCoV-19/Mexico/ROO-INMEGEN-06-02-66/2021	EPI_ISL_2692598	-	B.1.1.7	GRY	43	C222T,C241T,C913T,C2110T,C3037T,C3267T,C5388A,C5986T,T6954C,G7042T,C8389T,C12115T,C13517T,C14120T,C14408T,C14676T,C15279T,T16176C,G16900T,C17762T,C21077T,A23063T,C23271A,A23403G,C23604A,C23709T,T24506G,G24914C,T25180C,C25665T,G25785T,G26416T,C27972T,G28048T,A28095T,A28111G,G28280C,A28281T,T28282A,G28881A,G28882A,G28883	27	E:V58F,N:D3L,N:R203K,N:G204R,N:S235F,ORF1a:T1001I,ORF1a:A1708D,ORF1a:I2230T,ORF1a:M2259I,ORF1b:T17I,ORF1b:P218L,ORF1b:P314L,ORF1b:G1145C,ORF1b:A1432V,ORF1b:T2537I,ORF3a:W131C,ORF8:Q27*,ORF8:R52I,ORF8:K68*,ORF8:Y73C,S:N501Y,S:A570D,S:D614G,S:P681H,S:T716I,S:S982A,S:D11	6	704
hCoV-19/Mexico/ROO-INMEGEN-06-02-69/2021	EPI_ISL_2692601	-	B.1.1.7	GRY	40	C222T,C241T,C913T,C2110T,C3037T,C3267T,C4423T,C5388A,C5986T,C6681T,T6954C,G7042T,A10359G,C14120T,C14408T,C14676T,C15279T,T16176C,G16207T,C17762T,A23063T,C23271A,A23403G,C23604A,G23608T,C23709T,T24506G,G24914C,T25180C,C27972T,G28048T,A28095T,A28111G,G28280C,A28281T,T28282A,G28881A,G28882A,G28883C,C28977T	25	N:D3L,N:R203K,N:G204R,N:S235F,ORF1a:T1001I,ORF1a:A1708D,ORF1a:A2139V,ORF1a:I2230T,ORF1a:M2259I,ORF1a:K3365R,ORF1b:P218L,ORF1b:P314L,ORF1b:A914S,ORF1b:A1432V,ORF8:Q27*,ORF8:R52I,ORF8:K68*,ORF8:Y73C,S:N501Y,S:A570D,S:D614G,S:P681H,S:T716I,S:S982A,S:D1118H	6	675
hCoV-19/Mexico/ROO-INMEGEN-06-02-7/2021	EPI_ISL_2692545	-	B.1.1.7	GRY	35	C241T,C913T,C2453T,C3037T,C3267T,C5388A,C5986T,T6954C,C9611T,C9893T,C14408T,C14676T,C15279T,T16176C,C21855T,G21974C,A23063T,C23271A,A23403G,C23604A,C23709T,T24506G,G24914C,C27972T,G28048T,A28111G,G28280C,A28281T,T28282A,A28295G,C28775T,G28881A,G28882A,G28883C,C28977T	25	N:D3L,N:N8D,N:P168S,N:R203K,N:G204R,N:S235F,ORF1a:L730F,ORF1a:T1001I,ORF1a:A1708D,ORF1a:I2230T,ORF1a:L3116F,ORF1a:L3210F,ORF1b:P314L,ORF8:Q27*,ORF8:R52I,ORF8:Y73C,S:S98F,S:D138H,S:N501Y,S:A570D,S:D614G,S:P681H,S:T716I,S:S982A,S:D1118H	6	541
hCoV-19/Mexico/ROO-INMEGEN-06-02-77/2021	EPI_ISL_2692607	-	B.1.1.7	GRY	42	C241T,C913T,C2110T,C2143T,C3037T,G3047T,C3267T,C5388A,C5986T,C6730T,G7042T,C7945T,A10195G,C10834T,C14120T,C14408T,C14676T,C15279T,T16176C,T20445C,A23063T,C23271A,A23403G,C23604A,C23709T,T24506G,G24914C,C27972T,G28048T,G28075T,A28095T,A28111G,C28236T,G28280C,A28281T,T28282A,G28881A,G28882A,G28883C,C28896G,C28977T,C296	24	N:D3L,N:R203K,N:G204R,N:A208G,N:S235F,ORF1a:D928Y,ORF1a:T1001I,ORF1a:A1708D,ORF1a:M2259I,ORF1b:P218L,ORF1b:P314L,ORF8:Q27*,ORF8:R52I,ORF8:C61F,ORF8:K68*,ORF8:Y73C,ORF8:R115C,S:N501Y,S:A570D,S:D614G,S:P681H,S:T716I,S:S982A,S:D1118H	6	120
hCoV-19/Mexico/ROO-INMEGEN-06-02-82/2021	EPI_ISL_2692612	-	B.1.1.7	GRY	34	C241T,C913T,C3037T,C3267T,A3350G,C5388A,C5986T,T6954C,G14202A,C14408T,C14676T,C15279T,T16176C,C17002T,A17615G,G22081A,A23063T,C23271A,A23403G,C23604A,C23709T,T24506G,G24914C,G27467C,C27972T,G28048T,A28111G,G28280C,A28281T,T28282A,G28881A,G28882A,G28883C,C28977T	22	N:D3L,N:R203K,N:G204R,N:S235F,ORF1a:T1001I,ORF1a:S1029G,ORF1a:A1708D,ORF1a:I2230T,ORF1b:P314L,ORF1b:L1179F,ORF1b:K1383R,ORF7a:R25T,ORF8:Q27*,ORF8:R52I,ORF8:Y73C,S:N501Y,S:A570D,S:D614G,S:P681H,S:T716I,S:S982A,S:D1118H	6	1891
hCoV-19/Mexico/ROO-INMEGEN-06-02-99/2021	EPI_ISL_2692627	-	B.1.1.7	GRY	43	C241T,C913T,C2110T,C2143T,C3037T,G3047T,C3267T,C5388A,C5986T,C6730T,T6954C,G7042T,C7945T,A10195G,C10834T,C14120T,C14408T,C14676T,C15279T,T16176C,T20445C,A23063T,C23271A,A23403G,C23604A,C23709T,T24506G,G24914C,C27972T,G28048T,G28075T,A28095T,A28111G,C28236T,G28280C,A28281T,T28282A,G28881A,G28882A,G28883C,C28896G,C28977	25	N:D3L,N:R203K,N:G204R,N:A208G,N:S235F,ORF1a:D928Y,ORF1a:T1001I,ORF1a:A1708D,ORF1a:I2230T,ORF1a:M2259I,ORF1b:P218L,ORF1b:P314L,ORF8:Q27*,ORF8:R52I,ORF8:C61F,ORF8:K68*,ORF8:Y73C,ORF8:R115C,S:N501Y,S:A570D,S:D614G,S:P681H,S:T716I,S:S982A,S:D1118H	6	42
hCoV-19/Mexico/ROO-INMEGEN-06-03-289/2021	EPI_ISL_2810251	-	B.1.1.7	GRY	36	C241T,C673T,C913T,C2453T,C3037T,C3267T,C5388A,C5986T,T6954C,C9611T,C9893T,C14408T,C14676T,C15279T,T16176C,G17027T,C21855T,G21974C,A23063T,C23271A,A23403G,C23604A,C23709T,T24506G,G24914C,C27972T,G28048T,A28111G,G28280C,A28281T,T28282A,A28295G,G28881A,G28882A,G28883C,C28977T	25	N:D3L,N:N8D,N:R203K,N:G204R,N:S235F,ORF1a:L730F,ORF1a:T1001I,ORF1a:A1708D,ORF1a:I2230T,ORF1a:L3116F,ORF1a:L3210F,ORF1b:P314L,ORF1b:S1187I,ORF8:Q27*,ORF8:R52I,ORF8:Y73C,S:S98F,S:D138H,S:N501Y,S:A570D,S:D614G,S:P681H,S:T716I,S:S982A,S:D1118H	6	478
hCoV-19/Mexico/ROO-INMEGEN-06-03-296/2021	EPI_ISL_2810258	-	B.1.1.7	GR	36	C241T,C254T,C913T,C2453T,C3037T,C3267T,C5388A,C5986T,T6954C,C9611T,C14408T,C14676T,C15279T,T16176C,T18099C,C21855T,G21974C,C23271A,A23403G,C23604A,C23709T,T24506G,G24914C,C25609T,C27389T,C27972T,G28048T,A28111G,G28280C,A28281T,T28282A,A28295G,G28881A,G28882A,G28883C,C28977T	23	N:D3L,N:N8D,N:R203K,N:G204R,N:S235F,ORF1a:L730F,ORF1a:T1001I,ORF1a:A1708D,ORF1a:I2230T,ORF1a:L3116F,ORF1b:P314L,ORF3a:L73F,ORF8:Q27*,ORF8:R52I,ORF8:Y73C,S:S98F,S:D138H,S:A570D,S:D614G,S:P681H,S:T716I,S:S982A,S:D1118H	6	436
hCoV-19/Mexico/ROO-INMEGEN-06-03-320/2021	EPI_ISL_2810278	-	B.1.1.7	GRY	38	C222T,C241T,C913T,C2110T,C3037T,C3267T,C4423T,C5388A,C5986T,C6681T,T6954C,G7042T,A10359G,C14120T,C14408T,C14676T,C15279T,T16176C,C17762T,A23063T,C23271A,A23403G,C23604A,G23608T,C23709T,T24506G,G24914C,T25180C,C27972T,G28048T,A28111G,G28280C,A28281T,T28282A,G28881A,G28882A,G28883C,C28977T	23	N:D3L,N:R203K,N:G204R,N:S235F,ORF1a:T1001I,ORF1a:A1708D,ORF1a:A2139V,ORF1a:I2230T,ORF1a:M2259I,ORF1a:K3365R,ORF1b:P218L,ORF1b:P314L,ORF1b:A1432V,ORF8:Q27*,ORF8:R52I,ORF8:Y73C,S:N501Y,S:A570D,S:D614G,S:P681H,S:T716I,S:S982A,S:D1118H	6	12
hCoV-19/Mexico/ROO-INMEGEN-06-04-14/2021	EPI_ISL_2894164	-	B.1.1.7	GR	29	C241T,C913T,C3037T,C3267T,C5388A,C5986T,A6319G,T6954C,A8848G,C14408T,C14676T,C15279T,T16176C,C23271A,A23403G,C23604A,C23709T,T24506G,G24914C,G27870T,C27972T,G28048T,A28111G,T28282A,G28881A,G28882A,G28883C,C28977T,G29715T	18	N:D3E,N:R203K,N:G204R,N:S235F,ORF1a:T1001I,ORF1a:A1708D,ORF1a:I2230T,ORF1b:P314L,ORF7b:E39*,ORF8:Q27*,ORF8:R52I,ORF8:Y73C,S:A570D,S:D614G,S:P681H,S:T716I,S:S982A,S:D1118H	6	412
hCoV-19/Mexico/ROO-INMEGEN-06-04-313/2021	EPI_ISL_2894360	-	B.1.1.7	GRY	36	C241T,C673T,C913T,C2453T,C3037T,C3267T,C5388A,C5986T,T6954C,C9611T,C9893T,C14408T,C14676T,C15279T,T16176C,G17027T,C21855T,G21974C,A23063T,C23271A,A23403G,C23604A,C23709T,T24506G,G24914C,C27972T,G28048T,A28111G,G28280C,A28281T,T28282A,A28295G,G28881A,G28882A,G28883C,C28977T	25	N:D3L,N:N8D,N:R203K,N:G204R,N:S235F,ORF1a:L730F,ORF1a:T1001I,ORF1a:A1708D,ORF1a:I2230T,ORF1a:L3116F,ORF1a:L3210F,ORF1b:P314L,ORF1b:S1187I,ORF8:Q27*,ORF8:R52I,ORF8:Y73C,S:S98F,S:D138H,S:N501Y,S:A570D,S:D614G,S:P681H,S:T716I,S:S982A,S:D1118H	6	16

hCoV-19/Mexico/ROO-INMEGEN-06-04-316/2021	EPI_ISL_2894363	-	B.1.1.7	GRY	40	C241T,C913T,C2110T,C3037T,C3267T,C5388A,C5986T,T6954C,G7042T,A10195G,C10279T,C14120T,C14408T,C14676T,C15279T,T16176C,C16887T,G17427T,A19892G,A23063T,C23271A,A23403G,C23604A,C23709T,T24506G,G24914C,G27632T,A27943G,C27972T,G28048T,G28075T,A28095T,A28111G,G28280C,A28281T,T28282A,G28881A,G28882A,G28883C,C28977T	25	N:D3L,N:R203K,N:G204R,N:S235F,ORF1a:T1001I,ORF1a:A1708D,ORF1a:I2230T,ORF1a:M2259I,ORF1b:P218L,ORF1b:P314L,ORF1b:D2142G,ORF7a:R80I,ORF8:H17R,ORF8:Q27*,ORF8:R52I,ORF8:C61F,ORF8:K68*,ORF8:Y73C,S:N501Y,S:A570D,S:D614G,S:P681H,S:T716I,S:S982A,S:D1118H	6	0
hCoV-19/Mexico/ROO-INMEGEN-06-04-317/2021	EPI_ISL_2894364	-	B.1.1.7	GRY	40	T99C,C241T,C913T,C3037T,C3267T,C5284T,C5388A,C5986T,T6954C,A9238G,C10369T,C14408T,C14676T,C15279T,T16176C,C17004T,A17615G,G20056A,A21276G,A23063T,C23271A,A23403G,C23604A,C23709T,T24506G,G24914C,C25413T,G26062T,G26065T,C27972T,G28048T,A28111G,G28280C,A28281T,T28282A,G28881A,G28882A,G28883C,C28977T,G29777T	22	N:D3L,N:R203K,N:G204R,N:S235F,ORF1a:T1001I,ORF1a:A1708D,ORF1a:I2230T,ORF1b:P314L,ORF1b:K1383R,ORF1b:G2197S,ORF3a:G224C,ORF3a:V225F,ORF8:Q27*,ORF8:R52I,ORF8:Y73C,S:N501Y,S:A570D,S:D614G,S:P681H,S:T716I,S:S982A,S:D1118H	6	56
hCoV-19/Mexico/ROO-INMEGEN-06-04-320/2021	EPI_ISL_2894366	-	B.1.1.7	GRY	41	C241T,C913T,C2110T,C2143T,C3037T,G3047T,C3267T,C5388A,C5986T,C6730T,C7945T,A10195G,C10834T,C14120T,C14408T,C14676T,C15279T,T16176C,T20445C,A23063T,C23271A,A23403G,C23604A,C23709T,T24506G,G24914C,C27972T,G28048T,G28075T,A28095T,A28111G,C28236T,G28280C,A28281T,T28282A,G28881A,G28882A,G28883C,C28896G,C28977T,C29642T	23	N:D3L,N:R203K,N:G204R,N:A208G,N:S235F,ORF1a:D928Y,ORF1a:T1001I,ORF1a:A1708D,ORF1b:P218L,ORF1b:P314L,ORF8:Q27*,ORF8:R52I,ORF8:C61F,ORF8:K68*,ORF8:Y73C,ORF8:R115C,S:N501Y,S:A570D,S:D614G,S:P681H,S:T716I,S:S982A,S:D1118H	6	138
hCoV-19/Mexico/ROO-INMEGEN-06-04-321/2021	EPI_ISL_2894367	-	B.1.1.7	GRY	41	C222T,C241T,C715T,C913T,A977G,C2110T,C3037T,C3267T,C4423T,C5388A,C5986T,C6681T,T6954C,G7042T,A10359G,C14120T,C14408T,C14676T,C15279T,T16176C,C17762T,A23063T,C23271A,A23403G,C23604A,G23608T,C23709T,T24506G,G24914C,T25180C,C27972T,G28048T,A28095T,A28111G,G28280C,A28281T,T28282A,G28881A,G28882A,G28883C,C28977T	25	N:D3L,N:R203K,N:G204R,N:S235F,ORF1a:I238V,ORF1a:T1001I,ORF1a:A1708D,ORF1a:A2139V,ORF1a:I2230T,ORF1a:M2259I,ORF1a:K3365R,ORF1b:P218L,ORF1b:P314L,ORF1b:A1432V,ORF8:Q27*,ORF8:R52I,ORF8:K68*,ORF8:Y73C,S:N501Y,S:A570D,S:D614G,S:P681H,S:T716I,S:S982A,S:D1118H	6	4
hCoV-19/Mexico/ROO-INMEGEN-06-04-322/2021	EPI_ISL_2894368	-	B.1.1.7	GRY	39	C64T,C241T,C841T,C913T,C3037T,C3267T,A3946G,C5388A,C5986T,T6954C,C8320T,C11758T,C12400T,A12507G,C14408T,C14676T,C15279T,T16176C,T20733C,C21812T,A23063T,C23271A,A23403G,C23604A,C23709T,T24506G,G24914C,C25511T,C27972T,G28048T,A28111G,G28280C,A28281T,T28282A,G28881A,G28882A,G28883C,C28977T,C29732T	20	N:D3L,N:R203K,N:G204R,N:S235F,ORF1a:T1001I,ORF1a:A1708D,ORF1a:I2230T,ORF1a:K4081R,ORF1b:P314L,ORF3a:S40L,ORF8:Q27*,ORF8:R52I,ORF8:Y73C,S:N501Y,S:A570D,S:D614G,S:P681H,S:T716I,S:S982A,S:D1118H	6	0
hCoV-19/Mexico/ROO-INMEGEN-06-04-323/2021	EPI_ISL_2894369	-	B.1.1.7	GRY	43	C241T,C913T,C2110T,C2143T,C3037T,G3047T,C3267T,C5388A,C5986T,C6636T,C6730T,C7945T,A10195G,C10834T,C14120T,C14408T,C14676T,C15279T,T16176C,C18458T,T20445C,A23063T,C23271A,A23403G,C23604A,C23709T,T24506G,G24914C,C27972T,G28048T,G28075T,A28095T,A28111G,C28236T,G28280C,A28281T,T28282A,G28881A,G28882A,G28883C,C28896G,C289	25	N:D3L,N:R203K,N:G204R,N:A208G,N:S235F,ORF1a:D928Y,ORF1a:T1001I,ORF1a:A1708D,ORF1a:T2124I,ORF1b:P218L,ORF1b:P314L,ORF1b:P1664L,ORF8:Q27*,ORF8:R52I,ORF8:C61F,ORF8:K68*,ORF8:Y73C,ORF8:R115C,S:N501Y,S:A570D,S:D614G,S:P681H,S:T716I,S:S982A,S:D1118H	6	173
hCoV-19/Mexico/ROO-INMEGEN-06-04-54/2021	EPI_ISL_2894198	-	B.1.1.7	GRY	39	C241T,C913T,A2105G,C2110T,C3037T,C3267T,C5388A,C5986T,T6954C,G7042T,A10195G,C10279T,C14120T,C14408T,C14676T,C15279T,T16176C,C16887T,G17427T,A23063T,C23271A,A23403G,C23604A,C23709T,T24506G,G24914C,C27972T,C28000T,G28048T,G28075T,A28095T,A28111G,G28280C,A28281T,T28282A,G28881A,G28882A,G28883C,C28977T	24	N:D3L,N:R203K,N:G204R,N:S235F,ORF1a:T614A,ORF1a:T1001I,ORF1a:A1708D,ORF1a:I2230T,ORF1a:M2259I,ORF1b:P218L,ORF1b:P314L,ORF8:Q27*,ORF8:P36L,ORF8:R52I,ORF8:C61F,ORF8:K68*,ORF8:Y73C,S:N501Y,S:A570D,S:D614G,S:P681H,S:T716I,S:S982A,S:D1118H	6	5
hCoV-19/Mexico/ROO-INMEGEN-06-04-62/2021	EPI_ISL_2894206	-	B.1.1.7	GRY	40	C241T,C913T,C2110T,C3037T,C3267T,C5388A,C5986T,T6954C,G7042T,C7113T,A13947G,C14120T,C14408T,C14676T,C15279T,T16176C,C17762T,A23063T,T23071C,C23271A,A23403G,C23604A,C23709T,T24506G,G24914C,A25718G,G26020T,C27972T,G28048T,A28095T,A28111G,C28201T,G28280C,A28281T,T28282A,G28881A,G28882A,G28883C,C28977T,C29367T	27	N:D3L,N:R203K,N:G204R,N:S235F,N:P365L,ORF1a:T1001I,ORF1a:A1708D,ORF1a:I2230T,ORF1a:M2259I,ORF1a:T2283I,ORF1b:P218L,ORF1b:P314L,ORF1b:A1432V,ORF3a:Y109C,ORF3a:D210Y,ORF8:Q27*,ORF8:R52I,ORF8:K68*,ORF8:Y73C,ORF8:S103L,S:N501Y,S:A570D,S:D614G,S:P681H,S:T716I,S:S982A,S:D11	6	193
hCoV-19/Mexico/ROO-INMEGEN-06-04-7/2021	EPI_ISL_2894157	-	B.1.1.7	GR	40	C222T,C241T,C913T,A977G,C2110T,C3037T,C3267T,C4423T,C5388A,C5986T,C6681T,T6954C,G7042T,A10359G,C14120T,C14408T,C14676T,C15279T,T16176C,C17762T,C23271A,A23403G,C23604A,G23608T,C23709T,T24506G,G24914C,G25012T,T25180C,C27972T,G28048T,A28095T,A28111G,G28280C,A28281T,T28282A,G28881A,G28882A,G28883C,C28977T	25	N:D3L,N:R203K,N:G204R,N:S235F,ORF1a:I238V,ORF1a:T1001I,ORF1a:A1708D,ORF1a:A2139V,ORF1a:I2230T,ORF1a:M2259I,ORF1a:K3365R,ORF1b:P218L,ORF1b:P314L,ORF1b:A1432V,ORF8:Q27*,ORF8:R52I,ORF8:K68*,ORF8:Y73C,S:A570D,S:D614G,S:P681H,S:T716I,S:S982A,S:D1118H,S:E1150D	6	393
hCoV-19/Mexico/ROO-INMEGEN-16-288/2021	EPI_ISL_2978599	-	B.1.1.7	GR	38	C222T,C241T,C913T,C2110T,C3037T,C3267T,C5388A,C5986T,T6954C,A7933G,T9127C,C9891T,C12809T,C14120T,C14408T,C14676T,C15279T,T16176C,C23271A,A23403G,T23425C,C23604A,C23709T,C24099T,T24506G,G24914C,G27364C,C27972T,G28048T,A28111G,G28280C,A28281T,T28282A,C28291T,G28881A,G28882A,G28883C,C28977T	23	N:D3L,N:R203K,N:G204R,N:S235F,ORF1a:T1001I,ORF1a:A1708D,ORF1a:I2230T,ORF1a:A3209V,ORF1a:L4182F,ORF1b:P218L,ORF1b:P314L,ORF6:E55Q,ORF8:Q27*,ORF8:R52I,ORF8:Y73C,ORF9b:P3L,S:A570D,S:D614G,S:P681H,S:T716I,S:A846V,S:S982A,S:D1118H	6	852
hCoV-19/Mexico/ROO-INMEGEN-16-295/2021	EPI_ISL_2978606	-	B.1.1.7	GRY	43	C222T,C241T,C913T,C2110T,C3037T,C3267T,C5388A,C5986T,T6954C,A7933G,T9127C,C9891T,C12809T,C14120T,C14408T,C14676T,C15279T,T16176C,C22716A,T22717C,A23063T,C23271A,A23403G,T23425C,C23604A,C23709T,C24099T,T24506G,G24914C,A27345C,G27364C,C27972T,G28048T,A28095T,A28111G,G28280C,A28281T,T28282A,C28291T,G28881A,G28882A,G28883	27	N:D3L,N:R203K,N:G204R,N:S235F,ORF1a:T1001I,ORF1a:A1708D,ORF1a:I2230T,ORF1a:A3209V,ORF1a:L4182F,ORF1b:P218L,ORF1b:P314L,ORF6:K48N,ORF6:E55Q,ORF8:Q27*,ORF8:R52I,ORF8:K68*,ORF8:Y73C,ORF9b:P3L,S:T385N,S:N501Y,S:A570D,S:D614G,S:P681H,S:T716I,S:A846V,S:S982A,S:D1118H	6	0
hCoV-19/Mexico/ROO-INMEGEN-16-319/2021	EPI_ISL_2978626	-	B.1.1.7	GR	46	C241T,C913T,C2110T,G3004T,C3037T,C3267T,C5388A,C5986T,A6823G,T6954C,G7042T,C7765T,C10789T,C14120T,C14408T,C14676T,G14857T,C15279T,G15672C,C15810T,T16176C,G20679T,T22219C,G22567T,C23271A,A23403G,C23604A,C23709T,T24506G,G24914C,C25731T,C26110T,C26735T,C27002T,C27972T,G28048T,A28095T,A28111G,G28280C,A28281T,T28282A,A28804G,G28881A,G28882A,G28883C,C28977T	25	N:D3L,N:R203K,N:G204R,N:S235F,ORF1a:E913D,ORF1a:T1001I,ORF1a:A1708D,ORF1a:I2230T,ORF1a:M2259I,ORF1b:P218L,ORF1b:P314L,ORF1b:V464F,ORF1b:E735D,ORF3a:P240S,ORF8:Q27*,ORF8:R52I,ORF8:K68*,ORF8:Y73C,S:L335F,S:A570D,S:D614G,S:P681H,S:T716I,S:S982A,S:D1118H	6	403
hCoV-19/Mexico/ROO-INMEGEN-16-350/2021	EPI_ISL_2978654	-	B.1.1.7	GRY	39	C241T,C823T,C913T,T1104C,C2094T,C2453T,C3037T,C3267T,C5388A,C5986T,T6954C,C9611T,C14408T,C14676T,C15279T,T16176C,T17009C,C21855T,G21974C,A23063T,C23271A,A23403G,C23604A,C23709T,C24374T,T24506G,G24914C,C27972T,G28048T,A28111G,G28280C,A28281T,T28282A,A28295G,G28881A,G28882A,G28883C,C28977T,C29144T	27	N:D3L,N:N8D,N:R203K,N:G204R,N:S235F,ORF1a:I280T,ORF1a:S610L,ORF1a:L730F,ORF1a:T1001I,ORF1a:A1708D,ORF1a:I2230T,ORF1a:L3116F,ORF1b:P314L,ORF1b:I1181T,ORF8:Q27*,ORF8:R52I,ORF8:Y73C,S:S98F,S:D138H,S:N501Y,S:A570D,S:D614G,S:P681H,S:T716I,S:L938F,S:S982A,S:D1118H	6	0
hCoV-19/Mexico/SEARCH-101109/2021	EPI_ISL_2329458	-	B.1.1.7	GRY	32	C241T,C913T,C3037T,C3267T,C5388A,C5986T,T6954C,C14408T,C14676T,C15279T,T16176C,A23063T,C23271A,A23403G,C23604A,C23709T,T24506G,G24914C,G25690T,G25726T,C27972T,G28048T,A28111G,G28280C,A28281T,T28282A,A28352G,G28396T,G28881A,G28882A,G28883C	22	N:D3L,N:N27D,N:R203K,N:G204R,N:S235F,ORF1a:T1001I,ORF1a:A1708D,ORF1a:I2230T,ORF1b:P314L,ORF3a:G100C,ORF3a:V112F,ORF8:Q27*,ORF8:R52I,ORF8:Y73C,ORF9b:G38V,S:N501Y,S:A570D,S:D614G,S:P681H,S:T716I,S:S982A,S:	6	0
hCoV-19/Mexico/SEARCH-101405/2021	EPI_ISL_2422818	-	B.1.1.7	GRY	33	C241T,C913T,C3037T,C3267T,C5388A,C5986T,T6954C,C11580T,C14408T,C14676T,C15279T,T16176C,A23063T,C23271A,A23403G,C23604A,C23709T,T24506G,G24914C,G25690T,G25726T,C27972T,G28048T,A28111G,G28280C,A28281T,T28282A,A28352G,G28396T,G28881A,G28882A,G28883C,C28977T	23	N:D3L,N:N27D,N:R203K,N:G204R,N:S235F,ORF1a:T1001I,ORF1a:A1708D,ORF1a:I2230T,ORF1a:T3772I,ORF1b:P314L,ORF3a:G100C,ORF3a:V112F,ORF8:Q27*,ORF8:R52I,ORF8:Y73C,ORF9b:G38V,S:N501Y,S:A570D,S:D614G,S:P681H,S:T716I,S:S982A,S:D1118H	6	0
hCoV-19/Mexico/SEARCH-101440/2021	EPI_ISL_2422832	-	Q.3	GRY	37	G149T,C241T,G347A,C913T,C3037T,C3267T,G5023A,C5388A,C5986T,T6954C,G10396T,C14408T,C14676T,C15279T,T16176C,A17615G,G20962A,C21108T,A23063T,C23271A,A23403G,C23604A,C23709T,T24506G,G24914C,G26730C,C27972T,G28048T,A28111G,G28280C,A28281T,T28282A,G28881A,G28882A,G28883C,C28977T,G29440T	24	M:V70L,N:D3L,N:R203K,N:G204R,N:S235F,N:Q389H,ORF1a:V28I,ORF1a:T1001I,ORF1a:M1586I,ORF1a:A1708D,ORF1a:I2230T,ORF1b:P314L,ORF1b:K1383R,ORF1b:D2499N,ORF8:Q27*,ORF8:R52I,ORF8:Y73C,S:N501Y,S:A570D,S:D614G,S:P681H,S:T716I,S:S982A,S:D1118H	6	0
hCoV-19/Mexico/SIN_CIAD_HJ1385/2021	EPI_ISL_2026411	-	B.1.1.7	GRY	34	C241T,C913T,C3037T,C3267T,C5388A,C5986T,T6954C,C9298T,C14408T,C14676T,C15279T,T16176C,A17153G,C19554T,G22094A,A23063T,C23271A,A23403G,C23604A,C23709T,C24374T,T24506G,G24914C,C27972T,G28048T,A28111G,G28280C,A28281T,T28282A,G28774T,G28881A,G28882A,G28883C,C28977T	22	N:D3L,N:L167F,N:R203K,N:G204R,N:S235F,ORF1a:T1001I,ORF1a:A1708D,ORF1a:I2230T,ORF1b:P314L,ORF1b:Y1229C,ORF8:Q27*,ORF8:R52I,ORF8:Y73C,S:D178N,S:N501Y,S:A570D,S:D614G,S:P681H,S:T716I,S:L938F,S:S982A,S:D1118H	6	57
hCoV-19/Mexico/SIN_CIAD_HJ1490/2021	EPI_ISL_2026413	-	B.1.1.7	GRY	34	C241T,C913T,C3037T,C3267T,C5388A,C5986T,T6954C,C9298T,C14408T,C14676T,C15279T,T16176C,A17153G,C19554T,G22094A,A23063T,C23271A,A23403G,C23604A,C23709T,C24374T,T24506G,G24914C,C27972T,G28048T,A28111G,G28280C,A28281T,T28282A,G28774T,G28881A,G28882A,G28883C,C28977T	22	N:D3L,N:L167F,N:R203K,N:G204R,N:S235F,ORF1a:T1001I,ORF1a:A1708D,ORF1a:I2230T,ORF1b:P314L,ORF1b:Y1229C,ORF8:Q27*,ORF8:R52I,ORF8:Y73C,S:D178N,S:N501Y,S:A570D,S:D614G,S:P681H,S:T716I,S:L938F,S:S982A,S:D1118H	6	0
hCoV-19/Mexico/SIN_CIAD_HJ1500/2021	EPI_ISL_2026415	-	B.1.1.7	GRY	34	C241T,C913T,C3037T,C3267T,C5388A,C5986T,T6954C,C9298T,C14408T,C14676T,C15279T,T16176C,A17153G,C19554T,G22094A,A23063T,C23271A,A23403G,C23604A,C23709T,C24374T,T24506G,G24914C,C27972T,G28048T,A28111G,G28280C,A28281T,T28282A,G28774T,G28881A,G28882A,G28883C,C28977T	22	N:D3L,N:L167F,N:R203K,N:G204R,N:S235F,ORF1a:T1001I,ORF1a:A1708D,ORF1a:I2230T,ORF1b:P314L,ORF1b:Y1229C,ORF8:Q27*,ORF8:R52I,ORF8:Y73C,S:D178N,S:N501Y,S:A570D,S:D614G,S:P681H,S:T716I,S:L938F,S:S982A,S:D1118H	6	0

hCoV-19/Mexico/SIN_CIAD_HJ1512/2021	EPI_ISL_2026417	-	B.1.1.7	GRY	34	C241T,C913T,C3037T,C3267T,C5388A,C5986T,T6954C,C9298T,C14408T,C14676T,C15279T,T16176C,A17153G,C19554T,G22094A,A23063T,C23271A,A23403G,C23604A,C23709T,C24374T,T24506G,G24914C,C27972T,G28048T,A28111G,G28280C,A28281T,T28282A,G28774T,G28881A,G28882A,G28883C,C28977T	22	N:D3L,N:L167F,N:R203K,N:G204R,N:S235F,ORF1a:T1001I,ORF1a:A1708D,ORF1a:I2230T,ORF1b:P314L,ORF1b:Y1229C,ORF8:Q27*,ORF8:R52I,ORF8:Y73C,S:D178N,S:N501Y,S:A570D,S:D614G,S:P681H,S:T716I,S:L938F,S:S982A,S:D1118H	6	156
hCoV-19/Mexico/SIN_CIAD_HJ1592/2021	EPI_ISL_2249239	-	B.1.1.7	GRY	33	C241T,C913T,C2453T,C3037T,C3267T,C5388A,T6954C,C9611T,C14408T,C14676T,C15279T,T16176C,C21855T,G21974C,A23063T,C23271A,A23403G,C23604A,C23709T,T24506G,G24914C,G25273T,C27972T,G28048T,A28111G,G28280C,A28281T,T28282A,A28295G,G28881A,G28882A,G28883C,C28977T	24	N:D3L,N:N8D,N:R203K,N:G204R,N:S235F,ORF1a:L730F,ORF1a:T1001I,ORF1a:A1708D,ORF1a:I2230T,ORF1a:L3116F,ORF1b:P314L,ORF8:Q27*,ORF8:R52I,ORF8:Y73C,S:S98F,S:D138H,S:N501Y,S:A570D,S:D614G,S:P681H,S:T716I,S:S982A,S:D1118H,S:M1237I	6	462
hCoV-19/Mexico/SIN_CIAD_HJ1599/2021	EPI_ISL_2249241	-	B.1.1.7	GRY	36	C241T,C913T,C3037T,C3267T,C5388A,C5986T,G6421T,T6954C,T7204C,C9298T,C14408T,C14676T,C15279T,T16176C,A17153G,C19554T,G22094A,A23063T,C23271A,A23403G,C23604A,C23709T,C24374T,T24506G,G24914C,C27972T,G28048T,A28111G,G28280C,A28281T,T28282A,G28774T,G28881A,G28882A,G28883C,C28977T	22	N:D3L,N:L167F,N:R203K,N:G204R,N:S235F,ORF1a:T1001I,ORF1a:A1708D,ORF1a:I2230T,ORF1b:P314L,ORF1b:Y1229C,ORF8:Q27*,ORF8:R52I,ORF8:Y73C,S:D178N,S:N501Y,S:A570D,S:D614G,S:P681H,S:T716I,S:L938F,S:S982A,S:D1118H	6	0
hCoV-19/Mexico/SIN_CIAD_HJ1602/2021	EPI_ISL_2249242	-	B.1.1.7	GRY	32	C241T,C913T,C3037T,C3267T,C5388A,C5986T,T6954C,C10748T,C14408T,C14649T,C14676T,C15279T,T16176C,C18348T,A23063T,C23271A,A23403G,C23604A,C23709T,T24506G,G24914C,C27972T,G28048T,A28111G,G28280C,A28281T,T28282A,G28881A,G28882A,G28883C,G28908T	20	N:D3L,N:R203K,N:G204R,N:G212V,N:S235F,ORF1a:T1001I,ORF1a:A1708D,ORF1a:I2230T,ORF1a:L3495F,ORF1b:P314L,ORF8:Q27*,ORF8:R52I,ORF8:Y73C,S:N501Y,S:A570D,S:D614G,S:P681H,S:T716I,S:S982A,S:D1118H	6	11
hCoV-19/Mexico/SIN_CIAD_HJ1603/2021	EPI_ISL_2249243	-	B.1.1.7	GRY	32	C241T,C913T,C3037T,C3267T,C5388A,C5986T,T6954C,C10748T,C14408T,C14649T,C14676T,C15279T,T16176C,C18348T,A23063T,C23271A,A23403G,C23604A,C23709T,T24506G,G24914C,C27972T,G28048T,A28111G,G28280C,A28281T,T28282A,G28881A,G28882A,G28883C,G28908T	20	N:D3L,N:R203K,N:G204R,N:G212V,N:S235F,ORF1a:T1001I,ORF1a:A1708D,ORF1a:I2230T,ORF1a:L3495F,ORF1b:P314L,ORF8:Q27*,ORF8:R52I,ORF8:Y73C,S:N501Y,S:A570D,S:D614G,S:P681H,S:T716I,S:S982A,S:D1118H	6	1
hCoV-19/Mexico/SIN_CIAD_HJ1616/2021	EPI_ISL_2249254	-	B.1.1.7	GRY	38	C241T,C913T,C3037T,C3267T,C5388A,C5986T,G6421T,T7204C,A8165T,C9298T,C14408T,C14676T,C15279T,T16176C,G16402A,A16403C,A17153G,C19554T,G22094A,A23063T,C23271A,A23403G,C23604A,C23709T,C24374T,T24506G,G24914C,C27972T,G28048T,A28111G,G28280C,A28281T,T28282A,G28774T,G28881A,G28882A,G28883C,C28977T	23	N:D3L,N:L167F,N:R203K,N:G204R,N:S235F,ORF1a:T1001I,ORF1a:A1708D,ORF1a:I2634F,ORF1b:P314L,ORF1b:D979T,ORF1b:Y1229C,ORF8:Q27*,ORF8:R52I,ORF8:Y73C,S:D178N,S:N501Y,S:A570D,S:D614G,S:P681H,S:T716I,S:L938F,S:S982A,S:D1118H	6	2
hCoV-19/Mexico/SIN_CIAD_ID2302/2021	EPI_ISL_2249234	-	B.1.1.7	GRY	33	C241T,C913T,C3037T,C3267T,C5388A,C5986T,T6954C,C9298T,C14408T,C14676T,C15279T,T16176C,A17153G,C19554T,A23063T,C23271A,A23403G,C23604A,C23709T,C24374T,T24506G,G24914C,C27972T,G28048T,A28111G,G28280C,A28281T,T28282A,G28774T,G28881A,G28882A,G	21	N:D3L,N:L167F,N:R203K,N:G204R,N:S235F,ORF1a:T1001I,ORF1a:A1708D,ORF1a:I2230T,ORF1b:P314L,ORF1b:Y1229C,ORF8:Q27*,ORF8:R52I,ORF8:Y73C,S:N501Y,S:A570D,S:D614G,S:P681H,S:T716I,S:L938F,S:S982A,S:D1118H	6	0
hCoV-19/Mexico/SIN_CIAD_S6572/2021	EPI_ISL_2026422	-	B.1.1.7	GR	38	C241T,C913T,C2453T,A2722G,C3037T,C3267T,G4256T,T4785C,C5388A,C5986T,T6954C,T9594C,C14408T,C14676T,C15279T,T16176C,C19206T,C21855T,A23063T,C23271A,A23403G,C23604A,C23709T,T24506G,G24914C,G25273T,C27972T,G28048T,A28111G,G28280C,A28281T,T28282A,A28295G,G28881A,G28882A,G28883C,C28977T,T29699G	25	N:D3L,N:N8D,N:R203K,N:G204R,N:S235F,ORF1a:L730F,ORF1a:T1001I,ORF1a:G1331C,ORF1a:L1507P,ORF1a:A1708D,ORF1a:I2230T,ORF1a:L3110S,ORF1b:P314L,ORF8:Q27*,ORF8:R52I,ORF8:Y73C,S:S98F,S:N501Y,S:A570D,S:D614G,S:P681H,S:T716I,S:S982A,S:D1118H,S:M1237I	1	2864
hCoV-19/Mexico/SIN_CIAD_S6600/2021	EPI_ISL_2026432	-	Q.3	GR	36	C241T,C913T,C3037T,C3267T,G5023A,C5388A,C5986T,C6539T,T6954C,G10396T,C14117T,C14408T,C14676T,C15279T,T16176C,A17615G,G20962A,A23063T,C23271A,A23403G,C23604A,C23709T,T24506G,G24914C,G26730C,C27972T,G28048T,A28111G,G28280C,A28281T,T28282A,G28881A,G28882A,G28883C,T28902C,C28977T	18	M:V70L,N:D3L,N:R203K,N:G204R,N:M210T,N:S235F,ORF1a:T1001I,ORF1a:M1586I,ORF1a:A1708D,ORF1a:H2092Y,ORF1a:I2230T,ORF1b:T217M,ORF1b:P314L,ORF1b:K1383R,ORF1b:D2499N,ORF8:Q27*,ORF8:R52I,ORF8:Y73C	3	0
hCoV-19/Mexico/SIN_CIAD_S6625/2021	EPI_ISL_2026420	-	B.1.1.7	GR	46	C241T,C913T,C2453T,C3037T,C3267T,T4861C,C5388A,C5986T,T6954C,C9166T,C9611T,T11288A,G11291A,T11296G,C14408T,C14676T,C15279T,T16176C,C18387T,A21766C,C21767T,A21768C,G21770A,C21855T,T21992G,A21993T,A23063T,C23271A,A23403G,C23604A,C23709T,T24506G,G24914C,T27215C,C27643T,C27972T,G28048T,A28111G,G28280C,A28281T,T28282A,A28295G,G28881A,G28882A,G28883C,C28977T	30	N:D3L,N:N8D,N:R203K,N:G204R,N:S235F,ORF1a:L730F,ORF1a:T1001I,ORF1a:A1708D,ORF1a:I2230T,ORF1a:L3116F,ORF1a:S3675T,ORF1a:G3676S,ORF1a:F3677L,ORF1b:P314L,ORF6:V5A,ORF7a:P84S,ORF8:Q27*,ORF8:R52I,ORF8:Y73C,S:H69S,S:V70I,S:S98F,S:Y144V,S:N501Y,S:A570D,S:D614G,S:P681H,S:T716I,S:S982A,S:D1118H	0	43
hCoV-19/Mexico/SIN_CIAD_S6643/2021	EPI_ISL_2026418	-	B.1.1.7	GRY	36	C241T,C913T,C2453T,C3037T,C3267T,C5388A,C5986T,T6954C,C9166T,C9611T,C14408T,C14676T,C15279T,T16176C,C18387T,C21855T,G21974C,A23063T,C23271A,A23403G,C23604A,C23709T,T24506G,G24914C,C27643T,C27972T,G28048T,A28111G,G28280C,A28281T,T28282A,A28295G,G28881A,G28882A,G28883C,C28977T	24	N:D3L,N:N8D,N:R203K,N:G204R,N:S235F,ORF1a:L730F,ORF1a:T1001I,ORF1a:A1708D,ORF1a:I2230T,ORF1a:L3116F,ORF1b:P314L,ORF7a:P84S,ORF8:Q27*,ORF8:R52I,ORF8:Y73C,S:S98F,S:D138H,S:N501Y,S:A570D,S:D614G,S:P681H,S:T716I,S:S982A,S:D1118H	6	33
hCoV-19/Mexico/SIN_CIAD_S6662/2021	EPI_ISL_2249230	-	B.1.1.7	GRY	36	C241T,C913T,C2453T,C3037T,C3267T,C5388A,C5986T,T6954C,C9166T,C9611T,C14408T,C14676T,C15279T,T16176C,G18255T,C21855T,G21974C,A23063T,C23271A,A23403G,C23604A,C23709T,T24506G,G24914C,C27643T,C27972T,G28048T,A28111G,G28280C,A28281T,T28282A,A28295G,G28881A,G28882A,G28883C,C28977T	25	N:D3L,N:N8D,N:R203K,N:G204R,N:S235F,ORF1a:L730F,ORF1a:T1001I,ORF1a:A1708D,ORF1a:I2230T,ORF1a:L3116F,ORF1b:P314L,ORF1b:M1596I,ORF7a:P84S,ORF8:Q27*,ORF8:R52I,ORF8:Y73C,S:S98F,S:D138H,S:N501Y,S:A570D,S:D614G,S:P681H,S:T716I,S:S982A,S:D1118H	6	0
hCoV-19/Mexico/SIN_CIAD_S6698/2021	EPI_ISL_2249236	-	B.1.1.7	GRY	36	C241T,C913T,C2453T,C3037T,C3267T,C5388A,C5986T,T6954C,C9611T,C14408T,C14676T,C15279T,T16176C,C21855T,G21974C,A23063T,C23271A,A23403G,C23604A,C23709T,T24506G,G24914C,G25273T,C27972T,G28048T,A28111G,G28280C,A28281T,T28282A,A28295G,A28877T,G28878C,G28881A,G28882A,G28883C,C28977T	24	N:D3L,N:N8D,N:R203K,N:G204R,N:S235F,ORF1a:L730F,ORF1a:T1001I,ORF1a:A1708D,ORF1a:I2230T,ORF1a:L3116F,ORF1b:P314L,ORF8:Q27*,ORF8:R52I,ORF8:Y73C,S:S98F,S:D138H,S:N501Y,S:A570D,S:D614G,S:P681H,S:T716I,S:S982A,S:D1118H,S:M1237I	6	82
hCoV-19/Mexico/SIN_CIAD_S6699/2021	EPI_ISL_2277642	-	B.1.1.7	GRY	36	C241T,C913T,C2453T,C3037T,C3267T,C5388A,C5986T,T6954C,C9611T,C14408T,C14676T,C15279T,T16176C,C21855T,G21974C,A23063T,C23271A,A23403G,C23604A,C23709T,T24506G,G24914C,G25273T,C27972T,G28048T,A28111G,G28280C,A28281T,T28282A,A28295G,A28877T,G28878C,G28881A,G28882A,G28883C,C28977T	24	N:D3L,N:N8D,N:R203K,N:G204R,N:S235F,ORF1a:L730F,ORF1a:T1001I,ORF1a:A1708D,ORF1a:I2230T,ORF1a:L3116F,ORF1b:P314L,ORF8:Q27*,ORF8:R52I,ORF8:Y73C,S:S98F,S:D138H,S:N501Y,S:A570D,S:D614G,S:P681H,S:T716I,S:S982A,S:D1118H,S:M1237I	6	48
hCoV-19/Mexico/SIN_CIAD_S6706/2021	EPI_ISL_2249240	-	B.1.1.7	GRY	36	C241T,C913T,C3037T,C3267T,C5388A,C5986T,G6421T,T6954C,T7204C,C9298T,C14408T,C14676T,C15279T,T16176C,A17153G,C19554T,G22094A,A23063T,C23271A,A23403G,C23604A,C23709T,C24374T,T24506G,G24914C,C27972T,G28048T,A28111G,G28280C,A28281T,T28282A,G28774T,G28881A,G28882A,G28883C,C28977T	22	N:D3L,N:L167F,N:R203K,N:G204R,N:S235F,ORF1a:T1001I,ORF1a:A1708D,ORF1a:I2230T,ORF1b:P314L,ORF1b:Y1229C,ORF8:Q27*,ORF8:R52I,ORF8:Y73C,S:D178N,S:N501Y,S:A570D,S:D614G,S:P681H,S:T716I,S:L938F,S:S982A,S:D1118H	6	1
hCoV-19/Mexico/SIN_CIAD_S6741/2021	EPI_ISL_2249251	-	B.1.1.7	GRY	40	C241T,C512T,C913T,C2453T,G2527T,C3037T,C3267T,C3990T,C5388A,C5986T,T6954C,C9611T,C14408T,C14676T,C15279T,T16176C,C21855T,G21974C,A23063T,C23271A,A23403G,C23604A,C23709T,T24506G,G24914C,G25273T,C27972T,G28048T,A28111G,G28280C,A28281T,T28282A,A28295G,A28877T,G28878C,G28881A,G28882A,G28883C,C28977T,A29796T	27	N:D3L,N:N8D,N:R203K,N:G204R,N:S235F,ORF1a:H83Y,ORF1a:L730F,ORF1a:E754D,ORF1a:T1001I,ORF1a:T1242I,ORF1a:A1708D,ORF1a:I2230T,ORF1a:L3116F,ORF1b:P314L,ORF8:Q27*,ORF8:R52I,ORF8:Y73C,S:S98F,S:D138H,S:N501Y,S:A570D,S:D614G,S:P681H,S:T716I,S:S982A,S:D1118H,S:M1237I	6	0
hCoV-19/Mexico/SIN-InDRE_FB14031_S2069/2021	EPI_ISL_2283709	-	B.1.1.7	GRY	31	C241T,C913T,C3037T,C3267T,A3492G,C5388A,C5986T,T6954C,C14408T,C14676T,C15279T,T16176C,C21639A,A23063T,C23271A,A23403G,C23604A,C23709T,C24374T,T24506G,G24914C,C27972T,G28048T,A28111G,G28280C,A28281T,T28282A,G28881A,G28882A,G28883C,C28977T	21	N:D3L,N:R203K,N:G204R,N:S235F,ORF1a:T1001I,ORF1a:N1076S,ORF1a:A1708D,ORF1a:I2230T,ORF1b:P314L,ORF8:Q27*,ORF8:R52I,ORF8:Y73C,S:P26H,S:N501Y,S:A570D,S:D614G,S:P681H,S:T716I,S:L938F,S:S982A,S:D1118H	6	0
hCoV-19/Mexico/SIN-InDRE_FB14032_S2070/2021	EPI_ISL_2283710	-	B.1.1.7	GRY	31	C241T,C913T,C3037T,C3267T,A3492G,C5388A,C5986T,T6954C,C14408T,C14676T,C15279T,T16176C,C21639A,A23063T,C23271A,A23403G,C23604A,C23709T,C24374T,T24506G,G24914C,C27972T,G28048T,A28111G,G28280C,A28281T,T28282A,G28881A,G28882A,G28883C,C28977T	21	N:D3L,N:R203K,N:G204R,N:S235F,ORF1a:T1001I,ORF1a:N1076S,ORF1a:A1708D,ORF1a:I2230T,ORF1b:P314L,ORF8:Q27*,ORF8:R52I,ORF8:Y73C,S:P26H,S:N501Y,S:A570D,S:D614G,S:P681H,S:T716I,S:L938F,S:S982A,S:D1118H	6	0
hCoV-19/Mexico/SIN-InDRE_FB14033_S1757/2021	EPI_ISL_2101896	-	B.1.1.7	GRY	34	C241T,C913T,C2143T,C3037T,C3267T,C5388A,C5986T,T6954C,C14408T,C14676T,T15096C,C15279T,T16176C,A23063T,C23271A,A23403G,C23604A,C23709T,T24506G,G24914C,G25785T,C27476T,C27972T,G28048T,A28095T,A28111G,G28280C,A28281T,T28282A,G28881A,G28882A,G	21	N:D3L,N:R203K,N:G204R,N:S235F,ORF1a:T1001I,ORF1a:A1708D,ORF1a:I2230T,ORF1b:P314L,ORF3a:W131C,ORF7a:T28I,ORF8:Q27*,ORF8:R52I,ORF8:K68*,ORF8:Y73C,S:N501Y,S:A570D,S:D614G,S:P681H,S:T716I,S:S982A,S:D1118H	6	0
hCoV-19/Mexico/SIN-InDRE_FB15070_S2058/2021	EPI_ISL_2283700	-	B.1.1.7	GRY	37	C241T,C913T,C2110T,C2544T,C3037T,C3267T,C5388A,C5986T,T6954C,C12809T,C14120T,C14178T,C14408T,A14493G,C14676T,C15279T,T16176C,A23063T,C23271A,A23403G,C23604A,C23709T,T24506G,G24914C,C26388T,T26604C,C27972T,G28048T,A28095T,A28111G,G28280C,A28281T,T28282A,G28881A,G28882A,G28883C,C28977T	23	M:F28L,N:D3L,N:R203K,N:G204R,N:S235F,ORF1a:T760I,ORF1a:T1001I,ORF1a:A1708D,ORF1a:I2230T,ORF1a:L4182F,ORF1b:P218L,ORF1b:P314L,ORF8:Q27*,ORF8:R52I,ORF8:K68*,ORF8:Y73C,S:N501Y,S:A570D,S:D614G,S:P681H,S:T716I,S:S982A,S:D1118H	6	7

hCoV-19/Mexico/SIN-InDRE_FB15071_S2059/2021	EPI_ISL_2283701	-	B.1.1.7	GRY	39	C241T,C913T,C2453T,G2967A,C3037T,C3267T,C5388A,C5986T,T6954C,C9611T,C12415T,C14408T,C14676T,C15279T,T16176C,G20580A,C21855T,G21974C,A23063T,C23271A,A23403G,C23604A,C23709T,T24506G,G24914C,C27972T,G28048T,A28111G,G28280C,A28281T,T28282A,A28295G,C28606T,G28881A,G28882A,G28883C,C28977T,G29862T,A29866C	24	N:D3L,N:N8D,N:R203K,N:G204R,N:S235F,ORF1a:L730F,ORF1a:S901N,ORF1a:T1001I,ORF1a:A1708D,ORF1a:I2230T,ORF1a:L3116F,ORF1b:P314L,ORF8:Q27*,ORF8:R52I,ORF8:Y73C,S:S98F,S:D138H,S:N501Y,S:A570D,S:D614G,S:P681H,S:T716I,S:S982A,S:D1118H	6	0
hCoV-19/Mexico/SIN-InDRE_FB15074_S2060/2021	EPI_ISL_2283702	-	B.1.1.7	GRY	36	C241T,C913T,C2453T,C3037T,C3267T,C5388A,C5986T,T6954C,C9611T,C12415T,C14408T,C14676T,C15279T,T16176C,G20580A,C21855T,G21974C,A23063T,C23271A,A23403G,C23604A,C23709T,T24506G,G24914C,C27972T,G28048T,A28111G,G28280C,A28281T,T28282A,A28295G,C28606T,G28881A,G28882A,G28883C,C28977T	23	N:D3L,N:N8D,N:R203K,N:G204R,N:S235F,ORF1a:L730F,ORF1a:T1001I,ORF1a:A1708D,ORF1a:I2230T,ORF1a:L3116F,ORF1b:P314L,ORF8:Q27*,ORF8:R52I,ORF8:Y73C,S:S98F,S:D138H,S:N501Y,S:A570D,S:D614G,S:P681H,S:T716I,S:S982A,S:D1118H	6	2
hCoV-19/Mexico/SIN-InDRE_FB15075_S2061/2021	EPI_ISL_2283703	-	B.1.1.7	GRY	36	C241T,C913T,C3037T,C3267T,C5388A,C5986T,T6954C,C8950T,C14408T,C14676T,C15279T,T16176C,C19524T,C21234T,C21575T,A23063T,C23271A,A23403G,C23604A,C23709T,T24506G,G24914C,C25844T,C27577T,G27670T,C27972T,G28048T,A28111G,G28280C,A28281T,T28282A,G28881A,G28882A,G28883C,C28977T,T29524C	22	N:D3L,N:R203K,N:G204R,N:S235F,ORF1a:T1001I,ORF1a:A1708D,ORF1a:I2230T,ORF1b:P314L,ORF3a:T151I,ORF7a:Q62*,ORF7a:V93F,ORF8:Q27*,ORF8:R52I,ORF8:Y73C,S:L5F,S:N501Y,S:A570D,S:D614G,S:P681H,S:T716I,S:S982A,S:D1118H	6	4
hCoV-19/Mexico/SIN-InDRE_FB16917_S3067/2021	EPI_ISL_2663333	-	B.1.1.7	GRY	36	C241T,C913T,G1306T,C3037T,C3267T,A3492G,C5388A,C5986T,G6901A,T6954C,C14408T,C14676T,C15279T,T16176C,C21639A,G22801T,A23063T,C23271A,A23403G,C23604A,C23709T,C24374T,T24506G,G24914C,A25336C,A25998G,C27972T,G28048T,A28111G,G28280C,A28281T,T28282A,G28881A,G28882A,G28883C,C28977T	23	N:D3L,N:R203K,N:G204R,N:S235F,ORF1a:E347D,ORF1a:T1001I,ORF1a:N1076S,ORF1a:A1708D,ORF1a:I2230T,ORF1b:P314L,ORF8:Q27*,ORF8:R52I,ORF8:Y73C,S:P26H,S:N501Y,S:A570D,S:D614G,S:P681H,S:T716I,S:L938F,S:S982A,S:D1118H,S:E1258D	6	45
hCoV-19/Mexico/SIN-InDRE_FB16918_S3068/2021	EPI_ISL_2663334	-	B.1.1.7	GRY	35	C241T,C706T,C913T,C3037T,C3267T,A3492G,C5388A,C5986T,T6954C,C10834T,C14408T,C14676T,C15279T,T16176C,C21639A,C21846T,A23063T,C23271A,A23403G,C23604A,C23709T,C24374T,T24506G,G24914C,A25164T,C27972T,G28048T,A28111G,G28280C,A28281T,T28282A,G28881A,G28882A,G28883C,C28977T	23	N:D3L,N:R203K,N:G204R,N:S235F,ORF1a:T1001I,ORF1a:N1076S,ORF1a:A1708D,ORF1a:I2230T,ORF1b:P314L,ORF8:Q27*,ORF8:R52I,ORF8:Y73C,S:P26H,S:T95I,S:N501Y,S:A570D,S:D614G,S:P681H,S:T716I,S:L938F,S:S982A,S:D1118H,S:Q1201L	6	530
hCoV-19/Mexico/SIN-InDRE_FB17388_S3483/2021	EPI_ISL_2778998	-	B.1.1.7	GRY	35	C241T,C245T,C913T,C3037T,C3267T,A3492G,C5388A,C5986T,T6954C,C14408T,C14676T,C15279T,T16176C,C21639A,A23063T,C23271A,A23403G,C23604A,C23709T,C24374T,T24506G,G24914C,G25334C,A25336C,G25337C,C27972T,G28048T,A28111G,G28280C,A28281T,T28282A,G28881A,G28882A,G28883C,C28977T	23	N:D3L,N:R203K,N:G204R,N:S235F,ORF1a:T1001I,ORF1a:N1076S,ORF1a:A1708D,ORF1a:I2230T,ORF1b:P314L,ORF8:Q27*,ORF8:R52I,ORF8:Y73C,S:P26H,S:N501Y,S:A570D,S:D614G,S:P681H,S:T716I,S:L938F,S:S982A,S:D1118H,S:E1258H,S:D1259H	6	0
hCoV-19/Mexico/SIN-InDRE_FB17473_S3490/2021	EPI_ISL_2779005	-	B.1.1.7	GRY	38	C241T,C913T,C3037T,C3267T,C5388A,C5986T,C6525T,T6954C,C9298T,A10807G,G13385T,C14408T,C14676T,C15279T,T16176C,A18802G,A23063T,C23271A,A23403G,C23604A,C23709T,C24374T,T24506G,G24914C,A25336C,G25337C,T26486C,C27972T,G28048T,A28111G,G28280C,A28281T,T28282A,G28881A,G28882A,G28883C,C28977T,C29144T	24	N:D3L,N:R203K,N:G204R,N:S235F,ORF1a:T1001I,ORF1a:A1708D,ORF1a:T2087I,ORF1a:I2230T,ORF1a:G4374C,ORF1b:P314L,ORF1b:S1779G,ORF8:Q27*,ORF8:R52I,ORF8:Y73C,S:N501Y,S:A570D,S:D614G,S:P681H,S:T716I,S:L938F,S:S982A,S:D1118H,S:E1258D,S:D1259H	6	0
hCoV-19/Mexico/SIN-InDRE_FB17477_S3491/2021	EPI_ISL_2779006	-	B.1.1.7	GRY	39	C241T,C913T,C3037T,C3267T,C5388A,C5812T,C5986T,T6954C,C8950T,C14408T,C14676T,C15279T,T16176C,C20594T,C21234T,C21575T,A23063T,C23271A,A23403G,C23604A,C23709T,T24506G,G24914C,A25336C,G25337C,C25844T,C27577T,G27670T,C27972T,G28048T,A28111G,G28280C,A28281T,T28282A,G28881A,G28882A,G28883C,C28977T,T29524C	25	N:D3L,N:R203K,N:G204R,N:S235F,ORF1a:T1001I,ORF1a:A1708D,ORF1a:I2230T,ORF1b:P314L,ORF1b:T2376I,ORF3a:T151I,ORF7a:Q62*,ORF7a:V93F,ORF8:Q27*,ORF8:R52I,ORF8:Y73C,S:L5F,S:N501Y,S:A570D,S:D614G,S:P681H,S:T716I,S:S982A,S:D1118H,S:E1258D,S:D1259H	6	0
hCoV-19/Mexico/SIN-InDRE_FB17478_S3492/2021	EPI_ISL_2779007	-	B.1.1.7	GRY	35	C241T,C913T,C3037T,C3267T,A3492G,C5388A,C5986T,T6954C,C10332T,C14408T,C14676T,C15279T,T16176C,C21639A,A23063T,C23271A,A23403G,C23604A,C23709T,C24374T,T24506G,G24914C,A25164T,A25336C,C25728T,C27972T,G28048T,A28111G,G28280C,A28281T,T28282A,G28881A,G28882A,G28883C,C28977T	24	N:D3L,N:R203K,N:G204R,N:S235F,ORF1a:T1001I,ORF1a:N1076S,ORF1a:A1708D,ORF1a:I2230T,ORF1a:T3356I,ORF1b:P314L,ORF8:Q27*,ORF8:R52I,ORF8:Y73C,S:P26H,S:N501Y,S:A570D,S:D614G,S:P681H,S:T716I,S:L938F,S:S982A,S:D1118H,S:Q1201L,S:E1258D	6	0
hCoV-19/Mexico/SIN-InDRE_FB18120_S3959/2021	EPI_ISL_2858973	-	B.1.1.7	GRY	35	C241T,C913T,C2453T,C3037T,C3267T,C5388A,A5570G,C5986T,T6954C,C9611T,C14408T,C14676T,C15279T,T16176C,C21855T,G21974C,A23063T,C23271A,A23403G,C23604A,C23709T,T24506G,G24914C,G25273T,C27972T,G28048T,A28111G,G28280C,A28281T,T28282A,A28295G,G28881A,G28882A,G28883C,C28977T	25	N:D3L,N:N8D,N:R203K,N:G204R,N:S235F,ORF1a:L730F,ORF1a:T1001I,ORF1a:A1708D,ORF1a:M1769V,ORF1a:I2230T,ORF1a:L3116F,ORF1b:P314L,ORF8:Q27*,ORF8:R52I,ORF8:Y73C,S:S98F,S:D138H,S:N501Y,S:A570D,S:D614G,S:P681H,S:T716I,S:S982A,S:D1118H,S:M1237I	6	0
hCoV-19/Mexico/SIN-InDRE_FB19379_S4505/2021	EPI_ISL_3033359	-	B.1.1.7	GRY	32	C241T,C913T,C3037T,C3267T,C5388A,C5986T,T6954C,C14408T,C14676T,C15279T,T16176C,G19677T,A23063T,C23086T,C23271A,A23403G,C23604A,C23709T,T24506G,G24914C,C26833T,C27972T,G28048T,A28111G,G28280C,A28281T,T28282A,G28881A,G28882A,G28883C,C28977T,	20	M:A104V,N:D3L,N:R203K,N:G204R,N:S235F,ORF1a:T1001I,ORF1a:A1708D,ORF1a:I2230T,ORF1b:P314L,ORF1b:Q2070H,ORF8:Q27*,ORF8:R52I,ORF8:Y73C,S:N501Y,S:A570D,S:D614G,S:P681H,S:T716I,S:S982A,S:D1118H	6	0
hCoV-19/Mexico/SIN-InDRE_FB19380_S4506/2021	EPI_ISL_3033360	-	B.1.1.7	GRY	33	C241T,C913T,C3037T,C3267T,A3492G,C5388A,C5986T,T6954C,T11186C,C14408T,C14676T,C15279T,T16176C,C21639A,C22326T,A23063T,C23271A,A23403G,C23604A,C23709T,C24374T,T24506G,G24914C,C27972T,G28048T,A28111G,G28280C,A28281T,T28282A,G28881A,G28882A,G	22	N:D3L,N:R203K,N:G204R,N:S235F,ORF1a:T1001I,ORF1a:N1076S,ORF1a:A1708D,ORF1a:I2230T,ORF1b:P314L,ORF8:Q27*,ORF8:R52I,ORF8:Y73C,S:P26H,S:S255F,S:N501Y,S:A570D,S:D614G,S:P681H,S:T716I,S:L938F,S:S982A,S:D1118H	6	0
hCoV-19/Mexico/SLP-InDRE_FB16030_S2429/2021	EPI_ISL_2455953	-	B.1.1.7	GRY	40	C222T,C241T,C913T,C2110T,C3037T,C3267T,C3743T,C5388A,C5986T,T6954C,G7042T,C14120T,C14408T,C14676T,A15046G,C15279T,T16176C,C17762T,C21846T,A23063T,C23271A,A23403G,C23604A,C23709T,T24506G,G24914C,T25180C,G25311T,A25336C,C27741T,C27972T,G28048T,A28111G,G28280C,A28281T,T28282A,G28881A,G28882A,G28883C,C28977T	26	N:D3L,N:R203K,N:G204R,N:S235F,ORF1a:T1001I,ORF1a:H1160Y,ORF1a:A1708D,ORF1a:I2230T,ORF1a:M2259I,ORF1b:P218L,ORF1b:P314L,ORF1b:I527V,ORF1b:A1432V,ORF8:Q27*,ORF8:R52I,ORF8:Y73C,S:T95I,S:N501Y,S:A570D,S:D614G,S:P681H,S:T716I,S:S982A,S:D1118H,S:C1250F,S:E1258D	6	3
hCoV-19/Mexico/SLP-InDRE_FB16823_S2780/2021	EPI_ISL_2533806	-	B.1.1.7	GRY	36	C241T,C751T,C913T,C2110T,C3037T,C3267T,C5388A,C5986T,T6954C,G7042T,G9929A,A10195G,C14120T,C14408T,C14676T,C15279T,T16176C,A23063T,C23271A,A23403G,C23604A,C23709T,T24506G,G24914C,C27972T,G28048T,G28075T,A28111G,G28280C,A28281T,T28282A,T28688C,G28881A,G28882A,G28883C,C28977T	22	N:D3L,N:R203K,N:G204R,N:S235F,ORF1a:T1001I,ORF1a:A1708D,ORF1a:I2230T,ORF1a:M2259I,ORF1a:D3222N,ORF1b:P218L,ORF1b:P314L,ORF8:Q27*,ORF8:R52I,ORF8:C61F,ORF8:Y73C,S:N501Y,S:A570D,S:D614G,S:P681H,S:T716I,S:S982A,S:D1118H	6	0
hCoV-19/Mexico/SLP-InDRE_FB18337_S3973/2021	EPI_ISL_2858987	-	B.1.1.7	GRY	39	C241T,G896A,C913T,C3037T,C3267T,C5388A,C5986T,C6706T,T6954C,C10279T,C12295T,C14408T,C14676T,C15279T,T16176C,C16468T,C16887T,A17615G,G18891T,A23063T,C23271A,A23403G,C23604A,C23709T,T24506G,G24914C,C25904T,G27703T,C27972T,G28048T,A28111G,G28280C,A28281T,T28282A,G28881A,G28882A,G28883C,C28977T,C29409T	25	N:D3L,N:R203K,N:G204R,N:S235F,N:T379I,ORF1a:A211T,ORF1a:T1001I,ORF1a:A1708D,ORF1a:I2230T,ORF1b:P314L,ORF1b:P1001S,ORF1b:K1383R,ORF1b:E1808D,ORF3a:S171L,ORF7a:V104F,ORF8:Q27*,ORF8:R52I,ORF8:Y73C,S:N501Y,S:A570D,S:D614G,S:P681H,S:T716I,S:S982A,S:D1118H	6	4
hCoV-19/Mexico/SLP-InDRE_FB18338_S3974/2021	EPI_ISL_2858988	-	B.1.1.7	GRY	40	C241T,G896A,C913T,C3037T,C3267T,C5388A,C5986T,C6706T,T6954C,C10279T,C12295T,C14408T,C14676T,C15279T,T16176C,C16468T,C16887T,A17615G,G18891T,A23063T,C23271A,A23403G,C23604A,C23709T,T24506G,G24914C,A25336C,C25904T,G27703T,C27972T,G28048T,A28111G,G28280C,A28281T,T28282A,G28881A,G28882A,G28883C,C28977T,C29409T	26	N:D3L,N:R203K,N:G204R,N:S235F,N:T379I,ORF1a:A211T,ORF1a:T1001I,ORF1a:A1708D,ORF1a:I2230T,ORF1b:P314L,ORF1b:P1001S,ORF1b:K1383R,ORF1b:E1808D,ORF3a:S171L,ORF7a:V104F,ORF8:Q27*,ORF8:R52I,ORF8:Y73C,S:N501Y,S:A570D,S:D614G,S:P681H,S:T716I,S:S982A,S:D1118H,S:E1258D	6	4
hCoV-19/Mexico/SLP-InDRE_FB18343_S3975/2021	EPI_ISL_2858989	-	B.1.1.7	GRY	40	C241T,C913T,C2110T,C3037T,C3267T,C5388A,C5986T,C6120T,T6954C,G7042T,A10195G,C14120T,C14408T,C14676T,C15279T,C15933T,T16176C,C21597T,A23063T,C23271A,A23403G,C23604A,C23709T,T24506G,G24914C,A25336C,G25855T,A27745G,G27762T,C27972T,G28048T,G28075T,A28111G,G28280C,A28281T,T28282A,G28881A,G28882A,G28883C,C28977T	27	N:D3L,N:R203K,N:G204R,N:S235F,ORF1a:T1001I,ORF1a:A1708D,ORF1a:S1952L,ORF1a:I2230T,ORF1a:M2259I,ORF1b:P218L,ORF1b:P314L,ORF3a:D155Y,ORF7a:R118G,ORF7b:E3*,ORF8:Q27*,ORF8:R52I,ORF8:C61F,ORF8:Y73C,S:S12F,S:N501Y,S:A570D,S:D614G,S:P681H,S:T716I,S:S982A,S:D1118H,S:E1258D	6	0
hCoV-19/Mexico/SLP-InDRE_FB18344_S3976/2021	EPI_ISL_2858990	-	B.1.1.7	GRY	40	C241T,C906T,C913T,C2147A,C3037T,C3267T,A5111G,C5388A,C5986T,T6954C,A12162G,C14408T,C14676T,C15279T,C15720T,G16122T,T16176C,A17615G,G20578T,A21137G,A23063T,C23271A,A23403G,C23604A,C23709T,T24506G,G24914C,G25135T,A25336C,C26110T,C27972T,G28048T,A28111G,G28280C,A28281T,T28282A,G28881A,G28882A,G28883C,C28977T	29	N:D3L,N:R203K,N:G204R,N:S235F,ORF1a:T214I,ORF1a:L628I,ORF1a:T1001I,ORF1a:K1616E,ORF1a:A1708D,ORF1a:I2230T,ORF1a:Q3966R,ORF1b:P314L,ORF1b:E885D,ORF1b:K1383R,ORF1b:V2371L,ORF1b:K2557R,ORF3a:P240S,ORF8:Q27*,ORF8:R52I,ORF8:Y73C,S:N501Y,S:A570D,S:D614G,S:P681H,S:T716I,S:S982A,S:D1118H,S:K1191N,S:E1258D	6	0
hCoV-19/Mexico/SON-H-3296/2021	EPI_ISL_2894601	-	B.1.1.7	G	22	C241T,C913T,C3037T,C3267T,C5388A,C6525T,A10807G,C14408T,C14676T,C15279T,T16176C,C23271A,A23403G,C23709T,C24374T,T24506G,G24914C,A28111G,G28280C,A28281T,T28282A,	12	N:D3L,ORF1a:T1001I,ORF1a:A1708D,ORF1a:T2087I,ORF1b:P314L,ORF8:Y73C,S:A570D,S:D614G,S:T716I,S:L938F,S:S982A,S:D1118H	6	4675
hCoV-19/Mexico/SON-InDRE_FB13111_S1494/2021	EPI_ISL_1805472	-	B.1.1.7	GRY	41	C203T,C241T,C913T,G1820A,C3037T,C3267T,C5388A,C5986T,T6954C,G9526T,C11171T,A12162G,C14408T,C14676T,C15279T,C15720T,T16176C,A17615G,T19363C,A21137G,A23063T,C23248T,C23271A,A23403G,C23604A,C23709T,T24506G,G24914C,G25135T,A25336C,G25337C,C27972T,G28048T,A28111G,G28280C,A28281T,T28282A,G28881A,G28882A,G28883C,C28977T	27	N:D3L,N:R203K,N:G204R,N:S235F,ORF1a:G519S,ORF1a:T1001I,ORF1a:A1708D,ORF1a:I2230T,ORF1a:M3087I,ORF1a:L3636F,ORF1a:Q3966R,ORF1b:P314L,ORF1b:K1383R,ORF1b:K2557R,ORF8:Q27*,ORF8:R52I,ORF8:Y73C,S:N501Y,S:A570D,S:D614G,S:P681H,S:T716I,S:S982A,S:D1118H,S:K1191N,S:E1258D,S:D1	6	0

hCoV-19/Mexico/SON-InDRE_FB14085_S1758/2021	EPI_ISL_2101897	-	B.1.1.7	GRY	32	C241T,C913T,C3037T,C3267T,C5388A,C5986T,C6525T,T6954C,C9298T,C14408T,C14676T,C15279T,T16176C,A23063T,C23271A,A23403G,C23604A,C23709T,C24374T,T24506G,G24914C,T26486C,C27972T,G28048T,A28111G,G28280C,A28281T,T28282A,G28881A,G28882A,G28883C,C2	20	N:D3L,N:R203K,N:G204R,N:S235F,ORF1a:T1001I,ORF1a:A1708D,ORF1a:T2087I,ORF1a:I2230T,ORF1b:P314L,ORF8:Q27*,ORF8:R52I,ORF8:Y73C,S:N501Y,S:A570D,S:D614G,S:P681H,S:T716I,S:L938F,S:S982A,S:D1118H	6	0
hCoV-19/Mexico/SON-InDRE_FB16350_S3474/2021	EPI_ISL_2778989	-	B.1.1.7	GRY	37	C241T,C913T,C3037T,C3267T,C5388A,C5986T,T6954C,C14408T,C14649T,C14676T,C15279T,T16176C,C16997T,C18348T,A23063T,C23271A,A23403G,C23604A,C23709T,T24506G,G24914C,A25336C,G25337C,G25767T,C27944T,C27972T,G28001T,G28048T,A28111G,G28280C,A28281T,T28282A,G28881A,G28882A,G28883C,C28977T,C29451T	23	N:D3L,N:R203K,N:G204R,N:S235F,N:T393I,ORF1a:T1001I,ORF1a:A1708D,ORF1a:I2230T,ORF1b:P314L,ORF1b:P1177L,ORF3a:M125I,ORF8:Q27*,ORF8:R52I,ORF8:Y73C,S:N501Y,S:A570D,S:D614G,S:P681H,S:T716I,S:S982A,S:D1118H,S:E1258D,S:D1259H	6	0
hCoV-19/Mexico/SON-InDRE_FB17150_S3136/2021	EPI_ISL_2663403	-	B.1.1.7	GRY	40	C203T,C241T,C913T,G1820A,C3037T,G3251T,C3267T,C5388A,C5986T,T6954C,G9526T,A12162G,C14408T,C14676T,C15279T,C15720T,T16176C,A17615G,T19363C,A21137G,A23063T,C23248T,C23271A,A23403G,C23604A,C23709T,T24506G,G24914C,G25135T,T26861C,C27972T,G28048T,A28111G,G28280C,A28281T,T28282A,G28881A,G28882A,G28883C,C28977T	25	N:D3L,N:R203K,N:G204R,N:S235F,ORF1a:G519S,ORF1a:D996Y,ORF1a:T1001I,ORF1a:A1708D,ORF1a:I2230T,ORF1a:M3087I,ORF1a:Q3966R,ORF1b:P314L,ORF1b:K1383R,ORF1b:K2557R,ORF8:Q27*,ORF8:R52I,ORF8:Y73C,S:N501Y,S:A570D,S:D614G,S:P681H,S:T716I,S:S982A,S:D1118H,S:K1191N	6	0
hCoV-19/Mexico/SON-InDRE_FB17167_S3092/2021	EPI_ISL_2663359	-	B.1.1.7	GRY	40	C203T,C241T,C913T,G1820A,C3037T,G3251T,C3267T,C5388A,C5986T,T6954C,G9526T,A12162G,C14408T,C14676T,C15279T,C15720T,T16176C,A17615G,T19363C,G20635C,A21137G,A23063T,C23248T,C23271A,A23403G,C23604A,C23709T,T24506G,G24914C,G25135T,C27972T,G28048T,A28111G,G28280C,A28281T,T28282A,G28881A,G28882A,G28883C,C28977T	26	N:D3L,N:R203K,N:G204R,N:S235F,ORF1a:G519S,ORF1a:D996Y,ORF1a:T1001I,ORF1a:A1708D,ORF1a:I2230T,ORF1a:M3087I,ORF1a:Q3966R,ORF1b:P314L,ORF1b:K1383R,ORF1b:E2390Q,ORF1b:K2557R,ORF8:Q27*,ORF8:R52I,ORF8:Y73C,S:N501Y,S:A570D,S:D614G,S:P681H,S:T716I,S:S982A,S:D1118H,S:K1191N	6	0
hCoV-19/Mexico/SON-InDRE_FB17171_S3087/2021	EPI_ISL_2663354	-	B.1.1.7	GRY	38	C241T,C913T,C3037T,C3267T,C5388A,C5986T,C6196T,T6954C,C7765T,A8848G,C14408T,C14676T,C15279T,T16176C,C16750T,G19816T,G22901A,A23063T,C23271A,A23403G,C23604A,C23709T,T24506G,G24914C,G25217T,G26727T,T27285C,C27972T,G28048T,A28111G,G28280C,A28281T,T28282A,G28881A,G28882A,G28883C,C28977T,G29715T	23	M:A69S,N:D3L,N:R203K,N:G204R,N:S235F,ORF1a:T1001I,ORF1a:A1708D,ORF1a:I2230T,ORF1b:P314L,ORF1b:P1095S,ORF1b:V2117L,ORF8:Q27*,ORF8:R52I,ORF8:Y73C,S:G447S,S:N501Y,S:A570D,S:D614G,S:P681H,S:T716I,S:S982A,S:D1118H,S:G1219C	6	0
hCoV-19/Mexico/SON-InDRE_FB17175_S3088/2021	EPI_ISL_2663355	-	B.1.1.7	GRY	33	C241T,C913T,C3037T,C3267T,A3845G,C5388A,C5986T,C6525T,T6954C,C9298T,C14408T,C14676T,C15279T,T16176C,A23063T,C23271A,A23403G,C23604A,C23709T,C24374T,T24506G,G24914C,T26486C,C27972T,G28048T,A28111G,G28280C,A28281T,T28282A,G28881A,G28882A,G288	21	N:D3L,N:R203K,N:G204R,N:S235F,ORF1a:T1001I,ORF1a:K1194E,ORF1a:A1708D,ORF1a:T2087I,ORF1a:I2230T,ORF1b:P314L,ORF8:Q27*,ORF8:R52I,ORF8:Y73C,S:N501Y,S:A570D,S:D614G,S:P681H,S:T716I,S:L938F,S:S982A,S:D1118H	6	0
hCoV-19/Mexico/SON-InDRE_FB18023_S3947/2021	EPI_ISL_2858961	-	B.1.1.7	GRY	35	C241T,C913T,C3037T,C3267T,C5388A,C5986T,C6525T,T6954C,C9298T,C14408T,C14676T,C15279T,T16176C,A23063T,C23271A,A23403G,C23604A,C23709T,C24374T,T24506G,G24914C,A25336C,G25337C,T26486C,C27972T,G28048T,A28111G,G28280C,A28281T,T28282A,G28881A,G28882A,G28883C,C28977T,C29144T	22	N:D3L,N:R203K,N:G204R,N:S235F,ORF1a:T1001I,ORF1a:A1708D,ORF1a:T2087I,ORF1a:I2230T,ORF1b:P314L,ORF8:Q27*,ORF8:R52I,ORF8:Y73C,S:N501Y,S:A570D,S:D614G,S:P681H,S:T716I,S:L938F,S:S982A,S:D1118H,S:E1258D,S:D1259H	6	0
hCoV-19/Mexico/SON-InDRE_FB18274_S4135/2021	EPI_ISL_2920706	-	B.1.1.7	GRY	42	C241T,C913T,C2110T,C3037T,C3177T,C3267T,C4890T,C5388A,C5986T,T6954C,G7042T,G9929A,A10195G,C12741T,C14120T,C14408T,C14676T,C15279T,T16176C,C20628G,G22104T,A23063T,C23271A,A23403G,C23604A,C23709T,T24506G,G24914C,C27972T,C28000A,G28048T,G28075T,A28095T,A28111G,G28280C,A28281T,T28282A,T28688C,G28881A,G28882A,G28883C,C2897	28	N:D3L,N:R203K,N:G204R,N:S235F,ORF1a:P971L,ORF1a:T1001I,ORF1a:T1542I,ORF1a:A1708D,ORF1a:I2230T,ORF1a:M2259I,ORF1a:D3222N,ORF1a:T4159I,ORF1b:P218L,ORF1b:P314L,ORF8:Q27*,ORF8:P36Q,ORF8:R52I,ORF8:C61F,ORF8:K68*,ORF8:Y73C,S:G181V,S:N501Y,S:A570D,S:D614G,S:P681H,S:T716I,S:S9	6	0
hCoV-19/Mexico/SON-InDRE_FB18291_S3967/2021	EPI_ISL_2858981	-	Q.3	GRY	37	C241T,C913T,C3037T,C3267T,G5023A,C5388A,C5986T,T6954C,A9085G,G10396T,C14408T,C14676T,C15279T,T16176C,A17615G,G20962A,A23063T,C23271A,A23403G,C23604A,C23709T,T24506G,G24914C,A25336C,G25987T,G26730C,C26826T,C27972T,G28048T,A28111G,G28280C,A28281T,T28282A,G28881A,G28882A,G28883C,C28977T	24	M:V70L,N:D3L,N:R203K,N:G204R,N:S235F,ORF1a:T1001I,ORF1a:M1586I,ORF1a:A1708D,ORF1a:I2230T,ORF1b:P314L,ORF1b:K1383R,ORF1b:D2499N,ORF3a:D199Y,ORF8:Q27*,ORF8:R52I,ORF8:Y73C,S:N501Y,S:A570D,S:D614G,S:P681H,S:T716I,S:S982A,S:D1118H,S:E1258D	6	0
hCoV-19/Mexico/SON-InDRE_FB18293_S3968/2021	EPI_ISL_2858982	-	B.1.1.7	GRY	33	C241T,C913T,C3037T,C3267T,A3492G,C5388A,C5986T,T6954C,C14408T,C14676T,C15279T,T16176C,C18804T,C21639A,A23063T,C23271A,A23403G,C23604A,C23709T,C24374T,T24506G,G24914C,A25164T,C27972T,G28048T,A28111G,G28280C,A28281T,T28282A,G28881A,G28882A,G	22	N:D3L,N:R203K,N:G204R,N:S235F,ORF1a:T1001I,ORF1a:N1076S,ORF1a:A1708D,ORF1a:I2230T,ORF1b:P314L,ORF8:Q27*,ORF8:R52I,ORF8:Y73C,S:P26H,S:N501Y,S:A570D,S:D614G,S:P681H,S:T716I,S:L938F,S:S982A,S:D1118H,S:Q1201	6	0
hCoV-19/Mexico/TAB-InDRE_FB16513_S2742/2021	EPI_ISL_2533768	-	B.1.1.7	GRY	37	C241T,C913T,C2110T,C3037T,C3267T,C5388A,C5986T,T6954C,G7042T,A10195G,C10279T,C14120T,C14408T,C14676T,C15279T,T16176C,C16887T,G17427T,A23063T,C23271A,A23403G,C23604A,C23709T,T24506G,G24914C,G25052T,C27972T,G28048T,G28075T,A28111G,G28280C,A28281T,T28282A,G28881A,G28882A,G28883C,C28977T	22	N:D3L,N:R203K,N:G204R,N:S235F,ORF1a:T1001I,ORF1a:A1708D,ORF1a:I2230T,ORF1a:M2259I,ORF1b:P218L,ORF1b:P314L,ORF8:Q27*,ORF8:R52I,ORF8:C61F,ORF8:Y73C,S:N501Y,S:A570D,S:D614G,S:P681H,S:T716I,S:S982A,S:D1118H,S:V1164F	6	0
hCoV-19/Mexico/TAB-InDRE_FB16514_S2743/2021	EPI_ISL_2533769	-	B.1.1.7	GRY	37	C241T,C913T,C2110T,C3037T,C3267T,C5388A,C5986T,T6954C,G7042T,A10195G,C10279T,C14120T,C14408T,C14676T,C15279T,T16176C,C16887T,G17427T,G21423A,A23063T,C23271A,A23403G,C23604A,C23709T,T24506G,G24914C,C27972T,G28048T,G28075T,A28111G,G28280C,A28281T,T28282A,G28881A,G28882A,G28883C,C28977T	21	N:D3L,N:R203K,N:G204R,N:S235F,ORF1a:T1001I,ORF1a:A1708D,ORF1a:I2230T,ORF1a:M2259I,ORF1b:P218L,ORF1b:P314L,ORF8:Q27*,ORF8:R52I,ORF8:C61F,ORF8:Y73C,S:N501Y,S:A570D,S:D614G,S:P681H,S:T716I,S:S982A,S:D1118H	6	0
hCoV-19/Mexico/TAB-InDRE_FB16926_S3069/2021	EPI_ISL_2663336	-	B.1.1.7	GRY	41	C241T,C913T,C2110T,C3037T,C3267T,C5388A,C5986T,T6954C,G7042T,G8174A,A10195G,C10279T,C14120T,C14408T,C14676T,C15279T,T16176C,C16887T,G17427T,A23063T,C23271A,A23403G,C23604A,C23709T,T24506G,G24914C,A25097G,A25336C,G25337C,C27972T,G28048T,G28075T,A28111G,G28280C,A28281T,T28282A,G28881A,G28882A,G28883C,C28977T,A29700G	25	N:D3L,N:R203K,N:G204R,N:S235F,ORF1a:T1001I,ORF1a:A1708D,ORF1a:I2230T,ORF1a:M2259I,ORF1a:A2637T,ORF1b:P218L,ORF1b:P314L,ORF8:Q27*,ORF8:R52I,ORF8:C61F,ORF8:Y73C,S:N501Y,S:A570D,S:D614G,S:P681H,S:T716I,S:S982A,S:D1118H,S:I1179V,S:E1258D,S:D1259H	6	0
hCoV-19/Mexico/TAB-InDRE_FB16929_S3070/2021	EPI_ISL_2663337	-	B.1.1.7	GRY	40	C241T,C913T,C2110T,C3037T,C3267T,C5388A,C5986T,T6954C,G7042T,A8031G,A10195G,C10279T,C14120T,C14408T,C14676T,C15279T,T16176C,C16887T,G17427T,A23063T,C23271A,A23403G,C23604A,C23709T,T24506G,G24914C,G25052T,A25336C,C27972T,G28048T,G28075T,A28111G,G28280C,A28281T,T28282A,G28881A,G28882A,G28883C,C28977T,C29085T	25	N:D3L,N:R203K,N:G204R,N:S235F,N:T271I,ORF1a:T1001I,ORF1a:A1708D,ORF1a:I2230T,ORF1a:M2259I,ORF1a:K2589R,ORF1b:P218L,ORF1b:P314L,ORF8:Q27*,ORF8:R52I,ORF8:C61F,ORF8:Y73C,S:N501Y,S:A570D,S:D614G,S:P681H,S:T716I,S:S982A,S:D1118H,S:V1164F,S:E1258D	6	0
hCoV-19/Mexico/TAB-InDRE_FB16931_S3071/2021	EPI_ISL_2663338	-	B.1.1.7	GRY	43	C241T,C913T,C3037T,C3267T,A3337C,A5229G,C5388A,C5986T,G7393T,A7863G,G8600T,G13858T,C14408T,C14676T,C15279T,T16176C,T17007C,A17615G,A23063T,A23118G,C23271A,A23403G,C23604A,C23709T,C24378T,T24506G,G24914C,A25336C,G25494T,C25587T,C25624T,G26167T,G26849T,C27972T,G28048T,A28111G,G28280C,A28281T,T28282A,G28881A,G28882A,G28883C,C28977T	29	M:M109I,N:D3L,N:R203K,N:G204R,N:S235F,ORF1a:T1001I,ORF1a:E1024D,ORF1a:K1655R,ORF1a:A1708D,ORF1a:K2533R,ORF1a:V2779L,ORF1b:D131Y,ORF1b:P314L,ORF1b:K1383R,ORF3a:H78Y,ORF3a:V259L,ORF8:Q27*,ORF8:R52I,ORF8:Y73C,S:N501Y,S:H519R,S:A570D,S:D614G,S:P681H,S:T716I,S:S939F,S:S982A,S:D1118H,S:E1258D	6	0
hCoV-19/Mexico/TAB-InDRE_FB16944_S3072/2021	EPI_ISL_2663339	-	B.1.1.7	GRY	37	T78G,C241T,C913T,C2110T,C3037T,C3267T,C5388A,C5986T,T6954C,G7042T,A10195G,C10279T,C14120T,C14408T,C14676T,C15279T,T16176C,C16887T,G17427T,A23063T,C23271A,A23403G,C23604A,C23709T,T24506G,G24914C,C27972T,G28048T,G28075T,A28111G,G28280C,A28281T,T28282A,G28881A,G28882A,G28883C,C28977T	21	N:D3L,N:R203K,N:G204R,N:S235F,ORF1a:T1001I,ORF1a:A1708D,ORF1a:I2230T,ORF1a:M2259I,ORF1b:P218L,ORF1b:P314L,ORF8:Q27*,ORF8:R52I,ORF8:C61F,ORF8:Y73C,S:N501Y,S:A570D,S:D614G,S:P681H,S:T716I,S:S982A,S:D1118H	6	2
hCoV-19/Mexico/TAB-InDRE_FB16947_S3073/2021	EPI_ISL_2663340	-	B.1.1.7	GRY	36	C241T,C913T,C3037T,C3267T,C5388A,C5986T,T6954C,C7564T,C9967T,C11195T,C14408T,C14676T,T15096C,C15279T,T16176C,G21901A,C22987T,A23063T,C23271A,A23403G,C23604A,C23709T,T24506G,G24914C,A25336C,C27972T,G28048T,A28111G,G28280C,A28281T,T28282A,G28881A,G28882A,G28883C,G28884C,C28977T	20	N:D3L,N:R203K,N:G204P,N:S235F,ORF1a:T1001I,ORF1a:A1708D,ORF1a:I2230T,ORF1a:L3644F,ORF1b:P314L,ORF8:Q27*,ORF8:R52I,ORF8:Y73C,S:N501Y,S:A570D,S:D614G,S:P681H,S:T716I,S:S982A,S:D1118H,S:E1258D	6	0
hCoV-19/Mexico/TAB-InDRE_FB16948_S3074/2021	EPI_ISL_2663341	-	B.1.1.7	GRY	34	C241T,C913T,C3037T,C3267T,C5388A,C5986T,C6196T,C6268T,T6954C,A8848G,C14408T,C14676T,C15279T,T16176C,A23063T,C23271A,A23403G,C23604A,C23709T,T24506G,G24914C,A25336C,G25337C,C27972T,G28048T,A28111G,G28280C,A28281T,T28282A,G28881A,G28882A,G2	20	N:D3L,N:R203K,N:G204R,N:S235F,ORF1a:T1001I,ORF1a:A1708D,ORF1a:I2230T,ORF1b:P314L,ORF8:Q27*,ORF8:R52I,ORF8:Y73C,S:N501Y,S:A570D,S:D614G,S:P681H,S:T716I,S:S982A,S:D1118H,S:E1258D,S:D1259H	6	0
hCoV-19/Mexico/TAB-InDRE_FB17013_S3079/2021	EPI_ISL_2663346	-	B.1.1.7	GRY	41	C241T,C913T,C2110T,C3037T,C3267T,C5388A,C5986T,T6954C,G7042T,A10195G,C10279T,A12555G,C14120T,C14408T,C14676T,C15180T,C15279T,T16176C,C16887T,G17427T,A23063T,C23271A,A23403G,C23604A,C23709T,C24023T,T24506G,G24914C,C27223T,C27972T,G28048T,G28075T,A28095T,A28111G,G28280C,A28281T,T28282A,G28881A,G28882A,G28883C,C28977T	24	N:D3L,N:R203K,N:G204R,N:S235F,ORF1a:T1001I,ORF1a:A1708D,ORF1a:I2230T,ORF1a:M2259I,ORF1a:E4097G,ORF1b:P218L,ORF1b:P314L,ORF6:Q8*,ORF8:Q27*,ORF8:R52I,ORF8:C61F,ORF8:K68*,ORF8:Y73C,S:N501Y,S:A570D,S:D614G,S:P681H,S:T716I,S:S982A,S:D1118H	6	0
hCoV-19/Mexico/TAB-InDRE_FB17014_S3080/2021	EPI_ISL_2663347	-	B.1.1.7	GRY	40	C241T,C913T,C1926T,C2110T,C3037T,C3267T,C5388A,C5986T,T6954C,G7042T,A10195G,C10279T,C14120T,C14408T,C14676T,C15279T,T16176C,C16887T,G17427T,A23063T,C23271A,A23403G,C23604A,C23709T,T24506G,G24914C,G25395T,A27943G,C27972T,G28048T,G28075T,A28095T,A28111G,G28280C,A28281T,T28282A,G28881A,G28882A,G28883C,C28977T	25	N:D3L,N:R203K,N:G204R,N:S235F,ORF1a:T554I,ORF1a:T1001I,ORF1a:A1708D,ORF1a:I2230T,ORF1a:M2259I,ORF1b:P218L,ORF1b:P314L,ORF3a:M1I,ORF8:H17R,ORF8:Q27*,ORF8:R52I,ORF8:C61F,ORF8:K68*,ORF8:Y73C,S:N501Y,S:A570D,S:D614G,S:P681H,S:T716I,S:S982A,S:D1118H	6	0

hCoV-19/Mexico/TAB-InDRE_FB17020_S3081/2021	EPI_ISL_2663348	-	B.1.1.7	GRY	41	C241T,C913T,C2110T,C3037T,C3267T,C4113T,C5388A,C5986T,T6954C,G7042T,G7295T,C9430T,A10195G,C10279T,C14120T,C14408T,C14676T,C15279T,T16176C,C16887T,G17427T,T18012C,A23063T,C23271A,A23403G,C23604A,C23709T,T24506G,G24914C,C27972T,G28048T,G28075T,A28095T,A28111G,G28280C,A28281T,T28282A,G28881A,G28882A,G28883C,C28977T	24	N:D3L,N:R203K,N:G204R,N:S235F,ORF1a:T1001I,ORF1a:A1283V,ORF1a:A1708D,ORF1a:I2230T,ORF1a:M2259I,ORF1a:A2344S,ORF1b:P218L,ORF1b:P314L,ORF8:Q27*,ORF8:R52I,ORF8:C61F,ORF8:K68*,ORF8:Y73C,S:N501Y,S:A570D,S:D614G,S:P681H,S:T716I,S:S982A,S:D1118H	6	0
hCoV-19/Mexico/TAB-InDRE_FB17030_S3084/2021	EPI_ISL_2663351	-	B.1.1.7	GRY	39	C241T,C913T,G1696A,C2110T,C3037T,C3267T,C5388A,C5986T,T6954C,G7042T,A10195G,C10279T,C12473T,C14120T,C14408T,C14676T,C15279T,T16176C,C16887T,G17427T,A23063T,C23271A,A23403G,C23604A,C23709T,T24506G,G24914C,C27972T,G28048T,G28075T,A28095T,A28111G,G28280C,A28281T,T28282A,G28881A,G28882A,G28883C,C28977T	22	N:D3L,N:R203K,N:G204R,N:S235F,ORF1a:T1001I,ORF1a:A1708D,ORF1a:I2230T,ORF1a:M2259I,ORF1b:P218L,ORF1b:P314L,ORF8:Q27*,ORF8:R52I,ORF8:C61F,ORF8:K68*,ORF8:Y73C,S:N501Y,S:A570D,S:D614G,S:P681H,S:T716I,S:S982A,S:D1118H	6	0
hCoV-19/Mexico/TAB-InDRE_FB17043_S3082/2021	EPI_ISL_2663349	-	B.1.1.7	GRY	42	C241T,C913T,C2110T,C3037T,C3267T,C4113T,C5388A,C5986T,T6954C,G7042T,C9430T,C9532T,A10195G,C10279T,C14120T,C14408T,C14676T,C15279T,T16176C,C16887T,T16944C,G17427T,C22088T,A23063T,C23271A,A23403G,C23604A,C23709T,T24506G,G24914C,C27972T,G28048T,G28075T,A28095T,A28111G,G28280C,A28281T,T28282A,G28881A,G28882A,G28883C,C2897	24	N:D3L,N:R203K,N:G204R,N:S235F,ORF1a:T1001I,ORF1a:A1283V,ORF1a:A1708D,ORF1a:I2230T,ORF1a:M2259I,ORF1b:P218L,ORF1b:P314L,ORF8:Q27*,ORF8:R52I,ORF8:C61F,ORF8:K68*,ORF8:Y73C,S:L176F,S:N501Y,S:A570D,S:D614G,S:P681H,S:T716I,S:S982A,S:D1118H	6	0
hCoV-19/Mexico/TAB-InDRE_FB17298_S3469/2021	EPI_ISL_2778984	-	B.1.1.7	GRY	40	C241T,C913T,C2110T,C3037T,C3267T,C5388A,C5986T,C6016T,T6954C,G7042T,C8917T,A10195G,C10279T,C14120T,C14408T,C14676T,C15279T,T16176C,C16887T,G17427T,A23063T,C23271A,A23403G,C23604A,C23709T,T24506G,G24914C,A25336C,G25337C,C27972T,G28048T,G28075T,A28111G,G28280C,A28281T,T28282A,G28881A,G28882A,G28883C,C28977T	23	N:D3L,N:R203K,N:G204R,N:S235F,ORF1a:T1001I,ORF1a:A1708D,ORF1a:I2230T,ORF1a:M2259I,ORF1b:P218L,ORF1b:P314L,ORF8:Q27*,ORF8:R52I,ORF8:C61F,ORF8:Y73C,S:N501Y,S:A570D,S:D614G,S:P681H,S:T716I,S:S982A,S:D1118H,S:E1258D,S:D1259H	6	0
hCoV-19/Mexico/TAB-InDRE_FB17299_S3470/2021	EPI_ISL_2778985	-	B.1.1.7	GRY	40	C241T,C913T,C3037T,C3267T,C5388A,C5986T,C6196T,C6268T,T6954C,A8315G,A8848G,C10125T,G10261A,C14408T,C14676T,C15279T,T16176C,A23063T,C23271A,A23403G,C23604A,C23709T,T24506G,G24914C,A25336C,G25337C,C25544T,C26801T,C27972T,G28048T,A28111G,T28245G,G28280C,A28281T,T28282A,G28881A,G28882A,G28883C,C28977T,G29715T	24	N:D3L,N:R203K,N:G204R,N:S235F,ORF1a:T1001I,ORF1a:A1708D,ORF1a:I2230T,ORF1a:T2684A,ORF1a:T3287I,ORF1b:P314L,ORF3a:A51V,ORF8:Q27*,ORF8:R52I,ORF8:Y73C,ORF8:L118V,S:N501Y,S:A570D,S:D614G,S:P681H,S:T716I,S:S982A,S:D1118H,S:E1258D,S:D1259H	6	0
hCoV-19/Mexico/TAB-InDRE_FB17300_S3471/2021	EPI_ISL_2778986	-	B.1.1.7	GRY	41	C241T,C913T,C3037T,C3267T,C5388A,C5986T,C6196T,C6268T,T6954C,A8315G,A8848G,C10125T,G10261A,C14408T,C14676T,C15279T,T16176C,A23063T,C23271A,A23403G,C23604A,C23709T,T24506G,G24914C,A25336C,G25337C,C25544T,C26801T,C27972T,G28048T,A28111G,T28245G,T28251C,G28280C,A28281T,T28282A,G28881A,G28882A,G28883C,C28977T,G29715T	25	N:D3L,N:R203K,N:G204R,N:S235F,ORF1a:T1001I,ORF1a:A1708D,ORF1a:I2230T,ORF1a:T2684A,ORF1a:T3287I,ORF1b:P314L,ORF3a:A51V,ORF8:Q27*,ORF8:R52I,ORF8:Y73C,ORF8:L118V,ORF8:F120L,S:N501Y,S:A570D,S:D614G,S:P681H,S:T716I,S:S982A,S:D1118H,S:E1258D,S:D1259H	6	0
hCoV-19/Mexico/TAB-InDRE_FB17301_S3472/2021	EPI_ISL_2778987	-	B.1.1.7	GRY	39	C241T,C913T,C2110T,C3037T,C3267T,C4084T,C5388A,C5986T,T6954C,G7042T,A10195G,C10279T,G13459A,C14120T,C14408T,C14676T,C15279T,T16176C,C16887T,G17427T,A23063T,C23271A,A23403G,C23604A,C23709T,T24506G,G24914C,A25336C,C27972T,G28048T,G28075T,A28111G,G28280C,A28281T,T28282A,G28881A,G28882A,G28883C,C28977T	22	N:D3L,N:R203K,N:G204R,N:S235F,ORF1a:T1001I,ORF1a:A1708D,ORF1a:I2230T,ORF1a:M2259I,ORF1b:P218L,ORF1b:P314L,ORF8:Q27*,ORF8:R52I,ORF8:C61F,ORF8:Y73C,S:N501Y,S:A570D,S:D614G,S:P681H,S:T716I,S:S982A,S:D1118H,S:E1258D	6	0
hCoV-19/Mexico/TAB-InDRE_FB17315_S3473/2021	EPI_ISL_2778988	-	B.1.1.7	GRY	38	C241T,C913T,C2110T,C3037T,C3267T,C5388A,C5986T,T6954C,G7042T,A10195G,C14120T,C14408T,C14676T,C15279T,T16176C,C18647T,A23063T,C23271A,A23403G,C23604A,C23709T,T24506G,G24914C,A25336C,G25337C,A25592T,G25906T,C27972T,G28048T,G28075T,A28111G,G28280C,A28281T,T28282A,G28881A,G28882A,G28883C,C28977T	26	N:D3L,N:R203K,N:G204R,N:S235F,ORF1a:T1001I,ORF1a:A1708D,ORF1a:I2230T,ORF1a:M2259I,ORF1b:P218L,ORF1b:P314L,ORF1b:P1727L,ORF3a:K67M,ORF3a:G172C,ORF8:Q27*,ORF8:R52I,ORF8:C61F,ORF8:Y73C,S:N501Y,S:A570D,S:D614G,S:P681H,S:T716I,S:S982A,S:D1118H,S:E1258D,S:D1259H	6	0
hCoV-19/Mexico/TAB-InDRE_FB17673_S3509/2021	EPI_ISL_2779024	-	B.1.1.7	GRY	39	C241T,C913T,C2110T,C3037T,C3267T,G4504T,C5388A,C5986T,T6954C,G7042T,C9430T,A10195G,C10279T,C14120T,C14408T,C14676T,C15279T,T16176C,C16887T,G17427T,A23063T,C23271A,A23403G,C23604A,C23709T,T24506G,G24914C,C27972T,G28048T,G28075T,A28111G,G28280C,A28281T,T28282A,G28881A,G28882A,G28883C,C28977T,C29149T	22	N:D3L,N:R203K,N:G204R,N:S235F,ORF1a:T1001I,ORF1a:E1413D,ORF1a:A1708D,ORF1a:I2230T,ORF1a:M2259I,ORF1b:P218L,ORF1b:P314L,ORF8:Q27*,ORF8:R52I,ORF8:C61F,ORF8:Y73C,S:N501Y,S:A570D,S:D614G,S:P681H,S:T716I,S:S982A,S:D1118H	6	0
hCoV-19/Mexico/TAB-InDRE_FB17674_S3510/2021	EPI_ISL_2779025	-	B.1.1.7	GRY	40	C241T,C913T,C2110T,C3037T,C3267T,C5388A,C5986T,C6628T,T6954C,G7042T,G8174A,A10195G,C10279T,C14120T,C14408T,C14676T,C15279T,T16176C,C16887T,G17427T,A23063T,C23271A,A23403G,C23604A,C23709T,T24506G,G24914C,A25097G,C27972T,G28048T,G28075T,A28111G,G28280C,A28281T,T28282A,G28881A,G28882A,G28883C,C28977T,A29700G	23	N:D3L,N:R203K,N:G204R,N:S235F,ORF1a:T1001I,ORF1a:A1708D,ORF1a:I2230T,ORF1a:M2259I,ORF1a:A2637T,ORF1b:P218L,ORF1b:P314L,ORF8:Q27*,ORF8:R52I,ORF8:C61F,ORF8:Y73C,S:N501Y,S:A570D,S:D614G,S:P681H,S:T716I,S:S982A,S:D1118H,S:I1179V	6	0
hCoV-19/Mexico/TAB-InDRE_FB17675_S3511/2021	EPI_ISL_2779026	-	B.1.1.7	GRY	39	C241T,C913T,C2110T,C3037T,C3267T,C5388A,C5986T,T6954C,G7042T,G8174A,A10195G,C10279T,C14120T,C14408T,C14676T,C15279T,T16176C,C16887T,G17427T,A23063T,C23271A,A23403G,C23604A,C23709T,T24506G,G24914C,A25097G,C27972T,G28048T,G28075T,A28111G,G28280C,A28281T,T28282A,G28881A,G28882A,G28883C,C28977T,A29700G	23	N:D3L,N:R203K,N:G204R,N:S235F,ORF1a:T1001I,ORF1a:A1708D,ORF1a:I2230T,ORF1a:M2259I,ORF1a:A2637T,ORF1b:P218L,ORF1b:P314L,ORF8:Q27*,ORF8:R52I,ORF8:C61F,ORF8:Y73C,S:N501Y,S:A570D,S:D614G,S:P681H,S:T716I,S:S982A,S:D1118H,S:I1179V	6	0
hCoV-19/Mexico/TAB-InDRE_FB17677_S3512/2021	EPI_ISL_2779027	-	B.1.1.7	GRY	39	C241T,C913T,C2110T,C3037T,C3267T,C5388A,C5986T,T6954C,G7042T,A10195G,C10279T,C14120T,C14408T,C14676T,C15279T,T16176C,C16887T,G17427T,A23063T,C23271A,A23403G,C23604A,C23709T,T24506G,G24914C,A25336C,C27972T,G28048T,G28075T,A28111G,G28280C,A28281T,T28282A,C28606T,G28881A,G28882A,G28883C,C28977T,G29260T	22	N:D3L,N:R203K,N:G204R,N:S235F,ORF1a:T1001I,ORF1a:A1708D,ORF1a:I2230T,ORF1a:M2259I,ORF1b:P218L,ORF1b:P314L,ORF8:Q27*,ORF8:R52I,ORF8:C61F,ORF8:Y73C,S:N501Y,S:A570D,S:D614G,S:P681H,S:T716I,S:S982A,S:D1118H,S:E1258D	6	0
hCoV-19/Mexico/TAB-InDRE_FB17680_S3513/2021	EPI_ISL_2779028	-	B.1.1.7	GRY	38	C241T,C913T,A1677C,C2110T,C3037T,C3267T,C5388A,C5986T,T6954C,G7042T,A10195G,C10279T,C14120T,C14408T,C14676T,C15279T,T16176C,C16887T,G17427T,A23063T,C23271A,A23403G,C23604A,C23709T,T24506G,G24914C,G25052T,C27972T,G28048T,G28075T,A28111G,G28280C,A28281T,T28282A,G28881A,G28882A,G28883C,C28977T	23	N:D3L,N:R203K,N:G204R,N:S235F,ORF1a:E471A,ORF1a:T1001I,ORF1a:A1708D,ORF1a:I2230T,ORF1a:M2259I,ORF1b:P218L,ORF1b:P314L,ORF8:Q27*,ORF8:R52I,ORF8:C61F,ORF8:Y73C,S:N501Y,S:A570D,S:D614G,S:P681H,S:T716I,S:S982A,S:D1118H,S:V1164F	6	0
hCoV-19/Mexico/TAB-InDRE_FB17687_S3514/2021	EPI_ISL_2779029	-	B.1.1.7	GRY	38	C241T,C913T,C2110T,C3037T,C3267T,C5388A,C5986T,C6016T,T6954C,G7042T,A10195G,C10279T,C14120T,C14408T,C14676T,C15279T,T16176C,C16887T,G17427T,A23063T,C23271A,A23403G,C23604A,C23709T,T24506G,G24914C,A25336C,C27972T,G28048T,G28075T,A28111G,G28280C,A28281T,T28282A,G28881A,G28882A,G28883C,C28977T	22	N:D3L,N:R203K,N:G204R,N:S235F,ORF1a:T1001I,ORF1a:A1708D,ORF1a:I2230T,ORF1a:M2259I,ORF1b:P218L,ORF1b:P314L,ORF8:Q27*,ORF8:R52I,ORF8:C61F,ORF8:Y73C,S:N501Y,S:A570D,S:D614G,S:P681H,S:T716I,S:S982A,S:D1118H,S:E1258D	6	0
hCoV-19/Mexico/TAB-InDRE_FB17688_S3515/2021	EPI_ISL_2779030	-	B.1.1.7	GRY	42	C241T,G343T,C913T,G1820A,C3037T,C3267T,C5388A,C5986T,T6954C,A10323G,C10993T,C11704T,C14408T,C14676T,T15096C,C15279T,T16176C,C17733T,G17790T,T18327C,C18687T,A23063T,C23271A,A23403G,C23604A,C23709T,T24506G,G24914C,A25336C,G25337C,C27549T,C27972T,G28048T,A28111G,G28280C,A28281T,T28282A,G28541T,G28881A,G28882A,G28883C,C2	25	N:D3L,N:A90S,N:R203K,N:G204R,N:S235F,ORF1a:G519S,ORF1a:T1001I,ORF1a:A1708D,ORF1a:I2230T,ORF1a:K3353R,ORF1b:P314L,ORF1b:Q1441H,ORF8:Q27*,ORF8:R52I,ORF8:Y73C,ORF9b:E86D,S:N501Y,S:A570D,S:D614G,S:P681H,S:T716I,S:S982A,S:D1118H,S:E1258D,S:D1259H	6	0
hCoV-19/Mexico/TAB-InDRE_FB17689_S3516/2021	EPI_ISL_2779031	-	B.1.1.7	GRY	38	C241T,C913T,C2110T,C3037T,C3267T,C5388A,C5986T,T6954C,G7042T,A10195G,C10279T,C14120T,C14408T,C14676T,C15279T,T16176C,C16887T,G17427T,A23063T,C23271A,A23403G,C23604A,C23709T,T24506G,G24914C,G25052T,A25336C,C27972T,G28048T,G28075T,A28111G,G28280C,A28281T,T28282A,G28881A,G28882A,G28883C,C28977T	23	N:D3L,N:R203K,N:G204R,N:S235F,ORF1a:T1001I,ORF1a:A1708D,ORF1a:I2230T,ORF1a:M2259I,ORF1b:P218L,ORF1b:P314L,ORF8:Q27*,ORF8:R52I,ORF8:C61F,ORF8:Y73C,S:N501Y,S:A570D,S:D614G,S:P681H,S:T716I,S:S982A,S:D1118H,S:V1164F,S:E1258D	6	0
hCoV-19/Mexico/TAB-InDRE_FB17693_S3517/2021	EPI_ISL_2779032	-	B.1.1.7	GRY	39	C241T,C913T,C2110T,C3037T,C3267T,G4504T,C5388A,C5986T,T6954C,G7042T,A10195G,C10279T,C14120T,C14408T,C14676T,C15279T,T16176C,C16887T,G17427T,A23063T,C23271A,A23403G,C23604A,C23709T,T24506G,G24914C,A25336C,C27972T,G28048T,G28075T,A28111G,G28280C,A28281T,T28282A,G28881A,G28882A,G28883C,C28977T,C29149T	23	N:D3L,N:R203K,N:G204R,N:S235F,ORF1a:T1001I,ORF1a:E1413D,ORF1a:A1708D,ORF1a:I2230T,ORF1a:M2259I,ORF1b:P218L,ORF1b:P314L,ORF8:Q27*,ORF8:R52I,ORF8:C61F,ORF8:Y73C,S:N501Y,S:A570D,S:D614G,S:P681H,S:T716I,S:S982A,S:D1118H,S:E1258D	6	0
hCoV-19/Mexico/TAB-InDRE_FB17694_S3518/2021	EPI_ISL_2779033	-	B.1.1.7	GRY	39	C241T,C913T,C2110T,C3037T,C3267T,C5388A,C5986T,T6954C,G7042T,G8174A,A10195G,C10279T,C14120T,C14408T,C14676T,C15279T,T16176C,C16887T,G17427T,A23063T,C23271A,A23403G,C23604A,C23709T,T24506G,G24914C,A25336C,G25337C,C27972T,G28048T,G28075T,A28111G,G28280C,A28281T,T28282A,G28881A,G28882A,G28883C,C28977T	24	N:D3L,N:R203K,N:G204R,N:S235F,ORF1a:T1001I,ORF1a:A1708D,ORF1a:I2230T,ORF1a:M2259I,ORF1a:A2637T,ORF1b:P218L,ORF1b:P314L,ORF8:Q27*,ORF8:R52I,ORF8:C61F,ORF8:Y73C,S:N501Y,S:A570D,S:D614G,S:P681H,S:T716I,S:S982A,S:D1118H,S:E1258D,S:D1259H	6	0
hCoV-19/Mexico/TAB-InDRE_FB17796_S3528/2021	EPI_ISL_2779043	-	B.1.1.7	GRY	38	C241T,C913T,G1696A,C2110T,C3037T,C3267T,C5388A,C5986T,T6954C,G7042T,A10195G,C10279T,C12473T,C14120T,C14408T,C14676T,C15279T,T16176C,C16887T,G17427T,A23063T,C23271A,A23403G,C23604A,C23709T,T24506G,G24914C,C27972T,G28048T,G28075T,A28111G,G28280C,A28281T,T28282A,G28881A,G28882A,G28883C,C28977T	21	N:D3L,N:R203K,N:G204R,N:S235F,ORF1a:T1001I,ORF1a:A1708D,ORF1a:I2230T,ORF1a:M2259I,ORF1b:P218L,ORF1b:P314L,ORF8:Q27*,ORF8:R52I,ORF8:C61F,ORF8:Y73C,S:N501Y,S:A570D,S:D614G,S:P681H,S:T716I,S:S982A,S:D1118H	6	0

hCoV-19/Mexico/TAB-InDRE_FB17798_S3529/2021	EPI_ISL_2779044	-	B.1.1.7	GRY	37	C241T,C913T,C2110T,C3037T,C3267T,C5388A,C5986T,T6954C,G7042T,A10195G,C10279T,C14120T,C14408T,C14676T,C15279T,T16176C,C16887T,G17427T,A23063T,C23271A,A23403G,C23604A,C23709T,T24506G,G24914C,C27972T,G28048T,G28075T,A28095T,A28111G,G28280C,A28281T,T28282A,G28881A,G28882A,G28883C,C28977T	22	N:D3L,N:R203K,N:G204R,N:S235F,ORF1a:T1001I,ORF1a:A1708D,ORF1a:I2230T,ORF1a:M2259I,ORF1b:P218L,ORF1b:P314L,ORF8:Q27*,ORF8:R52I,ORF8:C61F,ORF8:K68*,ORF8:Y73C,S:N501Y,S:A570D,S:D614G,S:P681H,S:T716I,S:S982A,S:D1118H	6	0
hCoV-19/Mexico/TAB-InDRE_FB17801_S3530/2021	EPI_ISL_2779045	-	B.1.1.7	GRY	34	C241T,C913T,C2110T,C3037T,C3267T,C5388A,C5986T,C6016T,T6954C,G7042T,A10195G,C10279T,C14120T,C14408T,C14676T,C15279T,T16176C,C16887T,G17427T,A23063T,C23271A,A23403G,C23604A,C23709T,T24506G,G24914C,C27972T,G28048T,G28075T,A28111G,G28881A,G28	20	N:R203K,N:G204R,N:S235F,ORF1a:T1001I,ORF1a:A1708D,ORF1a:I2230T,ORF1a:M2259I,ORF1b:P218L,ORF1b:P314L,ORF8:Q27*,ORF8:R52I,ORF8:C61F,ORF8:Y73C,S:N501Y,S:A570D,S:D614G,S:P681H,S:T716I,S:S982A,S:D1118H	6	90
hCoV-19/Mexico/TAB-InDRE_FB17802_S3531/2021	EPI_ISL_2779046	-	B.1.1.7	GRY	36	C241T,C913T,C3037T,C3267T,C5388A,A5563G,C5986T,C6196T,T6954C,A8848G,T11135C,C14408T,C14676T,C15279T,T16176C,C19512T,A23063T,C23114T,C23271A,A23403G,C23604A,C23709T,T24506G,G24914C,G25996T,C27972T,G28048T,A28111G,G28280C,A28281T,T28282A,G28881A,G28882A,G28883C,C28977T,G29715T	20	N:D3L,N:R203K,N:G204R,N:S235F,ORF1a:T1001I,ORF1a:A1708D,ORF1a:I2230T,ORF1a:F3624L,ORF1b:P314L,ORF3a:V202L,ORF8:Q27*,ORF8:R52I,ORF8:Y73C,S:N501Y,S:A570D,S:D614G,S:P681H,S:T716I,S:S982A,S:D1118H	6	0
hCoV-19/Mexico/TAB-InDRE_FB17985_S3850/2021	EPI_ISL_2835057	-	B.1.1.7	GR	41	C241T,G343T,C913T,G1820A,C3037T,C3267T,C5388A,C5986T,T6954C,A10323G,C10993T,C11704T,C14408T,C14676T,T15096C,C15279T,T16176C,C17733T,G17790T,T18327C,C18687T,A23063T,C23271A,A23403G,C23604A,C23709T,T24506G,G24914C,A25336C,G25337C,C27972T,G28048T,A28111G,G28280C,A28281T,T28282A,G28541T,G28881A,G28882A,G28883C,C28977T	25	N:D3L,N:A90S,N:R203K,N:G204R,N:S235F,ORF1a:G519S,ORF1a:T1001I,ORF1a:A1708D,ORF1a:I2230T,ORF1a:K3353R,ORF1b:P314L,ORF1b:Q1441H,ORF8:Q27*,ORF8:R52I,ORF8:Y73C,ORF9b:E86D,S:N501Y,S:A570D,S:D614G,S:P681H,S:T716I,S:S982A,S:D1118H,S:E1258D,S:D1259H	4	40
hCoV-19/Mexico/TAB-InDRE_FB17995_S3943/2021	EPI_ISL_2858957	-	B.1.1.7	GRY	38	C241T,C913T,C2110T,C3037T,C3267T,C5388A,C5986T,T6954C,G7042T,A10195G,C10279T,C14120T,C14408T,C14676T,C15279T,T16176C,C16887T,G17427T,A23063T,C23271A,A23403G,C23604A,C23709T,T24506G,G24914C,A25336C,C27972T,G28048T,G28075T,A28111G,G28280C,A28281T,T28282A,C28606T,G28881A,G28882A,G28883C,C28977T	22	N:D3L,N:R203K,N:G204R,N:S235F,ORF1a:T1001I,ORF1a:A1708D,ORF1a:I2230T,ORF1a:M2259I,ORF1b:P218L,ORF1b:P314L,ORF8:Q27*,ORF8:R52I,ORF8:C61F,ORF8:Y73C,S:N501Y,S:A570D,S:D614G,S:P681H,S:T716I,S:S982A,S:D1118H,S:E1258D	6	0
hCoV-19/Mexico/TAB-InDRE_FB17999_S3944/2021	EPI_ISL_2858958	-	B.1.1.7	GRY	39	C241T,C913T,C2110T,C3037T,C3267T,G4504T,C5388A,C5986T,T6954C,G7042T,A10195G,C10279T,C14120T,C14408T,C14676T,C15279T,T16176C,C16887T,G17427T,A23063T,C23271A,A23403G,C23604A,C23709T,T24506G,G24914C,A25336C,C27972T,G28048T,G28075T,A28111G,G28280C,A28281T,T28282A,G28881A,G28882A,G28883C,C28977T,C29149T	23	N:D3L,N:R203K,N:G204R,N:S235F,ORF1a:T1001I,ORF1a:E1413D,ORF1a:A1708D,ORF1a:I2230T,ORF1a:M2259I,ORF1b:P218L,ORF1b:P314L,ORF8:Q27*,ORF8:R52I,ORF8:C61F,ORF8:Y73C,S:N501Y,S:A570D,S:D614G,S:P681H,S:T716I,S:S982A,S:D1118H,S:E1258D	6	0
hCoV-19/Mexico/TAB-InDRE_FB18008_S3945/2021	EPI_ISL_2858959	-	B.1.1.7	GRY	42	C241T,C913T,C2110T,C3037T,C3267T,C4113T,C5388A,C5986T,T6954C,G7042T,C9430T,A10195G,C10279T,C14120T,C14408T,C14676T,C15279T,T16176C,C16887T,T16944C,G17427T,A23063T,C23271A,A23403G,T23560C,C23604A,C23709T,C24237T,T24506G,G24914C,A25336C,C27972T,G28048T,G28075T,A28111G,G28280C,A28281T,T28282A,G28881A,G28882A,G28883C,C28	24	N:D3L,N:R203K,N:G204R,N:S235F,ORF1a:T1001I,ORF1a:A1283V,ORF1a:A1708D,ORF1a:I2230T,ORF1a:M2259I,ORF1b:P218L,ORF1b:P314L,ORF8:Q27*,ORF8:R52I,ORF8:C61F,ORF8:Y73C,S:N501Y,S:A570D,S:D614G,S:P681H,S:T716I,S:A892V,S:S982A,S:D1118H,S:E1258D	6	0
hCoV-19/Mexico/TAB-InDRE_FB18030_S3948/2021	EPI_ISL_2858962	-	B.1.1.7	GRY	39	C241T,C913T,C2110T,C3037T,C3267T,G4504T,C5388A,C5986T,T6954C,G7042T,A10195G,C10279T,C14120T,C14408T,C14676T,C15279T,T16176C,C16887T,G17427T,A23063T,C23271A,A23403G,C23604A,C23709T,T24506G,G24914C,A25336C,C27972T,G28048T,G28075T,A28111G,G28280C,A28281T,T28282A,G28881A,G28882A,G28883C,C28977T,C29149T	23	N:D3L,N:R203K,N:G204R,N:S235F,ORF1a:T1001I,ORF1a:E1413D,ORF1a:A1708D,ORF1a:I2230T,ORF1a:M2259I,ORF1b:P218L,ORF1b:P314L,ORF8:Q27*,ORF8:R52I,ORF8:C61F,ORF8:Y73C,S:N501Y,S:A570D,S:D614G,S:P681H,S:T716I,S:S982A,S:D1118H,S:E1258D	6	0
hCoV-19/Mexico/TAB-InDRE_FB18032_S3949/2021	EPI_ISL_2858963	-	B.1.1.7	GRY	38	C241T,C913T,C2110T,C3037T,C3267T,C5388A,C5986T,C6573T,T6954C,G7042T,A10195G,C10279T,C14120T,C14408T,C14676T,C15279T,T16176C,C16887T,G17427T,A23063T,C23271A,A23403G,C23604A,C23709T,T24506G,G24914C,A25336C,C27972T,G28048T,G28075T,A28111G,G28280C,A28281T,T28282A,G28881A,G28882A,G28883C,C28977T	23	N:D3L,N:R203K,N:G204R,N:S235F,ORF1a:T1001I,ORF1a:A1708D,ORF1a:S2103F,ORF1a:I2230T,ORF1a:M2259I,ORF1b:P218L,ORF1b:P314L,ORF8:Q27*,ORF8:R52I,ORF8:C61F,ORF8:Y73C,S:N501Y,S:A570D,S:D614G,S:P681H,S:T716I,S:S982A,S:D1118H,S:E1258D	6	0
hCoV-19/Mexico/TAB-InDRE_FB18051_S3950/2021	EPI_ISL_2858964	-	B.1.1.7	GRY	40	C241T,C913T,G1264T,C2110T,C3037T,C3267T,G5194T,C5388A,C5986T,T6954C,G7042T,A10195G,C10279T,C12468T,C14120T,C14408T,C14676T,C15279T,T16176C,C16887T,G17427T,A23063T,C23271A,A23403G,C23604A,C23709T,T24506G,G24914C,C27972T,G28048T,G28075T,A28095T,A28111G,G28280C,A28281T,T28282A,G28881A,G28882A,G28883C,C28977T	23	N:D3L,N:R203K,N:G204R,N:S235F,ORF1a:T1001I,ORF1a:A1708D,ORF1a:I2230T,ORF1a:M2259I,ORF1a:A4068V,ORF1b:P218L,ORF1b:P314L,ORF8:Q27*,ORF8:R52I,ORF8:C61F,ORF8:K68*,ORF8:Y73C,S:N501Y,S:A570D,S:D614G,S:P681H,S:T716I,S:S982A,S:D1118H	6	0
hCoV-19/Mexico/TAB-InDRE_FB18182_S3960/2021	EPI_ISL_2858974	-	B.1.1.7	GRY	41	A38C,C241T,C913T,C3037T,C3267T,C5388A,C5944T,C5986T,T6954C,C9967T,C13585T,C14408T,G14500T,C14676T,T15096C,C15279T,T16176C,G17658T,G21901A,C22987T,C22993T,A23063T,C23271A,A23403G,C23604A,C23709T,T24506G,G24914C,G25218T,C27972T,G28048T,A28095T,A28111G,G28280C,A28281T,T28282A,G28881A,G28882A,G28883C,G28884C,C28977T	22	N:D3L,N:R203K,N:G204P,N:S235F,ORF1a:T1001I,ORF1a:A1708D,ORF1a:I2230T,ORF1b:P314L,ORF1b:V345L,ORF1b:M1397I,ORF8:Q27*,ORF8:R52I,ORF8:K68*,ORF8:Y73C,S:N501Y,S:A570D,S:D614G,S:P681H,S:T716I,S:S982A,S:D1118H,S:G1219V	6	0
hCoV-19/Mexico/TAB-InDRE_FB18192_S3961/2021	EPI_ISL_2858975	-	B.1.1.7	GRY	40	C241T,C913T,C3037T,C3267T,C5388A,A5935G,C5986T,C6196T,C6268T,T6954C,A8315G,A8848G,C10125T,G10261A,C14408T,C14676T,C15279T,T16176C,A23063T,C23271A,A23403G,C23604A,C23709T,T24506G,G24914C,C25544T,C26801T,C27945T,C27972T,G28048T,A28111G,T28245G,G28280C,A28281T,T28282A,G28881A,G28882A,G28883C,C28977T,G29715T	23	N:D3L,N:R203K,N:G204R,N:S235F,ORF1a:T1001I,ORF1a:A1708D,ORF1a:I2230T,ORF1a:T2684A,ORF1a:T3287I,ORF1b:P314L,ORF3a:A51V,ORF8:Q18*,ORF8:Q27*,ORF8:R52I,ORF8:Y73C,ORF8:L118V,S:N501Y,S:A570D,S:D614G,S:P681H,S:T716I,S:S982A,S:D1118H	6	0
hCoV-19/Mexico/TAB-InDRE_FB18197_S3962/2021	EPI_ISL_2858976	-	B.1.1.7	GRY	42	C241T,C313T,C913T,C2110T,C3037T,C3267T,C5388A,C5986T,T6954C,G7042T,C7834T,C9693T,A10195G,C10279T,C14120T,C14408T,C14676T,C15279T,T16176C,C16887T,G17427T,A23063T,C23271A,A23403G,C23604A,C23709T,T24506G,G24914C,C26622T,C27972T,G28048T,G28075T,A28095T,A28111G,G28280C,A28281T,T28282A,G28881A,G28882A,G28883C,C28977T,C29774	24	M:L34F,N:D3L,N:R203K,N:G204R,N:S235F,ORF1a:T1001I,ORF1a:A1708D,ORF1a:I2230T,ORF1a:M2259I,ORF1a:A3143V,ORF1b:P218L,ORF1b:P314L,ORF8:Q27*,ORF8:R52I,ORF8:C61F,ORF8:K68*,ORF8:Y73C,S:N501Y,S:A570D,S:D614G,S:P681H,S:T716I,S:S982A,S:D1118H	6	0
hCoV-19/Mexico/TAB-InDRE_FB18207_S3963/2021	EPI_ISL_2858977	-	B.1.1.7	GRY	41	C241T,C913T,C2110T,C3037T,C3267T,C5388A,C5986T,T6954C,G7042T,A10195G,C10279T,C10725A,C13432T,C14120T,C14408T,C14676T,C15279T,T16176C,C16887T,G17427T,C22388T,A23063T,C23271A,A23403G,C23604A,C23709T,T24506G,G24914C,A25336C,C27972T,G28048T,G28075T,A28111G,G28280C,A28281T,T28282A,C28606T,G28881A,G28882A,G28883C,C28977T	23	N:D3L,N:R203K,N:G204R,N:S235F,ORF1a:T1001I,ORF1a:A1708D,ORF1a:I2230T,ORF1a:M2259I,ORF1a:T3487N,ORF1b:P218L,ORF1b:P314L,ORF8:Q27*,ORF8:R52I,ORF8:C61F,ORF8:Y73C,S:N501Y,S:A570D,S:D614G,S:P681H,S:T716I,S:S982A,S:D1118H,S:E1258D	6	0
hCoV-19/Mexico/TAB-InDRE_FB18209_S3964/2021	EPI_ISL_2858978	-	B.1.1.7	GRY	40	C241T,C913T,C3037T,C3267T,C5388A,C5986T,C6196T,C6268T,T6954C,A8315G,A8848G,C10125T,G10261A,C12295T,C14408T,C14676T,C15279T,T16176C,A23063T,C23271A,A23403G,C23604A,C23709T,T24506G,G24914C,A25336C,C25544T,C26801T,C27972T,G28048T,A28111G,T28245G,G28280C,A28281T,T28282A,G28881A,G28882A,G28883C,C28977T,G29715T	23	N:D3L,N:R203K,N:G204R,N:S235F,ORF1a:T1001I,ORF1a:A1708D,ORF1a:I2230T,ORF1a:T2684A,ORF1a:T3287I,ORF1b:P314L,ORF3a:A51V,ORF8:Q27*,ORF8:R52I,ORF8:Y73C,ORF8:L118V,S:N501Y,S:A570D,S:D614G,S:P681H,S:T716I,S:S982A,S:D1118H,S:E1258D	6	4
hCoV-19/Mexico/TAB-InDRE_FB18330_S3970/2021	EPI_ISL_2858984	-	B.1.1.7	GRY	41	C241T,C913T,A1677C,C2110T,C3037T,C3267T,C5388A,C5986T,T6954C,G7042T,A10195G,C10279T,C14120T,C14408T,C14676T,C15279T,C15579T,T16176C,C16887T,G17427T,A23063T,C23271A,A23403G,C23604A,C23709T,T24506G,G24914C,G25052T,A25336C,C27689T,C27972T,G28048T,G28075T,A28111G,G28280C,A28281T,T28282A,G28881A,G28882A,G28883C,C28977T	25	N:D3L,N:R203K,N:G204R,N:S235F,ORF1a:E471A,ORF1a:T1001I,ORF1a:A1708D,ORF1a:I2230T,ORF1a:M2259I,ORF1b:P218L,ORF1b:P314L,ORF7a:P99L,ORF8:Q27*,ORF8:R52I,ORF8:C61F,ORF8:Y73C,S:N501Y,S:A570D,S:D614G,S:P681H,S:T716I,S:S982A,S:D1118H,S:V1164F,S:E1258D	6	0
hCoV-19/Mexico/TAB-InDRE_FB18331_S3971/2021	EPI_ISL_2858985	-	B.1.1.7	GRY	42	C241T,C913T,C2110T,C3037T,C3267T,C5388A,C5986T,T6954C,G7042T,C7834T,A10195G,C10279T,C14120T,C14408T,C14676T,C15279T,T16176C,C16887T,G17427T,A22010G,G22205T,A23063T,C23271A,A23403G,C23604A,C23709T,T24506G,G24914C,A25336C,G27506T,C27972T,G28048T,G28075T,A28111G,G28280C,A28281T,T28282A,G28881A,G28882A,G28883C,C28977T,C29	25	N:D3L,N:R203K,N:G204R,N:S235F,ORF1a:T1001I,ORF1a:A1708D,ORF1a:I2230T,ORF1a:M2259I,ORF1b:P218L,ORF1b:P314L,ORF7a:G38V,ORF8:Q27*,ORF8:R52I,ORF8:C61F,ORF8:Y73C,S:K150E,S:D215Y,S:N501Y,S:A570D,S:D614G,S:P681H,S:T716I,S:S982A,S:D1118H,S:E1258D	6	0
hCoV-19/Mexico/TAB-InDRE_FB18332_S3972/2021	EPI_ISL_2858986	-	B.1.1.7	GRY	39	C241T,C913T,G1264T,C2110T,C3037T,C3267T,G5194T,C5388A,C5986T,T6954C,G7042T,A10195G,C10279T,C12468T,C14120T,C14408T,C14676T,C15279T,T16176C,C16887T,G17427T,A23063T,C23271A,A23403G,C23604A,C23709T,T24506G,G24914C,C27972T,G28048T,G28075T,A28111G,G28280C,A28281T,T28282A,G28881A,G28882A,G28883C,C28977T	22	N:D3L,N:R203K,N:G204R,N:S235F,ORF1a:T1001I,ORF1a:A1708D,ORF1a:I2230T,ORF1a:M2259I,ORF1a:A4068V,ORF1b:P218L,ORF1b:P314L,ORF8:Q27*,ORF8:R52I,ORF8:C61F,ORF8:Y73C,S:N501Y,S:A570D,S:D614G,S:P681H,S:T716I,S:S982A,S:D1118H	6	0
hCoV-19/Mexico/TAB-InDRE_FB18381_S4143/2021	EPI_ISL_2920714	-	B.1.1.7	GRY	41	C241T,C913T,C2110T,C3037T,C3267T,C4113T,C5388A,C5986T,T6954C,G7042T,C9430T,A10195G,C10279T,C14120T,C14408T,C14676T,C15279T,T16176C,C16887T,T16944C,G17427T,A23063T,C23271A,A23403G,C23604A,C23709T,T24506G,G24914C,G26065T,C27972T,G28048T,G28075T,A28095T,A28111G,G28280C,A28281T,T28282A,G28881A,G28882A,G28883C,C28977T	24	N:D3L,N:R203K,N:G204R,N:S235F,ORF1a:T1001I,ORF1a:A1283V,ORF1a:A1708D,ORF1a:I2230T,ORF1a:M2259I,ORF1b:P218L,ORF1b:P314L,ORF3a:V225F,ORF8:Q27*,ORF8:R52I,ORF8:C61F,ORF8:K68*,ORF8:Y73C,S:N501Y,S:A570D,S:D614G,S:P681H,S:T716I,S:S982A,S:D1118H	6	0

hCoV-19/Mexico/TAB-InDRE_FB18382_S4144/2021	EPI_ISL_2920715	-	B.1.1.7	GRY	41	C241T,C913T,C2110T,C3037T,C3267T,C4113T,C5388A,C5986T,T6954C,G7042T,C9430T,A10195G,C10279T,C14120T,C14408T,C14676T,C15279T,T16176C,C16887T,T16944C,G17427T,A23063T,C23271A,A23403G,C23604A,C23709T,T24506G,G24914C,G26065T,C27972T,G28048T,G28075T,A28095T,A28111G,G28280C,A28281T,T28282A,G28881A,G28882A,G28883C,C28977T	24	N:D3L,N:R203K,N:G204R,N:S235F,ORF1a:T1001I,ORF1a:A1283V,ORF1a:A1708D,ORF1a:I2230T,ORF1a:M2259I,ORF1b:P218L,ORF1b:P314L,ORF3a:V225F,ORF8:Q27*,ORF8:R52I,ORF8:C61F,ORF8:K68*,ORF8:Y73C,S:N501Y,S:A570D,S:D614G,S:P681H,S:T716I,S:S982A,S:D1118H	6	0
hCoV-19/Mexico/TAB-InDRE_FB18389_S4145/2021	EPI_ISL_2920716	-	B.1.1.7	GRY	40	C241T,C913T,C2110T,C3037T,C3267T,G4504T,C5388A,C5986T,C6091T,T6954C,G7042T,A10195G,C10279T,C14120T,C14408T,C14676T,C15279T,T16176C,C16887T,G17427T,A23063T,C23271A,A23403G,C23604A,C23709T,T24506G,G24914C,C27972T,G28048T,G28075T,A28095T,A28111G,G28280C,A28281T,T28282A,G28881A,G28882A,G28883C,C28977T,C29149T	23	N:D3L,N:R203K,N:G204R,N:S235F,ORF1a:T1001I,ORF1a:E1413D,ORF1a:A1708D,ORF1a:I2230T,ORF1a:M2259I,ORF1b:P218L,ORF1b:P314L,ORF8:Q27*,ORF8:R52I,ORF8:C61F,ORF8:K68*,ORF8:Y73C,S:N501Y,S:A570D,S:D614G,S:P681H,S:T716I,S:S982A,S:D1118H	6	0
hCoV-19/Mexico/TAB-InDRE_FB18397_S4146/2021	EPI_ISL_2920717	-	B.1.1.7	GRY	40	C241T,C913T,C3037T,C3267T,C5388A,C5986T,T6954C,C9693T,A11351G,C11442T,C11572T,C14408T,C14676T,C15279T,T16176C,G17058A,G18651A,T23042C,A23063T,C23271A,A23403G,C23604A,C23709T,T24506G,G24914C,G25218T,G25429T,C25553T,C27615T,C27972T,G28048T,A28095T,A28111G,G28280C,A28281T,T28282A,G28881A,G28882A,G28883C,C28977T	27	N:D3L,N:R203K,N:G204R,N:S235F,ORF1a:T1001I,ORF1a:A1708D,ORF1a:I2230T,ORF1a:A3143V,ORF1a:T3696A,ORF1a:A3726V,ORF1b:P314L,ORF1b:M1197I,ORF3a:V13L,ORF3a:A54V,ORF8:Q27*,ORF8:R52I,ORF8:K68*,ORF8:Y73C,S:S494P,S:N501Y,S:A570D,S:D614G,S:P681H,S:T716I,S:S982A,S:D1118H,S:G1219V	6	0
hCoV-19/Mexico/TAB-InDRE_FB18408_S4147/2021	EPI_ISL_2920718	-	B.1.1.7	GR	39	C241T,C913T,C2110T,C3037T,C3267T,G4504T,C5388A,C5986T,T6954C,G7042T,A10195G,C10279T,C14120T,C14408T,C14676T,C15279T,T16176C,C16887T,G17427T,A23063T,C23271A,A23403G,C23604A,C23709T,T24506G,G24914C,A25336C,C27972T,G28048T,G28075T,A28111G,G28280C,A28281T,T28282A,G28881A,G28882A,G28883C,C28977T,C29149T	23	N:D3L,N:R203K,N:G204R,N:S235F,ORF1a:T1001I,ORF1a:E1413D,ORF1a:A1708D,ORF1a:I2230T,ORF1a:M2259I,ORF1b:P218L,ORF1b:P314L,ORF8:Q27*,ORF8:R52I,ORF8:C61F,ORF8:Y73C,S:N501Y,S:A570D,S:D614G,S:P681H,S:T716I,S:S982A,S:D1118H,S:E1258D	4	395
hCoV-19/Mexico/TAB-InDRE_FB18500_S4148/2021	EPI_ISL_2920719	-	B.1.1.7	GRY	39	C241T,C913T,C2453T,C3037T,C3267T,C5388A,C5986T,T6954C,C8917T,C9611T,C14408T,C14676T,C15279T,G16068T,T16176C,G17562T,C20930T,C21110T,C21855T,A23063T,C23271A,A23403G,C23604A,C23709T,T24506G,G24914C,A25336C,G25337C,C27972T,G28048T,A28111G,G28280C,A28281T,T28282A,A28295G,G28881A,G28882A,G28883C,C28977T	27	N:D3L,N:N8D,N:R203K,N:G204R,N:S235F,ORF1a:L730F,ORF1a:T1001I,ORF1a:A1708D,ORF1a:I2230T,ORF1a:L3116F,ORF1b:P314L,ORF1b:E867D,ORF1b:T2488M,ORF1b:T2548I,ORF8:Q27*,ORF8:R52I,ORF8:Y73C,S:S98F,S:N501Y,S:A570D,S:D614G,S:P681H,S:T716I,S:S982A,S:D1118H,S:E1258D,S:D1259H	6	0
hCoV-19/Mexico/TAB-InDRE_FB18518_S4149/2021	EPI_ISL_2920720	-	B.1.1.7	GRY	42	C241T,C913T,C3037T,C3267T,C5388A,C5986T,C6196T,C6268T,T6954C,A8315G,A8848G,C10125T,G10261A,A12461G,C14408T,C14676T,C15279T,T16176C,A23063T,C23271A,A23403G,C23604A,C23709T,T24506G,G24914C,A25336C,G25337C,C25544T,C26801T,C27945T,C27972T,G28048T,A28111G,T28245G,G28280C,A28281T,T28282A,G28881A,G28882A,G28883C,C28977T,G2	26	N:D3L,N:R203K,N:G204R,N:S235F,ORF1a:T1001I,ORF1a:A1708D,ORF1a:I2230T,ORF1a:T2684A,ORF1a:T3287I,ORF1a:T4066A,ORF1b:P314L,ORF3a:A51V,ORF8:Q18*,ORF8:Q27*,ORF8:R52I,ORF8:Y73C,ORF8:L118V,S:N501Y,S:A570D,S:D614G,S:P681H,S:T716I,S:S982A,S:D1118H,S:E1258D,S:D1259H	6	0
hCoV-19/Mexico/TAB-InDRE_FB18526_S4150/2021	EPI_ISL_2920721	-	B.1.1.7	GRY	39	C241T,C913T,C2110T,C3037T,C3267T,C4084T,C5388A,C5986T,T6954C,G7042T,A10195G,C10279T,C14120T,C14408T,C14676T,C15279T,T16176C,C16887T,G17427T,A23063T,C23271A,A23403G,C23604A,C23709T,T24506G,G24914C,A25336C,G25337C,C27972T,G28048T,G28075T,A28111G,G28280C,A28281T,T28282A,G28881A,G28882A,G28883C,C28977T	23	N:D3L,N:R203K,N:G204R,N:S235F,ORF1a:T1001I,ORF1a:A1708D,ORF1a:I2230T,ORF1a:M2259I,ORF1b:P218L,ORF1b:P314L,ORF8:Q27*,ORF8:R52I,ORF8:C61F,ORF8:Y73C,S:N501Y,S:A570D,S:D614G,S:P681H,S:T716I,S:S982A,S:D1118H,S:E1258D,S:D1259H	6	172
hCoV-19/Mexico/TAB-InDRE_FB18527_S4151/2021	EPI_ISL_2920722	-	B.1.1.7	GRY	39	C241T,C913T,C2110T,C3037T,C3267T,C5388A,C5986T,T6954C,G7042T,A10195G,C10279T,C14120T,C14408T,C14676T,C15279T,T16176C,C16887T,G17427T,A23063T,C23271A,A23403G,C23604A,C23709T,T24506G,G24914C,G25334C,A25336C,G25337C,C27972T,G28048T,G28075T,A28111G,G28280C,A28281T,T28282A,G28881A,G28882A,G28883C,C28977T	23	N:D3L,N:R203K,N:G204R,N:S235F,ORF1a:T1001I,ORF1a:A1708D,ORF1a:I2230T,ORF1a:M2259I,ORF1b:P218L,ORF1b:P314L,ORF8:Q27*,ORF8:R52I,ORF8:C61F,ORF8:Y73C,S:N501Y,S:A570D,S:D614G,S:P681H,S:T716I,S:S982A,S:D1118H,S:E1258H,S:D1259H	6	28
hCoV-19/Mexico/TAB-InDRE_FB18528_S4152/2021	EPI_ISL_2920723	-	B.1.1.7	GRY	37	C241T,C913T,C2110T,C3037T,C3267T,C5388A,C5986T,T6954C,G7042T,A10195G,C10279T,C14120T,C14408T,C14676T,C15279T,T16176C,C16887T,G17427T,A23063T,C23271A,A23403G,C23604A,C23709T,T24506G,G24914C,A25336C,C27972T,G28048T,G28075T,A28111G,G28280C,A28281T,T28282A,G28881A,G28882A,G28883C,C28977T	22	N:D3L,N:R203K,N:G204R,N:S235F,ORF1a:T1001I,ORF1a:A1708D,ORF1a:I2230T,ORF1a:M2259I,ORF1b:P218L,ORF1b:P314L,ORF8:Q27*,ORF8:R52I,ORF8:C61F,ORF8:Y73C,S:N501Y,S:A570D,S:D614G,S:P681H,S:T716I,S:S982A,S:D1118H,S:E1258D	6	0
hCoV-19/Mexico/TAB-InDRE_FB19055_S4487/2021	EPI_ISL_3033341	-	B.1.1.7	GRY	37	C241T,C913T,G1820A,C2453T,C3037T,C3267T,C5388A,C5986T,T6954C,C9611T,C9893T,C14408T,C14676T,C15279T,T16176C,T18429C,C21575T,C21855T,G21974C,A23063T,C23271A,A23403G,C23604A,C23709T,T24506G,G24914C,C27972T,G28048T,A28111G,G28280C,A28281T,T28282A,A28295G,G28881A,G28882A,G28883C,C28977T	26	N:D3L,N:N8D,N:R203K,N:G204R,N:S235F,ORF1a:G519S,ORF1a:L730F,ORF1a:T1001I,ORF1a:A1708D,ORF1a:I2230T,ORF1a:L3116F,ORF1a:L3210F,ORF1b:P314L,ORF8:Q27*,ORF8:R52I,ORF8:Y73C,S:L5F,S:S98F,S:D138H,S:N501Y,S:A570D,S:D614G,S:P681H,S:T716I,S:S982A,S:D1118H	6	117
hCoV-19/Mexico/TAB-InDRE_FB19065_S4488/2021	EPI_ISL_3033342	-	B.1.1.7	GRY	40	C241T,C913T,G1696A,C2110T,C3037T,C3267T,C5388A,C5986T,T6954C,G7042T,C8499T,A10195G,C10279T,C14120T,C14408T,C14676T,C15279T,T16176C,C16887T,G17427T,A23063T,C23271A,A23403G,C23604A,C23709T,T24506G,G24914C,C26801T,C27972T,G28048T,G28075T,A28095T,A28111G,G28280C,A28281T,T28282A,G28881A,G28882A,G28883C,C28977T	23	N:D3L,N:R203K,N:G204R,N:S235F,ORF1a:T1001I,ORF1a:A1708D,ORF1a:I2230T,ORF1a:M2259I,ORF1a:A2745V,ORF1b:P218L,ORF1b:P314L,ORF8:Q27*,ORF8:R52I,ORF8:C61F,ORF8:K68*,ORF8:Y73C,S:N501Y,S:A570D,S:D614G,S:P681H,S:T716I,S:S982A,S:D1118H	6	0
hCoV-19/Mexico/TAB-InDRE_FB19070_S4489/2021	EPI_ISL_3033343	-	B.1.1.7	GRY	42	C241T,C913T,C920T,C2110T,C3037T,C3267T,C5388A,C5986T,T6954C,G7042T,G8174A,A10195G,C10279T,C14120T,C14408T,C14676T,C15279T,T16176C,C16887T,G17427T,C21914T,A23063T,C23271A,A23403G,C23604A,C23709T,T24506G,G24914C,A25097G,C27972T,G28048T,G28075T,A28095T,A28111G,G28280C,A28281T,T28282A,G28881A,G28882A,G28883C,C28977T,A2970	25	N:D3L,N:R203K,N:G204R,N:S235F,ORF1a:T1001I,ORF1a:A1708D,ORF1a:I2230T,ORF1a:M2259I,ORF1a:A2637T,ORF1b:P218L,ORF1b:P314L,ORF8:Q27*,ORF8:R52I,ORF8:C61F,ORF8:K68*,ORF8:Y73C,S:L118F,S:N501Y,S:A570D,S:D614G,S:P681H,S:T716I,S:S982A,S:D1118H,S:I1179V	6	0
hCoV-19/Mexico/TAB-InDRE_FB19071_S4490/2021	EPI_ISL_3033344	-	B.1.1.7	GRY	39	C241T,C913T,A1981C,C2110T,C3037T,C3267T,C5388A,C5986T,T6954C,G7042T,A10195G,C10279T,C14120T,C14408T,C14676T,C15279T,T16176C,C16887T,G17427T,A23063T,C23271A,A23403G,C23604A,C23709T,T24506G,G24914C,G25052T,C27972T,G28048T,G28075T,A28095T,A28111G,G28280C,A28281T,T28282A,G28881A,G28882A,G28883C,C28977T	23	N:D3L,N:R203K,N:G204R,N:S235F,ORF1a:T1001I,ORF1a:A1708D,ORF1a:I2230T,ORF1a:M2259I,ORF1b:P218L,ORF1b:P314L,ORF8:Q27*,ORF8:R52I,ORF8:C61F,ORF8:K68*,ORF8:Y73C,S:N501Y,S:A570D,S:D614G,S:P681H,S:T716I,S:S982A,S:D1118H,S:V1164F	6	0
hCoV-19/Mexico/TAB-InDRE_FB19078_S4491/2021	EPI_ISL_3033345	-	B.1.1.7	GRY	41	T154C,C241T,C313T,C913T,C3037T,C3267T,A5026T,C5388A,C5944T,C5986T,T6954C,C7564T,C8127T,C9967T,C13585T,C14408T,C14676T,T15096C,C15279T,T16176C,G21901A,C22987T,A23063T,C23271A,A23403G,C23604A,C23709T,T24506G,G24914C,C27972T,G28048T,A28095T,A28111G,G28280C,A28281T,T28282A,G28881A,G28882A,G28883C,G28884C,C28977T	20	N:D3L,N:R203K,N:G204P,N:S235F,ORF1a:T1001I,ORF1a:A1708D,ORF1a:I2230T,ORF1a:A2621V,ORF1b:P314L,ORF8:Q27*,ORF8:R52I,ORF8:K68*,ORF8:Y73C,S:N501Y,S:A570D,S:D614G,S:P681H,S:T716I,S:S982A,S:D1118H	6	126
hCoV-19/Mexico/TAB-InDRE_FB19079_S4492/2021	EPI_ISL_3033346	-	B.1.1.7	GRY	38	C241T,C913T,C3037T,C3267T,C5388A,C5944T,C5986T,T6954C,C9967T,C13585T,C14408T,C14676T,T15096C,C15279T,T16176C,G17658T,G21901A,C22987T,C22993T,A23063T,C23271A,A23403G,C23604A,C23709T,T24506G,G24914C,C27972T,G28048T,A28095T,A28111G,G28280C,A28281T,T28282A,G28881A,G28882A,G28883C,G28884C,C28977T	20	N:D3L,N:R203K,N:G204P,N:S235F,ORF1a:T1001I,ORF1a:A1708D,ORF1a:I2230T,ORF1b:P314L,ORF1b:M1397I,ORF8:Q27*,ORF8:R52I,ORF8:K68*,ORF8:Y73C,S:N501Y,S:A570D,S:D614G,S:P681H,S:T716I,S:S982A,S:D1118H	6	0
hCoV-19/Mexico/TAB-InDRE_FB19298_S4503/2021	EPI_ISL_3033357	-	B.1.1.7	GRY	43	C241T,C913T,C2110T,C3037T,C3267T,G4300T,C4900T,C5388A,C5986T,T6954C,T7984C,C10747T,C14120T,C14408T,C14676T,C15279T,T16176C,C19390T,A23063T,C23271A,T23323C,A23403G,C23604A,C23709T,T24506G,G24914C,C26176T,C26313T,C27737T,C27972T,G28048T,A28095T,A28111G,G28280C,A28281T,T28282A,G28817T,A28877T,G28878C,G28881A,G28882A,G28	24	N:D3L,N:A182S,N:R203K,N:G204R,N:S235F,ORF1a:T1001I,ORF1a:A1708D,ORF1a:I2230T,ORF1b:P218L,ORF1b:P314L,ORF1b:P1975S,ORF3a:P262S,ORF7a:T115I,ORF8:Q27*,ORF8:R52I,ORF8:K68*,ORF8:Y73C,S:N501Y,S:A570D,S:D614G,S:P681H,S:T716I,S:S982A,S:D1118H	6	0
hCoV-19/Mexico/TAB-InDRE_FB19319_S4504/2021	EPI_ISL_3033358	-	B.1.1.7	GRY	40	C241T,C913T,C2110T,C3037T,C3267T,G4504T,C5388A,C5986T,T6954C,G7042T,A8628G,A10195G,C10279T,C14120T,C14408T,C14676T,C15279T,T16176C,C16887T,G17427T,A23063T,C23271A,A23403G,C23604A,C23709T,T24506G,G24914C,C27972T,G28048T,G28075T,A28095T,A28111G,G28280C,A28281T,T28282A,G28881A,G28882A,G28883C,C28977T,C29149T	24	N:D3L,N:R203K,N:G204R,N:S235F,ORF1a:T1001I,ORF1a:E1413D,ORF1a:A1708D,ORF1a:I2230T,ORF1a:M2259I,ORF1a:Y2788C,ORF1b:P218L,ORF1b:P314L,ORF8:Q27*,ORF8:R52I,ORF8:C61F,ORF8:K68*,ORF8:Y73C,S:N501Y,S:A570D,S:D614G,S:P681H,S:T716I,S:S982A,S:D1118H	6	0
hCoV-19/Mexico/TAB-InDRE_FB19485_S4510/2021	EPI_ISL_3033363	-	B.1.1.7	GRY	42	C241T,C913T,C2110T,C2143T,C3037T,G3047T,C3267T,C5388A,C5986T,C6730T,T6954C,C7945T,A10195G,C10834T,C14120T,C14408T,C14676T,C15279T,T16176C,T20445C,A23063T,C23271A,A23403G,C23604A,C23709T,T24506G,G24914C,C27972T,G28048T,G28075T,A28095T,A28111G,C28236T,G28280C,A28281T,T28282A,G28881A,G28882A,G28883C,C28896G,C28977T,C296	24	N:D3L,N:R203K,N:G204R,N:A208G,N:S235F,ORF1a:D928Y,ORF1a:T1001I,ORF1a:A1708D,ORF1a:I2230T,ORF1b:P218L,ORF1b:P314L,ORF8:Q27*,ORF8:R52I,ORF8:C61F,ORF8:K68*,ORF8:Y73C,ORF8:R115C,S:N501Y,S:A570D,S:D614G,S:P681H,S:T716I,S:S982A,S:D1118H	6	0
hCoV-19/Mexico/TAB-InDRE_FB19500_S4511/2021	EPI_ISL_3033364	-	B.1.1.7	GRY	43	C241T,C913T,C2110T,C3037T,C3267T,C5388A,C5986T,C6016T,T6954C,G7042T,G8174A,A10195G,C10279T,C14120T,C14408T,C14676T,C15279T,T16176C,C16887T,G17427T,C18131T,A23063T,C23271A,A23403G,C23604A,C23709T,T24506G,G24914C,A25097G,C27972T,G28048T,G28075T,A28095T,A28111G,G28280C,A28281T,T28282A,G28812A,G28881A,G28882A,G28883C,C28	26	N:D3L,N:S180N,N:R203K,N:G204R,N:S235F,ORF1a:T1001I,ORF1a:A1708D,ORF1a:I2230T,ORF1a:M2259I,ORF1a:A2637T,ORF1b:P218L,ORF1b:P314L,ORF1b:T1555I,ORF8:Q27*,ORF8:R52I,ORF8:C61F,ORF8:K68*,ORF8:Y73C,S:N501Y,S:A570D,S:D614G,S:P681H,S:T716I,S:S982A,S:D1118H,S:I1179V	6	0

hCoV-19/Mexico/TAB-InDRE_FB19673_S4516/2021	EPI_ISL_3033369	-	B.1.1.7	GRY	39	C241T,C913T,C2110T,C3037T,C3267T,C5388A,C5986T,T6954C,G7042T,A10195G,C10279T,C14120T,C14408T,C14676T,C15279T,T16176C,C16887T,G17427T,A23063T,C23271A,A23403G,C23604A,C23709T,T24506G,G24914C,C27972T,G28048T,G28075T,A28095T,A28111G,G28280C,A28281T,T28282A,G28307A,C28606T,G28881A,G28882A,G28883C,C28977T	24	N:D3L,N:A12T,N:R203K,N:G204R,N:S235F,ORF1a:T1001I,ORF1a:A1708D,ORF1a:I2230T,ORF1a:M2259I,ORF1b:P218L,ORF1b:P314L,ORF8:Q27*,ORF8:R52I,ORF8:C61F,ORF8:K68*,ORF8:Y73C,ORF9b:M8I,S:N501Y,S:A570D,S:D614G,S:P681H,S:T716I,S:S982A,S:D1118H	6	0
hCoV-19/Mexico/TAB-INMEGEN-05-06-245/2021	EPI_ISL_2603720	-	B.1.1.7	GRY	39	C241T,C913T,C2110T,C3037T,C3267T,C5388A,C5986T,C6016T,T6954C,G7042T,A10195G,C10279T,C14120T,C14408T,C14676T,C15279T,T16176C,C16887T,G17427T,A23063T,C23271A,A23403G,C23604A,C23709T,T24506G,G24914C,C27577T,C27972T,G28048T,G28075T,A28095T,A28111G,G28280C,A28281T,T28282A,G28881A,G28882A,G28883C,C28977T	23	N:D3L,N:R203K,N:G204R,N:S235F,ORF1a:T1001I,ORF1a:A1708D,ORF1a:I2230T,ORF1a:M2259I,ORF1b:P218L,ORF1b:P314L,ORF7a:Q62*,ORF8:Q27*,ORF8:R52I,ORF8:C61F,ORF8:K68*,ORF8:Y73C,S:N501Y,S:A570D,S:D614G,S:P681H,S:T716I,S:S982A,S:D1118H	6	0
hCoV-19/Mexico/TAB-INMEGEN-05-06-253/2021	EPI_ISL_2603724	-	B.1.1.7	GRY	37	C241T,C913T,C2110T,C3037T,C3267T,C5388A,C5986T,T6954C,G7042T,A10195G,C10279T,C14120T,C14408T,C14676T,C15279T,T16176C,C16887T,G17427T,A23063T,C23271A,A23403G,C23604A,C23709T,T24506G,G24914C,C27972T,G28048T,G28075T,A28095T,A28111G,G28280C,A28281T,T28282A,G28881A,G28882A,G28883C,C28977T	22	N:D3L,N:R203K,N:G204R,N:S235F,ORF1a:T1001I,ORF1a:A1708D,ORF1a:I2230T,ORF1a:M2259I,ORF1b:P218L,ORF1b:P314L,ORF8:Q27*,ORF8:R52I,ORF8:C61F,ORF8:K68*,ORF8:Y73C,S:N501Y,S:A570D,S:D614G,S:P681H,S:T716I,S:S982A,S:D1118H	6	187
hCoV-19/Mexico/TAB-INMEGEN-05-06-261/2021	EPI_ISL_2603732	-	B.1.1.7	GRY	38	C241T,C913T,C2110T,C3037T,C3267T,C5388A,C5986T,T6954C,G7042T,G8174A,A10195G,C10279T,C14120T,C14408T,C14676T,C15279T,T16176C,C16887T,G17427T,A23063T,C23271A,A23403G,C23604A,C23709T,T24506G,G24914C,C27972T,G28048T,G28075T,A28095T,A28111G,G28280C,A28281T,T28282A,G28881A,G28882A,G28883C,C28977T	23	N:D3L,N:R203K,N:G204R,N:S235F,ORF1a:T1001I,ORF1a:A1708D,ORF1a:I2230T,ORF1a:M2259I,ORF1a:A2637T,ORF1b:P218L,ORF1b:P314L,ORF8:Q27*,ORF8:R52I,ORF8:C61F,ORF8:K68*,ORF8:Y73C,S:N501Y,S:A570D,S:D614G,S:P681H,S:T716I,S:S982A,S:D1118H	6	182
hCoV-19/Mexico/TAB-INMEGEN-06-01-211/2021	EPI_ISL_2616918	-	B.1.1.7	GRY	38	C241T,C913T,C2110T,C3037T,C3267T,C5388A,C5986T,T6954C,G7042T,A10195G,C10279T,C14120T,C14408T,C14676T,C15279T,T16176C,C16887T,G17427T,A23063T,C23271A,A23403G,C23604A,C23709T,T24506G,G24914C,G25052T,C27972T,G28048T,G28075T,A28081G,A28111G,G28280C,A28281T,T28282A,G28881A,G28882A,G28883C,C28977T	23	N:D3L,N:R203K,N:G204R,N:S235F,ORF1a:T1001I,ORF1a:A1708D,ORF1a:I2230T,ORF1a:M2259I,ORF1b:P218L,ORF1b:P314L,ORF8:Q27*,ORF8:R52I,ORF8:C61F,ORF8:D63G,ORF8:Y73C,S:N501Y,S:A570D,S:D614G,S:P681H,S:T716I,S:S982A,S:D1118H,S:V1164F	6	540
hCoV-19/Mexico/TAB-INMEGEN-06-01-219/2021	EPI_ISL_2616902	-	B.1.1.7	GRY	37	C241T,C913T,C2110T,C3037T,C3267T,C5388A,C5986T,T6954C,G7042T,A10195G,C10279T,C14120T,C14408T,C14676T,C15279T,T16176C,C16887T,G17427T,A23063T,C23271A,A23403G,C23604A,C23709T,T24506G,G24914C,G25494T,C27972T,G28048T,G28075T,A28111G,G28280C,A28281T,T28282A,G28881A,G28882A,G28883C,C28977T	21	N:D3L,N:R203K,N:G204R,N:S235F,ORF1a:T1001I,ORF1a:A1708D,ORF1a:I2230T,ORF1a:M2259I,ORF1b:P218L,ORF1b:P314L,ORF8:Q27*,ORF8:R52I,ORF8:C61F,ORF8:Y73C,S:N501Y,S:A570D,S:D614G,S:P681H,S:T716I,S:S982A,S:D1118H	6	617
hCoV-19/Mexico/TAB-INMEGEN-06-01-243/2021	EPI_ISL_2616917	-	B.1.1.7	GRY	38	C241T,C913T,C2110T,C3037T,C3267T,C5388A,C5986T,T6954C,G7042T,G8174A,A10195G,C10279T,C14120T,C14408T,C14676T,C15279T,T16176C,C16887T,G17427T,A23063T,C23271A,A23403G,C23604A,C23709T,T24506G,G24914C,C27972T,G28048T,G28075T,A28095T,A28111G,G28280C,A28281T,T28282A,G28881A,G28882A,G28883C,C28977T	23	N:D3L,N:R203K,N:G204R,N:S235F,ORF1a:T1001I,ORF1a:A1708D,ORF1a:I2230T,ORF1a:M2259I,ORF1a:A2637T,ORF1b:P218L,ORF1b:P314L,ORF8:Q27*,ORF8:R52I,ORF8:C61F,ORF8:K68*,ORF8:Y73C,S:N501Y,S:A570D,S:D614G,S:P681H,S:T716I,S:S982A,S:D1118H	6	554
hCoV-19/Mexico/TAB-INMEGEN-06-01-348/2021	EPI_ISL_2616988	-	B.1.1.7	G	34	C241T,C913T,C2110T,C3037T,C3267T,C5388A,C5986T,G7042T,C7834T,A10195G,C10279T,C14120T,C14408T,C14676T,C15279T,T16176C,C16887T,G17427T,C23271A,A23403G,C23604A,C23709T,T24506G,G24914C,C27972T,G28048T,G28075T,A28095T,A28111G,G28280C,A28281T,T28	18	N:D3L,N:S235F,ORF1a:T1001I,ORF1a:A1708D,ORF1a:M2259I,ORF1b:P218L,ORF1b:P314L,ORF8:Q27*,ORF8:R52I,ORF8:C61F,ORF8:K68*,ORF8:Y73C,S:A570D,S:D614G,S:P681H,S:T716I,S:S982A,S:D1118H	6	1917
hCoV-19/Mexico/TAB-INMEGEN-06-01-364/2021	EPI_ISL_2616972	-	B.1.1.7	GRY	38	C241T,C913T,C2110T,C3037T,C3267T,C5388A,C5986T,T6954C,G7042T,A10195G,C10279T,C14120T,C14408T,C14676T,C15279T,T16176C,C16887T,G17427T,A23063T,C23271A,A23403G,C23604A,C23709T,T24506G,G24914C,C27972T,G28048T,G28075T,G28085T,A28095T,A28111G,G28280C,A28281T,T28282A,G28881A,G28882A,G28883C,C28977T	23	N:D3L,N:R203K,N:G204R,N:S235F,ORF1a:T1001I,ORF1a:A1708D,ORF1a:I2230T,ORF1a:M2259I,ORF1b:P218L,ORF1b:P314L,ORF8:Q27*,ORF8:R52I,ORF8:C61F,ORF8:E64D,ORF8:K68*,ORF8:Y73C,S:N501Y,S:A570D,S:D614G,S:P681H,S:T716I,S:S982A,S:D1118H	6	7
hCoV-19/Mexico/TAB-INMEGEN-06-02-102/2021	EPI_ISL_2692630	-	B.1.1.7	GRY	37	C241T,C913T,C2110T,C3037T,C3267T,C5388A,C5986T,T6954C,G7042T,G8174A,A10195G,C10279T,C14120T,C14408T,C14676T,C15279T,T16176C,C16887T,G17427T,A23063T,C23271A,A23403G,C23604A,C23709T,T24506G,G24914C,C27972T,G28048T,G28075T,A28111G,G28280C,A28281T,T28282A,G28881A,G28882A,G28883C,C28977T	22	N:D3L,N:R203K,N:G204R,N:S235F,ORF1a:T1001I,ORF1a:A1708D,ORF1a:I2230T,ORF1a:M2259I,ORF1a:A2637T,ORF1b:P218L,ORF1b:P314L,ORF8:Q27*,ORF8:R52I,ORF8:C61F,ORF8:Y73C,S:N501Y,S:A570D,S:D614G,S:P681H,S:T716I,S:S982A,S:D1118H	6	0
hCoV-19/Mexico/TAB-INMEGEN-06-02-158/2021	EPI_ISL_2692674	-	B.1.1.7	GRY	37	C241T,C913T,C2110T,C3037T,C3267T,C5388A,C5986T,T6954C,G7042T,A10195G,C10279T,C14120T,C14408T,C14676T,C15279T,T16176C,C16887T,G17427T,A23063T,C23271A,A23403G,C23604A,C23709T,T24506G,G24914C,C27972T,G28048T,G28075T,A28111G,G28280C,A28281T,T28282A,C28606T,G28881A,G28882A,G28883C,C28977T	21	N:D3L,N:R203K,N:G204R,N:S235F,ORF1a:T1001I,ORF1a:A1708D,ORF1a:I2230T,ORF1a:M2259I,ORF1b:P218L,ORF1b:P314L,ORF8:Q27*,ORF8:R52I,ORF8:C61F,ORF8:Y73C,S:N501Y,S:A570D,S:D614G,S:P681H,S:T716I,S:S982A,S:D1118H	6	156
hCoV-19/Mexico/TAB-INMEGEN-06-02-327/2021	EPI_ISL_2692813	-	B.1.1.7	GRY	38	C222T,C241T,C913T,C2110T,C3037T,C3267T,C4206T,C5388A,C5986T,T6954C,C7006T,G7042T,C9165T,C14120T,C14408T,C14676T,C15279T,T16176C,C17762T,A23063T,C23271A,A23403G,C23604A,C23709T,T24506G,G24914C,T25180C,C27972T,G28048T,A28095T,A28111G,G28280C,A28281T,T28282A,G28881A,G28882A,G28883C,C28977T	24	N:D3L,N:R203K,N:G204R,N:S235F,ORF1a:T1001I,ORF1a:A1314V,ORF1a:A1708D,ORF1a:I2230T,ORF1a:M2259I,ORF1a:T2967I,ORF1b:P218L,ORF1b:P314L,ORF1b:A1432V,ORF8:Q27*,ORF8:R52I,ORF8:K68*,ORF8:Y73C,S:N501Y,S:A570D,S:D614G,S:P681H,S:T716I,S:S982A,S:D1118H	6	0
hCoV-19/Mexico/TAB-INMEGEN-06-03-343/2021	EPI_ISL_2810298	-	B.1.1.7	GR	37	C222T,C241T,C913T,C2110T,C3037T,C3267T,C5388A,G6476T,T6954C,G7042T,C12789T,C14120T,C14408T,C14676T,C15240T,C15279T,G15372T,T16176C,C17762T,C23271A,A23403G,C23604A,C23709T,T24506G,G24914C,T25180C,C26885T,C27972T,G28048T,A28111G,G28280C,A28281T,T28282A,G28881A,G28882A,G28883C,C28977T	22	N:D3L,N:R203K,N:G204R,N:S235F,ORF1a:T1001I,ORF1a:A1708D,ORF1a:V2071F,ORF1a:I2230T,ORF1a:M2259I,ORF1a:T4175I,ORF1b:P218L,ORF1b:P314L,ORF1b:A1432V,ORF8:Q27*,ORF8:R52I,ORF8:Y73C,S:A570D,S:D614G,S:P681H,S:T716I,S:S982A,S:D1118H	6	2569
hCoV-19/Mexico/TAB-INMEGEN-06-03-352/2021	EPI_ISL_2810305	-	B.1.1.7	GRY	39	C241T,C913T,C2110T,C3037T,C3267T,C5388A,C5986T,T6954C,G7042T,C9344T,A10195G,C10279T,C14120T,C14408T,C14676T,C15279T,T16176C,C16887T,G17427T,A23063T,C23271A,A23403G,C23604A,C23709T,T24506G,G24914C,G25494T,C27972T,G28048T,G28075T,A28095T,A28111G,G28280C,A28281T,T28282A,G28881A,G28882A,G28883C,C28977T	23	N:D3L,N:R203K,N:G204R,N:S235F,ORF1a:T1001I,ORF1a:A1708D,ORF1a:I2230T,ORF1a:M2259I,ORF1a:L3027F,ORF1b:P218L,ORF1b:P314L,ORF8:Q27*,ORF8:R52I,ORF8:C61F,ORF8:K68*,ORF8:Y73C,S:N501Y,S:A570D,S:D614G,S:P681H,S:T716I,S:S982A,S:D1118H	6	0
hCoV-19/Mexico/TAB-INMEGEN-06-03-360/2021	EPI_ISL_2810312	-	B.1.1.7	GRY	38	C241T,C913T,C2110T,C3037T,C3267T,C5388A,C5986T,T6954C,G7042T,A10195G,C10279T,C14120T,C14408T,C14676T,C15279T,T16176C,C16887T,G17427T,A23063T,C23271A,A23403G,C23604A,C23709T,T24506G,G24914C,C27972T,G28048T,G28075T,A28095T,A28111G,G28280C,A28281T,T28282A,C28606T,G28881A,G28882A,G28883C,C28977T	22	N:D3L,N:R203K,N:G204R,N:S235F,ORF1a:T1001I,ORF1a:A1708D,ORF1a:I2230T,ORF1a:M2259I,ORF1b:P218L,ORF1b:P314L,ORF8:Q27*,ORF8:R52I,ORF8:C61F,ORF8:K68*,ORF8:Y73C,S:N501Y,S:A570D,S:D614G,S:P681H,S:T716I,S:S982A,S:D1118H	6	0
hCoV-19/Mexico/TAB-INMEGEN-06-04-326/2021	EPI_ISL_2894372	-	B.1.1.7	GRY	39	C241T,C913T,A1759G,C3037T,C3267T,C5388A,C5944T,C5986T,T6954C,C9967T,C13585T,C14408T,C14676T,T15096C,C15279T,T16176C,G17658T,G21901A,C22987T,C22993T,A23063T,C23271A,A23403G,C23604A,C23709T,T24506G,G24914C,C27972T,G28048T,A28095T,A28111G,G28280C,A28281T,T28282A,G28881A,G28882A,G28883C,G28884C,C28977T	20	N:D3L,N:R203K,N:G204P,N:S235F,ORF1a:T1001I,ORF1a:A1708D,ORF1a:I2230T,ORF1b:P314L,ORF1b:M1397I,ORF8:Q27*,ORF8:R52I,ORF8:K68*,ORF8:Y73C,S:N501Y,S:A570D,S:D614G,S:P681H,S:T716I,S:S982A,S:D1118H	6	0
hCoV-19/Mexico/TAB-INMEGEN-06-04-329/2021	EPI_ISL_2894375	-	B.1.1.7	GRY	39	C241T,C913T,A1759G,C3037T,C3267T,C5388A,C5944T,C5986T,T6954C,C9967T,C13585T,C14408T,C14676T,T15096C,C15279T,T16176C,G17658T,G21901A,C22987T,C22993T,A23063T,C23271A,A23403G,C23604A,C23709T,T24506G,G24914C,C27972T,G28048T,A28095T,A28111G,G28280C,A28281T,T28282A,G28881A,G28882A,G28883C,G28884C,C28977T	20	N:D3L,N:R203K,N:G204P,N:S235F,ORF1a:T1001I,ORF1a:A1708D,ORF1a:I2230T,ORF1b:P314L,ORF1b:M1397I,ORF8:Q27*,ORF8:R52I,ORF8:K68*,ORF8:Y73C,S:N501Y,S:A570D,S:D614G,S:P681H,S:T716I,S:S982A,S:D1118H	6	19
hCoV-19/Mexico/TAB-INMEGEN-16-306/2021	EPI_ISL_2978616	-	B.1.1.7	GRY	37	C241T,C913T,C2110T,C3037T,C3267T,C5388A,C5986T,T6954C,G7042T,A10195G,C10279T,C14120T,C14408T,C14676T,C15279T,T16176C,C16887T,G17427T,A23063T,C23271A,A23403G,C23604A,C23709T,T24506G,G24914C,C27972T,G28048T,G28075T,A28095T,A28111G,G28280C,A28281T,T28282A,G28881A,G28882A,G28883C,C28977T	22	N:D3L,N:R203K,N:G204R,N:S235F,ORF1a:T1001I,ORF1a:A1708D,ORF1a:I2230T,ORF1a:M2259I,ORF1b:P218L,ORF1b:P314L,ORF8:Q27*,ORF8:R52I,ORF8:C61F,ORF8:K68*,ORF8:Y73C,S:N501Y,S:A570D,S:D614G,S:P681H,S:T716I,S:S982A,S:D1118H	6	1
hCoV-19/Mexico/TAM-InDRE_236/2021	EPI_ISL_961466	-	B.1.1.7	GRY	32	C241T,C913T,C3037T,C3267T,C5388A,C5986T,T6954C,C14408T,C14676T,C15279T,T16176C,A17615G,C18032T,A23063T,C23271A,A23403G,C23604A,C23709T,T24506G,G24914C,G26730C,C27972T,G28048T,A28111G,G28280C,A28281T,T28282A,C28603T,G28881A,G28882A,G28883C,	21	M:V70L,N:D3L,N:R203K,N:G204R,N:S235F,ORF1a:T1001I,ORF1a:A1708D,ORF1a:I2230T,ORF1b:P314L,ORF1b:K1383R,ORF1b:T1522I,ORF8:Q27*,ORF8:R52I,ORF8:Y73C,S:N501Y,S:A570D,S:D614G,S:P681H,S:T716I,S:S982A,S:D1118H	6	0
hCoV-19/Mexico/TAM-InDRE_244/2021	EPI_ISL_1008713	-	B.1.1.7	GRY	34	C241T,C292T,C913T,C3037T,C3267T,C5388A,C5986T,T6954C,C14408T,C14676T,C15279T,T16176C,A23063T,C23170T,C23271A,A23403G,C23604A,C23709T,T24506G,G24914C,C25626T,C27972T,G28048T,A28111G,G28280C,A28281T,T28282A,A28295C,G28881A,G28882A,G28883C,C2	20	N:D3L,N:N8H,N:R203K,N:G204R,N:S235F,ORF1a:T1001I,ORF1a:A1708D,ORF1a:I2230T,ORF1b:P314L,ORF8:Q27*,ORF8:R52I,ORF8:Y73C,ORF9b:K4N,S:N501Y,S:A570D,S:D614G,S:P681H,S:T716I,S:S982A,S:D1118H	6	294

hCoV-19/Mexico/TAM-InDRE_F108L_S569/2021	EPI_ISL_1334382	-	B.1.1.7	GRY	33	C241T,C292T,C913T,C3037T,C3267T,C5388A,C5986T,T6954C,C14408T,C14676T,C15279T,T16176C,T16563A,A23063T,C23170T,C23271A,A23403G,C23604A,C23709T,T24506G,G24914C,C25626T,C27972T,G28048T,A28111G,G28280C,A28281T,T28282A,A28295C,G28881A,G28882A,G28	20	N:D3L,N:N8H,N:R203K,N:G204R,N:S235F,ORF1a:T1001I,ORF1a:A1708D,ORF1a:I2230T,ORF1b:P314L,ORF8:Q27*,ORF8:R52I,ORF8:Y73C,ORF9b:K4N,S:N501Y,S:A570D,S:D614G,S:P681H,S:T716I,S:S982A,S:D1118H	6	9
hCoV-19/Mexico/TAM-InDRE_F111L_S570/2021	EPI_ISL_1334383	-	B.1.1.7	GRY	34	C241T,C292T,C913T,C3037T,C3267T,C5388A,C5986T,T6954C,C14408T,C14676T,C15279T,T16176C,T16563A,G18782T,A23063T,C23170T,C23271A,A23403G,C23604A,C23709T,T24506G,G24914C,C25626T,C27972T,G28048T,A28111G,G28280C,A28281T,T28282A,A28295C,G28881A,G28	21	N:D3L,N:N8H,N:R203K,N:G204R,N:S235F,ORF1a:T1001I,ORF1a:A1708D,ORF1a:I2230T,ORF1b:P314L,ORF1b:G1772V,ORF8:Q27*,ORF8:R52I,ORF8:Y73C,ORF9b:K4N,S:N501Y,S:A570D,S:D614G,S:P681H,S:T716I,S:S982A,S:D1118H	6	0
hCoV-19/Mexico/TAM-InDRE_F112L_S571/2021	EPI_ISL_1334384	-	B.1.1.7	GRY	34	C241T,C292T,C913T,C3037T,C3267T,C5388A,C5986T,T6954C,C14408T,C14676T,C15279T,T16176C,T16563A,G22317A,A23063T,C23170T,C23271A,A23403G,C23604A,C23709T,T24506G,G24914C,C25626T,C27972T,G28048T,A28111G,G28280C,A28281T,T28282A,A28295C,G28881A,G28	21	N:D3L,N:N8H,N:R203K,N:G204R,N:S235F,ORF1a:T1001I,ORF1a:A1708D,ORF1a:I2230T,ORF1b:P314L,ORF8:Q27*,ORF8:R52I,ORF8:Y73C,ORF9b:K4N,S:G252D,S:N501Y,S:A570D,S:D614G,S:P681H,S:T716I,S:S982A,S:D1118H	6	117
hCoV-19/Mexico/TAM-InDRE_F114L_S572/2021	EPI_ISL_1334385	-	B.1.1.7	GRY	35	C241T,C913T,C2334T,C2485T,C3037T,C3267T,C5388A,C5986T,G6894T,T6954C,T8603C,C14408T,C14676T,C15279T,T16176C,A17615G,C18555T,A23063T,C23271A,A23403G,C23604A,C23709T,T24506G,G24914C,G25437T,C27972T,G28048T,A28111G,G28280C,A28281T,T28282A,G28881A,G28882A,G28883C,C28977T	23	N:D3L,N:R203K,N:G204R,N:S235F,ORF1a:A690V,ORF1a:T1001I,ORF1a:A1708D,ORF1a:C2210F,ORF1a:I2230T,ORF1a:F2780L,ORF1b:P314L,ORF1b:K1383R,ORF3a:L15F,ORF8:Q27*,ORF8:R52I,ORF8:Y73C,S:N501Y,S:A570D,S:D614G,S:P681H,S:T716I,S:S982A,S:D1118H	6	0
hCoV-19/Mexico/TAM-InDRE_F11757_S901/2021	EPI_ISL_1423511	-	B.1.1.7	GRY	35	C241T,C913T,C2110T,C3037T,C3267T,C5388A,C5986T,T6954C,G7042T,A10195G,C14120T,C14408T,C14676T,C15279T,C15933T,T16176C,A23063T,C23271A,A23403G,C23604A,C23709T,T24506G,G24914C,A27745G,C27972T,G28048T,G28075T,A28111G,G28280C,A28281T,T28282A,G28881A,G28882A,G28883C,C28977T	22	N:D3L,N:R203K,N:G204R,N:S235F,ORF1a:T1001I,ORF1a:A1708D,ORF1a:I2230T,ORF1a:M2259I,ORF1b:P218L,ORF1b:P314L,ORF7a:R118G,ORF8:Q27*,ORF8:R52I,ORF8:C61F,ORF8:Y73C,S:N501Y,S:A570D,S:D614G,S:P681H,S:T716I,S:S982A,S:D1118H	6	0
hCoV-19/Mexico/TAM-InDRE_F86L_S567/2021	EPI_ISL_1334380	-	B.1.1.7	GRY	36	C241T,C292T,C913T,G1820A,C3037T,C3267T,C5388A,C5986T,T6954C,C14408T,C14676T,C15279T,T16176C,T16563A,T22861C,A23063T,C23170T,C23271A,A23403G,C23604A,C23709T,T24436C,T24506G,G24914C,C25626T,C27972T,G28048T,A28111G,G28280C,A28281T,T28282A,A28295C,G28881A,G28882A,G28883C,C28977T	21	N:D3L,N:N8H,N:R203K,N:G204R,N:S235F,ORF1a:G519S,ORF1a:T1001I,ORF1a:A1708D,ORF1a:I2230T,ORF1b:P314L,ORF8:Q27*,ORF8:R52I,ORF8:Y73C,ORF9b:K4N,S:N501Y,S:A570D,S:D614G,S:P681H,S:T716I,S:S982A,S:D1118H	6	9
hCoV-19/Mexico/TAM-InDRE_FB14042_S2071/2021	EPI_ISL_2283711	-	B.1.1.7	GRY	39	C241T,C913T,C2110T,C3037T,C3267T,C5388A,C5986T,T6954C,G7042T,A10195G,T12946C,C14120T,C14408T,C14676T,C15279T,T16176C,C20946T,A23063T,C23271A,A23403G,C23604A,C23709T,T24506G,G24872T,G24914C,A25165C,C26607T,C27972T,G28048T,G28075T,A28095T,A28111G,G28280C,A28281T,T28282A,G28881A,G28882A,G28883C,C28977T	25	M:L29F,N:D3L,N:R203K,N:G204R,N:S235F,ORF1a:T1001I,ORF1a:A1708D,ORF1a:I2230T,ORF1a:M2259I,ORF1b:P218L,ORF1b:P314L,ORF8:Q27*,ORF8:R52I,ORF8:C61F,ORF8:K68*,ORF8:Y73C,S:N501Y,S:A570D,S:D614G,S:P681H,S:T716I,S:S982A,S:V1104L,S:D1118H,S:Q1201H	6	0
hCoV-19/Mexico/TAM-InDRE_FB14045_S2072/2021	EPI_ISL_2283712	-	B.1.1.7	GRY	34	C241T,C292T,C913T,C2147A,C3037T,C3267T,A3963G,C5388A,C14408T,C14676T,C15279T,T16176C,T16563A,G22249A,A23063T,C23170T,C23271A,A23403G,C23604A,C23709T,T24506G,G24914C,C25626T,A26612G,C27972T,G28048T,A28111G,G28280C,A28281T,T28282A,A28295C,G2	20	N:D3L,N:N8H,N:R203K,N:G204R,ORF1a:L628I,ORF1a:T1001I,ORF1a:K1233R,ORF1a:A1708D,ORF1b:P314L,ORF8:Q27*,ORF8:R52I,ORF8:Y73C,ORF9b:K4N,S:N501Y,S:A570D,S:D614G,S:P681H,S:T716I,S:S982A,S:D1118H	6	840
hCoV-19/Mexico/TAM-InDRE_FB14051_S2073/2021	EPI_ISL_2283713	-	B.1.1.7	GRY	36	C241T,C913T,C2110T,C3037T,C3267T,C5388A,C5986T,T6954C,G7042T,A10195G,C14120T,C14408T,C14676T,C15279T,C15933T,T16176C,A23063T,C23271A,A23403G,C23604A,C23709T,T24506G,G24914C,A27745G,C27972T,G28048T,G28075T,A28095T,A28111G,G28280C,A28281T,T28282A,G28881A,G28882A,G28883C,C28977T	23	N:D3L,N:R203K,N:G204R,N:S235F,ORF1a:T1001I,ORF1a:A1708D,ORF1a:I2230T,ORF1a:M2259I,ORF1b:P218L,ORF1b:P314L,ORF7a:R118G,ORF8:Q27*,ORF8:R52I,ORF8:C61F,ORF8:K68*,ORF8:Y73C,S:N501Y,S:A570D,S:D614G,S:P681H,S:T716I,S:S982A,S:D1118H	6	0
hCoV-19/Mexico/TAM-InDRE_FB14052_S2074/2021	EPI_ISL_2283714	-	B.1.1.7	GRY	34	C241T,C913T,C2110T,C3037T,C3267T,C5388A,C5986T,T6954C,A10195G,C14120T,C14408T,C14676T,C15279T,C15933T,T16176C,A23063T,C23271A,A23403G,C23604A,C23709T,T24506G,G24914C,A27745G,C27972T,G28048T,G28075T,A28111G,G28280C,A28281T,T28282A,G28881A,G	21	N:D3L,N:R203K,N:G204R,N:S235F,ORF1a:T1001I,ORF1a:A1708D,ORF1a:I2230T,ORF1b:P218L,ORF1b:P314L,ORF7a:R118G,ORF8:Q27*,ORF8:R52I,ORF8:C61F,ORF8:Y73C,S:N501Y,S:A570D,S:D614G,S:P681H,S:T716I,S:S982A,S:D1118H	6	556
hCoV-19/Mexico/TAM-InDRE_FB14054_S2075/2021	EPI_ISL_2283715	-	B.1.1.7	GRY	37	C241T,C913T,C2110T,C3037T,C3267T,C5388A,C5986T,T6954C,G7042T,A10195G,C14120T,C14408T,C14676T,C15279T,C15933T,T16176C,A21137G,A23063T,C23271A,A23403G,C23604A,C23709T,T24506G,G24914C,A27745G,C27972T,G28048T,G28075T,A28095T,A28111G,G28280C,A28281T,T28282A,G28881A,G28882A,G28883C,C28977T	24	N:D3L,N:R203K,N:G204R,N:S235F,ORF1a:T1001I,ORF1a:A1708D,ORF1a:I2230T,ORF1a:M2259I,ORF1b:P218L,ORF1b:P314L,ORF1b:K2557R,ORF7a:R118G,ORF8:Q27*,ORF8:R52I,ORF8:C61F,ORF8:K68*,ORF8:Y73C,S:N501Y,S:A570D,S:D614G,S:P681H,S:T716I,S:S982A,S:D1118H	6	0
hCoV-19/Mexico/TAM-InDRE_FB14060_S2076/2021	EPI_ISL_2283716	-	B.1.1.7	GRY	37	C241T,G242T,C292T,C913T,C3037T,C3267T,C5388A,C5986T,T6954C,C9994T,G12345A,C14408T,C14676T,C15279T,T16176C,T16563A,A23063T,C23170T,C23271A,A23403G,C23604A,C23709T,T24506G,G24914C,C25626T,C27972T,G28048T,A28111G,G28280C,A28281T,T28282A,A28295C,T28825G,G28881A,G28882A,G28883C,C28977T	21	N:D3L,N:N8H,N:R203K,N:G204R,N:S235F,ORF1a:T1001I,ORF1a:A1708D,ORF1a:I2230T,ORF1a:S4027N,ORF1b:P314L,ORF8:Q27*,ORF8:R52I,ORF8:Y73C,ORF9b:K4N,S:N501Y,S:A570D,S:D614G,S:P681H,S:T716I,S:S982A,S:D1118H	6	0
hCoV-19/Mexico/TAM-InDRE_FB14700_S2045/2021	EPI_ISL_2283687	-	B.1.1.7	GRY	38	C241T,C913T,C2110T,C3037T,C3267T,C5388A,C5986T,T6954C,G7042T,A10195G,C14120T,C14408T,C14676T,C15279T,C15933T,T16176C,G22104C,A23063T,C23271A,A23403G,C23604A,C23709T,T24506G,G24914C,A27745G,C27972T,G28048T,G28075T,A28095T,A28111G,G28280C,A28281T,T28282A,G28881A,G28882A,G28883C,C28977T,T29867A	24	N:D3L,N:R203K,N:G204R,N:S235F,ORF1a:T1001I,ORF1a:A1708D,ORF1a:I2230T,ORF1a:M2259I,ORF1b:P218L,ORF1b:P314L,ORF7a:R118G,ORF8:Q27*,ORF8:R52I,ORF8:C61F,ORF8:K68*,ORF8:Y73C,S:G181A,S:N501Y,S:A570D,S:D614G,S:P681H,S:T716I,S:S982A,S:D1118H	6	0
hCoV-19/Mexico/TAM-InDRE_FB14924_S2391/2021	EPI_ISL_2455916	-	B.1.1.7	GRY	34	C241T,C913T,C3037T,C3267T,C5388A,C5986T,T6954C,C14408T,C14676T,C15279T,T16176C,C18501T,C21622T,A23063T,C23271A,A23403G,C23533T,C23604A,C23709T,C24381T,T24506G,G24914C,G25290T,A25336C,C27972T,G28048T,A28111G,G28280C,A28281T,T28282A,G28881A,G28882A,G28883C,C28977T	21	N:D3L,N:R203K,N:G204R,N:S235F,ORF1a:T1001I,ORF1a:A1708D,ORF1a:I2230T,ORF1b:P314L,ORF8:Q27*,ORF8:R52I,ORF8:Y73C,S:N501Y,S:A570D,S:D614G,S:P681H,S:T716I,S:S940F,S:S982A,S:D1118H,S:C1243F,S:E1258D	6	0
hCoV-19/Mexico/TAM-InDRE_FB14930_S2051/2021	EPI_ISL_2283693	-	B.1.1.7	GRY	41	C241T,C913T,C2110T,C3037T,C3267T,C5388A,C5986T,T6954C,G7042T,A10195G,C10809T,C14120T,C14408T,G14515T,C14676T,C15279T,C15933T,T16176C,G22103C,C22450T,A23063T,C23271A,A23403G,C23604A,C23709T,T24506G,G24914C,A27745G,C27972T,G28048T,G28075T,A28111G,G28280C,A28281T,T28282A,G28881A,G28882A,G28883C,C28977T,G29862T,A29866C	25	N:D3L,N:R203K,N:G204R,N:S235F,ORF1a:T1001I,ORF1a:A1708D,ORF1a:I2230T,ORF1a:M2259I,ORF1a:P3515L,ORF1b:P218L,ORF1b:P314L,ORF1b:V350L,ORF7a:R118G,ORF8:Q27*,ORF8:R52I,ORF8:C61F,ORF8:Y73C,S:G181R,S:N501Y,S:A570D,S:D614G,S:P681H,S:T716I,S:S982A,S:D1118H	6	0
hCoV-19/Mexico/TAM-InDRE_FB14936_S2591/2021	EPI_ISL_2492487	-	B.1.1	GR	33	C241T,C913T,C1288T,C2110T,C3037T,C3267T,C5388A,T6954C,C6997T,G7042T,A10195G,C14120T,C14408T,C14676T,C15279T,T16176C,A23063T,C23271A,A23403G,C23604A,T24506G,G24914C,A25336C,C27972T,G28075T,A28111G,G28280C,A28281T,T28282A,G28881A,G28882A,G28	16	N:D3L,N:R203K,N:G204R,N:S235F,ORF1b:P218L,ORF1b:P314L,ORF8:Q27*,ORF8:C61F,ORF8:Y73C,S:N501Y,S:A570D,S:D614G,S:P681H,S:S982A,S:D1118H,S:E1258D	0	1483
hCoV-19/Mexico/TAM-InDRE_FB14939_S2393/2021	EPI_ISL_2455918	-	B.1.1.7	GRY	40	C241T,C913T,T1918C,C2110T,C3037T,C3267T,C4575T,C4719T,C5388A,C5986T,T6954C,G7042T,A10195G,C11750T,C14120T,C14408T,C14676T,C15279T,T16176C,A23063T,C23271A,A23403G,C23604A,C23709T,T24506G,G24914C,A25336C,G26153T,T27380C,C27972T,G28048T,G28075T,A28111G,G28280C,A28281T,T28282A,G28881A,G28882A,G28883C,C28977T	27	N:D3L,N:R203K,N:G204R,N:S235F,ORF1a:T1001I,ORF1a:T1437I,ORF1a:A1485V,ORF1a:A1708D,ORF1a:I2230T,ORF1a:M2259I,ORF1a:L3829F,ORF1b:P218L,ORF1b:P314L,ORF3a:G254V,ORF6:I60T,ORF8:Q27*,ORF8:R52I,ORF8:C61F,ORF8:Y73C,S:N501Y,S:A570D,S:D614G,S:P681H,S:T716I,S:S982A,S:D1118H,S:E125	6	0
hCoV-19/Mexico/TAM-InDRE_FB15935_S2398/2021	EPI_ISL_2455923	-	B.1.1.7	GRY	36	C241T,C292T,C913T,C3037T,C3267T,C5388A,C5986T,T6954C,C8389T,C14408T,C14676T,C15279T,T16176C,T16563A,T22861C,A23063T,C23170T,C23271A,A23403G,C23604A,C23709T,T24506G,G24914C,A25336C,C25626T,C27972T,G28048T,A28111G,G28280C,A28281T,T28282A,A28295C,G28881A,G28882A,G28883C,C28977T	21	N:D3L,N:N8H,N:R203K,N:G204R,N:S235F,ORF1a:T1001I,ORF1a:A1708D,ORF1a:I2230T,ORF1b:P314L,ORF8:Q27*,ORF8:R52I,ORF8:Y73C,ORF9b:K4N,S:N501Y,S:A570D,S:D614G,S:P681H,S:T716I,S:S982A,S:D1118H,S:E1258D	6	6
hCoV-19/Mexico/TAM-InDRE_FB15937_S2399/2021	EPI_ISL_2455924	-	B.1.1.7	GRY	41	C241T,T907C,C913T,C1473T,C2110T,C3037T,C3267T,C5388A,C5986T,T6954C,G7042T,A10195G,C14120T,C14408T,C14676T,C15279T,C15933T,T16176C,C21597T,A23063T,C23271A,A23403G,C23604A,C23709T,T24506G,G24914C,A25336C,G25855T,G26774T,A27745G,C27972T,G28048T,G28075T,A28111G,G28280C,A28281T,T28282A,G28881A,G28882A,G28883C,C28977T	27	M:M84I,N:D3L,N:R203K,N:G204R,N:S235F,ORF1a:T403I,ORF1a:T1001I,ORF1a:A1708D,ORF1a:I2230T,ORF1a:M2259I,ORF1b:P218L,ORF1b:P314L,ORF3a:D155Y,ORF7a:R118G,ORF8:Q27*,ORF8:R52I,ORF8:C61F,ORF8:Y73C,S:S12F,S:N501Y,S:A570D,S:D614G,S:P681H,S:T716I,S:S982A,S:D1118H,S:E1258D	6	0
hCoV-19/Mexico/TAM-InDRE_FB15938_S2400/2021	EPI_ISL_2455925	-	B.1.1.7	GRY	39	C241T,C913T,C2110T,C3037T,C3267T,C5388A,C5986T,T6954C,G7042T,A10195G,C14120T,C14408T,C14676T,C15279T,C15933T,T16176C,A19865G,A23063T,C23271A,A23403G,C23604A,C23709T,T24506G,G24914C,A25336C,A27745G,C27972T,G28048T,G28075T,A28111G,G28280C,A28281T,T28282A,G28487A,G28881A,G28882A,G28883C,C28977T,C29367A	26	N:D3L,N:V72I,N:R203K,N:G204R,N:S235F,N:P365Q,ORF1a:T1001I,ORF1a:A1708D,ORF1a:I2230T,ORF1a:M2259I,ORF1b:P218L,ORF1b:P314L,ORF1b:N2133S,ORF7a:R118G,ORF8:Q27*,ORF8:R52I,ORF8:C61F,ORF8:Y73C,S:N501Y,S:A570D,S:D614G,S:P681H,S:T716I,S:S982A,S:D1118H,S:E1258D	6	17

hCoV-19/Mexico/TAM-InDRE_FB15942_S2406/2021	EPI_ISL_2455930	-	B.1.1.7	GRY	41	C241T,C913T,C2110T,C3037T,C3267T,C5388A,C5986T,C6651T,T6954C,G7042T,A10195G,C12855T,C14120T,C14408T,C14676T,C15279T,C15933T,T16176C,G19872T,A23063T,C23271A,A23403G,C23604A,C23709T,T24506G,G24914C,A25336C,T25717G,C26936T,A27745G,C27972T,G28048T,G28075T,A28111G,G28280C,A28281T,T28282A,G28881A,G28882A,G28883C,C28977T	26	N:D3L,N:R203K,N:G204R,N:S235F,ORF1a:T1001I,ORF1a:A1708D,ORF1a:A2129V,ORF1a:I2230T,ORF1a:M2259I,ORF1a:P4197L,ORF1b:P218L,ORF1b:P314L,ORF3a:Y109D,ORF7a:R118G,ORF8:Q27*,ORF8:R52I,ORF8:C61F,ORF8:Y73C,S:N501Y,S:A570D,S:D614G,S:P681H,S:T716I,S:S982A,S:D1118H,S:E1258D	6	29
hCoV-19/Mexico/TAM-InDRE_FB15943_S2407/2021	EPI_ISL_2455931	-	B.1.1.7	GRY	42	C110T,C241T,C913T,C2110T,C2607T,C3037T,C3267T,C3543T,C5388A,C5986T,C6762T,T6954C,G7042T,A10195G,C10341T,C14120T,C14408T,C14676T,C15279T,T16176C,A23063T,C23271A,A23403G,C23604A,C23709T,T24506G,G24914C,A25336C,C26645T,C27804T,C27972T,G28048T,G28075T,A28111G,G28280C,A28281T,T28282A,G28881A,G28882A,G28883C,C28977T,G29751T	26	N:D3L,N:R203K,N:G204R,N:S235F,ORF1a:T781I,ORF1a:T1001I,ORF1a:T1093I,ORF1a:A1708D,ORF1a:T2166I,ORF1a:I2230T,ORF1a:M2259I,ORF1a:P3359L,ORF1b:P218L,ORF1b:P314L,ORF8:Q27*,ORF8:R52I,ORF8:C61F,ORF8:Y73C,S:N501Y,S:A570D,S:D614G,S:P681H,S:T716I,S:S982A,S:D1118H,S:E1258D	6	0
hCoV-19/Mexico/TAM-InDRE_FB15944_S2408/2021	EPI_ISL_2455932	-	B.1.1.7	GRY	41	C241T,C292T,C913T,G1820A,C3037T,C3267T,A3963G,C5388A,C5986T,T6954C,C14408T,C14676T,C15279T,T16176C,T16563A,G22249A,A23063T,C23170T,C23271A,A23403G,C23604A,C23709T,T24506G,G24914C,A25336C,C25626T,G26506T,A26612G,C27972T,G28048T,A28111G,G28116T,G28280C,A28281T,T28282A,A28295C,C28657T,G28881A,G28882A,G28883C,C28977T	24	N:D3L,N:N8H,N:R203K,N:G204R,N:S235F,ORF1a:G519S,ORF1a:T1001I,ORF1a:K1233R,ORF1a:A1708D,ORF1a:I2230T,ORF1b:P314L,ORF8:Q27*,ORF8:R52I,ORF8:Y73C,ORF8:D75Y,ORF9b:K4N,S:N501Y,S:A570D,S:D614G,S:P681H,S:T716I,S:S982A,S:D1118H,S:E1258D	6	14
hCoV-19/Mexico/TAM-InDRE_FB15955_S2409/2021	EPI_ISL_2455933	-	B.1.1.7	GRY	38	C241T,C913T,C2110T,C3037T,C3267T,C5388A,C5986T,T6954C,G7042T,A10195G,C10279T,C14120T,C14408T,C14676T,C15279T,T16176C,C16887T,G17427T,G21624T,A23063T,C23271A,A23403G,C23604A,C23709T,T24506G,G24914C,A25336C,C27972T,G28048T,G28075T,A28111G,G28280C,A28281T,T28282A,G28881A,G28882A,G28883C,C28977T	23	N:D3L,N:R203K,N:G204R,N:S235F,ORF1a:T1001I,ORF1a:A1708D,ORF1a:I2230T,ORF1a:M2259I,ORF1b:P218L,ORF1b:P314L,ORF8:Q27*,ORF8:R52I,ORF8:C61F,ORF8:Y73C,S:R21I,S:N501Y,S:A570D,S:D614G,S:P681H,S:T716I,S:S982A,S:D1118H,S:E1258D	6	0
hCoV-19/Mexico/TAM-InDRE_FB16011_S2411/2021	EPI_ISL_2455935	-	B.1.1.7	GRY	40	C241T,C913T,C2110T,C2143T,C3037T,C3267T,C5388A,C5986T,T6954C,G7042T,C7945T,A10195G,C10834T,C14120T,C14408T,A14613G,C14676T,C15279T,T16176C,A23063T,C23271A,A23403G,C23604A,C23709T,T24506G,G24914C,A25336C,C27972T,G28048T,G28075T,A28111G,C28236T,G28280C,A28281T,T28282A,G28881A,G28882A,G28883C,C28977T,C29642T	23	N:D3L,N:R203K,N:G204R,N:S235F,ORF1a:T1001I,ORF1a:A1708D,ORF1a:I2230T,ORF1a:M2259I,ORF1b:P218L,ORF1b:P314L,ORF8:Q27*,ORF8:R52I,ORF8:C61F,ORF8:Y73C,ORF8:R115C,S:N501Y,S:A570D,S:D614G,S:P681H,S:T716I,S:S982A,S:D1118H,S:E1258D	6	0
hCoV-19/Mexico/TAM-InDRE_FB16013_S2412/2021	EPI_ISL_2455936	-	B.1.1.7	GRY	38	C241T,C292T,C913T,C3037T,C3267T,C5388A,C5986T,T6954C,C8389T,A10323G,C14408T,C14676T,C15279T,T16176C,T16563A,G20464A,T22861C,A23063T,C23170T,C23271A,A23403G,C23604A,C23709T,T24506G,G24914C,A25336C,C25626T,C27972T,G28048T,A28111G,G28280C,A28281T,T28282A,A28295C,G28881A,G28882A,G28883C,C28977T	23	N:D3L,N:N8H,N:R203K,N:G204R,N:S235F,ORF1a:T1001I,ORF1a:A1708D,ORF1a:I2230T,ORF1a:K3353R,ORF1b:P314L,ORF1b:D2333N,ORF8:Q27*,ORF8:R52I,ORF8:Y73C,ORF9b:K4N,S:N501Y,S:A570D,S:D614G,S:P681H,S:T716I,S:S982A,S:D1118H,S:E1258D	6	4
hCoV-19/Mexico/TAM-InDRE_FB16014_S2413/2021	EPI_ISL_2455937	-	B.1.1.7	GRY	36	C241T,C913T,C2110T,C3037T,C3267T,C5388A,C5986T,T6954C,G7042T,A10195G,C14120T,C14408T,C14676T,C15279T,C15933T,T16176C,A23063T,C23271A,A23403G,C23604A,C23709T,T24506G,G24914C,A25336C,A27745G,C27972T,G28048T,G28075T,A28111G,G28280C,A28281T,T28282A,G28881A,G28882A,G28883C,C28977T	23	N:D3L,N:R203K,N:G204R,N:S235F,ORF1a:T1001I,ORF1a:A1708D,ORF1a:I2230T,ORF1a:M2259I,ORF1b:P218L,ORF1b:P314L,ORF7a:R118G,ORF8:Q27*,ORF8:R52I,ORF8:C61F,ORF8:Y73C,S:N501Y,S:A570D,S:D614G,S:P681H,S:T716I,S:S982A,S:D1118H,S:E1258D	6	11
hCoV-19/Mexico/TAM-InDRE_FB16015_S2414/2021	EPI_ISL_2455938	-	B.1.1.7	GRY	40	C241T,C292T,T649C,C913T,C1471T,C3037T,C3267T,C5388A,C5986T,T6954C,C12115T,C14408T,C14676T,C15279T,T16176C,T16563A,T17343C,T17835C,C19269T,A23063T,C23170T,C23271A,G23401T,A23403G,C23604A,C23709T,T24506G,G24914C,A25336C,C25626T,C26885T,G28280C,A28281T,T28282A,A28295C,G28881A,G28882A,G28883C,C28977T,C29200T	19	N:D3L,N:N8H,N:R203K,N:G204R,N:S235F,ORF1a:T1001I,ORF1a:A1708D,ORF1a:I2230T,ORF1b:P314L,ORF9b:K4N,S:N501Y,S:A570D,S:Q613H,S:D614G,S:P681H,S:T716I,S:S982A,S:D1118H,S:E1258D	6	309
hCoV-19/Mexico/TAM-InDRE_FB16016_S2415/2021	EPI_ISL_2455939	-	B.1.1.7	GRY	42	C241T,T907C,C913T,C1473T,C2110T,C3037T,C3267T,C5388A,C5986T,T6954C,G7042T,A10195G,C14120T,C14408T,C14676T,C15279T,C15933T,T16176C,C21597T,A23063T,C23271A,A23403G,C23604A,C23709T,T24506G,G24914C,A25336C,G25855T,G26774T,C27577T,A27745G,C27972T,G28048T,G28075T,A28111G,G28280C,A28281T,T28282A,G28881A,G28882A,G28883C,C289	28	M:M84I,N:D3L,N:R203K,N:G204R,N:S235F,ORF1a:T403I,ORF1a:T1001I,ORF1a:A1708D,ORF1a:I2230T,ORF1a:M2259I,ORF1b:P218L,ORF1b:P314L,ORF3a:D155Y,ORF7a:Q62*,ORF7a:R118G,ORF8:Q27*,ORF8:R52I,ORF8:C61F,ORF8:Y73C,S:S12F,S:N501Y,S:A570D,S:D614G,S:P681H,S:T716I,S:S982A,S:D1118H,S:E125	6	60
hCoV-19/Mexico/TAM-InDRE_FB16017_S2416/2021	EPI_ISL_2455940	-	B.1.1.7	GRY	41	T201A,C241T,C913T,C1076T,C2110T,C3037T,C3267T,A5116G,C5388A,C5986T,C6388T,T6954C,G7042T,A10195G,C11224T,G11335A,C14120T,C14408T,C14676T,A14856G,C15279T,T16176C,A23063T,C23271A,A23403G,C23604A,C23709T,T24506G,G24914C,A25336C,C27972T,G28048T,G28075T,A28111G,G28280C,A28281T,T28282A,G28881A,G28882A,G28883C,C28977T	23	N:D3L,N:R203K,N:G204R,N:S235F,ORF1a:P271S,ORF1a:T1001I,ORF1a:A1708D,ORF1a:I2230T,ORF1a:M2259I,ORF1b:P218L,ORF1b:P314L,ORF8:Q27*,ORF8:R52I,ORF8:C61F,ORF8:Y73C,S:N501Y,S:A570D,S:D614G,S:P681H,S:T716I,S:S982A,S:D1118H,S:E1258D	6	133
hCoV-19/Mexico/TAM-InDRE_FB16018_S2417/2021	EPI_ISL_2455941	-	B.1.1.7	GRY	39	C241T,C292T,C913T,C1568T,C2727T,C3037T,C3267T,C5388A,C5986T,T6954C,C14408T,C14676T,C15279T,T16176C,T16563A,T21236A,C21597T,T22861C,A23063T,C23170T,C23271A,A23403G,C23604A,C23709T,T24506G,G24914C,C25626T,T25686A,C27972T,G28048T,A28111G,G28280C,A28281T,T28282A,A28295C,G28881A,G28882A,G28883C,C28977T	24	N:D3L,N:N8H,N:R203K,N:G204R,N:S235F,ORF1a:H435Y,ORF1a:T821I,ORF1a:T1001I,ORF1a:A1708D,ORF1a:I2230T,ORF1b:P314L,ORF1b:F2590Y,ORF8:Q27*,ORF8:R52I,ORF8:Y73C,ORF9b:K4N,S:S12F,S:N501Y,S:A570D,S:D614G,S:P681H,S:T716I,S:S982A,S:D1118H	6	125
hCoV-19/Mexico/TAM-InDRE_FB16019_S2418/2021	EPI_ISL_2455942	-	B.1.1.7	GRY	38	C241T,C292T,C913T,C2156T,T2446A,C3037T,C3267T,C5388A,C5986T,T6954C,T8431G,C14408T,C14676T,C15279T,T16176C,T16563A,C16580T,A23063T,C23170T,C23271A,A23403G,C23604A,C23709T,T24506G,G24914C,A25336C,C25626T,C27972T,G28048T,A28111G,G28280C,A28281T,T28282A,A28295C,G28881A,G28882A,G28883C,C28977T	23	N:D3L,N:N8H,N:R203K,N:G204R,N:S235F,ORF1a:L631F,ORF1a:T1001I,ORF1a:A1708D,ORF1a:I2230T,ORF1b:P314L,ORF1b:T1038I,ORF8:Q27*,ORF8:R52I,ORF8:Y73C,ORF9b:K4N,S:N501Y,S:A570D,S:D614G,S:P681H,S:T716I,S:S982A,S:D1118H,S:E1258D	6	0
hCoV-19/Mexico/TAM-InDRE_FB16020_S2419/2021	EPI_ISL_2455943	-	B.1.1.7	GRY	41	C241T,C913T,C2110T,C3037T,C3267T,T3508G,C5388A,C5986T,T6954C,G7042T,A10195G,C14120T,C14408T,C14676T,C15279T,C15933T,T16176C,G22113T,C22450T,A23063T,C23271A,A23403G,C23604A,C23625T,C23709T,T24506G,G24914C,A25336C,A27745G,T27939C,C27972T,G28048T,G28075T,A28111G,G28280C,A28281T,T28282A,G28881A,G28882A,G28883C,C28977T	27	N:D3L,N:R203K,N:G204R,N:S235F,ORF1a:T1001I,ORF1a:N1081K,ORF1a:A1708D,ORF1a:I2230T,ORF1a:M2259I,ORF1b:P218L,ORF1b:P314L,ORF7a:R118G,ORF8:F16L,ORF8:Q27*,ORF8:R52I,ORF8:C61F,ORF8:Y73C,S:G184V,S:N501Y,S:A570D,S:D614G,S:P681H,S:A688V,S:T716I,S:S982A,S:D1118H,S:E1258D	6	0
hCoV-19/Mexico/TAM-InDRE_FB16021_S2420/2021	EPI_ISL_2455944	-	B.1.1.7	GRY	38	C241T,C913T,C3037T,C3267T,C5388A,C5986T,T6954C,C9967T,C14408T,C14676T,T15096C,C15279T,T16176C,C18681T,T21236A,G21901A,C22987T,C22993T,A23063T,C23271A,A23403G,C23604A,C23709T,T24506G,G24914C,A25336C,C27972T,G28048T,A28111G,G28280C,A28281T,T28282A,G28378T,G28881A,G28882A,G28883C,G28884C,C28977T	21	N:D3L,N:R203K,N:G204P,N:S235F,ORF1a:T1001I,ORF1a:A1708D,ORF1a:I2230T,ORF1b:P314L,ORF1b:F2590Y,ORF8:Q27*,ORF8:R52I,ORF8:Y73C,ORF9b:R32L,S:N501Y,S:A570D,S:D614G,S:P681H,S:T716I,S:S982A,S:D1118H,S:E1258D	6	39
hCoV-19/Mexico/TAM-InDRE_FB16022_S2421/2021	EPI_ISL_2455945	-	B.1.1.7	GRY	37	C241T,C292T,C913T,C3037T,C3267T,C5388A,C5986T,T6954C,G12345A,C14408T,C14676T,C15279T,T16176C,T16563A,C20259T,T21967G,A23063T,C23170T,C23271A,A23403G,C23604A,C23709T,T24506G,G24914C,A25336C,C25626T,C27972T,G28048T,A28111G,G28280C,A28281T,T28282A,A28295C,G28881A,G28882A,G28883C,C28977T	23	N:D3L,N:N8H,N:R203K,N:G204R,N:S235F,ORF1a:T1001I,ORF1a:A1708D,ORF1a:I2230T,ORF1a:S4027N,ORF1b:P314L,ORF8:Q27*,ORF8:R52I,ORF8:Y73C,ORF9b:K4N,S:F135L,S:N501Y,S:A570D,S:D614G,S:P681H,S:T716I,S:S982A,S:D1118H,S:E1258D	6	0
hCoV-19/Mexico/TAM-InDRE_FB16023_S2422/2021	EPI_ISL_2455946	-	B.1.1.7	GRY	34	C241T,C913T,C3037T,C3267T,C5388A,C5986T,T6954C,C14408T,C14676T,C15279T,T16176C,C18348T,A23063T,C23271A,A23403G,C23604A,C23709T,G23811A,T24506G,G24914C,G24971A,A25336C,C27972T,G28048T,A28111G,G28209T,G28280C,A28281T,T28282A,G28881A,G28882A,G28883C,C28977T,C29632T	22	N:D3L,N:R203K,N:G204R,N:S235F,ORF1a:T1001I,ORF1a:A1708D,ORF1a:I2230T,ORF1b:P314L,ORF8:Q27*,ORF8:R52I,ORF8:Y73C,ORF8:E106*,S:N501Y,S:A570D,S:D614G,S:P681H,S:T716I,S:S750N,S:S982A,S:D1118H,S:V1137I,S:E1258D	6	0
hCoV-19/Mexico/TAM-InDRE_FB16024_S2423/2021	EPI_ISL_2455947	-	B.1.1.7	GRY	41	C241T,C913T,C2110T,C3037T,C3267T,C5388A,C5986T,T6954C,G7042T,C9430T,A10195G,C12043T,C14120T,C14408T,C14676T,C15279T,C15933T,T16176C,C22450T,A23063T,C23271A,A23403G,C23604A,C23709T,T24506G,G24914C,G25217T,A25336C,A27745G,C27972T,G28048T,G28075T,A28111G,G28280C,A28281T,T28282A,G28881A,G28882A,G28883C,C28977T,G29688T	24	N:D3L,N:R203K,N:G204R,N:S235F,ORF1a:T1001I,ORF1a:A1708D,ORF1a:I2230T,ORF1a:M2259I,ORF1b:P218L,ORF1b:P314L,ORF7a:R118G,ORF8:Q27*,ORF8:R52I,ORF8:C61F,ORF8:Y73C,S:N501Y,S:A570D,S:D614G,S:P681H,S:T716I,S:S982A,S:D1118H,S:G1219C,S:E1258D	6	0
hCoV-19/Mexico/TAM-InDRE_FB16025_S2424/2021	EPI_ISL_2455948	-	B.1.1.7	GRY	37	C241T,C292T,C913T,C3037T,C3267T,C5388A,C5986T,T6954C,G12345A,C14408T,C14676T,C15279T,T16176C,T16563A,C20259T,A23063T,C23170T,C23271A,A23403G,C23604A,C23709T,T24506G,G24914C,A25336C,C25626T,C27972T,G28048T,A28111G,G28280C,A28281T,T28282A,A28295C,G28881A,G28882A,G28883C,C28957T,C28977T	22	N:D3L,N:N8H,N:R203K,N:G204R,N:S235F,ORF1a:T1001I,ORF1a:A1708D,ORF1a:I2230T,ORF1a:S4027N,ORF1b:P314L,ORF8:Q27*,ORF8:R52I,ORF8:Y73C,ORF9b:K4N,S:N501Y,S:A570D,S:D614G,S:P681H,S:T716I,S:S982A,S:D1118H,S:E1258D	6	0
hCoV-19/Mexico/TAM-InDRE_FB16026_S2425/2021	EPI_ISL_2455949	-	B.1.1.7	GRY	37	C241T,C292T,C913T,C2156T,T2446A,C3037T,C3267T,C5388A,C5986T,T6954C,C14408T,C14676T,C15279T,T16176C,T16563A,C16580T,A23063T,C23170T,C23271A,A23403G,C23604A,C23709T,T24506G,G24914C,A25336C,C25626T,C27972T,G28048T,A28111G,G28280C,A28281T,T28282A,A28295C,G28881A,G28882A,G28883C,C28977T	23	N:D3L,N:N8H,N:R203K,N:G204R,N:S235F,ORF1a:L631F,ORF1a:T1001I,ORF1a:A1708D,ORF1a:I2230T,ORF1b:P314L,ORF1b:T1038I,ORF8:Q27*,ORF8:R52I,ORF8:Y73C,ORF9b:K4N,S:N501Y,S:A570D,S:D614G,S:P681H,S:T716I,S:S982A,S:D1118H,S:E1258D	6	0

hCoV-19/Mexico/TAM-InDRE_FB16027_S2426/2021	EPI_ISL_2455950	-	B.1.1.7	GRY	38	C241T,C292T,C913T,C3037T,C3267T,C5388A,C5986T,T6954C,C14408T,C14676T,C15279T,T16176C,T16563A,T21763C,T22861C,A23063T,C23170T,C23271A,A23403G,C23604A,C23709T,T24506G,G24914C,A25336C,C25626T,C26885T,C27972T,G28048T,A28111G,G28280C,A28281T,T28282A,A28295C,G28881A,G28882A,G28883C,C28977T,T29107C	21	N:D3L,N:N8H,N:R203K,N:G204R,N:S235F,ORF1a:T1001I,ORF1a:A1708D,ORF1a:I2230T,ORF1b:P314L,ORF8:Q27*,ORF8:R52I,ORF8:Y73C,ORF9b:K4N,S:N501Y,S:A570D,S:D614G,S:P681H,S:T716I,S:S982A,S:D1118H,S:E1258D	6	0
hCoV-19/Mexico/TAM-InDRE_FB16028_S2427/2021	EPI_ISL_2455951	-	B.1.1.7	GRY	36	C241T,C913T,C3037T,C3267T,C5388A,C5986T,T6954C,C14408T,C14676T,C15279T,A15860T,T16176C,C18348T,T19301C,A23063T,C23271A,A23403G,C23604A,C23709T,G23811A,T24506G,G24914C,G24971A,A25336C,C27972T,G28048T,A28111G,G28209T,G28280C,A28281T,T28282A,G28881A,G28882A,G28883C,C28977T,C29632T	24	N:D3L,N:R203K,N:G204R,N:S235F,ORF1a:T1001I,ORF1a:A1708D,ORF1a:I2230T,ORF1b:P314L,ORF1b:K798I,ORF1b:V1945A,ORF8:Q27*,ORF8:R52I,ORF8:Y73C,ORF8:E106*,S:N501Y,S:A570D,S:D614G,S:P681H,S:T716I,S:S750N,S:S982A,S:D1118H,S:V1137I,S:E1258D	6	0
hCoV-19/Mexico/TAM-InDRE_FB16029_S2428/2021	EPI_ISL_2455952	-	B.1.1.7	GRY	38	C241T,C913T,C1437T,C2453T,C3037T,C3267T,C5388A,C5986T,T6954C,C9611T,C14408T,C14676T,C15279T,T16176C,T19301C,C21008T,C21855T,G21974C,A23063T,C23271A,A23403G,C23604A,C23709T,T24506G,G24914C,A25336C,C27643T,C27972T,G28048T,A28111G,G28280C,A28281T,T28282A,A28295G,G28881A,G28882A,G28883C,C28977T	28	N:D3L,N:N8D,N:R203K,N:G204R,N:S235F,ORF1a:S391F,ORF1a:L730F,ORF1a:T1001I,ORF1a:A1708D,ORF1a:I2230T,ORF1a:L3116F,ORF1b:P314L,ORF1b:V1945A,ORF1b:T2514I,ORF7a:P84S,ORF8:Q27*,ORF8:R52I,ORF8:Y73C,S:S98F,S:D138H,S:N501Y,S:A570D,S:D614G,S:P681H,S:T716I,S:S982A,S:D1118H,S:E1258	6	134
hCoV-19/Mexico/TAM-InDRE_FB16031_S2430/2021	EPI_ISL_2455954	-	B.1.1.7	GRY	34	C241T,C913T,C3037T,C3267T,C5388A,C5986T,T6954C,C14408T,C14676T,C15279T,T16176C,C18348T,A23063T,C23271A,A23403G,C23604A,C23709T,G23811A,T24506G,G24914C,G24971A,A25336C,C27972T,G28048T,A28111G,G28209T,G28280C,A28281T,T28282A,G28881A,G28882A,G28883C,C28977T,C29632T	22	N:D3L,N:R203K,N:G204R,N:S235F,ORF1a:T1001I,ORF1a:A1708D,ORF1a:I2230T,ORF1b:P314L,ORF8:Q27*,ORF8:R52I,ORF8:Y73C,ORF8:E106*,S:N501Y,S:A570D,S:D614G,S:P681H,S:T716I,S:S750N,S:S982A,S:D1118H,S:V1137I,S:E1258D	6	23
hCoV-19/Mexico/TAM-InDRE_FB16032_S2431/2021	EPI_ISL_2455955	-	B.1.1.7	GRY	37	C241T,C292T,G663T,C913T,T1837C,C3037T,C3267T,C5388A,C5986T,T6954C,C14408T,C14676T,C15279T,T16176C,T16563A,A23063T,C23170T,C23271A,A23403G,C23604A,C23709T,T24506G,G24914C,A25336C,C25626T,C27972T,G28048T,A28111G,G28280C,A28281T,T28282A,A28295C,G28881A,G28882A,G28883C,C28977T,C29762T	22	N:D3L,N:N8H,N:R203K,N:G204R,N:S235F,ORF1a:G133V,ORF1a:T1001I,ORF1a:A1708D,ORF1a:I2230T,ORF1b:P314L,ORF8:Q27*,ORF8:R52I,ORF8:Y73C,ORF9b:K4N,S:N501Y,S:A570D,S:D614G,S:P681H,S:T716I,S:S982A,S:D1118H,S:E1258D	10	43
hCoV-19/Mexico/TAM-InDRE_FB16033_S2432/2021	EPI_ISL_2455956	-	B.1.1.7	GRY	41	C241T,C913T,C2110T,C3037T,G3230A,G3231A,T3232A,C3267T,C5388A,C5986T,T6954C,G7042T,A10195G,C14120T,C14408T,C14676T,C15279T,C15933T,T16176C,C19602T,G22104C,A23063T,C23271A,A23403G,C23604A,C23709T,T24506G,G24914C,A25336C,A27745G,C27972T,G28048T,G28075T,A28111G,G28280C,A28281T,T28282A,G28881A,G28882A,G28883C,C28977T	25	N:D3L,N:R203K,N:G204R,N:S235F,ORF1a:G989K,ORF1a:T1001I,ORF1a:A1708D,ORF1a:I2230T,ORF1a:M2259I,ORF1b:P218L,ORF1b:P314L,ORF7a:R118G,ORF8:Q27*,ORF8:R52I,ORF8:C61F,ORF8:Y73C,S:G181A,S:N501Y,S:A570D,S:D614G,S:P681H,S:T716I,S:S982A,S:D1118H,S:E1258D	6	18
hCoV-19/Mexico/TAM-InDRE_FB16034_S2433/2021	EPI_ISL_2455957	-	B.1.1.7	GRY	37	C241T,C913T,C1437T,C2453T,C3037T,C3267T,C5388A,C5986T,T6954C,C9611T,C14408T,C14676T,C15279T,T16176C,C21008T,C21855T,G21974C,A23063T,C23271A,A23403G,C23604A,C23709T,T24506G,G24914C,A25336C,C27643T,C27972T,G28048T,A28111G,G28280C,A28281T,T28282A,A28295G,G28881A,G28882A,G28883C,C28977T	27	N:D3L,N:N8D,N:R203K,N:G204R,N:S235F,ORF1a:S391F,ORF1a:L730F,ORF1a:T1001I,ORF1a:A1708D,ORF1a:I2230T,ORF1a:L3116F,ORF1b:P314L,ORF1b:T2514I,ORF7a:P84S,ORF8:Q27*,ORF8:R52I,ORF8:Y73C,S:S98F,S:D138H,S:N501Y,S:A570D,S:D614G,S:P681H,S:T716I,S:S982A,S:D1118H,S:E1258D	6	127
hCoV-19/Mexico/TAM-InDRE_FB16326_S3938/2021	EPI_ISL_2858952	-	B.1.1.7	GRY	37	C241T,C913T,C2110T,C3037T,C3267T,C5388A,C5986T,T6954C,G7042T,A10195G,C14120T,C14408T,C14676T,C15279T,C15933T,T16176C,A23063T,C23271A,A23403G,C23604A,C23709T,G24095T,T24506G,G24914C,A25336C,A27745G,C27972T,G28048T,G28075T,A28111G,G28280C,A28281T,T28282A,G28881A,G28882A,G28883C,C28977T	24	N:D3L,N:R203K,N:G204R,N:S235F,ORF1a:T1001I,ORF1a:A1708D,ORF1a:I2230T,ORF1a:M2259I,ORF1b:P218L,ORF1b:P314L,ORF7a:R118G,ORF8:Q27*,ORF8:R52I,ORF8:C61F,ORF8:Y73C,S:N501Y,S:A570D,S:D614G,S:P681H,S:T716I,S:A845S,S:S982A,S:D1118H,S:E1258D	6	199
hCoV-19/Mexico/TAM-InDRE_FB16329_S3939/2021	EPI_ISL_2858953	-	B.1.1.7	GRY	41	C241T,C913T,C2110T,C3037T,G3230A,G3231A,T3232A,C3267T,C5388A,C5986T,T6954C,G7042T,A10195G,C14120T,C14408T,C14676T,C15279T,C15933T,T16176C,C19602T,G22104C,A23063T,C23271A,A23403G,C23604A,C23709T,T24506G,G24914C,A25336C,A27745G,C27972T,G28048T,G28075T,A28111G,G28280C,A28281T,T28282A,G28881A,G28882A,G28883C,C28977T	25	N:D3L,N:R203K,N:G204R,N:S235F,ORF1a:G989K,ORF1a:T1001I,ORF1a:A1708D,ORF1a:I2230T,ORF1a:M2259I,ORF1b:P218L,ORF1b:P314L,ORF7a:R118G,ORF8:Q27*,ORF8:R52I,ORF8:C61F,ORF8:Y73C,S:G181A,S:N501Y,S:A570D,S:D614G,S:P681H,S:T716I,S:S982A,S:D1118H,S:E1258D	6	224
hCoV-19/Mexico/TAM-InDRE_FB16726_S2771/2021	EPI_ISL_2533797	-	B.1.1.7	GRY	42	C241T,C913T,C2110T,C3037T,C3267T,C5388A,C5986T,T6954C,G7042T,A10195G,C11455T,C14120T,C14408T,C14676T,C15279T,C15933T,T16176C,C18687T,T21823C,G22103C,C22450T,A23063T,C23271A,A23403G,C23604A,C23709T,T24506G,G24914C,A27038T,A27745G,C27972T,G28048T,G28075T,A28095T,A28111G,G28280C,A28281T,T28282A,G28881A,G28882A,G28883C,C2	24	N:D3L,N:R203K,N:G204R,N:S235F,ORF1a:T1001I,ORF1a:A1708D,ORF1a:I2230T,ORF1a:M2259I,ORF1b:P218L,ORF1b:P314L,ORF7a:R118G,ORF8:Q27*,ORF8:R52I,ORF8:C61F,ORF8:K68*,ORF8:Y73C,S:G181R,S:N501Y,S:A570D,S:D614G,S:P681H,S:T716I,S:S982A,S:D1118H	6	0
hCoV-19/Mexico/TAM-InDRE_FB16727_S2772/2021	EPI_ISL_2533798	-	B.1.1.7	GRY	40	T201A,C241T,C913T,G1976T,C2110T,C3037T,C3267T,C4438T,C5388A,C5477T,C5986T,C6388T,T6954C,G7042T,A10195G,C14120T,C14408T,C14676T,C15279T,T16176C,A23063T,C23271A,A23403G,C23604A,C23709T,T24506G,G24914C,C27972T,G28048T,G28075T,A28095T,A28111G,G28280C,A28281T,T28282A,G28881A,G28882A,G28883C,G28917A,C28977T	25	N:D3L,N:R203K,N:G204R,N:G215D,N:S235F,ORF1a:D571Y,ORF1a:T1001I,ORF1a:A1708D,ORF1a:H1738Y,ORF1a:I2230T,ORF1a:M2259I,ORF1b:P218L,ORF1b:P314L,ORF8:Q27*,ORF8:R52I,ORF8:C61F,ORF8:K68*,ORF8:Y73C,S:N501Y,S:A570D,S:D614G,S:P681H,S:T716I,S:S982A,S:D1118H	6	0
hCoV-19/Mexico/TAM-InDRE_FB16728_S2773/2021	EPI_ISL_2533799	-	B.1.1.7	GRY	38	C241T,C913T,C2110T,C2862T,C3037T,C3267T,C5388A,C5986T,T6954C,G7042T,A10195G,C14120T,C14408T,C14676T,C15279T,C15933T,T16176C,C22450T,A23063T,C23271A,A23403G,C23604A,C23709T,T24506G,G24914C,A27745G,C27972T,G28048T,G28075T,A28095T,A28111G,G28280C,A28281T,T28282A,G28881A,G28882A,G28883C,C28977T	24	N:D3L,N:R203K,N:G204R,N:S235F,ORF1a:T866I,ORF1a:T1001I,ORF1a:A1708D,ORF1a:I2230T,ORF1a:M2259I,ORF1b:P218L,ORF1b:P314L,ORF7a:R118G,ORF8:Q27*,ORF8:R52I,ORF8:C61F,ORF8:K68*,ORF8:Y73C,S:N501Y,S:A570D,S:D614G,S:P681H,S:T716I,S:S982A,S:D1118H	6	0
hCoV-19/Mexico/TAM-InDRE_FB16733_S2774/2021	EPI_ISL_2533800	-	B.1.1.7	GRY	42	C241T,T907C,C913T,C1473T,C2110T,C3037T,C3267T,C5388A,C5986T,T6954C,G7042T,A10195G,C14120T,C14408T,C14676T,C15279T,C15933T,T16176C,C21597T,A23063T,C23271A,A23403G,C23604A,C23709T,T24506G,G24914C,G25855T,G26774T,C27577T,A27745G,C27972T,G28048T,G28075T,A28095T,A28111G,G28280C,A28281T,T28282A,G28881A,G28882A,G28883C,C2897	28	M:M84I,N:D3L,N:R203K,N:G204R,N:S235F,ORF1a:T403I,ORF1a:T1001I,ORF1a:A1708D,ORF1a:I2230T,ORF1a:M2259I,ORF1b:P218L,ORF1b:P314L,ORF3a:D155Y,ORF7a:Q62*,ORF7a:R118G,ORF8:Q27*,ORF8:R52I,ORF8:C61F,ORF8:K68*,ORF8:Y73C,S:S12F,S:N501Y,S:A570D,S:D614G,S:P681H,S:T716I,S:S982A,S:D11	6	0
hCoV-19/Mexico/TAM-InDRE_FB16734_S2775/2021	EPI_ISL_2533801	-	B.1.1.7	GRY	40	C241T,C913T,C2110T,C3037T,C3267T,C5388A,C5986T,T6954C,G7042T,A10195G,C14120T,C14408T,C14676T,C15279T,C15933T,T16176C,C18687T,T21823C,G22103C,C22450T,A23063T,C23271A,A23403G,C23604A,C23709T,T24506G,G24914C,A27745G,C27972T,G28048T,G28075T,A28095T,A28111G,G28280C,A28281T,T28282A,G28881A,G28882A,G28883C,C28977T	24	N:D3L,N:R203K,N:G204R,N:S235F,ORF1a:T1001I,ORF1a:A1708D,ORF1a:I2230T,ORF1a:M2259I,ORF1b:P218L,ORF1b:P314L,ORF7a:R118G,ORF8:Q27*,ORF8:R52I,ORF8:C61F,ORF8:K68*,ORF8:Y73C,S:G181R,S:N501Y,S:A570D,S:D614G,S:P681H,S:T716I,S:S982A,S:D1118H	6	0
hCoV-19/Mexico/TAM-InDRE_FB17060_S3083/2021	EPI_ISL_2663350	-	B.1.1.7	GRY	40	C92T,C241T,C913T,C2110T,C2939T,C3037T,C3267T,G4904T,C5388A,C5986T,T6954C,G7042T,A10195G,C14120T,C14408T,C14676T,C15279T,C15933T,T16176C,A23063T,C23271A,A23403G,C23604A,C23709T,T24506G,G24914C,T27438C,A27745G,C27972T,G28048T,G28075T,A28095T,A28111G,G28280C,A28281T,T28282A,G28881A,G28882A,G28883C,C28977T	25	N:D3L,N:R203K,N:G204R,N:S235F,ORF1a:P892S,ORF1a:T1001I,ORF1a:D1547Y,ORF1a:A1708D,ORF1a:I2230T,ORF1a:M2259I,ORF1b:P218L,ORF1b:P314L,ORF7a:R118G,ORF8:Q27*,ORF8:R52I,ORF8:C61F,ORF8:K68*,ORF8:Y73C,S:N501Y,S:A570D,S:D614G,S:P681H,S:T716I,S:S982A,S:D1118H	6	0
hCoV-19/Mexico/TAM-InDRE_FB17076_S3133/2021	EPI_ISL_2663400	-	B.1.1.7	GRY	39	C241T,C913T,C2110T,C3037T,C3267T,C5388A,C5986T,T6954C,G7042T,G7846A,A10195G,C14120T,C14408T,C14676T,C15279T,C15933T,T16176C,A23063T,C23271A,A23403G,C23604A,C23709T,T24506G,G24914C,C26110T,A27745G,C27972T,G28048T,G28075T,A28095T,A28111G,G28280C,A28281T,T28282A,G28487A,G28881A,G28882A,G28883C,C28977T	25	N:D3L,N:V72I,N:R203K,N:G204R,N:S235F,ORF1a:T1001I,ORF1a:A1708D,ORF1a:I2230T,ORF1a:M2259I,ORF1b:P218L,ORF1b:P314L,ORF3a:P240S,ORF7a:R118G,ORF8:Q27*,ORF8:R52I,ORF8:C61F,ORF8:K68*,ORF8:Y73C,S:N501Y,S:A570D,S:D614G,S:P681H,S:T716I,S:S982A,S:D1118H	6	0
hCoV-19/Mexico/TAM-InDRE_FB17077_S3134/2021	EPI_ISL_2663401	-	B.1.1.7	GRY	40	C241T,C913T,C2110T,C3037T,C3267T,G3974A,C5388A,C5986T,T6954C,G7042T,A10195G,C14120T,C14408T,C14676T,C15279T,C15933T,T16176C,C16869T,A23063T,C23271A,A23403G,C23604A,C23709T,T24506G,G24914C,C25792T,A27745G,C27972T,G28048T,G28075T,A28095T,A28111G,G28280C,A28281T,T28282A,G28881A,G28882A,G28883C,C28977T,G29422C	25	N:D3L,N:R203K,N:G204R,N:S235F,ORF1a:T1001I,ORF1a:E1237K,ORF1a:A1708D,ORF1a:I2230T,ORF1a:M2259I,ORF1b:P218L,ORF1b:P314L,ORF3a:R134C,ORF7a:R118G,ORF8:Q27*,ORF8:R52I,ORF8:C61F,ORF8:K68*,ORF8:Y73C,S:N501Y,S:A570D,S:D614G,S:P681H,S:T716I,S:S982A,S:D1118H	6	0
hCoV-19/Mexico/TAM-InDRE_FB17417_S3927/2021	EPI_ISL_2858941	-	B.1.1.7	GRY	35	C241T,C292T,C913T,C3037T,C3267T,C5388A,C5986T,T6954C,C14408T,C14676T,C15279T,T16176C,T16563A,T22861C,A23063T,C23170T,C23271A,A23403G,C23604A,C23709T,T24506G,G24914C,C25626T,G27463T,C27972T,G28048T,A28111G,G28280C,A28281T,T28282A,A28295C,G28881A,G28882A,G28883C,C28977T	21	N:D3L,N:N8H,N:R203K,N:G204R,N:S235F,ORF1a:T1001I,ORF1a:A1708D,ORF1a:I2230T,ORF1b:P314L,ORF7a:V24F,ORF8:Q27*,ORF8:R52I,ORF8:Y73C,ORF9b:K4N,S:N501Y,S:A570D,S:D614G,S:P681H,S:T716I,S:S982A,S:D1118H	6	0
hCoV-19/Mexico/TAM-InDRE_FB17423_S3928/2021	EPI_ISL_2858942	-	B.1.1.7	GRY	43	T201A,C241T,C913T,C1076T,C2110T,C3037T,C3267T,A5116G,C5388A,C5986T,C6388T,T6954C,G7042T,A10195G,C10332T,C11224T,G11335A,C14120T,C14408T,C14676T,A14856G,C15279T,T16176C,G17511A,A23063T,C23271A,A23403G,C23604A,C23709T,T24506G,G24914C,A25336C,C27972T,G28048T,G28075T,A28111G,G28280C,A28281T,T28282A,G28881A,G28882A,G28883C	24	N:D3L,N:R203K,N:G204R,N:S235F,ORF1a:P271S,ORF1a:T1001I,ORF1a:A1708D,ORF1a:I2230T,ORF1a:M2259I,ORF1a:T3356I,ORF1b:P218L,ORF1b:P314L,ORF8:Q27*,ORF8:R52I,ORF8:C61F,ORF8:Y73C,S:N501Y,S:A570D,S:D614G,S:P681H,S:T716I,S:S982A,S:D1118H,S:E1258D	6	0

hCoV-19/Mexico/TAM-InDRE_FB17425_S3929/2021	EPI_ISL_2858943	-	B.1.1.7	GRY	41	C241T,C913T,C2110T,C3037T,C3267T,C5388A,C5986T,T6954C,G7042T,A10195G,C14120T,C14184T,C14408T,C14676T,C15279T,C15933T,T16176C,C21597T,A23063T,C23271A,A23403G,C23604A,C23709T,T24506G,G24914C,C25413T,C25710T,G25855T,A27745G,G27762T,C27972T,G28048T,G28075T,A28111G,G28280C,A28281T,T28282A,G28881A,G28882A,G28883C,C28977T	25	N:D3L,N:R203K,N:G204R,N:S235F,ORF1a:T1001I,ORF1a:A1708D,ORF1a:I2230T,ORF1a:M2259I,ORF1b:P218L,ORF1b:P314L,ORF3a:D155Y,ORF7a:R118G,ORF7b:E3*,ORF8:Q27*,ORF8:R52I,ORF8:C61F,ORF8:Y73C,S:S12F,S:N501Y,S:A570D,S:D614G,S:P681H,S:T716I,S:S982A,S:D1118H	6	0
hCoV-19/Mexico/TAM-InDRE_FB17463_S3487/2021	EPI_ISL_2779002	-	B.1.1.7	GRY	43	C241T,C913T,C2110T,C3037T,C3267T,G4201T,C5388A,G5554T,C5986T,T6954C,G7042T,A10195G,G12385T,C14120T,C14408T,C14676T,C15279T,C15933T,T16176C,G22103C,A23063T,C23271A,A23403G,C23604A,C23709T,T24506G,G24914C,A25336C,G25337C,A27745G,G27798A,C27972T,G28048T,G28075T,A28111G,G28280C,A28281T,T28282A,G28881A,G28882A,G28883C,C28977T,C29670T	29	N:D3L,N:R203K,N:G204R,N:S235F,ORF1a:T1001I,ORF1a:M1312I,ORF1a:A1708D,ORF1a:K1763N,ORF1a:I2230T,ORF1a:M2259I,ORF1a:L4040F,ORF1b:P218L,ORF1b:P314L,ORF7a:R118G,ORF7b:A15T,ORF8:Q27*,ORF8:R52I,ORF8:C61F,ORF8:Y73C,S:G181R,S:N501Y,S:A570D,S:D614G,S:P681H,S:T716I,S:S982A,S:D1118H,S:E1258D,S:D1259H	6	0
hCoV-19/Mexico/TAM-InDRE_FB17466_S3488/2021	EPI_ISL_2779003	-	B.1.1.7	GRY	43	C110T,C241T,C913T,C2110T,C3037T,C3267T,C3543T,C5388A,C5986T,T6954C,G7042T,A10195G,C10341T,C14120T,C14408T,C14676T,C15279T,T16176C,A17113G,C20451T,A23063T,C23271A,A23403G,C23604A,C23709T,T24506G,G24872T,G24914C,A25336C,G25337C,C26645T,C27972T,G28048T,G28075T,A28111G,G28280C,A28281T,T28282A,C28603T,G28881A,G28882A,G2888	27	N:D3L,N:R203K,N:G204R,N:S235F,ORF1a:T1001I,ORF1a:T1093I,ORF1a:A1708D,ORF1a:I2230T,ORF1a:M2259I,ORF1a:P3359L,ORF1b:P218L,ORF1b:P314L,ORF1b:I1216V,ORF8:Q27*,ORF8:R52I,ORF8:C61F,ORF8:Y73C,S:N501Y,S:A570D,S:D614G,S:P681H,S:T716I,S:S982A,S:V1104L,S:D1118H,S:E1258D,S:D1259H	6	0
hCoV-19/Mexico/TAM-InDRE_FB17471_S3489/2021	EPI_ISL_2779004	-	B.1.1.7	GRY	47	T201A,C241T,C913T,C1076T,C2110T,C3037T,C3267T,A5116G,C5388A,C5986T,C6388T,T6954C,G7042T,A10195G,C11224T,G11335A,C14120T,C14408T,C14676T,A14856G,C15279T,T16176C,G17511A,G22240A,A23063T,C23271A,A23403G,C23604A,C23709T,T24506G,G24914C,A25336C,G25337C,A25450C,C27213T,C27972T,G28048T,G28075T,A28111G,G28280C,A28281T,T28282A,G28881A,G28882A,G28883C,C28977T,G29543T	25	N:D3L,N:R203K,N:G204R,N:S235F,ORF1a:P271S,ORF1a:T1001I,ORF1a:A1708D,ORF1a:I2230T,ORF1a:M2259I,ORF1b:P218L,ORF1b:P314L,ORF3a:I20L,ORF8:Q27*,ORF8:R52I,ORF8:C61F,ORF8:Y73C,S:N501Y,S:A570D,S:D614G,S:P681H,S:T716I,S:S982A,S:D1118H,S:E1258D,S:D1259H	6	0
hCoV-19/Mexico/TAM-InDRE_FB18098_S3951/2021	EPI_ISL_2858965	-	B.1.1.7	GRY	44	T201A,C241T,G529T,C913T,C1076T,C2110T,C3037T,C3267T,A5116G,C5388A,C5986T,C6388T,T6954C,G7042T,A10195G,C11224T,G11335A,C11572T,C14120T,C14408T,C14676T,A14856G,C15279T,T16176C,G17511A,A23063T,C23271A,A23403G,C23604A,C23709T,T24506G,G24914C,C27972T,G28048T,G28075T,A28095T,A28111G,G28280C,A28281T,T28282A,G28881A,G28882A,G	23	N:D3L,N:R203K,N:G204R,N:S235F,ORF1a:P271S,ORF1a:T1001I,ORF1a:A1708D,ORF1a:I2230T,ORF1a:M2259I,ORF1b:P218L,ORF1b:P314L,ORF8:Q27*,ORF8:R52I,ORF8:C61F,ORF8:K68*,ORF8:Y73C,S:N501Y,S:A570D,S:D614G,S:P681H,S:T716I,S:S982A,S:D1118H	6	0
hCoV-19/Mexico/TAM-InDRE_FB18103_S3952/2021	EPI_ISL_2858966	-	B.1.1.7	GRY	37	C241T,C913T,C2110T,C3037T,C3267T,G4904T,C5388A,C5986T,T6954C,G7042T,A10195G,C14120T,C14408T,C14676T,C15279T,C15933T,T16176C,A23063T,C23271A,A23403G,C23604A,C23709T,T24506G,G24914C,A27745G,C27972T,G28048T,G28075T,A28095T,A28111G,G28280C,A28281T,T28282A,G28881A,G28882A,G28883C,C28977T	24	N:D3L,N:R203K,N:G204R,N:S235F,ORF1a:T1001I,ORF1a:D1547Y,ORF1a:A1708D,ORF1a:I2230T,ORF1a:M2259I,ORF1b:P218L,ORF1b:P314L,ORF7a:R118G,ORF8:Q27*,ORF8:R52I,ORF8:C61F,ORF8:K68*,ORF8:Y73C,S:N501Y,S:A570D,S:D614G,S:P681H,S:T716I,S:S982A,S:D1118H	6	0
hCoV-19/Mexico/TAM-InDRE_FB18104_S3953/2021	EPI_ISL_2858967	-	B.1.1.7	GRY	39	C241T,C913T,C1960T,C2110T,C3037T,C3267T,C5388A,C5986T,T6954C,G7042T,A10195G,C14120T,C14408T,C14676T,C15279T,C15933T,T16176C,A23063T,C23271A,A23403G,C23604A,C23709T,T24506G,G24914C,C26110T,A27745G,C27972T,G28048T,G28075T,A28095T,A28111G,G28280C,A28281T,T28282A,G28487A,G28881A,G28882A,G28883C,C28977T	25	N:D3L,N:V72I,N:R203K,N:G204R,N:S235F,ORF1a:T1001I,ORF1a:A1708D,ORF1a:I2230T,ORF1a:M2259I,ORF1b:P218L,ORF1b:P314L,ORF3a:P240S,ORF7a:R118G,ORF8:Q27*,ORF8:R52I,ORF8:C61F,ORF8:K68*,ORF8:Y73C,S:N501Y,S:A570D,S:D614G,S:P681H,S:T716I,S:S982A,S:D1118H	6	0
hCoV-19/Mexico/TAM-InDRE_FB18105_S3954/2021	EPI_ISL_2858968	-	B.1.1.7	GRY	39	C241T,C913T,C2110T,C3037T,C3267T,C5388A,C5986T,T6954C,G7042T,C7318T,A10195G,C14120T,C14408T,C14676T,C15279T,C15933T,T16176C,C22450T,A23063T,C23271A,A23403G,C23604A,C23709T,T24506G,G24914C,A27745G,A27770T,C27972T,G28048T,G28075T,A28095T,A28111G,G28280C,A28281T,T28282A,G28881A,G28882A,G28883C,C28977T	23	N:D3L,N:R203K,N:G204R,N:S235F,ORF1a:T1001I,ORF1a:A1708D,ORF1a:I2230T,ORF1a:M2259I,ORF1b:P218L,ORF1b:P314L,ORF7a:R118G,ORF8:Q27*,ORF8:R52I,ORF8:C61F,ORF8:K68*,ORF8:Y73C,S:N501Y,S:A570D,S:D614G,S:P681H,S:T716I,S:S982A,S:D1118H	6	0
hCoV-19/Mexico/TAM-InDRE_FB18106_S3955/2021	EPI_ISL_2858969	-	B.1.1.7	GRY	42	C241T,C913T,C2110T,C3037T,C3267T,C5388A,C5986T,C6120T,T6954C,G7042T,A10195G,C14120T,C14408T,C14676T,C15279T,C15933T,T16176C,C21597T,A23063T,C23271A,A23403G,C23604A,C23709T,T24506G,G24914C,G25855T,A27745G,G27762T,C27972T,G28048T,G28075T,A28095T,A28111G,G28280C,A28281T,T28282A,G28881A,G28882A,G28883C,G28884T,C28977T,G2	27	N:D3L,N:R203K,N:G204L,N:S235F,ORF1a:T1001I,ORF1a:A1708D,ORF1a:S1952L,ORF1a:I2230T,ORF1a:M2259I,ORF1b:P218L,ORF1b:P314L,ORF3a:D155Y,ORF7a:R118G,ORF7b:E3*,ORF8:Q27*,ORF8:R52I,ORF8:C61F,ORF8:K68*,ORF8:Y73C,S:S12F,S:N501Y,S:A570D,S:D614G,S:P681H,S:T716I,S:S982A,S:D1118H	6	0
hCoV-19/Mexico/TAM-InDRE_FB18115_S3956/2021	EPI_ISL_2858970	-	B.1.1.7	GRY	38	C241T,C913T,C2110T,C3037T,C3267T,C5388A,C5986T,T6954C,G7042T,A10195G,C14120T,C14408T,C14676T,C15279T,C15933T,T16176C,G22103C,C22450T,A23063T,C23271A,A23403G,C23604A,C23709T,T24506G,G24914C,A27745G,C27972T,G28048T,G28075T,A28095T,A28111G,G28280C,A28281T,T28282A,G28881A,G28882A,G28883C,C28977T	24	N:D3L,N:R203K,N:G204R,N:S235F,ORF1a:T1001I,ORF1a:A1708D,ORF1a:I2230T,ORF1a:M2259I,ORF1b:P218L,ORF1b:P314L,ORF7a:R118G,ORF8:Q27*,ORF8:R52I,ORF8:C61F,ORF8:K68*,ORF8:Y73C,S:G181R,S:N501Y,S:A570D,S:D614G,S:P681H,S:T716I,S:S982A,S:D1118H	6	0
hCoV-19/Mexico/TAM-InDRE_FB18116_S3957/2021	EPI_ISL_2858971	-	B.1.1.7	GRY	40	A156G,C241T,C913T,C2110T,C3037T,C3267T,C5388A,C5392T,C5986T,T6954C,G7042T,A10195G,C14120T,C14408T,C14676T,C15279T,C15933T,T16176C,C22450T,A23063T,C23271A,A23403G,C23604A,C23709T,T24506G,G24620T,G24914C,A27745G,C27972T,G28048T,G28075T,A28095T,A28111G,G28280C,A28281T,T28282A,G28881A,G28882A,G28883C,C28977T	24	N:D3L,N:R203K,N:G204R,N:S235F,ORF1a:T1001I,ORF1a:A1708D,ORF1a:I2230T,ORF1a:M2259I,ORF1b:P218L,ORF1b:P314L,ORF7a:R118G,ORF8:Q27*,ORF8:R52I,ORF8:C61F,ORF8:K68*,ORF8:Y73C,S:N501Y,S:A570D,S:D614G,S:P681H,S:T716I,S:S982A,S:A1020S,S:D1118H	7	0
hCoV-19/Mexico/TAM-InDRE_FB18133_S4122/2021	EPI_ISL_2920693	-	B.1.1.7	GRY	39	C241T,C913T,C2110T,C2847T,C3037T,C3267T,C5388A,C5986T,T6954C,G7042T,A10195G,C14120T,C14408T,C14676T,C15279T,C15933T,T16176C,C22450T,A23063T,C23271A,A23403G,C23604A,C23709T,T24506G,G24914C,A25336C,G25337C,A27745G,C27972T,G28048T,G28075T,A28111G,G28280C,A28281T,T28282A,G28881A,G28882A,G28883C,C28977T	25	N:D3L,N:R203K,N:G204R,N:S235F,ORF1a:T861I,ORF1a:T1001I,ORF1a:A1708D,ORF1a:I2230T,ORF1a:M2259I,ORF1b:P218L,ORF1b:P314L,ORF7a:R118G,ORF8:Q27*,ORF8:R52I,ORF8:C61F,ORF8:Y73C,S:N501Y,S:A570D,S:D614G,S:P681H,S:T716I,S:S982A,S:D1118H,S:E1258D,S:D1259H	6	0
hCoV-19/Mexico/TAM-InDRE_FB18134_S4123/2021	EPI_ISL_2920694	-	B.1.1.7	GRY	41	C241T,C690T,C913T,C2110T,C3037T,C3267T,C5388A,C5986T,T6954C,G7042T,A10195G,C14120T,C14408T,C14676T,C15279T,C15933T,T16176C,A21137G,T21823C,G22103C,C22450T,A23063T,C23271A,A23403G,C23604A,C23709T,T24506G,G24914C,A25336C,A27745G,C27972T,G28048T,G28075T,A28111G,G28280C,A28281T,T28282A,G28881A,G28882A,G28883C,C28977T	26	N:D3L,N:R203K,N:G204R,N:S235F,ORF1a:S142L,ORF1a:T1001I,ORF1a:A1708D,ORF1a:I2230T,ORF1a:M2259I,ORF1b:P218L,ORF1b:P314L,ORF1b:K2557R,ORF7a:R118G,ORF8:Q27*,ORF8:R52I,ORF8:C61F,ORF8:Y73C,S:G181R,S:N501Y,S:A570D,S:D614G,S:P681H,S:T716I,S:S982A,S:D1118H,S:E1258D	6	0
hCoV-19/Mexico/TAM-InDRE_FB18137_S4124/2021	EPI_ISL_2920695	-	B.1.1.7	GRY	41	C241T,C913T,C2110T,C3037T,C3267T,C5388A,C5986T,C6120T,T6954C,G7042T,C10039A,A10195G,C11750T,C14120T,C14408T,C14676T,C15279T,C15933T,T16176C,C21597T,A23063T,C23271A,A23403G,C23604A,C23709T,T24506G,G24914C,G25855T,A27745G,G27762T,C27972T,G28048T,G28075T,A28111G,G28280C,A28281T,T28282A,G28881A,G28882A,G28883C,C28977T	27	N:D3L,N:R203K,N:G204R,N:S235F,ORF1a:T1001I,ORF1a:A1708D,ORF1a:S1952L,ORF1a:I2230T,ORF1a:M2259I,ORF1a:L3829F,ORF1b:P218L,ORF1b:P314L,ORF3a:D155Y,ORF7a:R118G,ORF7b:E3*,ORF8:Q27*,ORF8:R52I,ORF8:C61F,ORF8:Y73C,S:S12F,S:N501Y,S:A570D,S:D614G,S:P681H,S:T716I,S:S982A,S:D1118	6	0
hCoV-19/Mexico/TAM-InDRE_FB18138_S4125/2021	EPI_ISL_2920696	-	B.1.1.7	GRY	44	T201A,C241T,G529T,C913T,C1076T,C2110T,C3037T,C3267T,A5116G,C5388A,C5986T,C6388T,T6954C,G7042T,A10195G,C11224T,G11335A,C14120T,C14408T,C14676T,A14856G,C15279T,T16176C,G17511A,A23063T,C23271A,A23403G,C23604A,C23709T,T24506G,G24914C,A25336C,G25337C,C27972T,G28048T,G28075T,A28111G,G28280C,A28281T,T28282A,G28881A,G28882A,	24	N:D3L,N:R203K,N:G204R,N:S235F,ORF1a:P271S,ORF1a:T1001I,ORF1a:A1708D,ORF1a:I2230T,ORF1a:M2259I,ORF1b:P218L,ORF1b:P314L,ORF8:Q27*,ORF8:R52I,ORF8:C61F,ORF8:Y73C,S:N501Y,S:A570D,S:D614G,S:P681H,S:T716I,S:S982A,S:D1118H,S:E1258D,S:D1259H	6	0
hCoV-19/Mexico/TAM-InDRE_FB18142_S4126/2021	EPI_ISL_2920697	-	B.1.1.7	GRY	42	C241T,C913T,C2110T,C3037T,C3267T,C5388A,C5986T,T6954C,G7042T,A10195G,C14120T,C14408T,C14676T,C15279T,C15933T,T16176C,T17268C,C18687T,T21823C,G22103C,C22450T,A23063T,C23271A,A23403G,C23604A,C23709T,T24506G,G24914C,A25336C,G25337C,A27745G,C27972T,G28048T,G28075T,A28111G,G28280C,A28281T,T28282A,G28881A,G28882A,G28883C,	25	N:D3L,N:R203K,N:G204R,N:S235F,ORF1a:T1001I,ORF1a:A1708D,ORF1a:I2230T,ORF1a:M2259I,ORF1b:P218L,ORF1b:P314L,ORF7a:R118G,ORF8:Q27*,ORF8:R52I,ORF8:C61F,ORF8:Y73C,S:G181R,S:N501Y,S:A570D,S:D614G,S:P681H,S:T716I,S:S982A,S:D1118H,S:E1258D,S:D1259H	6	0
hCoV-19/Mexico/TAM-InDRE_FB18143_S4127/2021	EPI_ISL_2920698	-	B.1.1.7	GRY	40	C241T,C913T,C2110T,C3037T,C3267T,C5388A,C5986T,G6839A,T6954C,G7042T,A10195G,C14120T,C14408T,C14676T,C15279T,C15933T,T16176C,G22103C,C22450T,A23063T,C23271A,A23403G,C23604A,C23709T,T24506G,G24914C,A25336C,G25337C,A27745G,C27972T,G28048T,G28075T,A28111G,G28280C,A28281T,T28282A,G28881A,G28882A,G28883C,C28977T	26	N:D3L,N:R203K,N:G204R,N:S235F,ORF1a:T1001I,ORF1a:A1708D,ORF1a:A2192T,ORF1a:I2230T,ORF1a:M2259I,ORF1b:P218L,ORF1b:P314L,ORF7a:R118G,ORF8:Q27*,ORF8:R52I,ORF8:C61F,ORF8:Y73C,S:G181R,S:N501Y,S:A570D,S:D614G,S:P681H,S:T716I,S:S982A,S:D1118H,S:E1258D,S:D1259H	6	0
hCoV-19/Mexico/TAM-InDRE_FB18144_S4128/2021	EPI_ISL_2920699	-	B.1.1.7	GRY	40	C241T,C823T,C913T,C1758T,C2110T,G3004T,C3037T,C3267T,C5388A,C5986T,T6954C,G7042T,C9559T,C14120T,C14408T,C14676T,C15279T,T16176C,A23063T,C23271A,A23403G,C23604A,C23709T,C24099T,C24208T,T24506G,G24914C,A25336C,C26110T,G27877T,C27972T,G28048T,A28111G,G28280C,A28281T,T28282A,G28881A,G28882A,G28883C,C28977T	26	N:D3L,N:R203K,N:G204R,N:S235F,ORF1a:A498V,ORF1a:E913D,ORF1a:T1001I,ORF1a:A1708D,ORF1a:I2230T,ORF1a:M2259I,ORF1b:P218L,ORF1b:P314L,ORF3a:P240S,ORF7b:C41F,ORF8:Q27*,ORF8:R52I,ORF8:Y73C,S:N501Y,S:A570D,S:D614G,S:P681H,S:T716I,S:A846V,S:S982A,S:D1118H,S:E1258D	6	0

hCoV-19/Mexico/TAM-InDRE_FB18145_S4129/2021	EPI_ISL_2920700	-	B.1.1.7	GRY	38	C241T,C913T,C2110T,C3037T,C3267T,C5388A,C5986T,T6954C,G7042T,A10195G,C14120T,C14408T,C14676T,C15279T,C15933T,T16176C,C22450T,A23063T,C23271A,A23403G,C23604A,C23709T,T24506G,G24914C,A25336C,C26885T,A27745G,C27972T,G28048T,G28075T,A28111G,G28280C,A28281T,T28282A,G28881A,G28882A,G28883C,C28977T	23	N:D3L,N:R203K,N:G204R,N:S235F,ORF1a:T1001I,ORF1a:A1708D,ORF1a:I2230T,ORF1a:M2259I,ORF1b:P218L,ORF1b:P314L,ORF7a:R118G,ORF8:Q27*,ORF8:R52I,ORF8:C61F,ORF8:Y73C,S:N501Y,S:A570D,S:D614G,S:P681H,S:T716I,S:S982A,S:D1118H,S:E1258D	6	0
hCoV-19/Mexico/TAM-InDRE_FB18146_S4130/2021	EPI_ISL_2920701	-	B.1.1.7	GRY	40	C241T,C913T,C2110T,C3037T,C3267T,C5388A,C5986T,T6954C,G7042T,A10195G,C14120T,C14408T,C14676T,C15279T,C15933T,T16176C,C18687T,T21823C,G22103C,C22450T,A23063T,C23271A,A23403G,C23604A,C23709T,T24506G,G24914C,A25336C,A27745G,C27972T,G28048T,G28075T,A28111G,G28280C,A28281T,T28282A,G28881A,G28882A,G28883C,C28977T	24	N:D3L,N:R203K,N:G204R,N:S235F,ORF1a:T1001I,ORF1a:A1708D,ORF1a:I2230T,ORF1a:M2259I,ORF1b:P218L,ORF1b:P314L,ORF7a:R118G,ORF8:Q27*,ORF8:R52I,ORF8:C61F,ORF8:Y73C,S:G181R,S:N501Y,S:A570D,S:D614G,S:P681H,S:T716I,S:S982A,S:D1118H,S:E1258D	6	0
hCoV-19/Mexico/TAM-InDRE_FB18874_S4154/2021	EPI_ISL_2920725	-	B.1.1.7	GRY	37	C241T,C292T,C913T,C3037T,C3267T,C5388A,C5986T,C6896T,T6954C,C14408T,C14676T,C15279T,T16176C,T16563A,T22861C,A23063T,C23170T,C23271A,A23403G,C23604A,C23709T,T24506G,G24914C,A25336C,C25626T,C27972T,G28048T,A28111G,G28280C,A28281T,T28282A,A28295C,A28858G,G28881A,G28882A,G28883C,C28977T	21	N:D3L,N:N8H,N:R203K,N:G204R,N:S235F,ORF1a:T1001I,ORF1a:A1708D,ORF1a:I2230T,ORF1b:P314L,ORF8:Q27*,ORF8:R52I,ORF8:Y73C,ORF9b:K4N,S:N501Y,S:A570D,S:D614G,S:P681H,S:T716I,S:S982A,S:D1118H,S:E1258D	6	0
hCoV-19/Mexico/TAM-InDRE_FB18878_S4155/2021	EPI_ISL_2920726	-	B.1.1.7	GRY	40	C241T,C913T,C2110T,C3037T,C3267T,C5388A,C5986T,G6839A,T6954C,G7042T,A10195G,C14120T,C14408T,C14676T,C15279T,C15933T,T16176C,G22103C,C22450T,A23063T,C23271A,A23403G,C23604A,C23709T,T24506G,G24914C,A25336C,G25337C,A27745G,C27972T,G28048T,G28075T,A28111G,G28280C,A28281T,T28282A,G28881A,G28882A,G28883C,C28977T	26	N:D3L,N:R203K,N:G204R,N:S235F,ORF1a:T1001I,ORF1a:A1708D,ORF1a:A2192T,ORF1a:I2230T,ORF1a:M2259I,ORF1b:P218L,ORF1b:P314L,ORF7a:R118G,ORF8:Q27*,ORF8:R52I,ORF8:C61F,ORF8:Y73C,S:G181R,S:N501Y,S:A570D,S:D614G,S:P681H,S:T716I,S:S982A,S:D1118H,S:E1258D,S:D1259H	6	0
hCoV-19/Mexico/TAM-InDRE_FB18879_S4156/2021	EPI_ISL_2920727	-	B.1.1.7	GRY	39	C241T,C913T,C2110T,C2847T,C3037T,C3267T,C5388A,C5986T,T6954C,G7042T,A10195G,C14120T,C14408T,C14676T,C15279T,C15933T,T16176C,C22450T,A23063T,C23271A,A23403G,C23604A,C23709T,T24506G,G24914C,A25336C,G25337C,A27745G,C27972T,G28048T,G28075T,A28111G,G28280C,A28281T,T28282A,G28881A,G28882A,G28883C,C28977T	25	N:D3L,N:R203K,N:G204R,N:S235F,ORF1a:T861I,ORF1a:T1001I,ORF1a:A1708D,ORF1a:I2230T,ORF1a:M2259I,ORF1b:P218L,ORF1b:P314L,ORF7a:R118G,ORF8:Q27*,ORF8:R52I,ORF8:C61F,ORF8:Y73C,S:N501Y,S:A570D,S:D614G,S:P681H,S:T716I,S:S982A,S:D1118H,S:E1258D,S:D1259H	6	0
hCoV-19/Mexico/TAM-InDRE_FB18890_S4157/2021	EPI_ISL_2920728	-	B.1.1.7	GRY	39	C241T,C913T,C2110T,C2847T,C3037T,C3267T,C5388A,C5986T,T6954C,G7042T,A10195G,C14120T,C14408T,C14676T,C15279T,C15933T,T16176C,C22450T,A23063T,C23271A,A23403G,C23604A,C23709T,T24506G,G24914C,A25336C,G25337C,A27745G,C27972T,G28048T,G28075T,A28111G,G28280C,A28281T,T28282A,G28881A,G28882A,G28883C,C28977T	25	N:D3L,N:R203K,N:G204R,N:S235F,ORF1a:T861I,ORF1a:T1001I,ORF1a:A1708D,ORF1a:I2230T,ORF1a:M2259I,ORF1b:P218L,ORF1b:P314L,ORF7a:R118G,ORF8:Q27*,ORF8:R52I,ORF8:C61F,ORF8:Y73C,S:N501Y,S:A570D,S:D614G,S:P681H,S:T716I,S:S982A,S:D1118H,S:E1258D,S:D1259H	6	0
hCoV-19/Mexico/TAM-InDRE_FB18891_S4158/2021	EPI_ISL_2920729	-	B.1.1.7	GRY	36	C241T,C913T,C2110T,C3037T,C3267T,C5388A,C5986T,T6954C,G7042T,A10195G,C14120T,C14408T,C14676T,C15279T,C15933T,T16176C,A23063T,C23271A,A23403G,C23604A,C23709T,T24506G,G24914C,A25336C,C26110T,A27745G,C27972T,G28048T,G28075T,A28111G,G28262A,G28487A,G28881A,G28882A,G28883C,C28977T	24	N:V72I,N:R203K,N:G204R,N:S235F,ORF1a:T1001I,ORF1a:A1708D,ORF1a:I2230T,ORF1a:M2259I,ORF1b:P218L,ORF1b:P314L,ORF3a:P240S,ORF7a:R118G,ORF8:Q27*,ORF8:R52I,ORF8:C61F,ORF8:Y73C,S:N501Y,S:A570D,S:D614G,S:P681H,S:T716I,S:S982A,S:D1118H,S:E1258D	6	3
hCoV-19/Mexico/TAM-InDRE_FB18896_S4159/2021	EPI_ISL_2920730	-	B.1.1.7	GRY	38	C241T,C913T,C2110T,C3037T,C3267T,C5388A,C5986T,T6954C,G7042T,A10195G,C14120T,C14408T,C14676T,C15279T,C15933T,T16176C,C22450T,A23063T,C23271A,A23403G,C23604A,C23709T,T24506G,G24914C,A25336C,G25337C,A27745G,C27972T,G28048T,G28075T,A28111G,G28280C,A28281T,T28282A,G28881A,G28882A,G28883C,C28977T	24	N:D3L,N:R203K,N:G204R,N:S235F,ORF1a:T1001I,ORF1a:A1708D,ORF1a:I2230T,ORF1a:M2259I,ORF1b:P218L,ORF1b:P314L,ORF7a:R118G,ORF8:Q27*,ORF8:R52I,ORF8:C61F,ORF8:Y73C,S:N501Y,S:A570D,S:D614G,S:P681H,S:T716I,S:S982A,S:D1118H,S:E1258D,S:D1259H	6	0
hCoV-19/Mexico/TAM-InDRE_FB18898_S4469/2021	EPI_ISL_3033323	-	B.1.1.7	GRY	41	C241T,C913T,C2110T,C3037T,C3267T,C5388A,C5986T,G6839A,T6954C,G7042T,G7723A,A10195G,C14120T,C14408T,C14676T,C15279T,C15933T,T16176C,G22103C,C22450T,A23063T,C23271A,A23403G,C23604A,C23709T,T24506G,G24914C,A27745G,C27972T,G28048T,G28075T,A28095T,A28111G,G28280C,A28281T,T28282A,G28881A,G28882A,G28883C,C28977T,C29523T	26	N:D3L,N:R203K,N:G204R,N:S235F,N:T417I,ORF1a:T1001I,ORF1a:A1708D,ORF1a:A2192T,ORF1a:I2230T,ORF1a:M2259I,ORF1b:P218L,ORF1b:P314L,ORF7a:R118G,ORF8:Q27*,ORF8:R52I,ORF8:C61F,ORF8:K68*,ORF8:Y73C,S:G181R,S:N501Y,S:A570D,S:D614G,S:P681H,S:T716I,S:S982A,S:D1118H	6	0
hCoV-19/Mexico/TAM-InDRE_FB18904_S4470/2021	EPI_ISL_3033324	-	B.1.1.7	GRY	36	C241T,C292T,C913T,C3037T,C3267T,C5388A,C5986T,T6954C,T11149C,C14408T,C14676T,C15279T,T16176C,T16563A,T22016C,T22861C,A23063T,C23170T,C23271A,A23403G,C23604A,C23709T,T24506G,G24914C,C25626T,C27972T,G28048T,A28111G,G28280C,A28281T,T28282A,A28295C,G28881A,G28882A,G28883C,C28977T	21	N:D3L,N:N8H,N:R203K,N:G204R,N:S235F,ORF1a:T1001I,ORF1a:A1708D,ORF1a:I2230T,ORF1b:P314L,ORF8:Q27*,ORF8:R52I,ORF8:Y73C,ORF9b:K4N,S:W152R,S:N501Y,S:A570D,S:D614G,S:P681H,S:T716I,S:S982A,S:D1118H	6	0
hCoV-19/Mexico/TAM-InDRE_FB18905_S4471/2021	EPI_ISL_3033325	-	B.1.1.7	GRY	43	C241T,C913T,C2110T,T2218C,C3037T,G3230A,G3231A,T3232A,C3267T,C5388A,C5986T,T6954C,G7042T,C9118T,A10195G,C14120T,C14408T,C14676T,C15279T,C15933T,T16176C,C19602T,G22104C,A23063T,C23271A,A23403G,C23604A,C23709T,T24506G,G24914C,A27745G,C27972T,G28048T,G28075T,A28095T,A28111G,G28280C,A28281T,T28282A,G28881A,G28882A,G28883C,	25	N:D3L,N:R203K,N:G204R,N:S235F,ORF1a:G989K,ORF1a:T1001I,ORF1a:A1708D,ORF1a:I2230T,ORF1a:M2259I,ORF1b:P218L,ORF1b:P314L,ORF7a:R118G,ORF8:Q27*,ORF8:R52I,ORF8:C61F,ORF8:K68*,ORF8:Y73C,S:G181A,S:N501Y,S:A570D,S:D614G,S:P681H,S:T716I,S:S982A,S:D1118H	6	0
hCoV-19/Mexico/TAM-InDRE_FB18909_S4472/2021	EPI_ISL_3033326	-	B.1.1.7	GRY	40	C241T,C913T,C2110T,C3037T,C3267T,C5388A,C5986T,T6954C,G7042T,A10195G,C14120T,C14408T,C14676T,C15279T,C15933T,T16176C,G22103C,T22334A,C22450T,A23063T,C23271A,A23403G,C23604A,C23709T,T24506G,G24914C,C27584T,A27745G,C27972T,G28048T,G28075T,A28095T,A28111G,G28280C,A28281T,T28282A,G28881A,G28882A,G28883C,C28977T	26	N:D3L,N:R203K,N:G204R,N:S235F,ORF1a:T1001I,ORF1a:A1708D,ORF1a:I2230T,ORF1a:M2259I,ORF1b:P218L,ORF1b:P314L,ORF7a:A64V,ORF7a:R118G,ORF8:Q27*,ORF8:R52I,ORF8:C61F,ORF8:K68*,ORF8:Y73C,S:G181R,S:W258R,S:N501Y,S:A570D,S:D614G,S:P681H,S:T716I,S:S982A,S:D1118H	6	0
hCoV-19/Mexico/TAM-InDRE_FB18910_S4473/2021	EPI_ISL_3033327	-	B.1.1.7	GRY	40	C241T,C913T,C2110T,C3037T,C3267T,C5388A,C5986T,T6954C,G7042T,A10195G,C14120T,C14408T,C14676T,C15279T,C15933T,T16176C,C18687T,T21823C,G22103C,C22450T,A23063T,C23271A,A23403G,C23604A,C23709T,T24506G,G24914C,A27745G,C27972T,G28048T,G28075T,A28095T,A28111G,G28280C,A28281T,T28282A,G28881A,G28882A,G28883C,C28977T	24	N:D3L,N:R203K,N:G204R,N:S235F,ORF1a:T1001I,ORF1a:A1708D,ORF1a:I2230T,ORF1a:M2259I,ORF1b:P218L,ORF1b:P314L,ORF7a:R118G,ORF8:Q27*,ORF8:R52I,ORF8:C61F,ORF8:K68*,ORF8:Y73C,S:G181R,S:N501Y,S:A570D,S:D614G,S:P681H,S:T716I,S:S982A,S:D1118H	6	0
hCoV-19/Mexico/TAM-InDRE_FB18911_S4474/2021	EPI_ISL_3033328	-	B.1.1.7	GRY	41	C241T,C913T,C2110T,C3037T,C3267T,C5388A,C5986T,T6954C,G7042T,A10195G,C10834T,G10870T,C14120T,C14408T,C14676T,C15279T,C15933T,T16176C,C16869T,A23063T,C23271A,A23403G,C23604A,C23709T,T24506G,G24914C,G26951T,A27745G,C27972T,G28048T,G28075T,A28095T,A28111G,G28280C,A28281T,T28282A,G28881A,G28882A,G28883C,C28977T,G29422C	23	N:D3L,N:R203K,N:G204R,N:S235F,ORF1a:T1001I,ORF1a:A1708D,ORF1a:I2230T,ORF1a:M2259I,ORF1b:P218L,ORF1b:P314L,ORF7a:R118G,ORF8:Q27*,ORF8:R52I,ORF8:C61F,ORF8:K68*,ORF8:Y73C,S:N501Y,S:A570D,S:D614G,S:P681H,S:T716I,S:S982A,S:D1118H	6	0
hCoV-19/Mexico/TAM-InDRE_FB18912_S4475/2021	EPI_ISL_3033329	-	B.1.1.7	GRY	41	C241T,C913T,C2110T,C3037T,C3267T,C5388A,C5986T,T6954C,G7042T,A10195G,C10834T,G10870T,C14120T,C14408T,C14676T,C15279T,C15933T,T16176C,C16869T,A23063T,C23271A,A23403G,C23604A,C23709T,T24506G,G24914C,G26951T,A27745G,C27972T,G28048T,G28075T,A28095T,A28111G,G28280C,A28281T,T28282A,G28881A,G28882A,G28883C,C28977T,G29422C	23	N:D3L,N:R203K,N:G204R,N:S235F,ORF1a:T1001I,ORF1a:A1708D,ORF1a:I2230T,ORF1a:M2259I,ORF1b:P218L,ORF1b:P314L,ORF7a:R118G,ORF8:Q27*,ORF8:R52I,ORF8:C61F,ORF8:K68*,ORF8:Y73C,S:N501Y,S:A570D,S:D614G,S:P681H,S:T716I,S:S982A,S:D1118H	6	0
hCoV-19/Mexico/TAM-InDRE_FB18922_S4476/2021	EPI_ISL_3033330	-	B.1.1.7	GRY	39	C241T,C913T,C2110T,C3037T,C3267T,C5388A,C5986T,T6954C,G7042T,A10195G,C14120T,C14408T,C14676T,C15279T,C15933T,T16176C,C21597T,C22224T,A23063T,C23271A,A23403G,C23604A,C23709T,T24506G,G24914C,G25855T,A27745G,C27972T,G28048T,G28075T,A28095T,A28111G,G28280C,A28281T,T28282A,G28881A,G28882A,G28883C,C28977T	26	N:D3L,N:R203K,N:G204R,N:S235F,ORF1a:T1001I,ORF1a:A1708D,ORF1a:I2230T,ORF1a:M2259I,ORF1b:P218L,ORF1b:P314L,ORF3a:D155Y,ORF7a:R118G,ORF8:Q27*,ORF8:R52I,ORF8:C61F,ORF8:K68*,ORF8:Y73C,S:S12F,S:S221L,S:N501Y,S:A570D,S:D614G,S:P681H,S:T716I,S:S982A,S:D1118H	6	0
hCoV-19/Mexico/TAM-InDRE_FB18925_S4477/2021	EPI_ISL_3033331	-	B.1.1.7	GRY	40	C241T,C913T,C2110T,C3037T,C3267T,C5388A,C5986T,T6954C,G7042T,A10195G,C14120T,C14408T,C14676T,C15279T,C15933T,T16176C,C18687T,T21823C,G22103C,C22450T,A23063T,C23271A,A23403G,C23604A,C23709T,T24506G,G24914C,A27745G,C27972T,G28048T,G28075T,A28095T,A28111G,G28280C,A28281T,T28282A,G28881A,G28882A,G28883C,C28977T	24	N:D3L,N:R203K,N:G204R,N:S235F,ORF1a:T1001I,ORF1a:A1708D,ORF1a:I2230T,ORF1a:M2259I,ORF1b:P218L,ORF1b:P314L,ORF7a:R118G,ORF8:Q27*,ORF8:R52I,ORF8:C61F,ORF8:K68*,ORF8:Y73C,S:G181R,S:N501Y,S:A570D,S:D614G,S:P681H,S:T716I,S:S982A,S:D1118H	6	0
hCoV-19/Mexico/TAM-InDRE-94/2020	EPI_ISL_794592	-	B.1.1.7	GRY	33	C241T,C913T,C2110T,C3037T,C3267T,C5388A,C5986T,T6954C,T7984C,C14120T,C14408T,C14676T,C15279T,T16176C,C19390T,A23063T,C23271A,A23403G,C23604A,C23709T,T24506G,G24914C,C27972T,G28048T,A28095T,A28111G,G28280C,A28281T,T28282A,G28881A,G28882A,G28	21	N:D3L,N:R203K,N:G204R,N:S235F,ORF1a:T1001I,ORF1a:A1708D,ORF1a:I2230T,ORF1b:P218L,ORF1b:P314L,ORF1b:P1975S,ORF8:Q27*,ORF8:R52I,ORF8:K68*,ORF8:Y73C,S:N501Y,S:A570D,S:D614G,S:P681H,S:T716I,S:S982A,S:D1118H	6	0
hCoV-19/Mexico/TAM-INMEGEN-06-01-347/2021	EPI_ISL_2616961	-	B.1.1.7	G	35	C241T,C913T,C2110T,C3037T,C3267T,C5388A,C5986T,T6954C,G7042T,C7318T,A10195G,C14120T,C14408T,C14676T,C15279T,C15933T,T16176C,A23063T,C23271A,A23403G,C23604A,C23709T,T24506G,G24914C,A27745G,C27972T,G28048T,G28075T,A28095T,A28111G,G28280C,A28281T,T28282A,G28881A,C28977T	21	N:D3L,N:S235F,ORF1a:T1001I,ORF1a:A1708D,ORF1a:I2230T,ORF1a:M2259I,ORF1b:P218L,ORF1b:P314L,ORF7a:R118G,ORF8:Q27*,ORF8:R52I,ORF8:C61F,ORF8:K68*,ORF8:Y73C,S:N501Y,S:A570D,S:D614G,S:P681H,S:T716I,S:S982A,S:D1118H	6	308

hCoV-19/Mexico/VER-InDRE_FB14026_S1753/2021	EPI_ISL_2101892	-	B.1.1.7	GRY	35		25		6	0
hCoV-19/Mexico/VER-InDRE_FB17431_S3930/2021	EPI_ISL_2858944	-	B.1.1.7	GRY	38		23		6	0
hCoV-19/Mexico/VER-InDRE_FB17434_S3931/2021	EPI_ISL_2858945	-	B.1.1.7	GRY	38		27		6	0
hCoV-19/Mexico/VER-INMEGEN-05-06-207/2021	EPI_ISL_2603696	-	B.1.1.7	GR	38		27		6	724
hCoV-19/Mexico/VER-INMEGEN-05-06-231/2021	EPI_ISL_2603713	-	B.1.1.7	GR	39		24		6	1446
hCoV-19/Mexico/VER-INMEGEN-05-06-246/2021	EPI_ISL_2881841	-	B.1.1.7	GR	36		25		6	2281
hCoV-19/Mexico/VER-INMEGEN-05-06-254/2021	EPI_ISL_2603725	-	B.1.1.7	GRY	40		24		6	0
hCoV-19/Mexico/VER-INMEGEN-05-06-255/2021	EPI_ISL_2603726	-	B.1.1.7	GRY	34		21		6	152
hCoV-19/Mexico/VER-INMEGEN-06-01-368/2021	EPI_ISL_2616973	-	B.1.1.7	GRY	42		26		6	0
hCoV-19/Mexico/VER-INMEGEN-06-02-173/2021	EPI_ISL_2692689	-	B.1.1.7	GRY	40		25		6	34
hCoV-19/Mexico/VER-INMEGEN-06-02-372/2021	EPI_ISL_2692848	-	B.1.1.7	GRY	37		26		6	375
hCoV-19/Mexico/VER-INMEGEN-06-02-373/2021	EPI_ISL_2692849	-	B.1.1.7	GRY	41		26		6	0
hCoV-19/Mexico/VER-INMEGEN-06-02-374/2021	EPI_ISL_2692850	-	B.1.1.7	GR	39		24		6	762
hCoV-19/Mexico/VER-INMEGEN-06-02-375/2021	EPI_ISL_2692851	-	B.1.1.7	GR	40		27		6	857
hCoV-19/Mexico/VER-INMEGEN-06-02-376/2021	EPI_ISL_2692852	-	B.1.1.7	GRY	38		27		6	0
hCoV-19/Mexico/VER-INMEGEN-06-04-13/2021	EPI_ISL_2894163	-	B.1.1.7	GRY	33		19		6	126
hCoV-19/Mexico/VER-INMEGEN-06-04-287/2021	EPI_ISL_2894334	-	B.1.1.7	GRY	40		26		6	190
hCoV-19/Mexico/VER-INMEGEN-06-04-324/2021	EPI_ISL_2894370	-	B.1.1.7	GRY	39		25		6	0
hCoV-19/Mexico/VER-INMEGEN-06-04-6/2021	EPI_ISL_2894156	-	B.1.1.7	GR	37		25		6	411

hCoV-19/Mexico/VER-InDRE_FB14026_S1753/2021	C241T,C913T,C2453T,C3037T,C3267T,C5388A,C5986T,T6954C,C9611T,C14408T,C14676T,C15279T,T16176C,C21855T,G21974C,A23063T,C23271A,A23403G,C23604A,C23709T,T24506G,G24914C,C27643T,C27972T,G28048T,A28111G,G28280C,A28281T,T28282A,A28295G,A28626G,G28881A,G28882A,G28883C,C28977T	N:D3L,N:N8D,N:E118G,N:R203K,N:G204R,N:S235F,ORF1a:L730F,ORF1a:T1001I,ORF1a:A1708D,ORF1a:I2230T,ORF1a:L3116F,ORF1b:P314L,ORF7a:P84S,ORF8:Q27*,ORF8:R52I,ORF8:Y73C,S:S98F,S:D138H,S:N501Y,S:A570D,S:D614G,S:P681H,S:T716I,S:S982A,S:D1118H
hCoV-19/Mexico/VER-InDRE_FB17431_S3930/2021	C241T,C913T,C2110T,G3004T,C3037T,C3267T,C5388A,C5986T,T6954C,G7042T,C10525T,C10789T,C14120T,C14408T,C14676T,C15279T,T16176C,A23063T,C23271A,A23403G,G23593T,C23604A,C23709T,T24506G,G24914C,C26110T,C26735T,C27972T,G28048T,A28111G,G28280C,A28281T,T28282A,A28804G,G28881A,G28882A,G28883C,C28977T	N:D3L,N:R203K,N:G204R,N:S235F,ORF1a:E913D,ORF1a:T1001I,ORF1a:A1708D,ORF1a:I2230T,ORF1a:M2259I,ORF1b:P218L,ORF1b:P314L,ORF3a:P240S,ORF8:Q27*,ORF8:R52I,ORF8:Y73C,S:N501Y,S:A570D,S:D614G,S:Q677H,S:P681H,S:T716I,S:S982A,S:D1118H
hCoV-19/Mexico/VER-InDRE_FB17434_S3931/2021	C241T,C913T,C2453T,C3037T,C3267T,C3695T,C5388A,C5986T,T6954C,C9611T,C14408T,C14676T,C15279T,T16176C,C21008T,C21855T,G21974C,A23063T,C23271A,A23403G,C23604A,C23709T,T24506G,G24914C,A25336C,G26529T,C27643T,C27972T,G28048T,A28111G,G28280C,A28281T,T28282A,A28295G,G28881A,G28882A,G28883C,C28977T	M:D3Y,N:D3L,N:N8D,N:R203K,N:G204R,N:S235F,ORF1a:L730F,ORF1a:T1001I,ORF1a:A1708D,ORF1a:I2230T,ORF1a:L3116F,ORF1b:P314L,ORF1b:T2514I,ORF7a:P84S,ORF8:Q27*,ORF8:R52I,ORF8:Y73C,S:S98F,S:D138H,S:N501Y,S:A570D,S:D614G,S:P681H,S:T716I,S:S982A,S:D1118H,S:E1258D
hCoV-19/Mexico/VER-INMEGEN-05-06-207/2021	C241T,C913T,A2317T,C2453T,C3037T,C3267T,C5388A,C5986T,C6433T,T6954C,C9611T,C11750T,C14408T,C14676T,C15279T,T16176C,G19518T,C21008T,C21855T,G21974C,C23271A,A23403G,C23604A,C23709T,T24506G,G24914C,C27643T,C27972T,G28048T,A28111G,G28280C,A28281T,T28282A,A28295G,G28881A,G28882A,G28883C,C28977T	N:D3L,N:N8D,N:R203K,N:G204R,N:S235F,ORF1a:K684N,ORF1a:L730F,ORF1a:T1001I,ORF1a:A1708D,ORF1a:I2230T,ORF1a:L3116F,ORF1a:L3829F,ORF1b:P314L,ORF1b:L2017F,ORF1b:T2514I,ORF7a:P84S,ORF8:Q27*,ORF8:R52I,ORF8:Y73C,S:S98F,S:D138H,S:A570D,S:D614G,S:P681H,S:T716I,S:S982A,S:D1118H
hCoV-19/Mexico/VER-INMEGEN-05-06-231/2021	C241T,C913T,C2110T,G3004T,C3037T,C3267T,C5388A,C5986T,T6954C,G7042T,T8672C,C10525T,C10789T,C14120T,C14408T,C14676T,C15279T,T16176C,C23271A,A23403G,G23593T,C23604A,C23709T,T24506G,G24914C,C26110T,C26735T,C27972T,G28048T,A28095T,A28111G,G28280C,A28281T,T28282A,A28804G,G28881A,G28882A,G28883C,C28977T	N:D3L,N:R203K,N:G204R,N:S235F,ORF1a:E913D,ORF1a:T1001I,ORF1a:A1708D,ORF1a:I2230T,ORF1a:M2259I,ORF1a:S2803P,ORF1b:P218L,ORF1b:P314L,ORF3a:P240S,ORF8:Q27*,ORF8:R52I,ORF8:K68*,ORF8:Y73C,S:A570D,S:D614G,S:Q677H,S:P681H,S:T716I,S:S982A,S:D1118H
hCoV-19/Mexico/VER-INMEGEN-05-06-246/2021	C241T,C913T,A2317T,C2453T,C3037T,C3267T,C5388A,C5986T,C6433T,T6954C,C9611T,C11750T,C14408T,C14676T,C15279T,T16176C,C21008T,C21855T,G21974C,C23271A,A23403G,C23604A,C23709T,T24506G,G24914C,C27972T,G28048T,A28111G,G28280C,A28281T,T28282A,A28295G,G28881A,G28882A,G28883C,C28977T	N:D3L,N:N8D,N:R203K,N:G204R,N:S235F,ORF1a:K684N,ORF1a:L730F,ORF1a:T1001I,ORF1a:A1708D,ORF1a:I2230T,ORF1a:L3116F,ORF1a:L3829F,ORF1b:P314L,ORF1b:T2514I,ORF8:Q27*,ORF8:R52I,ORF8:Y73C,S:S98F,S:D138H,S:A570D,S:D614G,S:P681H,S:T716I,S:S982A,S:D1118H
hCoV-19/Mexico/VER-INMEGEN-05-06-254/2021	C241T,C913T,C2110T,G3004T,C3037T,C3267T,C5388A,C5986T,T6954C,G7042T,C7609T,C10525T,C10789T,C14120T,C14408T,C14676T,C15279T,T16176C,A23063T,C23271A,A23403G,G23593T,C23604A,C23709T,T24506G,G24914C,C26110T,C26735T,C27972T,G28048T,A28095T,A28111G,G28280C,A28281T,T28282A,A28804G,G28881A,G28882A,G28883C,C28977T	N:D3L,N:R203K,N:G204R,N:S235F,ORF1a:E913D,ORF1a:T1001I,ORF1a:A1708D,ORF1a:I2230T,ORF1a:M2259I,ORF1b:P218L,ORF1b:P314L,ORF3a:P240S,ORF8:Q27*,ORF8:R52I,ORF8:K68*,ORF8:Y73C,S:N501Y,S:A570D,S:D614G,S:Q677H,S:P681H,S:T716I,S:S982A,S:D1118H
hCoV-19/Mexico/VER-INMEGEN-05-06-255/2021	C241T,C913T,C3037T,C3267T,A3492G,C4331T,C5388A,C5986T,T6954C,C14408T,C14676T,C15279T,T16176C,C18555T,C21639A,A23063T,C23271A,A23403G,C23604A,C23709T,C24374T,T24506G,G24914C,C27972T,G28048T,A28111G,G28280C,A28281T,T28282A,G28881A,G28882A,G2	N:D3L,N:R203K,N:G204R,N:S235F,ORF1a:T1001I,ORF1a:N1076S,ORF1a:A1708D,ORF1a:I2230T,ORF1b:P314L,ORF8:Q27*,ORF8:R52I,ORF8:Y73C,S:P26H,S:N501Y,S:A570D,S:D614G,S:P681H,S:T716I,S:L938F,S:S982A,S:D1118H
hCoV-19/Mexico/VER-INMEGEN-06-01-368/2021	C241T,C913T,C2110T,G3004T,C3037T,C3267T,C5388A,C5986T,T6954C,G7042T,C9996T,C10525T,C10789T,C14120T,C14408T,C14676T,C15279T,T16176C,A23063T,C23271A,A23403G,G23593T,C23604A,C23709T,T24506G,G24914C,C25844T,C26110T,C26735T,C27389T,C27972T,G28048T,A28095T,A28111G,G28280C,A28281T,T28282A,A28804G,G28881A,G28882A,G28883C,C289	N:D3L,N:R203K,N:G204R,N:S235F,ORF1a:E913D,ORF1a:T1001I,ORF1a:A1708D,ORF1a:I2230T,ORF1a:M2259I,ORF1a:S3244L,ORF1b:P218L,ORF1b:P314L,ORF3a:T151I,ORF3a:P240S,ORF8:Q27*,ORF8:R52I,ORF8:K68*,ORF8:Y73C,S:N501Y,S:A570D,S:D614G,S:Q677H,S:P681H,S:T716I,S:S982A,S:D1118H
hCoV-19/Mexico/VER-INMEGEN-06-02-173/2021	C241T,C913T,C2110T,G3004T,C3037T,C3267T,C5388A,C5986T,T6954C,G7042T,C9996T,C10525T,C10789T,C14120T,C14408T,C14676T,C15279T,T16176C,A23063T,C23271A,A23403G,G23593T,C23604A,C23709T,T24506G,G24914C,C25844T,C26110T,C26735T,C27972T,G28048T,A28111G,G28280C,A28281T,T28282A,A28804G,G28881A,G28882A,G28883C,C28977T	N:D3L,N:R203K,N:G204R,N:S235F,ORF1a:E913D,ORF1a:T1001I,ORF1a:A1708D,ORF1a:I2230T,ORF1a:M2259I,ORF1a:S3244L,ORF1b:P218L,ORF1b:P314L,ORF3a:T151I,ORF3a:P240S,ORF8:Q27*,ORF8:R52I,ORF8:Y73C,S:N501Y,S:A570D,S:D614G,S:Q677H,S:P681H,S:T716I,S:S982A,S:D1118H
hCoV-19/Mexico/VER-INMEGEN-06-02-372/2021	C241T,C913T,C2453T,C3037T,C3267T,C5388A,C5986T,C6433T,C6573T,T6954C,C9611T,C14408T,C14676T,C15279T,T16176C,C21008T,C21855T,G21974C,A23063T,C23271A,A23403G,C23604A,C23709T,T24506G,G24914C,C27643T,C27972T,G28048T,A28111G,G28280C,A28281T,T28282A,A28295G,G28881A,G28882A,G28883C,C28977T	N:D3L,N:N8D,N:R203K,N:G204R,N:S235F,ORF1a:L730F,ORF1a:T1001I,ORF1a:A1708D,ORF1a:S2103F,ORF1a:I2230T,ORF1a:L3116F,ORF1b:P314L,ORF1b:T2514I,ORF7a:P84S,ORF8:Q27*,ORF8:R52I,ORF8:Y73C,S:S98F,S:D138H,S:N501Y,S:A570D,S:D614G,S:P681H,S:T716I,S:S982A,S:D1118H
hCoV-19/Mexico/VER-INMEGEN-06-02-373/2021	C241T,C913T,C2110T,G3004T,C3037T,C3267T,C5388A,C5986T,T6954C,G7042T,C9996T,C10525T,C10789T,C14120T,C14408T,C14676T,C15279T,T16176C,A23063T,C23271A,A23403G,G23593T,C23604A,C23709T,T24506G,G24914C,C25844T,C26110T,C26735T,C27972T,G28048T,A28095T,A28111G,G28280C,A28281T,T28282A,A28804G,G28881A,G28882A,G28883C,C28977T	N:D3L,N:R203K,N:G204R,N:S235F,ORF1a:E913D,ORF1a:T1001I,ORF1a:A1708D,ORF1a:I2230T,ORF1a:M2259I,ORF1a:S3244L,ORF1b:P218L,ORF1b:P314L,ORF3a:T151I,ORF3a:P240S,ORF8:Q27*,ORF8:R52I,ORF8:K68*,ORF8:Y73C,S:N501Y,S:A570D,S:D614G,S:Q677H,S:P681H,S:T716I,S:S982A,S:D1118H
hCoV-19/Mexico/VER-INMEGEN-06-02-374/2021	C241T,C913T,C2110T,G3004T,C3037T,C3267T,C5388A,C5986T,T6954C,G7042T,C9996T,C10525T,C10789T,C14120T,C14408T,C14676T,C15279T,T16176C,C23271A,A23403G,G23593T,C23604A,C23709T,T24506G,G24914C,C25844T,C26110T,C26735T,C27972T,G28048T,A28111G,G28280C,A28281T,T28282A,A28804G,G28881A,G28882A,G28883C,C28977T	N:D3L,N:R203K,N:G204R,N:S235F,ORF1a:E913D,ORF1a:T1001I,ORF1a:A1708D,ORF1a:I2230T,ORF1a:M2259I,ORF1a:S3244L,ORF1b:P218L,ORF1b:P314L,ORF3a:T151I,ORF3a:P240S,ORF8:Q27*,ORF8:R52I,ORF8:Y73C,S:A570D,S:D614G,S:Q677H,S:P681H,S:T716I,S:S982A,S:D1118H
hCoV-19/Mexico/VER-INMEGEN-06-02-375/2021	C203T,C241T,C913T,G1820A,C3037T,C3267T,G5023T,C5388A,G5924A,C5986T,C6317T,T6954C,G9526T,A12162G,A13712G,C14408T,C14676T,C15279T,C15720T,T16176C,A17615G,A21137G,C23248T,C23271A,A23403G,C23604A,C23709T,T24506G,G24914C,G25135T,C27972T,G28048T,A28111G,G28280C,A28281T,T28282A,G28881A,G28882A,G28883C,C28977T	N:D3L,N:R203K,N:G204R,N:S235F,ORF1a:G519S,ORF1a:T1001I,ORF1a:M1586I,ORF1a:A1708D,ORF1a:V1887I,ORF1a:P2018S,ORF1a:I2230T,ORF1a:M3087I,ORF1a:Q3966R,ORF1b:K82R,ORF1b:P314L,ORF1b:K1383R,ORF1b:K2557R,ORF8:Q27*,ORF8:R52I,ORF8:Y73C,S:A570D,S:D614G,S:P681H,S:T716I,S:S982A,S:D1118H,S:K1191N
hCoV-19/Mexico/VER-INMEGEN-06-02-376/2021	C241T,C913T,A2317T,C2453T,C3037T,C3267T,C5388A,C5986T,C6433T,T6954C,C9611T,C11750T,C14408T,C14676T,C15279T,T16176C,C21008T,C21855T,G21974C,A23063T,C23271A,A23403G,C23604A,C23709T,T24506G,G24914C,C27643T,C27972T,G28048T,A28111G,G28280C,A28281T,T28282A,A28295G,G28881A,G28882A,G28883C,C28977T	N:D3L,N:N8D,N:R203K,N:G204R,N:S235F,ORF1a:K684N,ORF1a:L730F,ORF1a:T1001I,ORF1a:A1708D,ORF1a:I2230T,ORF1a:L3116F,ORF1a:L3829F,ORF1b:P314L,ORF1b:T2514I,ORF7a:P84S,ORF8:Q27*,ORF8:R52I,ORF8:Y73C,S:S98F,S:D138H,S:N501Y,S:A570D,S:D614G,S:P681H,S:T716I,S:S982A,S:D1118H
hCoV-19/Mexico/VER-INMEGEN-06-04-13/2021	C241T,C913T,C3037T,C3267T,C5388A,C5986T,C6196T,T6954C,A8848G,C14408T,C14676T,C15279T,T16176C,A23063T,C23114T,C23271A,A23403G,C23604A,C23709T,T24506G,G24914C,G25244C,C27972T,G28048T,A28111G,G28280C,A28281T,T28282A,G28881A,G28882A,G28883C,C	N:D3L,N:R203K,N:G204R,N:S235F,ORF1a:T1001I,ORF1a:A1708D,ORF1a:I2230T,ORF1b:P314L,ORF8:Q27*,ORF8:R52I,ORF8:Y73C,S:N501Y,S:A570D,S:D614G,S:P681H,S:T716I,S:S982A,S:D1118H,S:V1228L
hCoV-19/Mexico/VER-INMEGEN-06-04-287/2021	C203T,C241T,C913T,G1820A,C3037T,C3267T,G5023T,C5388A,G5924A,C5986T,T6954C,G9526T,A12162G,C14408T,C14676T,C15279T,C15720T,T16176C,A17615G,T19363C,A21137G,A23063T,C23248T,C23271A,A23403G,C23604A,C23709T,T24506G,G24914C,G25135T,C27972T,G28048T,A28111G,G28280C,A28281T,T28282A,G28881A,G28882A,G28883C,C28977T	N:D3L,N:R203K,N:G204R,N:S235F,ORF1a:G519S,ORF1a:T1001I,ORF1a:M1586I,ORF1a:A1708D,ORF1a:V1887I,ORF1a:I2230T,ORF1a:M3087I,ORF1a:Q3966R,ORF1b:P314L,ORF1b:K1383R,ORF1b:K2557R,ORF8:Q27*,ORF8:R52I,ORF8:Y73C,S:N501Y,S:A570D,S:D614G,S:P681H,S:T716I,S:S982A,S:D1118H,S:K1191N
hCoV-19/Mexico/VER-INMEGEN-06-04-324/2021	C203T,C241T,C913T,G1820A,C3037T,C3267T,C5388A,G5924A,C5986T,T6954C,G9526T,A12162G,C14408T,C14676T,C15279T,C15720T,T16176C,A17615G,T19363C,A21137G,A23063T,C23248T,C23271A,A23403G,C23604A,C23709T,T24506G,G24914C,G25135T,C27972T,G28048T,A28111G,G28280C,A28281T,T28282A,G28881A,G28882A,G28883C,C28977T	N:D3L,N:R203K,N:G204R,N:S235F,ORF1a:G519S,ORF1a:T1001I,ORF1a:A1708D,ORF1a:V1887I,ORF1a:I2230T,ORF1a:M3087I,ORF1a:Q3966R,ORF1b:P314L,ORF1b:K1383R,ORF1b:K2557R,ORF8:Q27*,ORF8:R52I,ORF8:Y73C,S:N501Y,S:A570D,S:D614G,S:P681H,S:T716I,S:S982A,S:D1118H,S:K1191N
hCoV-19/Mexico/VER-INMEGEN-06-04-6/2021	C241T,C913T,C2453T,C3037T,C3267T,C3695T,C5388A,C5986T,T6954C,C9611T,C14408T,C14676T,C15279T,T16176C,C21008T,C21855T,G21974C,C23271A,A23403G,C23604A,C23709T,T24506G,G24914C,A25048G,G26529T,C27643T,C27972T,G28048T,A28111G,G28280C,A28281T,T28282A,A28295G,G28881A,G28882A,G28883C,C28977T	M:D3Y,N:D3L,N:N8D,N:R203K,N:G204R,N:S235F,ORF1a:L730F,ORF1a:T1001I,ORF1a:A1708D,ORF1a:I2230T,ORF1a:L3116F,ORF1b:P314L,ORF1b:T2514I,ORF7a:P84S,ORF8:Q27*,ORF8:R52I,ORF8:Y73C,S:S98F,S:D138H,S:A570D,S:D614G,S:P681H,S:T716I,S:S982A,S:D1118H

hCoV-19/Mexico/VER-INMEGEN-16-284/2021	EPI_ISL_2978595	-	B.1.1.7	GRY	41	C241T,C913T,C2110T,C2223T,G3004T,C3037T,C3267T,C5388A,C5672T,C5986T,T6954C,G7042T,C10525T,C10789T,C14120T,C14408T,C14676T,C15279T,T16176C,A23063T,C23271A,A23403G,G23593T,C23604A,C23709T,T24506G,G24914C,C26110T,C26735T,C27972T,G28048T,A28095T,A28111G,G28280C,A28281T,T28282A,A28804G,G28881A,G28882A,G28883C,C28977T	26	N:D3L,N:R203K,N:G204R,N:S235F,ORF1a:S653L,ORF1a:E913D,ORF1a:T1001I,ORF1a:A1708D,ORF1a:P1803S,ORF1a:I2230T,ORF1a:M2259I,ORF1b:P218L,ORF1b:P314L,ORF3a:P240S,ORF8:Q27*,ORF8:R52I,ORF8:K68*,ORF8:Y73C,S:N501Y,S:A570D,S:D614G,S:Q677H,S:P681H,S:T716I,S:S982A,S:D1118H	6	87
hCoV-19/Mexico/VER-INMEGEN-16-302/2021	EPI_ISL_2978612	-	B.1.1.7	GR	40	C203T,C241T,C913T,G1820A,C3037T,C3267T,C5388A,G5924A,C5986T,T6954C,G9526T,C12119T,A12162G,C14408T,C14676T,C15279T,C15720T,T16176C,A17615G,T19363C,A21137G,C23248T,C23271A,A23403G,C23604A,C23709T,T24506G,G24914C,G25135T,T27634C,C27972T,G28048T,A28111G,G28280C,A28281T,T28282A,G28881A,G28882A,G28883C,C28977T	26	N:D3L,N:R203K,N:G204R,N:S235F,ORF1a:G519S,ORF1a:T1001I,ORF1a:A1708D,ORF1a:V1887I,ORF1a:I2230T,ORF1a:M3087I,ORF1a:P3952S,ORF1a:Q3966R,ORF1b:P314L,ORF1b:K1383R,ORF1b:K2557R,ORF7a:S81P,ORF8:Q27*,ORF8:R52I,ORF8:Y73C,S:A570D,S:D614G,S:P681H,S:T716I,S:S982A,S:D1118H,S:K11	6	2628
hCoV-19/Mexico/VER-INMEGEN-16-310/2021	EPI_ISL_2978619	-	B.1.1.7	GRY	41	C203T,C241T,C913T,G1820A,C3037T,C3267T,C5388A,G5924A,C5986T,T6954C,G9526T,C10030T,C12119T,A12162G,C14408T,C14676T,C15279T,C15720T,T16176C,A17615G,T19363C,A21137G,A23063T,C23248T,C23271A,A23403G,C23604A,C23709T,T24506G,G24914C,G25135T,C27972T,G28048T,A28111G,G28280C,A28281T,T28282A,G28881A,G28882A,G28883C,C28977T	26	N:D3L,N:R203K,N:G204R,N:S235F,ORF1a:G519S,ORF1a:T1001I,ORF1a:A1708D,ORF1a:V1887I,ORF1a:I2230T,ORF1a:M3087I,ORF1a:P3952S,ORF1a:Q3966R,ORF1b:P314L,ORF1b:K1383R,ORF1b:K2557R,ORF8:Q27*,ORF8:R52I,ORF8:Y73C,S:N501Y,S:A570D,S:D614G,S:P681H,S:T716I,S:S982A,S:D1118H,S:K1191N	6	382
hCoV-19/Mexico/VER-INMEGEN-16-322/2021	EPI_ISL_2978629	-	B.1.1.7	GR	34	C241T,C913T,C3037T,C3267T,G4145A,C5388A,C5986T,T6954C,A7479G,C14408T,C14676T,T15096C,C15279T,T16176C,C17676T,C23271A,A23403G,C23604A,C23709T,A24070T,T24506G,G24914C,C25624T,C27972T,G28048T,A28111G,G28280C,A28281T,T28282A,G28881A,G28882A,G28883C,G28884C,C28977T	22	N:D3L,N:R203K,N:G204P,N:S235F,ORF1a:T1001I,ORF1a:G1294S,ORF1a:A1708D,ORF1a:I2230T,ORF1a:N2405S,ORF1b:P314L,ORF3a:L15V,ORF3a:H78Y,ORF8:Q27*,ORF8:R52I,ORF8:Y73C,S:A570D,S:D614G,S:P681H,S:T716I,S:Q836H,S:S982A,S:D1118H	10	1933
hCoV-19/Mexico/VER-INMEGEN-16-338/2021	EPI_ISL_2978643	-	B.1.1.7	GRY	36	C241T,C913T,C3037T,C3267T,G4145A,C5388A,C5986T,T6954C,A7479G,C14408T,C14676T,T15096C,C15279T,T16176C,C17676T,A23063T,C23271A,A23403G,C23604A,C23709T,A24070T,T24506G,G24914C,C25624T,C27972T,G28048T,A28095T,A28111G,G28280C,A28281T,T28282A,G28881A,G28882A,G28883C,G28884C,C28977T	24	N:D3L,N:R203K,N:G204P,N:S235F,ORF1a:T1001I,ORF1a:G1294S,ORF1a:A1708D,ORF1a:I2230T,ORF1a:N2405S,ORF1b:P314L,ORF3a:L15V,ORF3a:H78Y,ORF8:Q27*,ORF8:R52I,ORF8:K68*,ORF8:Y73C,S:N501Y,S:A570D,S:D614G,S:P681H,S:T716I,S:Q836H,S:S982A,S:D1118H	10	170
hCoV-19/Mexico/YUC-Biomedicos-6140701/2021	EPI_ISL_2918940	-	B.1.1.7	GRY	39	C241T,C913T,C2110T,C3037T,C3267T,C5388A,C5986T,T6954C,G7042T,A10195G,C10279T,C14120T,C14408T,C14676T,C15279T,T16176C,C16887T,G17427T,A23063T,C23271A,A23403G,C23604A,C23709T,T24506G,G24914C,A27943G,C27972T,G28048T,G28075T,A28095T,A28111G,G28280C,A28281T,T28282A,G28881A,G28882A,G28883C,C28977T,G29477T	24	N:D3L,N:R203K,N:G204R,N:S235F,N:D402Y,ORF1a:T1001I,ORF1a:A1708D,ORF1a:I2230T,ORF1a:M2259I,ORF1b:P218L,ORF1b:P314L,ORF8:H17R,ORF8:Q27*,ORF8:R52I,ORF8:C61F,ORF8:K68*,ORF8:Y73C,S:N501Y,S:A570D,S:D614G,S:P681H,S:T716I,S:S982A,S:D1118H	6	0
hCoV-19/Mexico/YUC-Biomedicos-6150004/2021	EPI_ISL_2928339	-	B.1.1.7	GRY	38	C241T,C913T,C2453T,C3037T,C3267T,C5388A,C5986T,T6954C,C9611T,C9893T,C13860T,C14408T,C14676T,C15279T,T16176C,T18429C,C21855T,G21974C,A23063T,C23271A,A23403G,C23604A,C23709T,T24506G,G24914C,C26822T,C27972T,G28048T,A28111G,G28280C,A28281T,T28282A,A28295G,G28881A,G28882A,G28883C,C28977T,C29200T	24	N:D3L,N:N8D,N:R203K,N:G204R,N:S235F,ORF1a:L730F,ORF1a:T1001I,ORF1a:A1708D,ORF1a:I2230T,ORF1a:L3116F,ORF1a:L3210F,ORF1b:P314L,ORF8:Q27*,ORF8:R52I,ORF8:Y73C,S:S98F,S:D138H,S:N501Y,S:A570D,S:D614G,S:P681H,S:T716I,S:S982A,S:D1118H	6	0
hCoV-19/Mexico/YUC-Biomedicos-6220333/2021	EPI_ISL_2928304	-	B.1.1.7	GRY	42	C241T,C913T,C2110T,C2508T,C3037T,C3267T,C4776T,C5388A,C5986T,T6954C,G7042T,C8964T,A10195G,C10279T,C14120T,C14408T,C14676T,C15279T,T16176C,C16887T,G17427T,A23063T,C23271A,A23403G,C23604A,C23709T,T24506G,G24914C,A25859G,A27943G,C27972T,G28048T,G28075T,A28095T,A28111G,G28280C,A28281T,T28282A,G28881A,G28882A,G28883C,C2897	27	N:D3L,N:R203K,N:G204R,N:S235F,ORF1a:P748L,ORF1a:T1001I,ORF1a:T1504I,ORF1a:A1708D,ORF1a:I2230T,ORF1a:M2259I,ORF1a:S2900L,ORF1b:P218L,ORF1b:P314L,ORF3a:Y156C,ORF8:H17R,ORF8:Q27*,ORF8:R52I,ORF8:C61F,ORF8:K68*,ORF8:Y73C,S:N501Y,S:A570D,S:D614G,S:P681H,S:T716I,S:S982A,S:D11	6	0
hCoV-19/Mexico/YUC-InDRE_F11421_S904/2021	EPI_ISL_1423514	-	B.1.1.7	GRY	39	C241T,C913T,C3037T,C3267T,C5388A,C5986T,T6954C,C9693T,C10138T,C11572T,C14408T,C14676T,C15279T,T16176C,G18651A,T23042C,A23063T,C23271A,A23403G,C23604A,C23709T,T24506G,G24914C,C25006T,G25218T,C25553T,G26107T,C27615T,C27972T,G28048T,A28111G,G28280C,A28281T,T28282A,G28881A,G28882A,G28883C,C28977T,C29739T	23	N:D3L,N:R203K,N:G204R,N:S235F,ORF1a:T1001I,ORF1a:A1708D,ORF1a:I2230T,ORF1a:A3143V,ORF1b:P314L,ORF3a:A54V,ORF3a:E239*,ORF8:Q27*,ORF8:R52I,ORF8:Y73C,S:S494P,S:N501Y,S:A570D,S:D614G,S:P681H,S:T716I,S:S982A,S:D1118H,S:G1219V	6	139
hCoV-19/Mexico/YUC-InDRE_F12194_S1195/2021	EPI_ISL_1626802	-	B.1.1.7	GRY	37	C241T,C913T,C2453T,C3037T,C3267T,C5388A,C5986T,T6954C,C9611T,C14408T,C14676T,C15279T,C15925T,T16176C,C19185T,C21855T,G21974C,A23063T,C23271A,A23403G,C23604A,C23709T,T24506G,G24914C,C27972T,G28048T,A28111G,G28280C,A28281T,T28282A,A28295G,C28720T,G28881A,G28882A,G28883C,C28977T,G29527T	25	N:D3L,N:N8D,N:R203K,N:G204R,N:S235F,N:Q418H,ORF1a:L730F,ORF1a:T1001I,ORF1a:A1708D,ORF1a:I2230T,ORF1a:L3116F,ORF1b:P314L,ORF1b:L820F,ORF8:Q27*,ORF8:R52I,ORF8:Y73C,S:S98F,S:D138H,S:N501Y,S:A570D,S:D614G,S:P681H,S:T716I,S:S982A,S:D1118H	6	42
hCoV-19/Mexico/YUC-InDRE_FB15125_S2056/2021	EPI_ISL_2283698	-	B.1.1.7	GRY	35	C241T,C913T,C1223T,C2453T,C3037T,C3267T,C5388A,C5986T,T6954C,C9611T,C14408T,C14676T,C15279T,T16176C,C21855T,G21974C,A23063T,C23271A,A23403G,C23604A,C23709T,T24506G,G24914C,C27972T,G28048T,A28111G,G28280C,A28281T,T28282A,A28295G,G28460T,G28881A,G28882A,G28883C,C28977T	26	N:D3L,N:N8D,N:D63Y,N:R203K,N:G204R,N:S235F,ORF1a:L320F,ORF1a:L730F,ORF1a:T1001I,ORF1a:A1708D,ORF1a:I2230T,ORF1a:L3116F,ORF1b:P314L,ORF8:Q27*,ORF8:R52I,ORF8:Y73C,ORF9b:K59N,S:S98F,S:D138H,S:N501Y,S:A570D,S:D614G,S:P681H,S:T716I,S:S982A,S:D1118H	6	67
hCoV-19/Mexico/YUC-InDRE_FB15167_S2057/2021	EPI_ISL_2283699	-	B.1.1.7	GRY	30	C241T,C913T,C3037T,C3267T,C5388A,C5986T,T6954C,C14408T,C14676T,C15279T,T16176C,A23063T,C23271A,A23403G,C23604A,C23709T,T24506G,G24914C,G25690T,C27972T,G28048T,A28111G,G28280C,A28281T,T28282A,G28396T,G28881A,G28882A,G28883C,C28977T	20	N:D3L,N:R203K,N:G204R,N:S235F,ORF1a:T1001I,ORF1a:A1708D,ORF1a:I2230T,ORF1b:P314L,ORF3a:G100C,ORF8:Q27*,ORF8:R52I,ORF8:Y73C,ORF9b:G38V,S:N501Y,S:A570D,S:D614G,S:P681H,S:T716I,S:S982A,S:D1118H	6	0
hCoV-19/Mexico/YUC-InDRE_FB15328_S2079/2021	EPI_ISL_2283719	-	B.1.1.7	GRY	39	C241T,C913T,C2110T,G3004T,C3037T,C3267T,C5388A,C5986T,T6954C,G7042T,C10789T,C14120T,C14408T,C14676T,G14857T,C15279T,T16176C,G22567T,A23063T,C23271A,A23403G,C23604A,C23709T,T24506G,G24914C,A25336C,C26110T,C26735T,C27972T,G28048T,A28111G,G28280C,A28281T,T28282A,A28804G,G28881A,G28882A,G28883C,C28977T	25	N:D3L,N:R203K,N:G204R,N:S235F,ORF1a:E913D,ORF1a:T1001I,ORF1a:A1708D,ORF1a:I2230T,ORF1a:M2259I,ORF1b:P218L,ORF1b:P314L,ORF1b:V464F,ORF3a:P240S,ORF8:Q27*,ORF8:R52I,ORF8:Y73C,S:L335F,S:N501Y,S:A570D,S:D614G,S:P681H,S:T716I,S:S982A,S:D1118H,S:E1258D	6	0
hCoV-19/Mexico/YUC-InDRE_FB16549_S3111/2021	EPI_ISL_2663378	-	B.1.1.7	GRY	40	C241T,C913T,C2453T,C3037T,C3267T,C3784T,C5388A,C5986T,T6954C,C8334T,C9611T,C14408T,C14676T,C15279T,T16176C,G18984T,C21855T,C21952T,G21974C,A23063T,C23271A,T23323A,A23403G,C23604A,C23709T,T24506G,G24914C,C27972T,G28048T,A28111G,G28280C,A28281T,T28282A,A28295G,G28881A,G28882A,G28883C,C28977T,C29253T,G29405C	27	N:D3L,N:N8D,N:R203K,N:G204R,N:S235F,N:S327L,N:E378Q,ORF1a:L730F,ORF1a:T1001I,ORF1a:A1708D,ORF1a:I2230T,ORF1a:A2690V,ORF1a:L3116F,ORF1b:P314L,ORF1b:M1839I,ORF8:Q27*,ORF8:R52I,ORF8:Y73C,S:S98F,S:D138H,S:N501Y,S:A570D,S:D614G,S:P681H,S:T716I,S:S982A,S:D1118H	6	0
hCoV-19/Mexico/YUC-InDRE_FB16551_S3112/2021	EPI_ISL_2663379	-	B.1.1.7	GRY	33	C241T,C913T,C3037T,C3267T,C5388A,C5986T,C6196T,T6954C,A8848G,T12730A,C14408T,C14676T,C15279T,T16176C,A23063T,C23114T,C23271A,A23403G,C23604A,C23709T,T24506G,G24914C,C27972T,G28048T,A28111G,G28280C,A28281T,T28282A,G28881A,G28882A,G28883C,C2	18	N:D3L,N:R203K,N:G204R,N:S235F,ORF1a:T1001I,ORF1a:A1708D,ORF1a:I2230T,ORF1b:P314L,ORF8:Q27*,ORF8:R52I,ORF8:Y73C,S:N501Y,S:A570D,S:D614G,S:P681H,S:T716I,S:S982A,S:D1118H	6	0
hCoV-19/Mexico/YUC-InDRE_FB16573_S2744/2021	EPI_ISL_2533770	-	B.1.1.7	GRY	31	C241T,C913T,C3037T,C3267T,C3736T,C5388A,C5986T,T6954C,C14408T,C14676T,C15279T,T16176C,A17615G,C21621T,A23063T,C23271A,A23403G,C23604A,C23709T,T24506G,G24914C,C27972T,G28048T,A28111G,G28280C,A28281T,T28282A,G28881A,G28882A,G28883C,C28977T	20	N:D3L,N:R203K,N:G204R,N:S235F,ORF1a:T1001I,ORF1a:A1708D,ORF1a:I2230T,ORF1b:P314L,ORF1b:K1383R,ORF8:Q27*,ORF8:R52I,ORF8:Y73C,S:T20I,S:N501Y,S:A570D,S:D614G,S:P681H,S:T716I,S:S982A,S:D1118H	6	0
hCoV-19/Mexico/YUC-InDRE_FB16575_S2745/2021	EPI_ISL_2533771	-	B.1.1.7	GRY	41	C241T,C913T,G2281A,C3037T,C3267T,C5388A,C5986T,T6954C,A12162G,C13665T,C14408T,C14676T,C15279T,C15720T,T16176C,A17615G,C18747T,C20844T,A21137G,A23063T,C23271A,A23403G,C23604A,C23709T,T24506G,G24914C,G25135T,C26110T,C26521T,G26828T,C27972T,G28048T,A28111G,G28280C,A28281T,T28282A,G28881A,G28882A,G28883C,C28977T,C29095T	23	N:D3L,N:R203K,N:G204R,N:S235F,ORF1a:T1001I,ORF1a:A1708D,ORF1a:I2230T,ORF1a:Q3966R,ORF1b:P314L,ORF1b:K1383R,ORF1b:K2557R,ORF3a:P240S,ORF8:Q27*,ORF8:R52I,ORF8:Y73C,S:N501Y,S:A570D,S:D614G,S:P681H,S:T716I,S:S982A,S:D1118H,S:K1191N	6	0
hCoV-19/Mexico/YUC-InDRE_FB16578_S2746/2021	EPI_ISL_2533772	-	B.1.1.7	GRY	38	C241T,C913T,C2453T,C3037T,G3145A,C3267T,T3508G,C5388A,C5986T,T6954C,C9611T,C10263T,C12114T,C14408T,C14676T,C15279T,T16176C,C21855T,G21974C,A23063T,C23271A,A23403G,C23604A,C23709T,T24506G,G24914C,C27972T,G28048T,A28111G,G28280C,A28281T,T28282A,A28295G,G28881A,G28882A,G28883C,C28977T,C29733T	26	N:D3L,N:N8D,N:R203K,N:G204R,N:S235F,ORF1a:L730F,ORF1a:T1001I,ORF1a:N1081K,ORF1a:A1708D,ORF1a:I2230T,ORF1a:L3116F,ORF1a:A3333V,ORF1a:S3950F,ORF1b:P314L,ORF8:Q27*,ORF8:R52I,ORF8:Y73C,S:S98F,S:D138H,S:N501Y,S:A570D,S:D614G,S:P681H,S:T716I,S:S982A,S:D1118H	6	0
hCoV-19/Mexico/YUC-InDRE_FB16582_S2747/2021	EPI_ISL_2533773	-	B.1.1.7	GRY	36	C241T,C913T,C2453T,C3037T,C3267T,C5388A,C5986T,T6954C,C9611T,C11941T,C14408T,C14676T,C15279T,T16176C,C21855T,G21974C,A23063T,C23271A,A23403G,C23604A,C23709T,T24506G,G24914C,C25886T,C27972T,G28048T,G28077T,A28111G,G28280C,A28281T,T28282A,A28295G,G28881A,G28882A,G28883C,C28977T	25	N:D3L,N:N8D,N:R203K,N:G204R,N:S235F,ORF1a:L730F,ORF1a:T1001I,ORF1a:A1708D,ORF1a:I2230T,ORF1a:L3116F,ORF1b:P314L,ORF3a:S165F,ORF8:Q27*,ORF8:R52I,ORF8:V62L,ORF8:Y73C,S:S98F,S:D138H,S:N501Y,S:A570D,S:D614G,S:P681H,S:T716I,S:S982A,S:D1118H	6	0
hCoV-19/Mexico/YUC-InDRE_FB16584_S2748/2021	EPI_ISL_2533774	-	B.1.1.7	GRY	40	C241T,C913T,C2469T,C3037T,C3267T,T4966C,C5388A,C5986T,T6954C,C7299T,C7945T,C12099T,C14184T,C14408T,C14676T,T15096C,C15279T,T16176C,A23063T,C23271A,A23403G,C23604A,C23709T,G24140C,T24506G,G24914C,C27972T,G28048T,A28095T,A28111G,G28280C,A28281T,T28282A,A28877T,G28878C,G28881A,G28882A,G28883C,C28977T,C29722T	23	N:D3L,N:R203K,N:G204R,N:S235F,ORF1a:A735V,ORF1a:T1001I,ORF1a:A1708D,ORF1a:I2230T,ORF1a:A2345V,ORF1a:A3945V,ORF1b:P314L,ORF8:Q27*,ORF8:R52I,ORF8:K68*,ORF8:Y73C,S:N501Y,S:A570D,S:D614G,S:P681H,S:T716I,S:V860L,S:S982A,S:D1118H	6	0

hCoV-19/Mexico/YUC-InDRE_FB16588_S2749/2021	EPI_ISL_2533775	-	B.1.1.7	GRY	37	C241T,C913T,C2453T,C3037T,C3267T,C5388A,C5986T,T6954C,C9611T,C9893T,C14408T,C14676T,C15279T,T16176C,T18429C,C21855T,G21974C,A23063T,C23271A,A23403G,C23604A,C23709T,C23929T,T24506G,G24914C,C27972T,G28048T,A28111G,G28280C,A28281T,T28282A,A28295G,G28881A,G28882A,G28883C,C28977T,G29474T	25	N:D3L,N:N8D,N:R203K,N:G204R,N:S235F,N:D401Y,ORF1a:L730F,ORF1a:T1001I,ORF1a:A1708D,ORF1a:I2230T,ORF1a:L3116F,ORF1a:L3210F,ORF1b:P314L,ORF8:Q27*,ORF8:R52I,ORF8:Y73C,S:S98F,S:D138H,S:N501Y,S:A570D,S:D614G,S:P681H,S:T716I,S:S982A,S:D1118H	6	0
hCoV-19/Mexico/YUC-InDRE_FB16589_S2750/2021	EPI_ISL_2533776	-	B.1.1.7	GRY	41	C241T,C913T,C2110T,G3004T,C3037T,C3267T,C5388A,G5704T,C5986T,T6954C,G7042T,C10789T,C14120T,C14408T,C14676T,G14857T,C15279T,T16176C,G21538T,G22567T,A23063T,C23271A,A23403G,C23604A,C23709T,T24506G,G24914C,C26110T,C26735T,C27972T,G28048T,A28095T,A28111G,G28280C,A28281T,T28282A,A28804G,G28881A,G28882A,G28883C,C28977T	27	N:D3L,N:R203K,N:G204R,N:S235F,ORF1a:E913D,ORF1a:T1001I,ORF1a:A1708D,ORF1a:Q1813H,ORF1a:I2230T,ORF1a:M2259I,ORF1b:P218L,ORF1b:P314L,ORF1b:V464F,ORF1b:V2691F,ORF3a:P240S,ORF8:Q27*,ORF8:R52I,ORF8:K68*,ORF8:Y73C,S:L335F,S:N501Y,S:A570D,S:D614G,S:P681H,S:T716I,S:S982A,S:D1	6	0
hCoV-19/Mexico/YUC-InDRE_FB16598_S2751/2021	EPI_ISL_2533777	-	B.1.1.7	GRY	41	C241T,C913T,C2110T,G3004T,C3037T,C3267T,C5388A,G5704T,C5986T,T6954C,G7042T,C10789T,C14120T,C14408T,C14676T,G14857T,C15279T,T16176C,G21538T,G22567T,A23063T,C23271A,A23403G,C23604A,C23709T,T24506G,G24914C,C26110T,C26735T,C27972T,G28048T,A28095T,A28111G,G28280C,A28281T,T28282A,A28804G,G28881A,G28882A,G28883C,C28977T	27	N:D3L,N:R203K,N:G204R,N:S235F,ORF1a:E913D,ORF1a:T1001I,ORF1a:A1708D,ORF1a:Q1813H,ORF1a:I2230T,ORF1a:M2259I,ORF1b:P218L,ORF1b:P314L,ORF1b:V464F,ORF1b:V2691F,ORF3a:P240S,ORF8:Q27*,ORF8:R52I,ORF8:K68*,ORF8:Y73C,S:L335F,S:N501Y,S:A570D,S:D614G,S:P681H,S:T716I,S:S982A,S:D1	6	0
hCoV-19/Mexico/YUC-InDRE_FB16601_S2752/2021	EPI_ISL_2533778	-	B.1.1.7	GRY	42	C241T,C897T,C913T,C2469T,C3037T,C3267T,T4966C,C5388A,C5986T,T6954C,C7299T,C7945T,C12099T,C14184T,C14408T,C14676T,T15096C,C15279T,T16176C,C19269T,A23063T,C23271A,A23403G,C23604A,C23709T,G24140C,T24506G,G24914C,C27972T,G28048T,A28095T,A28111G,G28280C,A28281T,T28282A,A28877T,G28878C,G28881A,G28882A,G28883C,C28977T,C29722	24	N:D3L,N:R203K,N:G204R,N:S235F,ORF1a:A211V,ORF1a:A735V,ORF1a:T1001I,ORF1a:A1708D,ORF1a:I2230T,ORF1a:A2345V,ORF1a:A3945V,ORF1b:P314L,ORF8:Q27*,ORF8:R52I,ORF8:K68*,ORF8:Y73C,S:N501Y,S:A570D,S:D614G,S:P681H,S:T716I,S:V860L,S:S982A,S:D1118H	6	0
hCoV-19/Mexico/YUC-InDRE_FB16607_S2753/2021	EPI_ISL_2533779	-	B.1.1.7	GRY	36	C241T,C254T,C913T,C2453T,C3037T,C3267T,C5388A,C5986T,T6954C,C9611T,C14408T,C14676T,C15279T,T16176C,T18099C,C21855T,G21974C,A23063T,C23271A,A23403G,C23604A,C23709T,T24506G,G24914C,C25609T,C27972T,G28048T,A28111G,G28280C,A28281T,T28282A,A28295G,G28881A,G28882A,G28883C,C28977T	24	N:D3L,N:N8D,N:R203K,N:G204R,N:S235F,ORF1a:L730F,ORF1a:T1001I,ORF1a:A1708D,ORF1a:I2230T,ORF1a:L3116F,ORF1b:P314L,ORF3a:L73F,ORF8:Q27*,ORF8:R52I,ORF8:Y73C,S:S98F,S:D138H,S:N501Y,S:A570D,S:D614G,S:P681H,S:T716I,S:S982A,S:D1118H	6	0
hCoV-19/Mexico/YUC-InDRE_FB16611_S2754/2021	EPI_ISL_2533780	-	B.1.1.7	GRY	41	C241T,C913T,C2453T,C3037T,C3093T,C3267T,T4159C,C5388A,C5986T,T6954C,G8653T,C9611T,C9907T,C14408T,C14676T,C15279T,C16175T,T16176C,C21855T,G21974C,A23063T,C23271A,A23403G,C23604A,C23709T,T24506G,G24914C,C25609T,C26469T,G27758T,C27972T,G28048T,A28111G,G28280C,A28281T,T28282A,A28295G,G28881A,G28882A,G28883C,C28977T	29	N:D3L,N:N8D,N:R203K,N:G204R,N:S235F,ORF1a:L730F,ORF1a:P943L,ORF1a:T1001I,ORF1a:A1708D,ORF1a:I2230T,ORF1a:M2796I,ORF1a:L3116F,ORF1b:P314L,ORF1b:T903I,ORF3a:L73F,ORF7a:*122L,ORF7b:M1I,ORF8:Q27*,ORF8:R52I,ORF8:Y73C,S:S98F,S:D138H,S:N501Y,S:A570D,S:D614G,S:P681H,S:T716I,S:S9	6	0
hCoV-19/Mexico/YUC-InDRE_FB16614_S2755/2021	EPI_ISL_2533781	-	B.1.1.7	GRY	42	C241T,C913T,C2110T,C3037T,C3267T,C5388A,C5986T,C6701T,T6954C,G7042T,C9962T,C11750T,C14120T,C14408T,C14676T,C15279T,T16176C,G16416A,G19656T,C21721T,C23059G,A23063T,C23271A,A23403G,C23604A,C23709T,T24506G,G24914C,G26065T,C27972T,G28048T,C28054T,A28095T,A28111G,G28280C,A28281T,T28282A,C28367T,G28881A,G28882A,G28883C,C28977T	28	N:D3L,N:R32C,N:R203K,N:G204R,N:S235F,ORF1a:T1001I,ORF1a:A1708D,ORF1a:L2146F,ORF1a:I2230T,ORF1a:M2259I,ORF1a:H3233Y,ORF1a:L3829F,ORF1b:P218L,ORF1b:P314L,ORF1b:K2063N,ORF3a:V225F,ORF8:Q27*,ORF8:R52I,ORF8:S54L,ORF8:K68*,ORF8:Y73C,S:N501Y,S:A570D,S:D614G,S:P681H,S:T716I,S:S982A,S:D1118H	6	0
hCoV-19/Mexico/YUC-InDRE_FB16617_S2756/2021	EPI_ISL_2533782	-	B.1.1.7	GRY	35	C241T,C913T,C2453T,C3037T,C3267T,C5388A,C5986T,T6954C,C9611T,C14408T,C14676T,C15279T,T16176C,C21077T,C21855T,G21974C,C22987T,A23063T,C23271A,A23403G,C23604A,C23709T,T24506G,G24914C,C27972T,G28048T,A28111G,G28280C,A28281T,T28282A,A28295G,G28881A,G28882A,G28883C,C28977T	24	N:D3L,N:N8D,N:R203K,N:G204R,N:S235F,ORF1a:L730F,ORF1a:T1001I,ORF1a:A1708D,ORF1a:I2230T,ORF1a:L3116F,ORF1b:P314L,ORF1b:T2537I,ORF8:Q27*,ORF8:R52I,ORF8:Y73C,S:S98F,S:D138H,S:N501Y,S:A570D,S:D614G,S:P681H,S:T716I,S:S982A,S:D1118H	6	0
hCoV-19/Mexico/YUC-InDRE_FB16638_S2757/2021	EPI_ISL_2533783	-	B.1.1.7	GRY	34	C241T,C913T,C2453T,C3037T,C3267T,C5388A,C5934T,C5986T,T6954C,C9611T,C14408T,C14676T,C15279T,T16176C,C21855T,G21974C,A23063T,C23271A,A23403G,C23604A,C23709T,T24506G,G24914C,C27972T,G28048T,A28111G,G28280C,A28281T,T28282A,A28295G,G28881A,G28882A,G28883C,C28977T	24	N:D3L,N:N8D,N:R203K,N:G204R,N:S235F,ORF1a:L730F,ORF1a:T1001I,ORF1a:A1708D,ORF1a:T1890I,ORF1a:I2230T,ORF1a:L3116F,ORF1b:P314L,ORF8:Q27*,ORF8:R52I,ORF8:Y73C,S:S98F,S:D138H,S:N501Y,S:A570D,S:D614G,S:P681H,S:T716I,S:S982A,S:D1118H	6	0
hCoV-19/Mexico/YUC-InDRE_FB16653_S2758/2021	EPI_ISL_2533784	-	B.1.1.7	GRY	35	C241T,C913T,C2453T,C3037T,C3267T,C5388A,C5934T,C5986T,T6954C,C9611T,C14408T,C14676T,C15279T,T16176C,C21855T,G21974C,A23063T,C23271A,A23403G,C23604A,C23709T,T24506G,G24914C,C27972T,G28048T,A28111G,G28280C,A28281T,T28282A,A28295G,G28881A,G28882A,G28883C,C28977T,C29754T	24	N:D3L,N:N8D,N:R203K,N:G204R,N:S235F,ORF1a:L730F,ORF1a:T1001I,ORF1a:A1708D,ORF1a:T1890I,ORF1a:I2230T,ORF1a:L3116F,ORF1b:P314L,ORF8:Q27*,ORF8:R52I,ORF8:Y73C,S:S98F,S:D138H,S:N501Y,S:A570D,S:D614G,S:P681H,S:T716I,S:S982A,S:D1118H	6	0
hCoV-19/Mexico/YUC-InDRE_FB16657_S2759/2021	EPI_ISL_2533785	-	B.1.1.7	GRY	36	C241T,C913T,C2453T,C3037T,C3267T,C5388A,C5986T,T6954C,C9611T,C14408T,C14676T,C15279T,T16176C,G18651T,C21304T,C21855T,G21974C,A23063T,C23271A,A23403G,C23410T,C23604A,C23709T,T24506G,G24914C,C27972T,G28048T,A28111G,G28280C,A28281T,T28282A,A28295G,G28881A,G28882A,G28883C,C28977T	25	N:D3L,N:N8D,N:R203K,N:G204R,N:S235F,ORF1a:L730F,ORF1a:T1001I,ORF1a:A1708D,ORF1a:I2230T,ORF1a:L3116F,ORF1b:P314L,ORF1b:E1728D,ORF1b:R2613C,ORF8:Q27*,ORF8:R52I,ORF8:Y73C,S:S98F,S:D138H,S:N501Y,S:A570D,S:D614G,S:P681H,S:T716I,S:S982A,S:D1118H	6	0
hCoV-19/Mexico/YUC-InDRE_FB16659_S2760/2021	EPI_ISL_2533786	-	B.1.1.7	GRY	38	C241T,C913T,C3037T,C3267T,C4752T,C5388A,C5986T,T6954C,C9693T,C11572T,C14408T,C14676T,C15279T,T16176C,C18060A,G18651A,T23042C,A23063T,C23271A,A23403G,C23604A,C23709T,T24506G,G24914C,G25218T,C25553T,C27615T,C27972T,G28048T,A28095T,A28111G,G28280C,A28281T,T28282A,G28881A,G28882A,G28883C,C28977T	24	N:D3L,N:R203K,N:G204R,N:S235F,ORF1a:T1001I,ORF1a:T1496I,ORF1a:A1708D,ORF1a:I2230T,ORF1a:A3143V,ORF1b:P314L,ORF3a:A54V,ORF8:Q27*,ORF8:R52I,ORF8:K68*,ORF8:Y73C,S:S494P,S:N501Y,S:A570D,S:D614G,S:P681H,S:T716I,S:S982A,S:D1118H,S:G1219V	6	0
hCoV-19/Mexico/YUC-InDRE_FB16661_S2761/2021	EPI_ISL_2533787	-	B.1.1.7	GRY	46	C241T,C913T,C2110T,G3004T,C3037T,C3267T,C5388A,C5986T,T6954C,G7042T,C7765T,C10789T,C14120T,C14408T,C14676T,G14857T,C15279T,G15672C,C15810T,T16176C,G20679T,T22219C,G22567T,A23063T,C23271A,A23403G,C23604A,C23709T,T24506G,G24914C,C25731T,C26110T,C26735T,C27002T,C27972T,G28048T,A28095T,A28111G,G28280C,A28281T,T28282A,A28804G,G28881A,G28882A,G28883C,C28977T	26	N:D3L,N:R203K,N:G204R,N:S235F,ORF1a:E913D,ORF1a:T1001I,ORF1a:A1708D,ORF1a:I2230T,ORF1a:M2259I,ORF1b:P218L,ORF1b:P314L,ORF1b:V464F,ORF1b:E735D,ORF3a:P240S,ORF8:Q27*,ORF8:R52I,ORF8:K68*,ORF8:Y73C,S:L335F,S:N501Y,S:A570D,S:D614G,S:P681H,S:T716I,S:S982A,S:D1118H	6	0
hCoV-19/Mexico/YUC-InDRE_FB16665_S2762/2021	EPI_ISL_2533788	-	B.1.1.7	GRY	38	C222T,C241T,C913T,C2110T,T2785C,C3037T,C3267T,C4206T,C5388A,C5986T,T6954C,C7006T,G7042T,C14120T,C14408T,C14676T,C15279T,T16176C,C17762T,A23063T,C23271A,A23403G,C23604A,C23709T,T24506G,G24914C,T25180C,C27972T,G28048T,A28095T,A28111G,G28280C,A28281T,T28282A,G28881A,G28882A,G28883C,C28977T	23	N:D3L,N:R203K,N:G204R,N:S235F,ORF1a:T1001I,ORF1a:A1314V,ORF1a:A1708D,ORF1a:I2230T,ORF1a:M2259I,ORF1b:P218L,ORF1b:P314L,ORF1b:A1432V,ORF8:Q27*,ORF8:R52I,ORF8:K68*,ORF8:Y73C,S:N501Y,S:A570D,S:D614G,S:P681H,S:T716I,S:S982A,S:D1118H	6	0
hCoV-19/Mexico/YUC-InDRE_FB16666_S2763/2021	EPI_ISL_2533789	-	B.1.1.7	GRY	46	C241T,C913T,C2110T,G3004T,C3037T,C3267T,C5388A,C5986T,T6954C,G7042T,C7765T,C10789T,C14120T,C14408T,C14676T,G14857T,C15279T,G15672C,C15810T,T16176C,G20679T,T22219C,G22567T,A23063T,C23271A,A23403G,C23604A,C23709T,T24506G,G24914C,C25731T,C26110T,C26735T,C27002T,C27972T,G28048T,A28095T,A28111G,G28280C,A28281T,T28282A,A28804G,G28881A,G28882A,G28883C,C28977T	26	N:D3L,N:R203K,N:G204R,N:S235F,ORF1a:E913D,ORF1a:T1001I,ORF1a:A1708D,ORF1a:I2230T,ORF1a:M2259I,ORF1b:P218L,ORF1b:P314L,ORF1b:V464F,ORF1b:E735D,ORF3a:P240S,ORF8:Q27*,ORF8:R52I,ORF8:K68*,ORF8:Y73C,S:L335F,S:N501Y,S:A570D,S:D614G,S:P681H,S:T716I,S:S982A,S:D1118H	6	0
hCoV-19/Mexico/YUC-InDRE_FB16667_S2764/2021	EPI_ISL_2533790	-	B.1.1.7	GRY	40	C241T,C913T,C2469T,C3037T,C3267T,T4966C,C5388A,C5986T,T6954C,C7299T,C7945T,C12099T,C14184T,C14408T,C14676T,T15096C,C15279T,T16176C,A23063T,C23271A,A23403G,C23604A,C23709T,G24140C,T24506G,G24914C,C27972T,G28048T,A28095T,A28111G,G28280C,A28281T,T28282A,A28877T,G28878C,G28881A,G28882A,G28883C,C28977T,C29722T	23	N:D3L,N:R203K,N:G204R,N:S235F,ORF1a:A735V,ORF1a:T1001I,ORF1a:A1708D,ORF1a:I2230T,ORF1a:A2345V,ORF1a:A3945V,ORF1b:P314L,ORF8:Q27*,ORF8:R52I,ORF8:K68*,ORF8:Y73C,S:N501Y,S:A570D,S:D614G,S:P681H,S:T716I,S:V860L,S:S982A,S:D1118H	6	0
hCoV-19/Mexico/YUC-InDRE_FB16672_S2765/2021	EPI_ISL_2533791	-	B.1.1.7	GRY	38	C241T,C913T,C2453T,C3037T,C3267T,C5388A,C5986T,A6575T,T6954C,C9611T,G11146T,C14408T,C14676T,C15279T,T16176C,C20930T,C21855T,G21974C,A23063T,C23271A,A23403G,C23604A,C23709T,T24506G,G24914C,C27972T,G28048T,A28111G,G28280C,A28281T,T28282A,A28295G,G28881A,G28882A,G28883C,C28977T,G29033T,C29642T	27	N:D3L,N:N8D,N:R203K,N:G204R,N:S235F,N:A254S,ORF1a:L730F,ORF1a:T1001I,ORF1a:A1708D,ORF1a:S2104C,ORF1a:I2230T,ORF1a:L3116F,ORF1a:M3627I,ORF1b:P314L,ORF1b:T2488M,ORF8:Q27*,ORF8:R52I,ORF8:Y73C,S:S98F,S:D138H,S:N501Y,S:A570D,S:D614G,S:P681H,S:T716I,S:S982A,S:D1118H	6	0
hCoV-19/Mexico/YUC-InDRE_FB16674_S2766/2021	EPI_ISL_2533792	-	B.1.1.7	GRY	35	C241T,C913T,C2453T,C3037T,C3267T,C5388A,C5986T,A6575T,T6954C,C9611T,C14408T,C14676T,C15279T,T16176C,C20930T,C21855T,G21974C,A23063T,C23271A,A23403G,C23604A,C23709T,T24506G,G24914C,C27972T,G28048T,A28111G,G28280C,A28281T,T28282A,A28295G,G28881A,G28882A,G28883C,C28977T	25	N:D3L,N:N8D,N:R203K,N:G204R,N:S235F,ORF1a:L730F,ORF1a:T1001I,ORF1a:A1708D,ORF1a:S2104C,ORF1a:I2230T,ORF1a:L3116F,ORF1b:P314L,ORF1b:T2488M,ORF8:Q27*,ORF8:R52I,ORF8:Y73C,S:S98F,S:D138H,S:N501Y,S:A570D,S:D614G,S:P681H,S:T716I,S:S982A,S:D1118H	6	0

hCoV-19/Mexico/YUC-InDRE_FB16678_S2767/2021	EPI_ISL_2533793	-	B.1.1.7	GRY	36	C241T,C254T,C913T,C2453T,C3037T,C3267T,C5388A,C5986T,T6954C,C9611T,C14408T,C14676T,C15279T,T16176C,T18099C,C21855T,G21974C,A23063T,C23271A,A23403G,C23604A,C23709T,T24506G,G24914C,C25609T,C27972T,G28048T,A28111G,G28280C,A28281T,T28282A,A28295G,G28881A,G28882A,G28883C,C28977T	24	N:D3L,N:N8D,N:R203K,N:G204R,N:S235F,ORF1a:L730F,ORF1a:T1001I,ORF1a:A1708D,ORF1a:I2230T,ORF1a:L3116F,ORF1b:P314L,ORF3a:L73F,ORF8:Q27*,ORF8:R52I,ORF8:Y73C,S:S98F,S:D138H,S:N501Y,S:A570D,S:D614G,S:P681H,S:T716I,S:S982A,S:D1118H	6	0
hCoV-19/Mexico/YUC-InDRE_FB16682_S2768/2021	EPI_ISL_2533794	-	B.1.1.7	GRY	38	C241T,C823T,C913T,C1758T,C2110T,G3004T,C3037T,C3267T,C5388A,C5986T,T6954C,G7042T,C9559T,C14120T,C14408T,C14676T,C15279T,T16176C,G18651T,A23063T,C23271A,A23403G,C23604A,C23709T,T24506G,G24914C,C26110T,C27972T,G28048T,A28095T,A28111G,G28280C,A28281T,T28282A,G28881A,G28882A,G28883C,C28977T	25	N:D3L,N:R203K,N:G204R,N:S235F,ORF1a:A498V,ORF1a:E913D,ORF1a:T1001I,ORF1a:A1708D,ORF1a:I2230T,ORF1a:M2259I,ORF1b:P218L,ORF1b:P314L,ORF1b:E1728D,ORF3a:P240S,ORF8:Q27*,ORF8:R52I,ORF8:K68*,ORF8:Y73C,S:N501Y,S:A570D,S:D614G,S:P681H,S:T716I,S:S982A,S:D1118H	6	0
hCoV-19/Mexico/YUC-InDRE_FB16683_S2769/2021	EPI_ISL_2533795	-	B.1.1.7	GRY	36	C241T,C254T,C913T,C2453T,C3037T,C3267T,C5388A,C5986T,T6954C,C9611T,C14408T,C14676T,C15279T,T16176C,T18099C,C21855T,G21974C,A23063T,C23271A,A23403G,C23604A,C23709T,T24506G,G24914C,C25609T,C27972T,G28048T,A28111G,G28280C,A28281T,T28282A,A28295G,G28881A,G28882A,G28883C,C28977T	24	N:D3L,N:N8D,N:R203K,N:G204R,N:S235F,ORF1a:L730F,ORF1a:T1001I,ORF1a:A1708D,ORF1a:I2230T,ORF1a:L3116F,ORF1b:P314L,ORF3a:L73F,ORF8:Q27*,ORF8:R52I,ORF8:Y73C,S:S98F,S:D138H,S:N501Y,S:A570D,S:D614G,S:P681H,S:T716I,S:S982A,S:D1118H	6	0
hCoV-19/Mexico/YUC-InDRE_FB16684_S2770/2021	EPI_ISL_2533796	-	B.1.1.7	GRY	36	C241T,C254T,C913T,C2453T,C3037T,C3267T,C5388A,C5986T,T6954C,C9611T,C14408T,C14676T,C15279T,T16176C,T18099C,C21855T,G21974C,A23063T,C23271A,A23403G,C23604A,C23709T,T24506G,G24914C,C25609T,C27972T,G28048T,A28111G,G28280C,A28281T,T28282A,A28295G,G28881A,G28882A,G28883C,C28977T	24	N:D3L,N:N8D,N:R203K,N:G204R,N:S235F,ORF1a:L730F,ORF1a:T1001I,ORF1a:A1708D,ORF1a:I2230T,ORF1a:L3116F,ORF1b:P314L,ORF3a:L73F,ORF8:Q27*,ORF8:R52I,ORF8:Y73C,S:S98F,S:D138H,S:N501Y,S:A570D,S:D614G,S:P681H,S:T716I,S:S982A,S:D1118H	6	0
hCoV-19/Mexico/YUC-InDRE_FB17112_S3135/2021	EPI_ISL_2663402	-	B.1.1.7	GRY	38	C241T,C913T,C2469T,C3037T,C3267T,T4966C,C5388A,C5986T,T6954C,C7299T,C7945T,C12099T,C14184T,C14408T,C14676T,T15096C,C15279T,T16176C,A23063T,C23271A,A23403G,C23604A,C23709T,G24140C,T24506G,G24914C,C27972T,G28048T,A28095T,A28111G,G28280C,A28281T,T28282A,G28881A,G28882A,G28883C,C28977T,C29722T	23	N:D3L,N:R203K,N:G204R,N:S235F,ORF1a:A735V,ORF1a:T1001I,ORF1a:A1708D,ORF1a:I2230T,ORF1a:A2345V,ORF1a:A3945V,ORF1b:P314L,ORF8:Q27*,ORF8:R52I,ORF8:K68*,ORF8:Y73C,S:N501Y,S:A570D,S:D614G,S:P681H,S:T716I,S:V860L,S:S982A,S:D1118H	6	0
hCoV-19/Mexico/YUC-InDRE_FB17453_S3932/2021	EPI_ISL_2858946	-	B.1.1.7	GRY	34	C241T,C913T,C2453T,C3037T,C3267T,C5388A,C5986T,T6954C,C9611T,G12163T,C14408T,C14676T,C15279T,T16176C,C21855T,G21974C,A23063T,C23271A,A23403G,C23604A,C23709T,T24506G,G24914C,C27972T,G28048T,A28111G,G28280C,A28281T,T28282A,A28295G,G28881A,G28882A,G28883C,C28977T	24	N:D3L,N:N8D,N:R203K,N:G204R,N:S235F,ORF1a:L730F,ORF1a:T1001I,ORF1a:A1708D,ORF1a:I2230T,ORF1a:L3116F,ORF1a:Q3966H,ORF1b:P314L,ORF8:Q27*,ORF8:R52I,ORF8:Y73C,S:S98F,S:D138H,S:N501Y,S:A570D,S:D614G,S:P681H,S:T716I,S:S982A,S:D1118H	6	0
hCoV-19/Mexico/YUC-InDRE_FB17480_S3493/2021	EPI_ISL_2779008	-	B.1.1.7	GRY	38	C241T,C913T,C2453T,C3037T,C3267T,C5388A,C5986T,T6954C,C9611T,C14408T,C14676T,C15279T,C16017T,T16176C,C21855T,A23063T,C23271A,A23403G,C23604A,C23709T,T24506G,G24914C,A25336C,G25337C,C25886T,C27972T,G28048T,G28077T,A28111G,G28280C,A28281T,T28282A,A28295G,C28657T,G28881A,G28882A,G28883C,C28977T	26	N:D3L,N:N8D,N:R203K,N:G204R,N:S235F,ORF1a:L730F,ORF1a:T1001I,ORF1a:A1708D,ORF1a:I2230T,ORF1a:L3116F,ORF1b:P314L,ORF3a:S165F,ORF8:Q27*,ORF8:R52I,ORF8:V62L,ORF8:Y73C,S:S98F,S:N501Y,S:A570D,S:D614G,S:P681H,S:T716I,S:S982A,S:D1118H,S:E1258D,S:D1259H	6	0
hCoV-19/Mexico/YUC-InDRE_FB17482_S3494/2021	EPI_ISL_2779009	-	B.1.1.7	GRY	34	C241T,C913T,C2453T,C3037T,C3267T,C5388A,C5986T,T6954C,C9611T,C14408T,C14676T,C15279T,T16176C,C21855T,A23063T,C23271A,A23403G,C23604A,C23709T,T24506G,G24914C,A25336C,G25337C,C27972T,G28048T,A28111G,G28280C,A28281T,T28282A,A28295G,G28881A,G28882A,G28883C,C28977T	24	N:D3L,N:N8D,N:R203K,N:G204R,N:S235F,ORF1a:L730F,ORF1a:T1001I,ORF1a:A1708D,ORF1a:I2230T,ORF1a:L3116F,ORF1b:P314L,ORF8:Q27*,ORF8:R52I,ORF8:Y73C,S:S98F,S:N501Y,S:A570D,S:D614G,S:P681H,S:T716I,S:S982A,S:D1118H,S:E1258D,S:D1259H	6	0
hCoV-19/Mexico/YUC-InDRE_FB17487_S3495/2021	EPI_ISL_2779010	-	B.1.1.7	GRY	48	C241T,C913T,A1163C,C2110T,A2863G,G3004T,C3037T,C3267T,C5388A,C5986T,T6954C,G7042T,C7765T,C10789T,C14120T,C14408T,C14676T,G14857T,C15279T,G15672C,C15810T,T16176C,G20679T,T22219C,G22567T,A23063T,C23271A,A23403G,C23604A,C23709T,T24506G,G24914C,A25336C,G25337C,C25731T,C26110T,C26735T,C27972T,G28048T,A28111G,G28280C,A28281T,T28282A,A28804G,G28881A,G28882A,G28883C,C28977T	28	N:D3L,N:R203K,N:G204R,N:S235F,ORF1a:I300L,ORF1a:E913D,ORF1a:T1001I,ORF1a:A1708D,ORF1a:I2230T,ORF1a:M2259I,ORF1b:P218L,ORF1b:P314L,ORF1b:V464F,ORF1b:E735D,ORF3a:P240S,ORF8:Q27*,ORF8:R52I,ORF8:Y73C,S:L335F,S:N501Y,S:A570D,S:D614G,S:P681H,S:T716I,S:S982A,S:D1118H,S:E1258D,S:D1259H	6	0
hCoV-19/Mexico/YUC-InDRE_FB17495_S3496/2021	EPI_ISL_2779011	-	B.1.1.7	GRY	48	C241T,C913T,C2110T,G3004T,C3037T,C3267T,C5388A,C5654T,C5986T,T6954C,G7042T,C7765T,C10789T,C14120T,C14408T,C14676T,G14857T,C15279T,G15672C,C15810T,T16176C,G20679T,T22219C,G22567T,A23063T,C23271A,A23403G,C23604A,C23709T,T24506G,G24914C,A25336C,G25337C,C25731T,C26110T,C26735T,C27002T,C27972T,G28048T,A28111G,G28280C,A28281T,T28282A,A28804G,G28881A,G28882A,G28883C,C28977T	27	N:D3L,N:R203K,N:G204R,N:S235F,ORF1a:E913D,ORF1a:T1001I,ORF1a:A1708D,ORF1a:I2230T,ORF1a:M2259I,ORF1b:P218L,ORF1b:P314L,ORF1b:V464F,ORF1b:E735D,ORF3a:P240S,ORF8:Q27*,ORF8:R52I,ORF8:Y73C,S:L335F,S:N501Y,S:A570D,S:D614G,S:P681H,S:T716I,S:S982A,S:D1118H,S:E1258D,S:D1259H	6	0
hCoV-19/Mexico/YUC-InDRE_FB17498_S3497/2021	EPI_ISL_2779012	-	B.1.1.7	GRY	41	C241T,G353A,C913T,C2453T,C3037T,C3267T,C5388A,C5986T,T6954C,C9611T,C9893T,C14408T,C14676T,C15279T,T16176C,C21855T,G21974C,A23063T,C23271A,A23403G,C23604A,C23709T,T24506G,G24914C,A25336C,G25440T,C25844T,G27382C,A27383T,T27384C,C27972T,G28048T,A28111G,G28280C,A28281T,T28282A,A28295G,G28881A,G28882A,G28883C,C28977T	29	N:D3L,N:N8D,N:R203K,N:G204R,N:S235F,ORF1a:G30S,ORF1a:L730F,ORF1a:T1001I,ORF1a:A1708D,ORF1a:I2230T,ORF1a:L3116F,ORF1a:L3210F,ORF1b:P314L,ORF3a:K16N,ORF3a:T151I,ORF6:D61L,ORF8:Q27*,ORF8:R52I,ORF8:Y73C,S:S98F,S:D138H,S:N501Y,S:A570D,S:D614G,S:P681H,S:T716I,S:S982A,S:D1118H	6	0
hCoV-19/Mexico/YUC-InDRE_FB17499_S3498/2021	EPI_ISL_2779013	-	B.1.1.7	GRY	39	C241T,C913T,C2110T,C3037T,C3267T,C5388A,C5986T,T6954C,G7042T,A10195G,C10279T,C14120T,C14408T,C14676T,C15279T,T16176C,C16887T,G17427T,A23063T,C23271A,A23403G,C23604A,C23709T,T24506G,G24914C,A25336C,G25337C,A27943G,C27972T,G28048T,G28075T,A28111G,G28280C,A28281T,T28282A,G28881A,G28882A,G28883C,C28977T	24	N:D3L,N:R203K,N:G204R,N:S235F,ORF1a:T1001I,ORF1a:A1708D,ORF1a:I2230T,ORF1a:M2259I,ORF1b:P218L,ORF1b:P314L,ORF8:H17R,ORF8:Q27*,ORF8:R52I,ORF8:C61F,ORF8:Y73C,S:N501Y,S:A570D,S:D614G,S:P681H,S:T716I,S:S982A,S:D1118H,S:E1258D,S:D1259H	6	0
hCoV-19/Mexico/YUC-InDRE_FB17502_S3499/2021	EPI_ISL_2779014	-	B.1.1.7	GRY	41	C241T,C913T,C2469T,C3037T,C3267T,T4966C,C5388A,C5986T,T6954C,C7299T,C7945T,C12099T,C14184T,C14408T,C14676T,T15096C,C15279T,T16176C,A23063T,C23271A,A23403G,C23604A,C23709T,G24140C,T24506G,G24914C,A25336C,G25337C,C27972T,G28048T,A28111G,G28280C,A28281T,T28282A,A28877T,G28878C,G28881A,G28882A,G28883C,C28977T,C29722T	24	N:D3L,N:R203K,N:G204R,N:S235F,ORF1a:A735V,ORF1a:T1001I,ORF1a:A1708D,ORF1a:I2230T,ORF1a:A2345V,ORF1a:A3945V,ORF1b:P314L,ORF8:Q27*,ORF8:R52I,ORF8:Y73C,S:N501Y,S:A570D,S:D614G,S:P681H,S:T716I,S:V860L,S:S982A,S:D1118H,S:E1258D,S:D1259H	6	0
hCoV-19/Mexico/YUC-InDRE_FB17506_S3500/2021	EPI_ISL_2779015	-	B.1.1.7	GRY	39	A107T,C241T,C913T,C2453T,C3037T,C3267T,C5388A,C5986T,T6954C,C9611T,C14408T,C14676T,C15279T,T16176C,C18568T,C21855T,G21974C,A23063T,C23271A,A23403G,C23604A,C23709T,T24506G,G24914C,A25336C,G25337C,C27536T,G27754T,C27972T,G28048T,A28111G,G28280C,A28281T,T28282A,A28295G,G28881A,G28882A,G28883C,C28977T	28	N:D3L,N:N8D,N:R203K,N:G204R,N:S235F,ORF1a:L730F,ORF1a:T1001I,ORF1a:A1708D,ORF1a:I2230T,ORF1a:L3116F,ORF1b:P314L,ORF1b:L1701F,ORF7a:P48L,ORF7a:E121*,ORF8:Q27*,ORF8:R52I,ORF8:Y73C,S:S98F,S:D138H,S:N501Y,S:A570D,S:D614G,S:P681H,S:T716I,S:S982A,S:D1118H,S:E1258D,S:D1259H	6	0
hCoV-19/Mexico/YUC-InDRE_FB17511_S3501/2021	EPI_ISL_2779016	-	B.1.1.7	GRY	49	C241T,C913T,C2110T,G3004T,C3037T,C3267T,G4288T,C5388A,C5986T,T6954C,G7042T,C7765T,C10789T,T11125C,C14120T,C14408T,C14676T,G14857T,C15279T,G15672C,C15810T,T16176C,G20679T,T22219C,G22567T,A23063T,C23271A,A23403G,C23604A,C23709T,T24506G,G24914C,A25336C,G25337C,C25731T,C26110T,C26735T,C27002T,C27972T,G28048T,A28111G,G28280C,A28281T,T28282A,A28804G,G28881A,G28882A,G28883C,C28977T	28	N:D3L,N:R203K,N:G204R,N:S235F,ORF1a:E913D,ORF1a:T1001I,ORF1a:E1341D,ORF1a:A1708D,ORF1a:I2230T,ORF1a:M2259I,ORF1b:P218L,ORF1b:P314L,ORF1b:V464F,ORF1b:E735D,ORF3a:P240S,ORF8:Q27*,ORF8:R52I,ORF8:Y73C,S:L335F,S:N501Y,S:A570D,S:D614G,S:P681H,S:T716I,S:S982A,S:D1118H,S:E1258D,S:D1259H	6	0
hCoV-19/Mexico/YUC-InDRE_FB17518_S3502/2021	EPI_ISL_2779017	-	B.1.1.7	GRY	38	C241T,C913T,C2453T,C3037T,G3083A,C3267T,C5388A,C5986T,A6575T,T6954C,C9611T,C14408T,C14676T,C15279T,T16176C,C20930T,C21707T,C21855T,A23063T,C23271A,A23403G,C23604A,C23709T,T24506G,G24914C,A25336C,G25337C,C27972T,G28048T,A28111G,G28280C,A28281T,T28282A,A28295G,G28881A,G28882A,G28883C,C28977T	28	N:D3L,N:N8D,N:R203K,N:G204R,N:S235F,ORF1a:L730F,ORF1a:E940K,ORF1a:T1001I,ORF1a:A1708D,ORF1a:S2104C,ORF1a:I2230T,ORF1a:L3116F,ORF1b:P314L,ORF1b:T2488M,ORF8:Q27*,ORF8:R52I,ORF8:Y73C,S:H49Y,S:S98F,S:N501Y,S:A570D,S:D614G,S:P681H,S:T716I,S:S982A,S:D1118H,S:E1258D,S:D1259H	6	0
hCoV-19/Mexico/YUC-InDRE_FB17531_S3503/2021	EPI_ISL_2779018	-	B.1.1.7	GRY	40	C241T,C913T,C2110T,C3037T,C3267T,C5388A,C5986T,T6954C,G7042T,A10195G,C10279T,C14120T,C14408T,C14676T,C15279T,T16176C,C16887T,G17427T,G21604T,A23063T,C23271A,A23403G,C23604A,C23709T,T24506G,G24914C,A25336C,C25413T,A27943G,C27972T,G28048T,G28075T,A28111G,G28280C,A28281T,T28282A,G28881A,G28882A,G28883C,C28977T	24	N:D3L,N:R203K,N:G204R,N:S235F,ORF1a:T1001I,ORF1a:A1708D,ORF1a:I2230T,ORF1a:M2259I,ORF1b:P218L,ORF1b:P314L,ORF8:H17R,ORF8:Q27*,ORF8:R52I,ORF8:C61F,ORF8:Y73C,S:Q14H,S:N501Y,S:A570D,S:D614G,S:P681H,S:T716I,S:S982A,S:D1118H,S:E1258D	6	0
hCoV-19/Mexico/YUC-InDRE_FB17561_S3505/2021	EPI_ISL_2779020	-	B.1.1.7	GRY	48	C241T,C913T,C2110T,G3004T,C3037T,C3267T,A4108G,C5388A,C5986T,T6954C,G7042T,C7765T,C10789T,C14120T,C14408T,C14676T,G14857T,C15279T,G15672C,C15810T,T16176C,G20679T,T22219C,G22567T,A23063T,C23271A,A23403G,C23604A,C23709T,T24506G,G24914C,A25336C,C25731T,C26110T,C26735T,C27002T,C27972T,G28048T,A28111G,G28280C,A28281T,T28282A,G28727T,A28804G,G28881A,G28882A,G28883C,C28977T	27	N:D3L,N:A152S,N:R203K,N:G204R,N:S235F,ORF1a:E913D,ORF1a:T1001I,ORF1a:A1708D,ORF1a:I2230T,ORF1a:M2259I,ORF1b:P218L,ORF1b:P314L,ORF1b:V464F,ORF1b:E735D,ORF3a:P240S,ORF8:Q27*,ORF8:R52I,ORF8:Y73C,S:L335F,S:N501Y,S:A570D,S:D614G,S:P681H,S:T716I,S:S982A,S:D1118H,S:E1258D	6	0

hCoV-19/Mexico/YUC-InDRE_FB17570_S3506/2021	EPI_ISL_2779021	-	B.1.1.7	GRY	44	C241T,C913T,C2110T,G3004T,C3037T,C3267T,C4055T,C5388A,C5986T,T6954C,G7042T,C7765T,C10789T,C14120T,C14408T,C14676T,G14857T,C15279T,T16176C,G20208T,T22219C,G22567T,A23063T,C23271A,A23403G,C23604A,C23709T,T24506G,G24914C,A25336C,C25731T,C26110T,C26735T,C27972T,G28048T,A28111G,G28280C,A28281T,T28282A,A28804G,G28881A,G28882	27	N:D3L,N:R203K,N:G204R,N:S235F,ORF1a:E913D,ORF1a:T1001I,ORF1a:H1264Y,ORF1a:A1708D,ORF1a:I2230T,ORF1a:M2259I,ORF1b:P218L,ORF1b:P314L,ORF1b:V464F,ORF1b:Q2247H,ORF3a:P240S,ORF8:Q27*,ORF8:R52I,ORF8:Y73C,S:L335F,S:N501Y,S:A570D,S:D614G,S:P681H,S:T716I,S:S982A,S:D1118H,S:E12	6	0
hCoV-19/Mexico/YUC-InDRE_FB17577_S3507/2021	EPI_ISL_2779022	-	B.1.1.7	GRY	53	C241T,C913T,C2110T,G3004T,C3037T,C3267T,C5388A,C5986T,T6954C,G7042T,C7765T,C10789T,G11837T,T12283C,C14120T,C14408T,C14676T,G14857T,C15279T,G15672C,C15810T,T16176C,G20164T,G20679T,C21587T,T22219C,G22567T,A23063T,C23271A,A23403G,C23604A,C23709T,T24506G,G24914C,A25336C,G25337C,C25731T,C26110T,C26735T,C27002T,C27972T,G28048T,A28111G,G28280C,A28281T,T28282A,A28804G,A28877T,G28878C,G28881A,G28882A,G2[illegible]	30	N:D3L,N:R203K,N:G204R,N:S235F,ORF1a:E913D,ORF1a:T1001I,ORF1a:A1708D,ORF1a:I2230T,ORF1a:M2259I,ORF1a:V3858L,ORF1b:P218L,ORF1b:P314L,ORF1b:V464F,ORF1b:E735D,ORF1b:V2233F,ORF3a:P240S,ORF8:Q27*,ORF8:R52I,ORF8:Y73C,S:P9S,S:L335F,S:N501Y,S:A570D,S:D614G,S:P681H,S:T716I,S:S982A,S:D1118H,S:E1258D,S:D1259H	6	0
hCoV-19/Mexico/YUC-InDRE_FB17580_S3508/2021	EPI_ISL_2779023	-	B.1.1.7	GRY	39	C241T,C254T,C913T,C2453T,C3037T,C3267T,C5388A,C5986T,T6954C,C9611T,C14408T,C14676T,C15279T,T16176C,T18099C,C21855T,G21974C,A23063T,C23271A,A23403G,C23604A,C23709T,T24506G,G24914C,A25336C,G25523T,G25526T,C25609T,C27972T,G28048T,A28111G,G28280C,A28281T,T28282A,A28295G,G28881A,G28882A,G28883C,C28977T	27	N:D3L,N:N8D,N:R203K,N:G204R,N:S235F,ORF1a:L730F,ORF1a:T1001I,ORF1a:A1708D,ORF1a:I2230T,ORF1a:L3116F,ORF1b:P314L,ORF3a:G44V,ORF3a:W45L,ORF3a:L73F,ORF8:Q27*,ORF8:R52I,ORF8:Y73C,S:S98F,S:D138H,S:N501Y,S:A570D,S:D614G,S:P681H,S:T716I,S:S982A,S:D1118H,S:E1258D	6	0
hCoV-19/Mexico/YUC-InDRE_FB18961_S4481/2021	EPI_ISL_3033335	-	B.1.1.7	GRY	41	C241T,C823T,C913T,C1758T,C2110T,G3004T,C3037T,C3267T,C5388A,C5986T,T6954C,G7042T,C9559T,C13329T,C14120T,C14408T,C14676T,C15279T,T16176C,A23063T,C23271A,A23403G,C23604A,C23709T,T24506G,G24914C,C26110T,G27382C,A27383T,T27384C,C27972T,G28048T,A28095T,A28111G,G28280C,A28281T,T28282A,G28881A,G28882A,G28883C,C28977T	26	N:D3L,N:R203K,N:G204R,N:S235F,ORF1a:A498V,ORF1a:E913D,ORF1a:T1001I,ORF1a:A1708D,ORF1a:I2230T,ORF1a:M2259I,ORF1a:T4355I,ORF1b:P218L,ORF1b:P314L,ORF3a:P240S,ORF6:D61L,ORF8:Q27*,ORF8:R52I,ORF8:K68*,ORF8:Y73C,S:N501Y,S:A570D,S:D614G,S:P681H,S:T716I,S:S982A,S:D1118H	6	0
hCoV-19/Mexico/YUC-InDRE_FB18962_S4482/2021	EPI_ISL_3033336	-	B.1.1.7	GRY	47	C241T,C913T,C2110T,G3004T,C3037T,C3267T,C5388A,C5986T,T6954C,G7042T,C7765T,C10789T,C14120T,C14408T,C14676T,G14857T,C15279T,G15672C,C15810T,T16176C,C19524T,G20679T,T22219C,G22567T,A23063T,C23271A,A23403G,C23604A,C23709T,T24506G,G24914C,C25731T,C26110T,C26735T,C27002T,C27972T,G28048T,A28095T,A28111G,G28280C,A28281T,T28282A,A28804G,G28881A,G28882A,G28883C,C28977T	26	N:D3L,N:R203K,N:G204R,N:S235F,ORF1a:E913D,ORF1a:T1001I,ORF1a:A1708D,ORF1a:I2230T,ORF1a:M2259I,ORF1b:P218L,ORF1b:P314L,ORF1b:V464F,ORF1b:E735D,ORF3a:P240S,ORF8:Q27*,ORF8:R52I,ORF8:K68*,ORF8:Y73C,S:L335F,S:N501Y,S:A570D,S:D614G,S:P681H,S:T716I,S:S982A,S:D1118H	6	0
hCoV-19/Mexico/YUC-InDRE_FB18966_S4483/2021	EPI_ISL_3033337	-	B.1.1.7	GRY	47	C241T,C913T,C2110T,G3004T,C3037T,C3267T,C5388A,C5986T,T6954C,G7042T,C7765T,C10789T,C14120T,C14408T,C14676T,G14857T,C15279T,G15672C,C15810T,T16176C,C19602T,G20679T,T22219C,G22567T,A23063T,C23271A,A23403G,C23604A,C23709T,T24506G,G24914C,C25731T,C26110T,C26735T,C27002T,C27972T,G28048T,A28095T,A28111G,G28280C,A28281T,T28282A,A28804G,G28881A,G28882A,G28883C,C28977T	26	N:D3L,N:R203K,N:G204R,N:S235F,ORF1a:E913D,ORF1a:T1001I,ORF1a:A1708D,ORF1a:I2230T,ORF1a:M2259I,ORF1b:P218L,ORF1b:P314L,ORF1b:V464F,ORF1b:E735D,ORF3a:P240S,ORF8:Q27*,ORF8:R52I,ORF8:K68*,ORF8:Y73C,S:L335F,S:N501Y,S:A570D,S:D614G,S:P681H,S:T716I,S:S982A,S:D1118H	6	0
hCoV-19/Mexico/YUC-InDRE_FB19001_S4484/2021	EPI_ISL_3033338	-	B.1.1.7	GRY	48	C241T,C913T,C2110T,G3004T,C3037T,C3267T,C5388A,C5548T,C5986T,A6823G,T6954C,G7042T,C7765T,C10789T,C14120T,C14408T,C14676T,G14857T,C15279T,G15672C,C15810T,T16176C,G20679T,T22219C,G22567T,A23063T,C23271A,A23403G,C23604A,C23709T,T24506G,G24914C,C25731T,C26110T,C26735T,C27002T,C27972T,G28048T,A28095T,A28111G,G28280C,A28281T,T28282A,A28804G,G28881A,G28882A,G28883C,C28977T	26	N:D3L,N:R203K,N:G204R,N:S235F,ORF1a:E913D,ORF1a:T1001I,ORF1a:A1708D,ORF1a:I2230T,ORF1a:M2259I,ORF1b:P218L,ORF1b:P314L,ORF1b:V464F,ORF1b:E735D,ORF3a:P240S,ORF8:Q27*,ORF8:R52I,ORF8:K68*,ORF8:Y73C,S:L335F,S:N501Y,S:A570D,S:D614G,S:P681H,S:T716I,S:S982A,S:D1118H	6	0
hCoV-19/Mexico/YUC-InDRE_FB19002_S4485/2021	EPI_ISL_3033339	-	B.1.1.7	GRY	48	C241T,C913T,C2110T,G3004T,C3037T,C3267T,C5388A,C5548T,C5986T,A6823G,T6954C,G7042T,C7765T,C10789T,C14120T,C14408T,C14676T,G14857T,C15279T,G15672C,C15810T,T16176C,G20679T,T22219C,G22567T,A23063T,C23271A,A23403G,C23604A,C23709T,T24506G,G24914C,C25731T,C26110T,C26735T,C27002T,C27972T,G28048T,A28095T,A28111G,G28280C,A28281T,T28282A,A28804G,G28881A,G28882A,G28883C,C28977T	26	N:D3L,N:R203K,N:G204R,N:S235F,ORF1a:E913D,ORF1a:T1001I,ORF1a:A1708D,ORF1a:I2230T,ORF1a:M2259I,ORF1b:P218L,ORF1b:P314L,ORF1b:V464F,ORF1b:E735D,ORF3a:P240S,ORF8:Q27*,ORF8:R52I,ORF8:K68*,ORF8:Y73C,S:L335F,S:N501Y,S:A570D,S:D614G,S:P681H,S:T716I,S:S982A,S:D1118H	6	0
hCoV-19/Mexico/YUC-InDRE_FB19014_S4486/2021	EPI_ISL_3033340	-	B.1.1.7	GRY	39	C241T,C913T,C2110T,C3037T,C3267T,C5388A,C5986T,T6954C,G7042T,C9042T,A10195G,C10279T,C14120T,C14408T,C14676T,C15279T,T16176C,C16887T,G17427T,A23063T,C23271A,A23403G,C23604A,C23709T,T24506G,G24914C,A27943G,C27972T,G28048T,G28075T,A28095T,A28111G,G28280C,A28281T,T28282A,G28881A,G28882A,G28883C,C28977T	24	N:D3L,N:R203K,N:G204R,N:S235F,ORF1a:T1001I,ORF1a:A1708D,ORF1a:I2230T,ORF1a:M2259I,ORF1a:S2926F,ORF1b:P218L,ORF1b:P314L,ORF8:H17R,ORF8:Q27*,ORF8:R52I,ORF8:C61F,ORF8:K68*,ORF8:Y73C,S:N501Y,S:A570D,S:D614G,S:P681H,S:T716I,S:S982A,S:D1118H	6	0
hCoV-19/Mexico/YUC-INMEGEN-05-06-196/2021	EPI_ISL_2603685	-	B.1.1.7	GRY	48	C241T,C913T,C2110T,G3004T,C3037T,C3267T,C3583T,C5388A,C5986T,T6954C,G7042T,C7765T,C10789T,C14120T,C14408T,C14676T,G14857T,C15279T,G15672C,C15810T,T16176C,G20679T,T22219C,G22567T,A23063T,C23271A,A23403G,C23604A,C23709T,T24506G,G24914C,C25731T,C26110T,C26735T,C27002T,A27353C,C27972T,G28048T,A28095T,A28111G,G28280C,A28281T,T28282A,A28804G,G28881A,G28882A,G28883C,C28977T	27	N:D3L,N:R203K,N:G204R,N:S235F,ORF1a:E913D,ORF1a:T1001I,ORF1a:A1708D,ORF1a:I2230T,ORF1a:M2259I,ORF1b:P218L,ORF1b:P314L,ORF1b:V464F,ORF1b:E735D,ORF3a:P240S,ORF6:Q51P,ORF8:Q27*,ORF8:R52I,ORF8:K68*,ORF8:Y73C,S:L335F,S:N501Y,S:A570D,S:D614G,S:P681H,S:T716I,S:S982A,S:D1118H	6	277
hCoV-19/Mexico/YUC-INMEGEN-05-06-222/2021	EPI_ISL_2603706	-	B.1.1.7	GRY	46	C241T,C913T,C2110T,G3004T,C3037T,C3267T,C5388A,C5986T,T6954C,G7042T,C7765T,C10789T,C14120T,C14408T,C14676T,G14857T,C15279T,G15672C,C15810T,T16176C,G20679T,T22219C,G22567T,A23063T,C23271A,A23403G,C23604A,C23709T,T24506G,G24914C,C25731T,C26110T,C26735T,C27002T,C27972T,G28048T,A28095T,A28111G,G28280C,A28281T,T28282A,A28804G,G28881A,G28882A,G28883C,C28977T	26	N:D3L,N:R203K,N:G204R,N:S235F,ORF1a:E913D,ORF1a:T1001I,ORF1a:A1708D,ORF1a:I2230T,ORF1a:M2259I,ORF1b:P218L,ORF1b:P314L,ORF1b:V464F,ORF1b:E735D,ORF3a:P240S,ORF8:Q27*,ORF8:R52I,ORF8:K68*,ORF8:Y73C,S:L335F,S:N501Y,S:A570D,S:D614G,S:P681H,S:T716I,S:S982A,S:D1118H	6	10
hCoV-19/Mexico/YUC-INMEGEN-05-06-269/2021	EPI_ISL_2603739	-	B.1.1.7	GRY	38	C241T,C913T,C2110T,C3037T,C3267T,C5388A,C5986T,T6954C,G7042T,A10195G,C10279T,C14120T,C14408T,C14676T,C15279T,T16176C,C16887T,G17427T,A23063T,C23271A,A23403G,C23604A,C23709T,T24506G,G24914C,A27943G,C27972T,G28048T,G28075T,A28095T,A28111G,G28280C,A28281T,T28282A,G28881A,G28882A,G28883C,C28977T	23	N:D3L,N:R203K,N:G204R,N:S235F,ORF1a:T1001I,ORF1a:A1708D,ORF1a:I2230T,ORF1a:M2259I,ORF1b:P218L,ORF1b:P314L,ORF8:H17R,ORF8:Q27*,ORF8:R52I,ORF8:C61F,ORF8:K68*,ORF8:Y73C,S:N501Y,S:A570D,S:D614G,S:P681H,S:T716I,S:S982A,S:D1118H	6	0
hCoV-19/Mexico/YUC-INMEGEN-05-06-277/2021	EPI_ISL_2603746	-	B.1.1.7	GRY	46	C241T,C913T,C2110T,G3004T,C3037T,C3267T,C5388A,C5986T,T6954C,G7042T,C7765T,C10789T,C14120T,C14408T,C14676T,G14857T,C15279T,G15672C,C15810T,T16176C,G20679T,T22219C,G22567T,A23063T,C23271A,A23403G,C23604A,C23709T,T24506G,G24914C,C25731T,C26110T,C26735T,C27002T,C27972T,G28048T,A28095T,A28111G,G28280C,A28281T,T28282A,A28804G,G28881A,G28882A,G28883C,C28977T	26	N:D3L,N:R203K,N:G204R,N:S235F,ORF1a:E913D,ORF1a:T1001I,ORF1a:A1708D,ORF1a:I2230T,ORF1a:M2259I,ORF1b:P218L,ORF1b:P314L,ORF1b:V464F,ORF1b:E735D,ORF3a:P240S,ORF8:Q27*,ORF8:R52I,ORF8:K68*,ORF8:Y73C,S:L335F,S:N501Y,S:A570D,S:D614G,S:P681H,S:T716I,S:S982A,S:D1118H	6	11
hCoV-19/Mexico/YUC-INMEGEN-06-01-258/2021	EPI_ISL_2616939	-	B.1.1.7	GRY	35	C241T,C913T,C2453T,C3037T,C3267T,C5388A,C5986T,T6954C,C9611T,C14408T,C14676T,C15279T,T16176C,G18651T,C21304T,C21855T,G21974C,A23063T,C23271A,A23403G,C23410T,C23604A,C23709T,C24023T,T24506G,G24914C,C27972T,G28048T,A28111G,T28282A,A28295G,G28881A,G28882A,G28883C,C28977T	24	N:N8D,N:R203K,N:G204R,N:S235F,ORF1a:L730F,ORF1a:T1001I,ORF1a:A1708D,ORF1a:I2230T,ORF1a:L3116F,ORF1b:P314L,ORF1b:E1728D,ORF1b:R2613C,ORF8:Q27*,ORF8:R52I,ORF8:Y73C,S:S98F,S:D138H,S:N501Y,S:A570D,S:D614G,S:P681H,S:T716I,S:S982A,S:D1118H	6	411
hCoV-19/Mexico/YUC-INMEGEN-06-01-271/2021	EPI_ISL_2616910	-	B.1.1.7	GRY	44	C241T,C913T,C2110T,G3004T,C3037T,C3267T,T3613C,C4055T,C5388A,C5986T,T6954C,G7042T,C7765T,C10789T,C14120T,C14408T,C14676T,G14857T,C15279T,T16176C,G20208T,T22219C,G22567T,A23063T,C23271A,A23403G,C23604A,C23709T,T24506G,G24914C,C25731T,C26110T,C26735T,C27972T,G28048T,A28111G,G28280C,A28281T,T28282A,A28804G,G28881A,G28882	26	N:D3L,N:R203K,N:G204R,N:S235F,ORF1a:E913D,ORF1a:T1001I,ORF1a:H1264Y,ORF1a:A1708D,ORF1a:I2230T,ORF1a:M2259I,ORF1b:P218L,ORF1b:P314L,ORF1b:V464F,ORF1b:Q2247H,ORF3a:P240S,ORF8:Q27*,ORF8:R52I,ORF8:Y73C,S:L335F,S:N501Y,S:A570D,S:D614G,S:P681H,S:T716I,S:S982A,S:D1118H	6	585
hCoV-19/Mexico/YUC-INMEGEN-06-01-284/2021	EPI_ISL_2616967	-	B.1.1.7	GRY	37	C241T,C823T,C913T,C1758T,C2110T,G3004T,C3037T,C3267T,C5388A,C5986T,T6954C,G7042T,C9559T,C14120T,C14408T,C14676T,C15279T,T16176C,A23063T,C23271A,A23403G,C23604A,C23709T,T24506G,G24914C,C26110T,C27972T,G28048T,A28095T,A28111G,G28280C,A28281T,T28282A,G28881A,G28882A,G28883C,C28977T	24	N:D3L,N:R203K,N:G204R,N:S235F,ORF1a:A498V,ORF1a:E913D,ORF1a:T1001I,ORF1a:A1708D,ORF1a:I2230T,ORF1a:M2259I,ORF1b:P218L,ORF1b:P314L,ORF3a:P240S,ORF8:Q27*,ORF8:R52I,ORF8:K68*,ORF8:Y73C,S:N501Y,S:A570D,S:D614G,S:P681H,S:T716I,S:S982A,S:D1118H	6	218

hCoV-19/Mexico/YUC-INMEGEN-06-01-299/2021	EPI_ISL_2616968	-	B.1.1.7	GRY	48	C241T,C913T,C2110T,G3004T,C3037T,C3267T,C5388A,C5986T,T6954C,G7042T,C7765T,C10789T,C14120T,C14408T,C14676T,G14857T,C15279T,G15672C,C15810T,T16176C,G20679T,T22219C,G22567T,A23063T,C23271A,A23403G,C23604A,C23709T,T24506G,G24914C,C25731T,C26110T,G26506T,C26735T,C26882T,C27002T,C27972T,G28048T,A28095T,A28111G,G28280C,A28281T,T28282A,A28804G,G28881A,G28882A,G28883C,C28977T	26	N:D3L,N:R203K,N:G204R,N:S235F,ORF1a:E913D,ORF1a:T1001I,ORF1a:A1708D,ORF1a:I2230T,ORF1a:M2259I,ORF1b:P218L,ORF1b:P314L,ORF1b:V464F,ORF1b:E735D,ORF3a:P240S,ORF8:Q27*,ORF8:R52I,ORF8:K68*,ORF8:Y73C,S:L335F,S:N501Y,S:A570D,S:D614G,S:P681H,S:T716I,S:S982A,S:D1118H	6	162
hCoV-19/Mexico/YUC-INMEGEN-06-01-307/2021	EPI_ISL_2616932	-	B.1.1.7	GRY	35	C241T,C913T,C2453T,C3037T,C3267T,C5388A,C5986T,T6954C,C9611T,C9893T,C13860T,C14408T,C14676T,C15279T,T16176C,T18429C,C21855T,G21974C,A23063T,C23271A,A23403G,C23604A,C23709T,T24506G,G24914C,C26822T,C27972T,G28048T,A28111G,T28282A,A28295G,G28881A,G28882A,G28883C,C28977T	23	N:N8D,N:R203K,N:G204R,N:S235F,ORF1a:L730F,ORF1a:T1001I,ORF1a:A1708D,ORF1a:I2230T,ORF1a:L3116F,ORF1a:L3210F,ORF1b:P314L,ORF8:Q27*,ORF8:R52I,ORF8:Y73C,S:S98F,S:D138H,S:N501Y,S:A570D,S:D614G,S:P681H,S:T716I,S:S982A,S:D1118H	6	459
hCoV-19/Mexico/YUC-INMEGEN-06-01-319/2021	EPI_ISL_2616984	-	B.1.1.7	G	42	C241T,C913T,C2110T,G3004T,C3037T,C3267T,C5388A,C5986T,T6954C,G7042T,C7765T,C10789T,C14120T,C14408T,C14676T,G14857T,C15279T,G15672C,C15810T,T16176C,G20679T,T22219C,G22567T,C23271A,A23403G,C23604A,C23709T,T24506G,G24914C,C25731T,C26110T,C26735T,C27002T,C27972T,G28048T,A28095T,A28111G,G28280C,A28281T,T28282A,A28804G,C289	23	N:D3L,N:S235F,ORF1a:E913D,ORF1a:T1001I,ORF1a:A1708D,ORF1a:I2230T,ORF1a:M2259I,ORF1b:P218L,ORF1b:P314L,ORF1b:V464F,ORF1b:E735D,ORF3a:P240S,ORF8:Q27*,ORF8:R52I,ORF8:K68*,ORF8:Y73C,S:L335F,S:A570D,S:D614G,S:P681H,S:T716I,S:S982A,S:D1118H	6	2184
hCoV-19/Mexico/YUC-INMEGEN-06-01-330/2021	EPI_ISL_2616970	-	B.1.1.7	GRY	46	C241T,C913T,C2110T,G3004T,C3037T,C3267T,C5388A,C5986T,T6954C,G7042T,C7765T,C10789T,C14120T,C14408T,C14676T,G14857T,C15279T,G15672C,C15810T,T16176C,G20679T,T22219C,G22567T,A23063T,C23271A,A23403G,C23604A,C23709T,T24506G,G24914C,C25731T,C26110T,C26735T,C27002T,C27972T,G28048T,A28095T,A28111G,G28280C,A28281T,T28282A,A28804G,G28881A,G28882A,G28883C,C28977T	26	N:D3L,N:R203K,N:G204R,N:S235F,ORF1a:E913D,ORF1a:T1001I,ORF1a:A1708D,ORF1a:I2230T,ORF1a:M2259I,ORF1b:P218L,ORF1b:P314L,ORF1b:V464F,ORF1b:E735D,ORF3a:P240S,ORF8:Q27*,ORF8:R52I,ORF8:K68*,ORF8:Y73C,S:L335F,S:N501Y,S:A570D,S:D614G,S:P681H,S:T716I,S:S982A,S:D1118H	6	8
hCoV-19/Mexico/YUC-INMEGEN-06-01-338/2021	EPI_ISL_2616966	-	B.1.1.7	GRY	34	C241T,C913T,C2453T,C3037T,C3267T,C5388A,C5934T,C5986T,T6954C,C9611T,C14408T,C14676T,C15279T,T16176C,G20352A,C20946T,C21855T,G21974C,A23063T,C23271A,A23403G,C23604A,C23709T,T24506G,G24914C,C27972T,G28048T,A28111G,T28282A,A28295G,G28881A,G28882A,G28883C,C28977T	23	N:N8D,N:R203K,N:G204R,N:S235F,ORF1a:L730F,ORF1a:T1001I,ORF1a:A1708D,ORF1a:T1890I,ORF1a:I2230T,ORF1a:L3116F,ORF1b:P314L,ORF8:Q27*,ORF8:R52I,ORF8:Y73C,S:S98F,S:D138H,S:N501Y,S:A570D,S:D614G,S:P681H,S:T716I,S:S982A,S:D1118H	6	3
hCoV-19/Mexico/YUC-INMEGEN-06-02-116/2021	EPI_ISL_2692641	-	B.1.1.7	GR	45	C241T,C913T,C2110T,G3004T,C3037T,C3267T,C5388A,C5986T,T6954C,G7042T,C7765T,C10789T,C14120T,C14408T,C14676T,G14857T,C15279T,G15672C,C15810T,T16176C,G20679T,T22219C,G22567T,C23271A,A23403G,C23604A,C23709T,T24506G,G24914C,C25731T,C26110T,G26506T,C26735T,C27002T,C27972T,G28048T,A28111G,G28280C,A28281T,T28282A,A28804G,G288	24	N:D3L,N:R203K,N:G204R,N:S235F,ORF1a:E913D,ORF1a:T1001I,ORF1a:A1708D,ORF1a:I2230T,ORF1a:M2259I,ORF1b:P218L,ORF1b:P314L,ORF1b:V464F,ORF1b:E735D,ORF3a:P240S,ORF8:Q27*,ORF8:R52I,ORF8:Y73C,S:L335F,S:A570D,S:D614G,S:P681H,S:T716I,S:S982A,S:D1118H	6	1573
hCoV-19/Mexico/YUC-INMEGEN-06-02-136/2021	EPI_ISL_2692657	-	B.1.1.7	GRY	49	C241T,C913T,C2110T,G3004T,C3037T,C3267T,C5388A,C5986T,T6954C,G7042T,C7303T,C7765T,C10789T,C14120T,C14408T,C14676T,G14857T,C15279T,G15672C,C15810T,T16176C,G20679T,T22219C,G22567T,A23063T,C23271A,A23403G,C23604A,C23709T,T24506G,G24914C,C25731T,C26110T,G26506T,C26735T,C26882T,C27002T,C27972T,G28048T,A28095T,A28111G,G28280C,A28281T,T28282A,A28804G,G28881A,G28882A,G28883C,C28977T	26	N:D3L,N:R203K,N:G204R,N:S235F,ORF1a:E913D,ORF1a:T1001I,ORF1a:A1708D,ORF1a:I2230T,ORF1a:M2259I,ORF1b:P218L,ORF1b:P314L,ORF1b:V464F,ORF1b:E735D,ORF3a:P240S,ORF8:Q27*,ORF8:R52I,ORF8:K68*,ORF8:Y73C,S:L335F,S:N501Y,S:A570D,S:D614G,S:P681H,S:T716I,S:S982A,S:D1118H	6	21
hCoV-19/Mexico/YUC-INMEGEN-06-02-141/2021	EPI_ISL_2692662	-	B.1.1.7	GRY	47	C241T,C913T,C2110T,G3004T,C3037T,C3267T,C5388A,C5986T,T6954C,G7042T,C7765T,C10789T,C14120T,C14408T,C14676T,G14857T,C15279T,G15672C,C15810T,T16176C,G20679T,T22219C,G22567T,A23063T,C23271A,A23403G,C23604A,C23709T,T24506G,T24769C,G24914C,C25731T,C26110T,C26735T,C27002T,C27972T,G28048T,A28095T,A28111G,G28280C,A28281T,T28282A,A28804G,G28881A,G28882A,G28883C,C28977T	26	N:D3L,N:R203K,N:G204R,N:S235F,ORF1a:E913D,ORF1a:T1001I,ORF1a:A1708D,ORF1a:I2230T,ORF1a:M2259I,ORF1b:P218L,ORF1b:P314L,ORF1b:V464F,ORF1b:E735D,ORF3a:P240S,ORF8:Q27*,ORF8:R52I,ORF8:K68*,ORF8:Y73C,S:L335F,S:N501Y,S:A570D,S:D614G,S:P681H,S:T716I,S:S982A,S:D1118H	6	0
hCoV-19/Mexico/YUC-INMEGEN-06-02-160/2021	EPI_ISL_2692676	-	B.1.1.7	GRY	46	C241T,C913T,C2110T,G3004T,C3037T,C3267T,C5388A,C5986T,T6954C,G7042T,C7765T,C10789T,C14120T,C14408T,C14676T,G14857T,C15279T,G15672C,C15810T,T16176C,G20679T,T22219C,G22567T,A23063T,C23271A,A23403G,C23604A,C23709T,T24506G,G24914C,C25731T,C26110T,C26735T,C27002T,C27972T,G28048T,A28095T,A28111G,G28280C,A28281T,T28282A,A28804G,G28881A,G28882A,G28883C,C28977T	26	N:D3L,N:R203K,N:G204R,N:S235F,ORF1a:E913D,ORF1a:T1001I,ORF1a:A1708D,ORF1a:I2230T,ORF1a:M2259I,ORF1b:P218L,ORF1b:P314L,ORF1b:V464F,ORF1b:E735D,ORF3a:P240S,ORF8:Q27*,ORF8:R52I,ORF8:K68*,ORF8:Y73C,S:L335F,S:N501Y,S:A570D,S:D614G,S:P681H,S:T716I,S:S982A,S:D1118H	6	152
hCoV-19/Mexico/YUC-INMEGEN-06-02-165/2021	EPI_ISL_2692681	-	B.1.1.7	GRY	46	C241T,C913T,C2110T,G3004T,C3037T,C3267T,C5388A,C5986T,T6954C,G7042T,C7765T,C10789T,C14120T,C14408T,C14676T,G14857T,C15279T,G15672C,C15810T,T16176C,G20679T,T22219C,G22567T,A23063T,C23271A,A23403G,C23604A,C23709T,T24506G,G24914C,C25731T,C26110T,C26735T,C27002T,C27972T,G28048T,A28095T,A28111G,G28280C,A28281T,T28282A,A28804G,G28881A,G28882A,G28883C,C28977T	26	N:D3L,N:R203K,N:G204R,N:S235F,ORF1a:E913D,ORF1a:T1001I,ORF1a:A1708D,ORF1a:I2230T,ORF1a:M2259I,ORF1b:P218L,ORF1b:P314L,ORF1b:V464F,ORF1b:E735D,ORF3a:P240S,ORF8:Q27*,ORF8:R52I,ORF8:K68*,ORF8:Y73C,S:L335F,S:N501Y,S:A570D,S:D614G,S:P681H,S:T716I,S:S982A,S:D1118H	6	18
hCoV-19/Mexico/YUC-INMEGEN-06-02-168/2021	EPI_ISL_2692684	-	B.1.1.7	GRY	46	C241T,C913T,C2110T,G3004T,C3037T,C3267T,C5388A,C5986T,T6954C,G7042T,C7765T,C10789T,C14120T,C14408T,C14676T,G14857T,C15279T,G15672C,C15810T,T16176C,G20679T,T22219C,G22567T,A23063T,C23271A,A23403G,C23604A,C23709T,T24506G,G24914C,C25731T,C26110T,C26735T,C27002T,C27972T,G28048T,A28095T,A28111G,G28280C,A28281T,T28282A,A28804G,G28881A,G28882A,G28883C,C28977T	26	N:D3L,N:R203K,N:G204R,N:S235F,ORF1a:E913D,ORF1a:T1001I,ORF1a:A1708D,ORF1a:I2230T,ORF1a:M2259I,ORF1b:P218L,ORF1b:P314L,ORF1b:V464F,ORF1b:E735D,ORF3a:P240S,ORF8:Q27*,ORF8:R52I,ORF8:K68*,ORF8:Y73C,S:L335F,S:N501Y,S:A570D,S:D614G,S:P681H,S:T716I,S:S982A,S:D1118H	6	0
hCoV-19/Mexico/YUC-INMEGEN-06-02-18/2021	EPI_ISL_2692555	-	B.1.1.7	GRY	48	C203T,C241T,C913T,C2110T,G3004T,C3037T,C3267T,C3992T,C5388A,C5986T,T6954C,G7042T,C7765T,C10789T,C14120T,C14408T,C14676T,G14857T,C15279T,G15672C,C15810T,T16176C,G20679T,T22219C,G22567T,A23063T,C23271A,A23403G,C23604A,C23709T,T24506G,G24914C,C25731T,C26110T,C26735T,C27002T,C27972T,G28048T,A28095T,A28111G,G28280C,A28281T,T28282A,A28804G,G28881A,G28882A,G28883C,C28977T	26	N:D3L,N:R203K,N:G204R,N:S235F,ORF1a:E913D,ORF1a:T1001I,ORF1a:A1708D,ORF1a:I2230T,ORF1a:M2259I,ORF1b:P218L,ORF1b:P314L,ORF1b:V464F,ORF1b:E735D,ORF3a:P240S,ORF8:Q27*,ORF8:R52I,ORF8:K68*,ORF8:Y73C,S:L335F,S:N501Y,S:A570D,S:D614G,S:P681H,S:T716I,S:S982A,S:D1118H	6	19
hCoV-19/Mexico/YUC-INMEGEN-06-02-184/2021	EPI_ISL_2692697	-	B.1.1.7	GRY	49	C241T,C913T,C2110T,G3004T,C3037T,C3267T,C5388A,C5986T,T6954C,G7042T,C7765T,C10789T,C14120T,C14408T,C14676T,G14857T,C15279T,G15672C,C15810T,T16176C,G19300T,G20679T,T22219C,G22567T,A23063T,C23271A,A23403G,C23604A,C23709T,T24506G,G24914C,C25731T,C26110T,G26506T,C26735T,C26882T,C27002T,C27972T,G28048T,A28095T,A28111G,G28280C,A28281T,T28282A,A28804G,G28881A,G28882A,G28883C,C28977T	27	N:D3L,N:R203K,N:G204R,N:S235F,ORF1a:E913D,ORF1a:T1001I,ORF1a:A1708D,ORF1a:I2230T,ORF1a:M2259I,ORF1b:P218L,ORF1b:P314L,ORF1b:V464F,ORF1b:E735D,ORF1b:V1945L,ORF3a:P240S,ORF8:Q27*,ORF8:R52I,ORF8:K68*,ORF8:Y73C,S:L335F,S:N501Y,S:A570D,S:D614G,S:P681H,S:T716I,S:S982A,S:D1118H	6	31
hCoV-19/Mexico/YUC-INMEGEN-06-02-333/2021	EPI_ISL_2692817	-	B.1.1.7	GRY	37	C241T,C913T,C3037T,C3267T,C5388A,C5944T,C5986T,T6954C,C9967T,C13585T,C14408T,C14676T,T15096C,C15279T,T16176C,G21901A,C22987T,A23063T,C23271A,A23403G,C23604A,C23709T,T24506G,G24914C,T27931C,C27972T,G28048T,A28095T,A28111G,G28280C,A28281T,T28282A,G28881A,G28882A,G28883C,G28884C,C28977T	20	N:D3L,N:R203K,N:G204P,N:S235F,ORF1a:T1001I,ORF1a:A1708D,ORF1a:I2230T,ORF1b:P314L,ORF8:V13A,ORF8:Q27*,ORF8:R52I,ORF8:K68*,ORF8:Y73C,S:N501Y,S:A570D,S:D614G,S:P681H,S:T716I,S:S982A,S:D1118H	6	0
hCoV-19/Mexico/YUC-INMEGEN-06-02-334/2021	EPI_ISL_2692818	-	B.1.1.7	G	38	C241T,C913T,C2469T,C3037T,C3157T,C3267T,T4966C,C5388A,C5986T,T6954C,C7299T,C7945T,C12099T,C14184T,C14408T,C14676T,T15096C,C15279T,T16176C,C17934A,A23063T,C23271A,A23403G,C23604A,C23709T,G24140C,T24506G,G24914C,G26779T,C27972T,G28048T,A28111G,G28280C,A28281T,T28282A,A28877T,C28977T,C29722T	21	M:C86F,N:D3L,N:S235F,ORF1a:A735V,ORF1a:T1001I,ORF1a:A1708D,ORF1a:I2230T,ORF1a:A2345V,ORF1a:A3945V,ORF1b:P314L,ORF8:Q27*,ORF8:R52I,ORF8:Y73C,S:N501Y,S:A570D,S:D614G,S:P681H,S:T716I,S:V860L,S:S982A,S:D1118H	6	153
hCoV-19/Mexico/YUC-INMEGEN-06-02-35/2021	EPI_ISL_2692570	-	B.1.1.7	GRY	37	C241T,C254T,C337T,C913T,C2453T,C3037T,C3267T,C5388A,C5986T,T6954C,C9611T,C14408T,C14676T,C15279T,T16176C,T18099C,C21855T,G21974C,A23063T,C23271A,A23403G,C23604A,C23709T,T24506G,G24914C,C25609T,C27972T,G28048T,A28111G,G28280C,A28281T,T28282A,A28295G,G28881A,G28882A,G28883C,C28977T	24	N:D3L,N:N8D,N:R203K,N:G204R,N:S235F,ORF1a:L730F,ORF1a:T1001I,ORF1a:A1708D,ORF1a:I2230T,ORF1a:L3116F,ORF1b:P314L,ORF3a:L73F,ORF8:Q27*,ORF8:R52I,ORF8:Y73C,S:S98F,S:D138H,S:N501Y,S:A570D,S:D614G,S:P681H,S:T716I,S:S982A,S:D1118H	6	0

hCoV-19/Mexico/YUC-INMEGEN-06-02-39/2021	EPI_ISL_2692574	-	B.1.1.7	G	35	C241T,C913T,C2469T,C3037T,C3267T,T4966C,C5388A,C5986T,T6954C,C7299T,C7945T,C12099T,C14184T,C14408T,C14676T,T15096C,C15279T,T16176C,A23063T,C23271A,A23403G,C23604A,C23709T,G24140C,T24506G,G24914C,C27972T,G28048T,A28095T,A28111G,G28280C,A28281T,T28282A,C28977T,C29722T	21	N:D3L,N:S235F,ORF1a:A735V,ORF1a:T1001I,ORF1a:A1708D,ORF1a:I2230T,ORF1a:A2345V,ORF1a:A3945V,ORF1b:P314L,ORF8:Q27*,ORF8:R52I,ORF8:K68*,ORF8:Y73C,S:N501Y,S:A570D,S:D614G,S:P681H,S:T716I,S:V860L,S:S982A,S:D1118H	6	9
hCoV-19/Mexico/YUC-INMEGEN-06-02-65/2021	EPI_ISL_2692597	-	B.1.1.7	GRY	48	C203T,C241T,C913T,C2110T,G3004T,C3037T,C3267T,C3992T,C5388A,C5986T,T6954C,G7042T,C7765T,C10789T,C14120T,C14408T,C14676T,G14857T,C15279T,G15672C,C15810T,T16176C,G20679T,T22219C,G22567T,A23063T,C23271A,A23403G,C23604A,C23709T,T24506G,G24914C,C25731T,C26110T,C26735T,C27002T,C27972T,G28048T,A28095T,A28111G,G28280C,A28281T,T28282A,A28804G,G28881A,G28882A,G28883C,C28977T	26	N:D3L,N:R203K,N:G204R,N:S235F,ORF1a:E913D,ORF1a:T1001I,ORF1a:A1708D,ORF1a:I2230T,ORF1a:M2259I,ORF1b:P218L,ORF1b:P314L,ORF1b:V464F,ORF1b:E735D,ORF3a:P240S,ORF8:Q27*,ORF8:R52I,ORF8:K68*,ORF8:Y73C,S:L335F,S:N501Y,S:A570D,S:D614G,S:P681H,S:T716I,S:S982A,S:D1118H	6	219
hCoV-19/Mexico/YUC-INMEGEN-06-02-67/2021	EPI_ISL_2692599	-	B.1.1.7	GRY	47	C241T,C913T,C2110T,G3004T,C3037T,C3267T,C5388A,C5986T,T6954C,G7042T,C7765T,C10789T,C14120T,C14408T,C14676T,G14857T,C15279T,G15672C,C15810T,T16176C,G20679T,T22219C,G22567T,A23063T,C23271A,A23403G,C23604A,C23709T,T24506G,T24769C,G24914C,C25731T,C26110T,C26735T,C27002T,C27972T,G28048T,A28095T,A28111G,G28280C,A28281T,T28282A,A28804G,G28881A,G28882A,G28883C,C28977T	26	N:D3L,N:R203K,N:G204R,N:S235F,ORF1a:E913D,ORF1a:T1001I,ORF1a:A1708D,ORF1a:I2230T,ORF1a:M2259I,ORF1b:P218L,ORF1b:P314L,ORF1b:V464F,ORF1b:E735D,ORF3a:P240S,ORF8:Q27*,ORF8:R52I,ORF8:K68*,ORF8:Y73C,S:L335F,S:N501Y,S:A570D,S:D614G,S:P681H,S:T716I,S:S982A,S:D1118H	6	655
hCoV-19/Mexico/YUC-INMEGEN-06-02-88/2021	EPI_ISL_2692618	-	B.1.1.7	GRY	45	C241T,C913T,C2110T,G3004T,C3037T,C3267T,C5388A,C5986T,T6954C,G7042T,C7765T,C10789T,C14120T,C14408T,C14676T,G14857T,C15279T,G15672C,C15810T,T16176C,G20679T,T22219C,G22567T,A23063T,C23271A,A23403G,C23604A,C23709T,T24506G,G24914C,C25731T,C26110T,C26735T,C27002T,C27972T,G28048T,A28111G,G28280C,A28281T,T28282A,A28804G,G288	25	N:D3L,N:R203K,N:G204R,N:S235F,ORF1a:E913D,ORF1a:T1001I,ORF1a:A1708D,ORF1a:I2230T,ORF1a:M2259I,ORF1b:P218L,ORF1b:P314L,ORF1b:V464F,ORF1b:E735D,ORF3a:P240S,ORF8:Q27*,ORF8:R52I,ORF8:Y73C,S:L335F,S:N501Y,S:A570D,S:D614G,S:P681H,S:T716I,S:S982A,S:D1118H	6	613
hCoV-19/Mexico/YUC-INMEGEN-06-02-9/2021	EPI_ISL_2692547	-	B.1.1.7	GRY	39	C241T,C913T,C3037T,C3267T,C4752T,C5388A,C5986T,T6954C,C9693T,C11572T,C14408T,C14676T,C15279T,T16176C,C18060A,G18651A,T23042C,A23063T,C23271A,A23403G,C23604A,C23709T,T24506G,G24914C,G25218T,C25553T,G25660C,C27615T,C27972T,G28048T,A28095T,A28111G,G28280C,A28281T,T28282A,G28881A,G28882A,G28883C,C28977T	25	N:D3L,N:R203K,N:G204R,N:S235F,ORF1a:T1001I,ORF1a:T1496I,ORF1a:A1708D,ORF1a:I2230T,ORF1a:A3143V,ORF1b:P314L,ORF3a:A54V,ORF3a:V90L,ORF8:Q27*,ORF8:R52I,ORF8:K68*,ORF8:Y73C,S:S494P,S:N501Y,S:A570D,S:D614G,S:P681H,S:T716I,S:S982A,S:D1118H,S:G1219V	6	0
hCoV-19/Mexico/YUC-INMEGEN-06-04-289/2021	EPI_ISL_2894336	-	B.1.1.7	GRY	39	C241T,C913T,C2110T,C3037T,C3267T,C5388A,C5986T,T6954C,G7042T,A10195G,C10279T,C14120T,C14408T,C14676T,C15279T,T16176C,C16887T,G17427T,A23063T,C23271A,A23403G,C23604A,C23709T,T24506G,G24914C,A27943G,C27972T,G28048T,G28075T,A28095T,A28111G,G28280C,A28281T,T28282A,G28541T,G28881A,G28882A,G28883C,C28977T	25	N:D3L,N:A90S,N:R203K,N:G204R,N:S235F,ORF1a:T1001I,ORF1a:A1708D,ORF1a:I2230T,ORF1a:M2259I,ORF1b:P218L,ORF1b:P314L,ORF8:H17R,ORF8:Q27*,ORF8:R52I,ORF8:C61F,ORF8:K68*,ORF8:Y73C,ORF9b:E86D,S:N501Y,S:A570D,S:D614G,S:P681H,S:T716I,S:S982A,S:D1118H	6	0
hCoV-19/Mexico/YUC-INMEGEN-06-04-29/2021	EPI_ISL_2894176	-	B.1.1.7	GRY	40	C203T,C241T,C913T,G1820A,C3037T,C3267T,G5023T,C5388A,G5924A,C5986T,T6954C,G9526T,A12162G,C14408T,C14676T,C15279T,C15720T,T16176C,A17615G,T19363C,A21137G,A23063T,C23248T,C23271A,A23403G,C23604A,C23709T,T24506G,G24914C,G25135T,C27972T,G28048T,A28111G,G28280C,A28281T,T28282A,G28881A,G28882A,G28883C,C28977T	26	N:D3L,N:R203K,N:G204R,N:S235F,ORF1a:G519S,ORF1a:T1001I,ORF1a:M1586I,ORF1a:A1708D,ORF1a:V1887I,ORF1a:I2230T,ORF1a:M3087I,ORF1a:Q3966R,ORF1b:P314L,ORF1b:K1383R,ORF1b:K2557R,ORF8:Q27*,ORF8:R52I,ORF8:Y73C,S:N501Y,S:A570D,S:D614G,S:P681H,S:T716I,S:S982A,S:D1118H,S:K1191N	6	116
hCoV-19/Mexico/YUC-INMEGEN-06-04-293/2021	EPI_ISL_2894340	-	B.1.1.7	GRY	38	C241T,C913T,C2110T,C3037T,C3267T,C5388A,C5986T,T6954C,G7042T,A10195G,C10279T,C14120T,C14408T,C14676T,C15279T,T16176C,C16887T,G17427T,A23063T,C23271A,A23403G,C23604A,C23709T,T24506G,G24914C,A27943G,C27972T,G28048T,G28075T,A28095T,A28111G,G28280C,A28281T,T28282A,G28881A,G28882A,G28883C,C28977T	23	N:D3L,N:R203K,N:G204R,N:S235F,ORF1a:T1001I,ORF1a:A1708D,ORF1a:I2230T,ORF1a:M2259I,ORF1b:P218L,ORF1b:P314L,ORF8:H17R,ORF8:Q27*,ORF8:R52I,ORF8:C61F,ORF8:K68*,ORF8:Y73C,S:N501Y,S:A570D,S:D614G,S:P681H,S:T716I,S:S982A,S:D1118H	6	157
hCoV-19/Mexico/YUC-INMEGEN-06-04-296/2021	EPI_ISL_2894343	-	B.1.1.7	GRY	38	C241T,C913T,C2110T,C3037T,C3267T,C5388A,C5986T,T6954C,G7042T,A10195G,C10279T,C14120T,C14408T,C14676T,C15279T,T16176C,C16887T,G17427T,A23063T,C23271A,A23403G,C23604A,C23709T,T24506G,G24914C,A27943G,C27972T,G28048T,G28075T,A28095T,A28111G,G28280C,A28281T,T28282A,G28881A,G28882A,G28883C,C28977T	23	N:D3L,N:R203K,N:G204R,N:S235F,ORF1a:T1001I,ORF1a:A1708D,ORF1a:I2230T,ORF1a:M2259I,ORF1b:P218L,ORF1b:P314L,ORF8:H17R,ORF8:Q27*,ORF8:R52I,ORF8:C61F,ORF8:K68*,ORF8:Y73C,S:N501Y,S:A570D,S:D614G,S:P681H,S:T716I,S:S982A,S:D1118H	6	0
hCoV-19/Mexico/YUC-INMEGEN-06-04-299/2021	EPI_ISL_2894346	-	B.1.1.7	GRY	45	C241T,C913T,C2110T,G3004T,C3037T,C3267T,C4055T,C5388A,C5986T,T6954C,G7042T,C7765T,C8980T,C10789T,C14120T,C14408T,C14676T,G14857T,C15279T,T16176C,G20208T,T22219C,G22567T,A23063T,C23271A,A23403G,C23604A,C23709T,T24506G,G24914C,C25731T,C26110T,C26735T,C27972T,G28048T,A28095T,A28111G,G28280C,A28281T,T28282A,A28804G,G28881	27	N:D3L,N:R203K,N:G204R,N:S235F,ORF1a:E913D,ORF1a:T1001I,ORF1a:H1264Y,ORF1a:A1708D,ORF1a:I2230T,ORF1a:M2259I,ORF1b:P218L,ORF1b:P314L,ORF1b:V464F,ORF1b:Q2247H,ORF3a:P240S,ORF8:Q27*,ORF8:R52I,ORF8:K68*,ORF8:Y73C,S:L335F,S:N501Y,S:A570D,S:D614G,S:P681H,S:T716I,S:S982A,S:D1	6	0
hCoV-19/Mexico/YUC-INMEGEN-06-04-308/2021	EPI_ISL_2894355	-	B.1.1.7	GRY	38	C241T,C913T,C2110T,C3037T,C3267T,C5388A,C5986T,T6954C,G7042T,A10195G,C10279T,C14120T,C14408T,C14676T,C15279T,T16176C,C16887T,G17427T,A23063T,C23271A,A23403G,C23604A,C23709T,T24506G,G24914C,A27943G,C27972T,G28048T,G28075T,A28095T,A28111G,G28280C,A28281T,T28282A,G28881A,G28882A,G28883C,C28977T	23	N:D3L,N:R203K,N:G204R,N:S235F,ORF1a:T1001I,ORF1a:A1708D,ORF1a:I2230T,ORF1a:M2259I,ORF1b:P218L,ORF1b:P314L,ORF8:H17R,ORF8:Q27*,ORF8:R52I,ORF8:C61F,ORF8:K68*,ORF8:Y73C,S:N501Y,S:A570D,S:D614G,S:P681H,S:T716I,S:S982A,S:D1118H	6	0
hCoV-19/Mexico/YUC-INMEGEN-06-04-310/2021	EPI_ISL_2894357	-	B.1.1.7	GRY	47	C241T,C913T,C2110T,G3004T,C3037T,C3267T,C5388A,C5986T,T6954C,G7042T,C7765T,C10789T,C14120T,C14408T,C14676T,G14857T,C15279T,G15672C,C15810T,T16176C,C19602T,G20679T,T22219C,G22567T,A23063T,C23271A,A23403G,C23604A,C23709T,T24506G,G24914C,C25731T,C26110T,C26735T,C27002T,C27972T,G28048T,A28095T,A28111G,G28280C,A28281T,T28282A,A28804G,G28881A,G28882A,G28883C,C28977T	26	N:D3L,N:R203K,N:G204R,N:S235F,ORF1a:E913D,ORF1a:T1001I,ORF1a:A1708D,ORF1a:I2230T,ORF1a:M2259I,ORF1b:P218L,ORF1b:P314L,ORF1b:V464F,ORF1b:E735D,ORF3a:P240S,ORF8:Q27*,ORF8:R52I,ORF8:K68*,ORF8:Y73C,S:L335F,S:N501Y,S:A570D,S:D614G,S:P681H,S:T716I,S:S982A,S:D1118H	6	262
hCoV-19/Mexico/YUC-INMEGEN-06-04-338/2021	EPI_ISL_2894382	-	B.1.1.7	GRY	44	C241T,C913T,C2110T,G3004T,C3037T,C3267T,C4055T,C5388A,C5986T,T6954C,G7042T,C7765T,C10789T,C14120T,C14408T,C14676T,G14857T,C15279T,T16176C,G20208T,T22219C,G22567T,A23063T,C23271A,A23403G,C23604A,C23709T,T24506G,G24914C,C25731T,C26110T,C26735T,C27972T,G28048T,A28095T,A28111G,G28280C,A28281T,T28282A,A28804G,G28881A,G28882	27	N:D3L,N:R203K,N:G204R,N:S235F,ORF1a:E913D,ORF1a:T1001I,ORF1a:H1264Y,ORF1a:A1708D,ORF1a:I2230T,ORF1a:M2259I,ORF1b:P218L,ORF1b:P314L,ORF1b:V464F,ORF1b:Q2247H,ORF3a:P240S,ORF8:Q27*,ORF8:R52I,ORF8:K68*,ORF8:Y73C,S:L335F,S:N501Y,S:A570D,S:D614G,S:P681H,S:T716I,S:S982A,S:D1	6	0
hCoV-19/Mexico/YUC-INMEGEN-06-04-345/2021	EPI_ISL_2894389	-	B.1.1.7	GRY	38	C241T,C913T,C2110T,C3037T,C3267T,C5388A,C5986T,T6954C,G7042T,A10195G,C10279T,C14120T,C14408T,C14676T,C15279T,T16176C,C16887T,G17427T,A23063T,C23271A,A23403G,C23604A,C23709T,T24506G,G24914C,A27943G,C27972T,G28048T,G28075T,A28095T,A28111G,G28280C,A28281T,T28282A,G28881A,G28882A,G28883C,C28977T	23	N:D3L,N:R203K,N:G204R,N:S235F,ORF1a:T1001I,ORF1a:A1708D,ORF1a:I2230T,ORF1a:M2259I,ORF1b:P218L,ORF1b:P314L,ORF8:H17R,ORF8:Q27*,ORF8:R52I,ORF8:C61F,ORF8:K68*,ORF8:Y73C,S:N501Y,S:A570D,S:D614G,S:P681H,S:T716I,S:S982A,S:D1118H	6	0
hCoV-19/Mexico/YUC-INMEGEN-06-04-348/2021	EPI_ISL_2894392	-	B.1.1.7	GRY	38	C241T,C913T,C2110T,C3037T,C3267T,C5388A,C5986T,T6954C,G7042T,A10195G,C10279T,C14120T,C14408T,C14676T,C15279T,T16176C,C16887T,G17427T,A23063T,C23271A,A23403G,C23604A,C23709T,T24506G,G24914C,A27943G,C27972T,G28048T,G28075T,A28095T,A28111G,G28280C,A28281T,T28282A,G28881A,G28882A,G28883C,C28977T	23	N:D3L,N:R203K,N:G204R,N:S235F,ORF1a:T1001I,ORF1a:A1708D,ORF1a:I2230T,ORF1a:M2259I,ORF1b:P218L,ORF1b:P314L,ORF8:H17R,ORF8:Q27*,ORF8:R52I,ORF8:C61F,ORF8:K68*,ORF8:Y73C,S:N501Y,S:A570D,S:D614G,S:P681H,S:T716I,S:S982A,S:D1118H	6	0
hCoV-19/Mexico/YUC-INMEGEN-06-04-349/2021	EPI_ISL_2894393	-	B.1.1.7	GRY	38	C241T,C913T,C2110T,C3037T,C3267T,C5388A,C5986T,T6954C,G7042T,A10195G,C10279T,C14120T,C14408T,C14676T,C15279T,T16176C,C16887T,G17427T,A23063T,C23271A,A23403G,C23604A,C23709T,T24506G,G24914C,A27943G,C27972T,G28048T,G28075T,A28095T,A28111G,G28280C,A28281T,T28282A,G28881A,G28882A,G28883C,C28977T	23	N:D3L,N:R203K,N:G204R,N:S235F,ORF1a:T1001I,ORF1a:A1708D,ORF1a:I2230T,ORF1a:M2259I,ORF1b:P218L,ORF1b:P314L,ORF8:H17R,ORF8:Q27*,ORF8:R52I,ORF8:C61F,ORF8:K68*,ORF8:Y73C,S:N501Y,S:A570D,S:D614G,S:P681H,S:T716I,S:S982A,S:D1118H	6	0
hCoV-19/Mexico/YUC-INMEGEN-06-04-43/2021	EPI_ISL_2894187	-	B.1.1.7	GR	43	C241T,C913T,C2110T,G3004T,C3037T,C3267T,C4099T,C5388A,C5986T,T6954C,G7042T,G8846A,C10789T,C14120T,C14408T,C14676T,G14857T,C15279T,T16176C,C18555T,A19838C,G22567T,C23271A,A23403G,C23604A,C23709T,T24506G,G24914C,C26110T,C26735T,C27625T,C27972T,G28048T,A28095T,A28111G,G28280C,A28281T,T28282A,A28804G,G28881A,G28882A,G2888	27	N:D3L,N:R203K,N:G204R,N:S235F,ORF1a:E913D,ORF1a:T1001I,ORF1a:A1708D,ORF1a:I2230T,ORF1a:M2259I,ORF1a:E2861K,ORF1b:P218L,ORF1b:P314L,ORF1b:V464F,ORF1b:N2124T,ORF3a:P240S,ORF7a:R78C,ORF8:Q27*,ORF8:R52I,ORF8:K68*,ORF8:Y73C,S:L335F,S:A570D,S:D614G,S:P681H,S:T716I,S:S982A,S:	10	684
hCoV-19/Mexico/YUC-INMEGEN-06-04-59/2021	EPI_ISL_2894203	-	B.1.1.7	GR	34	C241T,C913T,C2453T,C3037T,C3267T,C5388A,C5986T,T6954C,C9611T,C14408T,C14676T,C15279T,T16176C,G18651T,C21855T,G21974C,C23271A,A23403G,C23410T,C23604A,C23709T,T24506G,G24914C,C27972T,G28048T,A28111G,G28280C,A28281T,T28282A,A28295G,G28881A,G28882A,G28883C,C28977T	23	N:D3L,N:N8D,N:R203K,N:G204R,N:S235F,ORF1a:L730F,ORF1a:T1001I,ORF1a:A1708D,ORF1a:I2230T,ORF1a:L3116F,ORF1b:P314L,ORF1b:E1728D,ORF8:Q27*,ORF8:R52I,ORF8:Y73C,S:S98F,S:D138H,S:A570D,S:D614G,S:P681H,S:T716I,S:S982A,S:D1118H	6	1420

hCoV-19/Mexico/YUC-INMEGEN-06-04-75/2021	EPI_ISL_2894219	-	B.1.1.7	GR	38	C241T,C913T,C2110T,C3037T,C3267T,C5388A,C5986T,T6954C,G7042T,A10195G,C10279T,C14120T,C14408T,C14676T,C15279T,T16176C,C16887T,G17427T,T21775C,C23271A,A23403G,C23604A,C23709T,T24506G,G24914C,A27943G,C27972T,G28048T,G28075T,A28095T,A28111G,G28280C,A28281T,T28282A,G28881A,G28882A,G28883C,C28977T	22	N:D3L,N:R203K,N:G204R,N:S235F,ORF1a:T1001I,ORF1a:A1708D,ORF1a:I2230T,ORF1a:M2259I,ORF1b:P218L,ORF1b:P314L,ORF8:H17R,ORF8:Q27*,ORF8:R52I,ORF8:C61F,ORF8:K68*,ORF8:Y73C,S:A570D,S:D614G,S:P681H,S:T716I,S:S982A,S:D1118H	6	414
hCoV-19/Mexico/YUC-INMEGEN-06-04-84/2021	EPI_ISL_2894227	-	B.1.1.7	GRY	39	C241T,C254T,C913T,C2453T,C3037T,C3267T,C5388A,C5986T,T6954C,C9611T,C14408T,C14676T,C15279T,T16176C,T18099C,C21855T,G21974C,A23063T,C23271A,A23403G,C23604A,C23709T,C23991T,T24506G,G24914C,C25609T,C27128T,C27972T,G28048T,A28111G,G28280C,A28281T,T28282A,A28295G,G28337T,G28881A,G28882A,G28883C,C28977T	27	N:D3L,N:N8D,N:D22Y,N:R203K,N:G204R,N:S235F,ORF1a:L730F,ORF1a:T1001I,ORF1a:A1708D,ORF1a:I2230T,ORF1a:L3116F,ORF1b:P314L,ORF3a:L73F,ORF8:Q27*,ORF8:R52I,ORF8:Y73C,ORF9b:Q18H,S:S98F,S:D138H,S:N501Y,S:A570D,S:D614G,S:P681H,S:T716I,S:S810L,S:S982A,S:D1118H	6	181
hCoV-19/Mexico/YUC-INMEGEN-16-301/2021	EPI_ISL_2978611	-	B.1.1.7	GR	36	C241T,C913T,C2110T,C3037T,C3267T,C5388A,C5986T,T6954C,G7042T,A10195G,C10279T,C14120T,C14408T,C14676T,C15279T,T16176C,C16887T,G17427T,C23271A,A23403G,C23604A,C23709T,T24506G,G24914C,A27943G,C27972T,G28048T,G28075T,A28111G,G28280C,A28281T,T28282A,G28881A,G28882A,G28883C,C28977T	21	N:D3L,N:R203K,N:G204R,N:S235F,ORF1a:T1001I,ORF1a:A1708D,ORF1a:I2230T,ORF1a:M2259I,ORF1b:P218L,ORF1b:P314L,ORF8:H17R,ORF8:Q27*,ORF8:R52I,ORF8:C61F,ORF8:Y73C,S:A570D,S:D614G,S:P681H,S:T716I,S:S982A,S:D1118H	6	416
hCoV-19/Mexico/YUC-INMEGEN-16-333/2021	EPI_ISL_2978638	-	B.1.1.7	GR	35	C241T,C913T,C2110T,C3037T,C3267T,C5388A,C5986T,T6954C,G7042T,A10195G,C10279T,C14120T,C14408T,C14676T,C15279T,T16176C,G17427T,C23271A,A23403G,C23604A,C23709T,T24506G,G24914C,A27943G,C27972T,G28048T,G28075T,A28111G,G28280C,A28281T,T28282A,G28881A,G28882A,G28883C,C28977T	21	N:D3L,N:R203K,N:G204R,N:S235F,ORF1a:T1001I,ORF1a:A1708D,ORF1a:I2230T,ORF1a:M2259I,ORF1b:P218L,ORF1b:P314L,ORF8:H17R,ORF8:Q27*,ORF8:R52I,ORF8:C61F,ORF8:Y73C,S:A570D,S:D614G,S:P681H,S:T716I,S:S982A,S:D1118H	6	2700
hCoV-19/Mexico/YUC-INMEGEN-16-340/2021	EPI_ISL_2978645	-	B.1.1.7	GR	36	C241T,C254T,C913T,C2453T,C3037T,C3267T,C5388A,C5986T,T6954C,C9611T,C14408T,C14676T,C15279T,T16176C,T18099C,C21855T,G21974C,C23271A,A23403G,C23604A,C23709T,T24506G,G24914C,C25609T,A27449G,C27972T,G28048T,A28111G,G28280C,A28281T,T28282A,A28295G,G28881A,G28882A,G28883C,C28977T	24	N:D3L,N:N8D,N:R203K,N:G204R,N:S235F,ORF1a:L730F,ORF1a:T1001I,ORF1a:A1708D,ORF1a:I2230T,ORF1a:L3116F,ORF1b:P314L,ORF3a:L73F,ORF7a:H19R,ORF8:Q27*,ORF8:R52I,ORF8:Y73C,S:S98F,S:D138H,S:A570D,S:D614G,S:P681H,S:T716I,S:S982A,S:D1118H	6	3046
hCoV-19/Mexico/YUC-INMEGEN-16-352/2021	EPI_ISL_2978656	-	B.1.1.7	GRY	39	C241T,C913T,C2453T,C3037T,C3267T,C5388A,C5822T,C5986T,T6954C,A8995G,C9611T,C9893T,C14408T,C14676T,C15279T,T16176C,C17550T,C21855T,G21974C,A23063T,C23271A,A23403G,C23604A,C23709T,T24506G,G24914C,G25440T,G26051A,C27972T,G28048T,A28111G,G28280C,A28281T,T28282A,A28295G,G28881A,G28882A,G28883C,C28977T	27	N:D3L,N:N8D,N:R203K,N:G204R,N:S235F,ORF1a:L730F,ORF1a:T1001I,ORF1a:A1708D,ORF1a:L1853F,ORF1a:I2230T,ORF1a:L3116F,ORF1a:L3210F,ORF1b:P314L,ORF3a:K16N,ORF3a:S220N,ORF8:Q27*,ORF8:R52I,ORF8:Y73C,S:S98F,S:D138H,S:N501Y,S:A570D,S:D614G,S:P681H,S:T716I,S:S982A,S:D1118H	6	0
hCoV-19/Mexico/YUC-INMEGEN-16-357/2021	EPI_ISL_2978661	-	B.1.1.7	GRY	45	C241T,C913T,C2110T,G3004T,C3037T,C3267T,C4055T,C5388A,C5986T,T6954C,G7042T,C7765T,C8980T,C10789T,C14120T,C14408T,C14676T,G14857T,C15279T,T16176C,G20208T,C21736T,T22219C,G22567T,A23063T,C23271A,A23403G,C23604A,C23709T,T24506G,G24914C,C25731T,C26110T,C26735T,C27972T,G28048T,A28111G,G28280C,A28281T,T28282A,A28804G,G28881A,	26	N:D3L,N:R203K,N:G204R,N:S235F,ORF1a:E913D,ORF1a:T1001I,ORF1a:H1264Y,ORF1a:A1708D,ORF1a:I2230T,ORF1a:M2259I,ORF1b:P218L,ORF1b:P314L,ORF1b:V464F,ORF1b:Q2247H,ORF3a:P240S,ORF8:Q27*,ORF8:R52I,ORF8:Y73C,S:L335F,S:N501Y,S:A570D,S:D614G,S:P681H,S:T716I,S:S982A,S:D1118H	6	368
hCoV-19/Mexico/YUC-INMEGEN-16-361/2021	EPI_ISL_2978665	-	B.1.1.7	G	36	C241T,C913T,C2469T,C3037T,C3267T,T4966C,C5388A,C5986T,T6954C,C7299T,C7945T,C12099T,C14184T,C14408T,C14676T,T15096C,C15279T,T16176C,A23063T,C23271A,A23403G,C23604A,C23709T,G24140C,T24506G,G24914C,C27972T,G28048T,A28095T,A28111G,G28280C,A28281T,T28282A,A28877T,C28977T,C29722T	21	N:D3L,N:S235F,ORF1a:A735V,ORF1a:T1001I,ORF1a:A1708D,ORF1a:I2230T,ORF1a:A2345V,ORF1a:A3945V,ORF1b:P314L,ORF8:Q27*,ORF8:R52I,ORF8:K68*,ORF8:Y73C,S:N501Y,S:A570D,S:D614G,S:P681H,S:T716I,S:V860L,S:S982A,S:D1118H	6	6
hCoV-19/Mexico/YUC-NYGC-1163-SM/2021	EPI_ISL_3023819	-	B.1.1.7	GRY	36	C241T,C913T,C2453T,C3037T,C3267T,C5388A,C5986T,T6954C,C9611T,C14408T,C14676T,C15279T,T16176C,G21255C,C21855T,G21974C,A23063T,C23271A,A23403G,C23533T,C23604A,C23709T,T24506G,G24914C,C27513T,C27972T,G28048T,A28111G,G28280C,A28281T,T28282A,A28295G,G28881A,G28882A,G28883C,C28977T	23	N:D3L,N:N8D,N:R203K,N:G204R,N:S235F,ORF1a:L730F,ORF1a:T1001I,ORF1a:A1708D,ORF1a:I2230T,ORF1a:L3116F,ORF1b:P314L,ORF8:Q27*,ORF8:R52I,ORF8:Y73C,S:S98F,S:D138H,S:N501Y,S:A570D,S:D614G,S:P681H,S:T716I,S:S982A,S:D1118H	6	0
hCoV-19/Mexico/YUC-NYGC-1206-SM/2021	EPI_ISL_3023815	-	B.1.1.7	GRY	34	C241T,C913T,C2453T,C3037T,C3267T,C5388A,C5986T,T6523C,T6954C,C9611T,C14408T,C14676T,C15279T,T16176C,C21855T,G21974C,A23063T,C23271A,A23403G,C23604A,C23709T,T24506G,G24914C,C27972T,G28048T,A28111G,G28280C,A28281T,T28282A,A28295G,G28881A,G28	23	N:D3L,N:N8D,N:R203K,N:G204R,N:S235F,ORF1a:L730F,ORF1a:T1001I,ORF1a:A1708D,ORF1a:I2230T,ORF1a:L3116F,ORF1b:P314L,ORF8:Q27*,ORF8:R52I,ORF8:Y73C,S:S98F,S:D138H,S:N501Y,S:A570D,S:D614G,S:P681H,S:T716I,S:S982	6	0
hCoV-19/Mexico/YUC-NYGC-1211-SM/2021	EPI_ISL_3023983	-	B.1.1.7	GRY	35	C241T,C913T,C2453T,C3037T,C3267T,C5388A,C5986T,T6954C,C9611T,C14408T,C14676T,C15279T,T16176C,C18115T,C21855T,G21974C,A23063T,C23271A,A23403G,C23604A,C23709T,T24506G,G24914C,C27972T,G28048T,A28111G,G28280C,A28281T,T28282A,A28295G,C28348T,G28881A,G28882A,G28883C,C28977T	25	N:D3L,N:N8D,N:R203K,N:G204R,N:S235F,ORF1a:L730F,ORF1a:T1001I,ORF1a:A1708D,ORF1a:I2230T,ORF1a:L3116F,ORF1b:P314L,ORF1b:H1550Y,ORF8:Q27*,ORF8:R52I,ORF8:Y73C,ORF9b:A22V,S:S98F,S:D138H,S:N501Y,S:A570D,S:D614G,S:P681H,S:T716I,S:S982A,S:D1118H	6	0
hCoV-19/Mexico/YUC-NYGC-1225-SM/2021	EPI_ISL_3023816	-	B.1.1.7	GRY	34	C241T,C913T,C2453T,C3037T,C3267T,C5388A,C5986T,T6523C,T6954C,C9611T,C14408T,C14676T,C15279T,T16176C,C21855T,G21974C,A23063T,C23271A,A23403G,C23604A,C23709T,T24506G,G24914C,C27972T,G28048T,A28111G,G28280C,A28281T,T28282A,A28295G,G28881A,G28	23	N:D3L,N:N8D,N:R203K,N:G204R,N:S235F,ORF1a:L730F,ORF1a:T1001I,ORF1a:A1708D,ORF1a:I2230T,ORF1a:L3116F,ORF1b:P314L,ORF8:Q27*,ORF8:R52I,ORF8:Y73C,S:S98F,S:D138H,S:N501Y,S:A570D,S:D614G,S:P681H,S:T716I,S:S982	6	0
hCoV-19/Mexico/YUC-NYGC-1232-SM/2021	EPI_ISL_3023948	-	B.1.1.7	GRY	34	C241T,C913T,C2453T,C3037T,C3267T,C5388A,C5986T,T6523C,T6954C,C9611T,C14408T,C14676T,C15279T,T16176C,C21855T,G21974C,A23063T,C23271A,A23403G,C23604A,C23709T,T24506G,G24914C,C27972T,G28048T,A28111G,G28280C,A28281T,T28282A,A28295G,G28881A,G28	23	N:D3L,N:N8D,N:R203K,N:G204R,N:S235F,ORF1a:L730F,ORF1a:T1001I,ORF1a:A1708D,ORF1a:I2230T,ORF1a:L3116F,ORF1b:P314L,ORF8:Q27*,ORF8:R52I,ORF8:Y73C,S:S98F,S:D138H,S:N501Y,S:A570D,S:D614G,S:P681H,S:T716I,S:S982	6	0
hCoV-19/Mexico/YUC-NYGC-14211-21/2021	EPI_ISL_3024031	-	B.1.1.7	GRY	39	C241T,C913T,C3037T,C3267T,C5388A,C5986T,C9693T,C10138T,C11572T,C14408T,C14676T,C15279T,T16176C,G18651A,T23042C,A23063T,C23271A,A23403G,C23604A,C23709T,T24506G,G24914C,C25006T,G25218T,C25553T,G26107T,C27615T,C27972T,G28048T,A28095T,A28111G,G28280C,A28281T,T28282A,G28881A,G28882A,G28883C,C28977T,C29739T	23	N:D3L,N:R203K,N:G204R,N:S235F,ORF1a:T1001I,ORF1a:A1708D,ORF1a:A3143V,ORF1b:P314L,ORF3a:A54V,ORF3a:E239*,ORF8:Q27*,ORF8:R52I,ORF8:K68*,ORF8:Y73C,S:S494P,S:N501Y,S:A570D,S:D614G,S:P681H,S:T716I,S:S982A,S:D1118H,S:G1219V	6	179
hCoV-19/Mexico/YUC-NYGC-14212-21/2021	EPI_ISL_3024035	-	B.1.1.7	GRY	40	C241T,C913T,C3037T,C3267T,C5388A,C5986T,T6954C,C9693T,C10138T,C11572T,C14408T,C14676T,C15279T,T16176C,G18651A,T23042C,A23063T,C23271A,A23403G,C23604A,C23709T,T24506G,G24914C,C25006T,G25218T,C25553T,G26107T,C27615T,C27972T,G28048T,A28095T,A28111G,G28280C,A28281T,T28282A,G28881A,G28882A,G28883C,C28977T,C29739T	24	N:D3L,N:R203K,N:G204R,N:S235F,ORF1a:T1001I,ORF1a:A1708D,ORF1a:I2230T,ORF1a:A3143V,ORF1b:P314L,ORF3a:A54V,ORF3a:E239*,ORF8:Q27*,ORF8:R52I,ORF8:K68*,ORF8:Y73C,S:S494P,S:N501Y,S:A570D,S:D614G,S:P681H,S:T716I,S:S982A,S:D1118H,S:G1219V	6	153
hCoV-19/Mexico/YUC-NYGC-14715-21/2021	EPI_ISL_3024078	-	B.1.1.7	GRY	36	C241T,C913T,C2453T,C3037T,C3267T,C5388A,C5986T,T6954C,C14408T,C14676T,C15279T,C15925T,T16176C,C21855T,G21974C,A23063T,C23271A,A23403G,C23604A,C23709T,T24506G,G24914C,G25244T,C27972T,G28048T,A28111G,G28280C,A28281T,T28282A,A28295G,C28720T,G28881A,G28882A,G28883C,C28977T,G29527T	25	N:D3L,N:N8D,N:R203K,N:G204R,N:S235F,N:Q418H,ORF1a:L730F,ORF1a:T1001I,ORF1a:A1708D,ORF1a:I2230T,ORF1b:P314L,ORF1b:L820F,ORF8:Q27*,ORF8:R52I,ORF8:Y73C,S:S98F,S:D138H,S:N501Y,S:A570D,S:D614G,S:P681H,S:T716I,S:S982A,S:D1118H,S:V1228L	6	1062
hCoV-19/Mexico/YUC-NYGC-15751-21/2021	EPI_ISL_3024024	-	B.1.1.7	GRY	39	C241T,C913T,C2110T,G3004T,C3037T,C3267T,C5388A,C5986T,T6954C,G7042T,C10789T,C14120T,C14408T,C14676T,G14857T,C15279T,T16176C,G22567T,A23063T,C23271A,A23403G,C23604A,C23709T,T24506G,G24914C,C26110T,C26735T,C27972T,G28048T,A28095T,A28111G,G28280C,A28281T,T28282A,A28804G,G28881A,G28882A,G28883C,C28977T	25	N:D3L,N:R203K,N:G204R,N:S235F,ORF1a:E913D,ORF1a:T1001I,ORF1a:A1708D,ORF1a:I2230T,ORF1a:M2259I,ORF1b:P218L,ORF1b:P314L,ORF1b:V464F,ORF3a:P240S,ORF8:Q27*,ORF8:R52I,ORF8:K68*,ORF8:Y73C,S:L335F,S:N501Y,S:A570D,S:D614G,S:P681H,S:T716I,S:S982A,S:D1118H	6	131
hCoV-19/Mexico/YUC-NYGC-8474-21/2021	EPI_ISL_3024018	-	B.1.1.7	GRY	34	C241T,C913T,C3037T,C3267T,C4900T,C5388A,C5986T,T6954C,A12162G,C14408T,C14676T,C15279T,C15720T,T16176C,A17615G,A21137G,A23063T,C23271A,A23403G,C23604A,C23709T,T24506G,G24914C,G25135T,C27972T,G28048T,A28111G,G28280C,A28281T,T28282A,G28881A,G28882A,G28883C,C28977T	22	N:D3L,N:R203K,N:G204R,N:S235F,ORF1a:T1001I,ORF1a:A1708D,ORF1a:I2230T,ORF1a:Q3966R,ORF1b:P314L,ORF1b:K1383R,ORF1b:K2557R,ORF8:Q27*,ORF8:R52I,ORF8:Y73C,S:N501Y,S:A570D,S:D614G,S:P681H,S:T716I,S:S982A,S:D1118H,S:K1191N	6	111
hCoV-19/Mexico/ZAC-InDRE_F10107_S573/2021	EPI_ISL_1335757	-	B.1.1.7	GRY	33	C241T,C913T,C3037T,C3267T,C5388A,C5986T,G6362A,T6954C,C12970T,C13176T,C14408T,C14676T,C15279T,T16176C,A23063T,C23271A,A23403G,C23604A,C23709T,C24023T,C24054T,T24506G,G24914C,C27972T,G28048T,A28111G,G28280C,A28281T,T28282A,G28881A,G28882A,G	21	N:D3L,N:R203K,N:G204R,N:S235F,ORF1a:T1001I,ORF1a:A1708D,ORF1a:A2033T,ORF1a:I2230T,ORF1a:T4304I,ORF1b:P314L,ORF8:Q27*,ORF8:R52I,ORF8:Y73C,S:N501Y,S:A570D,S:D614G,S:P681H,S:T716I,S:A831V,S:S982A,S:D1118H	6	5
hCoV-19/Mexico/ZAC-InDRE_FB16088_S2458/2021	EPI_ISL_2484166	-	B.1.1.7	GRY	38	C241T,C913T,C3037T,C3267T,C5388A,C5986T,G6362A,T6954C,C12970T,C13176T,C14408T,C14676T,C15279T,T16176C,C17977T,C18377T,A23063T,C23271A,A23403G,C23604A,C23709T,C24023T,C24054T,T24506G,G24914C,A25336C,C27046T,C27972T,G28048T,A28111G,G28209T,G28280C,A28281T,T28282A,G28881A,G28882A,G28883C,C28977T	26	M:T175M,N:D3L,N:R203K,N:G204R,N:S235F,ORF1a:T1001I,ORF1a:A1708D,ORF1a:A2033T,ORF1a:I2230T,ORF1a:T4304I,ORF1b:P314L,ORF1b:L1504F,ORF1b:T1637I,ORF8:Q27*,ORF8:R52I,ORF8:Y73C,ORF8:E106*,S:N501Y,S:A570D,S:D614G,S:P681H,S:T716I,S:A831V,S:S982A,S:D1118H,S:E1258D	6	0

hCoV-19/Mexico/ZAC-InDRE_FB17736_S4113/2021	EPI_ISL_2920684	-	B.1.1.7	GRY	41	G90A,C241T,C913T,C2453T,C3037T,C3267T,C5388A,C5986T,T6954C,C9611T,C14408T,C14676T,C15279T,T16176C,C18115T,G21795T,C21855T,T22469C,A23063T,C23271A,A23403G,C23604A,C23709T,T24506G,G24914C,A25336C,G25337C,C27389T,C27972T,G28048T,A28111G,G28280C,A28281T,T28282A,A28295G,C28348T,G28881A,G28882A,G28883C,C28977T,C29098T	27	N:D3L,N:N8D,N:R203K,N:G204R,N:S235F,ORF1a:L730F,ORF1a:T1001I,ORF1a:A1708D,ORF1a:I2230T,ORF1a:L3116F,ORF1b:P314L,ORF1b:H1550Y,ORF8:Q27*,ORF8:R52I,ORF8:Y73C,ORF9b:A22V,S:R78M,S:S98F,S:N501Y,S:A570D,S:D614G,S:P681H,S:T716I,S:S982A,S:D1118H,S:E1258D,S:D1259H	6	0

Table S2. Patient demographic and clinical characteristics of Mexican sequences classified as B.1.1.7 reported as of July 24, 2021. The information of the 473 genome sequences generated by us are shown in blue.

Virus name	Accession ID	Collection date	Location	Host	Additional location information	Gender	Patient age	Patient status	Specimen
hCoV-19/Mexico/BCN-IBT_IMSS_2004/2021	EPI_ISL_2801856	04/06/21	North America / Mexico / Baja Californi	Human		Male	35	Ambulatory	Nasopharyngeal and pharyngeal swabs
hCoV-19/Mexico/BCN-IBT_IMSS_2033/2021	EPI_ISL_2801878	08/06/21	North America / Mexico / Baja Californi	Human		Female	29	Ambulatory	Nasopharyngeal and pharyngeal swabs
hCoV-19/Mexico/BCN-LANGEBIO_IMSS_1438/2021	EPI_ISL_2942560	18/06/21	North America / Mexico / Baja Californi	Human		Female	32	Ambulatory	Nasopharyngeal and pharyngeal swabs
hCoV-19/Mexico/BCN-LANGEBIO_IMSS_1439/2021	EPI_ISL_2942561	21/06/21	North America / Mexico / Baja Californi	Human		Male	47	Ambulatory	Nasopharyngeal and pharyngeal swabs
hCoV-19/Mexico/BCN-LANGEBIO_IMSS_1802/2021	EPI_ISL_2942877	29/06/21	North America / Mexico / Baja Californi	Human		Male	38	Ambulatory	Nasopharyngeal and pharyngeal swabs
hCoV-19/Mexico/BCS_IBT_IMSS_1673/2021	EPI_ISL_2681213	29/05/21	rth America / Mexico / Baja California S	Human		Male	51	Ambulatory	Nasopharyngeal and pharyngeal swabs
hCoV-19/Mexico/BCS_INER_IMSS_00663/2021	EPI_ISL_1585658	17/03/21	rth America / Mexico / Baja California S	Human		Female	32	Ambulatory	Nasopharyngeal and oropharyngeal swabs
hCoV-19/Mexico/BCS-IBT_IMSS_1951/2021	EPI_ISL_2801812	02/06/21	rth America / Mexico / Baja California S	Human		Female	36	Ambulatory	Nasopharyngeal and pharyngeal swabs
hCoV-19/Mexico/BCS-IBT_IMSS_1970/2021	EPI_ISL_2801828	02/06/21	rth America / Mexico / Baja California S	Human		Female	34	Ambulatory	Nasopharyngeal and pharyngeal swabs
hCoV-19/Mexico/CAM_IBT_IMSS_10304-NC/2021	EPI_ISL_2681370	02/06/21	North America / Mexico / Campeche	Human		Female	39	Hospitalized	Nasopharyngeal and pharyngeal swabs
hCoV-19/Mexico/CAM_IBT_IMSS_1746/2021	EPI_ISL_2681118	01/06/21	North America / Mexico / Campeche	Human		Male	40	Ambulatory	Nasopharyngeal and pharyngeal swabs
hCoV-19/Mexico/CAM_INER_IMSS_1117/2021	EPI_ISL_2490327	29/04/21	North America / Mexico / Campeche	Human		Female	56	Ambulatory	Nasopharyngeal and oropharyngeal swabs
hCoV-19/Mexico/CAM_INER_IMSS_1141/2021	EPI_ISL_2490328	06/05/21	North America / Mexico / Campeche	Human		Female	26	Ambulatory	Nasopharyngeal and oropharyngeal swabs
hCoV-19/Mexico/CAM_INER_IMSS_1151/2021	EPI_ISL_2490329	10/05/21	North America / Mexico / Campeche	Human		Female	22	Ambulatory	Nasopharyngeal and oropharyngeal swabs
hCoV-19/Mexico/CAM_LANGEBIO_IMSS_0625/2021	EPI_ISL_2402042	21/04/21	North America / Mexico / Campeche	Human		Male	27	Ambulatory	Nasopharyngeal and pharyngeal swabs
hCoV-19/Mexico/CAM_LANGEBIO_IMSS_08144-NC/2	EPI_ISL_2969870	26/04/21	North America / Mexico / Campeche	Human		Female	30	Ambulatory	Nasopharyngeal and pharyngeal swabs
hCoV-19/Mexico/CAM-IBT_IMSS_1865/2021	EPI_ISL_2801583	03/06/21	North America / Mexico / Campeche	Human		Female	30	Ambulatory	Nasopharyngeal and pharyngeal swabs
hCoV-19/Mexico/CAM-IBT_IMSS_1876/2021	EPI_ISL_2801584	04/06/21	North America / Mexico / Campeche	Human		Female	84	Hospitalized	Nasopharyngeal and pharyngeal swabs
hCoV-19/Mexico/CAM-IBT_IMSS_2098/2021	EPI_ISL_2801645	07/06/21	North America / Mexico / Campeche	Human		Female	65	Hospitalized	Nasopharyngeal and pharyngeal swabs
hCoV-19/Mexico/CAM-IBT_IMSS_2102/2021	EPI_ISL_2801649	07/06/21	North America / Mexico / Campeche	Human		Female	54	Ambulatory	Nasopharyngeal and pharyngeal swabs
hCoV-19/Mexico/CAM-LANGEBIO_IMSS_0940/2021	EPI_ISL_2671501	20/05/21	North America / Mexico / Campeche	Human		Female	58	Ambulatory	Nasopharyngeal and pharyngeal swabs
hCoV-19/Mexico/CAM-LANGEBIO_IMSS_1190/2021	EPI_ISL_2942351	18/06/21	North America / Mexico / Campeche	Human		Female	32	Ambulatory	Nasopharyngeal and pharyngeal swabs
hCoV-19/Mexico/CAM-LANGEBIO_IMSS_1205/2021	EPI_ISL_2942366	18/06/21	North America / Mexico / Campeche	Human		Male	32	Ambulatory	Nasopharyngeal and pharyngeal swabs
hCoV-19/Mexico/CAM-LANGEBIO_IMSS_1207/2021	EPI_ISL_2942368	20/06/21	North America / Mexico / Campeche	Human		Female	49	Ambulatory	Nasopharyngeal and pharyngeal swabs
hCoV-19/Mexico/CAM-LANGEBIO_IMSS_1228/2021	EPI_ISL_2942389	21/06/21	North America / Mexico / Campeche	Human		Female	22	Ambulatory	Nasopharyngeal and pharyngeal swabs
hCoV-19/Mexico/CAM-LANGEBIO_IMSS_1231/2021	EPI_ISL_2942392	21/06/21	North America / Mexico / Campeche	Human		Male	32	Ambulatory	Nasopharyngeal and pharyngeal swabs
hCoV-19/Mexico/CAM-LANGEBIO_IMSS_1548/2021	EPI_ISL_2942659	26/06/21	North America / Mexico / Campeche	Human		Male	45	Hospitalized	Nasopharyngeal and pharyngeal swabs
hCoV-19/Mexico/CAM-LANGEBIO_IMSS_1571/2021	EPI_ISL_2942682	26/06/21	North America / Mexico / Campeche	Human		Female	8	Ambulatory	Nasopharyngeal and pharyngeal swabs
hCoV-19/Mexico/CHH_IBT_IMSS_1367/2021	EPI_ISL_2391727	12/03/21	North America / Mexico / Chihuahua	Human		Female	25	Ambulatory	Nasopharyngeal and pharyngeal swabs
hCoV-19/Mexico/CHH_IBT_IMSS_1370/2021	EPI_ISL_2391639	11/03/21	North America / Mexico / Chihuahua	Human		Male	83	Hospitalized	Nasopharyngeal and pharyngeal swabs
hCoV-19/Mexico/CHH_IBT_IMSS_1423/2021	EPI_ISL_2681357	31/05/21	North America / Mexico / Chihuahua	Human		Male	20	Ambulatory	Nasopharyngeal and pharyngeal swabs
hCoV-19/Mexico/CHH_IBT_IMSS_1429/2021	EPI_ISL_2681316	02/06/21	North America / Mexico / Chihuahua	Human		Female	70	Hospitalized	Nasopharyngeal and pharyngeal swabs
hCoV-19/Mexico/CHH_IBT_IMSS_1430/2021	EPI_ISL_2681289	02/06/21	North America / Mexico / Chihuahua	Human		Female	39	Hospitalized	Nasopharyngeal and pharyngeal swabs
hCoV-19/Mexico/CHH_IBT_IMSS_1431/2021	EPI_ISL_2681359	02/06/21	North America / Mexico / Chihuahua	Human		Female	56	Hospitalized	Nasopharyngeal and pharyngeal swabs
hCoV-19/Mexico/CHH_IBT_IMSS_1432/2021	EPI_ISL_2681137	27/05/21	North America / Mexico / Chihuahua	Human		Female	22	Ambulatory	Nasopharyngeal and pharyngeal swabs
hCoV-19/Mexico/CHH_IBT_IMSS_1434/2021	EPI_ISL_2681103	26/05/21	North America / Mexico / Chihuahua	Human		Female	61	Hospitalized	Nasopharyngeal and pharyngeal swabs
hCoV-19/Mexico/CHH_IBT_IMSS_1435/2021	EPI_ISL_2681265	26/05/21	North America / Mexico / Chihuahua	Human		Female	61	Hospitalized	Nasopharyngeal and pharyngeal swabs
hCoV-19/Mexico/CHH_IBT_IMSS_1436/2021	EPI_ISL_2681277	27/05/21	North America / Mexico / Chihuahua	Human		Female	80	Hospitalized	Nasopharyngeal and pharyngeal swabs
hCoV-19/Mexico/CHH_IBT_IMSS_1442/2021	EPI_ISL_2681248	31/05/21	North America / Mexico / Chihuahua	Human		Male	21	Ambulatory	Nasopharyngeal and pharyngeal swabs
hCoV-19/Mexico/CHH_IBT_IMSS_1443/2021	EPI_ISL_2681219	02/06/21	North America / Mexico / Chihuahua	Human		Male	27	Ambulatory	Nasopharyngeal and pharyngeal swabs
hCoV-19/Mexico/CHH_IBT_IMSS_1444/2021	EPI_ISL_2681104	01/06/21	North America / Mexico / Chihuahua	Human		Female	22	Ambulatory	Nasopharyngeal and pharyngeal swabs
hCoV-19/Mexico/CHH_INER_IMSS_00382/2021	EPI_ISL_1585392	05/03/21	North America / Mexico / Chihuahua	Human		Male	33	Ambulatory	Nasopharyngeal and oropharyngeal swabs
hCoV-19/Mexico/CHH_INER_IMSS_00383/2021	EPI_ISL_1585393	05/03/21	North America / Mexico / Chihuahua	Human		Female	31	Ambulatory	Nasopharyngeal and oropharyngeal swabs
hCoV-19/Mexico/CHH_INER_IMSS_00384/2021	EPI_ISL_1585394	08/03/21	North America / Mexico / Chihuahua	Human		Male	48	Hospitalized	Nasopharyngeal and oropharyngeal swabs
hCoV-19/Mexico/CHH_INER_IMSS_00390/2021	EPI_ISL_1585400	09/03/21	North America / Mexico / Chihuahua	Human		Female	23	Ambulatory	Nasopharyngeal and oropharyngeal swabs
hCoV-19/Mexico/CHH_INER_IMSS_00743/2021	EPI_ISL_2091198	04/04/21	North America / Mexico / Chihuahua	Human		Male	65	Hospitalized	Nasopharyngeal and oropharyngeal swabs
hCoV-19/Mexico/CHH_INER_IMSS_1042/2021	EPI_ISL_2490312	02/05/21	North America / Mexico / Chihuahua	Human		Male	70	Hospitalized	Nasopharyngeal and oropharyngeal swabs

hCoV-19/Mexico/CHH_INER_IMSS_1044/2021	EPI_ISL_2490338	02/05/21	North America / Mexico / Chihuahua	Human	Male	50	Ambulatory	Nasopharyngeal swab
hCoV-19/Mexico/CHH_INER_IMSS_1055/2021	EPI_ISL_2490314	03/05/21	North America / Mexico / Chihuahua	Human	Female	13	Hospitalized	Nasopharyngeal and oropharyngeal swabs
hCoV-19/Mexico/CHH_INER_IMSS_1060/2021	EPI_ISL_2490317	03/05/21	North America / Mexico / Chihuahua	Human	Male	61	Hospitalized	Nasopharyngeal and oropharyngeal swabs
hCoV-19/Mexico/CHH_INER_IMSS_1061/2021	EPI_ISL_2490318	03/05/21	North America / Mexico / Chihuahua	Human	Male	45	Hospitalized	Nasopharyngeal and oropharyngeal swabs
hCoV-19/Mexico/CHH_INER_IMSS_1062/2021	EPI_ISL_2490319	04/05/21	North America / Mexico / Chihuahua	Human	Female	41	Hospitalized	Nasopharyngeal and oropharyngeal swabs
hCoV-19/Mexico/CHH_INER_IMSS_1078/2021	EPI_ISL_2490321	06/05/21	North America / Mexico / Chihuahua	Human	Female	50	Ambulatory	Nasopharyngeal and oropharyngeal swabs
hCoV-19/Mexico/CHH_INER_IMSS_1082/2021	EPI_ISL_2490322	07/05/21	North America / Mexico / Chihuahua	Human	Female	53	Hospitalized	Nasopharyngeal and oropharyngeal swabs
hCoV-19/Mexico/CHH_INER_IMSS_1093/2021	EPI_ISL_2490323	02/05/21	North America / Mexico / Chihuahua	Human	Male	87	Hospitalized	Nasopharyngeal and oropharyngeal swabs
hCoV-19/Mexico/CHH_INER_IMSS_1094/2021	EPI_ISL_2490324	04/05/21	North America / Mexico / Chihuahua	Human	Female	59	Hospitalized	Nasopharyngeal and oropharyngeal swabs
hCoV-19/Mexico/CHH_INER_IMSS_1095/2021	EPI_ISL_2490325	09/05/21	North America / Mexico / Chihuahua	Human	Male	77	Hospitalized	Nasopharyngeal and oropharyngeal swabs
hCoV-19/Mexico/CHH_INER_IMSS_1103/2021	EPI_ISL_2490326	11/05/21	North America / Mexico / Chihuahua	Human	Female	69	Hospitalized	Nasopharyngeal and oropharyngeal swabs
hCoV-19/Mexico/CHH_LANGEBIO_IMSS_0275/2021	EPI_ISL_1661984	25/03/21	North America / Mexico / Chihuahua	Human	Female	30	Ambulatory	Nasopharyngeal swab
hCoV-19/Mexico/CHH_LANGEBIO_IMSS_0278/2021	EPI_ISL_1661987	26/03/21	North America / Mexico / Chihuahua	Human	Female	87	Hospitalized	Nasopharyngeal swab
hCoV-19/Mexico/CHH_LANGEBIO_IMSS_0279/2021	EPI_ISL_1661988	26/03/21	North America / Mexico / Chihuahua	Human	Female	56	Hospitalized	Nasopharyngeal swab
hCoV-19/Mexico/CHH_LANGEBIO_IMSS_0515/2021	EPI_ISL_2401942	19/04/21	North America / Mexico / Chihuahua	Human	Female	34	Hospitalized	Pharyngeal swab
hCoV-19/Mexico/CHH_LANGEBIO_IMSS_0530/2021	EPI_ISL_2401957	20/04/21	North America / Mexico / Chihuahua	Human	Male	66	Hospitalized	Pharyngeal swab
hCoV-19/Mexico/CHH_LANGEBIO_IMSS_0559/2021	EPI_ISL_2401981	24/04/21	North America / Mexico / Chihuahua	Human	Male	80	Hospitalized	Nasopharyngeal and pharyngeal swabs
hCoV-19/Mexico/CHH_LANGEBIO_IMSS_0561/2021	EPI_ISL_2401983	21/04/21	North America / Mexico / Chihuahua	Human	Male	67	Hospitalized	Nasopharyngeal swab
hCoV-19/Mexico/CHH_LANGEBIO_IMSS_0572/2021	EPI_ISL_2401992	23/04/21	North America / Mexico / Chihuahua	Human	Male	75	Ambulatory	Pharyngeal swab
hCoV-19/Mexico/CHH_LANGEBIO_IMSS_0580/2021	EPI_ISL_2402000	27/04/21	North America / Mexico / Chihuahua	Human	Female	33	Ambulatory	Nasopharyngeal and pharyngeal swabs
hCoV-19/Mexico/CHH_LANGEBIO_IMSS_0582/2021	EPI_ISL_2402002	26/04/21	North America / Mexico / Chihuahua	Human	Male	71	Hospitalized	Nasopharyngeal swab
hCoV-19/Mexico/CHH_LANGEBIO_IMSS_45998-NC/2	EPI_ISL_2969876	22/03/21	North America / Mexico / Chihuahua	Human	Male	76	Hospitalized	Nasopharyngeal and pharyngeal swabs
hCoV-19/Mexico/CHH_LANGEBIO_IMSS_46001-NC/2	EPI_ISL_2969877	22/03/21	North America / Mexico / Chihuahua	Human	Male	49	Hospitalized	Nasopharyngeal and pharyngeal swabs
hCoV-19/Mexico/CHH-IBT_IMSS_1799/2021	EPI_ISL_2801678	07/06/21	North America / Mexico / Chihuahua	Human	Female	43	Ambulatory	Nasopharyngeal swab
hCoV-19/Mexico/CHH-IBT_IMSS_1813/2021	EPI_ISL_2801692	08/06/21	North America / Mexico / Chihuahua	Human	Male	83	Hospitalized	Nasopharyngeal and pharyngeal swabs
hCoV-19/Mexico/CHH-INER-IMSS-00220/2021	EPI_ISL_1279266	10/02/21	North America / Mexico / Chihuahua	Human	Male	41	Hospitalized	Nasopharyngeal swab
hCoV-19/Mexico/CHH-LANGEBIO_IMSS_0835/2021	EPI_ISL_2671505	13/05/21	North America / Mexico / Chihuahua	Human	Female	59	Hospitalized	Nasopharyngeal and pharyngeal swabs
hCoV-19/Mexico/CHH-LANGEBIO_IMSS_0836/2021	EPI_ISL_2671506	14/05/21	North America / Mexico / Chihuahua	Human	Male	53	Ambulatory	Nasopharyngeal and pharyngeal swabs
hCoV-19/Mexico/CHH-LANGEBIO_IMSS_0840/2021	EPI_ISL_2671508	14/05/21	North America / Mexico / Chihuahua	Human	Male	73	Hospitalized	Nasopharyngeal and pharyngeal swabs
hCoV-19/Mexico/CHH-LANGEBIO_IMSS_0841/2021	EPI_ISL_2671509	14/05/21	North America / Mexico / Chihuahua	Human	Male	79	Hospitalized	Nasopharyngeal and pharyngeal swabs
hCoV-19/Mexico/CHH-LANGEBIO_IMSS_0842/2021	EPI_ISL_2671510	12/05/21	North America / Mexico / Chihuahua	Human	Male	52	Hospitalized	Nasopharyngeal and pharyngeal swabs
hCoV-19/Mexico/CHH-LANGEBIO_IMSS_0866/2021	EPI_ISL_2671515	15/05/21	North America / Mexico / Chihuahua	Human	Female	50	Hospitalized	Nasopharyngeal and pharyngeal swabs
hCoV-19/Mexico/CHH-LANGEBIO_IMSS_0868/2021	EPI_ISL_2671517	19/05/21	North America / Mexico / Chihuahua	Human	Female	51	Hospitalized	Nasopharyngeal and pharyngeal swabs
hCoV-19/Mexico/CHH-LANGEBIO_IMSS_0871/2021	EPI_ISL_2671519	21/05/21	North America / Mexico / Chihuahua	Human	Male	48	Hospitalized	Pharyngeal swab
hCoV-19/Mexico/CHH-LANGEBIO_IMSS_0879/2021	EPI_ISL_2671521	20/05/21	North America / Mexico / Chihuahua	Human	Female	50	Hospitalized	Nasopharyngeal and pharyngeal swabs
hCoV-19/Mexico/CHH-LANGEBIO_IMSS_0887/2021	EPI_ISL_2671523	21/05/21	North America / Mexico / Chihuahua	Human	Female	57	Hospitalized	Nasopharyngeal and pharyngeal swabs
hCoV-19/Mexico/CHH-LANGEBIO_IMSS_0889/2021	EPI_ISL_2671525	24/05/21	North America / Mexico / Chihuahua	Human	Male	45	Hospitalized	Nasopharyngeal and pharyngeal swabs
hCoV-19/Mexico/CHH-LANGEBIO_IMSS_1366/2021	EPI_ISL_2942497	16/06/21	North America / Mexico / Chihuahua	Human	Female	91	Hospitalized	Nasopharyngeal swab
hCoV-19/Mexico/CHH-LANGEBIO_IMSS_1379/2021	EPI_ISL_2942507	22/06/21	North America / Mexico / Chihuahua	Human	Male	42	Hospitalized	Nasopharyngeal and pharyngeal swabs
hCoV-19/Mexico/CHH-LANGEBIO_IMSS_1681/2021	EPI_ISL_2942772	27/06/21	North America / Mexico / Chihuahua	Human	Male	59	Hospitalized	Nasopharyngeal and pharyngeal swabs
hCoV-19/Mexico/CMX_IBT_IMSS_11022-NC/2021	EPI_ISL_2681369	17/05/21	North America / Mexico / Mexico City	Human	Female	71	Hospitalized	Nasopharyngeal swab
hCoV-19/Mexico/CMX_IBT_IMSS_1133/2021	EPI_ISL_2391710	30/04/21	North America / Mexico / Mexico City	Human	Female	52	Hospitalized	Nasopharyngeal and pharyngeal swabs
hCoV-19/Mexico/CMX_IBT_IMSS_1134/2021	EPI_ISL_2391660	30/04/21	North America / Mexico / Mexico City	Human	Male	84	Hospitalized	Nasopharyngeal and pharyngeal swabs
hCoV-19/Mexico/CMX_IBT_IMSS_1156/2021	EPI_ISL_2391508	01/05/21	North America / Mexico / Mexico City	Human	Male	57	Hospitalized	Pharyngeal swab
hCoV-19/Mexico/CMX_IBT_IMSS_1263/2021	EPI_ISL_2391617	07/05/21	North America / Mexico / Mexico City	Human	Male	39	Hospitalized	Pharyngeal swab
hCoV-19/Mexico/CMX_IBT_IMSS_1281/2021	EPI_ISL_2391572	27/04/21	North America / Mexico / Mexico City	Human	Female	43	Ambulatory	Nasopharyngeal and pharyngeal swabs
hCoV-19/Mexico/CMX_IBT_IMSS_1510/2021	EPI_ISL_2681352	19/05/21	North America / Mexico / Mexico City	Human	Male	54	Hospitalized	Nasopharyngeal and pharyngeal swabs
hCoV-19/Mexico/CMX_IBT_IMSS_1532/2021	EPI_ISL_2681058	25/05/21	North America / Mexico / Mexico City	Human	Female	45	Ambulatory	Nasopharyngeal and pharyngeal swabs
hCoV-19/Mexico/CMX_IBT_IMSS_1535/2021	EPI_ISL_2681165	25/05/21	North America / Mexico / Mexico City	Human	Female	60	Ambulatory	Pharyngeal swab
hCoV-19/Mexico/CMX_IBT_IMSS_1538/2021	EPI_ISL_2681310	24/05/21	North America / Mexico / Mexico City	Human	Female	45	Hospitalized	Nasopharyngeal and pharyngeal swabs
hCoV-19/Mexico/CMX_IBT_IMSS_1554/2021	EPI_ISL_2681167	26/05/21	North America / Mexico / Mexico City	Human	Female	42	Ambulatory	Nasopharyngeal and pharyngeal swabs
hCoV-19/Mexico/CMX_IBT_IMSS_1556/2021	EPI_ISL_2681313	26/05/21	North America / Mexico / Mexico City	Human	Female	23	Ambulatory	Nasopharyngeal and pharyngeal swabs

hCoV-19/Mexico/CMX_IBT_IMSS_1627/2021	EPI_ISL_2681216	31/05/21	North America / Mexico / Mexico City	Human	Male	41	Hospitalized	Nasopharyngeal and pharyngeal swabs
hCoV-19/Mexico/CMX_IBT_IMSS_1636/2021	EPI_ISL_2681195	31/05/21	North America / Mexico / Mexico City	Human	Male	41	Ambulatory	Nasopharyngeal and pharyngeal swabs
hCoV-19/Mexico/CMX_IBT_IMSS_1637/2021	EPI_ISL_2681174	31/05/21	North America / Mexico / Mexico City	Human	Female	17	Ambulatory	Nasopharyngeal and pharyngeal swabs
hCoV-19/Mexico/CMX_IBT_IMSS_1638/2021	EPI_ISL_2681249	31/05/21	North America / Mexico / Mexico City	Human	Female	46	Ambulatory	Nasopharyngeal and pharyngeal swabs
hCoV-19/Mexico/CMX_IBT_IMSS_1643/2021	EPI_ISL_2681226	01/06/21	North America / Mexico / Mexico City	Human	Male	2	Ambulatory	Nasopharyngeal and pharyngeal swabs
hCoV-19/Mexico/CMX_IBT_IMSS_1644/2021	EPI_ISL_2681132	01/06/21	North America / Mexico / Mexico City	Human	Female	22	Ambulatory	Nasopharyngeal and pharyngeal swabs
hCoV-19/Mexico/CMX_IBT_IMSS_1679/2021	EPI_ISL_2681062	02/06/21	North America / Mexico / Mexico City	Human	Female	81	Ambulatory	Nasopharyngeal and pharyngeal swabs
hCoV-19/Mexico/CMX_IBT_IMSS_1686/2021	EPI_ISL_2681066	03/06/21	North America / Mexico / Mexico City	Human	Male	34	Ambulatory	Nasopharyngeal and pharyngeal swabs
hCoV-19/Mexico/CMX_INER_IMSS_00652/2021	EPI_ISL_1585647	21/03/21	North America / Mexico / Mexico City	Human	Male	47	Hospitalized	Nasopharyngeal and oropharyngeal swabs
hCoV-19/Mexico/CMX_INER_IMSS_00878/2021	EPI_ISL_2091329	08/04/21	North America / Mexico / Mexico City	Human	Male	83	Hospitalized	Nasopharyngeal swab
hCoV-19/Mexico/CMX_INER_IMSS_00979/2021	EPI_ISL_2091419	16/04/21	North America / Mexico / Mexico City	Human	Male	0	Ambulatory	Oropharyngeal swab
hCoV-19/Mexico/CMX_INER_IMSS_1254/2021	EPI_ISL_2490349	10/05/21	North America / Mexico / Mexico City	Human	Female	26	Ambulatory	Nasopharyngeal and oropharyngeal swabs
hCoV-19/Mexico/CMX-IBT_IMSS_15419-NC/2021	EPI_ISL_2801784	01/06/21	North America / Mexico / Mexico City	Human	Female	74	Ambulatory	Pharyngeal swab
hCoV-19/Mexico/CMX-IBT_IMSS_1898/2021	EPI_ISL_2801765	31/05/21	North America / Mexico / Mexico City	Human	Female	37	Ambulatory	Nasopharyngeal and pharyngeal swabs
hCoV-19/Mexico/CMX-IBT_IMSS_1917/2021	EPI_ISL_2801781	01/06/21	North America / Mexico / Mexico City	Human	Female	37	Ambulatory	Nasopharyngeal and pharyngeal swabs
hCoV-19/Mexico/CMX-IBT_IMSS_1931/2021	EPI_ISL_2801797	04/06/21	North America / Mexico / Mexico City	Human	Male	78	Ambulatory	Nasopharyngeal and pharyngeal swabs
hCoV-19/Mexico/CMX-INER-IBT-0189/2021	EPI_ISL_2346447	18/04/21	North America / Mexico / Mexico City	Human	Male	40	Hospitalized	Oropharyngeal swab
hCoV-19/Mexico/CMX-INER-IBT-0191/2021	EPI_ISL_2346449	18/04/21	North America / Mexico / Mexico City	Human	Male	63	Hospitalized	Oropharyngeal swab
hCoV-19/Mexico/CMX-INER-IBT-0192/2021	EPI_ISL_2346450	18/04/21	North America / Mexico / Mexico City	Human	Male	65	Hospitalized	Oropharyngeal swab
hCoV-19/Mexico/CMX-INER-IBT-0226/2021	EPI_ISL_2490585	11/05/21	North America / Mexico / Mexico City	Human	Female	46	Hospitalized	Oropharyngeal swab
hCoV-19/Mexico/CMX-INER-IBT-0244/2021	EPI_ISL_2648114	02/05/21	North America / Mexico / Mexico City	Human	Female	65	unknown	Oropharyngeal swab
hCoV-19/Mexico/CMX-INER-IBT-0249/2021	EPI_ISL_2648118	01/06/21	North America / Mexico / Mexico City	Human	Male	31	Ambulatory	Oropharyngeal swab
hCoV-19/Mexico/CMX-INER-IBT-0250/2021	EPI_ISL_2648119	21/04/21	North America / Mexico / Mexico City	Human	Male	86	unknown	Oropharyngeal swab
hCoV-19/Mexico/CMX-INER-IBT-0273/2021	EPI_ISL_2777228	14/06/21	North America / Mexico / Mexico City	Human	Male	19	unknown	Oropharyngeal swab
hCoV-19/Mexico/CMX-LANGEBIO_IMSS_1049/2021	EPI_ISL_2671526	17/05/21	North America / Mexico / Mexico City	Human	Female	35	Ambulatory	Nasopharyngeal and pharyngeal swabs
hCoV-19/Mexico/CMX-LANGEBIO_IMSS_1069/2021	EPI_ISL_2671531	17/05/21	North America / Mexico / Mexico City	Human	Male	58	Hospitalized	Pharyngeal swab
hCoV-19/Mexico/CMX-LANGEBIO_IMSS_1117/2021	EPI_ISL_2671536	20/05/21	North America / Mexico / Mexico City	Human	Male	49	Ambulatory	Nasopharyngeal and pharyngeal swabs
hCoV-19/Mexico/CMX-LANGEBIO_IMSS_1122/2021	EPI_ISL_2671538	20/05/21	North America / Mexico / Mexico City	Human	Male	55	Hospitalized	Pharyngeal swab
hCoV-19/Mexico/CMX-LANGEBIO_IMSS_1461/2021	EPI_ISL_2942580	08/06/21	North America / Mexico / Mexico City	Human	Male	30	Ambulatory	Nasopharyngeal and pharyngeal swabs
hCoV-19/Mexico/CMX-LANGEBIO_IMSS_1463/2021	EPI_ISL_2942582	07/06/21	North America / Mexico / Mexico City	Human	Female	47	Ambulatory	Nasopharyngeal and pharyngeal swabs
hCoV-19/Mexico/CMX-LANGEBIO_IMSS_1466/2021	EPI_ISL_2942584	07/06/21	North America / Mexico / Mexico City	Human	Female	83	Hospitalized	Nasopharyngeal and pharyngeal swabs
hCoV-19/Mexico/COA_IBT_IMSS_1400/2021	EPI_ISL_2681095	29/05/21	North America / Mexico / Coahuila	Human	Male	34	Ambulatory	Nasopharyngeal and pharyngeal swabs
hCoV-19/Mexico/COA_INER_IMSS_1085/2021	EPI_ISL_2490340	08/05/21	North America / Mexico / Coahuila	Human	Female	39	Ambulatory	Nasopharyngeal and oropharyngeal swabs
hCoV-19/Mexico/COA_LANGEBIO_IMSS_0535/2021	EPI_ISL_2401962	22/04/21	North America / Mexico / Coahuila	Human	Male	80	Ambulatory	Nasopharyngeal and pharyngeal swabs
hCoV-19/Mexico/COA_LANGEBIO_IMSS_0575/2021	EPI_ISL_2401995	28/04/21	North America / Mexico / Coahuila	Human	Female	55	Hospitalized	Nasopharyngeal and pharyngeal swabs
hCoV-19/Mexico/COA_LANGEBIO_IMSS_0592/2021	EPI_ISL_2402011	17/04/21	North America / Mexico / Coahuila	Human	Female	62	Ambulatory	Nasopharyngeal and pharyngeal swabs
hCoV-19/Mexico/COA_LANGEBIO_IMSS_0594/2021	EPI_ISL_2402013	16/04/21	North America / Mexico / Coahuila	Human	Male	45	Hospitalized	Nasopharyngeal and pharyngeal swabs
hCoV-19/Mexico/COA_LANGEBIO_IMSS_0595/2021	EPI_ISL_2402014	17/04/21	North America / Mexico / Coahuila	Human	Female	55	Hospitalized	Nasopharyngeal and pharyngeal swabs
hCoV-19/Mexico/COA_LANGEBIO_IMSS_69504-NC/2	EPI_ISL_2969925	30/06/21	North America / Mexico / Coahuila	Human	Female	27	Ambulatory	Nasopharyngeal and pharyngeal swabs
hCoV-19/Mexico/COA-IBT_IMSS_1820/2021	EPI_ISL_2801696	11/06/21	North America / Mexico / Coahuila	Human	Male	47	Ambulatory	Nasopharyngeal and pharyngeal swabs
hCoV-19/Mexico/COA-LANGEBIO_IMSS_0874/2021	EPI_ISL_2671546	23/05/21	North America / Mexico / Coahuila	Human	Female	57	Hospitalized	Nasopharyngeal and pharyngeal swabs
hCoV-19/Mexico/COA-LANGEBIO_IMSS_1345/2021	EPI_ISL_2942482	12/06/21	North America / Mexico / Coahuila	Human	Male	87	Hospitalized	Nasopharyngeal and pharyngeal swabs
hCoV-19/Mexico/COA-LANGEBIO_IMSS_1377/2021	EPI_ISL_2942505	24/06/21	North America / Mexico / Coahuila	Human	Female	31	Hospitalized	Nasopharyngeal and pharyngeal swabs
hCoV-19/Mexico/COA-LANGEBIO_IMSS_1381/2021	EPI_ISL_2942509	25/06/21	North America / Mexico / Coahuila	Human	Male	18	Ambulatory	Nasopharyngeal and pharyngeal swabs
hCoV-19/Mexico/COA-LANGEBIO_IMSS_1672/2021	EPI_ISL_2942765	27/06/21	North America / Mexico / Coahuila	Human	Female	48	Ambulatory	Nasopharyngeal and pharyngeal swabs
hCoV-19/Mexico/COA-LANGEBIO_IMSS_1675/2021	EPI_ISL_2942768	26/06/21	North America / Mexico / Coahuila	Human	Male	63	Ambulatory	Nasopharyngeal and pharyngeal swabs
hCoV-19/Mexico/COA-LANGEBIO_IMSS_1696/2021	EPI_ISL_2942785	30/06/21	North America / Mexico / Coahuila	Human	Female	77	Hospitalized	Nasopharyngeal and pharyngeal swabs
hCoV-19/Mexico/COL_LANGEBIO_IMSS_0767/2021	EPI_ISL_2402178	30/04/21	North America / Mexico / Colima	Human	Female	50	Hospitalized	Nasopharyngeal and pharyngeal swabs
hCoV-19/Mexico/COL-IBT_IMSS_1847/2021	EPI_ISL_2801720	02/06/21	North America / Mexico / Colima	Human	Male	30	Hospitalized	Nasopharyngeal and pharyngeal swabs
hCoV-19/Mexico/COL-IBT_IMSS_1868/2021	EPI_ISL_2801737	06/06/21	North America / Mexico / Colima	Human	Male	74	Hospitalized	Nasopharyngeal and pharyngeal swabs
hCoV-19/Mexico/COL-LANGEBIO_IMSS_1011/2021	EPI_ISL_2671550	19/05/21	North America / Mexico / Colima	Human	Male	30	Hospitalized	Nasopharyngeal and pharyngeal swabs
hCoV-19/Mexico/COL-LANGEBIO_IMSS_1033/2021	EPI_ISL_2671554	24/05/21	North America / Mexico / Colima	Human	Male	45	Ambulatory	Nasopharyngeal and pharyngeal swabs

hCoV-19/Mexico/COL-LANGEBIO_IMSS_1045/2021	EPI_ISL_2671556	29/05/21	North America / Mexico / Colima	Human	Male	53	Hospitalized	Nasopharyngeal and pharyngeal swabs
hCoV-19/Mexico/COL-LANGEBIO_IMSS_1046/2021	EPI_ISL_2671557	29/05/21	North America / Mexico / Colima	Human	Female	35	Hospitalized	Nasopharyngeal and pharyngeal swabs
hCoV-19/Mexico/COL-LANGEBIO_IMSS_1266/2021	EPI_ISL_2942424	16/06/21	North America / Mexico / Colima	Human	Female	59	Hospitalized	Nasopharyngeal and pharyngeal swabs
hCoV-19/Mexico/DUR_IBT_IMSS_1409/2021	EPI_ISL_2681086	01/06/21	North America / Mexico / Durango	Human	Female	67	Hospitalized	Nasopharyngeal and pharyngeal swabs
hCoV-19/Mexico/DUR_INER_IMSS_1050/2021	EPI_ISL_2490339	06/05/21	North America / Mexico / Durango	Human	Male	27	Ambulatory	Nasopharyngeal and oropharyngeal swabs
hCoV-19/Mexico/DUR_LANGEBIO_IMSS_0551/2021	EPI_ISL_2401974	24/04/21	North America / Mexico / Durango	Human	Male	73	Hospitalized	Nasopharyngeal and pharyngeal swabs
hCoV-19/Mexico/DUR-LANGEBIO_IMSS_0824/2021	EPI_ISL_2671558	15/05/21	North America / Mexico / Durango	Human	Female	58	Hospitalized	Nasopharyngeal and pharyngeal swabs
hCoV-19/Mexico/DUR-LANGEBIO_IMSS_0870/2021	EPI_ISL_2671559	21/05/21	North America / Mexico / Durango	Human	Male	43	Ambulatory	Nasopharyngeal and pharyngeal swabs
hCoV-19/Mexico/DUR-LANGEBIO_IMSS_1371/2021	EPI_ISL_2942501	21/06/21	North America / Mexico / Durango	Human	Male	46	Ambulatory	Nasopharyngeal and pharyngeal swabs
hCoV-19/Mexico/GRO-IBT_IMSS_1934/2021	EPI_ISL_2801799	03/06/21	North America / Mexico / Guerrero	Human	Female	32	Ambulatory	Nasopharyngeal and pharyngeal swabs
hCoV-19/Mexico/GRO-IBT_IMSS_2023/2021	EPI_ISL_2801870	07/06/21	North America / Mexico / Guerrero	Human	Male	33	Ambulatory	Nasopharyngeal and pharyngeal swabs
hCoV-19/Mexico/GRO-LANGEBIO_IMSS_1391/2021	EPI_ISL_2942518	16/06/21	North America / Mexico / Guerrero	Human	Male	53	Ambulatory	Nasopharyngeal and pharyngeal swabs
hCoV-19/Mexico/GUA_INER_IMSS_00836/2021	EPI_ISL_2091287	05/04/21	North America / Mexico / Guanajuato	Human	Male	24	Ambulatory	Nasopharyngeal and oropharyngeal swabs
hCoV-19/Mexico/GUA_INER_IMSS_00867/2021	EPI_ISL_2091318	16/04/21	North America / Mexico / Guanajuato	Human	Female	58	Hospitalized	Nasopharyngeal and oropharyngeal swabs
hCoV-19/Mexico/GUA-LANGEBIO_IMSS_1001/2021	EPI_ISL_2671564	17/05/21	North America / Mexico / Guanajuato	Human	Female	68	Hospitalized	Nasopharyngeal and pharyngeal swabs
hCoV-19/Mexico/GUA-LANGEBIO_IMSS_1003/2021	EPI_ISL_2671565	17/05/21	North America / Mexico / Guanajuato	Human	Male	71	Ambulatory	Nasopharyngeal and pharyngeal swabs
hCoV-19/Mexico/GUA-LANGEBIO_IMSS_1013/2021	EPI_ISL_2671567	21/05/21	North America / Mexico / Guanajuato	Human	Male	50	Hospitalized	Nasopharyngeal and pharyngeal swabs
hCoV-19/Mexico/HID-LANGEBIO_IMSS_1451/2021	EPI_ISL_2942571	25/06/21	North America / Mexico / Hidalgo	Human	Male	11	Ambulatory	Nasopharyngeal swab
hCoV-19/Mexico/JAL_INER_IMSS_00822/2021	EPI_ISL_2091273	09/04/21	North America / Mexico / Jalisco	Human	Male	46	Ambulatory	Nasopharyngeal and oropharyngeal swabs
hCoV-19/Mexico/JAL_LANGEBIO_IMSS_0755/2021	EPI_ISL_2402166	27/04/21	North America / Mexico / Jalisco	Human	Male	56	Hospitalized	Nasopharyngeal and pharyngeal swabs
hCoV-19/Mexico/JAL-IBT_IMSS_1852/2021	EPI_ISL_2801725	03/06/21	North America / Mexico / Jalisco	Human	Male	24	Hospitalized	Nasopharyngeal and pharyngeal swabs
hCoV-19/Mexico/JAL-IBT_IMSS_1881/2021	EPI_ISL_2801747	11/06/21	North America / Mexico / Jalisco	Human	Male	30	Ambulatory	Nasopharyngeal and pharyngeal swabs
hCoV-19/Mexico/JAL-LANGEBIO_IMSS_1009/2021	EPI_ISL_2671572	21/05/21	North America / Mexico / Jalisco	Human	Female	25	Ambulatory	Nasopharyngeal and pharyngeal swabs
hCoV-19/Mexico/JAL-LANGEBIO_IMSS_1015/2021	EPI_ISL_2671573	21/05/21	North America / Mexico / Jalisco	Human	Male	22	Ambulatory	Nasopharyngeal and pharyngeal swabs
hCoV-19/Mexico/JAL-LANGEBIO_IMSS_1024/2021	EPI_ISL_2671577	25/05/21	North America / Mexico / Jalisco	Human	Female	54	Ambulatory	Nasopharyngeal and pharyngeal swabs
hCoV-19/Mexico/JAL-LANGEBIO_IMSS_1032/2021	EPI_ISL_2671581	25/05/21	North America / Mexico / Jalisco	Human	Female	89	Ambulatory	Nasopharyngeal swab
hCoV-19/Mexico/JAL-LANGEBIO_IMSS_1247/2021	EPI_ISL_2942408	15/06/21	North America / Mexico / Jalisco	Human	Female	40	Ambulatory	Nasopharyngeal and pharyngeal swabs
hCoV-19/Mexico/JAL-LANGEBIO_IMSS_1257/2021	EPI_ISL_2942416	15/06/21	North America / Mexico / Jalisco	Human	Female	41	Ambulatory	Nasopharyngeal and pharyngeal swabs
hCoV-19/Mexico/JAL-LANGEBIO_IMSS_1281/2021	EPI_ISL_2942436	20/06/21	North America / Mexico / Jalisco	Human	Male	66	Hospitalized	Nasopharyngeal and pharyngeal swabs
hCoV-19/Mexico/JAL-LANGEBIO_IMSS_1317/2021	EPI_ISL_2942464	25/06/21	North America / Mexico / Jalisco	Human	Male	38	Ambulatory	Nasopharyngeal and pharyngeal swabs
hCoV-19/Mexico/JAL-LANGEBIO_IMSS_1621/2021	EPI_ISL_2942725	29/06/21	North America / Mexico / Jalisco	Human	Male	39	Hospitalized	Nasopharyngeal and pharyngeal swabs
hCoV-19/Mexico/MEX_IBT_IMSS_1135/2021	EPI_ISL_2391531	30/04/21	lorth America / Mexico / State of Mexic	Human	Female	78	Hospitalized	Pharyngeal swab
hCoV-19/Mexico/MEX_IBT_IMSS_1249/2021	EPI_ISL_2391647	06/05/21	lorth America / Mexico / State of Mexic	Human	Male	51	Ambulatory	Nasopharyngeal and pharyngeal swabs
hCoV-19/Mexico/MEX_IBT_IMSS_1512/2021	EPI_ISL_2681069	24/05/21	lorth America / Mexico / State of Mexic	Human	Female	54	Ambulatory	Nasopharyngeal and pharyngeal swabs
hCoV-19/Mexico/MEX_IBT_IMSS_1513/2021	EPI_ISL_2681215	21/05/21	lorth America / Mexico / State of Mexic	Human	Male	43	Hospitalized	Nasopharyngeal and pharyngeal swabs
hCoV-19/Mexico/MEX_IBT_IMSS_1571/2021	EPI_ISL_2681111	27/05/21	lorth America / Mexico / State of Mexic	Human	Female	33	Hospitalized	Nasopharyngeal and pharyngeal swabs
hCoV-19/Mexico/MEX_IBT_IMSS_1667/2021	EPI_ISL_2681238	31/05/21	lorth America / Mexico / State of Mexic	Human	Male	58	Hospitalized	Nasopharyngeal and pharyngeal swabs
hCoV-19/Mexico/MEX_INER_IMSS_00690/2021	EPI_ISL_2091146	19/03/21	lorth America / Mexico / State of Mexic	Human	Male	66	Hospitalized	Nasopharyngeal and oropharyngeal swabs
hCoV-19/Mexico/MEX_LANGEBIO_IMSS_20813-NC/2	EPI_ISL_2969962	18/06/21	lorth America / Mexico / State of Mexic	Human	Male	27	Hospitalized	Nasopharyngeal and pharyngeal swabs
hCoV-19/Mexico/MEX-IBT_IMSS_14917-NC/2021	EPI_ISL_2801753	27/05/21	lorth America / Mexico / State of Mexic	Human	Female	58	Ambulatory	Nasopharyngeal and pharyngeal swabs
hCoV-19/Mexico/MEX-LANGEBIO_IMSS_1085/2021	EPI_ISL_2671594	18/05/21	lorth America / Mexico / State of Mexic	Human	Female	82	Hospitalized	Nasopharyngeal and pharyngeal swabs
hCoV-19/Mexico/MEX-LANGEBIO_IMSS_1114/2021	EPI_ISL_2671601	19/05/21	lorth America / Mexico / State of Mexic	Human	Male	46	Ambulatory	Nasopharyngeal and pharyngeal swabs
hCoV-19/Mexico/MEX-LANGEBIO_IMSS_1121/2021	EPI_ISL_2671605	19/05/21	lorth America / Mexico / State of Mexic	Human	Male	33	Ambulatory	Nasopharyngeal and pharyngeal swabs
hCoV-19/Mexico/MEX-LANGEBIO_IMSS_1474/2021	EPI_ISL_2942592	08/06/21	lorth America / Mexico / State of Mexic	Human	Female	62	Hospitalized	Nasopharyngeal and pharyngeal swabs
hCoV-19/Mexico/MEX-LANGEBIO_IMSS_1475/2021	EPI_ISL_2942593	08/06/21	lorth America / Mexico / State of Mexic	Human	Male	40	Hospitalized	Nasopharyngeal and pharyngeal swabs
hCoV-19/Mexico/MEX-LANGEBIO_IMSS_1476/2021	EPI_ISL_2942594	08/06/21	lorth America / Mexico / State of Mexic	Human	Female	59	Hospitalized	Nasopharyngeal and pharyngeal swabs
hCoV-19/Mexico/MEX-LANGEBIO_IMSS_1479/2021	EPI_ISL_2942596	08/06/21	lorth America / Mexico / State of Mexic	Human	Male	31	Ambulatory	Nasopharyngeal swab
hCoV-19/Mexico/MEX-LANGEBIO_IMSS_1576/2021	EPI_ISL_2942687	27/06/21	lorth America / Mexico / State of Mexic	Human	Male	40	Hospitalized	Nasopharyngeal and pharyngeal swabs
hCoV-19/Mexico/MIC_INER_IMSS_1184/2021	EPI_ISL_2490332	07/05/21	North America / Mexico / Michoacan	Human	Female	38	Ambulatory	Nasopharyngeal and oropharyngeal swabs
hCoV-19/Mexico/MOR_IBT_IMSS_1570/2021	EPI_ISL_2681243	26/05/21	North America / Mexico / Morelos	Human	Male	67	Ambulatory	Nasopharyngeal and pharyngeal swabs
hCoV-19/Mexico/MOR_LANGEBIO_IMSS_91231-NC/2	EPI_ISL_2969986	25/03/21	North America / Mexico / Morelos	Human	Male	40	Hospitalized	Nasopharyngeal and pharyngeal swabs
hCoV-19/Mexico/NAY-IBT_IMSS_1850/2021	EPI_ISL_2801723	01/06/21	North America / Mexico / Nayarit	Human	Female	44	Ambulatory	Nasopharyngeal and pharyngeal swabs

hCoV-19/Mexico/NAY-IBT_IMSS_1851/2021	EPI_ISL_2801724	02/06/21	North America / Mexico / Nayarit	Human	Female	35	Ambulatory	Nasopharyngeal and pharyngeal swabs
hCoV-19/Mexico/NAY-LANGEBIO_IMSS_1041/2021	EPI_ISL_2671618	28/05/21	North America / Mexico / Nayarit	Human	Female	22	Ambulatory	Nasopharyngeal and pharyngeal swabs
hCoV-19/Mexico/NLE_IBT_IMSS_1404/2021	EPI_ISL_2681127	01/06/21	North America / Mexico / Nuevo Leon	Human	Female	21	Hospitalized	Nasopharyngeal swab
hCoV-19/Mexico/NLE_IBT_IMSS_1411/2021	EPI_ISL_2681303	02/06/21	North America / Mexico / Nuevo Leon	Human	Female	47	Ambulatory	Nasopharyngeal and pharyngeal swabs
hCoV-19/Mexico/NLE_IBT_IMSS_1415/2021	EPI_ISL_2681197	01/06/21	North America / Mexico / Nuevo Leon	Human	Male	34	Ambulatory	Nasopharyngeal and pharyngeal swabs
hCoV-19/Mexico/NLE_IBT_IMSS_1418/2021	EPI_ISL_2681096	02/06/21	North America / Mexico / Nuevo Leon	Human	Male	27	Ambulatory	Nasopharyngeal and pharyngeal swabs
hCoV-19/Mexico/NLE_IBT_IMSS_1426/2021	EPI_ISL_2681102	02/06/21	North America / Mexico / Nuevo Leon	Human	Female	53	Ambulatory	Nasopharyngeal and pharyngeal swabs
hCoV-19/Mexico/NLE_IBT_IMSS_1427/2021	EPI_ISL_2681308	02/06/21	North America / Mexico / Nuevo Leon	Human	Male	19	Ambulatory	Nasopharyngeal and pharyngeal swabs
hCoV-19/Mexico/NLE_IBT_IMSS_1749/2021	EPI_ISL_2681358	23/05/21	North America / Mexico / Nuevo Leon	Human	Male	41	Hospitalized	Nasopharyngeal and pharyngeal swabs
hCoV-19/Mexico/NLE_INER_IMSS_00735/2021	EPI_ISL_2091191	08/04/21	North America / Mexico / Nuevo Leon	Human	Male	86	Hospitalized	Nasopharyngeal and oropharyngeal swabs
hCoV-19/Mexico/NLE_INER_IMSS_00746/2021	EPI_ISL_2091201	12/04/21	North America / Mexico / Nuevo Leon	Human	Male	93	Hospitalized	Nasopharyngeal and oropharyngeal swabs
hCoV-19/Mexico/NLE_INER_IMSS_00753/2021	EPI_ISL_2091207	12/04/21	North America / Mexico / Nuevo Leon	Human	Female	39	Hospitalized	Nasopharyngeal and oropharyngeal swabs
hCoV-19/Mexico/NLE_INER_IMSS_00765/2021	EPI_ISL_2091219	15/04/21	North America / Mexico / Nuevo Leon	Human	Male	74	Hospitalized	Nasopharyngeal and oropharyngeal swabs
hCoV-19/Mexico/NLE_INER_IMSS_00768/2021	EPI_ISL_2091222	17/04/21	North America / Mexico / Nuevo Leon	Human	Female	92	Hospitalized	Nasopharyngeal and oropharyngeal swabs
hCoV-19/Mexico/NLE_INER_IMSS_1018/2021	EPI_ISL_2490337	02/05/21	North America / Mexico / Nuevo Leon	Human	Female	87	Ambulatory	Nasopharyngeal and oropharyngeal swabs
hCoV-19/Mexico/NLE_INER_IMSS_1027/2021	EPI_ISL_2835916	03/05/21	North America / Mexico / Nuevo Leon	Human	Male	25	Ambulatory	Nasopharyngeal swab
hCoV-19/Mexico/NLE_INER_IMSS_1049/2021	EPI_ISL_2490313	06/05/21	North America / Mexico / Nuevo Leon	Human	Male	31	Ambulatory	Nasopharyngeal and oropharyngeal swabs
hCoV-19/Mexico/NLE_INER_IMSS_1056/2021	EPI_ISL_2490315	07/05/21	North America / Mexico / Nuevo Leon	Human	Female	32	Ambulatory	Nasopharyngeal and oropharyngeal swabs
hCoV-19/Mexico/NLE_INER_IMSS_1058/2021	EPI_ISL_2490316	07/05/21	North America / Mexico / Nuevo Leon	Human	Male	84	Hospitalized	Nasopharyngeal and oropharyngeal swabs
hCoV-19/Mexico/NLE_INER_IMSS_1073/2021	EPI_ISL_2490320	09/05/21	North America / Mexico / Nuevo Leon	Human	Female	41	Hospitalized	Nasopharyngeal and oropharyngeal swabs
hCoV-19/Mexico/NLE_INER_IMSS_1091/2021	EPI_ISL_2490341	11/05/21	North America / Mexico / Nuevo Leon	Human	Female	12	Hospitalized	Nasopharyngeal and oropharyngeal swabs
hCoV-19/Mexico/NLE_INER_IMSS_1102/2021	EPI_ISL_2490342	12/05/21	North America / Mexico / Nuevo Leon	Human	Female	62	Ambulatory	Nasopharyngeal and oropharyngeal swabs
hCoV-19/Mexico/NLE_LANGEBIO_IMSS_0504/2021	EPI_ISL_2401933	20/04/21	North America / Mexico / Nuevo Leon	Human	Male	50	Hospitalized	Nasopharyngeal and pharyngeal swabs
hCoV-19/Mexico/NLE_LANGEBIO_IMSS_0508/2021	EPI_ISL_2401937	19/04/21	North America / Mexico / Nuevo Leon	Human	Male	74	Ambulatory	Nasopharyngeal and pharyngeal swabs
hCoV-19/Mexico/NLE_LANGEBIO_IMSS_0520/2021	EPI_ISL_2401947	22/04/21	North America / Mexico / Nuevo Leon	Human	Male	90	Hospitalized	Nasopharyngeal swab
hCoV-19/Mexico/NLE_LANGEBIO_IMSS_0538/2021	EPI_ISL_2401965	24/04/21	North America / Mexico / Nuevo Leon	Human	Female	81	Hospitalized	Nasopharyngeal and pharyngeal swabs
hCoV-19/Mexico/NLE_LANGEBIO_IMSS_0554/2021	EPI_ISL_2401977	27/04/21	North America / Mexico / Nuevo Leon	Human	Male	33	Hospitalized	Nasopharyngeal and pharyngeal swabs
hCoV-19/Mexico/NLE_LANGEBIO_IMSS_0555/2021	EPI_ISL_2401978	27/04/21	North America / Mexico / Nuevo Leon	Human	Female	58	Hospitalized	Nasopharyngeal and pharyngeal swabs
hCoV-19/Mexico/NLE_LANGEBIO_IMSS_0565/2021	EPI_ISL_2401987	27/04/21	North America / Mexico / Nuevo Leon	Human	Male	72	Hospitalized	Nasopharyngeal and pharyngeal swabs
hCoV-19/Mexico/NLE_LANGEBIO_IMSS_0568/2021	EPI_ISL_2401990	28/04/21	North America / Mexico / Nuevo Leon	Human	Female	31	Ambulatory	Nasopharyngeal and pharyngeal swabs
hCoV-19/Mexico/NLE_LANGEBIO_IMSS_0586/2021	EPI_ISL_2402006	02/05/21	North America / Mexico / Nuevo Leon	Human	Male	49	Hospitalized	Nasopharyngeal swab
hCoV-19/Mexico/NLE_LANGEBIO_IMSS_0587/2021	EPI_ISL_2402007	02/05/21	North America / Mexico / Nuevo Leon	Human	Male	55	Hospitalized	Nasopharyngeal and pharyngeal swabs
hCoV-19/Mexico/NLE_LANGEBIO_IMSS_61186-NC/2	EPI_ISL_2970006	19/05/21	North America / Mexico / Nuevo Leon	Human	Female	56	Hospitalized	Nasopharyngeal and pharyngeal swabs
hCoV-19/Mexico/NLE-IBT_IMSS_1764/2021	EPI_ISL_2801661	03/06/21	North America / Mexico / Nuevo Leon	Human	Male	48	Hospitalized	Nasopharyngeal and pharyngeal swabs
hCoV-19/Mexico/NLE-IBT_IMSS_1768/2021	EPI_ISL_2801663	03/06/21	North America / Mexico / Nuevo Leon	Human	Female	45	Ambulatory	Pharyngeal swab
hCoV-19/Mexico/NLE-IBT_IMSS_1772/2021	EPI_ISL_2801666	04/06/21	North America / Mexico / Nuevo Leon	Human	Female	80	Ambulatory	Nasopharyngeal and pharyngeal swabs
hCoV-19/Mexico/NLE-IBT_IMSS_1774/2021	EPI_ISL_2801667	04/06/21	North America / Mexico / Nuevo Leon	Human	Female	51	Hospitalized	Nasopharyngeal swab
hCoV-19/Mexico/NLE-IBT_IMSS_1779/2021	EPI_ISL_2801668	08/06/21	North America / Mexico / Nuevo Leon	Human	Male	36	Ambulatory	Nasopharyngeal and pharyngeal swabs
hCoV-19/Mexico/NLE-IBT_IMSS_1786/2021	EPI_ISL_2801670	08/06/21	North America / Mexico / Nuevo Leon	Human	Female	57	Ambulatory	Pharyngeal swab
hCoV-19/Mexico/NLE-IBT_IMSS_1796/2021	EPI_ISL_2801675	07/06/21	North America / Mexico / Nuevo Leon	Human	Male	33	Ambulatory	Nasopharyngeal and pharyngeal swabs
hCoV-19/Mexico/NLE-IBT_IMSS_1797/2021	EPI_ISL_2801676	10/06/21	North America / Mexico / Nuevo Leon	Human	Male	84	Ambulatory	Nasopharyngeal and pharyngeal swabs
hCoV-19/Mexico/NLE-IBT_IMSS_1800/2021	EPI_ISL_2801679	10/06/21	North America / Mexico / Nuevo Leon	Human	Female	53	Hospitalized	Nasopharyngeal and pharyngeal swabs
hCoV-19/Mexico/NLE-IBT_IMSS_1809/2021	EPI_ISL_2801685	10/06/21	North America / Mexico / Nuevo Leon	Human	Female	61	Hospitalized	Nasopharyngeal and pharyngeal swabs
hCoV-19/Mexico/NLE-IBT_IMSS_1810/2021	EPI_ISL_2801686	10/06/21	North America / Mexico / Nuevo Leon	Human	Male	32	Ambulatory	Nasopharyngeal and pharyngeal swabs
hCoV-19/Mexico/NLE-IBT_IMSS_66028-NC/2021	EPI_ISL_2801687	11/06/21	North America / Mexico / Nuevo Leon	Human	Female	37	Ambulatory	Nasopharyngeal and pharyngeal swabs
hCoV-19/Mexico/NLE-LANGEBIO_IMSS_0829/2021	EPI_ISL_2671625	17/05/21	North America / Mexico / Nuevo Leon	Human	Female	49	Hospitalized	Nasopharyngeal and pharyngeal swabs
hCoV-19/Mexico/NLE-LANGEBIO_IMSS_0833/2021	EPI_ISL_2671626	18/05/21	North America / Mexico / Nuevo Leon	Human	Female	49	Ambulatory	Nasopharyngeal and pharyngeal swabs
hCoV-19/Mexico/NLE-LANGEBIO_IMSS_0851/2021	EPI_ISL_2671631	20/05/21	North America / Mexico / Nuevo Leon	Human	Male	26	Ambulatory	Nasopharyngeal and pharyngeal swabs
hCoV-19/Mexico/NLE-LANGEBIO_IMSS_0857/2021	EPI_ISL_2671633	21/05/21	North America / Mexico / Nuevo Leon	Human	Female	21	Hospitalized	Nasopharyngeal and pharyngeal swabs
hCoV-19/Mexico/NLE-LANGEBIO_IMSS_1355/2021	EPI_ISL_2942486	17/06/21	North America / Mexico / Nuevo Leon	Human	Male	53	Hospitalized	Nasopharyngeal and pharyngeal swabs
hCoV-19/Mexico/NLE-LANGEBIO_IMSS_1357/2021	EPI_ISL_2942488	17/06/21	North America / Mexico / Nuevo Leon	Human	Male	45	Ambulatory	Nasopharyngeal swab
hCoV-19/Mexico/NLE-LANGEBIO_IMSS_1365/2021	EPI_ISL_2942496	18/06/21	North America / Mexico / Nuevo Leon	Human	Male	53	Hospitalized	Nasopharyngeal and pharyngeal swabs

hCoV-19/Mexico/NLE-LANGEBIO_IMSS_1660/2021	EPI_ISL_2942757	21/06/21	North America / Mexico / Nuevo Leon	Human	Female	21	Ambulatory	Nasopharyngeal and pharyngeal swabs
hCoV-19/Mexico/NLE-LANGEBIO_IMSS_1666/2021	EPI_ISL_2942762	28/06/21	North America / Mexico / Nuevo Leon	Human	Female	12	Ambulatory	Nasopharyngeal and pharyngeal swabs
hCoV-19/Mexico/NLE-LANGEBIO_IMSS_1680/2021	EPI_ISL_2942771	01/07/21	North America / Mexico / Nuevo Leon	Human	Female	42	Ambulatory	Nasopharyngeal and pharyngeal swabs
hCoV-19/Mexico/NLE-LANGEBIO_IMSS_1688/2021	EPI_ISL_2942778	02/07/21	North America / Mexico / Nuevo Leon	Human	Male	18	Ambulatory	Nasopharyngeal and pharyngeal swabs
hCoV-19/Mexico/OAX_INER_IMSS_1120/2021	EPI_ISL_2490345	29/04/21	North America / Mexico / Oaxaca	Human	Male	37	Ambulatory	Nasopharyngeal and oropharyngeal swabs
hCoV-19/Mexico/OAX_LANGEBIO_IMSS_0779/2021	EPI_ISL_2402190	20/04/21	North America / Mexico / Oaxaca	Human	Male	71	Ambulatory	Nasopharyngeal and pharyngeal swabs
hCoV-19/Mexico/OAX_LANGEBIO_IMSS_0812/2021	EPI_ISL_2402222	23/04/21	North America / Mexico / Oaxaca	Human	Female	53	Ambulatory	Nasopharyngeal and pharyngeal swabs
hCoV-19/Mexico/OAX-IBT_IMSS_1929/2021	EPI_ISL_2801795	31/05/21	North America / Mexico / Oaxaca	Human	Female	38	Ambulatory	Nasopharyngeal and pharyngeal swabs
hCoV-19/Mexico/OAX-IBT_IMSS_1980/2021	EPI_ISL_2801837	03/06/21	North America / Mexico / Oaxaca	Human	Female	55	Ambulatory	Nasopharyngeal and pharyngeal swabs
hCoV-19/Mexico/OAX-IBT_IMSS_1981/2021	EPI_ISL_2801838	04/06/21	North America / Mexico / Oaxaca	Human	Female	29	Ambulatory	Nasopharyngeal and pharyngeal swabs
hCoV-19/Mexico/OAX-LANGEBIO_IMSS_1126/2021	EPI_ISL_2671639	19/05/21	North America / Mexico / Oaxaca	Human	Male	51	Ambulatory	Nasopharyngeal and pharyngeal swabs
hCoV-19/Mexico/PUE_IBT_IMSS_1252/2021	EPI_ISL_2391628	06/05/21	North America / Mexico / Puebla	Human	Male	72	Hospitalized	Nasopharyngeal and pharyngeal swabs
hCoV-19/Mexico/PUE_IBT_IMSS_1254/2021	EPI_ISL_2391580	06/05/21	North America / Mexico / Puebla	Human	Male	35	Ambulatory	Nasopharyngeal swab
hCoV-19/Mexico/PUE_IBT_IMSS_1522/2021	EPI_ISL_2681162	22/05/21	North America / Mexico / Puebla	Human	Male	38	Hospitalized	Nasopharyngeal and pharyngeal swabs
hCoV-19/Mexico/PUE_IBT_IMSS_1523/2021	EPI_ISL_2681191	22/05/21	North America / Mexico / Puebla	Human	Male	45	Hospitalized	Nasopharyngeal and pharyngeal swabs
hCoV-19/Mexico/PUE_IBT_IMSS_1549/2021	EPI_ISL_2681347	25/05/21	North America / Mexico / Puebla	Human	Female	82	Hospitalized	Nasopharyngeal and pharyngeal swabs
hCoV-19/Mexico/PUE_IBT_IMSS_1676/2021	EPI_ISL_2681149	01/06/21	North America / Mexico / Puebla	Human	Female	65	Hospitalized	Nasopharyngeal and pharyngeal swabs
hCoV-19/Mexico/PUE_INER_IMSS_1277/2021	EPI_ISL_2490350	09/05/21	North America / Mexico / Puebla	Human	Male	71	Hospitalized	Nasopharyngeal and oropharyngeal swabs
hCoV-19/Mexico/PUE_INER_IMSS_1306/2021	EPI_ISL_2490351	10/05/21	North America / Mexico / Puebla	Human	Female	34	Ambulatory	Nasopharyngeal swab
hCoV-19/Mexico/PUE_INER_IMSS_1322/2021	EPI_ISL_2490352	12/05/21	North America / Mexico / Puebla	Human	Female	27	Ambulatory	Nasopharyngeal swab
hCoV-19/Mexico/PUE_INER_IMSS_1323/2021	EPI_ISL_2490353	08/05/21	North America / Mexico / Puebla	Human	Female	43	Ambulatory	Nasopharyngeal and oropharyngeal swabs
hCoV-19/Mexico/PUE_LANGEBIO_IMSS_0438/2021	EPI_ISL_1662170	23/03/21	North America / Mexico / Puebla	Human	Female	29	Ambulatory	Nasopharyngeal swab
hCoV-19/Mexico/PUE_LANGEBIO_IMSS_0610/2021	EPI_ISL_2402028	17/04/21	North America / Mexico / Puebla	Human	Female	87	Hospitalized	Nasopharyngeal and pharyngeal swabs
hCoV-19/Mexico/PUE-IBT_IMSS_1889/2021	EPI_ISL_2801758	27/05/21	North America / Mexico / Puebla	Human	Male	39	Ambulatory	Nasopharyngeal and pharyngeal swabs
hCoV-19/Mexico/PUE-IBT_IMSS_1911/2021	EPI_ISL_2801776	31/05/21	North America / Mexico / Puebla	Human	Female	42	Ambulatory	Nasopharyngeal and pharyngeal swabs
hCoV-19/Mexico/PUE-IBT_IMSS_1913/2021	EPI_ISL_2801778	29/05/21	North America / Mexico / Puebla	Human	Male	37	Hospitalized	Nasopharyngeal and pharyngeal swabs
hCoV-19/Mexico/PUE-IBT_IMSS_1944/2021	EPI_ISL_2801806	03/06/21	North America / Mexico / Puebla	Human	Male	66	Hospitalized	Nasopharyngeal and pharyngeal swabs
hCoV-19/Mexico/PUE-IBT_IMSS_2022/2021	EPI_ISL_2801869	06/06/21	North America / Mexico / Puebla	Human	Male	34	Ambulatory	Nasopharyngeal and pharyngeal swabs
hCoV-19/Mexico/PUE-LANGEBIO_IMSS_1091/2021	EPI_ISL_2671642	18/05/21	North America / Mexico / Puebla	Human	Female	73	Hospitalized	Nasopharyngeal and pharyngeal swabs
hCoV-19/Mexico/PUE-LANGEBIO_IMSS_1093/2021	EPI_ISL_2671644	17/05/21	North America / Mexico / Puebla	Human	Male	51	Ambulatory	Nasopharyngeal and pharyngeal swabs
hCoV-19/Mexico/PUE-LANGEBIO_IMSS_1153/2021	EPI_ISL_2671652	21/05/21	North America / Mexico / Puebla	Human	Male	46	Ambulatory	Nasopharyngeal and pharyngeal swabs
hCoV-19/Mexico/PUE-LANGEBIO_IMSS_1387/2021	EPI_ISL_2942515	11/06/21	North America / Mexico / Puebla	Human	Female	38	Hospitalized	Nasopharyngeal and pharyngeal swabs
hCoV-19/Mexico/PUE-LANGEBIO_IMSS_1853/2021	EPI_ISL_2942920	01/07/21	North America / Mexico / Puebla	Human	Female	18	Hospitalized	Nasopharyngeal and pharyngeal swabs
hCoV-19/Mexico/QUE_LANGEBIO_IMSS_0516/2021	EPI_ISL_2401943	19/04/21	North America / Mexico / Queretaro	Human	Male	62	Hospitalized	Pharyngeal swab
hCoV-19/Mexico/QUE_LANGEBIO_IMSS_0810/2021	EPI_ISL_2402220	23/04/21	North America / Mexico / Queretaro	Human	Male	48	Ambulatory	Nasopharyngeal and pharyngeal swabs
hCoV-19/Mexico/QUE_LANGEBIO_IMSS_0811/2021	EPI_ISL_2402221	26/04/21	North America / Mexico / Queretaro	Human	Male	50	Hospitalized	Bronchoalveolar lavage
hCoV-19/Mexico/QUE-IBT_IMSS_1993/2021	EPI_ISL_2801848	08/06/21	North America / Mexico / Queretaro	Human	Female	36	Ambulatory	Nasopharyngeal and pharyngeal swabs
hCoV-19/Mexico/QUE-IBT_IMSS_1994/2021	EPI_ISL_2801849	08/06/21	North America / Mexico / Queretaro	Human	Female	53	Ambulatory	Nasopharyngeal and pharyngeal swabs
hCoV-19/Mexico/ROO_IBT_IMSS_1716/2021	EPI_ISL_2681136	31/05/21	North America / Mexico / Quintana Roo	Human	Female	27	Ambulatory	Nasopharyngeal swab
hCoV-19/Mexico/ROO_IBT_IMSS_1729/2021	EPI_ISL_2681233	28/05/21	North America / Mexico / Quintana Roo	Human	Female	50	Hospitalized	Nasopharyngeal and pharyngeal swabs
hCoV-19/Mexico/ROO_IBT_IMSS_1732/2021	EPI_ISL_2681050	31/05/21	North America / Mexico / Quintana Roo	Human	Male	48	Ambulatory	Nasopharyngeal and pharyngeal swabs
hCoV-19/Mexico/ROO_IBT_IMSS_1733/2021	EPI_ISL_2681133	31/05/21	North America / Mexico / Quintana Roo	Human	Female	27	Ambulatory	Nasopharyngeal and pharyngeal swabs
hCoV-19/Mexico/ROO_IBT_IMSS_1745/2021	EPI_ISL_2681176	30/05/21	North America / Mexico / Quintana Roo	Human	Male	40	Hospitalized	Nasopharyngeal and pharyngeal swabs
hCoV-19/Mexico/ROO_INER_IMSS_00420/2021	EPI_ISL_1585428	05/03/21	North America / Mexico / Quintana Roo	Human	Female	32	Hospitalized	Nasopharyngeal and oropharyngeal swabs
hCoV-19/Mexico/ROO_INER_IMSS_00436/2021	EPI_ISL_1585444	10/03/21	North America / Mexico / Quintana Roo	Human	Female	50	Ambulatory	Nasopharyngeal and oropharyngeal swabs
hCoV-19/Mexico/ROO_INER_IMSS_00771/2021	EPI_ISL_2091225	27/03/21	North America / Mexico / Quintana Roo	Human	Male	37	Ambulatory	Nasopharyngeal and oropharyngeal swabs
hCoV-19/Mexico/ROO_INER_IMSS_00790/2021	EPI_ISL_2091243	03/04/21	North America / Mexico / Quintana Roo	Human	Female	59	Ambulatory	Nasopharyngeal and oropharyngeal swabs
hCoV-19/Mexico/ROO_INER_IMSS_00791/2021	EPI_ISL_2091244	08/04/21	North America / Mexico / Quintana Roo	Human	Male	57	Hospitalized	Nasopharyngeal and oropharyngeal swabs
hCoV-19/Mexico/ROO_INER_IMSS_00792/2021	EPI_ISL_2091245	08/04/21	North America / Mexico / Quintana Roo	Human	Female	42	Hospitalized	Nasopharyngeal and oropharyngeal swabs
hCoV-19/Mexico/ROO_INER_IMSS_1130/2021	EPI_ISL_2490311	05/05/21	North America / Mexico / Quintana Roo	Human	Male	63	Hospitalized	Nasopharyngeal and oropharyngeal swabs
hCoV-19/Mexico/ROO_INER_IMSS_1152/2021	EPI_ISL_2490330	06/05/21	North America / Mexico / Quintana Roo	Human	Female	42	Ambulatory	Nasopharyngeal and oropharyngeal swabs
hCoV-19/Mexico/ROO_INER_IMSS_1162/2021	EPI_ISL_2490347	11/05/21	North America / Mexico / Quintana Roo	Human	Male	43	Hospitalized	Nasopharyngeal and oropharyngeal swabs

hCoV-19/Mexico/ROO_LANGEBIO_IMSS_07605-NC/2	EPI_ISL_2970025	17/04/21	North America / Mexico / Quintana Roc	Human		Female	45	Hospitalized	Nasopharyngeal and pharyngeal swabs
hCoV-19/Mexico/ROO-IBT_IMSS_1756/2021	EPI_ISL_2801655	07/06/21	North America / Mexico / Quintana Roc	Human		Male	62	Hospitalized	Nasopharyngeal and pharyngeal swabs
hCoV-19/Mexico/ROO-IBT_IMSS_1812/2021	EPI_ISL_2801577	02/06/21	North America / Mexico / Quintana Roc	Human		Male	49	Hospitalized	Nasopharyngeal and pharyngeal swabs
hCoV-19/Mexico/ROO-IBT_IMSS_1825/2021	EPI_ISL_2801579	02/06/21	North America / Mexico / Quintana Roc	Human		Male	37	Ambulatory	Nasopharyngeal and pharyngeal swabs
hCoV-19/Mexico/ROO-IBT_IMSS_1947/2021	EPI_ISL_2801592	03/06/21	North America / Mexico / Quintana Roc	Human		Female	25	Ambulatory	Nasopharyngeal and pharyngeal swabs
hCoV-19/Mexico/ROO-IBT_IMSS_1969/2021	EPI_ISL_2801594	03/06/21	North America / Mexico / Quintana Roc	Human		Male	56	Ambulatory	Nasopharyngeal and pharyngeal swabs
hCoV-19/Mexico/ROO-IBT_IMSS_2068/2021	EPI_ISL_2801619	04/06/21	North America / Mexico / Quintana Roc	Human		Male	28	Ambulatory	Nasopharyngeal and pharyngeal swabs
hCoV-19/Mexico/ROO-IBT_IMSS_2093/2021	EPI_ISL_2801641	07/06/21	North America / Mexico / Quintana Roc	Human		Female	45	Ambulatory	Nasopharyngeal and pharyngeal swabs
hCoV-19/Mexico/ROO-IBT_IMSS_2106/2021	EPI_ISL_2801653	05/06/21	North America / Mexico / Quintana Roc	Human		Female	37	Hospitalized	Nasopharyngeal and pharyngeal swabs
hCoV-19/Mexico/ROO-LANGEBIO_IMSS_0900/2021	EPI_ISL_2671655	18/05/21	North America / Mexico / Quintana Roc	Human		Female	31	Ambulatory	Nasopharyngeal and pharyngeal swabs
hCoV-19/Mexico/ROO-LANGEBIO_IMSS_0907/2021	EPI_ISL_2671660	17/05/21	North America / Mexico / Quintana Roc	Human		Female	45	Ambulatory	Nasopharyngeal and pharyngeal swabs
hCoV-19/Mexico/ROO-LANGEBIO_IMSS_0926/2021	EPI_ISL_2671671	19/05/21	North America / Mexico / Quintana Roc	Human		Female	25	Ambulatory	Nasopharyngeal and pharyngeal swabs
hCoV-19/Mexico/ROO-LANGEBIO_IMSS_0928/2021	EPI_ISL_2671673	19/05/21	North America / Mexico / Quintana Roc	Human		Female	41	Ambulatory	Nasopharyngeal and pharyngeal swabs
hCoV-19/Mexico/ROO-LANGEBIO_IMSS_0937/2021	EPI_ISL_2671680	21/05/21	North America / Mexico / Quintana Roc	Human		Male	34	Ambulatory	Nasopharyngeal and pharyngeal swabs
hCoV-19/Mexico/ROO-LANGEBIO_IMSS_0939/2021	EPI_ISL_2671682	20/05/21	North America / Mexico / Quintana Roc	Human		Female	28	Hospitalized	Nasopharyngeal and pharyngeal swabs
hCoV-19/Mexico/ROO-LANGEBIO_IMSS_0960/2021	EPI_ISL_2671690	24/05/21	North America / Mexico / Quintana Roc	Human		Male	22	Ambulatory	Nasopharyngeal and pharyngeal swabs
hCoV-19/Mexico/ROO-LANGEBIO_IMSS_0972/2021	EPI_ISL_2671696	26/05/21	North America / Mexico / Quintana Roc	Human		Male	35	Ambulatory	Nasopharyngeal and pharyngeal swabs
hCoV-19/Mexico/ROO-LANGEBIO_IMSS_1237/2021	EPI_ISL_2942398	22/06/21	North America / Mexico / Quintana Roc	Human		Male	32	Ambulatory	Nasopharyngeal and pharyngeal swabs
hCoV-19/Mexico/ROO-LANGEBIO_IMSS_1545/2021	EPI_ISL_2942656	26/06/21	North America / Mexico / Quintana Roc	Human		Female	20	Hospitalized	Nasopharyngeal and pharyngeal swabs
hCoV-19/Mexico/ROO-LANGEBIO_IMSS_1578/2021	EPI_ISL_2942689	27/06/21	North America / Mexico / Quintana Roc	Human		Female	37	Ambulatory	Nasopharyngeal and pharyngeal swabs
hCoV-19/Mexico/ROO-LANGEBIO_IMSS_1590/2021	EPI_ISL_2942701	28/06/21	North America / Mexico / Quintana Roc	Human		Female	52	Ambulatory	Nasopharyngeal and pharyngeal swabs
hCoV-19/Mexico/ROO-LANGEBIO_IMSS_1595/2021	EPI_ISL_2942704	29/06/21	North America / Mexico / Quintana Roc	Human		Male	26	Ambulatory	Nasopharyngeal and pharyngeal swabs
hCoV-19/Mexico/SIN_CIAD-CLN_D0035/2021	EPI_ISL_2680920	01/06/21	orth America / Mexico / Sinaloa / Ahom	Human		Female	42	unknown	Oropharyngeal swab
hCoV-19/Mexico/SIN_CIAD-CLN_D0511/2021	EPI_ISL_2927967	11/06/21	rth America / Mexico / Sinaloa / Culiac	Human		Female	23	unknown	Oropharyngeal swab
hCoV-19/Mexico/SIN_CIAD-CLN_D0557/2021	EPI_ISL_2927968	12/06/21	rth America / Mexico / Sinaloa / Culiac	Human		Female	52	unknown	Oropharyngeal swab
hCoV-19/Mexico/SIN_CIAD-CLN_D1476/2021	EPI_ISL_2680923	30/05/21	rth America / Mexico / Sinaloa / Culiac	Human		Female	49	unknown	Oropharyngeal swab
hCoV-19/Mexico/SIN_CIAD-CLN_D1477/2021	EPI_ISL_2680924	30/05/21	rth America / Mexico / Sinaloa / Culiac	Human		Female	54	unknown	Oropharyngeal swab
hCoV-19/Mexico/SIN_CIAD-CLN_D1511/2021	EPI_ISL_2680927	31/05/21	rth America / Mexico / Sinaloa / Culiac	Human		Female	77	unknown	Oropharyngeal swab
hCoV-19/Mexico/SIN_CIAD-CLN_F0851/2021	EPI_ISL_2533936	19/05/21	North America / Mexico / Sinaloa	Human	Other: Ahome	Male	46	unknown	Oropharyngeal swab
hCoV-19/Mexico/SIN_CIAD-CLN_F0854/2021	EPI_ISL_2680928	03/06/21	orth America / Mexico / Sinaloa / Ahom	Human		Female	52	unknown	Oropharyngeal swab
hCoV-19/Mexico/SIN_CIAD-CLN_S0600/2021	EPI_ISL_2927971	21/06/21	rth America / Mexico / Sinaloa / Culiac	Human		Male	22	unknown	Oropharyngeal swab
hCoV-19/Mexico/SIN_CIAD-MZT_HJ1711/2021	EPI_ISL_2533917	26/05/21	North America / Mexico / Sinaloa	Human	ther: Mazatla	Female	59	unknown	Oropharyngeal swab
hCoV-19/Mexico/SIN_CIAD-MZT_HJ1739/2021	EPI_ISL_2680932	02/06/21	rth America / Mexico / Sinaloa / Mazatl	Human		Male	49	Ambulatory	Oropharyngeal swab
hCoV-19/Mexico/SIN_CIAD-MZT_HJ1742/2021	EPI_ISL_2680933	02/06/21	rth America / Mexico / Sinaloa / Mazatl	Human		Male	41	Ambulatory	Oropharyngeal swab
hCoV-19/Mexico/SIN_CIAD-MZT_HJ1755/2021	EPI_ISL_2680936	04/06/21	rth America / Mexico / Sinaloa / Mazatl	Human		Female	38	Ambulatory	Oropharyngeal swab
hCoV-19/Mexico/SIN_CIAD-MZT_HJ1757/2021	EPI_ISL_2894554	04/06/21	rth America / Mexico / Sinaloa / Mazatl	Human		Female	43	Ambulatory	Oropharyngeal swab
hCoV-19/Mexico/SIN_CIAD-MZT_HJ1776/2021	EPI_ISL_2894557	07/06/21	rth America / Mexico / Sinaloa / Mazatl	Human		Male	31	Ambulatory	Oropharyngeal swab
hCoV-19/Mexico/SIN_CIAD-MZT_HJ1782/2021	EPI_ISL_2894558	09/06/21	rth America / Mexico / Sinaloa / Mazatl	Human		Male	25	Ambulatory	Oropharyngeal swab
hCoV-19/Mexico/SIN_CIAD-MZT_HJ1922/2021	EPI_ISL_2927976	23/06/21	rth America / Mexico / Sinaloa / Mazatl	Human		Female	54	unknown	Oropharyngeal swab
hCoV-19/Mexico/SIN_CIAD-MZT_ID2430/2021	EPI_ISL_2533913	15/05/21	North America / Mexico / Sinaloa	Human	ther: Mazatla	Male	63	unknown	Oropharyngeal swab
hCoV-19/Mexico/SIN_CIAD-MZT_ID2531/2021	EPI_ISL_2533918	27/05/21	North America / Mexico / Sinaloa	Human	ther: Mazatla	Male	43	unknown	Oropharyngeal swab
hCoV-19/Mexico/SIN_CIAD-MZT_ID2728/2021	EPI_ISL_2927972	21/06/21	rth America / Mexico / Sinaloa / Mazatl	Human		Male	14	unknown	Oropharyngeal swab
hCoV-19/Mexico/SIN_CIAD-MZT_S6810/2021	EPI_ISL_2533914	16/05/21	North America / Mexico / Sinaloa	Human	ther: Mazatla	Male	37	unknown	Oropharyngeal swab
hCoV-19/Mexico/SIN_CIAD-MZT_S6840/2021	EPI_ISL_2533915	20/05/21	North America / Mexico / Sinaloa	Human	ther: Mazatla	Male	37	unknown	Oropharyngeal swab
hCoV-19/Mexico/SIN_CIAD-MZT_S6867/2021	EPI_ISL_2533916	21/05/21	North America / Mexico / Sinaloa	Human	ther: Mazatla	Male	37	unknown	Oropharyngeal swab
hCoV-19/Mexico/SIN_CIAD-MZT_S6921/2021	EPI_ISL_2533919	28/05/21	North America / Mexico / Sinaloa	Human	ther: Mazatla	Male	31	unknown	Oropharyngeal swab
hCoV-19/Mexico/SIN_CIAD-MZT_S7016/2021	EPI_ISL_2680938	05/06/21	rth America / Mexico / Sinaloa / Mazatl	Human		Male	33	Ambulatory	Oropharyngeal swab
hCoV-19/Mexico/SIN_CIAD-MZT_S7028/2021	EPI_ISL_2680941	05/06/21	rth America / Mexico / Sinaloa / Mazatl	Human		Male	31	Ambulatory	Oropharyngeal swab
hCoV-19/Mexico/SIN_CIAD-MZT_S7158/2021	EPI_ISL_2927974	22/06/21	rth America / Mexico / Sinaloa / Mazatl	Human		Male	39	unknown	Oropharyngeal swab
hCoV-19/Mexico/SIN_INER_IMSS_1180/2021	EPI_ISL_2490331	10/05/21	North America / Mexico / Sinaloa	Human		Male	43	Hospitalized	Nasopharyngeal and oropharyngeal swabs
hCoV-19/Mexico/SIN-CIAD-CLN_D0174/2021	EPI_ISL_2987687	03/06/21	rth America / Mexico / Sinaloa / Guasa	Human		Female	52	unknown	Oropharyngeal swab
hCoV-19/Mexico/SIN-CIAD-CLN_F0865/2021	EPI_ISL_2987694	25/06/21	orth America / Mexico / Sinaloa / Ahom	Human		Male	33	unknown	Oropharyngeal swab

hCoV-19/Mexico/SIN-CIAD-MZT_HJ1803/2021	EPI_ISL_2987696	15/06/21	rth America / Mexico / Sinaloa / Mazatl	Human	Female	26	unknown	Oropharyngeal swab
hCoV-19/Mexico/SIN-CIAD-MZT_S7079/2021	EPI_ISL_2987713	11/06/21	rth America / Mexico / Sinaloa / Mazatl	Human	Male	21	unknown	Oropharyngeal swab
hCoV-19/Mexico/SIN-CIAD-MZT_S7103/2021	EPI_ISL_2987717	14/06/21	rth America / Mexico / Sinaloa / Mazatl	Human	Female	52	unknown	Oropharyngeal swab
hCoV-19/Mexico/SIN-CIAD-MZT_S7104/2021	EPI_ISL_2987718	14/06/21	rth America / Mexico / Sinaloa / Mazatl	Human	Female	47	unknown	Oropharyngeal swab
hCoV-19/Mexico/SIN-CIAD-MZT_S7130/2021	EPI_ISL_2987721	18/06/21	rth America / Mexico / Sinaloa / Mazatl	Human	Male	57	unknown	Oropharyngeal swab
hCoV-19/Mexico/SIN-IBT_IMSS_1832/2021	EPI_ISL_2801707	29/05/21	North America / Mexico / Sinaloa	Human	Male	90	Hospitalized	Nasopharyngeal and pharyngeal swabs
hCoV-19/Mexico/SIN-IBT_IMSS_1871/2021	EPI_ISL_2801739	07/06/21	North America / Mexico / Sinaloa	Human	Female	44	Hospitalized	Nasopharyngeal and pharyngeal swabs
hCoV-19/Mexico/SIN-IBT_IMSS_1879/2021	EPI_ISL_2801745	08/06/21	North America / Mexico / Sinaloa	Human	Female	17	Ambulatory	Nasopharyngeal and pharyngeal swabs
hCoV-19/Mexico/SIN-LANGEBIO_IMSS_1269/2021	EPI_ISL_2942427	15/06/21	North America / Mexico / Sinaloa	Human	Male	38	Hospitalized	Nasopharyngeal and pharyngeal swabs
hCoV-19/Mexico/SIN-LANGEBIO_IMSS_1277/2021	EPI_ISL_2942433	17/06/21	North America / Mexico / Sinaloa	Human	Male	71	Hospitalized	Nasopharyngeal and pharyngeal swabs
hCoV-19/Mexico/SIN-LANGEBIO_IMSS_1291/2021	EPI_ISL_2942444	22/06/21	North America / Mexico / Sinaloa	Human	Female	52	Ambulatory	Nasopharyngeal and pharyngeal swabs
hCoV-19/Mexico/SIN-LANGEBIO_IMSS_1293/2021	EPI_ISL_2942445	22/06/21	North America / Mexico / Sinaloa	Human	Male	43	Ambulatory	Nasopharyngeal and pharyngeal swabs
hCoV-19/Mexico/SIN-LANGEBIO_IMSS_1305/2021	EPI_ISL_2942455	22/06/21	North America / Mexico / Sinaloa	Human	Female	20	Ambulatory	Nasopharyngeal and pharyngeal swabs
hCoV-19/Mexico/SLP_IBT_IMSS_1474/2021	EPI_ISL_2681212	27/05/21	lorth America / Mexico / San Luis Poto:	Human	Male	77	Hospitalized	Nasopharyngeal and pharyngeal swabs
hCoV-19/Mexico/SLP-IBT_IMSS_1823/2021	EPI_ISL_2801699	06/06/21	lorth America / Mexico / San Luis Poto:	Human	Male	19	Ambulatory	Nasopharyngeal and pharyngeal swabs
hCoV-19/Mexico/SLP-IBT_IMSS_1824/2021	EPI_ISL_2801700	07/06/21	lorth America / Mexico / San Luis Poto:	Human	Female	48	Hospitalized	Nasopharyngeal and pharyngeal swabs
hCoV-19/Mexico/SLP-IBT_IMSS_1830/2021	EPI_ISL_2801705	10/06/21	lorth America / Mexico / San Luis Poto:	Human	Female	40	Ambulatory	Nasopharyngeal and pharyngeal swabs
hCoV-19/Mexico/SLP-LANGEBIO_IMSS_0974/2021	EPI_ISL_2671700	13/05/21	lorth America / Mexico / San Luis Poto:	Human	Male	31	Hospitalized	Nasopharyngeal and pharyngeal swabs
hCoV-19/Mexico/SLP-LANGEBIO_IMSS_1326/2021	EPI_ISL_2942471	15/06/21	lorth America / Mexico / San Luis Poto:	Human	Male	40	Ambulatory	Nasopharyngeal and pharyngeal swabs
hCoV-19/Mexico/SLP-LANGEBIO_IMSS_1327/2021	EPI_ISL_2942472	16/06/21	lorth America / Mexico / San Luis Poto:	Human	Male	46	Hospitalized	Nasopharyngeal and pharyngeal swabs
hCoV-19/Mexico/SLP-LANGEBIO_IMSS_1333/2021	EPI_ISL_2942476	24/06/21	lorth America / Mexico / San Luis Poto:	Human	Female	40	Ambulatory	Nasopharyngeal and pharyngeal swabs
hCoV-19/Mexico/SLP-LANGEBIO_IMSS_1334/2021	EPI_ISL_2942477	24/06/21	lorth America / Mexico / San Luis Poto:	Human	Female	45	Hospitalized	Nasopharyngeal and pharyngeal swabs
hCoV-19/Mexico/SON_CIAD-HMO_16010/2021	EPI_ISL_2927979	15/06/21	rth America / Mexico / Sonora / Hermos	Human	Female	49	unknown	Oropharyngeal swab
hCoV-19/Mexico/SON_CIAD-HMO_16011/2021	EPI_ISL_2927980	15/06/21	rth America / Mexico / Sonora / Hermos	Human	Male	24	unknown	Oropharyngeal swab
hCoV-19/Mexico/SON_CIAD-HMO_2555/2021	EPI_ISL_2894566	04/06/21	rth America / Mexico / Sonora / Hermos	Human	Female	8	Ambulatory	Oropharyngeal swab
hCoV-19/Mexico/SON_CIAD-HMO_2613/2021	EPI_ISL_2894569	11/06/21	rth America / Mexico / Sonora / Hermos	Human	Female	39	Ambulatory	Oropharyngeal swab
hCoV-19/Mexico/SON-IBT_IMSS_1840/2021	EPI_ISL_2801715	30/05/21	North America / Mexico / Sonora	Human	Female	44	Hospitalized	Nasopharyngeal and pharyngeal swabs
hCoV-19/Mexico/SON-LANGEBIO_IMSS_1288/2021	EPI_ISL_2942442	21/06/21	North America / Mexico / Sonora	Human	Male	32	Hospitalized	Nasopharyngeal swab
hCoV-19/Mexico/SON-LANGEBIO_IMSS_1289/2021	EPI_ISL_2942443	20/06/21	North America / Mexico / Sonora	Human	Female	26	Hospitalized	Nasopharyngeal and pharyngeal swabs
hCoV-19/Mexico/TAB_IBT_IMSS_10132-NC/2021	EPI_ISL_2681374	31/05/21	North America / Mexico / Tabasco	Human	Male	66	Hospitalized	Nasopharyngeal and pharyngeal swabs
hCoV-19/Mexico/TAB_IBT_IMSS_1351/2021	EPI_ISL_2391728	12/04/21	North America / Mexico / Tabasco	Human	Female	26	Ambulatory	Nasopharyngeal and pharyngeal swabs
hCoV-19/Mexico/TAB_INER_IMSS_1105/2021	EPI_ISL_2490343	28/04/21	North America / Mexico / Tabasco	Human	Female	57	Hospitalized	Nasopharyngeal and oropharyngeal swabs
hCoV-19/Mexico/TAB_INER_IMSS_1124/2021	EPI_ISL_2490346	02/05/21	North America / Mexico / Tabasco	Human	Female	30	Hospitalized	Nasopharyngeal and oropharyngeal swabs
hCoV-19/Mexico/TAB_LANGEBIO_IMSS_0647/2021	EPI_ISL_2402064	25/04/21	North America / Mexico / Tabasco	Human	Female	63	Ambulatory	Nasopharyngeal and pharyngeal swabs
hCoV-19/Mexico/TAB_LANGEBIO_IMSS_09609-NC/2	EPI_ISL_2970045	22/05/21	North America / Mexico / Tabasco	Human	Male	53	Ambulatory	Nasopharyngeal and pharyngeal swabs
hCoV-19/Mexico/TAB-LANGEBIO_IMSS_0911/2021	EPI_ISL_2671705	19/05/21	North America / Mexico / Tabasco	Human	Male	43	Hospitalized	Nasopharyngeal and pharyngeal swabs
hCoV-19/Mexico/TAB-LANGEBIO_IMSS_1519/2021	EPI_ISL_2942632	23/06/21	North America / Mexico / Tabasco	Human	Male	67	Hospitalized	Nasopharyngeal and pharyngeal swabs
hCoV-19/Mexico/TAB-LANGEBIO_IMSS_1539/2021	EPI_ISL_2942650	24/06/21	North America / Mexico / Tabasco	Human	Female	33	Ambulatory	Nasopharyngeal and pharyngeal swabs
hCoV-19/Mexico/TAM_IBT_IMSS_1373/2021	EPI_ISL_2391732	06/04/21	North America / Mexico / Tamaulipas	Human	Male	23	Ambulatory	Nasopharyngeal and pharyngeal swabs
hCoV-19/Mexico/TAM_LANGEBIO_IMSS_0049/2021	EPI_ISL_1351540	03/03/21	North America / Mexico / Jalisco	Human	Male	57	Hospitalized	Nasopharyngeal and pharyngeal swabs
hCoV-19/Mexico/TAM_LANGEBIO_IMSS_38905-NC/2	EPI_ISL_2970048	02/03/21	North America / Mexico / Tamaulipas	Human	Female	24	Ambulatory	Nasopharyngeal and pharyngeal swabs
hCoV-19/Mexico/TAM_LANGEBIO_IMSS_67638-NC/2	EPI_ISL_2970051	20/06/21	North America / Mexico / Tamaulipas	Human	Male	35	Ambulatory	Nasopharyngeal and pharyngeal swabs
hCoV-19/Mexico/TAM-IBT_IMSS_1792/2021	EPI_ISL_2801673	08/06/21	North America / Mexico / Tamaulipas	Human	Male	44	Ambulatory	Nasopharyngeal and pharyngeal swabs
hCoV-19/Mexico/TAM-LANGEBIO_IMSS_0849/2021	EPI_ISL_2671717	18/05/21	North America / Mexico / Tamaulipas	Human	Male	65	Ambulatory	Nasopharyngeal and pharyngeal swabs
hCoV-19/Mexico/TAM-LANGEBIO_IMSS_0854/2021	EPI_ISL_2671718	19/05/21	North America / Mexico / Tamaulipas	Human	Male	60	Ambulatory	Nasopharyngeal and pharyngeal swabs
hCoV-19/Mexico/TAM-LANGEBIO_IMSS_0884/2021	EPI_ISL_2671722	25/05/21	North America / Mexico / Tamaulipas	Human	Female	58	Ambulatory	Nasopharyngeal and pharyngeal swabs
hCoV-19/Mexico/TAM-LANGEBIO_IMSS_1368/2021	EPI_ISL_2942498	16/06/21	North America / Mexico / Tamaulipas	Human	Male	31	Ambulatory	Nasopharyngeal and pharyngeal swabs
hCoV-19/Mexico/TAM-LANGEBIO_IMSS_1375/2021	EPI_ISL_2942504	21/06/21	North America / Mexico / Tamaulipas	Human	Male	52	Ambulatory	Nasopharyngeal and pharyngeal swabs
hCoV-19/Mexico/TAM-LANGEBIO_IMSS_1671/2021	EPI_ISL_2942764	26/06/21	North America / Mexico / Tamaulipas	Human	Female	53	Ambulatory	Nasopharyngeal and pharyngeal swabs
hCoV-19/Mexico/TLA-LANGEBIO_IMSS_1065/2021	EPI_ISL_2671723	17/05/21	North America / Mexico / Tlaxcala	Human	Male	22	Ambulatory	Nasopharyngeal and pharyngeal swabs
hCoV-19/Mexico/VER_IBT_IMSS_1010/2021	EPI_ISL_1811518	08/04/21	North America / Mexico / Veracruz	Human	Male	49	Hospitalized	Nasopharyngeal and oropharyngeal swabs
hCoV-19/Mexico/VER_IBT_IMSS_1146/2021	EPI_ISL_2391570	22/04/21	North America / Mexico / Veracruz	Human	Female	28	Ambulatory	Nasopharyngeal and pharyngeal swabs

hCoV-19/Mexico/VER_IBT_IMSS_1214/2021	EPI_ISL_2391651	29/04/21	North America / Mexico / Veracruz	Human	Male	54	Ambulatory	Nasopharyngeal and pharyngeal swabs
hCoV-19/Mexico/VER_IBT_IMSS_1326/2021	EPI_ISL_2391668	28/04/21	North America / Mexico / Veracruz	Human	Female	21	Ambulatory	Nasopharyngeal and pharyngeal swabs
hCoV-19/Mexico/VER_IBT_IMSS_1330/2021	EPI_ISL_2391554	28/04/21	North America / Mexico / Veracruz	Human	Male	41	Ambulatory	Nasopharyngeal and pharyngeal swabs
hCoV-19/Mexico/VER_IBT_IMSS_1331/2021	EPI_ISL_2391555	29/04/21	North America / Mexico / Veracruz	Human	Female	65	Ambulatory	Nasopharyngeal and pharyngeal swabs
hCoV-19/Mexico/VER_IBT_IMSS_1333/2021	EPI_ISL_2391694	27/04/21	North America / Mexico / Veracruz	Human	Male	68	Hospitalized	Nasopharyngeal and pharyngeal swabs
hCoV-19/Mexico/VER_IBT_IMSS_1650/2021	EPI_ISL_2681350	30/05/21	North America / Mexico / Veracruz	Human	Female	46	Hospitalized	Nasopharyngeal and pharyngeal swabs
hCoV-19/Mexico/VER_IBT_IMSS_1663/2021	EPI_ISL_2681147	28/05/21	North America / Mexico / Veracruz	Human	Male	55	Ambulatory	Nasopharyngeal and pharyngeal swabs
hCoV-19/Mexico/VER_IBT_IMSS_1664/2021	EPI_ISL_2681180	30/05/21	North America / Mexico / Veracruz	Human	Female	54	Ambulatory	Nasopharyngeal and pharyngeal swabs
hCoV-19/Mexico/VER_INER_IMSS_00966/2021	EPI_ISL_2091407	14/04/21	North America / Mexico / Veracruz	Human	Male	68	Hospitalized	Nasopharyngeal and oropharyngeal swabs
hCoV-19/Mexico/VER_LANGEBIO_IMSS_0428/2021	EPI_ISL_1662210	23/03/21	North America / Mexico / Veracruz	Human	Male	43	Ambulatory	Nasopharyngeal swab
hCoV-19/Mexico/VER_LANGEBIO_IMSS_11722-NC/2	EPI_ISL_2970055	18/05/21	North America / Mexico / Veracruz	Human	Male	55	Hospitalized	Nasopharyngeal and pharyngeal swabs
hCoV-19/Mexico/VER-IBT_IMSS_1892/2021	EPI_ISL_2801760	30/05/21	North America / Mexico / Veracruz	Human	Female	70	Ambulatory	Nasopharyngeal and pharyngeal swabs
hCoV-19/Mexico/VER-IBT_IMSS_1923/2021	EPI_ISL_2801788	01/06/21	North America / Mexico / Veracruz	Human	Female	59	Hospitalized	Nasopharyngeal and pharyngeal swabs
hCoV-19/Mexico/VER-IBT_IMSS_1983/2021	EPI_ISL_2801841	04/06/21	North America / Mexico / Veracruz	Human	Male	28	Ambulatory	Nasopharyngeal and pharyngeal swabs
hCoV-19/Mexico/VER-IBT_IMSS_1990/2021	EPI_ISL_2801847	04/06/21	North America / Mexico / Veracruz	Human	Female	44	Ambulatory	Nasopharyngeal and pharyngeal swabs
hCoV-19/Mexico/VER-IBT_IMSS_1998/2021	EPI_ISL_2801851	06/06/21	North America / Mexico / Veracruz	Human	Female	48	Ambulatory	Nasopharyngeal and pharyngeal swabs
hCoV-19/Mexico/VER-IBT_IMSS_2043/2021	EPI_ISL_2801887	08/06/21	North America / Mexico / Veracruz	Human	Female	57	Ambulatory	Nasopharyngeal and pharyngeal swabs
hCoV-19/Mexico/VER-LANGEBIO_IMSS_1103/2021	EPI_ISL_2671737	18/05/21	North America / Mexico / Veracruz	Human	Male	38	Hospitalized	Nasopharyngeal and pharyngeal swabs
hCoV-19/Mexico/VER-LANGEBIO_IMSS_1105/2021	EPI_ISL_2671739	18/05/21	North America / Mexico / Veracruz	Human	Female	38	Ambulatory	Nasopharyngeal and pharyngeal swabs
hCoV-19/Mexico/VER-LANGEBIO_IMSS_1134/2021	EPI_ISL_2671744	20/05/21	North America / Mexico / Veracruz	Human	Male	39	Ambulatory	Nasopharyngeal and pharyngeal swabs
hCoV-19/Mexico/VER-LANGEBIO_IMSS_1424/2021	EPI_ISL_2942548	18/06/21	North America / Mexico / Veracruz	Human	Male	42	Ambulatory	Nasopharyngeal and pharyngeal swabs
hCoV-19/Mexico/VER-LANGEBIO_IMSS_1426/2021	EPI_ISL_2942550	20/06/21	North America / Mexico / Veracruz	Human	Male	40	Ambulatory	Nasopharyngeal swab
hCoV-19/Mexico/VER-LANGEBIO_IMSS_1428/2021	EPI_ISL_2942551	18/06/21	North America / Mexico / Veracruz	Human	Female	41	Ambulatory	Nasopharyngeal and pharyngeal swabs
hCoV-19/Mexico/VER-LANGEBIO_IMSS_1750/2021	EPI_ISL_2942831	24/06/21	North America / Mexico / Veracruz	Human	Male	33	Ambulatory	Nasopharyngeal and pharyngeal swabs
hCoV-19/Mexico/YUC_IBT_IMSS_10287-NC/2021	EPI_ISL_2681375	03/06/21	North America / Mexico / Yucatan	Human	Male	26	Ambulatory	Nasopharyngeal and pharyngeal swabs
hCoV-19/Mexico/YUC_IBT_IMSS_1346/2021	EPI_ISL_2391720	01/04/21	North America / Mexico / Yucatan	Human	Female	57	Hospitalized	Nasopharyngeal swab
hCoV-19/Mexico/YUC_IBT_IMSS_1727/2021	EPI_ISL_2681108	01/06/21	North America / Mexico / Yucatan	Human	Male	39	Ambulatory	Nasopharyngeal and pharyngeal swabs
hCoV-19/Mexico/YUC_IBT_IMSS_1748/2021	EPI_ISL_2681328	04/06/21	North America / Mexico / Yucatan	Human	Male	23	Ambulatory	Nasopharyngeal and pharyngeal swabs
hCoV-19/Mexico/YUC_INER_IMSS_00782/2021	EPI_ISL_2091236	08/04/21	North America / Mexico / Yucatan	Human	Male	31	Ambulatory	Nasopharyngeal and oropharyngeal swabs
hCoV-19/Mexico/YUC_INER_IMSS_1108/2021	EPI_ISL_2490344	29/04/21	North America / Mexico / Yucatan	Human	Male	37	Ambulatory	Nasopharyngeal and oropharyngeal swabs
hCoV-19/Mexico/YUC_LANGEBIO_IMSS_0596/2021	EPI_ISL_2402015	15/04/21	North America / Mexico / Yucatan	Human	Female	39	Ambulatory	Nasopharyngeal and pharyngeal swabs
hCoV-19/Mexico/YUC-IBT_IMSS_1845/2021	EPI_ISL_2801564	04/06/21	North America / Mexico / Yucatan	Human	Female	62	Hospitalized	Nasopharyngeal and pharyngeal swabs
hCoV-19/Mexico/YUC-IBT_IMSS_2008/2021	EPI_ISL_2801598	05/06/21	North America / Mexico / Yucatan	Human	Female	64	Hospitalized	Nasopharyngeal swab
hCoV-19/Mexico/YUC-IBT_IMSS_2027/2021	EPI_ISL_2801600	05/06/21	North America / Mexico / Yucatan	Human	Male	70	Hospitalized	Nasopharyngeal swab
hCoV-19/Mexico/YUC-IBT_IMSS_2048/2021	EPI_ISL_2801601	05/06/21	North America / Mexico / Yucatan	Human	Female	56	Hospitalized	Nasopharyngeal and pharyngeal swabs
hCoV-19/Mexico/YUC-IBT_IMSS_2049/2021	EPI_ISL_2801602	06/06/21	North America / Mexico / Yucatan	Human	Male	90	Hospitalized	Nasopharyngeal and pharyngeal swabs
hCoV-19/Mexico/YUC-LANGEBIO_IMSS_0942/2021	EPI_ISL_2671750	24/05/21	North America / Mexico / Yucatan	Human	Male	26	Ambulatory	Nasopharyngeal and pharyngeal swabs
hCoV-19/Mexico/YUC-LANGEBIO_IMSS_0964/2021	EPI_ISL_2671754	26/05/21	North America / Mexico / Yucatan	Human	Male	45	Ambulatory	Nasopharyngeal and pharyngeal swabs
hCoV-19/Mexico/YUC-LANGEBIO_IMSS_1240/2021	EPI_ISL_2942401	23/06/21	North America / Mexico / Yucatan	Human	Male	35	Ambulatory	Nasopharyngeal and pharyngeal swabs
hCoV-19/Mexico/YUC-LANGEBIO_IMSS_1245/2021	EPI_ISL_2942406	24/06/21	North America / Mexico / Yucatan	Human	Male	53	Ambulatory	Nasopharyngeal swab
hCoV-19/Mexico/YUC-LANGEBIO_IMSS_1562/2021	EPI_ISL_2942673	28/06/21	North America / Mexico / Yucatan	Human	Male	20	Ambulatory	Nasopharyngeal and pharyngeal swabs
hCoV-19/Mexico/YUC-LANGEBIO_IMSS_1586/2021	EPI_ISL_2942697	30/06/21	North America / Mexico / Yucatan	Human	Female	23	Ambulatory	Nasopharyngeal swab
hCoV-19/Mexico/ZAC_IBT_IMSS_1462/2021	EPI_ISL_2681120	25/05/21	North America / Mexico / Zacatecas	Human	Male	63	Ambulatory	Nasopharyngeal and pharyngeal swabs
hCoV-19/Mexico/ZAC_IBT_IMSS_1470/2021	EPI_ISL_2681121	26/05/21	North America / Mexico / Zacatecas	Human	Female	20	Ambulatory	Nasopharyngeal and pharyngeal swabs
hCoV-19/Mexico/ZAC_IBT_IMSS_1472/2021	EPI_ISL_2681092	28/05/21	North America / Mexico / Zacatecas	Human	Male	45	Ambulatory	Nasopharyngeal and pharyngeal swabs
hCoV-19/Mexico/ZAC_IBT_IMSS_1480/2021	EPI_ISL_2681142	31/05/21	North America / Mexico / Zacatecas	Human	Female	22	Ambulatory	Nasopharyngeal and pharyngeal swabs
hCoV-19/Mexico/ZAC_IBT_IMSS_1483/2021	EPI_ISL_2681190	31/05/21	North America / Mexico / Zacatecas	Human	Female	78	Ambulatory	Nasopharyngeal and pharyngeal swabs
hCoV-19/Mexico/ZAC_INER_IMSS_1210/2021	EPI_ISL_2835939	07/05/21	North America / Mexico / Zacatecas	Human	Female	45	Ambulatory	Nasopharyngeal and oropharyngeal swabs
hCoV-19/Mexico/ZAC_INER_IMSS_1217/2021	EPI_ISL_2835941	08/05/21	North America / Mexico / Zacatecas	Human	Female	20	Ambulatory	Nasopharyngeal and oropharyngeal swabs
hCoV-19/Mexico/ZAC_INER_IMSS_1225/2021	EPI_ISL_2835942	11/05/21	North America / Mexico / Zacatecas	Human	Male	45	Ambulatory	Nasopharyngeal and oropharyngeal swabs
hCoV-19/Mexico/ZAC_INER_IMSS_1230/2021	EPI_ISL_2490333	12/05/21	North America / Mexico / Zacatecas	Human	Female	33	Ambulatory	Nasopharyngeal and oropharyngeal swabs
hCoV-19/Mexico/ZAC_INER_IMSS_1235/2021	EPI_ISL_2835943	07/05/21	North America / Mexico / Zacatecas	Human	Male	39	Ambulatory	Nasopharyngeal and oropharyngeal swabs

hCoV-19/Mexico/ZAC_INER_IMSS_1237/2021	EPI_ISL_2490348	13/05/21	North America / Mexico / Zacatecas	Human	Female	33	Ambulatory	Nasopharyngeal and oropharyngeal swabs
hCoV-19/Mexico/ZAC_INER_IMSS_1244/2021	EPI_ISL_2835945	15/05/21	North America / Mexico / Zacatecas	Human	Male	24	Ambulatory	Nasopharyngeal and oropharyngeal swabs
hCoV-19/Mexico/ZAC_LANGEBIO_IMSS_0301/2021	EPI_ISL_1662226	28/03/21	North America / Mexico / Zacatecas	Human	Male	39	Ambulatory	Nasopharyngeal swab
hCoV-19/Mexico/ZAC_LANGEBIO_IMSS_0305/2021	EPI_ISL_1662230	30/03/21	North America / Mexico / Zacatecas	Human	Female	55	Ambulatory	Nasopharyngeal swab
hCoV-19/Mexico/ZAC_LANGEBIO_IMSS_0704/2021	EPI_ISL_2402118	29/04/21	North America / Mexico / Zacatecas	Human	Female	50	Hospitalized	Nasopharyngeal and pharyngeal swabs
hCoV-19/Mexico/ZAC-IBT_IMSS_11154-NC/2021	EPI_ISL_2801703	09/06/21	North America / Mexico / Zacatecas	Human	Female	19	Ambulatory	Nasopharyngeal and pharyngeal swabs
hCoV-19/Mexico/ZAC-IBT_IMSS_1821/2021	EPI_ISL_2801697	06/06/21	North America / Mexico / Zacatecas	Human	Female	36	Ambulatory	Nasopharyngeal and pharyngeal swabs
hCoV-19/Mexico/ZAC-IBT_IMSS_1826/2021	EPI_ISL_2801701	08/06/21	North America / Mexico / Zacatecas	Human	Male	29	Ambulatory	Nasopharyngeal and pharyngeal swabs
hCoV-19/Mexico/ZAC-IBT_IMSS_1827/2021	EPI_ISL_2801702	08/06/21	North America / Mexico / Zacatecas	Human	Male	47	Ambulatory	Nasopharyngeal and pharyngeal swabs
hCoV-19/Mexico/ZAC-IBT_IMSS_1831/2021	EPI_ISL_2801706	13/06/21	North America / Mexico / Zacatecas	Human	Male	47	Hospitalized	Nasopharyngeal and pharyngeal swabs
hCoV-19/Mexico/ZAC-LANGEBIO_IMSS_0977/2021	EPI_ISL_2671760	17/05/21	North America / Mexico / Zacatecas	Human	Female	52	Ambulatory	Nasopharyngeal and pharyngeal swabs
hCoV-19/Mexico/ZAC-INER-IMSS-00023/2021	EPI_ISL_1287761	10/02/21	North America / Mexico / Zacatecas	Human	Female	59	Ambulatory	Nasopharyngeal and oropharyngeal swabs
hCoV-19/Mexico/ZAC-INER-IMSS-00026/2021	EPI_ISL_1279265	11/02/21	North America / Mexico / Zacatecas	Human	Female	39	Ambulatory	Nasopharyngeal and oropharyngeal swabs
hCoV-19/Mexico/ZAC-INER-IMSS-00050/2021	EPI_ISL_1287762	14/02/21	North America / Mexico / Zacatecas	Human	Female	92	Ambulatory	Nasopharyngeal and oropharyngeal swabs
hCoV-19/Mexico/ZAC-INER-IMSS-00059/2021	EPI_ISL_1287763	15/02/21	North America / Mexico / Zacatecas	Human	Male	41	Ambulatory	Nasopharyngeal and oropharyngeal swabs
hCoV-19/Mexico/ZAC-INER-IMSS-00078/2021	EPI_ISL_1287764	16/02/21	North America / Mexico / Zacatecas	Human	Female	66	Ambulatory	Nasopharyngeal and oropharyngeal swabs
hCoV-19/Mexico/ZAC-INER-IMSS-00079/2021	EPI_ISL_1287765	16/02/21	North America / Mexico / Zacatecas	Human	Male	53	Ambulatory	Nasopharyngeal and oropharyngeal swabs
hCoV-19/Mexico/ZAC-INER-IMSS-00086/2021	EPI_ISL_1287767	17/02/21	North America / Mexico / Zacatecas	Human	Female	38	Ambulatory	Nasopharyngeal and oropharyngeal swabs
hCoV-19/Mexico/BCN-CICESE-M1409/2021	EPI_ISL_2455241	29/04/21	North America / Mexico / Baja California	Human	Male	68	unknown	Oropharyngeal swab
hCoV-19/Mexico/BCN-CICESE-M1411/2021	EPI_ISL_2455242	30/04/21	North America / Mexico / Baja California	Human	Female	35	unknown	Oropharyngeal swab
hCoV-19/Mexico/BCN-InDRE_FB13738_S1741/2021	EPI_ISL_2101880	19/04/21	North America / Mexico / Baja California	Human	Female	93	Deceased	Oropharyngeal swab
hCoV-19/Mexico/BCN-InDRE_FB16371_S2477/2021	EPI_ISL_2455998	17/05/21	North America / Mexico / Baja California	Human	Female	67	unknown	Oropharyngeal swab
hCoV-19/Mexico/BCN-InDRE_FB16375_S2478/2021	EPI_ISL_2455999	19/05/21	North America / Mexico / Baja California	Human	Female	28	unknown	Oropharyngeal swab
hCoV-19/Mexico/BCN-InDRE_FB16376_S2479/2021	EPI_ISL_2456000	18/05/21	North America / Mexico / Baja California	Human	Female	67	unknown	Oropharyngeal swab
hCoV-19/Mexico/BCN-InDRE_FB17815_S4114/2021	EPI_ISL_2920685	11/06/21	North America / Mexico / Baja California	Human	Female	19	Hospitalized	Oropharyngeal swab
hCoV-19/Mexico/BCN-InDRE_FB18286_S3966/2021	EPI_ISL_2858980	18/06/21	North America / Mexico / Baja California	Human	Male	38	Hospitalized	Oropharyngeal swab
hCoV-19/Mexico/BCN-InDRE_FB19191_S4500/2021	EPI_ISL_3033354	28/06/21	North America / Mexico / Baja California	Human	Female	32	Hospitalized	Oropharyngeal swab
hCoV-19/Mexico/BCS-InDRE_FB17119_S3086/2021	EPI_ISL_2663353	01/06/21	rth America / Mexico / Baja California S	Human	Female	35	Hospitalized	Oropharyngeal swab
hCoV-19/Mexico/BCS-InDRE_FB19249_S4501/2021	EPI_ISL_3033355	21/06/21	rth America / Mexico / Baja California S	Human	Female	49	Hospitalized	Oropharyngeal swab
hCoV-19/Mexico/BCS-InDRE_FB19255_S4502/2021	EPI_ISL_3033356	23/06/21	rth America / Mexico / Baja California S	Human	Male	71	Hospitalized	Oropharyngeal swab
hCoV-19/Mexico/CAM-InDRE_FB14881_S2388/2021	EPI_ISL_2455913	10/05/21	North America / Mexico / Campeche	Human	Female	9	unknown	Oropharyngeal swab
hCoV-19/Mexico/CAM-InDRE_FB14886_S2047/2021	EPI_ISL_2283689	05/05/21	North America / Mexico / Campeche	Human	Male	24	Hospitalized	Oropharyngeal swab
hCoV-19/Mexico/CAM-InDRE_FB17371_S3924/2021	EPI_ISL_2858938	09/06/21	North America / Mexico / Campeche	Human	Male	65	Hospitalized	Oropharyngeal swab
hCoV-19/Mexico/CAM-InDRE_FB17391_S3486/2021	EPI_ISL_2779001	02/06/21	North America / Mexico / Campeche	Human	Male	63	Hospitalized	Oropharyngeal swab
hCoV-19/Mexico/CAM-InDRE_FB18927_S4478/2021	EPI_ISL_3033332	16/06/21	North America / Mexico / Campeche	Human	Female	60	Hospitalized	Oropharyngeal swab
hCoV-19/Mexico/CAM-InDRE_FB18929_S4479/2021	EPI_ISL_3033333	18/06/21	North America / Mexico / Campeche	Human	Female	60	Hospitalized	Oropharyngeal swab
hCoV-19/Mexico/CAM-InDRE_FB18939_S4480/2021	EPI_ISL_3033334	25/06/21	North America / Mexico / Campeche	Human	Female	31	Hospitalized	Oropharyngeal swab
hCoV-19/Mexico/CHH-InDRE_F11326_S902/2021	EPI_ISL_1423512	02/03/21	North America / Mexico / Chihuahua	Human	Male	41	Hospitalized	Oropharyngeal swab
hCoV-19/Mexico/CHH-InDRE_F11642_S896/2021	EPI_ISL_1423506	08/03/21	North America / Mexico / Chihuahua	Human	Male	81	Hospitalized	Oropharyngeal swab
hCoV-19/Mexico/CHH-InDRE_F11646_S897/2021	EPI_ISL_1423507	07/03/21	North America / Mexico / Chihuahua	Human	Female	54	Hospitalized	Oropharyngeal swab
hCoV-19/Mexico/CHH-InDRE_F11654_S898/2021	EPI_ISL_1423508	09/03/21	North America / Mexico / Chihuahua	Human	Female	47	Hospitalized	Oropharyngeal swab
hCoV-19/Mexico/CHH-InDRE_F11656_S899/2021	EPI_ISL_1423509	08/03/21	North America / Mexico / Chihuahua	Human	Male	60	Hospitalized	Oropharyngeal swab
hCoV-19/Mexico/CHH-InDRE_F12155_S1187/2021	EPI_ISL_1626794	12/03/21	North America / Mexico / Chihuahua	Human	Male	63	Hospitalized	Oropharyngeal swab
hCoV-19/Mexico/CHH-InDRE_F12156_S1188/2021	EPI_ISL_1626795	15/03/21	North America / Mexico / Chihuahua	Human	Female	82	Hospitalized	Oropharyngeal swab
hCoV-19/Mexico/CHH-InDRE_F12158_S1189/2021	EPI_ISL_1626796	17/03/21	North America / Mexico / Chihuahua	Human	Female	54	Hospitalized	Oropharyngeal swab
hCoV-19/Mexico/CHH-InDRE_F12160_S1190/2021	EPI_ISL_1626797	17/03/21	North America / Mexico / Chihuahua	Human	Female	40	Hospitalized	Oropharyngeal swab
hCoV-19/Mexico/CHH-InDRE_F12161_S1191/2021	EPI_ISL_1626798	17/03/21	North America / Mexico / Chihuahua	Human	Female	48	unknown	Oropharyngeal swab
hCoV-19/Mexico/CHH-InDRE_F12165_S1192/2021	EPI_ISL_1626799	17/03/21	North America / Mexico / Chihuahua	Human	Male	59	Hospitalized	Oropharyngeal swab
hCoV-19/Mexico/CHH-InDRE_F12168_S1193/2021	EPI_ISL_1626800	17/03/21	North America / Mexico / Chihuahua	Human	Female	41	Hospitalized	Oropharyngeal swab
hCoV-19/Mexico/CHH-InDRE_F12169_S1194/2021	EPI_ISL_1626801	17/03/21	North America / Mexico / Chihuahua	Human	Female	68	Hospitalized	Oropharyngeal swab
hCoV-19/Mexico/CHH-InDRE_F12356_S1196/2021	EPI_ISL_1626803	22/03/21	North America / Mexico / Chihuahua	Human	Male	59	Hospitalized	Oropharyngeal swab
hCoV-19/Mexico/CHH-InDRE_F12359_S1197/2021	EPI_ISL_1626804	22/03/21	North America / Mexico / Chihuahua	Human	Male	40	Hospitalized	Oropharyngeal swab

hCoV-19/Mexico/CHH-InDRE_F12366_S1198/2021	EPI_ISL_1626805	23/03/21	North America / Mexico / Chihuahua	Human	Male	38	Hospitalized	Oropharyngeal swab
hCoV-19/Mexico/CHH-InDRE_FB12868_S1488/2021	EPI_ISL_1805466	30/03/21	North America / Mexico / Chihuahua	Human	Female	19	Hospitalized	Oropharyngeal swab
hCoV-19/Mexico/CHH-InDRE_FB12871_S1489/2021	EPI_ISL_1805467	02/04/21	North America / Mexico / Chihuahua	Human	Female	41	Hospitalized	Oropharyngeal swab
hCoV-19/Mexico/CHH-InDRE_FB12877_S1490/2021	EPI_ISL_1805468	04/04/21	North America / Mexico / Chihuahua	Human	Male	22	Hospitalized	Oropharyngeal swab
hCoV-19/Mexico/CHH-InDRE_FB12878_S1491/2021	EPI_ISL_1805469	02/04/21	North America / Mexico / Chihuahua	Human	Male	37	Hospitalized	Oropharyngeal swab
hCoV-19/Mexico/CHH-InDRE_FB13242_S1496/2021	EPI_ISL_1805474	05/04/21	North America / Mexico / Chihuahua	Human	Female	70	Hospitalized	Oropharyngeal swab
hCoV-19/Mexico/CHH-InDRE_FB13243_S1497/2021	EPI_ISL_1805475	05/04/21	North America / Mexico / Chihuahua	Human	Male	49	Hospitalized	Oropharyngeal swab
hCoV-19/Mexico/CHH-InDRE_FB13246_S1498/2021	EPI_ISL_1805476	06/04/21	North America / Mexico / Chihuahua	Human	Female	44	unknown	Oropharyngeal swab
hCoV-19/Mexico/CHH-InDRE_FB13247_S1499/2021	EPI_ISL_1805477	06/04/21	North America / Mexico / Chihuahua	Human	Male	30	Hospitalized	Oropharyngeal swab
hCoV-19/Mexico/CHH-InDRE_FB13252_S1500/2021	EPI_ISL_1805478	09/04/21	North America / Mexico / Chihuahua	Human	Male	68	unknown	Oropharyngeal swab
hCoV-19/Mexico/CHH-InDRE_FB13253_S1501/2021	EPI_ISL_1805479	09/04/21	North America / Mexico / Chihuahua	Human	Male	73	unknown	Oropharyngeal swab
hCoV-19/Mexico/CHH-InDRE_FB13662_S1732/2021	EPI_ISL_2101871	13/04/21	North America / Mexico / Chihuahua	Human	Female	29	Hospitalized	Oropharyngeal swab
hCoV-19/Mexico/CHH-InDRE_FB13663_S1733/2021	EPI_ISL_2101872	13/04/21	North America / Mexico / Chihuahua	Human	Male	45	Hospitalized	Oropharyngeal swab
hCoV-19/Mexico/CHH-InDRE_FB13668_S1734/2021	EPI_ISL_2101873	14/04/21	North America / Mexico / Chihuahua	Human	Female	57	Hospitalized	Oropharyngeal swab
hCoV-19/Mexico/CHH-InDRE_FB13671_S1735/2021	EPI_ISL_2101874	14/04/21	North America / Mexico / Chihuahua	Human	Female	46	unknown	Oropharyngeal swab
hCoV-19/Mexico/CHH-InDRE_FB13672_S1736/2021	EPI_ISL_2101875	14/04/21	North America / Mexico / Chihuahua	Human	Female	24	Hospitalized	Oropharyngeal swab
hCoV-19/Mexico/CHH-InDRE_FB13678_S1751/2021	EPI_ISL_2101890	16/04/21	North America / Mexico / Chihuahua	Human	Female	27	Hospitalized	Oropharyngeal swab
hCoV-19/Mexico/CHH-InDRE_FB13679_S1737/2021	EPI_ISL_2101876	18/04/21	North America / Mexico / Chihuahua	Human	Female	64	Hospitalized	Oropharyngeal swab
hCoV-19/Mexico/CHH-InDRE_FB13688_S1738/2021	EPI_ISL_2101877	08/04/21	North America / Mexico / Chihuahua	Human	Female	27	Hospitalized	Oropharyngeal swab
hCoV-19/Mexico/CHH-InDRE_FB13702_S1739/2021	EPI_ISL_2101878	15/04/21	North America / Mexico / Chihuahua	Human	Female	31	unknown	Oropharyngeal swab
hCoV-19/Mexico/CHH-InDRE_FB13814_S1742/2021	EPI_ISL_2101881	20/04/21	North America / Mexico / Chihuahua	Human	Female	42	Hospitalized	Oropharyngeal swab
hCoV-19/Mexico/CHH-InDRE_FB13819_S1743/2021	EPI_ISL_2101882	21/04/21	North America / Mexico / Chihuahua	Human	Female	55	Hospitalized	Oropharyngeal swab
hCoV-19/Mexico/CHH-InDRE_FB13820_S1744/2021	EPI_ISL_2101883	21/04/21	North America / Mexico / Chihuahua	Human	Female	28	Hospitalized	Oropharyngeal swab
hCoV-19/Mexico/CHH-InDRE_FB13822_S1745/2021	EPI_ISL_2101884	20/04/21	North America / Mexico / Chihuahua	Human	Female	78	Hospitalized	Oropharyngeal swab
hCoV-19/Mexico/CHH-InDRE_FB13825_S1746/2021	EPI_ISL_2101885	21/04/21	North America / Mexico / Chihuahua	Human	Female	65	Hospitalized	Oropharyngeal swab
hCoV-19/Mexico/CHH-InDRE_FB13827_S1747/2021	EPI_ISL_2101886	21/04/21	North America / Mexico / Chihuahua	Human	Male	16	Hospitalized	Oropharyngeal swab
hCoV-19/Mexico/CHH-InDRE_FB13832_S1748/2021	EPI_ISL_2101887	21/04/21	North America / Mexico / Chihuahua	Human	Male	85	Hospitalized	Oropharyngeal swab
hCoV-19/Mexico/CHH-InDRE_FB13835_S1749/2021	EPI_ISL_2101888	22/04/21	North America / Mexico / Chihuahua	Human	Female	53	Hospitalized	Oropharyngeal swab
hCoV-19/Mexico/CHH-InDRE_FB13840_S1750/2021	EPI_ISL_2101889	20/04/21	North America / Mexico / Chihuahua	Human	Female	48	unknown	Oropharyngeal swab
hCoV-19/Mexico/CHH-InDRE_FB14222_S1764/2021	EPI_ISL_2101903	26/04/21	North America / Mexico / Chihuahua	Human	Male	77	unknown	Oropharyngeal swab
hCoV-19/Mexico/CHH-InDRE_FB14223_S1765/2021	EPI_ISL_2101904	26/04/21	North America / Mexico / Chihuahua	Human	Female	55	unknown	Oropharyngeal swab
hCoV-19/Mexico/CHH-InDRE_FB14877_S2086/2021	EPI_ISL_2283725	05/04/21	North America / Mexico / Chihuahua	Human	Male	24	Hospitalized	Oropharyngeal swab
hCoV-19/Mexico/CHH-InDRE_FB14878_S2389/2021	EPI_ISL_2455914	05/05/21	North America / Mexico / Chihuahua	Human	Female	24	Hospitalized	Oropharyngeal swab
hCoV-19/Mexico/CHH-InDRE_FB14879_S2392/2021	EPI_ISL_2455917	07/05/21	North America / Mexico / Chihuahua	Human	Male	33	Hospitalized	Oropharyngeal swab
hCoV-19/Mexico/CHH-InDRE_FB14880_S2401/2021	EPI_ISL_2455926	09/05/21	North America / Mexico / Chihuahua	Human	Female	53	unknown	Oropharyngeal swab
hCoV-19/Mexico/CHH-InDRE_FB14901_S2402/2021	EPI_ISL_2455927	05/05/21	North America / Mexico / Chihuahua	Human	Female	62	Hospitalized	Oropharyngeal swab
hCoV-19/Mexico/CHH-InDRE_FB14906_S2403/2021	EPI_ISL_2455928	05/05/21	North America / Mexico / Chihuahua	Human	Male	40	Hospitalized	Oropharyngeal swab
hCoV-19/Mexico/CHH-InDRE_FB14908_S2404/2021	EPI_ISL_2484164	05/05/21	North America / Mexico / Chihuahua	Human	Male	45	Hospitalized	Oropharyngeal swab
hCoV-19/Mexico/CHH-InDRE_FB14909_S2405/2021	EPI_ISL_2455929	05/05/21	North America / Mexico / Chihuahua	Human	Female	58	Hospitalized	Oropharyngeal swab
hCoV-19/Mexico/CHH-InDRE_FB14913_S2390/2021	EPI_ISL_2455915	05/05/21	North America / Mexico / Chihuahua	Human	Female	32	Hospitalized	Oropharyngeal swab
hCoV-19/Mexico/CHH-InDRE_FB14915_S2049/2021	EPI_ISL_2283691	02/05/21	North America / Mexico / Chihuahua	Human	Female	58	Hospitalized	Oropharyngeal swab
hCoV-19/Mexico/CHH-InDRE_FB14918_S2050/2021	EPI_ISL_2283692	05/05/21	North America / Mexico / Chihuahua	Human	Male	80	Deceased	Oropharyngeal swab
hCoV-19/Mexico/CHH-InDRE_FB15893_S2394/2021	EPI_ISL_2455919	11/05/21	North America / Mexico / Chihuahua	Human	Male	49	Hospitalized	Oropharyngeal swab
hCoV-19/Mexico/CHH-InDRE_FB15895_S2395/2021	EPI_ISL_2455920	13/05/21	North America / Mexico / Chihuahua	Human	Female	22	Hospitalized	Oropharyngeal swab
hCoV-19/Mexico/CHH-InDRE_FB16251_S2733/2021	EPI_ISL_2533759	18/05/21	North America / Mexico / Chihuahua	Human	Male	17	Hospitalized	Oropharyngeal swab
hCoV-19/Mexico/CHH-InDRE_FB16252_S2734/2021	EPI_ISL_2533760	18/05/21	North America / Mexico / Chihuahua	Human	Male	49	Hospitalized	Oropharyngeal swab
hCoV-19/Mexico/CHH-InDRE_FB16254_S2735/2021	EPI_ISL_2533761	18/05/21	North America / Mexico / Chihuahua	Human	Male	49	Hospitalized	Oropharyngeal swab
hCoV-19/Mexico/CHH-InDRE_FB16255_S2736/2021	EPI_ISL_2533762	19/05/21	North America / Mexico / Chihuahua	Human	Male	18	Hospitalized	Oropharyngeal swab
hCoV-19/Mexico/CHH-InDRE_FB16972_S3127/2021	EPI_ISL_2663394	25/05/21	North America / Mexico / Chihuahua	Human	Male	59	Hospitalized	Oropharyngeal swab
hCoV-19/Mexico/CHH-InDRE_FB16976_S3128/2021	EPI_ISL_2663395	28/05/21	North America / Mexico / Chihuahua	Human	Male	38	Hospitalized	Oropharyngeal swab
hCoV-19/Mexico/CHH-InDRE_FB16978_S3129/2021	EPI_ISL_2663396	29/05/21	North America / Mexico / Chihuahua	Human	Male	53	Hospitalized	Oropharyngeal swab
hCoV-19/Mexico/CHH-InDRE_FB16984_S3130/2021	EPI_ISL_2663397	04/06/21	North America / Mexico / Chihuahua	Human	Male	24	Hospitalized	Oropharyngeal swab

hCoV-19/Mexico/CHH-InDRE_FB16985_S3131/2021	EPI_ISL_2663398	05/06/21	North America / Mexico / Chihuahua	Human	Male	58	Deceased	Oropharyngeal swab
hCoV-19/Mexico/CHH-InDRE_FB16989_S3940/2021	EPI_ISL_2858954	26/05/21	North America / Mexico / Chihuahua	Human	Male	43	Hospitalized	Oropharyngeal swab
hCoV-19/Mexico/CHH-InDRE_FB16992_S3941/2021	EPI_ISL_2858955	31/05/21	North America / Mexico / Chihuahua	Human	Female	47	Hospitalized	Oropharyngeal swab
hCoV-19/Mexico/CHH-InDRE_FB16993_S3942/2021	EPI_ISL_2858956	29/05/21	North America / Mexico / Chihuahua	Human	Female	49	Hospitalized	Oropharyngeal swab
hCoV-19/Mexico/CHH-InDRE_FB17409_S3925/2021	EPI_ISL_2858939	10/06/21	North America / Mexico / Chihuahua	Human	Male	41	Hospitalized	Oropharyngeal swab
hCoV-19/Mexico/CHH-InDRE_FB17410_S3926/2021	EPI_ISL_2858940	11/06/21	North America / Mexico / Chihuahua	Human	Male	63	Hospitalized	Oropharyngeal swab
hCoV-19/Mexico/CHH-InDRE_FB18221_S4131/2021	EPI_ISL_2920702	11/06/21	North America / Mexico / Chihuahua	Human	Female	38	Hospitalized	Oropharyngeal swab
hCoV-19/Mexico/CHH-InDRE_FB18222_S4132/2021	EPI_ISL_2920703	09/06/21	North America / Mexico / Chihuahua	Human	Female	36	Hospitalized	Oropharyngeal swab
hCoV-19/Mexico/CHH-InDRE_FB18224_S4133/2021	EPI_ISL_2920704	16/06/21	North America / Mexico / Chihuahua	Human	Male	69	Hospitalized	Oropharyngeal swab
hCoV-19/Mexico/CHH-InDRE_FB18225_S3965/2021	EPI_ISL_2858979	11/06/21	North America / Mexico / Chihuahua	Human	Male	59	Hospitalized	Oropharyngeal swab
hCoV-19/Mexico/CHH-INMEGEN-InDRE-04-04-126/20	EPI_ISL_1628624	05/04/21	North America / Mexico / Chihuahua	Human	Male	58	unknown	Oropharyngeal swab
hCoV-19/Mexico/CHP-InDRE_FB17387_S3482/2021	EPI_ISL_2778997	09/06/21	North America / Mexico / Chiapas	Human	Male	29	Hospitalized	Oropharyngeal swab
hCoV-19/Mexico/CMX-InDRE_F12295_S1200/2021	EPI_ISL_1626807	20/03/21	North America / Mexico / Mexico City	Human	Female	41	unknown	Oropharyngeal swab
hCoV-19/Mexico/CMX-InDRE_F34543_S900/2021	EPI_ISL_1423510	21/03/21	North America / Mexico / Mexico City	Human	Female	30	Hospitalized	Oropharyngeal swab
hCoV-19/Mexico/CMX-InDRE_FB13260_S1502/2021	EPI_ISL_1805480	10/04/21	North America / Mexico / Mexico City	Human	Female	56	Hospitalized	Oropharyngeal swab
hCoV-19/Mexico/CMX-InDRE_FB16175_S2435/2021	EPI_ISL_2455959	20/05/21	North America / Mexico / Mexico City	Human	Female	51	Hospitalized	Oropharyngeal swab
hCoV-19/Mexico/CMX-InDRE_FB16521_S3061/2021	EPI_ISL_2663328	25/05/21	North America / Mexico / Mexico City	Human	Male	38	Hospitalized	Oropharyngeal swab
hCoV-19/Mexico/CMX-InDRE_FB16522_S3062/2021	EPI_ISL_2663329	25/05/21	North America / Mexico / Mexico City	Human	Female	17	Hospitalized	Oropharyngeal swab
hCoV-19/Mexico/CMX-InDRE_FB16523_S3063/2021	EPI_ISL_2663330	31/05/21	North America / Mexico / Mexico City	Human	Female	40	Hospitalized	Oropharyngeal swab
hCoV-19/Mexico/CMX-InDRE_FB16969_S3126/2021	EPI_ISL_2663393	07/06/21	North America / Mexico / Mexico City	Human	Male	21	Hospitalized	Oropharyngeal swab
hCoV-19/Mexico/CMX-InDRE_FB17954_S3934/2021	EPI_ISL_2858948	08/06/21	North America / Mexico / Mexico City	Human	Male	63	Hospitalized	Oropharyngeal swab
hCoV-19/Mexico/CMX-InDRE_FB17958_S3935/2021	EPI_ISL_2858949	24/05/21	North America / Mexico / Mexico City	Human	Female	13	Hospitalized	Oropharyngeal swab
hCoV-19/Mexico/CMX-InDRE_FB17960_S3936/2021	EPI_ISL_2858950	26/05/21	North America / Mexico / Mexico City	Human	Female	58	Hospitalized	Oropharyngeal swab
hCoV-19/Mexico/CMX-InDRE_FB17970_S3937/2021	EPI_ISL_2858951	07/06/21	North America / Mexico / Mexico City	Human	Female	71	Hospitalized	Oropharyngeal swab
hCoV-19/Mexico/CMX-InDRE_FB19386_S4507/2021	EPI_ISL_3033361	14/06/21	North America / Mexico / Mexico City	Human	Female	68	Hospitalized	Oropharyngeal swab
hCoV-19/Mexico/CMX-InDRE_FB19393_S4508/2021	EPI_ISL_3046076	17/06/21	North America / Mexico / Mexico City	Human	Male	41	Hospitalized	Oropharyngeal swab
hCoV-19/Mexico/CMX-InDRE_FB19400_S4509/2021	EPI_ISL_3033362	18/06/21	North America / Mexico / Mexico City	Human	Female	47	Hospitalized	Oropharyngeal swab
hCoV-19/Mexico/CMX-InDRE_FD45086_S1508/2021	EPI_ISL_1805486	06/04/21	North America / Mexico / Mexico City	Human	Female	29	Hospitalized	Oropharyngeal swab
hCoV-19/Mexico/CMX-InDRE_FD49735_S1767/2021	EPI_ISL_2101906	12/04/21	North America / Mexico / Mexico City	Human	Female	32	Hospitalized	Oropharyngeal swab
hCoV-19/Mexico/CMX-InDRE_FD54664_S1768/2021	EPI_ISL_2101907	20/04/21	North America / Mexico / Mexico City	Human	Male	36	Hospitalized	Oropharyngeal swab
hCoV-19/Mexico/CMX-InDRE_FD54888_S1769/2021	EPI_ISL_2101908	19/04/21	North America / Mexico / Mexico City	Human	Female	59	Hospitalized	Oropharyngeal swab
hCoV-19/Mexico/CMX-InDRE_FD55350_S1770/2021	EPI_ISL_2101909	22/04/21	North America / Mexico / Mexico City	Human	Male	49	Hospitalized	Oropharyngeal swab
hCoV-19/Mexico/CMX-InDRE_FD57542_S1771/2021	EPI_ISL_2101910	26/04/21	North America / Mexico / Mexico City	Human	Female	53	Hospitalized	Oropharyngeal swab
hCoV-19/Mexico/CMX-InDRE_FD58317_S1772/2021	EPI_ISL_2101911	27/04/21	North America / Mexico / Mexico City	Human	Male	15	Hospitalized	Oropharyngeal swab
hCoV-19/Mexico/CMX-InDRE_FD58467_S1773/2021	EPI_ISL_2101912	27/04/21	North America / Mexico / Mexico City	Human	Male	46	Hospitalized	Oropharyngeal swab
hCoV-19/Mexico/CMX-InDRE_FD63274_S2065/2021	EPI_ISL_2283705	05/05/21	North America / Mexico / Mexico City	Human	Female	45	Hospitalized	Oropharyngeal swab
hCoV-19/Mexico/CMX-InDRE_FD63747_S2064/2021	EPI_ISL_2283704	11/03/21	North America / Mexico / Mexico City	Human	Male	53	Hospitalized	Oropharyngeal swab
hCoV-19/Mexico/CMX-InDRE_FD63907_S2067/2021	EPI_ISL_2283707	07/05/21	North America / Mexico / Mexico City	Human	Female	39	Hospitalized	Oropharyngeal swab
hCoV-19/Mexico/CMX-InDRE_FD64062_S2068/2021	EPI_ISL_2283708	07/05/21	North America / Mexico / Mexico City	Human	Female	55	Hospitalized	Oropharyngeal swab
hCoV-19/Mexico/CMX-InDRE_FD64177_S2066/2021	EPI_ISL_2283706	07/05/21	North America / Mexico / Mexico City	Human	Male	19	Hospitalized	Oropharyngeal swab
hCoV-19/Mexico/CMX-InDRE_FD64253_S2063/2021	EPI_ISL_2295589	05/03/21	North America / Mexico / Mexico City	Human	Female	22	Hospitalized	Oropharyngeal swab
hCoV-19/Mexico/CMX-InDRE_FD64705_S2062/2021	EPI_ISL_2295588	09/05/21	North America / Mexico / Mexico City	Human	Male	43	Hospitalized	Oropharyngeal swab
hCoV-19/Mexico/CMX-InDRE_FD69482_S2781/2021	EPI_ISL_2533807	17/05/21	North America / Mexico / Mexico City	Human	Male	31	Hospitalized	Oropharyngeal swab
hCoV-19/Mexico/CMX-InDRE_FD69743_S3075/2021	EPI_ISL_2663342	19/05/21	North America / Mexico / Mexico City	Human	Male	50	Hospitalized	Oropharyngeal swab
hCoV-19/Mexico/CMX-InDRE_FD71018_S2782/2021	EPI_ISL_2533808	19/05/21	North America / Mexico / Mexico City	Human	Female	45	Hospitalized	Oropharyngeal swab
hCoV-19/Mexico/CMX-InDRE_FD71149_S2784/2021	EPI_ISL_2533810	19/05/21	North America / Mexico / Mexico City	Human	Female	48	Hospitalized	Oropharyngeal swab
hCoV-19/Mexico/CMX-InDRE_FD71474_S2785/2021	EPI_ISL_2533811	20/05/21	North America / Mexico / Mexico City	Human	Female	40	Hospitalized	Oropharyngeal swab
hCoV-19/Mexico/CMX-InDRE_FD71481_S3076/2021	EPI_ISL_2663343	20/05/21	North America / Mexico / Mexico City	Human	Male	71	Hospitalized	Oropharyngeal swab
hCoV-19/Mexico/CMX-InDRE_FD71675_S3077/2021	EPI_ISL_2663344	20/05/21	North America / Mexico / Mexico City	Human	Male	59	Hospitalized	Oropharyngeal swab
hCoV-19/Mexico/CMX-InDRE_FD72271_S3091/2021	EPI_ISL_2663358	21/05/21	North America / Mexico / Mexico City	Human	Female	30	Hospitalized	Oropharyngeal swab
hCoV-19/Mexico/CMX-InDRE_FD72328_S2786/2021	EPI_ISL_2533812	21/05/21	North America / Mexico / Mexico City	Human	Female	53	Hospitalized	Oropharyngeal swab
hCoV-19/Mexico/CMX-InDRE_FD72355_S2787/2021	EPI_ISL_2533813	22/05/21	North America / Mexico / Mexico City	Human	Female	38	Hospitalized	Oropharyngeal swab

hCoV-19/Mexico/CMX-InDRE_FD72360_S2788/2021	EPI_ISL_2533814	22/05/21	North America / Mexico / Mexico City	Human	Male	38	Hospitalized	Oropharyngeal swab
hCoV-19/Mexico/CMX-InDRE_FD72362_S2789/2021	EPI_ISL_2533815	20/05/21	North America / Mexico / Mexico City	Human	Male	71	Hospitalized	Oropharyngeal swab
hCoV-19/Mexico/CMX-InDRE_FD77651_S4136/2021	EPI_ISL_2920707	23/06/21	North America / Mexico / Mexico City	Human	Female	23	Hospitalized	Oropharyngeal swab
hCoV-19/Mexico/CMX-InDRE_FD77860_S4137/2021	EPI_ISL_2920708	25/06/21	North America / Mexico / Mexico City	Human	Female	45	Hospitalized	Oropharyngeal swab
hCoV-19/Mexico/CMX-InDRE_FD77861_S4138/2021	EPI_ISL_2920709	25/06/21	North America / Mexico / Mexico City	Human	Female	40	Hospitalized	Oropharyngeal swab
hCoV-19/Mexico/CMX-InDRE_FD77862_S4139/2021	EPI_ISL_2920710	25/06/21	North America / Mexico / Mexico City	Human	Male	13	Hospitalized	Oropharyngeal swab
hCoV-19/Mexico/CMX-InDRE_FD77863_S4140/2021	EPI_ISL_2920711	25/06/21	North America / Mexico / Mexico City	Human	Male	18	Hospitalized	Oropharyngeal swab
hCoV-19/Mexico/CMX-InDRE_FD77882_S4141/2021	EPI_ISL_2920712	24/06/21	North America / Mexico / Mexico City	Human	Male	25	Hospitalized	Oropharyngeal swab
hCoV-19/Mexico/CMX-INER-INMEGEN-00136/2021	EPI_ISL_1824482	07/04/21	North America / Mexico / Mexico City	Human	Male	58	unknown	Oropharyngeal swab
hCoV-19/Mexico/CMX-INER-INMEGEN-00149/2021	EPI_ISL_1824495	09/04/21	North America / Mexico / Mexico City	Human	Male	49	unknown	Oropharyngeal swab
hCoV-19/Mexico/CMX-INER-INMEGEN-00159/2021	EPI_ISL_2160651	16/04/21	North America / Mexico / Mexico City	Human	Male	50	unknown	Oropharyngeal swab
hCoV-19/Mexico/CMX-INER-INMEGEN-00164/2021	EPI_ISL_2160659	16/04/21	North America / Mexico / Mexico City	Human	Female	58	unknown	Oropharyngeal swab
hCoV-19/Mexico/CMX-INER-INMEGEN-00167/2021	EPI_ISL_2160665	19/04/21	North America / Mexico / Mexico City	Human	Male	53	unknown	Oropharyngeal swab
hCoV-19/Mexico/CMX-INER-INMEGEN-00203/2021	EPI_ISL_2341315	15/04/21	North America / Mexico / Mexico City	Human	Male	25	unknown	Oropharyngeal swab
hCoV-19/Mexico/CMX-INER-INMEGEN-00227/2021	EPI_ISL_2646128	18/05/21	North America / Mexico / Mexico City	Human	Male	46	unknown	Oropharyngeal swab
hCoV-19/Mexico/CMX-INMEGEN-04-01-327/2021	EPI_ISL_1585917	29/03/21	North America / Mexico / Mexico City	Human	Male	49	unknown	Oropharyngeal swab
hCoV-19/Mexico/CMX-INMEGEN-04-04-55/2021	EPI_ISL_1626614	07/04/21	North America / Mexico / Mexico City	Human	Male	20	unknown	Oropharyngeal swab
hCoV-19/Mexico/CMX-INMEGEN-04-05-47/2021	EPI_ISL_1711297	13/04/21	North America / Mexico / Mexico City	Human	Female	26	unknown	Oropharyngeal swab
hCoV-19/Mexico/CMX-INMEGEN-04-05-49/2021	EPI_ISL_1711301	13/04/21	North America / Mexico / Mexico City	Human	Male	34	unknown	Oropharyngeal swab
hCoV-19/Mexico/CMX-INMEGEN-04-05-6/2021	EPI_ISL_1711300	12/04/21	North America / Mexico / Mexico City	Human	Female	38	unknown	Oropharyngeal swab
hCoV-19/Mexico/CMX-INMEGEN-04-05-63/2021	EPI_ISL_1711298	14/04/21	North America / Mexico / Mexico City	Human	Male	52	unknown	Oropharyngeal swab
hCoV-19/Mexico/CMX-INMEGEN-04-05-71/2021	EPI_ISL_1711302	15/04/21	North America / Mexico / Mexico City	Human	Female	45	unknown	Oropharyngeal swab
hCoV-19/Mexico/CMX-INMEGEN-04-05-72/2021	EPI_ISL_1711299	15/04/21	North America / Mexico / Mexico City	Human	Male	48	unknown	Oropharyngeal swab
hCoV-19/Mexico/CMX-INMEGEN-04-06-38/2021	EPI_ISL_1807344	21/04/21	North America / Mexico / Mexico City	Human	Female	54	unknown	Oropharyngeal swab
hCoV-19/Mexico/CMX-INMEGEN-04-06-73/2021	EPI_ISL_1807363	19/04/21	North America / Mexico / Mexico City	Human	Male	53	unknown	Oropharyngeal swab
hCoV-19/Mexico/CMX-INMEGEN-04-07-195/2021	EPI_ISL_2080627	23/04/21	North America / Mexico / Mexico City	Human	Female	57	unknown	Oropharyngeal swab
hCoV-19/Mexico/CMX-INMEGEN-04-07-230/2021	EPI_ISL_2080647	26/04/21	North America / Mexico / Mexico City	Human	Female	24	unknown	Oropharyngeal swab
hCoV-19/Mexico/CMX-INMEGEN-04-07-243/2021	EPI_ISL_2080658	26/04/21	North America / Mexico / Mexico City	Human	Female	54	unknown	Oropharyngeal swab
hCoV-19/Mexico/CMX-INMEGEN-04-07-292/2021	EPI_ISL_2080689	28/04/21	North America / Mexico / Mexico City	Human	Female	21	unknown	Oropharyngeal swab
hCoV-19/Mexico/CMX-INMEGEN-05-01-108/2021	EPI_ISL_2230864	28/04/21	North America / Mexico / Mexico City	Human	Female	41	unknown	Oropharyngeal swab
hCoV-19/Mexico/CMX-INMEGEN-05-01-177/2021	EPI_ISL_2230880	01/05/21	North America / Mexico / Mexico City	Human	Female	57	unknown	Oropharyngeal swab
hCoV-19/Mexico/CMX-INMEGEN-05-01-19/2021	EPI_ISL_2230820	06/05/21	North America / Mexico / Mexico City	Human	Male	55	unknown	Oropharyngeal swab
hCoV-19/Mexico/CMX-INMEGEN-05-01-192/2021	EPI_ISL_2105723	06/05/21	North America / Mexico / Mexico City	Human	Male	59	unknown	Oropharyngeal swab
hCoV-19/Mexico/CMX-INMEGEN-05-01-221/2021	EPI_ISL_2105741	06/05/21	North America / Mexico / Mexico City	Human	Male	22	unknown	Oropharyngeal swab
hCoV-19/Mexico/CMX-INMEGEN-05-01-226/2021	EPI_ISL_2105746	27/04/21	North America / Mexico / Mexico City	Human	Male	59	unknown	Oropharyngeal swab
hCoV-19/Mexico/CMX-INMEGEN-05-01-228/2021	EPI_ISL_2105748	27/04/21	North America / Mexico / Mexico City	Human	Female	31	unknown	Oropharyngeal swab
hCoV-19/Mexico/CMX-INMEGEN-05-01-229/2021	EPI_ISL_2105749	27/04/21	North America / Mexico / Mexico City	Human	Female	23	unknown	Oropharyngeal swab
hCoV-19/Mexico/CMX-INMEGEN-05-01-260/2021	EPI_ISL_2105773	28/04/21	North America / Mexico / Mexico City	Human	Male	51	unknown	Oropharyngeal swab
hCoV-19/Mexico/CMX-INMEGEN-05-01-262/2021	EPI_ISL_2105775	28/04/21	North America / Mexico / Mexico City	Human	Female	19	unknown	Oropharyngeal swab
hCoV-19/Mexico/CMX-INMEGEN-05-01-272/2021	EPI_ISL_2105784	28/04/21	North America / Mexico / Mexico City	Human	Female	11	unknown	Oropharyngeal swab
hCoV-19/Mexico/CMX-INMEGEN-05-01-301/2021	EPI_ISL_2105810	29/04/21	North America / Mexico / Mexico City	Human	Male	37	unknown	Oropharyngeal swab
hCoV-19/Mexico/CMX-INMEGEN-05-01-316/2021	EPI_ISL_2105824	01/05/21	North America / Mexico / Mexico City	Human	Female	63	unknown	Oropharyngeal swab
hCoV-19/Mexico/CMX-INMEGEN-05-01-319/2021	EPI_ISL_2105826	01/05/21	North America / Mexico / Mexico City	Human	Female	30	unknown	Oropharyngeal swab
hCoV-19/Mexico/CMX-INMEGEN-05-01-42/2021	EPI_ISL_2230892	03/05/21	North America / Mexico / Mexico City	Human	Male	20	unknown	Oropharyngeal swab
hCoV-19/Mexico/CMX-INMEGEN-05-01-74/2021	EPI_ISL_2277643	04/05/21	North America / Mexico / Mexico City	Human	Male	57	unknown	Oropharyngeal swab
hCoV-19/Mexico/CMX-INMEGEN-05-01-87/2021	EPI_ISL_2277644	06/05/21	North America / Mexico / Mexico City	Human	Female	48	unknown	Oropharyngeal swab
hCoV-19/Mexico/CMX-INMEGEN-05-01-98/2021	EPI_ISL_2230863	28/04/21	North America / Mexico / Mexico City	Human	Female	47	unknown	Oropharyngeal swab
hCoV-19/Mexico/CMX-INMEGEN-05-02-101/2021	EPI_ISL_2230870	29/04/21	North America / Mexico / Mexico City	Human	Male	20	unknown	Oropharyngeal swab
hCoV-19/Mexico/CMX-INMEGEN-05-02-107/2021	EPI_ISL_2881754	29/04/21	North America / Mexico / Mexico City	Human	Female	21	unknown	Oropharyngeal swab
hCoV-19/Mexico/CMX-INMEGEN-05-02-108/2021	EPI_ISL_2230830	29/04/21	North America / Mexico / Mexico City	Human	Female	29	unknown	Oropharyngeal swab
hCoV-19/Mexico/CMX-INMEGEN-05-02-109/2021	EPI_ISL_2230872	29/04/21	North America / Mexico / Mexico City	Human	Male	25	unknown	Oropharyngeal swab
hCoV-19/Mexico/CMX-INMEGEN-05-02-122/2021	EPI_ISL_2230874	29/04/21	North America / Mexico / Mexico City	Human	Male	25	unknown	Oropharyngeal swab

hCoV-19/Mexico/CMX-INMEGEN-05-02-123/2021	EPI_ISL_2230875	29/04/21	North America / Mexico / Mexico City	Human	Male	19	unknown	Oropharyngeal swab
hCoV-19/Mexico/CMX-INMEGEN-05-02-159/2021	EPI_ISL_2230882	01/05/21	North America / Mexico / Mexico City	Human	Male	58	unknown	Oropharyngeal swab
hCoV-19/Mexico/CMX-INMEGEN-05-02-172/2021	EPI_ISL_2230884	02/05/21	North America / Mexico / Mexico City	Human	Male	21	unknown	Oropharyngeal swab
hCoV-19/Mexico/CMX-INMEGEN-05-02-29/2021	EPI_ISL_2230868	05/05/21	North America / Mexico / Mexico City	Human	Male	30	unknown	Oropharyngeal swab
hCoV-19/Mexico/CMX-INMEGEN-05-02-35/2021	EPI_ISL_2230869	05/05/21	North America / Mexico / Mexico City	Human	Female	22	unknown	Oropharyngeal swab
hCoV-19/Mexico/CMX-INMEGEN-05-03-104/2021	EPI_ISL_2319256	08/05/21	North America / Mexico / Mexico City	Human	Male	53	unknown	Oropharyngeal swab
hCoV-19/Mexico/CMX-INMEGEN-05-03-161/2021	EPI_ISL_2319173	06/05/21	North America / Mexico / Mexico City	Human	Male	20	unknown	Oropharyngeal swab
hCoV-19/Mexico/CMX-INMEGEN-05-03-342/2021	EPI_ISL_2319403	06/05/21	North America / Mexico / Mexico City	Human	Male	48	unknown	Oropharyngeal swab
hCoV-19/Mexico/CMX-INMEGEN-05-03-350/2021	EPI_ISL_2319405	06/05/21	North America / Mexico / Mexico City	Human	Female	47	unknown	Oropharyngeal swab
hCoV-19/Mexico/CMX-INMEGEN-05-03-351/2021	EPI_ISL_2319406	06/05/21	North America / Mexico / Mexico City	Human	Male	30	unknown	Oropharyngeal swab
hCoV-19/Mexico/CMX-INMEGEN-05-03-51/2021	EPI_ISL_2319210	07/05/21	North America / Mexico / Mexico City	Human	Female	53	unknown	Oropharyngeal swab
hCoV-19/Mexico/CMX-INMEGEN-05-03-53/2021	EPI_ISL_2319212	07/05/21	North America / Mexico / Mexico City	Human	Male	21	unknown	Oropharyngeal swab
hCoV-19/Mexico/CMX-INMEGEN-05-03-54/2021	EPI_ISL_2319213	08/05/21	North America / Mexico / Mexico City	Human	Female	32	unknown	Oropharyngeal swab
hCoV-19/Mexico/CMX-INMEGEN-05-03-61/2021	EPI_ISL_2881761	08/05/21	North America / Mexico / Mexico City	Human	Male	31	unknown	Oropharyngeal swab
hCoV-19/Mexico/CMX-INMEGEN-05-03-64/2021	EPI_ISL_2319221	08/05/21	North America / Mexico / Mexico City	Human	Female	29	unknown	Oropharyngeal swab
hCoV-19/Mexico/CMX-INMEGEN-05-03-68/2021	EPI_ISL_2319225	08/05/21	North America / Mexico / Mexico City	Human	Female	54	unknown	Oropharyngeal swab
hCoV-19/Mexico/CMX-INMEGEN-05-03-8/2021	EPI_ISL_2319177	07/05/21	North America / Mexico / Mexico City	Human	Female	27	unknown	Oropharyngeal swab
hCoV-19/Mexico/CMX-INMEGEN-05-04-15/2021	EPI_ISL_2349893	11/05/21	North America / Mexico / Mexico City	Human	Female	29	unknown	Oropharyngeal swab
hCoV-19/Mexico/CMX-INMEGEN-05-04-170/2021	EPI_ISL_2350004	14/05/21	North America / Mexico / Mexico City	Human	Female	30	unknown	Oropharyngeal swab
hCoV-19/Mexico/CMX-INMEGEN-05-04-172/2021	EPI_ISL_2350006	14/05/21	North America / Mexico / Mexico City	Human	Female	56	unknown	Oropharyngeal swab
hCoV-19/Mexico/CMX-INMEGEN-05-04-177/2021	EPI_ISL_2350009	13/05/21	North America / Mexico / Mexico City	Human	Female	58	unknown	Oropharyngeal swab
hCoV-19/Mexico/CMX-INMEGEN-05-04-179/2021	EPI_ISL_2350010	14/05/21	North America / Mexico / Mexico City	Human	Female	46	unknown	Oropharyngeal swab
hCoV-19/Mexico/CMX-INMEGEN-05-04-18/2021	EPI_ISL_2349896	11/05/21	North America / Mexico / Mexico City	Human	Female	25	unknown	Oropharyngeal swab
hCoV-19/Mexico/CMX-INMEGEN-05-04-215/2021	EPI_ISL_2350035	15/05/21	North America / Mexico / Mexico City	Human	Male	38	unknown	Oropharyngeal swab
hCoV-19/Mexico/CMX-INMEGEN-05-04-217/2021	EPI_ISL_2350036	15/05/21	North America / Mexico / Mexico City	Human	Male	52	unknown	Oropharyngeal swab
hCoV-19/Mexico/CMX-INMEGEN-05-04-234/2021	EPI_ISL_2350048	17/05/21	North America / Mexico / Mexico City	Human	Male	36	unknown	Oropharyngeal swab
hCoV-19/Mexico/CMX-INMEGEN-05-04-300/2021	EPI_ISL_2350092	13/05/21	North America / Mexico / Mexico City	Human	Male	19	unknown	Oropharyngeal swab
hCoV-19/Mexico/CMX-INMEGEN-05-04-321/2021	EPI_ISL_2350105	14/05/21	North America / Mexico / Mexico City	Human	Male	48	unknown	Oropharyngeal swab
hCoV-19/Mexico/CMX-INMEGEN-05-04-364/2021	EPI_ISL_2350131	17/05/21	North America / Mexico / Mexico City	Human	Female	31	unknown	Oropharyngeal swab
hCoV-19/Mexico/CMX-INMEGEN-05-04-40/2021	EPI_ISL_2349916	12/05/21	North America / Mexico / Mexico City	Human	Male	23	unknown	Oropharyngeal swab
hCoV-19/Mexico/CMX-INMEGEN-05-04-64/2021	EPI_ISL_2349930	12/05/21	North America / Mexico / Mexico City	Human	Male	43	unknown	Oropharyngeal swab
hCoV-19/Mexico/CMX-INMEGEN-05-04-75/2021	EPI_ISL_2349939	12/05/21	North America / Mexico / Mexico City	Human	Female	33	unknown	Oropharyngeal swab
hCoV-19/Mexico/CMX-INMEGEN-05-04-78/2021	EPI_ISL_2349941	12/05/21	North America / Mexico / Mexico City	Human	Female	25	unknown	Oropharyngeal swab
hCoV-19/Mexico/CMX-INMEGEN-05-04-80/2021	EPI_ISL_2349943	12/05/21	North America / Mexico / Mexico City	Human	Female	37	unknown	Oropharyngeal swab
hCoV-19/Mexico/CMX-INMEGEN-05-05-111/2021	EPI_ISL_2881801	21/05/21	North America / Mexico / Mexico City	Human	Male	47	unknown	Oropharyngeal swab
hCoV-19/Mexico/CMX-INMEGEN-05-05-130/2021	EPI_ISL_2444478	21/05/21	North America / Mexico / Mexico City	Human	Female	26	unknown	Oropharyngeal swab
hCoV-19/Mexico/CMX-INMEGEN-05-05-15/2021	EPI_ISL_2444407	23/05/21	North America / Mexico / Mexico City	Human	Male	20	unknown	Oropharyngeal swab
hCoV-19/Mexico/CMX-INMEGEN-05-05-155/2021	EPI_ISL_2444493	21/05/21	North America / Mexico / Mexico City	Human	Female	64	unknown	Oropharyngeal swab
hCoV-19/Mexico/CMX-INMEGEN-05-05-165/2021	EPI_ISL_2881805	21/05/21	North America / Mexico / Mexico City	Human	Male	27	unknown	Oropharyngeal swab
hCoV-19/Mexico/CMX-INMEGEN-05-05-167/2021	EPI_ISL_2444501	21/05/21	North America / Mexico / Mexico City	Human	Female	51	unknown	Oropharyngeal swab
hCoV-19/Mexico/CMX-INMEGEN-05-05-182/2021	EPI_ISL_2444508	21/05/21	North America / Mexico / Mexico City	Human	Male	55	unknown	Oropharyngeal swab
hCoV-19/Mexico/CMX-INMEGEN-05-05-19/2021	EPI_ISL_2444411	22/05/21	North America / Mexico / Mexico City	Human	Male	47	unknown	Oropharyngeal swab
hCoV-19/Mexico/CMX-INMEGEN-05-05-206/2021	EPI_ISL_2550753	20/05/21	North America / Mexico / Mexico City	Human	Male	1	unknown	Oropharyngeal swab
hCoV-19/Mexico/CMX-INMEGEN-05-05-21/2021	EPI_ISL_2444412	22/05/21	North America / Mexico / Mexico City	Human	Female	60	unknown	Oropharyngeal swab
hCoV-19/Mexico/CMX-INMEGEN-05-05-232/2021	EPI_ISL_2444538	20/05/21	North America / Mexico / Mexico City	Human	Female	25	unknown	Oropharyngeal swab
hCoV-19/Mexico/CMX-INMEGEN-05-05-239/2021	EPI_ISL_2444542	20/05/21	North America / Mexico / Mexico City	Human	Male	34	unknown	Oropharyngeal swab
hCoV-19/Mexico/CMX-INMEGEN-05-05-280/2021	EPI_ISL_2444559	20/05/21	North America / Mexico / Mexico City	Human	Female	29	unknown	Oropharyngeal swab
hCoV-19/Mexico/CMX-INMEGEN-05-05-29/2021	EPI_ISL_2444417	22/05/21	North America / Mexico / Mexico City	Human	Male	47	unknown	Oropharyngeal swab
hCoV-19/Mexico/CMX-INMEGEN-05-05-92/2021	EPI_ISL_2444459	22/05/21	North America / Mexico / Mexico City	Human	Female	26	unknown	Oropharyngeal swab
hCoV-19/Mexico/CMX-INMEGEN-05-05-97/2021	EPI_ISL_2444461	21/05/21	North America / Mexico / Mexico City	Human	Female	29	unknown	Oropharyngeal swab
hCoV-19/Mexico/CMX-INMEGEN-05-06-172/2021	EPI_ISL_2603665	25/05/21	North America / Mexico / Mexico City	Human	Male	40	unknown	Oropharyngeal swab
hCoV-19/Mexico/CMX-INMEGEN-05-06-184/2021	EPI_ISL_2603675	25/05/21	North America / Mexico / Mexico City	Human	Male	25	unknown	Oropharyngeal swab

hCoV-19/Mexico/CMX-INMEGEN-05-06-185/2021	EPI_ISL_2603676	25/05/21	North America / Mexico / Mexico City	Human	Female	37	unknown	Oropharyngeal swab
hCoV-19/Mexico/CMX-INMEGEN-05-06-187/2021	EPI_ISL_2603677	25/05/21	North America / Mexico / Mexico City	Human	Male	26	unknown	Oropharyngeal swab
hCoV-19/Mexico/CMX-INMEGEN-05-06-200/2021	EPI_ISL_2603689	27/05/21	North America / Mexico / Mexico City	Human	Female	29	unknown	Oropharyngeal swab
hCoV-19/Mexico/CMX-INMEGEN-05-06-202/2021	EPI_ISL_2603691	28/05/21	North America / Mexico / Mexico City	Human	Female	13	unknown	Oropharyngeal swab
hCoV-19/Mexico/CMX-INMEGEN-05-06-22/2021	EPI_ISL_2603611	24/05/21	North America / Mexico / Mexico City	Human	Female	65	unknown	Oropharyngeal swab
hCoV-19/Mexico/CMX-INMEGEN-05-06-267/2021	EPI_ISL_2603737	28/05/21	North America / Mexico / Mexico City	Human	Female	41	unknown	Oropharyngeal swab
hCoV-19/Mexico/CMX-INMEGEN-05-06-273/2021	EPI_ISL_2603742	28/05/21	North America / Mexico / Mexico City	Human	Female	11	unknown	Oropharyngeal swab
hCoV-19/Mexico/CMX-INMEGEN-05-06-279/2021	EPI_ISL_2603748	27/05/21	North America / Mexico / Mexico City	Human	Male	29	unknown	Oropharyngeal swab
hCoV-19/Mexico/CMX-INMEGEN-05-06-28/2021	EPI_ISL_2603615	25/05/21	North America / Mexico / Mexico City	Human	Female	28	unknown	Oropharyngeal swab
hCoV-19/Mexico/CMX-INMEGEN-05-06-280/2021	EPI_ISL_2603749	28/05/21	North America / Mexico / Mexico City	Human	Male	21	unknown	Oropharyngeal swab
hCoV-19/Mexico/CMX-INMEGEN-05-06-281/2021	EPI_ISL_2603750	28/05/21	North America / Mexico / Mexico City	Human	Male	31	unknown	Oropharyngeal swab
hCoV-19/Mexico/CMX-INMEGEN-05-06-286/2021	EPI_ISL_2603754	29/05/21	North America / Mexico / Mexico City	Human	Male	23	unknown	Oropharyngeal swab
hCoV-19/Mexico/CMX-INMEGEN-05-06-296/2021	EPI_ISL_2603760	29/05/21	North America / Mexico / Mexico City	Human	Male	45	unknown	Oropharyngeal swab
hCoV-19/Mexico/CMX-INMEGEN-05-06-298/2021	EPI_ISL_2603762	29/05/21	North America / Mexico / Mexico City	Human	Female	35	unknown	Oropharyngeal swab
hCoV-19/Mexico/CMX-INMEGEN-05-06-332/2021	EPI_ISL_2603786	28/05/21	North America / Mexico / Mexico City	Human	Male	38	unknown	Oropharyngeal swab
hCoV-19/Mexico/CMX-INMEGEN-05-06-342/2021	EPI_ISL_2603793	29/05/21	North America / Mexico / Mexico City	Human	Male	32	unknown	Oropharyngeal swab
hCoV-19/Mexico/CMX-INMEGEN-05-06-369/2021	EPI_ISL_2603813	29/05/21	North America / Mexico / Mexico City	Human	Male	38	unknown	Oropharyngeal swab
hCoV-19/Mexico/CMX-INMEGEN-05-06-40/2021	EPI_ISL_2603625	26/05/21	North America / Mexico / Mexico City	Human	Female	52	unknown	Oropharyngeal swab
hCoV-19/Mexico/CMX-INMEGEN-05-06-47/2021	EPI_ISL_2603630	27/05/21	North America / Mexico / Mexico City	Human	Female	48	unknown	Oropharyngeal swab
hCoV-19/Mexico/CMX-INMEGEN-05-06-55/2021	EPI_ISL_2603634	27/05/21	North America / Mexico / Mexico City	Human	Female	46	unknown	Oropharyngeal swab
hCoV-19/Mexico/CMX-INMEGEN-05-06-61/2021	EPI_ISL_2603636	27/05/21	North America / Mexico / Mexico City	Human	Female	26	unknown	Oropharyngeal swab
hCoV-19/Mexico/CMX-INMEGEN-05-06-66/2021	EPI_ISL_2603641	27/05/21	North America / Mexico / Mexico City	Human	Female	24	unknown	Oropharyngeal swab
hCoV-19/Mexico/CMX-INMEGEN-05-06-72/2021	EPI_ISL_2603646	28/05/21	North America / Mexico / Mexico City	Human	Male	62	unknown	Oropharyngeal swab
hCoV-19/Mexico/CMX-INMEGEN-05-06-73/2021	EPI_ISL_2603647	21/05/21	North America / Mexico / Mexico City	Human	Male	35	unknown	Oropharyngeal swab
hCoV-19/Mexico/CMX-INMEGEN-05-06-76/2021	EPI_ISL_2603650	27/05/21	North America / Mexico / Mexico City	Human	Male	35	unknown	Oropharyngeal swab
hCoV-19/Mexico/CMX-INMEGEN-05-06-77/2021	EPI_ISL_2603651	27/05/21	North America / Mexico / Mexico City	Human	Male	56	unknown	Oropharyngeal swab
hCoV-19/Mexico/CMX-INMEGEN-05-06-78/2021	EPI_ISL_2603652	28/05/21	North America / Mexico / Mexico City	Human	Male	28	unknown	Oropharyngeal swab
hCoV-19/Mexico/CMX-INMEGEN-05-06-79/2021	EPI_ISL_2603653	28/05/21	North America / Mexico / Mexico City	Human	Male	54	unknown	Oropharyngeal swab
hCoV-19/Mexico/CMX-INMEGEN-05-06-8/2021	EPI_ISL_2603600	24/05/21	North America / Mexico / Mexico City	Human	Female	19	unknown	Oropharyngeal swab
hCoV-19/Mexico/CMX-INMEGEN-05-06-84/2021	EPI_ISL_2603655	29/05/21	North America / Mexico / Mexico City	Human	Male	37	unknown	Oropharyngeal swab
hCoV-19/Mexico/CMX-INMEGEN-05-06-93/2021	EPI_ISL_2603658	31/05/21	North America / Mexico / Mexico City	Human	Male	33	unknown	Oropharyngeal swab
hCoV-19/Mexico/CMX-INMEGEN-06-01-132/2021	EPI_ISL_2616957	02/06/21	North America / Mexico / Mexico City	Human	Female	29	unknown	Oropharyngeal swab
hCoV-19/Mexico/CMX-INMEGEN-06-01-23/2021	EPI_ISL_2616862	01/06/21	North America / Mexico / Mexico City	Human	Male	30	unknown	Oropharyngeal swab
hCoV-19/Mexico/CMX-INMEGEN-06-01-30/2021	EPI_ISL_2616927	01/06/21	North America / Mexico / Mexico City	Human	Male	55	unknown	Oropharyngeal swab
hCoV-19/Mexico/CMX-INMEGEN-06-01-40/2021	EPI_ISL_2616914	29/05/21	North America / Mexico / Mexico City	Human	Female	22	unknown	Oropharyngeal swab
hCoV-19/Mexico/CMX-INMEGEN-06-01-58/2021	EPI_ISL_2616976	31/05/21	North America / Mexico / Mexico City	Human	Female	43	unknown	Oropharyngeal swab
hCoV-19/Mexico/CMX-INMEGEN-06-01-62/2021	EPI_ISL_2616899	01/06/21	North America / Mexico / Mexico City	Human	Male	20	unknown	Oropharyngeal swab
hCoV-19/Mexico/CMX-INMEGEN-06-01-73/2021	EPI_ISL_2616940	30/05/21	North America / Mexico / Mexico City	Human	Male	45	unknown	Oropharyngeal swab
hCoV-19/Mexico/CMX-INMEGEN-06-01-80/2021	EPI_ISL_2616996	30/05/21	North America / Mexico / Mexico City	Human	Female	5	unknown	Oropharyngeal swab
hCoV-19/Mexico/CMX-INMEGEN-06-02-190/2021	EPI_ISL_2692703	13/06/21	North America / Mexico / Mexico City	Human	Female	30	unknown	Oropharyngeal swab
hCoV-19/Mexico/CMX-INMEGEN-06-02-203/2021	EPI_ISL_2692715	14/06/21	North America / Mexico / Mexico City	Human	Female	28	unknown	Oropharyngeal swab
hCoV-19/Mexico/CMX-INMEGEN-06-02-228/2021	EPI_ISL_2692739	13/06/21	North America / Mexico / Mexico City	Human	Female	38	unknown	Oropharyngeal swab
hCoV-19/Mexico/CMX-INMEGEN-06-02-246/2021	EPI_ISL_2692754	16/06/21	North America / Mexico / Mexico City	Human	Female	23	unknown	Oropharyngeal swab
hCoV-19/Mexico/CMX-INMEGEN-06-02-304/2021	EPI_ISL_2692795	26/05/21	North America / Mexico / Mexico City	Human	Male	30	unknown	Oropharyngeal swab
hCoV-19/Mexico/CMX-INMEGEN-06-02-305/2021	EPI_ISL_2692796	26/05/21	North America / Mexico / Mexico City	Human	Female	25	unknown	Oropharyngeal swab
hCoV-19/Mexico/CMX-INMEGEN-06-02-308/2021	EPI_ISL_2692798	26/05/21	North America / Mexico / Mexico City	Human	Male	22	unknown	Oropharyngeal swab
hCoV-19/Mexico/CMX-INMEGEN-06-03-138/2021	EPI_ISL_2810128	04/06/21	North America / Mexico / Mexico City	Human	Male	29	unknown	Oropharyngeal swab
hCoV-19/Mexico/CMX-INMEGEN-06-03-147/2021	EPI_ISL_2810137	04/06/21	North America / Mexico / Mexico City	Human	Male	19	unknown	Oropharyngeal swab
hCoV-19/Mexico/CMX-INMEGEN-06-03-150/2021	EPI_ISL_2810140	05/06/21	North America / Mexico / Mexico City	Human	Female	20	unknown	Oropharyngeal swab
hCoV-19/Mexico/CMX-INMEGEN-06-03-155/2021	EPI_ISL_2810143	04/06/21	North America / Mexico / Mexico City	Human	Female	19	unknown	Oropharyngeal swab
hCoV-19/Mexico/CMX-INMEGEN-06-03-163/2021	EPI_ISL_2810149	14/06/21	North America / Mexico / Mexico City	Human	Female	18	unknown	Oropharyngeal swab
hCoV-19/Mexico/CMX-INMEGEN-06-03-189/2021	EPI_ISL_2842783	23/06/21	North America / Mexico / Mexico City	Human	Male	44	unknown	Oropharyngeal swab

hCoV-19/Mexico/CMX-INMEGEN-06-03-212/2021	EPI_ISL_2842785	24/06/21	North America / Mexico / Mexico City	Human	Female	47	unknown	Oropharyngeal swab
hCoV-19/Mexico/CMX-INMEGEN-06-03-228/2021	EPI_ISL_2842803	02/06/21	North America / Mexico / Mexico City	Human	Female	23	unknown	Oropharyngeal swab
hCoV-19/Mexico/CMX-INMEGEN-06-03-284/2021	EPI_ISL_2810246	23/06/21	North America / Mexico / Mexico City	Human	Male	24	unknown	Oropharyngeal swab
hCoV-19/Mexico/CMX-INMEGEN-06-03-319/2021	EPI_ISL_2810277	09/06/21	North America / Mexico / Mexico City	Human	Female	29	unknown	Oropharyngeal swab
hCoV-19/Mexico/CMX-INMEGEN-06-03-32/2021	EPI_ISL_2810050	24/06/21	North America / Mexico / Mexico City	Human	Female	25	unknown	Oropharyngeal swab
hCoV-19/Mexico/CMX-INMEGEN-06-03-33/2021	EPI_ISL_2810051	24/06/21	North America / Mexico / Mexico City	Human	Male	45	unknown	Oropharyngeal swab
hCoV-19/Mexico/CMX-INMEGEN-06-03-38/2021	EPI_ISL_2810055	24/06/21	North America / Mexico / Mexico City	Human	Male	29	unknown	Oropharyngeal swab
hCoV-19/Mexico/CMX-INMEGEN-06-03-39/2021	EPI_ISL_2810056	24/06/21	lorth America / Mexico / State of Mexic	Human	Female	20	unknown	Oropharyngeal swab
hCoV-19/Mexico/CMX-INMEGEN-06-03-41/2021	EPI_ISL_2810058	24/06/21	North America / Mexico / Mexico City	Human	Female	34	unknown	Oropharyngeal swab
hCoV-19/Mexico/CMX-INMEGEN-06-03-65/2021	EPI_ISL_2810074	24/06/21	North America / Mexico / Mexico City	Human	Female	56	unknown	Oropharyngeal swab
hCoV-19/Mexico/CMX-INMEGEN-06-04-107/2021	EPI_ISL_2894237	21/06/21	North America / Mexico / Mexico City	Human	Male	46	unknown	Oropharyngeal swab
hCoV-19/Mexico/CMX-INMEGEN-06-04-191/2021	EPI_ISL_2894261	22/06/21	North America / Mexico / Mexico City	Human	Female	21	unknown	Oropharyngeal swab
hCoV-19/Mexico/CMX-INMEGEN-06-04-208/2021	EPI_ISL_2894273	25/06/21	North America / Mexico / Mexico City	Human	Male	50	unknown	Oropharyngeal swab
hCoV-19/Mexico/CMX-INMEGEN-06-04-209/2021	EPI_ISL_2894274	25/06/21	North America / Mexico / Mexico City	Human	Male	45	unknown	Oropharyngeal swab
hCoV-19/Mexico/CMX-INMEGEN-06-04-64/2021	EPI_ISL_2894208	09/06/21	North America / Mexico / Mexico City	Human	Female	23	unknown	Oropharyngeal swab
hCoV-19/Mexico/CMX-INMEGEN-06-04-66/2021	EPI_ISL_2894210	10/06/21	North America / Mexico / Mexico City	Human	Male	21	unknown	Oropharyngeal swab
hCoV-19/Mexico/CMX-INMEGEN-16-21/2021	EPI_ISL_2978376	26/06/21	North America / Mexico / Mexico City	Human	Female	18	unknown	Oropharyngeal swab
hCoV-19/Mexico/CMX-INMEGEN-16-75/2021	EPI_ISL_2978423	05/07/21	North America / Mexico / Mexico City	Human	Female	31	unknown	Oropharyngeal swab
hCoV-19/Mexico/CMX-INMEGEN-InDRE-04-04-99/202	EPI_ISL_1628598	05/04/21	North America / Mexico / Mexico City	Human	Female	55	unknown	Oropharyngeal swab
hCoV-19/Mexico/CMX-INMEGEN-InDRE-04-06-165/20	EPI_ISL_1810898	16/04/21	North America / Mexico / Mexico City	Human	Male	52	Hospitalized	Oropharyngeal swab
hCoV-19/Mexico/CMX-INMEGEN-InDRE-04-06-166/20	EPI_ISL_1810899	16/04/21	North America / Mexico / Mexico City	Human	Female	52	Hospitalized	Oropharyngeal swab
hCoV-19/Mexico/CMX-INMEGEN-InDRE-04-06-175/20	EPI_ISL_1810908	16/04/21	North America / Mexico / Mexico City	Human	Male	27	Hospitalized	Oropharyngeal swab
hCoV-19/Mexico/CMX-INMEGEN-InDRE-04-06-182/20	EPI_ISL_1810915	17/04/21	North America / Mexico / Mexico City	Human	Female	39	Hospitalized	Oropharyngeal swab
hCoV-19/Mexico/CMX-INMEGEN-INDRE-05-03-195/20	EPI_ISL_2319317	10/05/21	North America / Mexico / Mexico City	Human	Female	29	unknown	Oropharyngeal swab
hCoV-19/Mexico/CMX-INMEGEN-INDRE-05-03-209/20	EPI_ISL_2319330	09/05/21	North America / Mexico / Mexico City	Human	Male	48	unknown	Oropharyngeal swab
hCoV-19/Mexico/CMX-INMEGEN-INDRE-05-03-211/20	EPI_ISL_2319331	07/05/21	North America / Mexico / Mexico City	Human	Female	74	unknown	Oropharyngeal swab
hCoV-19/Mexico/CMX-INMEGEN-INDRE-05-03-212/20	EPI_ISL_2319332	10/05/21	North America / Mexico / Mexico City	Human	Male	30	unknown	Oropharyngeal swab
hCoV-19/Mexico/CMX-INMEGEN-INDRE-05-03-233/20	EPI_ISL_2319347	12/05/21	North America / Mexico / Mexico City	Human	Female	64	unknown	Oropharyngeal swab
hCoV-19/Mexico/CMX-INMEGEN-INDRE-05-03-249/20	EPI_ISL_2319359	12/05/21	North America / Mexico / Mexico City	Human	Male	67	unknown	Oropharyngeal swab
hCoV-19/Mexico/CMX-INMEGEN-INDRE-05-03-253/20	EPI_ISL_2319363	11/05/21	North America / Mexico / Mexico City	Human	Female	30	unknown	Oropharyngeal swab
hCoV-19/Mexico/CMX-INMEGEN-INDRE-05-03-271/20	EPI_ISL_2319371	14/05/21	North America / Mexico / Mexico City	Human	Male	16	unknown	Oropharyngeal swab
hCoV-19/Mexico/CMX-INMEGEN-INDRE-05-03-275/20	EPI_ISL_2319375	14/05/21	North America / Mexico / Mexico City	Human	Male	53	unknown	Oropharyngeal swab
hCoV-19/Mexico/COA-InDRE_FB13031_S1493/2021	EPI_ISL_1805471	09/04/21	North America / Mexico / Coahuila	Human	Female	38	Hospitalized	Oropharyngeal swab
hCoV-19/Mexico/COA-InDRE_FB14127_S1762/2021	EPI_ISL_2101901	25/04/21	North America / Mexico / Coahuila	Human	Female	60	Hospitalized	Oropharyngeal swab
hCoV-19/Mexico/COA-InDRE_FB16336_S2737/2021	EPI_ISL_2533763	20/05/21	North America / Mexico / Coahuila	Human	Male	39	Hospitalized	Oropharyngeal swab
hCoV-19/Mexico/COA-InDRE_FB16772_S2777/2021	EPI_ISL_2533803	24/05/21	North America / Mexico / Coahuila	Human	Female	38	Hospitalized	Oropharyngeal swab
hCoV-19/Mexico/COA-InDRE_FB17411_S3519/2021	EPI_ISL_2779034	03/06/21	North America / Mexico / Coahuila	Human	Male	24	Hospitalized	Oropharyngeal swab
hCoV-19/Mexico/COA-InDRE_FB17711_S4112/2021	EPI_ISL_2920683	08/06/21	North America / Mexico / Coahuila	Human	Female	34	Hospitalized	Oropharyngeal swab
hCoV-19/Mexico/COA-InDRE_FB18231_S4134/2021	EPI_ISL_2920705	17/06/21	North America / Mexico / Coahuila	Human	Male	56	Hospitalized	Oropharyngeal swab
hCoV-19/Mexico/COL-InDRE_FB15039_S2055/2021	EPI_ISL_2283697	06/05/21	North America / Mexico / Colima	Human	Male	77	Hospitalized	Oropharyngeal swab
hCoV-19/Mexico/COL-InDRE_FB16092_S2459/2021	EPI_ISL_2455980	18/05/21	North America / Mexico / Colima	Human	Male	35	Hospitalized	Oropharyngeal swab
hCoV-19/Mexico/COL-InDRE_FB16093_S3078/2021	EPI_ISL_2663345	11/05/21	North America / Mexico / Colima	Human	Male	58	Hospitalized	Oropharyngeal swab
hCoV-19/Mexico/COL-InDRE_FB16402_S2480/2021	EPI_ISL_2456001	20/05/21	North America / Mexico / Colima	Human	Female	62	unknown	Oropharyngeal swab
hCoV-19/Mexico/COL-InDRE_FB17828_S4115/2021	EPI_ISL_2920686	09/06/21	North America / Mexico / Colima	Human	Female	26	Hospitalized	Oropharyngeal swab
hCoV-19/Mexico/DUR-InDRE_FB17381_S3478/2021	EPI_ISL_2778993	09/06/21	North America / Mexico / Durango	Human	Male	28	Hospitalized	Oropharyngeal swab
hCoV-19/Mexico/DUR-InDRE_FB17382_S3479/2021	EPI_ISL_2778994	09/06/21	North America / Mexico / Durango	Human	Male	19	Hospitalized	Oropharyngeal swab
hCoV-19/Mexico/DUR-InDRE_FB17386_S3481/2021	EPI_ISL_2778996	09/06/21	North America / Mexico / Durango	Human	Female	18	Hospitalized	Oropharyngeal swab
hCoV-19/Mexico/DUR-InDRE_FB17390_S3485/2021	EPI_ISL_2779000	09/06/21	North America / Mexico / Durango	Human	Male	20	Hospitalized	Oropharyngeal swab
hCoV-19/Mexico/GRO-InDRE_F48G_S568/2021	EPI_ISL_1334381	21/01/21	North America / Mexico / Guerrero	Human	Male	53	Deceased	Oropharyngeal swab
hCoV-19/Mexico/GUA-InDRE_F10833_S574/2021	EPI_ISL_1334386	22/02/21	North America / Mexico / Guanajuato	Human	Female	48	Hospitalized	Oropharyngeal swab
hCoV-19/Mexico/GUA-InDRE_F12716_S1202/2021	EPI_ISL_1626809	02/04/21	North America / Mexico / Guanajuato	Human	Female	57	Hospitalized	Oropharyngeal swab
hCoV-19/Mexico/GUA-InDRE_FB13877_S2078/2021	EPI_ISL_2283718	19/04/21	North America / Mexico / Guanajuato	Human	Male	86	Hospitalized	Oropharyngeal swab

hCoV-19/Mexico/GUA-InDRE_FB13881_S1754/2021	EPI_ISL_2101893	20/04/21	North America / Mexico / Guanajuato	Human	Male	47	Hospitalized	Oropharyngeal swab
hCoV-19/Mexico/GUA-InDRE_FB13904_S2077/2021	EPI_ISL_2283717	20/04/21	North America / Mexico / Guanajuato	Human	Male	41	Hospitalized	Oropharyngeal swab
hCoV-19/Mexico/GUA-InDRE_FB13907_S1755/2021	EPI_ISL_2101894	21/04/21	North America / Mexico / Guanajuato	Human	Male	29	Hospitalized	Oropharyngeal swab
hCoV-19/Mexico/GUA-InDRE_FB14636_S2081/2021	EPI_ISL_2295590	30/04/21	North America / Mexico / Guanajuato	Human	Male	25	Hospitalized	Oropharyngeal swab
hCoV-19/Mexico/GUA-InDRE_FB14800_S2082/2021	EPI_ISL_2283721	03/05/21	North America / Mexico / Guanajuato	Human	Male	54	Hospitalized	Oropharyngeal swab
hCoV-19/Mexico/GUA-InDRE_FB14824_S2083/2021	EPI_ISL_2283722	06/05/21	North America / Mexico / Guanajuato	Human	Female	25	Hospitalized	Oropharyngeal swab
hCoV-19/Mexico/GUA-InDRE_FB14826_S2470/2021	EPI_ISL_2455991	05/05/21	North America / Mexico / Guanajuato	Human	Female	31	Hospitalized	Oropharyngeal swab
hCoV-19/Mexico/GUA-InDRE_FB14832_S3059/2021	EPI_ISL_2663326	06/05/21	North America / Mexico / Guanajuato	Human	Male	46	Hospitalized	Oropharyngeal swab
hCoV-19/Mexico/GUA-InDRE_FB14839_S2469/2021	EPI_ISL_2455990	05/05/21	North America / Mexico / Guanajuato	Human	Male	57	Hospitalized	Oropharyngeal swab
hCoV-19/Mexico/GUA-InDRE_FB16209_S2465/2021	EPI_ISL_2455986	17/05/21	North America / Mexico / Guanajuato	Human	Male	54	Hospitalized	Oropharyngeal swab
hCoV-19/Mexico/GUA-InDRE_FB16216_S2466/2021	EPI_ISL_2455987	18/05/21	North America / Mexico / Guanajuato	Human	Male	36	Hospitalized	Oropharyngeal swab
hCoV-19/Mexico/GUA-InDRE_FB16219_S2467/2021	EPI_ISL_2455988	19/05/21	North America / Mexico / Guanajuato	Human	Male	20	Hospitalized	Oropharyngeal swab
hCoV-19/Mexico/GUA-InDRE_FB16459_S3475/2021	EPI_ISL_2778990	24/05/21	North America / Mexico / Guanajuato	Human	Female	62	Hospitalized	Oropharyngeal swab
hCoV-19/Mexico/GUA-InDRE_FB16460_S3779/2021	EPI_ISL_2779281	25/05/21	North America / Mexico / Guanajuato	Human	Male	54	Hospitalized	Oropharyngeal swab
hCoV-19/Mexico/GUA-InDRE_FB16463_S3476/2021	EPI_ISL_2778991	25/05/21	North America / Mexico / Guanajuato	Human	Female	53	Hospitalized	Oropharyngeal swab
hCoV-19/Mexico/GUA-InDRE_FB16465_S3477/2021	EPI_ISL_2778992	26/05/21	North America / Mexico / Guanajuato	Human	Male	16	Hospitalized	Oropharyngeal swab
hCoV-19/Mexico/GUA-InDRE_FB16466_S3504/2021	EPI_ISL_2779019	26/05/21	North America / Mexico / Guanajuato	Human	Female	22	Hospitalized	Oropharyngeal swab
hCoV-19/Mexico/GUA-InDRE_FB16891_S3116/2021	EPI_ISL_2663383	31/05/21	North America / Mexico / Guanajuato	Human	Female	50	Hospitalized	Oropharyngeal swab
hCoV-19/Mexico/GUA-InDRE_FB16894_S3117/2021	EPI_ISL_2663384	28/05/21	North America / Mexico / Guanajuato	Human	Female	55	Hospitalized	Oropharyngeal swab
hCoV-19/Mexico/GUA-InDRE_FB16899_S3118/2021	EPI_ISL_2663385	31/05/21	North America / Mexico / Guanajuato	Human	Female	46	Hospitalized	Oropharyngeal swab
hCoV-19/Mexico/GUA-InDRE_FB16904_S3119/2021	EPI_ISL_2663386	02/06/21	North America / Mexico / Guanajuato	Human	Male	29	Hospitalized	Oropharyngeal swab
hCoV-19/Mexico/GUA-InDRE_FB16908_S3120/2021	EPI_ISL_2663387	03/06/21	North America / Mexico / Guanajuato	Human	Female	67	Hospitalized	Oropharyngeal swab
hCoV-19/Mexico/GUA-InDRE_FB16911_S3121/2021	EPI_ISL_2663388	03/06/21	North America / Mexico / Guanajuato	Human	Female	51	Hospitalized	Oropharyngeal swab
hCoV-19/Mexico/GUA-InDRE_FB17325_S3532/2021	EPI_ISL_2779047	07/06/21	North America / Mexico / Guanajuato	Human	Female	50	Hospitalized	Oropharyngeal swab
hCoV-19/Mexico/GUA-InDRE_FB17346_S3533/2021	EPI_ISL_2779048	11/06/21	North America / Mexico / Guanajuato	Human	Female	80	Deceased	Oropharyngeal swab
hCoV-19/Mexico/HID-InDRE_F12574_S1201/2021	EPI_ISL_1626808	28/03/21	North America / Mexico / Hidalgo	Human	Male	29	unknown	Oropharyngeal swab
hCoV-19/Mexico/HID-InDRE_FB13039_S1505/2021	EPI_ISL_1805483	06/04/21	North America / Mexico / Hidalgo	Human	Male	18	unknown	Oropharyngeal swab
hCoV-19/Mexico/HID-InDRE_FB13996_S2048/2021	EPI_ISL_2283690	19/04/21	North America / Mexico / Hidalgo	Human	Male	76	Hospitalized	Oropharyngeal swab
hCoV-19/Mexico/HID-InDRE_FB14798_S2046/2021	EPI_ISL_2283688	05/05/21	North America / Mexico / Hidalgo	Human	Male	73	Hospitalized	Oropharyngeal swab
hCoV-19/Mexico/HID-InDRE_FB17207_S3093/2021	EPI_ISL_2663360	21/05/21	North America / Mexico / Hidalgo	Human	Male	36	Hospitalized	Oropharyngeal swab
hCoV-19/Mexico/HID-InDRE_FB17212_S3094/2021	EPI_ISL_2663361	26/05/21	North America / Mexico / Hidalgo	Human	Male	44	Hospitalized	Oropharyngeal swab
hCoV-19/Mexico/HID-InDRE_FB17213_S3095/2021	EPI_ISL_2663362	26/05/21	North America / Mexico / Hidalgo	Human	Female	42	Hospitalized	Oropharyngeal swab
hCoV-19/Mexico/HID-InDRE_FB17214_S3096/2021	EPI_ISL_2663363	26/05/21	North America / Mexico / Hidalgo	Human	Female	64	Hospitalized	Oropharyngeal swab
hCoV-19/Mexico/HID-InDRE_FB17221_S3097/2021	EPI_ISL_2663364	28/05/21	North America / Mexico / Hidalgo	Human	Female	54	Deceased	Oropharyngeal swab
hCoV-19/Mexico/HID-InDRE_FB17223_S3098/2021	EPI_ISL_2663365	29/05/21	North America / Mexico / Hidalgo	Human	Male	57	Deceased	Oropharyngeal swab
hCoV-19/Mexico/HID-InDRE_FB17224_S3099/2021	EPI_ISL_2663366	29/05/21	North America / Mexico / Hidalgo	Human	Male	52	Hospitalized	Oropharyngeal swab
hCoV-19/Mexico/HID-InDRE_FB17225_S3100/2021	EPI_ISL_2663367	29/05/21	North America / Mexico / Hidalgo	Human	Male	54	Deceased	Oropharyngeal swab
hCoV-19/Mexico/HID-InDRE_FB17226_S3101/2021	EPI_ISL_2663368	31/05/21	North America / Mexico / Hidalgo	Human	Male	26	Hospitalized	Oropharyngeal swab
hCoV-19/Mexico/HID-InDRE_FB17234_S3102/2021	EPI_ISL_2663369	05/06/21	North America / Mexico / Hidalgo	Human	Female	43	Hospitalized	Oropharyngeal swab
hCoV-19/Mexico/HID-InDRE_FB17235_S3103/2021	EPI_ISL_2663370	05/06/21	North America / Mexico / Hidalgo	Human	Male	15	Hospitalized	Oropharyngeal swab
hCoV-19/Mexico/HID-InDRE_FB17239_S3104/2021	EPI_ISL_2663371	08/06/21	North America / Mexico / Hidalgo	Human	Female	17	Hospitalized	Oropharyngeal swab
hCoV-19/Mexico/HID-InDRE_FB17241_S3105/2021	EPI_ISL_2663372	08/06/21	North America / Mexico / Hidalgo	Human	Female	40	Hospitalized	Oropharyngeal swab
hCoV-19/Mexico/HID-InDRE_FB17242_S3106/2021	EPI_ISL_2663373	08/06/21	North America / Mexico / Hidalgo	Human	Female	67	Hospitalized	Oropharyngeal swab
hCoV-19/Mexico/HID-InDRE_FB17245_S3107/2021	EPI_ISL_2663374	09/06/21	North America / Mexico / Hidalgo	Human	Male	43	Hospitalized	Oropharyngeal swab
hCoV-19/Mexico/HID-InDRE_FB17246_S3108/2021	EPI_ISL_2663375	09/06/21	North America / Mexico / Hidalgo	Human	Male	29	Hospitalized	Oropharyngeal swab
hCoV-19/Mexico/HID-InDRE_FB17887_S3933/2021	EPI_ISL_2858947	14/06/21	North America / Mexico / Hidalgo	Human	Male	51	Hospitalized	Oropharyngeal swab
hCoV-19/Mexico/HID-InDRE_FB19125_S4493/2021	EPI_ISL_3033347	21/06/21	North America / Mexico / Hidalgo	Human	Male	53	Hospitalized	Oropharyngeal swab
hCoV-19/Mexico/HID-InDRE_FB19127_S4494/2021	EPI_ISL_3033348	22/06/21	North America / Mexico / Hidalgo	Human	Male	17	Hospitalized	Oropharyngeal swab
hCoV-19/Mexico/HID-InDRE_FB19128_S4495/2021	EPI_ISL_3033349	22/06/21	North America / Mexico / Hidalgo	Human	Female	43	Hospitalized	Oropharyngeal swab
hCoV-19/Mexico/HID-InDRE_FB19136_S4496/2021	EPI_ISL_3033350	25/06/21	North America / Mexico / Hidalgo	Human	Male	61	Hospitalized	Oropharyngeal swab
hCoV-19/Mexico/HID-InDRE_FB19137_S4497/2021	EPI_ISL_3033351	25/06/21	North America / Mexico / Hidalgo	Human	Male	38	Deceased	Oropharyngeal swab
hCoV-19/Mexico/HID-InDRE_FB19139_S4498/2021	EPI_ISL_3033352	25/06/21	North America / Mexico / Hidalgo	Human	Female	55	Hospitalized	Oropharyngeal swab

hCoV-19/Mexico/HID-INMEGEN-05-02-116/2021	EPI_ISL_2230873	29/04/21	North America / Mexico / Hidalgo	Human	Female	39	unknown	Oropharyngeal swab
hCoV-19/Mexico/HID-INMEGEN-05-02-154/2021	EPI_ISL_2230876	30/04/21	North America / Mexico / Hidalgo	Human	Female	22	unknown	Oropharyngeal swab
hCoV-19/Mexico/HID-INMEGEN-05-02-171/2021	EPI_ISL_2230878	02/05/21	North America / Mexico / Hidalgo	Human	Female	21	unknown	Oropharyngeal swab
hCoV-19/Mexico/HID-INMEGEN-05-02-179/2021	EPI_ISL_2230885	02/05/21	North America / Mexico / Hidalgo	Human	Female	20	unknown	Oropharyngeal swab
hCoV-19/Mexico/HID-INMEGEN-05-03-156/2021	EPI_ISL_2319289	09/05/21	North America / Mexico / Hidalgo	Human	Female	54	unknown	Oropharyngeal swab
hCoV-19/Mexico/HID-INMEGEN-05-03-157/2021	EPI_ISL_2319290	09/05/21	North America / Mexico / Hidalgo	Human	Female	21	unknown	Oropharyngeal swab
hCoV-19/Mexico/HID-INMEGEN-05-03-160/2021	EPI_ISL_2319293	09/05/21	North America / Mexico / Hidalgo	Human	Female	26	unknown	Oropharyngeal swab
hCoV-19/Mexico/HID-INMEGEN-05-03-29/2021	EPI_ISL_2319191	07/05/21	North America / Mexico / Hidalgo	Human	Female	44	unknown	Oropharyngeal swab
hCoV-19/Mexico/HID-INMEGEN-05-03-32/2021	EPI_ISL_2319193	07/05/21	North America / Mexico / Hidalgo	Human	Female	69	unknown	Oropharyngeal swab
hCoV-19/Mexico/HID-INMEGEN-05-04-25/2021	EPI_ISL_2349903	11/05/21	North America / Mexico / Hidalgo	Human	Female	55	unknown	Oropharyngeal swab
hCoV-19/Mexico/HID-INMEGEN-05-04-37/2021	EPI_ISL_2349913	12/05/21	North America / Mexico / Hidalgo	Human	Male	59	unknown	Oropharyngeal swab
hCoV-19/Mexico/HID-INMEGEN-05-05-141/2021	EPI_ISL_2444484	21/05/21	North America / Mexico / Hidalgo	Human	Male	39	unknown	Oropharyngeal swab
hCoV-19/Mexico/HID-INMEGEN-05-05-71/2021	EPI_ISL_2444449	23/05/21	North America / Mexico / Hidalgo	Human	Male	60	unknown	Oropharyngeal swab
hCoV-19/Mexico/HID-INMEGEN-05-06-371/2021	EPI_ISL_2603814	28/05/21	North America / Mexico / Hidalgo	Human	Female	30	unknown	Oropharyngeal swab
hCoV-19/Mexico/HID-INMEGEN-06-03-153/2021	EPI_ISL_2928491	03/06/21	North America / Mexico / Hidalgo	Human	Female	22	unknown	Oropharyngeal swab
hCoV-19/Mexico/HID-INMEGEN-06-03-173/2021	EPI_ISL_2928492	14/06/21	North America / Mexico / Hidalgo	Human	Male	28	unknown	Oropharyngeal swab
hCoV-19/Mexico/HID-INMEGEN-06-03-186/2021	EPI_ISL_2810168	04/06/21	North America / Mexico / Hidalgo	Human	Female	45	unknown	Oropharyngeal swab
hCoV-19/Mexico/JAL-InDRE_F11689_S895/2021	EPI_ISL_1423505	06/03/21	North America / Mexico / Jalisco	Human	Male	56	Hospitalized	Oropharyngeal swab
hCoV-19/Mexico/JAL-InDRE_FB13392_S1763/2021	EPI_ISL_2101902	16/04/21	North America / Mexico / Jalisco	Human	Male	51	unknown	Oropharyngeal swab
hCoV-19/Mexico/JAL-InDRE_FB13396_S1730/2021	EPI_ISL_2101869	19/04/21	North America / Mexico / Jalisco	Human	Female	83	Hospitalized	Oropharyngeal swab
hCoV-19/Mexico/JAL-InDRE_FB13967_S1756/2021	EPI_ISL_2101895	21/04/21	North America / Mexico / Jalisco	Human	Male	78	Hospitalized	Oropharyngeal swab
hCoV-19/Mexico/JAL-InDRE_FB13972_S1752/2021	EPI_ISL_2101891	21/04/21	North America / Mexico / Jalisco	Human	Male	78	Deceased	Oropharyngeal swab
hCoV-19/Mexico/JAL-InDRE_FB14113_S1761/2021	EPI_ISL_2101900	26/04/21	North America / Mexico / Jalisco	Human	Female	41	Hospitalized	Oropharyngeal swab
hCoV-19/Mexico/JAL-InDRE_FB14278_S1766/2021	EPI_ISL_2101905	27/04/21	North America / Mexico / Jalisco	Human	Male	70	unknown	Oropharyngeal swab
hCoV-19/Mexico/JAL-InDRE_FB14563_S2044/2021	EPI_ISL_2283686	29/04/21	North America / Mexico / Jalisco	Human	Male	51	unknown	Oropharyngeal swab
hCoV-19/Mexico/JAL-InDRE_FB16243_S2434/2021	EPI_ISL_2455958	22/05/21	North America / Mexico / Jalisco	Human	Female	70	unknown	Oropharyngeal swab
hCoV-19/Mexico/JAL-InDRE_FB16511_S2741/2021	EPI_ISL_2533767	30/05/21	North America / Mexico / Jalisco	Human	Male	75	Hospitalized	Oropharyngeal swab
hCoV-19/Mexico/JAL-InDRE_FB16739_S2776/2021	EPI_ISL_2533802	22/05/21	North America / Mexico / Jalisco	Human	Male	55	unknown	Oropharyngeal swab
hCoV-19/Mexico/JAL-InDRE_FB18117_S3958/2021	EPI_ISL_2858972	12/06/21	North America / Mexico / Jalisco	Human	Female	25	unknown	Oropharyngeal swab
hCoV-19/Mexico/MEX-InDRE_FB17389_S3484/2021	EPI_ISL_2778999	09/06/21	lorth America / Mexico / State of Mexic	Human	Male	23	Hospitalized	Oropharyngeal swab
hCoV-19/Mexico/MEX-InDRE_FD68596_S2471/2021	EPI_ISL_2455992	14/05/21	lorth America / Mexico / State of Mexic	Human	Male	34	Hospitalized	Oropharyngeal swab
hCoV-19/Mexico/MEX-InDRE_FD71099_S2783/2021	EPI_ISL_2533809	19/05/21	lorth America / Mexico / State of Mexic	Human	Female	40	Hospitalized	Oropharyngeal swab
hCoV-19/Mexico/MEX-INMEGEN-05-01-227/2021	EPI_ISL_2105747	27/04/21	lorth America / Mexico / State of Mexic	Human	Male	48	unknown	Oropharyngeal swab
hCoV-19/Mexico/MEX-INMEGEN-05-01-240/2021	EPI_ISL_2105759	27/04/21	lorth America / Mexico / State of Mexic	Human	Male	29	unknown	Oropharyngeal swab
hCoV-19/Mexico/MEX-INMEGEN-05-01-256/2021	EPI_ISL_2178757	28/04/21	lorth America / Mexico / State of Mexic	Human	Female	25	unknown	Oropharyngeal swab
hCoV-19/Mexico/MEX-INMEGEN-05-01-266/2021	EPI_ISL_2105778	28/04/21	lorth America / Mexico / State of Mexic	Human	Male	20	unknown	Oropharyngeal swab
hCoV-19/Mexico/MEX-INMEGEN-05-01-278/2021	EPI_ISL_2105790	28/04/21	lorth America / Mexico / State of Mexic	Human	Male	25	unknown	Oropharyngeal swab
hCoV-19/Mexico/MEX-INMEGEN-05-01-283/2021	EPI_ISL_2105795	28/04/21	lorth America / Mexico / State of Mexic	Human	Male	49	unknown	Oropharyngeal swab
hCoV-19/Mexico/MEX-INMEGEN-05-01-309/2021	EPI_ISL_2105817	30/04/21	lorth America / Mexico / State of Mexic	Human	Male	48	unknown	Oropharyngeal swab
hCoV-19/Mexico/MEX-INMEGEN-05-01-314/2021	EPI_ISL_2105822	01/05/21	lorth America / Mexico / State of Mexic	Human	Male	38	unknown	Oropharyngeal swab
hCoV-19/Mexico/MEX-INMEGEN-05-01-326/2021	EPI_ISL_2105832	01/05/21	lorth America / Mexico / State of Mexic	Human	Male	33	unknown	Oropharyngeal swab
hCoV-19/Mexico/MEX-INMEGEN-05-01-345/2021	EPI_ISL_2105850	03/05/21	lorth America / Mexico / State of Mexic	Human	Female	53	unknown	Oropharyngeal swab
hCoV-19/Mexico/MEX-INMEGEN-05-01-349/2021	EPI_ISL_2105853	03/05/21	lorth America / Mexico / State of Mexic	Human	Male	21	unknown	Oropharyngeal swab
hCoV-19/Mexico/MEX-INMEGEN-05-01-366/2021	EPI_ISL_2105867	03/05/21	lorth America / Mexico / State of Mexic	Human	Male	25	unknown	Oropharyngeal swab
hCoV-19/Mexico/MEX-INMEGEN-05-02-1/2021	EPI_ISL_2230865	03/05/21	lorth America / Mexico / State of Mexic	Human	Male	26	unknown	Oropharyngeal swab
hCoV-19/Mexico/MEX-INMEGEN-05-02-104/2021	EPI_ISL_2230871	29/04/21	lorth America / Mexico / State of Mexic	Human	Male	21	unknown	Oropharyngeal swab
hCoV-19/Mexico/MEX-INMEGEN-05-02-135/2021	EPI_ISL_2230832	30/04/21	lorth America / Mexico / State of Mexic	Human	Male	61	unknown	Oropharyngeal swab
hCoV-19/Mexico/MEX-INMEGEN-05-02-162/2021	EPI_ISL_2230883	01/05/21	lorth America / Mexico / State of Mexic	Human	Male	27	unknown	Oropharyngeal swab
hCoV-19/Mexico/MEX-INMEGEN-05-02-166/2021	EPI_ISL_2230877	02/05/21	lorth America / Mexico / State of Mexic	Human	Female	30	unknown	Oropharyngeal swab
hCoV-19/Mexico/MEX-INMEGEN-05-02-168/2021	EPI_ISL_2230806	02/05/21	lorth America / Mexico / State of Mexic	Human	Female	58	unknown	Oropharyngeal swab
hCoV-19/Mexico/MEX-INMEGEN-05-02-175/2021	EPI_ISL_2230825	02/05/21	lorth America / Mexico / State of Mexic	Human	Female	24	unknown	Oropharyngeal swab
hCoV-19/Mexico/MEX-INMEGEN-05-02-187/2021	EPI_ISL_2230879	03/05/21	lorth America / Mexico / State of Mexic	Human	Female	16	unknown	Oropharyngeal swab

hCoV-19/Mexico/MEX-INMEGEN-05-02-2/2021	EPI_ISL_2230866	03/05/21	lorth America / Mexico / State of Mexic	Human	Female	25	unknown	Oropharyngeal swab
hCoV-19/Mexico/MEX-INMEGEN-05-02-23/2021	EPI_ISL_2230841	05/05/21	lorth America / Mexico / State of Mexic	Human	Male	78	unknown	Oropharyngeal swab
hCoV-19/Mexico/MEX-INMEGEN-05-02-24/2021	EPI_ISL_2230867	05/05/21	lorth America / Mexico / State of Mexic	Human	Female	56	unknown	Oropharyngeal swab
hCoV-19/Mexico/MEX-INMEGEN-05-02-47/2021	EPI_ISL_2230881	05/05/21	lorth America / Mexico / State of Mexic	Human	Female	43	unknown	Oropharyngeal swab
hCoV-19/Mexico/MEX-INMEGEN-05-03-103/2021	EPI_ISL_2319255	08/05/21	lorth America / Mexico / State of Mexic	Human	Male	32	unknown	Oropharyngeal swab
hCoV-19/Mexico/MEX-INMEGEN-05-03-118/2021	EPI_ISL_2319264	09/05/21	lorth America / Mexico / State of Mexic	Human	Male	31	unknown	Oropharyngeal swab
hCoV-19/Mexico/MEX-INMEGEN-05-03-127/2021	EPI_ISL_2319273	09/05/21	lorth America / Mexico / State of Mexic	Human	Female	49	unknown	Oropharyngeal swab
hCoV-19/Mexico/MEX-INMEGEN-05-03-15/2021	EPI_ISL_2319183	07/05/21	lorth America / Mexico / State of Mexic	Human	Male	51	unknown	Oropharyngeal swab
hCoV-19/Mexico/MEX-INMEGEN-05-03-153/2021	EPI_ISL_2341033	09/05/21	rth America / Mexico / Estado de Mexi	Human	Female	30	unknown	Oropharyngeal swab
hCoV-19/Mexico/MEX-INMEGEN-05-03-167/2021	EPI_ISL_2319298	10/05/21	lorth America / Mexico / State of Mexic	Human	Male	52	unknown	Oropharyngeal swab
hCoV-19/Mexico/MEX-INMEGEN-05-03-287/2021	EPI_ISL_2319383	05/05/21	lorth America / Mexico / State of Mexic	Human	Male	31	unknown	Oropharyngeal swab
hCoV-19/Mexico/MEX-INMEGEN-05-03-292/2021	EPI_ISL_2319385	05/05/21	lorth America / Mexico / State of Mexic	Human	Female	34	unknown	Oropharyngeal swab
hCoV-19/Mexico/MEX-INMEGEN-05-03-300/2021	EPI_ISL_2881779	05/05/21	lorth America / Mexico / State of Mexic	Human	Female	29	unknown	Oropharyngeal swab
hCoV-19/Mexico/MEX-INMEGEN-05-03-328/2021	EPI_ISL_2319396	05/05/21	lorth America / Mexico / State of Mexic	Human	Female	22	unknown	Oropharyngeal swab
hCoV-19/Mexico/MEX-INMEGEN-05-03-332/2021	EPI_ISL_2319400	06/05/21	lorth America / Mexico / State of Mexic	Human	Male	36	unknown	Oropharyngeal swab
hCoV-19/Mexico/MEX-INMEGEN-05-03-340/2021	EPI_ISL_2319402	06/05/21	lorth America / Mexico / State of Mexic	Human	Male	52	unknown	Oropharyngeal swab
hCoV-19/Mexico/MEX-INMEGEN-05-03-347/2021	EPI_ISL_2341034	06/05/21	rth America / Mexico / Estado de Mexi	Human	Female	39	unknown	Oropharyngeal swab
hCoV-19/Mexico/MEX-INMEGEN-05-03-360/2021	EPI_ISL_2319411	06/05/21	lorth America / Mexico / State of Mexic	Human	Male	55	unknown	Oropharyngeal swab
hCoV-19/Mexico/MEX-INMEGEN-05-03-368/2021	EPI_ISL_2319412	06/05/21	lorth America / Mexico / State of Mexic	Human	Female	23	unknown	Oropharyngeal swab
hCoV-19/Mexico/MEX-INMEGEN-05-03-4/2021	EPI_ISL_2319174	07/05/21	lorth America / Mexico / State of Mexic	Human	Female	28	unknown	Oropharyngeal swab
hCoV-19/Mexico/MEX-INMEGEN-05-03-43/2021	EPI_ISL_2319202	07/05/21	lorth America / Mexico / State of Mexic	Human	Male	28	unknown	Oropharyngeal swab
hCoV-19/Mexico/MEX-INMEGEN-05-03-46/2021	EPI_ISL_2319205	07/05/21	lorth America / Mexico / State of Mexic	Human	Female	59	unknown	Oropharyngeal swab
hCoV-19/Mexico/MEX-INMEGEN-05-03-5/2021	EPI_ISL_2319175	07/05/21	lorth America / Mexico / State of Mexic	Human	Female	40	unknown	Oropharyngeal swab
hCoV-19/Mexico/MEX-INMEGEN-05-03-69/2021	EPI_ISL_2319226	08/05/21	lorth America / Mexico / State of Mexic	Human	Female	30	unknown	Oropharyngeal swab
hCoV-19/Mexico/MEX-INMEGEN-05-03-72/2021	EPI_ISL_2319229	08/05/21	lorth America / Mexico / State of Mexic	Human	Male	38	unknown	Oropharyngeal swab
hCoV-19/Mexico/MEX-INMEGEN-05-03-73/2021	EPI_ISL_2319230	08/05/21	lorth America / Mexico / State of Mexic	Human	Female	26	unknown	Oropharyngeal swab
hCoV-19/Mexico/MEX-INMEGEN-05-03-91/2021	EPI_ISL_2319245	08/05/21	lorth America / Mexico / State of Mexic	Human	Male	49	unknown	Oropharyngeal swab
hCoV-19/Mexico/MEX-INMEGEN-05-04-100/2021	EPI_ISL_2349957	14/05/21	lorth America / Mexico / State of Mexic	Human	Male	58	unknown	Oropharyngeal swab
hCoV-19/Mexico/MEX-INMEGEN-05-04-103/2021	EPI_ISL_2349960	13/05/21	lorth America / Mexico / State of Mexic	Human	Female	47	unknown	Oropharyngeal swab
hCoV-19/Mexico/MEX-INMEGEN-05-04-105/2021	EPI_ISL_2349961	13/05/21	lorth America / Mexico / State of Mexic	Human	Female	53	unknown	Oropharyngeal swab
hCoV-19/Mexico/MEX-INMEGEN-05-04-110/2021	EPI_ISL_2349965	13/05/21	lorth America / Mexico / State of Mexic	Human	Female	36	unknown	Oropharyngeal swab
hCoV-19/Mexico/MEX-INMEGEN-05-04-112/2021	EPI_ISL_2349966	13/05/21	lorth America / Mexico / State of Mexic	Human	Male	49	unknown	Oropharyngeal swab
hCoV-19/Mexico/MEX-INMEGEN-05-04-113/2021	EPI_ISL_2349967	13/05/21	lorth America / Mexico / State of Mexic	Human	Male	44	unknown	Oropharyngeal swab
hCoV-19/Mexico/MEX-INMEGEN-05-04-133/2021	EPI_ISL_2349979	14/05/21	lorth America / Mexico / State of Mexic	Human	Male	53	unknown	Oropharyngeal swab
hCoV-19/Mexico/MEX-INMEGEN-05-04-156/2021	EPI_ISL_2443076	14/05/21	lorth America / Mexico / State of Mexic	Human	Male	58	unknown	Oropharyngeal swab
hCoV-19/Mexico/MEX-INMEGEN-05-04-165/2021	EPI_ISL_2350000	15/05/21	lorth America / Mexico / State of Mexic	Human	Male	42	unknown	Oropharyngeal swab
hCoV-19/Mexico/MEX-INMEGEN-05-04-189/2021	EPI_ISL_2350017	15/05/21	lorth America / Mexico / State of Mexic	Human	Male	35	unknown	Oropharyngeal swab
hCoV-19/Mexico/MEX-INMEGEN-05-04-197/2021	EPI_ISL_2350022	15/05/21	lorth America / Mexico / State of Mexic	Human	Male	32	unknown	Oropharyngeal swab
hCoV-19/Mexico/MEX-INMEGEN-05-04-200/2021	EPI_ISL_2350025	15/05/21	lorth America / Mexico / State of Mexic	Human	Female	33	unknown	Oropharyngeal swab
hCoV-19/Mexico/MEX-INMEGEN-05-04-204/2021	EPI_ISL_2350027	15/05/21	lorth America / Mexico / State of Mexic	Human	Male	46	unknown	Oropharyngeal swab
hCoV-19/Mexico/MEX-INMEGEN-05-04-21/2021	EPI_ISL_2349899	11/05/21	lorth America / Mexico / State of Mexic	Human	Male	57	unknown	Oropharyngeal swab
hCoV-19/Mexico/MEX-INMEGEN-05-04-214/2021	EPI_ISL_2350034	15/05/21	lorth America / Mexico / State of Mexic	Human	Male	29	unknown	Oropharyngeal swab
hCoV-19/Mexico/MEX-INMEGEN-05-04-229/2021	EPI_ISL_2350045	15/05/21	lorth America / Mexico / State of Mexic	Human	Male	10	unknown	Oropharyngeal swab
hCoV-19/Mexico/MEX-INMEGEN-05-04-235/2021	EPI_ISL_2350049	17/05/21	lorth America / Mexico / State of Mexic	Human	Female	37	unknown	Oropharyngeal swab
hCoV-19/Mexico/MEX-INMEGEN-05-04-245/2021	EPI_ISL_2350057	15/05/21	lorth America / Mexico / State of Mexic	Human	Female	14	unknown	Oropharyngeal swab
hCoV-19/Mexico/MEX-INMEGEN-05-04-248/2021	EPI_ISL_2350059	15/05/21	lorth America / Mexico / State of Mexic	Human	Female	58	unknown	Oropharyngeal swab
hCoV-19/Mexico/MEX-INMEGEN-05-04-253/2021	EPI_ISL_2350062	15/05/21	lorth America / Mexico / State of Mexic	Human	Male	40	unknown	Oropharyngeal swab
hCoV-19/Mexico/MEX-INMEGEN-05-04-258/2021	EPI_ISL_2350065	17/05/21	lorth America / Mexico / State of Mexic	Human	Female	33	unknown	Oropharyngeal swab
hCoV-19/Mexico/MEX-INMEGEN-05-04-262/2021	EPI_ISL_2350068	15/05/21	lorth America / Mexico / State of Mexic	Human	Female	33	unknown	Oropharyngeal swab
hCoV-19/Mexico/MEX-INMEGEN-05-04-283/2021	EPI_ISL_2350081	13/05/21	lorth America / Mexico / State of Mexic	Human	Female	25	unknown	Oropharyngeal swab
hCoV-19/Mexico/MEX-INMEGEN-05-04-285/2021	EPI_ISL_2350082	13/05/21	lorth America / Mexico / State of Mexic	Human	Male	44	unknown	Oropharyngeal swab
hCoV-19/Mexico/MEX-INMEGEN-05-04-292/2021	EPI_ISL_2350087	13/05/21	lorth America / Mexico / State of Mexic	Human	Male	50	unknown	Oropharyngeal swab

hCoV-19/Mexico/MEX-INMEGEN-05-04-296/2021	EPI_ISL_2350090	13/05/21	lorth America / Mexico / State of Mexic	Human	Male	22	unknown	Oropharyngeal swab
hCoV-19/Mexico/MEX-INMEGEN-05-04-337/2021	EPI_ISL_2350115	17/05/21	lorth America / Mexico / State of Mexic	Human	Female	7	unknown	Oropharyngeal swab
hCoV-19/Mexico/MEX-INMEGEN-05-04-341/2021	EPI_ISL_2350118	17/05/21	lorth America / Mexico / State of Mexic	Human	Female	37	unknown	Oropharyngeal swab
hCoV-19/Mexico/MEX-INMEGEN-05-04-355/2021	EPI_ISL_2350123	17/05/21	lorth America / Mexico / State of Mexic	Human	Female	61	unknown	Oropharyngeal swab
hCoV-19/Mexico/MEX-INMEGEN-05-04-357/2021	EPI_ISL_2350124	17/05/21	lorth America / Mexico / State of Mexic	Human	Female	42	unknown	Oropharyngeal swab
hCoV-19/Mexico/MEX-INMEGEN-05-04-36/2021	EPI_ISL_2349912	12/05/21	lorth America / Mexico / State of Mexic	Human	Male	19	unknown	Oropharyngeal swab
hCoV-19/Mexico/MEX-INMEGEN-05-04-373/2021	EPI_ISL_2350138	05/05/21	lorth America / Mexico / State of Mexic	Human	Male	13	unknown	Oropharyngeal swab
hCoV-19/Mexico/MEX-INMEGEN-05-04-53/2021	EPI_ISL_2349922	12/05/21	lorth America / Mexico / State of Mexic	Human	Female	44	unknown	Oropharyngeal swab
hCoV-19/Mexico/MEX-INMEGEN-05-04-61/2021	EPI_ISL_2349927	12/05/21	lorth America / Mexico / State of Mexic	Human	Male	28	unknown	Oropharyngeal swab
hCoV-19/Mexico/MEX-INMEGEN-05-04-69/2021	EPI_ISL_2349934	12/05/21	lorth America / Mexico / State of Mexic	Human	Male	33	unknown	Oropharyngeal swab
hCoV-19/Mexico/MEX-INMEGEN-05-04-82/2021	EPI_ISL_2349945	12/05/21	lorth America / Mexico / State of Mexic	Human	Male	41	unknown	Oropharyngeal swab
hCoV-19/Mexico/MEX-INMEGEN-05-04-85/2021	EPI_ISL_2349947	12/05/21	lorth America / Mexico / State of Mexic	Human	Male	32	unknown	Oropharyngeal swab
hCoV-19/Mexico/MEX-INMEGEN-05-04-94/2021	EPI_ISL_2349952	11/05/21	lorth America / Mexico / State of Mexic	Human	Male	32	unknown	Oropharyngeal swab
hCoV-19/Mexico/MEX-INMEGEN-05-04-95/2021	EPI_ISL_2349953	13/05/21	lorth America / Mexico / State of Mexic	Human	Male	25	unknown	Oropharyngeal swab
hCoV-19/Mexico/MEX-INMEGEN-05-05-102/2021	EPI_ISL_2444462	21/05/21	lorth America / Mexico / State of Mexic	Human	Female	18	unknown	Oropharyngeal swab
hCoV-19/Mexico/MEX-INMEGEN-05-05-112/2021	EPI_ISL_2444469	21/05/21	lorth America / Mexico / State of Mexic	Human	Female	29	unknown	Oropharyngeal swab
hCoV-19/Mexico/MEX-INMEGEN-05-05-113/2021	EPI_ISL_2444470	21/05/21	lorth America / Mexico / State of Mexic	Human	Female	51	unknown	Oropharyngeal swab
hCoV-19/Mexico/MEX-INMEGEN-05-05-136/2021	EPI_ISL_2550747	21/05/21	lorth America / Mexico / State of Mexic	Human	Male	31	unknown	Oropharyngeal swab
hCoV-19/Mexico/MEX-INMEGEN-05-05-148/2021	EPI_ISL_2444489	21/05/21	lorth America / Mexico / State of Mexic	Human	Female	26	unknown	Oropharyngeal swab
hCoV-19/Mexico/MEX-INMEGEN-05-05-149/2021	EPI_ISL_2881804	21/05/21	lorth America / Mexico / State of Mexic	Human	Female	28	unknown	Oropharyngeal swab
hCoV-19/Mexico/MEX-INMEGEN-05-05-153/2021	EPI_ISL_2444492	21/05/21	lorth America / Mexico / State of Mexic	Human	Female	48	unknown	Oropharyngeal swab
hCoV-19/Mexico/MEX-INMEGEN-05-05-156/2021	EPI_ISL_2444494	21/05/21	lorth America / Mexico / State of Mexic	Human	Male	38	unknown	Oropharyngeal swab
hCoV-19/Mexico/MEX-INMEGEN-05-05-161/2021	EPI_ISL_2444497	21/05/21	lorth America / Mexico / State of Mexic	Human	Male	5	unknown	Oropharyngeal swab
hCoV-19/Mexico/MEX-INMEGEN-05-05-166/2021	EPI_ISL_2444500	21/05/21	lorth America / Mexico / State of Mexic	Human	Male	58	unknown	Oropharyngeal swab
hCoV-19/Mexico/MEX-INMEGEN-05-05-169/2021	EPI_ISL_2444502	21/05/21	lorth America / Mexico / State of Mexic	Human	Female	36	unknown	Oropharyngeal swab
hCoV-19/Mexico/MEX-INMEGEN-05-05-200/2021	EPI_ISL_2550752	20/05/21	lorth America / Mexico / State of Mexic	Human	Male	53	unknown	Oropharyngeal swab
hCoV-19/Mexico/MEX-INMEGEN-05-05-204/2021	EPI_ISL_2444521	20/05/21	lorth America / Mexico / State of Mexic	Human	Male	29	unknown	Oropharyngeal swab
hCoV-19/Mexico/MEX-INMEGEN-05-05-205/2021	EPI_ISL_2444522	20/05/21	lorth America / Mexico / State of Mexic	Human	Female	21	unknown	Oropharyngeal swab
hCoV-19/Mexico/MEX-INMEGEN-05-05-212/2021	EPI_ISL_2444525	20/05/21	lorth America / Mexico / State of Mexic	Human	Male	37	unknown	Oropharyngeal swab
hCoV-19/Mexico/MEX-INMEGEN-05-05-224/2021	EPI_ISL_2444534	20/05/21	lorth America / Mexico / State of Mexic	Human	Male	35	unknown	Oropharyngeal swab
hCoV-19/Mexico/MEX-INMEGEN-05-05-229/2021	EPI_ISL_2550754	20/05/21	lorth America / Mexico / State of Mexic	Human	Female	32	unknown	Oropharyngeal swab
hCoV-19/Mexico/MEX-INMEGEN-05-05-247/2021	EPI_ISL_2444546	20/05/21	lorth America / Mexico / State of Mexic	Human	Male	26	unknown	Oropharyngeal swab
hCoV-19/Mexico/MEX-INMEGEN-05-05-279/2021	EPI_ISL_2444558	20/05/21	lorth America / Mexico / State of Mexic	Human	Female	49	unknown	Oropharyngeal swab
hCoV-19/Mexico/MEX-INMEGEN-05-05-3/2021	EPI_ISL_2444399	21/05/21	lorth America / Mexico / State of Mexic	Human	Male	45	unknown	Oropharyngeal swab
hCoV-19/Mexico/MEX-INMEGEN-05-05-30/2021	EPI_ISL_2444418	23/05/21	lorth America / Mexico / State of Mexic	Human	Female	33	unknown	Oropharyngeal swab
hCoV-19/Mexico/MEX-INMEGEN-05-05-34/2021	EPI_ISL_2444421	21/05/21	lorth America / Mexico / State of Mexic	Human	Female	32	unknown	Oropharyngeal swab
hCoV-19/Mexico/MEX-INMEGEN-05-05-39/2021	EPI_ISL_2444424	23/05/21	lorth America / Mexico / State of Mexic	Human	Male	32	unknown	Oropharyngeal swab
hCoV-19/Mexico/MEX-INMEGEN-05-05-40/2021	EPI_ISL_2444425	21/05/21	lorth America / Mexico / State of Mexic	Human	Male	47	unknown	Oropharyngeal swab
hCoV-19/Mexico/MEX-INMEGEN-05-05-42/2021	EPI_ISL_2444426	21/05/21	lorth America / Mexico / State of Mexic	Human	Male	57	unknown	Oropharyngeal swab
hCoV-19/Mexico/MEX-INMEGEN-05-05-46/2021	EPI_ISL_2444428	22/05/21	lorth America / Mexico / State of Mexic	Human	Female	41	unknown	Oropharyngeal swab
hCoV-19/Mexico/MEX-INMEGEN-05-05-47/2021	EPI_ISL_2444429	23/05/21	lorth America / Mexico / State of Mexic	Human	Male	44	unknown	Oropharyngeal swab
hCoV-19/Mexico/MEX-INMEGEN-05-05-50/2021	EPI_ISL_2444432	21/05/21	lorth America / Mexico / State of Mexic	Human	Male	18	unknown	Oropharyngeal swab
hCoV-19/Mexico/MEX-INMEGEN-05-05-58/2021	EPI_ISL_2444438	21/05/21	lorth America / Mexico / State of Mexic	Human	Male	58	unknown	Oropharyngeal swab
hCoV-19/Mexico/MEX-INMEGEN-05-05-72/2021	EPI_ISL_2444450	21/05/21	lorth America / Mexico / State of Mexic	Human	Male	52	unknown	Oropharyngeal swab
hCoV-19/Mexico/MEX-INMEGEN-05-05-96/2021	EPI_ISL_2550741	21/05/21	lorth America / Mexico / State of Mexic	Human	Male	24	unknown	Oropharyngeal swab
hCoV-19/Mexico/MEX-INMEGEN-05-06-193/2021	EPI_ISL_2603682	27/05/21	lorth America / Mexico / State of Mexic	Human	Male	31	unknown	Oropharyngeal swab
hCoV-19/Mexico/MEX-INMEGEN-05-06-218/2021	EPI_ISL_2881838	28/05/21	lorth America / Mexico / State of Mexic	Human	Male	31	unknown	Oropharyngeal swab
hCoV-19/Mexico/MEX-INMEGEN-05-06-234/2021	EPI_ISL_2603715	28/05/21	lorth America / Mexico / State of Mexic	Human	Female	34	unknown	Oropharyngeal swab
hCoV-19/Mexico/MEX-INMEGEN-05-06-235/2021	EPI_ISL_2603716	28/05/21	lorth America / Mexico / State of Mexic	Human	Male	54	unknown	Oropharyngeal swab
hCoV-19/Mexico/MEX-INMEGEN-05-06-250/2021	EPI_ISL_2603723	28/05/21	lorth America / Mexico / State of Mexic	Human	Male	32	unknown	Oropharyngeal swab
hCoV-19/Mexico/MEX-INMEGEN-05-06-310/2021	EPI_ISL_2603772	29/05/21	lorth America / Mexico / State of Mexic	Human	Female	40	unknown	Oropharyngeal swab
hCoV-19/Mexico/MEX-INMEGEN-05-06-336/2021	EPI_ISL_2603787	29/05/21	lorth America / Mexico / State of Mexic	Human	Male	49	unknown	Oropharyngeal swab

hCoV-19/Mexico/MEX-INMEGEN-05-06-340/2021	EPI_ISL_2603791	28/05/21	Iorth America / Mexico / State of Mexic	Human	Female	32	unknown	Oropharyngeal swab
hCoV-19/Mexico/MEX-INMEGEN-05-06-345/2021	EPI_ISL_2603796	29/05/21	Iorth America / Mexico / State of Mexic	Human	Female	31	unknown	Oropharyngeal swab
hCoV-19/Mexico/MEX-INMEGEN-05-06-356/2021	EPI_ISL_2603803	28/05/21	Iorth America / Mexico / State of Mexic	Human	Male	46	unknown	Oropharyngeal swab
hCoV-19/Mexico/MEX-INMEGEN-05-06-357/2021	EPI_ISL_2603804	28/05/21	Iorth America / Mexico / State of Mexic	Human	Female	54	unknown	Oropharyngeal swab
hCoV-19/Mexico/MEX-INMEGEN-06-01-10/2021	EPI_ISL_2616828	30/05/21	Iorth America / Mexico / State of Mexic	Human	Male	12	unknown	Oropharyngeal swab
hCoV-19/Mexico/MEX-INMEGEN-06-01-117/2021	EPI_ISL_2616849	02/06/21	Iorth America / Mexico / State of Mexic	Human	Male	6	unknown	Oropharyngeal swab
hCoV-19/Mexico/MEX-INMEGEN-06-01-148/2021	EPI_ISL_2616949	02/06/21	Iorth America / Mexico / State of Mexic	Human	Female	19	unknown	Oropharyngeal swab
hCoV-19/Mexico/MEX-INMEGEN-06-01-154/2021	EPI_ISL_2616888	02/06/21	Iorth America / Mexico / State of Mexic	Human	Female	52	unknown	Oropharyngeal swab
hCoV-19/Mexico/MEX-INMEGEN-06-01-25/2021	EPI_ISL_2616824	30/05/21	Iorth America / Mexico / State of Mexic	Human	Female	48	unknown	Oropharyngeal swab
hCoV-19/Mexico/MEX-INMEGEN-06-01-34/2021	EPI_ISL_2616978	31/05/21	Iorth America / Mexico / State of Mexic	Human	Male	44	unknown	Oropharyngeal swab
hCoV-19/Mexico/MEX-INMEGEN-06-01-77/2021	EPI_ISL_2616998	01/06/21	Iorth America / Mexico / State of Mexic	Human	Female	26	unknown	Oropharyngeal swab
hCoV-19/Mexico/MEX-INMEGEN-06-01-9/2021	EPI_ISL_2616850	30/05/21	Iorth America / Mexico / State of Mexic	Human	Male	16	unknown	Oropharyngeal swab
hCoV-19/Mexico/MEX-INMEGEN-06-02-194/2021	EPI_ISL_2778956	16/06/21	Iorth America / Mexico / State of Mexic	Human	Male	66	unknown	Oropharyngeal swab
hCoV-19/Mexico/MEX-INMEGEN-06-02-240/2021	EPI_ISL_2692750	16/06/21	Iorth America / Mexico / State of Mexic	Human	Male	32	unknown	Oropharyngeal swab
hCoV-19/Mexico/MEX-INMEGEN-06-02-293/2021	EPI_ISL_2692787	26/05/21	Iorth America / Mexico / State of Mexic	Human	Female	52	unknown	Oropharyngeal swab
hCoV-19/Mexico/MEX-INMEGEN-06-03-129/2021	EPI_ISL_2810121	03/06/21	Iorth America / Mexico / State of Mexic	Human	Female	37	unknown	Oropharyngeal swab
hCoV-19/Mexico/MEX-INMEGEN-06-03-135/2021	EPI_ISL_2810126	03/06/21	Iorth America / Mexico / State of Mexic	Human	Male	28	unknown	Oropharyngeal swab
hCoV-19/Mexico/MEX-INMEGEN-06-03-136/2021	EPI_ISL_2810127	03/06/21	Iorth America / Mexico / State of Mexic	Human	Female	34	unknown	Oropharyngeal swab
hCoV-19/Mexico/MEX-INMEGEN-06-03-159/2021	EPI_ISL_2810146	03/06/21	Iorth America / Mexico / State of Mexic	Human	Male	26	unknown	Oropharyngeal swab
hCoV-19/Mexico/MEX-INMEGEN-06-03-160/2021	EPI_ISL_2810147	03/06/21	Iorth America / Mexico / State of Mexic	Human	Male	15	unknown	Oropharyngeal swab
hCoV-19/Mexico/MEX-INMEGEN-06-03-185/2021	EPI_ISL_2810167	03/06/21	Iorth America / Mexico / State of Mexic	Human	Female	62	unknown	Oropharyngeal swab
hCoV-19/Mexico/MEX-INMEGEN-06-03-193/2021	EPI_ISL_2810174	22/06/21	Iorth America / Mexico / State of Mexic	Human	Female	67	unknown	Oropharyngeal swab
hCoV-19/Mexico/MEX-INMEGEN-06-03-62/2021	EPI_ISL_2810072	24/06/21	Iorth America / Mexico / State of Mexic	Human	Female	27	unknown	Oropharyngeal swab
hCoV-19/Mexico/MEX-INMEGEN-06-03-69/2021	EPI_ISL_2810078	24/06/21	Iorth America / Mexico / State of Mexic	Human	Female	26	unknown	Oropharyngeal swab
hCoV-19/Mexico/MEX-INMEGEN-06-04-375/2021	EPI_ISL_2894414	28/06/21	Iorth America / Mexico / State of Mexic	Human	Female	33	unknown	Oropharyngeal swab
hCoV-19/Mexico/MEX-INMEGEN-16-260/2021	EPI_ISL_2978574	05/07/21	Iorth America / Mexico / State of Mexic	Human	Male	16	unknown	Oropharyngeal swab
hCoV-19/Mexico/MEX-INMEGEN-16-262/2021	EPI_ISL_2978576	05/07/21	Iorth America / Mexico / State of Mexic	Human	Male	57	unknown	Oropharyngeal swab
hCoV-19/Mexico/MEX-INMEGEN-16-263/2021	EPI_ISL_2978577	05/07/21	Iorth America / Mexico / State of Mexic	Human	Female	23	unknown	Oropharyngeal swab
hCoV-19/Mexico/MEX-INMEGEN-16-99/2021	EPI_ISL_2978445	28/06/21	Iorth America / Mexico / State of Mexic	Human	Female	44	unknown	Oropharyngeal swab
hCoV-19/Mexico/MIC-InDRE_FB14989_S2053/2021	EPI_ISL_2283695	07/04/21	North America / Mexico / Michoacan	Human	Male	56	Hospitalized	Oropharyngeal swab
hCoV-19/Mexico/MIC-InDRE_FB14998_S2054/2021	EPI_ISL_2283696	13/04/21	North America / Mexico / Michoacan	Human	Male	30	unknown	Oropharyngeal swab
hCoV-19/Mexico/MIC-InDRE_FB18021_S3946/2021	EPI_ISL_2858960	18/06/21	North America / Mexico / Michoacan	Human	Male	57	Hospitalized	Oropharyngeal swab
hCoV-19/Mexico/MOR-InDRE_FB13639_S1731/2021	EPI_ISL_2101870	10/04/21	North America / Mexico / Morelos	Human	Male	64	Hospitalized	Oropharyngeal swab
hCoV-19/Mexico/MOR-InDRE_FB16889_S3115/2021	EPI_ISL_2663382	03/06/21	North America / Mexico / Morelos	Human	Male	32	Hospitalized	Oropharyngeal swab
hCoV-19/Mexico/NAY-InDRE_FB17385_S3480/2021	EPI_ISL_2778995	09/06/21	North America / Mexico / Nayarit	Human	Male	20	Hospitalized	Oropharyngeal swab
hCoV-19/Mexico/NLE-InDRE_237/2021	EPI_ISL_961467	18/01/21	North America / Mexico / Nuevo Leon	Human	Female	48	Hospitalized	Oropharyngeal swab
hCoV-19/Mexico/NLE-InDRE_238/2021	EPI_ISL_961468	18/01/21	North America / Mexico / Nuevo Leon	Human	Male	50	Hospitalized	Oropharyngeal swab
hCoV-19/Mexico/NLE-InDRE_240/2021	EPI_ISL_961469	18/01/21	North America / Mexico / Nuevo Leon	Human	Male	18	Hospitalized	Oropharyngeal swab
hCoV-19/Mexico/NLE-InDRE_F11681_S1199/2021	EPI_ISL_1626806	09/03/21	North America / Mexico / Nuevo Leon	Human	Female	17	Hospitalized	Oropharyngeal swab
hCoV-19/Mexico/NLE-InDRE_FB13119_S1506/2021	EPI_ISL_1805484	26/03/21	North America / Mexico / Nuevo Leon	Human	Male	55	unknown	Oropharyngeal swab
hCoV-19/Mexico/NLE-InDRE_FB13201_S1495/2021	EPI_ISL_1805473	05/04/21	North America / Mexico / Nuevo Leon	Human	Female	92	unknown	Oropharyngeal swab
hCoV-19/Mexico/NLE-InDRE_FB14845_S2084/2021	EPI_ISL_2283723	05/04/21	North America / Mexico / Nuevo Leon	Human	Female	41	Hospitalized	Oropharyngeal swab
hCoV-19/Mexico/NLE-InDRE_FB14848_S2085/2021	EPI_ISL_2283724	05/04/21	North America / Mexico / Nuevo Leon	Human	Female	23	Hospitalized	Oropharyngeal swab
hCoV-19/Mexico/NLE-InDRE_FB16177_S2461/2021	EPI_ISL_2455982	17/05/21	North America / Mexico / Nuevo Leon	Human	Male	69	Hospitalized	Oropharyngeal swab
hCoV-19/Mexico/NLE-InDRE_FB16180_S2462/2021	EPI_ISL_2455983	18/05/21	North America / Mexico / Nuevo Leon	Human	Male	65	Hospitalized	Oropharyngeal swab
hCoV-19/Mexico/NLE-InDRE_FB16187_S2463/2021	EPI_ISL_2455984	17/05/21	North America / Mexico / Nuevo Leon	Human	Female	44	Hospitalized	Oropharyngeal swab
hCoV-19/Mexico/NLE-InDRE_FB16191_S2464/2021	EPI_ISL_2455985	19/05/21	North America / Mexico / Nuevo Leon	Human	Male	24	Hospitalized	Oropharyngeal swab
hCoV-19/Mexico/NLE-InDRE_FB16492_S3060/2021	EPI_ISL_2663327	25/05/21	North America / Mexico / Nuevo Leon	Human	Male	48	Hospitalized	Oropharyngeal swab
hCoV-19/Mexico/NLE-InDRE_FB16950_S3122/2021	EPI_ISL_2663389	29/05/21	North America / Mexico / Nuevo Leon	Human	Male	46	Hospitalized	Oropharyngeal swab
hCoV-19/Mexico/NLE-InDRE_FB16953_S3123/2021	EPI_ISL_2663390	01/06/21	North America / Mexico / Nuevo Leon	Human	Female	38	Hospitalized	Oropharyngeal swab
hCoV-19/Mexico/NLE-InDRE_FB16961_S3124/2021	EPI_ISL_2663391	02/06/21	North America / Mexico / Nuevo Leon	Human	Female	39	Hospitalized	Oropharyngeal swab
hCoV-19/Mexico/NLE-InDRE_FB16964_S3125/2021	EPI_ISL_2663392	03/06/21	North America / Mexico / Nuevo Leon	Human	Male	53	Hospitalized	Oropharyngeal swab

hCoV-19/Mexico/NLE-InDRE_FB17360_S3921/2021	EPI_ISL_2858935	07/06/21	North America / Mexico / Nuevo Leon	Human		Female	22	Hospitalized	Oropharyngeal swab
hCoV-19/Mexico/NLE-InDRE_FB17366_S3922/2021	EPI_ISL_2858936	08/06/21	North America / Mexico / Nuevo Leon	Human		Male	50	Hospitalized	Oropharyngeal swab
hCoV-19/Mexico/NLE-InDRE_FB17368_S3923/2021	EPI_ISL_2858937	10/06/21	North America / Mexico / Nuevo Leon	Human		Female	40	Hospitalized	Oropharyngeal swab
hCoV-19/Mexico/NLE-InDRE_FB18082_S4116/2021	EPI_ISL_2920687	15/06/21	North America / Mexico / Nuevo Leon	Human		Female	71	Hospitalized	Oropharyngeal swab
hCoV-19/Mexico/NLE-InDRE_FB18085_S4117/2021	EPI_ISL_2920688	15/06/21	North America / Mexico / Nuevo Leon	Human		Male	30	Hospitalized	Oropharyngeal swab
hCoV-19/Mexico/NLE-InDRE_FB18090_S4118/2021	EPI_ISL_2920689	14/06/21	North America / Mexico / Nuevo Leon	Human		Female	56	Hospitalized	Oropharyngeal swab
hCoV-19/Mexico/NLE-InDRE_FB18093_S4119/2021	EPI_ISL_2920690	15/06/21	North America / Mexico / Nuevo Leon	Human		Female	25	Hospitalized	Oropharyngeal swab
hCoV-19/Mexico/NLE-InDRE_FB18094_S4120/2021	EPI_ISL_2920691	16/06/21	North America / Mexico / Nuevo Leon	Human		Female	22	Hospitalized	Oropharyngeal swab
hCoV-19/Mexico/NLE-InDRE_FB18095_S4121/2021	EPI_ISL_2920692	16/06/21	North America / Mexico / Nuevo Leon	Human		Female	45	Hospitalized	Oropharyngeal swab
hCoV-19/Mexico/NLE-InDRE_FB18317_S3969/2021	EPI_ISL_2858983	02/06/21	North America / Mexico / Nuevo Leon	Human		Male	53	Hospitalized	Oropharyngeal swab
hCoV-19/Mexico/NLE-LESPNL-20210407010/2021	EPI_ISL_1911302	13/03/21	North America / Mexico / Nuevo Leon	Human	"DR. BERNAI	Female	50	Hospitalized	Nasopharyngeal and/or oropharyngeal exudate
hCoV-19/Mexico/NLE-LESPNL-20210512-417/2021	EPI_ISL_2102612	05/05/21	North America / Mexico / Nuevo Leon	Human	› "Dr. Bernardc	Male	77	Ambulatory	Nasopharyngeal and/or oropharyngeal exudate
hCoV-19/Mexico/NLE-LESPNL-20210512-431/2021	EPI_ISL_2102626	30/04/21	North America / Mexico / Nuevo Leon	Human	U. Nueva Mor	Female	17	Ambulatory	Nasopharyngeal and/or oropharyngeal exudate
hCoV-19/Mexico/NLE-LESPNL-20210512-433/2021	EPI_ISL_2102628	07/05/21	North America / Mexico / Nuevo Leon	Human	U. Nueva Mor	Male	59	Ambulatory	Nasopharyngeal and/or oropharyngeal exudate
hCoV-19/Mexico/NLE-LESPNL-20210512-435/2021	EPI_ISL_2102630	06/05/21	North America / Mexico / Nuevo Leon	Human	U. Nueva Mor	Male	58	Ambulatory	Nasopharyngeal and/or oropharyngeal exudate
hCoV-19/Mexico/NLE-LESPNL-20210512-439/2021	EPI_ISL_2102633	06/05/21	North America / Mexico / Nuevo Leon	Human	U. Nueva Mor	Male	60	Ambulatory	Nasopharyngeal and/or oropharyngeal exudate
hCoV-19/Mexico/NLE-LESPNL-20210512-453/2021	EPI_ISL_2102645	02/05/21	North America / Mexico / Nuevo Leon	Human	Terminal	Male	41	Ambulatory	Nasopharyngeal and/or oropharyngeal exudate
hCoV-19/Mexico/NLE-LESPNL-20210512-454/2021	EPI_ISL_2102646	02/05/21	North America / Mexico / Nuevo Leon	Human	Terminal	Female	41	Ambulatory	Nasopharyngeal and/or oropharyngeal exudate
hCoV-19/Mexico/NLE-LESPNL-20210512-455/2021	EPI_ISL_2102647	03/05/21	North America / Mexico / Nuevo Leon	Human	Terminal	Male	75	Ambulatory	Nasopharyngeal and/or oropharyngeal exudate
hCoV-19/Mexico/NLE-LESPNL-20210512-458/2021	EPI_ISL_2102650	04/05/21	North America / Mexico / Nuevo Leon	Human	Terminal	Male	51	Ambulatory	Nasopharyngeal and/or oropharyngeal exudate
hCoV-19/Mexico/NLE-LESPNL-20210512-460/2021	EPI_ISL_2102652	05/05/21	North America / Mexico / Nuevo Leon	Human	Terminal	Male	32	Ambulatory	Nasopharyngeal and/or oropharyngeal exudate
hCoV-19/Mexico/NLE-UANL-067/2021	EPI_ISL_1789696	11/04/21	North America / Mexico / Nuevo Leon	Human		Male	19	Ambulatory	Oropharyngeal swab
hCoV-19/Mexico/NLE-UANL-068/2021	EPI_ISL_1789697	11/04/21	North America / Mexico / Nuevo Leon	Human		Male	16	Ambulatory	Oropharyngeal swab
hCoV-19/Mexico/NLE-UANL-075/2021	EPI_ISL_1789699	19/04/21	North America / Mexico / Nuevo Leon	Human		Male	40	Ambulatory	Oropharyngeal swab
hCoV-19/Mexico/OAX-InDRE_FB13216_S1507/2021	EPI_ISL_1805485	01/04/21	North America / Mexico / Oaxaca	Human		Male	29	unknown	Oropharyngeal swab
hCoV-19/Mexico/OAX-InDRE_FB13300_S1504/2021	EPI_ISL_1805482	08/04/21	North America / Mexico / Oaxaca	Human		Female	30	Hospitalized	Oropharyngeal swab
hCoV-19/Mexico/OAX-InDRE_FB14523_S2037/2021	EPI_ISL_2283681	27/03/21	North America / Mexico / Oaxaca	Human		Female	35	Hospitalized	Oropharyngeal swab
hCoV-19/Mexico/OAX-InDRE_FB14526_S2038/2021	EPI_ISL_2283682	31/03/21	North America / Mexico / Oaxaca	Human		Male	31	Hospitalized	Oropharyngeal swab
hCoV-19/Mexico/OAX-InDRE_FB14528_S2039/2021	EPI_ISL_2283683	12/04/21	North America / Mexico / Oaxaca	Human		Male	30	Hospitalized	Oropharyngeal swab
hCoV-19/Mexico/OAX-InDRE_FB14529_S2040/2021	EPI_ISL_2283684	15/04/21	North America / Mexico / Oaxaca	Human		Female	22	Hospitalized	Oropharyngeal swab
hCoV-19/Mexico/OAX-InDRE_FB14532_S2042/2021	EPI_ISL_2283685	22/04/21	North America / Mexico / Oaxaca	Human		Female	24	Hospitalized	Oropharyngeal swab
hCoV-19/Mexico/OAX-InDRE_FB14970_S2052/2021	EPI_ISL_2283694	08/05/21	North America / Mexico / Oaxaca	Human		Male	21	Hospitalized	Oropharyngeal swab
hCoV-19/Mexico/OAX-InDRE_FB15918_S2397/2021	EPI_ISL_2455922	13/05/21	North America / Mexico / Oaxaca	Human		Male	34	Hospitalized	Oropharyngeal swab
hCoV-19/Mexico/OAX-InDRE_FB17118_S3085/2021	EPI_ISL_2663352	16/05/21	North America / Mexico / Oaxaca	Human		Male	52	Hospitalized	Oropharyngeal swab
hCoV-19/Mexico/OAX-InDRE_FB18623_S4153/2021	EPI_ISL_2920724	19/06/21	North America / Mexico / Oaxaca	Human		Male	33	Hospitalized	Oropharyngeal swab
hCoV-19/Mexico/PUE-InDRE_FB16502_S2472/2021	EPI_ISL_2455993	27/04/21	North America / Mexico / Puebla	Human		Female	83	unknown	Oropharyngeal swab
hCoV-19/Mexico/PUE-InDRE_FB16507_S2473/2021	EPI_ISL_2455994	09/05/21	North America / Mexico / Puebla	Human		Male	39	unknown	Oropharyngeal swab
hCoV-19/Mexico/PUE-InDRE_FB17005_S3132/2021	EPI_ISL_2663399	02/06/21	North America / Mexico / Puebla	Human		Male	50	Hospitalized	Oropharyngeal swab
hCoV-19/Mexico/PUE-InDRE_FB17749_S3520/2021	EPI_ISL_2779035	11/06/21	North America / Mexico / Puebla	Human		Male	58	Deceased	Oropharyngeal swab
hCoV-19/Mexico/PUE-InDRE_FB17750_S3521/2021	EPI_ISL_2779036	11/06/21	North America / Mexico / Puebla	Human		Male	60	Deceased	Oropharyngeal swab
hCoV-19/Mexico/PUE-InDRE_FB17754_S3522/2021	EPI_ISL_2779037	11/06/21	North America / Mexico / Puebla	Human		Female	39	Hospitalized	Oropharyngeal swab
hCoV-19/Mexico/PUE-InDRE_FB17755_S3523/2021	EPI_ISL_2779038	11/06/21	North America / Mexico / Puebla	Human		Female	8	Hospitalized	Oropharyngeal swab
hCoV-19/Mexico/PUE-InDRE_FB17756_S3524/2021	EPI_ISL_2779039	11/06/21	North America / Mexico / Puebla	Human		Female	13	Hospitalized	Oropharyngeal swab
hCoV-19/Mexico/PUE-InDRE_FB17764_S3525/2021	EPI_ISL_2779040	11/06/21	North America / Mexico / Puebla	Human		Female	17	Hospitalized	Oropharyngeal swab
hCoV-19/Mexico/PUE-InDRE_FB17766_S3526/2021	EPI_ISL_2779041	11/06/21	North America / Mexico / Puebla	Human		Male	69	Hospitalized	Oropharyngeal swab
hCoV-19/Mexico/PUE-InDRE_FB17767_S3527/2021	EPI_ISL_2779042	11/06/21	North America / Mexico / Puebla	Human		Male	42	Hospitalized	Oropharyngeal swab
hCoV-19/Mexico/PUE-InDRE_FB19607_S4512/2021	EPI_ISL_3033365	16/06/21	North America / Mexico / Puebla	Human		Male	46	Hospitalized	Oropharyngeal swab
hCoV-19/Mexico/PUE-InDRE_FB19622_S4513/2021	EPI_ISL_3033366	21/06/21	North America / Mexico / Puebla	Human		Female	43	Hospitalized	Oropharyngeal swab
hCoV-19/Mexico/PUE-InDRE_FB19629_S4514/2021	EPI_ISL_3033367	22/06/21	North America / Mexico / Puebla	Human		Female	24	Hospitalized	Oropharyngeal swab
hCoV-19/Mexico/PUE-InDRE_FB19630_S4515/2021	EPI_ISL_3033368	22/06/21	North America / Mexico / Puebla	Human		Female	24	Hospitalized	Oropharyngeal swab
hCoV-19/Mexico/QUE-InDRE_F11371_S903/2021	EPI_ISL_1423513	05/03/21	North America / Mexico / Queretaro	Human		Female	76	Hospitalized	Oropharyngeal swab
hCoV-19/Mexico/QUE-InDRE_FB12849_S1487/2021	EPI_ISL_1805465	06/04/21	North America / Mexico / Queretaro	Human		Female	17	Hospitalized	Oropharyngeal swab

hCoV-19/Mexico/QUE-InDRE_FB14548_S2468/2021	EPI_ISL_2455989	01/05/21	North America / Mexico / Queretaro	Human	Female	41	Hospitalized	Oropharyngeal swab
hCoV-19/Mexico/QUE-InDRE_FB14550_S2080/2021	EPI_ISL_2283720	01/05/21	North America / Mexico / Queretaro	Human	Female	50	Hospitalized	Oropharyngeal swab
hCoV-19/Mexico/QUE-InDRE_FB15517_S2371/2021	EPI_ISL_2455896	20/04/21	North America / Mexico / Queretaro	Human	Female	33	Hospitalized	Oropharyngeal swab
hCoV-19/Mexico/QUE-InDRE_FB15562_S2372/2021	EPI_ISL_2455897	22/04/21	North America / Mexico / Queretaro	Human	Female	41	Hospitalized	Oropharyngeal swab
hCoV-19/Mexico/QUE-InDRE_FB15604_S2373/2021	EPI_ISL_2455898	25/04/21	North America / Mexico / Queretaro	Human	Female	40	Hospitalized	Oropharyngeal swab
hCoV-19/Mexico/QUE-InDRE_FB15641_S2374/2021	EPI_ISL_2455899	27/04/21	North America / Mexico / Queretaro	Human	Female	31	Hospitalized	Oropharyngeal swab
hCoV-19/Mexico/QUE-InDRE_FB15653_S2375/2021	EPI_ISL_2455900	27/04/21	North America / Mexico / Queretaro	Human	Female	36	Hospitalized	Oropharyngeal swab
hCoV-19/Mexico/QUE-InDRE_FB15659_S2376/2021	EPI_ISL_2455901	27/04/21	North America / Mexico / Queretaro	Human	Female	48	Hospitalized	Oropharyngeal swab
hCoV-19/Mexico/QUE-InDRE_FB15667_S2377/2021	EPI_ISL_2455902	27/04/21	North America / Mexico / Queretaro	Human	Male	50	Hospitalized	Oropharyngeal swab
hCoV-19/Mexico/QUE-InDRE_FB15699_S2378/2021	EPI_ISL_2455903	30/04/21	North America / Mexico / Queretaro	Human	Male	32	Hospitalized	Oropharyngeal swab
hCoV-19/Mexico/QUE-InDRE_FB15700_S2379/2021	EPI_ISL_2455904	01/05/21	North America / Mexico / Queretaro	Human	Female	41	Hospitalized	Oropharyngeal swab
hCoV-19/Mexico/QUE-InDRE_FB15725_S2380/2021	EPI_ISL_2455905	03/05/21	North America / Mexico / Queretaro	Human	Female	41	Hospitalized	Oropharyngeal swab
hCoV-19/Mexico/QUE-InDRE_FB15757_S2381/2021	EPI_ISL_2455906	04/05/21	North America / Mexico / Queretaro	Human	Female	35	Hospitalized	Oropharyngeal swab
hCoV-19/Mexico/QUE-InDRE_FB15774_S2382/2021	EPI_ISL_2455907	05/05/21	North America / Mexico / Queretaro	Human	Male	22	Hospitalized	Oropharyngeal swab
hCoV-19/Mexico/QUE-InDRE_FB15777_S2383/2021	EPI_ISL_2455908	05/05/21	North America / Mexico / Queretaro	Human	Male	57	Hospitalized	Oropharyngeal swab
hCoV-19/Mexico/QUE-InDRE_FB15789_S2384/2021	EPI_ISL_2455909	06/05/21	North America / Mexico / Queretaro	Human	Male	35	Hospitalized	Oropharyngeal swab
hCoV-19/Mexico/QUE-InDRE_FB15790_S2385/2021	EPI_ISL_2455910	06/05/21	North America / Mexico / Queretaro	Human	Female	32	Hospitalized	Oropharyngeal swab
hCoV-19/Mexico/QUE-InDRE_FB15807_S2386/2021	EPI_ISL_2455911	13/05/21	North America / Mexico / Queretaro	Human	Female	28	Hospitalized	Oropharyngeal swab
hCoV-19/Mexico/QUE-InDRE_FB15808_S2387/2021	EPI_ISL_2455912	14/05/21	North America / Mexico / Queretaro	Human	Female	15	Hospitalized	Oropharyngeal swab
hCoV-19/Mexico/QUE-InDRE_FB16146_S2439/2021	EPI_ISL_2455963	17/05/21	North America / Mexico / Queretaro	Human	Male	34	Hospitalized	Oropharyngeal swab
hCoV-19/Mexico/QUE-InDRE_FB16154_S2438/2021	EPI_ISL_2455962	19/05/21	North America / Mexico / Queretaro	Human	Female	27	Hospitalized	Oropharyngeal swab
hCoV-19/Mexico/QUE-InDRE_FB16156_S2437/2021	EPI_ISL_2455961	19/05/21	North America / Mexico / Queretaro	Human	Male	50	Hospitalized	Oropharyngeal swab
hCoV-19/Mexico/QUE-InDRE_FB16157_S2436/2021	EPI_ISL_2455960	19/05/21	North America / Mexico / Queretaro	Human	Female	47	Hospitalized	Oropharyngeal swab
hCoV-19/Mexico/QUE-InDRE_FB16164_S2460/2021	EPI_ISL_2455981	17/05/21	North America / Mexico / Queretaro	Human	Female	17	Hospitalized	Oropharyngeal swab
hCoV-19/Mexico/QUE-InDRE_FB16431_S2481/2021	EPI_ISL_2456002	24/05/21	North America / Mexico / Queretaro	Human	Male	48	unknown	Oropharyngeal swab
hCoV-19/Mexico/QUE-InDRE_FB16434_S2738/2021	EPI_ISL_2533764	25/05/21	North America / Mexico / Queretaro	Human	Female	33	Hospitalized	Oropharyngeal swab
hCoV-19/Mexico/QUE-InDRE_FB16436_S2739/2021	EPI_ISL_2533765	20/05/21	North America / Mexico / Queretaro	Human	Female	49	Hospitalized	Oropharyngeal swab
hCoV-19/Mexico/QUE-InDRE_FB16437_S2740/2021	EPI_ISL_2533766	26/05/21	North America / Mexico / Queretaro	Human	Female	52	Hospitalized	Oropharyngeal swab
hCoV-19/Mexico/QUE-InDRE_FB16868_S3113/2021	EPI_ISL_2663380	30/05/21	North America / Mexico / Queretaro	Human	Male	64	Hospitalized	Oropharyngeal swab
hCoV-19/Mexico/QUE-InDRE_FB16876_S3114/2021	EPI_ISL_2663381	04/06/21	North America / Mexico / Queretaro	Human	Male	49	Hospitalized	Oropharyngeal swab
hCoV-19/Mexico/QUE-InDRE_FB16879_S3064/2021	EPI_ISL_2709686	01/06/21	North America / Mexico / Queretaro	Human	Male	55	Hospitalized	Oropharyngeal swab
hCoV-19/Mexico/QUE-InDRE_FB16881_S3065/2021	EPI_ISL_2663331	03/06/21	North America / Mexico / Queretaro	Human	Female	43	Hospitalized	Oropharyngeal swab
hCoV-19/Mexico/QUE-InDRE_FB16884_S3066/2021	EPI_ISL_2663332	04/06/21	North America / Mexico / Queretaro	Human	Female	53	Hospitalized	Oropharyngeal swab
hCoV-19/Mexico/QUE-InDRE_FB17253_S3534/2021	EPI_ISL_2779049	10/06/21	North America / Mexico / Queretaro	Human	Male	12	Hospitalized	Oropharyngeal swab
hCoV-19/Mexico/QUE-InDRE_FB17258_S3109/2021	EPI_ISL_2663376	14/02/21	North America / Mexico / Queretaro	Human	Male	59	Hospitalized	Oropharyngeal swab
hCoV-19/Mexico/QUE-InDRE_FB17259_S3110/2021	EPI_ISL_2663377	10/06/21	North America / Mexico / Queretaro	Human	Female	31	Hospitalized	Oropharyngeal swab
hCoV-19/Mexico/QUE-InDRE_FB18359_S4142/2021	EPI_ISL_2920713	25/06/21	North America / Mexico / Queretaro	Human	Female	15	Hospitalized	Oropharyngeal swab
hCoV-19/Mexico/ROO-InDRE_FB12917_S1492/2021	EPI_ISL_1805470	02/04/21	North America / Mexico / Quintana Roc	Human	Male	53	Hospitalized	Oropharyngeal swab
hCoV-19/Mexico/ROO-InDRE_FB13273_S1503/2021	EPI_ISL_1805481	06/04/21	North America / Mexico / Quintana Roc	Human	Male	53	Deceased	Oropharyngeal swab
hCoV-19/Mexico/ROO-InDRE_FB13727_S1740/2021	EPI_ISL_2101879	17/04/21	North America / Mexico / Quintana Roc	Human	Female	43	Hospitalized	Oropharyngeal swab
hCoV-19/Mexico/ROO-InDRE_FB14100_S1759/2021	EPI_ISL_2101898	21/04/21	North America / Mexico / Quintana Roc	Human	Female	74	Hospitalized	Oropharyngeal swab
hCoV-19/Mexico/ROO-InDRE_FB14101_S1760/2021	EPI_ISL_2101899	20/04/21	North America / Mexico / Quintana Roc	Human	Male	85	Deceased	Oropharyngeal swab
hCoV-19/Mexico/ROO-InDRE_FB16369_S2476/2021	EPI_ISL_2455997	15/05/21	North America / Mexico / Quintana Roc	Human	Female	46	unknown	Oropharyngeal swab
hCoV-19/Mexico/ROO-InDRE_FB16793_S2778/2021	EPI_ISL_2533804	26/05/21	North America / Mexico / Quintana Roc	Human	Male	58	Hospitalized	Oropharyngeal swab
hCoV-19/Mexico/ROO-InDRE_FB16795_S2779/2021	EPI_ISL_2533805	26/05/21	North America / Mexico / Quintana Roc	Human	Female	46	Hospitalized	Oropharyngeal swab
hCoV-19/Mexico/ROO-InDRE_FB17190_S3089/2021	EPI_ISL_2663356	03/06/21	North America / Mexico / Quintana Roc	Human	Male	42	Hospitalized	Oropharyngeal swab
hCoV-19/Mexico/ROO-InDRE_FB17192_S3090/2021	EPI_ISL_2663357	01/06/21	North America / Mexico / Quintana Roc	Human	Female	30	Hospitalized	Oropharyngeal swab
hCoV-19/Mexico/ROO-InDRE_FB19165_S4499/2021	EPI_ISL_3033353	21/06/21	North America / Mexico / Quintana Roc	Human	Male	55	Hospitalized	Oropharyngeal swab
hCoV-19/Mexico/ROO-INMEGEN-05-06-205/2021	EPI_ISL_2603694	01/06/21	North America / Mexico / Quintana Roc	Human	Female	46	unknown	Oropharyngeal swab
hCoV-19/Mexico/ROO-INMEGEN-05-06-212/2021	EPI_ISL_2603700	01/06/21	North America / Mexico / Quintana Roc	Human	Male	31	unknown	Oropharyngeal swab
hCoV-19/Mexico/ROO-INMEGEN-05-06-268/2021	EPI_ISL_2603738	31/05/21	North America / Mexico / Quintana Roc	Human	Male	34	unknown	Oropharyngeal swab
hCoV-19/Mexico/ROO-INMEGEN-06-01-197/2021	EPI_ISL_2616800	02/06/21	North America / Mexico / Quintana Roc	Human	Female	39	unknown	Oropharyngeal swab

hCoV-19/Mexico/ROO-INMEGEN-06-01-205/2021	EPI_ISL_2616938	01/06/21	North America / Mexico / Quintana Roo	Human	Male	27	unknown	Oropharyngeal swab
hCoV-19/Mexico/ROO-INMEGEN-06-01-212/2021	EPI_ISL_2616851	02/06/21	North America / Mexico / Quintana Roo	Human	Male	42	unknown	Oropharyngeal swab
hCoV-19/Mexico/ROO-INMEGEN-06-01-220/2021	EPI_ISL_2616979	02/06/21	North America / Mexico / Quintana Roo	Human	Male	51	unknown	Oropharyngeal swab
hCoV-19/Mexico/ROO-INMEGEN-06-01-224/2021	EPI_ISL_2616981	02/06/21	North America / Mexico / Quintana Roo	Human	Female	38	unknown	Oropharyngeal swab
hCoV-19/Mexico/ROO-INMEGEN-06-01-233/2021	EPI_ISL_2616887	03/06/21	North America / Mexico / Quintana Roo	Human	Male	52	unknown	Oropharyngeal swab
hCoV-19/Mexico/ROO-INMEGEN-06-01-241/2021	EPI_ISL_2616867	03/06/21	North America / Mexico / Quintana Roo	Human	Male	35	unknown	Oropharyngeal swab
hCoV-19/Mexico/ROO-INMEGEN-06-01-253/2021	EPI_ISL_2616898	01/06/21	North America / Mexico / Quintana Roo	Human	Female	37	unknown	Oropharyngeal swab
hCoV-19/Mexico/ROO-INMEGEN-06-01-268/2021	EPI_ISL_2616994	02/06/21	North America / Mexico / Quintana Roo	Human	Male	38	unknown	Oropharyngeal swab
hCoV-19/Mexico/ROO-INMEGEN-06-01-301/2021	EPI_ISL_2616969	03/06/21	North America / Mexico / Quintana Roo	Human	Female	11	unknown	Oropharyngeal swab
hCoV-19/Mexico/ROO-INMEGEN-06-01-309/2021	EPI_ISL_2616841	03/06/21	North America / Mexico / Quintana Roo	Human	Female	39	unknown	Oropharyngeal swab
hCoV-19/Mexico/ROO-INMEGEN-06-01-322/2021	EPI_ISL_2616965	03/06/21	North America / Mexico / Quintana Roo	Human	Female	28	unknown	Oropharyngeal swab
hCoV-19/Mexico/ROO-INMEGEN-06-01-342/2021	EPI_ISL_2616963	04/06/21	North America / Mexico / Quintana Roo	Human	Male	53	unknown	Oropharyngeal swab
hCoV-19/Mexico/ROO-INMEGEN-06-01-353/2021	EPI_ISL_2616971	05/06/21	North America / Mexico / Quintana Roo	Human	Male	38	unknown	Oropharyngeal swab
hCoV-19/Mexico/ROO-INMEGEN-06-02-104/2021	EPI_ISL_2692631	07/06/21	North America / Mexico / Quintana Roo	Human	Male	22	unknown	Oropharyngeal swab
hCoV-19/Mexico/ROO-INMEGEN-06-02-106/2021	EPI_ISL_2692633	08/06/21	North America / Mexico / Quintana Roo	Human	Male	48	unknown	Oropharyngeal swab
hCoV-19/Mexico/ROO-INMEGEN-06-02-130/2021	EPI_ISL_2692651	08/06/21	North America / Mexico / Quintana Roo	Human	Female	40	unknown	Oropharyngeal swab
hCoV-19/Mexico/ROO-INMEGEN-06-02-146/2021	EPI_ISL_2692665	08/06/21	North America / Mexico / Quintana Roo	Human	Female	38	unknown	Oropharyngeal swab
hCoV-19/Mexico/ROO-INMEGEN-06-02-183/2021	EPI_ISL_2692696	07/06/21	North America / Mexico / Quintana Roo	Human	Male	43	unknown	Oropharyngeal swab
hCoV-19/Mexico/ROO-INMEGEN-06-02-320/2021	EPI_ISL_2692807	01/06/21	North America / Mexico / Quintana Roo	Human	Male	30	unknown	Oropharyngeal swab
hCoV-19/Mexico/ROO-INMEGEN-06-02-40/2021	EPI_ISL_2692575	05/06/21	North America / Mexico / Quintana Roo	Human	Female	35	unknown	Oropharyngeal swab
hCoV-19/Mexico/ROO-INMEGEN-06-02-58/2021	EPI_ISL_2692591	06/06/21	North America / Mexico / Quintana Roo	Human	Male	47	unknown	Oropharyngeal swab
hCoV-19/Mexico/ROO-INMEGEN-06-02-66/2021	EPI_ISL_2692598	06/06/21	North America / Mexico / Quintana Roo	Human	Female	30	unknown	Oropharyngeal swab
hCoV-19/Mexico/ROO-INMEGEN-06-02-69/2021	EPI_ISL_2692601	07/06/21	North America / Mexico / Quintana Roo	Human	Female	45	unknown	Oropharyngeal swab
hCoV-19/Mexico/ROO-INMEGEN-06-02-7/2021	EPI_ISL_2692545	07/06/21	North America / Mexico / Quintana Roo	Human	Female	42	unknown	Oropharyngeal swab
hCoV-19/Mexico/ROO-INMEGEN-06-02-77/2021	EPI_ISL_2692607	07/06/21	North America / Mexico / Quintana Roo	Human	Male	19	unknown	Oropharyngeal swab
hCoV-19/Mexico/ROO-INMEGEN-06-02-82/2021	EPI_ISL_2692612	05/06/21	North America / Mexico / Quintana Roo	Human	Male	29	unknown	Oropharyngeal swab
hCoV-19/Mexico/ROO-INMEGEN-06-02-99/2021	EPI_ISL_2692627	08/06/21	North America / Mexico / Quintana Roo	Human	Female	39	unknown	Oropharyngeal swab
hCoV-19/Mexico/ROO-INMEGEN-06-03-289/2021	EPI_ISL_2810251	09/06/21	North America / Mexico / Quintana Roo	Human	Male	23	unknown	Oropharyngeal swab
hCoV-19/Mexico/ROO-INMEGEN-06-03-296/2021	EPI_ISL_2810258	08/06/21	North America / Mexico / Quintana Roo	Human	Male	54	unknown	Oropharyngeal swab
hCoV-19/Mexico/ROO-INMEGEN-06-03-320/2021	EPI_ISL_2810278	09/06/21	North America / Mexico / Quintana Roo	Human	Male	50	unknown	Oropharyngeal swab
hCoV-19/Mexico/ROO-INMEGEN-06-04-14/2021	EPI_ISL_2894164	10/06/21	North America / Mexico / Quintana Roo	Human	Male	26	unknown	Oropharyngeal swab
hCoV-19/Mexico/ROO-INMEGEN-06-04-313/2021	EPI_ISL_2894360	11/06/21	North America / Mexico / Quintana Roo	Human	Female	27	unknown	Oropharyngeal swab
hCoV-19/Mexico/ROO-INMEGEN-06-04-316/2021	EPI_ISL_2894363	11/06/21	North America / Mexico / Quintana Roo	Human	Female	55	unknown	Oropharyngeal swab
hCoV-19/Mexico/ROO-INMEGEN-06-04-317/2021	EPI_ISL_2894364	11/06/21	North America / Mexico / Quintana Roo	Human	Female	32	unknown	Oropharyngeal swab
hCoV-19/Mexico/ROO-INMEGEN-06-04-320/2021	EPI_ISL_2894366	11/06/21	North America / Mexico / Quintana Roo	Human	Female	41	unknown	Oropharyngeal swab
hCoV-19/Mexico/ROO-INMEGEN-06-04-321/2021	EPI_ISL_2894367	12/06/21	North America / Mexico / Quintana Roo	Human	Female	32	unknown	Oropharyngeal swab
hCoV-19/Mexico/ROO-INMEGEN-06-04-322/2021	EPI_ISL_2894368	12/06/21	North America / Mexico / Quintana Roo	Human	Male	46	unknown	Oropharyngeal swab
hCoV-19/Mexico/ROO-INMEGEN-06-04-323/2021	EPI_ISL_2894369	12/06/21	North America / Mexico / Quintana Roo	Human	Male	43	unknown	Oropharyngeal swab
hCoV-19/Mexico/ROO-INMEGEN-06-04-54/2021	EPI_ISL_2894198	10/06/21	North America / Mexico / Quintana Roo	Human	Male	29	unknown	Oropharyngeal swab
hCoV-19/Mexico/ROO-INMEGEN-06-04-62/2021	EPI_ISL_2894206	10/06/21	North America / Mexico / Quintana Roo	Human	Female	75	unknown	Oropharyngeal swab
hCoV-19/Mexico/ROO-INMEGEN-06-04-7/2021	EPI_ISL_2894157	10/06/21	North America / Mexico / Quintana Roo	Human	Male	31	unknown	Oropharyngeal swab
hCoV-19/Mexico/ROO-INMEGEN-16-288/2021	EPI_ISL_2978599	22/06/21	North America / Mexico / Quintana Roo	Human	Male	23	unknown	Oropharyngeal swab
hCoV-19/Mexico/ROO-INMEGEN-16-295/2021	EPI_ISL_2978606	22/06/21	North America / Mexico / Quintana Roo	Human	Male	51	unknown	Oropharyngeal swab
hCoV-19/Mexico/ROO-INMEGEN-16-319/2021	EPI_ISL_2978626	23/06/21	North America / Mexico / Quintana Roo	Human	Female	14	unknown	Oropharyngeal swab
hCoV-19/Mexico/ROO-INMEGEN-16-350/2021	EPI_ISL_2978654	23/06/21	North America / Mexico / Quintana Roo	Human	Male	28	unknown	Oropharyngeal swab
hCoV-19/Mexico/SEARCH-101109/2021	EPI_ISL_2329458	05/05/21	America / Mexico / Baja California / Tij	Human	unknown	unknown	unknown	Nasopharyngeal swab
hCoV-19/Mexico/SEARCH-101405/2021	EPI_ISL_2422818	10/05/21	America / Mexico / Baja California / Tij	Human	unknown	unknown	unknown	Oropharyngeal swab
hCoV-19/Mexico/SEARCH-101440/2021	EPI_ISL_2422832	11/05/21	America / Mexico / Baja California / Tij	Human	unknown	unknown	unknown	Oropharyngeal swab
hCoV-19/Mexico/SIN_CIAD_HJ1385/2021	EPI_ISL_2026411	05/04/21	rth America / Mexico / Sinaloa / Mazatl	Human	Male	62	unknown	Oropharyngeal swab
hCoV-19/Mexico/SIN_CIAD_HJ1490/2021	EPI_ISL_2026413	19/04/21	rth America / Mexico / Sinaloa / Mazatl	Human	Male	57	unknown	Oropharyngeal swab
hCoV-19/Mexico/SIN_CIAD_HJ1500/2021	EPI_ISL_2026415	19/04/21	rth America / Mexico / Sinaloa / Mazatl	Human	Male	48	unknown	Oropharyngeal swab
hCoV-19/Mexico/SIN_CIAD_HJ1512/2021	EPI_ISL_2026417	20/04/21	rth America / Mexico / Sinaloa / Mazatl	Human	Female	47	unknown	Oropharyngeal swab

hCoV-19/Mexico/SIN_CIAD_HJ1592/2021	EPI_ISL_2249239	04/05/21	rth America / Mexico / Sinaloa / Mazatl	Human	Male	38	unknown	Oropharyngeal swab
hCoV-19/Mexico/SIN_CIAD_HJ1599/2021	EPI_ISL_2249241	06/05/21	rth America / Mexico / Sinaloa / Mazatl	Human	Male	52	unknown	Oropharyngeal swab
hCoV-19/Mexico/SIN_CIAD_HJ1602/2021	EPI_ISL_2249242	06/05/21	rth America / Mexico / Sinaloa / Mazatl	Human	Male	37	unknown	Oropharyngeal swab
hCoV-19/Mexico/SIN_CIAD_HJ1603/2021	EPI_ISL_2249243	06/05/21	rth America / Mexico / Sinaloa / Mazatl	Human	Female	54	unknown	Oropharyngeal swab
hCoV-19/Mexico/SIN_CIAD_HJ1616/2021	EPI_ISL_2249254	10/05/21	rth America / Mexico / Sinaloa / Mazatl	Human	Female	61	unknown	Oropharyngeal swab
hCoV-19/Mexico/SIN_CIAD_ID2302/2021	EPI_ISL_2249234	03/05/21	rth America / Mexico / Sinaloa / Mazatl	Human	Female	75	unknown	Oropharyngeal swab
hCoV-19/Mexico/SIN_CIAD_S6572/2021	EPI_ISL_2026422	15/04/21	rth America / Mexico / Sinaloa / Mazatl	Human	Female	16	unknown	Oropharyngeal swab
hCoV-19/Mexico/SIN_CIAD_S6600/2021	EPI_ISL_2026432	20/04/21	rth America / Mexico / Sinaloa / Mazatl	Human	Male	43	unknown	Oropharyngeal swab
hCoV-19/Mexico/SIN_CIAD_S6625/2021	EPI_ISL_2026420	22/04/21	rth America / Mexico / Sinaloa / Mazatl	Human	Male	57	unknown	Oropharyngeal swab
hCoV-19/Mexico/SIN_CIAD_S6643/2021	EPI_ISL_2026418	26/04/21	rth America / Mexico / Sinaloa / Mazatl	Human	Female	57	unknown	Oropharyngeal swab
hCoV-19/Mexico/SIN_CIAD_S6662/2021	EPI_ISL_2249230	28/04/21	rth America / Mexico / Sinaloa / Mazatl	Human	Male	50	unknown	Oropharyngeal swab
hCoV-19/Mexico/SIN_CIAD_S6698/2021	EPI_ISL_2249236	03/05/21	rth America / Mexico / Sinaloa / Mazatl	Human	Male	23	unknown	Oropharyngeal swab
hCoV-19/Mexico/SIN_CIAD_S6699/2021	EPI_ISL_2277642	03/05/21	rth America / Mexico / Sinaloa / Mazatl	Human	Female	68	unknown	Oropharyngeal swab
hCoV-19/Mexico/SIN_CIAD_S6706/2021	EPI_ISL_2249240	04/05/21	rth America / Mexico / Sinaloa / Mazatl	Human	Male	52	unknown	Oropharyngeal swab
hCoV-19/Mexico/SIN_CIAD_S6741/2021	EPI_ISL_2249251	07/05/21	rth America / Mexico / Sinaloa / Mazatl	Human	Female	79	unknown	Oropharyngeal swab
hCoV-19/Mexico/SIN-InDRE_FB14031_S2069/2021	EPI_ISL_2283709	24/04/21	North America / Mexico / Sinaloa	Human	Female	19	Hospitalized	Oropharyngeal swab
hCoV-19/Mexico/SIN-InDRE_FB14032_S2070/2021	EPI_ISL_2283710	22/04/21	North America / Mexico / Sinaloa	Human	Male	26	Hospitalized	Oropharyngeal swab
hCoV-19/Mexico/SIN-InDRE_FB14033_S1757/2021	EPI_ISL_2101896	23/04/21	North America / Mexico / Sinaloa	Human	Male	21	Hospitalized	Oropharyngeal swab
hCoV-19/Mexico/SIN-InDRE_FB15070_S2058/2021	EPI_ISL_2283700	03/05/21	North America / Mexico / Sinaloa	Human	Male	59	Hospitalized	Oropharyngeal swab
hCoV-19/Mexico/SIN-InDRE_FB15071_S2059/2021	EPI_ISL_2283701	03/05/21	North America / Mexico / Sinaloa	Human	Female	29	Hospitalized	Oropharyngeal swab
hCoV-19/Mexico/SIN-InDRE_FB15074_S2060/2021	EPI_ISL_2283702	30/04/21	North America / Mexico / Sinaloa	Human	Female	57	Deceased	Oropharyngeal swab
hCoV-19/Mexico/SIN-InDRE_FB15075_S2061/2021	EPI_ISL_2283703	05/05/21	North America / Mexico / Sinaloa	Human	Male	19	Hospitalized	Oropharyngeal swab
hCoV-19/Mexico/SIN-InDRE_FB16917_S3067/2021	EPI_ISL_2663333	31/05/21	North America / Mexico / Sinaloa	Human	Male	58	Hospitalized	Oropharyngeal swab
hCoV-19/Mexico/SIN-InDRE_FB16918_S3068/2021	EPI_ISL_2663334	02/06/21	North America / Mexico / Sinaloa	Human	Female	42	Hospitalized	Oropharyngeal swab
hCoV-19/Mexico/SIN-InDRE_FB17388_S3483/2021	EPI_ISL_2778998	09/06/21	North America / Mexico / Sinaloa	Human	Male	31	Hospitalized	Oropharyngeal swab
hCoV-19/Mexico/SIN-InDRE_FB17473_S3490/2021	EPI_ISL_2779005	08/06/21	North America / Mexico / Sinaloa	Human	Male	63	Hospitalized	Oropharyngeal swab
hCoV-19/Mexico/SIN-InDRE_FB17477_S3491/2021	EPI_ISL_2779006	08/06/21	North America / Mexico / Sinaloa	Human	Female	36	Hospitalized	Oropharyngeal swab
hCoV-19/Mexico/SIN-InDRE_FB17478_S3492/2021	EPI_ISL_2779007	08/06/21	North America / Mexico / Sinaloa	Human	Male	11	Hospitalized	Oropharyngeal swab
hCoV-19/Mexico/SIN-InDRE_FB18120_S3959/2021	EPI_ISL_2858973	16/06/21	North America / Mexico / Sinaloa	Human	Female	71	Hospitalized	Oropharyngeal swab
hCoV-19/Mexico/SIN-InDRE_FB19379_S4505/2021	EPI_ISL_3033359	25/06/21	North America / Mexico / Sinaloa	Human	Female	41	Deceased	Oropharyngeal swab
hCoV-19/Mexico/SIN-InDRE_FB19380_S4506/2021	EPI_ISL_3033360	26/06/21	North America / Mexico / Sinaloa	Human	Female	29	Hospitalized	Oropharyngeal swab
hCoV-19/Mexico/SLP-InDRE_FB16030_S2429/2021	EPI_ISL_2455953	17/05/21	lorth America / Mexico / San Luis Potos	Human	Female	32	Hospitalized	Oropharyngeal swab
hCoV-19/Mexico/SLP-InDRE_FB16823_S2780/2021	EPI_ISL_2533806	19/05/21	lorth America / Mexico / San Luis Potos	Human	Male	45	Hospitalized	Oropharyngeal swab
hCoV-19/Mexico/SLP-InDRE_FB18337_S3973/2021	EPI_ISL_2858987	05/06/21	lorth America / Mexico / San Luis Potos	Human	Female	44	Hospitalized	Oropharyngeal swab
hCoV-19/Mexico/SLP-InDRE_FB18338_S3974/2021	EPI_ISL_2858988	07/06/21	lorth America / Mexico / San Luis Potos	Human	Female	52	Hospitalized	Oropharyngeal swab
hCoV-19/Mexico/SLP-InDRE_FB18343_S3975/2021	EPI_ISL_2858989	16/06/21	lorth America / Mexico / San Luis Potos	Human	Female	14	Hospitalized	Oropharyngeal swab
hCoV-19/Mexico/SLP-InDRE_FB18344_S3976/2021	EPI_ISL_2858990	19/06/21	lorth America / Mexico / San Luis Potos	Human	Female	23	Hospitalized	Oropharyngeal swab
hCoV-19/Mexico/SON-H-3296/2021	EPI_ISL_2894601	30/06/21	North America / Mexico / Sonora	Human	Female	19	unknown	Oropharyngeal swab
hCoV-19/Mexico/SON-InDRE_FB13111_S1494/2021	EPI_ISL_1805472	06/04/21	North America / Mexico / Sonora	Human	Male	58	Hospitalized	Oropharyngeal swab
hCoV-19/Mexico/SON-InDRE_FB14085_S1758/2021	EPI_ISL_2101897	25/04/21	North America / Mexico / Sonora	Human	Male	59	Deceased	Oropharyngeal swab
hCoV-19/Mexico/SON-InDRE_FB16350_S3474/2021	EPI_ISL_2778989	23/05/21	North America / Mexico / Sonora	Human	Female	45	Hospitalized	Oropharyngeal swab
hCoV-19/Mexico/SON-InDRE_FB17150_S3136/2021	EPI_ISL_2663403	30/05/21	North America / Mexico / Sonora	Human	Male	20	Hospitalized	Oropharyngeal swab
hCoV-19/Mexico/SON-InDRE_FB17167_S3092/2021	EPI_ISL_2663359	03/06/21	North America / Mexico / Sonora	Human	Male	33	Hospitalized	Oropharyngeal swab
hCoV-19/Mexico/SON-InDRE_FB17171_S3087/2021	EPI_ISL_2663354	04/06/21	North America / Mexico / Sonora	Human	Female	60	Hospitalized	Oropharyngeal swab
hCoV-19/Mexico/SON-InDRE_FB17175_S3088/2021	EPI_ISL_2663355	30/05/21	North America / Mexico / Sonora	Human	Male	33	Hospitalized	Oropharyngeal swab
hCoV-19/Mexico/SON-InDRE_FB18023_S3947/2021	EPI_ISL_2858961	15/06/21	North America / Mexico / Sonora	Human	Female	51	Hospitalized	Oropharyngeal swab
hCoV-19/Mexico/SON-InDRE_FB18274_S4135/2021	EPI_ISL_2920706	17/06/21	North America / Mexico / Sonora	Human	Male	16	Hospitalized	Oropharyngeal swab
hCoV-19/Mexico/SON-InDRE_FB18291_S3967/2021	EPI_ISL_2858981	14/06/21	North America / Mexico / Sonora	Human	Female	82	Hospitalized	Oropharyngeal swab
hCoV-19/Mexico/SON-InDRE_FB18293_S3968/2021	EPI_ISL_2858982	17/06/21	North America / Mexico / Sonora	Human	Female	63	Hospitalized	Oropharyngeal swab
hCoV-19/Mexico/TAB-InDRE_FB16513_S2742/2021	EPI_ISL_2533768	29/05/21	North America / Mexico / Tabasco	Human	Male	67	Hospitalized	Oropharyngeal swab
hCoV-19/Mexico/TAB-InDRE_FB16514_S2743/2021	EPI_ISL_2533769	29/05/21	North America / Mexico / Tabasco	Human	Female	73	Hospitalized	Oropharyngeal swab
hCoV-19/Mexico/TAB-InDRE_FB16926_S3069/2021	EPI_ISL_2663336	01/06/21	North America / Mexico / Tabasco	Human	Female	43	Hospitalized	Oropharyngeal swab

hCoV-19/Mexico/TAB-InDRE_FB16929_S3070/2021	EPI_ISL_2663337	01/06/21	North America / Mexico / Tabasco	Human	Female	64	Hospitalized	Oropharyngeal swab
hCoV-19/Mexico/TAB-InDRE_FB16931_S3071/2021	EPI_ISL_2663338	02/06/21	North America / Mexico / Tabasco	Human	Female	61	Hospitalized	Oropharyngeal swab
hCoV-19/Mexico/TAB-InDRE_FB16944_S3072/2021	EPI_ISL_2663339	03/06/21	North America / Mexico / Tabasco	Human	Male	75	Hospitalized	Oropharyngeal swab
hCoV-19/Mexico/TAB-InDRE_FB16947_S3073/2021	EPI_ISL_2663340	03/06/21	North America / Mexico / Tabasco	Human	Male	34	Hospitalized	Oropharyngeal swab
hCoV-19/Mexico/TAB-InDRE_FB16948_S3074/2021	EPI_ISL_2663341	03/06/21	North America / Mexico / Tabasco	Human	Male	19	Hospitalized	Oropharyngeal swab
hCoV-19/Mexico/TAB-InDRE_FB17013_S3079/2021	EPI_ISL_2663346	04/06/21	North America / Mexico / Tabasco	Human	Male	71	Hospitalized	Oropharyngeal swab
hCoV-19/Mexico/TAB-InDRE_FB17014_S3080/2021	EPI_ISL_2663347	04/06/21	North America / Mexico / Tabasco	Human	Female	25	Hospitalized	Oropharyngeal swab
hCoV-19/Mexico/TAB-InDRE_FB17020_S3081/2021	EPI_ISL_2663348	05/06/21	North America / Mexico / Tabasco	Human	Female	51	Hospitalized	Oropharyngeal swab
hCoV-19/Mexico/TAB-InDRE_FB17030_S3084/2021	EPI_ISL_2663351	07/06/21	North America / Mexico / Tabasco	Human	Female	49	unknown	Oropharyngeal swab
hCoV-19/Mexico/TAB-InDRE_FB17043_S3082/2021	EPI_ISL_2663349	07/06/21	North America / Mexico / Tabasco	Human	Female	2	Hospitalized	Oropharyngeal swab
hCoV-19/Mexico/TAB-InDRE_FB17298_S3469/2021	EPI_ISL_2778984	09/06/21	North America / Mexico / Tabasco	Human	Female	51	Hospitalized	Oropharyngeal swab
hCoV-19/Mexico/TAB-InDRE_FB17299_S3470/2021	EPI_ISL_2778985	09/06/21	North America / Mexico / Tabasco	Human	Male	73	Hospitalized	Oropharyngeal swab
hCoV-19/Mexico/TAB-InDRE_FB17300_S3471/2021	EPI_ISL_2778986	09/06/21	North America / Mexico / Tabasco	Human	Female	65	Hospitalized	Oropharyngeal swab
hCoV-19/Mexico/TAB-InDRE_FB17301_S3472/2021	EPI_ISL_2778987	09/06/21	North America / Mexico / Tabasco	Human	Male	44	Hospitalized	Oropharyngeal swab
hCoV-19/Mexico/TAB-InDRE_FB17315_S3473/2021	EPI_ISL_2778988	10/06/21	North America / Mexico / Tabasco	Human	Male	64	Hospitalized	Oropharyngeal swab
hCoV-19/Mexico/TAB-InDRE_FB17673_S3509/2021	EPI_ISL_2779024	12/06/21	North America / Mexico / Tabasco	Human	Female	52	Hospitalized	Oropharyngeal swab
hCoV-19/Mexico/TAB-InDRE_FB17674_S3510/2021	EPI_ISL_2779025	12/06/21	North America / Mexico / Tabasco	Human	Female	34	Hospitalized	Oropharyngeal swab
hCoV-19/Mexico/TAB-InDRE_FB17675_S3511/2021	EPI_ISL_2779026	12/06/21	North America / Mexico / Tabasco	Human	Female	34	Hospitalized	Oropharyngeal swab
hCoV-19/Mexico/TAB-InDRE_FB17677_S3512/2021	EPI_ISL_2779027	12/06/21	North America / Mexico / Tabasco	Human	Male	33	Hospitalized	Oropharyngeal swab
hCoV-19/Mexico/TAB-InDRE_FB17680_S3513/2021	EPI_ISL_2779028	11/06/21	North America / Mexico / Tabasco	Human	Male	37	Hospitalized	Oropharyngeal swab
hCoV-19/Mexico/TAB-InDRE_FB17687_S3514/2021	EPI_ISL_2779029	14/06/21	North America / Mexico / Tabasco	Human	Male	51	Hospitalized	Oropharyngeal swab
hCoV-19/Mexico/TAB-InDRE_FB17688_S3515/2021	EPI_ISL_2779030	14/06/21	North America / Mexico / Tabasco	Human	Female	24	Hospitalized	Oropharyngeal swab
hCoV-19/Mexico/TAB-InDRE_FB17689_S3516/2021	EPI_ISL_2779031	14/06/21	North America / Mexico / Tabasco	Human	Female	31	Hospitalized	Oropharyngeal swab
hCoV-19/Mexico/TAB-InDRE_FB17693_S3517/2021	EPI_ISL_2779032	14/06/21	North America / Mexico / Tabasco	Human	Male	43	Hospitalized	Oropharyngeal swab
hCoV-19/Mexico/TAB-InDRE_FB17694_S3518/2021	EPI_ISL_2779033	14/06/21	North America / Mexico / Tabasco	Human	Female	45	Hospitalized	Oropharyngeal swab
hCoV-19/Mexico/TAB-InDRE_FB17796_S3528/2021	EPI_ISL_2779043	10/06/21	North America / Mexico / Tabasco	Human	Male	54	Hospitalized	Oropharyngeal swab
hCoV-19/Mexico/TAB-InDRE_FB17798_S3529/2021	EPI_ISL_2779044	14/06/21	North America / Mexico / Tabasco	Human	Male	43	Hospitalized	Oropharyngeal swab
hCoV-19/Mexico/TAB-InDRE_FB17801_S3530/2021	EPI_ISL_2779045	11/06/21	North America / Mexico / Tabasco	Human	Female	64	Hospitalized	Oropharyngeal swab
hCoV-19/Mexico/TAB-InDRE_FB17802_S3531/2021	EPI_ISL_2779046	15/06/21	North America / Mexico / Tabasco	Human	Female	23	Hospitalized	Oropharyngeal swab
hCoV-19/Mexico/TAB-InDRE_FB17985_S3850/2021	EPI_ISL_2835057	16/06/21	North America / Mexico / Tabasco	Human	Female	30	Hospitalized	Oropharyngeal swab
hCoV-19/Mexico/TAB-InDRE_FB17995_S3943/2021	EPI_ISL_2858957	16/06/21	North America / Mexico / Tabasco	Human	Male	31	Hospitalized	Oropharyngeal swab
hCoV-19/Mexico/TAB-InDRE_FB17999_S3944/2021	EPI_ISL_2858958	16/06/21	North America / Mexico / Tabasco	Human	Male	44	Hospitalized	Oropharyngeal swab
hCoV-19/Mexico/TAB-InDRE_FB18008_S3945/2021	EPI_ISL_2858959	17/06/21	North America / Mexico / Tabasco	Human	Male	89	Hospitalized	Oropharyngeal swab
hCoV-19/Mexico/TAB-InDRE_FB18030_S3948/2021	EPI_ISL_2858962	17/06/21	North America / Mexico / Tabasco	Human	Female	39	Hospitalized	Oropharyngeal swab
hCoV-19/Mexico/TAB-InDRE_FB18032_S3949/2021	EPI_ISL_2858963	18/06/21	North America / Mexico / Tabasco	Human	Male	59	Hospitalized	Oropharyngeal swab
hCoV-19/Mexico/TAB-InDRE_FB18051_S3950/2021	EPI_ISL_2858964	19/06/21	North America / Mexico / Tabasco	Human	Male	32	Hospitalized	Oropharyngeal swab
hCoV-19/Mexico/TAB-InDRE_FB18182_S3960/2021	EPI_ISL_2858974	21/06/21	North America / Mexico / Tabasco	Human	Male	47	Hospitalized	Oropharyngeal swab
hCoV-19/Mexico/TAB-InDRE_FB18192_S3961/2021	EPI_ISL_2858975	21/06/21	North America / Mexico / Tabasco	Human	Female	42	Hospitalized	Oropharyngeal swab
hCoV-19/Mexico/TAB-InDRE_FB18197_S3962/2021	EPI_ISL_2858976	21/06/21	North America / Mexico / Tabasco	Human	Female	37	Hospitalized	Oropharyngeal swab
hCoV-19/Mexico/TAB-InDRE_FB18207_S3963/2021	EPI_ISL_2858977	21/06/21	North America / Mexico / Tabasco	Human	Female	40	Hospitalized	Oropharyngeal swab
hCoV-19/Mexico/TAB-InDRE_FB18209_S3964/2021	EPI_ISL_2858978	21/06/21	North America / Mexico / Tabasco	Human	Female	44	Hospitalized	Oropharyngeal swab
hCoV-19/Mexico/TAB-InDRE_FB18330_S3970/2021	EPI_ISL_2858984	22/05/21	North America / Mexico / Tabasco	Human	Male	28	Hospitalized	Oropharyngeal swab
hCoV-19/Mexico/TAB-InDRE_FB18331_S3971/2021	EPI_ISL_2858985	22/05/21	North America / Mexico / Tabasco	Human	Female	47	Hospitalized	Oropharyngeal swab
hCoV-19/Mexico/TAB-InDRE_FB18332_S3972/2021	EPI_ISL_2858986	22/05/21	North America / Mexico / Tabasco	Human	Male	62	Hospitalized	Oropharyngeal swab
hCoV-19/Mexico/TAB-InDRE_FB18381_S4143/2021	EPI_ISL_2920714	23/06/21	North America / Mexico / Tabasco	Human	Female	56	Hospitalized	Oropharyngeal swab
hCoV-19/Mexico/TAB-InDRE_FB18382_S4144/2021	EPI_ISL_2920715	23/06/21	North America / Mexico / Tabasco	Human	Male	56	Hospitalized	Oropharyngeal swab
hCoV-19/Mexico/TAB-InDRE_FB18389_S4145/2021	EPI_ISL_2920716	23/06/21	North America / Mexico / Tabasco	Human	Female	62	Hospitalized	Oropharyngeal swab
hCoV-19/Mexico/TAB-InDRE_FB18397_S4146/2021	EPI_ISL_2920717	23/06/21	North America / Mexico / Tabasco	Human	Male	62	Hospitalized	Oropharyngeal swab
hCoV-19/Mexico/TAB-InDRE_FB18408_S4147/2021	EPI_ISL_2920718	24/06/21	North America / Mexico / Tabasco	Human	Female	45	Hospitalized	Oropharyngeal swab
hCoV-19/Mexico/TAB-InDRE_FB18500_S4148/2021	EPI_ISL_2920719	22/06/21	North America / Mexico / Tabasco	Human	Female	60	Hospitalized	Oropharyngeal swab
hCoV-19/Mexico/TAB-InDRE_FB18518_S4149/2021	EPI_ISL_2920720	25/06/21	North America / Mexico / Tabasco	Human	Male	34	Hospitalized	Oropharyngeal swab
hCoV-19/Mexico/TAB-InDRE_FB18526_S4150/2021	EPI_ISL_2920721	25/06/21	North America / Mexico / Tabasco	Human	Female	68	Hospitalized	Oropharyngeal swab

hCoV-19/Mexico/TAB-InDRE_FB18527_S4151/2021	EPI_ISL_2920722	25/06/21	North America / Mexico / Tabasco	Human	Female	23	Hospitalized	Oropharyngeal swab
hCoV-19/Mexico/TAB-InDRE_FB18528_S4152/2021	EPI_ISL_2920723	25/06/21	North America / Mexico / Tabasco	Human	Female	42	Hospitalized	Oropharyngeal swab
hCoV-19/Mexico/TAB-InDRE_FB19055_S4487/2021	EPI_ISL_3033341	28/06/21	North America / Mexico / Tabasco	Human	Female	65	Hospitalized	Oropharyngeal swab
hCoV-19/Mexico/TAB-InDRE_FB19065_S4488/2021	EPI_ISL_3033342	28/06/21	North America / Mexico / Tabasco	Human	Female	58	Hospitalized	Oropharyngeal swab
hCoV-19/Mexico/TAB-InDRE_FB19070_S4489/2021	EPI_ISL_3033343	28/06/21	North America / Mexico / Tabasco	Human	Female	29	Hospitalized	Oropharyngeal swab
hCoV-19/Mexico/TAB-InDRE_FB19071_S4490/2021	EPI_ISL_3033344	28/06/21	North America / Mexico / Tabasco	Human	Male	32	Hospitalized	Oropharyngeal swab
hCoV-19/Mexico/TAB-InDRE_FB19078_S4491/2021	EPI_ISL_3033345	28/06/21	North America / Mexico / Tabasco	Human	Female	38	Hospitalized	Oropharyngeal swab
hCoV-19/Mexico/TAB-InDRE_FB19079_S4492/2021	EPI_ISL_3033346	28/06/21	North America / Mexico / Tabasco	Human	Female	57	Hospitalized	Oropharyngeal swab
hCoV-19/Mexico/TAB-InDRE_FB19298_S4503/2021	EPI_ISL_3033357	25/06/21	North America / Mexico / Tabasco	Human	Female	49	Hospitalized	Oropharyngeal swab
hCoV-19/Mexico/TAB-InDRE_FB19319_S4504/2021	EPI_ISL_3033358	29/06/21	North America / Mexico / Tabasco	Human	Male	35	Hospitalized	Oropharyngeal swab
hCoV-19/Mexico/TAB-InDRE_FB19485_S4510/2021	EPI_ISL_3033363	29/06/21	North America / Mexico / Tabasco	Human	Female	75	Hospitalized	Oropharyngeal swab
hCoV-19/Mexico/TAB-InDRE_FB19500_S4511/2021	EPI_ISL_3033364	01/07/21	North America / Mexico / Tabasco	Human	Male	49	Hospitalized	Oropharyngeal swab
hCoV-19/Mexico/TAB-InDRE_FB19673_S4516/2021	EPI_ISL_3033369	02/07/21	North America / Mexico / Tabasco	Human	Female	33	Hospitalized	Oropharyngeal swab
hCoV-19/Mexico/TAB-INMEGEN-05-06-245/2021	EPI_ISL_2603720	01/06/21	North America / Mexico / Tabasco	Human	Male	39	unknown	Oropharyngeal swab
hCoV-19/Mexico/TAB-INMEGEN-05-06-253/2021	EPI_ISL_2603724	01/06/21	North America / Mexico / Tabasco	Human	Female	67	unknown	Oropharyngeal swab
hCoV-19/Mexico/TAB-INMEGEN-05-06-261/2021	EPI_ISL_2603732	01/06/21	North America / Mexico / Tabasco	Human	Male	31	unknown	Oropharyngeal swab
hCoV-19/Mexico/TAB-INMEGEN-06-01-211/2021	EPI_ISL_2616918	03/06/21	North America / Mexico / Tabasco	Human	Female	19	unknown	Oropharyngeal swab
hCoV-19/Mexico/TAB-INMEGEN-06-01-219/2021	EPI_ISL_2616902	03/06/21	North America / Mexico / Tabasco	Human	Male	34	unknown	Oropharyngeal swab
hCoV-19/Mexico/TAB-INMEGEN-06-01-243/2021	EPI_ISL_2616917	03/06/21	North America / Mexico / Tabasco	Human	Male	39	unknown	Oropharyngeal swab
hCoV-19/Mexico/TAB-INMEGEN-06-01-348/2021	EPI_ISL_2616988	04/06/21	North America / Mexico / Tabasco	Human	Male	33	unknown	Oropharyngeal swab
hCoV-19/Mexico/TAB-INMEGEN-06-01-364/2021	EPI_ISL_2616972	04/06/21	North America / Mexico / Tabasco	Human	Female	40	unknown	Oropharyngeal swab
hCoV-19/Mexico/TAB-INMEGEN-06-02-102/2021	EPI_ISL_2692630	07/06/21	North America / Mexico / Tabasco	Human	Male	35	unknown	Oropharyngeal swab
hCoV-19/Mexico/TAB-INMEGEN-06-02-158/2021	EPI_ISL_2692674	07/06/21	North America / Mexico / Tabasco	Human	Male	22	unknown	Oropharyngeal swab
hCoV-19/Mexico/TAB-INMEGEN-06-02-327/2021	EPI_ISL_2692813	01/06/21	North America / Mexico / Tabasco	Human	Male	34	unknown	Oropharyngeal swab
hCoV-19/Mexico/TAB-INMEGEN-06-03-343/2021	EPI_ISL_2810298	08/06/21	North America / Mexico / Tabasco	Human	Female	27	unknown	Oropharyngeal swab
hCoV-19/Mexico/TAB-INMEGEN-06-03-352/2021	EPI_ISL_2810305	09/06/21	North America / Mexico / Tabasco	Human	Male	39	unknown	Oropharyngeal swab
hCoV-19/Mexico/TAB-INMEGEN-06-03-360/2021	EPI_ISL_2810312	09/06/21	North America / Mexico / Tabasco	Human	Male	52	unknown	Oropharyngeal swab
hCoV-19/Mexico/TAB-INMEGEN-06-04-326/2021	EPI_ISL_2894372	12/06/21	North America / Mexico / Tabasco	Human	Male	40	unknown	Oropharyngeal swab
hCoV-19/Mexico/TAB-INMEGEN-06-04-329/2021	EPI_ISL_2894375	12/06/21	North America / Mexico / Tabasco	Human	Male	68	unknown	Oropharyngeal swab
hCoV-19/Mexico/TAB-INMEGEN-16-306/2021	EPI_ISL_2978616	14/06/21	North America / Mexico / Tabasco	Human	Male	33	unknown	Oropharyngeal swab
hCoV-19/Mexico/TAM-InDRE_236/2021	EPI_ISL_961466	13/01/21	North America / Mexico / Tamaulipas	Human	Female	70	Hospitalized	Oropharyngeal swab
hCoV-19/Mexico/TAM-InDRE_244/2021	EPI_ISL_1008713	26/01/21	North America / Mexico / Tamaulipas	Human	Female	39	Hospitalized	Pharyngeal swab
hCoV-19/Mexico/TAM-InDRE_F108L_S569/2021	EPI_ISL_1334382	24/02/21	North America / Mexico / Tamaulipas	Human	Male	46	Hospitalized	Oropharyngeal swab
hCoV-19/Mexico/TAM-InDRE_F111L_S570/2021	EPI_ISL_1334383	01/03/21	North America / Mexico / Tamaulipas	Human	Female	35	Hospitalized	Oropharyngeal swab
hCoV-19/Mexico/TAM-InDRE_F112L_S571/2021	EPI_ISL_1334384	01/03/21	North America / Mexico / Tamaulipas	Human	Male	41	Hospitalized	Oropharyngeal swab
hCoV-19/Mexico/TAM-InDRE_F114L_S572/2021	EPI_ISL_1334385	24/02/21	North America / Mexico / Tamaulipas	Human	Male	44	Hospitalized	Oropharyngeal swab
hCoV-19/Mexico/TAM-InDRE_F11757_S901/2021	EPI_ISL_1423511	10/03/21	North America / Mexico / Tamaulipas	Human	Female	59	Hospitalized	Oropharyngeal swab
hCoV-19/Mexico/TAM-InDRE_F86L_S567/2021	EPI_ISL_1334380	17/02/21	North America / Mexico / Tamaulipas	Human	Male	35	Hospitalized	Oropharyngeal swab
hCoV-19/Mexico/TAM-InDRE_FB14042_S2071/2021	EPI_ISL_2283711	15/04/21	North America / Mexico / Tamaulipas	Human	Male	90	Deceased	Oropharyngeal swab
hCoV-19/Mexico/TAM-InDRE_FB14045_S2072/2021	EPI_ISL_2283712	15/04/21	North America / Mexico / Tamaulipas	Human	Female	50	Hospitalized	Oropharyngeal swab
hCoV-19/Mexico/TAM-InDRE_FB14051_S2073/2021	EPI_ISL_2283713	17/04/21	North America / Mexico / Tamaulipas	Human	Male	72	Deceased	Oropharyngeal swab
hCoV-19/Mexico/TAM-InDRE_FB14052_S2074/2021	EPI_ISL_2283714	18/04/21	North America / Mexico / Tamaulipas	Human	Male	44	Deceased	Oropharyngeal swab
hCoV-19/Mexico/TAM-InDRE_FB14054_S2075/2021	EPI_ISL_2283715	20/04/21	North America / Mexico / Tamaulipas	Human	Male	47	Hospitalized	Oropharyngeal swab
hCoV-19/Mexico/TAM-InDRE_FB14060_S2076/2021	EPI_ISL_2283716	16/04/21	North America / Mexico / Tamaulipas	Human	Female	37	Hospitalized	Oropharyngeal swab
hCoV-19/Mexico/TAM-InDRE_FB14700_S2045/2021	EPI_ISL_2283687	27/04/21	North America / Mexico / Tamaulipas	Human	Female	29	Hospitalized	Oropharyngeal swab
hCoV-19/Mexico/TAM-InDRE_FB14924_S2391/2021	EPI_ISL_2455916	05/05/21	North America / Mexico / Tamaulipas	Human	Female	74	Hospitalized	Oropharyngeal swab
hCoV-19/Mexico/TAM-InDRE_FB14930_S2051/2021	EPI_ISL_2283693	04/05/21	North America / Mexico / Tamaulipas	Human	Female	62	Hospitalized	Oropharyngeal swab
hCoV-19/Mexico/TAM-InDRE_FB14936_S2591/2021	EPI_ISL_2492487	05/05/21	North America / Mexico / Tamaulipas	Human	Female	42	Hospitalized	Oropharyngeal swab
hCoV-19/Mexico/TAM-InDRE_FB14939_S2393/2021	EPI_ISL_2455918	05/05/21	North America / Mexico / Tamaulipas	Human	Female	44	Hospitalized	Oropharyngeal swab
hCoV-19/Mexico/TAM-InDRE_FB15935_S2398/2021	EPI_ISL_2455923	06/05/21	North America / Mexico / Tamaulipas	Human	Female	33	Hospitalized	Oropharyngeal swab
hCoV-19/Mexico/TAM-InDRE_FB15937_S2399/2021	EPI_ISL_2455924	07/05/21	North America / Mexico / Tamaulipas	Human	Male	56	Hospitalized	Oropharyngeal swab
hCoV-19/Mexico/TAM-InDRE_FB15938_S2400/2021	EPI_ISL_2455925	07/05/21	North America / Mexico / Tamaulipas	Human	Female	31	Hospitalized	Oropharyngeal swab

hCoV-19/Mexico/TAM-InDRE_FB15942_S2406/2021	EPI_ISL_2455930	08/05/21	North America / Mexico / Tamaulipas	Human	Female	33	Hospitalized	Oropharyngeal swab
hCoV-19/Mexico/TAM-InDRE_FB15943_S2407/2021	EPI_ISL_2455931	07/05/21	North America / Mexico / Tamaulipas	Human	Female	73	Hospitalized	Oropharyngeal swab
hCoV-19/Mexico/TAM-InDRE_FB15944_S2408/2021	EPI_ISL_2455932	08/05/21	North America / Mexico / Tamaulipas	Human	Female	30	Hospitalized	Oropharyngeal swab
hCoV-19/Mexico/TAM-InDRE_FB15955_S2409/2021	EPI_ISL_2455933	08/05/21	North America / Mexico / Tamaulipas	Human	Male	59	Hospitalized	Oropharyngeal swab
hCoV-19/Mexico/TAM-InDRE_FB16011_S2411/2021	EPI_ISL_2455935	14/05/21	North America / Mexico / Tamaulipas	Human	Female	26	Hospitalized	Oropharyngeal swab
hCoV-19/Mexico/TAM-InDRE_FB16013_S2412/2021	EPI_ISL_2455936	14/05/21	North America / Mexico / Tamaulipas	Human	Male	51	unknown	Oropharyngeal swab
hCoV-19/Mexico/TAM-InDRE_FB16014_S2413/2021	EPI_ISL_2455937	15/05/21	North America / Mexico / Tamaulipas	Human	Female	52	Hospitalized	Oropharyngeal swab
hCoV-19/Mexico/TAM-InDRE_FB16015_S2414/2021	EPI_ISL_2455938	11/05/21	North America / Mexico / Tamaulipas	Human	Female	63	Hospitalized	Oropharyngeal swab
hCoV-19/Mexico/TAM-InDRE_FB16016_S2415/2021	EPI_ISL_2455939	17/05/21	North America / Mexico / Tamaulipas	Human	Female	37	Hospitalized	Oropharyngeal swab
hCoV-19/Mexico/TAM-InDRE_FB16017_S2416/2021	EPI_ISL_2455940	12/05/21	North America / Mexico / Tamaulipas	Human	Female	49	Hospitalized	Oropharyngeal swab
hCoV-19/Mexico/TAM-InDRE_FB16018_S2417/2021	EPI_ISL_2455941	12/05/21	North America / Mexico / Tamaulipas	Human	Male	27	Hospitalized	Oropharyngeal swab
hCoV-19/Mexico/TAM-InDRE_FB16019_S2418/2021	EPI_ISL_2455942	12/05/21	North America / Mexico / Tamaulipas	Human	Male	19	unknown	Oropharyngeal swab
hCoV-19/Mexico/TAM-InDRE_FB16020_S2419/2021	EPI_ISL_2455943	17/05/21	North America / Mexico / Tamaulipas	Human	Male	25	Hospitalized	Oropharyngeal swab
hCoV-19/Mexico/TAM-InDRE_FB16021_S2420/2021	EPI_ISL_2455944	17/05/21	North America / Mexico / Tamaulipas	Human	Male	60	Hospitalized	Oropharyngeal swab
hCoV-19/Mexico/TAM-InDRE_FB16022_S2421/2021	EPI_ISL_2455945	13/05/21	North America / Mexico / Tamaulipas	Human	Male	51	unknown	Oropharyngeal swab
hCoV-19/Mexico/TAM-InDRE_FB16023_S2422/2021	EPI_ISL_2455946	14/05/21	North America / Mexico / Tamaulipas	Human	Female	61	Hospitalized	Oropharyngeal swab
hCoV-19/Mexico/TAM-InDRE_FB16024_S2423/2021	EPI_ISL_2455947	12/05/21	North America / Mexico / Tamaulipas	Human	Male	57	Hospitalized	Oropharyngeal swab
hCoV-19/Mexico/TAM-InDRE_FB16025_S2424/2021	EPI_ISL_2455948	12/05/21	North America / Mexico / Tamaulipas	Human	Male	39	unknown	Oropharyngeal swab
hCoV-19/Mexico/TAM-InDRE_FB16026_S2425/2021	EPI_ISL_2455949	15/05/21	North America / Mexico / Tamaulipas	Human	Female	49	Hospitalized	Oropharyngeal swab
hCoV-19/Mexico/TAM-InDRE_FB16027_S2426/2021	EPI_ISL_2455950	13/05/21	North America / Mexico / Tamaulipas	Human	Female	37	Hospitalized	Oropharyngeal swab
hCoV-19/Mexico/TAM-InDRE_FB16028_S2427/2021	EPI_ISL_2455951	14/05/21	North America / Mexico / Tamaulipas	Human	Female	32	Hospitalized	Oropharyngeal swab
hCoV-19/Mexico/TAM-InDRE_FB16029_S2428/2021	EPI_ISL_2455952	17/05/21	North America / Mexico / Tamaulipas	Human	Male	43	Hospitalized	Oropharyngeal swab
hCoV-19/Mexico/TAM-InDRE_FB16031_S2430/2021	EPI_ISL_2455954	14/05/21	North America / Mexico / Tamaulipas	Human	Male	35	Hospitalized	Oropharyngeal swab
hCoV-19/Mexico/TAM-InDRE_FB16032_S2431/2021	EPI_ISL_2455955	12/05/21	North America / Mexico / Tamaulipas	Human	Female	39	Hospitalized	Oropharyngeal swab
hCoV-19/Mexico/TAM-InDRE_FB16033_S2432/2021	EPI_ISL_2455956	12/05/21	North America / Mexico / Tamaulipas	Human	Male	12	Hospitalized	Oropharyngeal swab
hCoV-19/Mexico/TAM-InDRE_FB16034_S2433/2021	EPI_ISL_2455957	17/05/21	North America / Mexico / Tamaulipas	Human	Female	17	unknown	Oropharyngeal swab
hCoV-19/Mexico/TAM-InDRE_FB16326_S3938/2021	EPI_ISL_2858952	14/05/21	North America / Mexico / Tamaulipas	Human	Female	42	Hospitalized	Oropharyngeal swab
hCoV-19/Mexico/TAM-InDRE_FB16329_S3939/2021	EPI_ISL_2858953	14/05/21	North America / Mexico / Tamaulipas	Human	Female	64	Hospitalized	Oropharyngeal swab
hCoV-19/Mexico/TAM-InDRE_FB16726_S2771/2021	EPI_ISL_2533797	20/05/21	North America / Mexico / Tamaulipas	Human	Female	50	Hospitalized	Oropharyngeal swab
hCoV-19/Mexico/TAM-InDRE_FB16727_S2772/2021	EPI_ISL_2533798	20/05/21	North America / Mexico / Tamaulipas	Human	Female	28	Hospitalized	Oropharyngeal swab
hCoV-19/Mexico/TAM-InDRE_FB16728_S2773/2021	EPI_ISL_2533799	21/05/21	North America / Mexico / Tamaulipas	Human	Female	67	Hospitalized	Oropharyngeal swab
hCoV-19/Mexico/TAM-InDRE_FB16733_S2774/2021	EPI_ISL_2533800	20/05/21	North America / Mexico / Tamaulipas	Human	Female	34	Hospitalized	Oropharyngeal swab
hCoV-19/Mexico/TAM-InDRE_FB16734_S2775/2021	EPI_ISL_2533801	25/05/21	North America / Mexico / Tamaulipas	Human	Female	54	Hospitalized	Oropharyngeal swab
hCoV-19/Mexico/TAM-InDRE_FB17060_S3083/2021	EPI_ISL_2663350	28/05/21	North America / Mexico / Tamaulipas	Human	Female	58	Hospitalized	Oropharyngeal swab
hCoV-19/Mexico/TAM-InDRE_FB17076_S3133/2021	EPI_ISL_2663400	31/05/21	North America / Mexico / Tamaulipas	Human	Female	42	Hospitalized	Oropharyngeal swab
hCoV-19/Mexico/TAM-InDRE_FB17077_S3134/2021	EPI_ISL_2663401	31/05/21	North America / Mexico / Tamaulipas	Human	Female	53	Hospitalized	Oropharyngeal swab
hCoV-19/Mexico/TAM-InDRE_FB17417_S3927/2021	EPI_ISL_2858941	06/06/21	North America / Mexico / Tamaulipas	Human	Male	35	Hospitalized	Oropharyngeal swab
hCoV-19/Mexico/TAM-InDRE_FB17423_S3928/2021	EPI_ISL_2858942	05/06/21	North America / Mexico / Tamaulipas	Human	Male	35	Hospitalized	Oropharyngeal swab
hCoV-19/Mexico/TAM-InDRE_FB17425_S3929/2021	EPI_ISL_2858943	07/06/21	North America / Mexico / Tamaulipas	Human	Male	46	Hospitalized	Oropharyngeal swab
hCoV-19/Mexico/TAM-InDRE_FB17463_S3487/2021	EPI_ISL_2779002	08/06/21	North America / Mexico / Tamaulipas	Human	Female	33	Hospitalized	Oropharyngeal swab
hCoV-19/Mexico/TAM-InDRE_FB17466_S3488/2021	EPI_ISL_2779003	02/06/21	North America / Mexico / Tamaulipas	Human	Female	52	Hospitalized	Oropharyngeal swab
hCoV-19/Mexico/TAM-InDRE_FB17471_S3489/2021	EPI_ISL_2779004	08/06/21	North America / Mexico / Tamaulipas	Human	Female	38	Hospitalized	Oropharyngeal swab
hCoV-19/Mexico/TAM-InDRE_FB18098_S3951/2021	EPI_ISL_2858965	14/06/21	North America / Mexico / Tamaulipas	Human	Female	30	Hospitalized	Oropharyngeal swab
hCoV-19/Mexico/TAM-InDRE_FB18103_S3952/2021	EPI_ISL_2858966	15/06/21	North America / Mexico / Tamaulipas	Human	Female	33	Hospitalized	Oropharyngeal swab
hCoV-19/Mexico/TAM-InDRE_FB18104_S3953/2021	EPI_ISL_2858967	15/06/21	North America / Mexico / Tamaulipas	Human	Male	40	Hospitalized	Oropharyngeal swab
hCoV-19/Mexico/TAM-InDRE_FB18105_S3954/2021	EPI_ISL_2858968	09/06/21	North America / Mexico / Tamaulipas	Human	Female	58	Hospitalized	Oropharyngeal swab
hCoV-19/Mexico/TAM-InDRE_FB18106_S3955/2021	EPI_ISL_2858969	14/06/21	North America / Mexico / Tamaulipas	Human	Male	59	Hospitalized	Oropharyngeal swab
hCoV-19/Mexico/TAM-InDRE_FB18115_S3956/2021	EPI_ISL_2858970	15/06/21	North America / Mexico / Tamaulipas	Human	Female	51	Hospitalized	Oropharyngeal swab
hCoV-19/Mexico/TAM-InDRE_FB18116_S3957/2021	EPI_ISL_2858971	16/06/21	North America / Mexico / Tamaulipas	Human	Female	40	Hospitalized	Oropharyngeal swab
hCoV-19/Mexico/TAM-InDRE_FB18133_S4122/2021	EPI_ISL_2920693	13/06/21	North America / Mexico / Tamaulipas	Human	Male	48	Hospitalized	Oropharyngeal swab
hCoV-19/Mexico/TAM-InDRE_FB18134_S4123/2021	EPI_ISL_2920694	12/06/21	North America / Mexico / Tamaulipas	Human	Female	45	Deceased	Oropharyngeal swab
hCoV-19/Mexico/TAM-InDRE_FB18137_S4124/2021	EPI_ISL_2920695	14/06/21	North America / Mexico / Tamaulipas	Human	Female	49	Hospitalized	Oropharyngeal swab

hCoV-19/Mexico/TAM-InDRE_FB18138_S4125/2021	EPI_ISL_2920696	09/06/21	North America / Mexico / Tamaulipas	Human		Male	26	Hospitalized	Oropharyngeal swab
hCoV-19/Mexico/TAM-InDRE_FB18142_S4126/2021	EPI_ISL_2920697	15/06/21	North America / Mexico / Tamaulipas	Human		Female	59	Hospitalized	Oropharyngeal swab
hCoV-19/Mexico/TAM-InDRE_FB18143_S4127/2021	EPI_ISL_2920698	15/06/21	North America / Mexico / Tamaulipas	Human		Male	63	Hospitalized	Oropharyngeal swab
hCoV-19/Mexico/TAM-InDRE_FB18144_S4128/2021	EPI_ISL_2920699	15/06/21	North America / Mexico / Tamaulipas	Human		Female	35	Hospitalized	Oropharyngeal swab
hCoV-19/Mexico/TAM-InDRE_FB18145_S4129/2021	EPI_ISL_2920700	15/06/21	North America / Mexico / Tamaulipas	Human		Male	48	Hospitalized	Oropharyngeal swab
hCoV-19/Mexico/TAM-InDRE_FB18146_S4130/2021	EPI_ISL_2920701	16/06/21	North America / Mexico / Tamaulipas	Human		Male	32	Hospitalized	Oropharyngeal swab
hCoV-19/Mexico/TAM-InDRE_FB18874_S4154/2021	EPI_ISL_2920725	16/06/21	North America / Mexico / Tamaulipas	Human		Male	32	Hospitalized	Oropharyngeal swab
hCoV-19/Mexico/TAM-InDRE_FB18878_S4155/2021	EPI_ISL_2920726	18/06/21	North America / Mexico / Tamaulipas	Human		Male	60	Hospitalized	Oropharyngeal swab
hCoV-19/Mexico/TAM-InDRE_FB18879_S4156/2021	EPI_ISL_2920727	18/06/21	North America / Mexico / Tamaulipas	Human		Female	36	Hospitalized	Oropharyngeal swab
hCoV-19/Mexico/TAM-InDRE_FB18890_S4157/2021	EPI_ISL_2920728	22/06/21	North America / Mexico / Tamaulipas	Human		Female	68	Hospitalized	Oropharyngeal swab
hCoV-19/Mexico/TAM-InDRE_FB18891_S4158/2021	EPI_ISL_2920729	22/06/21	North America / Mexico / Tamaulipas	Human		Female	46	Hospitalized	Oropharyngeal swab
hCoV-19/Mexico/TAM-InDRE_FB18896_S4159/2021	EPI_ISL_2920730	17/06/21	North America / Mexico / Tamaulipas	Human		Male	57	Hospitalized	Oropharyngeal swab
hCoV-19/Mexico/TAM-InDRE_FB18898_S4469/2021	EPI_ISL_3033323	18/06/21	North America / Mexico / Tamaulipas	Human		Female	44	Hospitalized	Oropharyngeal swab
hCoV-19/Mexico/TAM-InDRE_FB18904_S4470/2021	EPI_ISL_3033324	18/06/21	North America / Mexico / Tamaulipas	Human		Female	45	Hospitalized	Oropharyngeal swab
hCoV-19/Mexico/TAM-InDRE_FB18905_S4471/2021	EPI_ISL_3033325	18/06/21	North America / Mexico / Tamaulipas	Human		Female	53	Hospitalized	Oropharyngeal swab
hCoV-19/Mexico/TAM-InDRE_FB18909_S4472/2021	EPI_ISL_3033326	21/06/21	North America / Mexico / Tamaulipas	Human		Male	41	Hospitalized	Oropharyngeal swab
hCoV-19/Mexico/TAM-InDRE_FB18910_S4473/2021	EPI_ISL_3033327	21/06/21	North America / Mexico / Tamaulipas	Human		Female	49	Hospitalized	Oropharyngeal swab
hCoV-19/Mexico/TAM-InDRE_FB18911_S4474/2021	EPI_ISL_3033328	14/06/21	North America / Mexico / Tamaulipas	Human		Female	39	Hospitalized	Oropharyngeal swab
hCoV-19/Mexico/TAM-InDRE_FB18912_S4475/2021	EPI_ISL_3033329	14/06/21	North America / Mexico / Tamaulipas	Human		Male	40	Hospitalized	Oropharyngeal swab
hCoV-19/Mexico/TAM-InDRE_FB18922_S4476/2021	EPI_ISL_3033330	22/06/21	North America / Mexico / Tamaulipas	Human		Male	49	Hospitalized	Oropharyngeal swab
hCoV-19/Mexico/TAM-InDRE_FB18925_S4477/2021	EPI_ISL_3033331	23/06/21	North America / Mexico / Tamaulipas	Human		Female	55	Hospitalized	Oropharyngeal swab
hCoV-19/Mexico/TAM-InDRE-94/2020	EPI_ISL_794592	31/12/20	North America / Mexico / Tamaulipas	Human	Travel history	Male	56	Hospitalized	Nasopharyngeal and oropharyngeal swabs
hCoV-19/Mexico/TAM-INMEGEN-06-01-347/2021	EPI_ISL_2616961	04/06/21	North America / Mexico / Tamaulipas	Human		Female	57	unknown	Oropharyngeal swab
hCoV-19/Mexico/VER-InDRE_FB14026_S1753/2021	EPI_ISL_2101892	18/04/21	North America / Mexico / Veracruz	Human		Female	49	Hospitalized	Oropharyngeal swab
hCoV-19/Mexico/VER-InDRE_FB17431_S3930/2021	EPI_ISL_2858944	12/06/21	North America / Mexico / Veracruz	Human		Male	68	Deceased	Oropharyngeal swab
hCoV-19/Mexico/VER-InDRE_FB17434_S3931/2021	EPI_ISL_2858945	08/06/21	North America / Mexico / Veracruz	Human		Male	36	Hospitalized	Oropharyngeal swab
hCoV-19/Mexico/VER-INMEGEN-05-06-207/2021	EPI_ISL_2603696	31/05/21	North America / Mexico / Veracruz	Human		Male	38	unknown	Oropharyngeal swab
hCoV-19/Mexico/VER-INMEGEN-05-06-231/2021	EPI_ISL_2603713	31/05/21	North America / Mexico / Veracruz	Human		Female	34	unknown	Oropharyngeal swab
hCoV-19/Mexico/VER-INMEGEN-05-06-246/2021	EPI_ISL_2881841	01/06/21	North America / Mexico / Veracruz	Human		Female	34	unknown	Oropharyngeal swab
hCoV-19/Mexico/VER-INMEGEN-05-06-254/2021	EPI_ISL_2603725	31/05/21	North America / Mexico / Veracruz	Human		Female	54	unknown	Oropharyngeal swab
hCoV-19/Mexico/VER-INMEGEN-05-06-255/2021	EPI_ISL_2603726	27/05/21	North America / Mexico / Veracruz	Human		Male	25	unknown	Oropharyngeal swab
hCoV-19/Mexico/VER-INMEGEN-06-01-368/2021	EPI_ISL_2616973	05/06/21	North America / Mexico / Veracruz	Human		Male	25	unknown	Oropharyngeal swab
hCoV-19/Mexico/VER-INMEGEN-06-02-173/2021	EPI_ISL_2692689	08/06/21	North America / Mexico / Veracruz	Human		Female	25	unknown	Oropharyngeal swab
hCoV-19/Mexico/VER-INMEGEN-06-02-372/2021	EPI_ISL_2692848	09/06/21	North America / Mexico / Veracruz	Human		Female	38	unknown	Oropharyngeal swab
hCoV-19/Mexico/VER-INMEGEN-06-02-373/2021	EPI_ISL_2692849	09/06/21	North America / Mexico / Veracruz	Human		Male	40	unknown	Oropharyngeal swab
hCoV-19/Mexico/VER-INMEGEN-06-02-374/2021	EPI_ISL_2692850	09/06/21	North America / Mexico / Veracruz	Human		Male	47	unknown	Oropharyngeal swab
hCoV-19/Mexico/VER-INMEGEN-06-02-375/2021	EPI_ISL_2692851	09/06/21	North America / Mexico / Veracruz	Human		Male	37	unknown	Oropharyngeal swab
hCoV-19/Mexico/VER-INMEGEN-06-02-376/2021	EPI_ISL_2692852	09/06/21	North America / Mexico / Veracruz	Human		Female	25	unknown	Oropharyngeal swab
hCoV-19/Mexico/VER-INMEGEN-06-04-13/2021	EPI_ISL_2894163	09/06/21	North America / Mexico / Veracruz	Human		Female	34	unknown	Oropharyngeal swab
hCoV-19/Mexico/VER-INMEGEN-06-04-287/2021	EPI_ISL_2894334	12/06/21	North America / Mexico / Veracruz	Human		Female	39	unknown	Oropharyngeal swab
hCoV-19/Mexico/VER-INMEGEN-06-04-324/2021	EPI_ISL_2894370	13/06/21	North America / Mexico / Veracruz	Human		Male	30	unknown	Oropharyngeal swab
hCoV-19/Mexico/VER-INMEGEN-06-04-6/2021	EPI_ISL_2894156	09/06/21	North America / Mexico / Veracruz	Human		Male	25	unknown	Oropharyngeal swab
hCoV-19/Mexico/VER-INMEGEN-16-284/2021	EPI_ISL_2978595	14/06/21	North America / Mexico / Veracruz	Human		Female	30	unknown	Oropharyngeal swab
hCoV-19/Mexico/VER-INMEGEN-16-302/2021	EPI_ISL_2978612	16/06/21	North America / Mexico / Veracruz	Human		Female	34	unknown	Oropharyngeal swab
hCoV-19/Mexico/VER-INMEGEN-16-310/2021	EPI_ISL_2978619	16/06/21	North America / Mexico / Veracruz	Human		Female	26	unknown	Oropharyngeal swab
hCoV-19/Mexico/VER-INMEGEN-16-322/2021	EPI_ISL_2978629	14/06/21	North America / Mexico / Veracruz	Human		Male	19	unknown	Oropharyngeal swab
hCoV-19/Mexico/VER-INMEGEN-16-338/2021	EPI_ISL_2978643	14/06/21	North America / Mexico / Veracruz	Human		Female	60	unknown	Oropharyngeal swab
hCoV-19/Mexico/YUC-Biomedicos-6140701/2021	EPI_ISL_2918940	14/06/21	North America / Mexico / Yucatan	Human		Female	51	Hospitalized	Nasopharyngeal swab
hCoV-19/Mexico/YUC-Biomedicos-6150004/2021	EPI_ISL_2928339	15/06/21	North America / Mexico / Yucatan	Human	th America / M	Female	34	unknown	Nasopharyngeal swab
hCoV-19/Mexico/YUC-Biomedicos-6220333/2021	EPI_ISL_2928304	22/06/21	North America / Mexico / Yucatan	Human	th America / M	Male	49	unknown	Oropharyngeal swab
hCoV-19/Mexico/YUC-InDRE_F11421_S904/2021	EPI_ISL_1423514	01/03/21	North America / Mexico / Yucatan	Human		Female	67	Hospitalized	Oropharyngeal swab
hCoV-19/Mexico/YUC-InDRE_F12194_S1195/2021	EPI_ISL_1626802	22/03/21	North America / Mexico / Yucatan	Human		Female	33	Hospitalized	Oropharyngeal swab

hCoV-19/Mexico/YUC-InDRE_FB15125_S2056/2021	EPI_ISL_2283698	19/03/21	North America / Mexico / Yucatan	Human	Female	29	Hospitalized	Oropharyngeal swab
hCoV-19/Mexico/YUC-InDRE_FB15167_S2057/2021	EPI_ISL_2283699	24/03/21	North America / Mexico / Yucatan	Human	Male	45	Hospitalized	Oropharyngeal swab
hCoV-19/Mexico/YUC-InDRE_FB15328_S2079/2021	EPI_ISL_2283719	08/04/21	North America / Mexico / Yucatan	Human	Female	55	Hospitalized	Oropharyngeal swab
hCoV-19/Mexico/YUC-InDRE_FB16549_S3111/2021	EPI_ISL_2663378	28/05/21	North America / Mexico / Yucatan	Human	Female	22	Hospitalized	Oropharyngeal swab
hCoV-19/Mexico/YUC-InDRE_FB16551_S3112/2021	EPI_ISL_2663379	31/05/21	North America / Mexico / Yucatan	Human	Male	18	Hospitalized	Oropharyngeal swab
hCoV-19/Mexico/YUC-InDRE_FB16573_S2744/2021	EPI_ISL_2533770	17/05/21	North America / Mexico / Yucatan	Human	Male	15	Hospitalized	Oropharyngeal swab
hCoV-19/Mexico/YUC-InDRE_FB16575_S2745/2021	EPI_ISL_2533771	17/05/21	North America / Mexico / Yucatan	Human	Male	49	Hospitalized	Oropharyngeal swab
hCoV-19/Mexico/YUC-InDRE_FB16578_S2746/2021	EPI_ISL_2533772	17/05/21	North America / Mexico / Yucatan	Human	Male	38	Hospitalized	Oropharyngeal swab
hCoV-19/Mexico/YUC-InDRE_FB16582_S2747/2021	EPI_ISL_2533773	18/05/21	North America / Mexico / Yucatan	Human	Male	91	Deceased	Oropharyngeal swab
hCoV-19/Mexico/YUC-InDRE_FB16584_S2748/2021	EPI_ISL_2533774	18/05/21	North America / Mexico / Yucatan	Human	Female	57	Hospitalized	Oropharyngeal swab
hCoV-19/Mexico/YUC-InDRE_FB16588_S2749/2021	EPI_ISL_2533775	19/05/21	North America / Mexico / Yucatan	Human	Female	32	Hospitalized	Oropharyngeal swab
hCoV-19/Mexico/YUC-InDRE_FB16589_S2750/2021	EPI_ISL_2533776	18/05/21	North America / Mexico / Yucatan	Human	Female	76	Hospitalized	Oropharyngeal swab
hCoV-19/Mexico/YUC-InDRE_FB16598_S2751/2021	EPI_ISL_2533777	19/05/21	North America / Mexico / Yucatan	Human	Female	54	Hospitalized	Oropharyngeal swab
hCoV-19/Mexico/YUC-InDRE_FB16601_S2752/2021	EPI_ISL_2533778	19/05/21	North America / Mexico / Yucatan	Human	Female	57	Hospitalized	Oropharyngeal swab
hCoV-19/Mexico/YUC-InDRE_FB16607_S2753/2021	EPI_ISL_2533779	20/05/21	North America / Mexico / Yucatan	Human	Male	25	Hospitalized	Oropharyngeal swab
hCoV-19/Mexico/YUC-InDRE_FB16611_S2754/2021	EPI_ISL_2533780	20/05/21	North America / Mexico / Yucatan	Human	Male	23	Hospitalized	Oropharyngeal swab
hCoV-19/Mexico/YUC-InDRE_FB16614_S2755/2021	EPI_ISL_2533781	20/05/21	North America / Mexico / Yucatan	Human	Male	44	Hospitalized	Oropharyngeal swab
hCoV-19/Mexico/YUC-InDRE_FB16617_S2756/2021	EPI_ISL_2533782	20/05/21	North America / Mexico / Yucatan	Human	Female	52	Hospitalized	Oropharyngeal swab
hCoV-19/Mexico/YUC-InDRE_FB16638_S2757/2021	EPI_ISL_2533783	21/05/21	North America / Mexico / Yucatan	Human	Male	22	Hospitalized	Oropharyngeal swab
hCoV-19/Mexico/YUC-InDRE_FB16653_S2758/2021	EPI_ISL_2533784	24/05/21	North America / Mexico / Yucatan	Human	Female	52	Hospitalized	Oropharyngeal swab
hCoV-19/Mexico/YUC-InDRE_FB16657_S2759/2021	EPI_ISL_2533785	24/05/21	North America / Mexico / Yucatan	Human	Male	27	Hospitalized	Oropharyngeal swab
hCoV-19/Mexico/YUC-InDRE_FB16659_S2760/2021	EPI_ISL_2533786	24/05/21	North America / Mexico / Yucatan	Human	Male	38	Hospitalized	Oropharyngeal swab
hCoV-19/Mexico/YUC-InDRE_FB16661_S2761/2021	EPI_ISL_2533787	24/05/21	North America / Mexico / Yucatan	Human	Male	33	Hospitalized	Oropharyngeal swab
hCoV-19/Mexico/YUC-InDRE_FB16665_S2762/2021	EPI_ISL_2533788	24/05/21	North America / Mexico / Yucatan	Human	Female	40	Hospitalized	Oropharyngeal swab
hCoV-19/Mexico/YUC-InDRE_FB16666_S2763/2021	EPI_ISL_2533789	25/05/21	North America / Mexico / Yucatan	Human	Male	32	Hospitalized	Oropharyngeal swab
hCoV-19/Mexico/YUC-InDRE_FB16667_S2764/2021	EPI_ISL_2533790	25/05/21	North America / Mexico / Yucatan	Human	Female	45	Hospitalized	Oropharyngeal swab
hCoV-19/Mexico/YUC-InDRE_FB16672_S2765/2021	EPI_ISL_2533791	25/05/21	North America / Mexico / Yucatan	Human	Male	52	Hospitalized	Oropharyngeal swab
hCoV-19/Mexico/YUC-InDRE_FB16674_S2766/2021	EPI_ISL_2533792	25/05/21	North America / Mexico / Yucatan	Human	Female	21	Hospitalized	Oropharyngeal swab
hCoV-19/Mexico/YUC-InDRE_FB16678_S2767/2021	EPI_ISL_2533793	26/05/21	North America / Mexico / Yucatan	Human	Female	47	Hospitalized	Oropharyngeal swab
hCoV-19/Mexico/YUC-InDRE_FB16682_S2768/2021	EPI_ISL_2533794	26/05/21	North America / Mexico / Yucatan	Human	Male	93	Hospitalized	Oropharyngeal swab
hCoV-19/Mexico/YUC-InDRE_FB16683_S2769/2021	EPI_ISL_2533795	26/05/21	North America / Mexico / Yucatan	Human	Female	20	Hospitalized	Oropharyngeal swab
hCoV-19/Mexico/YUC-InDRE_FB16684_S2770/2021	EPI_ISL_2533796	26/05/21	North America / Mexico / Yucatan	Human	Female	21	Hospitalized	Oropharyngeal swab
hCoV-19/Mexico/YUC-InDRE_FB17112_S3135/2021	EPI_ISL_2663402	01/06/21	North America / Mexico / Yucatan	Human	Male	58	Hospitalized	Oropharyngeal swab
hCoV-19/Mexico/YUC-InDRE_FB17453_S3932/2021	EPI_ISL_2858946	14/06/21	North America / Mexico / Yucatan	Human	Male	55	Hospitalized	Oropharyngeal swab
hCoV-19/Mexico/YUC-InDRE_FB17480_S3493/2021	EPI_ISL_2779008	01/06/21	North America / Mexico / Yucatan	Human	Female	39	Hospitalized	Oropharyngeal swab
hCoV-19/Mexico/YUC-InDRE_FB17482_S3494/2021	EPI_ISL_2779009	01/06/21	North America / Mexico / Yucatan	Human	Female	27	Hospitalized	Oropharyngeal swab
hCoV-19/Mexico/YUC-InDRE_FB17487_S3495/2021	EPI_ISL_2779010	01/06/21	North America / Mexico / Yucatan	Human	Female	47	Hospitalized	Oropharyngeal swab
hCoV-19/Mexico/YUC-InDRE_FB17495_S3496/2021	EPI_ISL_2779011	01/06/21	North America / Mexico / Yucatan	Human	Male	22	Hospitalized	Oropharyngeal swab
hCoV-19/Mexico/YUC-InDRE_FB17498_S3497/2021	EPI_ISL_2779012	01/06/21	North America / Mexico / Yucatan	Human	Female	28	Hospitalized	Oropharyngeal swab
hCoV-19/Mexico/YUC-InDRE_FB17499_S3498/2021	EPI_ISL_2779013	01/06/21	North America / Mexico / Yucatan	Human	Female	21	Hospitalized	Oropharyngeal swab
hCoV-19/Mexico/YUC-InDRE_FB17502_S3499/2021	EPI_ISL_2779014	01/06/21	North America / Mexico / Yucatan	Human	Male	58	Hospitalized	Oropharyngeal swab
hCoV-19/Mexico/YUC-InDRE_FB17506_S3500/2021	EPI_ISL_2779015	01/06/21	North America / Mexico / Yucatan	Human	Female	24	Hospitalized	Oropharyngeal swab
hCoV-19/Mexico/YUC-InDRE_FB17511_S3501/2021	EPI_ISL_2779016	02/06/21	North America / Mexico / Yucatan	Human	Female	25	Hospitalized	Oropharyngeal swab
hCoV-19/Mexico/YUC-InDRE_FB17518_S3502/2021	EPI_ISL_2779017	03/06/21	North America / Mexico / Yucatan	Human	Male	18	Hospitalized	Oropharyngeal swab
hCoV-19/Mexico/YUC-InDRE_FB17531_S3503/2021	EPI_ISL_2779018	04/06/21	North America / Mexico / Yucatan	Human	Female	33	Hospitalized	Oropharyngeal swab
hCoV-19/Mexico/YUC-InDRE_FB17561_S3505/2021	EPI_ISL_2779020	07/06/21	North America / Mexico / Yucatan	Human	Male	37	Hospitalized	Oropharyngeal swab
hCoV-19/Mexico/YUC-InDRE_FB17570_S3506/2021	EPI_ISL_2779021	08/06/21	North America / Mexico / Yucatan	Human	Male	28	Hospitalized	Oropharyngeal swab
hCoV-19/Mexico/YUC-InDRE_FB17577_S3507/2021	EPI_ISL_2779022	09/06/21	North America / Mexico / Yucatan	Human	Female	34	Hospitalized	Oropharyngeal swab
hCoV-19/Mexico/YUC-InDRE_FB17580_S3508/2021	EPI_ISL_2779023	09/06/21	North America / Mexico / Yucatan	Human	Female	54	Hospitalized	Oropharyngeal swab
hCoV-19/Mexico/YUC-InDRE_FB18961_S4481/2021	EPI_ISL_3033335	14/06/21	North America / Mexico / Yucatan	Human	Female	57	Hospitalized	Oropharyngeal swab
hCoV-19/Mexico/YUC-InDRE_FB18962_S4482/2021	EPI_ISL_3033336	15/06/21	North America / Mexico / Yucatan	Human	Female	23	Hospitalized	Oropharyngeal swab
hCoV-19/Mexico/YUC-InDRE_FB18966_S4483/2021	EPI_ISL_3033337	15/06/21	North America / Mexico / Yucatan	Human	Male	46	Hospitalized	Oropharyngeal swab

hCoV-19/Mexico/YUC-InDRE_FB19001_S4484/2021	EPI_ISL_3033338	22/06/21	North America / Mexico / Yucatan	Human	Male	50	Hospitalized	Oropharyngeal swab
hCoV-19/Mexico/YUC-InDRE_FB19002_S4485/2021	EPI_ISL_3033339	22/06/21	North America / Mexico / Yucatan	Human	Male	49	Hospitalized	Oropharyngeal swab
hCoV-19/Mexico/YUC-InDRE_FB19014_S4486/2021	EPI_ISL_3033340	23/06/21	North America / Mexico / Yucatan	Human	Male	24	Hospitalized	Oropharyngeal swab
hCoV-19/Mexico/YUC-INMEGEN-05-06-196/2021	EPI_ISL_2603685	31/05/21	North America / Mexico / Yucatan	Human	Female	46	unknown	Oropharyngeal swab
hCoV-19/Mexico/YUC-INMEGEN-05-06-222/2021	EPI_ISL_2603706	01/06/21	North America / Mexico / Yucatan	Human	Male	41	unknown	Oropharyngeal swab
hCoV-19/Mexico/YUC-INMEGEN-05-06-269/2021	EPI_ISL_2603739	01/06/21	North America / Mexico / Yucatan	Human	Male	42	unknown	Oropharyngeal swab
hCoV-19/Mexico/YUC-INMEGEN-05-06-277/2021	EPI_ISL_2603746	01/06/21	North America / Mexico / Yucatan	Human	Male	30	unknown	Oropharyngeal swab
hCoV-19/Mexico/YUC-INMEGEN-06-01-258/2021	EPI_ISL_2616939	03/06/21	North America / Mexico / Yucatan	Human	Female	25	unknown	Oropharyngeal swab
hCoV-19/Mexico/YUC-INMEGEN-06-01-271/2021	EPI_ISL_2616910	02/06/21	North America / Mexico / Yucatan	Human	Female	32	unknown	Oropharyngeal swab
hCoV-19/Mexico/YUC-INMEGEN-06-01-284/2021	EPI_ISL_2616967	03/06/21	North America / Mexico / Yucatan	Human	Female	28	unknown	Oropharyngeal swab
hCoV-19/Mexico/YUC-INMEGEN-06-01-299/2021	EPI_ISL_2616968	03/06/21	North America / Mexico / Yucatan	Human	Male	23	unknown	Oropharyngeal swab
hCoV-19/Mexico/YUC-INMEGEN-06-01-307/2021	EPI_ISL_2616932	03/06/21	North America / Mexico / Yucatan	Human	Female	32	unknown	Oropharyngeal swab
hCoV-19/Mexico/YUC-INMEGEN-06-01-319/2021	EPI_ISL_2616984	04/06/21	North America / Mexico / Yucatan	Human	Male	39	unknown	Oropharyngeal swab
hCoV-19/Mexico/YUC-INMEGEN-06-01-330/2021	EPI_ISL_2616970	03/06/21	North America / Mexico / Yucatan	Human	Male	34	unknown	Oropharyngeal swab
hCoV-19/Mexico/YUC-INMEGEN-06-01-338/2021	EPI_ISL_2616966	03/06/21	North America / Mexico / Yucatan	Human	Female	41	unknown	Oropharyngeal swab
hCoV-19/Mexico/YUC-INMEGEN-06-02-116/2021	EPI_ISL_2692641	08/06/21	North America / Mexico / Yucatan	Human	Male	31	unknown	Oropharyngeal swab
hCoV-19/Mexico/YUC-INMEGEN-06-02-136/2021	EPI_ISL_2692657	07/06/21	North America / Mexico / Yucatan	Human	Male	25	unknown	Oropharyngeal swab
hCoV-19/Mexico/YUC-INMEGEN-06-02-141/2021	EPI_ISL_2692662	08/06/21	North America / Mexico / Yucatan	Human	Male	58	unknown	Oropharyngeal swab
hCoV-19/Mexico/YUC-INMEGEN-06-02-160/2021	EPI_ISL_2692676	07/06/21	North America / Mexico / Yucatan	Human	Female	44	unknown	Oropharyngeal swab
hCoV-19/Mexico/YUC-INMEGEN-06-02-165/2021	EPI_ISL_2692681	08/06/21	North America / Mexico / Yucatan	Human	Female	33	unknown	Oropharyngeal swab
hCoV-19/Mexico/YUC-INMEGEN-06-02-168/2021	EPI_ISL_2692684	07/06/21	North America / Mexico / Yucatan	Human	Male	46	unknown	Oropharyngeal swab
hCoV-19/Mexico/YUC-INMEGEN-06-02-18/2021	EPI_ISL_2692555	05/06/21	North America / Mexico / Yucatan	Human	Male	29	unknown	Oropharyngeal swab
hCoV-19/Mexico/YUC-INMEGEN-06-02-184/2021	EPI_ISL_2692697	07/06/21	North America / Mexico / Yucatan	Human	Male	20	unknown	Oropharyngeal swab
hCoV-19/Mexico/YUC-INMEGEN-06-02-333/2021	EPI_ISL_2692817	01/06/21	North America / Mexico / Yucatan	Human	Female	28	unknown	Oropharyngeal swab
hCoV-19/Mexico/YUC-INMEGEN-06-02-334/2021	EPI_ISL_2692818	01/06/21	North America / Mexico / Yucatan	Human	Male	30	unknown	Oropharyngeal swab
hCoV-19/Mexico/YUC-INMEGEN-06-02-35/2021	EPI_ISL_2692570	06/06/21	North America / Mexico / Yucatan	Human	Male	42	unknown	Oropharyngeal swab
hCoV-19/Mexico/YUC-INMEGEN-06-02-39/2021	EPI_ISL_2692574	07/06/21	North America / Mexico / Yucatan	Human	Male	28	unknown	Oropharyngeal swab
hCoV-19/Mexico/YUC-INMEGEN-06-02-65/2021	EPI_ISL_2692597	05/06/21	North America / Mexico / Yucatan	Human	Female	26	unknown	Oropharyngeal swab
hCoV-19/Mexico/YUC-INMEGEN-06-02-67/2021	EPI_ISL_2692599	06/06/21	\orth America / Mexico / Quintana Roc	Human	Male	22	unknown	Oropharyngeal swab
hCoV-19/Mexico/YUC-INMEGEN-06-02-88/2021	EPI_ISL_2692618	05/06/21	North America / Mexico / Yucatan	Human	Male	49	unknown	Oropharyngeal swab
hCoV-19/Mexico/YUC-INMEGEN-06-02-9/2021	EPI_ISL_2692547	05/06/21	North America / Mexico / Yucatan	Human	Female	21	unknown	Oropharyngeal swab
hCoV-19/Mexico/YUC-INMEGEN-06-04-289/2021	EPI_ISL_2894336	10/06/21	North America / Mexico / Yucatan	Human	Male	43	unknown	Oropharyngeal swab
hCoV-19/Mexico/YUC-INMEGEN-06-04-29/2021	EPI_ISL_2894176	09/06/21	North America / Mexico / Yucatan	Human	Female	24	unknown	Oropharyngeal swab
hCoV-19/Mexico/YUC-INMEGEN-06-04-293/2021	EPI_ISL_2894340	12/06/21	North America / Mexico / Yucatan	Human	Male	36	unknown	Oropharyngeal swab
hCoV-19/Mexico/YUC-INMEGEN-06-04-296/2021	EPI_ISL_2894343	12/06/21	North America / Mexico / Yucatan	Human	Female	6	unknown	Oropharyngeal swab
hCoV-19/Mexico/YUC-INMEGEN-06-04-299/2021	EPI_ISL_2894346	12/06/21	North America / Mexico / Yucatan	Human	Male	17	unknown	Oropharyngeal swab
hCoV-19/Mexico/YUC-INMEGEN-06-04-308/2021	EPI_ISL_2894355	11/06/21	North America / Mexico / Yucatan	Human	Male	57	unknown	Oropharyngeal swab
hCoV-19/Mexico/YUC-INMEGEN-06-04-310/2021	EPI_ISL_2894357	12/06/21	North America / Mexico / Yucatan	Human	Female	34	unknown	Oropharyngeal swab
hCoV-19/Mexico/YUC-INMEGEN-06-04-338/2021	EPI_ISL_2894382	12/06/21	North America / Mexico / Yucatan	Human	Female	47	unknown	Oropharyngeal swab
hCoV-19/Mexico/YUC-INMEGEN-06-04-345/2021	EPI_ISL_2894389	13/06/21	North America / Mexico / Yucatan	Human	Female	40	unknown	Oropharyngeal swab
hCoV-19/Mexico/YUC-INMEGEN-06-04-348/2021	EPI_ISL_2894392	13/06/21	North America / Mexico / Yucatan	Human	Female	32	unknown	Oropharyngeal swab
hCoV-19/Mexico/YUC-INMEGEN-06-04-349/2021	EPI_ISL_2894393	13/06/21	North America / Mexico / Yucatan	Human	Female	40	unknown	Oropharyngeal swab
hCoV-19/Mexico/YUC-INMEGEN-06-04-43/2021	EPI_ISL_2894187	09/06/21	North America / Mexico / Yucatan	Human	Male	51	unknown	Oropharyngeal swab
hCoV-19/Mexico/YUC-INMEGEN-06-04-59/2021	EPI_ISL_2894203	09/06/21	North America / Mexico / Yucatan	Human	Female	21	unknown	Oropharyngeal swab
hCoV-19/Mexico/YUC-INMEGEN-06-04-75/2021	EPI_ISL_2894219	09/06/21	North America / Mexico / Yucatan	Human	Male	39	unknown	Oropharyngeal swab
hCoV-19/Mexico/YUC-INMEGEN-06-04-84/2021	EPI_ISL_2894227	09/06/21	North America / Mexico / Yucatan	Human	Male	31	unknown	Oropharyngeal swab
hCoV-19/Mexico/YUC-INMEGEN-16-301/2021	EPI_ISL_2978611	16/06/21	North America / Mexico / Yucatan	Human	Female	33	unknown	Oropharyngeal swab
hCoV-19/Mexico/YUC-INMEGEN-16-333/2021	EPI_ISL_2978638	16/06/21	North America / Mexico / Yucatan	Human	Female	23	unknown	Oropharyngeal swab
hCoV-19/Mexico/YUC-INMEGEN-16-340/2021	EPI_ISL_2978645	16/06/21	North America / Mexico / Yucatan	Human	Male	36	unknown	Oropharyngeal swab
hCoV-19/Mexico/YUC-INMEGEN-16-352/2021	EPI_ISL_2978656	23/06/21	North America / Mexico / Yucatan	Human	Male	17	unknown	Oropharyngeal swab
hCoV-19/Mexico/YUC-INMEGEN-16-357/2021	EPI_ISL_2978661	16/06/21	North America / Mexico / Yucatan	Human	Male	22	unknown	Oropharyngeal swab
hCoV-19/Mexico/YUC-INMEGEN-16-361/2021	EPI_ISL_2978665	23/06/21	North America / Mexico / Yucatan	Human	Male	43	unknown	Oropharyngeal swab

hCoV-19/Mexico/YUC-NYGC-1163-SM/2021	EPI_ISL_3023819	26/05/21	North America / Mexico / Yucatan	Human		unknown	unknown	unknown	Oropharyngeal swab
hCoV-19/Mexico/YUC-NYGC-1206-SM/2021	EPI_ISL_3023815	28/04/21	North America / Mexico / Yucatan	Human	Other: Merida	Female	18	Hospitalized	Oropharyngeal swab
hCoV-19/Mexico/YUC-NYGC-1211-SM/2021	EPI_ISL_3023983	05/05/21	North America / Mexico / Yucatan	Human	Other: Merida	unknown	29	Hospitalized	Oropharyngeal swab
hCoV-19/Mexico/YUC-NYGC-1225-SM/2021	EPI_ISL_3023816	03/05/21	North America / Mexico / Yucatan	Human	Other: Merida	Female	48	Hospitalized	Oropharyngeal swab
hCoV-19/Mexico/YUC-NYGC-1232-SM/2021	EPI_ISL_3023948	05/05/21	North America / Mexico / Yucatan	Human	Other: Merida	Male	47	unknown	Oropharyngeal swab
hCoV-19/Mexico/YUC-NYGC-14211-21/2021	EPI_ISL_3024031	17/03/21	North America / Mexico / Yucatan	Human	Other: Chemax	Female	54	Hospitalized	Oropharyngeal swab
hCoV-19/Mexico/YUC-NYGC-14212-21/2021	EPI_ISL_3024035	17/03/21	North America / Mexico / Yucatan	Human	Other: Chemax	Female	52	Hospitalized	Oropharyngeal swab
hCoV-19/Mexico/YUC-NYGC-14715-21/2021	EPI_ISL_3024078	02/03/21	North America / Mexico / Yucatan	Human	Other: Merida	Female	38	Hospitalized	Oropharyngeal swab
hCoV-19/Mexico/YUC-NYGC-15751-21/2021	EPI_ISL_3024024	30/03/21	North America / Mexico / Yucatan	Human	Other: Tizimin	Male	29	Hospitalized	Oropharyngeal swab
hCoV-19/Mexico/YUC-NYGC-8474-21/2021	EPI_ISL_3024018	02/02/21	North America / Mexico / Yucatan	Human	Other: Merida	Male	44	Hospitalized	Oropharyngeal swab
hCoV-19/Mexico/ZAC-InDRE_F10107_S573/2021	EPI_ISL_1335757	15/02/21	North America / Mexico / Zacatecas	Human		Female	40	Hospitalized	Oropharyngeal swab
hCoV-19/Mexico/ZAC-InDRE_FB16088_S2458/2021	EPI_ISL_2484166	18/05/21	North America / Mexico / Zacatecas	Human		Female	79	Deceased	Oropharyngeal swab
hCoV-19/Mexico/ZAC-InDRE_FB17736_S4113/2021	EPI_ISL_2920684	14/06/21	North America / Mexico / Zacatecas	Human		Female	35	Hospitalized	Oropharyngeal swab

Table S3. Patient demographic and clinical characteristics of the sequences classified as B.1.1.519 collected in Mexico from January to June 2021.

Virus name	Accession ID	Collection date	Location	Host	Gender	Patient age	Patient status	Specimen
hCoV-19/Mexico/CMX-INMEGEN-02-02-08/2021	EPI_ISL_1040535	29/01/21	North America / Mexico / Mexico City	Human	Male	47	unknown	Nasopharyngeal swab
hCoV-19/Mexico/CMX-INMEGEN-02-02-10/2021	EPI_ISL_1040536	29/01/21	North America / Mexico / Mexico City	Human	Female	34	unknown	Nasopharyngeal swab
hCoV-19/Mexico/CMX-INMEGEN-02-02-15/2021	EPI_ISL_1040537	29/01/21	North America / Mexico / Mexico City	Human	Female	29	unknown	Nasopharyngeal swab
hCoV-19/Mexico/CMX-INMEGEN-02-02-16/2021	EPI_ISL_1040538	29/01/21	North America / Mexico / Mexico City	Human	Female	37	unknown	Nasopharyngeal swab
hCoV-19/Mexico/CMX-INMEGEN-02-02-21/2021	EPI_ISL_1040539	28/01/21	North America / Mexico / Mexico City	Human	Female	45	unknown	Nasopharyngeal swab
hCoV-19/Mexico/CMX-INMEGEN-02-02-07/2021	EPI_ISL_1040540	27/01/21	North America / Mexico / Mexico City	Human	Female	49	unknown	Nasopharyngeal swab
hCoV-19/Mexico/CMX-INMEGEN-02-02-06/2021	EPI_ISL_1040541	26/01/21	North America / Mexico / Mexico City	Human	Female	60	unknown	Nasopharyngeal swab
hCoV-19/Mexico/CMX-INMEGEN-02-02-09/2021	EPI_ISL_1040542	26/01/21	North America / Mexico / Mexico City	Human	Female	36	unknown	Nasopharyngeal swab
hCoV-19/Mexico/CMX-INMEGEN-02-02-23/2021	EPI_ISL_1040543	26/01/21	North America / Mexico / Mexico City	Human	Female	8	unknown	Nasopharyngeal swab
hCoV-19/Mexico/CMX-INMEGEN-02-02-14/2021	EPI_ISL_1040544	25/01/21	North America / Mexico / Mexico City	Human	Male	27	unknown	Nasopharyngeal swab
hCoV-19/Mexico/CMX-INMEGEN-02-02-22/2021	EPI_ISL_1040546	25/01/21	North America / Mexico / Mexico City	Human	Female	52	unknown	Nasopharyngeal swab
hCoV-19/Mexico/CMX-INMEGEN-02-02-05/2021	EPI_ISL_1040547	24/01/21	North America / Mexico / Mexico City	Human	Male	40	unknown	Nasopharyngeal swab
hCoV-19/Mexico/CMX-INMEGEN-02-02-13/2021	EPI_ISL_1040549	23/01/21	North America / Mexico / Mexico City	Human	Male	70	unknown	Nasopharyngeal swab
hCoV-19/Mexico/CMX-INMEGEN-02-02-20/2021	EPI_ISL_1040550	23/01/21	North America / Mexico / Mexico City	Human	Male	11	unknown	Nasopharyngeal swab
hCoV-19/Mexico/CMX-INMEGEN-02-02-12/2021	EPI_ISL_1040552	22/01/21	North America / Mexico / Mexico City	Human	Male	25	unknown	Nasopharyngeal swab
hCoV-19/Mexico/CMX-INMEGEN-02-02-19/2021	EPI_ISL_1040553	22/01/21	North America / Mexico / Mexico City	Human	Female	51	unknown	Nasopharyngeal swab
hCoV-19/Mexico/CMX-INMEGEN-02-02-01/2021	EPI_ISL_1040554	20/01/21	North America / Mexico / Mexico City	Human	Female	63	unknown	Nasopharyngeal swab
hCoV-19/Mexico/CMX-INMEGEN-02-02-02/2021	EPI_ISL_1040555	20/01/21	North America / Mexico / Mexico City	Human	Male	32	unknown	Nasopharyngeal swab
hCoV-19/Mexico/CMX-INMEGEN-02-02-17/2021	EPI_ISL_1040556	20/01/21	North America / Mexico / Mexico City	Human	Male	42	unknown	Nasopharyngeal swab
hCoV-19/Mexico/CMX-INMEGEN-01-04-11/2021	EPI_ISL_1040560	15/01/21	North America / Mexico / Mexico City	Human	Male	58	unknown	Nasopharyngeal swab
hCoV-19/Mexico/CMX-INMEGEN-01-04-15/2021	EPI_ISL_1040561	14/01/21	North America / Mexico / Mexico City	Human	Female	74	unknown	Nasopharyngeal swab
hCoV-19/Mexico/CMX-INMEGEN-01-04-12/2021	EPI_ISL_1040562	15/01/21	North America / Mexico / Mexico City	Human	Female	58	unknown	Nasopharyngeal swab
hCoV-19/Mexico/CMX-INMEGEN-01-04-16/2021	EPI_ISL_1040563	15/01/21	North America / Mexico / Mexico City	Human	Female	21	unknown	Nasopharyngeal swab
hCoV-19/Mexico/CMX-INMEGEN-01-04-13/2021	EPI_ISL_1040565	17/01/21	North America / Mexico / Mexico City	Human	Female	58	unknown	Nasopharyngeal swab
hCoV-19/Mexico/CMX-INMEGEN-01-04-05/2021	EPI_ISL_1040566	18/01/21	North America / Mexico / Mexico City	Human	Male	11	unknown	Nasopharyngeal swab
hCoV-19/Mexico/CMX-INMEGEN-01-04-06/2021	EPI_ISL_1040567	18/01/21	North America / Mexico / Mexico City	Human	Female	40	unknown	Nasopharyngeal swab
hCoV-19/Mexico/CMX-INMEGEN-01-04-08/2021	EPI_ISL_1040568	18/01/21	North America / Mexico / Mexico City	Human	Female	61	unknown	Nasopharyngeal swab
hCoV-19/Mexico/CMX-INMEGEN-01-04-14/2021	EPI_ISL_1040570	18/01/21	North America / Mexico / Mexico City	Human	Male	38	unknown	Nasopharyngeal swab
hCoV-19/Mexico/CMX-INMEGEN-01-04-17/2021	EPI_ISL_1040571	18/01/21	North America / Mexico / Mexico City	Human	Male	20	unknown	Nasopharyngeal swab
hCoV-19/Mexico/CMX-INMEGEN-01-04-04/2021	EPI_ISL_1040573	19/01/21	North America / Mexico / Mexico City	Human	Male	29	unknown	Nasopharyngeal swab
hCoV-19/Mexico/CMX-INMEGEN-01-04-07/2021	EPI_ISL_1040574	19/01/21	North America / Mexico / Mexico City	Human	Male	60	unknown	Nasopharyngeal swab
hCoV-19/Mexico/CMX-INMEGEN-01-04-10/2021	EPI_ISL_1040575	20/01/21	North America / Mexico / Mexico City	Human	Female	23	unknown	Nasopharyngeal swab
hCoV-19/Mexico/CMX-INMEGEN-01-04-19/2021	EPI_ISL_1040576	20/01/21	North America / Mexico / Mexico City	Human	Female	56	unknown	Nasopharyngeal swab
hCoV-19/Mexico/CMX-INMEGEN-01-04-20/2021	EPI_ISL_1040577	21/01/21	North America / Mexico / Mexico City	Human	Male	52	unknown	Nasopharyngeal swab
hCoV-19/Mexico/CMX-INMEGEN-01-05-06/2021	EPI_ISL_1040578	19/01/21	North America / Mexico / Mexico City	Human	Male	47	unknown	Nasopharyngeal swab
hCoV-19/Mexico/CMX-INMEGEN-01-05-07/2021	EPI_ISL_1040580	18/01/21	North America / Mexico / Mexico City	Human	Male	26	unknown	Nasopharyngeal swab
hCoV-19/Mexico/CMX-INMEGEN-01-05-19/2021	EPI_ISL_1040581	20/01/21	North America / Mexico / Mexico City	Human	Male	27	unknown	Nasopharyngeal swab
hCoV-19/Mexico/CMX-INMEGEN-01-05-20/2021	EPI_ISL_1040582	20/01/21	North America / Mexico / Mexico City	Human	Male	68	unknown	Nasopharyngeal swab
hCoV-19/Mexico/CMX-INMEGEN-01-05-23/2021	EPI_ISL_1040583	20/01/21	North America / Mexico / Mexico City	Human	Male	42	unknown	Nasopharyngeal swab
hCoV-19/Mexico/CMX-INMEGEN-01-05-08/2021	EPI_ISL_1040584	19/01/21	North America / Mexico / Mexico City	Human	Female	58	unknown	Nasopharyngeal swab
hCoV-19/Mexico/CMX-INMEGEN-01-05-11/2021	EPI_ISL_1040585	19/01/21	North America / Mexico / Mexico City	Human	Male	28	unknown	Nasopharyngeal swab

hCoV-19/Mexico/CMX-INMEGEN-01-05-12/2021	EPI_ISL_1040586	19/01/21	North America / Mexico / Mexico City	Human	Female	26	unknown		Nasopharyngeal swab
hCoV-19/Mexico/CMX-INMEGEN-01-05-05/2021	EPI_ISL_1040587	18/01/21	North America / Mexico / Mexico City	Human	Male	55	unknown		Nasopharyngeal swab
hCoV-19/Mexico/CMX-INMEGEN-01-05-14/2021	EPI_ISL_1040588	18/01/21	North America / Mexico / Mexico City	Human	Male	68	unknown		Nasopharyngeal swab
hCoV-19/Mexico/CMX-INMEGEN-01-05-15/2021	EPI_ISL_1040589	18/01/21	North America / Mexico / Mexico City	Human	Female	52	unknown		Nasopharyngeal swab
hCoV-19/Mexico/CMX-INMEGEN-01-05-16/2021	EPI_ISL_1040590	18/01/21	North America / Mexico / Mexico City	Human	Male	21	unknown		Nasopharyngeal swab
hCoV-19/Mexico/CMX-INMEGEN-01-05-01/2021	EPI_ISL_1040591	16/01/21	North America / Mexico / Mexico City	Human	Female	37	unknown		Nasopharyngeal swab
hCoV-19/Mexico/CMX-INMEGEN-01-05-04/2021	EPI_ISL_1040592	16/01/21	North America / Mexico / Mexico City	Human	Female	47	unknown		Nasopharyngeal swab
hCoV-19/Mexico/CMX-INMEGEN-01-05-02/2021	EPI_ISL_1040593	15/01/21	North America / Mexico / Mexico City	Human	Male	47	unknown		Nasopharyngeal swab
hCoV-19/Mexico/CMX-INMEGEN-01-05-03/2021	EPI_ISL_1040594	15/01/21	North America / Mexico / Mexico City	Human	Male	25	unknown		Nasopharyngeal swab
hCoV-19/Mexico/CMX-INMEGEN-01-05-09/2021	EPI_ISL_1040595	15/01/21	North America / Mexico / Mexico City	Human	Female	39	unknown		Nasopharyngeal swab
hCoV-19/Mexico/CMX-INMEGEN-01-05-18/2021	EPI_ISL_1040597	15/01/21	North America / Mexico / Mexico City	Human	Female	3	unknown		Nasopharyngeal swab
hCoV-19/Mexico/CMX-INMEGEN-01-05-17/2021	EPI_ISL_1040598	14/01/21	North America / Mexico / Mexico City	Human	Female	83	unknown		Nasopharyngeal swab
hCoV-19/Mexico/CMX-INMEGEN-01-05-21/2021	EPI_ISL_1040599	19/01/21	North America / Mexico / Mexico City	Human	Male	19	unknown		Nasopharyngeal swab
hCoV-19/Mexico/CMX-INMEGEN-02-01-22/2021	EPI_ISL_1040601	26/01/21	North America / Mexico / Mexico City	Human	Male	32	unknown		Nasopharyngeal swab
hCoV-19/Mexico/CMX-INMEGEN-02-01-20/2021	EPI_ISL_1040602	23/01/21	North America / Mexico / Mexico City	Human	Female	29	unknown		Nasopharyngeal swab
hCoV-19/Mexico/CMX-INMEGEN-02-01-16/2021	EPI_ISL_1040605	28/01/21	North America / Mexico / Mexico City	Human	Female	29	unknown		Nasopharyngeal swab
hCoV-19/Mexico/CMX-INMEGEN-02-01-14/2021	EPI_ISL_1040606	27/01/21	North America / Mexico / Mexico City	Human	Male	26	unknown		Nasopharyngeal swab
hCoV-19/Mexico/CMX-INMEGEN-02-01-15/2021	EPI_ISL_1040607	27/01/21	North America / Mexico / Mexico City	Human	Female	47	unknown		Nasopharyngeal swab
hCoV-19/Mexico/CMX-INMEGEN-02-01-13/2021	EPI_ISL_1040609	26/01/21	North America / Mexico / Mexico City	Human	Male	31	unknown		Nasopharyngeal swab
hCoV-19/Mexico/CMX-INMEGEN-02-01-21/2021	EPI_ISL_1040610	26/01/21	North America / Mexico / Mexico City	Human	Male	47	unknown		Nasopharyngeal swab
hCoV-19/Mexico/CMX-INMEGEN-02-01-10/2021	EPI_ISL_1040613	25/01/21	North America / Mexico / Mexico City	Human	Female	11	unknown		Nasopharyngeal swab
hCoV-19/Mexico/CMX-INMEGEN-02-01-11/2021	EPI_ISL_1040614	25/01/21	North America / Mexico / Mexico City	Human	Female	10	unknown		Nasopharyngeal swab
hCoV-19/Mexico/CMX-INMEGEN-02-01-23/2021	EPI_ISL_1040616	25/01/21	North America / Mexico / Mexico City	Human	Female	63	unknown		Nasopharyngeal swab
hCoV-19/Mexico/CMX-INMEGEN-02-01-08/2021	EPI_ISL_1040617	24/01/21	North America / Mexico / Mexico City	Human	Male	71	unknown		Nasopharyngeal swab
hCoV-19/Mexico/CMX-INMEGEN-02-01-09/2021	EPI_ISL_1040619	22/01/21	North America / Mexico / Mexico City	Human	Male	41	unknown		Nasopharyngeal swab
hCoV-19/Mexico/CMX-INMEGEN-02-01-18/2021	EPI_ISL_1040620	22/01/21	North America / Mexico / Mexico City	Human	Female	59	unknown		Nasopharyngeal swab
hCoV-19/Mexico/CMX-INMEGEN-02-01-02/2021	EPI_ISL_1040621	17/01/21	North America / Mexico / Mexico City	Human	Female	63	unknown		Nasopharyngeal swab
hCoV-19/Mexico/CMX-INMEGEN-02-01-03/2021	EPI_ISL_1040622	17/01/21	North America / Mexico / Mexico City	Human	Female	91	unknown		Nasopharyngeal swab
hCoV-19/Mexico/CMX-INMEGEN-02-03-09/2021	EPI_ISL_1040623	02/02/21	North America / Mexico / Mexico City	Human	Male	25	unknown		Nasopharyngeal swab
hCoV-19/Mexico/CMX-INMEGEN-02-03-15/2021	EPI_ISL_1040624	30/01/21	North America / Mexico / Mexico City	Human	Male	6	unknown		Nasopharyngeal swab
hCoV-19/Mexico/CMX-INMEGEN-02-03-11/2021	EPI_ISL_1040625	05/02/21	North America / Mexico / Mexico City	Human	Male	31	unknown		Nasopharyngeal swab
hCoV-19/Mexico/CMX-INMEGEN-02-03-12/2021	EPI_ISL_1040626	05/02/21	North America / Mexico / Mexico City	Human	Male	5	unknown		Nasopharyngeal swab
hCoV-19/Mexico/CMX-INMEGEN-02-03-02/2021	EPI_ISL_1040627	04/02/21	North America / Mexico / Mexico City	Human	Male	41	unknown		Nasopharyngeal swab
hCoV-19/Mexico/CMX-INMEGEN-02-03-03/2021	EPI_ISL_1040628	04/02/21	North America / Mexico / Mexico City	Human	Female	19	unknown		Nasopharyngeal swab
hCoV-19/Mexico/CMX-INMEGEN-02-03-04/2021	EPI_ISL_1040629	04/02/21	North America / Mexico / Mexico City	Human	Female	45	unknown		Nasopharyngeal swab
hCoV-19/Mexico/CMX-INMEGEN-02-03-10/2021	EPI_ISL_1040630	04/02/21	North America / Mexico / Mexico City	Human	Female	77	unknown		Nasopharyngeal swab
hCoV-19/Mexico/CMX-INMEGEN-02-03-08/2021	EPI_ISL_1040631	03/02/21	North America / Mexico / Mexico City	Human	Male	30	unknown		Nasopharyngeal swab
hCoV-19/Mexico/CMX-INMEGEN-02-03-22/2021	EPI_ISL_1040632	03/02/21	North America / Mexico / Mexico City	Human	Female	38	unknown		Nasopharyngeal swab
hCoV-19/Mexico/CMX-INMEGEN-02-03-23/2021	EPI_ISL_1040633	03/02/21	North America / Mexico / Mexico City	Human	Male	49	unknown		Nasopharyngeal swab
hCoV-19/Mexico/CMX-INMEGEN-02-03-14/2021	EPI_ISL_1040634	02/02/21	North America / Mexico / Mexico City	Human	Male	67	unknown		Nasopharyngeal swab
hCoV-19/Mexico/CMX-INMEGEN-02-03-20/2021	EPI_ISL_1040635	02/02/21	North America / Mexico / Mexico City	Human	Female	71	unknown		Nasopharyngeal swab
hCoV-19/Mexico/CMX-INMEGEN-02-03-21/2021	EPI_ISL_1040636	02/02/21	North America / Mexico / Mexico City	Human	Male	39	unknown		Nasopharyngeal swab
hCoV-19/Mexico/CMX-INMEGEN-02-03-01/2021	EPI_ISL_1040637	31/01/21	North America / Mexico / Mexico City	Human	Male	53	unknown		Nasopharyngeal swab
hCoV-19/Mexico/CMX-INMEGEN-02-03-06/2021	EPI_ISL_1040638	30/01/21	North America / Mexico / Mexico City	Human	Male	19	unknown		Nasopharyngeal swab

hCoV-19/Mexico/CMX-INMEGEN-02-03-16/2021	EPI_ISL_1040639	30/01/21	North America / Mexico / Mexico City	Human	Male	12	unknown	Nasopharyngeal swab
hCoV-19/Mexico/CMX-INMEGEN-02-03-05/2021	EPI_ISL_1040640	29/01/21	North America / Mexico / Mexico City	Human	Male	34	unknown	Nasopharyngeal swab
hCoV-19/Mexico/CMX-INMEGEN-02-03-17/2021	EPI_ISL_1040641	29/01/21	North America / Mexico / Mexico City	Human	Male	53	unknown	Nasopharyngeal swab
hCoV-19/Mexico/CMX-INMEGEN-02-03-19/2021	EPI_ISL_1040642	29/01/21	North America / Mexico / Mexico City	Human	Male	59	unknown	Nasopharyngeal swab
hCoV-19/Mexico/CMX-INMEGEN-02-03-18/2021	EPI_ISL_1040643	27/01/21	North America / Mexico / Mexico City	Human	Female	78	unknown	Nasopharyngeal swab
hCoV-19/Mexico/CMX-INER-SC239/2021	EPI_ISL_1040927	05/01/21	North America / Mexico / Mexico City	Human	Male	53	Live	Oropharyngeal swab
hCoV-19/Mexico/CMX-INMEGEN-02-04-17/2021	EPI_ISL_1055005	05/02/21	North America / Mexico / Mexico City	Human	Female	85	unknown	Oropharyngeal swab
hCoV-19/Mexico/CMX-INMEGEN-02-04-15/2021	EPI_ISL_1055006	05/02/21	North America / Mexico / Mexico City	Human	Female	54	unknown	Oropharyngeal swab
hCoV-19/Mexico/CMX-INMEGEN-02-04-16/2021	EPI_ISL_1055007	05/02/21	North America / Mexico / Mexico City	Human	Male	61	unknown	Oropharyngeal swab
hCoV-19/Mexico/CMX-INMEGEN-02-04-19/2021	EPI_ISL_1055008	05/02/21	North America / Mexico / Mexico City	Human	Female	24	unknown	Oropharyngeal swab
hCoV-19/Mexico/CMX-INMEGEN-02-04-02/2021	EPI_ISL_1055009	04/02/21	North America / Mexico / Mexico City	Human	Male	30	unknown	Oropharyngeal swab
hCoV-19/Mexico/CMX-INMEGEN-02-04-03/2021	EPI_ISL_1055010	04/02/21	North America / Mexico / Mexico City	Human	Female	33	unknown	Oropharyngeal swab
hCoV-19/Mexico/CMX-INMEGEN-02-04-04/2021	EPI_ISL_1055011	04/02/21	North America / Mexico / Mexico City	Human	Male	27	unknown	Oropharyngeal swab
hCoV-19/Mexico/CMX-INMEGEN-02-04-12/2021	EPI_ISL_1055012	04/02/21	North America / Mexico / Mexico City	Human	Male	27	unknown	Oropharyngeal swab
hCoV-19/Mexico/CMX-INMEGEN-02-04-13/2021	EPI_ISL_1055013	04/02/21	North America / Mexico / Mexico City	Human	Female	43	unknown	Oropharyngeal swab
hCoV-19/Mexico/CMX-INMEGEN-02-04-14/2021	EPI_ISL_1055014	04/02/21	North America / Mexico / Mexico City	Human	Male	51	unknown	Oropharyngeal swab
hCoV-19/Mexico/CMX-INMEGEN-02-04-21/2021	EPI_ISL_1055015	04/02/21	North America / Mexico / Mexico City	Human	Male	52	unknown	Oropharyngeal swab
hCoV-19/Mexico/CMX-INMEGEN-02-04-22/2021	EPI_ISL_1055016	04/02/21	North America / Mexico / Mexico City	Human	Female	27	unknown	Oropharyngeal swab
hCoV-19/Mexico/CMX-INMEGEN-02-04-01/2021	EPI_ISL_1055017	03/02/21	North America / Mexico / Mexico City	Human	Male	29	unknown	Oropharyngeal swab
hCoV-19/Mexico/CMX-INMEGEN-02-04-11/2021	EPI_ISL_1055019	03/02/21	North America / Mexico / Mexico City	Human	Female	44	unknown	Oropharyngeal swab
hCoV-19/Mexico/CMX-INMEGEN-02-04-20/2021	EPI_ISL_1055020	03/02/21	North America / Mexico / Mexico City	Human	Female	29	unknown	Oropharyngeal swab
hCoV-19/Mexico/CMX-INMEGEN-02-04-06/2021	EPI_ISL_1055021	02/02/21	North America / Mexico / Mexico City	Human	Female	37	unknown	Oropharyngeal swab
hCoV-19/Mexico/CMX-INMEGEN-02-04-09/2021	EPI_ISL_1055022	02/02/21	North America / Mexico / Mexico City	Human	Male	49	unknown	Oropharyngeal swab
hCoV-19/Mexico/CMX-INMEGEN-02-04-07/2021	EPI_ISL_1055024	29/01/21	North America / Mexico / Mexico City	Human	Female	56	unknown	Oropharyngeal swab
hCoV-19/Mexico/CMX-INMEGEN-02-04-08/2021	EPI_ISL_1055025	29/01/21	North America / Mexico / Mexico City	Human	Male	54	unknown	Oropharyngeal swab
hCoV-19/Mexico/CMX-INMEGEN-02-04-18/2021	EPI_ISL_1058046	05/02/21	North America / Mexico / Mexico City	Human	Female	51	unknown	
hCoV-19/Mexico/CMX-INMEGEN-02-05-19/2021	EPI_ISL_1060598	05/02/21	North America / Mexico / Mexico City	Human	Female	57	unknown	Oropharyngeal swab
hCoV-19/Mexico/CMX-INMEGEN-02-05-20/2021	EPI_ISL_1060599	05/02/21	North America / Mexico / Mexico City	Human	Male	46	unknown	Oropharyngeal swab
hCoV-19/Mexico/CMX-INMEGEN-02-05-10/2021	EPI_ISL_1060601	03/02/21	North America / Mexico / Mexico City	Human	Female	5	unknown	Oropharyngeal swab
hCoV-19/Mexico/CMX-INMEGEN-02-05-11/2021	EPI_ISL_1060602	03/02/21	North America / Mexico / Mexico City	Human	Male	24	unknown	Oropharyngeal swab
hCoV-19/Mexico/CMX-INMEGEN-02-05-12/2021	EPI_ISL_1060603	03/02/21	North America / Mexico / Mexico City	Human	Female	42	unknown	Oropharyngeal swab
hCoV-19/Mexico/CMX-INMEGEN-02-05-14/2021	EPI_ISL_1060604	03/02/21	North America / Mexico / Mexico City	Human	Male	63	unknown	Oropharyngeal swab
hCoV-19/Mexico/CMX-INMEGEN-02-05-15/2021	EPI_ISL_1060605	03/02/21	North America / Mexico / Mexico City	Human	Female	69	unknown	Oropharyngeal swab
hCoV-19/Mexico/CMX-INMEGEN-02-05-16/2021	EPI_ISL_1060606	03/02/21	North America / Mexico / Mexico City	Human	Female	45	unknown	Oropharyngeal swab
hCoV-19/Mexico/CMX-INMEGEN-02-05-17/2021	EPI_ISL_1060607	03/02/21	North America / Mexico / Mexico City	Human	Male	27	unknown	Oropharyngeal swab
hCoV-19/Mexico/CMX-INMEGEN-02-05-18/2021	EPI_ISL_1060608	03/02/21	North America / Mexico / Mexico City	Human	Male	41	unknown	Oropharyngeal swab
hCoV-19/Mexico/CMX-INMEGEN-02-05-21/2021	EPI_ISL_1060609	03/02/21	North America / Mexico / Mexico City	Human	Female	32	unknown	Oropharyngeal swab
hCoV-19/Mexico/CMX-INMEGEN-02-05-23/2021	EPI_ISL_1060610	03/02/21	North America / Mexico / Mexico City	Human	Female	50	unknown	Oropharyngeal swab
hCoV-19/Mexico/CMX-INMEGEN-02-05-22/2021	EPI_ISL_1060611	01/02/21	North America / Mexico / Mexico City	Human	Female	65	unknown	Oropharyngeal swab
hCoV-19/Mexico/QUE-InDRE_246/2021	EPI_ISL_1060697	14/01/21	North America / Mexico / Queretaro	Human	Female	34	unknown	Oropharyngeal swab
hCoV-19/Mexico/CMX-InDRE_248/2021	EPI_ISL_1060714	02/02/21	North America / Mexico / Mexico City	Human	Female	unknown	unknown	Oropharyngeal swab
hCoV-19/Mexico/CMX-InDRE_249/2021	EPI_ISL_1060716	28/01/21	North America / Mexico / Mexico City	Human	Female	unknown	unknown	Oropharyngeal swab
hCoV-19/Mexico/CMX-InDRE_312/2021	EPI_ISL_1060717	27/01/21	North America / Mexico / Mexico City	Human	Male	67	unknown	Oropharyngeal swab
hCoV-19/Mexico/CMX-INMEGEN-02-06-12/2021	EPI_ISL_1080433	04/02/21	North America / Mexico / Mexico City	Human	Male	14	unknown	Oropharyngeal swab

hCoV-19/Mexico/CMX-INMEGEN-02-06-13/2021	EPI_ISL_1080434	04/02/21	North America / Mexico / Mexico City	Human	Male	17	unknown	Oropharyngeal swab
hCoV-19/Mexico/CMX-INMEGEN-02-06-17/2021	EPI_ISL_1080435	04/02/21	North America / Mexico / Mexico City	Human	Female	25	unknown	Oropharyngeal swab
hCoV-19/Mexico/CMX-INMEGEN-02-06-04/2021	EPI_ISL_1080436	03/02/21	North America / Mexico / Mexico City	Human	Male	24	unknown	Oropharyngeal swab
hCoV-19/Mexico/CMX-INMEGEN-02-06-21/2021	EPI_ISL_1080437	08/02/21	North America / Mexico / Mexico City	Human	Female	22	unknown	Oropharyngeal swab
hCoV-19/Mexico/CMX-INMEGEN-02-06-03/2021	EPI_ISL_1080440	03/02/21	North America / Mexico / Mexico City	Human	Female	41	unknown	Oropharyngeal swab
hCoV-19/Mexico/CMX-INMEGEN-02-06-05/2021	EPI_ISL_1080441	03/02/21	North America / Mexico / Mexico City	Human	Male	43	unknown	Oropharyngeal swab
hCoV-19/Mexico/CMX-INMEGEN-02-06-07/2021	EPI_ISL_1080443	03/02/21	North America / Mexico / Mexico City	Human	Male	27	unknown	Oropharyngeal swab
hCoV-19/Mexico/CMX-INMEGEN-02-06-08/2021	EPI_ISL_1080444	03/02/21	North America / Mexico / Mexico City	Human	Female	54	unknown	Oropharyngeal swab
hCoV-19/Mexico/CMX-INMEGEN-02-06-09/2021	EPI_ISL_1080445	03/02/21	North America / Mexico / Mexico City	Human	Male	48	unknown	Oropharyngeal swab
hCoV-19/Mexico/CMX-INMEGEN-02-06-11/2021	EPI_ISL_1080447	01/02/21	North America / Mexico / Mexico City	Human	Female	40	unknown	Oropharyngeal swab
hCoV-19/Mexico/CMX-INMEGEN-02-06-14/2021	EPI_ISL_1080448	04/02/21	North America / Mexico / Mexico City	Human	Female	34	unknown	Oropharyngeal swab
hCoV-19/Mexico/CMX-INMEGEN-02-06-15/2021	EPI_ISL_1080449	04/02/21	North America / Mexico / Mexico City	Human	Male	25	unknown	Oropharyngeal swab
hCoV-19/Mexico/CMX-INMEGEN-02-06-16/2021	EPI_ISL_1080450	04/02/21	North America / Mexico / Mexico City	Human	Female	53	unknown	Oropharyngeal swab
hCoV-19/Mexico/CMX-INMEGEN-02-06-18/2021	EPI_ISL_1080451	05/02/21	North America / Mexico / Mexico City	Human	Male	7	unknown	Oropharyngeal swab
hCoV-19/Mexico/CMX-INMEGEN-02-06-19/2021	EPI_ISL_1080452	05/02/21	North America / Mexico / Mexico City	Human	Female	31	unknown	Oropharyngeal swab
hCoV-19/Mexico/CMX-INMEGEN-02-06-23/2021	EPI_ISL_1080453	08/02/21	North America / Mexico / Mexico City	Human	Male	51	unknown	Oropharyngeal swab
hCoV-19/Mexico/CMX-INMEGEN-02-07-01/2021	EPI_ISL_1081152	10/02/21	North America / Mexico / Mexico City	Human	Female	63	unknown	Oropharyngeal swab
hCoV-19/Mexico/CMX-INMEGEN-02-07-03/2021	EPI_ISL_1081154	10/02/21	North America / Mexico / Mexico City	Human	Female	68	unknown	Oropharyngeal swab
hCoV-19/Mexico/CMX-INMEGEN-02-07-04/2021	EPI_ISL_1081155	10/02/21	North America / Mexico / Mexico City	Human	Male	80	unknown	Oropharyngeal swab
hCoV-19/Mexico/CMX-INMEGEN-02-07-13/2021	EPI_ISL_1081156	10/02/21	North America / Mexico / Mexico City	Human	Female	29	unknown	Oropharyngeal swab
hCoV-19/Mexico/CMX-INMEGEN-02-07-17/2021	EPI_ISL_1081158	10/02/21	North America / Mexico / Mexico City	Human	Male	14	unknown	Oropharyngeal swab
hCoV-19/Mexico/CMX-INMEGEN-02-07-18/2021	EPI_ISL_1081159	10/02/21	North America / Mexico / Mexico City	Human	Female	28	unknown	Oropharyngeal swab
hCoV-19/Mexico/CMX-INMEGEN-02-07-19/2021	EPI_ISL_1081160	10/02/21	North America / Mexico / Mexico City	Human	Female	66	unknown	Oropharyngeal swab
hCoV-19/Mexico/CMX-INMEGEN-02-07-20/2021	EPI_ISL_1081161	10/02/21	North America / Mexico / Mexico City	Human	Male	50	unknown	Oropharyngeal swab
hCoV-19/Mexico/CMX-INMEGEN-02-07-07/2021	EPI_ISL_1081162	09/02/21	North America / Mexico / Mexico City	Human	Female	65	unknown	Oropharyngeal swab
hCoV-19/Mexico/CMX-INMEGEN-02-07-08/2021	EPI_ISL_1081163	09/02/21	North America / Mexico / Mexico City	Human	Female	75	unknown	Oropharyngeal swab
hCoV-19/Mexico/CMX-INMEGEN-02-07-10/2021	EPI_ISL_1081164	09/02/21	North America / Mexico / Mexico City	Human	Female	35	unknown	Oropharyngeal swab
hCoV-19/Mexico/CMX-INMEGEN-02-07-09/2021	EPI_ISL_1081165	08/02/21	North America / Mexico / Mexico City	Human	Female	47	unknown	Oropharyngeal swab
hCoV-19/Mexico/CMX-INMEGEN-02-07-11/2021	EPI_ISL_1081166	05/02/21	North America / Mexico / Mexico City	Human	Female	28	unknown	Oropharyngeal swab
hCoV-19/Mexico/CMX-INMEGEN-02-07-12/2021	EPI_ISL_1081167	05/02/21	North America / Mexico / Mexico City	Human	Female	30	unknown	Oropharyngeal swab
hCoV-19/Mexico/CMX-INMEGEN-02-07-22/2021	EPI_ISL_1081168	05/02/21	North America / Mexico / Mexico City	Human	Female	31	unknown	Oropharyngeal swab
hCoV-19/Mexico/CMX-INMEGEN-02-07-23/2021	EPI_ISL_1081169	05/02/21	North America / Mexico / Mexico City	Human	Male	27	unknown	Oropharyngeal swab
hCoV-19/Mexico/NLE-UANL-019/2021	EPI_ISL_1091253	15/01/21	North America / Mexico / Nuevo Leon	Human	Male	63	unknown	
hCoV-19/Mexico/CMX-INMEGEN-02-07-05/2021	EPI_ISL_1095493	07/02/21	North America / Mexico / Mexico City	Human	Female	29	unknown	Oropharyngeal swab
hCoV-19/Mexico/CMX-INMEGEN-02-07-06/2021	EPI_ISL_1095494	07/02/21	North America / Mexico / Mexico City	Human	Male	76	unknown	Oropharyngeal swab
hCoV-19/Mexico/CMX-INMEGEN-02-07-16/2021	EPI_ISL_1095495	10/02/21	North America / Mexico / Mexico City	Human	Male	66	unknown	Oropharyngeal swab
hCoV-19/Mexico/CMX-INMEGEN-02-09-05/2021	EPI_ISL_1095496	06/02/21	North America / Mexico / Mexico City	Human	Male	26	unknown	Oropharyngeal swab
hCoV-19/Mexico/CMX-INMEGEN-02-09-08/2021	EPI_ISL_1095497	08/02/21	North America / Mexico / Mexico City	Human	Female	30	unknown	Oropharyngeal swab
hCoV-19/Mexico/CMX-INMEGEN-02-09-09/2021	EPI_ISL_1095498	08/02/21	North America / Mexico / Mexico City	Human	Female	68	unknown	Oropharyngeal swab
hCoV-19/Mexico/CMX-INMEGEN-02-09-11/2021	EPI_ISL_1095499	08/02/21	North America / Mexico / Mexico City	Human	Female	23	unknown	Oropharyngeal swab
hCoV-19/Mexico/CMX-INMEGEN-02-09-12/2021	EPI_ISL_1095500	08/02/21	North America / Mexico / Mexico City	Human	Male	56	unknown	Oropharyngeal swab
hCoV-19/Mexico/CMX-INMEGEN-02-09-13/2021	EPI_ISL_1095501	08/02/21	North America / Mexico / Mexico City	Human	Female	24	unknown	Oropharyngeal swab
hCoV-19/Mexico/CMX-INMEGEN-02-09-14/2021	EPI_ISL_1095502	08/02/21	North America / Mexico / Mexico City	Human	Female	24	unknown	Oropharyngeal swab
hCoV-19/Mexico/CMX-INMEGEN-02-09-15/2021	EPI_ISL_1095503	08/02/21	North America / Mexico / Mexico City	Human	Male	25	unknown	Oropharyngeal swab

hCoV-19/Mexico/CMX-INMEGEN-02-09-16/2021	EPI_ISL_1095504	08/02/21	North America / Mexico / Mexico City	Human	Female	24	unknown	Oropharyngeal swab
hCoV-19/Mexico/CMX-INMEGEN-02-09-17/2021	EPI_ISL_1095505	08/02/21	North America / Mexico / Mexico City	Human	Female	24	unknown	Oropharyngeal swab
hCoV-19/Mexico/CMX-INMEGEN-02-09-19/2021	EPI_ISL_1095506	08/02/21	North America / Mexico / Mexico City	Human	Female	24	unknown	Oropharyngeal swab
hCoV-19/Mexico/CMX-INMEGEN-02-09-20/2021	EPI_ISL_1095507	08/02/21	North America / Mexico / Mexico City	Human	Female	24	unknown	Oropharyngeal swab
hCoV-19/Mexico/CMX-INMEGEN-02-09-02/2021	EPI_ISL_1095508	07/02/21	North America / Mexico / Mexico City	Human	Male	54	unknown	Oropharyngeal swab
hCoV-19/Mexico/CMX-INMEGEN-02-09-03/2021	EPI_ISL_1095509	06/02/21	North America / Mexico / Mexico City	Human	Male	44	unknown	Oropharyngeal swab
hCoV-19/Mexico/CMX-INMEGEN-02-09-04/2021	EPI_ISL_1095510	06/02/21	North America / Mexico / Mexico City	Human	Female	45	unknown	Oropharyngeal swab
hCoV-19/Mexico/CMX-INMEGEN-02-09-06/2021	EPI_ISL_1095511	05/02/21	North America / Mexico / Mexico City	Human	Male	24	unknown	Oropharyngeal swab
hCoV-19/Mexico/CMX-INMEGEN-02-09-21/2021	EPI_ISL_1095513	05/02/21	North America / Mexico / Mexico City	Human	Female	31	unknown	Oropharyngeal swab
hCoV-19/Mexico/CMX-INMEGEN-02-09-22/2021	EPI_ISL_1095514	05/02/21	North America / Mexico / Mexico City	Human	Male	28	unknown	Oropharyngeal swab
hCoV-19/Mexico/CMX-INMEGEN-02-09-23/2021	EPI_ISL_1095515	05/02/21	North America / Mexico / Mexico City	Human	Female	45	unknown	Oropharyngeal swab
hCoV-19/Mexico/CMX-INMEGEN-02-11-01/2021	EPI_ISL_1117353	08/02/21	North America / Mexico / Mexico City	Human	Male	19	unknown	Oropharyngeal swab
hCoV-19/Mexico/CMX-INMEGEN-02-11-02/2021	EPI_ISL_1117354	08/02/21	North America / Mexico / Mexico City	Human	Female	34	unknown	Oropharyngeal swab
hCoV-19/Mexico/CMX-INMEGEN-02-11-03/2021	EPI_ISL_1117355	08/02/21	North America / Mexico / Mexico City	Human	Female	15	unknown	Oropharyngeal swab
hCoV-19/Mexico/CMX-INMEGEN-02-11-04/2021	EPI_ISL_1117356	09/02/21	North America / Mexico / Mexico City	Human	Male	71	unknown	Oropharyngeal swab
hCoV-19/Mexico/CMX-INMEGEN-02-11-07/2021	EPI_ISL_1117359	09/02/21	North America / Mexico / Mexico City	Human	Female	64	unknown	Oropharyngeal swab
hCoV-19/Mexico/CMX-INMEGEN-02-11-08/2021	EPI_ISL_1117360	09/02/21	North America / Mexico / Mexico City	Human	Female	55	unknown	Oropharyngeal swab
hCoV-19/Mexico/CMX-INMEGEN-02-11-09/2021	EPI_ISL_1117361	08/02/21	North America / Mexico / Mexico City	Human	Male	48	unknown	Oropharyngeal swab
hCoV-19/Mexico/CMX-INMEGEN-02-11-10/2021	EPI_ISL_1117362	08/02/21	North America / Mexico / Mexico City	Human	Male	59	unknown	Oropharyngeal swab
hCoV-19/Mexico/CMX-INMEGEN-02-11-11/2021	EPI_ISL_1117363	08/02/21	North America / Mexico / Mexico City	Human	Male	65	unknown	Oropharyngeal swab
hCoV-19/Mexico/CMX-INMEGEN-02-11-13/2021	EPI_ISL_1117365	09/02/21	North America / Mexico / Mexico City	Human	Male	76	unknown	Oropharyngeal swab
hCoV-19/Mexico/CMX-INMEGEN-02-11-15/2021	EPI_ISL_1117367	05/02/21	North America / Mexico / Mexico City	Human	Female	66	unknown	Oropharyngeal swab
hCoV-19/Mexico/CMX-INMEGEN-02-11-16/2021	EPI_ISL_1117368	06/02/21	North America / Mexico / Mexico City	Human	Male	53	unknown	Oropharyngeal swab
hCoV-19/Mexico/CMX-INMEGEN-02-11-17/2021	EPI_ISL_1117369	06/02/21	North America / Mexico / Mexico City	Human	Female	54	unknown	Oropharyngeal swab
hCoV-19/Mexico/CMX-INMEGEN-02-11-18/2021	EPI_ISL_1117370	06/02/21	North America / Mexico / Mexico City	Human	Male	52	unknown	Oropharyngeal swab
hCoV-19/Mexico/CMX-INMEGEN-02-11-19/2021	EPI_ISL_1117371	10/02/21	North America / Mexico / Mexico City	Human	Female	57	unknown	Oropharyngeal swab
hCoV-19/Mexico/CMX-INMEGEN-02-11-20/2021	EPI_ISL_1117372	09/02/21	North America / Mexico / Mexico City	Human	Male	39	unknown	Oropharyngeal swab
hCoV-19/Mexico/CMX-INMEGEN-02-11-22/2021	EPI_ISL_1117374	10/02/21	North America / Mexico / Mexico City	Human	Female	43	unknown	Oropharyngeal swab
hCoV-19/Mexico/CMX-INMEGEN-02-11-23/2021	EPI_ISL_1117375	09/02/21	North America / Mexico / Mexico City	Human	Male	68	unknown	Oropharyngeal swab
hCoV-19/Mexico/CMX-INMEGEN-02-12-01/2021	EPI_ISL_1120878	12/02/21	North America / Mexico / Mexico City	Human	Female	28	unknown	Oropharyngeal swab
hCoV-19/Mexico/CMX-INMEGEN-02-12-02/2021	EPI_ISL_1120879	11/02/21	North America / Mexico / Mexico City	Human	Male	56	unknown	Oropharyngeal swab
hCoV-19/Mexico/CMX-INMEGEN-02-12-04/2021	EPI_ISL_1120881	10/02/21	North America / Mexico / Mexico City	Human	Male	39	unknown	Oropharyngeal swab
hCoV-19/Mexico/CMX-INMEGEN-02-12-05/2021	EPI_ISL_1120882	11/02/21	North America / Mexico / Mexico City	Human	Male	76	unknown	Oropharyngeal swab
hCoV-19/Mexico/CMX-INMEGEN-02-12-06/2021	EPI_ISL_1120883	10/02/21	North America / Mexico / Mexico City	Human	Female	63	unknown	Oropharyngeal swab
hCoV-19/Mexico/CMX-INMEGEN-02-12-07/2021	EPI_ISL_1120884	10/02/21	North America / Mexico / Mexico City	Human	Female	53	unknown	Oropharyngeal swab
hCoV-19/Mexico/CMX-INMEGEN-02-12-08/2021	EPI_ISL_1120885	10/02/21	North America / Mexico / Mexico City	Human	Male	47	unknown	Oropharyngeal swab
hCoV-19/Mexico/CMX-INMEGEN-02-12-09/2021	EPI_ISL_1120886	15/02/21	North America / Mexico / Mexico City	Human	Female	14	unknown	Oropharyngeal swab
hCoV-19/Mexico/CMX-INMEGEN-02-12-10/2021	EPI_ISL_1120887	15/02/21	North America / Mexico / Mexico City	Human	Female	67	unknown	Oropharyngeal swab
hCoV-19/Mexico/CMX-INMEGEN-02-12-11/2021	EPI_ISL_1120888	13/02/21	North America / Mexico / Mexico City	Human	Female	66	unknown	Oropharyngeal swab
hCoV-19/Mexico/CMX-INMEGEN-02-12-13/2021	EPI_ISL_1120889	13/02/21	North America / Mexico / Mexico City	Human	Female	21	unknown	Oropharyngeal swab
hCoV-19/Mexico/CMX-INMEGEN-02-12-15/2021	EPI_ISL_1120890	12/02/21	North America / Mexico / Mexico City	Human	Male	18	unknown	Oropharyngeal swab
hCoV-19/Mexico/CMX-INMEGEN-02-12-19/2021	EPI_ISL_1120891	13/02/21	North America / Mexico / Mexico City	Human	Female	66	unknown	Oropharyngeal swab
hCoV-19/Mexico/CMX-INMEGEN-02-12-21/2021	EPI_ISL_1120893	15/02/21	North America / Mexico / Mexico City	Human	Female	76	unknown	Oropharyngeal swab
hCoV-19/Mexico/CMX-INMEGEN-02-12-22/2021	EPI_ISL_1120894	15/02/21	North America / Mexico / Mexico City	Human	Male	45	unknown	Oropharyngeal swab

hCoV-19/Mexico/CMX-INMEGEN-02-12-23/2021	EPI_ISL_1120895	15/02/21	North America / Mexico / Mexico City	Human	Male	17	unknown	Oropharyngeal swab
hCoV-19/Mexico/CMX-INMEGEN-02-12-24/2021	EPI_ISL_1120896	15/02/21	North America / Mexico / Mexico City	Human	Female	28	unknown	Oropharyngeal swab
hCoV-19/Mexico/CMX-INMEGEN-02-12-14/2021	EPI_ISL_1133103	12/02/21	North America / Mexico / Mexico City	Human	Male	15	unknown	
hCoV-19/Mexico/CMX-INMEGEN-02-10-04/2021	EPI_ISL_1137460	05/02/21	North America / Mexico / Mexico City	Human	Female	22	unknown	Oropharyngeal swab
hCoV-19/Mexico/CMX-INMEGEN-02-10-14/2021	EPI_ISL_1137461	08/02/21	North America / Mexico / Mexico City	Human	Male	32	unknown	Oropharyngeal swab
hCoV-19/Mexico/CMX-INMEGEN-02-10-01/2021	EPI_ISL_1137462	06/02/21	North America / Mexico / Mexico City	Human	Male	38	unknown	Oropharyngeal swab
hCoV-19/Mexico/CMX-INMEGEN-02-10-02/2021	EPI_ISL_1137463	06/02/21	North America / Mexico / Mexico City	Human	Male	35	unknown	Oropharyngeal swab
hCoV-19/Mexico/CMX-INMEGEN-02-10-03/2021	EPI_ISL_1137464	07/02/21	North America / Mexico / Mexico City	Human	Female	27	unknown	Oropharyngeal swab
hCoV-19/Mexico/CMX-INMEGEN-02-10-06/2021	EPI_ISL_1137465	06/02/21	North America / Mexico / Mexico City	Human	Male	51	unknown	Oropharyngeal swab
hCoV-19/Mexico/CMX-INMEGEN-02-10-07/2021	EPI_ISL_1137466	07/02/21	North America / Mexico / Mexico City	Human	Male	18	unknown	Oropharyngeal swab
hCoV-19/Mexico/CMX-INMEGEN-02-10-09/2021	EPI_ISL_1137468	05/02/21	North America / Mexico / Mexico City	Human	Female	24	unknown	Oropharyngeal swab
hCoV-19/Mexico/CMX-INMEGEN-02-10-10/2021	EPI_ISL_1137469	05/02/21	North America / Mexico / Mexico City	Human	Female	27	unknown	Oropharyngeal swab
hCoV-19/Mexico/CMX-INMEGEN-02-10-11/2021	EPI_ISL_1137470	05/02/21	North America / Mexico / Mexico City	Human	Male	65	unknown	Oropharyngeal swab
hCoV-19/Mexico/CMX-INMEGEN-02-10-12/2021	EPI_ISL_1137471	08/02/21	North America / Mexico / Mexico City	Human	Male	44	unknown	Oropharyngeal swab
hCoV-19/Mexico/CMX-INMEGEN-02-10-13/2021	EPI_ISL_1137472	08/02/21	North America / Mexico / Mexico City	Human	Male	48	unknown	Oropharyngeal swab
hCoV-19/Mexico/CMX-INMEGEN-02-10-15/2021	EPI_ISL_1137473	08/02/21	North America / Mexico / Mexico City	Human	Female	62	unknown	Oropharyngeal swab
hCoV-19/Mexico/CMX-INMEGEN-02-10-16/2021	EPI_ISL_1137474	08/02/21	North America / Mexico / Mexico City	Human	Female	21	unknown	Oropharyngeal swab
hCoV-19/Mexico/QUE-InDRE_375/2021	EPI_ISL_1168453	24/01/21	North America / Mexico / Queretaro	Human	Male	36	Released	Oropharyngeal swab
hCoV-19/Mexico/QUE-InDRE_376/2021	EPI_ISL_1168454	24/01/21	North America / Mexico / Queretaro	Human	Male	34	Released	Oropharyngeal swab
hCoV-19/Mexico/QUE-InDRE_377/2021	EPI_ISL_1168455	24/01/21	North America / Mexico / Queretaro	Human	Female	32	Released	Oropharyngeal swab
hCoV-19/Mexico/QUE-InDRE_378/2021	EPI_ISL_1168456	24/01/21	North America / Mexico / Queretaro	Human	Female	37	Released	Oropharyngeal swab
hCoV-19/Mexico/QUE-InDRE_379/2021	EPI_ISL_1168457	24/01/21	North America / Mexico / Queretaro	Human	Male	73	Deceased	Oropharyngeal swab
hCoV-19/Mexico/QUE-InDRE_380/2021	EPI_ISL_1168458	25/01/21	North America / Mexico / Queretaro	Human	Female	52	Released	Oropharyngeal swab
hCoV-19/Mexico/QUE-InDRE_381/2021	EPI_ISL_1168459	25/01/21	North America / Mexico / Queretaro	Human	Male	44	Released	Oropharyngeal swab
hCoV-19/Mexico/QUE-InDRE_384/2021	EPI_ISL_1168462	25/01/21	North America / Mexico / Queretaro	Human	Male	41	Released	Oropharyngeal swab
hCoV-19/Mexico/QUE-InDRE_386/2021	EPI_ISL_1168464	25/01/21	North America / Mexico / Queretaro	Human	Female	3	Released	Oropharyngeal swab
hCoV-19/Mexico/QUE-InDRE_387/2021	EPI_ISL_1168465	26/01/21	North America / Mexico / Queretaro	Human	Female	35	Released	Oropharyngeal swab
hCoV-19/Mexico/NLE-InDRE_392/2021	EPI_ISL_1168470	19/01/21	North America / Mexico / Nuevo Leon	Human	Male	57	Released	Oropharyngeal swab
hCoV-19/Mexico/NLE-InDRE_395/2021	EPI_ISL_1168473	19/01/21	North America / Mexico / Nuevo Leon	Human	Male	22	Released	Oropharyngeal swab
hCoV-19/Mexico/NLE-InDRE_400/2021	EPI_ISL_1168478	21/01/21	North America / Mexico / Nuevo Leon	Human	Male	27	Released	Oropharyngeal swab
hCoV-19/Mexico/NLE-InDRE_403/2021	EPI_ISL_1168481	15/01/21	North America / Mexico / Nuevo Leon	Human	Female	59	Released	Oropharyngeal swab
hCoV-19/Mexico/COA-InDRE_410/2021	EPI_ISL_1168488	18/01/21	North America / Mexico / Coahuila	Human	Male	29	Released	Oropharyngeal swab
hCoV-19/Mexico/COA-InDRE_412/2021	EPI_ISL_1168490	18/01/21	North America / Mexico / Coahuila	Human	Male	31	Deceased	Oropharyngeal swab
hCoV-19/Mexico/VER-InDRE_415/2021	EPI_ISL_1168493	18/01/21	North America / Mexico / Veracruz	Human	Female	80	Live	Oropharyngeal swab
hCoV-19/Mexico/VER-InDRE_417/2021	EPI_ISL_1168495	20/01/21	North America / Mexico / Veracruz	Human	Male	45	Live	Oropharyngeal swab
hCoV-19/Mexico/VER-InDRE_418/2021	EPI_ISL_1168496	20/01/21	North America / Mexico / Veracruz	Human	Male	52	Hospitalized	Oropharyngeal swab
hCoV-19/Mexico/JAL-InDRE_421/2021	EPI_ISL_1168499	18/01/21	North America / Mexico / Jalisco	Human	Female	59	Released	Oropharyngeal swab
hCoV-19/Mexico/JAL-InDRE_426/2021	EPI_ISL_1168504	19/01/21	North America / Mexico / Jalisco	Human	Male	16	Released	Oropharyngeal swab
hCoV-19/Mexico/JAL-InDRE_431/2021	EPI_ISL_1168509	20/01/21	North America / Mexico / Jalisco	Human	Male	77	Hospitalized	Oropharyngeal swab
hCoV-19/Mexico/CHP-InDRE_443/2021	EPI_ISL_1168521	18/01/21	North America / Mexico / Chiapas	Human	Female	76	Live	Oropharyngeal swab
hCoV-19/Mexico/CHP-InDRE_444/2021	EPI_ISL_1168522	19/01/21	North America / Mexico / Chiapas	Human	Male	47	Deceased	Oropharyngeal swab
hCoV-19/Mexico/CHP-InDRE_445/2021	EPI_ISL_1168523	22/01/21	North America / Mexico / Chiapas	Human	Male	56	Deceased	Oropharyngeal swab
hCoV-19/Mexico/MOR-InDRE_448/2021	EPI_ISL_1168526	25/01/21	North America / Mexico / Morelos	Human	Female	38	Hospitalized	Oropharyngeal swab
hCoV-19/Mexico/HID-InDRE_461/2021	EPI_ISL_1168531	16/01/21	North America / Mexico / Hidalgo	Human	Female	31	Live	Oropharyngeal swab

hCoV-19/Mexico/MOR-InDRE_462/2021	EPI_ISL_1168532	15/01/21	North America / Mexico / Morelos	Human	Male	18	Released	Oropharyngeal swab
hCoV-19/Mexico/MOR-InDRE_465/2021	EPI_ISL_1168535	15/01/21	North America / Mexico / Morelos	Human	Female	54	Released	Oropharyngeal swab
hCoV-19/Mexico/MEX-InDRE_467/2021	EPI_ISL_1168537	16/01/21	North America / Mexico / State of Mexico	Human	Male	65	Hospitalized	Oropharyngeal swab
hCoV-19/Mexico/BCS-InDRE_468/2021	EPI_ISL_1168538	16/01/21	North America / Mexico / Baja California Sur	Human	Female	25	Released	Oropharyngeal swab
hCoV-19/Mexico/QUE-InDRE_473/2021	EPI_ISL_1168543	16/01/21	North America / Mexico / Queretaro	Human	Male	34	Released	Oropharyngeal swab
hCoV-19/Mexico/QUE-InDRE_474/2021	EPI_ISL_1168544	16/01/21	North America / Mexico / Queretaro	Human	Female	30	Released	Oropharyngeal swab
hCoV-19/Mexico/QUE-InDRE_475/2021	EPI_ISL_1168545	16/01/21	North America / Mexico / Queretaro	Human	Male	58	Released	Oropharyngeal swab
hCoV-19/Mexico/QUE-InDRE_479/2021	EPI_ISL_1168549	16/01/21	North America / Mexico / Queretaro	Human	Male	18	Released	Oropharyngeal swab
hCoV-19/Mexico/QUE-InDRE_480/2021	EPI_ISL_1168550	17/01/21	North America / Mexico / Queretaro	Human	Female	51	Deceased	Oropharyngeal swab
hCoV-19/Mexico/COA-InDRE_497/2021	EPI_ISL_1168567	18/01/21	North America / Mexico / Coahuila	Human	Female	35	Released	Oropharyngeal swab
hCoV-19/Mexico/YUC-InDRE_500/2021	EPI_ISL_1168570	18/01/21	North America / Mexico / Yucatan	Human	Female	31	Released	Oropharyngeal swab
hCoV-19/Mexico/YUC-InDRE_501/2021	EPI_ISL_1168571	18/01/21	North America / Mexico / Yucatan	Human	Female	41	Released	Oropharyngeal swab
hCoV-19/Mexico/YUC-InDRE_503/2021	EPI_ISL_1168573	18/01/21	North America / Mexico / Yucatan	Human	Male	46	Released	Oropharyngeal swab
hCoV-19/Mexico/COA-InDRE_511/2021	EPI_ISL_1168581	15/01/21	North America / Mexico / Coahuila	Human	Male	47	Released	Oropharyngeal swab
hCoV-19/Mexico/HID-InDRE_520/2021	EPI_ISL_1168590	21/01/21	North America / Mexico / Hidalgo	Human	Female	75	Live	Oropharyngeal swab
hCoV-19/Mexico/HID-InDRE_521/2021	EPI_ISL_1168591	23/01/21	North America / Mexico / Hidalgo	Human	Female	32	Live	Oropharyngeal swab
hCoV-19/Mexico/MOR-InDRE_523/2021	EPI_ISL_1168593	19/01/21	North America / Mexico / Morelos	Human	Female	58	Hospitalized	Oropharyngeal swab
hCoV-19/Mexico/MOR-InDRE_524/2021	EPI_ISL_1168594	19/01/21	North America / Mexico / Morelos	Human	Male	39	Released	Oropharyngeal swab
hCoV-19/Mexico/MOR-InDRE_525/2021	EPI_ISL_1168595	20/01/21	North America / Mexico / Morelos	Human	Male	46	Released	Oropharyngeal swab
hCoV-19/Mexico/MOR-InDRE_526/2021	EPI_ISL_1168596	21/01/21	North America / Mexico / Morelos	Human	Male	44	Released	Oropharyngeal swab
hCoV-19/Mexico/MOR-InDRE_528/2021	EPI_ISL_1168598	20/01/21	North America / Mexico / Morelos	Human	Female	49	Released	Oropharyngeal swab
hCoV-19/Mexico/MOR-InDRE_529/2021	EPI_ISL_1168599	21/01/21	North America / Mexico / Morelos	Human	Female	40	Released	Oropharyngeal swab
hCoV-19/Mexico/MOR-InDRE_530/2021	EPI_ISL_1168600	21/01/21	North America / Mexico / Morelos	Human	Male	87	Released	Oropharyngeal swab
hCoV-19/Mexico/MOR-InDRE_532/2021	EPI_ISL_1168602	22/01/21	North America / Mexico / Morelos	Human	Male	33	Released	Oropharyngeal swab
hCoV-19/Mexico/MOR-InDRE_533/2021	EPI_ISL_1168603	21/01/21	North America / Mexico / Morelos	Human	Female	77	Released	Oropharyngeal swab
hCoV-19/Mexico/MOR-InDRE_534/2021	EPI_ISL_1168604	25/01/21	North America / Mexico / Morelos	Human	Female	45	Released	Oropharyngeal swab
hCoV-19/Mexico/OAX-InDRE_536/2021	EPI_ISL_1168606	21/01/21	North America / Mexico / Oaxaca	Human	Male	32	unknown	Oropharyngeal swab
hCoV-19/Mexico/OAX-InDRE_537/2021	EPI_ISL_1168607	20/01/21	North America / Mexico / Oaxaca	Human	Male	59	Deceased	Oropharyngeal swab
hCoV-19/Mexico/MEX-InDRE_542/2021	EPI_ISL_1168612	22/01/21	North America / Mexico / State of Mexico	Human	Male	44	Live	Oropharyngeal swab
hCoV-19/Mexico/CAM-InDRE_544/2021	EPI_ISL_1168614	17/01/21	North America / Mexico / Campeche	Human	Male	59	Deceased	Oropharyngeal swab
hCoV-19/Mexico/COL-InDRE_545/2021	EPI_ISL_1168615	15/01/21	North America / Mexico / Colima	Human	Female	72	Deceased	Oropharyngeal swab
hCoV-19/Mexico/JAL-InDRE_552/2021	EPI_ISL_1168622	15/01/21	North America / Mexico / Jalisco	Human	Female	70	Released	Oropharyngeal swab
hCoV-19/Mexico/QUE-InDRE_554/2021	EPI_ISL_1168624	20/01/21	North America / Mexico / Queretaro	Human	Male	36	Released	Oropharyngeal swab
hCoV-19/Mexico/QUE-InDRE_556/2021	EPI_ISL_1168626	22/01/21	North America / Mexico / Queretaro	Human	Female	33	Released	Oropharyngeal swab
hCoV-19/Mexico/QUE-InDRE_557/2021	EPI_ISL_1168627	22/01/21	North America / Mexico / Queretaro	Human	Female	26	Released	Oropharyngeal swab
hCoV-19/Mexico/QUE-InDRE_558/2021	EPI_ISL_1168628	22/01/21	North America / Mexico / Queretaro	Human	Male	37	Released	Oropharyngeal swab
hCoV-19/Mexico/QUE-InDRE_561/2021	EPI_ISL_1168631	22/01/21	North America / Mexico / Queretaro	Human	Male	22	Released	Oropharyngeal swab
hCoV-19/Mexico/QUE-InDRE_562/2021	EPI_ISL_1168632	22/01/21	North America / Mexico / Queretaro	Human	Male	26	Released	Oropharyngeal swab
hCoV-19/Mexico/QUE-InDRE_563/2021	EPI_ISL_1168633	22/01/21	North America / Mexico / Queretaro	Human	Male	39	Live	Oropharyngeal swab
hCoV-19/Mexico/CMX-INMEGEN-03-01-01/2021	EPI_ISL_1168634	15/02/21	North America / Mexico / Mexico City	Human	Female	49	unknown	Oropharyngeal swab
hCoV-19/Mexico/CMX-INMEGEN-03-01-02/2021	EPI_ISL_1168635	15/02/21	North America / Mexico / Mexico City	Human	Male	64	unknown	Oropharyngeal swab
hCoV-19/Mexico/CMX-INMEGEN-03-01-03/2021	EPI_ISL_1168636	15/02/21	North America / Mexico / Mexico City	Human	Female	59	unknown	Oropharyngeal swab
hCoV-19/Mexico/CMX-INMEGEN-03-01-04/2021	EPI_ISL_1168637	12/02/21	North America / Mexico / Mexico City	Human	Male	57	unknown	Oropharyngeal swab
hCoV-19/Mexico/CMX-INMEGEN-03-01-05/2021	EPI_ISL_1168638	15/02/21	North America / Mexico / Mexico City	Human	Male	66	unknown	Oropharyngeal swab

hCoV-19/Mexico/CMX-INMEGEN-03-01-06/2021	EPI_ISL_1168639	15/02/21	North America / Mexico / Mexico City	Human	Male	66	unknown	Oropharyngeal swab
hCoV-19/Mexico/CMX-INMEGEN-03-01-07/2021	EPI_ISL_1168640	15/02/21	North America / Mexico / Mexico City	Human	Female	52	unknown	Oropharyngeal swab
hCoV-19/Mexico/CMX-INMEGEN-03-01-08/2021	EPI_ISL_1168641	15/02/21	North America / Mexico / Mexico City	Human	Female	60	unknown	Oropharyngeal swab
hCoV-19/Mexico/CMX-INMEGEN-03-01-09/2021	EPI_ISL_1168642	17/02/21	North America / Mexico / Mexico City	Human	Male	22	unknown	Oropharyngeal swab
hCoV-19/Mexico/CMX-INMEGEN-03-01-10/2021	EPI_ISL_1168643	17/02/21	North America / Mexico / Mexico City	Human	Female	46	unknown	Oropharyngeal swab
hCoV-19/Mexico/CMX-INMEGEN-03-01-11/2021	EPI_ISL_1168644	17/02/21	North America / Mexico / Mexico City	Human	Male	17	unknown	Oropharyngeal swab
hCoV-19/Mexico/CMX-INMEGEN-03-01-12/2021	EPI_ISL_1168645	18/02/21	North America / Mexico / Mexico City	Human	Female	49	unknown	Oropharyngeal swab
hCoV-19/Mexico/CMX-INMEGEN-03-01-17/2021	EPI_ISL_1168646	18/02/21	North America / Mexico / Mexico City	Human	Female	69	unknown	Oropharyngeal swab
hCoV-19/Mexico/CMX-INMEGEN-03-01-18/2021	EPI_ISL_1168647	18/02/21	North America / Mexico / Mexico City	Human	Male	13	unknown	Oropharyngeal swab
hCoV-19/Mexico/CMX-INMEGEN-03-01-19/2021	EPI_ISL_1168648	19/02/21	North America / Mexico / Mexico City	Human	Male	30	unknown	Oropharyngeal swab
hCoV-19/Mexico/CMX-INMEGEN-03-01-20/2021	EPI_ISL_1168649	19/02/21	North America / Mexico / Mexico City	Human	Female	74	unknown	Oropharyngeal swab
hCoV-19/Mexico/CMX-INMEGEN-03-01-21/2021	EPI_ISL_1168650	18/02/21	North America / Mexico / Mexico City	Human	Male	68	unknown	Oropharyngeal swab
hCoV-19/Mexico/CMX-INMEGEN-03-01-22/2021	EPI_ISL_1168651	18/02/21	North America / Mexico / Mexico City	Human	Male	53	unknown	Oropharyngeal swab
hCoV-19/Mexico/CMX-INMEGEN-03-01-23/2021	EPI_ISL_1168652	18/02/21	North America / Mexico / Mexico City	Human	Female	32	unknown	Oropharyngeal swab
hCoV-19/Mexico/CMX-INMEGEN-03-02-01/2021	EPI_ISL_1181700	19/02/21	North America / Mexico / Mexico City	Human	Female	49	unknown	Oropharyngeal swab
hCoV-19/Mexico/CMX-INMEGEN-03-02-02/2021	EPI_ISL_1181701	18/02/21	North America / Mexico / Mexico City	Human	Male	54	unknown	Oropharyngeal swab
hCoV-19/Mexico/CMX-INMEGEN-03-02-04/2021	EPI_ISL_1181702	19/02/21	North America / Mexico / Mexico City	Human	Female	49	unknown	Oropharyngeal swab
hCoV-19/Mexico/CMX-INMEGEN-03-02-05/2021	EPI_ISL_1181703	18/02/21	North America / Mexico / Mexico City	Human	Female	42	unknown	Oropharyngeal swab
hCoV-19/Mexico/CMX-INMEGEN-03-02-06/2021	EPI_ISL_1181704	18/02/21	North America / Mexico / Mexico City	Human	Female	41	unknown	Oropharyngeal swab
hCoV-19/Mexico/CMX-INMEGEN-03-02-07/2021	EPI_ISL_1181705	19/02/21	North America / Mexico / Mexico City	Human	Male	33	unknown	Oropharyngeal swab
hCoV-19/Mexico/CMX-INMEGEN-03-02-10/2021	EPI_ISL_1181707	16/02/21	North America / Mexico / Mexico City	Human	Male	38	unknown	Oropharyngeal swab
hCoV-19/Mexico/CMX-INMEGEN-03-02-11/2021	EPI_ISL_1181708	17/02/21	North America / Mexico / Mexico City	Human	Female	57	unknown	Oropharyngeal swab
hCoV-19/Mexico/CMX-INMEGEN-03-02-12/2021	EPI_ISL_1181709	17/02/21	North America / Mexico / Mexico City	Human	Male	58	unknown	Oropharyngeal swab
hCoV-19/Mexico/CMX-INMEGEN-03-02-13/2021	EPI_ISL_1181710	19/02/21	North America / Mexico / Mexico City	Human	Female	29	unknown	Oropharyngeal swab
hCoV-19/Mexico/CMX-INMEGEN-03-02-14/2021	EPI_ISL_1181711	20/02/21	North America / Mexico / Mexico City	Human	Female	20	unknown	Oropharyngeal swab
hCoV-19/Mexico/CMX-INMEGEN-03-02-15/2021	EPI_ISL_1181712	20/02/21	North America / Mexico / Mexico City	Human	Male	56	unknown	Oropharyngeal swab
hCoV-19/Mexico/CMX-INMEGEN-03-02-16/2021	EPI_ISL_1181713	20/02/21	North America / Mexico / Mexico City	Human	Male	42	unknown	Oropharyngeal swab
hCoV-19/Mexico/CMX-INMEGEN-03-02-17/2021	EPI_ISL_1181714	21/02/21	North America / Mexico / Mexico City	Human	Female	69	unknown	Oropharyngeal swab
hCoV-19/Mexico/CMX-INMEGEN-03-02-18/2021	EPI_ISL_1181715	22/02/21	North America / Mexico / Mexico City	Human	Male	52	unknown	Oropharyngeal swab
hCoV-19/Mexico/CMX-INMEGEN-03-02-19/2021	EPI_ISL_1181716	19/02/21	North America / Mexico / Mexico City	Human	Female	40	unknown	Oropharyngeal swab
hCoV-19/Mexico/CMX-INMEGEN-03-02-20/2021	EPI_ISL_1181717	22/02/21	North America / Mexico / Mexico City	Human	Female	27	unknown	Oropharyngeal swab
hCoV-19/Mexico/CMX-INMEGEN-03-02-21/2021	EPI_ISL_1181718	22/02/21	North America / Mexico / Mexico City	Human	Female	21	unknown	Oropharyngeal swab
hCoV-19/Mexico/CMX-INMEGEN-03-02-22/2021	EPI_ISL_1181719	20/02/21	North America / Mexico / Mexico City	Human	Male	21	unknown	Oropharyngeal swab
hCoV-19/Mexico/CMX-INMEGEN-03-02-23/2021	EPI_ISL_1193558	22/02/21	North America / Mexico / Mexico City	Human	Male	56	unknown	
hCoV-19/Mexico/CMX-INMEGEN-03-04-01/2021	EPI_ISL_1205203	23/02/21	North America / Mexico / Mexico City	Human	Male	81	unknown	Oropharyngeal swab
hCoV-19/Mexico/CMX-INMEGEN-03-04-02/2021	EPI_ISL_1205204	24/02/21	North America / Mexico / Mexico City	Human	Female	83	unknown	Oropharyngeal swab
hCoV-19/Mexico/CMX-INMEGEN-03-04-03/2021	EPI_ISL_1205205	23/02/21	North America / Mexico / Mexico City	Human	Male	19	unknown	Oropharyngeal swab
hCoV-19/Mexico/CMX-INMEGEN-03-04-04/2021	EPI_ISL_1205206	24/02/21	North America / Mexico / Mexico City	Human	Male	40	unknown	Oropharyngeal swab
hCoV-19/Mexico/CMX-INMEGEN-03-04-05/2021	EPI_ISL_1205207	23/02/21	North America / Mexico / Mexico City	Human	Female	62	unknown	Oropharyngeal swab
hCoV-19/Mexico/CMX-INMEGEN-03-04-06/2021	EPI_ISL_1205208	23/02/21	North America / Mexico / Mexico City	Human	Male	65	unknown	Oropharyngeal swab
hCoV-19/Mexico/CMX-INMEGEN-03-04-07/2021	EPI_ISL_1205209	15/02/21	North America / Mexico / Mexico City	Human	Female	59	unknown	Oropharyngeal swab
hCoV-19/Mexico/CMX-INMEGEN-03-04-08/2021	EPI_ISL_1205210	26/02/21	North America / Mexico / Mexico City	Human	Female	24	unknown	Oropharyngeal swab
hCoV-19/Mexico/CMX-INMEGEN-03-04-09/2021	EPI_ISL_1205211	26/02/21	North America / Mexico / Mexico City	Human	Male	25	unknown	Oropharyngeal swab
hCoV-19/Mexico/CMX-INMEGEN-03-04-10/2021	EPI_ISL_1205212	26/02/21	North America / Mexico / Mexico City	Human	Female	38	unknown	Oropharyngeal swab

hCoV-19/Mexico/CMX-INMEGEN-03-04-11/2021	EPI_ISL_1205213	26/02/21	North America / Mexico / Mexico City	Human	female	41	unknown	Oropharyngeal swab
hCoV-19/Mexico/CMX-INMEGEN-03-04-13/2021	EPI_ISL_1205215	27/02/21	North America / Mexico / Mexico City	Human	female	38	unknown	Oropharyngeal swab
hCoV-19/Mexico/CMX-INMEGEN-03-04-14/2021	EPI_ISL_1205216	27/02/21	North America / Mexico / Mexico City	Human	male	68	unknown	Oropharyngeal swab
hCoV-19/Mexico/CMX-INMEGEN-03-04-15/2021	EPI_ISL_1205217	27/02/21	North America / Mexico / Mexico City	Human	male	11	unknown	Oropharyngeal swab
hCoV-19/Mexico/CMX-InDRE_451/2021	EPI_ISL_1219711	22/01/21	North America / Mexico / Mexico City	Human	Male	52	Hospitalized	Oropharyngeal swab
hCoV-19/Mexico/CMX-InDRE_452/2021	EPI_ISL_1219712	22/01/21	North America / Mexico / Mexico City	Human	Male	86	Released	Oropharyngeal swab
hCoV-19/Mexico/CMX-INMEGEN-03-05-43/2021	EPI_ISL_1262631	26/02/21	North America / Mexico / Mexico City	Human	Male	44	unknown	Oropharyngeal swab
hCoV-19/Mexico/CMX-INMEGEN-03-05-85/2021	EPI_ISL_1262632	02/03/21	North America / Mexico / Mexico City	Human	Female	36	unknown	Oropharyngeal swab
hCoV-19/Mexico/CMX-INMEGEN-03-05-54/2021	EPI_ISL_1262633	01/03/21	North America / Mexico / Mexico City	Human	Male	32	unknown	Oropharyngeal swab
hCoV-19/Mexico/CMX-INMEGEN-03-05-82/2021	EPI_ISL_1262634	03/03/21	North America / Mexico / Mexico City	Human	Female	45	unknown	Oropharyngeal swab
hCoV-19/Mexico/CMX-INMEGEN-03-05-32/2021	EPI_ISL_1262635	04/03/21	North America / Mexico / Mexico City	Human	Female	13	unknown	Oropharyngeal swab
hCoV-19/Mexico/CMX-INMEGEN-03-05-75/2021	EPI_ISL_1262636	02/03/21	North America / Mexico / Mexico City	Human	Female	21	unknown	Oropharyngeal swab
hCoV-19/Mexico/CMX-INMEGEN-03-05-37/2021	EPI_ISL_1262638	26/02/21	North America / Mexico / Mexico City	Human	Male	49	unknown	Oropharyngeal swab
hCoV-19/Mexico/CMX-INMEGEN-03-05-88/2021	EPI_ISL_1262639	02/03/21	North America / Mexico / Mexico City	Human	Female	8	unknown	Oropharyngeal swab
hCoV-19/Mexico/CMX-INMEGEN-03-05-60/2021	EPI_ISL_1262640	01/03/21	North America / Mexico / Mexico City	Human	Male	42	unknown	Oropharyngeal swab
hCoV-19/Mexico/CMX-INMEGEN-03-05-76/2021	EPI_ISL_1262641	02/03/21	North America / Mexico / Mexico City	Human	Female	59	unknown	Oropharyngeal swab
hCoV-19/Mexico/CMX-INMEGEN-03-05-11/2021	EPI_ISL_1262642	24/02/21	North America / Mexico / Mexico City	Human	Male	30	unknown	Oropharyngeal swab
hCoV-19/Mexico/CMX-INMEGEN-03-05-34/2021	EPI_ISL_1262643	05/03/21	North America / Mexico / Mexico City	Human	Female	11	unknown	Oropharyngeal swab
hCoV-19/Mexico/CMX-INMEGEN-03-05-57/2021	EPI_ISL_1262644	05/03/21	North America / Mexico / Mexico City	Human	Male	29	unknown	Oropharyngeal swab
hCoV-19/Mexico/CMX-INMEGEN-03-05-29/2021	EPI_ISL_1262645	04/03/21	North America / Mexico / Mexico City	Human	Female	15	unknown	Oropharyngeal swab
hCoV-19/Mexico/CMX-INMEGEN-03-05-62/2021	EPI_ISL_1262646	04/03/21	North America / Mexico / Mexico City	Human	Female	13	unknown	Oropharyngeal swab
hCoV-19/Mexico/CMX-INMEGEN-03-05-73/2021	EPI_ISL_1262647	04/03/21	North America / Mexico / Mexico City	Human	Male	11	unknown	Oropharyngeal swab
hCoV-19/Mexico/CMX-INMEGEN-03-05-92/2021	EPI_ISL_1262648	04/03/21	North America / Mexico / Mexico City	Human	Female	24	unknown	Oropharyngeal swab
hCoV-19/Mexico/CMX-INMEGEN-03-05-06/2021	EPI_ISL_1262649	02/03/21	North America / Mexico / Mexico City	Human	Female	43	unknown	Oropharyngeal swab
hCoV-19/Mexico/CMX-INMEGEN-03-05-24/2021	EPI_ISL_1262650	02/03/21	North America / Mexico / Mexico City	Human	Female	50	unknown	Oropharyngeal swab
hCoV-19/Mexico/CMX-INMEGEN-03-05-31/2021	EPI_ISL_1262651	02/03/21	North America / Mexico / Mexico City	Human	Male	32	unknown	Oropharyngeal swab
hCoV-19/Mexico/CMX-INMEGEN-03-05-58/2021	EPI_ISL_1262652	02/03/21	North America / Mexico / Mexico City	Human	Male	42	unknown	Oropharyngeal swab
hCoV-19/Mexico/CMX-INMEGEN-03-05-61/2021	EPI_ISL_1262653	02/03/21	North America / Mexico / Mexico City	Human	Male	69	unknown	Oropharyngeal swab
hCoV-19/Mexico/CMX-INMEGEN-03-05-77/2021	EPI_ISL_1262654	02/03/21	North America / Mexico / Mexico City	Human	Male	44	unknown	Oropharyngeal swab
hCoV-19/Mexico/CMX-INMEGEN-03-05-78/2021	EPI_ISL_1262655	02/03/21	North America / Mexico / Mexico City	Human	Male	26	unknown	Oropharyngeal swab
hCoV-19/Mexico/CMX-INMEGEN-03-05-79/2021	EPI_ISL_1262656	02/03/21	North America / Mexico / Mexico City	Human	Male	36	unknown	Oropharyngeal swab
hCoV-19/Mexico/CMX-INMEGEN-03-05-80/2021	EPI_ISL_1262657	02/03/21	North America / Mexico / Mexico City	Human	Male	37	unknown	Oropharyngeal swab
hCoV-19/Mexico/CMX-INMEGEN-03-05-81/2021	EPI_ISL_1262658	02/03/21	North America / Mexico / Mexico City	Human	Male	44	unknown	Oropharyngeal swab
hCoV-19/Mexico/CMX-INMEGEN-03-05-83/2021	EPI_ISL_1262659	02/03/21	North America / Mexico / Mexico City	Human	Male	26	unknown	Oropharyngeal swab
hCoV-19/Mexico/CMX-INMEGEN-03-05-84/2021	EPI_ISL_1262660	02/03/21	North America / Mexico / Mexico City	Human	Female	22	unknown	Oropharyngeal swab
hCoV-19/Mexico/CMX-INMEGEN-03-05-86/2021	EPI_ISL_1262661	02/03/21	North America / Mexico / Mexico City	Human	Female	43	unknown	Oropharyngeal swab
hCoV-19/Mexico/CMX-INMEGEN-03-05-87/2021	EPI_ISL_1262662	02/03/21	North America / Mexico / Mexico City	Human	Female	36	unknown	Oropharyngeal swab
hCoV-19/Mexico/CMX-INMEGEN-03-05-20/2021	EPI_ISL_1262663	01/03/21	North America / Mexico / Mexico City	Human	Female	37	unknown	Oropharyngeal swab
hCoV-19/Mexico/CMX-INMEGEN-03-05-23/2021	EPI_ISL_1262664	01/03/21	North America / Mexico / Mexico City	Human	Female	62	unknown	Oropharyngeal swab
hCoV-19/Mexico/CMX-INMEGEN-03-05-25/2021	EPI_ISL_1262665	01/03/21	North America / Mexico / Mexico City	Human	Female	30	unknown	Oropharyngeal swab
hCoV-19/Mexico/CMX-INMEGEN-03-05-42/2021	EPI_ISL_1262666	01/03/21	North America / Mexico / Mexico City	Human	Female	74	unknown	Oropharyngeal swab
hCoV-19/Mexico/CMX-INMEGEN-03-05-44/2021	EPI_ISL_1262667	01/03/21	North America / Mexico / Mexico City	Human	Female	71	unknown	Oropharyngeal swab
hCoV-19/Mexico/CMX-INMEGEN-03-05-55/2021	EPI_ISL_1262669	01/03/21	North America / Mexico / Mexico City	Human	Female	41	unknown	Oropharyngeal swab
hCoV-19/Mexico/CMX-INMEGEN-03-05-59/2021	EPI_ISL_1262670	01/03/21	North America / Mexico / Mexico City	Human	Female	51	unknown	Oropharyngeal swab

hCoV-19/Mexico/CMX-INMEGEN-03-05-63/2021	EPI_ISL_1262671	01/03/21	North America / Mexico / Mexico City	Human	Male	65	unknown	Oropharyngeal swab
hCoV-19/Mexico/CMX-INMEGEN-03-05-64/2021	EPI_ISL_1262672	01/03/21	North America / Mexico / Mexico City	Human	Female	49	unknown	Oropharyngeal swab
hCoV-19/Mexico/CMX-INMEGEN-03-05-65/2021	EPI_ISL_1262673	01/03/21	North America / Mexico / Mexico City	Human	Female	26	unknown	Oropharyngeal swab
hCoV-19/Mexico/CMX-INMEGEN-03-05-66/2021	EPI_ISL_1262674	01/03/21	North America / Mexico / Mexico City	Human	Female	18	unknown	Oropharyngeal swab
hCoV-19/Mexico/CMX-INMEGEN-03-05-67/2021	EPI_ISL_1262675	01/03/21	North America / Mexico / Mexico City	Human	Female	58	unknown	Oropharyngeal swab
hCoV-19/Mexico/CMX-INMEGEN-03-05-68/2021	EPI_ISL_1262676	01/03/21	North America / Mexico / Mexico City	Human	Male	70	unknown	Oropharyngeal swab
hCoV-19/Mexico/CMX-INMEGEN-03-05-71/2021	EPI_ISL_1262677	01/03/21	North America / Mexico / Mexico City	Human	Female	29	unknown	Oropharyngeal swab
hCoV-19/Mexico/CMX-INMEGEN-03-05-91/2021	EPI_ISL_1262679	01/03/21	North America / Mexico / Mexico City	Human	Male	60	unknown	Oropharyngeal swab
hCoV-19/Mexico/CMX-INMEGEN-03-05-69/2021	EPI_ISL_1262680	28/02/21	North America / Mexico / Mexico City	Human	Female	55	unknown	Oropharyngeal swab
hCoV-19/Mexico/CMX-INMEGEN-03-05-72/2021	EPI_ISL_1262681	28/02/21	North America / Mexico / Mexico City	Human	Male	28	unknown	Oropharyngeal swab
hCoV-19/Mexico/CMX-INMEGEN-03-05-39/2021	EPI_ISL_1262682	27/02/21	North America / Mexico / Mexico City	Human	Male	44	unknown	Oropharyngeal swab
hCoV-19/Mexico/CMX-INMEGEN-03-05-49/2021	EPI_ISL_1262683	27/02/21	North America / Mexico / Mexico City	Human	Male	47	unknown	Oropharyngeal swab
hCoV-19/Mexico/CMX-INMEGEN-03-05-51/2021	EPI_ISL_1262684	27/02/21	North America / Mexico / Mexico City	Human	Female	43	unknown	Oropharyngeal swab
hCoV-19/Mexico/CMX-INMEGEN-03-05-35/2021	EPI_ISL_1262686	26/02/21	North America / Mexico / Mexico City	Human	Male	73	unknown	Oropharyngeal swab
hCoV-19/Mexico/CMX-INMEGEN-03-05-38/2021	EPI_ISL_1262687	26/02/21	North America / Mexico / Mexico City	Human	Male	23	unknown	Oropharyngeal swab
hCoV-19/Mexico/CMX-INMEGEN-03-05-41/2021	EPI_ISL_1262688	26/02/21	North America / Mexico / Mexico City	Human	Male	56	unknown	Oropharyngeal swab
hCoV-19/Mexico/CMX-INMEGEN-03-05-47/2021	EPI_ISL_1262689	26/02/21	North America / Mexico / Mexico City	Human	Male	50	unknown	Oropharyngeal swab
hCoV-19/Mexico/CMX-INMEGEN-03-05-48/2021	EPI_ISL_1262690	26/02/21	North America / Mexico / Mexico City	Human	Female	30	unknown	Oropharyngeal swab
hCoV-19/Mexico/CMX-INMEGEN-03-05-74/2021	EPI_ISL_1262691	26/02/21	North America / Mexico / Mexico City	Human	Male	53	unknown	Oropharyngeal swab
hCoV-19/Mexico/CMX-INMEGEN-03-05-89/2021	EPI_ISL_1262692	26/02/21	North America / Mexico / Mexico City	Human	Male	56	unknown	Oropharyngeal swab
hCoV-19/Mexico/CMX-INMEGEN-03-05-17/2021	EPI_ISL_1262693	25/02/21	North America / Mexico / Mexico City	Human	Male	62	unknown	Oropharyngeal swab
hCoV-19/Mexico/CMX-INMEGEN-03-05-22/2021	EPI_ISL_1262694	25/02/21	North America / Mexico / Mexico City	Human	Male	20	unknown	Oropharyngeal swab
hCoV-19/Mexico/CMX-INMEGEN-03-05-26/2021	EPI_ISL_1262695	25/02/21	North America / Mexico / Mexico City	Human	Male	65	unknown	Oropharyngeal swab
hCoV-19/Mexico/CMX-INMEGEN-03-05-27/2021	EPI_ISL_1262696	25/02/21	North America / Mexico / Mexico City	Human	Female	39	unknown	Oropharyngeal swab
hCoV-19/Mexico/CMX-INMEGEN-03-05-28/2021	EPI_ISL_1262697	25/02/21	North America / Mexico / Mexico City	Human	Male	41	unknown	Oropharyngeal swab
hCoV-19/Mexico/CMX-INMEGEN-03-05-30/2021	EPI_ISL_1262698	25/02/21	North America / Mexico / Mexico City	Human	Male	19	unknown	Oropharyngeal swab
hCoV-19/Mexico/CMX-INMEGEN-03-05-33/2021	EPI_ISL_1262699	25/02/21	North America / Mexico / Mexico City	Human	Male	49	unknown	Oropharyngeal swab
hCoV-19/Mexico/CMX-INMEGEN-03-05-36/2021	EPI_ISL_1262700	25/02/21	North America / Mexico / Mexico City	Human	Female	84	unknown	Oropharyngeal swab
hCoV-19/Mexico/CMX-INMEGEN-03-05-40/2021	EPI_ISL_1262701	25/02/21	North America / Mexico / Mexico City	Human	Female	46	unknown	Oropharyngeal swab
hCoV-19/Mexico/CMX-INMEGEN-03-05-01/2021	EPI_ISL_1262702	24/02/21	North America / Mexico / Mexico City	Human	Female	36	unknown	Oropharyngeal swab
hCoV-19/Mexico/CMX-INMEGEN-03-05-02/2021	EPI_ISL_1262703	24/02/21	North America / Mexico / Mexico City	Human	Female	15	unknown	Oropharyngeal swab
hCoV-19/Mexico/CMX-INMEGEN-03-05-03/2021	EPI_ISL_1262704	24/02/21	North America / Mexico / Mexico City	Human	Male	58	unknown	Oropharyngeal swab
hCoV-19/Mexico/CMX-INMEGEN-03-05-04/2021	EPI_ISL_1262705	24/02/21	North America / Mexico / Mexico City	Human	Male	28	unknown	Oropharyngeal swab
hCoV-19/Mexico/CMX-INMEGEN-03-05-05/2021	EPI_ISL_1262706	24/02/21	North America / Mexico / Mexico City	Human	Male	45	unknown	Oropharyngeal swab
hCoV-19/Mexico/CMX-INMEGEN-03-05-07/2021	EPI_ISL_1262707	24/02/21	North America / Mexico / Mexico City	Human	Male	50	unknown	Oropharyngeal swab
hCoV-19/Mexico/CMX-INMEGEN-03-05-08/2021	EPI_ISL_1262708	24/02/21	North America / Mexico / Mexico City	Human	Male	64	unknown	Oropharyngeal swab
hCoV-19/Mexico/CMX-INMEGEN-03-05-09/2021	EPI_ISL_1262709	24/02/21	North America / Mexico / Mexico City	Human	Male	9	unknown	Oropharyngeal swab
hCoV-19/Mexico/CMX-INMEGEN-03-05-10/2021	EPI_ISL_1262710	24/02/21	North America / Mexico / Mexico City	Human	Female	53	unknown	Oropharyngeal swab
hCoV-19/Mexico/CMX-INMEGEN-03-05-12/2021	EPI_ISL_1262711	24/02/21	North America / Mexico / Mexico City	Human	Female	58	unknown	Oropharyngeal swab
hCoV-19/Mexico/CMX-INMEGEN-03-05-13/2021	EPI_ISL_1262712	24/02/21	North America / Mexico / Mexico City	Human	Female	64	unknown	Oropharyngeal swab
hCoV-19/Mexico/CMX-INMEGEN-03-05-14/2021	EPI_ISL_1262713	24/02/21	North America / Mexico / Mexico City	Human	Male	34	unknown	Oropharyngeal swab
hCoV-19/Mexico/CMX-INMEGEN-03-05-15/2021	EPI_ISL_1262714	24/02/21	North America / Mexico / Mexico City	Human	Male	31	unknown	Oropharyngeal swab
hCoV-19/Mexico/CMX-INMEGEN-03-05-18/2021	EPI_ISL_1262715	24/02/21	North America / Mexico / Mexico City	Human	Male	35	unknown	Oropharyngeal swab
hCoV-19/Mexico/CMX-INMEGEN-03-05-19/2021	EPI_ISL_1262716	24/02/21	North America / Mexico / Mexico City	Human	Male	32	unknown	Oropharyngeal swab

hCoV-19/Mexico/CMX-INMEGEN-03-05-21/2021	ΞPI_ISL_1262717	11/02/21	North America / Mexico / Mexico City	Human	Male	7	unknown	Oropharyngeal swab
hCoV-19/Mexico/CMX-INMEGEN-03-06-12/2021	ΞPI_ISL_1265902	27/02/21	North America / Mexico / Mexico City	Human	Male	55	unknown	Oropharyngeal swab
hCoV-19/Mexico/CMX-INMEGEN-03-06-13/2021	ΞPI_ISL_1265903	26/02/21	North America / Mexico / Mexico City	Human	Female	47	unknown	Oropharyngeal swab
hCoV-19/Mexico/CMX-INMEGEN-03-06-14/2021	ΞPI_ISL_1265904	25/02/21	North America / Mexico / Mexico City	Human	Male	12	unknown	Oropharyngeal swab
hCoV-19/Mexico/CMX-INMEGEN-03-06-16/2021	ΞPI_ISL_1265906	26/02/21	North America / Mexico / Mexico City	Human	Male	41	unknown	Oropharyngeal swab
hCoV-19/Mexico/CMX-INMEGEN-03-06-17/2021	ΞPI_ISL_1265907	25/02/21	North America / Mexico / Mexico City	Human	Female	58	unknown	Oropharyngeal swab
hCoV-19/Mexico/CMX-INMEGEN-03-06-18/2021	ΞPI_ISL_1265908	25/02/21	North America / Mexico / Mexico City	Human	Female	35	unknown	Oropharyngeal swab
hCoV-19/Mexico/QUE-INER-IMSS-00112/2021	ΞPI_ISL_1279263	19/02/21	North America / Mexico / Queretaro	Human	Male	55	Ambulatory	Nasopharyngeal and oropharyngeal swab
hCoV-19/Mexico/PUE-INER-IMSS-00120/2021	ΞPI_ISL_1279268	20/02/21	North America / Mexico / Puebla	Human	Male	64	Hospitalized	Nasopharyngeal and oropharyngeal swab
hCoV-19/Mexico/SLP-INER-IMSS-00170/2021	ΞPI_ISL_127926ς	20/02/21	North America / Mexico / San Luis Potosi	Human	Male	48	Hospitalized	Nasopharyngeal and oropharyngeal swab
hCoV-19/Mexico/PUE-INER-IMSS-00155/2021	ΞPI_ISL_1279274	19/02/21	North America / Mexico / Puebla	Human	Male	33	Ambulatory	Nasopharyngeal and oropharyngeal swab
hCoV-19/Mexico/CAM-INER-IMSS-00368/2021	ΞPI_ISL_1279282	11/02/21	North America / Mexico / Campeche	Human	Male	30	Ambulatory	Nasopharyngeal and oropharyngeal swab
hCoV-19/Mexico/SLP-INER-IMSS-00162/2021	ΞPI_ISL_1279286	18/02/21	North America / Mexico / San Luis Potosi	Human	Male	53	Hospitalized	Nasopharyngeal and oropharyngeal swab
hCoV-19/Mexico/CMX-INER-IMSS-00179/2021	ΞPI_ISL_127929C	22/02/21	North America / Mexico / Mexico City	Human	Female	54	Hospitalized	Pharyngeal swab
hCoV-19/Mexico/CMX-INER-IMSS-00180/2021	ΞPI_ISL_1279291	22/02/21	North America / Mexico / Mexico City	Human	Male	81	Hospitalized	Pharyngeal swab
hCoV-19/Mexico/CMX-INER-IMSS-00181/2021	ΞPI_ISL_1279292	22/02/21	North America / Mexico / Mexico City	Human	Male	77	Hospitalized	Pharyngeal swab
hCoV-19/Mexico/VER-INER-IMSS-00354/2021	ΞPI_ISL_1279293	20/02/21	North America / Mexico / Veracruz	Human	Male	24	Ambulatory	Pharyngeal swab
hCoV-19/Mexico/ZAC-INER-IMSS-00055/2021	ΞPI_ISL_1279295	15/02/21	North America / Mexico / Zacatecas	Human	Male	52	Ambulatory	Nasopharyngeal and oropharyngeal swab
hCoV-19/Mexico/SLP-INER-IMSS-00337/2021	ΞPI_ISL_127929ς	23/02/21	North America / Mexico / San Luis Potosi	Human	Female	73	Hospitalized	Nasopharyngeal and oropharyngeal swab
hCoV-19/Mexico/NLE-INER-IMSS-00249/2021	ΞPI_ISL_1279302	16/02/21	North America / Mexico / Nuevo Leon	Human	Male	34	Ambulatory	Nasopharyngeal and oropharyngeal swab
hCoV-19/Mexico/ZAC-INER-IMSS-00009/2021	ΞPI_ISL_1279312	08/02/21	North America / Mexico / Zacatecas	Human	Male	54	Hospitalized	Nasopharyngeal and oropharyngeal swab
hCoV-19/Mexico/ZAC-INER-IMSS-00011/2021	ΞPI_ISL_1279313	09/02/21	North America / Mexico / Zacatecas	Human	Female	49	Ambulatory	Nasopharyngeal and oropharyngeal swab
hCoV-19/Mexico/ZAC-INER-IMSS-00015/2021	ΞPI_ISL_1279317	09/02/21	North America / Mexico / Zacatecas	Human	Female	66	Ambulatory	Nasopharyngeal and oropharyngeal swab
hCoV-19/Mexico/ZAC-INER-IMSS-00018/2021	ΞPI_ISL_127932C	10/02/21	North America / Mexico / Zacatecas	Human	Male	40	Ambulatory	Nasopharyngeal and oropharyngeal swab
hCoV-19/Mexico/ZAC-INER-IMSS-00024/2021	ΞPI_ISL_1279325	11/02/21	North America / Mexico / Zacatecas	Human	Male	44	Ambulatory	Nasopharyngeal and oropharyngeal swab
hCoV-19/Mexico/ZAC-INER-IMSS-00027/2021	ΞPI_ISL_1279326	11/02/21	North America / Mexico / Zacatecas	Human	Male	21	Ambulatory	Nasopharyngeal and oropharyngeal swab
hCoV-19/Mexico/ZAC-INER-IMSS-00028/2021	ΞPI_ISL_1279327	11/02/21	North America / Mexico / Zacatecas	Human	Male	35	Ambulatory	Nasopharyngeal and oropharyngeal swab
hCoV-19/Mexico/ZAC-INER-IMSS-00030/2021	ΞPI_ISL_127932ς	12/02/21	North America / Mexico / Zacatecas	Human	Male	24	Ambulatory	Nasopharyngeal and oropharyngeal swab
hCoV-19/Mexico/ZAC-INER-IMSS-00033/2021	ΞPI_ISL_1279332	11/02/21	North America / Mexico / Zacatecas	Human	Female	25	Ambulatory	Nasopharyngeal and oropharyngeal swab
hCoV-19/Mexico/ZAC-INER-IMSS-00034/2021	ΞPI_ISL_1279333	12/02/21	North America / Mexico / Zacatecas	Human	Male	50	Ambulatory	Nasopharyngeal and oropharyngeal swab
hCoV-19/Mexico/ZAC-INER-IMSS-00041/2021	ΞPI_ISL_127934C	13/02/21	North America / Mexico / Zacatecas	Human	Female	44	Ambulatory	Nasopharyngeal and oropharyngeal swab
hCoV-19/Mexico/ZAC-INER-IMSS-00042/2021	ΞPI_ISL_1279341	13/02/21	North America / Mexico / Zacatecas	Human	Female	35	Ambulatory	Nasopharyngeal and oropharyngeal swab
hCoV-19/Mexico/ZAC-INER-IMSS-00043/2021	ΞPI_ISL_1279342	13/02/21	North America / Mexico / Zacatecas	Human	Male	41	Ambulatory	Nasopharyngeal and oropharyngeal swab
hCoV-19/Mexico/ZAC-INER-IMSS-00044/2021	ΞPI_ISL_1279343	14/02/21	North America / Mexico / Zacatecas	Human	Male	77	Ambulatory	Nasopharyngeal and oropharyngeal swab
hCoV-19/Mexico/ZAC-INER-IMSS-00047/2021	ΞPI_ISL_1279346	14/02/21	North America / Mexico / Zacatecas	Human	Male	59	Hospitalized	Nasopharyngeal and oropharyngeal swab
hCoV-19/Mexico/ZAC-INER-IMSS-00048/2021	ΞPI_ISL_1279347	14/02/21	North America / Mexico / Zacatecas	Human	Female	71	Hospitalized	Nasopharyngeal and oropharyngeal swab
hCoV-19/Mexico/ZAC-INER-IMSS-00051/2021	ΞPI_ISL_127934ς	13/02/21	North America / Mexico / Zacatecas	Human	Female	44	Ambulatory	Nasopharyngeal and oropharyngeal swab
hCoV-19/Mexico/ZAC-INER-IMSS-00060/2021	ΞPI_ISL_1279353	15/02/21	North America / Mexico / Zacatecas	Human	Female	55	Ambulatory	Nasopharyngeal and oropharyngeal swab
hCoV-19/Mexico/ZAC-INER-IMSS-00061/2021	ΞPI_ISL_1279354	15/02/21	North America / Mexico / Zacatecas	Human	Male	43	Ambulatory	Nasopharyngeal and oropharyngeal swab
hCoV-19/Mexico/ZAC-INER-IMSS-00063/2021	ΞPI_ISL_1279356	15/02/21	North America / Mexico / Zacatecas	Human	Male	27	Ambulatory	Nasopharyngeal and oropharyngeal swab
hCoV-19/Mexico/ZAC-INER-IMSS-00067/2021	ΞPI_ISL_127936C	15/02/21	North America / Mexico / Zacatecas	Human	Female	60	Hospitalized	Nasopharyngeal and oropharyngeal swab
hCoV-19/Mexico/ZAC-INER-IMSS-00069/2021	ΞPI_ISL_1279361	16/02/21	North America / Mexico / Zacatecas	Human	Male	43	Ambulatory	Nasopharyngeal and oropharyngeal swab
hCoV-19/Mexico/ZAC-INER-IMSS-00070/2021	ΞPI_ISL_1279362	16/02/21	North America / Mexico / Zacatecas	Human	Female	46	Ambulatory	Nasopharyngeal and oropharyngeal swab
hCoV-19/Mexico/ZAC-INER-IMSS-00071/2021	ΞPI_ISL_1279363	16/02/21	North America / Mexico / Zacatecas	Human	Female	43	Ambulatory	Nasopharyngeal and oropharyngeal swab

hCoV-19/Mexico/ZAC-INER-IMSS-00076/2021	EPI_ISL_1279367	16/02/21	North America / Mexico / Zacatecas	Human	Female	26	Ambulatory	Nasopharyngeal and oropharyngeal swab
hCoV-19/Mexico/ZAC-INER-IMSS-00080/2021	EPI_ISL_1279368	16/02/21	North America / Mexico / Zacatecas	Human	Female	50	Ambulatory	Nasopharyngeal and oropharyngeal swab
hCoV-19/Mexico/ZAC-INER-IMSS-00085/2021	EPI_ISL_1279372	17/02/21	North America / Mexico / Zacatecas	Human	Male	38	Ambulatory	Nasopharyngeal and oropharyngeal swab
hCoV-19/Mexico/ZAC-INER-IMSS-00087/2021	EPI_ISL_1279373	17/02/21	North America / Mexico / Zacatecas	Human	Male	87	Hospitalized	Nasopharyngeal and oropharyngeal swab
hCoV-19/Mexico/ZAC-INER-IMSS-00090/2021	EPI_ISL_1279375	18/02/21	North America / Mexico / Zacatecas	Human	Female	40	Ambulatory	Nasopharyngeal and oropharyngeal swab
hCoV-19/Mexico/ZAC-INER-IMSS-00096/2021	EPI_ISL_1279381	18/02/21	North America / Mexico / Zacatecas	Human	Male	54	Ambulatory	Nasopharyngeal and oropharyngeal swab
hCoV-19/Mexico/MEX-INER-IMSS-00098/2021	EPI_ISL_1279383	22/02/21	North America / Mexico / State of Mexico	Human	Male	61	Ambulatory	Nasopharyngeal and oropharyngeal swab
hCoV-19/Mexico/MEX-INER-IMSS-00100/2021	EPI_ISL_1279384	22/02/21	North America / Mexico / State of Mexico	Human	Male	77	Hospitalized	Pharyngeal swab
hCoV-19/Mexico/MEX-INER-IMSS-00101/2021	EPI_ISL_1279385	22/02/21	North America / Mexico / State of Mexico	Human	Male	24	Ambulatory	Nasopharyngeal and oropharyngeal swab
hCoV-19/Mexico/CMX-INER-IMSS-00102/2021	EPI_ISL_1279386	22/02/21	North America / Mexico / Mexico City	Human	Female	49	Ambulatory	Pharyngeal swab
hCoV-19/Mexico/MEX-INER-IMSS-00103/2021	EPI_ISL_1279387	22/02/21	North America / Mexico / State of Mexico	Human	Female	31	Hospitalized	Nasopharyngeal and oropharyngeal swab
hCoV-19/Mexico/MEX-INER-IMSS-00104/2021	EPI_ISL_1279388	22/02/21	North America / Mexico / State of Mexico	Human	Male	66	Hospitalized	Nasopharyngeal and oropharyngeal swab
hCoV-19/Mexico/QUE-INER-IMSS-00105/2021	EPI_ISL_1279389	20/02/21	North America / Mexico / Queretaro	Human	Female	63	Hospitalized	Nasopharyngeal and oropharyngeal swab
hCoV-19/Mexico/MEX-INER-IMSS-00106/2021	EPI_ISL_1279390	22/02/21	North America / Mexico / State of Mexico	Human	Female	64	Hospitalized	Nasopharyngeal and oropharyngeal swab
hCoV-19/Mexico/MEX-INER-IMSS-00107/2021	EPI_ISL_1279391	22/02/21	North America / Mexico / State of Mexico	Human	Female	56	Hospitalized	Nasopharyngeal and oropharyngeal swab
hCoV-19/Mexico/QUE-INER-IMSS-00108/2021	EPI_ISL_1279392	20/02/21	North America / Mexico / Queretaro	Human	Female	67	Hospitalized	Nasopharyngeal and oropharyngeal swab
hCoV-19/Mexico/QUE-INER-IMSS-00110/2021	EPI_ISL_1279394	21/02/21	North America / Mexico / Queretaro	Human	Male	97	Hospitalized	Nasopharyngeal and oropharyngeal swab
hCoV-19/Mexico/QUE-INER-IMSS-00111/2021	EPI_ISL_1279395	21/02/21	North America / Mexico / Queretaro	Human	Female	53	Hospitalized	Nasopharyngeal and oropharyngeal swab
hCoV-19/Mexico/MEX-INER-IMSS-00113/2021	EPI_ISL_1279396	22/02/21	North America / Mexico / State of Mexico	Human	Male	50	Hospitalized	Nasopharyngeal and oropharyngeal swab
hCoV-19/Mexico/HID-INER-IMSS-00114/2021	EPI_ISL_1279397	22/02/21	North America / Mexico / Hidalgo	Human	Male	1	Ambulatory	Nasopharyngeal swab
hCoV-19/Mexico/PUE-INER-IMSS-00115/2021	EPI_ISL_1279398	20/02/21	North America / Mexico / Puebla	Human	Female	85	Hospitalized	Nasopharyngeal and oropharyngeal swab
hCoV-19/Mexico/PUE-INER-IMSS-00116/2021	EPI_ISL_1279399	20/02/21	North America / Mexico / Puebla	Human	Female	60	Hospitalized	Nasopharyngeal and oropharyngeal swab
hCoV-19/Mexico/PUE-INER-IMSS-00117/2021	EPI_ISL_1279400	20/02/21	North America / Mexico / Puebla	Human	Male	77	Hospitalized	Nasopharyngeal and oropharyngeal swab
hCoV-19/Mexico/PUE-INER-IMSS-00118/2021	EPI_ISL_1279401	20/02/21	North America / Mexico / Puebla	Human	Male	61	Hospitalized	Nasopharyngeal and oropharyngeal swab
hCoV-19/Mexico/PUE-INER-IMSS-00121/2021	EPI_ISL_1279403	17/02/21	North America / Mexico / Puebla	Human	Female	55	Ambulatory	Nasopharyngeal and oropharyngeal swab
hCoV-19/Mexico/PUE-INER-IMSS-00122/2021	EPI_ISL_1279404	18/02/21	North America / Mexico / Puebla	Human	Male	22	Ambulatory	Nasopharyngeal and oropharyngeal swab
hCoV-19/Mexico/PUE-INER-IMSS-00123/2021	EPI_ISL_1279405	18/02/21	North America / Mexico / Puebla	Human	Male	27	Ambulatory	Nasopharyngeal and oropharyngeal swab
hCoV-19/Mexico/PUE-INER-IMSS-00126/2021	EPI_ISL_1279407	19/02/21	North America / Mexico / Puebla	Human	Female	48	Ambulatory	Nasopharyngeal and oropharyngeal swab
hCoV-19/Mexico/PUE-INER-IMSS-00127/2021	EPI_ISL_1279408	20/02/21	North America / Mexico / Puebla	Human	Female	72	Ambulatory	Nasopharyngeal and oropharyngeal swab
hCoV-19/Mexico/PUE-INER-IMSS-00128/2021	EPI_ISL_1279409	20/02/21	North America / Mexico / Puebla	Human	Male	15	Ambulatory	Nasopharyngeal and oropharyngeal swab
hCoV-19/Mexico/PUE-INER-IMSS-00129/2021	EPI_ISL_1279410	21/02/21	North America / Mexico / Puebla	Human	Male	36	Ambulatory	Nasopharyngeal and oropharyngeal swab
hCoV-19/Mexico/PUE-INER-IMSS-00130/2021	EPI_ISL_1279411	19/02/21	North America / Mexico / Puebla	Human	Female	52	Ambulatory	Nasopharyngeal and oropharyngeal swab
hCoV-19/Mexico/PUE-INER-IMSS-00131/2021	EPI_ISL_1279412	19/02/21	North America / Mexico / Puebla	Human	Female	15	Ambulatory	Nasopharyngeal and oropharyngeal swab
hCoV-19/Mexico/PUE-INER-IMSS-00132/2021	EPI_ISL_1279413	19/02/21	North America / Mexico / Puebla	Human	Male	54	Ambulatory	Nasopharyngeal and oropharyngeal swab
hCoV-19/Mexico/PUE-INER-IMSS-00133/2021	EPI_ISL_1279414	20/02/21	North America / Mexico / Puebla	Human	Male	57	Ambulatory	Nasopharyngeal and oropharyngeal swab
hCoV-19/Mexico/PUE-INER-IMSS-00134/2021	EPI_ISL_1279415	20/02/21	North America / Mexico / Puebla	Human	Female	23	Ambulatory	Nasopharyngeal and oropharyngeal swab
hCoV-19/Mexico/PUE-INER-IMSS-00136/2021	EPI_ISL_1279416	20/02/21	North America / Mexico / Puebla	Human	Male	46	Ambulatory	Nasopharyngeal and oropharyngeal swab
hCoV-19/Mexico/PUE-INER-IMSS-00138/2021	EPI_ISL_1279417	20/02/21	North America / Mexico / Puebla	Human	Male	48	Ambulatory	Nasopharyngeal and oropharyngeal swab
hCoV-19/Mexico/PUE-INER-IMSS-00139/2021	EPI_ISL_1279418	20/02/21	North America / Mexico / Puebla	Human	Male	32	Ambulatory	Nasopharyngeal and oropharyngeal swab
hCoV-19/Mexico/PUE-INER-IMSS-00141/2021	EPI_ISL_1279420	17/02/21	North America / Mexico / Puebla	Human	Male	53	Hospitalized	Nasopharyngeal and oropharyngeal swab
hCoV-19/Mexico/PUE-INER-IMSS-00142/2021	EPI_ISL_1279421	17/02/21	North America / Mexico / Puebla	Human	Male	82	Hospitalized	Nasopharyngeal and oropharyngeal swab
hCoV-19/Mexico/PUE-INER-IMSS-00143/2021	EPI_ISL_1279422	17/02/21	North America / Mexico / Puebla	Human	Male	58	Hospitalized	Nasopharyngeal and oropharyngeal swab
hCoV-19/Mexico/PUE-INER-IMSS-00144/2021	EPI_ISL_1279423	16/02/21	North America / Mexico / Puebla	Human	Female	61	Hospitalized	Nasopharyngeal and oropharyngeal swab
hCoV-19/Mexico/PUE-INER-IMSS-00145/2021	EPI_ISL_1279424	18/02/21	North America / Mexico / Puebla	Human	Female	30	Ambulatory	Nasopharyngeal swab

hCoV-19/Mexico/PUE-INER-IMSS-00146/2021	EPI_ISL_1279425	18/02/21	North America / Mexico / Puebla	Human	Male	33	Ambulatory	Nasopharyngeal swab
hCoV-19/Mexico/PUE-INER-IMSS-00149/2021	EPI_ISL_1279427	17/02/21	North America / Mexico / Puebla	Human	Female	34	Ambulatory	Nasopharyngeal and oropharyngeal swab
hCoV-19/Mexico/PUE-INER-IMSS-00150/2021	EPI_ISL_1279428	20/02/21	North America / Mexico / Puebla	Human	Male	44	Ambulatory	Nasopharyngeal swab
hCoV-19/Mexico/PUE-INER-IMSS-00151/2021	EPI_ISL_1279429	20/02/21	North America / Mexico / Puebla	Human	Female	39	Ambulatory	Nasopharyngeal swab
hCoV-19/Mexico/PUE-INER-IMSS-00152/2021	EPI_ISL_1279430	18/02/21	North America / Mexico / Puebla	Human	Male	32	Ambulatory	Nasopharyngeal and oropharyngeal swab
hCoV-19/Mexico/PUE-INER-IMSS-00153/2021	EPI_ISL_1279431	19/02/21	North America / Mexico / Puebla	Human	Male	43	Ambulatory	Nasopharyngeal and oropharyngeal swab
hCoV-19/Mexico/PUE-INER-IMSS-00154/2021	EPI_ISL_1279432	19/02/21	North America / Mexico / Puebla	Human	Male	47	Ambulatory	Nasopharyngeal and oropharyngeal swab
hCoV-19/Mexico/PUE-INER-IMSS-00156/2021	EPI_ISL_1279433	20/02/21	North America / Mexico / Puebla	Human	Male	50	Ambulatory	Nasopharyngeal swab
hCoV-19/Mexico/PUE-INER-IMSS-00157/2021	EPI_ISL_1279434	20/02/21	North America / Mexico / Puebla	Human	Male	80	Ambulatory	Nasopharyngeal swab
hCoV-19/Mexico/PUE-INER-IMSS-00158/2021	EPI_ISL_1279435	20/02/21	North America / Mexico / Puebla	Human	Female	27	Ambulatory	Nasopharyngeal swab
hCoV-19/Mexico/PUE-INER-IMSS-00159/2021	EPI_ISL_1279436	20/02/21	North America / Mexico / Puebla	Human	Male	35	Ambulatory	Nasopharyngeal swab
hCoV-19/Mexico/PUE-INER-IMSS-00160/2021	EPI_ISL_1279437	20/02/21	North America / Mexico / Puebla	Human	Male	38	Ambulatory	Nasopharyngeal swab
hCoV-19/Mexico/PUE-INER-IMSS-00161/2021	EPI_ISL_1279438	20/02/21	North America / Mexico / Puebla	Human	Male	41	Ambulatory	Nasopharyngeal swab
hCoV-19/Mexico/ROO-INER-IMSS-00164/2021	EPI_ISL_1279440	10/02/21	North America / Mexico / Quintana Roo	Human	Female	62	Ambulatory	Nasopharyngeal and oropharyngeal swab
hCoV-19/Mexico/OAX-INER-IMSS-00165/2021	EPI_ISL_1279441	21/02/21	North America / Mexico / Oaxaca	Human	Male	48	Hospitalized	Nasopharyngeal and oropharyngeal swab
hCoV-19/Mexico/OAX-INER-IMSS-00166/2021	EPI_ISL_1279442	21/02/21	North America / Mexico / Oaxaca	Human	Female	23	Ambulatory	Nasopharyngeal and oropharyngeal swab
hCoV-19/Mexico/OAX-INER-IMSS-00167/2021	EPI_ISL_1279443	21/02/21	North America / Mexico / Oaxaca	Human	Male	28	Hospitalized	Nasopharyngeal and oropharyngeal swab
hCoV-19/Mexico/SLP-INER-IMSS-00168/2021	EPI_ISL_1279444	21/02/21	North America / Mexico / San Luis Potosi	Human	Female	50	Hospitalized	Nasopharyngeal and oropharyngeal swab
hCoV-19/Mexico/SLP-INER-IMSS-00171/2021	EPI_ISL_1279445	20/02/21	North America / Mexico / San Luis Potosi	Human	Male	78	Hospitalized	Nasopharyngeal and oropharyngeal swab
hCoV-19/Mexico/SLP-INER-IMSS-00173/2021	EPI_ISL_1279447	20/02/21	North America / Mexico / San Luis Potosi	Human	Female	53	Hospitalized	Nasopharyngeal and oropharyngeal swab
hCoV-19/Mexico/VER-INER-IMSS-00174/2021	EPI_ISL_1279448	15/02/21	North America / Mexico / Veracruz	Human	Female	81	Hospitalized	Nasopharyngeal and oropharyngeal swab
hCoV-19/Mexico/VER-INER-IMSS-00175/2021	EPI_ISL_1279449	17/02/21	North America / Mexico / Veracruz	Human	Male	50	Ambulatory	Nasopharyngeal and oropharyngeal swab
hCoV-19/Mexico/VER-INER-IMSS-00176/2021	EPI_ISL_1279450	19/02/21	North America / Mexico / Veracruz	Human	Male	68	Hospitalized	Nasopharyngeal and oropharyngeal swab
hCoV-19/Mexico/CMX-INER-IMSS-00178/2021	EPI_ISL_1279452	22/02/21	North America / Mexico / Mexico City	Human	Female	33	Hospitalized	Pharyngeal swab
hCoV-19/Mexico/OAX-INER-IMSS-00182/2021	EPI_ISL_1279453	20/02/21	North America / Mexico / Oaxaca	Human	Male	57	Hospitalized	Nasopharyngeal and oropharyngeal swab
hCoV-19/Mexico/OAX-INER-IMSS-00183/2021	EPI_ISL_1279454	20/02/21	North America / Mexico / Oaxaca	Human	Female	51	Ambulatory	Nasopharyngeal and oropharyngeal swab
hCoV-19/Mexico/CMX-INER-IMSS-00186/2021	EPI_ISL_1279457	23/02/21	North America / Mexico / Mexico City	Human	Female	72	Hospitalized	Pharyngeal swab
hCoV-19/Mexico/MEX-INER-IMSS-00187/2021	EPI_ISL_1279458	23/02/21	North America / Mexico / State of Mexico	Human	Female	58	Ambulatory	Pharyngeal swab
hCoV-19/Mexico/CMX-INER-IMSS-00188/2021	EPI_ISL_1279459	23/02/21	North America / Mexico / Mexico City	Human	Male	39	Ambulatory	Pharyngeal swab
hCoV-19/Mexico/CMX-INER-IMSS-00189/2021	EPI_ISL_1279460	23/02/21	North America / Mexico / Mexico City	Human	Female	100	Hospitalized	Pharyngeal swab
hCoV-19/Mexico/AGU-INER-IMSS-00190/2021	EPI_ISL_1279461	19/02/21	North America / Mexico / Aguascalientes	Human	Female	36	Ambulatory	Nasopharyngeal and oropharyngeal swab
hCoV-19/Mexico/DUR-INER-IMSS-00194/2021	EPI_ISL_1279465	08/02/21	North America / Mexico / Durango	Human	Female	35	Ambulatory	Nasopharyngeal and oropharyngeal swab
hCoV-19/Mexico/CHH-INER-IMSS-00195/2021	EPI_ISL_1279466	08/02/21	North America / Mexico / Chihuahua	Human	Female	30	Hospitalized	Nasopharyngeal and oropharyngeal swab
hCoV-19/Mexico/TAM-INER-IMSS-00209/2021	EPI_ISL_1279476	10/02/21	North America / Mexico / Tamaulipas	Human	Female	37	Ambulatory	Nasopharyngeal and oropharyngeal swab
hCoV-19/Mexico/COA-INER-IMSS-00211/2021	EPI_ISL_1279477	11/02/21	North America / Mexico / Coahuila	Human	Female	75	Ambulatory	Nasopharyngeal and oropharyngeal swab
hCoV-19/Mexico/SLP-INER-IMSS-00218/2021	EPI_ISL_1279484	10/02/21	North America / Mexico / San Luis Potosi	Human	Female	71	Ambulatory	Nasopharyngeal and oropharyngeal swab
hCoV-19/Mexico/NLE-INER-IMSS-00225/2021	EPI_ISL_1279490	15/02/21	North America / Mexico / Nuevo Leon	Human	Male	27	Ambulatory	Nasopharyngeal and oropharyngeal swab
hCoV-19/Mexico/TAM-INER-IMSS-00227/2021	EPI_ISL_1279492	12/02/21	North America / Mexico / Tamaulipas	Human	Female	53	Ambulatory	Pharyngeal swab
hCoV-19/Mexico/NLE-INER-IMSS-00232/2021	EPI_ISL_1279496	16/02/21	North America / Mexico / Nuevo Leon	Human	Female	47	Ambulatory	Nasopharyngeal and oropharyngeal swab
hCoV-19/Mexico/NLE-INER-IMSS-00234/2021	EPI_ISL_1279498	16/02/21	North America / Mexico / Nuevo Leon	Human	Male	70	Hospitalized	Nasopharyngeal and oropharyngeal swab
hCoV-19/Mexico/NLE-INER-IMSS-00236/2021	EPI_ISL_1279500	16/02/21	North America / Mexico / Nuevo Leon	Human	Female	62	Hospitalized	Nasopharyngeal and oropharyngeal swab
hCoV-19/Mexico/COA-INER-IMSS-00240/2021	EPI_ISL_1279504	13/02/21	North America / Mexico / Coahuila	Human	Female	31	Hospitalized	Nasopharyngeal and oropharyngeal swab
hCoV-19/Mexico/COA-INER-IMSS-00243/2021	EPI_ISL_1279506	15/02/21	North America / Mexico / Coahuila	Human	Female	34	Hospitalized	Nasopharyngeal and oropharyngeal swab
hCoV-19/Mexico/COA-INER-IMSS-00246/2021	EPI_ISL_1279509	13/02/21	North America / Mexico / Coahuila	Human	Female	44	Hospitalized	Nasopharyngeal and oropharyngeal swab

hCoV-19/Mexico/NLE-INER-IMSS-00252/2021	EPI_ISL_1279514	17/02/21	North America / Mexico / Nuevo Leon	Human	Female	77	Ambulatory	Nasopharyngeal and oropharyngeal swab
hCoV-19/Mexico/TAM-INER-IMSS-00254/2021	EPI_ISL_1279516	16/02/21	North America / Mexico / Tamaulipas	Human	Male	41	Ambulatory	Nasopharyngeal and oropharyngeal swab
hCoV-19/Mexico/TAM-INER-IMSS-00259/2021	EPI_ISL_1279521	18/02/21	North America / Mexico / Tamaulipas	Human	Male	27	Hospitalized	Nasopharyngeal and oropharyngeal swab
hCoV-19/Mexico/CHH-INER-IMSS-00272/2021	EPI_ISL_1279531	13/02/21	North America / Mexico / Chihuahua	Human	Female	68	Hospitalized	Nasopharyngeal and oropharyngeal swab
hCoV-19/Mexico/DUR-INER-IMSS-00274/2021	EPI_ISL_1279532	17/02/21	North America / Mexico / Durango	Human	Female	62	Hospitalized	Nasopharyngeal and oropharyngeal swab
hCoV-19/Mexico/DUR-INER-IMSS-00275/2021	EPI_ISL_1279533	16/02/21	North America / Mexico / Durango	Human	Female	33	Ambulatory	Nasopharyngeal and oropharyngeal swab
hCoV-19/Mexico/TAM-INER-IMSS-00276/2021	EPI_ISL_1279534	17/02/21	North America / Mexico / Tamaulipas	Human	Female	49	Hospitalized	Nasopharyngeal and oropharyngeal swab
hCoV-19/Mexico/TAM-INER-IMSS-00277/2021	EPI_ISL_1279535	12/02/21	North America / Mexico / Tamaulipas	Human	Female	28	Ambulatory	Nasopharyngeal and oropharyngeal swab
hCoV-19/Mexico/GRO-INER-IMSS-00280/2021	EPI_ISL_1279537	20/02/21	North America / Mexico / Guerrero	Human	Female	81	Hospitalized	Nasopharyngeal and oropharyngeal swab
hCoV-19/Mexico/PUE-INER-IMSS-00282/2021	EPI_ISL_1279538	19/02/21	North America / Mexico / Puebla	Human	Female	21	Ambulatory	Nasopharyngeal and oropharyngeal swab
hCoV-19/Mexico/TLA-INER-IMSS-00284/2021	EPI_ISL_1279539	22/02/21	North America / Mexico / Tlaxcala	Human	Male	62	Hospitalized	Nasopharyngeal and oropharyngeal swab
hCoV-19/Mexico/TLA-INER-IMSS-00285/2021	EPI_ISL_1279540	23/02/21	North America / Mexico / Tlaxcala	Human	Male	79	Hospitalized	Nasopharyngeal and oropharyngeal swab
hCoV-19/Mexico/VER-INER-IMSS-00286/2021	EPI_ISL_1279541	17/02/21	North America / Mexico / Veracruz	Human	Male	35	Ambulatory	Nasopharyngeal and oropharyngeal swab
hCoV-19/Mexico/VER-INER-IMSS-00287/2021	EPI_ISL_1279542	19/02/21	North America / Mexico / Veracruz	Human	Male	28	Ambulatory	Nasopharyngeal and oropharyngeal swab
hCoV-19/Mexico/VER-INER-IMSS-00290/2021	EPI_ISL_1279544	19/02/21	North America / Mexico / Veracruz	Human	Male	22	Ambulatory	Nasopharyngeal and oropharyngeal swab
hCoV-19/Mexico/VER-INER-IMSS-00291/2021	EPI_ISL_1279545	19/02/21	North America / Mexico / Veracruz	Human	Male	38	Ambulatory	Nasopharyngeal and oropharyngeal swab
hCoV-19/Mexico/VER-INER-IMSS-00293/2021	EPI_ISL_1279546	20/02/21	North America / Mexico / Veracruz	Human	Male	61	Hospitalized	Nasopharyngeal and oropharyngeal swab
hCoV-19/Mexico/VER-INER-IMSS-00294/2021	EPI_ISL_1279547	20/02/21	North America / Mexico / Veracruz	Human	Female	92	Hospitalized	Nasopharyngeal and oropharyngeal swab
hCoV-19/Mexico/VER-INER-IMSS-00295/2021	EPI_ISL_1279548	21/02/21	North America / Mexico / Veracruz	Human	Male	83	Hospitalized	Nasopharyngeal and oropharyngeal swab
hCoV-19/Mexico/VER-INER-IMSS-00298/2021	EPI_ISL_1279551	21/02/21	North America / Mexico / Veracruz	Human	Male	40	Hospitalized	Nasopharyngeal and oropharyngeal swab
hCoV-19/Mexico/VER-INER-IMSS-00299/2021	EPI_ISL_1279552	19/02/21	North America / Mexico / Veracruz	Human	Female	33	Ambulatory	Nasopharyngeal and oropharyngeal swab
hCoV-19/Mexico/VER-INER-IMSS-00300/2021	EPI_ISL_1279553	19/02/21	North America / Mexico / Veracruz	Human	Female	49	Ambulatory	Nasopharyngeal and oropharyngeal swab
hCoV-19/Mexico/VER-INER-IMSS-00301/2021	EPI_ISL_1279554	19/02/21	North America / Mexico / Veracruz	Human	Male	69	Hospitalized	Nasopharyngeal and oropharyngeal swab
hCoV-19/Mexico/VER-INER-IMSS-00302/2021	EPI_ISL_1279555	19/02/21	North America / Mexico / Veracruz	Human	Male	31	Hospitalized	Nasopharyngeal and oropharyngeal swab
hCoV-19/Mexico/VER-INER-IMSS-00303/2021	EPI_ISL_1279556	19/02/21	North America / Mexico / Veracruz	Human	Female	45	Ambulatory	Nasopharyngeal and oropharyngeal swab
hCoV-19/Mexico/GRO-INER-IMSS-00304/2021	EPI_ISL_1279557	20/02/21	North America / Mexico / Guerrero	Human	Male	47	Ambulatory	Nasopharyngeal and oropharyngeal swab
hCoV-19/Mexico/MOR-INER-IMSS-00305/2021	EPI_ISL_1279558	21/02/21	North America / Mexico / Morelos	Human	Male	83	Hospitalized	Nasopharyngeal and oropharyngeal swab
hCoV-19/Mexico/MEX-INER-IMSS-00306/2021	EPI_ISL_1279559	22/02/21	North America / Mexico / State of Mexico	Human	Female	34	Hospitalized	Nasopharyngeal and oropharyngeal swab
hCoV-19/Mexico/MOR-INER-IMSS-00307/2021	EPI_ISL_1279560	18/02/21	North America / Mexico / Morelos	Human	Male	44	Hospitalized	Nasopharyngeal and oropharyngeal swab
hCoV-19/Mexico/MEX-INER-IMSS-00308/2021	EPI_ISL_1279561	22/02/21	North America / Mexico / State of Mexico	Human	Female	82	Ambulatory	Nasopharyngeal and oropharyngeal swab
hCoV-19/Mexico/MEX-INER-IMSS-00309/2021	EPI_ISL_1279562	22/02/21	North America / Mexico / State of Mexico	Human	Female	63	Hospitalized	Nasopharyngeal and oropharyngeal swab
hCoV-19/Mexico/MEX-INER-IMSS-00310/2021	EPI_ISL_1279563	22/02/21	North America / Mexico / State of Mexico	Human	Female	70	Hospitalized	Nasopharyngeal and oropharyngeal swab
hCoV-19/Mexico/VER-INER-IMSS-00311/2021	EPI_ISL_1279564	19/02/21	North America / Mexico / Veracruz	Human	Female	37	Ambulatory	Nasopharyngeal and oropharyngeal swab
hCoV-19/Mexico/VER-INER-IMSS-00314/2021	EPI_ISL_1279566	22/02/21	North America / Mexico / Veracruz	Human	Female	82	Hospitalized	Nasopharyngeal and oropharyngeal swab
hCoV-19/Mexico/VER-INER-IMSS-00315/2021	EPI_ISL_1279567	22/02/21	North America / Mexico / Veracruz	Human	Female	74	Hospitalized	Nasopharyngeal and oropharyngeal swab
hCoV-19/Mexico/VER-INER-IMSS-00316/2021	EPI_ISL_1279568	22/02/21	North America / Mexico / Veracruz	Human	Male	55	Hospitalized	Nasopharyngeal and oropharyngeal swab
hCoV-19/Mexico/VER-INER-IMSS-00317/2021	EPI_ISL_1279569	22/02/21	North America / Mexico / Veracruz	Human	Female	65	Hospitalized	Nasopharyngeal and oropharyngeal swab
hCoV-19/Mexico/MEX-INER-IMSS-00320/2021	EPI_ISL_1279570	23/02/21	North America / Mexico / State of Mexico	Human	Female	48	Hospitalized	Pharyngeal swab
hCoV-19/Mexico/MEX-INER-IMSS-00321/2021	EPI_ISL_1279571	22/02/21	North America / Mexico / State of Mexico	Human	Female	59	Ambulatory	Nasopharyngeal and oropharyngeal swab
hCoV-19/Mexico/MEX-INER-IMSS-00322/2021	EPI_ISL_1279572	23/02/21	North America / Mexico / State of Mexico	Human	Female	32	Hospitalized	Pharyngeal swab
hCoV-19/Mexico/MEX-INER-IMSS-00323/2021	EPI_ISL_1279573	23/02/21	North America / Mexico / State of Mexico	Human	Male	45	Ambulatory	Nasopharyngeal and oropharyngeal swab
hCoV-19/Mexico/MEX-INER-IMSS-00326/2021	EPI_ISL_1279575	23/02/21	North America / Mexico / State of Mexico	Human	Male	69	Hospitalized	Nasopharyngeal and oropharyngeal swab
hCoV-19/Mexico/MEX-INER-IMSS-00327/2021	EPI_ISL_1279576	23/02/21	North America / Mexico / State of Mexico	Human	Male	48	Hospitalized	Nasopharyngeal and oropharyngeal swab
hCoV-19/Mexico/MEX-INER-IMSS-00329/2021	EPI_ISL_1279577	23/02/21	North America / Mexico / State of Mexico	Human	Male	47	Hospitalized	Pharyngeal swab

hCoV-19/Mexico/HID-INER-IMSS-00330/2021	EPI_ISL_1279578	22/02/21	North America / Mexico / Hidalgo	Human	Female	37	Hospitalized	Nasopharyngeal and oropharyngeal swab
hCoV-19/Mexico/HID-INER-IMSS-00332/2021	EPI_ISL_1279580	22/02/21	North America / Mexico / Hidalgo	Human	Male	94	Hospitalized	Nasopharyngeal and oropharyngeal swab
hCoV-19/Mexico/MOR-INER-IMSS-00333/2021	EPI_ISL_1279581	22/02/21	North America / Mexico / Morelos	Human	Male	67	Ambulatory	Nasopharyngeal and oropharyngeal swab
hCoV-19/Mexico/CMX-INER-IMSS-00334/2021	EPI_ISL_1279582	22/02/21	North America / Mexico / Mexico City	Human	Female	37	Ambulatory	Nasopharyngeal and oropharyngeal swab
hCoV-19/Mexico/MEX-INER-IMSS-00335/2021	EPI_ISL_1279583	22/02/21	North America / Mexico / State of Mexico	Human	Male	25	Ambulatory	Nasopharyngeal and oropharyngeal swab
hCoV-19/Mexico/SLP-INER-IMSS-00336/2021	EPI_ISL_1279584	23/02/21	North America / Mexico / San Luis Potosi	Human	Female	40	Hospitalized	Nasopharyngeal and oropharyngeal swab
hCoV-19/Mexico/MEX-INER-IMSS-00338/2021	EPI_ISL_1279585	23/02/21	North America / Mexico / State of Mexico	Human	Male	70	Ambulatory	Nasopharyngeal and oropharyngeal swab
hCoV-19/Mexico/MEX-INER-IMSS-00339/2021	EPI_ISL_1279586	23/02/21	North America / Mexico / State of Mexico	Human	Male	52	Ambulatory	Nasopharyngeal and oropharyngeal swab
hCoV-19/Mexico/CMX-INER-IMSS-00341/2021	EPI_ISL_1279587	24/02/21	North America / Mexico / Mexico City	Human	Female	89	Hospitalized	Pharyngeal swab
hCoV-19/Mexico/OAX-INER-IMSS-00343/2021	EPI_ISL_1279589	22/02/21	North America / Mexico / Oaxaca	Human	Male	62	Ambulatory	Nasopharyngeal and oropharyngeal swab
hCoV-19/Mexico/OAX-INER-IMSS-00344/2021	EPI_ISL_1279590	22/02/21	North America / Mexico / Oaxaca	Human	Female	60	Hospitalized	Nasopharyngeal and oropharyngeal swab
hCoV-19/Mexico/VER-INER-IMSS-00345/2021	EPI_ISL_1279591	22/02/21	North America / Mexico / Veracruz	Human	Male	78	Hospitalized	Nasopharyngeal and oropharyngeal swab
hCoV-19/Mexico/VER-INER-IMSS-00346/2021	EPI_ISL_1279592	23/02/21	North America / Mexico / Veracruz	Human	Female	37	Ambulatory	Nasopharyngeal and oropharyngeal swab
hCoV-19/Mexico/VER-INER-IMSS-00347/2021	EPI_ISL_1279593	22/02/21	North America / Mexico / Veracruz	Human	Female	59	Hospitalized	Nasopharyngeal and oropharyngeal swab
hCoV-19/Mexico/VER-INER-IMSS-00348/2021	EPI_ISL_1279594	22/02/21	North America / Mexico / Veracruz	Human	Male	43	Hospitalized	Nasopharyngeal and oropharyngeal swab
hCoV-19/Mexico/CMX-INER-IMSS-00349/2021	EPI_ISL_1279595	24/02/21	North America / Mexico / Mexico City	Human	Male	5	Ambulatory	Nasopharyngeal and oropharyngeal swab
hCoV-19/Mexico/CMX-INER-IMSS-00350/2021	EPI_ISL_1279596	24/02/21	North America / Mexico / Mexico City	Human	Female	38	Hospitalized	Nasopharyngeal and oropharyngeal swab
hCoV-19/Mexico/MEX-INER-IMSS-00351/2021	EPI_ISL_1279597	23/02/21	North America / Mexico / State of Mexico	Human	Female	40	Hospitalized	Pharyngeal swab
hCoV-19/Mexico/CMX-INER-IMSS-00352/2021	EPI_ISL_1279598	24/02/21	North America / Mexico / Mexico City	Human	Male	37	Hospitalized	Nasopharyngeal and oropharyngeal swab
hCoV-19/Mexico/VER-INER-IMSS-00353/2021	EPI_ISL_1279599	19/02/21	North America / Mexico / Veracruz	Human	Male	15	Ambulatory	Nasopharyngeal and oropharyngeal swab
hCoV-19/Mexico/CMX-INER-IMSS-00355/2021	EPI_ISL_1279600	23/02/21	North America / Mexico / Mexico City	Human	Female	62	Hospitalized	Pharyngeal swab
hCoV-19/Mexico/MEX-INER-IMSS-00356/2021	EPI_ISL_1279601	24/02/21	North America / Mexico / State of Mexico	Human	Female	49	Hospitalized	Pharyngeal swab
hCoV-19/Mexico/VER-INER-IMSS-00357/2021	EPI_ISL_1279602	18/02/21	North America / Mexico / Veracruz	Human	Male	76	Hospitalized	Nasopharyngeal and oropharyngeal swab
hCoV-19/Mexico/VER-INER-IMSS-00358/2021	EPI_ISL_1279603	18/02/21	North America / Mexico / Veracruz	Human	Male	82	Hospitalized	Nasopharyngeal and oropharyngeal swab
hCoV-19/Mexico/ROO-INER-IMSS-00359/2021	EPI_ISL_1279604	10/02/21	North America / Mexico / Quintana Roo	Human	Male	75	Hospitalized	Nasopharyngeal and oropharyngeal swab
hCoV-19/Mexico/YUC-INER-IMSS-00360/2021	EPI_ISL_1279605	12/02/21	North America / Mexico / Yucatan	Human	Male	21	Ambulatory	Nasopharyngeal swab
hCoV-19/Mexico/YUC-INER-IMSS-00361/2021	EPI_ISL_1279606	12/02/21	North America / Mexico / Yucatan	Human	Male	31	Ambulatory	Nasopharyngeal swab
hCoV-19/Mexico/YUC-INER-IMSS-00363/2021	EPI_ISL_1279608	12/02/21	North America / Mexico / Yucatan	Human	Female	33	Ambulatory	Nasopharyngeal and oropharyngeal swab
hCoV-19/Mexico/YUC-INER-IMSS-00364/2021	EPI_ISL_1279609	13/02/21	North America / Mexico / Yucatan	Human	Female	67	Hospitalized	Nasopharyngeal and oropharyngeal swab
hCoV-19/Mexico/YUC-INER-IMSS-00365/2021	EPI_ISL_1279610	13/02/21	North America / Mexico / Yucatan	Human	Male	45	Hospitalized	Nasopharyngeal and oropharyngeal swab
hCoV-19/Mexico/ROO-INER-IMSS-00367/2021	EPI_ISL_1279612	11/02/21	North America / Mexico / Quintana Roo	Human	Female	21	Ambulatory	Nasopharyngeal and oropharyngeal swab
hCoV-19/Mexico/YUC-INER-IMSS-00370/2021	EPI_ISL_1279613	14/02/21	North America / Mexico / Yucatan	Human	Female	90	Hospitalized	Nasopharyngeal and oropharyngeal swab
hCoV-19/Mexico/CAM-INER-IMSS-00371/2021	EPI_ISL_1279614	12/02/21	North America / Mexico / Campeche	Human	Male	34	Ambulatory	Nasopharyngeal and oropharyngeal swab
hCoV-19/Mexico/DUR-INER-IMSS-00273/2021	EPI_ISL_1287772	16/02/21	North America / Mexico / Durango	Human	Female	50	Ambulatory	Nasopharyngeal and oropharyngeal swab
hCoV-19/Mexico/TLA-INER-IMSS-00283/2021	EPI_ISL_1287775	22/02/21	North America / Mexico / Tlaxcala	Human	Male	63	Hospitalized	Nasopharyngeal and oropharyngeal swab
hCoV-19/Mexico/MEX-INER-IMSS-00328/2021	EPI_ISL_1287777	23/02/21	North America / Mexico / State of Mexico	Human	Female	41	Hospitalized	Nasopharyngeal and oropharyngeal swab
hCoV-19/Mexico/TLA-IBT-IMSS-137/2021	EPI_ISL_1288153	10/02/21	North America / Mexico / Tlaxcala	Human	Female	48	Ambulatory	Nasopharyngeal and oropharyngeal swab
hCoV-19/Mexico/MOR-IBT-IMSS-370/2021	EPI_ISL_1288156	09/02/21	North America / Mexico / Morelos	Human	Male	84	Hospitalized	Nasopharyngeal and oropharyngeal swab
hCoV-19/Mexico/MOR-IBT-IMSS-381/2021	EPI_ISL_1288157	09/02/21	North America / Mexico / Morelos	Human	Female	58	Hospitalized	Nasopharyngeal and oropharyngeal swab
hCoV-19/Mexico/MOR-IBT-IMSS-392/2021	EPI_ISL_1288158	09/02/21	North America / Mexico / Morelos	Human	Male	33	Ambulatory	Nasopharyngeal and oropharyngeal swab
hCoV-19/Mexico/MOR-IBT-IMSS-403/2021	EPI_ISL_1288159	10/02/21	North America / Mexico / Morelos	Human	Male	50	Ambulatory	Nasopharyngeal and oropharyngeal swab
hCoV-19/Mexico/MOR-IBT-IMSS-414/2021	EPI_ISL_1288160	09/02/21	North America / Mexico / Morelos	Human	Male	77	Hospitalized	Nasopharyngeal and oropharyngeal swab
hCoV-19/Mexico/OAX-IBT-IMSS-59/2021	EPI_ISL_1288164	09/02/21	North America / Mexico / Oaxaca	Human	Female	26	Ambulatory	Nasopharyngeal and oropharyngeal swab
hCoV-19/Mexico/OAX-IBT-IMSS-114/2021	EPI_ISL_1288168	09/02/21	North America / Mexico / Oaxaca	Human	Female	22	Ambulatory	Nasopharyngeal and oropharyngeal swab

hCoV-19/Mexico/OAX-IBT-IMSS-125/2021	EPI_ISL_1288169	09/02/21	North America / Mexico / Oaxaca	Human	Male	38	Ambulatory	Nasopharyngeal and oropharyngeal swab
hCoV-19/Mexico/OAX-IBT-IMSS-136/2021	EPI_ISL_1288170	08/02/21	North America / Mexico / Oaxaca	Human	Female	30	Ambulatory	Nasopharyngeal and oropharyngeal swab
hCoV-19/Mexico/OAX-IBT-IMSS-148/2021	EPI_ISL_1288171	08/02/21	North America / Mexico / Oaxaca	Human	Male	46	Ambulatory	Nasopharyngeal and oropharyngeal swab
hCoV-19/Mexico/OAX-IBT-IMSS-159/2021	EPI_ISL_1288172	08/02/21	North America / Mexico / Oaxaca	Human	Female	68	Hospitalized	Nasopharyngeal and oropharyngeal swab
hCoV-19/Mexico/OAX-IBT-IMSS-170/2021	EPI_ISL_1288173	09/02/21	North America / Mexico / Oaxaca	Human	Male	30	Ambulatory	Nasopharyngeal and oropharyngeal swab
hCoV-19/Mexico/OAX-IBT-IMSS-171/2021	EPI_ISL_1288174	09/02/21	North America / Mexico / Oaxaca	Human	Female	30	Ambulatory	Nasopharyngeal and oropharyngeal swab
hCoV-19/Mexico/GRO-IBT-IMSS-193/2021	EPI_ISL_1288176	09/02/21	North America / Mexico / Guerrero	Human	Male	36	Ambulatory	Nasopharyngeal and oropharyngeal swab
hCoV-19/Mexico/GRO-IBT-IMSS-204/2021	EPI_ISL_1288177	09/02/21	North America / Mexico / Guerrero	Human	Male	29	Ambulatory	Nasopharyngeal and oropharyngeal swab
hCoV-19/Mexico/GRO-IBT-IMSS-215/2021	EPI_ISL_1288178	07/02/21	North America / Mexico / Guerrero	Human	Male	79	Hospitalized	Oropharyngeal swab
hCoV-19/Mexico/BCS-IBT-IMSS-282/2021	EPI_ISL_1288182	09/02/21	North America / Mexico / Baja California Sur	Human	Male	36	Ambulatory	Oropharyngeal swab
hCoV-19/Mexico/SLP-IBT-IMSS-303/2021	EPI_ISL_1288184	09/02/21	North America / Mexico / San Luis Potosi	Human	Male	71	Hospitalized	Nasopharyngeal and oropharyngeal swab
hCoV-19/Mexico/SLP-IBT-IMSS-314/2021	EPI_ISL_1288185	10/02/21	North America / Mexico / San Luis Potosi	Human	Male	57	Ambulatory	Nasopharyngeal and oropharyngeal swab
hCoV-19/Mexico/SLP-IBT-IMSS-325/2021	EPI_ISL_1288186	10/02/21	North America / Mexico / San Luis Potosi	Human	Female	30	Ambulatory	Nasopharyngeal and oropharyngeal swab
hCoV-19/Mexico/SLP-IBT-IMSS-336/2021	EPI_ISL_1288187	10/02/21	North America / Mexico / San Luis Potosi	Human	Male	21	Ambulatory	Nasopharyngeal and oropharyngeal swab
hCoV-19/Mexico/SLP-IBT-IMSS-347/2021	EPI_ISL_1288188	10/02/21	North America / Mexico / San Luis Potosi	Human	Male	32	Ambulatory	Nasopharyngeal and oropharyngeal swab
hCoV-19/Mexico/CMX-IBT-IMSS-359/2021	EPI_ISL_1288189	10/02/21	North America / Mexico / Mexico City	Human	Female	23	Ambulatory	Nasopharyngeal and oropharyngeal swab
hCoV-19/Mexico/CMX-IBT-IMSS-361/2021	EPI_ISL_1288190	10/02/21	North America / Mexico / Mexico City	Human	Female	27	Ambulatory	Nasopharyngeal and oropharyngeal swab
hCoV-19/Mexico/CMX-IBT-IMSS-362/2021	EPI_ISL_1288191	10/02/21	North America / Mexico / Mexico City	Human	Female	39	Ambulatory	Nasopharyngeal and oropharyngeal swab
hCoV-19/Mexico/CMX-IBT-IMSS-363/2021	EPI_ISL_1288192	11/02/21	North America / Mexico / Mexico City	Human	Female	62	Ambulatory	Nasopharyngeal and oropharyngeal swab
hCoV-19/Mexico/CMX-IBT-IMSS-364/2021	EPI_ISL_1288193	12/02/21	North America / Mexico / Mexico City	Human	Female	65	Ambulatory	Nasopharyngeal and oropharyngeal swab
hCoV-19/Mexico/CMX-IBT-IMSS-365/2021	EPI_ISL_1288194	12/02/21	North America / Mexico / Mexico City	Human	Female	64	Ambulatory	Nasopharyngeal and oropharyngeal swab
hCoV-19/Mexico/CMX-IBT-IMSS-366/2021	EPI_ISL_1288195	12/02/21	North America / Mexico / Mexico City	Human	Male	75	Ambulatory	Nasopharyngeal and oropharyngeal swab
hCoV-19/Mexico/CMX-IBT-IMSS-367/2021	EPI_ISL_1288196	11/02/21	North America / Mexico / Mexico City	Human	Male	39	Hospitalized	Oropharyngeal swab
hCoV-19/Mexico/CMX-IBT-IMSS-368/2021	EPI_ISL_1288197	11/02/21	North America / Mexico / Mexico City	Human	Male	74	Hospitalized	Oropharyngeal swab
hCoV-19/Mexico/MEX-IBT-IMSS-369/2021	EPI_ISL_1288198	11/02/21	North America / Mexico / State of Mexico	Human	Female	63	Hospitalized	Oropharyngeal swab
hCoV-19/Mexico/MEX-IBT-IMSS-371/2021	EPI_ISL_1288199	11/02/21	North America / Mexico / State of Mexico	Human	Male	72	Hospitalized	Oropharyngeal swab
hCoV-19/Mexico/CMX-IBT-IMSS-372/2021	EPI_ISL_1288200	11/02/21	North America / Mexico / Mexico City	Human	Male	69	Hospitalized	Oropharyngeal swab
hCoV-19/Mexico/QUE-IBT-IMSS-374/2021	EPI_ISL_1288202	10/02/21	North America / Mexico / Queretaro	Human	Female	37	Ambulatory	Nasopharyngeal and oropharyngeal swab
hCoV-19/Mexico/QUE-IBT-IMSS-375/2021	EPI_ISL_1288203	10/02/21	North America / Mexico / Queretaro	Human	Male	61	Ambulatory	Nasopharyngeal and oropharyngeal swab
hCoV-19/Mexico/QUE-IBT-IMSS-376/2021	EPI_ISL_1288204	10/02/21	North America / Mexico / Queretaro	Human	Female	65	Ambulatory	Nasopharyngeal and oropharyngeal swab
hCoV-19/Mexico/MEX-IBT-IMSS-377/2021	EPI_ISL_1288205	11/02/21	North America / Mexico / State of Mexico	Human	Male	41	Ambulatory	Nasopharyngeal and oropharyngeal swab
hCoV-19/Mexico/MEX-IBT-IMSS-378/2021	EPI_ISL_1288206	11/02/21	North America / Mexico / State of Mexico	Human	Female	40	Ambulatory	Nasopharyngeal and oropharyngeal swab
hCoV-19/Mexico/MEX-IBT-IMSS-379/2021	EPI_ISL_1288207	10/02/21	North America / Mexico / State of Mexico	Human	Female	67	Ambulatory	Nasopharyngeal swab
hCoV-19/Mexico/MEX-IBT-IMSS-380/2021	EPI_ISL_1288208	10/02/21	North America / Mexico / State of Mexico	Human	Male	6	Ambulatory	Nasopharyngeal swab
hCoV-19/Mexico/MEX-IBT-IMSS-382/2021	EPI_ISL_1288209	11/02/21	North America / Mexico / State of Mexico	Human	Female	68	Ambulatory	Nasopharyngeal and oropharyngeal swab
hCoV-19/Mexico/MEX-IBT-IMSS-383/2021	EPI_ISL_1288210	10/02/21	North America / Mexico / State of Mexico	Human	Male	54	Ambulatory	Oropharyngeal swab
hCoV-19/Mexico/CMX-IBT-IMSS-384/2021	EPI_ISL_1288211	11/02/21	North America / Mexico / Mexico City	Human	Male	44	Ambulatory	Nasopharyngeal and oropharyngeal swab
hCoV-19/Mexico/CMX-IBT-IMSS-385/2021	EPI_ISL_1288212	12/02/21	North America / Mexico / Mexico City	Human	Male	68	Hospitalized	Nasopharyngeal and oropharyngeal swab
hCoV-19/Mexico/MOR-IBT-IMSS-386/2021	EPI_ISL_1288213	08/02/21	North America / Mexico / Morelos	Human	Male	76	Hospitalized	Nasopharyngeal and oropharyngeal swab
hCoV-19/Mexico/MOR-IBT-IMSS-387/2021	EPI_ISL_1288214	08/02/21	North America / Mexico / Morelos	Human	Female	79	Hospitalized	Nasopharyngeal and oropharyngeal swab
hCoV-19/Mexico/MOR-IBT-IMSS-388/2021	EPI_ISL_1288215	08/02/21	North America / Mexico / Morelos	Human	Female	67	OSPITALIZAD	Nasopharyngeal and oropharyngeal swab
hCoV-19/Mexico/MOR-IBT-IMSS-389/2021	EPI_ISL_1288216	09/02/21	North America / Mexico / Morelos	Human	Male	67	Ambulatory	Nasopharyngeal and oropharyngeal swab
hCoV-19/Mexico/MOR-IBT-IMSS-390/2021	EPI_ISL_1288217	09/02/21	North America / Mexico / Morelos	Human	Male	33	Hospitalized	Nasopharyngeal and oropharyngeal swab
hCoV-19/Mexico/GRO-IBT-IMSS-391/2021	EPI_ISL_1288218	10/02/21	North America / Mexico / Guerrero	Human	Male	75	Hospitalized	Nasopharyngeal and oropharyngeal swab

hCoV-19/Mexico/GRO-IBT-IMSS-393/2021	EPI_ISL_1288219	11/02/21	North America / Mexico / Guerrero	Human	Male	50	Hospitalized	Nasopharyngeal and oropharyngeal swab
hCoV-19/Mexico/GRO-IBT-IMSS-394/2021	EPI_ISL_1288220	09/02/21	North America / Mexico / Guerrero	Human	Male	46	Ambulatory	Nasopharyngeal and oropharyngeal swab
hCoV-19/Mexico/GRO-IBT-IMSS-395/2021	EPI_ISL_1288221	09/02/21	North America / Mexico / Guerrero	Human	Female	35	Ambulatory	Nasopharyngeal and oropharyngeal swab
hCoV-19/Mexico/GRO-IBT-IMSS-396/2021	EPI_ISL_1288222	10/02/21	North America / Mexico / Guerrero	Human	Male	63	Hospitalized	Nasopharyngeal and oropharyngeal swab
hCoV-19/Mexico/GRO-IBT-IMSS-404/2021	EPI_ISL_1288226	09/02/21	North America / Mexico / Guerrero	Human	Female	38	Ambulatory	Nasopharyngeal and oropharyngeal swab
hCoV-19/Mexico/GRO-IBT-IMSS-405/2021	EPI_ISL_1288227	09/02/21	North America / Mexico / Guerrero	Human	Female	70	Ambulatory	Nasopharyngeal and oropharyngeal swab
hCoV-19/Mexico/GRO-IBT-IMSS-406/2021	EPI_ISL_1288228	09/02/21	North America / Mexico / Guerrero	Human	Female	53	Ambulatory	Nasopharyngeal and oropharyngeal swab
hCoV-19/Mexico/GRO-IBT-IMSS-411/2021	EPI_ISL_1288233	09/02/21	North America / Mexico / Guerrero	Human	Female	58	Ambulatory	Nasopharyngeal and oropharyngeal swab
hCoV-19/Mexico/GRO-IBT-IMSS-412/2021	EPI_ISL_1288234	09/02/21	North America / Mexico / Guerrero	Human	Female	46	Ambulatory	Nasopharyngeal and oropharyngeal swab
hCoV-19/Mexico/GRO-IBT-IMSS-413/2021	EPI_ISL_1288235	09/02/21	North America / Mexico / Guerrero	Human	Male	71	Ambulatory	Nasopharyngeal and oropharyngeal swab
hCoV-19/Mexico/GRO-IBT-IMSS-415/2021	EPI_ISL_1288236	09/02/21	North America / Mexico / Guerrero	Human	Female	46	Ambulatory	Nasopharyngeal and oropharyngeal swab
hCoV-19/Mexico/GRO-IBT-IMSS-417/2021	EPI_ISL_1288238	09/02/21	North America / Mexico / Guerrero	Human	Male	30	Ambulatory	Nasopharyngeal and oropharyngeal swab
hCoV-19/Mexico/VER-IBT-IMSS-418/2021	EPI_ISL_1288239	10/02/21	North America / Mexico / Veracruz	Human	Female	62	Hospitalized	Nasopharyngeal and oropharyngeal swab
hCoV-19/Mexico/VER-IBT-IMSS-421/2021	EPI_ISL_1288242	10/02/21	North America / Mexico / Veracruz	Human	Female	67	Ambulatory	Nasopharyngeal and oropharyngeal swab
hCoV-19/Mexico/ROO-IBT-IMSS-422/2021	EPI_ISL_1288243	10/02/21	North America / Mexico / Quintana Roo	Human	Female	61	Hospitalized	Nasopharyngeal and oropharyngeal swab
hCoV-19/Mexico/MOR-IBT-IMSS-27/2021	EPI_ISL_1288246	10/02/21	North America / Mexico / Morelos	Human	Male	46	Hospitalized	Nasopharyngeal and oropharyngeal swab
hCoV-19/Mexico/HID-IBT-IMSS-28/2021	EPI_ISL_1288247	12/02/21	North America / Mexico / Hidalgo	Human	Female	63	Hospitalized	Oropharyngeal swab
hCoV-19/Mexico/MEX-IBT-IMSS-29/2021	EPI_ISL_1288248	12/02/21	North America / Mexico / State of Mexico	Human	Male	53	Hospitalized	Nasopharyngeal and oropharyngeal swab
hCoV-19/Mexico/MEX-IBT-IMSS-30/2021	EPI_ISL_1288249	11/02/21	North America / Mexico / State of Mexico	Human	Male	31	Ambulatory	Nasopharyngeal and oropharyngeal swab
hCoV-19/Mexico/TLA-IBT-IMSS-31/2021	EPI_ISL_1288250	11/02/21	North America / Mexico / Tlaxcala	Human	Male	47	Hospitalized	Nasopharyngeal and oropharyngeal swab
hCoV-19/Mexico/TLA-IBT-IMSS-32/2021	EPI_ISL_1288251	11/02/21	North America / Mexico / Tlaxcala	Human	Male	69	Hospitalized	Nasopharyngeal and oropharyngeal swab
hCoV-19/Mexico/TLA-IBT-IMSS-33/2021	EPI_ISL_1288252	11/02/21	North America / Mexico / Tlaxcala	Human	Male	48	Hospitalized	Nasopharyngeal and oropharyngeal swab
hCoV-19/Mexico/TLA-IBT-IMSS-34/2021	EPI_ISL_1288253	12/02/21	North America / Mexico / Tlaxcala	Human	Male	32	Ambulatory	Nasopharyngeal and oropharyngeal swab
hCoV-19/Mexico/PUE-IBT-IMSS-35/2021	EPI_ISL_1288254	11/02/21	North America / Mexico / Puebla	Human	Male	58	Ambulatory	Nasopharyngeal and oropharyngeal swab
hCoV-19/Mexico/PUE-IBT-IMSS-36/2021	EPI_ISL_1288255	10/02/21	North America / Mexico / Puebla	Human	Female	9	Ambulatory	Nasopharyngeal and oropharyngeal swab
hCoV-19/Mexico/PUE-IBT-IMSS-38/2021	EPI_ISL_1288256	11/02/21	North America / Mexico / Puebla	Human	Female	54	Ambulatory	Nasopharyngeal and oropharyngeal swab
hCoV-19/Mexico/PUE-IBT-IMSS-39/2021	EPI_ISL_1288257	11/02/21	North America / Mexico / Puebla	Human	Male	45	Ambulatory	Nasopharyngeal and oropharyngeal swab
hCoV-19/Mexico/PUE-IBT-IMSS-40/2021	EPI_ISL_1288258	11/02/21	North America / Mexico / Puebla	Human	Male	44	Ambulatory	Nasopharyngeal and oropharyngeal swab
hCoV-19/Mexico/PUE-IBT-IMSS-41/2021	EPI_ISL_1288259	11/02/21	North America / Mexico / Puebla	Human	Male	50	Ambulatory	Nasopharyngeal swab
hCoV-19/Mexico/PUE-IBT-IMSS-42/2021	EPI_ISL_1288260	11/02/21	North America / Mexico / Puebla	Human	Male	58	Ambulatory	Nasopharyngeal swab
hCoV-19/Mexico/PUE-IBT-IMSS-43/2021	EPI_ISL_1288261	11/02/21	North America / Mexico / Puebla	Human	Female	41	Ambulatory	Nasopharyngeal swab
hCoV-19/Mexico/PUE-IBT-IMSS-45/2021	EPI_ISL_1288263	11/02/21	North America / Mexico / Puebla	Human	Female	31	Ambulatory	Nasopharyngeal swab
hCoV-19/Mexico/PUE-IBT-IMSS-46/2021	EPI_ISL_1288264	11/02/21	North America / Mexico / Puebla	Human	Female	28	Ambulatory	Nasopharyngeal swab
hCoV-19/Mexico/PUE-IBT-IMSS-47/2021	EPI_ISL_1288265	10/02/21	North America / Mexico / Puebla	Human	Male	58	Hospitalized	Nasopharyngeal and oropharyngeal swab
hCoV-19/Mexico/PUE-IBT-IMSS-49/2021	EPI_ISL_1288266	10/02/21	North America / Mexico / Puebla	Human	Male	74	Hospitalized	Nasopharyngeal and oropharyngeal swab
hCoV-19/Mexico/PUE-IBT-IMSS-50/2021	EPI_ISL_1288267	11/02/21	North America / Mexico / Puebla	Human	Female	88	Ambulatory	Nasopharyngeal swab
hCoV-19/Mexico/PUE-IBT-IMSS-51/2021	EPI_ISL_1288268	10/02/21	North America / Mexico / Puebla	Human	Male	68	Ambulatory	Nasopharyngeal and oropharyngeal swab
hCoV-19/Mexico/PUE-IBT-IMSS-52/2021	EPI_ISL_1288269	11/02/21	North America / Mexico / Puebla	Human	Female	53	Ambulatory	Oropharyngeal swab
hCoV-19/Mexico/PUE-IBT-IMSS-53/2021	EPI_ISL_1288270	11/02/21	North America / Mexico / Puebla	Human	Female	26	Ambulatory	Oropharyngeal swab
hCoV-19/Mexico/PUE-IBT-IMSS-54/2021	EPI_ISL_1288271	11/02/21	North America / Mexico / Puebla	Human	Female	46	Ambulatory	Oropharyngeal swab
hCoV-19/Mexico/PUE-IBT-IMSS-55/2021	EPI_ISL_1288272	11/02/21	North America / Mexico / Puebla	Human	Female	34	Ambulatory	Nasopharyngeal and oropharyngeal swab
hCoV-19/Mexico/PUE-IBT-IMSS-56/2021	EPI_ISL_1288273	10/02/21	North America / Mexico / Puebla	Human	Male	47	Ambulatory	Nasopharyngeal and oropharyngeal swab
hCoV-19/Mexico/PUE-IBT-IMSS-57/2021	EPI_ISL_1288274	11/02/21	North America / Mexico / Puebla	Human	Female	33	Ambulatory	Nasopharyngeal and oropharyngeal swab
hCoV-19/Mexico/PUE-IBT-IMSS-58/2021	EPI_ISL_1288275	10/02/21	North America / Mexico / Puebla	Human	Female	38	Ambulatory	Nasopharyngeal and oropharyngeal swab

hCoV-19/Mexico/PUE-IBT-IMSS-60/2021	EPI_ISL_1288276	10/02/21	North America / Mexico / Puebla	Human	Female	67	Hospitalized	Nasopharyngeal and oropharyngeal swab
hCoV-19/Mexico/PUE-IBT-IMSS-61/2021	EPI_ISL_1288277	08/02/21	North America / Mexico / Puebla	Human	Female	69	Ambulatory	Nasopharyngeal and oropharyngeal swab
hCoV-19/Mexico/SLP-IBT-IMSS-68/2021	EPI_ISL_1288284	11/02/21	North America / Mexico / San Luis Potosi	Human	Female	46	Ambulatory	Nasopharyngeal and oropharyngeal swab
hCoV-19/Mexico/SLP-IBT-IMSS-69/2021	EPI_ISL_1288285	12/02/21	North America / Mexico / San Luis Potosi	Human	Female	25	Ambulatory	Nasopharyngeal and oropharyngeal swab
hCoV-19/Mexico/SLP-IBT-IMSS-71/2021	EPI_ISL_1288286	12/02/21	North America / Mexico / San Luis Potosi	Human	Male	86	Ambulatory	Nasopharyngeal and oropharyngeal swab
hCoV-19/Mexico/SLP-IBT-IMSS-73/2021	EPI_ISL_1288288	10/02/21	North America / Mexico / San Luis Potosi	Human	Female	76	Hospitalized	Nasopharyngeal and oropharyngeal swab
hCoV-19/Mexico/SLP-IBT-IMSS-74/2021	EPI_ISL_1288289	10/02/21	North America / Mexico / San Luis Potosi	Human	Male	42	Hospitalized	Nasopharyngeal and oropharyngeal swab
hCoV-19/Mexico/SLP-IBT-IMSS-75/2021	EPI_ISL_1288290	10/02/21	North America / Mexico / San Luis Potosi	Human	Female	39	Ambulatory	Nasopharyngeal and oropharyngeal swab
hCoV-19/Mexico/SLP-IBT-IMSS-76/2021	EPI_ISL_1288291	12/02/21	North America / Mexico / San Luis Potosi	Human	Female	54	Ambulatory	Nasopharyngeal and oropharyngeal swab
hCoV-19/Mexico/SLP-IBT-IMSS-77/2021	EPI_ISL_1288292	12/02/21	North America / Mexico / San Luis Potosi	Human	Female	45	Ambulatory	Nasopharyngeal and oropharyngeal swab
hCoV-19/Mexico/SLP-IBT-IMSS-78/2021	EPI_ISL_1288293	10/02/21	North America / Mexico / San Luis Potosi	Human	Male	34	Ambulatory	Nasopharyngeal and oropharyngeal swab
hCoV-19/Mexico/VER-IBT-IMSS-79/2021	EPI_ISL_1288294	10/02/21	North America / Mexico / Veracruz	Human	Female	62	Hospitalized	Nasopharyngeal and oropharyngeal swab
hCoV-19/Mexico/VER-IBT-IMSS-80/2021	EPI_ISL_1288295	08/02/21	North America / Mexico / Veracruz	Human	Male	66	Hospitalized	Nasopharyngeal and oropharyngeal swab
hCoV-19/Mexico/VER-IBT-IMSS-82/2021	EPI_ISL_1288296	10/02/21	North America / Mexico / Veracruz	Human	Female	47	Ambulatory	Nasopharyngeal and oropharyngeal swab
hCoV-19/Mexico/VER-IBT-IMSS-85/2021	EPI_ISL_1288299	10/02/21	North America / Mexico / Veracruz	Human	Male	55	Ambulatory	Nasopharyngeal swab
hCoV-19/Mexico/VER-IBT-IMSS-86/2021	EPI_ISL_1288300	10/02/21	North America / Mexico / Veracruz	Human	Female	68	Ambulatory	Nasopharyngeal swab
hCoV-19/Mexico/VER-IBT-IMSS-87/2021	EPI_ISL_1288301	10/02/21	North America / Mexico / Veracruz	Human	Female	43	Ambulatory	Nasopharyngeal swab
hCoV-19/Mexico/VER-IBT-IMSS-89/2021	EPI_ISL_1288303	10/02/21	North America / Mexico / Veracruz	Human	Male	32	Ambulatory	Nasopharyngeal and oropharyngeal swab
hCoV-19/Mexico/VER-IBT-IMSS-90/2021	EPI_ISL_1288304	10/02/21	North America / Mexico / Veracruz	Human	Male	44	Ambulatory	Nasopharyngeal and oropharyngeal swab
hCoV-19/Mexico/VER-IBT-IMSS-91/2021	EPI_ISL_1288305	10/02/21	North America / Mexico / Veracruz	Human	Male	40	Ambulatory	Nasopharyngeal and oropharyngeal swab
hCoV-19/Mexico/VER-IBT-IMSS-93/2021	EPI_ISL_1288306	10/02/21	North America / Mexico / Veracruz	Human	Male	20	Ambulatory	Nasopharyngeal and oropharyngeal swab
hCoV-19/Mexico/CHP-IBT-IMSS-95/2021	EPI_ISL_1288307	11/02/21	North America / Mexico / Chiapas	Human	Female	47	Ambulatory	Nasopharyngeal and oropharyngeal swab
hCoV-19/Mexico/BCS-IBT-IMSS-97/2021	EPI_ISL_1288309	08/02/21	North America / Mexico / Baja California Sur	Human	Female	37	Ambulatory	Nasopharyngeal and oropharyngeal swab
hCoV-19/Mexico/VER-IBT-IMSS-100/2021	EPI_ISL_1288311	10/02/21	North America / Mexico / Veracruz	Human	Female	72	Hospitalized	Nasopharyngeal and oropharyngeal swab
hCoV-19/Mexico/BCS-IBT-IMSS-102/2021	EPI_ISL_1288313	11/02/21	North America / Mexico / Baja California Sur	Human	Male	52	Ambulatory	Nasopharyngeal and oropharyngeal swab
hCoV-19/Mexico/VER-IBT-IMSS-105/2021	EPI_ISL_1288315	10/02/21	North America / Mexico / Veracruz	Human	Male	77	Ambulatory	Nasopharyngeal and oropharyngeal swab
hCoV-19/Mexico/BCS-IBT-IMSS-106/2021	EPI_ISL_1288316	11/02/21	North America / Mexico / Baja California Sur	Human	Male	60	Ambulatory	Nasopharyngeal and oropharyngeal swab
hCoV-19/Mexico/BCS-IBT-IMSS-109/2021	EPI_ISL_1288318	11/02/21	North America / Mexico / Baja California Sur	Human	Male	35	Ambulatory	Nasopharyngeal and oropharyngeal swab
hCoV-19/Mexico/VER-IBT-IMSS-110/2021	EPI_ISL_1288319	10/02/21	North America / Mexico / Veracruz	Human	Female	35	Ambulatory	Nasopharyngeal and oropharyngeal swab
hCoV-19/Mexico/VER-IBT-IMSS-112/2021	EPI_ISL_1288321	10/02/21	North America / Mexico / Veracruz	Human	Male	48	Ambulatory	Nasopharyngeal and oropharyngeal swab
hCoV-19/Mexico/VER-IBT-IMSS-113/2021	EPI_ISL_1288322	10/02/21	North America / Mexico / Veracruz	Human	Male	33	Ambulatory	Nasopharyngeal and oropharyngeal swab
hCoV-19/Mexico/VER-IBT-IMSS-115/2021	EPI_ISL_1288323	10/02/21	North America / Mexico / Veracruz	Human	Male	43	Ambulatory	Nasopharyngeal and oropharyngeal swab
hCoV-19/Mexico/VER-IBT-IMSS-116/2021	EPI_ISL_1288324	10/02/21	North America / Mexico / Veracruz	Human	Male	80	Ambulatory	Nasopharyngeal and oropharyngeal swab
hCoV-19/Mexico/VER-IBT-IMSS-117/2021	EPI_ISL_1288325	10/02/21	North America / Mexico / Veracruz	Human	Female	23	Ambulatory	Nasopharyngeal and oropharyngeal swab
hCoV-19/Mexico/CHP-IBT-IMSS-118/2021	EPI_ISL_1288326	10/02/21	North America / Mexico / Chiapas	Human	Male	44	Ambulatory	Nasopharyngeal and oropharyngeal swab
hCoV-19/Mexico/CHP-IBT-IMSS-119/2021	EPI_ISL_1288327	11/02/21	North America / Mexico / Chiapas	Human	Female	57	Ambulatory	Nasopharyngeal and oropharyngeal swab
hCoV-19/Mexico/PUE-IBT-IMSS-120/2021	EPI_ISL_1288328	10/02/21	North America / Mexico / Puebla	Human	Female	38	Ambulatory	Nasopharyngeal and oropharyngeal swab
hCoV-19/Mexico/CHP-IBT-IMSS-122/2021	EPI_ISL_1288329	11/02/21	North America / Mexico / Chiapas	Human	Male	23	Ambulatory	Nasopharyngeal and oropharyngeal swab
hCoV-19/Mexico/CHP-IBT-IMSS-123/2021	EPI_ISL_1288330	10/02/21	North America / Mexico / Chiapas	Human	Female	28	Ambulatory	Nasopharyngeal and oropharyngeal swab
hCoV-19/Mexico/PUE-IBT-IMSS-124/2021	EPI_ISL_1288331	11/02/21	North America / Mexico / Puebla	Human	Male	54	Ambulatory	Nasopharyngeal swab
hCoV-19/Mexico/PUE-IBT-IMSS-126/2021	EPI_ISL_1288332	10/02/21	North America / Mexico / Puebla	Human	Male	33	Ambulatory	Nasopharyngeal and oropharyngeal swab
hCoV-19/Mexico/PUE-IBT-IMSS-127/2021	EPI_ISL_1288333	11/02/21	North America / Mexico / Puebla	Human	Male	39	Ambulatory	Nasopharyngeal swab
hCoV-19/Mexico/PUE-IBT-IMSS-128/2021	EPI_ISL_1288334	11/02/21	North America / Mexico / Puebla	Human	Female	19	Ambulatory	Nasopharyngeal and oropharyngeal swab
hCoV-19/Mexico/PUE-IBT-IMSS-129/2021	EPI_ISL_1288335	10/02/21	North America / Mexico / Puebla	Human	Male	29	Ambulatory	Nasopharyngeal and oropharyngeal swab

hCoV-19/Mexico/PUE-IBT-IMSS-130/2021	EPI_ISL_1288336	10/02/21	North America / Mexico / Puebla	Human	Female	45	Ambulatory	Nasopharyngeal and oropharyngeal swab
hCoV-19/Mexico/PUE-IBT-IMSS-131/2021	EPI_ISL_1288337	11/02/21	North America / Mexico / Puebla	Human	Female	30	Ambulatory	Nasopharyngeal and oropharyngeal swab
hCoV-19/Mexico/PUE-IBT-IMSS-132/2021	EPI_ISL_1288338	11/02/21	North America / Mexico / Puebla	Human	Female	67	Ambulatory	Nasopharyngeal swab
hCoV-19/Mexico/PUE-IBT-IMSS-134/2021	EPI_ISL_1288340	11/02/21	North America / Mexico / Puebla	Human	Female	57	Ambulatory	Nasopharyngeal swab
hCoV-19/Mexico/PUE-IBT-IMSS-138/2021	EPI_ISL_1288341	10/02/21	North America / Mexico / Puebla	Human	Female	29	Ambulatory	Nasopharyngeal and oropharyngeal swab
hCoV-19/Mexico/PUE-IBT-IMSS-139/2021	EPI_ISL_1288342	10/02/21	North America / Mexico / Puebla	Human	Male	44	Ambulatory	Oropharyngeal swab
hCoV-19/Mexico/PUE-IBT-IMSS-140/2021	EPI_ISL_1288343	10/02/21	North America / Mexico / Puebla	Human	Male	37	Ambulatory	Nasopharyngeal and oropharyngeal swab
hCoV-19/Mexico/PUE-IBT-IMSS-141/2021	EPI_ISL_1288344	10/02/21	North America / Mexico / Puebla	Human	Female	37	Ambulatory	Nasopharyngeal and oropharyngeal swab
hCoV-19/Mexico/PUE-IBT-IMSS-142/2021	EPI_ISL_1288345	11/02/21	North America / Mexico / Puebla	Human	Female	52	Ambulatory	Nasopharyngeal and oropharyngeal swab
hCoV-19/Mexico/PUE-IBT-IMSS-144/2021	EPI_ISL_1288347	10/02/21	North America / Mexico / Puebla	Human	Male	62	Ambulatory	Nasopharyngeal and oropharyngeal swab
hCoV-19/Mexico/CMX-IBT-IMSS-147/2021	EPI_ISL_1288350	12/02/21	North America / Mexico / Mexico City	Human	Female	46	Ambulatory	Nasopharyngeal and oropharyngeal swab
hCoV-19/Mexico/VER-IBT-IMSS-150/2021	EPI_ISL_1288352	12/02/21	North America / Mexico / Veracruz	Human	Female	35	Ambulatory	Nasopharyngeal and oropharyngeal swab
hCoV-19/Mexico/OAX-IBT-IMSS-152/2021	EPI_ISL_1288354	11/02/21	North America / Mexico / Oaxaca	Human	Male	32	Ambulatory	Nasopharyngeal and oropharyngeal swab
hCoV-19/Mexico/VER-IBT-IMSS-153/2021	EPI_ISL_1288355	11/02/21	North America / Mexico / Veracruz	Human	Female	43	Ambulatory	Nasopharyngeal and oropharyngeal swab
hCoV-19/Mexico/GRO-IBT-IMSS-154/2021	EPI_ISL_1288356	11/02/21	North America / Mexico / Guerrero	Human	Female	60	Hospitalized	Nasopharyngeal and oropharyngeal swab
hCoV-19/Mexico/GRO-IBT-IMSS-155/2021	EPI_ISL_1288357	10/02/21	North America / Mexico / Guerrero	Human	Male	52	Ambulatory	Oropharyngeal swab
hCoV-19/Mexico/VER-IBT-IMSS-156/2021	EPI_ISL_1288358	12/02/21	North America / Mexico / Veracruz	Human	Male	53	Hospitalized	Nasopharyngeal and oropharyngeal swab
hCoV-19/Mexico/VER-IBT-IMSS-157/2021	EPI_ISL_1288359	12/02/21	North America / Mexico / Veracruz	Human	Female	65	Hospitalized	Nasopharyngeal and oropharyngeal swab
hCoV-19/Mexico/VER-IBT-IMSS-158/2021	EPI_ISL_1288360	11/02/21	North America / Mexico / Veracruz	Human	Male	31	Ambulatory	Nasopharyngeal and oropharyngeal swab
hCoV-19/Mexico/GRO-IBT-IMSS-161/2021	EPI_ISL_1288362	11/02/21	North America / Mexico / Guerrero	Human	Female	25	Ambulatory	Nasopharyngeal and oropharyngeal swab
hCoV-19/Mexico/VER-IBT-IMSS-162/2021	EPI_ISL_1288363	11/02/21	North America / Mexico / Veracruz	Human	Male	34	Ambulatory	Nasopharyngeal and oropharyngeal swab
hCoV-19/Mexico/VER-IBT-IMSS-163/2021	EPI_ISL_1288364	11/02/21	North America / Mexico / Veracruz	Human	Female	39	Ambulatory	Nasopharyngeal and oropharyngeal swab
hCoV-19/Mexico/MEX-IBT-IMSS-164/2021	EPI_ISL_1288365	11/02/21	North America / Mexico / State of Mexico	Human	Male	81	Hospitalized	Nasopharyngeal and oropharyngeal swab
hCoV-19/Mexico/MEX-IBT-IMSS-165/2021	EPI_ISL_1288366	11/02/21	North America / Mexico / State of Mexico	Human	Male	43	Hospitalized	Nasopharyngeal and oropharyngeal swab
hCoV-19/Mexico/MEX-IBT-IMSS-166/2021	EPI_ISL_1288367	11/02/21	North America / Mexico / State of Mexico	Human	Male	55	Hospitalized	Nasopharyngeal and oropharyngeal swab
hCoV-19/Mexico/MEX-IBT-IMSS-167/2021	EPI_ISL_1288368	11/02/21	North America / Mexico / State of Mexico	Human	Male	61	Hospitalized	Nasopharyngeal and oropharyngeal swab
hCoV-19/Mexico/MOR-IBT-IMSS-168/2021	EPI_ISL_1288369	25/02/21	North America / Mexico / Morelos	Human	Male	72	Ambulatory	Nasopharyngeal and oropharyngeal swab
hCoV-19/Mexico/MEX-IBT-IMSS-169/2021	EPI_ISL_1288370	12/02/21	North America / Mexico / State of Mexico	Human	Male	65	Hospitalized	Nasopharyngeal and oropharyngeal swab
hCoV-19/Mexico/MOR-IBT-IMSS-173/2021	EPI_ISL_1288372	12/02/21	North America / Mexico / Morelos	Human	Male	57	Hospitalized	Nasopharyngeal and oropharyngeal swab
hCoV-19/Mexico/MOR-IBT-IMSS-174/2021	EPI_ISL_1288373	11/02/21	North America / Mexico / Morelos	Human	Male	69	Hospitalized	Nasopharyngeal and oropharyngeal swab
hCoV-19/Mexico/MOR-IBT-IMSS-176/2021	EPI_ISL_1288375	11/02/21	North America / Mexico / Morelos	Human	Female	73	Ambulatory	Nasopharyngeal and oropharyngeal swab
hCoV-19/Mexico/MEX-IBT-IMSS-177/2021	EPI_ISL_1288376	10/02/21	North America / Mexico / State of Mexico	Human	Female	44	Ambulatory	Nasopharyngeal and oropharyngeal swab
hCoV-19/Mexico/MEX-IBT-IMSS-178/2021	EPI_ISL_1288377	10/02/21	North America / Mexico / State of Mexico	Human	Male	24	Ambulatory	Nasopharyngeal and oropharyngeal swab
hCoV-19/Mexico/MEX-IBT-IMSS-179/2021	EPI_ISL_1288378	10/02/21	North America / Mexico / State of Mexico	Human	Female	18	Ambulatory	Nasopharyngeal and oropharyngeal swab
hCoV-19/Mexico/MEX-IBT-IMSS-180/2021	EPI_ISL_1288379	10/02/21	North America / Mexico / State of Mexico	Human	Female	24	Ambulatory	Nasopharyngeal and oropharyngeal swab
hCoV-19/Mexico/MEX-IBT-IMSS-181/2021	EPI_ISL_1288380	10/02/21	North America / Mexico / State of Mexico	Human	Female	28	Ambulatory	Nasopharyngeal and oropharyngeal swab
hCoV-19/Mexico/MEX-IBT-IMSS-183/2021	EPI_ISL_1288381	10/02/21	North America / Mexico / State of Mexico	Human	Male	42	Ambulatory	Nasopharyngeal and oropharyngeal swab
hCoV-19/Mexico/MEX-IBT-IMSS-184/2021	EPI_ISL_1288382	11/02/21	North America / Mexico / State of Mexico	Human	Female	12	Ambulatory	Nasopharyngeal and oropharyngeal swab
hCoV-19/Mexico/MEX-IBT-IMSS-185/2021	EPI_ISL_1288383	11/02/21	North America / Mexico / State of Mexico	Human	Female	49	Ambulatory	Nasopharyngeal and oropharyngeal swab
hCoV-19/Mexico/MEX-IBT-IMSS-186/2021	EPI_ISL_1288384	11/02/21	North America / Mexico / State of Mexico	Human	Male	71	Hospitalized	Nasopharyngeal and oropharyngeal swab
hCoV-19/Mexico/MEX-IBT-IMSS-188/2021	EPI_ISL_1288386	11/02/21	North America / Mexico / State of Mexico	Human	Male	47	Ambulatory	Nasopharyngeal and oropharyngeal swab
hCoV-19/Mexico/MEX-IBT-IMSS-197/2021	EPI_ISL_1288394	12/02/21	North America / Mexico / State of Mexico	Human	Female	68	Hospitalized	Nasopharyngeal and oropharyngeal swab
hCoV-19/Mexico/MEX-IBT-IMSS-198/2021	EPI_ISL_1288395	12/02/21	North America / Mexico / State of Mexico	Human	Male	55	Hospitalized	Nasopharyngeal and oropharyngeal swab
hCoV-19/Mexico/MEX-IBT-IMSS-199/2021	EPI_ISL_1288396	12/02/21	North America / Mexico / State of Mexico	Human	Female	71	Hospitalized	Nasopharyngeal and oropharyngeal swab

hCoV-19/Mexico/MEX-IBT-IMSS-201/2021	EPI_ISL_1288398	12/02/21	North America / Mexico / State of Mexico	Human	Male	69	Hospitalized	Nasopharyngeal and oropharyngeal swab
hCoV-19/Mexico/MEX-IBT-IMSS-202/2021	EPI_ISL_1288399	12/02/21	North America / Mexico / State of Mexico	Human	Female	52	Hospitalized	Nasopharyngeal and oropharyngeal swab
hCoV-19/Mexico/MEX-IBT-IMSS-203/2021	EPI_ISL_1288400	11/02/21	North America / Mexico / State of Mexico	Human	Female	32	Ambulatory	Nasopharyngeal and oropharyngeal swab
hCoV-19/Mexico/MEX-IBT-IMSS-205/2021	EPI_ISL_1288401	11/02/21	North America / Mexico / State of Mexico	Human	Male	59	Hospitalized	Nasopharyngeal and oropharyngeal swab
hCoV-19/Mexico/MEX-IBT-IMSS-206/2021	EPI_ISL_1288402	11/02/21	North America / Mexico / State of Mexico	Human	Male	49	Ambulatory	Nasopharyngeal and oropharyngeal swab
hCoV-19/Mexico/MEX-IBT-IMSS-207/2021	EPI_ISL_1288403	11/02/21	North America / Mexico / State of Mexico	Human	Female	66	Hospitalized	Nasopharyngeal and oropharyngeal swab
hCoV-19/Mexico/MEX-IBT-IMSS-208/2021	EPI_ISL_1288404	11/02/21	North America / Mexico / State of Mexico	Human	Female	57	Ambulatory	Nasopharyngeal and oropharyngeal swab
hCoV-19/Mexico/MEX-IBT-IMSS-209/2021	EPI_ISL_1288405	11/02/21	North America / Mexico / State of Mexico	Human	Female	22	Ambulatory	Nasopharyngeal and oropharyngeal swab
hCoV-19/Mexico/MEX-IBT-IMSS-210/2021	EPI_ISL_1288406	11/02/21	North America / Mexico / State of Mexico	Human	Male	49	Ambulatory	Nasopharyngeal and oropharyngeal swab
hCoV-19/Mexico/MEX-IBT-IMSS-211/2021	EPI_ISL_1288407	11/02/21	North America / Mexico / State of Mexico	Human	Female	39	Ambulatory	Nasopharyngeal and oropharyngeal swab
hCoV-19/Mexico/MEX-IBT-IMSS-212/2021	EPI_ISL_1288408	11/02/21	North America / Mexico / State of Mexico	Human	Male	39	Ambulatory	Nasopharyngeal and oropharyngeal swab
hCoV-19/Mexico/MEX-IBT-IMSS-213/2021	EPI_ISL_1288409	10/02/21	North America / Mexico / State of Mexico	Human	Male	28	Ambulatory	Nasopharyngeal and oropharyngeal swab
hCoV-19/Mexico/MEX-IBT-IMSS-214/2021	EPI_ISL_1288410	06/02/21	North America / Mexico / State of Mexico	Human	Male	73	Hospitalized	Nasopharyngeal and oropharyngeal swab
hCoV-19/Mexico/CMX-IBT-IMSS-216/2021	EPI_ISL_1288411	03/02/21	North America / Mexico / Mexico City	Human	Male	58	Hospitalized	Nasopharyngeal and oropharyngeal swab
hCoV-19/Mexico/CMX-IBT-IMSS-217/2021	EPI_ISL_1288412	09/02/21	North America / Mexico / Mexico City	Human	Female	67	Hospitalized	Nasopharyngeal and oropharyngeal swab
hCoV-19/Mexico/MEX-IBT-IMSS-218/2021	EPI_ISL_1288413	13/02/21	North America / Mexico / State of Mexico	Human	Male	12	Ambulatory	Nasopharyngeal and oropharyngeal swab
hCoV-19/Mexico/CMX-IBT-IMSS-219/2021	EPI_ISL_1288414	13/02/21	North America / Mexico / Mexico City	Human	Male	61	Hospitalized	Oropharyngeal swab
hCoV-19/Mexico/CMX-IBT-IMSS-221/2021	EPI_ISL_1288415	13/02/21	North America / Mexico / Mexico City	Human	Female	44	Hospitalized	Oropharyngeal swab
hCoV-19/Mexico/MEX-IBT-IMSS-222/2021	EPI_ISL_1288416	13/02/21	North America / Mexico / State of Mexico	Human	Female	67	Ambulatory	Oropharyngeal swab
hCoV-19/Mexico/AGU-IBT-IMSS-223/2021	EPI_ISL_1288417	12/02/21	North America / Mexico / Mexico City	Human	Female	79	Hospitalized	Nasopharyngeal swab
hCoV-19/Mexico/CMX-IBT-IMSS-224/2021	EPI_ISL_1288418	12/02/21	North America / Mexico / Mexico City	Human	Female	81	Hospitalized	Nasopharyngeal swab
hCoV-19/Mexico/CMX-IBT-IMSS-225/2021	EPI_ISL_1288419	13/02/21	North America / Mexico / Mexico City	Human	Female	64	Ambulatory	Oropharyngeal swab
hCoV-19/Mexico/CMX-IBT-IMSS-227/2021	EPI_ISL_1288420	12/02/21	North America / Mexico / Mexico City	Human	Male	40	Hospitalized	Oropharyngeal swab
hCoV-19/Mexico/QUE-IBT-IMSS-228/2021	EPI_ISL_1288421	11/02/21	North America / Mexico / Queretaro	Human	Female	64	Hospitalized	Nasopharyngeal and oropharyngeal swab
hCoV-19/Mexico/QUE-IBT-IMSS-229/2021	EPI_ISL_1288422	11/02/21	North America / Mexico / Queretaro	Human	Female	86	Hospitalized	Nasopharyngeal and oropharyngeal swab
hCoV-19/Mexico/BCS-IBT-IMSS-230/2021	EPI_ISL_1288423	11/02/21	North America / Mexico / Baja California Sur	Human	Female	32	Ambulatory	Nasopharyngeal and oropharyngeal swab
hCoV-19/Mexico/BCS-IBT-IMSS-231/2021	EPI_ISL_1288424	11/02/21	North America / Mexico / Baja California Sur	Human	Male	31	Ambulatory	Nasopharyngeal and oropharyngeal swab
hCoV-19/Mexico/BCS-IBT-IMSS-232/2021	EPI_ISL_1288425	11/02/21	North America / Mexico / Baja California Sur	Human	Female	32	Ambulatory	Nasopharyngeal and oropharyngeal swab
hCoV-19/Mexico/BCS-IBT-IMSS-233/2021	EPI_ISL_1288426	11/02/21	North America / Mexico / Baja California Sur	Human	Female	52	Ambulatory	Nasopharyngeal and oropharyngeal swab
hCoV-19/Mexico/BCS-IBT-IMSS-236/2021	EPI_ISL_1288428	11/02/21	North America / Mexico / Baja California Sur	Human	Male	49	Ambulatory	Nasopharyngeal and oropharyngeal swab
hCoV-19/Mexico/BCS-IBT-IMSS-239/2021	EPI_ISL_1288429	11/02/21	North America / Mexico / Baja California Sur	Human	Male	27	Ambulatory	Nasopharyngeal and oropharyngeal swab
hCoV-19/Mexico/BCS-IBT-IMSS-240/2021	EPI_ISL_1288430	11/02/21	North America / Mexico / Baja California Sur	Human	Female	33	Ambulatory	Nasopharyngeal and oropharyngeal swab
hCoV-19/Mexico/BCS-IBT-IMSS-242/2021	EPI_ISL_1288431	10/02/21	North America / Mexico / Baja California Sur	Human	Female	28	Ambulatory	Nasopharyngeal and oropharyngeal swab
hCoV-19/Mexico/BCS-IBT-IMSS-245/2021	EPI_ISL_1288434	10/02/21	North America / Mexico / Baja California Sur	Human	Female	27	Ambulatory	Nasopharyngeal and oropharyngeal swab
hCoV-19/Mexico/BCS-IBT-IMSS-246/2021	EPI_ISL_1288435	12/02/21	North America / Mexico / Baja California Sur	Human	Female	28	Ambulatory	Nasopharyngeal and oropharyngeal swab
hCoV-19/Mexico/BCS-IBT-IMSS-247/2021	EPI_ISL_1288436	12/02/21	North America / Mexico / Baja California Sur	Human	Male	24	Ambulatory	Nasopharyngeal and oropharyngeal swab
hCoV-19/Mexico/BCS-IBT-IMSS-250/2021	EPI_ISL_1288438	12/02/21	North America / Mexico / Baja California Sur	Human	Male	40	Ambulatory	Nasopharyngeal and oropharyngeal swab
hCoV-19/Mexico/BCS-IBT-IMSS-251/2021	EPI_ISL_1288439	12/02/21	North America / Mexico / Baja California Sur	Human	Female	39	Ambulatory	Nasopharyngeal and oropharyngeal swab
hCoV-19/Mexico/BCS-IBT-IMSS-252/2021	EPI_ISL_1288440	12/02/21	North America / Mexico / Baja California Sur	Human	Female	36	Ambulatory	Nasopharyngeal and oropharyngeal swab
hCoV-19/Mexico/TLA-IBT-IMSS-253/2021	EPI_ISL_1288441	12/02/21	North America / Mexico / Tlaxcala	Human	Male	64	Hospitalized	Nasopharyngeal and oropharyngeal swab
hCoV-19/Mexico/HID-IBT-IMSS-254/2021	EPI_ISL_1288443	13/02/21	North America / Mexico / Hidalgo	Human	Female	57	Hospitalized	Oropharyngeal swab
hCoV-19/Mexico/VER-IBT-IMSS-255/2021	EPI_ISL_1288444	10/02/21	North America / Mexico / Veracruz	Human	Female	48	Ambulatory	Nasopharyngeal swab
hCoV-19/Mexico/VER-IBT-IMSS-256/2021	EPI_ISL_1288445	11/02/21	North America / Mexico / Veracruz	Human	Male	20	Ambulatory	Nasopharyngeal and oropharyngeal swab
hCoV-19/Mexico/VER-IBT-IMSS-257/2021	EPI_ISL_1288446	11/02/21	North America / Mexico / Veracruz	Human	Female	58	Ambulatory	Nasopharyngeal and oropharyngeal swab

hCoV-19/Mexico/VER-IBT-IMSS-258/2021	EPI_ISL_1288447	11/02/21	North America / Mexico / Veracruz	Human	Male	14	Ambulatory	Nasopharyngeal and oropharyngeal swab
hCoV-19/Mexico/VER-IBT-IMSS-259/2021	EPI_ISL_1288448	11/02/21	North America / Mexico / Veracruz	Human	Male	41	Ambulatory	Nasopharyngeal and oropharyngeal swab
hCoV-19/Mexico/VER-IBT-IMSS-261/2021	EPI_ISL_1288449	11/02/21	North America / Mexico / Veracruz	Human	Male	24	Ambulatory	Nasopharyngeal and oropharyngeal swab
hCoV-19/Mexico/VER-IBT-IMSS-262/2021	EPI_ISL_1288450	11/02/21	North America / Mexico / Veracruz	Human	Male	41	Ambulatory	Nasopharyngeal and oropharyngeal swab
hCoV-19/Mexico/VER-IBT-IMSS-263/2021	EPI_ISL_1288451	11/02/21	North America / Mexico / Veracruz	Human	Female	21	Ambulatory	Nasopharyngeal and oropharyngeal swab
hCoV-19/Mexico/HID-IBT-IMSS-265/2021	EPI_ISL_1288453	05/02/21	North America / Mexico / Hidalgo	Human	Female	73	Hospitalized	Nasopharyngeal and oropharyngeal swab
hCoV-19/Mexico/VER-IBT-IMSS-266/2021	EPI_ISL_1288454	11/02/21	North America / Mexico / Veracruz	Human	Male	33	Ambulatory	Nasopharyngeal and oropharyngeal swab
hCoV-19/Mexico/VER-IBT-IMSS-267/2021	EPI_ISL_1288455	11/02/21	North America / Mexico / Veracruz	Human	Female	34	Ambulatory	Nasopharyngeal and oropharyngeal swab
hCoV-19/Mexico/VER-IBT-IMSS-268/2021	EPI_ISL_1288456	11/02/21	North America / Mexico / Veracruz	Human	Female	21	Ambulatory	Nasopharyngeal and oropharyngeal swab
hCoV-19/Mexico/VER-IBT-IMSS-269/2021	EPI_ISL_1288457	11/02/21	North America / Mexico / Veracruz	Human	Male	37	Ambulatory	Nasopharyngeal and oropharyngeal swab
hCoV-19/Mexico/VER-IBT-IMSS-270/2021	EPI_ISL_1288458	11/02/21	North America / Mexico / Veracruz	Human	Male	31	Ambulatory	Nasopharyngeal and oropharyngeal swab
hCoV-19/Mexico/VER-IBT-IMSS-273/2021	EPI_ISL_1288460	11/02/21	North America / Mexico / Veracruz	Human	Male	39	Ambulatory	Nasopharyngeal and oropharyngeal swab
hCoV-19/Mexico/VER-IBT-IMSS-274/2021	EPI_ISL_1288461	11/02/21	North America / Mexico / Veracruz	Human	Male	27	Ambulatory	Nasopharyngeal and oropharyngeal swab
hCoV-19/Mexico/VER-IBT-IMSS-275/2021	EPI_ISL_1288462	12/02/21	North America / Mexico / Veracruz	Human	Female	61	Ambulatory	Nasopharyngeal and oropharyngeal swab
hCoV-19/Mexico/VER-IBT-IMSS-276/2021	EPI_ISL_1288463	12/02/21	North America / Mexico / Veracruz	Human	Female	26	Ambulatory	Nasopharyngeal and oropharyngeal swab
hCoV-19/Mexico/VER-IBT-IMSS-277/2021	EPI_ISL_1288464	12/02/21	North America / Mexico / Veracruz	Human	Female	58	Ambulatory	Nasopharyngeal and oropharyngeal swab
hCoV-19/Mexico/VER-IBT-IMSS-278/2021	EPI_ISL_1288465	11/02/21	North America / Mexico / Veracruz	Human	Female	41	Ambulatory	Nasopharyngeal and oropharyngeal swab
hCoV-19/Mexico/VER-IBT-IMSS-281/2021	EPI_ISL_1288468	11/02/21	North America / Mexico / Veracruz	Human	Female	20	Ambulatory	Nasopharyngeal and oropharyngeal swab
hCoV-19/Mexico/VER-IBT-IMSS-283/2021	EPI_ISL_1288469	11/02/21	North America / Mexico / Veracruz	Human	Male	30	Ambulatory	Nasopharyngeal and oropharyngeal swab
hCoV-19/Mexico/CMX-IBT-IMSS-284/2021	EPI_ISL_1288470	13/02/21	North America / Mexico / Mexico City	Human	Male	62	Ambulatory	Nasopharyngeal and oropharyngeal swab
hCoV-19/Mexico/CMX-IBT-IMSS-285/2021	EPI_ISL_1288471	14/02/21	North America / Mexico / Mexico City	Human	Male	77	Hospitalized	Nasopharyngeal and oropharyngeal swab
hCoV-19/Mexico/MEX-IBT-IMSS-286/2021	EPI_ISL_1288472	14/02/21	North America / Mexico / State of Mexico	Human	Male	67	Hospitalized	Nasopharyngeal and oropharyngeal swab
hCoV-19/Mexico/SLP-IBT-IMSS-287/2021	EPI_ISL_1288473	12/02/21	North America / Mexico / San Luis Potosi	Human	Female	71	Hospitalized	Nasopharyngeal and oropharyngeal swab
hCoV-19/Mexico/SLP-IBT-IMSS-288/2021	EPI_ISL_1288474	13/02/21	North America / Mexico / San Luis Potosi	Human	Female	68	Hospitalized	Nasopharyngeal and oropharyngeal swab
hCoV-19/Mexico/SLP-IBT-IMSS-289/2021	EPI_ISL_1288475	12/02/21	North America / Mexico / San Luis Potosi	Human	Male	65	Hospitalized	Nasopharyngeal and oropharyngeal swab
hCoV-19/Mexico/CMX-IBT-IMSS-290/2021	EPI_ISL_1288476	13/02/21	North America / Mexico / Mexico City	Human	Male	10	Ambulatory	Nasopharyngeal and oropharyngeal swab
hCoV-19/Mexico/CMX-IBT-IMSS-291/2021	EPI_ISL_1288477	12/02/21	North America / Mexico / Mexico City	Human	Female	44	Ambulatory	Nasopharyngeal and oropharyngeal swab
hCoV-19/Mexico/CMX-IBT-IMSS-293/2021	EPI_ISL_1288478	12/02/21	North America / Mexico / Mexico City	Human	Male	82	Hospitalized	Nasopharyngeal and oropharyngeal swab
hCoV-19/Mexico/CMX-IBT-IMSS-294/2021	EPI_ISL_1288479	12/02/21	North America / Mexico / Mexico City	Human	Female	52	Ambulatory	Nasopharyngeal and oropharyngeal swab
hCoV-19/Mexico/MEX-IBT-IMSS-295/2021	EPI_ISL_1288480	12/02/21	North America / Mexico / State of Mexico	Human	Male	41	Hospitalized	Nasopharyngeal and oropharyngeal swab
hCoV-19/Mexico/CMX-IBT-IMSS-296/2021	EPI_ISL_1288481	13/02/21	North America / Mexico / Mexico City	Human	Male	68	Hospitalized	Nasopharyngeal and oropharyngeal swab
hCoV-19/Mexico/CMX-IBT-IMSS-297/2021	EPI_ISL_1288482	13/02/21	North America / Mexico / Mexico City	Human	Female	56	Hospitalized	Nasopharyngeal and oropharyngeal swab
hCoV-19/Mexico/CMX-IBT-IMSS-298/2021	EPI_ISL_1288483	14/02/21	North America / Mexico / Mexico City	Human	Male	82	Hospitalized	Nasopharyngeal and oropharyngeal swab
hCoV-19/Mexico/CMX-IBT-IMSS-300/2021	EPI_ISL_1288485	13/02/21	North America / Mexico / Mexico City	Human	Male	74	Hospitalized	Nasopharyngeal and oropharyngeal swab
hCoV-19/Mexico/CMX-IBT-IMSS-301/2021	EPI_ISL_1288486	14/02/21	North America / Mexico / Mexico City	Human	Female	58	Ambulatory	Nasopharyngeal swab
hCoV-19/Mexico/OAX-IBT-IMSS-304/2021	EPI_ISL_1288488	12/02/21	North America / Mexico / Oaxaca	Human	Male	22	Ambulatory	Nasopharyngeal and oropharyngeal swab
hCoV-19/Mexico/OAX-IBT-IMSS-305/2021	EPI_ISL_1288489	12/02/21	North America / Mexico / Oaxaca	Human	Female	46	Ambulatory	Nasopharyngeal and oropharyngeal swab
hCoV-19/Mexico/OAX-IBT-IMSS-306/2021	EPI_ISL_1288490	12/02/21	North America / Mexico / Oaxaca	Human	Male	29	Ambulatory	Nasopharyngeal and oropharyngeal swab
hCoV-19/Mexico/BCS-IBT-IMSS-308/2021	EPI_ISL_1288491	12/02/21	North America / Mexico / Baja California Sur	Human	Male	47	Ambulatory	Nasopharyngeal and oropharyngeal swab
hCoV-19/Mexico/BCS-IBT-IMSS-309/2021	EPI_ISL_1288492	12/02/21	North America / Mexico / Baja California Sur	Human	Female	21	Ambulatory	Nasopharyngeal and oropharyngeal swab
hCoV-19/Mexico/MEX-IBT-IMSS-311/2021	EPI_ISL_1288494	12/02/21	North America / Mexico / State of Mexico	Human	Male	51	Ambulatory	Nasopharyngeal and oropharyngeal swab
hCoV-19/Mexico/MEX-IBT-IMSS-312/2021	EPI_ISL_1288495	14/02/21	North America / Mexico / State of Mexico	Human	Female	70	Hospitalized	Nasopharyngeal and oropharyngeal swab
hCoV-19/Mexico/MEX-IBT-IMSS-313/2021	EPI_ISL_1288496	13/02/21	North America / Mexico / State of Mexico	Human	Female	79	Hospitalized	Nasopharyngeal and oropharyngeal swab
hCoV-19/Mexico/MEX-IBT-IMSS-315/2021	EPI_ISL_1288497	14/02/21	North America / Mexico / State of Mexico	Human	Male	66	Hospitalized	Nasopharyngeal and oropharyngeal swab

hCoV-19/Mexico/CMX-IBT-IMSS-317/2021	EPI_ISL_1288499	13/02/21	North America / Mexico / Mexico City	Human	Male	72	Hospitalized	Nasopharyngeal and oropharyngeal swab
hCoV-19/Mexico/MEX-IBT-IMSS-318/2021	EPI_ISL_1288500	13/02/21	North America / Mexico / State of Mexico	Human	Male	62	Ambulatory	Nasopharyngeal and oropharyngeal swab
hCoV-19/Mexico/MOR-IBT-IMSS-319/2021	EPI_ISL_1288501	13/02/21	North America / Mexico / Morelos	Human	Male	65	Ambulatory	Nasopharyngeal and oropharyngeal swab
hCoV-19/Mexico/MOR-IBT-IMSS-321/2021	EPI_ISL_1288503	13/02/21	North America / Mexico / Morelos	Human	Female	65	Hospitalized	Nasopharyngeal and oropharyngeal swab
hCoV-19/Mexico/MOR-IBT-IMSS-322/2021	EPI_ISL_1288504	13/02/21	North America / Mexico / Morelos	Human	Female	67	Hospitalized	Nasopharyngeal and oropharyngeal swab
hCoV-19/Mexico/MOR-IBT-IMSS-323/2021	EPI_ISL_1288505	13/02/21	North America / Mexico / Morelos	Human	Male	52	Ambulatory	Nasopharyngeal and oropharyngeal swab
hCoV-19/Mexico/MOR-IBT-IMSS-324/2021	EPI_ISL_1288506	13/02/21	North America / Mexico / Morelos	Human	Male	54	Hospitalized	Nasopharyngeal and oropharyngeal swab
hCoV-19/Mexico/CMX-IBT-IMSS-326/2021	EPI_ISL_1288507	13/02/21	North America / Mexico / Mexico City	Human	Male	72	Hospitalized	Nasopharyngeal swab
hCoV-19/Mexico/CMX-IBT-IMSS-327/2021	EPI_ISL_1288508	13/02/21	North America / Mexico / Mexico City	Human	Female	62	Hospitalized	Nasopharyngeal swab
hCoV-19/Mexico/CMX-IBT-IMSS-328/2021	EPI_ISL_1288509	13/02/21	North America / Mexico / Mexico City	Human	Female	81	Hospitalized	Nasopharyngeal swab
hCoV-19/Mexico/CMX-IBT-IMSS-329/2021	EPI_ISL_1288510	13/02/21	North America / Mexico / Mexico City	Human	Male	39	Hospitalized	Nasopharyngeal swab
hCoV-19/Mexico/CMX-IBT-IMSS-330/2021	EPI_ISL_1288511	13/02/21	North America / Mexico / Mexico City	Human	Male	53	Hospitalized	Nasopharyngeal swab
hCoV-19/Mexico/CMX-IBT-IMSS-331/2021	EPI_ISL_1288512	13/02/21	North America / Mexico / Mexico City	Human	Male	52	Hospitalized	Nasopharyngeal swab
hCoV-19/Mexico/HID-IBT-IMSS-332/2021	EPI_ISL_1288513	13/02/21	North America / Mexico / Hidalgo	Human	Female	33	Ambulatory	Oropharyngeal swab
hCoV-19/Mexico/HID-IBT-IMSS-333/2021	EPI_ISL_1288514	13/02/21	North America / Mexico / Hidalgo	Human	Female	30	Ambulatory	Oropharyngeal swab
hCoV-19/Mexico/CMX-IBT-IMSS-334/2021	EPI_ISL_1288515	13/02/21	North America / Mexico / Mexico City	Human	Female	66	Ambulatory	Nasopharyngeal and oropharyngeal swab
hCoV-19/Mexico/MEX-IBT-IMSS-337/2021	EPI_ISL_1288516	12/02/21	North America / Mexico / State of Mexico	Human	Male	72	Hospitalized	Oropharyngeal swab
hCoV-19/Mexico/MEX-IBT-IMSS-339/2021	EPI_ISL_1288518	12/02/21	North America / Mexico / State of Mexico	Human	Female	26	Hospitalized	Nasopharyngeal and oropharyngeal swab
hCoV-19/Mexico/CMX-IBT-IMSS-340/2021	EPI_ISL_1288519	12/02/21	North America / Mexico / Mexico City	Human	Male	9	Ambulatory	Nasopharyngeal and oropharyngeal swab
hCoV-19/Mexico/CMX-IBT-IMSS-341/2021	EPI_ISL_1288520	14/02/21	North America / Mexico / Mexico City	Human	Female	7	Ambulatory	Nasopharyngeal and oropharyngeal swab
hCoV-19/Mexico/CMX-IBT-IMSS-342/2021	EPI_ISL_1288521	14/02/21	North America / Mexico / Mexico City	Human	Male	56	Hospitalized	Oropharyngeal swab
hCoV-19/Mexico/MEX-IBT-IMSS-343/2021	EPI_ISL_1288522	15/02/21	North America / Mexico / State of Mexico	Human	Male	67	Hospitalized	Nasopharyngeal swab
hCoV-19/Mexico/MEX-IBT-IMSS-344/2021	EPI_ISL_1288523	14/02/21	North America / Mexico / State of Mexico	Human	Male	67	Hospitalized	Nasopharyngeal and oropharyngeal swab
hCoV-19/Mexico/CMX-IBT-IMSS-345/2021	EPI_ISL_1288524	12/02/21	North America / Mexico / Mexico City	Human	Male	57	Ambulatory	Nasopharyngeal and oropharyngeal swab
hCoV-19/Mexico/CMX-IBT-IMSS-346/2021	EPI_ISL_1288525	12/02/21	North America / Mexico / Mexico City	Human	Female	65	Ambulatory	Nasopharyngeal and oropharyngeal swab
hCoV-19/Mexico/CMX-IBT-IMSS-350/2021	EPI_ISL_1288527	12/02/21	North America / Mexico / Mexico City	Human	Male	58	Ambulatory	Nasopharyngeal and oropharyngeal swab
hCoV-19/Mexico/VER-IBT-IMSS-353/2021	EPI_ISL_1288530	12/02/21	North America / Mexico / Veracruz	Human	Female	34	Ambulatory	Nasopharyngeal swab
hCoV-19/Mexico/VER-IBT-IMSS-354/2021	EPI_ISL_1288531	12/02/21	North America / Mexico / Veracruz	Human	Male	49	Ambulatory	Nasopharyngeal and oropharyngeal swab
hCoV-19/Mexico/MEX-IBT-IMSS-356/2021	EPI_ISL_1288533	14/02/21	North America / Mexico / State of Mexico	Human	Male	56	Hospitalized	Nasopharyngeal and oropharyngeal swab
hCoV-19/Mexico/BCN-SEARCH-7360/2021	EPI_ISL_1295671	10/02/21	orth America / Mexico / Baja California / Tijuan	Human	unknown	unknown	unknown	Nasal swab
hCoV-19/Mexico/BCN-SEARCH-7370/2021	EPI_ISL_1295676	08/02/21	orth America / Mexico / Baja California / Tijuan	Human	unknown	unknown	unknown	Nasal swab
hCoV-19/Mexico/CMX-INMEGEN-03-03-01/2021	EPI_ISL_1298451	20/02/21	North America / Mexico / Mexico City	Human	Female	30	unknown	Oropharyngeal swab
hCoV-19/Mexico/CMX-INMEGEN-03-03-02/2021	EPI_ISL_1298452	20/02/21	North America / Mexico / Mexico City	Human	Male	65	unknown	Oropharyngeal swab
hCoV-19/Mexico/CMX-INMEGEN-03-03-04/2021	EPI_ISL_1298454	19/02/21	North America / Mexico / Mexico City	Human	Female	59	unknown	Oropharyngeal swab
hCoV-19/Mexico/CMX-INMEGEN-03-03-05/2021	EPI_ISL_1298455	20/02/21	North America / Mexico / Mexico City	Human	Female	16	unknown	Oropharyngeal swab
hCoV-19/Mexico/CMX-INMEGEN-03-03-06/2021	EPI_ISL_1298456	20/02/21	North America / Mexico / Mexico City	Human	Female	69	unknown	Oropharyngeal swab
hCoV-19/Mexico/CMX-INMEGEN-03-03-07/2021	EPI_ISL_1298457	22/02/21	North America / Mexico / Mexico City	Human	Male	29	unknown	Oropharyngeal swab
hCoV-19/Mexico/CMX-INMEGEN-03-03-08/2021	EPI_ISL_1298458	22/02/21	North America / Mexico / Mexico City	Human	Male	68	unknown	Oropharyngeal swab
hCoV-19/Mexico/CMX-INMEGEN-03-03-09/2021	EPI_ISL_1298459	22/02/21	North America / Mexico / Mexico City	Human	Male	41	unknown	Oropharyngeal swab
hCoV-19/Mexico/CMX-INMEGEN-03-03-10/2021	EPI_ISL_1298460	22/02/21	North America / Mexico / Mexico City	Human	Male	50	unknown	Oropharyngeal swab
hCoV-19/Mexico/CMX-INMEGEN-03-03-11/2021	EPI_ISL_1298461	22/02/21	North America / Mexico / Mexico City	Human	Male	12	unknown	Oropharyngeal swab
hCoV-19/Mexico/CMX-INMEGEN-03-03-13/2021	EPI_ISL_1298463	22/02/21	North America / Mexico / Mexico City	Human	Female	64	unknown	Oropharyngeal swab
hCoV-19/Mexico/CMX-INMEGEN-03-03-14/2021	EPI_ISL_1298464	22/02/21	North America / Mexico / Mexico City	Human	Female	54	unknown	Oropharyngeal swab
hCoV-19/Mexico/CMX-INMEGEN-03-03-15/2021	EPI_ISL_1298465	20/02/21	North America / Mexico / Mexico City	Human	Male	49	unknown	Oropharyngeal swab

hCoV-19/Mexico/CMX-INMEGEN-03-03-16/2021	EPI_ISL_1298466	22/02/21	North America / Mexico / Mexico City	Human	Female	29	unknown	Oropharyngeal swab
hCoV-19/Mexico/CMX-INMEGEN-03-03-17/2021	EPI_ISL_1298467	23/02/21	North America / Mexico / Mexico City	Human	Female	59	unknown	Oropharyngeal swab
hCoV-19/Mexico/CMX-INMEGEN-03-03-18/2021	EPI_ISL_1298468	22/02/21	North America / Mexico / Mexico City	Human	Female	52	unknown	Oropharyngeal swab
hCoV-19/Mexico/CMX-INMEGEN-03-03-19/2021	EPI_ISL_1298469	23/02/21	North America / Mexico / Mexico City	Human	Male	6	unknown	Oropharyngeal swab
hCoV-19/Mexico/CMX-INMEGEN-03-03-20/2021	EPI_ISL_1298470	23/02/21	North America / Mexico / Mexico City	Human	Female	76	unknown	Oropharyngeal swab
hCoV-19/Mexico/CMX-INMEGEN-03-03-21/2021	EPI_ISL_1298471	19/02/21	North America / Mexico / Mexico City	Human	Female	24	unknown	Oropharyngeal swab
hCoV-19/Mexico/CMX-INMEGEN-03-03-22/2021	EPI_ISL_1298472	22/02/21	North America / Mexico / Mexico City	Human	Female	32	unknown	Oropharyngeal swab
hCoV-19/Mexico/CMX-INMEGEN-03-03-23/2021	EPI_ISL_1298473	24/02/21	North America / Mexico / Mexico City	Human	Male	40	unknown	Oropharyngeal swab
hCoV-19/Mexico/CMX-INMEGEN-03-05-16/2021	EPI_ISL_1300425	22/02/21	North America / Mexico / Mexico City	Human	Female	37	unknown	
hCoV-19/Mexico/CMX-IBT-IMSS-568/2021	EPI_ISL_1302156	02/02/21	North America / Mexico / Mexico City	Human	Male	46	Ambulatory	Nasopharyngeal and oropharyngeal swab
hCoV-19/Mexico/MEX-IBT-IMSS-547/2021	EPI_ISL_1302157	30/01/21	North America / Mexico / State of Mexico	Human	Male	23	Hospitalized	Pharyngeal swab
hCoV-19/Mexico/MEX-IBT-IMSS-553/2021	EPI_ISL_1302158	02/02/21	North America / Mexico / State of Mexico	Human	Female	65	Hospitalized	Nasopharyngeal and oropharyngeal swab
hCoV-19/Mexico/SLP-IBT-IMSS-560/2021	EPI_ISL_1302161	31/01/21	North America / Mexico / San Luis Potosi	Human	Female	25	Ambulatory	Nasopharyngeal and oropharyngeal swab
hCoV-19/Mexico/CMX-IBT-IMSS-535/2021	EPI_ISL_1302165	01/02/21	North America / Mexico / Mexico City	Human	Male	51	Ambulatory	Nasopharyngeal and oropharyngeal swab
hCoV-19/Mexico/MEX-IBT-IMSS-546/2021	EPI_ISL_1302169	31/01/21	North America / Mexico / State of Mexico	Human	Female	58	Hospitalized	Pharyngeal swab
hCoV-19/Mexico/MEX-IBT-IMSS-544/2021	EPI_ISL_1302172	29/01/21	North America / Mexico / State of Mexico	Human	Male	72	Hospitalized	Nasopharyngeal and oropharyngeal swab
hCoV-19/Mexico/PUE-IBT-IMSS-576/2021	EPI_ISL_1302173	30/01/21	North America / Mexico / Puebla	Human	Male	41	Ambulatory	Nasopharyngeal and oropharyngeal swab
hCoV-19/Mexico/CMX-IBT-IMSS-538/2021	EPI_ISL_1302178	31/01/21	North America / Mexico / Mexico City	Human	Female	75	Hospitalized	Nasopharyngeal and oropharyngeal swab
hCoV-19/Mexico/CMX-IBT-IMSS-572/2021	EPI_ISL_1302190	02/02/21	North America / Mexico / Mexico City	Human	Female	43	Ambulatory	Nasopharyngeal and oropharyngeal swab
hCoV-19/Mexico/CMX-INER-IBT-118/2021	EPI_ISL_1302192	06/01/21	North America / Mexico / Mexico City	Human	Female	unknown	Hospitalized	Nasopharyngeal and oropharyngeal swab
hCoV-19/Mexico/MEX-IBT-IMSS-545/2021	EPI_ISL_1302195	30/01/21	North America / Mexico / State of Mexico	Human	Female	37	Hospitalized	Nasopharyngeal and oropharyngeal swab
hCoV-19/Mexico/MEX-IBT-IMSS-525/2021	EPI_ISL_1302196	01/02/21	North America / Mexico / State of Mexico	Human	Female	82	Hospitalized	Pharyngeal swab
hCoV-19/Mexico/CMX-INER-IBT-127/2021	EPI_ISL_1302198	05/01/21	North America / Mexico / Mexico City	Human	Male	unknown	Hospitalized	Nasopharyngeal and oropharyngeal swab
hCoV-19/Mexico/CMX-IBT-IMSS-523/2021	EPI_ISL_1302203	31/01/21	North America / Mexico / Mexico City	Human	Male	71	Hospitalized	Pharyngeal swab
hCoV-19/Mexico/CMX-IBT-IMSS-530/2021	EPI_ISL_1302204	31/01/21	North America / Mexico / Mexico City	Human	Male	50	Ambulatory	Pharyngeal swab
hCoV-19/Mexico/BCN-IBT-IMSS-519/2021	EPI_ISL_1302220	30/01/21	North America / Mexico / Baja California	Human	Male	35	Ambulatory	Nasopharyngeal and oropharyngeal swab
hCoV-19/Mexico/CMX-IBT-IMSS-524/2021	EPI_ISL_1302221	03/02/21	North America / Mexico / Mexico City	Human	Female	25	Ambulatory	Nasopharyngeal and oropharyngeal swab
hCoV-19/Mexico/CMX-IBT-IMSS-529/2021	EPI_ISL_1302222	02/02/21	North America / Mexico / Mexico City	Human	Female	28	Hospitalized	Nasopharyngeal and oropharyngeal swab
hCoV-19/Mexico/CMX-IBT-IMSS-536/2021	EPI_ISL_1302223	01/02/21	North America / Mexico / Mexico City	Human	Male	45	Hospitalized	Nasopharyngeal and oropharyngeal swab
hCoV-19/Mexico/CMX-IBT-IMSS-563/2021	EPI_ISL_1302224	02/02/21	North America / Mexico / Mexico City	Human	Female	25	Ambulatory	Nasopharyngeal and oropharyngeal swab
hCoV-19/Mexico/CMX-IBT-IMSS-571/2021	EPI_ISL_1302225	02/02/21	North America / Mexico / Mexico City	Human	Female	27	Ambulatory	Nasopharyngeal and oropharyngeal swab
hCoV-19/Mexico/CMX-IBT-IMSS-573/2021	EPI_ISL_1302226	02/02/21	North America / Mexico / Mexico City	Human	Male	41	Ambulatory	Nasopharyngeal and oropharyngeal swab
hCoV-19/Mexico/CMX-INER-IBT-116/2021	EPI_ISL_1302227	04/01/21	North America / Mexico / Mexico City	Human	Male	unknown	Hospitalized	Nasopharyngeal and oropharyngeal swab
hCoV-19/Mexico/HID-IBT-IMSS-514/2021	EPI_ISL_1302235	31/01/21	North America / Mexico / Hidalgo	Human	Female	56	Hospitalized	Nasopharyngeal and oropharyngeal swab
hCoV-19/Mexico/MEX-IBT-IMSS-515/2021	EPI_ISL_1302236	29/01/21	North America / Mexico / State of Mexico	Human	Male	39	Hospitalized	Nasopharyngeal and oropharyngeal swab
hCoV-19/Mexico/SLP-IBT-IMSS-584/2021	EPI_ISL_1302237	30/01/21	North America / Mexico / San Luis Potosi	Human	Female	30	Ambulatory	Nasopharyngeal and oropharyngeal swab
hCoV-19/Mexico/VER-IBT-IMSS-527/2021	EPI_ISL_1302238	30/01/21	North America / Mexico / Veracruz	Human	Female	71	Hospitalized	Nasopharyngeal and oropharyngeal swab
hCoV-19/Mexico/CMX-IBT-IMSS-586/2021	EPI_ISL_1302244	03/02/21	North America / Mexico / Mexico City	Human	Male	53	Ambulatory	Nasopharyngeal and oropharyngeal swab
hCoV-19/Mexico/CMX-INER-IBT-131/2021	EPI_ISL_1302245	04/01/21	North America / Mexico / Mexico City	Human	Female	unknown	Hospitalized	Nasopharyngeal and oropharyngeal swab
hCoV-19/Mexico/MEX-IBT-IMSS-542/2021	EPI_ISL_1302248	01/02/21	North America / Mexico / State of Mexico	Human	Male	70	Hospitalized	Pharyngeal swab
hCoV-19/Mexico/MEX-IBT-IMSS-540/2021	EPI_ISL_1302251	01/02/21	North America / Mexico / State of Mexico	Human	Male	62	Hospitalized	Pharyngeal swab
hCoV-19/Mexico/VER-IBT-IMSS-555/2021	EPI_ISL_1302260	31/01/21	North America / Mexico / Veracruz	Human	Male	58	Hospitalized	Nasopharyngeal and oropharyngeal swab
hCoV-19/Mexico/CMX-IBT-IMSS-526/2021	EPI_ISL_1302261	01/02/21	North America / Mexico / Mexico City	Human	Male	75	Hospitalized	Pharyngeal swab
hCoV-19/Mexico/PUE-IBT-IMSS-575/2021	EPI_ISL_1302268	31/01/21	North America / Mexico / Puebla	Human	Female	40	Ambulatory	Pharyngeal swab

hCoV-19/Mexico/MEX-IBT-IMSS-550/2021	EPI_ISL_1302270	01/02/21	North America / Mexico / State of Mexico	Human	Male	52	Hospitalized	Nasopharyngeal and oropharyngeal swab
hCoV-19/Mexico/CMX-IBT-IMSS-518/2021	EPI_ISL_1302272	01/02/21	North America / Mexico / Mexico City	Human	Female	31	Ambulatory	Nasopharyngeal and oropharyngeal swab
hCoV-19/Mexico/CMX-INER-IBT-124/2021	EPI_ISL_1302273	04/01/21	North America / Mexico / Mexico City	Human	Female	unknown	Hospitalized	Nasopharyngeal and oropharyngeal swab
hCoV-19/Mexico/CMX-IBT-IMSS-528/2021	EPI_ISL_1302275	31/01/21	North America / Mexico / Mexico City	Human	Female	56	Ambulatory	Pharyngeal swab
hCoV-19/Mexico/CMX-INER-IBT-122/2021	EPI_ISL_1302278	04/01/21	North America / Mexico / Mexico City	Human	Male	unknown	Hospitalized	Nasopharyngeal and oropharyngeal swab
hCoV-19/Mexico/CMX-IBT-IMSS-567/2021	EPI_ISL_1302281	02/02/21	North America / Mexico / Mexico City	Human	Female	64	Ambulatory	Nasopharyngeal and oropharyngeal swab
hCoV-19/Mexico/QUE-IBT-IMSS-516/2021	EPI_ISL_1302282	31/01/21	North America / Mexico / Queretaro	Human	Male	56	Hospitalized	Nasopharyngeal and oropharyngeal swab
hCoV-19/Mexico/CMX-IBT-IMSS-521/2021	EPI_ISL_1302284	29/01/21	North America / Mexico / Mexico City	Human	Female	43	Ambulatory	Nasopharyngeal and oropharyngeal swab
hCoV-19/Mexico/SLP-IBT-IMSS-581/2021	EPI_ISL_1302285	01/02/21	North America / Mexico / San Luis Potosi	Human	Male	60	Ambulatory	Nasopharyngeal and oropharyngeal swab
hCoV-19/Mexico/PUE-IBT-IMSS-574/2021	EPI_ISL_1302288	31/01/21	North America / Mexico / Puebla	Human	Female	39	Ambulatory	Pharyngeal swab
hCoV-19/Mexico/CMX-IBT-IMSS-531/2021	EPI_ISL_1302294	29/01/21	North America / Mexico / Mexico City	Human	Female	32	Ambulatory	Nasopharyngeal and oropharyngeal swab
hCoV-19/Mexico/CMX-INER-IBT-132/2021	EPI_ISL_1302306	04/01/21	North America / Mexico / Mexico City	Human	Female	unknown	Hospitalized	Nasopharyngeal and oropharyngeal swab
hCoV-19/Mexico/SLP-IBT-IMSS-561/2021	EPI_ISL_1302311	31/01/21	North America / Mexico / San Luis Potosi	Human	Male	19	Ambulatory	Nasopharyngeal and oropharyngeal swab
hCoV-19/Mexico/CMX-IBT-IMSS-533/2021	EPI_ISL_1302315	01/02/21	North America / Mexico / Mexico City	Human	Male	61	Hospitalized	Nasopharyngeal and oropharyngeal swab
hCoV-19/Mexico/CMX-INER-IBT-119/2021	EPI_ISL_1302316	05/01/21	North America / Mexico / Mexico City	Human	Male	unknown	Hospitalized	Nasopharyngeal and oropharyngeal swab
hCoV-19/Mexico/SLP-IBT-IMSS-580/2021	EPI_ISL_1302318	01/02/21	North America / Mexico / San Luis Potosi	Human	Male	40	Ambulatory	Nasopharyngeal and oropharyngeal swab
hCoV-19/Mexico/CMX-IBT-IMSS-569/2021	EPI_ISL_1302324	02/02/21	North America / Mexico / Mexico City	Human	Female	51	Ambulatory	Nasopharyngeal and oropharyngeal swab
hCoV-19/Mexico/CMX-INER-IBT-117/2021	EPI_ISL_1302327	11/01/21	North America / Mexico / Mexico City	Human	Female	unknown	Hospitalized	Nasopharyngeal and oropharyngeal swab
hCoV-19/Mexico/SLP-IBT-IMSS-585/2021	EPI_ISL_1302332	30/01/21	North America / Mexico / San Luis Potosi	Human	Male	62	Hospitalized	Nasopharyngeal and oropharyngeal swab
hCoV-19/Mexico/VER-IBT-IMSS-556/2021	EPI_ISL_1302341	30/01/21	North America / Mexico / Veracruz	Human	Male	60	Hospitalized	Nasopharyngeal and oropharyngeal swab
hCoV-19/Mexico/CMX-IBT-IMSS-537/2021	EPI_ISL_1302347	01/02/21	North America / Mexico / Mexico City	Human	Male	80	Hospitalized	Pharyngeal swab
hCoV-19/Mexico/MEX-IBT-IMSS-543/2021	EPI_ISL_1302348	01/02/21	North America / Mexico / State of Mexico	Human	Male	58	Hospitalized	Pharyngeal swab
hCoV-19/Mexico/MEX-IBT-IMSS-549/2021	EPI_ISL_1302351	01/02/21	North America / Mexico / State of Mexico	Human	Male	56	Hospitalized	Nasopharyngeal and oropharyngeal swab
hCoV-19/Mexico/SLP-IBT-IMSS-562/2021	EPI_ISL_1302352	31/01/21	North America / Mexico / San Luis Potosi	Human	Female	58	Ambulatory	Nasopharyngeal and oropharyngeal swab
hCoV-19/Mexico/SLP-IBT-IMSS-583/2021	EPI_ISL_1302353	30/01/21	North America / Mexico / San Luis Potosi	Human	Male	53	Hospitalized	Nasopharyngeal and oropharyngeal swab
hCoV-19/Mexico/CMX-IBT-IMSS-520/2021	EPI_ISL_1302355	29/01/21	North America / Mexico / Mexico City	Human	Female	26	Ambulatory	Nasopharyngeal and oropharyngeal swab
hCoV-19/Mexico/MEX-IBT-IMSS-552/2021	EPI_ISL_1302359	01/02/21	North America / Mexico / State of Mexico	Human	Male	61	Hospitalized	Nasopharyngeal and oropharyngeal swab
hCoV-19/Mexico/CMX-INER-IBT-121/2021	EPI_ISL_1302360	04/01/21	North America / Mexico / Mexico City	Human	Female	unknown	Hospitalized	Nasopharyngeal and oropharyngeal swab
hCoV-19/Mexico/SLP-IBT-IMSS-578/2021	EPI_ISL_1302362	01/02/21	North America / Mexico / San Luis Potosi	Human	Female	67	Ambulatory	Nasopharyngeal and oropharyngeal swab
hCoV-19/Mexico/SLP-IBT-IMSS-582/2021	EPI_ISL_1302367	01/02/21	North America / Mexico / San Luis Potosi	Human	Male	70	Hospitalized	Nasopharyngeal and oropharyngeal swab
hCoV-19/Mexico/MEX-IBT-IMSS-541/2021	EPI_ISL_1302370	01/02/21	North America / Mexico / State of Mexico	Human	Female	58	Hospitalized	Pharyngeal swab
hCoV-19/Mexico/CMX-IBT-IMSS-570/2021	EPI_ISL_1302372	02/02/21	North America / Mexico / Mexico City	Human	Male	59	Ambulatory	Nasopharyngeal and oropharyngeal swab
hCoV-19/Mexico/CMX-INER-IBT-133/2021	EPI_ISL_1302374	04/01/21	North America / Mexico / Mexico City	Human	Female	unknown	Hospitalized	Nasopharyngeal and oropharyngeal swab
hCoV-19/Mexico/CMX-INER-IBT-129/2021	EPI_ISL_1302376	04/01/21	North America / Mexico / Mexico City	Human	Male	unknown	Hospitalized	Nasopharyngeal and oropharyngeal swab
hCoV-19/Mexico/CMX-IBT-IMSS-534/2021	EPI_ISL_1302378	01/02/21	North America / Mexico / Mexico City	Human	Female	49	Hospitalized	Nasopharyngeal and oropharyngeal swab
hCoV-19/Mexico/CMX-IBT-IMSS-565/2021	EPI_ISL_1302379	02/02/21	North America / Mexico / Mexico City	Human	Male	39	Ambulatory	Nasopharyngeal and oropharyngeal swab
hCoV-19/Mexico/CMX-INER-IBT-123/2021	EPI_ISL_1302387	05/01/21	North America / Mexico / Mexico City	Human	Male	unknown	Hospitalized	Nasopharyngeal and oropharyngeal swab
hCoV-19/Mexico/PUE-IBT-IMSS-577/2021	EPI_ISL_1302390	29/01/21	North America / Mexico / Puebla	Human	Female	56	Ambulatory	Nasopharyngeal and oropharyngeal swab
hCoV-19/Mexico/CMX-IBT-IMSS-566/2021	EPI_ISL_1302392	03/02/21	North America / Mexico / Mexico City	Human	Female	76	Hospitalized	Nasopharyngeal and oropharyngeal swab
hCoV-19/Mexico/MEX-IBT-IMSS-539/2021	EPI_ISL_1302393	02/02/21	North America / Mexico / State of Mexico	Human	Female	44	Hospitalized	Pharyngeal swab
hCoV-19/Mexico/QUE-IBT-IMSS-517/2021	EPI_ISL_1302401	31/01/21	North America / Mexico / Queretaro	Human	Male	87	Hospitalized	Nasopharyngeal and oropharyngeal swab
hCoV-19/Mexico/CMX-INMEGEN-03-05-46/2021	EPI_ISL_1310808	01/03/21	North America / Mexico / Mexico City	Human	Male	32	unknown	
hCoV-19/Mexico/CMX-INMEGEN-03-05-50/2021	EPI_ISL_1310811	27/02/21	North America / Mexico / Mexico City	Human	Male	22	unknown	
hCoV-19/Mexico/CMX-INMEGEN-03-08-01/2021	EPI_ISL_1315479	25/02/21	North America / Mexico / Mexico City	Human	Female	58	unknown	Oropharyngeal swab

hCoV-19/Mexico/CMX-INMEGEN-03-08-03/2021	EPI_ISL_1315481	01/03/21	North America / Mexico / Mexico City	Human	Female	38	unknown	Oropharyngeal swab
hCoV-19/Mexico/CMX-INMEGEN-03-08-04/2021	EPI_ISL_1315482	01/03/21	North America / Mexico / Mexico City	Human	Male	42	unknown	Oropharyngeal swab
hCoV-19/Mexico/CMX-INMEGEN-03-08-05/2021	EPI_ISL_1315483	01/03/21	North America / Mexico / Mexico City	Human	Female	59	unknown	Oropharyngeal swab
hCoV-19/Mexico/CMX-INMEGEN-03-08-06/2021	EPI_ISL_1315484	01/03/21	North America / Mexico / Mexico City	Human	Female	75	unknown	Oropharyngeal swab
hCoV-19/Mexico/CMX-INMEGEN-03-08-08/2021	EPI_ISL_1315485	01/03/21	North America / Mexico / Mexico City	Human	Female	27	unknown	Oropharyngeal swab
hCoV-19/Mexico/CMX-INMEGEN-03-08-09/2021	EPI_ISL_1315486	01/03/21	North America / Mexico / Mexico City	Human	Male	78	unknown	Oropharyngeal swab
hCoV-19/Mexico/CMX-INMEGEN-03-08-10/2021	EPI_ISL_1315487	26/02/21	North America / Mexico / Mexico City	Human	Female	24	unknown	Oropharyngeal swab
hCoV-19/Mexico/CMX-INMEGEN-03-08-11/2021	EPI_ISL_1315488	01/03/21	North America / Mexico / Mexico City	Human	Male	48	unknown	Oropharyngeal swab
hCoV-19/Mexico/CMX-INMEGEN-03-08-12/2021	EPI_ISL_1315489	02/03/21	North America / Mexico / Mexico City	Human	Female	20	unknown	Oropharyngeal swab
hCoV-19/Mexico/CMX-INMEGEN-03-08-13/2021	EPI_ISL_1315490	02/03/21	North America / Mexico / Mexico City	Human	Male	19	unknown	Oropharyngeal swab
hCoV-19/Mexico/CMX-INMEGEN-03-08-14/2021	EPI_ISL_1315491	02/03/21	North America / Mexico / Mexico City	Human	Female	61	unknown	Oropharyngeal swab
hCoV-19/Mexico/CMX-INMEGEN-03-08-15/2021	EPI_ISL_1315492	24/02/21	North America / Mexico / Mexico City	Human	Male	47	unknown	Oropharyngeal swab
hCoV-19/Mexico/CMX-INMEGEN-03-08-16/2021	EPI_ISL_1315493	24/02/21	North America / Mexico / Mexico City	Human	Male	73	unknown	Oropharyngeal swab
hCoV-19/Mexico/CMX-INMEGEN-03-08-17/2021	EPI_ISL_1315494	03/03/21	North America / Mexico / Mexico City	Human	Female	38	unknown	Oropharyngeal swab
hCoV-19/Mexico/CMX-INMEGEN-03-08-18/2021	EPI_ISL_1315495	02/03/21	North America / Mexico / Mexico City	Human	Male	32	unknown	Oropharyngeal swab
hCoV-19/Mexico/CMX-INMEGEN-03-08-19/2021	EPI_ISL_1315496	02/03/21	North America / Mexico / Mexico City	Human	Female	33	unknown	Oropharyngeal swab
hCoV-19/Mexico/CMX-INMEGEN-03-08-20/2021	EPI_ISL_1315497	02/03/21	North America / Mexico / Mexico City	Human	Female	49	unknown	Oropharyngeal swab
hCoV-19/Mexico/CMX-INMEGEN-03-08-21/2021	EPI_ISL_1315498	04/03/21	North America / Mexico / Mexico City	Human	Female	27	unknown	Oropharyngeal swab
hCoV-19/Mexico/CMX-INMEGEN-03-08-22/2021	EPI_ISL_1315499	03/03/21	North America / Mexico / Mexico City	Human	Male	54	unknown	Oropharyngeal swab
hCoV-19/Mexico/CMX-INMEGEN-03-08-23/2021	EPI_ISL_1315500	03/03/21	North America / Mexico / Mexico City	Human	Male	54	unknown	Oropharyngeal swab
hCoV-19/Mexico/CMX-INMEGEN-03-08-24/2021	EPI_ISL_1315501	03/03/21	North America / Mexico / Mexico City	Human	Female	47	unknown	Oropharyngeal swab
hCoV-19/Mexico/CMX-INMEGEN-03-08-25/2021	EPI_ISL_1315502	03/03/21	North America / Mexico / Mexico City	Human	Male	24	unknown	Oropharyngeal swab
hCoV-19/Mexico/CMX-INMEGEN-03-08-26/2021	EPI_ISL_1315503	04/03/21	North America / Mexico / Mexico City	Human	Male	50	unknown	Oropharyngeal swab
hCoV-19/Mexico/CMX-INMEGEN-03-08-27/2021	EPI_ISL_1315504	02/03/21	North America / Mexico / Mexico City	Human	Female	32	unknown	Oropharyngeal swab
hCoV-19/Mexico/CMX-INMEGEN-03-08-28/2021	EPI_ISL_1315505	03/03/21	North America / Mexico / Mexico City	Human	Male	77	unknown	Oropharyngeal swab
hCoV-19/Mexico/CMX-INMEGEN-03-08-29/2021	EPI_ISL_1315506	04/03/21	North America / Mexico / Mexico City	Human	Male	61	unknown	Oropharyngeal swab
hCoV-19/Mexico/CMX-INMEGEN-03-08-30/2021	EPI_ISL_1315507	03/03/21	North America / Mexico / Mexico City	Human	Male	65	unknown	Oropharyngeal swab
hCoV-19/Mexico/CMX-INMEGEN-03-08-33/2021	EPI_ISL_1315509	03/03/21	North America / Mexico / Mexico City	Human	Male	44	unknown	Oropharyngeal swab
hCoV-19/Mexico/CMX-INMEGEN-03-08-34/2021	EPI_ISL_1315510	03/03/21	North America / Mexico / Mexico City	Human	Male	33	unknown	Oropharyngeal swab
hCoV-19/Mexico/CMX-INMEGEN-03-08-35/2021	EPI_ISL_1315511	04/03/21	North America / Mexico / Mexico City	Human	Male	53	unknown	Oropharyngeal swab
hCoV-19/Mexico/CMX-INMEGEN-03-08-37/2021	EPI_ISL_1315512	03/03/21	North America / Mexico / Mexico City	Human	Female	26	unknown	Oropharyngeal swab
hCoV-19/Mexico/CMX-INMEGEN-03-08-38/2021	EPI_ISL_1315513	04/03/21	North America / Mexico / Mexico City	Human	Female	68	unknown	Oropharyngeal swab
hCoV-19/Mexico/CMX-INMEGEN-03-08-39/2021	EPI_ISL_1315514	03/03/21	North America / Mexico / Mexico City	Human	Female	35	unknown	Oropharyngeal swab
hCoV-19/Mexico/CMX-INMEGEN-03-08-40/2021	EPI_ISL_1315515	03/03/21	North America / Mexico / Mexico City	Human	Female	37	unknown	Oropharyngeal swab
hCoV-19/Mexico/CMX-INMEGEN-03-08-41/2021	EPI_ISL_1315516	03/03/21	North America / Mexico / Mexico City	Human	Male	47	unknown	Oropharyngeal swab
hCoV-19/Mexico/CMX-INMEGEN-03-08-42/2021	EPI_ISL_1315517	03/03/21	North America / Mexico / Mexico City	Human	Female	51	unknown	Oropharyngeal swab
hCoV-19/Mexico/CMX-INMEGEN-03-08-43/2021	EPI_ISL_1315518	03/03/21	North America / Mexico / Mexico City	Human	Male	41	unknown	Oropharyngeal swab
hCoV-19/Mexico/CMX-INMEGEN-03-08-44/2021	EPI_ISL_1315519	03/03/21	North America / Mexico / Mexico City	Human	Female	42	unknown	Oropharyngeal swab
hCoV-19/Mexico/CMX-INMEGEN-03-08-45/2021	EPI_ISL_1315520	01/03/21	North America / Mexico / Mexico City	Human	Female	17	unknown	Oropharyngeal swab
hCoV-19/Mexico/CMX-INMEGEN-03-08-46/2021	EPI_ISL_1315521	03/03/21	North America / Mexico / Mexico City	Human	Male	40	unknown	Oropharyngeal swab
hCoV-19/Mexico/CMX-INMEGEN-03-08-47/2021	EPI_ISL_1315522	26/02/21	North America / Mexico / Mexico City	Human	Female	44	unknown	Oropharyngeal swab
hCoV-19/Mexico/CMX-INMEGEN-03-08-48/2021	EPI_ISL_1315523	05/03/21	North America / Mexico / Mexico City	Human	Female	23	unknown	Oropharyngeal swab
hCoV-19/Mexico/CMX-INMEGEN-03-08-49/2021	EPI_ISL_1315524	04/03/21	North America / Mexico / Mexico City	Human	Male	50	unknown	Oropharyngeal swab
hCoV-19/Mexico/CMX-INMEGEN-03-08-50/2021	EPI_ISL_1315525	05/03/21	North America / Mexico / Mexico City	Human	Female	61	unknown	Oropharyngeal swab

hCoV-19/Mexico/CMX-INMEGEN-03-08-51/2021	EPI_ISL_1315526	04/03/21	North America / Mexico / Mexico City	Human	Male	46	unknown	Oropharyngeal swab
hCoV-19/Mexico/CMX-INMEGEN-03-08-52/2021	EPI_ISL_1315527	03/03/21	North America / Mexico / Mexico City	Human	Male	56	unknown	Oropharyngeal swab
hCoV-19/Mexico/CMX-INMEGEN-03-08-53/2021	EPI_ISL_1315528	03/03/21	North America / Mexico / Mexico City	Human	Male	56	unknown	Oropharyngeal swab
hCoV-19/Mexico/CMX-INMEGEN-03-08-57/2021	EPI_ISL_1315532	04/03/21	North America / Mexico / Mexico City	Human	Male	32	unknown	Oropharyngeal swab
hCoV-19/Mexico/CMX-INMEGEN-03-08-59/2021	EPI_ISL_1315534	04/03/21	North America / Mexico / Mexico City	Human	Female	10	unknown	Oropharyngeal swab
hCoV-19/Mexico/CMX-INMEGEN-03-08-60/2021	EPI_ISL_1315535	04/03/21	North America / Mexico / Mexico City	Human	Male	31	unknown	Oropharyngeal swab
hCoV-19/Mexico/CMX-INMEGEN-03-08-61/2021	EPI_ISL_1315536	04/03/21	North America / Mexico / Mexico City	Human	Female	93	unknown	Oropharyngeal swab
hCoV-19/Mexico/CMX-INMEGEN-03-08-62/2021	EPI_ISL_1315537	04/03/21	North America / Mexico / Mexico City	Human	Male	45	unknown	Oropharyngeal swab
hCoV-19/Mexico/CMX-INMEGEN-03-08-64/2021	EPI_ISL_1315539	04/03/21	North America / Mexico / Mexico City	Human	Male	42	unknown	Oropharyngeal swab
hCoV-19/Mexico/CMX-INMEGEN-03-08-65/2021	EPI_ISL_1315540	05/03/21	North America / Mexico / Mexico City	Human	Male	69	unknown	Oropharyngeal swab
hCoV-19/Mexico/CMX-INMEGEN-03-08-67/2021	EPI_ISL_1315541	05/03/21	North America / Mexico / Mexico City	Human	Female	38	unknown	Oropharyngeal swab
hCoV-19/Mexico/CMX-INMEGEN-03-08-68/2021	EPI_ISL_1315542	04/03/21	North America / Mexico / Mexico City	Human	Male	34	unknown	Oropharyngeal swab
hCoV-19/Mexico/CMX-INMEGEN-03-08-69/2021	EPI_ISL_1315543	05/03/21	North America / Mexico / Mexico City	Human	Female	50	unknown	Oropharyngeal swab
hCoV-19/Mexico/CMX-INMEGEN-03-08-70/2021	EPI_ISL_1315544	04/03/21	North America / Mexico / Mexico City	Human	Female	58	unknown	Oropharyngeal swab
hCoV-19/Mexico/CMX-INMEGEN-03-08-71/2021	EPI_ISL_1315545	09/03/21	North America / Mexico / Mexico City	Human	Female	23	unknown	Oropharyngeal swab
hCoV-19/Mexico/CMX-INMEGEN-03-08-72/2021	EPI_ISL_1315546	04/03/21	North America / Mexico / Mexico City	Human	Female	73	unknown	Oropharyngeal swab
hCoV-19/Mexico/CMX-INMEGEN-03-08-73/2021	EPI_ISL_1315547	04/03/21	North America / Mexico / Mexico City	Human	Female	37	unknown	Oropharyngeal swab
hCoV-19/Mexico/CMX-INMEGEN-03-08-74/2021	EPI_ISL_1315548	04/03/21	North America / Mexico / Mexico City	Human	Male	56	unknown	Oropharyngeal swab
hCoV-19/Mexico/CMX-INMEGEN-03-08-75/2021	EPI_ISL_1315549	04/03/21	North America / Mexico / Mexico City	Human	Male	34	unknown	Oropharyngeal swab
hCoV-19/Mexico/CMX-INMEGEN-03-08-76/2021	EPI_ISL_1315550	04/03/21	North America / Mexico / Mexico City	Human	Male	68	unknown	Oropharyngeal swab
hCoV-19/Mexico/CMX-INMEGEN-03-08-78/2021	EPI_ISL_1315552	04/03/21	North America / Mexico / Mexico City	Human	Male	18	unknown	Oropharyngeal swab
hCoV-19/Mexico/CMX-INMEGEN-03-08-79/2021	EPI_ISL_1315553	05/03/21	North America / Mexico / Mexico City	Human	Male	51	unknown	Oropharyngeal swab
hCoV-19/Mexico/CMX-INMEGEN-03-08-80/2021	EPI_ISL_1315554	05/03/21	North America / Mexico / Mexico City	Human	Male	34	unknown	Oropharyngeal swab
hCoV-19/Mexico/CMX-INMEGEN-03-08-81/2021	EPI_ISL_1315555	04/03/21	North America / Mexico / Mexico City	Human	Male	73	unknown	Oropharyngeal swab
hCoV-19/Mexico/CMX-INMEGEN-03-08-82/2021	EPI_ISL_1315556	03/03/21	North America / Mexico / Mexico City	Human	Female	45	unknown	Oropharyngeal swab
hCoV-19/Mexico/CMX-INMEGEN-03-08-83/2021	EPI_ISL_1315557	04/03/21	North America / Mexico / Mexico City	Human	Female	24	unknown	Oropharyngeal swab
hCoV-19/Mexico/CMX-INMEGEN-03-08-84/2021	EPI_ISL_1315558	04/03/21	North America / Mexico / Mexico City	Human	Female	20	unknown	Oropharyngeal swab
hCoV-19/Mexico/CMX-INMEGEN-03-08-85/2021	EPI_ISL_1315559	04/03/21	North America / Mexico / Mexico City	Human	Female	13	unknown	Oropharyngeal swab
hCoV-19/Mexico/CMX-INMEGEN-03-08-86/2021	EPI_ISL_1315560	04/03/21	North America / Mexico / Mexico City	Human	Male	11	unknown	Oropharyngeal swab
hCoV-19/Mexico/CMX-INMEGEN-03-08-87/2021	EPI_ISL_1315561	04/03/21	North America / Mexico / Mexico City	Human	Female	13	unknown	Oropharyngeal swab
hCoV-19/Mexico/CMX-INMEGEN-03-08-88/2021	EPI_ISL_1315562	04/03/21	North America / Mexico / Mexico City	Human	Female	15	unknown	Oropharyngeal swab
hCoV-19/Mexico/CMX-INMEGEN-03-08-89/2021	EPI_ISL_1315563	04/03/21	North America / Mexico / Mexico City	Human	Male	50	unknown	Oropharyngeal swab
hCoV-19/Mexico/CMX-INMEGEN-03-08-90/2021	EPI_ISL_1315564	05/03/21	North America / Mexico / Mexico City	Human	Male	30	unknown	Oropharyngeal swab
hCoV-19/Mexico/CMX-INMEGEN-03-08-91/2021	EPI_ISL_1315565	09/03/21	North America / Mexico / Mexico City	Human	Female	43	unknown	Oropharyngeal swab
hCoV-19/Mexico/CMX-INMEGEN-03-08-94/2021	EPI_ISL_1315567	05/03/21	North America / Mexico / Mexico City	Human	Male	50	unknown	Oropharyngeal swab
hCoV-19/Mexico/CMX-INMEGEN-03-08-95/2021	EPI_ISL_1315568	05/03/21	North America / Mexico / Mexico City	Human	Female	48	unknown	Oropharyngeal swab
hCoV-19/Mexico/CMX-INMEGEN-03-08-98/2021	EPI_ISL_1315569	04/03/21	North America / Mexico / Mexico City	Human	Male	32	unknown	Oropharyngeal swab
hCoV-19/Mexico/CMX-INMEGEN-03-08-99/2021	EPI_ISL_1315570	05/03/21	North America / Mexico / Mexico City	Human	Female	55	unknown	Oropharyngeal swab
hCoV-19/Mexico/CMX-INMEGEN-03-08-100/2021	EPI_ISL_1315571	08/03/21	North America / Mexico / Mexico City	Human	Male	22	unknown	Oropharyngeal swab
hCoV-19/Mexico/CMX-INMEGEN-03-08-101/2021	EPI_ISL_1315572	09/03/21	North America / Mexico / Mexico City	Human	Male	27	unknown	Oropharyngeal swab
hCoV-19/Mexico/GUA-InDRE_F10353_S633/2021	EPI_ISL_1340608	20/02/21	North America / Mexico / Guanajuato	Human	Male	51	Hospitalized	Oropharyngeal swab
hCoV-19/Mexico/GUA-InDRE_F10362_S636/2021	EPI_ISL_1340610	20/02/21	North America / Mexico / Guanajuato	Human	Male	74	Released	Oropharyngeal swab
hCoV-19/Mexico/GUA-InDRE_F84G_S609/2021	EPI_ISL_1340611	19/02/21	North America / Mexico / Guanajuato	Human	Male	60	Released	Oropharyngeal swab
hCoV-19/Mexico/CMX-InDRE_F95D_S614/2021	EPI_ISL_1340613	19/02/21	North America / Mexico / Mexico City	Human	Female	53	Released	Oropharyngeal swab

hCoV-19/Mexico/GUA-InDRE_F10357_S634/2021	EPI_ISL_1340614	19/02/21	North America / Mexico / Guanajuato	Human	Male	29	Released	Oropharyngeal swab
hCoV-19/Mexico/GUA-InDRE_F10367_S637/2021	EPI_ISL_1340616	19/02/21	North America / Mexico / Guanajuato	Human	Female	17	Released	Oropharyngeal swab
hCoV-19/Mexico/GUA-InDRE_F10395_S644/2021	EPI_ISL_1340617	19/02/21	North America / Mexico / Guanajuato	Human	Male	41	Released	Oropharyngeal swab
hCoV-19/Mexico/QUE-InDRE_F90B_S612/2021	EPI_ISL_1340619	18/02/21	North America / Mexico / Queretaro	Human	Female	51	Deceased	Oropharyngeal swab
hCoV-19/Mexico/CMX-InDRE_F115B_S615/2021	EPI_ISL_1340620	18/02/21	North America / Mexico / Mexico City	Human	Male	23	Released	Oropharyngeal swab
hCoV-19/Mexico/CMX-InDRE_F116B_S616/2021	EPI_ISL_1340621	18/02/21	North America / Mexico / Mexico City	Human	Male	54	Released	Oropharyngeal swab
hCoV-19/Mexico/CMX-InDRE_F117B_S617/2021	EPI_ISL_1340623	18/02/21	North America / Mexico / Mexico City	Human	Male	44	Released	Oropharyngeal swab
hCoV-19/Mexico/GUA-InDRE_F10348_S631/2021	EPI_ISL_1340624	18/02/21	North America / Mexico / Guanajuato	Human	Male	57	Hospitalized	Oropharyngeal swab
hCoV-19/Mexico/GUA-InDRE_F10358_S635/2021	EPI_ISL_1340626	18/02/21	North America / Mexico / Guanajuato	Human	Female	33	Released	Oropharyngeal swab
hCoV-19/Mexico/GUA-InDRE_F10391_S643/2021	EPI_ISL_1340627	18/02/21	North America / Mexico / Guanajuato	Human	Female	65	Released	Oropharyngeal swab
hCoV-19/Mexico/CMX-InDRE_F97I_S613/2021	EPI_ISL_1340629	17/02/21	North America / Mexico / Mexico City	Human	Female	unknown	Released	Oropharyngeal swab
hCoV-19/Mexico/GUA-InDRE_F10349_S632/2021	EPI_ISL_1340630	17/02/21	North America / Mexico / Guanajuato	Human	Female	44	Hospitalized	Oropharyngeal swab
hCoV-19/Mexico/GUA-InDRE_F10382_S641/2021	EPI_ISL_1340631	17/02/21	North America / Mexico / Guanajuato	Human	Male	39	Released	Oropharyngeal swab
hCoV-19/Mexico/GUA-InDRE_F10387_S642/2021	EPI_ISL_1340633	17/02/21	North America / Mexico / Guanajuato	Human	Male	38	Released	Oropharyngeal swab
hCoV-19/Mexico/QUE-InDRE_F88B_S610/2021	EPI_ISL_1340634	16/02/21	North America / Mexico / Queretaro	Human	Male	40	Released	Oropharyngeal swab
hCoV-19/Mexico/QUE-InDRE_F9843_S620/2021	EPI_ISL_1340636	16/02/21	North America / Mexico / Queretaro	Human	Male	24	Released	Oropharyngeal swab
hCoV-19/Mexico/QUE-InDRE_F9847_S621/2021	EPI_ISL_1340637	16/02/21	North America / Mexico / Queretaro	Human	Female	34	Released	Oropharyngeal swab
hCoV-19/Mexico/QUE-InDRE_F9848_S622/2021	EPI_ISL_1340639	16/02/21	North America / Mexico / Queretaro	Human	Female	39	Released	Oropharyngeal swab
hCoV-19/Mexico/HID-InDRE_F10298_S626/2021	EPI_ISL_1340640	16/02/21	North America / Mexico / Hidalgo	Human	Male	51	Deceased	Oropharyngeal swab
hCoV-19/Mexico/HID-InDRE_F10306_S627/2021	EPI_ISL_1340642	16/02/21	North America / Mexico / Hidalgo	Human	Male	68	Hospitalized	Oropharyngeal swab
hCoV-19/Mexico/GUA-InDRE_F10333_S628/2021	EPI_ISL_1340643	16/02/21	North America / Mexico / Guanajuato	Human	Female	43	Deceased	Oropharyngeal swab
hCoV-19/Mexico/GUA-InDRE_F10345_S629/2021	EPI_ISL_1340644	16/02/21	North America / Mexico / Guanajuato	Human	Male	52	Hospitalized	Oropharyngeal swab
hCoV-19/Mexico/GUA-InDRE_F10347_S630/2021	EPI_ISL_1340646	16/02/21	North America / Mexico / Guanajuato	Human	Female	90	Hospitalized	Oropharyngeal swab
hCoV-19/Mexico/GUA-InDRE_F10379_S638/2021	EPI_ISL_1340647	16/02/21	North America / Mexico / Guanajuato	Human	Female	18	Released	Oropharyngeal swab
hCoV-19/Mexico/GUA-InDRE_F10380_S639/2021	EPI_ISL_1340648	16/02/21	North America / Mexico / Guanajuato	Human	Male	21	Released	Oropharyngeal swab
hCoV-19/Mexico/GUA-InDRE_F10381_S640/2021	EPI_ISL_1340650	16/02/21	North America / Mexico / Guanajuato	Human	Male	33	Released	Oropharyngeal swab
hCoV-19/Mexico/PUE-InDRE_F10410_S645/2021	EPI_ISL_1340651	16/02/21	North America / Mexico / Puebla	Human	Male	49	Released	Oropharyngeal swab
hCoV-19/Mexico/PUE-InDRE_F10412_S646/2021	EPI_ISL_1340652	16/02/21	North America / Mexico / Puebla	Human	Female	63	Released	Oropharyngeal swab
hCoV-19/Mexico/QUE-InDRE_F89B_S611/2021	EPI_ISL_1340654	15/02/21	North America / Mexico / Queretaro	Human	Female	28	Released	Oropharyngeal swab
hCoV-19/Mexico/QUE-InDRE_F9839_S618/2021	EPI_ISL_1340655	15/02/21	North America / Mexico / Queretaro	Human	Male	58	Released	Oropharyngeal swab
hCoV-19/Mexico/QUE-InDRE_F9841_S619/2021	EPI_ISL_1340657	15/02/21	North America / Mexico / Queretaro	Human	Female	46	Released	Oropharyngeal swab
hCoV-19/Mexico/YUC-InDRE_F9950_S623/2021	EPI_ISL_1340658	15/02/21	North America / Mexico / Yucatan	Human	Female	44	Released	Oropharyngeal swab
hCoV-19/Mexico/ZAC-InDRE_F10108_S625/2021	EPI_ISL_1340660	15/02/21	North America / Mexico / Zacatecas	Human	Female	68	Released	Oropharyngeal swab
hCoV-19/Mexico/YUC-InDRE_F9951_S624/2021	EPI_ISL_1340661	14/02/21	North America / Mexico / Yucatan	Human	Female	39	Released	Oropharyngeal swab
hCoV-19/Mexico/TAM_LANGEBIO_IMSS_0003/2021	EPI_ISL_1351420	22/02/21	North America / Mexico / Nuevo Leon	Human	Female	42	Ambulatory	Pharyngeal and Nasopharyngeal swab
hCoV-19/Mexico/TAM_LANGEBIO_IMSS_0004/2021	EPI_ISL_1351422	22/02/21	North America / Mexico / Nuevo Leon	Human	Male	54	Hospitalized	Pharyngeal and Nasopharyngeal swab
hCoV-19/Mexico/TAM_LANGEBIO_IMSS_0006/2021	EPI_ISL_1351424	22/02/21	North America / Mexico / Nuevo Leon	Human	Male	19	Ambulatory	Pharyngeal and Nasopharyngeal swab
hCoV-19/Mexico/TAM_LANGEBIO_IMSS_0010/2021	EPI_ISL_1351433	25/02/21	North America / Mexico / Tamaulipas	Human	Female	24	Ambulatory	Pharyngeal and Nasopharyngeal swab
hCoV-19/Mexico/TAM_LANGEBIO_IMSS_0014/2021	EPI_ISL_1351441	03/03/21	North America / Mexico / Coahuila	Human	Male	36	Ambulatory	Pharyngeal and Nasopharyngeal swab
hCoV-19/Mexico/TAM_LANGEBIO_IMSS_0015/2021	EPI_ISL_1351443	02/03/21	North America / Mexico / Coahuila	Human	Male	55	Hospitalized	Pharyngeal and Nasopharyngeal swab
hCoV-19/Mexico/TAM_LANGEBIO_IMSS_0016/2021	EPI_ISL_1351445	03/03/21	North America / Mexico / Nuevo Leon	Human	Male	74	Hospitalized	Pharyngeal and Nasopharyngeal swab
hCoV-19/Mexico/TAM_LANGEBIO_IMSS_0021/2021	EPI_ISL_1351460	04/03/21	North America / Mexico / Nuevo Leon	Human	Female	28	Ambulatory	Pharyngeal and Nasopharyngeal swab
hCoV-19/Mexico/TAM_LANGEBIO_IMSS_0022/2021	EPI_ISL_1351462	04/03/21	North America / Mexico / Nuevo Leon	Human	Male	61	Hospitalized	Nasopharyngeal swab
hCoV-19/Mexico/TAM_LANGEBIO_IMSS_0023/2021	EPI_ISL_1351465	21/02/21	North America / Mexico / Yucatan	Human	Male	75	Hospitalized	Pharyngeal and Nasopharyngeal swab

CoV-19/Mexico/TAM_LANGEBIO_IMSS_0024/202	EPI_ISL_1351468	19/02/21	North America / Mexico / Yucatan	Human	Male	45	Ambulatory	Pharyngeal and Nasopharyngeal swab
CoV-19/Mexico/TAM_LANGEBIO_IMSS_0026/202	EPI_ISL_1351474	21/02/21	North America / Mexico / Quintana Roo	Human	Male	55	Hospitalized	Pharyngeal and Nasopharyngeal swab
CoV-19/Mexico/TAM_LANGEBIO_IMSS_0027/202	EPI_ISL_1351477	21/02/21	North America / Mexico / Quintana Roo	Human	Female	66	Hospitalized	Pharyngeal and Nasopharyngeal swab
CoV-19/Mexico/TAM_LANGEBIO_IMSS_0028/202	EPI_ISL_1351480	23/02/21	North America / Mexico / Yucatan	Human	Male	37	Ambulatory	Pharyngeal and Nasopharyngeal swab
CoV-19/Mexico/TAM_LANGEBIO_IMSS_0029/202	EPI_ISL_1351483	21/02/21	North America / Mexico / Quintana Roo	Human	Female	39	Hospitalized	Pharyngeal and Nasopharyngeal swab
CoV-19/Mexico/TAM_LANGEBIO_IMSS_0030/202	EPI_ISL_1351486	24/02/21	North America / Mexico / Yucatan	Human	Male	29	Ambulatory	Pharyngeal and Nasopharyngeal swab
CoV-19/Mexico/TAM_LANGEBIO_IMSS_0031/202	EPI_ISL_1351489	22/02/21	North America / Mexico / Quintana Roo	Human	Female	36	Ambulatory	Pharyngeal and Nasopharyngeal swab
CoV-19/Mexico/TAM_LANGEBIO_IMSS_0037/202	EPI_ISL_1351506	01/03/21	North America / Mexico / Quintana Roo	Human	Female	25	Hospitalized	Pharyngeal and Nasopharyngeal swab
CoV-19/Mexico/TAM_LANGEBIO_IMSS_0038/202	EPI_ISL_1351509	04/03/21	North America / Mexico / Yucatan	Human	Male	29	Ambulatory	Pharyngeal and Nasopharyngeal swab
CoV-19/Mexico/TAM_LANGEBIO_IMSS_0039/202	EPI_ISL_1351511	03/03/21	North America / Mexico / Jalisco	Human	Male	23	Ambulatory	Pharyngeal and Nasopharyngeal swab
CoV-19/Mexico/TAM_LANGEBIO_IMSS_0041/202	EPI_ISL_1351517	02/03/21	North America / Mexico / Jalisco	Human	Female	26	Hospitalized	Pharyngeal and Nasopharyngeal swab
CoV-19/Mexico/TAM_LANGEBIO_IMSS_0042/202	EPI_ISL_1351520	01/03/21	North America / Mexico / Guanajuato	Human	Male	67	Hospitalized	Pharyngeal and Nasopharyngeal swab
CoV-19/Mexico/TAM_LANGEBIO_IMSS_0043/202	EPI_ISL_1351523	27/02/21	North America / Mexico / Guanajuato	Human	Male	69	Hospitalized	Pharyngeal and Nasopharyngeal swab
CoV-19/Mexico/TAM_LANGEBIO_IMSS_0046/202	EPI_ISL_1351532	03/03/21	North America / Mexico / Jalisco	Human	Male	22	Hospitalized	Pharyngeal and Nasopharyngeal swab
CoV-19/Mexico/TAM_LANGEBIO_IMSS_0048/202	EPI_ISL_1351538	03/03/21	North America / Mexico / Jalisco	Human	Male	66	Hospitalized	Pharyngeal and Nasopharyngeal swab
CoV-19/Mexico/TAM_LANGEBIO_IMSS_0050/202	EPI_ISL_1351543	03/03/21	North America / Mexico / Jalisco	Human	Male	36	Ambulatory	Pharyngeal and Nasopharyngeal swab
CoV-19/Mexico/TAM_LANGEBIO_IMSS_0051/202	EPI_ISL_1351547	28/02/21	North America / Mexico / Sinaloa	Human	Female	29	Ambulatory	Pharyngeal and Nasopharyngeal swab
CoV-19/Mexico/TAM_LANGEBIO_IMSS_0054/202	EPI_ISL_1351555	02/03/21	North America / Mexico / Sinaloa	Human	Female	77	Hospitalized	Pharyngeal and Nasopharyngeal swab
CoV-19/Mexico/TAM_LANGEBIO_IMSS_0058/202	EPI_ISL_1351567	04/03/21	North America / Mexico / Jalisco	Human	Female	35	Ambulatory	Pharyngeal and Nasopharyngeal swab
CoV-19/Mexico/TAM_LANGEBIO_IMSS_0060/202	EPI_ISL_1351574	04/03/21	North America / Mexico / Jalisco	Human	Male	44	Hospitalized	Pharyngeal and Nasopharyngeal swab
CoV-19/Mexico/TAM_LANGEBIO_IMSS_0061/202	EPI_ISL_1351577	03/03/21	North America / Mexico / Michoacan	Human	Male	82	Hospitalized	Pharyngeal and Nasopharyngeal swab
CoV-19/Mexico/TAM_LANGEBIO_IMSS_0063/202	EPI_ISL_1351583	05/03/21	North America / Mexico / Jalisco	Human	Female	45	Ambulatory	Pharyngeal and Nasopharyngeal swab
CoV-19/Mexico/TAM_LANGEBIO_IMSS_0064/202	EPI_ISL_1351585	05/03/21	North America / Mexico / Jalisco	Human	Male	44	Ambulatory	Pharyngeal and Nasopharyngeal swab
CoV-19/Mexico/TAM_LANGEBIO_IMSS_0066/202	EPI_ISL_1351592	03/03/21	North America / Mexico / Jalisco	Human	Male	81	Hospitalized	Nasopharyngeal swab
CoV-19/Mexico/TAM_LANGEBIO_IMSS_0069/202	EPI_ISL_1351601	04/03/21	North America / Mexico / Jalisco	Human	Male	66	Hospitalized	Pharyngeal and Nasopharyngeal swab
CoV-19/Mexico/TAM_LANGEBIO_IMSS_0071/202	EPI_ISL_1351608	05/03/21	North America / Mexico / Jalisco	Human	Female	82	Hospitalized	Pharyngeal and Nasopharyngeal swab
CoV-19/Mexico/TAM_LANGEBIO_IMSS_0072/202	EPI_ISL_1351611	05/03/21	North America / Mexico / Jalisco	Human	Female	33	Ambulatory	Pharyngeal and Nasopharyngeal swab
CoV-19/Mexico/TAM_LANGEBIO_IMSS_0073/202	EPI_ISL_1351614	05/03/21	North America / Mexico / Jalisco	Human	Male	44	Hospitalized	Pharyngeal and Nasopharyngeal swab
CoV-19/Mexico/TAM_LANGEBIO_IMSS_0074/202	EPI_ISL_1351617	04/03/21	North America / Mexico / Sonora	Human	Female	58	Hospitalized	Pharyngeal and Nasopharyngeal swab
CoV-19/Mexico/TAM_LANGEBIO_IMSS_0076/202	EPI_ISL_1351623	04/03/21	North America / Mexico / Guanajuato	Human	Female	22	Ambulatory	Pharyngeal and Nasopharyngeal swab
CoV-19/Mexico/TAM_LANGEBIO_IMSS_0080/202	EPI_ISL_1351635	02/03/21	North America / Mexico / Sinaloa	Human	Female	33	Hospitalized	Nasopharyngeal swab
CoV-19/Mexico/TAM_LANGEBIO_IMSS_0081/202	EPI_ISL_1351638	03/03/21	North America / Mexico / Guanajuato	Human	Male	25	Ambulatory	Pharyngeal and Nasopharyngeal swab
CoV-19/Mexico/TAM_LANGEBIO_IMSS_0082/202	EPI_ISL_1351642	03/03/21	North America / Mexico / Guanajuato	Human	Male	59	Ambulatory	Pharyngeal and Nasopharyngeal swab
CoV-19/Mexico/TAM_LANGEBIO_IMSS_0083/202	EPI_ISL_1351645	04/03/21	North America / Mexico / Guanajuato	Human	Male	69	Ambulatory	Pharyngeal and Nasopharyngeal swab
CoV-19/Mexico/TAM_LANGEBIO_IMSS_0084/202	EPI_ISL_1351648	06/03/21	North America / Mexico / Jalisco	Human	Female	20	Ambulatory	Pharyngeal and Nasopharyngeal swab
CoV-19/Mexico/TAM_LANGEBIO_IMSS_0085/202	EPI_ISL_1351651	04/03/21	North America / Mexico / Jalisco	Human	Female	24	Ambulatory	Pharyngeal and Nasopharyngeal swab
CoV-19/Mexico/TAM_LANGEBIO_IMSS_0086/202	EPI_ISL_1351654	06/03/21	North America / Mexico / Jalisco	Human	Female	54	Hospitalized	Pharyngeal and Nasopharyngeal swab
CoV-19/Mexico/TAM_LANGEBIO_IMSS_0087/202	EPI_ISL_1351656	06/03/21	North America / Mexico / Jalisco	Human	Male	70	Hospitalized	Pharyngeal and Nasopharyngeal swab
CoV-19/Mexico/TAM_LANGEBIO_IMSS_0088/202	EPI_ISL_1351659	06/03/21	North America / Mexico / Jalisco	Human	Female	63	Hospitalized	Pharyngeal and Nasopharyngeal swab
CoV-19/Mexico/TAM_LANGEBIO_IMSS_0089/202	EPI_ISL_1351662	05/03/21	North America / Mexico / Guanajuato	Human	Male	70	Hospitalized	Pharyngeal and Nasopharyngeal swab
CoV-19/Mexico/TAM_LANGEBIO_IMSS_0090/202	EPI_ISL_1351665	04/03/21	North America / Mexico / Guanajuato	Human	Female	60	Ambulatory	Pharyngeal swab
CoV-19/Mexico/TAM_LANGEBIO_IMSS_0091/202	EPI_ISL_1351668	06/03/21	North America / Mexico / Guanajuato	Human	Male	31	Ambulatory	Pharyngeal and Nasopharyngeal swab
CoV-19/Mexico/TAM_LANGEBIO_IMSS_0092/202	EPI_ISL_1351670	05/03/21	North America / Mexico / Guanajuato	Human	Male	58	Ambulatory	Pharyngeal and Nasopharyngeal swab
CoV-19/Mexico/TAM_LANGEBIO_IMSS_0093/202	EPI_ISL_1351672	06/03/21	North America / Mexico / Jalisco	Human	Female	73	Hospitalized	Pharyngeal and Nasopharyngeal swab

CoV-19/Mexico/TAM_LANGEBIO_IMSS_0095/202	EPI_ISL_1351679	06/03/21	North America / Mexico / Jalisco	Human	Female	77	Deceased	Pharyngeal and Nasopharyngeal swab
CoV-19/Mexico/TAM_LANGEBIO_IMSS_0096/202	EPI_ISL_1351682	08/03/21	North America / Mexico / Jalisco	Human	Female	63	Ambulatory	Pharyngeal and Nasopharyngeal swab
CoV-19/Mexico/TAM_LANGEBIO_IMSS_0097/202	EPI_ISL_1351685	07/03/21	North America / Mexico / Jalisco	Human	Male	81	Hospitalized	Pharyngeal and Nasopharyngeal swab
CoV-19/Mexico/TAM_LANGEBIO_IMSS_0098/202	EPI_ISL_1351688	07/03/21	North America / Mexico / Nayarit	Human	Female	59	Hospitalized	Pharyngeal and Nasopharyngeal swab
CoV-19/Mexico/TAM_LANGEBIO_IMSS_0099/202	EPI_ISL_1351691	06/03/21	North America / Mexico / Guanajuato	Human	Male	34	Ambulatory	Pharyngeal and Nasopharyngeal swab
CoV-19/Mexico/TAM_LANGEBIO_IMSS_0101/202	EPI_ISL_1351697	06/03/21	North America / Mexico / Jalisco	Human	Female	67	Ambulatory	Pharyngeal and Nasopharyngeal swab
CoV-19/Mexico/TAM_LANGEBIO_IMSS_0104/202	EPI_ISL_1351706	08/03/21	North America / Mexico / Jalisco	Human	Male	63	Hospitalized	Pharyngeal and Nasopharyngeal swab
CoV-19/Mexico/TAM_LANGEBIO_IMSS_0106/202	EPI_ISL_1351713	03/03/21	North America / Mexico / San Luis Potosi	Human	Female	43	Ambulatory	Pharyngeal and Nasopharyngeal swab
CoV-19/Mexico/TAM_LANGEBIO_IMSS_0107/202	EPI_ISL_1351716	03/03/21	North America / Mexico / San Luis Potosi	Human	Female	78	Hospitalized	Pharyngeal and Nasopharyngeal swab
CoV-19/Mexico/TAM_LANGEBIO_IMSS_0108/202	EPI_ISL_1351719	03/03/21	North America / Mexico / San Luis Potosi	Human	Female	64	Hospitalized	Pharyngeal and Nasopharyngeal swab
CoV-19/Mexico/TAM_LANGEBIO_IMSS_0110/202	EPI_ISL_1351728	03/03/21	North America / Mexico / San Luis Potosi	Human	Female	67	Hospitalized	Pharyngeal and Nasopharyngeal swab
CoV-19/Mexico/TAM_LANGEBIO_IMSS_0111/202	EPI_ISL_1351731	05/03/21	North America / Mexico / Mexico City	Human	Male	53	Hospitalized	Pharyngeal and Nasopharyngeal swab
CoV-19/Mexico/TAM_LANGEBIO_IMSS_0112/202	EPI_ISL_1351734	04/03/21	North America / Mexico / Mexico City	Human	Male	29	Ambulatory	Pharyngeal and Nasopharyngeal swab
CoV-19/Mexico/TAM_LANGEBIO_IMSS_0113/202	EPI_ISL_1351737	04/03/21	North America / Mexico / Mexico City	Human	Male	25	Ambulatory	Pharyngeal and Nasopharyngeal swab
CoV-19/Mexico/TAM_LANGEBIO_IMSS_0114/202	EPI_ISL_1351739	04/03/21	North America / Mexico / Mexico City	Human	Male	50	Ambulatory	Pharyngeal and Nasopharyngeal swab
CoV-19/Mexico/TAM_LANGEBIO_IMSS_0116/202	EPI_ISL_1351746	04/03/21	North America / Mexico / Mexico City	Human	Male	68	Hospitalized	Pharyngeal and Nasopharyngeal swab
CoV-19/Mexico/TAM_LANGEBIO_IMSS_0117/202	EPI_ISL_1351749	04/03/21	North America / Mexico / Mexico City	Human	Male	42	Hospitalized	Bronchoalveolar lavage
CoV-19/Mexico/TAM_LANGEBIO_IMSS_0118/202	EPI_ISL_1351752	03/03/21	North America / Mexico / Mexico City	Human	Female	67	Hospitalized	Pharyngeal and Nasopharyngeal swab
CoV-19/Mexico/TAM_LANGEBIO_IMSS_0119/202	EPI_ISL_1351754	04/03/21	North America / Mexico / Mexico City	Human	Male	25	Ambulatory	Pharyngeal and Nasopharyngeal swab
CoV-19/Mexico/TAM_LANGEBIO_IMSS_0121/202	EPI_ISL_1351760	04/03/21	North America / Mexico / Mexico City	Human	Female	82	Ambulatory	Pharyngeal swab
CoV-19/Mexico/TAM_LANGEBIO_IMSS_0122/202	EPI_ISL_1351764	04/03/21	North America / Mexico / State of Mexico	Human	Female	50	Hospitalized	Nasopharyngeal swab
CoV-19/Mexico/TAM_LANGEBIO_IMSS_0124/202	EPI_ISL_1351770	04/03/21	North America / Mexico / Mexico City	Human	Male	49	Ambulatory	Pharyngeal swab
CoV-19/Mexico/TAM_LANGEBIO_IMSS_0125/202	EPI_ISL_1351774	05/03/21	North America / Mexico / Mexico City	Human	Female	47	Hospitalized	Pharyngeal swab
CoV-19/Mexico/TAM_LANGEBIO_IMSS_0126/202	EPI_ISL_1351777	04/03/21	North America / Mexico / State of Mexico	Human	Female	19	Hospitalized	Pharyngeal and Nasopharyngeal swab
CoV-19/Mexico/TAM_LANGEBIO_IMSS_0127/202	EPI_ISL_1351780	05/03/21	North America / Mexico / Mexico City	Human	Female	24	Ambulatory	Pharyngeal and Nasopharyngeal swab
CoV-19/Mexico/TAM_LANGEBIO_IMSS_0129/202	EPI_ISL_1351787	02/03/21	North America / Mexico / Veracruz	Human	Female	26	Ambulatory	Nasopharyngeal swab
CoV-19/Mexico/TAM_LANGEBIO_IMSS_0130/202	EPI_ISL_1351790	04/03/21	North America / Mexico / State of Mexico	Human	Female	49	Hospitalized	Pharyngeal and Nasopharyngeal swab
CoV-19/Mexico/TAM_LANGEBIO_IMSS_0132/202	EPI_ISL_1351796	04/03/21	North America / Mexico / State of Mexico	Human	Female	49	Ambulatory	Pharyngeal swab
CoV-19/Mexico/TAM_LANGEBIO_IMSS_0133/202	EPI_ISL_1351799	04/03/21	North America / Mexico / Puebla	Human	Male	17	Ambulatory	Pharyngeal and Nasopharyngeal swab
CoV-19/Mexico/TAM_LANGEBIO_IMSS_0134/202	EPI_ISL_1351802	04/03/21	North America / Mexico / Puebla	Human	Male	65	Ambulatory	Pharyngeal and Nasopharyngeal swab
CoV-19/Mexico/TAM_LANGEBIO_IMSS_0135/202	EPI_ISL_1351805	03/03/21	North America / Mexico / Puebla	Human	Female	33	Ambulatory	Pharyngeal and Nasopharyngeal swab
CoV-19/Mexico/TAM_LANGEBIO_IMSS_0136/202	EPI_ISL_1351809	04/03/21	North America / Mexico / Puebla	Human	Male	24	Ambulatory	Pharyngeal and Nasopharyngeal swab
CoV-19/Mexico/TAM_LANGEBIO_IMSS_0137/202	EPI_ISL_1351810	04/03/21	North America / Mexico / Puebla	Human	Female	33	Ambulatory	Pharyngeal and Nasopharyngeal swab
CoV-19/Mexico/TAM_LANGEBIO_IMSS_0138/202	EPI_ISL_1351811	03/03/21	North America / Mexico / Puebla	Human	Male	29	Ambulatory	Nasopharyngeal swab
CoV-19/Mexico/TAM_LANGEBIO_IMSS_0139/202	EPI_ISL_1351815	04/03/21	North America / Mexico / Puebla	Human	Female	52	Ambulatory	Pharyngeal and Nasopharyngeal swab
CoV-19/Mexico/TAM_LANGEBIO_IMSS_0140/202	EPI_ISL_1351818	04/03/21	North America / Mexico / Puebla	Human	Male	34	Ambulatory	Pharyngeal and Nasopharyngeal swab
CoV-19/Mexico/TAM_LANGEBIO_IMSS_0141/202	EPI_ISL_1351821	04/03/21	North America / Mexico / Puebla	Human	Female	46	Ambulatory	Pharyngeal and Nasopharyngeal swab
CoV-19/Mexico/TAM_LANGEBIO_IMSS_0143/202	EPI_ISL_1351827	03/03/21	North America / Mexico / Puebla	Human	Male	43	Ambulatory	Pharyngeal and Nasopharyngeal swab
CoV-19/Mexico/TAM_LANGEBIO_IMSS_0144/202	EPI_ISL_1351830	03/03/21	North America / Mexico / Puebla	Human	Male	35	Ambulatory	Pharyngeal and Nasopharyngeal swab
CoV-19/Mexico/TAM_LANGEBIO_IMSS_0145/202	EPI_ISL_1351833	02/03/21	North America / Mexico / Puebla	Human	Female	63	Ambulatory	Pharyngeal and Nasopharyngeal swab
CoV-19/Mexico/TAM_LANGEBIO_IMSS_0146/202	EPI_ISL_1351836	03/03/21	North America / Mexico / Puebla	Human	Male	15	Ambulatory	Pharyngeal and Nasopharyngeal swab
CoV-19/Mexico/TAM_LANGEBIO_IMSS_0147/202	EPI_ISL_1351839	02/03/21	North America / Mexico / Puebla	Human	Male	71	Hospitalized	Pharyngeal and Nasopharyngeal swab
CoV-19/Mexico/TAM_LANGEBIO_IMSS_0148/202	EPI_ISL_1351842	03/03/21	North America / Mexico / Puebla	Human	Female	70	Ambulatory	Pharyngeal and Nasopharyngeal swab
CoV-19/Mexico/TAM_LANGEBIO_IMSS_0149/202	EPI_ISL_1351845	02/03/21	North America / Mexico / Puebla	Human	Female	53	Hospitalized	Pharyngeal and Nasopharyngeal swab

CoV-19/Mexico/TAM_LANGEBIO_IMSS_0150/202	EPI_ISL_1351848	03/03/21	North America / Mexico / Puebla	Human	Female	47	Hospitalized	Pharyngeal and Nasopharyngeal swab
CoV-19/Mexico/TAM_LANGEBIO_IMSS_0151/202	EPI_ISL_1351851	03/03/21	North America / Mexico / Puebla	Human	Male	62	Hospitalized	Pharyngeal and Nasopharyngeal swab
CoV-19/Mexico/TAM_LANGEBIO_IMSS_0152/202	EPI_ISL_1351854	03/03/21	North America / Mexico / Puebla	Human	Male	48	Ambulatory	Pharyngeal and Nasopharyngeal swab
CoV-19/Mexico/TAM_LANGEBIO_IMSS_0153/202	EPI_ISL_1351857	03/03/21	North America / Mexico / Puebla	Human	Female	54	Ambulatory	Pharyngeal and Nasopharyngeal swab
CoV-19/Mexico/TAM_LANGEBIO_IMSS_0154/202	EPI_ISL_1351860	03/03/21	North America / Mexico / Puebla	Human	Female	26	Ambulatory	Pharyngeal and Nasopharyngeal swab
CoV-19/Mexico/TAM_LANGEBIO_IMSS_0155/202	EPI_ISL_1351863	04/03/21	North America / Mexico / Puebla	Human	Female	47	Ambulatory	Pharyngeal and Nasopharyngeal swab
CoV-19/Mexico/TAM_LANGEBIO_IMSS_0156/202	EPI_ISL_1351866	04/03/21	North America / Mexico / Puebla	Human	Male	72	Hospitalized	Pharyngeal and Nasopharyngeal swab
CoV-19/Mexico/TAM_LANGEBIO_IMSS_0158/202	EPI_ISL_1351872	01/03/21	North America / Mexico / Puebla	Human	Female	18	Ambulatory	Pharyngeal and Nasopharyngeal swab
CoV-19/Mexico/TAM_LANGEBIO_IMSS_0159/202	EPI_ISL_1351875	05/03/21	North America / Mexico / Tlaxcala	Human	Female	73	Ambulatory	Pharyngeal and Nasopharyngeal swab
CoV-19/Mexico/TAM_LANGEBIO_IMSS_0160/202	EPI_ISL_1351878	04/03/21	North America / Mexico / Tlaxcala	Human	Male	56	Hospitalized	Pharyngeal and Nasopharyngeal swab
CoV-19/Mexico/TAM_LANGEBIO_IMSS_0161/202	EPI_ISL_1351880	04/03/21	North America / Mexico / Tlaxcala	Human	Male	78	Ambulatory	Pharyngeal and Nasopharyngeal swab
CoV-19/Mexico/TAM_LANGEBIO_IMSS_0162/202	EPI_ISL_1351883	03/03/21	North America / Mexico / Tlaxcala	Human	Male	43	Hospitalized	Pharyngeal and Nasopharyngeal swab
CoV-19/Mexico/TAM_LANGEBIO_IMSS_0163/202	EPI_ISL_1351886	03/03/21	North America / Mexico / Tlaxcala	Human	Male	71	Hospitalized	Pharyngeal and Nasopharyngeal swab
CoV-19/Mexico/TAM_LANGEBIO_IMSS_0164/202	EPI_ISL_1351890	03/03/21	North America / Mexico / Veracruz	Human	Male	54	Hospitalized	Pharyngeal and Nasopharyngeal swab
CoV-19/Mexico/TAM_LANGEBIO_IMSS_0167/202	EPI_ISL_1351899	02/03/21	North America / Mexico / Veracruz	Human	Female	70	Hospitalized	Pharyngeal and Nasopharyngeal swab
CoV-19/Mexico/TAM_LANGEBIO_IMSS_0168/202	EPI_ISL_1351902	03/03/21	North America / Mexico / Oaxaca	Human	Female	77	Hospitalized	Pharyngeal and Nasopharyngeal swab
CoV-19/Mexico/TAM_LANGEBIO_IMSS_0169/202	EPI_ISL_1351905	03/03/21	North America / Mexico / Aguascalientes	Human	Male	45	Ambulatory	Pharyngeal and Nasopharyngeal swab
CoV-19/Mexico/TAM_LANGEBIO_IMSS_0170/202	EPI_ISL_1351908	01/03/21	North America / Mexico / Morelos	Human	Male	76	Hospitalized	Pharyngeal and Nasopharyngeal swab
CoV-19/Mexico/TAM_LANGEBIO_IMSS_0172/202	EPI_ISL_1351914	02/03/21	North America / Mexico / Morelos	Human	Female	45	Hospitalized	Pharyngeal and Nasopharyngeal swab
CoV-19/Mexico/TAM_LANGEBIO_IMSS_0175/202	EPI_ISL_1351924	03/03/21	North America / Mexico / Veracruz	Human	Female	68	Hospitalized	Pharyngeal and Nasopharyngeal swab
CoV-19/Mexico/TAM_LANGEBIO_IMSS_0176/202	EPI_ISL_1351927	04/03/21	North America / Mexico / Veracruz	Human	Female	57	Ambulatory	Pharyngeal and Nasopharyngeal swab
CoV-19/Mexico/TAM_LANGEBIO_IMSS_0177/202	EPI_ISL_1351930	03/03/21	North America / Mexico / Morelos	Human	Male	64	Hospitalized	Pharyngeal and Nasopharyngeal swab
CoV-19/Mexico/TAM_LANGEBIO_IMSS_0178/202	EPI_ISL_1351931	02/03/21	North America / Mexico / Morelos	Human	Male	55	Hospitalized	Pharyngeal and Nasopharyngeal swab
CoV-19/Mexico/TAM_LANGEBIO_IMSS_0180/202	EPI_ISL_1351935	03/03/21	North America / Mexico / Guerrero	Human	Male	79	Hospitalized	Pharyngeal and Nasopharyngeal swab
CoV-19/Mexico/TAM_LANGEBIO_IMSS_0182/202	EPI_ISL_1351941	05/03/21	North America / Mexico / State of Mexico	Human	Female	55	Hospitalized	Pharyngeal and Nasopharyngeal swab
CoV-19/Mexico/TAM_LANGEBIO_IMSS_0183/202	EPI_ISL_1351944	05/03/21	North America / Mexico / State of Mexico	Human	Female	53	Hospitalized	Pharyngeal and Nasopharyngeal swab
CoV-19/Mexico/TAM_LANGEBIO_IMSS_0184/202	EPI_ISL_1351947	05/03/21	North America / Mexico / State of Mexico	Human	Female	50	Hospitalized	Pharyngeal and Nasopharyngeal swab
CoV-19/Mexico/TAM_LANGEBIO_IMSS_0185/202	EPI_ISL_1351950	05/03/21	North America / Mexico / State of Mexico	Human	Male	21	Hospitalized	Pharyngeal and Nasopharyngeal swab
CoV-19/Mexico/TAM_LANGEBIO_IMSS_0187/202	EPI_ISL_1351952	04/03/21	North America / Mexico / Oaxaca	Human	Female	66	Hospitalized	Pharyngeal and Nasopharyngeal swab
CoV-19/Mexico/TAM_LANGEBIO_IMSS_0188/202	EPI_ISL_1351954	05/03/21	North America / Mexico / State of Mexico	Human	Female	43	Ambulatory	Pharyngeal and Nasopharyngeal swab
CoV-19/Mexico/TAM_LANGEBIO_IMSS_0189/202	EPI_ISL_1351956	03/03/21	North America / Mexico / San Luis Potosi	Human	Male	61	Hospitalized	Pharyngeal and Nasopharyngeal swab
CoV-19/Mexico/TAM_LANGEBIO_IMSS_0190/202	EPI_ISL_1351958	06/03/21	North America / Mexico / Mexico City	Human	Female	56	Hospitalized	Pharyngeal and Nasopharyngeal swab
CoV-19/Mexico/TAM_LANGEBIO_IMSS_0191/202	EPI_ISL_1351960	04/03/21	North America / Mexico / Mexico City	Human	Female	51	Ambulatory	Nasopharyngeal swab
CoV-19/Mexico/TAM_LANGEBIO_IMSS_0192/202	EPI_ISL_1351963	05/03/21	North America / Mexico / Mexico City	Human	Male	41	Hospitalized	Pharyngeal swab
CoV-19/Mexico/TAM_LANGEBIO_IMSS_0193/202	EPI_ISL_1351966	06/03/21	North America / Mexico / Mexico City	Human	Female	93	Ambulatory	Pharyngeal swab
CoV-19/Mexico/TAM_LANGEBIO_IMSS_0194/202	EPI_ISL_1351969	05/03/21	North America / Mexico / Mexico City	Human	Male	57	Ambulatory	Pharyngeal swab
CoV-19/Mexico/TAM_LANGEBIO_IMSS_0195/202	EPI_ISL_1351972	05/03/21	North America / Mexico / State of Mexico	Human	Male	66	Hospitalized	Pharyngeal and Nasopharyngeal swab
CoV-19/Mexico/TAM_LANGEBIO_IMSS_0196/202	EPI_ISL_1351975	02/03/21	North America / Mexico / Veracruz	Human	Male	24	Ambulatory	Nasopharyngeal swab
CoV-19/Mexico/TAM_LANGEBIO_IMSS_0198/202	EPI_ISL_1351981	05/03/21	North America / Mexico / Baja California Sur	Human	Male	23	Ambulatory	Pharyngeal and Nasopharyngeal swab
CoV-19/Mexico/TAM_LANGEBIO_IMSS_0199/202	EPI_ISL_1351984	05/03/21	North America / Mexico / Baja California Sur	Human	Male	20	Ambulatory	Pharyngeal and Nasopharyngeal swab
CoV-19/Mexico/TAM_LANGEBIO_IMSS_0200/202	EPI_ISL_1351987	05/03/21	North America / Mexico / Baja California Sur	Human	Male	39	Ambulatory	Pharyngeal and Nasopharyngeal swab
CoV-19/Mexico/TAM_LANGEBIO_IMSS_0201/202	EPI_ISL_1351990	05/03/21	North America / Mexico / Baja California Sur	Human	Male	29	Ambulatory	Pharyngeal and Nasopharyngeal swab
CoV-19/Mexico/TAM_LANGEBIO_IMSS_0202/202	EPI_ISL_1351993	04/03/21	North America / Mexico / Veracruz	Human	Male	60	Ambulatory	Pharyngeal and Nasopharyngeal swab
CoV-19/Mexico/TAM_LANGEBIO_IMSS_0203/202	EPI_ISL_1351997	04/03/21	North America / Mexico / Veracruz	Human	Female	34	Ambulatory	Pharyngeal and Nasopharyngeal swab

hCoV-19/Mexico/TAM_LANGEBIO_IMSS_0204/202	EPI_ISL_1352000	04/03/21	North America / Mexico / Veracruz	Human	Male	61	Ambulatory	Pharyngeal and Nasopharyngeal swab
hCoV-19/Mexico/TAM_LANGEBIO_IMSS_0206/202	EPI_ISL_1352006	05/03/21	North America / Mexico / Morelos	Human	Female	61	Hospitalized	Pharyngeal and Nasopharyngeal swab
hCoV-19/Mexico/TAM_LANGEBIO_IMSS_0207/202	EPI_ISL_1352009	04/03/21	North America / Mexico / Aguascalientes	Human	Female	25	Ambulatory	Pharyngeal and Nasopharyngeal swab
hCoV-19/Mexico/TAM_LANGEBIO_IMSS_0208/202	EPI_ISL_1352012	04/03/21	North America / Mexico / Aguascalientes	Human	Female	26	Ambulatory	Pharyngeal and Nasopharyngeal swab
hCoV-19/Mexico/TAM_LANGEBIO_IMSS_0209/202	EPI_ISL_1352015	05/03/21	North America / Mexico / Queretaro	Human	Male	69	Hospitalized	Pharyngeal and Nasopharyngeal swab
hCoV-19/Mexico/TAM_LANGEBIO_IMSS_0210/202	EPI_ISL_1352018	05/03/21	North America / Mexico / Queretaro	Human	Male	43	Hospitalized	Pharyngeal and Nasopharyngeal swab
hCoV-19/Mexico/TAM_LANGEBIO_IMSS_0211/202	EPI_ISL_1352020	05/03/21	North America / Mexico / Queretaro	Human	Male	42	Hospitalized	Pharyngeal and Nasopharyngeal swab
hCoV-19/Mexico/TAM_LANGEBIO_IMSS_0212/202	EPI_ISL_1352023	06/03/21	North America / Mexico / State of Mexico	Human	Female	56	Hospitalized	Nasopharyngeal swab
hCoV-19/Mexico/TAM_LANGEBIO_IMSS_0213/202	EPI_ISL_1352026	07/03/21	North America / Mexico / Mexico City	Human	Male	69	Hospitalized	Nasopharyngeal swab
hCoV-19/Mexico/TAM_LANGEBIO_IMSS_0214/202	EPI_ISL_1352029	07/03/21	North America / Mexico / Mexico City	Human	Female	76	Hospitalized	Nasopharyngeal swab
hCoV-19/Mexico/TAM_LANGEBIO_IMSS_0217/202	EPI_ISL_1352035	05/03/21	North America / Mexico / San Luis Potosi	Human	Male	63	Hospitalized	Pharyngeal and Nasopharyngeal swab
hCoV-19/Mexico/TAM_LANGEBIO_IMSS_0218/202	EPI_ISL_1352038	06/03/21	North America / Mexico / Hidalgo	Human	Female	67	Hospitalized	Pharyngeal and Nasopharyngeal swab
hCoV-19/Mexico/TAM_LANGEBIO_IMSS_0219/202	EPI_ISL_1352041	05/03/21	North America / Mexico / State of Mexico	Human	Male	65	Hospitalized	Pharyngeal and Nasopharyngeal swab
hCoV-19/Mexico/TAM_LANGEBIO_IMSS_0220/202	EPI_ISL_1352043	05/03/21	North America / Mexico / Mexico City	Human	Female	49	Hospitalized	Pharyngeal swab
hCoV-19/Mexico/TAM_LANGEBIO_IMSS_0221/202	EPI_ISL_1352047	06/03/21	North America / Mexico / Mexico City	Human	Female	74	Hospitalized	Pharyngeal swab
hCoV-19/Mexico/TAM_LANGEBIO_IMSS_0222/202	EPI_ISL_1352048	05/03/21	North America / Mexico / Baja California Sur	Human	Male	49	Hospitalized	Pharyngeal and Nasopharyngeal swab
hCoV-19/Mexico/TAM_LANGEBIO_IMSS_0223/202	EPI_ISL_1352050	08/03/21	North America / Mexico / Puebla	Human	Male	63	Hospitalized	Pharyngeal and Nasopharyngeal swab
hCoV-19/Mexico/TAM_LANGEBIO_IMSS_0224/202	EPI_ISL_1352051	06/03/21	North America / Mexico / Mexico City	Human	Female	69	Hospitalized	Pharyngeal and Nasopharyngeal swab
hCoV-19/Mexico/TAM_LANGEBIO_IMSS_0225/202	EPI_ISL_1352053	06/03/21	North America / Mexico / Mexico City	Human	Male	59	Ambulatory	Pharyngeal and Nasopharyngeal swab
hCoV-19/Mexico/TAM_LANGEBIO_IMSS_0226/202	EPI_ISL_1352055	08/03/21	North America / Mexico / Mexico City	Human	Female	71	Hospitalized	Nasopharyngeal swab
hCoV-19/Mexico/TAM_LANGEBIO_IMSS_0227/202	EPI_ISL_1352057	07/03/21	North America / Mexico / Mexico City	Human	Male	42	Hospitalized	Pharyngeal and Nasopharyngeal swab
hCoV-19/Mexico/TAM_LANGEBIO_IMSS_0228/202	EPI_ISL_1352059	07/03/21	North America / Mexico / Mexico City	Human	Female	83	Hospitalized	Pharyngeal and Nasopharyngeal swab
hCoV-19/Mexico/TAM_LANGEBIO_IMSS_0229/202	EPI_ISL_1352060	08/03/21	North America / Mexico / Mexico City	Human	Female	91	Hospitalized	Pharyngeal and Nasopharyngeal swab
hCoV-19/Mexico/TAM_LANGEBIO_IMSS_0230/202	EPI_ISL_1352062	03/03/21	North America / Mexico / Veracruz	Human	Male	73	Hospitalized	Pharyngeal and Nasopharyngeal swab
hCoV-19/Mexico/TAM_LANGEBIO_IMSS_0231/202	EPI_ISL_1352066	04/03/21	North America / Mexico / Veracruz	Human	Female	46	Ambulatory	Pharyngeal and Nasopharyngeal swab
hCoV-19/Mexico/TAM_LANGEBIO_IMSS_0232/202	EPI_ISL_1352068	06/03/21	North America / Mexico / Morelos	Human	Male	57	Ambulatory	Pharyngeal and Nasopharyngeal swab
hCoV-19/Mexico/TAM_LANGEBIO_IMSS_0233/202	EPI_ISL_1352070	04/03/21	North America / Mexico / San Luis Potosi	Human	Male	71	Hospitalized	Pharyngeal and Nasopharyngeal swab
hCoV-19/Mexico/TAM_LANGEBIO_IMSS_0234/202	EPI_ISL_1352072	04/03/21	North America / Mexico / San Luis Potosi	Human	Male	76	Hospitalized	Pharyngeal and Nasopharyngeal swab
hCoV-19/Mexico/TAM_LANGEBIO_IMSS_0235/202	EPI_ISL_1352076	07/03/21	North America / Mexico / Zacatecas	Human	Male	20	Ambulatory	Pharyngeal and Nasopharyngeal swab
hCoV-19/Mexico/TAM_LANGEBIO_IMSS_0236/202	EPI_ISL_1352079	05/03/21	North America / Mexico / Zacatecas	Human	Female	79	Ambulatory	Pharyngeal and Nasopharyngeal swab
hCoV-19/Mexico/TAM_LANGEBIO_IMSS_0237/202	EPI_ISL_1352083	06/03/21	North America / Mexico / Zacatecas	Human	Male	47	Hospitalized	Pharyngeal and Nasopharyngeal swab
hCoV-19/Mexico/TAM_LANGEBIO_IMSS_0238/202	EPI_ISL_1352085	05/03/21	North America / Mexico / Zacatecas	Human	Female	46	Ambulatory	Pharyngeal and Nasopharyngeal swab
hCoV-19/Mexico/GUA-InDRE_F10376_S662/2021	EPI_ISL_1359077	15/02/21	North America / Mexico / Guanajuato	Human	Female	20	Released	Oropharyngeal swab
hCoV-19/Mexico/GUA-InDRE_F10385_S667/2021	EPI_ISL_1359082	17/02/21	North America / Mexico / Guanajuato	Human	Female	60	Released	Oropharyngeal swab
hCoV-19/Mexico/GUA-InDRE_F10392_S671/2021	EPI_ISL_1359086	18/02/21	North America / Mexico / Guanajuato	Human	Male	43	Released	Oropharyngeal swab
hCoV-19/Mexico/MOR-InDRE_F10613_S685/2021	EPI_ISL_1365650	16/02/21	North America / Mexico / Morelos	Human	Male	67	Hospitalized	Oropharyngeal swab
hCoV-19/Mexico/MOR-InDRE_F10663_S690/2021	EPI_ISL_1365655	19/02/21	North America / Mexico / Morelos	Human	Male	71	Released	Oropharyngeal swab
hCoV-19/Mexico/VER-InDRE_F10678_S691/2021	EPI_ISL_1365656	15/02/21	North America / Mexico / Veracruz	Human	Male	24	Released	Oropharyngeal swab
hCoV-19/Mexico/CMX-InDRE_F10682_S692/2021	EPI_ISL_1365657	16/02/21	North America / Mexico / Mexico City	Human	Male	74	Deceased	Oropharyngeal swab
hCoV-19/Mexico/NLE-InDRE_F10811_S709/2021	EPI_ISL_1365674	23/02/21	North America / Mexico / Nuevo Leon	Human	Male	37	Released	Oropharyngeal swab
hCoV-19/Mexico/PUE-InDRE_F10413_S711/2021	EPI_ISL_1366660	21/02/21	North America / Mexico / Puebla	Human	Female	28	Released	Oropharyngeal swab
hCoV-19/Mexico/JAL-InDRE_F10432_S712/2021	EPI_ISL_1366661	16/02/21	North America / Mexico / Jalisco	Human	Male	78	Released	Oropharyngeal swab
hCoV-19/Mexico/JAL-InDRE_F10438_S713/2021	EPI_ISL_1366662	19/02/21	North America / Mexico / Jalisco	Human	Female	26	Released	Oropharyngeal swab
hCoV-19/Mexico/NLE-InDRE_F10502_S714/2021	EPI_ISL_1366663	15/02/21	North America / Mexico / Nuevo Leon	Human	Male	41	Hospitalized	Oropharyngeal swab

hCoV-19/Mexico/NLE-InDRE_F10511_S715/2021	EPI_ISL_1366664	17/02/21	North America / Mexico / Nuevo Leon	Human	Female	54	Released	Oropharyngeal swab
hCoV-19/Mexico/NLE-InDRE_F10523_S716/2021	EPI_ISL_1366665	16/02/21	North America / Mexico / Nuevo Leon	Human	Male	69	Released	Oropharyngeal swab
hCoV-19/Mexico/YUC-InDRE_F10540_S717/2021	EPI_ISL_1366666	19/02/21	North America / Mexico / Yucatan	Human	Female	74	Released	Oropharyngeal swab
hCoV-19/Mexico/QUE-InDRE_F10573_S718/2021	EPI_ISL_1366667	16/02/21	North America / Mexico / Queretaro	Human	Male	34	Released	Oropharyngeal swab
hCoV-19/Mexico/QUE-InDRE_F10577_S719/2021	EPI_ISL_1366668	16/02/21	North America / Mexico / Queretaro	Human	Male	50	Released	Oropharyngeal swab
hCoV-19/Mexico/QUE-InDRE_F10583_S720/2021	EPI_ISL_1366669	17/02/21	North America / Mexico / Queretaro	Human	Female	27	Released	Oropharyngeal swab
hCoV-19/Mexico/QUE-InDRE_F10598_S721/2021	EPI_ISL_1366670	19/02/21	North America / Mexico / Queretaro	Human	Female	56	Released	Oropharyngeal swab
hCoV-19/Mexico/QUE-InDRE_F10599_S722/2021	EPI_ISL_1366671	19/02/21	North America / Mexico / Queretaro	Human	Male	58	Released	Oropharyngeal swab
hCoV-19/Mexico/QUE-InDRE_F10602_S723/2021	EPI_ISL_1366672	20/02/21	North America / Mexico / Queretaro	Human	Male	38	Released	Oropharyngeal swab
hCoV-19/Mexico/QUE-InDRE_F10603_S724/2021	EPI_ISL_1366673	20/02/21	North America / Mexico / Queretaro	Human	Female	49	Released	Oropharyngeal swab
hCoV-19/Mexico/QUE-InDRE_F10607_S725/2021	EPI_ISL_1366674	21/02/21	North America / Mexico / Queretaro	Human	Female	29	Released	Oropharyngeal swab
hCoV-19/Mexico/QUE-InDRE_F10608_S726/2021	EPI_ISL_1366675	21/02/21	North America / Mexico / Queretaro	Human	Female	36	Hospitalized	Oropharyngeal swab
hCoV-19/Mexico/QUE-InDRE_F10614_S727/2021	EPI_ISL_1366676	23/02/21	North America / Mexico / Queretaro	Human	Male	34	Released	Oropharyngeal swab
hCoV-19/Mexico/MOR-InDRE_F10626_S728/2021	EPI_ISL_1366677	15/02/21	North America / Mexico / Morelos	Human	Female	74	Released	Oropharyngeal swab
hCoV-19/Mexico/MOR-InDRE_F10650_S729/2021	EPI_ISL_1366678	17/02/21	North America / Mexico / Morelos	Human	Male	76	Released	Oropharyngeal swab
hCoV-19/Mexico/MOR-InDRE_F10653_S730/2021	EPI_ISL_1366679	18/02/21	North America / Mexico / Morelos	Human	Female	28	Released	Oropharyngeal swab
hCoV-19/Mexico/MOR-InDRE_F10656_S731/2021	EPI_ISL_1366680	17/02/21	North America / Mexico / Morelos	Human	Male	83	Hospitalized	Oropharyngeal swab
hCoV-19/Mexico/MOR-InDRE_F10660_S732/2021	EPI_ISL_1366681	19/02/21	North America / Mexico / Morelos	Human	Male	17	Released	Oropharyngeal swab
hCoV-19/Mexico/VER-InDRE_F10672_S733/2021	EPI_ISL_1366682	19/02/21	North America / Mexico / Veracruz	Human	Female	75	Hospitalized	Oropharyngeal swab
hCoV-19/Mexico/VER-InDRE_F10673_S734/2021	EPI_ISL_1366683	20/02/21	North America / Mexico / Veracruz	Human	Male	45	Released	Oropharyngeal swab
hCoV-19/Mexico/VER-InDRE_F10674_S735/2021	EPI_ISL_1366684	20/02/21	North America / Mexico / Veracruz	Human	Female	70	Hospitalized	Oropharyngeal swab
hCoV-19/Mexico/VER-InDRE_F10679_S736/2021	EPI_ISL_1366685	16/02/21	North America / Mexico / Veracruz	Human	Female	77	Released	Oropharyngeal swab
hCoV-19/Mexico/VER-InDRE_F10681_S737/2021	EPI_ISL_1366686	21/02/21	North America / Mexico / Veracruz	Human	Female	74	Released	Oropharyngeal swab
hCoV-19/Mexico/CMX-InDRE_F10721_S738/2021	EPI_ISL_1366687	15/02/21	North America / Mexico / Mexico City	Human	Male	34	Released	Oropharyngeal swab
hCoV-19/Mexico/CMX-InDRE_F10724_S739/2021	EPI_ISL_1366688	15/02/21	North America / Mexico / Mexico City	Human	Male	45	Deceased	Oropharyngeal swab
hCoV-19/Mexico/CMX-InDRE_F10723_S740/2021	EPI_ISL_1366689	16/02/21	North America / Mexico / Mexico City	Human	Female	62	Released	Oropharyngeal swab
hCoV-19/Mexico/HID-InDRE_F10781_S741/2021	EPI_ISL_1366690	22/02/21	North America / Mexico / Hidalgo	Human	Female	50	Deceased	Oropharyngeal swab
hCoV-19/Mexico/HID-InDRE_F10786_S742/2021	EPI_ISL_1366691	23/02/21	North America / Mexico / Hidalgo	Human	Female	55	Released	Oropharyngeal swab
hCoV-19/Mexico/HID-InDRE_F10789_S743/2021	EPI_ISL_1366692	27/02/21	North America / Mexico / Hidalgo	Human	Male	47	Released	Oropharyngeal swab
hCoV-19/Mexico/NLE-InDRE_F10792_S744/2021	EPI_ISL_1366693	22/02/21	North America / Mexico / Nuevo Leon	Human	Male	51	Released	Oropharyngeal swab
hCoV-19/Mexico/NLE-InDRE_F10793_S745/2021	EPI_ISL_1366694	23/02/21	North America / Mexico / Nuevo Leon	Human	Female	46	Hospitalized	Oropharyngeal swab
hCoV-19/Mexico/NLE-InDRE_F10794_S746/2021	EPI_ISL_1366695	24/02/21	North America / Mexico / Nuevo Leon	Human	Female	36	Released	Oropharyngeal swab
hCoV-19/Mexico/NLE-InDRE_F10800_S747/2021	EPI_ISL_1366696	23/02/21	North America / Mexico / Nuevo Leon	Human	Male	66	Released	Oropharyngeal swab
hCoV-19/Mexico/NLE-InDRE_F10801_S748/2021	EPI_ISL_1366697	22/02/21	North America / Mexico / Nuevo Leon	Human	Female	60	Deceased	Oropharyngeal swab
hCoV-19/Mexico/NLE-InDRE_F10807_S749/2021	EPI_ISL_1366698	23/02/21	North America / Mexico / Nuevo Leon	Human	Male	52	Released	Oropharyngeal swab
hCoV-19/Mexico/NLE-InDRE_F10809_S750/2021	EPI_ISL_1366699	24/02/21	North America / Mexico / Nuevo Leon	Human	Female	52	Released	Oropharyngeal swab
hCoV-19/Mexico/GUA-InDRE_F10816_S751/2021	EPI_ISL_1366700	22/02/21	North America / Mexico / Guanajuato	Human	Male	65	Hospitalized	Oropharyngeal swab
hCoV-19/Mexico/MEX_LANGEBIO_IMSS_0239/202	EPI_ISL_1381234	06/03/21	North America / Mexico / State of Mexico	Human	Male	45	Ambulatory	Pharyngeal / Nasopharyngeal swab
hCoV-19/Mexico/CMX_LANGEBIO_IMSS_0240/202	EPI_ISL_1381235	05/03/21	North America / Mexico / Mexico City	Human	Male	78	Hospitalized	Nasopharyngeal swab
hCoV-19/Mexico/CMX_LANGEBIO_IMSS_0241/202	EPI_ISL_1381236	05/03/21	North America / Mexico / Mexico City	Human	Female	67	Hospitalized	Nasopharyngeal swab
hCoV-19/Mexico/CMX_LANGEBIO_IMSS_0242/202	EPI_ISL_1381237	05/03/21	North America / Mexico / Mexico City	Human	Female	61	Hospitalized	Nasopharyngeal swab
hCoV-19/Mexico/CMX_LANGEBIO_IMSS_0243/202	EPI_ISL_1381238	05/03/21	North America / Mexico / Mexico City	Human	Male	77	Hospitalized	Nasopharyngeal swab
hCoV-19/Mexico/CMX_LANGEBIO_IMSS_0244/202	EPI_ISL_1381239	05/03/21	North America / Mexico / Mexico City	Human	Male	39	Ambulatory	Pharyngeal swab
hCoV-19/Mexico/CMX_LANGEBIO_IMSS_0245/202	EPI_ISL_1381240	06/03/21	North America / Mexico / Mexico City	Human	Female	76	Hospitalized	Pharyngeal swab

ıCoV-19/Mexico/MEX_LANGEBIO_IMSS_0246/202	EPI_ISL_1381241	07/03/21	North America / Mexico / State of Mexico	Human	Female	63	Hospitalized	Pharyngeal / Nasopharyngeal swab
ıCoV-19/Mexico/MEX_LANGEBIO_IMSS_0247/202	EPI_ISL_1381242	07/03/21	North America / Mexico / State of Mexico	Human	Male	77	Hospitalized	Pharyngeal / Nasopharyngeal swab
ıCoV-19/Mexico/MEX_LANGEBIO_IMSS_0249/202	EPI_ISL_1381244	06/03/21	North America / Mexico / State of Mexico	Human	Male	52	Hospitalized	Pharyngeal / Nasopharyngeal swab
ıCoV-19/Mexico/CMX_LANGEBIO_IMSS_0250/202	EPI_ISL_1381245	07/03/21	North America / Mexico / Mexico City	Human	Male	78	Hospitalized	Nasopharyngeal swab
ıCoV-19/Mexico/CMX_LANGEBIO_IMSS_0251/202	EPI_ISL_1381246	07/03/21	North America / Mexico / Mexico City	Human	Male	59	Ambulatory	Pharyngeal swab
ıCoV-19/Mexico/MEX_LANGEBIO_IMSS_0252/202	EPI_ISL_1381247	04/03/21	North America / Mexico / State of Mexico	Human	Male	57	Ambulatory	Pharyngeal swab
ıCoV-19/Mexico/ZAC_LANGEBIO_IMSS_0255/202	EPI_ISL_1381250	04/03/21	North America / Mexico / Zacatecas	Human	Male	57	Ambulatory	Pharyngeal / Nasopharyngeal swab
ıCoV-19/Mexico/ZAC_LANGEBIO_IMSS_0256/202	EPI_ISL_1381251	04/03/21	North America / Mexico / Zacatecas	Human	Male	65	Ambulatory	Pharyngeal / Nasopharyngeal swab
ıCoV-19/Mexico/ZAC_LANGEBIO_IMSS_0257/202	EPI_ISL_1381252	04/03/21	North America / Mexico / Zacatecas	Human	Female	40	Ambulatory	Pharyngeal / Nasopharyngeal swab
ıCoV-19/Mexico/ZAC_LANGEBIO_IMSS_0259/202	EPI_ISL_1381254	04/03/21	North America / Mexico / Zacatecas	Human	Male	52	Ambulatory	Pharyngeal / Nasopharyngeal swab
ıCoV-19/Mexico/ZAC_LANGEBIO_IMSS_0260/202	EPI_ISL_1381255	03/03/21	North America / Mexico / Zacatecas	Human	Male	27	Ambulatory	Pharyngeal / Nasopharyngeal swab
ıCoV-19/Mexico/ZAC_LANGEBIO_IMSS_0263/202	EPI_ISL_1381258	02/03/21	North America / Mexico / Zacatecas	Human	Male	47	Ambulatory	Pharyngeal / Nasopharyngeal swab
hCoV-19/Mexico/CMX-INMEGEN-03-05-45/2021	EPI_ISL_1392922	01/03/21	North America / Mexico / Mexico City	Human	Female	68	unknown	
hCoV-19/Mexico/MIC-InDRE_F10346_S853/2021	EPI_ISL_1399259	17/02/21	North America / Mexico / Michoacan	Human	Male	65	Hospitalized	Oropharyngeal swab
hCoV-19/Mexico/GUA-InDRE_F10355_S856/2021	EPI_ISL_1399261	20/02/21	North America / Mexico / Guanajuato	Human	Female	60	Hospitalized	Oropharyngeal swab
hCoV-19/Mexico/MOR-InDRE_F10908_S864/2021	EPI_ISL_1399269	21/02/21	North America / Mexico / Morelos	Human	Male	64	Hospitalized	Oropharyngeal swab
hCoV-19/Mexico/CMX-InDRE_F11121_S877/2021	EPI_ISL_1399281	26/02/21	North America / Mexico / Mexico City	Human	Male	68	Released	Oropharyngeal swab
hCoV-19/Mexico/JAL-InDRE_F10948_S752/2021	EPI_ISL_1400298	21/02/21	North America / Mexico / Jalisco	Human	Female	66	Hospitalized	Oropharyngeal swab
hCoV-19/Mexico/CAM-InDRE_F10946_S753/2021	EPI_ISL_1400299	26/02/21	North America / Mexico / Campeche	Human	Female	54	Hospitalized	Oropharyngeal swab
hCoV-19/Mexico/MOR-InDRE_F10943_S754/2021	EPI_ISL_1400300	26/02/21	North America / Mexico / Morelos	Human	Male	71	Released	Oropharyngeal swab
hCoV-19/Mexico/MOR-InDRE_F10935_S755/2021	EPI_ISL_1400301	24/02/21	North America / Mexico / Morelos	Human	Male	58	Released	Oropharyngeal swab
hCoV-19/Mexico/MOR-InDRE_F10933_S756/2021	EPI_ISL_1400302	24/02/21	North America / Mexico / Morelos	Human	Male	63	Hospitalized	Oropharyngeal swab
hCoV-19/Mexico/MOR-InDRE_F10930_S757/2021	EPI_ISL_1400303	24/02/21	North America / Mexico / Morelos	Human	Male	67	Hospitalized	Oropharyngeal swab
hCoV-19/Mexico/GUA-InDRE_F10839_S758/2021	EPI_ISL_1400304	23/02/21	North America / Mexico / Guanajuato	Human	Female	17	Released	Oropharyngeal swab
hCoV-19/Mexico/MOR-InDRE_F10927_S759/2021	EPI_ISL_1400305	22/02/21	North America / Mexico / Morelos	Human	Male	46	Hospitalized	Oropharyngeal swab
hCoV-19/Mexico/MOR-InDRE_F10926_S760/2021	EPI_ISL_1400306	22/02/21	North America / Mexico / Morelos	Human	Female	33	Hospitalized	Oropharyngeal swab
hCoV-19/Mexico/MOR-InDRE_F10923_S761/2021	EPI_ISL_1400307	23/02/21	North America / Mexico / Morelos	Human	Male	31	Released	Oropharyngeal swab
hCoV-19/Mexico/MOR-InDRE_F10922_S762/2021	EPI_ISL_1400308	22/02/21	North America / Mexico / Morelos	Human	Male	45	Hospitalized	Oropharyngeal swab
hCoV-19/Mexico/MOR-InDRE_F10916_S763/2021	EPI_ISL_1400309	21/02/21	North America / Mexico / Morelos	Human	Female	47	Hospitalized	Oropharyngeal swab
hCoV-19/Mexico/PUE-InDRE_F10903_S765/2021	EPI_ISL_1400310	19/02/21	North America / Mexico / Puebla	Human	Male	48	Hospitalized	Oropharyngeal swab
hCoV-19/Mexico/PUE-InDRE_F10895_S767/2021	EPI_ISL_1400312	22/02/21	North America / Mexico / Puebla	Human	Female	86	Deceased	Oropharyngeal swab
hCoV-19/Mexico/PUE-InDRE_F10894_S768/2021	EPI_ISL_1400313	22/02/21	North America / Mexico / Puebla	Human	Male	36	Hospitalized	Oropharyngeal swab
hCoV-19/Mexico/GUA-InDRE_F10882_S769/2021	EPI_ISL_1400314	27/02/21	North America / Mexico / Guanajuato	Human	Female	27	Released	Oropharyngeal swab
hCoV-19/Mexico/GUA-InDRE_F10878_S770/2021	EPI_ISL_1400315	25/02/21	North America / Mexico / Guanajuato	Human	Female	58	Released	Oropharyngeal swab
hCoV-19/Mexico/GUA-InDRE_F10876_S772/2021	EPI_ISL_1400317	25/02/21	North America / Mexico / Guanajuato	Human	Male	60	Released	Oropharyngeal swab
hCoV-19/Mexico/GUA-InDRE_F10870_S773/2021	EPI_ISL_1400318	26/02/21	North America / Mexico / Guanajuato	Human	Female	30	Released	Oropharyngeal swab
hCoV-19/Mexico/GUA-InDRE_F10869_S774/2021	EPI_ISL_1400319	25/02/21	North America / Mexico / Guanajuato	Human	Female	53	Released	Oropharyngeal swab
hCoV-19/Mexico/GUA-InDRE_F10867_S775/2021	EPI_ISL_1400320	25/02/21	North America / Mexico / Guanajuato	Human	Female	29	Released	Oropharyngeal swab
hCoV-19/Mexico/GUA-InDRE_F10865_S776/2021	EPI_ISL_1400321	25/02/21	North America / Mexico / Guanajuato	Human	Male	33	Released	Oropharyngeal swab
hCoV-19/Mexico/GUA-InDRE_F10864_S777/2021	EPI_ISL_1400322	25/02/21	North America / Mexico / Guanajuato	Human	Female	60	Released	Oropharyngeal swab
hCoV-19/Mexico/GUA-InDRE_F10863_S778/2021	EPI_ISL_1400323	25/02/21	North America / Mexico / Guanajuato	Human	Male	42	Released	Oropharyngeal swab
hCoV-19/Mexico/GUA-InDRE_F10860_S779/2021	EPI_ISL_1400324	25/02/21	North America / Mexico / Guanajuato	Human	Female	61	Released	Oropharyngeal swab
hCoV-19/Mexico/GUA-InDRE_F10854_S783/2021	EPI_ISL_1400328	22/02/21	North America / Mexico / Guanajuato	Human	Female	57	Released	Oropharyngeal swab
hCoV-19/Mexico/GUA-InDRE_F10848_S784/2021	EPI_ISL_1400329	23/02/21	North America / Mexico / Guanajuato	Human	Male	33	Released	Oropharyngeal swab

hCoV-19/Mexico/GUA-InDRE_F10847_S785/2021	EPI_ISL_1400330	27/02/21	North America / Mexico / Guanajuato	Human	Female	57	Released	Oropharyngeal swab
hCoV-19/Mexico/GUA-InDRE_F10846_S786/2021	EPI_ISL_1400331	22/02/21	North America / Mexico / Guanajuato	Human	Female	60	Released	Oropharyngeal swab
hCoV-19/Mexico/GUA-InDRE_F10843_S788/2021	EPI_ISL_1400333	24/02/21	North America / Mexico / Guanajuato	Human	Female	31	Released	Oropharyngeal swab
hCoV-19/Mexico/GUA-InDRE_F10842_S789/2021	EPI_ISL_1400334	24/02/21	North America / Mexico / Guanajuato	Human	Male	11	Released	Oropharyngeal swab
hCoV-19/Mexico/GUA-InDRE_F10841_S790/2021	EPI_ISL_1400335	23/02/21	North America / Mexico / Guanajuato	Human	Female	49	Released	Oropharyngeal swab
hCoV-19/Mexico/GUA-InDRE_F10840_S791/2021	EPI_ISL_1400336	23/02/21	North America / Mexico / Guanajuato	Human	Male	46	Released	Oropharyngeal swab
hCoV-19/Mexico/GUA-InDRE_F10837_S792/2021	EPI_ISL_1400337	23/02/21	North America / Mexico / Guanajuato	Human	Female	18	Released	Oropharyngeal swab
hCoV-19/Mexico/GUA-InDRE_F10835_S793/2021	EPI_ISL_1400338	22/02/21	North America / Mexico / Guanajuato	Human	Female	53	Released	Oropharyngeal swab
hCoV-19/Mexico/GUA-InDRE_F10834_S794/2021	EPI_ISL_1400339	22/02/21	North America / Mexico / Guanajuato	Human	Female	70	Released	Oropharyngeal swab
hCoV-19/Mexico/GUA-InDRE_F10829_S796/2021	EPI_ISL_1400341	27/02/21	North America / Mexico / Guanajuato	Human	Female	66	Hospitalized	Oropharyngeal swab
hCoV-19/Mexico/GUA-InDRE_F10827_S797/2021	EPI_ISL_1400342	25/02/21	North America / Mexico / Guanajuato	Human	Male	71	Hospitalized	Oropharyngeal swab
hCoV-19/Mexico/GUA-InDRE_F10825_S798/2021	EPI_ISL_1400343	25/02/21	North America / Mexico / Guanajuato	Human	Female	87	Deceased	Oropharyngeal swab
hCoV-19/Mexico/GUA-InDRE_F10824_S799/2021	EPI_ISL_1400344	25/02/21	North America / Mexico / Guanajuato	Human	Female	55	Deceased	Oropharyngeal swab
hCoV-19/Mexico/GUA-InDRE_F10823_S800/2021	EPI_ISL_1400345	25/02/21	North America / Mexico / Guanajuato	Human	Male	69	Hospitalized	Oropharyngeal swab
hCoV-19/Mexico/GUA-InDRE_F10821_S801/2021	EPI_ISL_1400346	23/02/21	North America / Mexico / Guanajuato	Human	Female	59	Hospitalized	Oropharyngeal swab
hCoV-19/Mexico/GUA-InDRE_F10820_S802/2021	EPI_ISL_1400347	24/02/21	North America / Mexico / Guanajuato	Human	Male	78	Hospitalized	Oropharyngeal swab
hCoV-19/Mexico/CMX-INMEGEN-03-08-07/2021	EPI_ISL_1400795	01/03/21	North America / Mexico / Mexico City	Human	Female	49	unknown	Oropharyngeal swab
hCoV-19/Mexico/CMX-INMEGEN-03-08-32/2021	EPI_ISL_1400796	03/03/21	North America / Mexico / Mexico City	Human	Male	41	unknown	Oropharyngeal swab
hCoV-19/Mexico/CMX-INMEGEN-03-08-36/2021	EPI_ISL_1400797	03/03/21	North America / Mexico / Mexico City	Human	Male	34	unknown	Oropharyngeal swab
hCoV-19/Mexico/CMX-INMEGEN-03-08-66/2021	EPI_ISL_1400798	05/03/21	North America / Mexico / Mexico City	Human	Female	47	unknown	Oropharyngeal swab
hCoV-19/Mexico/CMX-INMEGEN-03-08-93/2021	EPI_ISL_1400799	05/03/21	North America / Mexico / Mexico City	Human	Female	34	unknown	Oropharyngeal swab
hCoV-19/Mexico/MOR-InDRE_F10942_S882/2021	EPI_ISL_1405899	25/02/21	North America / Mexico / Morelos	Human	Female	83	Hospitalized	Oropharyngeal swab
hCoV-19/Mexico/GUA-InDRE_F10866_S887/2021	EPI_ISL_1405904	25/02/21	North America / Mexico / Guanajuato	Human	Male	20	Released	Oropharyngeal swab
hCoV-19/Mexico/JAL-InDRE_F10958_S880/2021	EPI_ISL_1405911	24/02/21	North America / Mexico / Jalisco	Human	Female	64	Released	Oropharyngeal swab
hCoV-19/Mexico/CMX-INMEGEN-03-09-29/2021	EPI_ISL_1406083	11/03/21	North America / Mexico / Mexico City	Human	Male	21	unknown	Oropharyngeal swab
hCoV-19/Mexico/CMX-INMEGEN-03-09-30/2021	EPI_ISL_1406084	11/03/21	North America / Mexico / Mexico City	Human	Male	43	unknown	Oropharyngeal swab
hCoV-19/Mexico/CMX-INMEGEN-03-09-31/2021	EPI_ISL_1406085	11/03/21	North America / Mexico / Mexico City	Human	Male	63	unknown	Oropharyngeal swab
hCoV-19/Mexico/CMX-INMEGEN-03-09-33/2021	EPI_ISL_1406087	11/03/21	North America / Mexico / Mexico City	Human	Female	48	unknown	Oropharyngeal swab
hCoV-19/Mexico/CMX-INMEGEN-03-09-34/2021	EPI_ISL_1406088	11/03/21	North America / Mexico / Mexico City	Human	Female	25	unknown	Oropharyngeal swab
hCoV-19/Mexico/CMX-INMEGEN-03-09-35/2021	EPI_ISL_1406089	11/03/21	North America / Mexico / Mexico City	Human	Male	36	unknown	Oropharyngeal swab
hCoV-19/Mexico/CMX-INMEGEN-03-09-36/2021	EPI_ISL_1406090	11/03/21	North America / Mexico / Mexico City	Human	Male	59	unknown	Oropharyngeal swab
hCoV-19/Mexico/CMX-INMEGEN-03-09-37/2021	EPI_ISL_1406091	11/03/21	North America / Mexico / Mexico City	Human	Female	61	unknown	Oropharyngeal swab
hCoV-19/Mexico/CMX-INMEGEN-03-09-38/2021	EPI_ISL_1406092	11/03/21	North America / Mexico / Mexico City	Human	Female	22	unknown	Oropharyngeal swab
hCoV-19/Mexico/CMX-INMEGEN-03-09-39/2021	EPI_ISL_1406093	11/03/21	North America / Mexico / Mexico City	Human	Female	68	unknown	Oropharyngeal swab
hCoV-19/Mexico/CMX-INMEGEN-03-09-41/2021	EPI_ISL_1406094	11/03/21	North America / Mexico / Mexico City	Human	Female	56	unknown	Oropharyngeal swab
hCoV-19/Mexico/CMX-INMEGEN-03-09-42/2021	EPI_ISL_1406095	12/03/21	North America / Mexico / Mexico City	Human	Male	47	unknown	Oropharyngeal swab
hCoV-19/Mexico/CMX-INMEGEN-03-09-43/2021	EPI_ISL_1406096	12/03/21	North America / Mexico / Mexico City	Human	Male	81	unknown	Oropharyngeal swab
hCoV-19/Mexico/CMX-INMEGEN-03-09-44/2021	EPI_ISL_1406097	12/03/21	North America / Mexico / Mexico City	Human	Male	25	unknown	Oropharyngeal swab
hCoV-19/Mexico/CMX-INMEGEN-03-09-45/2021	EPI_ISL_1406098	12/03/21	North America / Mexico / Mexico City	Human	Male	58	unknown	Oropharyngeal swab
hCoV-19/Mexico/CMX-INMEGEN-03-09-46/2021	EPI_ISL_1406099	12/03/21	North America / Mexico / Mexico City	Human	Female	82	unknown	Oropharyngeal swab
hCoV-19/Mexico/CMX-INMEGEN-03-09-47/2021	EPI_ISL_1406100	12/03/21	North America / Mexico / Mexico City	Human	Male	55	unknown	Oropharyngeal swab
hCoV-19/Mexico/CMX-INMEGEN-03-09-48/2021	EPI_ISL_1406101	12/03/21	North America / Mexico / Mexico City	Human	Male	56	unknown	Oropharyngeal swab
hCoV-19/Mexico/CMX-INMEGEN-03-09-49/2021	EPI_ISL_1406102	12/03/21	North America / Mexico / Mexico City	Human	Male	34	unknown	Oropharyngeal swab
hCoV-19/Mexico/CMX-INMEGEN-03-09-50/2021	EPI_ISL_1406103	12/03/21	North America / Mexico / Mexico City	Human	Male	33	unknown	Oropharyngeal swab

hCoV-19/Mexico/CMX-INMEGEN-03-09-51/2021	EPI_ISL_1406104	12/03/21	North America / Mexico / Mexico City	Human	Male	59	unknown	Oropharyngeal swab
hCoV-19/Mexico/CMX-INMEGEN-03-09-52/2021	EPI_ISL_1406105	12/03/21	North America / Mexico / Mexico City	Human	Female	55	unknown	Oropharyngeal swab
hCoV-19/Mexico/CMX-INMEGEN-03-09-53/2021	EPI_ISL_1406106	12/03/21	North America / Mexico / Mexico City	Human	Female	59	unknown	Oropharyngeal swab
hCoV-19/Mexico/CMX-INMEGEN-03-09-54/2021	EPI_ISL_1406107	12/03/21	North America / Mexico / Mexico City	Human	Male	73	unknown	Oropharyngeal swab
hCoV-19/Mexico/CMX-INMEGEN-03-09-55/2021	EPI_ISL_1406108	12/03/21	North America / Mexico / Mexico City	Human	Male	71	unknown	Oropharyngeal swab
hCoV-19/Mexico/CMX-INMEGEN-03-09-57/2021	EPI_ISL_1406109	16/03/21	North America / Mexico / Mexico City	Human	Female	52	unknown	Oropharyngeal swab
hCoV-19/Mexico/CMX-INMEGEN-03-09-58/2021	EPI_ISL_1406110	16/03/21	North America / Mexico / Mexico City	Human	Female	39	unknown	Oropharyngeal swab
hCoV-19/Mexico/CMX-INMEGEN-03-09-59/2021	EPI_ISL_1406111	16/03/21	North America / Mexico / Mexico City	Human	Male	44	unknown	Oropharyngeal swab
hCoV-19/Mexico/CMX-INMEGEN-03-09-60/2021	EPI_ISL_1406112	16/03/21	North America / Mexico / Mexico City	Human	Male	59	unknown	Oropharyngeal swab
hCoV-19/Mexico/CMX-INMEGEN-03-09-61/2021	EPI_ISL_1406113	16/03/21	North America / Mexico / Mexico City	Human	Female	58	unknown	Oropharyngeal swab
hCoV-19/Mexico/CMX-INMEGEN-03-09-62/2021	EPI_ISL_1406114	16/03/21	North America / Mexico / Mexico City	Human	Female	54	unknown	Oropharyngeal swab
hCoV-19/Mexico/CMX-INMEGEN-03-09-63/2021	EPI_ISL_1406115	16/03/21	North America / Mexico / Mexico City	Human	Female	51	unknown	Oropharyngeal swab
hCoV-19/Mexico/CMX-INMEGEN-03-09-65/2021	EPI_ISL_1406116	16/03/21	North America / Mexico / Mexico City	Human	Female	90	unknown	Oropharyngeal swab
hCoV-19/Mexico/CMX-INMEGEN-03-09-66/2021	EPI_ISL_1406117	17/03/21	North America / Mexico / Mexico City	Human	Male	24	unknown	Oropharyngeal swab
hCoV-19/Mexico/CMX-INMEGEN-03-09-67/2021	EPI_ISL_1406118	17/03/21	North America / Mexico / Mexico City	Human	Male	56	unknown	Oropharyngeal swab
hCoV-19/Mexico/CMX-INMEGEN-03-09-68/2021	EPI_ISL_1406119	17/03/21	North America / Mexico / Mexico City	Human	Male	41	unknown	Oropharyngeal swab
hCoV-19/Mexico/CMX-INMEGEN-03-09-69/2021	EPI_ISL_1406120	17/03/21	North America / Mexico / Mexico City	Human	Male	45	unknown	Oropharyngeal swab
hCoV-19/Mexico/CMX-INMEGEN-03-09-70/2021	EPI_ISL_1406121	17/03/21	North America / Mexico / Mexico City	Human	Female	55	unknown	Oropharyngeal swab
hCoV-19/Mexico/CMX-INMEGEN-03-09-71/2021	EPI_ISL_1406122	17/03/21	North America / Mexico / Mexico City	Human	Female	66	unknown	Oropharyngeal swab
hCoV-19/Mexico/CMX-INMEGEN-03-09-73/2021	EPI_ISL_1406123	17/03/21	North America / Mexico / Mexico City	Human	Female	57	unknown	Oropharyngeal swab
hCoV-19/Mexico/CMX-INMEGEN-03-09-74/2021	EPI_ISL_1406124	17/03/21	North America / Mexico / Mexico City	Human	Female	50	unknown	Oropharyngeal swab
hCoV-19/Mexico/CMX-INMEGEN-03-09-75/2021	EPI_ISL_1406125	17/03/21	North America / Mexico / Mexico City	Human	Female	44	unknown	Oropharyngeal swab
hCoV-19/Mexico/CMX-INMEGEN-03-09-76/2021	EPI_ISL_1406126	15/02/21	North America / Mexico / Mexico City	Human	Male	55	unknown	Oropharyngeal swab
hCoV-19/Mexico/CMX-INMEGEN-03-09-77/2021	EPI_ISL_1406127	17/02/21	North America / Mexico / Mexico City	Human	Female	57	unknown	Oropharyngeal swab
hCoV-19/Mexico/CMX-INMEGEN-03-09-79/2021	EPI_ISL_1406128	17/02/21	North America / Mexico / Mexico City	Human	Male	40	unknown	Oropharyngeal swab
hCoV-19/Mexico/CMX-INMEGEN-03-09-80/2021	EPI_ISL_1406129	17/02/21	North America / Mexico / Mexico City	Human	Female	49	unknown	Oropharyngeal swab
hCoV-19/Mexico/CMX-INMEGEN-03-09-81/2021	EPI_ISL_1406130	17/02/21	North America / Mexico / Mexico City	Human	Female	16	unknown	Oropharyngeal swab
hCoV-19/Mexico/CMX-INMEGEN-03-09-82/2021	EPI_ISL_1406131	17/02/21	North America / Mexico / Mexico City	Human	Female	18	unknown	Oropharyngeal swab
hCoV-19/Mexico/CMX-INMEGEN-03-09-83/2021	EPI_ISL_1406132	17/02/21	North America / Mexico / Mexico City	Human	Female	3	unknown	Oropharyngeal swab
hCoV-19/Mexico/CMX-INMEGEN-03-09-84/2021	EPI_ISL_1406133	17/02/21	North America / Mexico / Mexico City	Human	Female	68	unknown	Oropharyngeal swab
hCoV-19/Mexico/CMX-INMEGEN-03-09-85/2021	EPI_ISL_1406134	17/02/21	North America / Mexico / Mexico City	Human	Female	52	unknown	Oropharyngeal swab
hCoV-19/Mexico/CMX-INMEGEN-03-09-86/2021	EPI_ISL_1406135	11/03/21	North America / Mexico / Mexico City	Human	Male	44	unknown	Oropharyngeal swab
hCoV-19/Mexico/CMX-INMEGEN-03-09-87/2021	EPI_ISL_1406136	12/03/21	North America / Mexico / Mexico City	Human	Female	22	unknown	Oropharyngeal swab
hCoV-19/Mexico/CMX-INMEGEN-03-09-88/2021	EPI_ISL_1406137	12/03/21	North America / Mexico / Mexico City	Human	Male	54	unknown	Oropharyngeal swab
hCoV-19/Mexico/CMX-INMEGEN-03-09-89/2021	EPI_ISL_1406138	18/03/21	North America / Mexico / Mexico City	Human	Male	23	unknown	Oropharyngeal swab
hCoV-19/Mexico/CMX-INMEGEN-03-09-90/2021	EPI_ISL_1406139	18/03/21	North America / Mexico / Mexico City	Human	Male	21	unknown	Oropharyngeal swab
hCoV-19/Mexico/CMX-INMEGEN-03-09-91/2021	EPI_ISL_1406140	18/03/21	North America / Mexico / Mexico City	Human	Male	45	unknown	Oropharyngeal swab
hCoV-19/Mexico/CMX-INMEGEN-03-09-92/2021	EPI_ISL_1406141	18/03/21	North America / Mexico / Mexico City	Human	Male	25	unknown	Oropharyngeal swab
hCoV-19/Mexico/CMX-INMEGEN-03-09-93/2021	EPI_ISL_1406142	18/03/21	North America / Mexico / Mexico City	Human	Male	52	unknown	Oropharyngeal swab
hCoV-19/Mexico/CMX-INER-INMEGEN-00001/2021	EPI_ISL_1406722	24/02/21	North America / Mexico / Mexico City	Human	Male	10	unknown	
hCoV-19/Mexico/CMX-INER-INMEGEN-00002/2021	EPI_ISL_1406723	24/02/21	North America / Mexico / Mexico City	Human	Female	69	unknown	
hCoV-19/Mexico/CMX-INER-INMEGEN-00003/2021	EPI_ISL_1406724	24/02/21	North America / Mexico / Mexico City	Human	Male	29	unknown	
hCoV-19/Mexico/CMX-INER-INMEGEN-00005/2021	EPI_ISL_1406725	25/02/21	North America / Mexico / Mexico City	Human	Female	36	unknown	
hCoV-19/Mexico/CMX-INER-INMEGEN-00006/2021	EPI_ISL_1406726	25/02/21	North America / Mexico / Mexico City	Human	Female	35	unknown	

hCoV-19/Mexico/CMX-INER-INMEGEN-00008/2021	EPI_ISL_1406727	27/02/21	North America / Mexico / Mexico City	Human	Male	55	unknown
hCoV-19/Mexico/CMX-INER-INMEGEN-00009/2021	EPI_ISL_1406728	28/02/21	North America / Mexico / Mexico City	Human	Male	13	unknown
hCoV-19/Mexico/CMX-INER-INMEGEN-00010/2021	EPI_ISL_1406729	27/02/21	North America / Mexico / Mexico City	Human	Female	38	unknown
hCoV-19/Mexico/CMX-INER-INMEGEN-00011/2021	EPI_ISL_1406730	26/02/21	North America / Mexico / Mexico City	Human	Male	62	unknown
hCoV-19/Mexico/CMX-INER-INMEGEN-00012/2021	EPI_ISL_1406731	27/02/21	North America / Mexico / Mexico City	Human	Male	68	unknown
hCoV-19/Mexico/CMX-INER-INMEGEN-00013/2021	EPI_ISL_1406732	27/02/21	North America / Mexico / Mexico City	Human	Female	30	unknown
hCoV-19/Mexico/CMX-INER-INMEGEN-00014/2021	EPI_ISL_1406733	01/03/21	North America / Mexico / Mexico City	Human	Female	36	unknown
hCoV-19/Mexico/CMX-INER-INMEGEN-00015/2021	EPI_ISL_1406734	02/03/21	North America / Mexico / Mexico City	Human	Female	44	unknown
hCoV-19/Mexico/CMX-INER-INMEGEN-00016/2021	EPI_ISL_1406735	02/03/21	North America / Mexico / Mexico City	Human	Female	31	unknown
hCoV-19/Mexico/CMX-INER-INMEGEN-00017/2021	EPI_ISL_1406736	02/03/21	North America / Mexico / Mexico City	Human	Male	59	unknown
hCoV-19/Mexico/CMX-INER-INMEGEN-00018/2021	EPI_ISL_1406737	02/03/21	North America / Mexico / Mexico City	Human	Male	22	unknown
hCoV-19/Mexico/CMX-INER-INMEGEN-00020/2021	EPI_ISL_1406738	02/03/21	North America / Mexico / Mexico City	Human	Female	41	unknown
hCoV-19/Mexico/CMX-INER-INMEGEN-00021/2021	EPI_ISL_1406739	02/03/21	North America / Mexico / Mexico City	Human	Female	38	unknown
hCoV-19/Mexico/CMX-INER-INMEGEN-00022/2021	EPI_ISL_1406740	03/03/21	North America / Mexico / Mexico City	Human	Female	53	unknown
hCoV-19/Mexico/CMX-INER-INMEGEN-00023/2021	EPI_ISL_1406741	03/03/21	North America / Mexico / Mexico City	Human	Female	26	unknown
hCoV-19/Mexico/CMX-INER-INMEGEN-00025/2021	EPI_ISL_1406743	03/03/21	North America / Mexico / Mexico City	Human	Female	22	unknown
hCoV-19/Mexico/CMX-INER-INMEGEN-00026/2021	EPI_ISL_1406744	03/03/21	North America / Mexico / Mexico City	Human	Male	65	unknown
hCoV-19/Mexico/CMX-INER-INMEGEN-00027/2021	EPI_ISL_1406745	03/03/21	North America / Mexico / Mexico City	Human	Female	36	unknown
hCoV-19/Mexico/CMX-INER-INMEGEN-00028/2021	EPI_ISL_1406746	04/03/21	North America / Mexico / Mexico City	Human	Female	64	unknown
hCoV-19/Mexico/CMX-INER-INMEGEN-00031/2021	EPI_ISL_1406747	03/03/21	North America / Mexico / Mexico City	Human	Male	22	unknown
hCoV-19/Mexico/CMX-INER-INMEGEN-00033/2021	EPI_ISL_1406748	03/03/21	North America / Mexico / Mexico City	Human	Male	33	unknown
hCoV-19/Mexico/CMX-INER-INMEGEN-00034/2021	EPI_ISL_1406749	03/03/21	North America / Mexico / Mexico City	Human	Female	22	unknown
hCoV-19/Mexico/CMX-INER-INMEGEN-00035/2021	EPI_ISL_1406750	03/03/21	North America / Mexico / Mexico City	Human	Male	23	unknown
hCoV-19/Mexico/CMX-INER-INMEGEN-00036/2021	EPI_ISL_1406751	11/02/21	North America / Mexico / Mexico City	Human	Male	22	unknown
hCoV-19/Mexico/CMX-INER-INMEGEN-00037/2021	EPI_ISL_1406752	17/02/21	North America / Mexico / Mexico City	Human	Female	57	unknown
hCoV-19/Mexico/CMX-INER-INMEGEN-00038/2021	EPI_ISL_1406753	17/02/21	North America / Mexico / Mexico City	Human	Female	49	unknown
hCoV-19/Mexico/CMX-INER-INMEGEN-00039/2021	EPI_ISL_1406754	17/02/21	North America / Mexico / Mexico City	Human	Male	40	unknown
hCoV-19/Mexico/CMX-INER-INMEGEN-00040/2021	EPI_ISL_1406755	17/02/21	North America / Mexico / Mexico City	Human	Female	18	unknown
hCoV-19/Mexico/CMX-INER-INMEGEN-00041/2021	EPI_ISL_1406756	17/02/21	North America / Mexico / Mexico City	Human	Female	43	unknown
hCoV-19/Mexico/CMX-INER-INMEGEN-00043/2021	EPI_ISL_1406757	17/02/21	North America / Mexico / Mexico City	Human	Male	34	unknown
hCoV-19/Mexico/CMX-INER-INMEGEN-00044/2021	EPI_ISL_1406758	17/02/21	North America / Mexico / Mexico City	Human	Female	36	unknown
hCoV-19/Mexico/CMX-INER-INMEGEN-00045/2021	EPI_ISL_1406759	18/02/21	North America / Mexico / Mexico City	Human	Female	45	unknown
hCoV-19/Mexico/CMX-INER-INMEGEN-00046/2021	EPI_ISL_1406760	19/02/21	North America / Mexico / Mexico City	Human	Female	15	unknown
hCoV-19/Mexico/CMX-INER-INMEGEN-00047/2021	EPI_ISL_1406761	19/02/21	North America / Mexico / Mexico City	Human	Male	10	unknown
hCoV-19/Mexico/CMX-INER-INMEGEN-00048/2021	EPI_ISL_1406762	19/02/21	North America / Mexico / Mexico City	Human	Female	18	unknown
hCoV-19/Mexico/CMX-INER-INMEGEN-00050/2021	EPI_ISL_1406763	21/02/21	North America / Mexico / Mexico City	Human	Female	50	unknown
hCoV-19/Mexico/CMX-INER-INMEGEN-00051/2021	EPI_ISL_1406764	21/02/21	North America / Mexico / Mexico City	Human	Male	20	unknown
hCoV-19/Mexico/CMX-INER-INMEGEN-00052/2021	EPI_ISL_1406765	22/02/21	North America / Mexico / Mexico City	Human	Male	27	unknown
hCoV-19/Mexico/CMX-INER-INMEGEN-00053/2021	EPI_ISL_1406766	22/02/21	North America / Mexico / Mexico City	Human	Female	20	unknown
hCoV-19/Mexico/CMX-INER-INMEGEN-00054/2021	EPI_ISL_1406767	23/02/21	North America / Mexico / Mexico City	Human	Male	46	unknown
hCoV-19/Mexico/CMX-INER-INMEGEN-00055/2021	EPI_ISL_1406768	23/02/21	North America / Mexico / Mexico City	Human	Male	56	unknown
hCoV-19/Mexico/CMX-INER-INMEGEN-00056/2021	EPI_ISL_1406769	23/02/21	North America / Mexico / Mexico City	Human	Female	74	unknown
hCoV-19/Mexico/CMX-INER-INMEGEN-00057/2021	EPI_ISL_1406770	23/02/21	North America / Mexico / Mexico City	Human	Male	37	unknown
hCoV-19/Mexico/CMX-INER-INMEGEN-00058/2021	EPI_ISL_1406771	23/02/21	North America / Mexico / Mexico City	Human	Female	70	unknown

hCoV-19/Mexico/CMX-INER-INMEGEN-00059/2021	EPI_ISL_1406772	23/02/21	North America / Mexico / Mexico City	Human	Female	83	unknown
hCoV-19/Mexico/CMX-INER-INMEGEN-00060/2021	EPI_ISL_1406773	23/02/21	North America / Mexico / Mexico City	Human	Male	59	unknown
hCoV-19/Mexico/CMX-INER-INMEGEN-00061/2021	EPI_ISL_1406774	23/02/21	North America / Mexico / Mexico City	Human	Female	50	unknown
hCoV-19/Mexico/CMX-INER-INMEGEN-00062/2021	EPI_ISL_1406775	24/02/21	North America / Mexico / Mexico City	Human	Female	21	unknown
hCoV-19/Mexico/CMX-INER-INMEGEN-00063/2021	EPI_ISL_1406776	25/02/21	North America / Mexico / Mexico City	Human	Female	39	unknown
hCoV-19/Mexico/CMX-INER-INMEGEN-00064/2021	EPI_ISL_1406777	01/03/21	North America / Mexico / Mexico City	Human	Female	24	unknown
hCoV-19/Mexico/QUE-IBT-IMSS-684/2021	EPI_ISL_1416332	05/03/21	North America / Mexico / Queretaro	Human	Male	53	Ambulatory
hCoV-19/Mexico/QUE-IBT-IMSS-889/2021	EPI_ISL_1416333	06/03/21	North America / Mexico / Queretaro	Human	Male	83	Hospitalized
hCoV-19/Mexico/QUE-IBT-IMSS-903/2021	EPI_ISL_1416334	06/03/21	North America / Mexico / Queretaro	Human	Male	64	Hospitalized
hCoV-19/Mexico/MEX-IBT-IMSS-914/2021	EPI_ISL_1416335	06/03/21	North America / Mexico / State of Mexico	Human	Male	73	Hospitalized
hCoV-19/Mexico/MEX-IBT-IMSS-925/2021	EPI_ISL_1416336	07/03/21	North America / Mexico / State of Mexico	Human	Female	65	Hospitalized
hCoV-19/Mexico/CMX-IBT-IMSS-933/2021	EPI_ISL_1416337	07/03/21	North America / Mexico / Mexico City	Human	Male	26	Ambulatory
hCoV-19/Mexico/MEX-IBT-IMSS-953/2021	EPI_ISL_1416338	03/03/21	North America / Mexico / State of Mexico	Human	Male	55	Ambulatory
hCoV-19/Mexico/PUE-IBT-IMSS-608/2021	EPI_ISL_1416339	05/03/21	North America / Mexico / Puebla	Human	Female	30	Ambulatory
hCoV-19/Mexico/PUE-IBT-IMSS-624/2021	EPI_ISL_1416340	05/03/21	North America / Mexico / Puebla	Human	Female	60	Ambulatory
hCoV-19/Mexico/PUE-IBT-IMSS-643/2021	EPI_ISL_1416341	05/03/21	North America / Mexico / Puebla	Human	Female	22	Ambulatory
hCoV-19/Mexico/PUE-IBT-IMSS-672/2021	EPI_ISL_1416342	04/03/21	North America / Mexico / Puebla	Human	Female	54	Ambulatory
hCoV-19/Mexico/PUE-IBT-IMSS-695/2021	EPI_ISL_1416344	04/03/21	North America / Mexico / Puebla	Human	Male	50	Ambulatory
hCoV-19/Mexico/PUE-IBT-IMSS-716/2021	EPI_ISL_1416346	05/03/21	North America / Mexico / Puebla	Human	Female	48	Ambulatory
hCoV-19/Mexico/PUE-IBT-IMSS-725/2021	EPI_ISL_1416347	04/03/21	North America / Mexico / Puebla	Human	Female	22	Ambulatory
hCoV-19/Mexico/PUE-IBT-IMSS-736/2021	EPI_ISL_1416348	04/03/21	North America / Mexico / Puebla	Human	Female	42	Ambulatory
hCoV-19/Mexico/PUE-IBT-IMSS-747/2021	EPI_ISL_1416349	04/03/21	North America / Mexico / Puebla	Human	Female	42	Ambulatory
hCoV-19/Mexico/PUE-IBT-IMSS-757/2021	EPI_ISL_1416350	04/03/21	North America / Mexico / Puebla	Human	Male	55	Ambulatory
hCoV-19/Mexico/PUE-IBT-IMSS-768/2021	EPI_ISL_1416351	04/03/21	North America / Mexico / Puebla	Human	Male	39	Ambulatory
hCoV-19/Mexico/PUE-IBT-IMSS-788/2021	EPI_ISL_1416353	05/03/21	North America / Mexico / Puebla	Human	Female	92	Hospitalized
hCoV-19/Mexico/PUE-IBT-IMSS-800/2021	EPI_ISL_1416354	05/03/21	North America / Mexico / Puebla	Human	Female	62	Hospitalized
hCoV-19/Mexico/PUE-IBT-IMSS-811/2021	EPI_ISL_1416355	06/03/21	North America / Mexico / Puebla	Human	Female	52	Ambulatory
hCoV-19/Mexico/PUE-IBT-IMSS-820/2021	EPI_ISL_1416356	07/03/21	North America / Mexico / Puebla	Human	Female	56	Hospitalized
hCoV-19/Mexico/PUE-IBT-IMSS-837/2021	EPI_ISL_1416358	07/03/21	North America / Mexico / Puebla	Human	Male	49	Ambulatory
hCoV-19/Mexico/PUE-IBT-IMSS-847/2021	EPI_ISL_1416359	05/03/21	North America / Mexico / Puebla	Human	Female	35	Ambulatory
hCoV-19/Mexico/PUE-IBT-IMSS-857/2021	EPI_ISL_1416360	05/03/21	North America / Mexico / Puebla	Human	Female	45	Ambulatory
hCoV-19/Mexico/PUE-IBT-IMSS-868/2021	EPI_ISL_1416361	05/03/21	North America / Mexico / Puebla	Human	Male	43	Ambulatory
hCoV-19/Mexico/PUE-IBT-IMSS-877/2021	EPI_ISL_1416362	06/03/21	North America / Mexico / Puebla	Human	Male	37	Ambulatory
hCoV-19/Mexico/PUE-IBT-IMSS-888/2021	EPI_ISL_1416363	05/03/21	North America / Mexico / Puebla	Human	Female	28	Ambulatory
hCoV-19/Mexico/PUE-IBT-IMSS-895/2021	EPI_ISL_1416364	06/03/21	North America / Mexico / Puebla	Human	Male	28	Ambulatory
hCoV-19/Mexico/PUE-IBT-IMSS-896/2021	EPI_ISL_1416365	05/03/21	North America / Mexico / Puebla	Human	Male	32	Ambulatory
hCoV-19/Mexico/PUE-IBT-IMSS-897/2021	EPI_ISL_1416366	05/03/21	North America / Mexico / Puebla	Human	Female	46	Ambulatory
hCoV-19/Mexico/PUE-IBT-IMSS-898/2021	EPI_ISL_1416367	05/03/21	North America / Mexico / Puebla	Human	Male	69	Ambulatory
hCoV-19/Mexico/PUE-IBT-IMSS-899/2021	EPI_ISL_1416368	05/03/21	North America / Mexico / Puebla	Human	Male	42	Ambulatory
hCoV-19/Mexico/PUE-IBT-IMSS-901/2021	EPI_ISL_1416370	05/03/21	North America / Mexico / Puebla	Human	Male	50	Ambulatory
hCoV-19/Mexico/PUE-IBT-IMSS-902/2021	EPI_ISL_1416371	05/03/21	North America / Mexico / Puebla	Human	Female	36	Ambulatory
hCoV-19/Mexico/PUE-IBT-IMSS-904/2021	EPI_ISL_1416372	04/03/21	North America / Mexico / Puebla	Human	Male	72	Ambulatory
hCoV-19/Mexico/PUE-IBT-IMSS-906/2021	EPI_ISL_1416374	05/03/21	North America / Mexico / Puebla	Human	Male	25	Ambulatory
hCoV-19/Mexico/PUE-IBT-IMSS-907/2021	EPI_ISL_1416375	04/03/21	North America / Mexico / Puebla	Human	Male	44	Ambulatory

hCoV-19/Mexico/PUE-IBT-IMSS-908/2021	EPI_ISL_1416376	05/03/21	North America / Mexico / Puebla	Human	Female	48	Ambulatory
hCoV-19/Mexico/PUE-IBT-IMSS-909/2021	EPI_ISL_1416377	04/03/21	North America / Mexico / Puebla	Human	Female	30	Ambulatory
hCoV-19/Mexico/PUE-IBT-IMSS-910/2021	EPI_ISL_1416378	04/03/21	North America / Mexico / Puebla	Human	Male	46	Ambulatory
hCoV-19/Mexico/PUE-IBT-IMSS-911/2021	EPI_ISL_1416379	04/03/21	North America / Mexico / Puebla	Human	Female	32	Ambulatory
hCoV-19/Mexico/PUE-IBT-IMSS-912/2021	EPI_ISL_1416380	04/03/21	North America / Mexico / Puebla	Human	Male	48	Ambulatory
hCoV-19/Mexico/PUE-IBT-IMSS-913/2021	EPI_ISL_1416381	04/03/21	North America / Mexico / Puebla	Human	Male	30	Ambulatory
hCoV-19/Mexico/MEX-IBT-IMSS-915/2021	EPI_ISL_1416382	07/03/21	North America / Mexico / State of Mexico	Human	Male	68	Hospitalized
hCoV-19/Mexico/MEX-IBT-IMSS-916/2021	EPI_ISL_1416383	07/03/21	North America / Mexico / State of Mexico	Human	Male	48	Hospitalized
hCoV-19/Mexico/MEX-IBT-IMSS-917/2021	EPI_ISL_1416384	08/03/21	North America / Mexico / State of Mexico	Human	Female	31	Ambulatory
hCoV-19/Mexico/SLP-IBT-IMSS-918/2021	EPI_ISL_1416385	08/03/21	North America / Mexico / San Luis Potosi	Human	Female	22	Ambulatory
hCoV-19/Mexico/SLP-IBT-IMSS-919/2021	EPI_ISL_1416386	08/03/21	North America / Mexico / San Luis Potosi	Human	Female	63	Hospitalized
hCoV-19/Mexico/SLP-IBT-IMSS-920/2021	EPI_ISL_1416387	08/03/21	North America / Mexico / San Luis Potosi	Human	Female	43	Hospitalized
hCoV-19/Mexico/MEX-IBT-IMSS-921/2021	EPI_ISL_1416388	07/03/21	North America / Mexico / State of Mexico	Human	Female	58	Hospitalized
hCoV-19/Mexico/MEX-IBT-IMSS-922/2021	EPI_ISL_1416389	04/03/21	North America / Mexico / State of Mexico	Human	Female	84	Hospitalized
hCoV-19/Mexico/MEX-IBT-IMSS-923/2021	EPI_ISL_1416390	09/03/21	North America / Mexico / State of Mexico	Human	Male	80	Ambulatory
hCoV-19/Mexico/CMX-IBT-IMSS-924/2021	EPI_ISL_1416391	07/03/21	North America / Mexico / Mexico City	Human	Female	49	Ambulatory
hCoV-19/Mexico/CMX-IBT-IMSS-926/2021	EPI_ISL_1416392	07/03/21	North America / Mexico / Mexico City	Human	Female	51	Ambulatory
hCoV-19/Mexico/CMX-IBT-IMSS-927/2021	EPI_ISL_1416393	07/03/21	North America / Mexico / Mexico City	Human	Female	51	Hospitalized
hCoV-19/Mexico/CMX-IBT-IMSS-928/2021	EPI_ISL_1416394	07/03/21	North America / Mexico / Mexico City	Human	Male	62	Hospitalized
hCoV-19/Mexico/CMX-IBT-IMSS-929/2021	EPI_ISL_1416395	07/03/21	North America / Mexico / Mexico City	Human	Male	80	Hospitalized
hCoV-19/Mexico/CMX-IBT-IMSS-930/2021	EPI_ISL_1416396	07/03/21	North America / Mexico / Mexico City	Human	Female	27	Ambulatory
hCoV-19/Mexico/MEX-IBT-IMSS-931/2021	EPI_ISL_1416397	05/03/21	North America / Mexico / State of Mexico	Human	Male	33	Ambulatory
hCoV-19/Mexico/MEX-IBT-IMSS-932/2021	EPI_ISL_1416398	05/03/21	North America / Mexico / State of Mexico	Human	Female	65	Ambulatory
hCoV-19/Mexico/MEX-IBT-IMSS-934/2021	EPI_ISL_1416399	08/03/21	North America / Mexico / State of Mexico	Human	Male	55	Hospitalized
hCoV-19/Mexico/CMX-IBT-IMSS-935/2021	EPI_ISL_1416400	07/03/21	North America / Mexico / Mexico City	Human	Female	77	Hospitalized
hCoV-19/Mexico/MEX-IBT-IMSS-936/2021	EPI_ISL_1416401	09/03/21	North America / Mexico / State of Mexico	Human	Male	33	Hospitalized
hCoV-19/Mexico/CMX-IBT-IMSS-937/2021	EPI_ISL_1416402	07/03/21	North America / Mexico / Mexico City	Human	Female	51	Hospitalized
hCoV-19/Mexico/CMX-IBT-IMSS-938/2021	EPI_ISL_1416403	07/03/21	North America / Mexico / Mexico City	Human	Female	46	Hospitalized
hCoV-19/Mexico/CMX-IBT-IMSS-939/2021	EPI_ISL_1416404	07/03/21	North America / Mexico / Mexico City	Human	Female	60	Ambulatory
hCoV-19/Mexico/MEX-IBT-IMSS-940/2021	EPI_ISL_1416405	05/03/21	North America / Mexico / State of Mexico	Human	Female	15	Hospitalized
hCoV-19/Mexico/MEX-IBT-IMSS-941/2021	EPI_ISL_1416406	08/03/21	North America / Mexico / State of Mexico	Human	Male	63	Hospitalized
hCoV-19/Mexico/MEX-IBT-IMSS-942/2021	EPI_ISL_1416407	09/03/21	North America / Mexico / State of Mexico	Human	Male	28	Hospitalized
hCoV-19/Mexico/CMX-IBT-IMSS-943/2021	EPI_ISL_1416408	07/03/21	North America / Mexico / Mexico City	Human	Female	48	Ambulatory
hCoV-19/Mexico/CMX-IBT-IMSS-944/2021	EPI_ISL_1416409	07/03/21	North America / Mexico / Mexico City	Human	Male	51	Ambulatory
hCoV-19/Mexico/QUE-IBT-IMSS-946/2021	EPI_ISL_1416411	06/03/21	North America / Mexico / Queretaro	Human	Male	36	Ambulatory
hCoV-19/Mexico/MEX-IBT-IMSS-947/2021	EPI_ISL_1416412	09/03/21	North America / Mexico / State of Mexico	Human	Male	63	Hospitalized
hCoV-19/Mexico/OAX-IBT-IMSS-949/2021	EPI_ISL_1416413	09/03/21	North America / Mexico / Oaxaca	Human	Male	47	Hospitalized
hCoV-19/Mexico/MEX-IBT-IMSS-950/2021	EPI_ISL_1416414	09/03/21	North America / Mexico / State of Mexico	Human	Female	63	Hospitalized
hCoV-19/Mexico/VER-IBT-IMSS-886/2021	EPI_ISL_1416415	09/03/21	North America / Mexico / Veracruz	Human	Male	55	Hospitalized
hCoV-19/Mexico/VER-IBT-IMSS-887/2021	EPI_ISL_1416416	09/03/21	North America / Mexico / Veracruz	Human	Female	48	Ambulatory
hCoV-19/Mexico/VER-IBT-IMSS-890/2021	EPI_ISL_1416417	09/03/21	North America / Mexico / Veracruz	Human	Female	38	Ambulatory
hCoV-19/Mexico/VER-IBT-IMSS-891/2021	EPI_ISL_1416418	09/03/21	North America / Mexico / Veracruz	Human	Male	23	Ambulatory
hCoV-19/Mexico/GRO-IBT-IMSS-951/2021	EPI_ISL_1416419	04/03/21	North America / Mexico / Guerrero	Human	Male	0	Ambulatory
hCoV-19/Mexico/VER-IBT-IMSS-588/2021	EPI_ISL_1416422	09/03/21	North America / Mexico / Veracruz	Human	Female	47	Hospitalized

hCoV-19/Mexico/VER-IBT-IMSS-589/2021	EPI_ISL_1416423	09/03/21	North America / Mexico / Veracruz	Human	Male	85	Hospitalized
hCoV-19/Mexico/VER-IBT-IMSS-591/2021	EPI_ISL_1416424	09/03/21	North America / Mexico / Veracruz	Human	Male	34	Ambulatory
hCoV-19/Mexico/VER-IBT-IMSS-592/2021	EPI_ISL_1416425	09/03/21	North America / Mexico / Veracruz	Human	Male	45	Ambulatory
hCoV-19/Mexico/VER-IBT-IMSS-892/2021	EPI_ISL_1416427	09/03/21	North America / Mexico / Veracruz	Human	Female	44	Ambulatory
hCoV-19/Mexico/VER-IBT-IMSS-594/2021	EPI_ISL_1416428	09/03/21	North America / Mexico / Veracruz	Human	Male	75	Hospitalized
hCoV-19/Mexico/VER-IBT-IMSS-595/2021	EPI_ISL_1416429	09/03/21	North America / Mexico / Veracruz	Human	Female	36	Ambulatory
hCoV-19/Mexico/VER-IBT-IMSS-596/2021	EPI_ISL_1416430	09/03/21	North America / Mexico / Veracruz	Human	Female	38	Ambulatory
hCoV-19/Mexico/VER-IBT-IMSS-598/2021	EPI_ISL_1416431	09/03/21	North America / Mexico / Veracruz	Human	Female	50	Hospitalized
hCoV-19/Mexico/VER-IBT-IMSS-599/2021	EPI_ISL_1416432	09/03/21	North America / Mexico / Veracruz	Human	Female	31	Ambulatory
hCoV-19/Mexico/VER-IBT-IMSS-602/2021	EPI_ISL_1416435	09/03/21	North America / Mexico / Veracruz	Human	Male	59	Hospitalized
hCoV-19/Mexico/MEX-IBT-IMSS-603/2021	EPI_ISL_1416436	08/03/21	North America / Mexico / State of Mexico	Human	Female	40	Hospitalized
hCoV-19/Mexico/VER-IBT-IMSS-604/2021	EPI_ISL_1416437	09/03/21	North America / Mexico / Veracruz	Human	Female	50	Hospitalized
hCoV-19/Mexico/VER-IBT-IMSS-605/2021	EPI_ISL_1416438	09/03/21	North America / Mexico / Veracruz	Human	Male	27	Ambulatory
hCoV-19/Mexico/VER-IBT-IMSS-606/2021	EPI_ISL_1416439	09/03/21	North America / Mexico / Veracruz	Human	Female	86	Hospitalized
hCoV-19/Mexico/VER-IBT-IMSS-607/2021	EPI_ISL_1416440	09/03/21	North America / Mexico / Veracruz	Human	Female	39	Ambulatory
hCoV-19/Mexico/SLP-IBT-IMSS-609/2021	EPI_ISL_1416441	08/03/21	North America / Mexico / San Luis Potosi	Human	Male	55	Hospitalized
hCoV-19/Mexico/SLP-IBT-IMSS-610/2021	EPI_ISL_1416442	08/03/21	North America / Mexico / San Luis Potosi	Human	Female	10	Ambulatory
hCoV-19/Mexico/SLP-IBT-IMSS-611/2021	EPI_ISL_1416443	08/03/21	North America / Mexico / San Luis Potosi	Human	Female	60	Hospitalized
hCoV-19/Mexico/SLP-IBT-IMSS-612/2021	EPI_ISL_1416444	08/03/21	North America / Mexico / San Luis Potosi	Human	Female	92	Hospitalized
hCoV-19/Mexico/SLP-IBT-IMSS-613/2021	EPI_ISL_1416445	08/03/21	North America / Mexico / San Luis Potosi	Human	Female	36	Hospitalized
hCoV-19/Mexico/SLP-IBT-IMSS-614/2021	EPI_ISL_1416446	08/03/21	North America / Mexico / San Luis Potosi	Human	Female	20	Ambulatory
hCoV-19/Mexico/SLP-IBT-IMSS-615/2021	EPI_ISL_1416447	08/03/21	North America / Mexico / San Luis Potosi	Human	Female	22	Hospitalized
hCoV-19/Mexico/PUE-IBT-IMSS-616/2021	EPI_ISL_1416448	06/03/21	North America / Mexico / Puebla	Human	Male	82	Ambulatory
hCoV-19/Mexico/PUE-IBT-IMSS-619/2021	EPI_ISL_1416450	08/03/21	North America / Mexico / Puebla	Human	Male	66	Ambulatory
hCoV-19/Mexico/PUE-IBT-IMSS-620/2021	EPI_ISL_1416451	07/03/21	North America / Mexico / Puebla	Human	Male	59	Ambulatory
hCoV-19/Mexico/PUE-IBT-IMSS-621/2021	EPI_ISL_1416452	06/03/21	North America / Mexico / Puebla	Human	Female	51	Ambulatory
hCoV-19/Mexico/VER-IBT-IMSS-622/2021	EPI_ISL_1416453	09/03/21	North America / Mexico / Veracruz	Human	Male	35	Ambulatory
hCoV-19/Mexico/VER-IBT-IMSS-623/2021	EPI_ISL_1416454	09/03/21	North America / Mexico / Veracruz	Human	Female	64	Ambulatory
hCoV-19/Mexico/PUE-IBT-IMSS-626/2021	EPI_ISL_1416456	07/03/21	North America / Mexico / Puebla	Human	Female	46	Ambulatory
hCoV-19/Mexico/PUE-IBT-IMSS-627/2021	EPI_ISL_1416457	08/03/21	North America / Mexico / Puebla	Human	Male	48	Ambulatory
hCoV-19/Mexico/PUE-IBT-IMSS-628/2021	EPI_ISL_1416458	05/03/21	North America / Mexico / Puebla	Human	Female	23	Ambulatory
hCoV-19/Mexico/PUE-IBT-IMSS-629/2021	EPI_ISL_1416459	05/03/21	North America / Mexico / Puebla	Human	Female	33	Ambulatory
hCoV-19/Mexico/PUE-IBT-IMSS-630/2021	EPI_ISL_1416460	08/03/21	North America / Mexico / Puebla	Human	Female	29	Ambulatory
hCoV-19/Mexico/PUE-IBT-IMSS-631/2021	EPI_ISL_1416461	05/03/21	North America / Mexico / Puebla	Human	Female	35	Ambulatory
hCoV-19/Mexico/PUE-IBT-IMSS-632/2021	EPI_ISL_1416462	08/03/21	North America / Mexico / Puebla	Human	Male	34	Ambulatory
hCoV-19/Mexico/MEX-IBT-IMSS-633/2021	EPI_ISL_1416463	09/03/21	North America / Mexico / State of Mexico	Human	Male	25	Ambulatory
hCoV-19/Mexico/MEX-IBT-IMSS-635/2021	EPI_ISL_1416464	07/03/21	North America / Mexico / State of Mexico	Human	Male	50	Hospitalized
hCoV-19/Mexico/MEX-IBT-IMSS-893/2021	EPI_ISL_1416465	07/03/21	North America / Mexico / State of Mexico	Human	Male	82	Hospitalized
hCoV-19/Mexico/MEX-IBT-IMSS-636/2021	EPI_ISL_1416466	07/03/21	North America / Mexico / State of Mexico	Human	Female	63	Hospitalized
hCoV-19/Mexico/MEX-IBT-IMSS-637/2021	EPI_ISL_1416467	08/03/21	North America / Mexico / State of Mexico	Human	Female	61	Hospitalized
hCoV-19/Mexico/BCS-IBT-IMSS-638/2021	EPI_ISL_1416468	07/03/21	North America / Mexico / Baja California Sur	Human	Male	37	Ambulatory
hCoV-19/Mexico/BCS-IBT-IMSS-639/2021	EPI_ISL_1416469	07/03/21	North America / Mexico / Baja California Sur	Human	Male	24	Ambulatory
hCoV-19/Mexico/BCS-IBT-IMSS-640/2021	EPI_ISL_1416470	07/03/21	North America / Mexico / Baja California Sur	Human	Male	35	Ambulatory
hCoV-19/Mexico/BCS-IBT-IMSS-641/2021	EPI_ISL_1416471	07/03/21	North America / Mexico / Baja California Sur	Human	Female	18	Ambulatory

hCoV-19/Mexico/BCS-IBT-IMSS-642/2021	EPI_ISL_1416472	07/03/21	North America / Mexico / Baja California Sur	Human	Female	89	Hospitalized
hCoV-19/Mexico/BCS-IBT-IMSS-644/2021	EPI_ISL_1416473	07/03/21	North America / Mexico / Baja California Sur	Human	Male	56	Ambulatory
hCoV-19/Mexico/BCS-IBT-IMSS-645/2021	EPI_ISL_1416474	07/03/21	North America / Mexico / Baja California Sur	Human	Male	46	Ambulatory
hCoV-19/Mexico/CMX-IBT-IMSS-646/2021	EPI_ISL_1416475	07/03/21	North America / Mexico / Mexico City	Human	Male	70	Hospitalized
hCoV-19/Mexico/CMX-IBT-IMSS-647/2021	EPI_ISL_1416476	07/03/21	North America / Mexico / Mexico City	Human	Male	40	Hospitalized
hCoV-19/Mexico/CMX-IBT-IMSS-648/2021	EPI_ISL_1416477	07/03/21	North America / Mexico / Mexico City	Human	Female	67	Hospitalized
hCoV-19/Mexico/CMX-IBT-IMSS-649/2021	EPI_ISL_1416478	07/03/21	North America / Mexico / Mexico City	Human	Male	74	Hospitalized
hCoV-19/Mexico/CMX-IBT-IMSS-650/2021	EPI_ISL_1416479	07/03/21	North America / Mexico / Mexico City	Human	Male	46	Hospitalized
hCoV-19/Mexico/CMX-IBT-IMSS-651/2021	EPI_ISL_1416480	07/03/21	North America / Mexico / Mexico City	Human	Male	73	Ambulatory
hCoV-19/Mexico/CMX-IBT-IMSS-652/2021	EPI_ISL_1416481	07/03/21	North America / Mexico / Mexico City	Human	Male	35	Ambulatory
hCoV-19/Mexico/CMX-IBT-IMSS-653/2021	EPI_ISL_1416482	07/03/21	North America / Mexico / Mexico City	Human	Female	45	Ambulatory
hCoV-19/Mexico/CMX-IBT-IMSS-654/2021	EPI_ISL_1416483	07/03/21	North America / Mexico / Mexico City	Human	Female	38	Ambulatory
hCoV-19/Mexico/CMX-IBT-IMSS-655/2021	EPI_ISL_1416484	07/03/21	North America / Mexico / Mexico City	Human	Female	39	Ambulatory
hCoV-19/Mexico/MEX-IBT-IMSS-656/2021	EPI_ISL_1416485	09/03/21	North America / Mexico / State of Mexico	Human	Male	40	Hospitalized
hCoV-19/Mexico/MEX-IBT-IMSS-657/2021	EPI_ISL_1416486	06/03/21	North America / Mexico / State of Mexico	Human	Male	70	Hospitalized
hCoV-19/Mexico/CMX-IBT-IMSS-658/2021	EPI_ISL_1416487	07/03/21	North America / Mexico / Mexico City	Human	Male	87	Hospitalized
hCoV-19/Mexico/CMX-IBT-IMSS-660/2021	EPI_ISL_1416488	07/03/21	North America / Mexico / Mexico City	Human	Female	80	Hospitalized
hCoV-19/Mexico/MEX-IBT-IMSS-662/2021	EPI_ISL_1416490	09/03/21	North America / Mexico / State of Mexico	Human	Female	57	Hospitalized
hCoV-19/Mexico/CMX-IBT-IMSS-663/2021	EPI_ISL_1416491	07/03/21	North America / Mexico / Mexico City	Human	Male	63	Hospitalized
hCoV-19/Mexico/CMX-IBT-IMSS-664/2021	EPI_ISL_1416492	07/03/21	North America / Mexico / Mexico City	Human	Male	26	Ambulatory
hCoV-19/Mexico/CMX-IBT-IMSS-665/2021	EPI_ISL_1416493	07/03/21	North America / Mexico / Mexico City	Human	Male	65	Hospitalized
hCoV-19/Mexico/QUE-IBT-IMSS-668/2021	EPI_ISL_1416496	06/03/21	North America / Mexico / Queretaro	Human	Male	25	Ambulatory
hCoV-19/Mexico/MEX-IBT-IMSS-669/2021	EPI_ISL_1416497	10/03/21	North America / Mexico / State of Mexico	Human	Male	68	Hospitalized
hCoV-19/Mexico/PUE-IBT-IMSS-670/2021	EPI_ISL_1416498	04/03/21	North America / Mexico / Puebla	Human	Male	66	Hospitalized
hCoV-19/Mexico/PUE-IBT-IMSS-671/2021	EPI_ISL_1416499	08/03/21	North America / Mexico / Puebla	Human	Male	50	Ambulatory
hCoV-19/Mexico/PUE-IBT-IMSS-673/2021	EPI_ISL_1416500	07/03/21	North America / Mexico / Puebla	Human	Female	59	Ambulatory
hCoV-19/Mexico/PUE-IBT-IMSS-674/2021	EPI_ISL_1416501	09/03/21	North America / Mexico / Puebla	Human	Male	49	Ambulatory
hCoV-19/Mexico/PUE-IBT-IMSS-675/2021	EPI_ISL_1416502	08/03/21	North America / Mexico / Puebla	Human	Male	61	Ambulatory
hCoV-19/Mexico/PUE-IBT-IMSS-676/2021	EPI_ISL_1416503	08/03/21	North America / Mexico / Puebla	Human	Male	46	Ambulatory
hCoV-19/Mexico/PUE-IBT-IMSS-677/2021	EPI_ISL_1416504	08/03/21	North America / Mexico / Puebla	Human	Female	49	Ambulatory
hCoV-19/Mexico/PUE-IBT-IMSS-678/2021	EPI_ISL_1416505	09/03/21	North America / Mexico / Puebla	Human	Male	23	Ambulatory
hCoV-19/Mexico/PUE-IBT-IMSS-679/2021	EPI_ISL_1416506	04/03/21	North America / Mexico / Puebla	Human	Male	39	Hospitalized
hCoV-19/Mexico/PUE-IBT-IMSS-680/2021	EPI_ISL_1416507	05/03/21	North America / Mexico / Puebla	Human	Male	79	Hospitalized
hCoV-19/Mexico/PUE-IBT-IMSS-681/2021	EPI_ISL_1416508	08/03/21	North America / Mexico / Puebla	Human	Male	30	Ambulatory
hCoV-19/Mexico/PUE-IBT-IMSS-682/2021	EPI_ISL_1416509	08/03/21	North America / Mexico / Puebla	Human	Male	63	Ambulatory
hCoV-19/Mexico/PUE-IBT-IMSS-685/2021	EPI_ISL_1416510	08/03/21	North America / Mexico / Puebla	Human	Male	22	Ambulatory
hCoV-19/Mexico/PUE-IBT-IMSS-686/2021	EPI_ISL_1416511	08/03/21	North America / Mexico / Puebla	Human	Female	23	Ambulatory
hCoV-19/Mexico/PUE-IBT-IMSS-687/2021	EPI_ISL_1416512	08/03/21	North America / Mexico / Puebla	Human	Male	51	Ambulatory
hCoV-19/Mexico/PUE-IBT-IMSS-688/2021	EPI_ISL_1416513	05/03/21	North America / Mexico / Puebla	Human	Female	36	Ambulatory
hCoV-19/Mexico/PUE-IBT-IMSS-689/2021	EPI_ISL_1416514	08/03/21	North America / Mexico / Puebla	Human	Female	51	Ambulatory
hCoV-19/Mexico/PUE-IBT-IMSS-690/2021	EPI_ISL_1416515	05/03/21	North America / Mexico / Puebla	Human	Female	60	Ambulatory
hCoV-19/Mexico/PUE-IBT-IMSS-691/2021	EPI_ISL_1416516	09/03/21	North America / Mexico / Puebla	Human	Male	35	Ambulatory
hCoV-19/Mexico/PUE-IBT-IMSS-693/2021	EPI_ISL_1416518	09/03/21	North America / Mexico / Puebla	Human	Female	40	Ambulatory
hCoV-19/Mexico/PUE-IBT-IMSS-694/2021	EPI_ISL_1416519	07/03/21	North America / Mexico / Puebla	Human	Male	47	Hospitalized

hCoV-19/Mexico/PUE-IBT-IMSS-696/2021	EPI_ISL_1416520	08/03/21	North America / Mexico / Puebla	Human	Female	41	Ambulatory
hCoV-19/Mexico/PUE-IBT-IMSS-697/2021	EPI_ISL_1416521	09/03/21	North America / Mexico / Puebla	Human	Female	57	Ambulatory
hCoV-19/Mexico/PUE-IBT-IMSS-698/2021	EPI_ISL_1416522	08/03/21	North America / Mexico / Puebla	Human	Male	40	Ambulatory
hCoV-19/Mexico/PUE-IBT-IMSS-699/2021	EPI_ISL_1416523	08/03/21	North America / Mexico / Puebla	Human	Male	54	Ambulatory
hCoV-19/Mexico/PUE-IBT-IMSS-700/2021	EPI_ISL_1416524	08/03/21	North America / Mexico / Puebla	Human	Female	50	Ambulatory
hCoV-19/Mexico/PUE-IBT-IMSS-701/2021	EPI_ISL_1416525	05/03/21	North America / Mexico / Puebla	Human	Male	56	Ambulatory
hCoV-19/Mexico/PUE-IBT-IMSS-702/2021	EPI_ISL_1416526	08/03/21	North America / Mexico / Puebla	Human	Male	22	Ambulatory
hCoV-19/Mexico/PUE-IBT-IMSS-703/2021	EPI_ISL_1416527	08/03/21	North America / Mexico / Puebla	Human	Male	49	Ambulatory
hCoV-19/Mexico/PUE-IBT-IMSS-704/2021	EPI_ISL_1416528	05/03/21	North America / Mexico / Puebla	Human	Male	64	Hospitalized
hCoV-19/Mexico/PUE-IBT-IMSS-705/2021	EPI_ISL_1416529	05/03/21	North America / Mexico / Puebla	Human	Female	43	Ambulatory
hCoV-19/Mexico/PUE-IBT-IMSS-707/2021	EPI_ISL_1416530	09/03/21	North America / Mexico / Puebla	Human	Male	41	Hospitalized
hCoV-19/Mexico/PUE-IBT-IMSS-708/2021	EPI_ISL_1416531	09/03/21	North America / Mexico / Puebla	Human	Female	82	Hospitalized
hCoV-19/Mexico/PUE-IBT-IMSS-709/2021	EPI_ISL_1416532	09/03/21	North America / Mexico / Puebla	Human	Female	57	Hospitalized
hCoV-19/Mexico/PUE-IBT-IMSS-710/2021	EPI_ISL_1416533	09/03/21	North America / Mexico / Puebla	Human	Female	69	Hospitalized
hCoV-19/Mexico/TLA-IBT-IMSS-711/2021	EPI_ISL_1416534	09/03/21	North America / Mexico / Tlaxcala	Human	Male	66	Hospitalized
hCoV-19/Mexico/VER-IBT-IMSS-712/2021	EPI_ISL_1416535	09/03/21	North America / Mexico / Veracruz	Human	Male	20	Ambulatory
hCoV-19/Mexico/VER-IBT-IMSS-713/2021	EPI_ISL_1416536	09/03/21	North America / Mexico / Veracruz	Human	Male	24	Ambulatory
hCoV-19/Mexico/VER-IBT-IMSS-715/2021	EPI_ISL_1416538	09/03/21	North America / Mexico / Veracruz	Human	Female	84	Hospitalized
hCoV-19/Mexico/VER-IBT-IMSS-717/2021	EPI_ISL_1416539	09/03/21	North America / Mexico / Veracruz	Human	Female	66	Hospitalized
hCoV-19/Mexico/MOR-IBT-IMSS-718/2021	EPI_ISL_1416540	09/03/21	North America / Mexico / Morelos	Human	Female	74	Hospitalized
hCoV-19/Mexico/MEX-IBT-IMSS-719/2021	EPI_ISL_1416541	10/03/21	North America / Mexico / State of Mexico	Human	Female	55	Ambulatory
hCoV-19/Mexico/HID-IBT-IMSS-721/2021	EPI_ISL_1416543	09/03/21	North America / Mexico / Hidalgo	Human	Female	49	Ambulatory
hCoV-19/Mexico/MEX-IBT-IMSS-724/2021	EPI_ISL_1416546	10/03/21	North America / Mexico / State of Mexico	Human	Female	32	Ambulatory
hCoV-19/Mexico/BCS-IBT-IMSS-726/2021	EPI_ISL_1416547	10/03/21	North America / Mexico / Baja California Sur	Human	Male	33	Ambulatory
hCoV-19/Mexico/BCS-IBT-IMSS-727/2021	EPI_ISL_1416548	10/03/21	North America / Mexico / Baja California Sur	Human	Male	61	Ambulatory
hCoV-19/Mexico/BCS-IBT-IMSS-728/2021	EPI_ISL_1416549	10/03/21	North America / Mexico / Baja California Sur	Human	Male	31	Ambulatory
hCoV-19/Mexico/SLP-IBT-IMSS-729/2021	EPI_ISL_1416550	08/03/21	North America / Mexico / San Luis Potosi	Human	Male	16	Hospitalized
hCoV-19/Mexico/SLP-IBT-IMSS-730/2021	EPI_ISL_1416551	08/03/21	North America / Mexico / San Luis Potosi	Human	Male	26	Ambulatory
hCoV-19/Mexico/SLP-IBT-IMSS-731/2021	EPI_ISL_1416552	08/03/21	North America / Mexico / San Luis Potosi	Human	Female	67	Hospitalized
hCoV-19/Mexico/SLP-IBT-IMSS-732/2021	EPI_ISL_1416553	08/03/21	North America / Mexico / San Luis Potosi	Human	Male	39	Hospitalized
hCoV-19/Mexico/SLP-IBT-IMSS-733/2021	EPI_ISL_1416554	08/03/21	North America / Mexico / San Luis Potosi	Human	Male	74	Hospitalized
hCoV-19/Mexico/CMX-IBT-IMSS-734/2021	EPI_ISL_1416555	07/03/21	North America / Mexico / Mexico City	Human	Male	49	Ambulatory
hCoV-19/Mexico/VER-IBT-IMSS-738/2021	EPI_ISL_1416558	09/03/21	North America / Mexico / Veracruz	Human	Male	63	Hospitalized
hCoV-19/Mexico/VER-IBT-IMSS-739/2021	EPI_ISL_1416559	09/03/21	North America / Mexico / Veracruz	Human	Female	36	Ambulatory
hCoV-19/Mexico/VER-IBT-IMSS-740/2021	EPI_ISL_1416560	09/03/21	North America / Mexico / Veracruz	Human	Male	46	Hospitalized
hCoV-19/Mexico/PUE-IBT-IMSS-741/2021	EPI_ISL_1416561	09/03/21	North America / Mexico / Puebla	Human	Male	61	Hospitalized
hCoV-19/Mexico/PUE-IBT-IMSS-742/2021	EPI_ISL_1416562	10/03/21	North America / Mexico / Puebla	Human	Male	58	Hospitalized
hCoV-19/Mexico/PUE-IBT-IMSS-743/2021	EPI_ISL_1416563	09/03/21	North America / Mexico / Puebla	Human	Male	42	Hospitalized
hCoV-19/Mexico/PUE-IBT-IMSS-744/2021	EPI_ISL_1416564	10/03/21	North America / Mexico / Puebla	Human	Female	38	Ambulatory
hCoV-19/Mexico/PUE-IBT-IMSS-745/2021	EPI_ISL_1416565	10/03/21	North America / Mexico / Puebla	Human	Female	42	Ambulatory
hCoV-19/Mexico/PUE-IBT-IMSS-746/2021	EPI_ISL_1416566	10/03/21	North America / Mexico / Puebla	Human	Female	52	Ambulatory
hCoV-19/Mexico/PUE-IBT-IMSS-749/2021	EPI_ISL_1416568	09/03/21	North America / Mexico / Puebla	Human	Female	23	Ambulatory
hCoV-19/Mexico/PUE-IBT-IMSS-751/2021	EPI_ISL_1416570	09/03/21	North America / Mexico / Puebla	Human	Male	42	Ambulatory
hCoV-19/Mexico/PUE-IBT-IMSS-753/2021	EPI_ISL_1416572	09/03/21	North America / Mexico / Puebla	Human	Female	48	Ambulatory

hCoV-19/Mexico/PUE-IBT-IMSS-754/2021	EPI_ISL_1416573	10/03/21	North America / Mexico / Puebla	Human	Female	38	Ambulatory
hCoV-19/Mexico/PUE-IBT-IMSS-755/2021	EPI_ISL_1416574	09/03/21	North America / Mexico / Puebla	Human	Male	68	Ambulatory
hCoV-19/Mexico/PUE-IBT-IMSS-756/2021	EPI_ISL_1416575	08/03/21	North America / Mexico / Puebla	Human	Male	49	Ambulatory
hCoV-19/Mexico/PUE-IBT-IMSS-758/2021	EPI_ISL_1416576	09/03/21	North America / Mexico / Puebla	Human	Female	53	Ambulatory
hCoV-19/Mexico/PUE-IBT-IMSS-759/2021	EPI_ISL_1416577	09/03/21	North America / Mexico / Puebla	Human	Female	41	Ambulatory
hCoV-19/Mexico/PUE-IBT-IMSS-760/2021	EPI_ISL_1416578	09/03/21	North America / Mexico / Puebla	Human	Female	43	Ambulatory
hCoV-19/Mexico/PUE-IBT-IMSS-761/2021	EPI_ISL_1416579	09/03/21	North America / Mexico / Puebla	Human	Female	40	Ambulatory
hCoV-19/Mexico/PUE-IBT-IMSS-763/2021	EPI_ISL_1416581	10/03/21	North America / Mexico / Puebla	Human	Male	56	Ambulatory
hCoV-19/Mexico/PUE-IBT-IMSS-764/2021	EPI_ISL_1416582	10/03/21	North America / Mexico / Puebla	Human	Female	25	Ambulatory
hCoV-19/Mexico/CMX-IBT-IMSS-766/2021	EPI_ISL_1416584	07/03/21	North America / Mexico / Mexico City	Human	Female	73	Hospitalized
hCoV-19/Mexico/CMX-IBT-IMSS-767/2021	EPI_ISL_1416585	07/03/21	North America / Mexico / Mexico City	Human	Male	69	Ambulatory
hCoV-19/Mexico/CMX-IBT-IMSS-769/2021	EPI_ISL_1416586	07/03/21	North America / Mexico / Mexico City	Human	Female	66	Ambulatory
hCoV-19/Mexico/CMX-IBT-IMSS-770/2021	EPI_ISL_1416587	07/03/21	North America / Mexico / Mexico City	Human	Male	40	Hospitalized
hCoV-19/Mexico/MEX-IBT-IMSS-771/2021	EPI_ISL_1416588	11/03/21	North America / Mexico / State of Mexico	Human	Male	48	Ambulatory
hCoV-19/Mexico/CMX-IBT-IMSS-772/2021	EPI_ISL_1416589	07/03/21	North America / Mexico / Mexico City	Human	Female	67	Hospitalized
hCoV-19/Mexico/CMX-IBT-IMSS-773/2021	EPI_ISL_1416590	07/03/21	North America / Mexico / Mexico City	Human	Male	64	Ambulatory
hCoV-19/Mexico/MEX-IBT-IMSS-774/2021	EPI_ISL_1416591	12/03/21	North America / Mexico / State of Mexico	Human	Female	88	Hospitalized
hCoV-19/Mexico/CMX-IBT-IMSS-775/2021	EPI_ISL_1416592	07/03/21	North America / Mexico / Mexico City	Human	Female	73	Hospitalized
hCoV-19/Mexico/CMX-IBT-IMSS-776/2021	EPI_ISL_1416593	07/03/21	North America / Mexico / Mexico City	Human	Male	18	Hospitalized
hCoV-19/Mexico/BCS-IBT-IMSS-777/2021	EPI_ISL_1416594	10/03/21	North America / Mexico / Baja California Sur	Human	Male	43	Ambulatory
hCoV-19/Mexico/VER-IBT-IMSS-779/2021	EPI_ISL_1416595	09/03/21	North America / Mexico / Veracruz	Human	Female	56	Ambulatory
hCoV-19/Mexico/QUE-IBT-IMSS-782/2021	EPI_ISL_1416596	06/03/21	North America / Mexico / Queretaro	Human	Female	30	Ambulatory
hCoV-19/Mexico/QUE-IBT-IMSS-783/2021	EPI_ISL_1416597	06/03/21	North America / Mexico / Queretaro	Human	Male	1	Ambulatory
hCoV-19/Mexico/MEX-IBT-IMSS-784/2021	EPI_ISL_1416598	10/03/21	North America / Mexico / State of Mexico	Human	Female	26	Ambulatory
hCoV-19/Mexico/BCS-IBT-IMSS-785/2021	EPI_ISL_1416599	10/03/21	North America / Mexico / Baja California Sur	Human	Male	58	Ambulatory
hCoV-19/Mexico/PUE-IBT-IMSS-787/2021	EPI_ISL_1416601	10/03/21	North America / Mexico / Puebla	Human	Male	50	Ambulatory
hCoV-19/Mexico/PUE-IBT-IMSS-790/2021	EPI_ISL_1416602	10/03/21	North America / Mexico / Puebla	Human	Female	31	Ambulatory
hCoV-19/Mexico/PUE-IBT-IMSS-791/2021	EPI_ISL_1416603	10/03/21	North America / Mexico / Puebla	Human	Male	38	Ambulatory
hCoV-19/Mexico/PUE-IBT-IMSS-792/2021	EPI_ISL_1416604	11/03/21	North America / Mexico / Puebla	Human	Male	28	Ambulatory
hCoV-19/Mexico/PUE-IBT-IMSS-793/2021	EPI_ISL_1416605	10/03/21	North America / Mexico / Puebla	Human	Female	42	Ambulatory
hCoV-19/Mexico/PUE-IBT-IMSS-794/2021	EPI_ISL_1416606	11/03/21	North America / Mexico / Puebla	Human	Male	25	Ambulatory
hCoV-19/Mexico/PUE-IBT-IMSS-795/2021	EPI_ISL_1416607	10/03/21	North America / Mexico / Puebla	Human	Male	51	Ambulatory
hCoV-19/Mexico/PUE-IBT-IMSS-798/2021	EPI_ISL_1416608	10/03/21	North America / Mexico / Puebla	Human	Male	61	Ambulatory
hCoV-19/Mexico/PUE-IBT-IMSS-799/2021	EPI_ISL_1416609	11/03/21	North America / Mexico / Puebla	Human	Female	31	Ambulatory
hCoV-19/Mexico/PUE-IBT-IMSS-801/2021	EPI_ISL_1416610	11/03/21	North America / Mexico / Puebla	Human	Male	25	Ambulatory
hCoV-19/Mexico/PUE-IBT-IMSS-804/2021	EPI_ISL_1416613	10/03/21	North America / Mexico / Puebla	Human	Male	25	Ambulatory
hCoV-19/Mexico/VER-IBT-IMSS-806/2021	EPI_ISL_1416614	09/03/21	North America / Mexico / Veracruz	Human	Male	37	Ambulatory
hCoV-19/Mexico/VER-IBT-IMSS-807/2021	EPI_ISL_1416615	09/03/21	North America / Mexico / Veracruz	Human	Female	66	Hospitalized
hCoV-19/Mexico/PUE-IBT-IMSS-808/2021	EPI_ISL_1416616	10/03/21	North America / Mexico / Puebla	Human	Female	57	Ambulatory
hCoV-19/Mexico/PUE-IBT-IMSS-809/2021	EPI_ISL_1416617	11/03/21	North America / Mexico / Puebla	Human	Male	29	Ambulatory
hCoV-19/Mexico/PUE-IBT-IMSS-810/2021	EPI_ISL_1416618	08/03/21	North America / Mexico / Puebla	Human	Male	80	Hospitalized
hCoV-19/Mexico/GRO-IBT-IMSS-812/2021	EPI_ISL_1416619	08/03/21	North America / Mexico / Guerrero	Human	Female	51	Ambulatory
hCoV-19/Mexico/PUE-IBT-IMSS-813/2021	EPI_ISL_1416620	08/03/21	North America / Mexico / Puebla	Human	Male	61	Hospitalized
hCoV-19/Mexico/OAX-IBT-IMSS-814/2021	EPI_ISL_1416621	08/03/21	North America / Mexico / Oaxaca	Human	Female	64	Hospitalized

hCoV-19/Mexico/PUE-IBT-IMSS-815/2021	EPI_ISL_1416622	09/03/21	North America / Mexico / Puebla	Human	Male	73	Hospitalized
hCoV-19/Mexico/PUE-IBT-IMSS-816/2021	EPI_ISL_1416623	04/03/21	North America / Mexico / Puebla	Human	Male	52	Hospitalized
hCoV-19/Mexico/PUE-IBT-IMSS-817/2021	EPI_ISL_1416624	10/03/21	North America / Mexico / Puebla	Human	Female	52	Hospitalized
hCoV-19/Mexico/PUE-IBT-IMSS-818/2021	EPI_ISL_1416625	11/03/21	North America / Mexico / Puebla	Human	Female	73	Hospitalized
hCoV-19/Mexico/PUE-IBT-IMSS-819/2021	EPI_ISL_1416626	11/03/21	North America / Mexico / Puebla	Human	Male	34	Hospitalized
hCoV-19/Mexico/PUE-IBT-IMSS-821/2021	EPI_ISL_1416627	11/03/21	North America / Mexico / Puebla	Human	Male	28	Ambulatory
hCoV-19/Mexico/SLP-IBT-IMSS-822/2021	EPI_ISL_1416628	08/03/21	North America / Mexico / San Luis Potosi	Human	Female	67	Hospitalized
hCoV-19/Mexico/MEX-IBT-IMSS-823/2021	EPI_ISL_1416629	12/03/21	North America / Mexico / State of Mexico	Human	Female	47	Hospitalized
hCoV-19/Mexico/GRO-IBT-IMSS-824/2021	EPI_ISL_1416630	06/03/21	North America / Mexico / Guerrero	Human	Male	45	Hospitalized
hCoV-19/Mexico/CHP-IBT-IMSS-825/2021	EPI_ISL_1416631	06/03/21	North America / Mexico / Chiapas	Human	Male	56	Ambulatory
hCoV-19/Mexico/MEX-IBT-IMSS-826/2021	EPI_ISL_1416632	13/03/21	North America / Mexico / State of Mexico	Human	Female	42	Hospitalized
hCoV-19/Mexico/CMX-IBT-IMSS-827/2021	EPI_ISL_1416633	07/03/21	North America / Mexico / Mexico City	Human	Male	67	Hospitalized
hCoV-19/Mexico/CMX-IBT-IMSS-829/2021	EPI_ISL_1416634	07/03/21	North America / Mexico / Mexico City	Human	Male	64	Hospitalized
hCoV-19/Mexico/QUE-IBT-IMSS-830/2021	EPI_ISL_1416635	06/03/21	North America / Mexico / Queretaro	Human	Male	40	Ambulatory
hCoV-19/Mexico/QUE-IBT-IMSS-831/2021	EPI_ISL_1416636	06/03/21	North America / Mexico / Queretaro	Human	Male	32	Ambulatory
hCoV-19/Mexico/QUE-IBT-IMSS-832/2021	EPI_ISL_1416637	06/03/21	North America / Mexico / Queretaro	Human	Female	58	Hospitalized
hCoV-19/Mexico/MOR-IBT-IMSS-834/2021	EPI_ISL_1416639	09/03/21	North America / Mexico / Morelos	Human	Female	49	Hospitalized
hCoV-19/Mexico/VER-IBT-IMSS-835/2021	EPI_ISL_1416640	09/03/21	North America / Mexico / Veracruz	Human	Female	42	Ambulatory
hCoV-19/Mexico/VER-IBT-IMSS-836/2021	EPI_ISL_1416641	09/03/21	North America / Mexico / Veracruz	Human	Female	36	Ambulatory
hCoV-19/Mexico/VER-IBT-IMSS-838/2021	EPI_ISL_1416642	09/03/21	North America / Mexico / Veracruz	Human	Female	49	Hospitalized
hCoV-19/Mexico/VER-IBT-IMSS-842/2021	EPI_ISL_1416643	09/03/21	North America / Mexico / Veracruz	Human	Female	53	Hospitalized
hCoV-19/Mexico/VER-IBT-IMSS-843/2021	EPI_ISL_1416644	09/03/21	North America / Mexico / Veracruz	Human	Female	66	Hospitalized
hCoV-19/Mexico/VER-IBT-IMSS-844/2021	EPI_ISL_1416645	09/03/21	North America / Mexico / Veracruz	Human	Male	55	Ambulatory
hCoV-19/Mexico/CMX-IBT-IMSS-845/2021	EPI_ISL_1416646	07/03/21	North America / Mexico / Mexico City	Human	Male	26	Ambulatory
hCoV-19/Mexico/MEX-IBT-IMSS-848/2021	EPI_ISL_1416648	13/03/21	North America / Mexico / State of Mexico	Human	Male	55	Hospitalized
hCoV-19/Mexico/MEX-IBT-IMSS-850/2021	EPI_ISL_1416650	13/03/21	North America / Mexico / State of Mexico	Human	Male	60	Hospitalized
hCoV-19/Mexico/CMX-IBT-IMSS-851/2021	EPI_ISL_1416651	07/03/21	North America / Mexico / Mexico City	Human	Male	83	Hospitalized
hCoV-19/Mexico/CMX-IBT-IMSS-852/2021	EPI_ISL_1416652	07/03/21	North America / Mexico / Mexico City	Human	Male	69	Hospitalized
hCoV-19/Mexico/CMX-IBT-IMSS-853/2021	EPI_ISL_1416653	07/03/21	North America / Mexico / Mexico City	Human	Male	48	Ambulatory
hCoV-19/Mexico/CMX-IBT-IMSS-854/2021	EPI_ISL_1416654	07/03/21	North America / Mexico / Mexico City	Human	Male	82	Hospitalized
hCoV-19/Mexico/MEX-IBT-IMSS-855/2021	EPI_ISL_1416655	05/03/21	North America / Mexico / State of Mexico	Human	Female	82	Hospitalized
hCoV-19/Mexico/CMX-IBT-IMSS-856/2021	EPI_ISL_1416656	07/03/21	North America / Mexico / Mexico City	Human	Female	38	Hospitalized
hCoV-19/Mexico/CMX-IBT-IMSS-858/2021	EPI_ISL_1416657	07/03/21	North America / Mexico / Mexico City	Human	Male	74	Hospitalized
hCoV-19/Mexico/CMX-IBT-IMSS-859/2021	EPI_ISL_1416658	07/03/21	North America / Mexico / Mexico City	Human	Male	80	Hospitalized
hCoV-19/Mexico/CMX-IBT-IMSS-860/2021	EPI_ISL_1416659	07/03/21	North America / Mexico / Mexico City	Human	Female	65	Hospitalized
hCoV-19/Mexico/CMX-IBT-IMSS-861/2021	EPI_ISL_1416660	07/03/21	North America / Mexico / Mexico City	Human	Male	63	Hospitalized
hCoV-19/Mexico/CMX-IBT-IMSS-862/2021	EPI_ISL_1416661	07/03/21	North America / Mexico / Mexico City	Human	Female	75	Hospitalized
hCoV-19/Mexico/VER-IBT-IMSS-864/2021	EPI_ISL_1416663	09/03/21	North America / Mexico / Veracruz	Human	Female	77	Hospitalized
hCoV-19/Mexico/HID-IBT-IMSS-865/2021	EPI_ISL_1416664	09/03/21	North America / Mexico / Hidalgo	Human	Male	79	Hospitalized
hCoV-19/Mexico/HID-IBT-IMSS-866/2021	EPI_ISL_1416665	09/03/21	North America / Mexico / Hidalgo	Human	Male	61	Hospitalized
hCoV-19/Mexico/HID-IBT-IMSS-867/2021	EPI_ISL_1416666	09/03/21	North America / Mexico / Hidalgo	Human	Male	61	Hospitalized
hCoV-19/Mexico/CMX-IBT-IMSS-870/2021	EPI_ISL_1416668	07/03/21	North America / Mexico / Mexico City	Human	Male	74	Hospitalized
hCoV-19/Mexico/MEX-IBT-IMSS-871/2021	EPI_ISL_1416669	13/03/21	North America / Mexico / State of Mexico	Human	Male	71	Ambulatory
hCoV-19/Mexico/MEX-IBT-IMSS-872/2021	EPI_ISL_1416670	14/03/21	North America / Mexico / State of Mexico	Human	Female	60	Hospitalized

hCoV-19/Mexico/MEX-IBT-IMSS-873/2021	EPI_ISL_1416671	12/03/21	North America / Mexico / State of Mexico	Human	Male	84	Hospitalized	
hCoV-19/Mexico/CMX-IBT-IMSS-874/2021	EPI_ISL_1416672	07/03/21	North America / Mexico / Mexico City	Human	Female	61	Hospitalized	
hCoV-19/Mexico/MOR-IBT-IMSS-875/2021	EPI_ISL_1416673	09/03/21	North America / Mexico / Morelos	Human	Male	68	Hospitalized	
hCoV-19/Mexico/MOR-IBT-IMSS-876/2021	EPI_ISL_1416674	09/03/21	North America / Mexico / Morelos	Human	Male	65	Hospitalized	
hCoV-19/Mexico/MOR-IBT-IMSS-878/2021	EPI_ISL_1416675	09/03/21	North America / Mexico / Morelos	Human	Female	27	Ambulatory	
hCoV-19/Mexico/CMX-IBT-IMSS-880/2021	EPI_ISL_1416677	07/03/21	North America / Mexico / Mexico City	Human	Female	62	Ambulatory	
hCoV-19/Mexico/MEX-IBT-IMSS-881/2021	EPI_ISL_1416678	15/03/21	North America / Mexico / State of Mexico	Human	Female	36	Hospitalized	
hCoV-19/Mexico/MEX-IBT-IMSS-882/2021	EPI_ISL_1416679	13/03/21	North America / Mexico / State of Mexico	Human	Male	64	Hospitalized	
hCoV-19/Mexico/MEX-IBT-IMSS-883/2021	EPI_ISL_1416680	12/03/21	North America / Mexico / State of Mexico	Human	Male	36	Hospitalized	
hCoV-19/Mexico/MEX-IBT-IMSS-884/2021	EPI_ISL_1416681	13/03/21	North America / Mexico / State of Mexico	Human	Male	79	Hospitalized	
hCoV-19/Mexico/MEX-IBT-IMSS-885/2021	EPI_ISL_1416682	15/03/21	North America / Mexico / State of Mexico	Human	Male	74	Hospitalized	
hCoV-19/Mexico/CMX-INMEGEN-03-09-94/2021	EPI_ISL_1416873	16/03/21	North America / Mexico / Mexico City	Human	Male	26	unknown	
hCoV-19/Mexico/CMX-INMEGEN-03-09-95/2021	EPI_ISL_1417442	16/03/21	North America / Mexico / Mexico City	Human	Male	32	unknown	
hCoV-19/Mexico/CMX-INER-INMEGEN-00004/2021	EPI_ISL_1423994	25/02/21	North America / Mexico / Mexico City	Human	Female	10	unknown	
hCoV-19/Mexico/CMX-INER-INMEGEN-00007/2021	EPI_ISL_1423995	26/02/21	North America / Mexico / Mexico City	Human	Male	12	unknown	
hCoV-19/Mexico/CMX-INER-INMEGEN-00019/2021	EPI_ISL_1423996	03/03/21	North America / Mexico / Mexico City	Human	Female	23	unknown	
hCoV-19/Mexico/CMX-INER-INMEGEN-00029/2021	EPI_ISL_1423997	03/03/21	North America / Mexico / Mexico City	Human	Female	35	unknown	
hCoV-19/Mexico/CMX-INER-INMEGEN-00049/2021	EPI_ISL_1424001	20/02/21	North America / Mexico / Mexico City	Human	Female	58	unknown	
hCoV-19/Mexico/YUC-InDRE_F9948_S805/2021	EPI_ISL_1424007	15/02/21	North America / Mexico / Yucatan	Human	Male	42	Released	Oropharyngeal swab
hCoV-19/Mexico/HID-InDRE_F10300_S806/2021	EPI_ISL_1424008	20/02/21	North America / Mexico / Hidalgo	Human	Female	48	Released	Oropharyngeal swab
hCoV-19/Mexico/HID-InDRE_F10309_S807/2021	EPI_ISL_1424009	18/02/21	North America / Mexico / Hidalgo	Human	Male	84	Deceased	Oropharyngeal swab
hCoV-19/Mexico/GUA-InDRE_F10343_S809/2021	EPI_ISL_1424011	16/02/21	North America / Mexico / Guanajuato	Human	Male	57	Hospitalized	Oropharyngeal swab
hCoV-19/Mexico/YUC-InDRE_F10541_S812/2021	EPI_ISL_1424014	21/02/21	North America / Mexico / Yucatan	Human	Male	31	Released	Oropharyngeal swab
hCoV-19/Mexico/QUE-InDRE_F10593_S814/2021	EPI_ISL_1424016	18/02/21	North America / Mexico / Queretaro	Human	Male	17	Released	Oropharyngeal swab
hCoV-19/Mexico/MOR-InDRE_F10634_S815/2021	EPI_ISL_1424017	16/02/21	North America / Mexico / Morelos	Human	Female	25	Released	Oropharyngeal swab
hCoV-19/Mexico/MOR-InDRE_F10665_S816/2021	EPI_ISL_1424018	21/02/21	North America / Mexico / Morelos	Human	Male	44	Hospitalized	Oropharyngeal swab
hCoV-19/Mexico/VER-InDRE_F10671_S817/2021	EPI_ISL_1424019	17/02/21	North America / Mexico / Veracruz	Human	Female	62	Released	Oropharyngeal swab
hCoV-19/Mexico/MOR-InDRE_F10934_S818/2021	EPI_ISL_1424020	24/02/21	North America / Mexico / Morelos	Human	Male	53	Released	Oropharyngeal swab
hCoV-19/Mexico/CHH-InDRE_F11012_S819/2021	EPI_ISL_1424021	23/02/21	North America / Mexico / Chiapas	Human	Male	43	Released	Oropharyngeal swab
hCoV-19/Mexico/QUE-InDRE_F11013_S820/2021	EPI_ISL_1424022	25/02/21	North America / Mexico / Queretaro	Human	Female	53	Hospitalized	Oropharyngeal swab
hCoV-19/Mexico/QUE-InDRE_F11015_S821/2021	EPI_ISL_1424023	25/02/21	North America / Mexico / Queretaro	Human	Female	39	Released	Oropharyngeal swab
hCoV-19/Mexico/QUE-InDRE_F11027_S822/2021	EPI_ISL_1424024	28/02/21	North America / Mexico / Queretaro	Human	Female	78	Released	Oropharyngeal swab
hCoV-19/Mexico/QUE-InDRE_F11019_S824/2021	EPI_ISL_1424026	26/02/21	North America / Mexico / Queretaro	Human	Female	36	Released	Oropharyngeal swab
hCoV-19/Mexico/QUE-InDRE_F11020_S825/2021	EPI_ISL_1424027	26/02/21	North America / Mexico / Queretaro	Human	Male	43	Released	Oropharyngeal swab
hCoV-19/Mexico/QUE-InDRE_F11022_S826/2021	EPI_ISL_1424028	26/02/21	North America / Mexico / Queretaro	Human	Female	47	Released	Oropharyngeal swab
hCoV-19/Mexico/QUE-InDRE_F11023_S827/2021	EPI_ISL_1424029	26/02/21	North America / Mexico / Queretaro	Human	Male	50	Hospitalized	Oropharyngeal swab
hCoV-19/Mexico/QUE-InDRE_F11024_S828/2021	EPI_ISL_1424030	27/02/21	North America / Mexico / Queretaro	Human	Male	49	Released	Oropharyngeal swab
hCoV-19/Mexico/QUE-InDRE_F11025_S829/2021	EPI_ISL_1424031	27/02/21	North America / Mexico / Queretaro	Human	Female	20	Released	Oropharyngeal swab
hCoV-19/Mexico/QUE-InDRE_F11016_S830/2021	EPI_ISL_1424032	25/02/21	North America / Mexico / Queretaro	Human	Male	62	Hospitalized	Oropharyngeal swab
hCoV-19/Mexico/QUE-InDRE_F11028_S831/2021	EPI_ISL_1424033	28/02/21	North America / Mexico / Queretaro	Human	Female	25	Released	Oropharyngeal swab
hCoV-19/Mexico/GUA-InDRE_F11032_S832/2021	EPI_ISL_1424034	01/03/21	North America / Mexico / Guanajuato	Human	Male	84	Hospitalized	Oropharyngeal swab
hCoV-19/Mexico/YUC-InDRE_F11038_S833/2021	EPI_ISL_1424035	01/03/21	North America / Mexico / Yucatan	Human	Male	36	Released	Oropharyngeal swab
hCoV-19/Mexico/YUC-InDRE_F11042_S834/2021	EPI_ISL_1424036	01/03/21	North America / Mexico / Yucatan	Human	Male	62	Released	Oropharyngeal swab
hCoV-19/Mexico/YUC-InDRE_F11043_S835/2021	EPI_ISL_1424037	01/03/21	North America / Mexico / Yucatan	Human	Male	39	Released	Oropharyngeal swab

hCoV-19/Mexico/TAM-InDRE_F11058_S837/2021	EPI_ISL_1424039	18/02/21	North America / Mexico / Tamaulipas	Human	Female	31	Released		Oropharyngeal swab
hCoV-19/Mexico/TAM-InDRE_F11059_S838/2021	EPI_ISL_1424040	19/02/21	North America / Mexico / Tamaulipas	Human	Male	36	Released		Oropharyngeal swab
hCoV-19/Mexico/TAM-InDRE_F11060_S839/2021	EPI_ISL_1424041	23/02/21	North America / Mexico / Tamaulipas	Human	Male	64	Released		Oropharyngeal swab
hCoV-19/Mexico/MOR-InDRE_F11061_S840/2021	EPI_ISL_1424042	25/02/21	North America / Mexico / Morelos	Human	Male	85	Deceased		Oropharyngeal swab
hCoV-19/Mexico/GRO-InDRE_F11063_S841/2021	EPI_ISL_1424043	24/02/21	North America / Mexico / Guerrero	Human	Male	67	Deceased		Oropharyngeal swab
hCoV-19/Mexico/VER-InDRE_F11084_S845/2021	EPI_ISL_1424046	19/02/21	North America / Mexico / Veracruz	Human	Female	70	Hospitalized		Oropharyngeal swab
hCoV-19/Mexico/VER-InDRE_F11087_S846/2021	EPI_ISL_1424047	24/02/21	North America / Mexico / Veracruz	Human	Male	60	Hospitalized		Oropharyngeal swab
hCoV-19/Mexico/VER-InDRE_F11090_S847/2021	EPI_ISL_1424048	24/02/21	North America / Mexico / Veracruz	Human	Female	71	Released		Oropharyngeal swab
hCoV-19/Mexico/VER-InDRE_F11093_S848/2021	EPI_ISL_1424049	25/02/21	North America / Mexico / Veracruz	Human	Female	50	Released		Oropharyngeal swab
hCoV-19/Mexico/BCN-InDRE_F11110_S849/2021	EPI_ISL_1424050	28/02/21	North America / Mexico / Baja California	Human	Male	27	Released		Oropharyngeal swab
hCoV-19/Mexico/CMX-InDRE_F11123_S851/2021	EPI_ISL_1424052	24/02/21	North America / Mexico / Mexico City	Human	Male	57	Hospitalized		Oropharyngeal swab
hCoV-19/Mexico/CMX-InDRE_F11124_S852/2021	EPI_ISL_1424053	25/02/21	North America / Mexico / Mexico City	Human	Male	48	Hospitalized		Oropharyngeal swab
hCoV-19/Mexico/BCN-SEARCH-8268/2021	EPI_ISL_1468958	03/03/21	orth America / Mexico / Baja California / Tijuan	Human	unknown	unknown	unknown		Nasal swab
hCoV-19/Mexico/SLP_UASLP_A019/2021	EPI_ISL_1469110	21/01/21	North America / Mexico / San Luis Potosi	Human	Male	37	Released		Oropharyngeal swab
hCoV-19/Mexico/SLP_UASLP_A007/2021	EPI_ISL_1469117	20/01/21	North America / Mexico / San Luis Potosi	Human	Female	40	Hospitalized		Oropharyngeal swab
hCoV-19/Mexico/SLP_UASLP_A009/2021	EPI_ISL_1469119	20/01/21	North America / Mexico / San Luis Potosi	Human	Female	44	Released		Oropharyngeal swab
hCoV-19/Mexico/SIN-CIAD-HJ1154/2021	EPI_ISL_1482628	25/02/21	North America / Mexico / Sinaloa	Human	Female	62	unknown		Oropharyngeal swab
hCoV-19/Mexico/SIN-CIAD-S6311/2021	EPI_ISL_1482629	17/03/21	North America / Mexico / Sinaloa	Human	Male	33	unknown		Oropharyngeal swab
hCoV-19/Mexico/SIN-CIAD-S6325/2021	EPI_ISL_1482630	18/03/21	North America / Mexico / Sinaloa	Human	Female	38	unknown		Oropharyngeal swab
hCoV-19/Mexico/SIN-CIAD-S6373/2021	EPI_ISL_1482631	23/03/21	North America / Mexico / Sinaloa	Human	Female	41	unknown		Oropharyngeal swab
hCoV-19/Mexico/NLE-InDRE_F11678_S926/2021	EPI_ISL_1483032	08/03/21	North America / Mexico / Nuevo Leon	Human	Female	80	Released		Oropharyngeal swab
hCoV-19/Mexico/NLE-InDRE_F11680_S927/2021	EPI_ISL_1483033	08/03/21	North America / Mexico / Nuevo Leon	Human	Female	50	Released		Oropharyngeal swab
hCoV-19/Mexico/NLE-InDRE_F11683_S928/2021	EPI_ISL_1483034	09/03/21	North America / Mexico / Nuevo Leon	Human	Male	36	Released		Oropharyngeal swab
hCoV-19/Mexico/NLE-InDRE_F11684_S929/2021	EPI_ISL_1483035	09/03/21	North America / Mexico / Nuevo Leon	Human	Female	17	Released		Oropharyngeal swab
hCoV-19/Mexico/NLE-InDRE_F11685_S930/2021	EPI_ISL_1483036	11/03/21	North America / Mexico / Nuevo Leon	Human	Male	11	Released		Oropharyngeal swab
hCoV-19/Mexico/NLE-InDRE_F11686_S931/2021	EPI_ISL_1483037	11/03/21	North America / Mexico / Nuevo Leon	Human	Female	28	Released		Oropharyngeal swab
hCoV-19/Mexico/YUC-InDRE_F11698_S932/2021	EPI_ISL_1483038	14/03/21	North America / Mexico / Yucatan	Human	Female	41	Released		Oropharyngeal swab
hCoV-19/Mexico/YUC-InDRE_F11702_S933/2021	EPI_ISL_1483039	14/03/21	North America / Mexico / Yucatan	Human	Female	86	Hospitalized		Oropharyngeal swab
hCoV-19/Mexico/NAY-InDRE_F11703_S934/2021	EPI_ISL_1483040	12/03/21	North America / Mexico / Nayarit	Human	Male	95	Deceased		Oropharyngeal swab
hCoV-19/Mexico/NAY-InDRE_F11704_S935/2021	EPI_ISL_1483041	10/03/21	North America / Mexico / Nayarit	Human	Male	66	Released		Oropharyngeal swab
hCoV-19/Mexico/SIN-InDRE_F11705_S936/2021	EPI_ISL_1483042	09/03/21	North America / Mexico / Sinaloa	Human	Male	63	Hospitalized		Oropharyngeal swab
hCoV-19/Mexico/SIN-InDRE_F11707_S937/2021	EPI_ISL_1483043	11/03/21	North America / Mexico / Sinaloa	Human	Female	21	Released		Oropharyngeal swab
hCoV-19/Mexico/QUE-InDRE_F11713_S938/2021	EPI_ISL_1483044	11/03/21	North America / Mexico / Queretaro	Human	Female	28	Released		Oropharyngeal swab
hCoV-19/Mexico/QUE-InDRE_F11716_S939/2021	EPI_ISL_1483045	12/03/21	North America / Mexico / Queretaro	Human	Female	44	Released		Oropharyngeal swab
hCoV-19/Mexico/QUE-InDRE_F11721_S940/2021	EPI_ISL_1483046	12/03/21	North America / Mexico / Queretaro	Human	Male	36	Released		Oropharyngeal swab
hCoV-19/Mexico/QUE-InDRE_F11722_S941/2021	EPI_ISL_1483047	13/03/21	North America / Mexico / Queretaro	Human	Female	65	Hospitalized		Oropharyngeal swab
hCoV-19/Mexico/GUA-InDRE_F11723_S942/2021	EPI_ISL_1483048	13/03/21	North America / Mexico / Guanajuato	Human	Male	55	Released		Oropharyngeal swab
hCoV-19/Mexico/QUE-InDRE_F11724_S943/2021	EPI_ISL_1483049	12/03/21	North America / Mexico / Queretaro	Human	Male	57	Released		Oropharyngeal swab
hCoV-19/Mexico/QUE-InDRE_F11735_S944/2021	EPI_ISL_1483050	15/03/21	North America / Mexico / Queretaro	Human	Male	20	Released		Oropharyngeal swab
hCoV-19/Mexico/VER-InDRE_F11484_S945/2021	EPI_ISL_1483055	06/03/21	North America / Mexico / Veracruz	Human	Male	88	Hospitalized		Oropharyngeal swab
hCoV-19/Mexico/VER-InDRE_F11486_S946/2021	EPI_ISL_1483056	01/03/21	North America / Mexico / Veracruz	Human	Male	36	Released		Oropharyngeal swab
hCoV-19/Mexico/VER-InDRE_F11488_S947/2021	EPI_ISL_1483057	06/03/21	North America / Mexico / Veracruz	Human	Female	43	Released		Oropharyngeal swab
hCoV-19/Mexico/HID-InDRE_F11501_S948/2021	EPI_ISL_1483058	07/03/21	North America / Mexico / Hidalgo	Human	Female	75	Deceased		Oropharyngeal swab
hCoV-19/Mexico/PUE-InDRE_F11503_S949/2021	EPI_ISL_1483059	08/03/21	North America / Mexico / Puebla	Human	Male	37	Live		Oropharyngeal swab

hCoV-19/Mexico/PUE-InDRE_F11504_S950/2021	EPI_ISL_1483060	08/03/21	North America / Mexico / Puebla	Human	Male	31	Released	Oropharyngeal swab
hCoV-19/Mexico/PUE-InDRE_F11505_S951/2021	EPI_ISL_1483061	08/03/21	North America / Mexico / Puebla	Human	Female	48	Live	Oropharyngeal swab
hCoV-19/Mexico/PUE-InDRE_F11506_S952/2021	EPI_ISL_1483062	08/03/21	North America / Mexico / Puebla	Human	Female	24	Released	Oropharyngeal swab
hCoV-19/Mexico/PUE-InDRE_F11508_S953/2021	EPI_ISL_1483063	09/03/21	North America / Mexico / Puebla	Human	Female	34	Released	Oropharyngeal swab
hCoV-19/Mexico/PUE-InDRE_F11509_S954/2021	EPI_ISL_1483064	11/03/21	North America / Mexico / Puebla	Human	Female	50	Live	Oropharyngeal swab
hCoV-19/Mexico/PUE-InDRE_F11510_S955/2021	EPI_ISL_1483065	12/03/21	North America / Mexico / Puebla	Human	Male	67	Hospitalized	Oropharyngeal swab
hCoV-19/Mexico/CAM-InDRE_F11511_S956/2021	EPI_ISL_1483066	08/03/21	North America / Mexico / Campeche	Human	Male	69	Hospitalized	Oropharyngeal swab
hCoV-19/Mexico/CAM-InDRE_F11513_S957/2021	EPI_ISL_1483067	12/03/21	North America / Mexico / Campeche	Human	Male	60	Hospitalized	Oropharyngeal swab
hCoV-19/Mexico/JAL-InDRE_F11516_S958/2021	EPI_ISL_1483068	08/03/21	North America / Mexico / Jalisco	Human	Male	28	Released	Oropharyngeal swab
hCoV-19/Mexico/JAL-InDRE_F11517_S959/2021	EPI_ISL_1483069	08/03/21	North America / Mexico / Jalisco	Human	Female	42	Released	Oropharyngeal swab
hCoV-19/Mexico/JAL-InDRE_F11519_S960/2021	EPI_ISL_1483070	09/03/21	North America / Mexico / Jalisco	Human	Male	72	Hospitalized	Oropharyngeal swab
hCoV-19/Mexico/JAL-InDRE_F11523_S961/2021	EPI_ISL_1483071	10/03/21	North America / Mexico / Jalisco	Human	Male	68	Hospitalized	Oropharyngeal swab
hCoV-19/Mexico/JAL-InDRE_F11524_S962/2021	EPI_ISL_1483072	10/03/21	North America / Mexico / Jalisco	Human	Male	85	Released	Oropharyngeal swab
hCoV-19/Mexico/GRO-InDRE_F11640_S963/2021	EPI_ISL_1483073	06/03/21	North America / Mexico / Guerrero	Human	Female	74	Deceased	Oropharyngeal swab
hCoV-19/Mexico/CHH-InDRE_F11643_S964/2021	EPI_ISL_1483074	05/03/21	North America / Mexico / Chihuahua	Human	Female	54	Released	Oropharyngeal swab
hCoV-19/Mexico/SIN_CIAD_ID1979/2021	EPI_ISL_1491362	19/03/21	North America / Mexico / Sinaloa / Mazatlan	Human	Male	53	unknown	Oropharyngeal swab
hCoV-19/Mexico/CHH-InDRE_F11645_S965/2021	EPI_ISL_1494626	07/03/21	North America / Mexico / Chihuahua	Human	Male	43	Live	Oropharyngeal swab
hCoV-19/Mexico/CHH-InDRE_F11650_S966/2021	EPI_ISL_1494627	09/03/21	North America / Mexico / Chihuahua	Human	Female	51	Released	Oropharyngeal swab
hCoV-19/Mexico/CHH-InDRE_F11653_S967/2021	EPI_ISL_1494628	08/03/21	North America / Mexico / Chihuahua	Human	Female	42	Released	Oropharyngeal swab
hCoV-19/Mexico/CHH-InDRE_F11659_S968/2021	EPI_ISL_1494629	10/03/21	North America / Mexico / Chihuahua	Human	Male	79	Hospitalized	Oropharyngeal swab
hCoV-19/Mexico/CHH-InDRE_F11661_S969/2021	EPI_ISL_1494630	08/03/21	North America / Mexico / Chihuahua	Human	Female	41	Live	Oropharyngeal swab
hCoV-19/Mexico/NLE-InDRE_F11664_S970/2021	EPI_ISL_1494631	08/03/21	North America / Mexico / Nuevo Leon	Human	Male	49	Hospitalized	Oropharyngeal swab
hCoV-19/Mexico/NLE-InDRE_F11665_S971/2021	EPI_ISL_1494632	05/03/21	North America / Mexico / Nuevo Leon	Human	Female	66	Released	Oropharyngeal swab
hCoV-19/Mexico/NLE-InDRE_F11668_S972/2021	EPI_ISL_1494633	09/03/21	North America / Mexico / Nuevo Leon	Human	Female	54	Hospitalized	Oropharyngeal swab
hCoV-19/Mexico/NLE-InDRE_F11669_S973/2021	EPI_ISL_1494634	10/03/21	North America / Mexico / Nuevo Leon	Human	Male	41	Released	Oropharyngeal swab
hCoV-19/Mexico/NLE-InDRE_F11671_S974/2021	EPI_ISL_1494635	10/03/21	North America / Mexico / Nuevo Leon	Human	Female	62	Released	Oropharyngeal swab
hCoV-19/Mexico/CMX-InDRE_F34680_S975/2021	EPI_ISL_1494636	17/03/21	North America / Mexico / Mexico City	Human	Male	56	Released	Oropharyngeal swab
hCoV-19/Mexico/CMX-InDRE_F34863_S976/2021	EPI_ISL_1494637	18/03/21	North America / Mexico / Mexico City	Human	Female	20	Released	Oropharyngeal swab
hCoV-19/Mexico/CMX-InDRE_F35831_S977/2021	EPI_ISL_1494638	18/03/21	North America / Mexico / Mexico City	Human	Male	30	released	Oropharyngeal swab
hCoV-19/Mexico/CMX-InDRE_F34412_S978/2021	EPI_ISL_1494639	16/03/21	North America / Mexico / Mexico City	Human	Female	34	Released	Oropharyngeal swab
hCoV-19/Mexico/CMX-InDRE_F34822_S979/2021	EPI_ISL_1494640	16/03/21	North America / Mexico / Mexico City	Human	Female	64	Released	Oropharyngeal swab
hCoV-19/Mexico/CMX-InDRE_F35419_S980/2021	EPI_ISL_1494641	18/03/21	North America / Mexico / Mexico City	Human	Male	55	Released	Oropharyngeal swab
hCoV-19/Mexico/CMX-InDRE_F35724_S981/2021	EPI_ISL_1494642	19/03/21	North America / Mexico / Mexico City	Human	Female	32	Released	Oropharyngeal swab
hCoV-19/Mexico/CMX-InDRE_F34219_S982/2021	EPI_ISL_1494643	16/03/21	North America / Mexico / Mexico City	Human	Male	33	Released	Oropharyngeal swab
hCoV-19/Mexico/CMX-InDRE_F34245_S983/2021	EPI_ISL_1494644	16/03/21	North America / Mexico / Mexico City	Human	Female	21	Released	Oropharyngeal swab
hCoV-19/Mexico/CMX-InDRE_F34262_S984/2021	EPI_ISL_1494645	16/03/21	North America / Mexico / Mexico City	Human	Male	33	Released	Oropharyngeal swab
hCoV-19/Mexico/CMX-InDRE_F34264_S985/2021	EPI_ISL_1494646	16/03/21	North America / Mexico / Mexico City	Human	Female	30	Released	Oropharyngeal swab
hCoV-19/Mexico/CMX-InDRE_F34276_S986/2021	EPI_ISL_1494647	16/03/21	North America / Mexico / Mexico City	Human	Female	53	Released	Oropharyngeal swab
hCoV-19/Mexico/NLE-InDRE_F11674_S987/2021	EPI_ISL_1494648	08/03/21	North America / Mexico / Nuevo Leon	Human	Male	62	Released	Oropharyngeal swab
hCoV-19/Mexico/NLE-InDRE_F11675_S988/2021	EPI_ISL_1494649	08/03/21	North America / Mexico / Nuevo Leon	Human	Male	22	Released	Oropharyngeal swab
hCoV-19/Mexico/TAM-InDRE_F11747_S989/2021	EPI_ISL_1494650	08/03/21	North America / Mexico / Tamaulipas	Human	Male	62	Hospitalized	Oropharyngeal swab
hCoV-19/Mexico/TAM-InDRE_F11749_S990/2021	EPI_ISL_1494651	09/03/21	North America / Mexico / Tamaulipas	Human	Male	55	Live	Oropharyngeal swab
hCoV-19/Mexico/TAM-InDRE_F11750_S991/2021	EPI_ISL_1494652	05/03/21	North America / Mexico / Tamaulipas	Human	Female	21	Live	Oropharyngeal swab
hCoV-19/Mexico/TAM-InDRE_F11752_S992/2021	EPI_ISL_1494653	08/03/21	North America / Mexico / Tamaulipas	Human	Male	26	Released	Oropharyngeal swab

hCoV-19/Mexico/TAM-InDRE_F11756_S993/2021	EPI_ISL_1494654	10/03/21	North America / Mexico / Tamaulipas	Human	Male	54	Released		Oropharyngeal swab
hCoV-19/Mexico/HID-InDRE_F11158_S994/2021	EPI_ISL_1494655	02/03/21	North America / Mexico / Hidalgo	Human	Female	43	Deceased		Oropharyngeal swab
hCoV-19/Mexico/HID-InDRE_F11162_S995/2021	EPI_ISL_1494656	04/03/21	North America / Mexico / Hidalgo	Human	Male	62	Released		Oropharyngeal swab
hCoV-19/Mexico/GUA-InDRE_F11175_S996/2021	EPI_ISL_1494657	02/03/21	North America / Mexico / Guanajuato	Human	Female	76	Hospitalized		Oropharyngeal swab
hCoV-19/Mexico/GUA-InDRE_F11176_S997/2021	EPI_ISL_1494658	03/03/21	North America / Mexico / Guanajuato	Human	Male	59	Hospitalized		Oropharyngeal swab
hCoV-19/Mexico/GUA-InDRE_F11178_S998/2021	EPI_ISL_1494659	04/03/21	North America / Mexico / Guanajuato	Human	Male	44	Hospitalized		Oropharyngeal swab
hCoV-19/Mexico/GUA-InDRE_F11184_S999/2021	EPI_ISL_1494660	01/03/21	North America / Mexico / Guanajuato	Human	Female	24	Released		Oropharyngeal swab
hCoV-19/Mexico/GUA-InDRE_F11185_S1000/2021	EPI_ISL_1494661	01/03/21	North America / Mexico / Guanajuato	Human	Female	28	Released		Oropharyngeal swab
hCoV-19/Mexico/GUA-InDRE_F11186_S1001/2021	EPI_ISL_1494662	02/03/21	North America / Mexico / Guanajuato	Human	Male	18	Released		Oropharyngeal swab
hCoV-19/Mexico/GUA-InDRE_F11188_S1002/2021	EPI_ISL_1494663	02/03/21	North America / Mexico / Guanajuato	Human	Male	46	Released		Oropharyngeal swab
hCoV-19/Mexico/GUA-InDRE_F11189_S1003/2021	EPI_ISL_1494664	03/03/21	North America / Mexico / Guanajuato	Human	Female	59	Released		Oropharyngeal swab
hCoV-19/Mexico/GUA-InDRE_F11193_S1004/2021	EPI_ISL_1494665	04/03/21	North America / Mexico / Guanajuato	Human	Female	28	Released		Oropharyngeal swab
hCoV-19/Mexico/GUA-InDRE_F11195_S1005/2021	EPI_ISL_1494666	04/03/21	North America / Mexico / Guanajuato	Human	Male	19	Released		Oropharyngeal swab
hCoV-19/Mexico/GUA-InDRE_F11196_S1006/2021	EPI_ISL_1494667	04/03/21	North America / Mexico / Guanajuato	Human	Male	68	Released		Oropharyngeal swab
hCoV-19/Mexico/GUA-InDRE_F11198_S1007/2021	EPI_ISL_1494668	05/03/21	North America / Mexico / Guanajuato	Human	Male	31	Released		Oropharyngeal swab
hCoV-19/Mexico/GUA-InDRE_F11199_S1008/2021	EPI_ISL_1494669	06/03/21	North America / Mexico / Guanajuato	Human	Female	49	Released		Oropharyngeal swab
hCoV-19/Mexico/SIN-InDRE_F11201_S1009/2021	EPI_ISL_1494670	01/03/21	North America / Mexico / Sinaloa	Human	Male	75	Deceased		Oropharyngeal swab
hCoV-19/Mexico/SIN-InDRE_F11203_S1010/2021	EPI_ISL_1494671	03/03/21	North America / Mexico / Sinaloa	Human	Male	63	Hospitalized		Oropharyngeal swab
hCoV-19/Mexico/SIN-InDRE_F11204_S1011/2021	EPI_ISL_1494672	05/03/21	North America / Mexico / Sinaloa	Human	Male	44	Deceased		Oropharyngeal swab
hCoV-19/Mexico/NLE-InDRE_F11208_S1012/2021	EPI_ISL_1494673	02/03/21	North America / Mexico / Nuevo Leon	Human	Female	60	Hospitalized		Oropharyngeal swab
oV-19/Mexico/SLP-UASLP-AH1COV2SS018_S7/20	EPI_ISL_1494729	11/01/21	North America / Mexico / San Luis Potosi	Human	Male	38	Hospitalized		
oV-19/Mexico/SLP-UASLP-AH1COV2SS019_S8/20	EPI_ISL_1494730	05/01/21	North America / Mexico / San Luis Potosi	Human	Female	51	Released		
hCoV-19/Mexico/CMX-INCMNSZ_COV159238/2021	EPI_ISL_1502815	11/02/21	North America / Mexico / Mexico City	Human	Male	66	Deceased		Nasopharyngeal swab
hCoV-19/Mexico/CMX-INCMNSZ_COV152225/2021	EPI_ISL_1503143	24/01/21	North America / Mexico / Mexico City	Human	Male	80	Released		Nasopharyngeal swab
hCoV-19/Mexico/NLE-InDRE_F11211_S1013/2021	EPI_ISL_1504058	02/03/21	North America / Mexico / Nuevo Leon	Human	Male	35	Hospitalized		Oropharyngeal swab
hCoV-19/Mexico/NLE-InDRE_F11212_S1014/2021	EPI_ISL_1504059	03/03/21	North America / Mexico / Nuevo Leon	Human	Male	70	Hospitalized		Oropharyngeal swab
hCoV-19/Mexico/NLE-InDRE_F11215_S1015/2021	EPI_ISL_1504060	04/03/21	North America / Mexico / Nuevo Leon	Human	Male	50	Hospitalized		Oropharyngeal swab
hCoV-19/Mexico/NLE-InDRE_F11218_S1016/2021	EPI_ISL_1504061	04/03/21	North America / Mexico / Nuevo Leon	Human	Female	57	Hospitalized		Oropharyngeal swab
hCoV-19/Mexico/NLE-InDRE_F11225_S1017/2021	EPI_ISL_1504062	02/03/21	North America / Mexico / Nuevo Leon	Human	Female	64	Released		Oropharyngeal swab
hCoV-19/Mexico/NLE-InDRE_F11226_S1018/2021	EPI_ISL_1504063	02/03/21	North America / Mexico / Nuevo Leon	Human	Female	56	Released		Oropharyngeal swab
hCoV-19/Mexico/NLE-InDRE_F11228_S1019/2021	EPI_ISL_1504064	02/03/21	North America / Mexico / Nuevo Leon	Human	Female	82	Released		Oropharyngeal swab
hCoV-19/Mexico/NLE-InDRE_F11231_S1020/2021	EPI_ISL_1504065	01/03/21	North America / Mexico / Nuevo Leon	Human	Male	18	Released		Oropharyngeal swab
hCoV-19/Mexico/NLE-InDRE_F11233_S1021/2021	EPI_ISL_1504066	01/03/21	North America / Mexico / Nuevo Leon	Human	Female	44	Released		Oropharyngeal swab
hCoV-19/Mexico/NLE-InDRE_F11235_S1022/2021	EPI_ISL_1504067	04/03/21	North America / Mexico / Nuevo Leon	Human	Female	20	Released		Oropharyngeal swab
hCoV-19/Mexico/MOR-InDRE_F11241_S1023/2021	EPI_ISL_1504068	01/03/21	North America / Mexico / Morelos	Human	Male	17	Released		Oropharyngeal swab
hCoV-19/Mexico/MOR-InDRE_F11243_S1024/2021	EPI_ISL_1504069	01/03/21	North America / Mexico / Morelos	Human	Female	54	Released		Oropharyngeal swab
hCoV-19/Mexico/MOR-InDRE_F11248_S1025/2021	EPI_ISL_1504070	01/03/21	North America / Mexico / Morelos	Human	Female	69	Hospitalized		Oropharyngeal swab
hCoV-19/Mexico/MOR-InDRE_F11250_S1026/2021	EPI_ISL_1504071	01/03/21	North America / Mexico / Morelos	Human	Male	56	Released		Oropharyngeal swab
hCoV-19/Mexico/MOR-InDRE_F11251_S1027/2021	EPI_ISL_1504072	02/03/21	North America / Mexico / Morelos	Human	Male	58	Released		Oropharyngeal swab
hCoV-19/Mexico/MOR-InDRE_F11252_S1028/2021	EPI_ISL_1504073	02/03/21	North America / Mexico / Morelos	Human	Male	47	Released		Oropharyngeal swab
hCoV-19/Mexico/MOR-InDRE_F11254_S1029/2021	EPI_ISL_1504074	02/03/21	North America / Mexico / Morelos	Human	Female	47	Released		Oropharyngeal swab
hCoV-19/Mexico/MOR-InDRE_F11256_S1030/2021	EPI_ISL_1504075	02/03/21	North America / Mexico / Morelos	Human	Male	50	Released		Oropharyngeal swab
hCoV-19/Mexico/MOR-InDRE_F11257_S1031/2021	EPI_ISL_1504076	02/03/21	North America / Mexico / Morelos	Human	Male	83	Hospitalized		Oropharyngeal swab
hCoV-19/Mexico/MOR-InDRE_F11261_S1032/2021	EPI_ISL_1504077	01/03/21	North America / Mexico / Morelos	Human	Female	21	Hospitalized		Oropharyngeal swab

hCoV-19/Mexico/MOR-InDRE_F11264_S1033/2021	EPI_ISL_1504078	02/03/21	North America / Mexico / Morelos	Human	Female	51	Released	Oropharyngeal swab
hCoV-19/Mexico/MOR-InDRE_F11265_S1034/2021	EPI_ISL_1504079	02/03/21	North America / Mexico / Morelos	Human	Female	51	Released	Oropharyngeal swab
hCoV-19/Mexico/MOR-InDRE_F11266_S1035/2021	EPI_ISL_1504080	03/03/21	North America / Mexico / Morelos	Human	Male	48	Hospitalized	Oropharyngeal swab
hCoV-19/Mexico/MOR-InDRE_F11267_S1036/2021	EPI_ISL_1504081	02/03/21	North America / Mexico / Morelos	Human	Female	58	Hospitalized	Oropharyngeal swab
hCoV-19/Mexico/MOR-InDRE_F11275_S1037/2021	EPI_ISL_1504082	03/03/21	North America / Mexico / Morelos	Human	Male	54	Hospitalized	Oropharyngeal swab
hCoV-19/Mexico/CMX-InDRE_F11309_S1038/2021	EPI_ISL_1504083	10/03/21	North America / Mexico / Mexico City	Human	Male	44	Released	Oropharyngeal swab
hCoV-19/Mexico/CHH-InDRE_F11316_S1039/2021	EPI_ISL_1504084	01/03/21	North America / Mexico / Chihuahua	Human	Female	53	Live	Oropharyngeal swab
hCoV-19/Mexico/CHH-InDRE_F11322_S1040/2021	EPI_ISL_1504085	01/03/21	North America / Mexico / Chihuahua	Human	Female	53	Released	Oropharyngeal swab
hCoV-19/Mexico/CHH-InDRE_F11325_S1041/2021	EPI_ISL_1504086	01/03/21	North America / Mexico / Chihuahua	Human	Female	39	Live	Oropharyngeal swab
hCoV-19/Mexico/CHH-InDRE_F11335_S1042/2021	EPI_ISL_1504087	01/03/21	North America / Mexico / Chihuahua	Human	Female	42	Hospitalized	Oropharyngeal swab
hCoV-19/Mexico/CHH-InDRE_F11337_S1043/2021	EPI_ISL_1504088	03/03/21	North America / Mexico / Chihuahua	Human	Male	96	Deceased	Oropharyngeal swab
hCoV-19/Mexico/QUE-InDRE_F11343_S1044/2021	EPI_ISL_1504089	03/03/21	North America / Mexico / Queretaro	Human	Male	84	Released	Oropharyngeal swab
hCoV-19/Mexico/QUE-InDRE_F11344_S1045/2021	EPI_ISL_1504090	03/03/21	North America / Mexico / Queretaro	Human	Male	62	Hospitalized	Oropharyngeal swab
hCoV-19/Mexico/QUE-InDRE_F11345_S1046/2021	EPI_ISL_1504091	03/03/21	North America / Mexico / Queretaro	Human	Male	31	Hospitalized	Oropharyngeal swab
hCoV-19/Mexico/QUE-InDRE_F11346_S1047/2021	EPI_ISL_1504092	03/03/21	North America / Mexico / Queretaro	Human	Female	47	Released	Oropharyngeal swab
hCoV-19/Mexico/QUE-InDRE_F11347_S1048/2021	EPI_ISL_1504093	03/03/21	North America / Mexico / Queretaro	Human	Male	44	Hospitalized	Oropharyngeal swab
hCoV-19/Mexico/QUE-InDRE_F11348_S1049/2021	EPI_ISL_1504094	04/03/21	North America / Mexico / Queretaro	Human	Female	50	Released	Oropharyngeal swab
hCoV-19/Mexico/QUE-InDRE_F11349_S1050/2021	EPI_ISL_1504095	04/03/21	North America / Mexico / Queretaro	Human	Female	59	Released	Oropharyngeal swab
hCoV-19/Mexico/QUE-InDRE_F11351_S1051/2021	EPI_ISL_1504096	05/03/21	North America / Mexico / Queretaro	Human	Male	39	Released	Oropharyngeal swab
hCoV-19/Mexico/QUE-InDRE_F11352_S1052/2021	EPI_ISL_1504097	05/03/21	North America / Mexico / Queretaro	Human	Female	40	Hospitalized	Oropharyngeal swab
hCoV-19/Mexico/QUE-InDRE_F11353_S1053/2021	EPI_ISL_1504098	05/03/21	North America / Mexico / Queretaro	Human	Female	37	Released	Oropharyngeal swab
hCoV-19/Mexico/QUE-InDRE_F11357_S1054/2021	EPI_ISL_1504099	06/03/21	North America / Mexico / Queretaro	Human	Male	27	Released	Oropharyngeal swab
hCoV-19/Mexico/QUE-InDRE_F11361_S1055/2021	EPI_ISL_1504100	06/03/21	North America / Mexico / Queretaro	Human	Male	49	Hospitalized	Oropharyngeal swab
hCoV-19/Mexico/QUE-InDRE_F11362_S1056/2021	EPI_ISL_1504101	08/03/21	North America / Mexico / Queretaro	Human	Male	57	Hospitalized	Oropharyngeal swab
hCoV-19/Mexico/QUE-InDRE_F11364_S1057/2021	EPI_ISL_1504102	08/03/21	North America / Mexico / Queretaro	Human	Female	81	Hospitalized	Oropharyngeal swab
hCoV-19/Mexico/QUE-InDRE_F11365_S1058/2021	EPI_ISL_1504103	08/03/21	North America / Mexico / Queretaro	Human	Female	80	Hospitalized	Oropharyngeal swab
hCoV-19/Mexico/GRO-InDRE_F11377_S1059/2021	EPI_ISL_1504104	01/03/21	North America / Mexico / Guerrero	Human	Male	67	Deceased	Oropharyngeal swab
hCoV-19/Mexico/BCS-InDRE_F11397_S1060/2021	EPI_ISL_1504105	03/03/21	North America / Mexico / Baja California Sur	Human	Female	41	Released	Oropharyngeal swab
hCoV-19/Mexico/ZAC-InDRE_F11430_S1061/2021	EPI_ISL_1504106	04/03/21	North America / Mexico / Zacatecas	Human	Female	54	Live	Oropharyngeal swab
hCoV-19/Mexico/ZAC-InDRE_F11433_S1062/2021	EPI_ISL_1504107	05/03/21	North America / Mexico / Zacatecas	Human	Female	63	Live	Oropharyngeal swab
hCoV-19/Mexico/BCN-InDRE_F11452_S1063/2021	EPI_ISL_1504108	05/03/21	North America / Mexico / Baja California	Human	Male	42	Hospitalized	Oropharyngeal swab
hCoV-19/Mexico/JAL-InDRE_F11470_S1064/2021	EPI_ISL_1504109	05/03/21	North America / Mexico / Jalisco	Human	Female	26	Released	Oropharyngeal swab
hCoV-19/Mexico/QUE-InDRE_F11715_S1065/2021	EPI_ISL_1504110	12/03/21	North America / Mexico / Queretaro	Human	Female	79	Hospitalized	Oropharyngeal swab
hCoV-19/Mexico/QUE-InDRE_F11731_S1066/2021	EPI_ISL_1504111	14/03/21	North America / Mexico / Queretaro	Human	Male	87	Deceased	Oropharyngeal swab
hCoV-19/Mexico/HID-InDRE_F11498_S1069/2021	EPI_ISL_1516763	08/03/21	North America / Mexico / Hidalgo	Human	Male	78	Deceased	Oropharyngeal swab
hCoV-19/Mexico/PUE-InDRE_F11502_S1070/2021	EPI_ISL_1516764	08/03/21	North America / Mexico / Puebla	Human	Male	75	Hospitalized	Oropharyngeal swab
hCoV-19/Mexico/CHH-InDRE_F11647_S1072/2021	EPI_ISL_1516766	06/03/21	North America / Mexico / Chihuahua	Human	Female	42	Live	Oropharyngeal swab
hCoV-19/Mexico/NLE-InDRE_F11676_S1079/2021	EPI_ISL_1516772	08/03/21	North America / Mexico / Nuevo Leon	Human	Female	64	Released	Oropharyngeal swab
hCoV-19/Mexico/CMX-InDRE_F34272_S1096/2021	EPI_ISL_1516788	16/03/21	North America / Mexico / Mexico City	Human	Female	19	Released	Oropharyngeal swab
hCoV-19/Mexico/MOR-InDRE_F11259_S1118/2021	EPI_ISL_1516809	01/03/21	North America / Mexico / Morelos	Human	Male	40	Hospitalized	Oropharyngeal swab
hCoV-19/Mexico/MOR-InDRE_F11273_S1121/2021	EPI_ISL_1516812	03/03/21	North America / Mexico / Morelos	Human	Female	41	Released	Oropharyngeal swab
hCoV-19/Mexico/MOR-InDRE_F11277_S1124/2021	EPI_ISL_1516815	04/03/21	North America / Mexico / Morelos	Human	Male	66	Released	Oropharyngeal swab
hCoV-19/Mexico/QUE-InDRE_F11356_S1133/2021	EPI_ISL_1516824	06/03/21	North America / Mexico / Queretaro	Human	Male	58	Released	Oropharyngeal swab
hCoV-19/Mexico/QUE-InDRE_F11366_S1136/2021	EPI_ISL_1532236	08/03/21	North America / Mexico / Queretaro	Human	Male	48	Hospitalized	Oro-pharyngeal swab

hCoV-19/Mexico/QUE-InDRE_F11373_S1139/2021	EPI_ISL_1532239	09/03/21	North America / Mexico / Queretaro	Human	Male	31	Released	Oro-pharyngeal swab
hCoV-19/Mexico/GRO-InDRE_F11378_S1140/2021	EPI_ISL_1532240	04/03/21	North America / Mexico / Guerrero	Human	Male	79	Released	Oro-pharyngeal swab
hCoV-19/Mexico/GRO-InDRE_F11380_S1141/2021	EPI_ISL_1532241	03/03/21	North America / Mexico / Guerrero	Human	Female	90	Deceased	Oro-pharyngeal swab
hCoV-19/Mexico/BCS-InDRE_F11398_S1143/2021	EPI_ISL_1532243	03/03/21	North America / Mexico / Baja California Sur	Human	Male	43	Released	Oro-pharyngeal swab
hCoV-19/Mexico/BCS-InDRE_F11399_S1144/2021	EPI_ISL_1532244	03/03/21	North America / Mexico / Baja California Sur	Human	Male	24	Released	Oro-pharyngeal swab
hCoV-19/Mexico/BCS-InDRE_F11402_S1145/2021	EPI_ISL_1532245	01/03/21	North America / Mexico / Baja California Sur	Human	Male	34	Released	Oro-pharyngeal swab
hCoV-19/Mexico/CHP-InDRE_F11406_S1146/2021	EPI_ISL_1532246	01/03/21	North America / Mexico / Chiapas	Human	Male	70	Hospitalized	Oro-pharyngeal swab
hCoV-19/Mexico/CHP-InDRE_F11407_S1147/2021	EPI_ISL_1532247	04/03/21	North America / Mexico / Chiapas	Human	Female	42	Live	Oro-pharyngeal swab
hCoV-19/Mexico/YUC-InDRE_F11416_S1148/2021	EPI_ISL_1532248	03/03/21	North America / Mexico / Yucatan	Human	Male	70	Hospitalized	Oro-pharyngeal swab
hCoV-19/Mexico/ZAC-InDRE_F11434_S1153/2021	EPI_ISL_1532253	05/03/21	North America / Mexico / Zacatecas	Human	Male	15	Live	Oro-pharyngeal swab
hCoV-19/Mexico/ZAC-InDRE_F11436_S1155/2021	EPI_ISL_1532255	05/03/21	North America / Mexico / Zacatecas	Human	Male	41	Live	Oro-pharyngeal swab
hCoV-19/Mexico/ZAC-InDRE_F11437_S1156/2021	EPI_ISL_1532256	05/03/21	North America / Mexico / Zacatecas	Human	Female	76	Live	Oro-pharyngeal swab
hCoV-19/Mexico/ZAC-InDRE_F11441_S1158/2021	EPI_ISL_1532258	09/03/21	North America / Mexico / Zacatecas	Human	Female	60	Released	Oro-pharyngeal swab
hCoV-19/Mexico/ZAC-InDRE_F11443_S1160/2021	EPI_ISL_1532260	09/03/21	North America / Mexico / Zacatecas	Human	Male	18	Live	Oro-pharyngeal swab
hCoV-19/Mexico/ZAC-InDRE_F11445_S1162/2021	EPI_ISL_1532262	08/03/21	North America / Mexico / Zacatecas	Human	Male	67	Hospitalized	Oro-pharyngeal swab
hCoV-19/Mexico/CMX-InDRE_F11460_S1165/2021	EPI_ISL_1532265	05/03/21	North America / Mexico / Mexico City	Human	Male	62	Deceased	Oro-pharyngeal swab
hCoV-19/Mexico/JAL-InDRE_F11462_S1166/2021	EPI_ISL_1532266	01/03/21	North America / Mexico / Jalisco	Human	Male	45	Hospitalized	Oro-pharyngeal swab
hCoV-19/Mexico/JAL-InDRE_F11464_S1167/2021	EPI_ISL_1532267	03/03/21	North America / Mexico / Jalisco	Human	Female	80	Hospitalized	Oro-pharyngeal swab
hCoV-19/Mexico/JAL-InDRE_F11469_S1169/2021	EPI_ISL_1532269	04/03/21	North America / Mexico / Jalisco	Human	Male	50	Released	Oro-pharyngeal swab
hCoV-19/Mexico/JAL-InDRE_F11520_S1170/2021	EPI_ISL_1532270	09/03/21	North America / Mexico / Jalisco	Human	Female	28	Released	Oro-pharyngeal swab
hCoV-19/Mexico/QUE-InDRE_F11710_S1172/2021	EPI_ISL_1532272	10/03/21	North America / Mexico / Queretaro	Human	Female	43	Released	Oro-pharyngeal swab
hCoV-19/Mexico/QUE-InDRE_F11711_S1173/2021	EPI_ISL_1532273	11/03/21	North America / Mexico / Queretaro	Human	Female	71	Released	Oro-pharyngeal swab
hCoV-19/Mexico/QUE-InDRE_F11718_S1175/2021	EPI_ISL_1532275	12/03/21	North America / Mexico / Queretaro	Human	Male	75	Hospitalized	Oro-pharyngeal swab
hCoV-19/Mexico/QUE-InDRE_F11725_S1176/2021	EPI_ISL_1532276	13/03/21	North America / Mexico / Queretaro	Human	Male	44	Hospitalized	Oro-pharyngeal swab
hCoV-19/Mexico/QUE-InDRE_F11726_S1177/2021	EPI_ISL_1532277	14/03/21	North America / Mexico / Queretaro	Human	Female	56	Released	Oro-pharyngeal swab
hCoV-19/Mexico/QUE-InDRE_F11729_S1178/2021	EPI_ISL_1532278	14/03/21	North America / Mexico / Queretaro	Human	Female	36	Released	Oro-pharyngeal swab
hCoV-19/Mexico/CMX-InDRE_F11761_S1179/2021	EPI_ISL_1532279	12/03/21	North America / Mexico / Mexico City	Human	Male	56	Released	Oro-pharyngeal swab
hCoV-19/Mexico/ROO-InDRE_F11811_S1181/2021	EPI_ISL_1532281	12/03/21	North America / Mexico / Quintana Roo	Human	Male	66	Deceased	Oro-pharyngeal swab
hCoV-19/Mexico/CMX-INMEGEN-03-09-01/2021	EPI_ISL_1558802	19/03/21	North America / Mexico / Mexico City	Human	Male	70	unknown	Oropharyngeal swab
hCoV-19/Mexico/CMX-INMEGEN-03-09-02/2021	EPI_ISL_1558803	19/03/21	North America / Mexico / Mexico City	Human	Male	65	unknown	Oropharyngeal swab
hCoV-19/Mexico/CMX-INMEGEN-03-09-03/2021	EPI_ISL_1558804	19/03/21	North America / Mexico / Mexico City	Human	Female	62	unknown	Oropharyngeal swab
hCoV-19/Mexico/CMX-INMEGEN-03-09-04/2021	EPI_ISL_1558805	19/03/21	North America / Mexico / Mexico City	Human	Male	48	unknown	Oropharyngeal swab
hCoV-19/Mexico/CMX-INMEGEN-03-09-05/2021	EPI_ISL_1558806	19/03/21	North America / Mexico / Mexico City	Human	Male	37	unknown	Oropharyngeal swab
hCoV-19/Mexico/CMX-INMEGEN-03-09-06/2021	EPI_ISL_1558807	19/03/21	North America / Mexico / Mexico City	Human	Male	16	unknown	Oropharyngeal swab
hCoV-19/Mexico/CMX-INMEGEN-03-09-07/2021	EPI_ISL_1558808	19/03/21	North America / Mexico / Mexico City	Human	Female	79	unknown	Oropharyngeal swab
hCoV-19/Mexico/CMX-INMEGEN-03-09-09/2021	EPI_ISL_1558809	19/03/21	North America / Mexico / Mexico City	Human	Female	33	unknown	Oropharyngeal swab
hCoV-19/Mexico/CMX-INMEGEN-03-09-10/2021	EPI_ISL_1558810	19/03/21	North America / Mexico / Mexico City	Human	Female	64	unknown	Oropharyngeal swab
hCoV-19/Mexico/CMX-INMEGEN-03-09-11/2021	EPI_ISL_1558811	19/03/21	North America / Mexico / Mexico City	Human	Female	59	unknown	Oropharyngeal swab
hCoV-19/Mexico/CMX-INMEGEN-03-09-12/2021	EPI_ISL_1558812	19/03/21	North America / Mexico / Mexico City	Human	Male	73	unknown	Oropharyngeal swab
hCoV-19/Mexico/CMX-INMEGEN-03-09-13/2021	EPI_ISL_1558813	18/03/21	North America / Mexico / Mexico City	Human	Male	56	unknown	Oropharyngeal swab
hCoV-19/Mexico/CMX-INMEGEN-03-09-14/2021	EPI_ISL_1558814	18/03/21	North America / Mexico / Mexico City	Human	Male	58	unknown	Oropharyngeal swab
hCoV-19/Mexico/CMX-INMEGEN-03-09-15/2021	EPI_ISL_1558815	18/03/21	North America / Mexico / Mexico City	Human	Male	65	unknown	Oropharyngeal swab
hCoV-19/Mexico/CMX-INMEGEN-03-09-16/2021	EPI_ISL_1558816	18/03/21	North America / Mexico / Mexico City	Human	Male	55	unknown	Oropharyngeal swab
hCoV-19/Mexico/CMX-INMEGEN-03-09-17/2021	EPI_ISL_1558817	18/03/21	North America / Mexico / Mexico City	Human	Female	74	unknown	Oropharyngeal swab

hCoV-19/Mexico/CMX-INMEGEN-03-09-18/2021	EPI_ISL_1558818	18/03/21	North America / Mexico / Mexico City	Human	Female	53	unknown	Oropharyngeal swab
hCoV-19/Mexico/CMX-INMEGEN-03-09-19/2021	EPI_ISL_1558819	18/03/21	North America / Mexico / Mexico City	Human	Female	36	unknown	Oropharyngeal swab
hCoV-19/Mexico/CMX-INMEGEN-03-09-20/2021	EPI_ISL_1558820	18/03/21	North America / Mexico / Mexico City	Human	Male	59	unknown	Oropharyngeal swab
hCoV-19/Mexico/CMX-INMEGEN-03-09-21/2021	EPI_ISL_1558821	18/03/21	North America / Mexico / Mexico City	Human	Male	47	unknown	Oropharyngeal swab
hCoV-19/Mexico/CMX-INMEGEN-03-09-22/2021	EPI_ISL_1558822	18/03/21	North America / Mexico / Mexico City	Human	Male	66	unknown	Oropharyngeal swab
hCoV-19/Mexico/CMX-INMEGEN-03-09-23/2021	EPI_ISL_1558823	18/03/21	North America / Mexico / Mexico City	Human	Female	70	unknown	Oropharyngeal swab
hCoV-19/Mexico/CMX-INMEGEN-03-09-24/2021	EPI_ISL_1558824	18/03/21	North America / Mexico / Mexico City	Human	Female	59	unknown	Oropharyngeal swab
hCoV-19/Mexico/CMX-INMEGEN-03-09-25/2021	EPI_ISL_1558825	17/03/21	North America / Mexico / Mexico City	Human	Female	61	unknown	Oropharyngeal swab
hCoV-19/Mexico/CMX-INMEGEN-03-09-27/2021	EPI_ISL_1558826	17/03/21	North America / Mexico / Mexico City	Human	Male	44	unknown	Oropharyngeal swab
hCoV-19/Mexico/CMX-INMEGEN-03-09-28/2021	EPI_ISL_1558827	17/03/21	North America / Mexico / Mexico City	Human	Male	72	unknown	Oropharyngeal swab
hCoV-19/Mexico/CHH_INER_IMSS_00372/2021	EPI_ISL_1585384	03/03/21	North America / Mexico / Chihuahua	Human	Female	45	Ambulatory	Nasopharyngeal swab
hCoV-19/Mexico/CHH_INER_IMSS_00374/2021	EPI_ISL_1585386	02/03/21	North America / Mexico / Chihuahua	Human	Male	35	Hospitalized	Nasopharyngeal and oropharyngeal swab
hCoV-19/Mexico/CHH_INER_IMSS_00377/2021	EPI_ISL_1585388	01/03/21	North America / Mexico / Chihuahua	Human	Male	54	Hospitalized	Nasopharyngeal and oropharyngeal swab
hCoV-19/Mexico/CHH_INER_IMSS_00378/2021	EPI_ISL_1585389	01/03/21	North America / Mexico / Chihuahua	Human	Female	52	Hospitalized	Nasopharyngeal and oropharyngeal swab
hCoV-19/Mexico/DUR_INER_IMSS_00385/2021	EPI_ISL_1585395	10/03/21	North America / Mexico / Durango	Human	Female	37	Ambulatory	Nasopharyngeal and oropharyngeal swab
hCoV-19/Mexico/DUR_INER_IMSS_00386/2021	EPI_ISL_1585396	11/03/21	North America / Mexico / Durango	Human	Male	85	Hospitalized	Nasopharyngeal and oropharyngeal swab
hCoV-19/Mexico/CHH_INER_IMSS_00387/2021	EPI_ISL_1585397	08/03/21	North America / Mexico / Chihuahua	Human	Male	46	Ambulatory	Oropharyngeal swab
hCoV-19/Mexico/COA_INER_IMSS_00388/2021	EPI_ISL_1585398	10/03/21	North America / Mexico / Coahuila	Human	Female	81	Ambulatory	Nasopharyngeal and oropharyngeal swab
hCoV-19/Mexico/CHH_INER_IMSS_00392/2021	EPI_ISL_1585402	08/03/21	North America / Mexico / Chihuahua	Human	Male	77	Ambulatory	Nasopharyngeal and oropharyngeal swab
hCoV-19/Mexico/CHH_INER_IMSS_00393/2021	EPI_ISL_1585403	07/03/21	North America / Mexico / Chihuahua	Human	Male	56	Ambulatory	Nasopharyngeal swab
hCoV-19/Mexico/CHH_INER_IMSS_00397/2021	EPI_ISL_1585407	09/03/21	North America / Mexico / Chihuahua	Human	Male	71	Hospitalized	Nasopharyngeal and oropharyngeal swab
hCoV-19/Mexico/NLE_INER_IMSS_00399/2021	EPI_ISL_1585409	15/03/21	North America / Mexico / Nuevo Leon	Human	Female	26	Hospitalized	Nasopharyngeal and oropharyngeal swab
hCoV-19/Mexico/NLE_INER_IMSS_00400/2021	EPI_ISL_1585410	15/03/21	North America / Mexico / Nuevo Leon	Human	Female	51	Hospitalized	Nasopharyngeal and oropharyngeal swab
hCoV-19/Mexico/NLE_INER_IMSS_00401/2021	EPI_ISL_1585411	16/03/21	North America / Mexico / Nuevo Leon	Human	Male	70	Ambulatory	Nasopharyngeal swab
hCoV-19/Mexico/NLE_INER_IMSS_00402/2021	EPI_ISL_1585412	16/03/21	North America / Mexico / Nuevo Leon	Human	Female	71	Hospitalized	Nasopharyngeal and oropharyngeal swab
hCoV-19/Mexico/COA_INER_IMSS_00405/2021	EPI_ISL_1585414	14/03/21	North America / Mexico / Coahuila	Human	Female	70	Hospitalized	Nasopharyngeal and oropharyngeal swab
hCoV-19/Mexico/COA_INER_IMSS_00406/2021	EPI_ISL_1585415	14/03/21	North America / Mexico / Coahuila	Human	Male	74	Ambulatory	Nasopharyngeal and oropharyngeal swab
hCoV-19/Mexico/DUR_INER_IMSS_00407/2021	EPI_ISL_1585416	16/03/21	North America / Mexico / Durango	Human	Male	60	Hospitalized	Nasopharyngeal and oropharyngeal swab
hCoV-19/Mexico/DUR_INER_IMSS_00408/2021	EPI_ISL_1585417	16/03/21	North America / Mexico / Durango	Human	Female	47	Ambulatory	Nasopharyngeal and oropharyngeal swab
hCoV-19/Mexico/TAM_INER_IMSS_00413/2021	EPI_ISL_1585421	17/03/21	North America / Mexico / Tamaulipas	Human	Female	37	Ambulatory	Nasopharyngeal and oropharyngeal swab
hCoV-19/Mexico/CHH_INER_IMSS_00417/2021	EPI_ISL_1585425	14/03/21	North America / Mexico / Chihuahua	Human	Male	47	Hospitalized	Nasopharyngeal and oropharyngeal swab
hCoV-19/Mexico/YUC_INER_IMSS_00421/2021	EPI_ISL_1585429	07/03/21	North America / Mexico / Yucatan	Human	Male	73	Hospitalized	Nasopharyngeal swab
hCoV-19/Mexico/ROO_INER_IMSS_00424/2021	EPI_ISL_1585432	08/03/21	North America / Mexico / Quintana Roo	Human	Female	69	Hospitalized	Nasopharyngeal swab
hCoV-19/Mexico/YUC_INER_IMSS_00425/2021	EPI_ISL_1585433	05/03/21	North America / Mexico / Yucatan	Human	Male	50	Deceased	Nasopharyngeal and oropharyngeal swab
hCoV-19/Mexico/TAB_INER_IMSS_00426/2021	EPI_ISL_1585434	04/03/21	North America / Mexico / Tabasco	Human	Male	27	Ambulatory	Nasopharyngeal and oropharyngeal swab
hCoV-19/Mexico/YUC_INER_IMSS_00427/2021	EPI_ISL_1585435	10/03/21	North America / Mexico / Yucatan	Human	Male	73	Hospitalized	Nasopharyngeal and oropharyngeal swab
hCoV-19/Mexico/YUC_INER_IMSS_00428/2021	EPI_ISL_1585436	10/03/21	North America / Mexico / Yucatan	Human	Male	85	Hospitalized	Oropharyngeal swab
hCoV-19/Mexico/YUC_INER_IMSS_00430/2021	EPI_ISL_1585438	10/03/21	North America / Mexico / Yucatan	Human	Male	36	Ambulatory	Nasopharyngeal and oropharyngeal swab
hCoV-19/Mexico/YUC_INER_IMSS_00431/2021	EPI_ISL_1585439	11/03/21	North America / Mexico / Yucatan	Human	Male	63	Hospitalized	Nasopharyngeal swab
hCoV-19/Mexico/YUC_INER_IMSS_00432/2021	EPI_ISL_1585440	11/03/21	North America / Mexico / Yucatan	Human	Female	61	Hospitalized	Nasopharyngeal and oropharyngeal swab
hCoV-19/Mexico/YUC_INER_IMSS_00433/2021	EPI_ISL_1585441	10/03/21	North America / Mexico / Yucatan	Human	Male	34	Hospitalized	Nasopharyngeal and oropharyngeal swab
hCoV-19/Mexico/ROO_INER_IMSS_00435/2021	EPI_ISL_1585443	09/03/21	North America / Mexico / Quintana Roo	Human	Female	72	Hospitalized	Nasopharyngeal and oropharyngeal swab
hCoV-19/Mexico/YUC_INER_IMSS_00439/2021	EPI_ISL_1585447	14/03/21	North America / Mexico / Yucatan	Human	Female	61	Deceased	Nasopharyngeal and oropharyngeal swab
hCoV-19/Mexico/ROO_INER_IMSS_00440/2021	EPI_ISL_1585448	12/03/21	North America / Mexico / Quintana Roo	Human	Male	69	Hospitalized	Nasopharyngeal and oropharyngeal swab

hCoV-19/Mexico/YUC_INER_IMSS_00442/2021	EPI_ISL_1585449	16/03/21	North America / Mexico / Yucatan	Human	Male	41	Ambulatory	Nasopharyngeal and oropharyngeal swab
hCoV-19/Mexico/YUC_INER_IMSS_00443/2021	EPI_ISL_1585450	15/03/21	North America / Mexico / Yucatan	Human	Female	67	Hospitalized	Nasopharyngeal swab
hCoV-19/Mexico/YUC_INER_IMSS_00444/2021	EPI_ISL_1585451	16/03/21	North America / Mexico / Yucatan	Human	Male	22	Hospitalized	Nasopharyngeal and oropharyngeal swab
hCoV-19/Mexico/YUC_INER_IMSS_00445/2021	EPI_ISL_1585452	17/03/21	North America / Mexico / Yucatan	Human	Male	21	Hospitalized	Nasopharyngeal swab
hCoV-19/Mexico/YUC_INER_IMSS_00446/2021	EPI_ISL_1585453	17/03/21	North America / Mexico / Yucatan	Human	Male	56	Hospitalized	Nasopharyngeal and oropharyngeal swab
hCoV-19/Mexico/YUC_INER_IMSS_00447/2021	EPI_ISL_1585454	17/03/21	North America / Mexico / Yucatan	Human	Female	63	Hospitalized	Nasopharyngeal swab
hCoV-19/Mexico/ZAC_INER_IMSS_00448/2021	EPI_ISL_1585455	07/03/21	North America / Mexico / Zacatecas	Human	Female	73	Hospitalized	Nasopharyngeal and oropharyngeal swab
hCoV-19/Mexico/ZAC_INER_IMSS_00449/2021	EPI_ISL_1585456	07/03/21	North America / Mexico / Zacatecas	Human	Female	31	Ambulatory	Nasopharyngeal and oropharyngeal swab
hCoV-19/Mexico/ZAC_INER_IMSS_00450/2021	EPI_ISL_1585457	08/03/21	North America / Mexico / Zacatecas	Human	Female	47	Ambulatory	Nasopharyngeal and oropharyngeal swab
hCoV-19/Mexico/ZAC_INER_IMSS_00453/2021	EPI_ISL_1585460	08/03/21	North America / Mexico / Zacatecas	Human	Female	37	Ambulatory	Nasopharyngeal and oropharyngeal swab
hCoV-19/Mexico/ZAC_INER_IMSS_00454/2021	EPI_ISL_1585461	08/03/21	North America / Mexico / Zacatecas	Human	Female	33	Ambulatory	Nasopharyngeal and oropharyngeal swab
hCoV-19/Mexico/ZAC_INER_IMSS_00456/2021	EPI_ISL_1585463	09/03/21	North America / Mexico / Zacatecas	Human	Female	32	Ambulatory	Nasopharyngeal and oropharyngeal swab
hCoV-19/Mexico/ZAC_INER_IMSS_00457/2021	EPI_ISL_1585464	10/03/21	North America / Mexico / Zacatecas	Human	Male	57	Ambulatory	Nasopharyngeal and oropharyngeal swab
hCoV-19/Mexico/ZAC_INER_IMSS_00461/2021	EPI_ISL_1585467	11/03/21	North America / Mexico / Zacatecas	Human	Male	79	Hospitalized	Nasopharyngeal and oropharyngeal swab
hCoV-19/Mexico/ZAC_INER_IMSS_00464/2021	EPI_ISL_1585470	14/03/21	North America / Mexico / Zacatecas	Human	Female	64	Hospitalized	Nasopharyngeal and oropharyngeal swab
hCoV-19/Mexico/ZAC_INER_IMSS_00465/2021	EPI_ISL_1585471	16/03/21	North America / Mexico / Zacatecas	Human	Male	93	Ambulatory	Nasopharyngeal and oropharyngeal swab
hCoV-19/Mexico/ZAC_INER_IMSS_00466/2021	EPI_ISL_1585472	17/03/21	North America / Mexico / Zacatecas	Human	Male	69	Hospitalized	Nasopharyngeal and oropharyngeal swab
hCoV-19/Mexico/ZAC_INER_IMSS_00467/2021	EPI_ISL_1585473	17/03/21	North America / Mexico / Zacatecas	Human	Male	48	Ambulatory	Nasopharyngeal and oropharyngeal swab
hCoV-19/Mexico/ZAC_INER_IMSS_00468/2021	EPI_ISL_1585474	17/03/21	North America / Mexico / Zacatecas	Human	Male	29	Ambulatory	Nasopharyngeal and oropharyngeal swab
hCoV-19/Mexico/ZAC_INER_IMSS_00469/2021	EPI_ISL_1585475	18/03/21	North America / Mexico / Zacatecas	Human	Female	74	Ambulatory	Nasopharyngeal and oropharyngeal swab
hCoV-19/Mexico/ZAC_INER_IMSS_00471/2021	EPI_ISL_1585477	17/03/21	North America / Mexico / Zacatecas	Human	Female	77	Ambulatory	Nasopharyngeal and oropharyngeal swab
hCoV-19/Mexico/ZAC_INER_IMSS_00472/2021	EPI_ISL_1585478	18/03/21	North America / Mexico / Zacatecas	Human	Male	76	Hospitalized	Nasopharyngeal and oropharyngeal swab
hCoV-19/Mexico/JAL_INER_IMSS_00478/2021	EPI_ISL_1585482	09/03/21	North America / Mexico / Jalisco	Human	Male	36	Hospitalized	Nasopharyngeal and oropharyngeal swab
hCoV-19/Mexico/JAL_INER_IMSS_00480/2021	EPI_ISL_1585484	08/03/21	North America / Mexico / Jalisco	Human	Female	84	Hospitalized	Nasopharyngeal and oropharyngeal swab
hCoV-19/Mexico/JAL_INER_IMSS_00486/2021	EPI_ISL_1585489	10/03/21	North America / Mexico / Jalisco	Human	Female	35	Hospitalized	Nasopharyngeal and oropharyngeal swab
hCoV-19/Mexico/JAL_INER_IMSS_00487/2021	EPI_ISL_1585490	11/03/21	North America / Mexico / Jalisco	Human	Male	36	Ambulatory	Nasopharyngeal and oropharyngeal swab
hCoV-19/Mexico/JAL_INER_IMSS_00488/2021	EPI_ISL_1585491	10/03/21	North America / Mexico / Jalisco	Human	Male	74	Hospitalized	Nasopharyngeal and oropharyngeal swab
hCoV-19/Mexico/GUA_INER_IMSS_00489/2021	EPI_ISL_1585492	09/03/21	North America / Mexico / Guanajuato	Human	Female	51	Ambulatory	Oropharyngeal swab
hCoV-19/Mexico/GUA_INER_IMSS_00491/2021	EPI_ISL_1585494	09/03/21	North America / Mexico / Guanajuato	Human	Male	79	Hospitalized	Nasopharyngeal and oropharyngeal swab
hCoV-19/Mexico/GUA_INER_IMSS_00492/2021	EPI_ISL_1585495	10/03/21	North America / Mexico / Guanajuato	Human	Male	85	Hospitalized	Nasopharyngeal and oropharyngeal swab
hCoV-19/Mexico/GUA_INER_IMSS_00494/2021	EPI_ISL_1585497	03/03/21	North America / Mexico / Guanajuato	Human	Female	59	Hospitalized	Nasopharyngeal and oropharyngeal swab
hCoV-19/Mexico/MIC_INER_IMSS_00496/2021	EPI_ISL_1585499	10/03/21	North America / Mexico / Michoacan	Human	Female	75	Hospitalized	Nasopharyngeal and oropharyngeal swab
hCoV-19/Mexico/JAL_INER_IMSS_00502/2021	EPI_ISL_1585505	12/03/21	North America / Mexico / Jalisco	Human	Male	32	Hospitalized	Nasopharyngeal and oropharyngeal swab
hCoV-19/Mexico/JAL_INER_IMSS_00503/2021	EPI_ISL_1585506	12/03/21	North America / Mexico / Jalisco	Human	Female	60	Ambulatory	Nasopharyngeal and oropharyngeal swab
hCoV-19/Mexico/GUA_INER_IMSS_00504/2021	EPI_ISL_1585507	11/03/21	North America / Mexico / Guanajuato	Human	Male	75	Hospitalized	Nasopharyngeal and oropharyngeal swab
hCoV-19/Mexico/COL_INER_IMSS_00505/2021	EPI_ISL_1585508	10/03/21	North America / Mexico / Colima	Human	Male	48	Hospitalized	Nasopharyngeal and oropharyngeal swab
hCoV-19/Mexico/GUA_INER_IMSS_00506/2021	EPI_ISL_1585509	11/03/21	North America / Mexico / Guanajuato	Human	Male	33	Ambulatory	Nasopharyngeal and oropharyngeal swab
hCoV-19/Mexico/GUA_INER_IMSS_00508/2021	EPI_ISL_1585511	11/03/21	North America / Mexico / Guanajuato	Human	Male	36	Ambulatory	Nasopharyngeal and oropharyngeal swab
hCoV-19/Mexico/JAL_INER_IMSS_00511/2021	EPI_ISL_1585514	12/03/21	North America / Mexico / Jalisco	Human	Male	78	Hospitalized	Nasopharyngeal and oropharyngeal swab
hCoV-19/Mexico/JAL_INER_IMSS_00514/2021	EPI_ISL_1585517	14/03/21	North America / Mexico / Jalisco	Human	Female	80	Hospitalized	Nasopharyngeal and oropharyngeal swab
hCoV-19/Mexico/JAL_INER_IMSS_00515/2021	EPI_ISL_1585518	16/03/21	North America / Mexico / Jalisco	Human	Male	38	Ambulatory	Nasopharyngeal and oropharyngeal swab
hCoV-19/Mexico/SON_INER_IMSS_00516/2021	EPI_ISL_1585519	11/03/21	North America / Mexico / Sonora	Human	Female	33	Ambulatory	Nasopharyngeal swab
hCoV-19/Mexico/COL_INER_IMSS_00519/2021	EPI_ISL_1585522	15/03/21	North America / Mexico / Colima	Human	Male	56	Hospitalized	Nasopharyngeal and oropharyngeal swab
hCoV-19/Mexico/JAL_INER_IMSS_00520/2021	EPI_ISL_1585523	15/03/21	North America / Mexico / Jalisco	Human	Male	91	Hospitalized	Nasopharyngeal and oropharyngeal swab

hCoV-19/Mexico/JAL_INER_IMSS_00521/2021	EPI_ISL_1585524	15/03/21	North America / Mexico / Jalisco	Human	Male	79	Ambulatory	Nasopharyngeal and oropharyngeal swab
hCoV-19/Mexico/JAL_INER_IMSS_00522/2021	EPI_ISL_1585525	16/03/21	North America / Mexico / Jalisco	Human	Female	54	Ambulatory	Nasopharyngeal and oropharyngeal swab
hCoV-19/Mexico/JAL_INER_IMSS_00523/2021	EPI_ISL_1585526	17/03/21	North America / Mexico / Jalisco	Human	Male	61	Hospitalized	Nasopharyngeal and oropharyngeal swab
hCoV-19/Mexico/GUA_INER_IMSS_00524/2021	EPI_ISL_1585527	16/03/21	North America / Mexico / Guanajuato	Human	Male	44	Ambulatory	Nasopharyngeal and oropharyngeal swab
hCoV-19/Mexico/JAL_INER_IMSS_00525/2021	EPI_ISL_1585528	16/03/21	North America / Mexico / Jalisco	Human	Male	78	Hospitalized	Nasopharyngeal and oropharyngeal swab
hCoV-19/Mexico/JAL_INER_IMSS_00526/2021	EPI_ISL_1585529	17/03/21	North America / Mexico / Jalisco	Human	Male	78	Ambulatory	Oropharyngeal swab
hCoV-19/Mexico/GUA_INER_IMSS_00528/2021	EPI_ISL_1585531	16/03/21	North America / Mexico / Guanajuato	Human	Male	30	Ambulatory	Nasopharyngeal and oropharyngeal swab
hCoV-19/Mexico/SON_INER_IMSS_00529/2021	EPI_ISL_1585532	15/03/21	North America / Mexico / Sonora	Human	Male	26	Ambulatory	Nasopharyngeal swab
hCoV-19/Mexico/SON_INER_IMSS_00531/2021	EPI_ISL_1585534	05/03/21	North America / Mexico / Sonora	Human	Female	61	Hospitalized	Oropharyngeal swab
hCoV-19/Mexico/JAL_INER_IMSS_00533/2021	EPI_ISL_1585535	13/03/21	North America / Mexico / Jalisco	Human	Male	68	Hospitalized	Nasopharyngeal swab
hCoV-19/Mexico/JAL_INER_IMSS_00537/2021	EPI_ISL_1585539	17/03/21	North America / Mexico / Jalisco	Human	Male	58	Hospitalized	Nasopharyngeal and oropharyngeal swab
hCoV-19/Mexico/JAL_INER_IMSS_00540/2021	EPI_ISL_1585542	18/03/21	North America / Mexico / Jalisco	Human	Female	54	Ambulatory	Nasopharyngeal and oropharyngeal swab
hCoV-19/Mexico/GUA_INER_IMSS_00542/2021	EPI_ISL_1585543	17/03/21	North America / Mexico / Guanajuato	Human	Female	65	Ambulatory	Nasopharyngeal and oropharyngeal swab
hCoV-19/Mexico/SON_INER_IMSS_00543/2021	EPI_ISL_1585544	16/03/21	North America / Mexico / Sonora	Human	Female	47	Ambulatory	Nasopharyngeal swab
hCoV-19/Mexico/SON_INER_IMSS_00549/2021	EPI_ISL_1585549	17/03/21	North America / Mexico / Sonora	Human	Female	40	Ambulatory	Nasopharyngeal and oropharyngeal swab
hCoV-19/Mexico/SIN_INER_IMSS_00550/2021	EPI_ISL_1585550	17/03/21	North America / Mexico / Sinaloa	Human	Female	72	Hospitalized	Nasopharyngeal swab
hCoV-19/Mexico/SON_INER_IMSS_00552/2021	EPI_ISL_1585552	15/03/21	North America / Mexico / Sonora	Human	Female	85	Hospitalized	Nasopharyngeal swab
hCoV-19/Mexico/SON_INER_IMSS_00553/2021	EPI_ISL_1585553	15/03/21	North America / Mexico / Sonora	Human	Male	71	Hospitalized	Nasopharyngeal swab
hCoV-19/Mexico/JAL_INER_IMSS_00556/2021	EPI_ISL_1585555	20/03/21	North America / Mexico / Jalisco	Human	Female	29	Hospitalized	Nasopharyngeal and oropharyngeal swab
hCoV-19/Mexico/JAL_INER_IMSS_00557/2021	EPI_ISL_1585556	20/03/21	North America / Mexico / Jalisco	Human	Male	85	Hospitalized	Nasopharyngeal and oropharyngeal swab
hCoV-19/Mexico/JAL_INER_IMSS_00558/2021	EPI_ISL_1585557	19/03/21	North America / Mexico / Jalisco	Human	Male	43	Hospitalized	Nasopharyngeal and oropharyngeal swab
hCoV-19/Mexico/JAL_INER_IMSS_00559/2021	EPI_ISL_1585558	19/03/21	North America / Mexico / Jalisco	Human	Male	66	Hospitalized	Nasopharyngeal and oropharyngeal swab
hCoV-19/Mexico/JAL_INER_IMSS_00560/2021	EPI_ISL_1585559	19/03/21	North America / Mexico / Jalisco	Human	Female	82	Hospitalized	Nasopharyngeal and oropharyngeal swab
hCoV-19/Mexico/JAL_INER_IMSS_00561/2021	EPI_ISL_1585560	20/03/21	North America / Mexico / Jalisco	Human	Male	67	Hospitalized	Nasopharyngeal and oropharyngeal swab
hCoV-19/Mexico/JAL_INER_IMSS_00562/2021	EPI_ISL_1585561	20/03/21	North America / Mexico / Jalisco	Human	Male	62	Hospitalized	Nasopharyngeal and oropharyngeal swab
hCoV-19/Mexico/JAL_INER_IMSS_00563/2021	EPI_ISL_1585562	21/03/21	North America / Mexico / Jalisco	Human	Female	35	Ambulatory	Nasopharyngeal and oropharyngeal swab
hCoV-19/Mexico/MOR_INER_IMSS_00565/2021	EPI_ISL_1585564	15/03/21	North America / Mexico / Morelos	Human	Male	62	Hospitalized	Nasopharyngeal and oropharyngeal swab
hCoV-19/Mexico/VER_INER_IMSS_00566/2021	EPI_ISL_1585565	12/03/21	North America / Mexico / Veracruz	Human	Male	26	Ambulatory	Nasopharyngeal and oropharyngeal swab
hCoV-19/Mexico/VER_INER_IMSS_00567/2021	EPI_ISL_1585566	16/03/21	North America / Mexico / Veracruz	Human	Female	80	Hospitalized	Nasopharyngeal and oropharyngeal swab
hCoV-19/Mexico/VER_INER_IMSS_00568/2021	EPI_ISL_1585567	14/03/21	North America / Mexico / Veracruz	Human	Female	58	Hospitalized	Nasopharyngeal and oropharyngeal swab
hCoV-19/Mexico/VER_INER_IMSS_00569/2021	EPI_ISL_1585568	15/03/21	North America / Mexico / Veracruz	Human	Male	80	Hospitalized	Nasopharyngeal and oropharyngeal swab
hCoV-19/Mexico/VER_INER_IMSS_00570/2021	EPI_ISL_1585569	15/03/21	North America / Mexico / Veracruz	Human	Female	66	Hospitalized	Nasopharyngeal and oropharyngeal swab
hCoV-19/Mexico/HID_INER_IMSS_00571/2021	EPI_ISL_1585570	17/03/21	North America / Mexico / Hidalgo	Human	Female	25	Ambulatory	Nasopharyngeal and oropharyngeal swab
hCoV-19/Mexico/HID_INER_IMSS_00572/2021	EPI_ISL_1585571	17/03/21	North America / Mexico / Hidalgo	Human	Male	36	Ambulatory	Oropharyngeal swab
hCoV-19/Mexico/HID_INER_IMSS_00573/2021	EPI_ISL_1585572	16/03/21	North America / Mexico / Hidalgo	Human	Male	90	Hospitalized	Nasopharyngeal and oropharyngeal swab
hCoV-19/Mexico/VER_INER_IMSS_00574/2021	EPI_ISL_1585573	13/03/21	North America / Mexico / Veracruz	Human	Male	34	Ambulatory	Nasopharyngeal swab
hCoV-19/Mexico/VER_INER_IMSS_00575/2021	EPI_ISL_1585574	13/03/21	North America / Mexico / Veracruz	Human	Male	30	Ambulatory	Nasopharyngeal swab
hCoV-19/Mexico/VER_INER_IMSS_00576/2021	EPI_ISL_1585575	12/03/21	North America / Mexico / Veracruz	Human	Female	26	Ambulatory	Nasopharyngeal and oropharyngeal swab
hCoV-19/Mexico/VER_INER_IMSS_00577/2021	EPI_ISL_1585576	12/03/21	North America / Mexico / Veracruz	Human	Female	23	Ambulatory	Nasopharyngeal and oropharyngeal swab
hCoV-19/Mexico/VER_INER_IMSS_00578/2021	EPI_ISL_1585577	16/03/21	North America / Mexico / Veracruz	Human	Male	25	Ambulatory	Nasopharyngeal and oropharyngeal swab
hCoV-19/Mexico/VER_INER_IMSS_00579/2021	EPI_ISL_1585578	16/03/21	North America / Mexico / Veracruz	Human	Male	46	Ambulatory	Nasopharyngeal and oropharyngeal swab
hCoV-19/Mexico/VER_INER_IMSS_00580/2021	EPI_ISL_1585579	16/03/21	North America / Mexico / Veracruz	Human	Male	55	Ambulatory	Nasopharyngeal and oropharyngeal swab
hCoV-19/Mexico/VER_INER_IMSS_00581/2021	EPI_ISL_1585580	16/03/21	North America / Mexico / Veracruz	Human	Male	33	Ambulatory	Nasopharyngeal and oropharyngeal swab
hCoV-19/Mexico/VER_INER_IMSS_00582/2021	EPI_ISL_1585581	16/03/21	North America / Mexico / Veracruz	Human	Male	53	Hospitalized	Nasopharyngeal and oropharyngeal swab

hCoV-19/Mexico/AGU_INER_IMSS_00583/2021	EPI_ISL_1585582	14/03/21	North America / Mexico / Aguascalientes	Human	Female	48	Ambulatory	Nasopharyngeal and oropharyngeal swab
hCoV-19/Mexico/TLA_INER_IMSS_00585/2021	EPI_ISL_1585584	16/03/21	North America / Mexico / Tlaxcala	Human	Male	56	Hospitalized	Nasopharyngeal and oropharyngeal swab
hCoV-19/Mexico/TLA_INER_IMSS_00586/2021	EPI_ISL_1585585	16/03/21	North America / Mexico / Tlaxcala	Human	Female	75	Hospitalized	Nasopharyngeal and oropharyngeal swab
hCoV-19/Mexico/TLA_INER_IMSS_00587/2021	EPI_ISL_1585586	17/03/21	North America / Mexico / Tlaxcala	Human	Male	75	Hospitalized	Nasopharyngeal and oropharyngeal swab
hCoV-19/Mexico/TLA_INER_IMSS_00589/2021	EPI_ISL_1585587	16/03/21	North America / Mexico / Tlaxcala	Human	Male	59	Hospitalized	Nasopharyngeal and oropharyngeal swab
hCoV-19/Mexico/TLA_INER_IMSS_00590/2021	EPI_ISL_1585588	14/03/21	North America / Mexico / Tlaxcala	Human	Female	52	Hospitalized	Nasopharyngeal and oropharyngeal swab
hCoV-19/Mexico/MOR_INER_IMSS_00592/2021	EPI_ISL_1585589	15/03/21	North America / Mexico / Morelos	Human	Female	80	Hospitalized	Oropharyngeal swab
hCoV-19/Mexico/VER_INER_IMSS_00593/2021	EPI_ISL_1585590	16/03/21	North America / Mexico / Veracruz	Human	Female	25	Ambulatory	Nasopharyngeal and oropharyngeal swab
hCoV-19/Mexico/VER_INER_IMSS_00594/2021	EPI_ISL_1585591	16/03/21	North America / Mexico / Veracruz	Human	Male	28	Ambulatory	Nasopharyngeal and oropharyngeal swab
hCoV-19/Mexico/VER_INER_IMSS_00595/2021	EPI_ISL_1585592	15/03/21	North America / Mexico / Veracruz	Human	Female	34	Hospitalized	Nasopharyngeal and oropharyngeal swab
hCoV-19/Mexico/VER_INER_IMSS_00596/2021	EPI_ISL_1585593	14/03/21	North America / Mexico / Veracruz	Human	Male	32	Hospitalized	Nasopharyngeal and oropharyngeal swab
hCoV-19/Mexico/VER_INER_IMSS_00597/2021	EPI_ISL_1585594	14/03/21	North America / Mexico / Veracruz	Human	Female	23	Ambulatory	Nasopharyngeal and oropharyngeal swab
hCoV-19/Mexico/SLP_INER_IMSS_00599/2021	EPI_ISL_1585596	18/03/21	North America / Mexico / San Luis Potosi	Human	Female	83	Hospitalized	Nasopharyngeal and oropharyngeal swab
hCoV-19/Mexico/SLP_INER_IMSS_00600/2021	EPI_ISL_1585597	12/03/21	North America / Mexico / San Luis Potosi	Human	Male	86	Hospitalized	Nasopharyngeal and oropharyngeal swab
hCoV-19/Mexico/MEX_INER_IMSS_00601/2021	EPI_ISL_1585598	18/03/21	North America / Mexico / State of Mexico	Human	Male	40	Ambulatory	Nasopharyngeal and oropharyngeal swab
hCoV-19/Mexico/TLA_INER_IMSS_00602/2021	EPI_ISL_1585599	17/03/21	North America / Mexico / Tlaxcala	Human	Male	66	Hospitalized	Nasopharyngeal and oropharyngeal swab
hCoV-19/Mexico/TLA_INER_IMSS_00603/2021	EPI_ISL_1585600	17/03/21	North America / Mexico / Tlaxcala	Human	Male	33	Hospitalized	Nasopharyngeal and oropharyngeal swab
hCoV-19/Mexico/SLP_INER_IMSS_00606/2021	EPI_ISL_1585603	18/03/21	North America / Mexico / San Luis Potosi	Human	Male	64	Hospitalized	Nasopharyngeal and oropharyngeal swab
hCoV-19/Mexico/SLP_INER_IMSS_00607/2021	EPI_ISL_1585604	17/03/21	North America / Mexico / San Luis Potosi	Human	Female	53	Ambulatory	Nasopharyngeal and oropharyngeal swab
hCoV-19/Mexico/SLP_INER_IMSS_00608/2021	EPI_ISL_1585605	16/03/21	North America / Mexico / San Luis Potosi	Human	Male	31	Ambulatory	Nasopharyngeal and oropharyngeal swab
hCoV-19/Mexico/SLP_INER_IMSS_00609/2021	EPI_ISL_1585606	16/03/21	North America / Mexico / San Luis Potosi	Human	Female	53	Ambulatory	Nasopharyngeal and oropharyngeal swab
hCoV-19/Mexico/MEX_INER_IMSS_00610/2021	EPI_ISL_1585607	18/03/21	North America / Mexico / State of Mexico	Human	Male	64	Hospitalized	Nasopharyngeal swab
hCoV-19/Mexico/MEX_INER_IMSS_00611/2021	EPI_ISL_1585608	19/03/21	North America / Mexico / State of Mexico	Human	Male	40	Ambulatory	Oropharyngeal swab
hCoV-19/Mexico/MEX_INER_IMSS_00612/2021	EPI_ISL_1585609	18/03/21	North America / Mexico / State of Mexico	Human	Male	37	Ambulatory	Oropharyngeal swab
hCoV-19/Mexico/MEX_INER_IMSS_00614/2021	EPI_ISL_1585611	18/03/21	North America / Mexico / State of Mexico	Human	Male	58	Ambulatory	Nasopharyngeal and oropharyngeal swab
hCoV-19/Mexico/VER_INER_IMSS_00616/2021	EPI_ISL_1585613	17/03/21	North America / Mexico / Veracruz	Human	Female	37	Ambulatory	Nasopharyngeal and oropharyngeal swab
hCoV-19/Mexico/HID_INER_IMSS_00617/2021	EPI_ISL_1585614	18/03/21	North America / Mexico / Hidalgo	Human	Male	72	Hospitalized	Oropharyngeal swab
hCoV-19/Mexico/HID_INER_IMSS_00618/2021	EPI_ISL_1585615	17/03/21	North America / Mexico / Hidalgo	Human	Female	64	Hospitalized	Nasopharyngeal and oropharyngeal swab
hCoV-19/Mexico/HID_INER_IMSS_00619/2021	EPI_ISL_1585616	18/03/21	North America / Mexico / Hidalgo	Human	Male	43	Hospitalized	Nasopharyngeal and oropharyngeal swab
hCoV-19/Mexico/MOR_INER_IMSS_00620/2021	EPI_ISL_1585617	17/03/21	North America / Mexico / Morelos	Human	Male	38	Hospitalized	Nasopharyngeal and oropharyngeal swab
hCoV-19/Mexico/GRO_INER_IMSS_00621/2021	EPI_ISL_1585618	19/03/21	North America / Mexico / Guerrero	Human	Male	48	Ambulatory	Nasopharyngeal and oropharyngeal swab
hCoV-19/Mexico/GRO_INER_IMSS_00622/2021	EPI_ISL_1585619	17/03/21	North America / Mexico / Guerrero	Human	Female	52	Ambulatory	Nasopharyngeal and oropharyngeal swab
hCoV-19/Mexico/SLP_INER_IMSS_00623/2021	EPI_ISL_1585620	18/03/21	North America / Mexico / San Luis Potosi	Human	Male	0	Hospitalized	Nasopharyngeal and oropharyngeal swab
hCoV-19/Mexico/GRO_INER_IMSS_00624/2021	EPI_ISL_1585621	19/03/21	North America / Mexico / Guerrero	Human	Male	86	Hospitalized	Nasopharyngeal and oropharyngeal swab
hCoV-19/Mexico/GRO_INER_IMSS_00625/2021	EPI_ISL_1585622	19/03/21	North America / Mexico / Guerrero	Human	Male	45	Ambulatory	Nasopharyngeal and oropharyngeal swab
hCoV-19/Mexico/SLP_INER_IMSS_00626/2021	EPI_ISL_1585623	18/03/21	North America / Mexico / San Luis Potosi	Human	Female	79	Hospitalized	Nasopharyngeal and oropharyngeal swab
hCoV-19/Mexico/SLP_INER_IMSS_00627/2021	EPI_ISL_1585624	16/03/21	North America / Mexico / San Luis Potosi	Human	Male	45	Hospitalized	Nasopharyngeal and oropharyngeal swab
hCoV-19/Mexico/TLA_INER_IMSS_00629/2021	EPI_ISL_1585626	19/03/21	North America / Mexico / Tlaxcala	Human	Male	49	Hospitalized	Nasopharyngeal and oropharyngeal swab
hCoV-19/Mexico/MEX_INER_IMSS_00631/2021	EPI_ISL_1585627	20/03/21	North America / Mexico / State of Mexico	Human	Male	73	Hospitalized	Nasopharyngeal and oropharyngeal swab
hCoV-19/Mexico/CMX_INER_IMSS_00632/2021	EPI_ISL_1585628	20/03/21	North America / Mexico / Mexico City	Human	Male	64	Hospitalized	Nasopharyngeal and oropharyngeal swab
hCoV-19/Mexico/MEX_INER_IMSS_00633/2021	EPI_ISL_1585629	18/03/21	North America / Mexico / State of Mexico	Human	Male	70	Hospitalized	Nasopharyngeal and oropharyngeal swab
hCoV-19/Mexico/VER_INER_IMSS_00634/2021	EPI_ISL_1585630	18/03/21	North America / Mexico / Veracruz	Human	Male	85	Hospitalized	Nasopharyngeal and oropharyngeal swab
hCoV-19/Mexico/AGU_INER_IMSS_00635/2021	EPI_ISL_1585631	18/03/21	North America / Mexico / Aguascalientes	Human	Male	52	Ambulatory	Nasopharyngeal and oropharyngeal swab
hCoV-19/Mexico/QUE_INER_IMSS_00636/2021	EPI_ISL_1585632	19/03/21	North America / Mexico / Queretaro	Human	Male	38	Hospitalized	Nasopharyngeal and oropharyngeal swab

hCoV-19/Mexico/QUE_INER_IMSS_00637/2021	EPI_ISL_1585633	19/03/21	North America / Mexico / Queretaro	Human	Male	85	Hospitalized	Nasopharyngeal and oropharyngeal swab
hCoV-19/Mexico/MEX_INER_IMSS_00638/2021	EPI_ISL_1585634	19/03/21	North America / Mexico / State of Mexico	Human	Male	68	Hospitalized	Nasopharyngeal and oropharyngeal swab
hCoV-19/Mexico/MEX_INER_IMSS_00639/2021	EPI_ISL_1585635	20/03/21	North America / Mexico / State of Mexico	Human	Female	83	Hospitalized	Nasopharyngeal and oropharyngeal swab
hCoV-19/Mexico/CMX_INER_IMSS_00640/2021	EPI_ISL_1585636	20/03/21	North America / Mexico / Mexico City	Human	Male	75	Hospitalized	Nasopharyngeal and oropharyngeal swab
hCoV-19/Mexico/CMX_INER_IMSS_00641/2021	EPI_ISL_1585637	20/03/21	North America / Mexico / Mexico City	Human	Male	34	Ambulatory	Oropharyngeal swab
hCoV-19/Mexico/MOR_INER_IMSS_00642/2021	EPI_ISL_1585638	18/03/21	North America / Mexico / Morelos	Human	Male	86	Hospitalized	Nasopharyngeal and oropharyngeal swab
hCoV-19/Mexico/CMX_INER_IMSS_00643/2021	EPI_ISL_1585639	20/03/21	North America / Mexico / Mexico City	Human	Male	47	Hospitalized	Oropharyngeal swab
hCoV-19/Mexico/MOR_INER_IMSS_00645/2021	EPI_ISL_1585640	19/03/21	North America / Mexico / Morelos	Human	Male	35	Hospitalized	Nasopharyngeal and oropharyngeal swab
hCoV-19/Mexico/MOR_INER_IMSS_00646/2021	EPI_ISL_1585641	17/03/21	North America / Mexico / Morelos	Human	Male	31	Hospitalized	Nasopharyngeal and oropharyngeal swab
hCoV-19/Mexico/MOR_INER_IMSS_00647/2021	EPI_ISL_1585642	17/03/21	North America / Mexico / Morelos	Human	Female	59	Hospitalized	Nasopharyngeal and oropharyngeal swab
hCoV-19/Mexico/MOR_INER_IMSS_00648/2021	EPI_ISL_1585643	18/03/21	North America / Mexico / Morelos	Human	Female	68	Hospitalized	Nasopharyngeal and oropharyngeal swab
hCoV-19/Mexico/HID_INER_IMSS_00649/2021	EPI_ISL_1585644	18/03/21	North America / Mexico / Hidalgo	Human	Female	42	Hospitalized	Nasopharyngeal and oropharyngeal swab
hCoV-19/Mexico/MEX_INER_IMSS_00650/2021	EPI_ISL_1585645	18/03/21	North America / Mexico / State of Mexico	Human	Female	59	Hospitalized	Nasopharyngeal and oropharyngeal swab
hCoV-19/Mexico/MEX_INER_IMSS_00651/2021	EPI_ISL_1585646	21/03/21	North America / Mexico / State of Mexico	Human	Male	88	Hospitalized	Nasopharyngeal and oropharyngeal swab
hCoV-19/Mexico/MEX_INER_IMSS_00653/2021	EPI_ISL_1585648	21/03/21	North America / Mexico / State of Mexico	Human	Male	65	Hospitalized	Nasopharyngeal and oropharyngeal swab
hCoV-19/Mexico/MEX_INER_IMSS_00654/2021	EPI_ISL_1585649	19/03/21	North America / Mexico / State of Mexico	Human	Female	58	Hospitalized	Nasopharyngeal and oropharyngeal swab
hCoV-19/Mexico/CMX_INER_IMSS_00655/2021	EPI_ISL_1585650	20/03/21	North America / Mexico / Mexico City	Human	Male	77	Hospitalized	Nasopharyngeal swab
hCoV-19/Mexico/CMX_INER_IMSS_00656/2021	EPI_ISL_1585651	20/03/21	North America / Mexico / Mexico City	Human	Female	57	Hospitalized	Nasopharyngeal swab
hCoV-19/Mexico/CMX_INER_IMSS_00657/2021	EPI_ISL_1585652	20/03/21	North America / Mexico / Mexico City	Human	Female	46	Hospitalized	Nasopharyngeal swab
hCoV-19/Mexico/CMX_INER_IMSS_00658/2021	EPI_ISL_1585653	21/03/21	North America / Mexico / Mexico City	Human	Female	66	Hospitalized	Nasopharyngeal swab
hCoV-19/Mexico/CMX_INER_IMSS_00659/2021	EPI_ISL_1585654	20/03/21	North America / Mexico / Mexico City	Human	Male	70	Hospitalized	Oropharyngeal swab
hCoV-19/Mexico/CMX_INER_IMSS_00660/2021	EPI_ISL_1585655	21/03/21	North America / Mexico / Mexico City	Human	Female	34	Hospitalized	Oropharyngeal swab
hCoV-19/Mexico/CMX_INER_IMSS_00661/2021	EPI_ISL_1585656	21/03/21	North America / Mexico / Mexico City	Human	Female	49	Hospitalized	Oropharyngeal swab
hCoV-19/Mexico/CMX_INER_IMSS_00662/2021	EPI_ISL_1585657	20/03/21	North America / Mexico / Mexico City	Human	Female	44	Hospitalized	Oropharyngeal swab
hCoV-19/Mexico/SLP_INER_IMSS_00664/2021	EPI_ISL_1585659	18/03/21	North America / Mexico / San Luis Potosi	Human	Male	84	Hospitalized	Nasopharyngeal and oropharyngeal swab
hCoV-19/Mexico/TLA_INER_IMSS_00665/2021	EPI_ISL_1585660	19/03/21	North America / Mexico / Tlaxcala	Human	Male	79	Hospitalized	Nasopharyngeal and oropharyngeal swab
hCoV-19/Mexico/CMX_INER_IMSS_00666/2021	EPI_ISL_1585661	20/03/21	North America / Mexico / Mexico City	Human	Female	72	Hospitalized	Nasopharyngeal and oropharyngeal swab
hCoV-19/Mexico/CMX_INER_IMSS_00667/2021	EPI_ISL_1585662	20/03/21	North America / Mexico / Mexico City	Human	Female	67	Hospitalized	Nasopharyngeal and oropharyngeal swab
hCoV-19/Mexico/CMX_INER_IMSS_00668/2021	EPI_ISL_1585663	19/03/21	North America / Mexico / Mexico City	Human	Male	31	Ambulatory	Nasopharyngeal and oropharyngeal swab
hCoV-19/Mexico/CMX_INER_IMSS_00669/2021	EPI_ISL_1585664	19/03/21	North America / Mexico / Mexico City	Human	Female	31	Ambulatory	Nasopharyngeal and oropharyngeal swab
hCoV-19/Mexico/CMX_INER_IMSS_00670/2021	EPI_ISL_1585665	21/03/21	North America / Mexico / Mexico City	Human	Female	65	Ambulatory	Oropharyngeal swab
hCoV-19/Mexico/OAX_INER_IMSS_00671/2021	EPI_ISL_1585666	19/03/21	North America / Mexico / Oaxaca	Human	Male	33	Ambulatory	Nasopharyngeal and oropharyngeal swab
hCoV-19/Mexico/CMX_INER_IMSS_00672/2021	EPI_ISL_1585667	19/03/21	North America / Mexico / Mexico City	Human	Male	48	Ambulatory	Nasopharyngeal and oropharyngeal swab
hCoV-19/Mexico/MEX_INER_IMSS_00673/2021	EPI_ISL_1585668	20/03/21	North America / Mexico / State of Mexico	Human	Female	50	Hospitalized	Oropharyngeal swab
hCoV-19/Mexico/CMX_INER_IMSS_00674/2021	EPI_ISL_1585669	19/03/21	North America / Mexico / Mexico City	Human	Male	30	Ambulatory	Oropharyngeal swab
hCoV-19/Mexico/CMX_INER_IMSS_00675/2021	EPI_ISL_1585670	19/03/21	North America / Mexico / Mexico City	Human	Female	62	Hospitalized	Nasopharyngeal and oropharyngeal swab
hCoV-19/Mexico/CMX-INMEGEN-03-11-01/2021	EPI_ISL_1591460	17/02/21	North America / Mexico / Mexico City	Human	Female	26	unknown	Oropharyngeal swab
hCoV-19/Mexico/CMX-INMEGEN-03-11-02/2021	EPI_ISL_1591461	18/02/21	North America / Mexico / Mexico City	Human	Male	59	unknown	Oropharyngeal swab
hCoV-19/Mexico/CMX-INMEGEN-03-11-03/2021	EPI_ISL_1591462	22/02/21	North America / Mexico / Mexico City	Human	Male	21	unknown	Oropharyngeal swab
hCoV-19/Mexico/CMX-INMEGEN-03-11-04/2021	EPI_ISL_1591463	25/02/21	North America / Mexico / Mexico City	Human	Male	13	unknown	Oropharyngeal swab
hCoV-19/Mexico/CMX-INMEGEN-03-11-05/2021	EPI_ISL_1591464	02/03/21	North America / Mexico / Mexico City	Human	Female	23	unknown	Oropharyngeal swab
hCoV-19/Mexico/CMX-INMEGEN-03-11-06/2021	EPI_ISL_1591465	01/03/21	North America / Mexico / Mexico City	Human	Female	45	unknown	Oropharyngeal swab
hCoV-19/Mexico/CMX-INMEGEN-03-11-07/2021	EPI_ISL_1591466	02/03/21	North America / Mexico / Mexico City	Human	Male	28	unknown	Oropharyngeal swab
hCoV-19/Mexico/CMX-INMEGEN-03-11-08/2021	EPI_ISL_1591467	26/02/21	North America / Mexico / Mexico City	Human	Female	23	unknown	Oropharyngeal swab

hCoV-19/Mexico/CMX-INMEGEN-03-11-09/2021	EPI_ISL_1591468	17/02/21	North America / Mexico / Mexico City	Human	Male	57	unknown	Oropharyngeal swab
hCoV-19/Mexico/CMX-INMEGEN-03-11-11/2021	EPI_ISL_1591470	22/02/21	North America / Mexico / Mexico City	Human	Female	47	unknown	Oropharyngeal swab
hCoV-19/Mexico/CMX-INMEGEN-03-11-12/2021	EPI_ISL_1591471	26/02/21	North America / Mexico / Mexico City	Human	Female	31	unknown	Oropharyngeal swab
hCoV-19/Mexico/CMX-INMEGEN-03-11-13/2021	EPI_ISL_1591472	03/03/21	North America / Mexico / Mexico City	Human	Female	66	unknown	Oropharyngeal swab
hCoV-19/Mexico/CMX-INMEGEN-03-11-14/2021	EPI_ISL_1591473	02/03/21	North America / Mexico / Mexico City	Human	Male	51	unknown	Oropharyngeal swab
hCoV-19/Mexico/CMX-INMEGEN-03-11-15/2021	EPI_ISL_1591474	02/03/21	North America / Mexico / Mexico City	Human	Female	25	unknown	Oropharyngeal swab
hCoV-19/Mexico/CMX-INMEGEN-03-11-16/2021	EPI_ISL_1591475	27/02/21	North America / Mexico / Mexico City	Human	Female	29	unknown	Oropharyngeal swab
hCoV-19/Mexico/CMX-INMEGEN-03-11-17/2021	EPI_ISL_1591476	16/02/21	North America / Mexico / Mexico City	Human	Male	24	unknown	Oropharyngeal swab
hCoV-19/Mexico/CMX-INMEGEN-03-11-18/2021	EPI_ISL_1591477	18/02/21	North America / Mexico / Mexico City	Human	Female	39	unknown	Oropharyngeal swab
hCoV-19/Mexico/CMX-INMEGEN-03-11-19/2021	EPI_ISL_1591478	22/02/21	North America / Mexico / Mexico City	Human	Female	41	unknown	Oropharyngeal swab
hCoV-19/Mexico/CMX-INMEGEN-03-11-20/2021	EPI_ISL_1591479	26/02/21	North America / Mexico / Mexico City	Human	Female	35	unknown	Oropharyngeal swab
hCoV-19/Mexico/CMX-INMEGEN-03-11-22/2021	EPI_ISL_1591480	01/03/21	North America / Mexico / Mexico City	Human	Male	46	unknown	Oropharyngeal swab
hCoV-19/Mexico/CMX-INMEGEN-03-11-23/2021	EPI_ISL_1591481	02/03/21	North America / Mexico / Mexico City	Human	Female	49	unknown	Oropharyngeal swab
hCoV-19/Mexico/CMX-INMEGEN-03-11-25/2021	EPI_ISL_1591482	17/02/21	North America / Mexico / Mexico City	Human	Male	72	unknown	Oropharyngeal swab
hCoV-19/Mexico/CMX-INMEGEN-03-11-26/2021	EPI_ISL_1591483	18/02/21	North America / Mexico / Mexico City	Human	Male	31	unknown	Oropharyngeal swab
hCoV-19/Mexico/CMX-INMEGEN-03-11-27/2021	EPI_ISL_1591484	22/02/21	North America / Mexico / Mexico City	Human	Male	13	unknown	Oropharyngeal swab
hCoV-19/Mexico/CMX-INMEGEN-03-11-28/2021	EPI_ISL_1591485	26/02/21	North America / Mexico / Mexico City	Human	Female	23	unknown	Oropharyngeal swab
hCoV-19/Mexico/CMX-INMEGEN-03-11-30/2021	EPI_ISL_1591487	02/03/21	North America / Mexico / Mexico City	Human	Female	43	unknown	Oropharyngeal swab
hCoV-19/Mexico/CMX-INMEGEN-03-11-32/2021	EPI_ISL_1591489	22/02/21	North America / Mexico / Mexico City	Human	Male	52	unknown	Oropharyngeal swab
hCoV-19/Mexico/CMX-INMEGEN-03-11-34/2021	EPI_ISL_1591490	19/02/21	North America / Mexico / Mexico City	Human	Female	50	unknown	Oropharyngeal swab
hCoV-19/Mexico/CMX-INMEGEN-03-11-35/2021	EPI_ISL_1591491	22/02/21	North America / Mexico / Mexico City	Human	Female	43	unknown	Oropharyngeal swab
hCoV-19/Mexico/CMX-INMEGEN-03-11-36/2021	EPI_ISL_1591492	02/03/21	North America / Mexico / Mexico City	Human	Female	72	unknown	Oropharyngeal swab
hCoV-19/Mexico/CMX-INMEGEN-03-11-37/2021	EPI_ISL_1591493	03/03/21	North America / Mexico / Mexico City	Human	Male	47	unknown	Oropharyngeal swab
hCoV-19/Mexico/CMX-INMEGEN-03-11-38/2021	EPI_ISL_1591494	02/03/21	North America / Mexico / Mexico City	Human	Female	18	unknown	Oropharyngeal swab
hCoV-19/Mexico/CMX-INMEGEN-03-11-40/2021	EPI_ISL_1591495	22/02/21	North America / Mexico / Mexico City	Human	Male	41	unknown	Oropharyngeal swab
hCoV-19/Mexico/CMX-INMEGEN-03-11-42/2021	EPI_ISL_1591496	19/02/21	North America / Mexico / Mexico City	Human	Male	50	unknown	Oropharyngeal swab
hCoV-19/Mexico/CMX-INMEGEN-03-11-43/2021	EPI_ISL_1591497	24/02/21	North America / Mexico / Mexico City	Human	Female	44	unknown	Oropharyngeal swab
hCoV-19/Mexico/CMX-INMEGEN-03-11-44/2021	EPI_ISL_1591498	02/03/21	North America / Mexico / Mexico City	Human	Female	47	unknown	Oropharyngeal swab
hCoV-19/Mexico/CMX-INMEGEN-03-11-45/2021	EPI_ISL_1591499	03/03/21	North America / Mexico / Mexico City	Human	Female	64	unknown	Oropharyngeal swab
hCoV-19/Mexico/CMX-INMEGEN-03-11-46/2021	EPI_ISL_1591500	02/03/21	North America / Mexico / Mexico City	Human	Male	19	unknown	Oropharyngeal swab
hCoV-19/Mexico/CMX-INMEGEN-03-11-47/2021	EPI_ISL_1591501	12/03/21	North America / Mexico / Mexico City	Human	Female	59	unknown	Oropharyngeal swab
hCoV-19/Mexico/CMX-INMEGEN-03-11-48/2021	EPI_ISL_1591502	05/04/21	North America / Mexico / Mexico City	Human	Female	36	unknown	Oropharyngeal swab
hCoV-19/Mexico/CMX-INMEGEN-03-11-49/2021	EPI_ISL_1591503	18/02/21	North America / Mexico / Mexico City	Human	Male	29	unknown	Oropharyngeal swab
hCoV-19/Mexico/CMX-INMEGEN-03-11-51/2021	EPI_ISL_1591505	23/02/21	North America / Mexico / Mexico City	Human	Female	39	unknown	Oropharyngeal swab
hCoV-19/Mexico/CMX-INMEGEN-03-11-52/2021	EPI_ISL_1591506	03/03/21	North America / Mexico / Mexico City	Human	Female	96	unknown	Oropharyngeal swab
hCoV-19/Mexico/CMX-INMEGEN-03-11-53/2021	EPI_ISL_1591507	03/03/21	North America / Mexico / Mexico City	Human	Male	61	unknown	Oropharyngeal swab
hCoV-19/Mexico/CMX-INMEGEN-03-11-54/2021	EPI_ISL_1591508	03/03/21	North America / Mexico / Mexico City	Human	Female	43	unknown	Oropharyngeal swab
hCoV-19/Mexico/CMX-INMEGEN-03-11-55/2021	EPI_ISL_1591509	12/03/21	North America / Mexico / Mexico City	Human	Female	64	unknown	Oropharyngeal swab
hCoV-19/Mexico/CMX-INMEGEN-03-11-56/2021	EPI_ISL_1591510	05/04/21	North America / Mexico / Mexico City	Human	Male	24	unknown	Oropharyngeal swab
hCoV-19/Mexico/CMX-INMEGEN-03-11-57/2021	EPI_ISL_1591511	18/02/21	North America / Mexico / Mexico City	Human	Female	71	unknown	Oropharyngeal swab
hCoV-19/Mexico/CMX-INMEGEN-03-11-59/2021	EPI_ISL_1591513	22/02/21	North America / Mexico / Mexico City	Human	Male	36	unknown	Oropharyngeal swab
hCoV-19/Mexico/CMX-INMEGEN-03-11-60/2021	EPI_ISL_1591514	02/03/21	North America / Mexico / Mexico City	Human	Male	69	unknown	Oropharyngeal swab
hCoV-19/Mexico/CMX-INMEGEN-03-11-62/2021	EPI_ISL_1591515	04/03/21	North America / Mexico / Mexico City	Human	Male	72	unknown	Oropharyngeal swab
hCoV-19/Mexico/CMX-INMEGEN-03-11-63/2021	EPI_ISL_1591516	12/03/21	North America / Mexico / Mexico City	Human	Female	74	unknown	Oropharyngeal swab

hCoV-19/Mexico/CMX-INMEGEN-03-11-64/2021	EPI_ISL_1591517	05/04/21	North America / Mexico / Mexico City	Human	Female	49	unknown	Oropharyngeal swab
hCoV-19/Mexico/CMX-INMEGEN-03-11-68/2021	EPI_ISL_1591521	02/03/21	North America / Mexico / Mexico City	Human	Female	79	unknown	Oropharyngeal swab
hCoV-19/Mexico/CMX-INMEGEN-03-11-70/2021	EPI_ISL_1591522	03/03/21	North America / Mexico / Mexico City	Human	Female	65	unknown	Oropharyngeal swab
hCoV-19/Mexico/CMX-INMEGEN-03-11-72/2021	EPI_ISL_1591523	05/04/21	North America / Mexico / Mexico City	Human	Female	49	unknown	Oropharyngeal swab
hCoV-19/Mexico/CMX-INMEGEN-03-11-73/2021	EPI_ISL_1591524	18/02/21	North America / Mexico / Mexico City	Human	Male	26	unknown	Oropharyngeal swab
hCoV-19/Mexico/CMX-INMEGEN-03-11-74/2021	EPI_ISL_1591525	18/02/21	North America / Mexico / Mexico City	Human	Female	42	unknown	Oropharyngeal swab
hCoV-19/Mexico/CMX-INMEGEN-03-11-76/2021	EPI_ISL_1591526	02/03/21	North America / Mexico / Mexico City	Human	Male	21	unknown	Oropharyngeal swab
hCoV-19/Mexico/CMX-INMEGEN-03-11-77/2021	EPI_ISL_1591527	04/03/21	North America / Mexico / Mexico City	Human	Female	88	unknown	Oropharyngeal swab
hCoV-19/Mexico/CMX-INMEGEN-03-11-78/2021	EPI_ISL_1591528	04/03/21	North America / Mexico / Mexico City	Human	Male	37	unknown	Oropharyngeal swab
hCoV-19/Mexico/CMX-INMEGEN-03-11-80/2021	EPI_ISL_1591529	05/04/21	North America / Mexico / Mexico City	Human	Female	44	unknown	Oropharyngeal swab
hCoV-19/Mexico/CMX-INMEGEN-03-11-81/2021	EPI_ISL_1591530	18/02/21	North America / Mexico / Mexico City	Human	Male	55	unknown	Oropharyngeal swab
hCoV-19/Mexico/CMX-INMEGEN-03-11-82/2021	EPI_ISL_1591531	18/02/21	North America / Mexico / Mexico City	Human	Male	26	unknown	Oropharyngeal swab
hCoV-19/Mexico/CMX-INMEGEN-03-11-83/2021	EPI_ISL_1591532	26/02/21	North America / Mexico / Mexico City	Human	Female	48	unknown	Oropharyngeal swab
hCoV-19/Mexico/CMX-INMEGEN-03-11-84/2021	EPI_ISL_1591533	01/03/21	North America / Mexico / Mexico City	Human	Male	49	unknown	Oropharyngeal swab
hCoV-19/Mexico/CMX-INMEGEN-03-11-85/2021	EPI_ISL_1591534	02/03/21	North America / Mexico / Mexico City	Human	Male	32	unknown	Oropharyngeal swab
hCoV-19/Mexico/CMX-INMEGEN-03-11-86/2021	EPI_ISL_1591535	04/03/21	North America / Mexico / Mexico City	Human	Female	67	unknown	Oropharyngeal swab
hCoV-19/Mexico/CMX-INMEGEN-03-11-87/2021	EPI_ISL_1591536	18/02/21	North America / Mexico / Mexico City	Human	Male	23	unknown	Oropharyngeal swab
hCoV-19/Mexico/CMX-INMEGEN-03-11-89/2021	EPI_ISL_1591537	18/02/21	North America / Mexico / Mexico City	Human	Female	58	unknown	Oropharyngeal swab
hCoV-19/Mexico/CMX-INMEGEN-03-11-90/2021	EPI_ISL_1591538	17/02/21	North America / Mexico / Mexico City	Human	Female	67	unknown	Oropharyngeal swab
hCoV-19/Mexico/CMX-INMEGEN-03-11-91/2021	EPI_ISL_1591539	26/02/21	North America / Mexico / Mexico City	Human	Female	50	unknown	Oropharyngeal swab
hCoV-19/Mexico/CMX-INMEGEN-03-11-92/2021	EPI_ISL_1591540	01/03/21	North America / Mexico / Mexico City	Human	Female	9	unknown	Oropharyngeal swab
hCoV-19/Mexico/CMX-INMEGEN-03-11-93/2021	EPI_ISL_1591541	02/03/21	North America / Mexico / Mexico City	Human	Female	27	unknown	Oropharyngeal swab
hCoV-19/Mexico/CMX-INMEGEN-03-11-94/2021	EPI_ISL_1591542	02/03/21	North America / Mexico / Mexico City	Human	Male	17	unknown	Oropharyngeal swab
hCoV-19/Mexico/CMX-INMEGEN-03-11-193/2021	EPI_ISL_1591543	24/03/21	North America / Mexico / Mexico City	Human	Female	20	unknown	Oropharyngeal swab
hCoV-19/Mexico/CMX-INMEGEN-03-11-194/2021	EPI_ISL_1591544	25/03/21	North America / Mexico / Mexico City	Human	Male	40	unknown	Oropharyngeal swab
hCoV-19/Mexico/CMX-INMEGEN-03-11-195/2021	EPI_ISL_1591545	25/03/21	North America / Mexico / Mexico City	Human	Female	77	unknown	Oropharyngeal swab
hCoV-19/Mexico/CMX-INMEGEN-03-11-196/2021	EPI_ISL_1591546	27/03/21	North America / Mexico / Mexico City	Human	Male	22	unknown	Oropharyngeal swab
hCoV-19/Mexico/CMX-INMEGEN-03-11-197/2021	EPI_ISL_1591547	29/03/21	North America / Mexico / Mexico City	Human	Male	73	unknown	Oropharyngeal swab
hCoV-19/Mexico/CMX-INMEGEN-03-11-198/2021	EPI_ISL_1591548	29/03/21	North America / Mexico / Mexico City	Human	Male	62	unknown	Oropharyngeal swab
hCoV-19/Mexico/CMX-INMEGEN-03-11-201/2021	EPI_ISL_1591550	24/03/21	North America / Mexico / Mexico City	Human	Male	23	unknown	Oropharyngeal swab
hCoV-19/Mexico/CMX-INMEGEN-03-11-202/2021	EPI_ISL_1591551	25/03/21	North America / Mexico / Mexico City	Human	Male	32	unknown	Oropharyngeal swab
hCoV-19/Mexico/CMX-INMEGEN-03-11-203/2021	EPI_ISL_1591552	25/03/21	North America / Mexico / Mexico City	Human	Female	54	unknown	Oropharyngeal swab
hCoV-19/Mexico/CMX-INMEGEN-03-11-204/2021	EPI_ISL_1591553	28/03/21	North America / Mexico / Mexico City	Human	Male	28	unknown	Oropharyngeal swab
hCoV-19/Mexico/CMX-INMEGEN-03-11-205/2021	EPI_ISL_1591554	29/03/21	North America / Mexico / Mexico City	Human	Female	29	unknown	Oropharyngeal swab
hCoV-19/Mexico/CMX-INMEGEN-03-11-206/2021	EPI_ISL_1591555	30/03/21	North America / Mexico / Mexico City	Human	Female	58	unknown	Oropharyngeal swab
hCoV-19/Mexico/CMX-INMEGEN-03-11-207/2021	EPI_ISL_1591556	24/03/21	North America / Mexico / Mexico City	Human	Female	32	unknown	Oropharyngeal swab
hCoV-19/Mexico/CMX-INMEGEN-03-11-209/2021	EPI_ISL_1591557	24/03/21	North America / Mexico / Mexico City	Human	Female	75	unknown	Oropharyngeal swab
hCoV-19/Mexico/CMX-INMEGEN-03-11-211/2021	EPI_ISL_1591559	25/03/21	North America / Mexico / Mexico City	Human	Female	48	unknown	Oropharyngeal swab
hCoV-19/Mexico/CMX-INMEGEN-03-11-212/2021	EPI_ISL_1591560	29/03/21	North America / Mexico / Mexico City	Human	Male	81	unknown	Oropharyngeal swab
hCoV-19/Mexico/CMX-INMEGEN-03-11-213/2021	EPI_ISL_1591561	29/03/21	North America / Mexico / Mexico City	Human	Female	50	unknown	Oropharyngeal swab
hCoV-19/Mexico/CMX-INMEGEN-03-11-214/2021	EPI_ISL_1591562	30/03/21	North America / Mexico / Mexico City	Human	Female	67	unknown	Oropharyngeal swab
hCoV-19/Mexico/CMX-INMEGEN-03-11-215/2021	EPI_ISL_1591563	24/03/21	North America / Mexico / Mexico City	Human	Male	63	unknown	Oropharyngeal swab
hCoV-19/Mexico/CMX-INMEGEN-03-11-216/2021	EPI_ISL_1591564	01/04/21	North America / Mexico / Mexico City	Human	Female	30	unknown	Oropharyngeal swab
hCoV-19/Mexico/CMX-INMEGEN-03-11-217/2021	EPI_ISL_1591565	24/03/21	North America / Mexico / Mexico City	Human	Female	25	unknown	Oropharyngeal swab

hCoV-19/Mexico/CMX-INMEGEN-03-11-218/2021	EPI_ISL_1591566	25/03/21	North America / Mexico / Mexico City	Human	Female	33	unknown	Oropharyngeal swab
hCoV-19/Mexico/CMX-INMEGEN-03-11-219/2021	EPI_ISL_1591567	25/03/21	North America / Mexico / Mexico City	Human	Male	48	unknown	Oropharyngeal swab
hCoV-19/Mexico/CMX-INMEGEN-03-11-220/2021	EPI_ISL_1591568	29/03/21	North America / Mexico / Mexico City	Human	Male	81	unknown	Oropharyngeal swab
hCoV-19/Mexico/CMX-INMEGEN-03-11-221/2021	EPI_ISL_1591569	29/03/21	North America / Mexico / Mexico City	Human	Male	32	unknown	Oropharyngeal swab
hCoV-19/Mexico/CMX-INMEGEN-03-11-222/2021	EPI_ISL_1591570	30/03/21	North America / Mexico / Mexico City	Human	Female	52	unknown	Oropharyngeal swab
hCoV-19/Mexico/CMX-INMEGEN-03-11-223/2021	EPI_ISL_1591571	24/03/21	North America / Mexico / Mexico City	Human	Female	61	unknown	Oropharyngeal swab
hCoV-19/Mexico/CMX-INMEGEN-03-11-225/2021	EPI_ISL_1591572	24/03/21	North America / Mexico / Mexico City	Human	Female	54	unknown	Oropharyngeal swab
hCoV-19/Mexico/CMX-INMEGEN-03-11-226/2021	EPI_ISL_1591573	25/03/21	North America / Mexico / Mexico City	Human	Female	58	unknown	Oropharyngeal swab
hCoV-19/Mexico/CMX-INMEGEN-03-11-227/2021	EPI_ISL_1591574	26/03/21	North America / Mexico / Mexico City	Human	Male	23	unknown	Oropharyngeal swab
hCoV-19/Mexico/CMX-INMEGEN-03-11-229/2021	EPI_ISL_1591575	29/03/21	North America / Mexico / Mexico City	Human	Female	81	unknown	Oropharyngeal swab
hCoV-19/Mexico/CMX-INMEGEN-03-11-230/2021	EPI_ISL_1591576	30/03/21	North America / Mexico / Mexico City	Human	Female	23	unknown	Oropharyngeal swab
hCoV-19/Mexico/CMX-INMEGEN-03-11-231/2021	EPI_ISL_1591577	24/03/21	North America / Mexico / Mexico City	Human	Female	73	unknown	Oropharyngeal swab
hCoV-19/Mexico/CMX-INMEGEN-03-11-233/2021	EPI_ISL_1591579	24/03/21	North America / Mexico / Mexico City	Human	Male	17	unknown	Oropharyngeal swab
hCoV-19/Mexico/CMX-INMEGEN-03-11-234/2021	EPI_ISL_1591580	25/03/21	North America / Mexico / Mexico City	Human	Female	60	unknown	Oropharyngeal swab
hCoV-19/Mexico/CMX-INMEGEN-03-11-235/2021	EPI_ISL_1591581	26/03/21	North America / Mexico / Mexico City	Human	Female	49	unknown	Oropharyngeal swab
hCoV-19/Mexico/CMX-INMEGEN-03-11-237/2021	EPI_ISL_1591582	29/03/21	North America / Mexico / Mexico City	Human	Male	23	unknown	Oropharyngeal swab
hCoV-19/Mexico/CMX-INMEGEN-03-11-238/2021	EPI_ISL_1591583	30/03/21	North America / Mexico / Mexico City	Human	Female	27	unknown	Oropharyngeal swab
hCoV-19/Mexico/CMX-INMEGEN-03-11-239/2021	EPI_ISL_1591584	24/03/21	North America / Mexico / Mexico City	Human	Female	71	unknown	Oropharyngeal swab
hCoV-19/Mexico/CMX-INMEGEN-03-11-240/2021	EPI_ISL_1591585	25/03/21	North America / Mexico / Mexico City	Human	Male	38	unknown	Oropharyngeal swab
hCoV-19/Mexico/CMX-INMEGEN-03-11-241/2021	EPI_ISL_1591586	24/03/21	North America / Mexico / Mexico City	Human	Male	33	unknown	Oropharyngeal swab
hCoV-19/Mexico/CMX-INMEGEN-03-11-242/2021	EPI_ISL_1591587	25/03/21	North America / Mexico / Mexico City	Human	Female	57	unknown	Oropharyngeal swab
hCoV-19/Mexico/CMX-INMEGEN-03-11-243/2021	EPI_ISL_1591588	26/03/21	North America / Mexico / Mexico City	Human	Male	44	unknown	Oropharyngeal swab
hCoV-19/Mexico/CMX-INMEGEN-03-11-244/2021	EPI_ISL_1591589	29/03/21	North America / Mexico / Mexico City	Human	Female	41	unknown	Oropharyngeal swab
hCoV-19/Mexico/CMX-INMEGEN-03-11-245/2021	EPI_ISL_1591590	29/03/21	North America / Mexico / Mexico City	Human	Female	52	unknown	Oropharyngeal swab
hCoV-19/Mexico/CMX-INMEGEN-03-11-246/2021	EPI_ISL_1591591	30/03/21	North America / Mexico / Mexico City	Human	Female	59	unknown	Oropharyngeal swab
hCoV-19/Mexico/CMX-INMEGEN-03-11-247/2021	EPI_ISL_1591592	24/03/21	North America / Mexico / Mexico City	Human	Male	42	unknown	Oropharyngeal swab
hCoV-19/Mexico/CMX-INMEGEN-03-11-248/2021	EPI_ISL_1591593	25/03/21	North America / Mexico / Mexico City	Human	Male	80	unknown	Oropharyngeal swab
hCoV-19/Mexico/CMX-INMEGEN-03-11-249/2021	EPI_ISL_1591594	24/03/21	North America / Mexico / Mexico City	Human	Male	30	unknown	Oropharyngeal swab
hCoV-19/Mexico/CMX-INMEGEN-03-11-250/2021	EPI_ISL_1591595	25/03/21	North America / Mexico / Mexico City	Human	Female	68	unknown	Oropharyngeal swab
hCoV-19/Mexico/CMX-INMEGEN-03-11-251/2021	EPI_ISL_1591596	26/03/21	North America / Mexico / Mexico City	Human	Female	31	unknown	Oropharyngeal swab
hCoV-19/Mexico/CMX-INMEGEN-03-11-252/2021	EPI_ISL_1591597	29/03/21	North America / Mexico / Mexico City	Human	Female	45	unknown	Oropharyngeal swab
hCoV-19/Mexico/CMX-INMEGEN-03-11-253/2021	EPI_ISL_1591598	29/03/21	North America / Mexico / Mexico City	Human	Female	21	unknown	Oropharyngeal swab
hCoV-19/Mexico/CMX-INMEGEN-03-11-254/2021	EPI_ISL_1591599	30/03/21	North America / Mexico / Mexico City	Human	Female	48	unknown	Oropharyngeal swab
hCoV-19/Mexico/CMX-INMEGEN-03-11-255/2021	EPI_ISL_1591600	24/03/21	North America / Mexico / Mexico City	Human	Female	52	unknown	Oropharyngeal swab
hCoV-19/Mexico/CMX-INMEGEN-03-11-257/2021	EPI_ISL_1591601	24/03/21	North America / Mexico / Mexico City	Human	Female	61	unknown	Oropharyngeal swab
hCoV-19/Mexico/CMX-INMEGEN-03-11-258/2021	EPI_ISL_1591602	25/03/21	North America / Mexico / Mexico City	Human	Male	51	unknown	Oropharyngeal swab
hCoV-19/Mexico/CMX-INMEGEN-03-11-259/2021	EPI_ISL_1591603	26/03/21	North America / Mexico / Mexico City	Human	Female	73	unknown	Oropharyngeal swab
hCoV-19/Mexico/CMX-INMEGEN-03-11-260/2021	EPI_ISL_1591604	29/03/21	North America / Mexico / Mexico City	Human	Male	61	unknown	Oropharyngeal swab
hCoV-19/Mexico/CMX-INMEGEN-03-11-261/2021	EPI_ISL_1591605	29/03/21	North America / Mexico / Mexico City	Human	Male	79	unknown	Oropharyngeal swab
hCoV-19/Mexico/CMX-INMEGEN-03-11-262/2021	EPI_ISL_1591606	30/03/21	North America / Mexico / Mexico City	Human	Male	43	unknown	Oropharyngeal swab
hCoV-19/Mexico/CMX-INMEGEN-03-11-263/2021	EPI_ISL_1591607	24/03/21	North America / Mexico / Mexico City	Human	Female	50	unknown	Oropharyngeal swab
hCoV-19/Mexico/CMX-INMEGEN-03-11-264/2021	EPI_ISL_1591608	25/03/21	North America / Mexico / Mexico City	Human	Male	62	unknown	Oropharyngeal swab
hCoV-19/Mexico/CMX-INMEGEN-03-11-265/2021	EPI_ISL_1591609	24/03/21	North America / Mexico / Mexico City	Human	Female	20	unknown	Oropharyngeal swab
hCoV-19/Mexico/CMX-INMEGEN-03-11-266/2021	EPI_ISL_1591610	01/04/21	North America / Mexico / Mexico City	Human	Female	83	unknown	Oropharyngeal swab

hCoV-19/Mexico/CMX-INMEGEN-03-11-268/2021	EPI_ISL_1591612	29/03/21	North America / Mexico / Mexico City	Human	Male	24	unknown	Oropharyngeal swab
hCoV-19/Mexico/CMX-INMEGEN-03-11-269/2021	EPI_ISL_1591613	29/03/21	North America / Mexico / Mexico City	Human	Male	46	unknown	Oropharyngeal swab
hCoV-19/Mexico/CMX-INMEGEN-03-11-270/2021	EPI_ISL_1591614	30/03/21	North America / Mexico / Mexico City	Human	Female	51	unknown	Oropharyngeal swab
hCoV-19/Mexico/CMX-INMEGEN-03-11-271/2021	EPI_ISL_1591615	24/03/21	North America / Mexico / Mexico City	Human	Male	54	unknown	Oropharyngeal swab
hCoV-19/Mexico/CMX-INMEGEN-03-11-273/2021	EPI_ISL_1591617	24/03/21	North America / Mexico / Mexico City	Human	Male	49	unknown	Oropharyngeal swab
hCoV-19/Mexico/CMX-INMEGEN-03-11-274/2021	EPI_ISL_1591618	25/03/21	North America / Mexico / Mexico City	Human	Female	51	unknown	Oropharyngeal swab
hCoV-19/Mexico/CMX-INMEGEN-03-11-275/2021	EPI_ISL_1591619	26/03/21	North America / Mexico / Mexico City	Human	Female	54	unknown	Oropharyngeal swab
hCoV-19/Mexico/CMX-INMEGEN-03-11-276/2021	EPI_ISL_1591620	29/03/21	North America / Mexico / Mexico City	Human	Male	58	unknown	Oropharyngeal swab
hCoV-19/Mexico/CMX-INMEGEN-03-11-277/2021	EPI_ISL_1591621	29/03/21	North America / Mexico / Mexico City	Human	Female	61	unknown	Oropharyngeal swab
hCoV-19/Mexico/CMX-INMEGEN-03-11-278/2021	EPI_ISL_1591622	30/03/21	North America / Mexico / Mexico City	Human	Male	17	unknown	Oropharyngeal swab
hCoV-19/Mexico/CMX-INMEGEN-03-11-279/2021	EPI_ISL_1591623	24/03/21	North America / Mexico / Mexico City	Human	Female	72	unknown	Oropharyngeal swab
hCoV-19/Mexico/CMX-INMEGEN-03-11-280/2021	EPI_ISL_1591624	25/03/21	North America / Mexico / Mexico City	Human	Male	31	unknown	Oropharyngeal swab
hCoV-19/Mexico/CMX-INMEGEN-03-11-282/2021	EPI_ISL_1591626	25/03/21	North America / Mexico / Mexico City	Human	Female	61	unknown	Oropharyngeal swab
hCoV-19/Mexico/CMX-INMEGEN-03-11-284/2021	EPI_ISL_1591627	29/03/21	North America / Mexico / Mexico City	Human	Male	73	unknown	Oropharyngeal swab
hCoV-19/Mexico/CMX-INMEGEN-03-11-285/2021	EPI_ISL_1591628	29/03/21	North America / Mexico / Mexico City	Human	Male	51	unknown	Oropharyngeal swab
hCoV-19/Mexico/CMX-INMEGEN-03-11-289/2021	EPI_ISL_1591630	01/04/21	North America / Mexico / Mexico City	Human	Female	62	unknown	Oropharyngeal swab
hCoV-19/Mexico/CMX-INMEGEN-03-11-290/2021	EPI_ISL_1591631	01/04/21	North America / Mexico / Mexico City	Human	Male	49	unknown	Oropharyngeal swab
hCoV-19/Mexico/CMX-INMEGEN-03-11-291/2021	EPI_ISL_1591632	02/04/21	North America / Mexico / Mexico City	Human	Female	74	unknown	Oropharyngeal swab
hCoV-19/Mexico/CMX-INMEGEN-03-11-293/2021	EPI_ISL_1591634	26/03/21	North America / Mexico / Mexico City	Human	Male	55	unknown	Oropharyngeal swab
hCoV-19/Mexico/CMX-INMEGEN-03-11-294/2021	EPI_ISL_1591635	29/03/21	North America / Mexico / Mexico City	Human	Male	50	unknown	Oropharyngeal swab
hCoV-19/Mexico/CMX-INMEGEN-03-11-295/2021	EPI_ISL_1591636	29/03/21	North America / Mexico / Mexico City	Human	Female	90	unknown	Oropharyngeal swab
hCoV-19/Mexico/CMX-INMEGEN-03-11-296/2021	EPI_ISL_1591637	31/03/21	North America / Mexico / Mexico City	Human	Male	45	unknown	Oropharyngeal swab
hCoV-19/Mexico/CMX-INMEGEN-03-11-297/2021	EPI_ISL_1591638	01/04/21	North America / Mexico / Mexico City	Human	Male	49	unknown	Oropharyngeal swab
hCoV-19/Mexico/CMX-INMEGEN-03-11-298/2021	EPI_ISL_1591639	01/04/21	North America / Mexico / Mexico City	Human	Female	73	unknown	Oropharyngeal swab
hCoV-19/Mexico/CMX-INMEGEN-03-11-299/2021	EPI_ISL_1591640	02/04/21	North America / Mexico / Mexico City	Human	Male	80	unknown	Oropharyngeal swab
hCoV-19/Mexico/CMX-INMEGEN-03-11-300/2021	EPI_ISL_1591641	26/03/21	North America / Mexico / Mexico City	Human	Male	58	unknown	Oropharyngeal swab
hCoV-19/Mexico/CMX-INMEGEN-03-11-301/2021	EPI_ISL_1591642	26/03/21	North America / Mexico / Mexico City	Human	Male	34	unknown	Oropharyngeal swab
hCoV-19/Mexico/CMX-INMEGEN-03-11-302/2021	EPI_ISL_1591643	29/03/21	North America / Mexico / Mexico City	Human	Male	32	unknown	Oropharyngeal swab
hCoV-19/Mexico/CMX-INMEGEN-03-11-303/2021	EPI_ISL_1591644	29/03/21	North America / Mexico / Mexico City	Human	Female	54	unknown	Oropharyngeal swab
hCoV-19/Mexico/CMX-INMEGEN-03-11-304/2021	EPI_ISL_1591645	31/03/21	North America / Mexico / Mexico City	Human	Female	68	unknown	Oropharyngeal swab
hCoV-19/Mexico/CMX-INMEGEN-03-11-305/2021	EPI_ISL_1591646	31/03/21	North America / Mexico / Mexico City	Human	Male	35	unknown	Oropharyngeal swab
hCoV-19/Mexico/CMX-INMEGEN-03-11-306/2021	EPI_ISL_1591647	01/04/21	North America / Mexico / Mexico City	Human	Male	77	unknown	Oropharyngeal swab
hCoV-19/Mexico/CMX-INMEGEN-03-11-307/2021	EPI_ISL_1591648	02/04/21	North America / Mexico / Mexico City	Human	Male	51	unknown	Oropharyngeal swab
hCoV-19/Mexico/CMX-INMEGEN-03-11-308/2021	EPI_ISL_1591649	26/03/21	North America / Mexico / Mexico City	Human	Male	59	unknown	Oropharyngeal swab
hCoV-19/Mexico/CMX-INMEGEN-03-11-309/2021	EPI_ISL_1591650	26/03/21	North America / Mexico / Mexico City	Human	Female	50	unknown	Oropharyngeal swab
hCoV-19/Mexico/CMX-INMEGEN-03-11-311/2021	EPI_ISL_1591652	29/03/21	North America / Mexico / Mexico City	Human	Male	58	unknown	Oropharyngeal swab
hCoV-19/Mexico/CMX-INMEGEN-03-11-312/2021	EPI_ISL_1591653	31/03/21	North America / Mexico / Mexico City	Human	Female	69	unknown	Oropharyngeal swab
hCoV-19/Mexico/CMX-INMEGEN-03-11-313/2021	EPI_ISL_1591654	31/03/21	North America / Mexico / Mexico City	Human	Male	25	unknown	Oropharyngeal swab
hCoV-19/Mexico/CMX-INMEGEN-03-11-314/2021	EPI_ISL_1591655	01/04/21	North America / Mexico / Mexico City	Human	Female	52	unknown	Oropharyngeal swab
hCoV-19/Mexico/CMX-INMEGEN-03-11-315/2021	EPI_ISL_1591656	02/04/21	North America / Mexico / Mexico City	Human	Female	42	unknown	Oropharyngeal swab
hCoV-19/Mexico/CMX-INMEGEN-03-11-316/2021	EPI_ISL_1591657	26/03/21	North America / Mexico / Mexico City	Human	Male	62	unknown	Oropharyngeal swab
hCoV-19/Mexico/CMX-INMEGEN-03-11-317/2021	EPI_ISL_1591658	01/04/21	North America / Mexico / Mexico City	Human	Female	46	unknown	Oropharyngeal swab
hCoV-19/Mexico/CMX-INMEGEN-03-11-318/2021	EPI_ISL_1591659	29/03/21	North America / Mexico / Mexico City	Human	Male	56	unknown	Oropharyngeal swab
hCoV-19/Mexico/CMX-INMEGEN-03-11-319/2021	EPI_ISL_1591660	29/03/21	North America / Mexico / Mexico City	Human	Male	38	unknown	Oropharyngeal swab

hCoV-19/Mexico/CMX-INMEGEN-03-11-320/2021	ΞPI_ISL_1591661	31/03/21	North America / Mexico / Mexico City	Human	Female	57	unknown		Oropharyngeal swab
hCoV-19/Mexico/CMX-INMEGEN-03-11-321/2021	ΞPI_ISL_1591662	31/03/21	North America / Mexico / Mexico City	Human	Male	49	unknown		Oropharyngeal swab
hCoV-19/Mexico/CMX-INMEGEN-03-11-322/2021	ΞPI_ISL_1591663	01/04/21	North America / Mexico / Mexico City	Human	Female	56	unknown		Oropharyngeal swab
hCoV-19/Mexico/CMX-INMEGEN-03-11-323/2021	ΞPI_ISL_1591664	02/04/21	North America / Mexico / Mexico City	Human	Female	24	unknown		Oropharyngeal swab
hCoV-19/Mexico/CMX-INMEGEN-03-11-324/2021	ΞPI_ISL_1591665	26/03/21	North America / Mexico / Mexico City	Human	Male	39	unknown		Oropharyngeal swab
hCoV-19/Mexico/CMX-INMEGEN-03-11-325/2021	ΞPI_ISL_1591666	29/03/21	North America / Mexico / Mexico City	Human	Female	63	unknown		Oropharyngeal swab
hCoV-19/Mexico/CMX-INMEGEN-03-11-326/2021	ΞPI_ISL_1591667	29/03/21	North America / Mexico / Mexico City	Human	Female	56	unknown		Oropharyngeal swab
hCoV-19/Mexico/CMX-INMEGEN-03-11-328/2021	ΞPI_ISL_1591668	31/03/21	North America / Mexico / Mexico City	Human	Male	49	unknown		Oropharyngeal swab
hCoV-19/Mexico/CMX-INMEGEN-03-11-329/2021	ΞPI_ISL_1591669	31/03/21	North America / Mexico / Mexico City	Human	Female	76	unknown		Oropharyngeal swab
hCoV-19/Mexico/CMX-INMEGEN-03-11-330/2021	ΞPI_ISL_1591670	01/04/21	North America / Mexico / Mexico City	Human	Female	35	unknown		Oropharyngeal swab
hCoV-19/Mexico/CMX-INMEGEN-03-11-331/2021	ΞPI_ISL_1591671	25/03/21	North America / Mexico / Mexico City	Human	Male	34	unknown		Oropharyngeal swab
hCoV-19/Mexico/CMX-INMEGEN-03-11-332/2021	ΞPI_ISL_1591672	26/03/21	North America / Mexico / Mexico City	Human	Male	83	unknown		Oropharyngeal swab
hCoV-19/Mexico/CMX-INMEGEN-03-11-333/2021	ΞPI_ISL_1591673	29/03/21	North America / Mexico / Mexico City	Human	Male	24	unknown		Oropharyngeal swab
hCoV-19/Mexico/CMX-INMEGEN-03-11-334/2021	ΞPI_ISL_1591674	29/03/21	North America / Mexico / Mexico City	Human	Male	23	unknown		Oropharyngeal swab
hCoV-19/Mexico/CMX-INMEGEN-03-11-335/2021	ΞPI_ISL_1591675	30/03/21	North America / Mexico / Mexico City	Human	Female	56	unknown		Oropharyngeal swab
hCoV-19/Mexico/CMX-INMEGEN-03-11-336/2021	ΞPI_ISL_1591676	31/03/21	North America / Mexico / Mexico City	Human	Female	63	unknown		Oropharyngeal swab
hCoV-19/Mexico/CMX-INMEGEN-03-11-337/2021	ΞPI_ISL_1591677	31/03/21	North America / Mexico / Mexico City	Human	Male	55	unknown		Oropharyngeal swab
hCoV-19/Mexico/CMX-INMEGEN-03-11-340/2021	ΞPI_ISL_1591679	26/03/21	North America / Mexico / Mexico City	Human	Male	36	unknown		Oropharyngeal swab
hCoV-19/Mexico/CMX-INMEGEN-03-11-341/2021	ΞPI_ISL_1591680	29/03/21	North America / Mexico / Mexico City	Human	Male	58	unknown		Oropharyngeal swab
hCoV-19/Mexico/CMX-INMEGEN-03-11-342/2021	ΞPI_ISL_1591681	29/03/21	North America / Mexico / Mexico City	Human	Female	27	unknown		Oropharyngeal swab
hCoV-19/Mexico/CMX-INMEGEN-03-11-343/2021	ΞPI_ISL_1591682	30/03/21	North America / Mexico / Mexico City	Human	Male	51	unknown		Oropharyngeal swab
hCoV-19/Mexico/CMX-INMEGEN-03-11-344/2021	ΞPI_ISL_1591683	31/03/21	North America / Mexico / Mexico City	Human	Female	68	unknown		Oropharyngeal swab
hCoV-19/Mexico/CMX-INMEGEN-03-11-345/2021	ΞPI_ISL_1591684	31/03/21	North America / Mexico / Mexico City	Human	Female	31	unknown		Oropharyngeal swab
hCoV-19/Mexico/CMX-INMEGEN-03-11-346/2021	ΞPI_ISL_1591685	01/04/21	North America / Mexico / Mexico City	Human	Male	25	unknown		Oropharyngeal swab
hCoV-19/Mexico/CMX-INMEGEN-03-11-347/2021	ΞPI_ISL_1591686	25/03/21	North America / Mexico / Mexico City	Human	Male	47	unknown		Oropharyngeal swab
hCoV-19/Mexico/CMX-INMEGEN-03-11-349/2021	ΞPI_ISL_1591687	29/03/21	North America / Mexico / Mexico City	Human	Female	72	unknown		Oropharyngeal swab
hCoV-19/Mexico/CMX-INMEGEN-03-11-350/2021	ΞPI_ISL_1591688	29/03/21	North America / Mexico / Mexico City	Human	Male	59	unknown		Oropharyngeal swab
hCoV-19/Mexico/CMX-INMEGEN-03-11-351/2021	ΞPI_ISL_1591689	30/03/21	North America / Mexico / Mexico City	Human	Male	51	unknown		Oropharyngeal swab
hCoV-19/Mexico/CMX-INMEGEN-03-11-352/2021	ΞPI_ISL_1591690	31/03/21	North America / Mexico / Mexico City	Human	Male	62	unknown		Oropharyngeal swab
hCoV-19/Mexico/CMX-INMEGEN-03-11-353/2021	ΞPI_ISL_1591691	31/03/21	North America / Mexico / Mexico City	Human	Male	24	unknown		Oropharyngeal swab
hCoV-19/Mexico/CMX-INMEGEN-03-11-355/2021	ΞPI_ISL_1591693	25/03/21	North America / Mexico / Mexico City	Human	Male	47	unknown		Oropharyngeal swab
hCoV-19/Mexico/CMX-INMEGEN-03-11-357/2021	ΞPI_ISL_1591694	29/03/21	North America / Mexico / Mexico City	Human	Male	76	unknown		Oropharyngeal swab
hCoV-19/Mexico/CMX-INMEGEN-03-11-358/2021	ΞPI_ISL_1591695	29/03/21	North America / Mexico / Mexico City	Human	Female	32	unknown		Oropharyngeal swab
hCoV-19/Mexico/CMX-INMEGEN-03-11-359/2021	ΞPI_ISL_1591696	30/03/21	North America / Mexico / Mexico City	Human	Male	57	unknown		Oropharyngeal swab
hCoV-19/Mexico/CMX-INMEGEN-03-11-360/2021	ΞPI_ISL_1591697	01/04/21	North America / Mexico / Mexico City	Human	Male	54	unknown		Oropharyngeal swab
hCoV-19/Mexico/CMX-INMEGEN-03-11-361/2021	ΞPI_ISL_1591698	01/04/21	North America / Mexico / Mexico City	Human	Male	49	unknown		Oropharyngeal swab
hCoV-19/Mexico/CMX-INMEGEN-03-11-362/2021	ΞPI_ISL_1591699	02/04/21	North America / Mexico / Mexico City	Human	Female	20	unknown		Oropharyngeal swab
hCoV-19/Mexico/CMX-INMEGEN-03-11-363/2021	ΞPI_ISL_1591700	25/03/21	North America / Mexico / Mexico City	Human	Female	57	unknown		Oropharyngeal swab
hCoV-19/Mexico/CMX-INMEGEN-03-11-365/2021	ΞPI_ISL_1591702	29/03/21	North America / Mexico / Mexico City	Human	Female	48	unknown		Oropharyngeal swab
hCoV-19/Mexico/CMX-INMEGEN-03-11-366/2021	ΞPI_ISL_1591703	29/03/21	North America / Mexico / Mexico City	Human	Female	72	unknown		Oropharyngeal swab
hCoV-19/Mexico/CMX-INMEGEN-03-11-367/2021	ΞPI_ISL_1591704	01/04/21	North America / Mexico / Mexico City	Human	Male	48	unknown		Oropharyngeal swab
hCoV-19/Mexico/CMX-INMEGEN-03-11-368/2021	ΞPI_ISL_1591705	03/04/21	North America / Mexico / Mexico City	Human	Male	38	unknown		Oropharyngeal swab
hCoV-19/Mexico/CMX-INMEGEN-03-11-369/2021	ΞPI_ISL_1591706	01/04/21	North America / Mexico / Mexico City	Human	Female	62	unknown		Oropharyngeal swab
hCoV-19/Mexico/CMX-INMEGEN-03-11-370/2021	ΞPI_ISL_1591707	02/04/21	North America / Mexico / Mexico City	Human	Female	46	unknown		Oropharyngeal swab

hCoV-19/Mexico/CMX-INMEGEN-03-11-373/2021	EPI_ISL_1591708	29/03/21	North America / Mexico / Mexico City	Human	Female	46	unknown	Oropharyngeal swab
hCoV-19/Mexico/CMX-INMEGEN-03-11-374/2021	EPI_ISL_1591709	29/03/21	North America / Mexico / Mexico City	Human	Female	67	unknown	Oropharyngeal swab
hCoV-19/Mexico/CMX-INMEGEN-03-11-375/2021	EPI_ISL_1591710	31/03/21	North America / Mexico / Mexico City	Human	Male	24	unknown	Oropharyngeal swab
hCoV-19/Mexico/CMX-INMEGEN-03-11-376/2021	EPI_ISL_1591711	02/04/21	North America / Mexico / Mexico City	Human	Female	55	unknown	Oropharyngeal swab
hCoV-19/Mexico/CMX-INMEGEN-03-11-377/2021	EPI_ISL_1591712	01/04/21	North America / Mexico / Mexico City	Human	Male	83	unknown	Oropharyngeal swab
hCoV-19/Mexico/CMX-INMEGEN-03-11-378/2021	EPI_ISL_1591713	02/04/21	North America / Mexico / Mexico City	Human	Male	54	unknown	Oropharyngeal swab
hCoV-19/Mexico/CMX-INMEGEN-03-11-380/2021	EPI_ISL_1591715	26/03/21	North America / Mexico / Mexico City	Human	Male	65	unknown	Oropharyngeal swab
hCoV-19/Mexico/CMX-INMEGEN-03-11-381/2021	EPI_ISL_1591716	29/03/21	North America / Mexico / Mexico City	Human	Female	51	unknown	Oropharyngeal swab
hCoV-19/Mexico/CMX-INMEGEN-03-11-382/2021	EPI_ISL_1591717	29/03/21	North America / Mexico / Mexico City	Human	Male	48	unknown	Oropharyngeal swab
hCoV-19/Mexico/CMX-INMEGEN-03-10-01/2021	EPI_ISL_1594356	22/03/21	North America / Mexico / Mexico City	Human	Male	46	unknown	Oropharyngeal swab
hCoV-19/Mexico/CMX-INMEGEN-03-10-02/2021	EPI_ISL_1594357	22/03/21	North America / Mexico / Mexico City	Human	Female	55	unknown	Oropharyngeal swab
hCoV-19/Mexico/CMX-INMEGEN-03-10-10/2021	EPI_ISL_1594358	22/03/21	North America / Mexico / Mexico City	Human	Male	50	unknown	Oropharyngeal swab
hCoV-19/Mexico/CMX-INMEGEN-03-10-17/2021	EPI_ISL_1594359	22/03/21	North America / Mexico / Mexico City	Human	Female	20	unknown	Oropharyngeal swab
hCoV-19/Mexico/CMX-INMEGEN-03-10-18/2021	EPI_ISL_1594360	22/03/21	North America / Mexico / Mexico City	Human	Male	53	unknown	Oropharyngeal swab
hCoV-19/Mexico/CMX-INMEGEN-03-10-25/2021	EPI_ISL_1594361	22/03/21	North America / Mexico / Mexico City	Human	Female	54	unknown	Oropharyngeal swab
hCoV-19/Mexico/CMX-INMEGEN-03-10-26/2021	EPI_ISL_1594362	22/03/21	North America / Mexico / Mexico City	Human	Female	78	unknown	Oropharyngeal swab
hCoV-19/Mexico/CMX-INMEGEN-03-10-33/2021	EPI_ISL_1594363	22/03/21	North America / Mexico / Mexico City	Human	Male	56	unknown	Oropharyngeal swab
hCoV-19/Mexico/CMX-INMEGEN-03-10-34/2021	EPI_ISL_1594364	23/03/21	North America / Mexico / Mexico City	Human	Male	58	unknown	Oropharyngeal swab
hCoV-19/Mexico/CMX-INMEGEN-03-10-41/2021	EPI_ISL_1594365	22/03/21	North America / Mexico / Mexico City	Human	Male	46	unknown	Oropharyngeal swab
hCoV-19/Mexico/CMX-INMEGEN-03-10-42/2021	EPI_ISL_1594366	23/03/21	North America / Mexico / Mexico City	Human	Female	78	unknown	Oropharyngeal swab
hCoV-19/Mexico/CMX-INMEGEN-03-10-49/2021	EPI_ISL_1594367	22/03/21	North America / Mexico / Mexico City	Human	Male	59	unknown	Oropharyngeal swab
hCoV-19/Mexico/CMX-INMEGEN-03-10-50/2021	EPI_ISL_1594368	23/03/21	North America / Mexico / Mexico City	Human	Female	51	unknown	Oropharyngeal swab
hCoV-19/Mexico/CMX-INMEGEN-03-10-57/2021	EPI_ISL_1594369	22/03/21	North America / Mexico / Mexico City	Human	Female	56	unknown	Oropharyngeal swab
hCoV-19/Mexico/CMX-INMEGEN-03-10-58/2021	EPI_ISL_1594370	23/03/21	North America / Mexico / Mexico City	Human	Female	39	unknown	Oropharyngeal swab
hCoV-19/Mexico/CMX-INMEGEN-03-10-65/2021	EPI_ISL_1594371	22/03/21	North America / Mexico / Mexico City	Human	Female	45	unknown	Oropharyngeal swab
hCoV-19/Mexico/CMX-INMEGEN-03-10-66/2021	EPI_ISL_1594372	23/03/21	North America / Mexico / Mexico City	Human	Female	55	unknown	Oropharyngeal swab
hCoV-19/Mexico/CMX-INMEGEN-03-10-73/2021	EPI_ISL_1594373	22/03/21	North America / Mexico / Mexico City	Human	Female	33	unknown	Oropharyngeal swab
hCoV-19/Mexico/CMX-INMEGEN-03-10-74/2021	EPI_ISL_1594374	23/03/21	North America / Mexico / Mexico City	Human	Female	74	unknown	Oropharyngeal swab
hCoV-19/Mexico/CMX-INMEGEN-03-10-81/2021	EPI_ISL_1594375	22/03/21	North America / Mexico / Mexico City	Human	Female	45	unknown	Oropharyngeal swab
hCoV-19/Mexico/CMX-INMEGEN-03-10-82/2021	EPI_ISL_1594376	23/03/21	North America / Mexico / Mexico City	Human	Female	47	unknown	Oropharyngeal swab
hCoV-19/Mexico/CMX-INMEGEN-03-10-89/2021	EPI_ISL_1594377	22/03/21	North America / Mexico / Mexico City	Human	Female	55	unknown	Oropharyngeal swab
hCoV-19/Mexico/CMX-INMEGEN-03-10-90/2021	EPI_ISL_1594378	23/03/21	North America / Mexico / Mexico City	Human	Male	76	unknown	Oropharyngeal swab
hCoV-19/Mexico/CHH_INER_IMSS_00379/2021	EPI_ISL_1595596	03/03/21	North America / Mexico / Chihuahua	Human	Male	52	Ambulatory	Nasopharyngeal and oropharyngeal swab
hCoV-19/Mexico/COL_INER_IMSS_00532/2021	EPI_ISL_1595604	13/03/21	North America / Mexico / Colima	Human	Female	73	Ambulatory	Nasopharyngeal and oropharyngeal swab
hCoV-19/Mexico/CMX-INMEGEN-03-11-208/2021	EPI_ISL_1615069	24/03/21	North America / Mexico / Mexico City	Human	Male	52	unknown	
hCoV-19/Mexico/BCS-InDRE_F12237_S1204/2021	EPI_ISL_1626811	20/03/21	North America / Mexico / Baja California Sur	Human	Female	58	Released	Oropharyngeal swab
hCoV-19/Mexico/BCS-InDRE_F12241_S1206/2021	EPI_ISL_1626813	20/03/21	North America / Mexico / Baja California Sur	Human	Female	20	Released	Oropharyngeal swab
hCoV-19/Mexico/SIN_CIAD_ID1978/2021	EPI_ISL_1627081	19/03/21	North America / Mexico / Sinaloa / Mazatlan	Human	Male	70	unknown	Oropharyngeal swab
hCoV-19/Mexico/SIN_CIAD_S6374/2021	EPI_ISL_1627082	23/03/21	North America / Mexico / Sinaloa / Mazatlan	Human	Male	45	unknown	Oropharyngeal swab
hCoV-19/Mexico/SIN_CIAD_S6436/2021	EPI_ISL_1627085	30/03/21	North America / Mexico / Sinaloa / Mazatlan	Human	Male	42	unknown	Oropharyngeal swab
hCoV-19/Mexico/CMX-INMEGEN-04-04-1/2021	EPI_ISL_1628520	18/02/21	North America / Mexico / Mexico City	Human	Male	66	unknown	Oropharyngeal swab
hCoV-19/Mexico/CMX-INMEGEN-04-04-4/2021	EPI_ISL_1628523	03/03/21	North America / Mexico / Mexico City	Human	Female	44	unknown	Oropharyngeal swab
hCoV-19/Mexico/CMX-INMEGEN-04-04-5/2021	EPI_ISL_1628524	05/04/21	North America / Mexico / Mexico City	Human	Female	53	unknown	Oropharyngeal swab
hCoV-19/Mexico/CMX-INMEGEN-04-04-6/2021	EPI_ISL_1628525	05/04/21	North America / Mexico / Mexico City	Human	Male	54	unknown	Oropharyngeal swab

hCoV-19/Mexico/CMX-INMEGEN-04-04-7/2021	EPI_ISL_1628526	06/04/21	North America / Mexico / Mexico City	Human	Female	69	unknown	Oropharyngeal swab
hCoV-19/Mexico/CMX-INMEGEN-04-04-8/2021	EPI_ISL_1628527	07/04/21	North America / Mexico / Mexico City	Human	Female	73	unknown	Oropharyngeal swab
hCoV-19/Mexico/CMX-INMEGEN-04-04-9/2021	EPI_ISL_1628528	24/02/21	North America / Mexico / Mexico City	Human	Male	38	unknown	Oropharyngeal swab
hCoV-19/Mexico/CMX-INMEGEN-04-04-11/2021	EPI_ISL_1628529	03/03/21	North America / Mexico / Mexico City	Human	Female	21	unknown	Oropharyngeal swab
hCoV-19/Mexico/CMX-INMEGEN-04-04-12/2021	EPI_ISL_1628530	03/03/21	North America / Mexico / Mexico City	Human	Female	23	unknown	Oropharyngeal swab
hCoV-19/Mexico/CMX-INMEGEN-04-04-13/2021	EPI_ISL_1628531	05/04/21	North America / Mexico / Mexico City	Human	Male	50	unknown	Oropharyngeal swab
hCoV-19/Mexico/CMX-INMEGEN-04-04-14/2021	EPI_ISL_1628532	05/04/21	North America / Mexico / Mexico City	Human	Male	35	unknown	Oropharyngeal swab
hCoV-19/Mexico/CMX-INMEGEN-04-04-15/2021	EPI_ISL_1628533	06/04/21	North America / Mexico / Mexico City	Human	Female	65	unknown	Oropharyngeal swab
hCoV-19/Mexico/CMX-INMEGEN-04-04-18/2021	EPI_ISL_1628534	25/02/21	North America / Mexico / Mexico City	Human	Male	56	unknown	Oropharyngeal swab
hCoV-19/Mexico/CMX-INMEGEN-04-04-19/2021	EPI_ISL_1628535	03/03/21	North America / Mexico / Mexico City	Human	Female	83	unknown	Oropharyngeal swab
hCoV-19/Mexico/CMX-INMEGEN-04-04-20/2021	EPI_ISL_1628536	03/03/21	North America / Mexico / Mexico City	Human	Female	52	unknown	Oropharyngeal swab
hCoV-19/Mexico/CMX-INMEGEN-04-04-21/2021	EPI_ISL_1628537	05/04/21	North America / Mexico / Mexico City	Human	Male	36	unknown	Oropharyngeal swab
hCoV-19/Mexico/CMX-INMEGEN-04-04-23/2021	EPI_ISL_1628539	06/04/21	North America / Mexico / Mexico City	Human	Female	73	unknown	Oropharyngeal swab
hCoV-19/Mexico/CMX-INMEGEN-04-04-24/2021	EPI_ISL_1628540	07/04/21	North America / Mexico / Mexico City	Human	Female	50	unknown	Oropharyngeal swab
hCoV-19/Mexico/CMX-INMEGEN-04-04-25/2021	EPI_ISL_1628541	24/02/21	North America / Mexico / Mexico City	Human	Male	41	unknown	Oropharyngeal swab
hCoV-19/Mexico/CMX-INMEGEN-04-04-27/2021	EPI_ISL_1628542	01/03/21	North America / Mexico / Mexico City	Human	Female	12	unknown	Oropharyngeal swab
hCoV-19/Mexico/CMX-INMEGEN-04-04-28/2021	EPI_ISL_1628543	05/04/21	North America / Mexico / Mexico City	Human	Male	52	unknown	Oropharyngeal swab
hCoV-19/Mexico/CMX-INMEGEN-04-04-29/2021	EPI_ISL_1628544	01/03/21	North America / Mexico / Mexico City	Human	Female	15	unknown	Oropharyngeal swab
hCoV-19/Mexico/CMX-INMEGEN-04-04-30/2021	EPI_ISL_1628545	06/04/21	North America / Mexico / Mexico City	Human	Male	72	unknown	Oropharyngeal swab
hCoV-19/Mexico/CMX-INMEGEN-04-04-31/2021	EPI_ISL_1628546	06/04/21	North America / Mexico / Mexico City	Human	Female	35	unknown	Oropharyngeal swab
hCoV-19/Mexico/CMX-INMEGEN-04-04-33/2021	EPI_ISL_1628548	24/02/21	North America / Mexico / Mexico City	Human	Male	43	unknown	Oropharyngeal swab
hCoV-19/Mexico/CMX-INMEGEN-04-04-34/2021	EPI_ISL_1628549	25/02/21	North America / Mexico / Mexico City	Human	Male	13	unknown	Oropharyngeal swab
hCoV-19/Mexico/CMX-INMEGEN-04-04-35/2021	EPI_ISL_1628550	02/03/21	North America / Mexico / Mexico City	Human	Male	22	unknown	Oropharyngeal swab
hCoV-19/Mexico/CMX-INMEGEN-04-04-36/2021	EPI_ISL_1628551	03/03/21	North America / Mexico / Mexico City	Human	Female	47	unknown	Oropharyngeal swab
hCoV-19/Mexico/CMX-INMEGEN-04-04-37/2021	EPI_ISL_1628552	05/04/21	North America / Mexico / Mexico City	Human	Male	71	unknown	Oropharyngeal swab
hCoV-19/Mexico/CMX-INMEGEN-04-04-38/2021	EPI_ISL_1628553	06/04/21	North America / Mexico / Mexico City	Human	Female	25	unknown	Oropharyngeal swab
hCoV-19/Mexico/CMX-INMEGEN-04-04-39/2021	EPI_ISL_1628554	06/04/21	North America / Mexico / Mexico City	Human	Female	53	unknown	Oropharyngeal swab
hCoV-19/Mexico/CMX-INMEGEN-04-04-41/2021	EPI_ISL_1628555	25/02/21	North America / Mexico / Mexico City	Human	Male	23	unknown	Oropharyngeal swab
hCoV-19/Mexico/CMX-INMEGEN-04-04-42/2021	EPI_ISL_1628556	26/02/21	North America / Mexico / Mexico City	Human	Female	56	unknown	Oropharyngeal swab
hCoV-19/Mexico/CMX-INMEGEN-04-04-43/2021	EPI_ISL_1628557	01/03/21	North America / Mexico / Mexico City	Human	Female	39	unknown	Oropharyngeal swab
hCoV-19/Mexico/CMX-INMEGEN-04-04-46/2021	EPI_ISL_1628558	06/04/21	North America / Mexico / Mexico City	Human	Female	41	unknown	Oropharyngeal swab
hCoV-19/Mexico/CMX-INMEGEN-04-04-48/2021	EPI_ISL_1628559	06/04/21	North America / Mexico / Mexico City	Human	Female	66	unknown	Oropharyngeal swab
hCoV-19/Mexico/CMX-INMEGEN-04-04-49/2021	EPI_ISL_1628560	25/02/21	North America / Mexico / Mexico City	Human	Female	22	unknown	Oropharyngeal swab
hCoV-19/Mexico/CMX-INMEGEN-04-04-50/2021	EPI_ISL_1628561	26/02/21	North America / Mexico / Mexico City	Human	Female	21	unknown	Oropharyngeal swab
hCoV-19/Mexico/CMX-INMEGEN-04-04-51/2021	EPI_ISL_1628562	02/03/21	North America / Mexico / Mexico City	Human	Male	50	unknown	Oropharyngeal swab
hCoV-19/Mexico/CMX-INMEGEN-04-04-52/2021	EPI_ISL_1628563	22/02/21	North America / Mexico / Mexico City	Human	Male	22	unknown	Oropharyngeal swab
hCoV-19/Mexico/CMX-INMEGEN-04-04-53/2021	EPI_ISL_1628564	05/04/21	North America / Mexico / Mexico City	Human	Male	77	unknown	Oropharyngeal swab
hCoV-19/Mexico/CMX-INMEGEN-04-04-56/2021	EPI_ISL_1628565	07/04/21	North America / Mexico / Mexico City	Human	Female	74	unknown	Oropharyngeal swab
hCoV-19/Mexico/CMX-INMEGEN-04-04-57/2021	EPI_ISL_1628566	24/02/21	North America / Mexico / Mexico City	Human	Male	36	unknown	Oropharyngeal swab
hCoV-19/Mexico/CMX-INMEGEN-04-04-60/2021	EPI_ISL_1628568	05/04/21	North America / Mexico / Mexico City	Human	Female	58	unknown	Oropharyngeal swab
hCoV-19/Mexico/CMX-INMEGEN-04-04-61/2021	EPI_ISL_1628569	05/04/21	North America / Mexico / Mexico City	Human	Male	48	unknown	Oropharyngeal swab
hCoV-19/Mexico/CMX-INMEGEN-04-04-62/2021	EPI_ISL_1628570	06/04/21	North America / Mexico / Mexico City	Human	Female	28	unknown	Oropharyngeal swab
hCoV-19/Mexico/CMX-INMEGEN-04-04-64/2021	EPI_ISL_1628572	06/04/21	North America / Mexico / Mexico City	Human	Female	15	unknown	Oropharyngeal swab
hCoV-19/Mexico/CMX-INMEGEN-04-04-65/2021	EPI_ISL_1628573	23/02/21	North America / Mexico / Mexico City	Human	Female	38	unknown	Oropharyngeal swab

hCoV-19/Mexico/CMX-INMEGEN-04-04-67/2021	EPI_ISL_1628574	03/03/21	North America / Mexico / Mexico City	Human	Female	50	unknown	Oropharyngeal swab
hCoV-19/Mexico/CMX-INMEGEN-04-04-68/2021	EPI_ISL_1628575	05/04/21	North America / Mexico / Mexico City	Human	Male	61	unknown	Oropharyngeal swab
hCoV-19/Mexico/CMX-INMEGEN-04-04-69/2021	EPI_ISL_1628576	05/04/21	North America / Mexico / Mexico City	Human	Female	60	unknown	Oropharyngeal swab
hCoV-19/Mexico/CMX-INMEGEN-04-04-71/2021	EPI_ISL_1628577	07/04/21	North America / Mexico / Mexico City	Human	Male	55	unknown	Oropharyngeal swab
hCoV-19/Mexico/CMX-INMEGEN-04-04-72/2021	EPI_ISL_1628578	06/04/21	North America / Mexico / Mexico City	Human	Female	50	unknown	Oropharyngeal swab
hCoV-19/Mexico/CMX-INMEGEN-04-04-73/2021	EPI_ISL_1628579	25/02/21	North America / Mexico / Mexico City	Human	Male	44	unknown	Oropharyngeal swab
hCoV-19/Mexico/CMX-INMEGEN-04-04-76/2021	EPI_ISL_1628581	05/04/21	North America / Mexico / Mexico City	Human	Female	73	unknown	Oropharyngeal swab
hCoV-19/Mexico/CMX-INMEGEN-04-04-77/2021	EPI_ISL_1628582	05/04/21	North America / Mexico / Mexico City	Human	Female	61	unknown	Oropharyngeal swab
hCoV-19/Mexico/CMX-INMEGEN-04-04-79/2021	EPI_ISL_1628583	07/04/21	North America / Mexico / Mexico City	Human	Male	20	unknown	Oropharyngeal swab
hCoV-19/Mexico/CMX-INMEGEN-04-04-80/2021	EPI_ISL_1628584	07/04/21	North America / Mexico / Mexico City	Human	Male	54	unknown	Oropharyngeal swab
hCoV-19/Mexico/CMX-INMEGEN-04-04-84/2021	EPI_ISL_1628585	05/04/21	North America / Mexico / Mexico City	Human	Female	50	unknown	Oropharyngeal swab
hCoV-19/Mexico/CMX-INMEGEN-04-04-86/2021	EPI_ISL_1628587	06/04/21	North America / Mexico / Mexico City	Human	Male	57	unknown	Oropharyngeal swab
hCoV-19/Mexico/CMX-INMEGEN-04-04-88/2021	EPI_ISL_1628589	07/04/21	North America / Mexico / Mexico City	Human	Male	75	unknown	Oropharyngeal swab
hCoV-19/Mexico/CMX-INMEGEN-04-04-89/2021	EPI_ISL_1628590	25/02/21	North America / Mexico / Mexico City	Human	Male	45	unknown	Oropharyngeal swab
hCoV-19/Mexico/CMX-INMEGEN-04-04-90/2021	EPI_ISL_1628591	27/02/21	North America / Mexico / Mexico City	Human	Male	54	unknown	Oropharyngeal swab
hCoV-19/Mexico/CMX-INMEGEN-04-04-91/2021	EPI_ISL_1628592	02/03/21	North America / Mexico / Mexico City	Human	Male	57	unknown	Oropharyngeal swab
hCoV-19/Mexico/CMX-INMEGEN-04-04-92/2021	EPI_ISL_1628593	05/04/21	North America / Mexico / Mexico City	Human	Female	12	unknown	Oropharyngeal swab
hCoV-19/Mexico/CMX-INMEGEN-04-04-93/2021	EPI_ISL_1628594	05/04/21	North America / Mexico / Mexico City	Human	Female	45	unknown	Oropharyngeal swab
hCoV-19/Mexico/CMX-INMEGEN-04-04-94/2021	EPI_ISL_1628595	06/04/21	North America / Mexico / Mexico City	Human	Female	23	unknown	Oropharyngeal swab
CoV-19/Mexico/CMX-INMEGEN-InDRE-04-04-97/202	EPI_ISL_1628596	04/04/21	North America / Mexico / Mexico City	Human	Male	69	unknown	Oropharyngeal swab
CoV-19/Mexico/MOR-INMEGEN-InDRE-04-04-98/202	EPI_ISL_1628597	04/04/21	North America / Mexico / Morelos	Human	Female	64	unknown	Oropharyngeal swab
CoV-19/Mexico/MEX-INMEGEN-InDRE-04-04-101/20	EPI_ISL_1628599	31/03/21	North America / Mexico / State of Mexico	Human	Male	41	unknown	Oropharyngeal swab
CoV-19/Mexico/MEX-INMEGEN-InDRE-04-04-102/20	EPI_ISL_1628600	31/03/21	North America / Mexico / State of Mexico	Human	Female	63	unknown	Oropharyngeal swab
CoV-19/Mexico/CMX-INMEGEN-InDRE-04-04-103/20	EPI_ISL_1628601	02/04/21	North America / Mexico / Mexico City	Human	Male	40	unknown	Oropharyngeal swab
CoV-19/Mexico/CMX-INMEGEN-InDRE-04-04-104/20	EPI_ISL_1628602	31/03/21	North America / Mexico / Mexico City	Human	Male	50	unknown	Oropharyngeal swab
CoV-19/Mexico/CMX-INMEGEN-InDRE-04-04-106/20	EPI_ISL_1628604	02/04/21	North America / Mexico / Mexico City	Human	Male	29	unknown	Oropharyngeal swab
CoV-19/Mexico/MEX-INMEGEN-InDRE-04-04-107/20	EPI_ISL_1628605	02/04/21	North America / Mexico / State of Mexico	Human	Female	57	unknown	Oropharyngeal swab
CoV-19/Mexico/CMX-INMEGEN-InDRE-04-04-108/20	EPI_ISL_1628606	02/04/21	North America / Mexico / Mexico City	Human	Female	55	unknown	Oropharyngeal swab
CoV-19/Mexico/CMX-INMEGEN-InDRE-04-04-109/20	EPI_ISL_1628607	02/04/21	North America / Mexico / Mexico City	Human	Male	53	unknown	Oropharyngeal swab
CoV-19/Mexico/CMX-INMEGEN-InDRE-04-04-110/20	EPI_ISL_1628608	02/04/21	North America / Mexico / Mexico City	Human	Female	30	unknown	Oropharyngeal swab
CoV-19/Mexico/TAB-INMEGEN-InDRE-04-04-111/20	EPI_ISL_1628609	04/04/21	North America / Mexico / Tabasco	Human	Female	37	unknown	Oropharyngeal swab
CoV-19/Mexico/MEX-INMEGEN-InDRE-04-04-112/20	EPI_ISL_1628610	05/04/21	North America / Mexico / State of Mexico	Human	Female	39	unknown	Oropharyngeal swab
CoV-19/Mexico/CMX-INMEGEN-InDRE-04-04-113/20	EPI_ISL_1628611	05/04/21	North America / Mexico / Mexico City	Human	Female	95	unknown	Oropharyngeal swab
CoV-19/Mexico/CMX-INMEGEN-InDRE-04-04-114/20	EPI_ISL_1628612	05/04/21	North America / Mexico / Mexico City	Human	Female	79	unknown	Oropharyngeal swab
CoV-19/Mexico/CMX-INMEGEN-InDRE-04-04-115/20	EPI_ISL_1628613	05/04/21	North America / Mexico / Mexico City	Human	Male	63	unknown	Oropharyngeal swab
CoV-19/Mexico/CMX-INMEGEN-InDRE-04-04-116/20	EPI_ISL_1628614	05/04/21	North America / Mexico / Mexico City	Human	Male	63	unknown	Oropharyngeal swab
CoV-19/Mexico/CMX-INMEGEN-InDRE-04-04-117/20	EPI_ISL_1628615	05/04/21	North America / Mexico / Mexico City	Human	Female	39	unknown	Oropharyngeal swab
CoV-19/Mexico/CMX-INMEGEN-InDRE-04-04-118/20	EPI_ISL_1628616	05/04/21	North America / Mexico / Mexico City	Human	Female	53	unknown	Oropharyngeal swab
CoV-19/Mexico/CMX-INMEGEN-InDRE-04-04-119/20	EPI_ISL_1628617	05/04/21	North America / Mexico / Mexico City	Human	Male	51	unknown	Oropharyngeal swab
CoV-19/Mexico/CMX-INMEGEN-InDRE-04-04-120/20	EPI_ISL_1628618	05/04/21	North America / Mexico / Mexico City	Human	Female	29	unknown	Oropharyngeal swab
CoV-19/Mexico/CMX-INMEGEN-InDRE-04-04-121/20	EPI_ISL_1628619	04/04/21	North America / Mexico / Mexico City	Human	Male	40	unknown	Oropharyngeal swab
CoV-19/Mexico/CMX-INMEGEN-InDRE-04-04-122/20	EPI_ISL_1628620	03/04/21	North America / Mexico / Mexico City	Human	Female	18	unknown	Oropharyngeal swab
CoV-19/Mexico/CMX-INMEGEN-InDRE-04-04-123/20	EPI_ISL_1628621	03/04/21	North America / Mexico / Mexico City	Human	Female	38	unknown	Oropharyngeal swab
CoV-19/Mexico/CMX-INMEGEN-InDRE-04-04-124/20	EPI_ISL_1628622	05/04/21	North America / Mexico / Mexico City	Human	Male	10	unknown	Oropharyngeal swab

CoV-19/Mexico/CMX-INMEGEN-InDRE-04-04-125/20	EPI_ISL_1628623	05/04/21	North America / Mexico / Mexico City	Human	Male	11	unknown	Oropharyngeal swab
CoV-19/Mexico/CMX-INMEGEN-InDRE-04-04-127/20	EPI_ISL_1628625	05/04/21	North America / Mexico / Mexico City	Human	Male	30	unknown	Oropharyngeal swab
CoV-19/Mexico/CMX-INMEGEN-InDRE-04-04-128/20	EPI_ISL_1628626	05/04/21	North America / Mexico / Mexico City	Human	Male	47	unknown	Oropharyngeal swab
CoV-19/Mexico/CMX-INMEGEN-InDRE-04-04-129/20	EPI_ISL_1628627	05/04/21	North America / Mexico / Mexico City	Human	Female	31	unknown	Oropharyngeal swab
CoV-19/Mexico/CMX-INMEGEN-InDRE-04-04-131/20	EPI_ISL_1628628	05/04/21	North America / Mexico / Mexico City	Human	Male	40	unknown	Oropharyngeal swab
CoV-19/Mexico/CMX-INMEGEN-InDRE-04-04-132/20	EPI_ISL_1628629	05/04/21	North America / Mexico / Mexico City	Human	Female	26	unknown	Oropharyngeal swab
CoV-19/Mexico/CMX-INMEGEN-InDRE-04-04-133/20	EPI_ISL_1628630	05/04/21	North America / Mexico / Mexico City	Human	Male	51	unknown	Oropharyngeal swab
CoV-19/Mexico/CMX-INMEGEN-InDRE-04-04-134/20	EPI_ISL_1628631	05/04/21	North America / Mexico / Mexico City	Human	Female	26	unknown	Oropharyngeal swab
CoV-19/Mexico/CMX-INMEGEN-InDRE-04-04-135/20	EPI_ISL_1628632	05/04/21	North America / Mexico / Mexico City	Human	Male	19	unknown	Oropharyngeal swab
CoV-19/Mexico/CMX-INMEGEN-InDRE-04-04-136/20	EPI_ISL_1628633	05/04/21	North America / Mexico / Mexico City	Human	Male	42	unknown	Oropharyngeal swab
CoV-19/Mexico/CMX-INMEGEN-InDRE-04-04-137/20	EPI_ISL_1628634	05/04/21	North America / Mexico / Mexico City	Human	Female	64	unknown	Oropharyngeal swab
CoV-19/Mexico/CMX-INMEGEN-InDRE-04-04-138/20	EPI_ISL_1628635	05/04/21	North America / Mexico / Mexico City	Human	Female	49	unknown	Oropharyngeal swab
CoV-19/Mexico/CMX-INMEGEN-InDRE-04-04-139/20	EPI_ISL_1628636	05/04/21	North America / Mexico / Mexico City	Human	Female	13	unknown	Oropharyngeal swab
CoV-19/Mexico/CMX-INMEGEN-InDRE-04-04-140/20	EPI_ISL_1628637	05/04/21	North America / Mexico / Mexico City	Human	Female	37	unknown	Oropharyngeal swab
CoV-19/Mexico/CMX-INMEGEN-InDRE-04-04-141/20	EPI_ISL_1628638	05/04/21	North America / Mexico / Mexico City	Human	Male	7	unknown	Oropharyngeal swab
CoV-19/Mexico/CMX-INMEGEN-InDRE-04-04-142/20	EPI_ISL_1628639	02/04/21	North America / Mexico / Mexico City	Human	Female	31	unknown	Oropharyngeal swab
CoV-19/Mexico/CMX-INMEGEN-InDRE-04-04-143/20	EPI_ISL_1628640	05/04/21	North America / Mexico / Mexico City	Human	Female	62	unknown	Oropharyngeal swab
CoV-19/Mexico/CMX-INMEGEN-InDRE-04-04-144/20	EPI_ISL_1628641	05/04/21	North America / Mexico / Mexico City	Human	Male	52	unknown	Oropharyngeal swab
CoV-19/Mexico/CMX-INMEGEN-InDRE-04-04-146/20	EPI_ISL_1628643	05/04/21	North America / Mexico / Mexico City	Human	Female	23	unknown	Oropharyngeal swab
CoV-19/Mexico/CMX-INMEGEN-InDRE-04-04-147/20	EPI_ISL_1628644	05/04/21	North America / Mexico / Mexico City	Human	Male	48	unknown	Oropharyngeal swab
CoV-19/Mexico/MEX-INMEGEN-InDRE-04-04-148/20	EPI_ISL_1628645	05/04/21	North America / Mexico / State of Mexico	Human	Male	25	unknown	Oropharyngeal swab
CoV-19/Mexico/CMX-INMEGEN-InDRE-04-04-149/20	EPI_ISL_1628646	05/04/21	North America / Mexico / Mexico City	Human	Female	47	unknown	Oropharyngeal swab
CoV-19/Mexico/CMX-INMEGEN-InDRE-04-04-150/20	EPI_ISL_1628647	05/04/21	North America / Mexico / Mexico City	Human	Male	45	unknown	Oropharyngeal swab
CoV-19/Mexico/CMX-INMEGEN-InDRE-04-04-151/20	EPI_ISL_1628648	05/04/21	North America / Mexico / Mexico City	Human	Male	55	unknown	Oropharyngeal swab
CoV-19/Mexico/CMX-INMEGEN-InDRE-04-04-152/20	EPI_ISL_1628649	05/04/21	North America / Mexico / Mexico City	Human	Female	35	unknown	Oropharyngeal swab
CoV-19/Mexico/CMX-INMEGEN-InDRE-04-04-153/20	EPI_ISL_1628650	05/04/21	North America / Mexico / Mexico City	Human	Female	75	unknown	Oropharyngeal swab
CoV-19/Mexico/CMX-INMEGEN-InDRE-04-04-154/20	EPI_ISL_1628651	05/04/21	North America / Mexico / Mexico City	Human	Male	37	unknown	Oropharyngeal swab
CoV-19/Mexico/CMX-INMEGEN-InDRE-04-04-155/20	EPI_ISL_1628652	05/04/21	North America / Mexico / Mexico City	Human	Female	47	unknown	Oropharyngeal swab
CoV-19/Mexico/CMX-INMEGEN-InDRE-04-04-156/20	EPI_ISL_1628653	05/04/21	North America / Mexico / Mexico City	Human	Female	49	unknown	Oropharyngeal swab
CoV-19/Mexico/CMX-INMEGEN-InDRE-04-04-157/20	EPI_ISL_1628654	05/04/21	North America / Mexico / Mexico City	Human	Female	57	unknown	Oropharyngeal swab
CoV-19/Mexico/MEX-INMEGEN-InDRE-04-04-158/20	EPI_ISL_1628655	05/04/21	North America / Mexico / State of Mexico	Human	Female	21	unknown	Oropharyngeal swab
CoV-19/Mexico/CMX-INMEGEN-InDRE-04-04-159/20	EPI_ISL_1628656	05/04/21	North America / Mexico / Mexico City	Human	Male	68	unknown	Oropharyngeal swab
CoV-19/Mexico/CMX-INMEGEN-InDRE-04-04-160/20	EPI_ISL_1628657	05/04/21	North America / Mexico / Mexico City	Human	Female	22	unknown	Oropharyngeal swab
CoV-19/Mexico/CMX-INMEGEN-InDRE-04-04-161/20	EPI_ISL_1628658	05/04/21	North America / Mexico / Mexico City	Human	Female	29	unknown	Oropharyngeal swab
CoV-19/Mexico/MEX-INMEGEN-InDRE-04-04-162/20	EPI_ISL_1628659	05/04/21	North America / Mexico / State of Mexico	Human	Female	17	unknown	Oropharyngeal swab
CoV-19/Mexico/CMX-INMEGEN-InDRE-04-04-163/20	EPI_ISL_1628660	05/04/21	North America / Mexico / Mexico City	Human	Female	42	unknown	Oropharyngeal swab
CoV-19/Mexico/CMX-INMEGEN-InDRE-04-04-164/20	EPI_ISL_1628661	05/04/21	North America / Mexico / Mexico City	Human	Female	58	unknown	Oropharyngeal swab
CoV-19/Mexico/GUA-INMEGEN-InDRE-04-04-165/20	EPI_ISL_1628662	02/04/21	North America / Mexico / Guanajuato	Human	Male	35	unknown	Oropharyngeal swab
CoV-19/Mexico/CMX-INMEGEN-InDRE-04-04-166/20	EPI_ISL_1628663	29/03/21	North America / Mexico / Mexico City	Human	Female	52	unknown	Oropharyngeal swab
CoV-19/Mexico/CMX-INMEGEN-InDRE-04-04-167/20	EPI_ISL_1628664	05/04/21	North America / Mexico / Mexico City	Human	Male	38	unknown	Oropharyngeal swab
CoV-19/Mexico/MEX-INMEGEN-InDRE-04-04-168/20	EPI_ISL_1628665	05/04/21	North America / Mexico / State of Mexico	Human	Female	39	unknown	Oropharyngeal swab
CoV-19/Mexico/CMX-INMEGEN-InDRE-04-04-170/20	EPI_ISL_1628666	05/04/21	North America / Mexico / Mexico City	Human	Male	54	unknown	Oropharyngeal swab
CoV-19/Mexico/CMX-INMEGEN-InDRE-04-04-172/20	EPI_ISL_1628667	05/04/21	North America / Mexico / Mexico City	Human	Female	29	unknown	Oropharyngeal swab
CoV-19/Mexico/CMX-INMEGEN-InDRE-04-04-173/20	EPI_ISL_1628668	05/04/21	North America / Mexico / Mexico City	Human	Female	37	unknown	Oropharyngeal swab

CoV-19/Mexico/CMX-INMEGEN-InDRE-04-04-174/20	EPI_ISL_1628669	06/04/21	North America / Mexico / Mexico City	Human	Male	51	unknown	Oropharyngeal swab
CoV-19/Mexico/CMX-INMEGEN-InDRE-04-04-175/20	EPI_ISL_1628670	05/04/21	North America / Mexico / Mexico City	Human	Male	35	unknown	Oropharyngeal swab
CoV-19/Mexico/CMX-INMEGEN-InDRE-04-04-176/20	EPI_ISL_1628671	05/04/21	North America / Mexico / Mexico City	Human	Female	25	unknown	Oropharyngeal swab
CoV-19/Mexico/CMX-INMEGEN-InDRE-04-04-177/20	EPI_ISL_1628672	05/04/21	North America / Mexico / Mexico City	Human	Male	55	unknown	Oropharyngeal swab
CoV-19/Mexico/CMX-INMEGEN-InDRE-04-04-178/20	EPI_ISL_1628673	05/04/21	North America / Mexico / Mexico City	Human	Female	31	unknown	Oropharyngeal swab
CoV-19/Mexico/CMX-INMEGEN-InDRE-04-04-179/20	EPI_ISL_1628674	05/04/21	North America / Mexico / Mexico City	Human	Male	29	unknown	Oropharyngeal swab
CoV-19/Mexico/CMX-INMEGEN-InDRE-04-04-181/20	EPI_ISL_1628675	05/04/21	North America / Mexico / Mexico City	Human	Female	59	unknown	Oropharyngeal swab
CoV-19/Mexico/CMX-INMEGEN-InDRE-04-04-182/20	EPI_ISL_1628676	04/04/21	North America / Mexico / Mexico City	Human	Male	39	unknown	Oropharyngeal swab
CoV-19/Mexico/CMX-INMEGEN-InDRE-04-04-183/20	EPI_ISL_1628677	04/04/21	North America / Mexico / Mexico City	Human	Female	24	unknown	Oropharyngeal swab
CoV-19/Mexico/CMX-INMEGEN-InDRE-04-04-184/20	EPI_ISL_1628678	06/04/21	North America / Mexico / Mexico City	Human	Female	25	unknown	Oropharyngeal swab
CoV-19/Mexico/CMX-INMEGEN-InDRE-04-04-185/20	EPI_ISL_1628679	06/04/21	North America / Mexico / Mexico City	Human	Female	49	unknown	Oropharyngeal swab
CoV-19/Mexico/CMX-INMEGEN-InDRE-04-04-186/20	EPI_ISL_1628680	06/04/21	North America / Mexico / Mexico City	Human	Female	35	unknown	Oropharyngeal swab
CoV-19/Mexico/CMX-INMEGEN-InDRE-04-04-187/20	EPI_ISL_1628681	06/04/21	North America / Mexico / Mexico City	Human	Female	27	unknown	Oropharyngeal swab
CoV-19/Mexico/CMX-INMEGEN-InDRE-04-04-188/20	EPI_ISL_1628682	06/04/21	North America / Mexico / Mexico City	Human	Female	41	unknown	Oropharyngeal swab
CoV-19/Mexico/MEX-INMEGEN-InDRE-04-04-189/20	EPI_ISL_1628683	06/04/21	North America / Mexico / State of Mexico	Human	Male	75	unknown	Oropharyngeal swab
CoV-19/Mexico/CMX-INMEGEN-InDRE-04-04-190/20	EPI_ISL_1628684	06/04/21	North America / Mexico / Mexico City	Human	Female	55	unknown	Oropharyngeal swab
hCoV-19/Mexico/CHP-InDRE_F11915_S1209/2021	EPI_ISL_1651229	14/03/21	North America / Mexico / Chiapas	Human	Female	63	Hospitalized	Oropharyngeal swab
hCoV-19/Mexico/CHP-InDRE_F11917_S1217/2021	EPI_ISL_1651230	16/03/21	North America / Mexico / Chiapas	Human	Female	50	Released	Oropharyngeal swab
hCoV-19/Mexico/PUE-InDRE_F11926_S1218/2021	EPI_ISL_1651231	16/03/21	North America / Mexico / Puebla	Human	Male	63	Hospitalized	Oropharyngeal swab
hCoV-19/Mexico/PUE-InDRE_F11929_S1219/2021	EPI_ISL_1651232	14/03/21	North America / Mexico / Puebla	Human	Female	87	Deceased	Oropharyngeal swab
hCoV-19/Mexico/PUE-InDRE_F11931_S1220/2021	EPI_ISL_1651233	16/03/21	North America / Mexico / Puebla	Human	Male	54	Hospitalized	Oropharyngeal swab
hCoV-19/Mexico/PUE-InDRE_F11932_S1221/2021	EPI_ISL_1651234	17/03/21	North America / Mexico / Puebla	Human	Male	41	Released	Oropharyngeal swab
hCoV-19/Mexico/NLE-InDRE_F11938_S1222/2021	EPI_ISL_1651235	16/03/21	North America / Mexico / Nuevo Leon	Human	Male	56	Hospitalized	Oropharyngeal swab
hCoV-19/Mexico/NLE-InDRE_F11939_S1223/2021	EPI_ISL_1651236	15/03/21	North America / Mexico / Nuevo Leon	Human	Female	45	Released	Oropharyngeal swab
hCoV-19/Mexico/NLE-InDRE_F11940_S1224/2021	EPI_ISL_1651237	15/03/21	North America / Mexico / Nuevo Leon	Human	Male	75	Released	Oropharyngeal swab
hCoV-19/Mexico/NLE-InDRE_F11941_S1225/2021	EPI_ISL_1651238	16/03/21	North America / Mexico / Nuevo Leon	Human	Male	44	Released	Oropharyngeal swab
hCoV-19/Mexico/NLE-InDRE_F11942_S1226/2021	EPI_ISL_1651239	16/03/21	North America / Mexico / Nuevo Leon	Human	Female	34	Released	Oropharyngeal swab
hCoV-19/Mexico/NLE-InDRE_F11943_S1227/2021	EPI_ISL_1651240	16/03/21	North America / Mexico / Nuevo Leon	Human	Male	34	Released	Oropharyngeal swab
hCoV-19/Mexico/NLE-InDRE_F11944_S1228/2021	EPI_ISL_1651241	15/03/21	North America / Mexico / Nuevo Leon	Human	Male	20	Released	Oropharyngeal swab
hCoV-19/Mexico/NLE-InDRE_F11948_S1229/2021	EPI_ISL_1651242	16/03/21	North America / Mexico / Nuevo Leon	Human	Female	28	Released	Oropharyngeal swab
hCoV-19/Mexico/NLE-InDRE_F11949_S1230/2021	EPI_ISL_1651243	16/03/21	North America / Mexico / Nuevo Leon	Human	Female	50	Released	Oropharyngeal swab
hCoV-19/Mexico/NLE-InDRE_F11950_S1231/2021	EPI_ISL_1651244	16/03/21	North America / Mexico / Nuevo Leon	Human	Male	50	Released	Oropharyngeal swab
hCoV-19/Mexico/NLE-InDRE_F11951_S1232/2021	EPI_ISL_1651245	16/03/21	North America / Mexico / Nuevo Leon	Human	Female	45	Released	Oropharyngeal swab
hCoV-19/Mexico/NLE-InDRE_F11952_S1233/2021	EPI_ISL_1651246	16/03/21	North America / Mexico / Nuevo Leon	Human	Male	64	Released	Oropharyngeal swab
hCoV-19/Mexico/NLE-InDRE_F11953_S1234/2021	EPI_ISL_1651247	16/03/21	North America / Mexico / Nuevo Leon	Human	Male	31	Released	Oropharyngeal swab
hCoV-19/Mexico/NLE-InDRE_F11955_S1235/2021	EPI_ISL_1651248	16/03/21	North America / Mexico / Nuevo Leon	Human	Female	23	Released	Oropharyngeal swab
hCoV-19/Mexico/NLE-InDRE_F11956_S1236/2021	EPI_ISL_1651249	17/03/21	North America / Mexico / Nuevo Leon	Human	Male	55	Released	Oropharyngeal swab
hCoV-19/Mexico/JAL-InDRE_F11988_S1237/2021	EPI_ISL_1651250	18/03/21	North America / Mexico / Jalisco	Human	Female	72	Hospitalized	Oropharyngeal swab
hCoV-19/Mexico/JAL-InDRE_F11991_S1238/2021	EPI_ISL_1651251	20/03/21	North America / Mexico / Jalisco	Human	Male	60	Released	Oropharyngeal swab
hCoV-19/Mexico/OAX-InDRE_F11998_S1239/2021	EPI_ISL_1651252	16/03/21	North America / Mexico / Oaxaca	Human	Male	19	Released	Oropharyngeal swab
hCoV-19/Mexico/SIN-InDRE_F12014_S1240/2021	EPI_ISL_1651253	14/03/21	North America / Mexico / Sinaloa	Human	Male	66	Hospitalized	Oropharyngeal swab
hCoV-19/Mexico/SON-InDRE_F12033_S1241/2021	EPI_ISL_1651254	17/03/21	North America / Mexico / Sonora	Human	Male	41	Released	Oropharyngeal swab
hCoV-19/Mexico/MOR-InDRE_F12082_S1242/2021	EPI_ISL_1651255	14/03/21	North America / Mexico / Morelos	Human	Male	77	Hospitalized	Oropharyngeal swab
hCoV-19/Mexico/MOR-InDRE_F12086_S1243/2021	EPI_ISL_1651256	15/03/21	North America / Mexico / Morelos	Human	Female	61	Hospitalized	Oropharyngeal swab

hCoV-19/Mexico/MOR-InDRE_F12088_S1244/2021	EPI_ISL_1651257	15/03/21	North America / Mexico / Morelos	Human	Female	56	Released	Oropharyngeal swab
hCoV-19/Mexico/MOR-InDRE_F12091_S1245/2021	EPI_ISL_1651258	16/03/21	North America / Mexico / Morelos	Human	Female	57	Hospitalized	Oropharyngeal swab
hCoV-19/Mexico/MOR-InDRE_F12093_S1246/2021	EPI_ISL_1651259	17/03/21	North America / Mexico / Morelos	Human	Female	45	Released	Oropharyngeal swab
hCoV-19/Mexico/MOR-InDRE_F12094_S1247/2021	EPI_ISL_1651260	15/03/21	North America / Mexico / Morelos	Human	Female	59	Hospitalized	Oropharyngeal swab
hCoV-19/Mexico/MOR-InDRE_F12095_S1248/2021	EPI_ISL_1651261	17/03/21	North America / Mexico / Morelos	Human	Male	50	Hospitalized	Oropharyngeal swab
hCoV-19/Mexico/MOR-InDRE_F12099_S1249/2021	EPI_ISL_1651262	18/03/21	North America / Mexico / Morelos	Human	Male	50	Released	Oropharyngeal swab
hCoV-19/Mexico/MOR-InDRE_F12100_S1250/2021	EPI_ISL_1651263	18/03/21	North America / Mexico / Morelos	Human	Male	49	Released	Oropharyngeal swab
hCoV-19/Mexico/MOR-InDRE_F12101_S1251/2021	EPI_ISL_1651264	18/03/21	North America / Mexico / Morelos	Human	Female	77	Released	Oropharyngeal swab
hCoV-19/Mexico/MOR-InDRE_F12103_S1252/2021	EPI_ISL_1651265	19/03/21	North America / Mexico / Morelos	Human	Male	44	Released	Oropharyngeal swab
hCoV-19/Mexico/MOR-InDRE_F12104_S1253/2021	EPI_ISL_1651266	19/03/21	North America / Mexico / Morelos	Human	Male	46	Released	Oropharyngeal swab
hCoV-19/Mexico/MOR-InDRE_F12105_S1254/2021	EPI_ISL_1651267	19/03/21	North America / Mexico / Morelos	Human	Male	68	Released	Oropharyngeal swab
hCoV-19/Mexico/MOR-InDRE_F12106_S1255/2021	EPI_ISL_1651268	19/03/21	North America / Mexico / Morelos	Human	Female	30	Released	Oropharyngeal swab
hCoV-19/Mexico/MOR-InDRE_F12107_S1256/2021	EPI_ISL_1651269	19/03/21	North America / Mexico / Morelos	Human	Male	92	Released	Oropharyngeal swab
hCoV-19/Mexico/MOR-InDRE_F12108_S1257/2021	EPI_ISL_1651270	18/03/21	North America / Mexico / Morelos	Human	Male	68	Hospitalized	Oropharyngeal swab
hCoV-19/Mexico/MOR-InDRE_F12109_S1258/2021	EPI_ISL_1651271	18/03/21	North America / Mexico / Morelos	Human	Male	70	Hospitalized	Oropharyngeal swab
hCoV-19/Mexico/MOR-InDRE_F12110_S1259/2021	EPI_ISL_1651272	19/03/21	North America / Mexico / Morelos	Human	Male	68	Hospitalized	Oropharyngeal swab
hCoV-19/Mexico/MOR-InDRE_F12113_S1260/2021	EPI_ISL_1651273	22/03/21	North America / Mexico / Morelos	Human	Female	49	Hospitalized	Oropharyngeal swab
hCoV-19/Mexico/MOR-InDRE_F12114_S1261/2021	EPI_ISL_1651274	22/03/21	North America / Mexico / Morelos	Human	Male	40	Released	Oropharyngeal swab
hCoV-19/Mexico/MOR-InDRE_F12115_S1262/2021	EPI_ISL_1651275	21/03/21	North America / Mexico / Morelos	Human	Female	58	Hospitalized	Oropharyngeal swab
hCoV-19/Mexico/MOR-InDRE_F12117_S1263/2021	EPI_ISL_1651276	19/03/21	North America / Mexico / Morelos	Human	Male	43	Released	Oropharyngeal swab
hCoV-19/Mexico/QUE-InDRE_F12126_S1264/2021	EPI_ISL_1651277	17/03/21	North America / Mexico / Queretaro	Human	Male	62	Released	Oropharyngeal swab
hCoV-19/Mexico/QUE-InDRE_F12127_S1265/2021	EPI_ISL_1651278	17/03/21	North America / Mexico / Queretaro	Human	Female	57	Released	Oropharyngeal swab
hCoV-19/Mexico/QUE-InDRE_F12128_S1266/2021	EPI_ISL_1651279	19/03/21	North America / Mexico / Queretaro	Human	Male	35	Released	Oropharyngeal swab
hCoV-19/Mexico/QUE-InDRE_F12130_S1267/2021	EPI_ISL_1651280	19/03/21	North America / Mexico / Queretaro	Human	Male	96	Hospitalized	Oropharyngeal swab
hCoV-19/Mexico/QUE-InDRE_F12133_S1268/2021	EPI_ISL_1651281	20/03/21	North America / Mexico / Queretaro	Human	Female	34	Released	Oropharyngeal swab
hCoV-19/Mexico/QUE-InDRE_F12134_S1269/2021	EPI_ISL_1651282	20/03/21	North America / Mexico / Queretaro	Human	Male	66	Released	Oropharyngeal swab
hCoV-19/Mexico/QUE-InDRE_F12137_S1270/2021	EPI_ISL_1651283	20/03/21	North America / Mexico / Queretaro	Human	Female	47	Deceased	Oropharyngeal swab
hCoV-19/Mexico/QUE-InDRE_F12138_S1271/2021	EPI_ISL_1651284	21/03/21	North America / Mexico / Queretaro	Human	Male	43	Released	Oropharyngeal swab
hCoV-19/Mexico/QUE-InDRE_F12140_S1272/2021	EPI_ISL_1651285	21/03/21	North America / Mexico / Queretaro	Human	Male	28	Released	Oropharyngeal swab
hCoV-19/Mexico/QUE-InDRE_F12143_S1273/2021	EPI_ISL_1651286	21/03/21	North America / Mexico / Queretaro	Human	Female	87	Hospitalized	Oropharyngeal swab
hCoV-19/Mexico/QUE-InDRE_F12147_S1274/2021	EPI_ISL_1651287	22/03/21	North America / Mexico / Queretaro	Human	Female	81	Deceased	Oropharyngeal swab
hCoV-19/Mexico/QUE-InDRE_F12148_S1275/2021	EPI_ISL_1651876	22/03/21	North America / Mexico / Queretaro	Human	Male	67	Hospitalized	Oropharyngeal swab
hCoV-19/Mexico/CHH-InDRE_F12153_S1276/2021	EPI_ISL_1651877	15/03/21	North America / Mexico / Chihuahua	Human	Female	61	Live	Oropharyngeal swab
hCoV-19/Mexico/CHH-InDRE_F12154_S1277/2021	EPI_ISL_1651878	15/03/21	North America / Mexico / Chihuahua	Human	Female	84	Hospitalized	Oropharyngeal swab
hCoV-19/Mexico/CHH-InDRE_F12163_S1278/2021	EPI_ISL_1651879	17/03/21	North America / Mexico / Chihuahua	Human	Female	41	Released	Oropharyngeal swab
hCoV-19/Mexico/CHH-InDRE_F12164_S1279/2021	EPI_ISL_1651880	17/03/21	North America / Mexico / Chihuahua	Human	Female	38	Released	Oropharyngeal swab
hCoV-19/Mexico/VER-InDRE_F12186_S1280/2021	EPI_ISL_1651881	17/03/21	North America / Mexico / Veracruz	Human	Female	54	Released	Oropharyngeal swab
hCoV-19/Mexico/VER-InDRE_F12187_S1281/2021	EPI_ISL_1651882	16/03/21	North America / Mexico / Veracruz	Human	Male	33	Live	Oropharyngeal swab
hCoV-19/Mexico/YUC-InDRE_F12189_S1282/2021	EPI_ISL_1651883	22/03/21	North America / Mexico / Yucatan	Human	Female	45	Released	Oropharyngeal swab
hCoV-19/Mexico/YUC-InDRE_F12191_S1283/2021	EPI_ISL_1651884	22/03/21	North America / Mexico / Yucatan	Human	Male	39	Released	Oropharyngeal swab
hCoV-19/Mexico/YUC-InDRE_F12192_S1284/2021	EPI_ISL_1651885	22/03/21	North America / Mexico / Yucatan	Human	Female	30	Released	Oropharyngeal swab
hCoV-19/Mexico/YUC-InDRE_F12195_S1285/2021	EPI_ISL_1651886	22/03/21	North America / Mexico / Yucatan	Human	Female	84	Hospitalized	Oropharyngeal swab
hCoV-19/Mexico/TAM-InDRE_F12200_S1286/2021	EPI_ISL_1651887	16/03/21	North America / Mexico / Tamaulipas	Human	Male	75	Hospitalized	Oropharyngeal swab
hCoV-19/Mexico/CMX-InDRE_F12208_S1287/2021	EPI_ISL_1651888	16/03/21	North America / Mexico / Mexico City	Human	Male	72	Hospitalized	Oropharyngeal swab

hCoV-19/Mexico/BCS-InDRE_F12235_S1289/2021	EPI_ISL_1651890	20/03/21	North America / Mexico / Baja California Sur	Human	Female	43	Released		Oropharyngeal swab
hCoV-19/Mexico/BCS-InDRE_F12236_S1290/2021	EPI_ISL_1651891	20/03/21	North America / Mexico / Baja California Sur	Human	Male	43	Released		Oropharyngeal swab
hCoV-19/Mexico/BCS-InDRE_F12240_S1292/2021	EPI_ISL_1651893	20/03/21	North America / Mexico / Baja California Sur	Human	Male	16	Released		Oropharyngeal swab
hCoV-19/Mexico/BCS-InDRE_F12243_S1294/2021	EPI_ISL_1651895	21/03/21	North America / Mexico / Baja California Sur	Human	Female	77	Released		Oropharyngeal swab
hCoV-19/Mexico/ROO-InDRE_F12254_S1295/2021	EPI_ISL_1651896	18/03/21	North America / Mexico / Quintana Roo	Human	Male	47	Released		Oropharyngeal swab
hCoV-19/Mexico/ROO-InDRE_F12255_S1296/2021	EPI_ISL_1651897	20/03/21	North America / Mexico / Quintana Roo	Human	Male	28	Released		Oropharyngeal swab
hCoV-19/Mexico/ROO-InDRE_F12256_S1297/2021	EPI_ISL_1651898	20/03/21	North America / Mexico / Quintana Roo	Human	Female	62	Released		Oropharyngeal swab
hCoV-19/Mexico/CMX-InDRE_F12277_S1298/2021	EPI_ISL_1651899	17/03/21	North America / Mexico / Mexico City	Human	Female	71	Released		Oropharyngeal swab
hCoV-19/Mexico/BCS-InDRE_F12278_S1299/2021	EPI_ISL_1651900	18/03/21	North America / Mexico / Baja California Sur	Human	Female	72	Released		Oropharyngeal swab
hCoV-19/Mexico/CMX-InDRE_F12282_S1300/2021	EPI_ISL_1651901	19/03/21	North America / Mexico / Mexico City	Human	Female	64	Released		Oropharyngeal swab
hCoV-19/Mexico/CMX-InDRE_F12283_S1301/2021	EPI_ISL_1651902	16/03/21	North America / Mexico / Mexico City	Human	Male	54	Released		Oropharyngeal swab
hCoV-19/Mexico/MEX-InDRE_F12284_S1302/2021	EPI_ISL_1651903	17/03/21	North America / Mexico / State of Mexico	Human	Female	87	Released		Oropharyngeal swab
hCoV-19/Mexico/MOR-InDRE_F12299_S1303/2021	EPI_ISL_1651904	19/03/21	North America / Mexico / Morelos	Human	Male	90	Hospitalized		Oropharyngeal swab
hCoV-19/Mexico/MOR-InDRE_F12306_S1304/2021	EPI_ISL_1651905	23/03/21	North America / Mexico / Morelos	Human	Female	55	Released		Oropharyngeal swab
hCoV-19/Mexico/MOR-InDRE_F12307_S1305/2021	EPI_ISL_1651906	23/03/21	North America / Mexico / Morelos	Human	Female	55	Released		Oropharyngeal swab
hCoV-19/Mexico/MOR-InDRE_F12310_S1306/2021	EPI_ISL_1651907	23/03/21	North America / Mexico / Morelos	Human	Male	81	Hospitalized		Oropharyngeal swab
hCoV-19/Mexico/MOR-InDRE_F12311_S1307/2021	EPI_ISL_1651908	22/03/21	North America / Mexico / Morelos	Human	Male	44	Hospitalized		Oropharyngeal swab
hCoV-19/Mexico/SIN_CIAD_ID1988/2021	EPI_ISL_1660619	19/03/21	North America / Mexico / Sinaloa	Human	Male	59	unknown		Oropharyngeal swab
hCoV-19/Mexico/SIN_CIAD_HJ1345/2021	EPI_ISL_1660622	26/03/21	North America / Mexico / Sinaloa	Human	Male	70	unknown		Oropharyngeal swab
hCoV-19/Mexico/SIN_CIAD_S6400/2021	EPI_ISL_1660623	26/03/21	North America / Mexico / Sinaloa	Human	Female	38	unknown		Oropharyngeal swab
hCoV-19/Mexico/SIN_CIAD_HJ1356/2021	EPI_ISL_1660624	29/03/21	North America / Mexico / Sinaloa	Human	Female	66	unknown		Oropharyngeal swab
hCoV-19/Mexico/SIN_CIAD_S6410/2021	EPI_ISL_1660626	27/03/21	North America / Mexico / Sinaloa	Human	Male	63	unknown		Oropharyngeal swab
hCoV-19/Mexico/SIN_CIAD_ID2083/2021	EPI_ISL_1660627	30/03/21	North America / Mexico / Sinaloa	Human	Male	23	unknown		Oropharyngeal swab
hCoV-19/Mexico/CMX-INMEGEN-03-09-26/2021	EPI_ISL_1661254	17/03/21	North America / Mexico / Mexico City	Human	Male	39	unknown		Oropharyngeal swab
hCoV-19/Mexico/MOR-InDRE_F12323_S1308/2021	EPI_ISL_1661474	25/03/21	North America / Mexico / Morelos	Human	Female	68	Released		Oropharyngeal swab
hCoV-19/Mexico/MOR-InDRE_F12318_S1309/2021	EPI_ISL_1661475	24/03/21	North America / Mexico / Morelos	Human	Female	61	Released		Oropharyngeal swab
hCoV-19/Mexico/MOR-InDRE_F12320_S1310/2021	EPI_ISL_1661476	24/03/21	North America / Mexico / Morelos	Human	Male	67	Hospitalized		Oropharyngeal swab
hCoV-19/Mexico/MOR-InDRE_F12312_S1311/2021	EPI_ISL_1661477	22/03/21	North America / Mexico / Morelos	Human	Female	64	Hospitalized		Oropharyngeal swab
hCoV-19/Mexico/PUE-InDRE_F12325_S1312/2021	EPI_ISL_1661478	26/03/21	North America / Mexico / Puebla	Human	Male	56	Hospitalized		Oropharyngeal swab
hCoV-19/Mexico/MOR-InDRE_F12326_S1313/2021	EPI_ISL_1661479	25/03/21	North America / Mexico / Morelos	Human	Female	72	Hospitalized		Oropharyngeal swab
hCoV-19/Mexico/MOR-InDRE_F12327_S1314/2021	EPI_ISL_1661480	25/03/21	North America / Mexico / Morelos	Human	Male	57	Hospitalized		Oropharyngeal swab
hCoV-19/Mexico/CHH-InDRE_F12357_S1315/2021	EPI_ISL_1661481	22/03/21	North America / Mexico / Chihuahua	Human	Female	36	Live		Oropharyngeal swab
hCoV-19/Mexico/CHH-InDRE_F12360_S1316/2021	EPI_ISL_1661482	22/03/21	North America / Mexico / Chihuahua	Human	Male	76	Hospitalized		Oropharyngeal swab
hCoV-19/Mexico/CHH-InDRE_F12362_S1317/2021	EPI_ISL_1661483	22/03/21	North America / Mexico / Chihuahua	Human	Female	25	Released		Oropharyngeal swab
hCoV-19/Mexico/CHH-InDRE_F12363_S1318/2021	EPI_ISL_1661484	22/03/21	North America / Mexico / Chihuahua	Human	Male	18	Released		Oropharyngeal swab
hCoV-19/Mexico/CHH-InDRE_F12364_S1319/2021	EPI_ISL_1661485	22/03/21	North America / Mexico / Chihuahua	Human	Female	40	Released		Oropharyngeal swab
hCoV-19/Mexico/CHH-InDRE_F12365_S1320/2021	EPI_ISL_1661486	23/03/21	North America / Mexico / Chihuahua	Human	Male	52	Released		Oropharyngeal swab
hCoV-19/Mexico/CHH-InDRE_F12368_S1321/2021	EPI_ISL_1661487	18/03/21	North America / Mexico / Chihuahua	Human	Female	32	Hospitalized		Oropharyngeal swab
hCoV-19/Mexico/CHH-InDRE_F12369_S1322/2021	EPI_ISL_1661488	18/03/21	North America / Mexico / Chihuahua	Human	Female	67	Hospitalized		Oropharyngeal swab
hCoV-19/Mexico/CHH-InDRE_F12370_S1323/2021	EPI_ISL_1661489	19/03/21	North America / Mexico / Chihuahua	Human	Female	74	Deceased		Oropharyngeal swab
hCoV-19/Mexico/CMX-InDRE_F12375_S1324/2021	EPI_ISL_1661490	16/03/21	North America / Mexico / Mexico City	Human	Female	65	Hospitalized		Oropharyngeal swab
hCoV-19/Mexico/MEX-InDRE_F12379_S1325/2021	EPI_ISL_1661491	18/03/21	North America / Mexico / State of Mexico	Human	Female	85	Hospitalized		Oropharyngeal swab
hCoV-19/Mexico/MEX-InDRE_F12380_S1326/2021	EPI_ISL_1661492	17/03/21	North America / Mexico / State of Mexico	Human	Female	59	Hospitalized		Oropharyngeal swab
hCoV-19/Mexico/CMX-InDRE_F12383_S1327/2021	EPI_ISL_1661493	17/03/21	North America / Mexico / Mexico City	Human	Male	86	Hospitalized		Oropharyngeal swab

hCoV-19/Mexico/CMX-InDRE_F12384_S1328/2021	EPI_ISL_1661494	17/03/21	North America / Mexico / Mexico City	Human	Male	46	Deceased	Oropharyngeal swab
hCoV-19/Mexico/GUA-InDRE_F12422_S1329/2021	EPI_ISL_1661495	20/03/21	North America / Mexico / Guanajuato	Human	Female	63	Deceased	Oropharyngeal swab
hCoV-19/Mexico/GUA-InDRE_F12425_S1330/2021	EPI_ISL_1661496	15/03/21	North America / Mexico / Guanajuato	Human	Male	39	Live	Oropharyngeal swab
hCoV-19/Mexico/GUA-InDRE_F12428_S1331/2021	EPI_ISL_1661497	16/03/21	North America / Mexico / Guanajuato	Human	Female	24	Live	Oropharyngeal swab
hCoV-19/Mexico/GUA-InDRE_F12430_S1332/2021	EPI_ISL_1661498	16/03/21	North America / Mexico / Guanajuato	Human	Male	83	Released	Oropharyngeal swab
hCoV-19/Mexico/GUA-InDRE_F12432_S1333/2021	EPI_ISL_1661499	24/03/21	North America / Mexico / Guanajuato	Human	Male	82	Released	Oropharyngeal swab
hCoV-19/Mexico/GUA-InDRE_F12435_S1334/2021	EPI_ISL_1661500	23/03/21	North America / Mexico / Guanajuato	Human	Male	65	Hospitalized	Oropharyngeal swab
hCoV-19/Mexico/GUA-InDRE_F12438_S1335/2021	EPI_ISL_1661501	16/03/21	North America / Mexico / Guanajuato	Human	Male	46	Live	Oropharyngeal swab
hCoV-19/Mexico/GUA-InDRE_F12440_S1336/2021	EPI_ISL_1661502	16/03/21	North America / Mexico / Guanajuato	Human	Female	77	Released	Oropharyngeal swab
hCoV-19/Mexico/GUA-InDRE_F12443_S1337/2021	EPI_ISL_1661503	16/03/21	North America / Mexico / Guanajuato	Human	Male	31	Released	Oropharyngeal swab
hCoV-19/Mexico/GUA-InDRE_F12455_S1338/2021	EPI_ISL_1661504	20/03/21	North America / Mexico / Guanajuato	Human	Female	77	Released	Oropharyngeal swab
hCoV-19/Mexico/GUA-InDRE_F12456_S1339/2021	EPI_ISL_1661505	19/03/21	North America / Mexico / Guanajuato	Human	Female	46	Released	Oropharyngeal swab
hCoV-19/Mexico/GUA-InDRE_F12457_S1340/2021	EPI_ISL_1661506	23/03/21	North America / Mexico / Guanajuato	Human	Female	49	Released	Oropharyngeal swab
hCoV-19/Mexico/GUA-InDRE_F12459_S1341/2021	EPI_ISL_1661507	23/03/21	North America / Mexico / Guanajuato	Human	Female	34	Live	Oropharyngeal swab
hCoV-19/Mexico/GUA-InDRE_F12460_S1342/2021	EPI_ISL_1661508	22/03/21	North America / Mexico / Guanajuato	Human	Female	21	Released	Oropharyngeal swab
hCoV-19/Mexico/GUA-InDRE_F12461_S1343/2021	EPI_ISL_1661509	22/03/21	North America / Mexico / Guanajuato	Human	Female	21	Released	Oropharyngeal swab
hCoV-19/Mexico/GUA-InDRE_F12462_S1344/2021	EPI_ISL_1661510	24/03/21	North America / Mexico / Guanajuato	Human	Female	46	Released	Oropharyngeal swab
hCoV-19/Mexico/GUA-InDRE_F12467_S1345/2021	EPI_ISL_1661511	25/03/21	North America / Mexico / Guanajuato	Human	Female	39	Released	Oropharyngeal swab
hCoV-19/Mexico/GUA-InDRE_F12468_S1346/2021	EPI_ISL_1661512	24/03/21	North America / Mexico / Guanajuato	Human	Female	53	Released	Oropharyngeal swab
hCoV-19/Mexico/GUA-InDRE_F12469_S1347/2021	EPI_ISL_1661513	24/03/21	North America / Mexico / Guanajuato	Human	Female	24	Released	Oropharyngeal swab
hCoV-19/Mexico/GUA-InDRE_F12470_S1348/2021	EPI_ISL_1661514	25/03/21	North America / Mexico / Guanajuato	Human	Female	27	Released	Oropharyngeal swab
hCoV-19/Mexico/GUA-InDRE_F12471_S1349/2021	EPI_ISL_1661515	25/03/21	North America / Mexico / Guanajuato	Human	Male	23	Released	Oropharyngeal swab
hCoV-19/Mexico/GUA-InDRE_F12472_S1350/2021	EPI_ISL_1661516	25/03/21	North America / Mexico / Guanajuato	Human	Female	56	Released	Oropharyngeal swab
hCoV-19/Mexico/GUA-InDRE_F12473_S1351/2021	EPI_ISL_1661517	26/03/21	North America / Mexico / Guanajuato	Human	Female	43	Released	Oropharyngeal swab
hCoV-19/Mexico/GUA-InDRE_F12476_S1352/2021	EPI_ISL_1661518	25/03/21	North America / Mexico / Guanajuato	Human	Female	51	Released	Oropharyngeal swab
hCoV-19/Mexico/GUA-InDRE_F12477_S1353/2021	EPI_ISL_1661519	25/03/21	North America / Mexico / Guanajuato	Human	Male	56	Released	Oropharyngeal swab
hCoV-19/Mexico/GUA-InDRE_F12479_S1354/2021	EPI_ISL_1661520	26/03/21	North America / Mexico / Guanajuato	Human	Female	36	Released	Oropharyngeal swab
hCoV-19/Mexico/MEX-InDRE_F12481_S1355/2021	EPI_ISL_1661521	27/03/21	North America / Mexico / State of Mexico	Human	Male	65	Hospitalized	Oropharyngeal swab
hCoV-19/Mexico/QUE-InDRE_F12484_S1356/2021	EPI_ISL_1661522	28/03/21	North America / Mexico / Queretaro	Human	Female	27	Released	Oropharyngeal swab
hCoV-19/Mexico/QUE-InDRE_F12485_S1357/2021	EPI_ISL_1661523	27/03/21	North America / Mexico / Queretaro	Human	Female	42	Released	Oropharyngeal swab
hCoV-19/Mexico/QUE-InDRE_F12486_S1358/2021	EPI_ISL_1661524	27/03/21	North America / Mexico / Queretaro	Human	Male	23	Hospitalized	Oropharyngeal swab
hCoV-19/Mexico/QUE-InDRE_F12487_S1359/2021	EPI_ISL_1661525	27/03/21	North America / Mexico / Queretaro	Human	Male	23	Released	Oropharyngeal swab
hCoV-19/Mexico/QUE-InDRE_F12488_S1360/2021	EPI_ISL_1661526	26/03/21	North America / Mexico / Queretaro	Human	Female	25	Released	Oropharyngeal swab
hCoV-19/Mexico/QUE-InDRE_F12489_S1361/2021	EPI_ISL_1661527	27/03/21	North America / Mexico / Queretaro	Human	Male	57	Released	Oropharyngeal swab
hCoV-19/Mexico/QUE-InDRE_F12492_S1362/2021	EPI_ISL_1661528	26/03/21	North America / Mexico / Queretaro	Human	Male	40	Released	Oropharyngeal swab
hCoV-19/Mexico/QUE-InDRE_F12493_S1363/2021	EPI_ISL_1661529	26/03/21	North America / Mexico / Queretaro	Human	Male	59	Released	Oropharyngeal swab
hCoV-19/Mexico/QUE-InDRE_F12494_S1364/2021	EPI_ISL_1661530	26/03/21	North America / Mexico / Queretaro	Human	Male	36	Released	Oropharyngeal swab
hCoV-19/Mexico/QUE-InDRE_F12495_S1365/2021	EPI_ISL_1661531	25/03/21	North America / Mexico / Queretaro	Human	Male	80	Hospitalized	Oropharyngeal swab
hCoV-19/Mexico/QUE-InDRE_F12496_S1366/2021	EPI_ISL_1661532	25/03/21	North America / Mexico / Queretaro	Human	Female	86	Hospitalized	Oropharyngeal swab
hCoV-19/Mexico/QUE-InDRE_F12497_S1367/2021	EPI_ISL_1661533	25/03/21	North America / Mexico / Queretaro	Human	Male	47	Released	Oropharyngeal swab
hCoV-19/Mexico/HID-InDRE_F12498_S1368/2021	EPI_ISL_1661534	25/03/21	North America / Mexico / Hidalgo	Human	Female	76	Hospitalized	Oropharyngeal swab
hCoV-19/Mexico/PUE-InDRE_F12504_S1369/2021	EPI_ISL_1661535	24/03/21	North America / Mexico / Puebla	Human	Female	50	Released	Oropharyngeal swab
hCoV-19/Mexico/ZAC-InDRE_F12509_S1370/2021	EPI_ISL_1661536	27/03/21	North America / Mexico / Zacatecas	Human	Male	18	Released	Oropharyngeal swab
hCoV-19/Mexico/ZAC-InDRE_F12510_S1371/2021	EPI_ISL_1661537	27/03/21	North America / Mexico / Zacatecas	Human	Female	39	Released	Oropharyngeal swab

hCoV-19/Mexico/ZAC-InDRE_F12519_S1372/2021	EPI_ISL_1661538	26/03/21	North America / Mexico / Zacatecas	Human	Female	51	Released	Oropharyngeal swab
hCoV-19/Mexico/HID-InDRE_F12521_S1373/2021	EPI_ISL_1661539	23/03/21	North America / Mexico / Hidalgo	Human	Male	39	Hospitalized	Oropharyngeal swab
hCoV-19/Mexico/HID-InDRE_F12522_S1374/2021	EPI_ISL_1661540	23/03/21	North America / Mexico / Hidalgo	Human	Male	23	Live	Oropharyngeal swab
hCoV-19/Mexico/HID-InDRE_F12523_S1375/2021	EPI_ISL_1661541	26/03/21	North America / Mexico / Hidalgo	Human	Female	89	Released	Oropharyngeal swab
hCoV-19/Mexico/MOR-InDRE_F12328_S1376/2021	EPI_ISL_1661542	26/03/21	North America / Mexico / Morelos	Human	Male	77	Released	Oropharyngeal swab
hCoV-19/Mexico/MOR-InDRE_F12329_S1377/2021	EPI_ISL_1661543	26/03/21	North America / Mexico / Morelos	Human	Male	73	Released	Oropharyngeal swab
hCoV-19/Mexico/ZAC-InDRE_F12512_S1378/2021	EPI_ISL_1661544	27/03/21	North America / Mexico / Zacatecas	Human	Female	27	Released	Oropharyngeal swab
hCoV-19/Mexico/ZAC-InDRE_F12514_S1379/2021	EPI_ISL_1661545	27/03/21	North America / Mexico / Zacatecas	Human	Female	35	Released	Oropharyngeal swab
hCoV-19/Mexico/ZAC-InDRE_F12515_S1380/2021	EPI_ISL_1661546	27/03/21	North America / Mexico / Zacatecas	Human	Female	51	Released	Oropharyngeal swab
hCoV-19/Mexico/ZAC-InDRE_F12516_S1381/2021	EPI_ISL_1661547	27/03/21	North America / Mexico / Zacatecas	Human	Female	27	Released	Oropharyngeal swab
hCoV-19/Mexico/MIC-InDRE_F11838_S1382/2021	EPI_ISL_1661548	16/03/21	North America / Mexico / Michoacan	Human	Female	62	Released	Oropharyngeal swab
hCoV-19/Mexico/CMX-InDRE_F12418_S1383/2021	EPI_ISL_1661549	18/03/21	North America / Mexico / Mexico City	Human	Female	51	unknown	Oropharyngeal swab
hCoV-19/Mexico/HID-InDRE_F12665_S1386/2021	EPI_ISL_1661551	01/04/21	North America / Mexico / Hidalgo	Human	Male	54	Released	Oropharyngeal swab
hCoV-19/Mexico/HID-InDRE_F12666_S1387/2021	EPI_ISL_1661552	03/04/21	North America / Mexico / Hidalgo	Human	Female	52	Hospitalized	Oropharyngeal swab
hCoV-19/Mexico/NLE-InDRE_F12688_S1388/2021	EPI_ISL_1661553	31/03/21	North America / Mexico / Nuevo Leon	Human	Female	49	Hospitalized	Oropharyngeal swab
hCoV-19/Mexico/NLE-InDRE_F12689_S1389/2021	EPI_ISL_1661554	31/03/21	North America / Mexico / Nuevo Leon	Human	Female	70	Hospitalized	Oropharyngeal swab
hCoV-19/Mexico/NLE-InDRE_F12690_S1390/2021	EPI_ISL_1661555	31/03/21	North America / Mexico / Nuevo Leon	Human	Male	44	Hospitalized	Oropharyngeal swab
hCoV-19/Mexico/NLE-InDRE_F12691_S1391/2021	EPI_ISL_1661556	31/03/21	North America / Mexico / Nuevo Leon	Human	Female	30	Hospitalized	Oropharyngeal swab
hCoV-19/Mexico/SLP-InDRE_F12692_S1392/2021	EPI_ISL_1661557	31/03/21	North America / Mexico / San Luis Potosi	Human	Female	74	Hospitalized	Oropharyngeal swab
hCoV-19/Mexico/NLE-InDRE_F12695_S1393/2021	EPI_ISL_1661558	30/03/21	North America / Mexico / Nuevo Leon	Human	Female	55	Released	Oropharyngeal swab
hCoV-19/Mexico/NLE-InDRE_F12696_S1394/2021	EPI_ISL_1661559	31/03/21	North America / Mexico / Nuevo Leon	Human	Female	55	Released	Oropharyngeal swab
hCoV-19/Mexico/NLE-InDRE_F12697_S1395/2021	EPI_ISL_1661560	31/03/21	North America / Mexico / Nuevo Leon	Human	Female	42	Released	Oropharyngeal swab
hCoV-19/Mexico/NLE-InDRE_F12698_S1396/2021	EPI_ISL_1661561	31/03/21	North America / Mexico / Nuevo Leon	Human	Female	22	Released	Oropharyngeal swab
hCoV-19/Mexico/NLE-InDRE_F12699_S1397/2021	EPI_ISL_1661562	31/03/21	North America / Mexico / Nuevo Leon	Human	Male	19	Released	Oropharyngeal swab
hCoV-19/Mexico/NLE-InDRE_F12700_S1398/2021	EPI_ISL_1661563	31/03/21	North America / Mexico / Nuevo Leon	Human	Male	71	Released	Oropharyngeal swab
hCoV-19/Mexico/NLE-InDRE_F12701_S1399/2021	EPI_ISL_1661564	31/03/21	North America / Mexico / Nuevo Leon	Human	Male	13	Released	Oropharyngeal swab
hCoV-19/Mexico/GUA-InDRE_F12706_S1400/2021	EPI_ISL_1661565	30/03/21	North America / Mexico / Guanajuato	Human	Female	76	Hospitalized	Oropharyngeal swab
hCoV-19/Mexico/GUA-InDRE_F12707_S1401/2021	EPI_ISL_1661566	31/03/21	North America / Mexico / Guanajuato	Human	Female	79	Hospitalized	Oropharyngeal swab
hCoV-19/Mexico/GUA-InDRE_F12712_S1402/2021	EPI_ISL_1661567	30/03/21	North America / Mexico / Guanajuato	Human	Male	88	Live	Oropharyngeal swab
hCoV-19/Mexico/GUA-InDRE_F12714_S1403/2021	EPI_ISL_1661568	30/03/21	North America / Mexico / Guanajuato	Human	Male	56	Released	Oropharyngeal swab
hCoV-19/Mexico/GUA-InDRE_F12715_S1404/2021	EPI_ISL_1661569	31/03/21	North America / Mexico / Guanajuato	Human	Male	21	Released	Oropharyngeal swab
hCoV-19/Mexico/GRO-InDRE_F12806_S1405/2021	EPI_ISL_1661570	30/03/21	North America / Mexico / Guerrero	Human	Male	83	Hospitalized	Oropharyngeal swab
hCoV-19/Mexico/AGU_LANGEBIO_IMSS_0460/2021	EPI_ISL_1661977	24/03/21	North America / Mexico / Aguascalientes	Human	Male	41	Ambulatory	Nasopharyngeal swab
hCoV-19/Mexico/AGU_LANGEBIO_IMSS_0487/2021	EPI_ISL_1661978	30/03/21	North America / Mexico / Aguascalientes	Human	Female	45	Ambulatory	Nasopharyngeal swab
hCoV-19/Mexico/BCS_LANGEBIO_IMSS_0356/2021	EPI_ISL_1661979	23/03/21	North America / Mexico / Baja California Sur	Human	Male	47	Hospitalized	Nasopharyngeal swab
hCoV-19/Mexico/BCS_LANGEBIO_IMSS_0368/2021	EPI_ISL_1661980	22/03/21	North America / Mexico / Baja California Sur	Human	Female	31	Ambulatory	Nasopharyngeal swab
hCoV-19/Mexico/CHH_LANGEBIO_IMSS_0259/2021	EPI_ISL_1661983	24/03/21	North America / Mexico / Chihuahua	Human	Female	43	Ambulatory	Nasopharyngeal swab
hCoV-19/Mexico/CHH_LANGEBIO_IMSS_0280/2021	EPI_ISL_1661989	28/03/21	North America / Mexico / Chihuahua	Human	Male	40	Hospitalized	Nasopharyngeal swab
hCoV-19/Mexico/CMX_LANGEBIO_IMSS_0372/2021	EPI_ISL_1661991	22/03/21	North America / Mexico / Mexico City	Human	Female	25	Ambulatory	Nasopharyngeal swab
hCoV-19/Mexico/CMX_LANGEBIO_IMSS_0385/2021	EPI_ISL_1661993	23/03/21	North America / Mexico / Mexico City	Human	Female	72	Hospitalized	Pharyngeal swab
hCoV-19/Mexico/CMX_LANGEBIO_IMSS_0415/2021	EPI_ISL_1661995	24/03/21	North America / Mexico / Mexico City	Human	Male	95	Hospitalized	Nasopharyngeal swab
hCoV-19/Mexico/CMX_LANGEBIO_IMSS_0416/2021	EPI_ISL_1661996	23/03/21	North America / Mexico / Mexico City	Human	Male	50	Ambulatory	Nasopharyngeal swab
hCoV-19/Mexico/CMX_LANGEBIO_IMSS_0417/2021	EPI_ISL_1661997	21/03/21	North America / Mexico / Mexico City	Human	Male	70	Hospitalized	Nasopharyngeal swab
hCoV-19/Mexico/CMX_LANGEBIO_IMSS_0419/2021	EPI_ISL_1661998	24/03/21	North America / Mexico / Mexico City	Human	Male	63	Hospitalized	Nasopharyngeal swab

CoV-19/Mexico/CMX_LANGEBIO_IMSS_0421/202	EPI_ISL_1661999	23/03/21	North America / Mexico / Mexico City	Human	Male	65	Hospitalized	Pharyngeal swab
CoV-19/Mexico/CMX_LANGEBIO_IMSS_0432/202	EPI_ISL_1662001	24/03/21	North America / Mexico / Mexico City	Human	Female	22	Ambulatory	Nasopharyngeal swab
CoV-19/Mexico/CMX_LANGEBIO_IMSS_0433/202	EPI_ISL_1662002	25/03/21	North America / Mexico / Mexico City	Human	Male	73	Ambulatory	Nasopharyngeal swab
CoV-19/Mexico/CMX_LANGEBIO_IMSS_0434/202	EPI_ISL_1662003	24/03/21	North America / Mexico / Mexico City	Human	Male	65	Hospitalized	Nasopharyngeal swab
CoV-19/Mexico/CMX_LANGEBIO_IMSS_0435/202	EPI_ISL_1662004	25/03/21	North America / Mexico / Mexico City	Human	Male	93	Hospitalized	Pharyngeal swab
CoV-19/Mexico/CMX_LANGEBIO_IMSS_0436/202	EPI_ISL_1662005	25/03/21	North America / Mexico / Mexico City	Human	Male	55	Hospitalized	Pharyngeal swab
CoV-19/Mexico/CMX_LANGEBIO_IMSS_0437/202	EPI_ISL_1662006	25/03/21	North America / Mexico / Mexico City	Human	Male	68	Ambulatory	Pharyngeal swab
CoV-19/Mexico/CMX_LANGEBIO_IMSS_0446/202	EPI_ISL_1662007	26/03/21	North America / Mexico / Mexico City	Human	Male	67	Hospitalized	Nasopharyngeal swab
CoV-19/Mexico/CMX_LANGEBIO_IMSS_0449/202	EPI_ISL_1662008	25/03/21	North America / Mexico / Mexico City	Human	Male	80	Hospitalized	Nasopharyngeal swab
CoV-19/Mexico/CMX_LANGEBIO_IMSS_0450/202	EPI_ISL_1662009	25/03/21	North America / Mexico / Mexico City	Human	Female	79	Hospitalized	Nasopharyngeal swab
CoV-19/Mexico/CMX_LANGEBIO_IMSS_0453/202	EPI_ISL_1662010	26/03/21	North America / Mexico / Mexico City	Human	Female	51	Hospitalized	Nasopharyngeal swab
CoV-19/Mexico/CMX_LANGEBIO_IMSS_0454/202	EPI_ISL_1662011	26/03/21	North America / Mexico / Mexico City	Human	Male	70	Hospitalized	Pharyngeal swab
CoV-19/Mexico/CMX_LANGEBIO_IMSS_0455/202	EPI_ISL_1662012	25/03/21	North America / Mexico / Mexico City	Human	Male	59	Hospitalized	Pharyngeal swab
CoV-19/Mexico/CMX_LANGEBIO_IMSS_0456/202	EPI_ISL_1662013	26/03/21	North America / Mexico / Mexico City	Human	Male	81	Hospitalized	Pharyngeal swab
CoV-19/Mexico/CMX_LANGEBIO_IMSS_0459/202	EPI_ISL_1662014	25/03/21	North America / Mexico / Mexico City	Human	Male	0	Ambulatory	Pharyngeal swab
CoV-19/Mexico/CMX_LANGEBIO_IMSS_0469/202	EPI_ISL_1662015	28/03/21	North America / Mexico / Mexico City	Human	Female	24	Hospitalized	Nasopharyngeal swab
CoV-19/Mexico/CMX_LANGEBIO_IMSS_0470/202	EPI_ISL_1662016	26/03/21	North America / Mexico / Mexico City	Human	Male	30	Ambulatory	Pharyngeal swab
CoV-19/Mexico/CMX_LANGEBIO_IMSS_0473/202	EPI_ISL_1662017	27/03/21	North America / Mexico / Mexico City	Human	Female	36	Ambulatory	Nasopharyngeal swab
CoV-19/Mexico/CMX_LANGEBIO_IMSS_0474/202	EPI_ISL_1662018	27/03/21	North America / Mexico / Mexico City	Human	Male	83	Hospitalized	Pharyngeal swab
CoV-19/Mexico/CMX_LANGEBIO_IMSS_0475/202	EPI_ISL_1662019	26/03/21	North America / Mexico / Mexico City	Human	Female	66	Hospitalized	Pharyngeal swab
CoV-19/Mexico/CMX_LANGEBIO_IMSS_0476/202	EPI_ISL_1662020	28/03/21	North America / Mexico / Mexico City	Human	Female	71	Ambulatory	Pharyngeal swab
CoV-19/Mexico/CMX_LANGEBIO_IMSS_0477/202	EPI_ISL_1662021	28/03/21	North America / Mexico / Mexico City	Human	Male	67	Hospitalized	Pharyngeal swab
CoV-19/Mexico/CMX_LANGEBIO_IMSS_0480/202	EPI_ISL_1662022	26/03/21	North America / Mexico / Mexico City	Human	Male	20	Ambulatory	Nasopharyngeal swab
CoV-19/Mexico/CMX_LANGEBIO_IMSS_0489/202	EPI_ISL_1662024	01/04/21	North America / Mexico / Mexico City	Human	Male	43	Hospitalized	Pharyngeal swab
CoV-19/Mexico/CMX_LANGEBIO_IMSS_0490/202	EPI_ISL_1662025	23/03/21	North America / Mexico / Mexico City	Human	Female	66	Hospitalized	Pharyngeal swab
CoV-19/Mexico/CMX_LANGEBIO_IMSS_0491/202	EPI_ISL_1662026	31/03/21	North America / Mexico / Mexico City	Human	Male	45	Hospitalized	Nasopharyngeal swab
CoV-19/Mexico/CMX_LANGEBIO_IMSS_0492/202	EPI_ISL_1662027	02/04/21	North America / Mexico / Mexico City	Human	Male	56	Hospitalized	Nasopharyngeal swab
CoV-19/Mexico/CMX_LANGEBIO_IMSS_0494/202	EPI_ISL_1662028	01/04/21	North America / Mexico / Mexico City	Human	Male	66	Hospitalized	Pharyngeal swab
CoV-19/Mexico/CMX_LANGEBIO_IMSS_0497/202	EPI_ISL_1662029	03/04/21	North America / Mexico / Mexico City	Human	Male	82	Ambulatory	Pharyngeal swab
CoV-19/Mexico/CMX_LANGEBIO_IMSS_0499/202	EPI_ISL_1662031	01/04/21	North America / Mexico / Mexico City	Human	Male	89	Hospitalized	Nasopharyngeal swab
CoV-19/Mexico/CMX_LANGEBIO_IMSS_0500/202	EPI_ISL_1662032	01/04/21	North America / Mexico / Mexico City	Human	Female	58	Hospitalized	Nasopharyngeal swab
CoV-19/Mexico/CMX_LANGEBIO_IMSS_0501/202	EPI_ISL_1662033	04/04/21	North America / Mexico / Mexico City	Human	Male	37	Hospitalized	Nasopharyngeal swab
CoV-19/Mexico/COA_LANGEBIO_IMSS_0239/202	EPI_ISL_1662035	22/03/21	North America / Mexico / Coahuila	Human	Male	52	Ambulatory	Nasopharyngeal swab
CoV-19/Mexico/COA_LANGEBIO_IMSS_0255/202	EPI_ISL_1662039	25/03/21	North America / Mexico / Coahuila	Human	Male	55	Hospitalized	Nasopharyngeal swab
CoV-19/Mexico/COA_LANGEBIO_IMSS_0261/202	EPI_ISL_1662040	28/03/21	North America / Mexico / Coahuila	Human	Male	49	Hospitalized	Nasopharyngeal swab
CoV-19/Mexico/COA_LANGEBIO_IMSS_0269/202	EPI_ISL_1662042	30/03/21	North America / Mexico / Coahuila	Human	Female	45	Ambulatory	Nasopharyngeal swab
CoV-19/Mexico/COA_LANGEBIO_IMSS_0270/202	EPI_ISL_1662043	30/03/21	North America / Mexico / Coahuila	Human	Male	50	Hospitalized	Nasopharyngeal swab
CoV-19/Mexico/COA_LANGEBIO_IMSS_0272/202	EPI_ISL_1662044	29/03/21	North America / Mexico / Coahuila	Human	Female	53	Hospitalized	Nasopharyngeal swab
CoV-19/Mexico/COA_LANGEBIO_IMSS_0273/202	EPI_ISL_1662045	30/03/21	North America / Mexico / Coahuila	Human	Male	26	Ambulatory	Nasopharyngeal swab
CoV-19/Mexico/COA_LANGEBIO_IMSS_0274/202	EPI_ISL_1662046	31/03/21	North America / Mexico / Coahuila	Human	Female	23	Ambulatory	Nasopharyngeal swab
CoV-19/Mexico/COA_LANGEBIO_IMSS_0284/202	EPI_ISL_1662048	01/04/21	North America / Mexico / Coahuila	Human	Male	44	Hospitalized	Nasopharyngeal swab
CoV-19/Mexico/DUR_LANGEBIO_IMSS_0248/202	EPI_ISL_1662049	24/03/21	North America / Mexico / Durango	Human	Male	30	Ambulatory	Nasopharyngeal swab
CoV-19/Mexico/DUR_LANGEBIO_IMSS_0249/202	EPI_ISL_1662050	23/03/21	North America / Mexico / Durango	Human	Male	73	Hospitalized	Nasopharyngeal swab
CoV-19/Mexico/DUR_LANGEBIO_IMSS_0256/202	EPI_ISL_1662051	26/03/21	North America / Mexico / Durango	Human	Male	45	Hospitalized	Nasopharyngeal swab

CoV-19/Mexico/DUR_LANGEBIO_IMSS_0258/202	EPI_ISL_1662053	26/03/21	North America / Mexico / Durango	Human	Male	23	Ambulatory	Nasopharyngeal swab
CoV-19/Mexico/DUR_LANGEBIO_IMSS_0265/202	EPI_ISL_1662055	29/03/21	North America / Mexico / Durango	Human	Male	19	Ambulatory	Nasopharyngeal swab
CoV-19/Mexico/DUR_LANGEBIO_IMSS_0266/202	EPI_ISL_1662056	29/03/21	North America / Mexico / Durango	Human	Female	40	Ambulatory	Nasopharyngeal swab
CoV-19/Mexico/GRO_LANGEBIO_IMSS_0424/202	EPI_ISL_1662058	21/03/21	North America / Mexico / Guerrero	Human	Female	66	Hospitalized	Nasopharyngeal swab
CoV-19/Mexico/GRO_LANGEBIO_IMSS_0425/202	EPI_ISL_1662059	22/03/21	North America / Mexico / Guerrero	Human	Female	68	Hospitalized	Nasopharyngeal swab
CoV-19/Mexico/GRO_LANGEBIO_IMSS_0444/202	EPI_ISL_1662060	23/03/21	North America / Mexico / Guerrero	Human	Male	84	Ambulatory	Nasopharyngeal swab
CoV-19/Mexico/GRO_LANGEBIO_IMSS_0485/202	EPI_ISL_1662061	28/03/21	North America / Mexico / Guerrero	Human	Male	52	Hospitalized	Pharyngeal swab
CoV-19/Mexico/GUA_LANGEBIO_IMSS_0308/202	EPI_ISL_1662062	08/03/21	North America / Mexico / Guanajuato	Human	Female	41	Ambulatory	Nasopharyngeal swab
CoV-19/Mexico/GUA_LANGEBIO_IMSS_0309/202	EPI_ISL_1662063	10/03/21	North America / Mexico / Guanajuato	Human	Male	66	Ambulatory	Nasopharyngeal swab
CoV-19/Mexico/GUA_LANGEBIO_IMSS_0310/202	EPI_ISL_1662064	09/03/21	North America / Mexico / Guanajuato	Human	Female	42	Ambulatory	Nasopharyngeal swab
CoV-19/Mexico/GUA_LANGEBIO_IMSS_0318/202	EPI_ISL_1662065	19/03/21	North America / Mexico / Guanajuato	Human	Male	72	Ambulatory	Nasopharyngeal swab
CoV-19/Mexico/GUA_LANGEBIO_IMSS_0321/202	EPI_ISL_1662066	18/03/21	North America / Mexico / Guanajuato	Human	Female	37	Ambulatory	Nasopharyngeal swab
CoV-19/Mexico/GUA_LANGEBIO_IMSS_0332/202	EPI_ISL_1662067	22/03/21	North America / Mexico / Guanajuato	Human	Male	56	Hospitalized	Nasopharyngeal swab
CoV-19/Mexico/GUA_LANGEBIO_IMSS_0344/202	EPI_ISL_1662068	23/03/21	North America / Mexico / Guanajuato	Human	Female	81	Ambulatory	Nasopharyngeal swab
CoV-19/Mexico/GUA_LANGEBIO_IMSS_0359/202	EPI_ISL_1662069	26/03/21	North America / Mexico / Guanajuato	Human	Female	31	Ambulatory	Nasopharyngeal swab
hCoV-19/Mexico/HID_LANGEBIO_IMSS_0389/2021	EPI_ISL_1662070	22/03/21	North America / Mexico / Hidalgo	Human	Male	34	Hospitalized	Pharyngeal swab
hCoV-19/Mexico/HID_LANGEBIO_IMSS_0452/2021	EPI_ISL_1662071	26/03/21	North America / Mexico / Hidalgo	Human	Female	45	Ambulatory	Pharyngeal swab
hCoV-19/Mexico/HID_LANGEBIO_IMSS_0468/2021	EPI_ISL_1662072	10/03/21	North America / Mexico / Hidalgo	Human	Male	61	Ambulatory	Pharyngeal swab
hCoV-19/Mexico/HID_LANGEBIO_IMSS_0472/2021	EPI_ISL_1662073	26/03/21	North America / Mexico / Hidalgo	Human	Female	64	Hospitalized	Nasopharyngeal swab
hCoV-19/Mexico/HID_LANGEBIO_IMSS_0493/2021	EPI_ISL_1662074	01/04/21	North America / Mexico / Hidalgo	Human	Male	25	Ambulatory	Nasopharyngeal swab
hCoV-19/Mexico/JAL_LANGEBIO_IMSS_0314/2021	EPI_ISL_1662075	13/03/21	North America / Mexico / Jalisco	Human	Female	49	Hospitalized	Nasopharyngeal swab
hCoV-19/Mexico/JAL_LANGEBIO_IMSS_0315/2021	EPI_ISL_1662076	14/03/21	North America / Mexico / Jalisco	Human	Female	54	Hospitalized	Nasopharyngeal swab
hCoV-19/Mexico/JAL_LANGEBIO_IMSS_0319/2021	EPI_ISL_1662078	22/03/21	North America / Mexico / Jalisco	Human	Female	70	Hospitalized	Pharyngeal swab
hCoV-19/Mexico/JAL_LANGEBIO_IMSS_0326/2021	EPI_ISL_1662081	23/03/21	North America / Mexico / Jalisco	Human	Female	42	Ambulatory	Nasopharyngeal swab
hCoV-19/Mexico/JAL_LANGEBIO_IMSS_0328/2021	EPI_ISL_1662083	23/03/21	North America / Mexico / Jalisco	Human	Female	56	Hospitalized	Nasopharyngeal swab
hCoV-19/Mexico/JAL_LANGEBIO_IMSS_0329/2021	EPI_ISL_1662084	23/03/21	North America / Mexico / Jalisco	Human	Female	24	Ambulatory	Nasopharyngeal swab
hCoV-19/Mexico/JAL_LANGEBIO_IMSS_0330/2021	EPI_ISL_1662085	23/03/21	North America / Mexico / Jalisco	Human	Female	97	Hospitalized	Nasopharyngeal swab
hCoV-19/Mexico/JAL_LANGEBIO_IMSS_0334/2021	EPI_ISL_1662087	23/03/21	North America / Mexico / Jalisco	Human	Male	54	Ambulatory	Nasopharyngeal swab
hCoV-19/Mexico/JAL_LANGEBIO_IMSS_0342/2021	EPI_ISL_1662088	25/03/21	North America / Mexico / Jalisco	Human	Female	51	Ambulatory	Nasopharyngeal swab
hCoV-19/Mexico/JAL_LANGEBIO_IMSS_0343/2021	EPI_ISL_1662089	25/03/21	North America / Mexico / Jalisco	Human	Female	35	Ambulatory	Nasopharyngeal swab
hCoV-19/Mexico/JAL_LANGEBIO_IMSS_0345/2021	EPI_ISL_1662090	24/03/21	North America / Mexico / Jalisco	Human	Male	71	Hospitalized	Nasopharyngeal swab
hCoV-19/Mexico/JAL_LANGEBIO_IMSS_0347/2021	EPI_ISL_1662092	25/03/21	North America / Mexico / Jalisco	Human	Female	24	Ambulatory	Nasopharyngeal swab
hCoV-19/Mexico/JAL_LANGEBIO_IMSS_0348/2021	EPI_ISL_1662093	25/03/21	North America / Mexico / Jalisco	Human	Male	39	Hospitalized	Nasopharyngeal swab
hCoV-19/Mexico/JAL_LANGEBIO_IMSS_0349/2021	EPI_ISL_1662094	25/03/21	North America / Mexico / Jalisco	Human	Male	75	Hospitalized	Pharyngeal swab
hCoV-19/Mexico/JAL_LANGEBIO_IMSS_0350/2021	EPI_ISL_1662095	25/03/21	North America / Mexico / Jalisco	Human	Female	71	Hospitalized	Pharyngeal swab
hCoV-19/Mexico/JAL_LANGEBIO_IMSS_0354/2021	EPI_ISL_1662096	25/03/21	North America / Mexico / Jalisco	Human	Male	76	Hospitalized	Nasopharyngeal swab
hCoV-19/Mexico/JAL_LANGEBIO_IMSS_0358/2021	EPI_ISL_1662097	26/03/21	North America / Mexico / Jalisco	Human	Male	44	Ambulatory	Nasopharyngeal swab
hCoV-19/Mexico/JAL_LANGEBIO_IMSS_0360/2021	EPI_ISL_1662098	27/03/21	North America / Mexico / Jalisco	Human	Male	88	Hospitalized	Pharyngeal swab
hCoV-19/Mexico/JAL_LANGEBIO_IMSS_0364/2021	EPI_ISL_1662100	29/03/21	North America / Mexico / Jalisco	Human	Female	30	Ambulatory	Nasopharyngeal swab
hCoV-19/Mexico/JAL_LANGEBIO_IMSS_0367/2021	EPI_ISL_1662103	29/03/21	North America / Mexico / Jalisco	Human	Male	59	Hospitalized	Nasopharyngeal swab
CoV-19/Mexico/MEX_LANGEBIO_IMSS_0373/202	EPI_ISL_1662104	22/03/21	North America / Mexico / State of Mexico	Human	Female	39	Ambulatory	Nasopharyngeal swab
CoV-19/Mexico/MEX_LANGEBIO_IMSS_0375/202	EPI_ISL_1662105	23/03/21	North America / Mexico / State of Mexico	Human	Male	21	Ambulatory	Nasopharyngeal swab
CoV-19/Mexico/MEX_LANGEBIO_IMSS_0377/202	EPI_ISL_1662106	22/03/21	North America / Mexico / State of Mexico	Human	Female	73	Hospitalized	Nasopharyngeal swab
CoV-19/Mexico/MEX_LANGEBIO_IMSS_0379/202	EPI_ISL_1662107	22/03/21	North America / Mexico / State of Mexico	Human	Male	64	Hospitalized	Nasopharyngeal swab

hCoV-19/Mexico/MEX_LANGEBIO_IMSS_0418/2021	EPI_ISL_1662108	23/03/21	North America / Mexico / State of Mexico	Human	Female	53	Hospitalized	Nasopharyngeal swab
hCoV-19/Mexico/MEX_LANGEBIO_IMSS_0422/2021	EPI_ISL_1662109	22/03/21	North America / Mexico / State of Mexico	Human	Male	86	Hospitalized	Nasopharyngeal swab
hCoV-19/Mexico/MEX_LANGEBIO_IMSS_0423/2021	EPI_ISL_1662110	22/03/21	North America / Mexico / State of Mexico	Human	Male	41	Hospitalized	Nasopharyngeal swab
hCoV-19/Mexico/MEX_LANGEBIO_IMSS_0426/2021	EPI_ISL_1662111	23/03/21	North America / Mexico / State of Mexico	Human	Male	46	Ambulatory	Nasopharyngeal swab
hCoV-19/Mexico/MEX_LANGEBIO_IMSS_0445/2021	EPI_ISL_1662112	26/03/21	North America / Mexico / State of Mexico	Human	Male	67	Hospitalized	Nasopharyngeal swab
hCoV-19/Mexico/MEX_LANGEBIO_IMSS_0447/2021	EPI_ISL_1662113	25/03/21	North America / Mexico / State of Mexico	Human	Male	52	Hospitalized	Nasopharyngeal swab
hCoV-19/Mexico/MEX_LANGEBIO_IMSS_0448/2021	EPI_ISL_1662114	24/03/21	North America / Mexico / State of Mexico	Human	Male	61	Hospitalized	Nasopharyngeal swab
hCoV-19/Mexico/MEX_LANGEBIO_IMSS_0451/2021	EPI_ISL_1662115	25/03/21	North America / Mexico / State of Mexico	Human	Female	71	Ambulatory	Nasopharyngeal swab
hCoV-19/Mexico/MEX_LANGEBIO_IMSS_0457/2021	EPI_ISL_1662116	25/03/21	North America / Mexico / State of Mexico	Human	Female	73	Hospitalized	Pharyngeal swab
hCoV-19/Mexico/MEX_LANGEBIO_IMSS_0478/2021	EPI_ISL_1662117	28/03/21	North America / Mexico / State of Mexico	Human	Female	51	Hospitalized	Nasopharyngeal swab
hCoV-19/Mexico/MEX_LANGEBIO_IMSS_0481/2021	EPI_ISL_1662118	26/03/21	North America / Mexico / State of Mexico	Human	Male	39	Hospitalized	Nasopharyngeal swab
hCoV-19/Mexico/MEX_LANGEBIO_IMSS_0483/2021	EPI_ISL_1662119	28/03/21	North America / Mexico / State of Mexico	Human	Male	64	Ambulatory	Nasopharyngeal swab
hCoV-19/Mexico/MEX_LANGEBIO_IMSS_0488/2021	EPI_ISL_1662120	31/03/21	North America / Mexico / State of Mexico	Human	Male	51	Hospitalized	Nasopharyngeal swab
hCoV-19/Mexico/MEX_LANGEBIO_IMSS_0496/2021	EPI_ISL_1662121	02/04/21	North America / Mexico / State of Mexico	Human	Male	50	Ambulatory	Nasopharyngeal swab
hCoV-19/Mexico/MEX_LANGEBIO_IMSS_0502/2021	EPI_ISL_1662122	31/03/21	North America / Mexico / State of Mexico	Human	Female	76	Ambulatory	Nasopharyngeal swab
hCoV-19/Mexico/MIC_LANGEBIO_IMSS_0323/2021	EPI_ISL_1662123	16/03/21	North America / Mexico / Michoacan	Human	Female	36	Ambulatory	Nasopharyngeal swab
hCoV-19/Mexico/MIC_LANGEBIO_IMSS_0331/2021	EPI_ISL_1662124	21/03/21	North America / Mexico / Michoacan	Human	Male	36	Hospitalized	Nasopharyngeal swab
hCoV-19/Mexico/MOR_LANGEBIO_IMSS_0380/2021	EPI_ISL_1662128	20/03/21	North America / Mexico / Morelos	Human	Male	52	Hospitalized	Nasopharyngeal swab
hCoV-19/Mexico/MOR_LANGEBIO_IMSS_0381/2021	EPI_ISL_1662129	21/03/21	North America / Mexico / Morelos	Human	Male	80	Hospitalized	Nasopharyngeal swab
hCoV-19/Mexico/MOR_LANGEBIO_IMSS_0471/2021	EPI_ISL_1662130	25/03/21	North America / Mexico / Morelos	Human	Male	64	Hospitalized	Nasopharyngeal swab
hCoV-19/Mexico/NAY_LANGEBIO_IMSS_0335/2021	EPI_ISL_1662132	24/03/21	North America / Mexico / Nayarit	Human	Male	56	Hospitalized	Nasopharyngeal swab
hCoV-19/Mexico/NLE_LANGEBIO_IMSS_0241/2021	EPI_ISL_1662133	24/03/21	North America / Mexico / Nuevo Leon	Human	Female	56	Ambulatory	Nasopharyngeal swab
hCoV-19/Mexico/NLE_LANGEBIO_IMSS_0244/2021	EPI_ISL_1662136	23/03/21	North America / Mexico / Nuevo Leon	Human	Female	80	Hospitalized	Nasopharyngeal swab
hCoV-19/Mexico/NLE_LANGEBIO_IMSS_0245/2021	EPI_ISL_1662137	24/03/21	North America / Mexico / Nuevo Leon	Human	Male	58	Ambulatory	Nasopharyngeal swab
hCoV-19/Mexico/NLE_LANGEBIO_IMSS_0246/2021	EPI_ISL_1662138	24/03/21	North America / Mexico / Nuevo Leon	Human	Male	71	Hospitalized	Nasopharyngeal swab
hCoV-19/Mexico/NLE_LANGEBIO_IMSS_0252/2021	EPI_ISL_1662141	25/03/21	North America / Mexico / Nuevo Leon	Human	Female	22	Ambulatory	Nasopharyngeal swab
hCoV-19/Mexico/NLE_LANGEBIO_IMSS_0260/2021	EPI_ISL_1662142	26/03/21	North America / Mexico / Nuevo Leon	Human	Female	29	Ambulatory	Nasopharyngeal swab
hCoV-19/Mexico/NLE_LANGEBIO_IMSS_0263/2021	EPI_ISL_1662143	30/03/21	North America / Mexico / Nuevo Leon	Human	Female	44	Hospitalized	Nasopharyngeal swab
hCoV-19/Mexico/OAX_LANGEBIO_IMSS_0382/2021	EPI_ISL_1662145	20/03/21	North America / Mexico / Oaxaca	Human	Female	22	Ambulatory	Nasopharyngeal swab
hCoV-19/Mexico/OAX_LANGEBIO_IMSS_0383/2021	EPI_ISL_1662146	20/03/21	North America / Mexico / Oaxaca	Human	Male	49	Ambulatory	Nasopharyngeal swab
hCoV-19/Mexico/OAX_LANGEBIO_IMSS_0461/2021	EPI_ISL_1662147	22/03/21	North America / Mexico / Oaxaca	Human	Male	57	Ambulatory	Nasopharyngeal swab
hCoV-19/Mexico/PUE_LANGEBIO_IMSS_0369/2021	EPI_ISL_1662149	18/03/21	North America / Mexico / Puebla	Human	Male	38	Ambulatory	Nasopharyngeal swab
hCoV-19/Mexico/PUE_LANGEBIO_IMSS_0370/2021	EPI_ISL_1662150	18/03/21	North America / Mexico / Puebla	Human	Male	59	Ambulatory	Nasopharyngeal swab
hCoV-19/Mexico/PUE_LANGEBIO_IMSS_0390/2021	EPI_ISL_1662151	19/03/21	North America / Mexico / Puebla	Human	Male	45	Ambulatory	Pharyngeal swab
hCoV-19/Mexico/PUE_LANGEBIO_IMSS_0391/2021	EPI_ISL_1662152	19/03/21	North America / Mexico / Puebla	Human	Male	40	Ambulatory	Pharyngeal swab
hCoV-19/Mexico/PUE_LANGEBIO_IMSS_0393/2021	EPI_ISL_1662153	19/03/21	North America / Mexico / Puebla	Human	Male	30	Ambulatory	Pharyngeal swab
hCoV-19/Mexico/PUE_LANGEBIO_IMSS_0394/2021	EPI_ISL_1662154	19/03/21	North America / Mexico / Puebla	Human	Male	49	Ambulatory	Pharyngeal swab
hCoV-19/Mexico/PUE_LANGEBIO_IMSS_0396/2021	EPI_ISL_1662155	22/03/21	North America / Mexico / Puebla	Human	Male	53	Ambulatory	Nasopharyngeal swab
hCoV-19/Mexico/PUE_LANGEBIO_IMSS_0397/2021	EPI_ISL_1662156	22/03/21	North America / Mexico / Puebla	Human	Female	84	Hospitalized	Nasopharyngeal swab
hCoV-19/Mexico/PUE_LANGEBIO_IMSS_0399/2021	EPI_ISL_1662158	21/03/21	North America / Mexico / Puebla	Human	Male	47	Hospitalized	Nasopharyngeal swab
hCoV-19/Mexico/PUE_LANGEBIO_IMSS_0400/2021	EPI_ISL_1662159	21/03/21	North America / Mexico / Puebla	Human	Female	37	Hospitalized	Nasopharyngeal swab
hCoV-19/Mexico/PUE_LANGEBIO_IMSS_0401/2021	EPI_ISL_1662160	22/03/21	North America / Mexico / Puebla	Human	Male	79	Hospitalized	Nasopharyngeal swab
hCoV-19/Mexico/PUE_LANGEBIO_IMSS_0402/2021	EPI_ISL_1662161	19/03/21	North America / Mexico / Puebla	Human	Male	72	Hospitalized	Nasopharyngeal swab
hCoV-19/Mexico/PUE_LANGEBIO_IMSS_0403/2021	EPI_ISL_1662162	19/03/21	North America / Mexico / Puebla	Human	Male	49	Hospitalized	Nasopharyngeal swab

hCoV-19/Mexico/PUE_LANGEBIO_IMSS_0405/202	EPI_ISL_1662164	22/03/21	North America / Mexico / Puebla	Human	Male	51	Ambulatory	Nasopharyngeal swab
hCoV-19/Mexico/PUE_LANGEBIO_IMSS_0406/202	EPI_ISL_1662165	22/03/21	North America / Mexico / Puebla	Human	Male	41	Ambulatory	Nasopharyngeal swab
hCoV-19/Mexico/PUE_LANGEBIO_IMSS_0407/202	EPI_ISL_1662166	21/03/21	North America / Mexico / Puebla	Human	Female	76	Ambulatory	Nasopharyngeal swab
hCoV-19/Mexico/PUE_LANGEBIO_IMSS_0408/202	EPI_ISL_1662167	21/03/21	North America / Mexico / Puebla	Human	Male	74	Ambulatory	Nasopharyngeal swab
hCoV-19/Mexico/PUE_LANGEBIO_IMSS_0409/202	EPI_ISL_1662168	20/03/21	North America / Mexico / Puebla	Human	Female	23	Ambulatory	Pharyngeal swab
hCoV-19/Mexico/PUE_LANGEBIO_IMSS_0410/202	EPI_ISL_1662169	19/03/21	North America / Mexico / Puebla	Human	Female	57	Ambulatory	Nasopharyngeal swab
hCoV-19/Mexico/PUE_LANGEBIO_IMSS_0439/202	EPI_ISL_1662171	23/03/21	North America / Mexico / Puebla	Human	Male	22	Ambulatory	Nasopharyngeal swab
hCoV-19/Mexico/PUE_LANGEBIO_IMSS_0441/202	EPI_ISL_1662173	23/03/21	North America / Mexico / Puebla	Human	Male	36	Ambulatory	Nasopharyngeal swab
hCoV-19/Mexico/PUE_LANGEBIO_IMSS_0442/202	EPI_ISL_1662174	23/03/21	North America / Mexico / Puebla	Human	Male	38	Ambulatory	Nasopharyngeal swab
hCoV-19/Mexico/PUE_LANGEBIO_IMSS_0463/202	EPI_ISL_1662175	25/03/21	North America / Mexico / Puebla	Human	Female	27	Ambulatory	Nasopharyngeal swab
hCoV-19/Mexico/PUE_LANGEBIO_IMSS_0464/202	EPI_ISL_1662176	25/03/21	North America / Mexico / Puebla	Human	Male	33	Ambulatory	Nasopharyngeal swab
hCoV-19/Mexico/PUE_LANGEBIO_IMSS_0465/202	EPI_ISL_1662177	25/03/21	North America / Mexico / Puebla	Human	Female	66	Ambulatory	Nasopharyngeal swab
hCoV-19/Mexico/PUE_LANGEBIO_IMSS_0466/202	EPI_ISL_1662178	24/03/21	North America / Mexico / Puebla	Human	Male	16	Ambulatory	Nasopharyngeal swab
hCoV-19/Mexico/PUE_LANGEBIO_IMSS_0484/202	EPI_ISL_1662179	30/03/21	North America / Mexico / Puebla	Human	Male	41	Ambulatory	Nasopharyngeal swab
hCoV-19/Mexico/PUE_LANGEBIO_IMSS_0486/202	EPI_ISL_1662180	29/03/21	North America / Mexico / Puebla	Human	Female	45	Ambulatory	Nasopharyngeal swab
hCoV-19/Mexico/QUE_LANGEBIO_IMSS_0420/202	EPI_ISL_1662181	22/03/21	North America / Mexico / Queretaro	Human	Male	46	Hospitalized	Nasopharyngeal swab
hCoV-19/Mexico/QUE_LANGEBIO_IMSS_0458/202	EPI_ISL_1662182	24/03/21	North America / Mexico / Queretaro	Human	Male	31	Hospitalized	Nasopharyngeal swab
hCoV-19/Mexico/QUE_LANGEBIO_IMSS_0495/202	EPI_ISL_1662183	30/03/21	North America / Mexico / Queretaro	Human	Female	34	Ambulatory	Nasopharyngeal swab
hCoV-19/Mexico/ROO_LANGEBIO_IMSS_0286/202	EPI_ISL_1662184	11/03/21	North America / Mexico / Quintana Roo	Human	Male	54	Hospitalized	Nasopharyngeal swab
hCoV-19/Mexico/SIN_LANGEBIO_IMSS_0311/2021	EPI_ISL_1662186	09/03/21	North America / Mexico / Sinaloa	Human	Female	77	Hospitalized	Nasopharyngeal swab
hCoV-19/Mexico/SIN_LANGEBIO_IMSS_0322/2021	EPI_ISL_1662187	21/03/21	North America / Mexico / Sinaloa	Human	Male	64	Hospitalized	Nasopharyngeal swab
hCoV-19/Mexico/SIN_LANGEBIO_IMSS_0337/2021	EPI_ISL_1662189	23/03/21	North America / Mexico / Sinaloa	Human	Male	65	Hospitalized	Nasopharyngeal swab
hCoV-19/Mexico/SIN_LANGEBIO_IMSS_0338/2021	EPI_ISL_1662190	23/03/21	North America / Mexico / Sinaloa	Human	Female	71	Hospitalized	Nasopharyngeal swab
hCoV-19/Mexico/SIN_LANGEBIO_IMSS_0340/2021	EPI_ISL_1662192	22/03/21	North America / Mexico / Sinaloa	Human	Female	60	Hospitalized	Nasopharyngeal swab
hCoV-19/Mexico/SIN_LANGEBIO_IMSS_0341/2021	EPI_ISL_1662193	19/03/21	North America / Mexico / Sinaloa	Human	Male	28	Ambulatory	Nasopharyngeal swab
hCoV-19/Mexico/SLP_LANGEBIO_IMSS_0387/202	EPI_ISL_1662194	22/03/21	North America / Mexico / San Luis Potosi	Human	Female	60	Hospitalized	Nasopharyngeal swab
hCoV-19/Mexico/SLP_LANGEBIO_IMSS_0443/202	EPI_ISL_1662195	24/03/21	North America / Mexico / San Luis Potosi	Human	Female	20	Ambulatory	Nasopharyngeal swab
hCoV-19/Mexico/SLP_LANGEBIO_IMSS_0479/202	EPI_ISL_1662196	27/03/21	North America / Mexico / San Luis Potosi	Human	Female	38	Ambulatory	Nasopharyngeal swab
hCoV-19/Mexico/SON_LANGEBIO_IMSS_0355/202	EPI_ISL_1662197	23/03/21	North America / Mexico / Sonora	Human	Female	40	Ambulatory	Nasopharyngeal swab
hCoV-19/Mexico/SON_LANGEBIO_IMSS_0363/202	EPI_ISL_1662198	25/03/21	North America / Mexico / Sonora	Human	Female	38	Ambulatory	Nasopharyngeal swab
hCoV-19/Mexico/TAM_LANGEBIO_IMSS_0271/202	EPI_ISL_1662199	29/03/21	North America / Mexico / Tamaulipas	Human	Female	57	Ambulatory	Nasopharyngeal swab
hCoV-19/Mexico/TAM_LANGEBIO_IMSS_0282/202	EPI_ISL_1662200	30/03/21	North America / Mexico / Tamaulipas	Human	Female	22	Ambulatory	Nasopharyngeal swab
hCoV-19/Mexico/TLA_LANGEBIO_IMSS_0371/2021	EPI_ISL_1662201	20/03/21	North America / Mexico / Tlaxcala	Human	Female	77	Ambulatory	Nasopharyngeal swab
hCoV-19/Mexico/VER_LANGEBIO_IMSS_0376/202	EPI_ISL_1662202	21/03/21	North America / Mexico / Veracruz	Human	Female	60	Hospitalized	Nasopharyngeal swab
hCoV-19/Mexico/VER_LANGEBIO_IMSS_0384/202	EPI_ISL_1662203	21/03/21	North America / Mexico / Veracruz	Human	Male	67	Hospitalized	Nasopharyngeal swab
hCoV-19/Mexico/VER_LANGEBIO_IMSS_0386/202	EPI_ISL_1662204	18/03/21	North America / Mexico / Veracruz	Human	Female	45	Ambulatory	Nasopharyngeal swab
hCoV-19/Mexico/VER_LANGEBIO_IMSS_0411/202	EPI_ISL_1662205	22/03/21	North America / Mexico / Veracruz	Human	Male	25	Hospitalized	Bronchoalveolar lavage
hCoV-19/Mexico/VER_LANGEBIO_IMSS_0412/202	EPI_ISL_1662206	22/03/21	North America / Mexico / Veracruz	Human	Male	48	Ambulatory	Nasopharyngeal swab
hCoV-19/Mexico/VER_LANGEBIO_IMSS_0413/202	EPI_ISL_1662207	23/03/21	North America / Mexico / Veracruz	Human	Male	25	Ambulatory	Nasopharyngeal swab
hCoV-19/Mexico/VER_LANGEBIO_IMSS_0414/202	EPI_ISL_1662208	23/03/21	North America / Mexico / Veracruz	Human	Male	73	Hospitalized	Bronchoalveolar lavage
hCoV-19/Mexico/VER_LANGEBIO_IMSS_0427/202	EPI_ISL_1662209	23/03/21	North America / Mexico / Veracruz	Human	Male	35	Ambulatory	Nasopharyngeal swab
hCoV-19/Mexico/VER_LANGEBIO_IMSS_0430/202	EPI_ISL_1662212	23/03/21	North America / Mexico / Veracruz	Human	Male	11	Ambulatory	Nasopharyngeal swab
hCoV-19/Mexico/YUC_LANGEBIO_IMSS_0287/202	EPI_ISL_1662214	22/03/21	North America / Mexico / Yucatan	Human	Male	60	Ambulatory	Nasopharyngeal swab
hCoV-19/Mexico/YUC_LANGEBIO_IMSS_0288/202	EPI_ISL_1662215	26/03/21	North America / Mexico / Yucatan	Human	Female	51	Hospitalized	Nasopharyngeal swab

hCoV-19/Mexico/YUC_LANGEBIO_IMSS_0289/2021	EPI_ISL_1662216	25/03/21	North America / Mexico / Yucatan	Human	Male	73	Hospitalized	Nasopharyngeal swab
hCoV-19/Mexico/YUC_LANGEBIO_IMSS_0290/2021	EPI_ISL_1662217	26/03/21	North America / Mexico / Yucatan	Human	Female	86	Hospitalized	Nasopharyngeal swab
hCoV-19/Mexico/YUC_LANGEBIO_IMSS_0291/2021	EPI_ISL_1662218	29/03/21	North America / Mexico / Yucatan	Human	Male	75	Ambulatory	Nasopharyngeal swab
hCoV-19/Mexico/YUC_LANGEBIO_IMSS_0293/2021	EPI_ISL_1662219	26/03/21	North America / Mexico / Yucatan	Human	Male	60	Hospitalized	Nasopharyngeal swab
hCoV-19/Mexico/YUC_LANGEBIO_IMSS_0294/2021	EPI_ISL_1662220	30/03/21	North America / Mexico / Yucatan	Human	Male	76	Hospitalized	Nasopharyngeal swab
hCoV-19/Mexico/ZAC_LANGEBIO_IMSS_0295/2021	EPI_ISL_1662221	22/03/21	North America / Mexico / Zacatecas	Human	Female	66	Hospitalized	Nasopharyngeal swab
hCoV-19/Mexico/ZAC_LANGEBIO_IMSS_0296/2021	EPI_ISL_1662222	22/03/21	North America / Mexico / Zacatecas	Human	Female	22	Hospitalized	Nasopharyngeal swab
hCoV-19/Mexico/ZAC_LANGEBIO_IMSS_0297/2021	EPI_ISL_1662223	22/03/21	North America / Mexico / Zacatecas	Human	Female	22	Ambulatory	Nasopharyngeal swab
hCoV-19/Mexico/ZAC_LANGEBIO_IMSS_0303/2021	EPI_ISL_1662228	29/03/21	North America / Mexico / Zacatecas	Human	Male	77	Hospitalized	Nasopharyngeal swab
hCoV-19/Mexico/ZAC_LANGEBIO_IMSS_0304/2021	EPI_ISL_1662229	29/03/21	North America / Mexico / Zacatecas	Human	Female	32	Ambulatory	Nasopharyngeal swab
hCoV-19/Mexico/ZAC_LANGEBIO_IMSS_0306/2021	EPI_ISL_1662231	02/04/21	North America / Mexico / Zacatecas	Human	Female	43	Ambulatory	Nasopharyngeal swab
hCoV-19/Mexico/ZAC_LANGEBIO_IMSS_0307/2021	EPI_ISL_1662232	01/04/21	North America / Mexico / Zacatecas	Human	Male	50	Hospitalized	Nasopharyngeal swab
hCoV-19/Mexico/CMX-INER-SC480/2021	EPI_ISL_1672849	17/02/21	North America / Mexico / Mexico City	Human	Male	86	Hospitalized	
hCoV-19/Mexico/CMX-INER-SC483/2021	EPI_ISL_1672864	18/02/21	North America / Mexico / Mexico City	Human	Male	62	Hospitalized	Nasopharyngeal swab
hCoV-19/Mexico/CMX-INER-SC486/2021	EPI_ISL_1672865	18/02/21	North America / Mexico / Mexico City	Human	Male	57	Deceased	Nasopharyngeal swab
hCoV-19/Mexico/CMX-INER-SC498/2021	EPI_ISL_1672866	19/02/21	North America / Mexico / Mexico City	Human	Female	67	Hospitalized	Nasopharyngeal swab
hCoV-19/Mexico/CHH-InDRE_F12367_S1444/2021	EPI_ISL_1673267	23/03/21	North America / Mexico / Chihuahua	Human	Male	37	Live	Oropharyngeal swab
hCoV-19/Mexico/CHH-InDRE_F12371_S1445/2021	EPI_ISL_1673268	22/03/21	North America / Mexico / Chihuahua	Human	Male	90	Released	Oropharyngeal swab
hCoV-19/Mexico/GUA-InDRE_F12427_S1446/2021	EPI_ISL_1673269	15/03/21	North America / Mexico / Guanajuato	Human	Female	49	Deceased	Oropharyngeal swab
hCoV-19/Mexico/QUE-InDRE_F12490_S1454/2021	EPI_ISL_1673274	27/03/21	North America / Mexico / Queretaro	Human	Female	63	Hospitalized	Oropharyngeal swab
hCoV-19/Mexico/CHP-InDRE_F11919_S1406/2021	EPI_ISL_1700795	18/03/21	North America / Mexico / Chiapas	Human	Female	76	Hospitalized	Oropharyngeal swab
hCoV-19/Mexico/NLE-InDRE_F11937_S1409/2021	EPI_ISL_1700798	15/03/21	North America / Mexico / Nuevo Leon	Human	Male	69	Hospitalized	Oropharyngeal swab
hCoV-19/Mexico/JAL-InDRE_F11989_S1411/2021	EPI_ISL_1700800	21/03/21	North America / Mexico / Jalisco	Human	Male	84	Deceased	Oropharyngeal swab
hCoV-19/Mexico/QUE-InDRE_F12125_S1415/2021	EPI_ISL_1700803	16/03/21	North America / Mexico / Queretaro	Human	Female	63	Released	Oropharyngeal swab
hCoV-19/Mexico/QUE-InDRE_F12131_S1416/2021	EPI_ISL_1700804	19/03/21	North America / Mexico / Queretaro	Human	Female	45	Released	Oropharyngeal swab
hCoV-19/Mexico/QUE-InDRE_F12141_S1418/2021	EPI_ISL_1700805	21/03/21	North America / Mexico / Queretaro	Human	Male	49	Released	Oropharyngeal swab
hCoV-19/Mexico/QUE-InDRE_F12146_S1419/2021	EPI_ISL_1700806	22/03/21	North America / Mexico / Queretaro	Human	Male	43	Released	Oropharyngeal swab
hCoV-19/Mexico/GRO-InDRE_F12175_S1423/2021	EPI_ISL_1700810	14/03/21	North America / Mexico / Guerrero	Human	Male	85	Deceased	Oropharyngeal swab
hCoV-19/Mexico/VER-InDRE_F12180_S1425/2021	EPI_ISL_1700811	14/03/21	North America / Mexico / Veracruz	Human	Female	70	Deceased	Oropharyngeal swab
hCoV-19/Mexico/VER-InDRE_F12182_S1426/2021	EPI_ISL_1700812	16/03/21	North America / Mexico / Veracruz	Human	Female	62	Deceased	Oropharyngeal swab
hCoV-19/Mexico/VER-InDRE_F12183_S1427/2021	EPI_ISL_1700813	16/03/21	North America / Mexico / Veracruz	Human	Female	27	Hospitalized	Oropharyngeal swab
hCoV-19/Mexico/COA-InDRE_F12230_S1433/2021	EPI_ISL_1700818	18/03/21	North America / Mexico / Coahuila	Human	Male	47	Released	Oropharyngeal swab
hCoV-19/Mexico/BCS-InDRE_F12244_S1434/2021	EPI_ISL_1700819	20/03/21	North America / Mexico / Baja California Sur	Human	Male	85	Released	Oropharyngeal swab
hCoV-19/Mexico/NAY-InDRE_F12257_S1438/2021	EPI_ISL_1700823	16/03/21	North America / Mexico / Nayarit	Human	Male	58	Hospitalized	Oropharyngeal swab
hCoV-19/Mexico/CMX-InDRE_F12262_S1439/2021	EPI_ISL_1700824	15/03/21	North America / Mexico / Mexico City	Human	Male	70	Released	Oropharyngeal swab
hCoV-19/Mexico/MEX-InDRE_F12287_S1440/2021	EPI_ISL_1700825	16/03/21	North America / Mexico / State of Mexico	Human	Male	24	Hospitalized	Oropharyngeal swab
hCoV-19/Mexico/MOR-InDRE_F12308_S1441/2021	EPI_ISL_1700826	23/03/21	North America / Mexico / Morelos	Human	Male	68	Released	Oropharyngeal swab
hCoV-19/Mexico/MOR-InDRE_F12309_S1442/2021	EPI_ISL_1700827	23/03/21	North America / Mexico / Morelos	Human	Female	74	Released	Oropharyngeal swab
hCoV-19/Mexico/QUE-InDRE_F12500_S1455/2021	EPI_ISL_1700831	23/03/21	North America / Mexico / Queretaro	Human	Female	60	Released	Oropharyngeal swab
hCoV-19/Mexico/QUE-InDRE_F12502_S1456/2021	EPI_ISL_1700832	23/03/21	North America / Mexico / Queretaro	Human	Male	39	Released	Oropharyngeal swab
hCoV-19/Mexico/ZAC-InDRE_F12520_S1457/2021	EPI_ISL_1700833	21/03/21	North America / Mexico / Zacatecas	Human	Male	57	Deceased	Oropharyngeal swab
hCoV-19/Mexico/MOR-InDRE_F12331_S1459/2021	EPI_ISL_1700835	28/03/21	North America / Mexico / Morelos	Human	Male	44	Hospitalized	Oropharyngeal swab
hCoV-19/Mexico/BCN-InDRE_F12593_S1461/2021	EPI_ISL_1700837	08/03/21	North America / Mexico / Baja California	Human	Female	42	unknown	Oropharyngeal swab
hCoV-19/Mexico/MOR-InDRE_F12112_S1473/2021	EPI_ISL_1700840	22/03/21	North America / Mexico / Morelos	Human	Female	26	Released	Oropharyngeal swab

hCoV-19/Mexico/ROO-InDRE_F12253_S1474/2021	EPI_ISL_1710963	17/03/21	North America / Mexico / Quintana Roo	Human	Male	42	Hospitalized		Oropharyngeal swab
hCoV-19/Mexico/CMX-InDRE_F12281_S1475/2021	EPI_ISL_1710964	19/03/21	North America / Mexico / Mexico City	Human	Female	14	Released		Oropharyngeal swab
hCoV-19/Mexico/CMX-InDRE_F12285_S1476/2021	EPI_ISL_1710965	18/03/21	North America / Mexico / Mexico City	Human	Male	52	Released		Oropharyngeal swab
hCoV-19/Mexico/CMX-InDRE_F12290_S1477/2021	EPI_ISL_1710966	16/03/21	North America / Mexico / Mexico City	Human	Female	79	Deceased		Oropharyngeal swab
hCoV-19/Mexico/MOR-InDRE_F12315_S1478/2021	EPI_ISL_1710967	23/03/21	North America / Mexico / Morelos	Human	Male	68	Hospitalized		Oropharyngeal swab
hCoV-19/Mexico/GUA-InDRE_F12463_S1479/2021	EPI_ISL_1710968	23/03/21	North America / Mexico / Guanajuato	Human	Male	36	Released		Oropharyngeal swab
hCoV-19/Mexico/GUA-InDRE_F12464_S1480/2021	EPI_ISL_1710969	24/03/21	North America / Mexico / Guanajuato	Human	Female	49	Released		Oropharyngeal swab
hCoV-19/Mexico/ZAC-InDRE_F12511_S1481/2021	EPI_ISL_1710970	27/03/21	North America / Mexico / Zacatecas	Human	Male	44	Released		Oropharyngeal swab
hCoV-19/Mexico/CMX-INMEGEN-04-05-23/2021	EPI_ISL_1711276	12/04/21	North America / Mexico / Mexico City	Human	Female	55	unknown		Oropharyngeal swab
hCoV-19/Mexico/CMX-INMEGEN-04-05-66/2021	EPI_ISL_1711277	14/04/21	North America / Mexico / Mexico City	Human	Female	19	unknown		Oropharyngeal swab
hCoV-19/Mexico/CMX-INMEGEN-04-05-44/2021	EPI_ISL_1711278	13/04/21	North America / Mexico / Mexico City	Human	Male	28	unknown		Oropharyngeal swab
hCoV-19/Mexico/CMX-INMEGEN-04-05-16/2021	EPI_ISL_1711279	12/04/21	North America / Mexico / Mexico City	Human	Female	20	unknown		Oropharyngeal swab
hCoV-19/Mexico/CMX-INMEGEN-04-05-13/2021	EPI_ISL_1711280	12/04/21	North America / Mexico / Mexico City	Human	Female	50	unknown		Oropharyngeal swab
hCoV-19/Mexico/CMX-INMEGEN-04-05-9/2021	EPI_ISL_1711282	12/04/21	North America / Mexico / Mexico City	Human	Male	47	unknown		Oropharyngeal swab
hCoV-19/Mexico/CMX-INMEGEN-04-05-52/2021	EPI_ISL_1711283	13/04/21	North America / Mexico / Mexico City	Human	Female	48	unknown		Oropharyngeal swab
hCoV-19/Mexico/CMX-INMEGEN-04-05-43/2021	EPI_ISL_1711284	13/04/21	North America / Mexico / Mexico City	Human	Male	41	unknown		Oropharyngeal swab
hCoV-19/Mexico/CMX-INMEGEN-04-05-21/2021	EPI_ISL_1711285	12/04/21	North America / Mexico / Mexico City	Human	Female	71	unknown		Oropharyngeal swab
hCoV-19/Mexico/CMX-INMEGEN-04-05-53/2021	EPI_ISL_1711286	13/04/21	North America / Mexico / Mexico City	Human	Female	44	unknown		Oropharyngeal swab
hCoV-19/Mexico/CMX-INMEGEN-04-05-67/2021	EPI_ISL_1711287	14/04/21	North America / Mexico / Mexico City	Human	Male	40	unknown		Oropharyngeal swab
hCoV-19/Mexico/CMX-INMEGEN-04-05-27/2021	EPI_ISL_1711288	12/04/21	North America / Mexico / Mexico City	Human	Female	67	unknown		Oropharyngeal swab
hCoV-19/Mexico/CMX-INMEGEN-04-05-19/2021	EPI_ISL_1711289	12/04/21	North America / Mexico / Mexico City	Human	Female	53	unknown		Oropharyngeal swab
hCoV-19/Mexico/CMX-INMEGEN-04-05-11/2021	EPI_ISL_1711290	12/04/21	North America / Mexico / Mexico City	Human	Male	22	unknown		Oropharyngeal swab
hCoV-19/Mexico/CMX-INMEGEN-04-05-38/2021	EPI_ISL_1711291	12/04/21	North America / Mexico / Mexico City	Human	Male	19	unknown		Oropharyngeal swab
hCoV-19/Mexico/CMX-INMEGEN-04-05-50/2021	EPI_ISL_1711292	13/04/21	North America / Mexico / Mexico City	Human	Female	28	unknown		Oropharyngeal swab
hCoV-19/Mexico/CMX-INMEGEN-04-05-31/2021	EPI_ISL_1711294	12/04/21	North America / Mexico / Mexico City	Human	Male	52	unknown		Oropharyngeal swab
hCoV-19/Mexico/CMX-INMEGEN-04-05-51/2021	EPI_ISL_1711295	13/04/21	North America / Mexico / Mexico City	Human	Female	58	unknown		Oropharyngeal swab
hCoV-19/Mexico/CMX-INMEGEN-04-05-55/2021	EPI_ISL_1711296	13/04/21	North America / Mexico / Mexico City	Human	Male	61	unknown		Oropharyngeal swab
hCoV-19/Mexico/CMX-INMEGEN-04-05-40/2021	EPI_ISL_1711303	12/04/21	North America / Mexico / Mexico City	Human	Female	15	unknown		Oropharyngeal swab
hCoV-19/Mexico/CMX-INMEGEN-04-05-12/2021	EPI_ISL_1711304	12/04/21	North America / Mexico / Mexico City	Human	Male	48	unknown		Oropharyngeal swab
hCoV-19/Mexico/CMX-INMEGEN-04-05-30/2021	EPI_ISL_1711305	12/04/21	North America / Mexico / Mexico City	Human	Male	46	unknown		Oropharyngeal swab
hCoV-19/Mexico/CMX-INMEGEN-04-05-7/2021	EPI_ISL_1711306	12/04/21	North America / Mexico / Mexico City	Human	Male	41	unknown		Oropharyngeal swab
hCoV-19/Mexico/CMX-INMEGEN-04-05-17/2021	EPI_ISL_1711308	12/04/21	North America / Mexico / Mexico City	Human	Female	20	unknown		Oropharyngeal swab
hCoV-19/Mexico/CMX-INMEGEN-04-05-15/2021	EPI_ISL_1711310	12/04/21	North America / Mexico / Mexico City	Human	Female	59	unknown		Oropharyngeal swab
hCoV-19/Mexico/CMX-INMEGEN-04-05-41/2021	EPI_ISL_1711316	13/04/21	North America / Mexico / Mexico City	Human	Male	72	unknown		Oropharyngeal swab
hCoV-19/Mexico/CMX-INMEGEN-04-05-42/2021	EPI_ISL_1711317	13/04/21	North America / Mexico / Mexico City	Human	Female	58	unknown		Oropharyngeal swab
hCoV-19/Mexico/CMX-INMEGEN-04-05-1/2021	EPI_ISL_1711318	09/04/21	North America / Mexico / Mexico City	Human	Female	37	unknown		Oropharyngeal swab
hCoV-19/Mexico/CMX-INMEGEN-04-05-2/2021	EPI_ISL_1711319	11/04/21	North America / Mexico / Mexico City	Human	Female	45	unknown		Oropharyngeal swab
hCoV-19/Mexico/CMX-INMEGEN-04-05-3/2021	EPI_ISL_1711320	12/04/21	North America / Mexico / Mexico City	Human	Male	59	unknown		Oropharyngeal swab
hCoV-19/Mexico/CMX-INMEGEN-04-05-4/2021	EPI_ISL_1711321	12/04/21	North America / Mexico / Mexico City	Human	Female	65	unknown		Oropharyngeal swab
hCoV-19/Mexico/CMX-INMEGEN-04-05-5/2021	EPI_ISL_1711322	12/04/21	North America / Mexico / Mexico City	Human	Male	47	unknown		Oropharyngeal swab
hCoV-19/Mexico/CMX-INMEGEN-04-05-10/2021	EPI_ISL_1711324	12/04/21	North America / Mexico / Mexico City	Human	Male	51	unknown		Oropharyngeal swab
hCoV-19/Mexico/CMX-INMEGEN-04-05-14/2021	EPI_ISL_1711325	12/04/21	North America / Mexico / Mexico City	Human	Female	65	unknown		Oropharyngeal swab
hCoV-19/Mexico/CMX-INMEGEN-04-05-18/2021	EPI_ISL_1711326	12/04/21	North America / Mexico / Mexico City	Human	Female	21	unknown		Oropharyngeal swab
hCoV-19/Mexico/CMX-INMEGEN-04-05-20/2021	EPI_ISL_1711327	12/04/21	North America / Mexico / Mexico City	Human	Female	56	unknown		Oropharyngeal swab

hCoV-19/Mexico/CMX-INMEGEN-04-05-24/2021	EPI_ISL_1711328	12/04/21	North America / Mexico / Mexico City	Human	Female	51	unknown	Oropharyngeal swab
hCoV-19/Mexico/CMX-INMEGEN-04-05-25/2021	EPI_ISL_1711329	12/04/21	North America / Mexico / Mexico City	Human	Female	19	unknown	Oropharyngeal swab
hCoV-19/Mexico/CMX-INMEGEN-04-05-26/2021	EPI_ISL_1711330	12/04/21	North America / Mexico / Mexico City	Human	Female	64	unknown	Oropharyngeal swab
hCoV-19/Mexico/CMX-INMEGEN-04-05-28/2021	EPI_ISL_1711331	12/04/21	North America / Mexico / Mexico City	Human	Female	37	unknown	Oropharyngeal swab
hCoV-19/Mexico/CMX-INMEGEN-04-05-29/2021	EPI_ISL_1711332	12/04/21	North America / Mexico / Mexico City	Human	Male	71	unknown	Oropharyngeal swab
hCoV-19/Mexico/CMX-INMEGEN-04-05-33/2021	EPI_ISL_1711333	12/04/21	North America / Mexico / Mexico City	Human	Male	46	unknown	Oropharyngeal swab
hCoV-19/Mexico/CMX-INMEGEN-04-05-36/2021	EPI_ISL_1711335	12/04/21	North America / Mexico / Mexico City	Human	Male	66	unknown	Oropharyngeal swab
hCoV-19/Mexico/CMX-INMEGEN-04-05-37/2021	EPI_ISL_1711336	12/04/21	North America / Mexico / Mexico City	Human	Male	69	unknown	Oropharyngeal swab
hCoV-19/Mexico/CMX-INMEGEN-04-05-39/2021	EPI_ISL_1711337	12/04/21	North America / Mexico / Mexico City	Human	Female	19	unknown	Oropharyngeal swab
hCoV-19/Mexico/CMX-INMEGEN-04-05-48/2021	EPI_ISL_1711339	13/04/21	North America / Mexico / Mexico City	Human	Male	53	unknown	Oropharyngeal swab
hCoV-19/Mexico/CMX-INMEGEN-04-05-54/2021	EPI_ISL_1711340	13/04/21	North America / Mexico / Mexico City	Human	Female	57	unknown	Oropharyngeal swab
hCoV-19/Mexico/CMX-INMEGEN-04-05-59/2021	EPI_ISL_1711341	14/04/21	North America / Mexico / Mexico City	Human	Male	62	unknown	Oropharyngeal swab
hCoV-19/Mexico/CMX-INMEGEN-04-05-60/2021	EPI_ISL_1711342	14/04/21	North America / Mexico / Mexico City	Human	Male	52	unknown	Oropharyngeal swab
hCoV-19/Mexico/CMX-INMEGEN-04-05-61/2021	EPI_ISL_1711343	14/04/21	North America / Mexico / Mexico City	Human	Female	76	unknown	Oropharyngeal swab
hCoV-19/Mexico/CMX-INMEGEN-04-05-62/2021	EPI_ISL_1711344	14/04/21	North America / Mexico / Mexico City	Human	Male	45	unknown	Oropharyngeal swab
hCoV-19/Mexico/CMX-INMEGEN-04-05-68/2021	EPI_ISL_1711345	14/04/21	North America / Mexico / Mexico City	Human	Male	59	unknown	Oropharyngeal swab
hCoV-19/Mexico/CMX-INMEGEN-04-05-73/2021	EPI_ISL_1711346	09/04/21	North America / Mexico / Mexico City	Human	Male	40	unknown	Oropharyngeal swab
hCoV-19/Mexico/CMX-INMEGEN-04-05-74/2021	EPI_ISL_1711347	09/04/21	North America / Mexico / Mexico City	Human	Female	73	unknown	Oropharyngeal swab
hCoV-19/Mexico/CMX-INMEGEN-03-10-09/2021	EPI_ISL_1758186	22/03/21	North America / Mexico / Mexico City	Human	Male	61	unknown	Oropharyngeal swab
hCoV-19/Mexico/BCN-SEARCH-8937/2021	EPI_ISL_1794747	30/03/21	orth America / Mexico / Baja California / Tijuan	Human	unknown	unknown	unknown	Nasal swab
hCoV-19/Mexico/CMX-INMEGEN-04-06-1/2021	EPI_ISL_1807319	20/04/21	North America / Mexico / Mexico City	Human	Male	53	unknown	Oropharyngeal swab
hCoV-19/Mexico/CMX-INMEGEN-04-06-5/2021	EPI_ISL_1807322	20/04/21	North America / Mexico / Mexico City	Human	Male	46	unknown	Oropharyngeal swab
hCoV-19/Mexico/CMX-INMEGEN-04-06-6/2021	EPI_ISL_1807323	20/04/21	North America / Mexico / Mexico City	Human	Male	68	unknown	Oropharyngeal swab
hCoV-19/Mexico/CMX-INMEGEN-04-06-7/2021	EPI_ISL_1807324	20/04/21	North America / Mexico / Mexico City	Human	Female	49	unknown	Oropharyngeal swab
hCoV-19/Mexico/CMX-INMEGEN-04-06-8/2021	EPI_ISL_1807325	20/04/21	North America / Mexico / Mexico City	Human	Male	22	unknown	Oropharyngeal swab
hCoV-19/Mexico/CMX-INMEGEN-04-06-9/2021	EPI_ISL_1807326	20/04/21	North America / Mexico / Mexico City	Human	Female	48	unknown	Oropharyngeal swab
hCoV-19/Mexico/CMX-INMEGEN-04-06-10/2021	EPI_ISL_1807327	20/04/21	North America / Mexico / Mexico City	Human	Female	36	unknown	Oropharyngeal swab
hCoV-19/Mexico/CMX-INMEGEN-04-06-12/2021	EPI_ISL_1807328	20/04/21	North America / Mexico / Mexico City	Human	Female	30	unknown	Oropharyngeal swab
hCoV-19/Mexico/CMX-INMEGEN-04-06-13/2021	EPI_ISL_1807329	20/04/21	North America / Mexico / Mexico City	Human	Male	51	unknown	Oropharyngeal swab
hCoV-19/Mexico/CMX-INMEGEN-04-06-14/2021	EPI_ISL_1807330	20/04/21	North America / Mexico / Mexico City	Human	Male	33	unknown	Oropharyngeal swab
hCoV-19/Mexico/CMX-INMEGEN-04-06-21/2021	EPI_ISL_1807331	21/04/21	North America / Mexico / Mexico City	Human	Female	39	unknown	Oropharyngeal swab
hCoV-19/Mexico/CMX-INMEGEN-04-06-22/2021	EPI_ISL_1807332	21/04/21	North America / Mexico / Mexico City	Human	Female	32	unknown	Oropharyngeal swab
hCoV-19/Mexico/CMX-INMEGEN-04-06-23/2021	EPI_ISL_1807333	21/04/21	North America / Mexico / Mexico City	Human	Female	33	unknown	Oropharyngeal swab
hCoV-19/Mexico/CMX-INMEGEN-04-06-25/2021	EPI_ISL_1807335	21/04/21	North America / Mexico / Mexico City	Human	Male	41	unknown	Oropharyngeal swab
hCoV-19/Mexico/CMX-INMEGEN-04-06-26/2021	EPI_ISL_1807336	21/04/21	North America / Mexico / Mexico City	Human	Male	27	unknown	Oropharyngeal swab
hCoV-19/Mexico/CMX-INMEGEN-04-06-27/2021	EPI_ISL_1807337	21/04/21	North America / Mexico / Mexico City	Human	Male	74	unknown	Oropharyngeal swab
hCoV-19/Mexico/CMX-INMEGEN-04-06-29/2021	EPI_ISL_1807338	21/04/21	North America / Mexico / Mexico City	Human	Female	56	unknown	Oropharyngeal swab
hCoV-19/Mexico/CMX-INMEGEN-04-06-30/2021	EPI_ISL_1807339	21/04/21	North America / Mexico / Mexico City	Human	Female	44	unknown	Oropharyngeal swab
hCoV-19/Mexico/CMX-INMEGEN-04-06-31/2021	EPI_ISL_1807340	21/04/21	North America / Mexico / Mexico City	Human	Male	45	unknown	Oropharyngeal swab
hCoV-19/Mexico/CMX-INMEGEN-04-06-32/2021	EPI_ISL_1807341	21/04/21	North America / Mexico / Mexico City	Human	Male	59	unknown	Oropharyngeal swab
hCoV-19/Mexico/CMX-INMEGEN-04-06-33/2021	EPI_ISL_1807342	21/04/21	North America / Mexico / Mexico City	Human	Male	49	unknown	Oropharyngeal swab
hCoV-19/Mexico/CMX-INMEGEN-04-06-35/2021	EPI_ISL_1807343	21/04/21	North America / Mexico / Mexico City	Human	Female	51	unknown	Oropharyngeal swab
hCoV-19/Mexico/CMX-INMEGEN-04-06-40/2021	EPI_ISL_1807345	16/04/21	North America / Mexico / Mexico City	Human	Male	21	unknown	Oropharyngeal swab
hCoV-19/Mexico/CMX-INMEGEN-04-06-44/2021	EPI_ISL_1807347	10/03/21	North America / Mexico / Mexico City	Human	Male	69	unknown	Oropharyngeal swab

hCoV-19/Mexico/CMX-INMEGEN-04-06-46/2021	EPI_ISL_1807348	22/04/21	North America / Mexico / Mexico City	Human	Male	39	unknown	Oropharyngeal swab
hCoV-19/Mexico/CMX-INMEGEN-04-06-47/2021	EPI_ISL_1807349	22/04/21	North America / Mexico / Mexico City	Human	Male	53	unknown	Oropharyngeal swab
hCoV-19/Mexico/CMX-INMEGEN-04-06-48/2021	EPI_ISL_1807350	22/04/21	North America / Mexico / Mexico City	Human	Female	40	unknown	Oropharyngeal swab
hCoV-19/Mexico/CMX-INMEGEN-04-06-49/2021	EPI_ISL_1807351	22/04/21	North America / Mexico / Mexico City	Human	Male	67	unknown	Oropharyngeal swab
hCoV-19/Mexico/CMX-INMEGEN-04-06-50/2021	EPI_ISL_1807352	22/04/21	North America / Mexico / Mexico City	Human	Male	34	unknown	Oropharyngeal swab
hCoV-19/Mexico/CMX-INMEGEN-04-06-51/2021	EPI_ISL_1807353	22/04/21	North America / Mexico / Mexico City	Human	Female	18	unknown	Oropharyngeal swab
hCoV-19/Mexico/CMX-INMEGEN-04-06-52/2021	EPI_ISL_1807354	22/04/21	North America / Mexico / Mexico City	Human	Female	52	unknown	Oropharyngeal swab
hCoV-19/Mexico/CMX-INMEGEN-04-06-54/2021	EPI_ISL_1807356	22/04/21	North America / Mexico / Mexico City	Human	Male	19	unknown	Oropharyngeal swab
hCoV-19/Mexico/CMX-INMEGEN-04-06-56/2021	EPI_ISL_1807357	22/04/21	North America / Mexico / Mexico City	Human	Female	50	unknown	Oropharyngeal swab
hCoV-19/Mexico/CMX-INMEGEN-04-06-66/2021	EPI_ISL_1807360	11/03/21	North America / Mexico / Mexico City	Human	Female	69	unknown	Oropharyngeal swab
hCoV-19/Mexico/CMX-INMEGEN-04-06-70/2021	EPI_ISL_1807361	22/03/21	North America / Mexico / Mexico City	Human	Female	53	unknown	Oropharyngeal swab
hCoV-19/Mexico/CMX-INMEGEN-04-06-78/2021	EPI_ISL_1807365	16/03/21	North America / Mexico / Mexico City	Human	Male	54	unknown	Oropharyngeal swab
hCoV-19/Mexico/CMX-INMEGEN-04-06-81/2021	EPI_ISL_1807366	16/03/21	North America / Mexico / Mexico City	Human	Male	70	unknown	Oropharyngeal swab
hCoV-19/Mexico/CMX-INMEGEN-04-06-82/2021	EPI_ISL_1807367	16/03/21	North America / Mexico / Mexico City	Human	Male	63	unknown	Oropharyngeal swab
hCoV-19/Mexico/CMX-INMEGEN-04-06-85/2021	EPI_ISL_1807369	12/03/21	North America / Mexico / Mexico City	Human	Female	48	unknown	Oropharyngeal swab
hCoV-19/Mexico/CMX-INMEGEN-04-06-86/2021	EPI_ISL_1807370	12/03/21	North America / Mexico / Mexico City	Human	Female	29	unknown	Oropharyngeal swab
hCoV-19/Mexico/CMX-INMEGEN-04-06-93/2021	EPI_ISL_1807373	11/03/21	North America / Mexico / Mexico City	Human	Female	46	unknown	Oropharyngeal swab
CoV-19/Mexico/CMX-INMEGEN-InDRE-04-06-97/202	EPI_ISL_1810831	14/04/21	North America / Mexico / Mexico City	Human	Female	34	Released	Oropharyngeal swab
CoV-19/Mexico/CMX-INMEGEN-InDRE-04-06-98/202	EPI_ISL_1810832	14/04/21	North America / Mexico / Mexico City	Human	Female	32	Released	Oropharyngeal swab
CoV-19/Mexico/CMX-INMEGEN-InDRE-04-06-99/202	EPI_ISL_1810833	14/04/21	North America / Mexico / Mexico City	Human	Female	19	Released	Oropharyngeal swab
CoV-19/Mexico/CMX-INMEGEN-InDRE-04-06-100/20	EPI_ISL_1810834	14/04/21	North America / Mexico / Mexico City	Human	Male	17	Released	Oropharyngeal swab
CoV-19/Mexico/CMX-INMEGEN-InDRE-04-06-101/20	EPI_ISL_1810835	14/04/21	North America / Mexico / Mexico City	Human	Female	37	Released	Oropharyngeal swab
CoV-19/Mexico/CMX-INMEGEN-InDRE-04-06-102/20	EPI_ISL_1810836	14/04/21	North America / Mexico / Mexico City	Human	Male	26	Released	Oropharyngeal swab
CoV-19/Mexico/CMX-INMEGEN-InDRE-04-06-104/20	EPI_ISL_1810838	14/04/21	North America / Mexico / Mexico City	Human	Female	44	Released	Oropharyngeal swab
CoV-19/Mexico/CMX-INMEGEN-InDRE-04-06-105/20	EPI_ISL_1810839	13/04/21	North America / Mexico / Mexico City	Human	Female	43	Released	Oropharyngeal swab
CoV-19/Mexico/MEX-INMEGEN-InDRE-04-06-106/20	EPI_ISL_1810840	14/04/21	North America / Mexico / State of Mexico	Human	Female	52	Released	Oropharyngeal swab
CoV-19/Mexico/CMX-INMEGEN-InDRE-04-06-107/20	EPI_ISL_1810841	14/04/21	North America / Mexico / Mexico City	Human	Male	42	Released	Oropharyngeal swab
CoV-19/Mexico/CMX-INMEGEN-InDRE-04-06-108/20	EPI_ISL_1810842	15/04/21	North America / Mexico / Mexico City	Human	Male	74	Hospitalized	Oropharyngeal swab
CoV-19/Mexico/CMX-INMEGEN-InDRE-04-06-109/20	EPI_ISL_1810843	14/04/21	North America / Mexico / Mexico City	Human	Male	39	Released	Oropharyngeal swab
CoV-19/Mexico/CMX-INMEGEN-InDRE-04-06-110/20	EPI_ISL_1810844	14/04/21	North America / Mexico / Mexico City	Human	Female	57	Live	Oropharyngeal swab
CoV-19/Mexico/CMX-INMEGEN-InDRE-04-06-111/20	EPI_ISL_1810845	14/04/21	North America / Mexico / Mexico City	Human	Female	22	Live	Oropharyngeal swab
CoV-19/Mexico/MEX-INMEGEN-InDRE-04-06-112/20	EPI_ISL_1810846	14/04/21	North America / Mexico / State of Mexico	Human	Female	28	Released	Oropharyngeal swab
CoV-19/Mexico/CMX-INMEGEN-InDRE-04-06-114/20	EPI_ISL_1810848	14/04/21	North America / Mexico / Mexico City	Human	Female	17	Released	Oropharyngeal swab
CoV-19/Mexico/CMX-INMEGEN-InDRE-04-06-115/20	EPI_ISL_1810849	14/04/21	North America / Mexico / Mexico City	Human	Male	24	Released	Oropharyngeal swab
CoV-19/Mexico/CMX-INMEGEN-InDRE-04-06-116/20	EPI_ISL_1810850	14/04/21	North America / Mexico / Mexico City	Human	Male	78	Live	Oropharyngeal swab
CoV-19/Mexico/CMX-INMEGEN-InDRE-04-06-119/20	EPI_ISL_1810852	13/04/21	North America / Mexico / Mexico City	Human	Female	46	Released	Oropharyngeal swab
CoV-19/Mexico/CMX-INMEGEN-InDRE-04-06-120/20	EPI_ISL_1810853	13/04/21	North America / Mexico / Mexico City	Human	Female	78	Released	Oropharyngeal swab
CoV-19/Mexico/MEX-INMEGEN-InDRE-04-06-121/20	EPI_ISL_1810854	13/04/21	North America / Mexico / State of Mexico	Human	Female	28	Released	Oropharyngeal swab
CoV-19/Mexico/CMX-INMEGEN-InDRE-04-06-122/20	EPI_ISL_1810855	13/04/21	North America / Mexico / Mexico City	Human	Male	19	Released	Oropharyngeal swab
CoV-19/Mexico/CMX-INMEGEN-InDRE-04-06-123/20	EPI_ISL_1810856	13/04/21	North America / Mexico / Mexico City	Human	Female	19	Released	Oropharyngeal swab
CoV-19/Mexico/CMX-INMEGEN-InDRE-04-06-125/20	EPI_ISL_1810858	13/04/21	North America / Mexico / Mexico City	Human	Female	41	Live	Oropharyngeal swab
CoV-19/Mexico/CMX-INMEGEN-InDRE-04-06-126/20	EPI_ISL_1810859	14/04/21	North America / Mexico / Mexico City	Human	Female	17	Released	Oropharyngeal swab
CoV-19/Mexico/CMX-INMEGEN-InDRE-04-06-127/20	EPI_ISL_1810860	14/04/21	North America / Mexico / Mexico City	Human	Male	23	Released	Oropharyngeal swab
CoV-19/Mexico/MEX-INMEGEN-InDRE-04-06-128/20	EPI_ISL_1810861	14/04/21	North America / Mexico / State of Mexico	Human	Male	42	Released	Oropharyngeal swab

CoV-19/Mexico/CMX-INMEGEN-InDRE-04-06-129/20	EPI_ISL_1810862	15/04/21	North America / Mexico / Mexico City	Human	Female	33	unknown	Oropharyngeal swab
CoV-19/Mexico/CMX-INMEGEN-InDRE-04-06-130/20	EPI_ISL_1810863	14/04/21	North America / Mexico / Mexico City	Human	Male	74	Released	Oropharyngeal swab
CoV-19/Mexico/CMX-INMEGEN-InDRE-04-06-131/20	EPI_ISL_1810864	14/04/21	North America / Mexico / Mexico City	Human	Female	68	Live	Oropharyngeal swab
CoV-19/Mexico/CMX-INMEGEN-InDRE-04-06-132/20	EPI_ISL_1810865	15/04/21	North America / Mexico / Mexico City	Human	Male	69	Live	Oropharyngeal swab
CoV-19/Mexico/CMX-INMEGEN-InDRE-04-06-133/20	EPI_ISL_1810866	15/04/21	North America / Mexico / Mexico City	Human	Female	71	Live	Oropharyngeal swab
CoV-19/Mexico/CMX-INMEGEN-InDRE-04-06-134/20	EPI_ISL_1810867	14/04/21	North America / Mexico / Mexico City	Human	Male	51	Hospitalized	Oropharyngeal swab
CoV-19/Mexico/CMX-INMEGEN-InDRE-04-06-135/20	EPI_ISL_1810868	14/04/21	North America / Mexico / Mexico City	Human	Female	88	Deceased	Oropharyngeal swab
CoV-19/Mexico/MEX-INMEGEN-InDRE-04-06-136/20	EPI_ISL_1810869	15/04/21	North America / Mexico / State of Mexico	Human	Male	41	Released	Oropharyngeal swab
CoV-19/Mexico/CMX-INMEGEN-InDRE-04-06-137/20	EPI_ISL_1810870	13/04/21	North America / Mexico / Mexico City	Human	Male	52	Live	Oropharyngeal swab
CoV-19/Mexico/CMX-INMEGEN-InDRE-04-06-138/20	EPI_ISL_1810871	14/04/21	North America / Mexico / Mexico City	Human	Male	60	Live	Oropharyngeal swab
CoV-19/Mexico/CMX-INMEGEN-InDRE-04-06-139/20	EPI_ISL_1810872	15/04/21	North America / Mexico / Mexico City	Human	Female	59	Released	Oropharyngeal swab
CoV-19/Mexico/CMX-INMEGEN-InDRE-04-06-141/20	EPI_ISL_1810874	15/04/21	North America / Mexico / Mexico City	Human	Male	37	Released	Oropharyngeal swab
CoV-19/Mexico/CMX-INMEGEN-InDRE-04-06-142/20	EPI_ISL_1810875	15/04/21	North America / Mexico / Mexico City	Human	Female	27	Released	Oropharyngeal swab
CoV-19/Mexico/CMX-INMEGEN-InDRE-04-06-144/20	EPI_ISL_1810877	15/04/21	North America / Mexico / Mexico City	Human	Female	35	Released	Oropharyngeal swab
CoV-19/Mexico/CMX-INMEGEN-InDRE-04-06-145/20	EPI_ISL_1810878	14/04/21	North America / Mexico / Mexico City	Human	Male	42	Released	Oropharyngeal swab
CoV-19/Mexico/CMX-INMEGEN-InDRE-04-06-146/20	EPI_ISL_1810879	15/04/21	North America / Mexico / Mexico City	Human	Male	46	Live	Oropharyngeal swab
CoV-19/Mexico/CMX-INMEGEN-InDRE-04-06-147/20	EPI_ISL_1810880	15/04/21	North America / Mexico / Mexico City	Human	Male	50	Deceased	Oropharyngeal swab
CoV-19/Mexico/CMX-INMEGEN-InDRE-04-06-148/20	EPI_ISL_1810881	15/04/21	North America / Mexico / Mexico City	Human	Male	52	Deceased	Oropharyngeal swab
CoV-19/Mexico/CMX-INMEGEN-InDRE-04-06-149/20	EPI_ISL_1810882	14/04/21	North America / Mexico / Mexico City	Human	Female	60	Hospitalized	Oropharyngeal swab
CoV-19/Mexico/MEX-INMEGEN-InDRE-04-06-151/20	EPI_ISL_1810884	15/04/21	North America / Mexico / State of Mexico	Human	Female	51	Hospitalized	Oropharyngeal swab
CoV-19/Mexico/CMX-INMEGEN-InDRE-04-06-152/20	EPI_ISL_1810885	15/04/21	North America / Mexico / Mexico City	Human	Male	75	Released	Oropharyngeal swab
CoV-19/Mexico/CMX-INMEGEN-InDRE-04-06-153/20	EPI_ISL_1810886	15/04/21	North America / Mexico / Mexico City	Human	Male	52	Live	Oropharyngeal swab
CoV-19/Mexico/CMX-INMEGEN-InDRE-04-06-154/20	EPI_ISL_1810887	15/04/21	North America / Mexico / Mexico City	Human	Male	32	Released	Oropharyngeal swab
CoV-19/Mexico/CMX-INMEGEN-InDRE-04-06-155/20	EPI_ISL_1810888	15/04/21	North America / Mexico / Mexico City	Human	Male	50	Live	Oropharyngeal swab
CoV-19/Mexico/CMX-INMEGEN-InDRE-04-06-156/20	EPI_ISL_1810889	15/04/21	North America / Mexico / Mexico City	Human	Male	41	Live	Oropharyngeal swab
CoV-19/Mexico/CMX-INMEGEN-InDRE-04-06-157/20	EPI_ISL_1810890	16/04/21	North America / Mexico / Mexico City	Human	Male	29	Released	Oropharyngeal swab
CoV-19/Mexico/MEX-INMEGEN-InDRE-04-06-158/20	EPI_ISL_1810891	15/04/21	North America / Mexico / State of Mexico	Human	Male	48	Released	Oropharyngeal swab
CoV-19/Mexico/CMX-INMEGEN-InDRE-04-06-159/20	EPI_ISL_1810892	16/04/21	North America / Mexico / Mexico City	Human	Female	64	Live	Oropharyngeal swab
CoV-19/Mexico/CMX-INMEGEN-InDRE-04-06-160/20	EPI_ISL_1810893	16/04/21	North America / Mexico / Mexico City	Human	Male	69	Live	Oropharyngeal swab
CoV-19/Mexico/CMX-INMEGEN-InDRE-04-06-161/20	EPI_ISL_1810894	16/04/21	North America / Mexico / Mexico City	Human	Male	50	Live	Oropharyngeal swab
CoV-19/Mexico/CMX-INMEGEN-InDRE-04-06-162/20	EPI_ISL_1810895	16/04/21	North America / Mexico / Mexico City	Human	Female	62	Live	Oropharyngeal swab
CoV-19/Mexico/CMX-INMEGEN-InDRE-04-06-163/20	EPI_ISL_1810896	15/04/21	North America / Mexico / Mexico City	Human	Female	5	Released	Oropharyngeal swab
CoV-19/Mexico/CMX-INMEGEN-InDRE-04-06-164/20	EPI_ISL_1810897	15/04/21	North America / Mexico / Mexico City	Human	Male	51	Deceased	Oropharyngeal swab
CoV-19/Mexico/MEX-INMEGEN-InDRE-04-06-167/20	EPI_ISL_1810900	16/04/21	North America / Mexico / State of Mexico	Human	Female	61	Hospitalized	Oropharyngeal swab
CoV-19/Mexico/CMX-INMEGEN-InDRE-04-06-168/20	EPI_ISL_1810901	16/04/21	North America / Mexico / Mexico City	Human	Female	70	Released	Oropharyngeal swab
CoV-19/Mexico/CMX-INMEGEN-InDRE-04-06-169/20	EPI_ISL_1810902	16/04/21	North America / Mexico / Mexico City	Human	Male	22	Released	Oropharyngeal swab
CoV-19/Mexico/CMX-INMEGEN-InDRE-04-06-172/20	EPI_ISL_1810905	17/04/21	North America / Mexico / Mexico City	Human	Female	21	Released	Oropharyngeal swab
CoV-19/Mexico/MEX-INMEGEN-InDRE-04-06-173/20	EPI_ISL_1810906	16/04/21	North America / Mexico / State of Mexico	Human	Female	14	Released	Oropharyngeal swab
CoV-19/Mexico/CMX-INMEGEN-InDRE-04-06-174/20	EPI_ISL_1810907	16/04/21	North America / Mexico / Mexico City	Human	Female	41	Released	Oropharyngeal swab
CoV-19/Mexico/CMX-INMEGEN-InDRE-04-06-176/20	EPI_ISL_1810909	17/04/21	North America / Mexico / Mexico City	Human	Male	53	Live	Oropharyngeal swab
CoV-19/Mexico/CMX-INMEGEN-InDRE-04-06-177/20	EPI_ISL_1810910	16/04/21	North America / Mexico / Mexico City	Human	Male	48	Live	Oropharyngeal swab
CoV-19/Mexico/CMX-INMEGEN-InDRE-04-06-178/20	EPI_ISL_1810911	17/04/21	North America / Mexico / Mexico City	Human	Male	47	Released	Oropharyngeal swab
CoV-19/Mexico/CMX-INMEGEN-InDRE-04-06-179/20	EPI_ISL_1810912	17/04/21	North America / Mexico / Mexico City	Human	Male	31	Released	Oropharyngeal swab
CoV-19/Mexico/CMX-INMEGEN-InDRE-04-06-180/20	EPI_ISL_1810913	17/04/21	North America / Mexico / Mexico City	Human	Female	43	Released	Oropharyngeal swab

oV-19/Mexico/CMX-INMEGEN-InDRE-04-06-183/20	EPI_ISL_1810916	16/04/21	North America / Mexico / Mexico City	Human	Female	13	Released	Oropharyngeal swab
oV-19/Mexico/CMX-INMEGEN-InDRE-04-06-184/20	EPI_ISL_1810917	16/04/21	North America / Mexico / Mexico City	Human	Male	29	Released	Oropharyngeal swab
oV-19/Mexico/MEX-INMEGEN-InDRE-04-06-186/20	EPI_ISL_1810919	15/04/21	North America / Mexico / State of Mexico	Human	Male	91	Hospitalized	Oropharyngeal swab
oV-19/Mexico/CMX-INMEGEN-InDRE-04-06-187/20	EPI_ISL_1810920	19/04/21	North America / Mexico / Mexico City	Human	Female	31	Released	Oropharyngeal swab
oV-19/Mexico/CMX-INMEGEN-InDRE-04-06-188/20	EPI_ISL_1810921	16/04/21	North America / Mexico / Mexico City	Human	Male	60	Released	Oropharyngeal swab
oV-19/Mexico/CMX-INMEGEN-InDRE-04-06-189/20	EPI_ISL_1810922	16/04/21	North America / Mexico / Mexico City	Human	Female	45	Live	Oropharyngeal swab
oV-19/Mexico/CMX-INMEGEN-InDRE-04-06-190/20	EPI_ISL_1810923	16/04/21	North America / Mexico / Mexico City	Human	Female	61	Live	Oropharyngeal swab
hCoV-19/Mexico/TLA_IBT_IMSS_1043/2021	EPI_ISL_1811379	03/04/21	North America / Mexico / Tlaxcala	Human	Female	35	Ambulatory	Nasopharyngeal and oropharyngeal swab
hCoV-19/Mexico/TLA_IBT_IMSS_1046/2021	EPI_ISL_1811380	03/04/21	North America / Mexico / Tlaxcala	Human	Male	66	Hospitalized	Nasopharyngeal and oropharyngeal swab
hCoV-19/Mexico/GRO_IBT_IMSS_1055/2021	EPI_ISL_1811381	03/04/21	North America / Mexico / Guerrero	Human	Male	44	Hospitalized	Nasopharyngeal and oropharyngeal swab
hCoV-19/Mexico/PUE_IBT_IMSS_1065/2021	EPI_ISL_1811382	30/03/21	North America / Mexico / Puebla	Human	Female	64	Ambulatory	Nasopharyngeal and oropharyngeal swab
hCoV-19/Mexico/PUE_IBT_IMSS_1087/2021	EPI_ISL_1811383	30/03/21	North America / Mexico / Puebla	Human	Male	63	Ambulatory	Nasopharyngeal and oropharyngeal swab
hCoV-19/Mexico/PUE_IBT_IMSS_1097/2021	EPI_ISL_1811384	30/03/21	North America / Mexico / Puebla	Human	Male	36	Ambulatory	Nasopharyngeal and oropharyngeal swab
hCoV-19/Mexico/PUE_IBT_IMSS_1107/2021	EPI_ISL_1811385	29/03/21	North America / Mexico / Puebla	Human	Female	49	Ambulatory	Nasopharyngeal and oropharyngeal swab
hCoV-19/Mexico/PUE_IBT_IMSS_1118/2021	EPI_ISL_1811386	30/03/21	North America / Mexico / Puebla	Human	Female	17	Ambulatory	Nasopharyngeal and oropharyngeal swab
hCoV-19/Mexico/PUE_IBT_IMSS_1129/2021	EPI_ISL_1811387	30/03/21	North America / Mexico / Puebla	Human	Female	41	Ambulatory	Nasopharyngeal and oropharyngeal swab
hCoV-19/Mexico/PUE_IBT_IMSS_963/2021	EPI_ISL_1811388	28/03/21	North America / Mexico / Puebla	Human	Male	83	Ambulatory	Nasopharyngeal and oropharyngeal swab
hCoV-19/Mexico/PUE_IBT_IMSS_984/2021	EPI_ISL_1811390	30/03/21	North America / Mexico / Puebla	Human	Female	29	Ambulatory	Nasopharyngeal and oropharyngeal swab
hCoV-19/Mexico/PUE_IBT_IMSS_995/2021	EPI_ISL_1811391	31/03/21	North America / Mexico / Puebla	Human	Male	58	Ambulatory	Oropharyngeal swab
hCoV-19/Mexico/PUE_IBT_IMSS_1004/2021	EPI_ISL_1811392	28/03/21	North America / Mexico / Puebla	Human	Male	57	Hospitalized	Nasopharyngeal and oropharyngeal swab
hCoV-19/Mexico/PUE_IBT_IMSS_1014/2021	EPI_ISL_1811393	31/03/21	North America / Mexico / Puebla	Human	Male	26	Ambulatory	Oropharyngeal swab
hCoV-19/Mexico/PUE_IBT_IMSS_1024/2021	EPI_ISL_1811394	01/04/21	North America / Mexico / Puebla	Human	Male	46	Ambulatory	Nasopharyngeal swab
hCoV-19/Mexico/PUE_IBT_IMSS_1035/2021	EPI_ISL_1811395	03/04/21	North America / Mexico / Puebla	Human	Female	52	Ambulatory	Nasopharyngeal and oropharyngeal swab
hCoV-19/Mexico/PUE_IBT_IMSS_1044/2021	EPI_ISL_1811396	31/03/21	North America / Mexico / Puebla	Human	Male	25	Ambulatory	Nasopharyngeal and oropharyngeal swab
hCoV-19/Mexico/PUE_IBT_IMSS_1045/2021	EPI_ISL_1811397	31/03/21	North America / Mexico / Puebla	Human	Male	30	Ambulatory	Nasopharyngeal and oropharyngeal swab
hCoV-19/Mexico/PUE_IBT_IMSS_1047/2021	EPI_ISL_1811398	31/03/21	North America / Mexico / Puebla	Human	Male	30	Ambulatory	Nasopharyngeal and oropharyngeal swab
hCoV-19/Mexico/PUE_IBT_IMSS_1049/2021	EPI_ISL_1811399	03/04/21	North America / Mexico / Puebla	Human	Male	75	Hospitalized	Nasopharyngeal and oropharyngeal swab
hCoV-19/Mexico/PUE_IBT_IMSS_1050/2021	EPI_ISL_1811400	04/04/21	North America / Mexico / Puebla	Human	Male	81	Hospitalized	Nasopharyngeal and oropharyngeal swab
hCoV-19/Mexico/PUE_IBT_IMSS_1051/2021	EPI_ISL_1811401	04/04/21	North America / Mexico / Puebla	Human	Female	56	Hospitalized	Nasopharyngeal and oropharyngeal swab
hCoV-19/Mexico/PUE_IBT_IMSS_1052/2021	EPI_ISL_1811402	02/04/21	North America / Mexico / Puebla	Human	Female	48	Ambulatory	Nasopharyngeal and oropharyngeal swab
hCoV-19/Mexico/PUE_IBT_IMSS_1054/2021	EPI_ISL_1811403	31/03/21	North America / Mexico / Puebla	Human	Male	71	Ambulatory	Nasopharyngeal and oropharyngeal swab
hCoV-19/Mexico/VER_IBT_IMSS_1056/2021	EPI_ISL_1811404	31/03/21	North America / Mexico / Veracruz	Human	Male	49	Ambulatory	Nasopharyngeal and oropharyngeal swab
hCoV-19/Mexico/CMX_IBT_IMSS_1058/2021	EPI_ISL_1811405	30/03/21	North America / Mexico / Mexico City	Human	Female	59	Ambulatory	Nasopharyngeal and oropharyngeal swab
hCoV-19/Mexico/CMX_IBT_IMSS_1059/2021	EPI_ISL_1811406	05/04/21	North America / Mexico / Mexico City	Human	Female	50	Ambulatory	Nasopharyngeal and oropharyngeal swab
hCoV-19/Mexico/CMX_IBT_IMSS_1060/2021	EPI_ISL_1811407	05/04/21	North America / Mexico / Mexico City	Human	Female	33	Ambulatory	Nasopharyngeal and oropharyngeal swab
hCoV-19/Mexico/CMX_IBT_IMSS_1061/2021	EPI_ISL_1811408	05/04/21	North America / Mexico / Mexico City	Human	Female	48	Ambulatory	Nasopharyngeal and oropharyngeal swab
hCoV-19/Mexico/CMX_IBT_IMSS_1062/2021	EPI_ISL_1811409	05/04/21	North America / Mexico / Mexico City	Human	Male	12	Ambulatory	Nasopharyngeal and oropharyngeal swab
hCoV-19/Mexico/CMX_IBT_IMSS_1063/2021	EPI_ISL_1811410	05/04/21	North America / Mexico / Mexico City	Human	Female	27	Ambulatory	Nasopharyngeal and oropharyngeal swab
hCoV-19/Mexico/CMX_IBT_IMSS_1064/2021	EPI_ISL_1811411	06/04/21	North America / Mexico / Mexico City	Human	Female	31	Hospitalized	Nasopharyngeal swab
hCoV-19/Mexico/MEX_IBT_IMSS_1066/2021	EPI_ISL_1811412	05/04/21	North America / Mexico / State of Mexico	Human	Male	64	Hospitalized	Nasopharyngeal and oropharyngeal swab
hCoV-19/Mexico/AGU_IBT_IMSS_1067/2021	EPI_ISL_1811413	04/04/21	North America / Mexico / Aguascalientes	Human	Male	29	Ambulatory	Nasopharyngeal and oropharyngeal swab
hCoV-19/Mexico/MEX_IBT_IMSS_1068/2021	EPI_ISL_1811414	05/04/21	North America / Mexico / State of Mexico	Human	Male	43	Ambulatory	Nasopharyngeal and oropharyngeal swab
hCoV-19/Mexico/MEX_IBT_IMSS_1069/2021	EPI_ISL_1811415	05/04/21	North America / Mexico / State of Mexico	Human	Female	46	Ambulatory	Nasopharyngeal and oropharyngeal swab
hCoV-19/Mexico/MOR_IBT_IMSS_1070/2021	EPI_ISL_1811416	04/04/21	North America / Mexico / Morelos	Human	Female	24	Ambulatory	Nasopharyngeal and oropharyngeal swab

hCoV-19/Mexico/VER_IBT_IMSS_1071/2021	EPI_ISL_1811417	31/03/21	North America / Mexico / Veracruz	Human	Female	46	Ambulatory	Nasopharyngeal and oropharyngeal swab
hCoV-19/Mexico/VER_IBT_IMSS_1072/2021	EPI_ISL_1811418	05/04/21	North America / Mexico / Veracruz	Human	Male	48	Ambulatory	Nasopharyngeal and oropharyngeal swab
hCoV-19/Mexico/QUE_IBT_IMSS_1074/2021	EPI_ISL_1811420	05/04/21	North America / Mexico / Queretaro	Human	Female	74	Hospitalized	Nasopharyngeal and oropharyngeal swab
hCoV-19/Mexico/HID_IBT_IMSS_1075/2021	EPI_ISL_1811421	03/04/21	North America / Mexico / Hidalgo	Human	Male	64	Hospitalized	Nasopharyngeal and oropharyngeal swab
hCoV-19/Mexico/HID_IBT_IMSS_1077/2021	EPI_ISL_1811422	03/04/21	North America / Mexico / Hidalgo	Human	Male	52	Hospitalized	Nasopharyngeal and oropharyngeal swab
hCoV-19/Mexico/CMX_IBT_IMSS_1078/2021	EPI_ISL_1811423	06/04/21	North America / Mexico / Mexico City	Human	Female	58	Hospitalized	Oropharyngeal swab
hCoV-19/Mexico/CMX_IBT_IMSS_1079/2021	EPI_ISL_1811424	06/04/21	North America / Mexico / Mexico City	Human	Male	63	Hospitalized	Oropharyngeal swab
hCoV-19/Mexico/CMX_IBT_IMSS_1080/2021	EPI_ISL_1811425	06/04/21	North America / Mexico / Mexico City	Human	Female	60	Hospitalized	Oropharyngeal swab
hCoV-19/Mexico/CMX_IBT_IMSS_1081/2021	EPI_ISL_1811426	05/04/21	North America / Mexico / Mexico City	Human	Female	53	Hospitalized	Oropharyngeal swab
hCoV-19/Mexico/MEX_IBT_IMSS_1082/2021	EPI_ISL_1811427	05/04/21	North America / Mexico / State of Mexico	Human	Male	45	Hospitalized	Nasopharyngeal and oropharyngeal swab
hCoV-19/Mexico/MEX_IBT_IMSS_1083/2021	EPI_ISL_1811428	06/04/21	North America / Mexico / State of Mexico	Human	Male	76	Hospitalized	Nasopharyngeal and oropharyngeal swab
hCoV-19/Mexico/MEX_IBT_IMSS_1084/2021	EPI_ISL_1811429	05/04/21	North America / Mexico / State of Mexico	Human	Male	68	Hospitalized	Oropharyngeal swab
hCoV-19/Mexico/MEX_IBT_IMSS_1085/2021	EPI_ISL_1811430	05/04/21	North America / Mexico / State of Mexico	Human	Female	42	Hospitalized	Nasopharyngeal and oropharyngeal swab
hCoV-19/Mexico/MEX_IBT_IMSS_1086/2021	EPI_ISL_1811431	05/04/21	North America / Mexico / State of Mexico	Human	Male	64	Hospitalized	Nasopharyngeal and oropharyngeal swab
hCoV-19/Mexico/SLP_IBT_IMSS_1088/2021	EPI_ISL_1811432	06/04/21	North America / Mexico / San Luis Potosi	Human	Female	24	Ambulatory	Nasopharyngeal and oropharyngeal swab
hCoV-19/Mexico/SLP_IBT_IMSS_1090/2021	EPI_ISL_1811434	04/04/21	North America / Mexico / San Luis Potosi	Human	Male	33	Hospitalized	Nasopharyngeal and oropharyngeal swab
hCoV-19/Mexico/PUE_IBT_IMSS_1091/2021	EPI_ISL_1811435	02/04/21	North America / Mexico / Puebla	Human	Female	32	Ambulatory	Oropharyngeal swab
hCoV-19/Mexico/PUE_IBT_IMSS_1092/2021	EPI_ISL_1811436	02/04/21	North America / Mexico / Puebla	Human	Female	40	Ambulatory	Oropharyngeal swab
hCoV-19/Mexico/PUE_IBT_IMSS_1093/2021	EPI_ISL_1811437	04/04/21	North America / Mexico / Puebla	Human	Female	31	Ambulatory	Oropharyngeal swab
hCoV-19/Mexico/PUE_IBT_IMSS_1094/2021	EPI_ISL_1811438	04/04/21	North America / Mexico / Puebla	Human	Male	53	Ambulatory	Oropharyngeal swab
hCoV-19/Mexico/PUE_IBT_IMSS_1095/2021	EPI_ISL_1811439	04/04/21	North America / Mexico / Puebla	Human	Female	68	Hospitalized	Nasopharyngeal and oropharyngeal swab
hCoV-19/Mexico/PUE_IBT_IMSS_1096/2021	EPI_ISL_1811440	01/04/21	North America / Mexico / Puebla	Human	Female	40	Ambulatory	Oropharyngeal swab
hCoV-19/Mexico/PUE_IBT_IMSS_1098/2021	EPI_ISL_1811441	03/04/21	North America / Mexico / Puebla	Human	Female	76	Ambulatory	Oropharyngeal swab
hCoV-19/Mexico/PUE_IBT_IMSS_1099/2021	EPI_ISL_1811442	03/04/21	North America / Mexico / Puebla	Human	Female	41	Ambulatory	Oropharyngeal swab
hCoV-19/Mexico/PUE_IBT_IMSS_1100/2021	EPI_ISL_1811443	03/04/21	North America / Mexico / Puebla	Human	Male	45	Ambulatory	Oropharyngeal swab
hCoV-19/Mexico/PUE_IBT_IMSS_1101/2021	EPI_ISL_1811444	02/04/21	North America / Mexico / Puebla	Human	Male	62	Hospitalized	Oropharyngeal swab
hCoV-19/Mexico/PUE_IBT_IMSS_1102/2021	EPI_ISL_1811445	04/04/21	North America / Mexico / Puebla	Human	Male	32	Ambulatory	Nasopharyngeal swab
hCoV-19/Mexico/PUE_IBT_IMSS_1103/2021	EPI_ISL_1811446	05/04/21	North America / Mexico / Puebla	Human	Female	46	Ambulatory	Nasopharyngeal and oropharyngeal swab
hCoV-19/Mexico/PUE_IBT_IMSS_1104/2021	EPI_ISL_1811447	05/04/21	North America / Mexico / Puebla	Human	Male	21	Ambulatory	Nasopharyngeal and oropharyngeal swab
hCoV-19/Mexico/PUE_IBT_IMSS_1105/2021	EPI_ISL_1811448	05/04/21	North America / Mexico / Puebla	Human	Male	37	Ambulatory	Nasopharyngeal and oropharyngeal swab
hCoV-19/Mexico/CMX_IBT_IMSS_1108/2021	EPI_ISL_1811450	05/04/21	North America / Mexico / Mexico City	Human	Female	34	Ambulatory	Nasopharyngeal and oropharyngeal swab
hCoV-19/Mexico/CMX_IBT_IMSS_1109/2021	EPI_ISL_1811451	06/04/21	North America / Mexico / Mexico City	Human	Male	42	Ambulatory	Nasopharyngeal and oropharyngeal swab
hCoV-19/Mexico/CMX_IBT_IMSS_1110/2021	EPI_ISL_1811452	06/04/21	North America / Mexico / Mexico City	Human	Male	37	Ambulatory	Nasopharyngeal and oropharyngeal swab
hCoV-19/Mexico/MEX_IBT_IMSS_1111/2021	EPI_ISL_1811453	06/04/21	North America / Mexico / State of Mexico	Human	Male	44	Hospitalized	Nasopharyngeal and oropharyngeal swab
hCoV-19/Mexico/VER_IBT_IMSS_1113/2021	EPI_ISL_1811455	05/04/21	North America / Mexico / Veracruz	Human	Male	77	Hospitalized	Nasopharyngeal and oropharyngeal swab
hCoV-19/Mexico/VER_IBT_IMSS_1114/2021	EPI_ISL_1811456	05/04/21	North America / Mexico / Veracruz	Human	Male	77	Hospitalized	Nasopharyngeal and oropharyngeal swab
hCoV-19/Mexico/CMX_IBT_IMSS_1115/2021	EPI_ISL_1811457	07/04/21	North America / Mexico / Mexico City	Human	Male	31	Ambulatory	Nasopharyngeal and oropharyngeal swab
hCoV-19/Mexico/MEX_IBT_IMSS_1117/2021	EPI_ISL_1811459	06/04/21	North America / Mexico / State of Mexico	Human	Male	36	Hospitalized	Nasopharyngeal swab
hCoV-19/Mexico/VER_IBT_IMSS_1121/2021	EPI_ISL_1811461	04/04/21	North America / Mexico / Veracruz	Human	Female	59	Ambulatory	Nasopharyngeal and oropharyngeal swab
hCoV-19/Mexico/VER_IBT_IMSS_1122/2021	EPI_ISL_1811462	05/04/21	North America / Mexico / Veracruz	Human	Male	39	Hospitalized	Nasopharyngeal and oropharyngeal swab
hCoV-19/Mexico/VER_IBT_IMSS_1123/2021	EPI_ISL_1811463	05/04/21	North America / Mexico / Veracruz	Human	Female	55	Ambulatory	Nasopharyngeal and oropharyngeal swab
hCoV-19/Mexico/AGU_IBT_IMSS_1124/2021	EPI_ISL_1811464	06/04/21	North America / Mexico / Aguascalientes	Human	Male	55	Ambulatory	Nasopharyngeal and oropharyngeal swab
hCoV-19/Mexico/AGU_IBT_IMSS_1126/2021	EPI_ISL_1811466	05/04/21	North America / Mexico / Aguascalientes	Human	Female	44	Ambulatory	Nasopharyngeal and oropharyngeal swab
hCoV-19/Mexico/QUE_IBT_IMSS_1127/2021	EPI_ISL_1811467	02/04/21	North America / Mexico / Queretaro	Human	Male	57	Hospitalized	Nasopharyngeal and oropharyngeal swab

hCoV-19/Mexico/MEX_IBT_IMSS_1128/2021	EPI_ISL_1811468	07/04/21	North America / Mexico / State of Mexico	Human	Male	51	Hospitalized	Nasopharyngeal and oropharyngeal swab
hCoV-19/Mexico/MEX_IBT_IMSS_954/2021	EPI_ISL_1811469	06/04/21	North America / Mexico / State of Mexico	Human	Female	63	Hospitalized	Oropharyngeal swab
hCoV-19/Mexico/CMX_IBT_IMSS_955/2021	EPI_ISL_1811470	06/04/21	North America / Mexico / Mexico City	Human	Female	83	Hospitalized	Oropharyngeal swab
hCoV-19/Mexico/HID_IBT_IMSS_956/2021	EPI_ISL_1811471	06/04/21	North America / Mexico / Hidalgo	Human	Male	48	Hospitalized	Oropharyngeal swab
hCoV-19/Mexico/HID_IBT_IMSS_957/2021	EPI_ISL_1811472	06/04/21	North America / Mexico / Hidalgo	Human	Male	47	Hospitalized	Nasopharyngeal and oropharyngeal swab
hCoV-19/Mexico/HID_IBT_IMSS_958/2021	EPI_ISL_1811473	06/04/21	North America / Mexico / Hidalgo	Human	Female	55	Hospitalized	Nasopharyngeal and oropharyngeal swab
hCoV-19/Mexico/HID_IBT_IMSS_959/2021	EPI_ISL_1811474	06/04/21	North America / Mexico / Hidalgo	Human	Male	69	Hospitalized	Nasopharyngeal and oropharyngeal swab
hCoV-19/Mexico/SLP_IBT_IMSS_960/2021	EPI_ISL_1811475	04/04/21	North America / Mexico / San Luis Potosi	Human	Male	46	Hospitalized	Nasopharyngeal and oropharyngeal swab
hCoV-19/Mexico/MEX_IBT_IMSS_961/2021	EPI_ISL_1811476	06/04/21	North America / Mexico / State of Mexico	Human	Male	61	Hospitalized	Oropharyngeal swab
hCoV-19/Mexico/TLA_IBT_IMSS_964/2021	EPI_ISL_1811478	07/04/21	North America / Mexico / Tlaxcala	Human	Female	79	Hospitalized	Nasopharyngeal and oropharyngeal swab
hCoV-19/Mexico/TLA_IBT_IMSS_965/2021	EPI_ISL_1811479	06/04/21	North America / Mexico / Tlaxcala	Human	Male	39	Hospitalized	Nasopharyngeal and oropharyngeal swab
hCoV-19/Mexico/TLA_IBT_IMSS_966/2021	EPI_ISL_1811480	05/04/21	North America / Mexico / Tlaxcala	Human	Female	57	Hospitalized	Nasopharyngeal and oropharyngeal swab
hCoV-19/Mexico/PUE_IBT_IMSS_967/2021	EPI_ISL_1811481	05/04/21	North America / Mexico / Puebla	Human	Male	61	Hospitalized	Nasopharyngeal and oropharyngeal swab
hCoV-19/Mexico/PUE_IBT_IMSS_968/2021	EPI_ISL_1811482	05/04/21	North America / Mexico / Puebla	Human	Male	75	Hospitalized	Nasopharyngeal and oropharyngeal swab
hCoV-19/Mexico/PUE_IBT_IMSS_969/2021	EPI_ISL_1811483	05/04/21	North America / Mexico / Puebla	Human	Male	61	Hospitalized	Nasopharyngeal and oropharyngeal swab
hCoV-19/Mexico/PUE_IBT_IMSS_970/2021	EPI_ISL_1811484	05/04/21	North America / Mexico / Puebla	Human	Female	47	Hospitalized	Nasopharyngeal and oropharyngeal swab
hCoV-19/Mexico/PUE_IBT_IMSS_971/2021	EPI_ISL_1811485	05/04/21	North America / Mexico / Puebla	Human	Male	39	Hospitalized	Nasopharyngeal and oropharyngeal swab
hCoV-19/Mexico/PUE_IBT_IMSS_972/2021	EPI_ISL_1811486	06/04/21	North America / Mexico / Puebla	Human	Male	23	Ambulatory	Nasopharyngeal and oropharyngeal swab
hCoV-19/Mexico/PUE_IBT_IMSS_974/2021	EPI_ISL_1811487	05/04/21	North America / Mexico / Puebla	Human	Male	39	Ambulatory	Nasopharyngeal and oropharyngeal swab
hCoV-19/Mexico/PUE_IBT_IMSS_975/2021	EPI_ISL_1811488	05/04/21	North America / Mexico / Puebla	Human	Male	74	Ambulatory	Nasopharyngeal and oropharyngeal swab
hCoV-19/Mexico/PUE_IBT_IMSS_976/2021	EPI_ISL_1811489	05/04/21	North America / Mexico / Puebla	Human	Male	25	Ambulatory	Oropharyngeal swab
hCoV-19/Mexico/PUE_IBT_IMSS_977/2021	EPI_ISL_1811490	04/04/21	North America / Mexico / Puebla	Human	Male	52	Hospitalized	Nasopharyngeal and oropharyngeal swab
hCoV-19/Mexico/PUE_IBT_IMSS_978/2021	EPI_ISL_1811491	04/04/21	North America / Mexico / Puebla	Human	Female	36	Hospitalized	Nasopharyngeal and oropharyngeal swab
hCoV-19/Mexico/PUE_IBT_IMSS_980/2021	EPI_ISL_1811493	30/03/21	North America / Mexico / Puebla	Human	Female	58	Hospitalized	Nasopharyngeal and oropharyngeal swab
hCoV-19/Mexico/PUE_IBT_IMSS_981/2021	EPI_ISL_1811494	03/04/21	North America / Mexico / Puebla	Human	Male	23	Hospitalized	Nasopharyngeal and oropharyngeal swab
hCoV-19/Mexico/PUE_IBT_IMSS_982/2021	EPI_ISL_1811495	29/03/21	North America / Mexico / Puebla	Human	Male	56	Hospitalized	Nasopharyngeal and oropharyngeal swab
hCoV-19/Mexico/PUE_IBT_IMSS_983/2021	EPI_ISL_1811496	05/04/21	North America / Mexico / Puebla	Human	Male	37	Ambulatory	Oropharyngeal swab
hCoV-19/Mexico/PUE_IBT_IMSS_985/2021	EPI_ISL_1811497	04/04/21	North America / Mexico / Puebla	Human	Male	35	Ambulatory	Oropharyngeal swab
hCoV-19/Mexico/PUE_IBT_IMSS_986/2021	EPI_ISL_1811498	05/04/21	North America / Mexico / Puebla	Human	Male	58	Ambulatory	Oropharyngeal swab
hCoV-19/Mexico/PUE_IBT_IMSS_987/2021	EPI_ISL_1811499	06/04/21	North America / Mexico / Puebla	Human	Male	48	Hospitalized	Nasopharyngeal and oropharyngeal swab
hCoV-19/Mexico/CMX_IBT_IMSS_989/2021	EPI_ISL_1811501	07/04/21	North America / Mexico / Mexico City	Human	Female	80	Hospitalized	Nasopharyngeal swab
hCoV-19/Mexico/CMX_IBT_IMSS_990/2021	EPI_ISL_1811502	07/04/21	North America / Mexico / Mexico City	Human	Male	54	Hospitalized	Oropharyngeal swab
hCoV-19/Mexico/CMX_IBT_IMSS_991/2021	EPI_ISL_1811503	07/04/21	North America / Mexico / Mexico City	Human	Female	65	Hospitalized	Nasopharyngeal swab
hCoV-19/Mexico/CMX_IBT_IMSS_993/2021	EPI_ISL_1811505	07/04/21	North America / Mexico / Mexico City	Human	Male	45	Hospitalized	Nasopharyngeal and oropharyngeal swab
hCoV-19/Mexico/CMX_IBT_IMSS_994/2021	EPI_ISL_1811506	08/04/21	North America / Mexico / Mexico City	Human	Female	55	Ambulatory	Nasopharyngeal and oropharyngeal swab
hCoV-19/Mexico/MEX_IBT_IMSS_996/2021	EPI_ISL_1811507	09/04/21	North America / Mexico / State of Mexico	Human	Female	26	Ambulatory	Nasopharyngeal and oropharyngeal swab
hCoV-19/Mexico/MEX_IBT_IMSS_997/2021	EPI_ISL_1811508	04/04/21	North America / Mexico / State of Mexico	Human	Male	68	Hospitalized	Nasopharyngeal and oropharyngeal swab
hCoV-19/Mexico/JAL_IBT_IMSS_998/2021	EPI_ISL_1811509	08/04/21	North America / Mexico / Jalisco	Human	Male	73	Hospitalized	Nasopharyngeal and oropharyngeal swab
hCoV-19/Mexico/MEX_IBT_IMSS_999/2021	EPI_ISL_1811510	08/04/21	North America / Mexico / State of Mexico	Human	Female	57	Hospitalized	Nasopharyngeal and oropharyngeal swab
hCoV-19/Mexico/CMX_IBT_IMSS_1002/2021	EPI_ISL_1811511	09/04/21	North America / Mexico / Mexico City	Human	Female	50	Ambulatory	Oropharyngeal swab
hCoV-19/Mexico/MEX_IBT_IMSS_1003/2021	EPI_ISL_1811512	09/04/21	North America / Mexico / State of Mexico	Human	Female	49	Ambulatory	Nasopharyngeal and oropharyngeal swab
hCoV-19/Mexico/MEX_IBT_IMSS_1005/2021	EPI_ISL_1811513	07/04/21	North America / Mexico / State of Mexico	Human	Female	78	Hospitalized	Nasopharyngeal and oropharyngeal swab
hCoV-19/Mexico/MEX_IBT_IMSS_1006/2021	EPI_ISL_1811514	06/04/21	North America / Mexico / State of Mexico	Human	Female	46	Ambulatory	Nasopharyngeal and oropharyngeal swab
hCoV-19/Mexico/MEX_IBT_IMSS_1007/2021	EPI_ISL_1811515	08/04/21	North America / Mexico / State of Mexico	Human	Female	63	Hospitalized	Nasopharyngeal and oropharyngeal swab

hCoV-19/Mexico/MEX_IBT_IMSS_1008/2021	EPI_ISL_1811516	08/04/21	North America / Mexico / State of Mexico	Human	Male	64	Hospitalized	Nasopharyngeal and oropharyngeal swab
hCoV-19/Mexico/CMX_IBT_IMSS_1009/2021	EPI_ISL_1811517	09/04/21	North America / Mexico / Mexico City	Human	Male	37	Ambulatory	Oropharyngeal swab
hCoV-19/Mexico/VER_IBT_IMSS_1013/2021	EPI_ISL_1811520	05/04/21	North America / Mexico / Veracruz	Human	Male	42	Ambulatory	Nasopharyngeal and oropharyngeal swab
hCoV-19/Mexico/MEX_IBT_IMSS_1016/2021	EPI_ISL_1811522	11/04/21	North America / Mexico / State of Mexico	Human	Female	67	Hospitalized	Nasopharyngeal and oropharyngeal swab
hCoV-19/Mexico/GRO_IBT_IMSS_1017/2021	EPI_ISL_1811523	09/04/21	North America / Mexico / Guerrero	Human	Female	68	Hospitalized	Nasopharyngeal and oropharyngeal swab
hCoV-19/Mexico/BCS_IBT_IMSS_1018/2021	EPI_ISL_1811524	09/04/21	North America / Mexico / Baja California Sur	Human	Male	45	Ambulatory	Nasopharyngeal and oropharyngeal swab
hCoV-19/Mexico/CMX_IBT_IMSS_1019/2021	EPI_ISL_1811525	10/04/21	North America / Mexico / Mexico City	Human	Male	76	Hospitalized	Nasopharyngeal swab
hCoV-19/Mexico/CMX_IBT_IMSS_1020/2021	EPI_ISL_1811526	10/04/21	North America / Mexico / Mexico City	Human	Female	56	Hospitalized	Nasopharyngeal swab
hCoV-19/Mexico/MEX_IBT_IMSS_1021/2021	EPI_ISL_1811527	10/04/21	North America / Mexico / State of Mexico	Human	Male	64	Hospitalized	Nasopharyngeal and oropharyngeal swab
hCoV-19/Mexico/TLA_IBT_IMSS_1022/2021	EPI_ISL_1811528	08/04/21	North America / Mexico / Tlaxcala	Human	Male	77	Hospitalized	Nasopharyngeal and oropharyngeal swab
hCoV-19/Mexico/TLA_IBT_IMSS_1023/2021	EPI_ISL_1811529	11/04/21	North America / Mexico / Tlaxcala	Human	Female	90	Hospitalized	Nasopharyngeal and oropharyngeal swab
hCoV-19/Mexico/TLA_IBT_IMSS_1025/2021	EPI_ISL_1811530	11/04/21	North America / Mexico / Tlaxcala	Human	Male	48	Hospitalized	Nasopharyngeal and oropharyngeal swab
hCoV-19/Mexico/BCS_IBT_IMSS_1026/2021	EPI_ISL_1811531	10/04/21	North America / Mexico / Baja California Sur	Human	Male	32	Ambulatory	Nasopharyngeal and oropharyngeal swab
hCoV-19/Mexico/PUE_IBT_IMSS_1027/2021	EPI_ISL_1811532	10/04/21	North America / Mexico / Puebla	Human	Female	92	Hospitalized	Nasopharyngeal and oropharyngeal swab
hCoV-19/Mexico/PUE_IBT_IMSS_1028/2021	EPI_ISL_1811533	10/04/21	North America / Mexico / Puebla	Human	Male	94	Hospitalized	Nasopharyngeal and oropharyngeal swab
hCoV-19/Mexico/PUE_IBT_IMSS_1029/2021	EPI_ISL_1811534	10/04/21	North America / Mexico / Puebla	Human	Male	82	Hospitalized	Nasopharyngeal and oropharyngeal swab
hCoV-19/Mexico/PUE_IBT_IMSS_1030/2021	EPI_ISL_1811535	09/04/21	North America / Mexico / Puebla	Human	Female	63	Hospitalized	Nasopharyngeal and oropharyngeal swab
hCoV-19/Mexico/PUE_IBT_IMSS_1031/2021	EPI_ISL_1811536	09/04/21	North America / Mexico / Puebla	Human	Male	49	Ambulatory	Oropharyngeal swab
hCoV-19/Mexico/PUE_IBT_IMSS_1032/2021	EPI_ISL_1811537	08/04/21	North America / Mexico / Puebla	Human	Female	92	Ambulatory	Oropharyngeal swab
hCoV-19/Mexico/PUE_IBT_IMSS_1033/2021	EPI_ISL_1811538	09/04/21	North America / Mexico / Puebla	Human	Female	34	Ambulatory	Oropharyngeal swab
hCoV-19/Mexico/PUE_IBT_IMSS_1034/2021	EPI_ISL_1811539	31/03/21	North America / Mexico / Puebla	Human	Female	42	Ambulatory	Nasopharyngeal and oropharyngeal swab
hCoV-19/Mexico/PUE_IBT_IMSS_1036/2021	EPI_ISL_1811540	05/04/21	North America / Mexico / Puebla	Human	Female	59	Hospitalized	Nasopharyngeal and oropharyngeal swab
hCoV-19/Mexico/PUE_IBT_IMSS_1037/2021	EPI_ISL_1811541	05/04/21	North America / Mexico / Puebla	Human	Female	45	Ambulatory	Nasopharyngeal and oropharyngeal swab
hCoV-19/Mexico/PUE_IBT_IMSS_1038/2021	EPI_ISL_1811542	07/04/21	North America / Mexico / Puebla	Human	Female	37	Ambulatory	Nasopharyngeal and oropharyngeal swab
hCoV-19/Mexico/PUE_IBT_IMSS_1040/2021	EPI_ISL_1811543	09/04/21	North America / Mexico / Puebla	Human	Male	51	Ambulatory	Nasopharyngeal and oropharyngeal swab
hCoV-19/Mexico/PUE_IBT_IMSS_1041/2021	EPI_ISL_1811544	09/04/21	North America / Mexico / Puebla	Human	Male	39	Ambulatory	Nasopharyngeal and oropharyngeal swab
hCoV-19/Mexico/CMX_IBT_IMSS_1042/2021	EPI_ISL_1811545	12/04/21	North America / Mexico / Mexico City	Human	Female	41	Ambulatory	Nasopharyngeal and oropharyngeal swab
hCoV-19/Mexico/QUE-InDRE_FB12853_S1527/2021	EPI_ISL_1821102	02/04/21	North America / Mexico / Queretaro	Human	Female	27	Hospitalized	Oropharyngeal swab
hCoV-19/Mexico/CHH-InDRE_FB12873_S1528/2021	EPI_ISL_1821103	03/04/21	North America / Mexico / Chihuahua	Human	Male	26	Released	Oropharyngeal swab
hCoV-19/Mexico/BCS-InDRE_FB12883_S1529/2021	EPI_ISL_1821104	03/04/21	North America / Mexico / Baja California Sur	Human	Male	27	Released	Oropharyngeal swab
hCoV-19/Mexico/BCS-InDRE_FB12888_S1530/2021	EPI_ISL_1821105	03/04/21	North America / Mexico / Baja California Sur	Human	Female	76	Released	Oropharyngeal swab
hCoV-19/Mexico/CMX-InDRE_FB12939_S1531/2021	EPI_ISL_1821106	01/04/21	North America / Mexico / Mexico City	Human	Female	30	Released	Oropharyngeal swab
hCoV-19/Mexico/COL-InDRE_FB12960_S1532/2021	EPI_ISL_1821107	01/04/21	North America / Mexico / Colima	Human	Male	68	Hospitalized	Oropharyngeal swab
hCoV-19/Mexico/GUA-InDRE_FB13062_S1533/2021	EPI_ISL_1821108	05/04/21	North America / Mexico / Guanajuato	Human	Male	63	Deceased	Oropharyngeal swab
hCoV-19/Mexico/GUA-InDRE_FB13064_S1534/2021	EPI_ISL_1821109	07/04/21	North America / Mexico / Guanajuato	Human	Female	72	Hospitalized	Oropharyngeal swab
hCoV-19/Mexico/GUA-InDRE_FB13072_S1535/2021	EPI_ISL_1821110	07/04/21	North America / Mexico / Guanajuato	Human	Male	20	Released	Oropharyngeal swab
hCoV-19/Mexico/GUA-InDRE_FB13074_S1536/2021	EPI_ISL_1821111	09/04/21	North America / Mexico / Guanajuato	Human	Male	51	Released	Oropharyngeal swab
hCoV-19/Mexico/NLE-InDRE_FB13079_S1537/2021	EPI_ISL_1821112	06/04/21	North America / Mexico / Nuevo Leon	Human	Female	53	Hospitalized	Oropharyngeal swab
hCoV-19/Mexico/NLE-InDRE_FB13081_S1539/2021	EPI_ISL_1821113	06/04/21	North America / Mexico / Nuevo Leon	Human	Male	71	Hospitalized	Oropharyngeal swab
hCoV-19/Mexico/NLE-InDRE_FB13084_S1540/2021	EPI_ISL_1821114	05/04/21	North America / Mexico / Nuevo Leon	Human	Male	31	Released	Oropharyngeal swab
hCoV-19/Mexico/NLE-InDRE_FB13085_S1541/2021	EPI_ISL_1821115	05/04/21	North America / Mexico / Nuevo Leon	Human	Male	46	Released	Oropharyngeal swab
hCoV-19/Mexico/NLE-InDRE_FB13086_S1542/2021	EPI_ISL_1821116	06/04/21	North America / Mexico / Nuevo Leon	Human	Female	35	Released	Oropharyngeal swab
hCoV-19/Mexico/NLE-InDRE_FB13090_S1543/2021	EPI_ISL_1821117	07/04/21	North America / Mexico / Nuevo Leon	Human	Female	58	Released	Oropharyngeal swab
hCoV-19/Mexico/NLE-InDRE_FB13091_S1544/2021	EPI_ISL_1821118	07/04/21	North America / Mexico / Nuevo Leon	Human	Male	61	Released	Oropharyngeal swab

hCoV-19/Mexico/PUE-InDRE_FB13093_S1546/2021	EPI_ISL_1821119	08/04/21	North America / Mexico / Puebla	Human	Male	56	Released	Oropharyngeal swab
hCoV-19/Mexico/PUE-InDRE_FB13096_S1547/2021	EPI_ISL_1821120	08/04/21	North America / Mexico / Puebla	Human	Female	45	Live	Oropharyngeal swab
hCoV-19/Mexico/PUE-InDRE_FB13098_S1548/2021	EPI_ISL_1821121	08/04/21	North America / Mexico / Puebla	Human	Male	31	Hospitalized	Oropharyngeal swab
hCoV-19/Mexico/PUE-InDRE_FB13099_S1549/2021	EPI_ISL_1821122	09/04/21	North America / Mexico / Puebla	Human	Female	98	Deceased	Oropharyngeal swab
hCoV-19/Mexico/GRO-InDRE_FB13100_S1550/2021	EPI_ISL_1821123	09/04/21	North America / Mexico / Guerrero	Human	Male	77	Hospitalized	Oropharyngeal swab
hCoV-19/Mexico/PUE-InDRE_FB13101_S1551/2021	EPI_ISL_1821124	10/04/21	North America / Mexico / Puebla	Human	Female	60	Deceased	Oropharyngeal swab
hCoV-19/Mexico/CHH-InDRE_FB13108_S1552/2021	EPI_ISL_1821125	05/04/21	North America / Mexico / Chihuahua	Human	Male	38	Live	Oropharyngeal swab
hCoV-19/Mexico/SON-InDRE_FB13110_S1553/2021	EPI_ISL_1821126	08/04/21	North America / Mexico / Sonora	Human	Male	59	Hospitalized	Oropharyngeal swab
hCoV-19/Mexico/CHH-InDRE_FB13112_S1554/2021	EPI_ISL_1821127	05/04/21	North America / Mexico / Chihuahua	Human	Male	18	Released	Oropharyngeal swab
hCoV-19/Mexico/SON-InDRE_FB13113_S1555/2021	EPI_ISL_1821128	08/04/21	North America / Mexico / Sonora	Human	Female	40	Released	Oropharyngeal swab
hCoV-19/Mexico/JAL-InDRE_FB13120_S1556/2021	EPI_ISL_1821129	08/04/21	North America / Mexico / Jalisco	Human	Male	20	Released	Oropharyngeal swab
hCoV-19/Mexico/JAL-InDRE_FB13121_S1557/2021	EPI_ISL_1821130	08/04/21	North America / Mexico / Jalisco	Human	Male	83	Hospitalized	Oropharyngeal swab
hCoV-19/Mexico/JAL-InDRE_FB13122_S1558/2021	EPI_ISL_1821131	11/04/21	North America / Mexico / Jalisco	Human	Female	46	Released	Oropharyngeal swab
hCoV-19/Mexico/QUE-InDRE_FB13125_S1559/2021	EPI_ISL_1821132	06/04/21	North America / Mexico / Queretaro	Human	Female	42	Released	Oropharyngeal swab
hCoV-19/Mexico/QUE-InDRE_FB13127_S1560/2021	EPI_ISL_1821133	06/04/21	North America / Mexico / Queretaro	Human	Male	73	Deceased	Oropharyngeal swab
hCoV-19/Mexico/QUE-InDRE_FB13128_S1561/2021	EPI_ISL_1821134	06/04/21	North America / Mexico / Queretaro	Human	Female	59	Hospitalized	Oropharyngeal swab
hCoV-19/Mexico/QUE-InDRE_FB13137_S1563/2021	EPI_ISL_1821135	11/04/21	North America / Mexico / Queretaro	Human	Male	58	Hospitalized	Oropharyngeal swab
hCoV-19/Mexico/QUE-InDRE_FB13141_S1564/2021	EPI_ISL_1821136	09/04/21	North America / Mexico / Queretaro	Human	Female	57	Released	Oropharyngeal swab
hCoV-19/Mexico/MOR-InDRE_FB13150_S1565/2021	EPI_ISL_1821137	04/04/21	North America / Mexico / Morelos	Human	Male	56	Hospitalized	Oropharyngeal swab
hCoV-19/Mexico/MOR-InDRE_FB13156_S1566/2021	EPI_ISL_1821138	05/04/21	North America / Mexico / Morelos	Human	Male	79	Hospitalized	Oropharyngeal swab
hCoV-19/Mexico/MOR-InDRE_FB13158_S1567/2021	EPI_ISL_1821139	05/04/21	North America / Mexico / Morelos	Human	Male	63	Hospitalized	Oropharyngeal swab
hCoV-19/Mexico/MOR-InDRE_FB13160_S1568/2021	EPI_ISL_1821140	06/04/21	North America / Mexico / Morelos	Human	Female	71	Hospitalized	Oropharyngeal swab
hCoV-19/Mexico/MOR-InDRE_FB13161_S1569/2021	EPI_ISL_1821141	06/04/21	North America / Mexico / Morelos	Human	Female	63	Hospitalized	Oropharyngeal swab
hCoV-19/Mexico/MOR-InDRE_FB13162_S1570/2021	EPI_ISL_1821142	06/04/21	North America / Mexico / Morelos	Human	Male	27	Hospitalized	Oropharyngeal swab
hCoV-19/Mexico/MOR-InDRE_FB13164_S1571/2021	EPI_ISL_1821143	07/04/21	North America / Mexico / Morelos	Human	Female	60	Hospitalized	Oropharyngeal swab
hCoV-19/Mexico/MOR-InDRE_FB13165_S1572/2021	EPI_ISL_1821144	07/04/21	North America / Mexico / Morelos	Human	Female	78	Hospitalized	Oropharyngeal swab
hCoV-19/Mexico/MOR-InDRE_FB13166_S1573/2021	EPI_ISL_1821145	07/04/21	North America / Mexico / Morelos	Human	Male	58	Hospitalized	Oropharyngeal swab
hCoV-19/Mexico/MOR-InDRE_FB13169_S1574/2021	EPI_ISL_1821146	08/04/21	North America / Mexico / Morelos	Human	Male	58	Hospitalized	Oropharyngeal swab
hCoV-19/Mexico/MOR-InDRE_FB13172_S1575/2021	EPI_ISL_1821147	09/04/21	North America / Mexico / Morelos	Human	Male	71	Hospitalized	Oropharyngeal swab
hCoV-19/Mexico/MOR-InDRE_FB13173_S1576/2021	EPI_ISL_1821148	08/04/21	North America / Mexico / Morelos	Human	Male	48	Released	Oropharyngeal swab
hCoV-19/Mexico/GRO-InDRE_FB13175_S1577/2021	EPI_ISL_1821149	03/04/21	North America / Mexico / Guerrero	Human	Male	65	Hospitalized	Oropharyngeal swab
hCoV-19/Mexico/GRO-InDRE_FB13177_S1578/2021	EPI_ISL_1821150	05/04/21	North America / Mexico / Guerrero	Human	Male	77	Live	Oropharyngeal swab
hCoV-19/Mexico/GRO-InDRE_FB13178_S1579/2021	EPI_ISL_1821151	06/04/21	North America / Mexico / Guerrero	Human	Male	84	Hospitalized	Oropharyngeal swab
hCoV-19/Mexico/YUC-InDRE_FB13181_S1580/2021	EPI_ISL_1821152	12/04/21	North America / Mexico / YucatÃ¡n	Human	Male	21	Released	Oropharyngeal swab
hCoV-19/Mexico/YUC-InDRE_FB13182_S1581/2021	EPI_ISL_1821153	12/04/21	North America / Mexico / YucatÃ¡n	Human	Female	24	Released	Oropharyngeal swab
hCoV-19/Mexico/YUC-InDRE_FB13184_S1582/2021	EPI_ISL_1821154	11/04/21	North America / Mexico / YucatÃ¡n	Human	Female	76	Released	Oropharyngeal swab
hCoV-19/Mexico/YUC-InDRE_FB13186_S1583/2021	EPI_ISL_1821155	12/04/21	North America / Mexico / YucatÃ¡n	Human	Female	36	Released	Oropharyngeal swab
hCoV-19/Mexico/SIN-InDRE_FB13194_S1584/2021	EPI_ISL_1821156	10/04/21	North America / Mexico / Sinaloa	Human	Female	45	Hospitalized	Oropharyngeal swab
hCoV-19/Mexico/SIN-InDRE_FB13195_S1585/2021	EPI_ISL_1821157	08/04/21	North America / Mexico / Sinaloa	Human	Male	60	Released	Oropharyngeal swab
hCoV-19/Mexico/TAM-InDRE_FB13198_S1586/2021	EPI_ISL_1821158	09/04/21	North America / Mexico / Tamaulipas	Human	Male	79	Hospitalized	Oropharyngeal swab
hCoV-19/Mexico/TAM-InDRE_FB13200_S1587/2021	EPI_ISL_1821159	04/04/21	North America / Mexico / Tamaulipas	Human	Female	36	Hospitalized	Oropharyngeal swab
hCoV-19/Mexico/TAM-InDRE_FB13203_S1588/2021	EPI_ISL_1821160	05/04/21	North America / Mexico / Tamaulipas	Human	Male	22	Released	Oropharyngeal swab
hCoV-19/Mexico/NAY-InDRE_FB13205_S1589/2021	EPI_ISL_1821161	10/04/21	North America / Mexico / Nayarit	Human	Female	37	Hospitalized	Oropharyngeal swab
hCoV-19/Mexico/OAX-InDRE_FB13221_S1590/2021	EPI_ISL_1821162	03/04/21	North America / Mexico / Oaxaca	Human	Female	64	Hospitalized	Oropharyngeal swab

hCoV-19/Mexico/VER-InDRE_FB13229_S1591/2021	EPI_ISL_1821163	05/04/21	North America / Mexico / Veracruz	Human	Male	85	Deceased	Oropharyngeal swab
hCoV-19/Mexico/VER-InDRE_FB13230_S1592/2021	EPI_ISL_1821164	06/04/21	North America / Mexico / Veracruz	Human	Male	65	Live	Oropharyngeal swab
hCoV-19/Mexico/VER-InDRE_FB13233_S1593/2021	EPI_ISL_1821165	05/04/21	North America / Mexico / Veracruz	Human	Male	26	Released	Oropharyngeal swab
hCoV-19/Mexico/TLA-InDRE_FB13239_S1594/2021	EPI_ISL_1821166	08/04/21	North America / Mexico / Tlaxcala	Human	Male	37	Released	Oropharyngeal swab
hCoV-19/Mexico/CHH-InDRE_FB13250_S1595/2021	EPI_ISL_1821167	06/04/21	North America / Mexico / Chihuahua	Human	Female	80	Hospitalized	Oropharyngeal swab
hCoV-19/Mexico/CHH-InDRE_FB13254_S1596/2021	EPI_ISL_1821168	12/04/21	North America / Mexico / Chihuahua	Human	Male	41	Released	Oropharyngeal swab
hCoV-19/Mexico/CMX-InDRE_FB13258_S1597/2021	EPI_ISL_1821169	07/04/21	North America / Mexico / Mexico City	Human	Male	56	Hospitalized	Oropharyngeal swab
hCoV-19/Mexico/CMX-InDRE_FB13259_S1598/2021	EPI_ISL_1821170	08/04/21	North America / Mexico / Mexico City	Human	Male	51	Hospitalized	Oropharyngeal swab
hCoV-19/Mexico/ROO-InDRE_FB13270_S1599/2021	EPI_ISL_1821171	06/04/21	North America / Mexico / Quintana Roo	Human	Male	66	Deceased	Oropharyngeal swab
hCoV-19/Mexico/ROO-InDRE_FB13272_S1600/2021	EPI_ISL_1821172	05/04/21	North America / Mexico / Quintana Roo	Human	Male	79	Hospitalized	Oropharyngeal swab
hCoV-19/Mexico/ZAC-InDRE_FB13275_S1601/2021	EPI_ISL_1821173	12/04/21	North America / Mexico / Zacatecas	Human	Female	20	Released	Oropharyngeal swab
hCoV-19/Mexico/ZAC-InDRE_FB13276_S1602/2021	EPI_ISL_1821174	13/04/21	North America / Mexico / Zacatecas	Human	Female	12	Released	Oropharyngeal swab
hCoV-19/Mexico/ZAC-InDRE_FB13277_S1603/2021	EPI_ISL_1821175	13/04/21	North America / Mexico / Zacatecas	Human	Male	47	Released	Oropharyngeal swab
hCoV-19/Mexico/ZAC-InDRE_FB13279_S1604/2021	EPI_ISL_1821176	13/04/21	North America / Mexico / Zacatecas	Human	Female	50	Released	Oropharyngeal swab
hCoV-19/Mexico/ZAC-InDRE_FB13280_S1605/2021	EPI_ISL_1821177	13/04/21	North America / Mexico / Zacatecas	Human	Female	77	Released	Oropharyngeal swab
hCoV-19/Mexico/ZAC-InDRE_FB13281_S1606/2021	EPI_ISL_1821178	07/04/21	North America / Mexico / Zacatecas	Human	Female	59	Hospitalized	Oropharyngeal swab
hCoV-19/Mexico/ZAC-InDRE_FB13283_S1607/2021	EPI_ISL_1821179	08/04/21	North America / Mexico / Zacatecas	Human	Female	29	Hospitalized	Oropharyngeal swab
hCoV-19/Mexico/BCS-InDRE_FB13288_S1608/2021	EPI_ISL_1821180	10/04/21	North America / Mexico / Baja California Sur	Human	Male	46	Released	Oropharyngeal swab
hCoV-19/Mexico/BCS-InDRE_FB13289_S1609/2021	EPI_ISL_1821181	10/04/21	North America / Mexico / Baja California Sur	Human	Male	36	Released	Oropharyngeal swab
hCoV-19/Mexico/BCS-InDRE_FB13291_S1610/2021	EPI_ISL_1821182	10/04/21	North America / Mexico / Baja California Sur	Human	Female	33	Released	Oropharyngeal swab
hCoV-19/Mexico/BCS-InDRE_FB13292_S1611/2021	EPI_ISL_1821183	10/04/21	North America / Mexico / Baja California Sur	Human	Female	17	Released	Oropharyngeal swab
hCoV-19/Mexico/BCS-InDRE_FB13293_S1612/2021	EPI_ISL_1821184	10/04/21	North America / Mexico / Baja California Sur	Human	Male	12	Released	Oropharyngeal swab
hCoV-19/Mexico/JAL-InDRE_FB13302_S1613/2021	EPI_ISL_1821185	14/04/21	North America / Mexico / Jalisco	Human	Female	79	Released	Oropharyngeal swab
hCoV-19/Mexico/JAL-InDRE_FB13303_S1614/2021	EPI_ISL_1821186	07/04/21	North America / Mexico / Jalisco	Human	Male	81	Hospitalized	Oropharyngeal swab
hCoV-19/Mexico/CMX-InDRE_FB13304_S1615/2021	EPI_ISL_1821187	03/04/21	North America / Mexico / Mexico City	Human	Female	36	Released	Oropharyngeal swab
hCoV-19/Mexico/CMX-InDRE_FB13305_S1616/2021	EPI_ISL_1821188	02/04/21	North America / Mexico / Mexico City	Human	Female	48	Released	Oropharyngeal swab
hCoV-19/Mexico/CMX-InDRE_FB13309_S1617/2021	EPI_ISL_1821189	05/04/21	North America / Mexico / Mexico City	Human	Female	45	Live	Oropharyngeal swab
hCoV-19/Mexico/CMX-InDRE_FB13315_S1618/2021	EPI_ISL_1821190	06/04/21	North America / Mexico / Mexico City	Human	Male	58	Released	Oropharyngeal swab
hCoV-19/Mexico/QUE-InDRE_FB11295_S1619/2021	EPI_ISL_1821191	06/04/21	North America / Mexico / Queretaro	Human	Male	62	unknown	Oropharyngeal swab
hCoV-19/Mexico/QUE-InDRE_FB11296_S1620/2021	EPI_ISL_1821192	06/04/21	North America / Mexico / Queretaro	Human	Male	12	unknown	Oropharyngeal swab
hCoV-19/Mexico/TAB-InDRE_FB13054_S1621/2021	EPI_ISL_1821193	30/03/21	North America / Mexico / Tabasco	Human	Male	42	unknown	Oropharyngeal swab
hCoV-19/Mexico/HID-InDRE_FB12659_S1624/2021	EPI_ISL_1821194	31/03/21	North America / Mexico / Hidalgo	Human	Female	38	Live	Oropharyngeal swab
hCoV-19/Mexico/HID-InDRE_FB12660_S1625/2021	EPI_ISL_1821195	02/04/21	North America / Mexico / Hidalgo	Human	Female	52	Released	Oropharyngeal swab
hCoV-19/Mexico/HID-InDRE_FB12661_S1626/2021	EPI_ISL_1821196	03/04/21	North America / Mexico / Hidalgo	Human	Male	56	Deceased	Oropharyngeal swab
hCoV-19/Mexico/JAL-InDRE_FB13056_S1627/2021	EPI_ISL_1821197	27/03/21	North America / Mexico / Jalisco	Human	Male	53	unknown	Oropharyngeal swab
hCoV-19/Mexico/JAL-InDRE_FB13058_S1628/2021	EPI_ISL_1821198	30/03/21	North America / Mexico / Jalisco	Human	Female	37	unknown	Oropharyngeal swab
hCoV-19/Mexico/SIN-InDRE_FB13059_S1629/2021	EPI_ISL_1821199	31/03/21	North America / Mexico / Sinaloa	Human	Male	57	Released	Oropharyngeal swab
hCoV-19/Mexico/JAL-InDRE_FB13060_S1630/2021	EPI_ISL_1821200	02/04/21	North America / Mexico / Jalisco	Human	Male	64	unknown	Oropharyngeal swab
hCoV-19/Mexico/BCN-InDRE_FB13333_S1631/2021	EPI_ISL_1821201	10/04/21	North America / Mexico / Baja California	Human	Female	26	Released	Oropharyngeal swab
hCoV-19/Mexico/BCN-InDRE_FB13334_S1632/2021	EPI_ISL_1821202	12/04/21	North America / Mexico / Baja California	Human	Female	37	Released	Oropharyngeal swab
hCoV-19/Mexico/BCN-InDRE_FB13335_S1633/2021	EPI_ISL_1821203	13/04/21	North America / Mexico / Baja California	Human	Female	66	Released	Oropharyngeal swab
hCoV-19/Mexico/CMX-INER-INMEGEN-00065/2021	EPI_ISL_1824419	19/02/21	North America / Mexico / Mexico City	Human	Male	70	unknown	
hCoV-19/Mexico/CMX-INER-INMEGEN-00066/2021	EPI_ISL_1824420	19/02/21	North America / Mexico / Mexico City	Human	Male	64	unknown	
hCoV-19/Mexico/CMX-INER-INMEGEN-00067/2021	EPI_ISL_1824421	19/02/21	North America / Mexico / Mexico City	Human	Female	62	unknown	

hCoV-19/Mexico/CMX-INER-INMEGEN-00068/2021	EPI_ISL_1824422	19/02/21	North America / Mexico / Mexico City	Human	Male	48	unknown
hCoV-19/Mexico/CMX-INER-INMEGEN-00069/2021	EPI_ISL_1824423	19/02/21	North America / Mexico / Mexico City	Human	Male	37	unknown
hCoV-19/Mexico/CMX-INER-INMEGEN-00070/2021	EPI_ISL_1824424	19/02/21	North America / Mexico / Mexico City	Human	Male	16	unknown
hCoV-19/Mexico/CMX-INER-INMEGEN-00071/2021	EPI_ISL_1824425	19/02/21	North America / Mexico / Mexico City	Human	Female	79	unknown
hCoV-19/Mexico/CMX-INER-INMEGEN-00072/2021	EPI_ISL_1824426	19/02/21	North America / Mexico / Mexico City	Human	Female	54	unknown
hCoV-19/Mexico/CMX-INER-INMEGEN-00073/2021	EPI_ISL_1824427	19/02/21	North America / Mexico / Mexico City	Human	Female	33	unknown
hCoV-19/Mexico/CMX-INER-INMEGEN-00075/2021	EPI_ISL_1824428	19/02/21	North America / Mexico / Mexico City	Human	Female	59	unknown
hCoV-19/Mexico/CMX-INER-INMEGEN-00076/2021	EPI_ISL_1824429	19/02/21	North America / Mexico / Mexico City	Human	Male	73	unknown
hCoV-19/Mexico/CMX-INER-INMEGEN-00077/2021	EPI_ISL_1824430	18/03/21	North America / Mexico / Mexico City	Human	Male	56	unknown
hCoV-19/Mexico/CMX-INER-INMEGEN-00078/2021	EPI_ISL_1824431	18/03/21	North America / Mexico / Mexico City	Human	Male	58	unknown
hCoV-19/Mexico/CMX-INER-INMEGEN-00079/2021	EPI_ISL_1824432	18/03/21	North America / Mexico / Mexico City	Human	Male	65	unknown
hCoV-19/Mexico/CMX-INER-INMEGEN-00081/2021	EPI_ISL_1824433	18/03/21	North America / Mexico / Mexico City	Human	Female	74	unknown
hCoV-19/Mexico/CMX-INER-INMEGEN-00082/2021	EPI_ISL_1824434	18/03/21	North America / Mexico / Mexico City	Human	Female	53	unknown
hCoV-19/Mexico/CMX-INER-INMEGEN-00083/2021	EPI_ISL_1824435	18/03/21	North America / Mexico / Mexico City	Human	Female	36	unknown
hCoV-19/Mexico/CMX-INER-INMEGEN-00084/2021	EPI_ISL_1824436	18/03/21	North America / Mexico / Mexico City	Human	Male	59	unknown
hCoV-19/Mexico/CMX-INER-INMEGEN-00085/2021	EPI_ISL_1824437	18/03/21	North America / Mexico / Mexico City	Human	Male	47	unknown
hCoV-19/Mexico/CMX-INER-INMEGEN-00086/2021	EPI_ISL_1824438	18/03/21	North America / Mexico / Mexico City	Human	Male	66	unknown
hCoV-19/Mexico/CMX-INER-INMEGEN-00089/2021	EPI_ISL_1824439	11/03/21	North America / Mexico / Mexico City	Human	Female	59	unknown
hCoV-19/Mexico/CMX-INER-INMEGEN-00090/2021	EPI_ISL_1824440	11/03/21	North America / Mexico / Mexico City	Human	Male	54	unknown
hCoV-19/Mexico/CMX-INER-INMEGEN-00091/2021	EPI_ISL_1824441	12/03/21	North America / Mexico / Mexico City	Human	Female	52	unknown
hCoV-19/Mexico/CMX-INER-INMEGEN-00092/2021	EPI_ISL_1824442	17/02/21	North America / Mexico / Mexico City	Human	Male	57	unknown
hCoV-19/Mexico/CMX-INER-INMEGEN-00093/2021	EPI_ISL_1824443	17/02/21	North America / Mexico / Mexico City	Human	Male	34	unknown
hCoV-19/Mexico/CMX-INER-INMEGEN-00094/2021	EPI_ISL_1824444	17/02/21	North America / Mexico / Mexico City	Human	Female	26	unknown
hCoV-19/Mexico/CMX-INER-INMEGEN-00099/2021	EPI_ISL_1824445	18/02/21	North America / Mexico / Mexico City	Human	Male	29	unknown
hCoV-19/Mexico/CMX-INER-INMEGEN-00100/2021	EPI_ISL_1824446	18/02/21	North America / Mexico / Mexico City	Human	Male	26	unknown
hCoV-19/Mexico/CMX-INER-INMEGEN-00101/2021	EPI_ISL_1824447	18/02/21	North America / Mexico / Mexico City	Human	Female	55	unknown
hCoV-19/Mexico/CMX-INER-INMEGEN-00102/2021	EPI_ISL_1824448	18/02/21	North America / Mexico / Mexico City	Human	Male	31	unknown
hCoV-19/Mexico/CMX-INER-INMEGEN-00103/2021	EPI_ISL_1824449	18/02/21	North America / Mexico / Mexico City	Human	Female	39	unknown
hCoV-19/Mexico/CMX-INER-INMEGEN-00104/2021	EPI_ISL_1824450	18/02/21	North America / Mexico / Mexico City	Human	Female	42	unknown
hCoV-19/Mexico/CMX-INER-INMEGEN-00105/2021	EPI_ISL_1824451	22/03/21	North America / Mexico / Mexico City	Human	Male	61	unknown
hCoV-19/Mexico/CMX-INER-INMEGEN-00106/2021	EPI_ISL_1824452	22/03/21	North America / Mexico / Mexico City	Human	Female	19	unknown
hCoV-19/Mexico/CMX-INER-INMEGEN-00107/2021	EPI_ISL_1824453	22/03/21	North America / Mexico / Mexico City	Human	Male	46	unknown
hCoV-19/Mexico/CMX-INER-INMEGEN-00109/2021	EPI_ISL_1824455	22/03/21	North America / Mexico / Mexico City	Human	Female	18	unknown
hCoV-19/Mexico/CMX-INER-INMEGEN-00110/2021	EPI_ISL_1824456	22/03/21	North America / Mexico / Mexico City	Human	Female	45	unknown
hCoV-19/Mexico/CMX-INER-INMEGEN-00111/2021	EPI_ISL_1824457	22/03/21	North America / Mexico / Mexico City	Human	Female	62	unknown
hCoV-19/Mexico/CMX-INER-INMEGEN-00112/2021	EPI_ISL_1824458	22/03/21	North America / Mexico / Mexico City	Human	Female	56	unknown
hCoV-19/Mexico/CMX-INER-INMEGEN-00113/2021	EPI_ISL_1824459	22/03/21	North America / Mexico / Mexico City	Human	Female	20	unknown
hCoV-19/Mexico/CMX-INER-INMEGEN-00114/2021	EPI_ISL_1824460	22/03/21	North America / Mexico / Mexico City	Human	Female	51	unknown
hCoV-19/Mexico/CMX-INER-INMEGEN-00115/2021	EPI_ISL_1824461	22/03/21	North America / Mexico / Mexico City	Human	Female	71	unknown
hCoV-19/Mexico/CMX-INER-INMEGEN-00116/2021	EPI_ISL_1824462	22/03/21	North America / Mexico / Mexico City	Human	Female	67	unknown
hCoV-19/Mexico/CMX-INER-INMEGEN-00117/2021	EPI_ISL_1824463	22/03/21	North America / Mexico / Mexico City	Human	Male	49	unknown
hCoV-19/Mexico/CMX-INER-INMEGEN-00118/2021	EPI_ISL_1824464	22/03/21	North America / Mexico / Mexico City	Human	Male	25	unknown
hCoV-19/Mexico/CMX-INER-INMEGEN-00119/2021	EPI_ISL_1824465	22/03/21	North America / Mexico / Mexico City	Human	Male	26	unknown
hCoV-19/Mexico/CMX-INER-INMEGEN-00121/2021	EPI_ISL_1824467	24/03/21	North America / Mexico / Mexico City	Human	Male	21	unknown

hCoV-19/Mexico/CMX-INER-INMEGEN-00122/2021	EPI_ISL_1824468	25/03/21	North America / Mexico / Mexico City	Human	Female	27	unknown	
hCoV-19/Mexico/CMX-INER-INMEGEN-00123/2021	EPI_ISL_1824469	29/03/21	North America / Mexico / Mexico City	Human	Male	55	unknown	
hCoV-19/Mexico/CMX-INER-INMEGEN-00124/2021	EPI_ISL_1824470	31/03/21	North America / Mexico / Mexico City	Human	Female	28	unknown	
hCoV-19/Mexico/CMX-INER-INMEGEN-00125/2021	EPI_ISL_1824471	01/04/21	North America / Mexico / Mexico City	Human	Female	68	unknown	
hCoV-19/Mexico/CMX-INER-INMEGEN-00126/2021	EPI_ISL_1824472	05/04/21	North America / Mexico / Mexico City	Human	Female	35	unknown	
hCoV-19/Mexico/CMX-INER-INMEGEN-00127/2021	EPI_ISL_1824473	05/04/21	North America / Mexico / Mexico City	Human	Male	58	unknown	
hCoV-19/Mexico/CMX-INER-INMEGEN-00128/2021	EPI_ISL_1824474	06/04/21	North America / Mexico / Mexico City	Human	Male	56	unknown	
hCoV-19/Mexico/CMX-INER-INMEGEN-00129/2021	EPI_ISL_1824475	08/04/21	North America / Mexico / Mexico City	Human	Male	86	unknown	
hCoV-19/Mexico/CMX-INER-INMEGEN-00130/2021	EPI_ISL_1824476	08/04/21	North America / Mexico / Mexico City	Human	Female	52	unknown	
hCoV-19/Mexico/CMX-INER-INMEGEN-00132/2021	EPI_ISL_1824478	07/04/21	North America / Mexico / Mexico City	Human	Female	46	unknown	
hCoV-19/Mexico/CMX-INER-INMEGEN-00133/2021	EPI_ISL_1824479	07/04/21	North America / Mexico / Mexico City	Human	Female	34	unknown	
hCoV-19/Mexico/CMX-INER-INMEGEN-00134/2021	EPI_ISL_1824480	07/04/21	North America / Mexico / Mexico City	Human	Female	49	unknown	
hCoV-19/Mexico/CMX-INER-INMEGEN-00135/2021	EPI_ISL_1824481	07/04/21	North America / Mexico / Mexico City	Human	Female	71	unknown	
hCoV-19/Mexico/CMX-INER-INMEGEN-00137/2021	EPI_ISL_1824483	07/04/21	North America / Mexico / Mexico City	Human	Male	56	unknown	
hCoV-19/Mexico/CMX-INER-INMEGEN-00138/2021	EPI_ISL_1824484	08/04/21	North America / Mexico / Mexico City	Human	Male	61	unknown	
hCoV-19/Mexico/CMX-INER-INMEGEN-00140/2021	EPI_ISL_1824486	08/04/21	North America / Mexico / Mexico City	Human	Female	56	unknown	
hCoV-19/Mexico/CMX-INER-INMEGEN-00141/2021	EPI_ISL_1824487	08/04/21	North America / Mexico / Mexico City	Human	Female	47	unknown	
hCoV-19/Mexico/CMX-INER-INMEGEN-00143/2021	EPI_ISL_1824489	08/04/21	North America / Mexico / Mexico City	Human	Female	50	unknown	
hCoV-19/Mexico/CMX-INER-INMEGEN-00144/2021	EPI_ISL_1824490	08/04/21	North America / Mexico / Mexico City	Human	Male	24	unknown	
hCoV-19/Mexico/CMX-INER-INMEGEN-00145/2021	EPI_ISL_1824491	08/04/21	North America / Mexico / Mexico City	Human	Male	64	unknown	
hCoV-19/Mexico/CMX-INER-INMEGEN-00146/2021	EPI_ISL_1824492	08/04/21	North America / Mexico / Mexico City	Human	Female	66	unknown	
hCoV-19/Mexico/CMX-INER-INMEGEN-00147/2021	EPI_ISL_1824493	08/04/21	North America / Mexico / Mexico City	Human	Male	31	unknown	
hCoV-19/Mexico/CMX-INER-INMEGEN-00148/2021	EPI_ISL_1824494	08/04/21	North America / Mexico / Mexico City	Human	Female	68	unknown	
hCoV-19/Mexico/CMX-INER-INMEGEN-00150/2021	EPI_ISL_1824496	09/04/21	North America / Mexico / Mexico City	Human	Female	58	unknown	
hCoV-19/Mexico/CMX-INER-INMEGEN-00151/2021	EPI_ISL_1824497	09/04/21	North America / Mexico / Mexico City	Human	Female	46	unknown	
hCoV-19/Mexico/CMX-INER-INMEGEN-00152/2021	EPI_ISL_1824498	09/04/21	North America / Mexico / Mexico City	Human	Male	60	unknown	
hCoV-19/Mexico/NLE-InDRE_FB13080_S1538/2021	EPI_ISL_1857099	06/04/21	North America / Mexico / Nuevo Leon	Human	Male	51	Hospitalized	Oropharyngeal swab
hCoV-19/Mexico/PUE-InDRE_FB13092_S1545/2021	EPI_ISL_1857100	08/04/21	North America / Mexico / Puebla	Human	Female	27	Released	Oropharyngeal swab
hCoV-19/Mexico/GUA-InDRE_FB13134_S1562/2021	EPI_ISL_1857101	10/04/21	North America / Mexico / Guanajuato	Human	Female	60	Hospitalized	Oropharyngeal swab
hCoV-19/Mexico/CMX-InDRE_FD48254_S1622/2021	EPI_ISL_1857102	09/04/21	North America / Mexico / Mexico City	Human	Female	35	Released	Oropharyngeal swab
hCoV-19/Mexico/CMX-InDRE_FD49446_S1623/2021	EPI_ISL_1857103	12/04/21	North America / Mexico / Mexico City	Human	Female	37	Released	Oropharyngeal swab
hCoV-19/Mexico/CMX-InDRE_FD46181_S1634/2021	EPI_ISL_1857104	07/04/21	North America / Mexico / Mexico City	Human	Male	26	Released	Oropharyngeal swab
hCoV-19/Mexico/CMX-InDRE_FD46182_S1635/2021	EPI_ISL_1857105	07/04/21	North America / Mexico / Mexico City	Human	Female	24	Released	Oropharyngeal swab
hCoV-19/Mexico/TAM-InDRE_FB12784_S1636/2021	EPI_ISL_1857200	30/03/21	North America / Mexico / Tamaulipas	Human	Male	34	Released	Oropharyngeal swab
hCoV-19/Mexico/GRO-InDRE_FB12806_S1637/2021	EPI_ISL_1857201	30/03/21	North America / Mexico / Guerrero	Human	Male	83	Hospitalized	Oropharyngeal swab
hCoV-19/Mexico/YUC-InDRE_FB12810_S1638/2021	EPI_ISL_1857202	03/04/21	North America / Mexico / Yucatan	Human	Female	58	Released	Oropharyngeal swab
hCoV-19/Mexico/YUC-InDRE_FB12814_S1640/2021	EPI_ISL_1857204	05/04/21	North America / Mexico / Yucatan	Human	Male	54	Released	Oropharyngeal swab
hCoV-19/Mexico/YUC-InDRE_FB12815_S1641/2021	EPI_ISL_1857205	05/04/21	North America / Mexico / Yucatan	Human	Female	70	Released	Oropharyngeal swab
hCoV-19/Mexico/YUC-InDRE_FB12816_S1642/2021	EPI_ISL_1857206	03/04/21	North America / Mexico / Yucatan	Human	Female	68	Hospitalized	Oropharyngeal swab
hCoV-19/Mexico/CHP-InDRE_FB12823_S1643/2021	EPI_ISL_1857207	02/04/21	North America / Mexico / Chiapas	Human	Male	48	Hospitalized	Oropharyngeal swab
hCoV-19/Mexico/QUE-InDRE_FB12830_S1645/2021	EPI_ISL_1857208	30/03/21	North America / Mexico / Queretaro	Human	Female	32	Released	Oropharyngeal swab
hCoV-19/Mexico/QUE-InDRE_FB12831_S1646/2021	EPI_ISL_1857209	31/03/21	North America / Mexico / Queretaro	Human	Male	38	Released	Oropharyngeal swab
hCoV-19/Mexico/QUE-InDRE_FB12833_S1647/2021	EPI_ISL_1857210	31/03/21	North America / Mexico / Queretaro	Human	Male	74	Released	Oropharyngeal swab
hCoV-19/Mexico/QUE-InDRE_FB12834_S1648/2021	EPI_ISL_1857211	31/03/21	North America / Mexico / Queretaro	Human	Male	56	Released	Oropharyngeal swab

hCoV-19/Mexico/QUE-InDRE_FB12835_S1649/2021	EPI_ISL_1857212	31/03/21	North America / Mexico / Queretaro	Human	Male	29	Live		Oropharyngeal swab
hCoV-19/Mexico/QUE-InDRE_FB12837_S1650/2021	EPI_ISL_1857213	01/04/21	North America / Mexico / Queretaro	Human	Male	75	Deceased		Oropharyngeal swab
hCoV-19/Mexico/QUE-InDRE_FB12838_S1651/2021	EPI_ISL_1857214	01/04/21	North America / Mexico / Queretaro	Human	Female	46	Live		Oropharyngeal swab
hCoV-19/Mexico/QUE-InDRE_FB12839_S1652/2021	EPI_ISL_1857215	02/04/21	North America / Mexico / Queretaro	Human	Male	56	Hospitalized		Oropharyngeal swab
hCoV-19/Mexico/QUE-InDRE_FB12840_S1653/2021	EPI_ISL_1857216	02/04/21	North America / Mexico / Queretaro	Human	Male	65	Hospitalized		Oropharyngeal swab
hCoV-19/Mexico/CMX-InDRE_FB12841_S1654/2021	EPI_ISL_1857217	02/04/21	North America / Mexico / Mexico City	Human	Male	68	Live		Oropharyngeal swab
hCoV-19/Mexico/QUE-InDRE_FB12844_S1655/2021	EPI_ISL_1857218	04/04/21	North America / Mexico / Queretaro	Human	Male	42	Released		Oropharyngeal swab
hCoV-19/Mexico/CHH-InDRE_FB12865_S1656/2021	EPI_ISL_1857219	30/03/21	North America / Mexico / Chihuahua	Human	Male	48	Hospitalized		Oropharyngeal swab
hCoV-19/Mexico/CHH-InDRE_FB12869_S1657/2021	EPI_ISL_1857220	31/03/21	North America / Mexico / Chihuahua	Human	Female	27	Released		Oropharyngeal swab
hCoV-19/Mexico/CHH-InDRE_FB12874_S1658/2021	EPI_ISL_1857221	03/04/21	North America / Mexico / Chihuahua	Human	Female	87	Released		Oropharyngeal swab
hCoV-19/Mexico/BCS-InDRE_FB12885_S1661/2021	EPI_ISL_1857224	03/04/21	North America / Mexico / Baja California Sur	Human	Female	42	Released		Oropharyngeal swab
hCoV-19/Mexico/BCS-InDRE_FB12887_S1662/2021	EPI_ISL_1857225	03/04/21	North America / Mexico / Baja California Sur	Human	Female	35	Live		Oropharyngeal swab
hCoV-19/Mexico/BCS-InDRE_FB12891_S1663/2021	EPI_ISL_1857226	03/04/21	North America / Mexico / Baja California Sur	Human	Female	22	Hospitalized		Oropharyngeal swab
hCoV-19/Mexico/BCS-InDRE_FB12892_S1664/2021	EPI_ISL_1857227	04/04/21	North America / Mexico / Baja California Sur	Human	Male	72	Hospitalized		Oropharyngeal swab
hCoV-19/Mexico/ROO-InDRE_FB12918_S1665/2021	EPI_ISL_1857228	31/03/21	North America / Mexico / Quintana Roo	Human	Male	77	Live		Oropharyngeal swab
hCoV-19/Mexico/JAL-InDRE_FB12920_S1667/2021	EPI_ISL_1857230	05/04/21	North America / Mexico / Jalisco	Human	Female	60	Hospitalized		Oropharyngeal swab
hCoV-19/Mexico/JAL-InDRE_FB12921_S1668/2021	EPI_ISL_1857231	30/03/21	North America / Mexico / Jalisco	Human	Male	70	Released		Oropharyngeal swab
hCoV-19/Mexico/COA-InDRE_FB13030_S1669/2021	EPI_ISL_1857232	04/04/21	North America / Mexico / Coahuila	Human	Male	94	Released		Oropharyngeal swab
hCoV-19/Mexico/HID-InDRE_FB13033_S1670/2021	EPI_ISL_1857233	06/04/21	North America / Mexico / Hidalgo	Human	Male	41	Released		Oropharyngeal swab
hCoV-19/Mexico/HID-InDRE_FB13036_S1671/2021	EPI_ISL_1857234	06/04/21	North America / Mexico / Hidalgo	Human	Male	66	Released		Oropharyngeal swab
hCoV-19/Mexico/BCS-InDRE_FB13043_S1672/2021	EPI_ISL_1857235	30/03/21	North America / Mexico / Baja California Sur	Human	Female	37	Released		Oropharyngeal swab
hCoV-19/Mexico/GUA-InDRE_FB13061_S1673/2021	EPI_ISL_1857236	03/04/21	North America / Mexico / Guanajuato	Human	Male	65	Hospitalized		Oropharyngeal swab
hCoV-19/Mexico/GUA-InDRE_FB13063_S1674/2021	EPI_ISL_1857237	05/04/21	North America / Mexico / Guanajuato	Human	Male	55	Live		Oropharyngeal swab
hCoV-19/Mexico/GUA-InDRE_FB13067_S1675/2021	EPI_ISL_1857238	09/04/21	North America / Mexico / Guanajuato	Human	Male	75	Hospitalized		Oropharyngeal swab
hCoV-19/Mexico/GUA-InDRE_FB13068_S1676/2021	EPI_ISL_1857239	01/04/21	North America / Mexico / Guanajuato	Human	Male	65	Released		Oropharyngeal swab
hCoV-19/Mexico/PUE-InDRE_FB13097_S1680/2021	EPI_ISL_1857242	08/04/21	North America / Mexico / Puebla	Human	Female	44	Hospitalized		Oropharyngeal swab
hCoV-19/Mexico/PUE-InDRE_FB13102_S1681/2021	EPI_ISL_1857243	11/04/21	North America / Mexico / Puebla	Human	Female	74	Deceased		Oropharyngeal swab
hCoV-19/Mexico/JAL-InDRE_FB13124_S1682/2021	EPI_ISL_1857244	12/04/21	North America / Mexico / Jalisco	Human	Female	70	Released		Oropharyngeal swab
hCoV-19/Mexico/QUE-InDRE_FB13126_S1683/2021	EPI_ISL_1857245	06/04/21	North America / Mexico / Queretaro	Human	Male	62	Deceased		Oropharyngeal swab
hCoV-19/Mexico/QUE-InDRE_FB13129_S1684/2021	EPI_ISL_1857246	08/04/21	North America / Mexico / Queretaro	Human	Female	49	Released		Oropharyngeal swab
hCoV-19/Mexico/MOR-InDRE_FB13148_S1688/2021	EPI_ISL_1857248	02/04/21	North America / Mexico / Morelos	Human	Female	68	Hospitalized		Oropharyngeal swab
hCoV-19/Mexico/MOR-InDRE_FB13167_S1689/2021	EPI_ISL_1857249	07/04/21	North America / Mexico / Morelos	Human	Male	53	Hospitalized		Oropharyngeal swab
hCoV-19/Mexico/MOR-InDRE_FB13168_S1690/2021	EPI_ISL_1857250	07/04/21	North America / Mexico / Morelos	Human	Male	68	Hospitalized		Oropharyngeal swab
hCoV-19/Mexico/MOR-InDRE_FB13171_S1691/2021	EPI_ISL_1857251	09/04/21	North America / Mexico / Morelos	Human	Female	29	Released		Oropharyngeal swab
hCoV-19/Mexico/OAX-InDRE_FB13219_S1699/2021	EPI_ISL_1857255	05/04/21	North America / Mexico / Oaxaca	Human	Male	49	Released		Oropharyngeal swab
hCoV-19/Mexico/OAX-InDRE_FB13220_S1700/2021	EPI_ISL_1857256	03/04/21	North America / Mexico / Oaxaca	Human	Female	71	Released		Oropharyngeal swab
hCoV-19/Mexico/TLA-InDRE_FB13241_S1702/2021	EPI_ISL_1857257	12/04/21	North America / Mexico / Tlaxcala	Human	Female	51	Released		Oropharyngeal swab
hCoV-19/Mexico/CHH-InDRE_FB13244_S1703/2021	EPI_ISL_1857258	05/04/21	North America / Mexico / Chihuahua	Human	Male	28	Released		Oropharyngeal swab
hCoV-19/Mexico/CHH-InDRE_FB13248_S1704/2021	EPI_ISL_1857259	05/04/21	North America / Mexico / Chihuahua	Human	Female	70	Hospitalized		Oropharyngeal swab
hCoV-19/Mexico/CHH-InDRE_FB13251_S1705/2021	EPI_ISL_1857260	09/04/21	North America / Mexico / Chihuahua	Human	Male	55	Released		Oropharyngeal swab
hCoV-19/Mexico/CHH-InDRE_FB13256_S1707/2021	EPI_ISL_1857262	11/04/21	North America / Mexico / Chihuahua	Human	Male	73	Deceased		Oropharyngeal swab
hCoV-19/Mexico/ZAC-InDRE_FB13278_S1709/2021	EPI_ISL_1857264	13/04/21	North America / Mexico / Zacatecas	Human	Male	56	Released		Oropharyngeal swab
hCoV-19/Mexico/BCS-InDRE_FB13294_S1711/2021	EPI_ISL_1857266	10/04/21	North America / Mexico / Baja California Sur	Human	Male	51	Released		Oropharyngeal swab
hCoV-19/Mexico/BCS-InDRE_FB13299_S1712/2021	EPI_ISL_1857267	10/04/21	North America / Mexico / Baja California Sur	Human	Male	6	Released		Oropharyngeal swab

ıCoV-19/Mexico/CMX-InDRE_FB13306_S1713/202	EPI_ISL_1857268	05/04/21	North America / Mexico / Mexico City	Human	Male	65	Released	Oropharyngeal swab
ıCoV-19/Mexico/MEX-InDRE_FB13324_S1715/202	EPI_ISL_1857270	04/04/21	North America / Mexico / State of Mexico	Human	Female	66	Deceased	Oropharyngeal swab
ıCoV-19/Mexico/CMX-InDRE_FD53894_S1717/202	EPI_ISL_1857272	13/04/21	North America / Mexico / Mexico City	Human	Female	45	Released	Oropharyngeal swab
ıCoV-19/Mexico/CMX-InDRE_FD49359_S1718/202	EPI_ISL_1857273	12/04/21	North America / Mexico / Mexico City	Human	Female	33	Released	Oropharyngeal swab
ıCoV-19/Mexico/CMX-InDRE_FD49545_S1719/202	EPI_ISL_1857274	12/04/21	North America / Mexico / Mexico City	Human	Male	62	Released	Oropharyngeal swab
ıCoV-19/Mexico/CMX-InDRE_FD49549_S1720/202	EPI_ISL_1857275	13/04/21	North America / Mexico / Mexico City	Human	Female	47	Live	Oropharyngeal swab
hCoV-19/Mexico/CMX-INMEGEN-04-04-66/2021	EPI_ISL_1857295	26/02/21	North America / Mexico / Mexico City	Human	Female	59	unknown	Oropharyngeal swab
hCoV-19/Mexico/NLE-LESPNL-20210407045/2021	EPI_ISL_1911253	25/03/21	North America / Mexico / Nuevo Leon	Human	Male	35	RY - UNDER T	asopharyngeal and/or oropharyngeal exudat
hCoV-19/Mexico/NLE-LESPNL-20210407035/2021	EPI_ISL_1911254	27/03/21	North America / Mexico / Nuevo Leon	Human	Male	20	RY - UNDER T	asopharyngeal and/or oropharyngeal exudat
hCoV-19/Mexico/NLE-LESPNL-20210407027/2021	EPI_ISL_1911255	26/03/21	North America / Mexico / Nuevo Leon	Human	Female	68	RY - UNDER T	asopharyngeal and/or oropharyngeal exudat
hCoV-19/Mexico/NLE-LESPNL-20210407024/2021	EPI_ISL_1911259	10/03/21	North America / Mexico / Nuevo Leon	Human	Female	47	RY - UNDER T	asopharyngeal and/or oropharyngeal exudat
hCoV-19/Mexico/NLE-LESPNL-20210407022/2021	EPI_ISL_1911262	24/03/21	North America / Mexico / Nuevo Leon	Human	Female	55	RY - UNDER T	asopharyngeal and/or oropharyngeal exudat
hCoV-19/Mexico/NLE-LESPNL-20210407012/2021	EPI_ISL_1911267	18/03/21	North America / Mexico / Nuevo Leon	Human	Female	57	NT - HOME M	asopharyngeal and/or oropharyngeal exudat
hCoV-19/Mexico/NLE-LESPNL-20210407034/2021	EPI_ISL_1911268	20/03/21	North America / Mexico / Nuevo Leon	Human	Male	52	NT - HOME M	asopharyngeal and/or oropharyngeal exudat
hCoV-19/Mexico/NLE-LESPNL-20210407042/2021	EPI_ISL_1911269	26/03/21	North America / Mexico / Nuevo Leon	Human	Male	28	NT - HOME M	asopharyngeal and/or oropharyngeal exudat
hCoV-19/Mexico/NLE-LESPNL-20210407023/2021	EPI_ISL_1911274	29/03/21	North America / Mexico / Nuevo Leon	Human	Male	27	NT - HOME M	asopharyngeal and/or oropharyngeal exudat
hCoV-19/Mexico/NLE-LESPNL-20210407002/2021	EPI_ISL_1911278	10/03/21	North America / Mexico / Nuevo Leon	Human	Female	36	NT - HOME M	asopharyngeal and/or oropharyngeal exudat
hCoV-19/Mexico/NLE-LESPNL-20210407026/2021	EPI_ISL_1911279	31/03/21	North America / Mexico / Nuevo Leon	Human	Male	22	NT - HOME M	asopharyngeal and/or oropharyngeal exudat
hCoV-19/Mexico/NLE-LESPNL-20210407059/2021	EPI_ISL_1911285	30/03/21	North America / Mexico / Nuevo Leon	Human	Female	43	NT - HOME M	asopharyngeal and/or oropharyngeal exudat
hCoV-19/Mexico/NLE-LESPNL-20210407005/2021	EPI_ISL_1911287	28/03/21	North America / Mexico / Nuevo Leon	Human	Male	62	NT - HOME M	asopharyngeal and/or oropharyngeal exudat
hCoV-19/Mexico/NLE-LESPNL-20210407039/2021	EPI_ISL_1911288	30/03/21	North America / Mexico / Nuevo Leon	Human	Male	51	NT - HOME M	asopharyngeal and/or oropharyngeal exudat
hCoV-19/Mexico/NLE-LESPNL-20210407047/2021	EPI_ISL_1911290	24/03/21	North America / Mexico / Nuevo Leon	Human	Male	61	NT - HOME M	asopharyngeal and/or oropharyngeal exudat
hCoV-19/Mexico/NLE-LESPNL-20210407028/2021	EPI_ISL_1911291	17/03/21	North America / Mexico / Nuevo Leon	Human	Female	49	NT - HOME M	asopharyngeal and/or oropharyngeal exudat
hCoV-19/Mexico/NLE-LESPNL-20210407013/2021	EPI_ISL_1911293	16/03/21	North America / Mexico / Nuevo Leon	Human	Female	21	NT - HOME M	asopharyngeal and/or oropharyngeal exudat
hCoV-19/Mexico/NLE-LESPNL-20210407031/2021	EPI_ISL_1911300	17/03/21	North America / Mexico / Nuevo Leon	Human	Female	35	LIZED - SERI	asopharyngeal and/or oropharyngeal exudat
hCoV-19/Mexico/NLE-LESPNL-20210407008/2021	EPI_ISL_1911307	19/03/21	North America / Mexico / Nuevo Leon	Human	Male	75	LIZED - SERI	asopharyngeal and/or oropharyngeal exudat
hCoV-19/Mexico/NLE-LESPNL-20210407001/2021	EPI_ISL_1911310	18/03/21	North America / Mexico / Nuevo Leon	Human	Female	70	ED - NON-SE	asopharyngeal and/or oropharyngeal exudat
hCoV-19/Mexico/CMX-INER-INMEGEN-00074/2021	EPI_ISL_1970838	19/02/21	North America / Mexico / Mexico City	Human	Female	64	unknown	
hCoV-19/Mexico/CMX-INER-INMEGEN-00080/2021	EPI_ISL_1970839	18/03/21	North America / Mexico / Mexico City	Human	Male	55	unknown	
hCoV-19/Mexico/CMX-INER-INMEGEN-00087/2021	EPI_ISL_1970840	18/03/21	North America / Mexico / Mexico City	Human	Female	70	unknown	
hCoV-19/Mexico/CMX-INER-INMEGEN-00088/2021	EPI_ISL_1970841	17/03/21	North America / Mexico / Mexico City	Human	Female	44	unknown	
hCoV-19/Mexico/SIN_CIAD_HJ1324/2021	EPI_ISL_1972328	23/03/21	North America / Mexico / Sinaloa	Human	Male	43	unknown	Oropharyngeal swab
hCoV-19/Mexico/SIN_CIAD_HJ1326/2021	EPI_ISL_1972329	23/03/21	North America / Mexico / Sinaloa	Human	Male	47	unknown	Oropharyngeal swab
hCoV-19/Mexico/SIN_CIAD_HJ1439/2021	EPI_ISL_1972330	12/04/21	North America / Mexico / Sinaloa	Human	Female	43	unknown	Oropharyngeal swab
hCoV-19/Mexico/SIN_CIAD_HJ1450/2021	EPI_ISL_1972331	14/04/21	North America / Mexico / Sinaloa	Human	Male	36	unknown	Oropharyngeal swab
hCoV-19/Mexico/SIN_CIAD_HJ1453/2021	EPI_ISL_1972332	14/04/21	North America / Mexico / Sinaloa	Human	Male	22	unknown	Oropharyngeal swab
hCoV-19/Mexico/SIN_CIAD_HJ1455/2021	EPI_ISL_1972333	14/04/21	North America / Mexico / Sinaloa	Human	Female	48	unknown	Oropharyngeal swab
hCoV-19/Mexico/SIN_CIAD_HJ1457/2021	EPI_ISL_1972334	14/04/21	North America / Mexico / Sinaloa	Human	Male	79	unknown	Oropharyngeal swab
hCoV-19/Mexico/SIN_CIAD_ID2018/2021	EPI_ISL_1972335	24/03/21	North America / Mexico / Sinaloa	Human	Male	82	unknown	Oropharyngeal swab
hCoV-19/Mexico/SIN_CIAD_ID2135/2021	EPI_ISL_1972337	07/04/21	North America / Mexico / Sinaloa	Human	Female	70	unknown	Oropharyngeal swab
hCoV-19/Mexico/SIN_CIAD_S6387/2021	EPI_ISL_1972338	24/03/21	North America / Mexico / Sinaloa	Human	Male	35	unknown	Oropharyngeal swab
hCoV-19/Mexico/SIN_CIAD_S6499/2021	EPI_ISL_1972339	07/04/21	North America / Mexico / Sinaloa	Human	Female	42	unknown	Oropharyngeal swab
hCoV-19/Mexico/SIN_CIAD_S6653/2021	EPI_ISL_2026396	27/04/21	North America / Mexico / Sinaloa / Mazatlan	Human	Male	52	unknown	Oropharyngeal swab
hCoV-19/Mexico/SIN_CIAD_S6644/2021	EPI_ISL_2026398	26/04/21	North America / Mexico / Sinaloa / Mazatlan	Human	Male	35	unknown	Oropharyngeal swab

hCoV-19/Mexico/SIN_CIAD_HJ1458/2021	EPI_ISL_2026402	14/04/21	North America / Mexico / Sinaloa / Mazatlan	Human	Female	68	unknown	Oropharyngeal swab
hCoV-19/Mexico/SIN_CIAD_ID2087/2021	EPI_ISL_2026406	31/03/21	North America / Mexico / Sinaloa / Mazatlan	Human	Female	20	unknown	Oropharyngeal swab
hCoV-19/Mexico/SIN_CIAD_S6558/2021	EPI_ISL_2026424	14/04/21	North America / Mexico / Sinaloa / Mazatlan	Human	Male	29	unknown	Oropharyngeal swab
hCoV-19/Mexico/SIN_CIAD_HJ1327/2021	EPI_ISL_2026426	23/03/21	North America / Mexico / Sinaloa / Mazatlan	Human	Female	39	unknown	Oropharyngeal swab
hCoV-19/Mexico/SIN_CIAD_S6526/2021	EPI_ISL_2026429	09/04/21	North America / Mexico / Sinaloa / Mazatlan	Human	Male	34	unknown	Oropharyngeal swab
hCoV-19/Mexico/SIN_CIAD_HJ1491/2021	EPI_ISL_2026440	19/04/21	North America / Mexico / Sinaloa / Mazatlan	Human	Female	78	unknown	Oropharyngeal swab
hCoV-19/Mexico/SIN_CIAD_S6435/2021	EPI_ISL_2026442	30/03/21	North America / Mexico / Sinaloa / Mazatlan	Human	Female	32	unknown	Oropharyngeal swab
hCoV-19/Mexico/SIN_CIAD_S6468/2021	EPI_ISL_2026444	06/04/21	North America / Mexico / Sinaloa / Mazatlan	Human	Male	69	unknown	Oropharyngeal swab
hCoV-19/Mexico/SIN_CIAD_HJ1484/2021	EPI_ISL_2026446	16/04/21	North America / Mexico / Sinaloa / Mazatlan	Human	Female	16	unknown	Oropharyngeal swab
hCoV-19/Mexico/SIN_CIAD_HJ1492/2021	EPI_ISL_2026448	19/04/21	North America / Mexico / Sinaloa / Mazatlan	Human	Male	82	unknown	Oropharyngeal swab
hCoV-19/Mexico/SIN_CIAD_HJ1498/2021	EPI_ISL_2026450	19/04/21	North America / Mexico / Sinaloa / Mazatlan	Human	Female	31	unknown	Oropharyngeal swab
hCoV-19/Mexico/SIN_CIAD_HJ1502/2021	EPI_ISL_2026452	19/04/21	North America / Mexico / Sinaloa / Mazatlan	Human	Female	58	unknown	Oropharyngeal swab
hCoV-19/Mexico/SIN_CIAD_HJ1507/2021	EPI_ISL_2026454	20/04/21	North America / Mexico / Sinaloa / Mazatlan	Human	Female	59	unknown	Oropharyngeal swab
hCoV-19/Mexico/SIN_CIAD_S6634/2021	EPI_ISL_2026456	23/04/21	North America / Mexico / Sinaloa / Mazatlan	Human	Male	54	unknown	Oropharyngeal swab
hCoV-19/Mexico/SIN_CIAD_HJ1556/2021	EPI_ISL_2026458	27/04/21	North America / Mexico / Sinaloa / Mazatlan	Human	Male	28	unknown	Oropharyngeal swab
hCoV-19/Mexico/SIN_CIAD_S6654/2021	EPI_ISL_2026460	27/04/21	North America / Mexico / Sinaloa / Mazatlan	Human	Female	53	unknown	Oropharyngeal swab
hCoV-19/Mexico/CMX-INMEGEN-03-11-61/2021	EPI_ISL_2080616	04/03/21	North America / Mexico / Mexico City	Human	Female	46	unknown	Oropharyngeal swab
hCoV-19/Mexico/CMX-INMEGEN-03-11-256/2021	EPI_ISL_2080617	25/03/21	North America / Mexico / Mexico City	Human	Female	69	unknown	Oropharyngeal swab
hCoV-19/Mexico/CMX-INMEGEN-04-07-191/2021	EPI_ISL_2080624	23/04/21	North America / Mexico / Mexico City	Human	Female	38	unknown	Oropharyngeal swab
hCoV-19/Mexico/CMX-INMEGEN-04-07-192/2021	EPI_ISL_2080625	23/04/21	North America / Mexico / Mexico City	Human	Male	25	unknown	Oropharyngeal swab
hCoV-19/Mexico/CMX-INMEGEN-04-07-193/2021	EPI_ISL_2080626	23/04/21	North America / Mexico / Mexico City	Human	Male	30	unknown	Oropharyngeal swab
hCoV-19/Mexico/CMX-INMEGEN-04-07-196/2021	EPI_ISL_2080628	23/04/21	North America / Mexico / Mexico City	Human	Female	39	unknown	Oropharyngeal swab
hCoV-19/Mexico/CMX-INMEGEN-04-07-197/2021	EPI_ISL_2080629	23/04/21	North America / Mexico / Mexico City	Human	Female	22	unknown	Oropharyngeal swab
hCoV-19/Mexico/CMX-INMEGEN-04-07-198/2021	EPI_ISL_2080630	23/04/21	North America / Mexico / Mexico City	Human	Female	53	unknown	Oropharyngeal swab
hCoV-19/Mexico/CMX-INMEGEN-04-07-200/2021	EPI_ISL_2080631	23/04/21	North America / Mexico / Mexico City	Human	Male	34	unknown	Oropharyngeal swab
hCoV-19/Mexico/CMX-INMEGEN-04-07-205/2021	EPI_ISL_2080632	23/04/21	North America / Mexico / Mexico City	Human	Female	39	unknown	Oropharyngeal swab
hCoV-19/Mexico/CMX-INMEGEN-04-07-206/2021	EPI_ISL_2080633	23/04/21	North America / Mexico / Mexico City	Human	Female	66	unknown	Oropharyngeal swab
hCoV-19/Mexico/CMX-INMEGEN-04-07-207/2021	EPI_ISL_2080634	23/04/21	North America / Mexico / Mexico City	Human	Male	42	unknown	Oropharyngeal swab
hCoV-19/Mexico/CMX-INMEGEN-04-07-214/2021	EPI_ISL_2080636	23/04/21	North America / Mexico / Mexico City	Human	Female	45	unknown	Oropharyngeal swab
hCoV-19/Mexico/CMX-INMEGEN-04-07-215/2021	EPI_ISL_2080637	23/04/21	North America / Mexico / Mexico City	Human	Male	45	unknown	Oropharyngeal swab
hCoV-19/Mexico/CMX-INMEGEN-04-07-216/2021	EPI_ISL_2080638	23/04/21	North America / Mexico / Mexico City	Human	Male	48	unknown	Oropharyngeal swab
hCoV-19/Mexico/CMX-INMEGEN-04-07-217/2021	EPI_ISL_2080639	23/04/21	North America / Mexico / Mexico City	Human	Male	22	unknown	Oropharyngeal swab
hCoV-19/Mexico/CMX-INMEGEN-04-07-218/2021	EPI_ISL_2080640	23/04/21	North America / Mexico / Mexico City	Human	Male	51	unknown	Oropharyngeal swab
hCoV-19/Mexico/CMX-INMEGEN-04-07-219/2021	EPI_ISL_2080641	23/04/21	North America / Mexico / Mexico City	Human	Male	59	unknown	Oropharyngeal swab
hCoV-19/Mexico/CMX-INMEGEN-04-07-221/2021	EPI_ISL_2080642	23/04/21	North America / Mexico / Mexico City	Human	Male	29	unknown	Oropharyngeal swab
hCoV-19/Mexico/CMX-INMEGEN-04-07-223/2021	EPI_ISL_2080643	26/04/21	North America / Mexico / Mexico City	Human	Male	49	unknown	Oropharyngeal swab
hCoV-19/Mexico/CMX-INMEGEN-04-07-227/2021	EPI_ISL_2080645	26/04/21	North America / Mexico / Mexico City	Human	Female	16	unknown	Oropharyngeal swab
hCoV-19/Mexico/CMX-INMEGEN-04-07-231/2021	EPI_ISL_2080648	26/04/21	North America / Mexico / Mexico City	Human	Female	49	unknown	Oropharyngeal swab
hCoV-19/Mexico/CMX-INMEGEN-04-07-232/2021	EPI_ISL_2080649	26/04/21	North America / Mexico / Mexico City	Human	Female	34	unknown	Oropharyngeal swab
hCoV-19/Mexico/CMX-INMEGEN-04-07-233/2021	EPI_ISL_2080650	26/04/21	North America / Mexico / Mexico City	Human	Male	32	unknown	Oropharyngeal swab
hCoV-19/Mexico/CMX-INMEGEN-04-07-234/2021	EPI_ISL_2080651	26/04/21	North America / Mexico / Mexico City	Human	Male	28	unknown	Oropharyngeal swab
hCoV-19/Mexico/CMX-INMEGEN-04-07-236/2021	EPI_ISL_2080652	26/04/21	North America / Mexico / Mexico City	Human	Male	53	unknown	Oropharyngeal swab
hCoV-19/Mexico/CMX-INMEGEN-04-07-237/2021	EPI_ISL_2080653	26/04/21	North America / Mexico / Mexico City	Human	Male	25	unknown	Oropharyngeal swab
hCoV-19/Mexico/CMX-INMEGEN-04-07-238/2021	EPI_ISL_2080654	26/04/21	North America / Mexico / Mexico City	Human	Female	33	unknown	Oropharyngeal swab

hCoV-19/Mexico/CMX-INMEGEN-04-07-239/2021	EPI_ISL_2080655	26/04/21	North America / Mexico / Mexico City	Human	Female	45	unknown	Oropharyngeal swab
hCoV-19/Mexico/CMX-INMEGEN-04-07-240/2021	EPI_ISL_2080656	26/04/21	North America / Mexico / Mexico City	Human	Female	72	unknown	Oropharyngeal swab
hCoV-19/Mexico/CMX-INMEGEN-04-07-241/2021	EPI_ISL_2080657	26/04/21	North America / Mexico / Mexico City	Human	Female	22	unknown	Oropharyngeal swab
hCoV-19/Mexico/CMX-INMEGEN-04-07-244/2021	EPI_ISL_2080659	26/04/21	North America / Mexico / Mexico City	Human	Female	55	unknown	Oropharyngeal swab
hCoV-19/Mexico/CMX-INMEGEN-04-07-245/2021	EPI_ISL_2080660	26/04/21	North America / Mexico / Mexico City	Human	Female	58	unknown	Oropharyngeal swab
hCoV-19/Mexico/CMX-INMEGEN-04-07-246/2021	EPI_ISL_2080661	26/04/21	North America / Mexico / Mexico City	Human	Male	28	unknown	Oropharyngeal swab
hCoV-19/Mexico/CMX-INMEGEN-04-07-247/2021	EPI_ISL_2080662	26/04/21	North America / Mexico / Mexico City	Human	Male	33	unknown	Oropharyngeal swab
hCoV-19/Mexico/CMX-INMEGEN-04-07-248/2021	EPI_ISL_2080663	26/04/21	North America / Mexico / Mexico City	Human	Male	23	unknown	Oropharyngeal swab
hCoV-19/Mexico/CMX-INMEGEN-04-07-249/2021	EPI_ISL_2080664	26/04/21	North America / Mexico / Mexico City	Human	Female	39	unknown	Oropharyngeal swab
hCoV-19/Mexico/CMX-INMEGEN-04-07-250/2021	EPI_ISL_2080665	26/04/21	North America / Mexico / Mexico City	Human	Male	52	unknown	Oropharyngeal swab
hCoV-19/Mexico/CMX-INMEGEN-04-07-252/2021	EPI_ISL_2080666	26/04/21	North America / Mexico / Mexico City	Human	Male	32	unknown	Oropharyngeal swab
hCoV-19/Mexico/CMX-INMEGEN-04-07-255/2021	EPI_ISL_2080667	26/04/21	North America / Mexico / Mexico City	Human	Male	35	unknown	Oropharyngeal swab
hCoV-19/Mexico/CMX-INMEGEN-04-07-256/2021	EPI_ISL_2080668	26/04/21	North America / Mexico / Mexico City	Human	Male	54	unknown	Oropharyngeal swab
hCoV-19/Mexico/CMX-INMEGEN-04-07-258/2021	EPI_ISL_2080669	26/04/21	North America / Mexico / Mexico City	Human	Female	52	unknown	Oropharyngeal swab
hCoV-19/Mexico/CMX-INMEGEN-04-07-259/2021	EPI_ISL_2080670	26/04/21	North America / Mexico / Mexico City	Human	Female	52	unknown	Oropharyngeal swab
hCoV-19/Mexico/CMX-INMEGEN-04-07-262/2021	EPI_ISL_2080672	26/04/21	North America / Mexico / Mexico City	Human	Male	52	unknown	Oropharyngeal swab
hCoV-19/Mexico/CMX-INMEGEN-04-07-263/2021	EPI_ISL_2080673	26/04/21	North America / Mexico / Mexico City	Human	Male	40	unknown	Oropharyngeal swab
hCoV-19/Mexico/CMX-INMEGEN-04-07-264/2021	EPI_ISL_2080674	27/04/21	North America / Mexico / Mexico City	Human	Male	53	unknown	Oropharyngeal swab
hCoV-19/Mexico/CMX-INMEGEN-04-07-265/2021	EPI_ISL_2080675	27/04/21	North America / Mexico / Mexico City	Human	Male	50	unknown	Oropharyngeal swab
hCoV-19/Mexico/CMX-INMEGEN-04-07-266/2021	EPI_ISL_2080676	27/04/21	North America / Mexico / Mexico City	Human	Male	29	unknown	Oropharyngeal swab
hCoV-19/Mexico/CMX-INMEGEN-04-07-268/2021	EPI_ISL_2080677	27/04/21	North America / Mexico / Mexico City	Human	Female	36	unknown	Oropharyngeal swab
hCoV-19/Mexico/CMX-INMEGEN-04-07-269/2021	EPI_ISL_2080678	27/04/21	North America / Mexico / Mexico City	Human	Female	17	unknown	Oropharyngeal swab
hCoV-19/Mexico/CMX-INMEGEN-04-07-270/2021	EPI_ISL_2080679	27/04/21	North America / Mexico / Mexico City	Human	Female	45	unknown	Oropharyngeal swab
hCoV-19/Mexico/CMX-INMEGEN-04-07-271/2021	EPI_ISL_2080680	27/04/21	North America / Mexico / Mexico City	Human	Male	43	unknown	Oropharyngeal swab
hCoV-19/Mexico/CMX-INMEGEN-04-07-273/2021	EPI_ISL_2080681	27/04/21	North America / Mexico / Mexico City	Human	Male	77	unknown	Oropharyngeal swab
hCoV-19/Mexico/CMX-INMEGEN-04-07-283/2021	EPI_ISL_2080682	27/04/21	North America / Mexico / Mexico City	Human	Female	55	unknown	Oropharyngeal swab
hCoV-19/Mexico/CMX-INMEGEN-04-07-285/2021	EPI_ISL_2080683	28/04/21	North America / Mexico / Mexico City	Human	Female	20	unknown	Oropharyngeal swab
hCoV-19/Mexico/CMX-INMEGEN-04-07-286/2021	EPI_ISL_2080684	28/04/21	North America / Mexico / Mexico City	Human	Male	41	unknown	Oropharyngeal swab
hCoV-19/Mexico/CMX-INMEGEN-04-07-287/2021	EPI_ISL_2080685	28/04/21	North America / Mexico / Mexico City	Human	Male	26	unknown	Oropharyngeal swab
hCoV-19/Mexico/CMX-INMEGEN-04-07-288/2021	EPI_ISL_2080686	28/04/21	North America / Mexico / Mexico City	Human	Female	57	unknown	Oropharyngeal swab
hCoV-19/Mexico/CMX-INMEGEN-04-07-289/2021	EPI_ISL_2080687	28/04/21	North America / Mexico / Mexico City	Human	Male	33	unknown	Oropharyngeal swab
hCoV-19/Mexico/CMX-INMEGEN-04-07-290/2021	EPI_ISL_2080688	28/04/21	North America / Mexico / Mexico City	Human	Female	37	unknown	Oropharyngeal swab
hCoV-19/Mexico/CMX-INMEGEN-04-07-293/2021	EPI_ISL_2080690	28/04/21	North America / Mexico / Mexico City	Human	Male	59	unknown	Oropharyngeal swab
hCoV-19/Mexico/CMX-INMEGEN-04-07-294/2021	EPI_ISL_2080691	28/04/21	North America / Mexico / Mexico City	Human	Female	37	unknown	Oropharyngeal swab
hCoV-19/Mexico/CMX-INMEGEN-04-07-295/2021	EPI_ISL_2080692	28/04/21	North America / Mexico / Mexico City	Human	Female	23	unknown	Oropharyngeal swab
hCoV-19/Mexico/CMX-INMEGEN-04-07-297/2021	EPI_ISL_2080694	28/04/21	North America / Mexico / Mexico City	Human	Male	49	unknown	Oropharyngeal swab
CoV-19/Mexico/CMX-INMEGEN-InDRE-04-04-169/20	EPI_ISL_2090648	05/04/21	North America / Mexico / Mexico City	Human	Male	24	unknown	Oropharyngeal swab
CoV-19/Mexico/CMX-INMEGEN-InDRE-04-04-171/20	EPI_ISL_2090649	05/04/21	North America / Mexico / Mexico City	Human	Male	56	unknown	Oropharyngeal swab
hCoV-19/Mexico/CMX_INER_IMSS_00677/2021	EPI_ISL_2091133	22/02/21	North America / Mexico / Mexico City	Human	Male	17	Ambulatory	Oropharyngeal swab
hCoV-19/Mexico/PUE_INER_IMSS_00678/2021	EPI_ISL_2091134	19/02/21	North America / Mexico / Puebla	Human	Male	38	Ambulatory	Nasopharyngeal and oropharyngeal swab
hCoV-19/Mexico/CMX_INER_IMSS_00681/2021	EPI_ISL_2091137	24/02/21	North America / Mexico / Mexico City	Human	Female	33	Ambulatory	Nasopharyngeal and oropharyngeal swab
hCoV-19/Mexico/CHH_INER_IMSS_00682/2021	EPI_ISL_2091138	09/03/21	North America / Mexico / Chihuahua	Human	Male	42	Hospitalized	Nasopharyngeal and oropharyngeal swab
hCoV-19/Mexico/JAL_INER_IMSS_00684/2021	EPI_ISL_2091140	15/03/21	North America / Mexico / Jalisco	Human	Male	74	Hospitalized	Nasopharyngeal and oropharyngeal swab
hCoV-19/Mexico/GUA_INER_IMSS_00685/2021	EPI_ISL_2091141	16/03/21	North America / Mexico / Guanajuato	Human	Female	71	Ambulatory	Nasopharyngeal and oropharyngeal swab

hCoV-19/Mexico/OAX_INER_IMSS_00687/2021	EPI_ISL_2091143	17/03/21	North America / Mexico / Oaxaca	Human	Male	75	Hospitalized	Nasopharyngeal swab
hCoV-19/Mexico/HID_INER_IMSS_00688/2021	EPI_ISL_2091144	19/03/21	North America / Mexico / Hidalgo	Human	Male	52	Hospitalized	Nasopharyngeal and oropharyngeal swab
hCoV-19/Mexico/MIC_INER_IMSS_00689/2021	EPI_ISL_2091145	16/03/21	North America / Mexico / Michoacan	Human	Female	91	Hospitalized	Nasopharyngeal and oropharyngeal swab
hCoV-19/Mexico/MEX_INER_IMSS_00691/2021	EPI_ISL_2091147	21/03/21	North America / Mexico / State of Mexico	Human	Male	51	Hospitalized	Nasopharyngeal and oropharyngeal swab
hCoV-19/Mexico/QUE_INER_IMSS_00692/2021	EPI_ISL_2091148	22/03/21	North America / Mexico / Queretaro	Human	Female	85	Hospitalized	Nasopharyngeal and oropharyngeal swab
hCoV-19/Mexico/CMX_INER_IMSS_00693/2021	EPI_ISL_2091149	22/03/21	North America / Mexico / Mexico City	Human	Female	69	Hospitalized	Nasopharyngeal and oropharyngeal swab
hCoV-19/Mexico/MEX_INER_IMSS_00694/2021	EPI_ISL_2091150	22/03/21	North America / Mexico / State of Mexico	Human	Female	67	Hospitalized	Nasopharyngeal and oropharyngeal swab
hCoV-19/Mexico/CMX_INER_IMSS_00695/2021	EPI_ISL_2091151	22/03/21	North America / Mexico / Mexico City	Human	Male	57	Ambulatory	Nasopharyngeal and oropharyngeal swab
hCoV-19/Mexico/MEX_INER_IMSS_00696/2021	EPI_ISL_2091152	21/03/21	North America / Mexico / State of Mexico	Human	Female	67	Hospitalized	Nasopharyngeal and oropharyngeal swab
hCoV-19/Mexico/CMX_INER_IMSS_00697/2021	EPI_ISL_2091153	21/03/21	North America / Mexico / Mexico City	Human	Female	39	Hospitalized	Oropharyngeal swab
hCoV-19/Mexico/CMX_INER_IMSS_00698/2021	EPI_ISL_2091154	22/03/21	North America / Mexico / Mexico City	Human	Male	62	Hospitalized	Oropharyngeal swab
hCoV-19/Mexico/CMX_INER_IMSS_00699/2021	EPI_ISL_2091155	22/03/21	North America / Mexico / Mexico City	Human	Male	50	Hospitalized	Oropharyngeal swab
hCoV-19/Mexico/CMX_INER_IMSS_00700/2021	EPI_ISL_2091156	22/03/21	North America / Mexico / Mexico City	Human	Female	59	Hospitalized	Oropharyngeal swab
hCoV-19/Mexico/CMX_INER_IMSS_00701/2021	EPI_ISL_2091157	21/03/21	North America / Mexico / Mexico City	Human	Male	71	Hospitalized	Oropharyngeal swab
hCoV-19/Mexico/CMX_INER_IMSS_00702/2021	EPI_ISL_2091158	21/03/21	North America / Mexico / Mexico City	Human	Female	54	Hospitalized	Oropharyngeal swab
hCoV-19/Mexico/CMX_INER_IMSS_00703/2021	EPI_ISL_2091159	20/03/21	North America / Mexico / Mexico City	Human	Female	89	Hospitalized	Oropharyngeal swab
hCoV-19/Mexico/NLE_INER_IMSS_00704/2021	EPI_ISL_2091160	04/04/21	North America / Mexico / Nuevo Leon	Human	Female	57	Ambulatory	Nasopharyngeal swab
hCoV-19/Mexico/NLE_INER_IMSS_00705/2021	EPI_ISL_2091161	04/04/21	North America / Mexico / Nuevo Leon	Human	Male	80	Hospitalized	Nasopharyngeal swab
hCoV-19/Mexico/NLE_INER_IMSS_00707/2021	EPI_ISL_2091163	05/04/21	North America / Mexico / Nuevo Leon	Human	Female	45	Ambulatory	Nasopharyngeal and oropharyngeal swab
hCoV-19/Mexico/NLE_INER_IMSS_00708/2021	EPI_ISL_2091164	05/04/21	North America / Mexico / Nuevo Leon	Human	Male	58	Ambulatory	Nasopharyngeal and oropharyngeal swab
hCoV-19/Mexico/NLE_INER_IMSS_00709/2021	EPI_ISL_2091165	05/04/21	North America / Mexico / Nuevo Leon	Human	Male	71	Hospitalized	Nasopharyngeal and oropharyngeal swab
hCoV-19/Mexico/NLE_INER_IMSS_00710/2021	EPI_ISL_2091166	05/04/21	North America / Mexico / Nuevo Leon	Human	Male	68	Hospitalized	Nasopharyngeal swab
hCoV-19/Mexico/NLE_INER_IMSS_00711/2021	EPI_ISL_2091167	05/04/21	North America / Mexico / Nuevo Leon	Human	Female	39	Hospitalized	Nasopharyngeal and oropharyngeal swab
hCoV-19/Mexico/NLE_INER_IMSS_00713/2021	EPI_ISL_2091169	05/04/21	North America / Mexico / Nuevo Leon	Human	Male	44	Ambulatory	Nasopharyngeal swab
hCoV-19/Mexico/NLE_INER_IMSS_00714/2021	EPI_ISL_2091170	05/04/21	North America / Mexico / Nuevo Leon	Human	Male	58	Hospitalized	Nasopharyngeal and oropharyngeal swab
hCoV-19/Mexico/NLE_INER_IMSS_00715/2021	EPI_ISL_2091171	02/04/21	North America / Mexico / Nuevo Leon	Human	Male	89	Hospitalized	Nasopharyngeal and oropharyngeal swab
hCoV-19/Mexico/DUR_INER_IMSS_00717/2021	EPI_ISL_2091173	01/04/21	North America / Mexico / Durango	Human	Male	90	Ambulatory	Nasopharyngeal and oropharyngeal swab
hCoV-19/Mexico/NLE_INER_IMSS_00719/2021	EPI_ISL_2091175	06/04/21	North America / Mexico / Nuevo Leon	Human	Male	26	Ambulatory	Nasopharyngeal and oropharyngeal swab
hCoV-19/Mexico/NLE_INER_IMSS_00720/2021	EPI_ISL_2091176	07/04/21	North America / Mexico / Nuevo Leon	Human	Female	36	Hospitalized	Nasopharyngeal and oropharyngeal swab
hCoV-19/Mexico/NLE_INER_IMSS_00721/2021	EPI_ISL_2091177	07/04/21	North America / Mexico / Nuevo Leon	Human	Male	79	Hospitalized	Nasopharyngeal and oropharyngeal swab
hCoV-19/Mexico/NLE_INER_IMSS_00722/2021	EPI_ISL_2091178	07/04/21	North America / Mexico / Nuevo Leon	Human	Male	48	Ambulatory	Nasopharyngeal and oropharyngeal swab
hCoV-19/Mexico/DUR_INER_IMSS_00724/2021	EPI_ISL_2091180	06/04/21	North America / Mexico / Durango	Human	Male	85	Hospitalized	Nasopharyngeal and oropharyngeal swab
hCoV-19/Mexico/TAM_INER_IMSS_00726/2021	EPI_ISL_2091182	07/04/21	North America / Mexico / Tamaulipas	Human	Male	14	Hospitalized	Nasopharyngeal swab
hCoV-19/Mexico/NLE_INER_IMSS_00727/2021	EPI_ISL_2091183	07/04/21	North America / Mexico / Nuevo Leon	Human	Male	31	Ambulatory	Nasopharyngeal and oropharyngeal swab
hCoV-19/Mexico/COA_INER_IMSS_00728/2021	EPI_ISL_2091184	06/04/21	North America / Mexico / Coahuila	Human	Male	62	Ambulatory	Nasopharyngeal and oropharyngeal swab
hCoV-19/Mexico/DUR_INER_IMSS_00729/2021	EPI_ISL_2091185	07/04/21	North America / Mexico / Durango	Human	Female	66	Hospitalized	Nasopharyngeal and oropharyngeal swab
hCoV-19/Mexico/COA_INER_IMSS_00730/2021	EPI_ISL_2091186	05/04/21	North America / Mexico / Coahuila	Human	Female	50	Ambulatory	Nasopharyngeal and oropharyngeal swab
hCoV-19/Mexico/NLE_INER_IMSS_00731/2021	EPI_ISL_2091187	08/04/21	North America / Mexico / Nuevo Leon	Human	Female	76	Ambulatory	Nasopharyngeal and oropharyngeal swab
hCoV-19/Mexico/NLE_INER_IMSS_00732/2021	EPI_ISL_2091188	08/04/21	North America / Mexico / Nuevo Leon	Human	Female	88	Hospitalized	Nasopharyngeal swab
hCoV-19/Mexico/CHH_INER_IMSS_00734/2021	EPI_ISL_2091190	04/04/21	North America / Mexico / Chihuahua	Human	Male	60	Ambulatory	Nasopharyngeal swab
hCoV-19/Mexico/NLE_INER_IMSS_00736/2021	EPI_ISL_2091192	08/04/21	North America / Mexico / Nuevo Leon	Human	Female	61	Hospitalized	Nasopharyngeal and oropharyngeal swab
hCoV-19/Mexico/DUR_INER_IMSS_00737/2021	EPI_ISL_2091193	08/04/21	North America / Mexico / Durango	Human	Female	66	Hospitalized	Nasopharyngeal and oropharyngeal swab
hCoV-19/Mexico/DUR_INER_IMSS_00738/2021	EPI_ISL_2091194	07/04/21	North America / Mexico / Durango	Human	Female	64	Hospitalized	Nasopharyngeal and oropharyngeal swab
hCoV-19/Mexico/CHH_INER_IMSS_00739/2021	EPI_ISL_2091195	06/04/21	North America / Mexico / Chihuahua	Human	Female	73	Hospitalized	Nasopharyngeal and oropharyngeal swab

hCoV-19/Mexico/CHH_INER_IMSS_00742/2021	EPI_ISL_2091197	06/04/21	North America / Mexico / Chihuahua	Human	Male	71	Hospitalized	Nasopharyngeal and oropharyngeal swab
hCoV-19/Mexico/NLE_INER_IMSS_00747/2021	EPI_ISL_2091202	12/04/21	North America / Mexico / Nuevo Leon	Human	Female	39	Hospitalized	Nasopharyngeal and oropharyngeal swab
hCoV-19/Mexico/NLE_INER_IMSS_00748/2021	EPI_ISL_2091203	12/04/21	North America / Mexico / Nuevo Leon	Human	Male	85	Hospitalized	Nasopharyngeal and oropharyngeal swab
hCoV-19/Mexico/NLE_INER_IMSS_00749/2021	EPI_ISL_2091204	12/04/21	North America / Mexico / Nuevo Leon	Human	Female	52	Ambulatory	Nasopharyngeal and oropharyngeal swab
hCoV-19/Mexico/NLE_INER_IMSS_00750/2021	EPI_ISL_2091205	12/04/21	North America / Mexico / Nuevo Leon	Human	Female	74	Hospitalized	Nasopharyngeal and oropharyngeal swab
hCoV-19/Mexico/NLE_INER_IMSS_00752/2021	EPI_ISL_2091206	13/04/21	North America / Mexico / Nuevo Leon	Human	Female	54	Hospitalized	Nasopharyngeal and oropharyngeal swab
hCoV-19/Mexico/COA_INER_IMSS_00755/2021	EPI_ISL_2091209	11/04/21	North America / Mexico / Coahuila	Human	Female	77	Hospitalized	Nasopharyngeal and oropharyngeal swab
hCoV-19/Mexico/NLE_INER_IMSS_00756/2021	EPI_ISL_2091210	13/04/21	North America / Mexico / Nuevo Leon	Human	Female	70	Hospitalized	Nasopharyngeal and oropharyngeal swab
hCoV-19/Mexico/NLE_INER_IMSS_00757/2021	EPI_ISL_2091211	13/04/21	North America / Mexico / Nuevo Leon	Human	Female	65	Hospitalized	Nasopharyngeal and oropharyngeal swab
hCoV-19/Mexico/CHH_INER_IMSS_00759/2021	EPI_ISL_2091213	06/04/21	North America / Mexico / Chihuahua	Human	Female	33	Ambulatory	Nasopharyngeal and oropharyngeal swab
hCoV-19/Mexico/CHH_INER_IMSS_00760/2021	EPI_ISL_2091214	11/04/21	North America / Mexico / Chihuahua	Human	Female	25	Ambulatory	Nasopharyngeal swab
hCoV-19/Mexico/NLE_INER_IMSS_00761/2021	EPI_ISL_2091215	15/04/21	North America / Mexico / Nuevo Leon	Human	Female	76	Ambulatory	Nasopharyngeal and oropharyngeal swab
hCoV-19/Mexico/NLE_INER_IMSS_00764/2021	EPI_ISL_2091218	15/04/21	North America / Mexico / Nuevo Leon	Human	Male	69	Ambulatory	Nasopharyngeal and oropharyngeal swab
hCoV-19/Mexico/CHH_INER_IMSS_00766/2021	EPI_ISL_2091220	12/04/21	North America / Mexico / Chihuahua	Human	Male	65	Hospitalized	Nasopharyngeal swab
hCoV-19/Mexico/NLE_INER_IMSS_00769/2021	EPI_ISL_2091223	16/04/21	North America / Mexico / Nuevo Leon	Human	Female	24	Ambulatory	Nasopharyngeal and oropharyngeal swab
hCoV-19/Mexico/NLE_INER_IMSS_00770/2021	EPI_ISL_2091224	16/04/21	North America / Mexico / Nuevo Leon	Human	Female	42	Hospitalized	Nasopharyngeal and oropharyngeal swab
hCoV-19/Mexico/YUC_INER_IMSS_00772/2021	EPI_ISL_2091226	31/03/21	North America / Mexico / Yucatan	Human	Male	45	Ambulatory	Nasopharyngeal and oropharyngeal swab
hCoV-19/Mexico/YUC_INER_IMSS_00773/2021	EPI_ISL_2091227	31/03/21	North America / Mexico / Yucatan	Human	Female	81	Hospitalized	Nasopharyngeal and oropharyngeal swab
hCoV-19/Mexico/YUC_INER_IMSS_00775/2021	EPI_ISL_2091229	04/04/21	North America / Mexico / Yucatan	Human	Female	71	Hospitalized	Nasopharyngeal and oropharyngeal swab
hCoV-19/Mexico/YUC_INER_IMSS_00776/2021	EPI_ISL_2091230	06/04/21	North America / Mexico / Yucatan	Human	Female	47	Ambulatory	Nasopharyngeal and oropharyngeal swab
hCoV-19/Mexico/YUC_INER_IMSS_00777/2021	EPI_ISL_2091231	07/04/21	North America / Mexico / Yucatan	Human	Female	45	Ambulatory	Nasopharyngeal and oropharyngeal swab
hCoV-19/Mexico/TAB_INER_IMSS_00779/2021	EPI_ISL_2091233	07/04/21	North America / Mexico / Tabasco	Human	Female	52	Hospitalized	Nasopharyngeal and oropharyngeal swab
hCoV-19/Mexico/YUC_INER_IMSS_00780/2021	EPI_ISL_2091234	08/04/21	North America / Mexico / Yucatan	Human	Male	26	Ambulatory	Nasopharyngeal and oropharyngeal swab
hCoV-19/Mexico/YUC_INER_IMSS_00781/2021	EPI_ISL_2091235	08/04/21	North America / Mexico / Yucatan	Human	Male	62	Ambulatory	Nasopharyngeal and oropharyngeal swab
hCoV-19/Mexico/YUC_INER_IMSS_00783/2021	EPI_ISL_2091237	09/04/21	North America / Mexico / Yucatan	Human	Female	57	Ambulatory	Nasopharyngeal and oropharyngeal swab
hCoV-19/Mexico/YUC_INER_IMSS_00784/2021	EPI_ISL_2091238	09/04/21	North America / Mexico / Yucatan	Human	Female	55	Ambulatory	Nasopharyngeal and oropharyngeal swab
hCoV-19/Mexico/ROO_INER_IMSS_00787/2021	EPI_ISL_2091240	07/04/21	North America / Mexico / Quintana Roo	Human	Male	34	Hospitalized	Nasopharyngeal and oropharyngeal swab
hCoV-19/Mexico/CAM_INER_IMSS_00788/2021	EPI_ISL_2091241	07/04/21	North America / Mexico / Campeche	Human	Female	56	Hospitalized	Nasopharyngeal and oropharyngeal swab
hCoV-19/Mexico/TAB_INER_IMSS_00793/2021	EPI_ISL_2091246	08/04/21	North America / Mexico / Tabasco	Human	Male	35	Ambulatory	Nasopharyngeal and oropharyngeal swab
hCoV-19/Mexico/YUC_INER_IMSS_00794/2021	EPI_ISL_2091247	10/04/21	North America / Mexico / Yucatan	Human	Male	75	Hospitalized	Nasopharyngeal and oropharyngeal swab
hCoV-19/Mexico/CAM_INER_IMSS_00795/2021	EPI_ISL_2091248	09/04/21	North America / Mexico / Campeche	Human	Male	49	Ambulatory	Nasopharyngeal and oropharyngeal swab
hCoV-19/Mexico/CAM_INER_IMSS_00796/2021	EPI_ISL_2091249	09/04/21	North America / Mexico / Campeche	Human	Male	40	Ambulatory	Nasopharyngeal and oropharyngeal swab
hCoV-19/Mexico/CAM_INER_IMSS_00797/2021	EPI_ISL_2091250	09/04/21	North America / Mexico / Campeche	Human	Male	46	Ambulatory	Nasopharyngeal and oropharyngeal swab
hCoV-19/Mexico/YUC_INER_IMSS_00798/2021	EPI_ISL_2091251	12/04/21	North America / Mexico / Yucatan	Human	Female	58	Ambulatory	Nasopharyngeal and oropharyngeal swab
hCoV-19/Mexico/CAM_INER_IMSS_00799/2021	EPI_ISL_2091252	10/04/21	North America / Mexico / Campeche	Human	Male	33	Ambulatory	Nasopharyngeal and oropharyngeal swab
hCoV-19/Mexico/CAM_INER_IMSS_00800/2021	EPI_ISL_2091253	10/04/21	North America / Mexico / Campeche	Human	Male	24	Ambulatory	Nasopharyngeal and oropharyngeal swab
hCoV-19/Mexico/TAB_INER_IMSS_00802/2021	EPI_ISL_2091254	10/04/21	North America / Mexico / Tabasco	Human	Female	55	Hospitalized	Nasopharyngeal and oropharyngeal swab
hCoV-19/Mexico/YUC_INER_IMSS_00803/2021	EPI_ISL_2091255	13/04/21	North America / Mexico / Yucatan	Human	Female	40	Ambulatory	Nasopharyngeal and oropharyngeal swab
hCoV-19/Mexico/YUC_INER_IMSS_00804/2021	EPI_ISL_2091256	12/04/21	North America / Mexico / Yucatan	Human	Female	34	Ambulatory	Nasopharyngeal and oropharyngeal swab
hCoV-19/Mexico/NAY_INER_IMSS_00807/2021	EPI_ISL_2091259	05/04/21	North America / Mexico / Nayarit	Human	Female	74	Hospitalized	Nasopharyngeal and oropharyngeal swab
hCoV-19/Mexico/GUA_INER_IMSS_00809/2021	EPI_ISL_2091260	04/04/21	North America / Mexico / Guanajuato	Human	Male	51	Hospitalized	Nasopharyngeal and oropharyngeal swab
hCoV-19/Mexico/GUA_INER_IMSS_00810/2021	EPI_ISL_2091261	01/04/21	North America / Mexico / Guanajuato	Human	Male	50	Ambulatory	Nasopharyngeal and oropharyngeal swab
hCoV-19/Mexico/JAL_INER_IMSS_00811/2021	EPI_ISL_2091262	04/04/21	North America / Mexico / Jalisco	Human	Female	37	Hospitalized	Nasopharyngeal and oropharyngeal swab
hCoV-19/Mexico/JAL_INER_IMSS_00812/2021	EPI_ISL_2091263	31/03/21	North America / Mexico / Jalisco	Human	Female	64	Hospitalized	Nasopharyngeal swab

hCoV-19/Mexico/SIN_INER_IMSS_00813/2021	EPI_ISL_2091264	04/04/21	North America / Mexico / Sinaloa	Human	Male	44	Ambulatory	Nasopharyngeal and oropharyngeal swab
hCoV-19/Mexico/SIN_INER_IMSS_00814/2021	EPI_ISL_2091265	04/04/21	North America / Mexico / Sinaloa	Human	Female	52	Hospitalized	Nasopharyngeal and oropharyngeal swab
hCoV-19/Mexico/JAL_INER_IMSS_00815/2021	EPI_ISL_2091266	08/04/21	North America / Mexico / Jalisco	Human	Male	28	Ambulatory	Nasopharyngeal and oropharyngeal swab
hCoV-19/Mexico/JAL_INER_IMSS_00816/2021	EPI_ISL_2091267	08/04/21	North America / Mexico / Jalisco	Human	Female	72	Hospitalized	Nasopharyngeal and oropharyngeal swab
hCoV-19/Mexico/SIN_INER_IMSS_00817/2021	EPI_ISL_2091268	07/04/21	North America / Mexico / Sinaloa	Human	Female	54	Hospitalized	Nasopharyngeal and oropharyngeal swab
hCoV-19/Mexico/JAL_INER_IMSS_00818/2021	EPI_ISL_2091269	08/04/21	North America / Mexico / Jalisco	Human	Female	53	Hospitalized	Nasopharyngeal and oropharyngeal swab
hCoV-19/Mexico/SIN_INER_IMSS_00820/2021	EPI_ISL_2091271	06/04/21	North America / Mexico / Sinaloa	Human	Male	67	Hospitalized	Nasopharyngeal and oropharyngeal swab
hCoV-19/Mexico/JAL_INER_IMSS_00821/2021	EPI_ISL_2091272	09/04/21	North America / Mexico / Jalisco	Human	Female	33	Ambulatory	Nasopharyngeal and oropharyngeal swab
hCoV-19/Mexico/JAL_INER_IMSS_00823/2021	EPI_ISL_2091274	09/04/21	North America / Mexico / Jalisco	Human	Male	42	Ambulatory	Nasopharyngeal and oropharyngeal swab
hCoV-19/Mexico/JAL_INER_IMSS_00825/2021	EPI_ISL_2091276	09/04/21	North America / Mexico / Jalisco	Human	Male	74	Hospitalized	Nasopharyngeal and oropharyngeal swab
hCoV-19/Mexico/NAY_INER_IMSS_00827/2021	EPI_ISL_2091278	07/04/21	North America / Mexico / Nayarit	Human	Male	31	Ambulatory	Nasopharyngeal and oropharyngeal swab
hCoV-19/Mexico/JAL_INER_IMSS_00829/2021	EPI_ISL_2091280	09/04/21	North America / Mexico / Jalisco	Human	Male	76	Hospitalized	Nasopharyngeal and oropharyngeal swab
hCoV-19/Mexico/GUA_INER_IMSS_00832/2021	EPI_ISL_2091283	09/04/21	North America / Mexico / Guanajuato	Human	Female	64	Hospitalized	Nasopharyngeal and oropharyngeal swab
hCoV-19/Mexico/JAL_INER_IMSS_00833/2021	EPI_ISL_2091284	10/04/21	North America / Mexico / Jalisco	Human	Female	62	Hospitalized	Oropharyngeal swab
hCoV-19/Mexico/JAL_INER_IMSS_00835/2021	EPI_ISL_2091286	11/04/21	North America / Mexico / Jalisco	Human	Male	14	Ambulatory	Nasopharyngeal and oropharyngeal swab
hCoV-19/Mexico/JAL_INER_IMSS_00838/2021	EPI_ISL_2091289	12/04/21	North America / Mexico / Jalisco	Human	Female	57	Ambulatory	Nasopharyngeal and oropharyngeal swab
hCoV-19/Mexico/SIN_INER_IMSS_00842/2021	EPI_ISL_2091293	11/04/21	North America / Mexico / Sinaloa	Human	Female	52	Hospitalized	Nasopharyngeal and oropharyngeal swab
hCoV-19/Mexico/JAL_INER_IMSS_00843/2021	EPI_ISL_2091294	12/04/21	North America / Mexico / Jalisco	Human	Female	45	Hospitalized	Nasopharyngeal and oropharyngeal swab
hCoV-19/Mexico/JAL_INER_IMSS_00844/2021	EPI_ISL_2091295	12/04/21	North America / Mexico / Jalisco	Human	Male	76	Hospitalized	Nasopharyngeal and oropharyngeal swab
hCoV-19/Mexico/JAL_INER_IMSS_00845/2021	EPI_ISL_2091296	12/04/21	North America / Mexico / Jalisco	Human	Female	87	Hospitalized	Nasopharyngeal and oropharyngeal swab
hCoV-19/Mexico/GUA_INER_IMSS_00847/2021	EPI_ISL_2091298	09/04/21	North America / Mexico / Guanajuato	Human	Female	46	Hospitalized	Nasopharyngeal and oropharyngeal swab
hCoV-19/Mexico/GUA_INER_IMSS_00848/2021	EPI_ISL_2091299	12/04/21	North America / Mexico / Guanajuato	Human	Male	24	Ambulatory	Oropharyngeal swab
hCoV-19/Mexico/MIC_INER_IMSS_00849/2021	EPI_ISL_2091300	08/04/21	North America / Mexico / Michoacan	Human	Male	71	Hospitalized	Nasopharyngeal and oropharyngeal swab
hCoV-19/Mexico/NAY_INER_IMSS_00850/2021	EPI_ISL_2091301	13/04/21	North America / Mexico / Nayarit	Human	Male	50	Hospitalized	Nasopharyngeal and oropharyngeal swab
hCoV-19/Mexico/SON_INER_IMSS_00851/2021	EPI_ISL_2091302	12/04/21	North America / Mexico / Sonora	Human	Male	49	Hospitalized	Nasopharyngeal swab
hCoV-19/Mexico/JAL_INER_IMSS_00852/2021	EPI_ISL_2091303	14/04/21	North America / Mexico / Jalisco	Human	Female	82	Hospitalized	Nasopharyngeal and oropharyngeal swab
hCoV-19/Mexico/JAL_INER_IMSS_00853/2021	EPI_ISL_2091304	14/04/21	North America / Mexico / Jalisco	Human	Female	52	Hospitalized	Oropharyngeal swab
hCoV-19/Mexico/JAL_INER_IMSS_00854/2021	EPI_ISL_2091305	12/04/21	North America / Mexico / Jalisco	Human	Male	99	Hospitalized	Nasopharyngeal and oropharyngeal swab
hCoV-19/Mexico/MIC_INER_IMSS_00855/2021	EPI_ISL_2091306	08/04/21	North America / Mexico / Michoacan	Human	Male	53	Ambulatory	Nasopharyngeal and oropharyngeal swab
hCoV-19/Mexico/MIC_INER_IMSS_00856/2021	EPI_ISL_2091307	12/04/21	North America / Mexico / Michoacan	Human	Male	55	Hospitalized	Nasopharyngeal and oropharyngeal swab
hCoV-19/Mexico/JAL_INER_IMSS_00858/2021	EPI_ISL_2091309	14/04/21	North America / Mexico / Jalisco	Human	Male	61	Hospitalized	Nasopharyngeal and oropharyngeal swab
hCoV-19/Mexico/JAL_INER_IMSS_00859/2021	EPI_ISL_2091310	15/04/21	North America / Mexico / Jalisco	Human	Male	49	Ambulatory	Nasopharyngeal and oropharyngeal swab
hCoV-19/Mexico/GUA_INER_IMSS_00860/2021	EPI_ISL_2091311	13/04/21	North America / Mexico / Guanajuato	Human	Male	30	Ambulatory	Nasopharyngeal and oropharyngeal swab
hCoV-19/Mexico/JAL_INER_IMSS_00862/2021	EPI_ISL_2091313	15/04/21	North America / Mexico / Jalisco	Human	Male	39	Ambulatory	Nasopharyngeal and oropharyngeal swab
hCoV-19/Mexico/NAY_INER_IMSS_00863/2021	EPI_ISL_2091314	14/04/21	North America / Mexico / Nayarit	Human	Female	48	Hospitalized	Nasopharyngeal and oropharyngeal swab
hCoV-19/Mexico/GUA_INER_IMSS_00864/2021	EPI_ISL_2091315	14/04/21	North America / Mexico / Guanajuato	Human	Female	40	Ambulatory	Nasopharyngeal and oropharyngeal swab
hCoV-19/Mexico/GUA_INER_IMSS_00865/2021	EPI_ISL_2091316	15/04/21	North America / Mexico / Guanajuato	Human	Female	53	Ambulatory	Nasopharyngeal and oropharyngeal swab
hCoV-19/Mexico/MIC_INER_IMSS_00868/2021	EPI_ISL_2091319	16/04/21	North America / Mexico / Michoacan	Human	Female	83	Hospitalized	Nasopharyngeal and oropharyngeal swab
hCoV-19/Mexico/PUE_INER_IMSS_00869/2021	EPI_ISL_2091320	06/04/21	North America / Mexico / Puebla	Human	Female	31	Ambulatory	Oropharyngeal swab
hCoV-19/Mexico/PUE_INER_IMSS_00870/2021	EPI_ISL_2091321	06/04/21	North America / Mexico / Puebla	Human	Male	35	Ambulatory	Nasopharyngeal and oropharyngeal swab
hCoV-19/Mexico/PUE_INER_IMSS_00871/2021	EPI_ISL_2091322	06/04/21	North America / Mexico / Puebla	Human	Female	32	Ambulatory	Nasopharyngeal and oropharyngeal swab
hCoV-19/Mexico/CMX_INER_IMSS_00872/2021	EPI_ISL_2091323	09/04/21	North America / Mexico / Mexico City	Human	Male	69	Hospitalized	Nasopharyngeal and oropharyngeal swab
hCoV-19/Mexico/CMX_INER_IMSS_00873/2021	EPI_ISL_2091324	08/04/21	North America / Mexico / Mexico City	Human	Female	54	Ambulatory	Nasopharyngeal and oropharyngeal swab
hCoV-19/Mexico/CMX_INER_IMSS_00874/2021	EPI_ISL_2091325	08/04/21	North America / Mexico / Mexico City	Human	Male	36	Ambulatory	Nasopharyngeal and oropharyngeal swab

hCoV-19/Mexico/CMX_INER_IMSS_00875/2021	EPI_ISL_2091326	08/04/21	North America / Mexico / Mexico City	Human	Male	75	Hospitalized	Nasopharyngeal and oropharyngeal swab
hCoV-19/Mexico/CMX_INER_IMSS_00876/2021	EPI_ISL_2091327	08/04/21	North America / Mexico / Mexico City	Human	Female	55	Ambulatory	Nasopharyngeal swab
hCoV-19/Mexico/CMX_INER_IMSS_00877/2021	EPI_ISL_2091328	08/04/21	North America / Mexico / Mexico City	Human	Male	69	Hospitalized	Nasopharyngeal swab
hCoV-19/Mexico/CMX_INER_IMSS_00879/2021	EPI_ISL_2091330	08/04/21	North America / Mexico / Mexico City	Human	Male	47	Hospitalized	Oropharyngeal swab
hCoV-19/Mexico/CMX_INER_IMSS_00880/2021	EPI_ISL_2091331	09/04/21	North America / Mexico / Mexico City	Human	Female	26	Ambulatory	Nasopharyngeal and oropharyngeal swab
hCoV-19/Mexico/MEX_INER_IMSS_00881/2021	EPI_ISL_2091332	07/04/21	North America / Mexico / State of Mexico	Human	Male	30	Hospitalized	Oropharyngeal swab
hCoV-19/Mexico/AGU_INER_IMSS_00882/2021	EPI_ISL_2091333	05/04/21	North America / Mexico / Aguascalientes	Human	Male	71	Ambulatory	Nasopharyngeal and oropharyngeal swab
hCoV-19/Mexico/AGU_INER_IMSS_00883/2021	EPI_ISL_2091334	05/04/21	North America / Mexico / Aguascalientes	Human	Female	29	Ambulatory	Nasopharyngeal and oropharyngeal swab
hCoV-19/Mexico/PUE_INER_IMSS_00885/2021	EPI_ISL_2091335	07/04/21	North America / Mexico / Puebla	Human	Male	87	Hospitalized	Nasopharyngeal and oropharyngeal swab
hCoV-19/Mexico/PUE_INER_IMSS_00886/2021	EPI_ISL_2091336	07/04/21	North America / Mexico / Puebla	Human	Female	52	Hospitalized	Nasopharyngeal and oropharyngeal swab
hCoV-19/Mexico/PUE_INER_IMSS_00887/2021	EPI_ISL_2091337	08/04/21	North America / Mexico / Puebla	Human	Male	30	Ambulatory	Nasopharyngeal swab
hCoV-19/Mexico/MEX_INER_IMSS_00888/2021	EPI_ISL_2091338	09/04/21	North America / Mexico / State of Mexico	Human	Male	64	Hospitalized	Nasopharyngeal and oropharyngeal swab
hCoV-19/Mexico/PUE_INER_IMSS_00889/2021	EPI_ISL_2091339	07/04/21	North America / Mexico / Puebla	Human	Male	59	Ambulatory	Nasopharyngeal and oropharyngeal swab
hCoV-19/Mexico/PUE_INER_IMSS_00891/2021	EPI_ISL_2091340	08/04/21	North America / Mexico / Puebla	Human	Female	47	Ambulatory	Nasopharyngeal and oropharyngeal swab
hCoV-19/Mexico/PUE_INER_IMSS_00892/2021	EPI_ISL_2091341	08/04/21	North America / Mexico / Puebla	Human	Female	31	Ambulatory	Nasopharyngeal and oropharyngeal swab
hCoV-19/Mexico/VER_INER_IMSS_00893/2021	EPI_ISL_2091342	08/04/21	North America / Mexico / Veracruz	Human	Male	30	Ambulatory	Nasopharyngeal and oropharyngeal swab
hCoV-19/Mexico/CMX_INER_IMSS_00894/2021	EPI_ISL_2091343	09/04/21	North America / Mexico / Mexico City	Human	Male	58	Ambulatory	Nasopharyngeal and oropharyngeal swab
hCoV-19/Mexico/CMX_INER_IMSS_00895/2021	EPI_ISL_2091344	10/04/21	North America / Mexico / Mexico City	Human	Female	88	Hospitalized	Oropharyngeal swab
hCoV-19/Mexico/CMX_INER_IMSS_00896/2021	EPI_ISL_2091345	10/04/21	North America / Mexico / Mexico City	Human	Male	83	Hospitalized	Oropharyngeal swab
hCoV-19/Mexico/CMX_INER_IMSS_00897/2021	EPI_ISL_2091346	10/04/21	North America / Mexico / Mexico City	Human	Female	67	Hospitalized	Oropharyngeal swab
hCoV-19/Mexico/CMX_INER_IMSS_00898/2021	EPI_ISL_2091347	10/04/21	North America / Mexico / Mexico City	Human	Male	44	Ambulatory	Oropharyngeal swab
hCoV-19/Mexico/CMX_INER_IMSS_00899/2021	EPI_ISL_2091348	08/04/21	North America / Mexico / Mexico City	Human	Male	42	Ambulatory	Nasopharyngeal and oropharyngeal swab
hCoV-19/Mexico/CMX_INER_IMSS_00900/2021	EPI_ISL_2091349	12/04/21	North America / Mexico / Mexico City	Human	Male	68	Hospitalized	Nasopharyngeal and oropharyngeal swab
hCoV-19/Mexico/CMX_INER_IMSS_00901/2021	EPI_ISL_2091350	11/04/21	North America / Mexico / Mexico City	Human	Male	64	Hospitalized	Nasopharyngeal and oropharyngeal swab
hCoV-19/Mexico/CMX_INER_IMSS_00902/2021	EPI_ISL_2091351	10/04/21	North America / Mexico / Mexico City	Human	Male	68	Ambulatory	Nasopharyngeal and oropharyngeal swab
hCoV-19/Mexico/CMX_INER_IMSS_00903/2021	EPI_ISL_2091352	10/04/21	North America / Mexico / Mexico City	Human	Female	43	Ambulatory	Oropharyngeal swab
hCoV-19/Mexico/MEX_INER_IMSS_00904/2021	EPI_ISL_2091353	12/04/21	North America / Mexico / State of Mexico	Human	Female	61	Hospitalized	Nasopharyngeal and oropharyngeal swab
hCoV-19/Mexico/CMX_INER_IMSS_00905/2021	EPI_ISL_2091354	08/04/21	North America / Mexico / Mexico City	Human	Female	86	Ambulatory	Nasopharyngeal and oropharyngeal swab
hCoV-19/Mexico/CMX_INER_IMSS_00906/2021	EPI_ISL_2091355	08/04/21	North America / Mexico / Mexico City	Human	Male	44	Ambulatory	Nasopharyngeal and oropharyngeal swab
hCoV-19/Mexico/CMX_INER_IMSS_00907/2021	EPI_ISL_2091356	09/04/21	North America / Mexico / Mexico City	Human	Male	39	Ambulatory	Nasopharyngeal and oropharyngeal swab
hCoV-19/Mexico/CMX_INER_IMSS_00908/2021	EPI_ISL_2091357	09/04/21	North America / Mexico / Mexico City	Human	Male	14	Ambulatory	Nasopharyngeal and oropharyngeal swab
hCoV-19/Mexico/CMX_INER_IMSS_00909/2021	EPI_ISL_2091358	09/04/21	North America / Mexico / Mexico City	Human	Male	19	Ambulatory	Nasopharyngeal and oropharyngeal swab
hCoV-19/Mexico/CMX_INER_IMSS_00910/2021	EPI_ISL_2091359	09/04/21	North America / Mexico / Mexico City	Human	Female	43	Ambulatory	Nasopharyngeal and oropharyngeal swab
hCoV-19/Mexico/PUE_INER_IMSS_00911/2021	EPI_ISL_2091360	09/04/21	North America / Mexico / Puebla	Human	Male	19	Ambulatory	Nasopharyngeal and oropharyngeal swab
hCoV-19/Mexico/PUE_INER_IMSS_00912/2021	EPI_ISL_2091361	10/04/21	North America / Mexico / Puebla	Human	Female	24	Ambulatory	Nasopharyngeal and oropharyngeal swab
hCoV-19/Mexico/CMX_INER_IMSS_00914/2021	EPI_ISL_2091362	12/04/21	North America / Mexico / Mexico City	Human	Male	37	Ambulatory	Nasopharyngeal and oropharyngeal swab
hCoV-19/Mexico/CMX_INER_IMSS_00915/2021	EPI_ISL_2091363	12/04/21	North America / Mexico / Mexico City	Human	Female	74	Hospitalized	Nasopharyngeal swab
hCoV-19/Mexico/CMX_INER_IMSS_00916/2021	EPI_ISL_2091364	13/04/21	North America / Mexico / Mexico City	Human	Female	73	Hospitalized	Nasopharyngeal and oropharyngeal swab
hCoV-19/Mexico/CMX_INER_IMSS_00917/2021	EPI_ISL_2091365	13/04/21	North America / Mexico / Mexico City	Human	Female	59	Hospitalized	Nasopharyngeal swab
hCoV-19/Mexico/CMX_INER_IMSS_00918/2021	EPI_ISL_2091366	13/04/21	North America / Mexico / Mexico City	Human	Male	74	Hospitalized	Nasopharyngeal swab
hCoV-19/Mexico/VER_INER_IMSS_00919/2021	EPI_ISL_2091367	13/04/21	North America / Mexico / Veracruz	Human	Male	54	Hospitalized	Nasopharyngeal and oropharyngeal swab
hCoV-19/Mexico/VER_INER_IMSS_00921/2021	EPI_ISL_2091369	13/04/21	North America / Mexico / Veracruz	Human	Male	16	Ambulatory	Nasopharyngeal and oropharyngeal swab
hCoV-19/Mexico/PUE_INER_IMSS_00922/2021	EPI_ISL_2091370	13/04/21	North America / Mexico / Puebla	Human	Female	27	Hospitalized	Nasopharyngeal and oropharyngeal swab
hCoV-19/Mexico/PUE_INER_IMSS_00926/2021	EPI_ISL_2091371	13/04/21	North America / Mexico / Puebla	Human	Male	29	Hospitalized	Nasopharyngeal and oropharyngeal swab

hCoV-19/Mexico/PUE_INER_IMSS_00927/2021	EPI_ISL_2091372	13/04/21	North America / Mexico / Puebla	Human	Male	46	Hospitalized	Nasopharyngeal and oropharyngeal swab
hCoV-19/Mexico/PUE_INER_IMSS_00928/2021	EPI_ISL_2091373	12/04/21	North America / Mexico / Puebla	Human	Female	22	Ambulatory	Oropharyngeal swab
hCoV-19/Mexico/PUE_INER_IMSS_00929/2021	EPI_ISL_2091374	13/04/21	North America / Mexico / Puebla	Human	Female	29	Ambulatory	Oropharyngeal swab
hCoV-19/Mexico/PUE_INER_IMSS_00930/2021	EPI_ISL_2091375	12/04/21	North America / Mexico / Puebla	Human	Female	42	Ambulatory	Nasopharyngeal and oropharyngeal swab
hCoV-19/Mexico/PUE_INER_IMSS_00931/2021	EPI_ISL_2091376	12/04/21	North America / Mexico / Puebla	Human	Male	41	Ambulatory	Nasopharyngeal swab
hCoV-19/Mexico/MEX_INER_IMSS_00932/2021	EPI_ISL_2091377	12/04/21	North America / Mexico / State of Mexico	Human	Female	29	Ambulatory	Nasopharyngeal and oropharyngeal swab
hCoV-19/Mexico/SLP_INER_IMSS_00933/2021	EPI_ISL_2091378	12/04/21	North America / Mexico / San Luis Potosi	Human	Female	62	Hospitalized	Nasopharyngeal and oropharyngeal swab
hCoV-19/Mexico/SLP_INER_IMSS_00934/2021	EPI_ISL_2091379	12/04/21	North America / Mexico / San Luis Potosi	Human	Female	66	Hospitalized	Nasopharyngeal and oropharyngeal swab
hCoV-19/Mexico/CMX_INER_IMSS_00936/2021	EPI_ISL_2091381	13/04/21	North America / Mexico / Mexico City	Human	Female	56	Ambulatory	Nasopharyngeal and oropharyngeal swab
hCoV-19/Mexico/CMX_INER_IMSS_00937/2021	EPI_ISL_2091382	14/04/21	North America / Mexico / Mexico City	Human	Female	61	Hospitalized	Nasopharyngeal and oropharyngeal swab
hCoV-19/Mexico/CMX_INER_IMSS_00938/2021	EPI_ISL_2091383	14/04/21	North America / Mexico / Mexico City	Human	Female	64	Hospitalized	Nasopharyngeal and oropharyngeal swab
hCoV-19/Mexico/MEX_INER_IMSS_00939/2021	EPI_ISL_2091384	12/04/21	North America / Mexico / State of Mexico	Human	Female	56	Ambulatory	Oropharyngeal swab
hCoV-19/Mexico/MEX_INER_IMSS_00941/2021	EPI_ISL_2091385	14/04/21	North America / Mexico / State of Mexico	Human	Male	33	Ambulatory	Oropharyngeal swab
hCoV-19/Mexico/MEX_INER_IMSS_00942/2021	EPI_ISL_2091386	13/04/21	North America / Mexico / State of Mexico	Human	Female	66	Ambulatory	Oropharyngeal swab
hCoV-19/Mexico/MEX_INER_IMSS_00943/2021	EPI_ISL_2091387	13/04/21	North America / Mexico / State of Mexico	Human	Female	49	Ambulatory	Oropharyngeal swab
hCoV-19/Mexico/MEX_INER_IMSS_00944/2021	EPI_ISL_2091388	13/04/21	North America / Mexico / State of Mexico	Human	Male	58	Ambulatory	Oropharyngeal swab
hCoV-19/Mexico/MOR_INER_IMSS_00945/2021	EPI_ISL_2091389	14/04/21	North America / Mexico / Morelos	Human	Male	85	Hospitalized	Nasopharyngeal and oropharyngeal swab
hCoV-19/Mexico/MOR_INER_IMSS_00946/2021	EPI_ISL_2091390	14/04/21	North America / Mexico / Morelos	Human	Female	52	Hospitalized	Nasopharyngeal and oropharyngeal swab
hCoV-19/Mexico/CHP_INER_IMSS_00947/2021	EPI_ISL_2091391	13/04/21	North America / Mexico / Chiapas	Human	Female	51	Ambulatory	Nasopharyngeal and oropharyngeal swab
hCoV-19/Mexico/PUE_INER_IMSS_00948/2021	EPI_ISL_2091392	14/04/21	North America / Mexico / Puebla	Human	Male	55	Hospitalized	Nasopharyngeal and oropharyngeal swab
hCoV-19/Mexico/PUE_INER_IMSS_00949/2021	EPI_ISL_2091393	14/04/21	North America / Mexico / Puebla	Human	Male	35	Hospitalized	Nasopharyngeal and oropharyngeal swab
hCoV-19/Mexico/PUE_INER_IMSS_00950/2021	EPI_ISL_2091394	14/04/21	North America / Mexico / Puebla	Human	Male	54	Hospitalized	Nasopharyngeal and oropharyngeal swab
hCoV-19/Mexico/PUE_INER_IMSS_00951/2021	EPI_ISL_2091395	14/04/21	North America / Mexico / Puebla	Human	Female	55	Hospitalized	Nasopharyngeal and oropharyngeal swab
hCoV-19/Mexico/PUE_INER_IMSS_00953/2021	EPI_ISL_2091396	14/04/21	North America / Mexico / Puebla	Human	Male	30	Hospitalized	Nasopharyngeal and oropharyngeal swab
hCoV-19/Mexico/PUE_INER_IMSS_00955/2021	EPI_ISL_2091397	12/04/21	North America / Mexico / Puebla	Human	Female	13	Ambulatory	Oropharyngeal swab
hCoV-19/Mexico/PUE_INER_IMSS_00956/2021	EPI_ISL_2091398	13/04/21	North America / Mexico / Puebla	Human	Female	50	Ambulatory	Oropharyngeal swab
hCoV-19/Mexico/PUE_INER_IMSS_00957/2021	EPI_ISL_2091399	13/04/21	North America / Mexico / Puebla	Human	Female	30	Ambulatory	Nasopharyngeal and oropharyngeal swab
hCoV-19/Mexico/PUE_INER_IMSS_00959/2021	EPI_ISL_2091400	14/04/21	North America / Mexico / Puebla	Human	Male	53	Ambulatory	Nasopharyngeal and oropharyngeal swab
hCoV-19/Mexico/PUE_INER_IMSS_00960/2021	EPI_ISL_2091401	14/04/21	North America / Mexico / Puebla	Human	Female	28	Ambulatory	Oropharyngeal swab
hCoV-19/Mexico/PUE_INER_IMSS_00961/2021	EPI_ISL_2091402	13/04/21	North America / Mexico / Puebla	Human	Female	28	Ambulatory	Oropharyngeal swab
hCoV-19/Mexico/PUE_INER_IMSS_00962/2021	EPI_ISL_2091403	13/04/21	North America / Mexico / Puebla	Human	Female	26	Ambulatory	Oropharyngeal swab
hCoV-19/Mexico/MEX_INER_IMSS_00964/2021	EPI_ISL_2091405	15/04/21	North America / Mexico / State of Mexico	Human	Male	34	Ambulatory	Nasopharyngeal and oropharyngeal swab
hCoV-19/Mexico/VER_INER_IMSS_00965/2021	EPI_ISL_2091406	14/04/21	North America / Mexico / Veracruz	Human	Female	37	Ambulatory	Nasopharyngeal and oropharyngeal swab
hCoV-19/Mexico/CMX_INER_IMSS_00967/2021	EPI_ISL_2091408	16/04/21	North America / Mexico / Mexico City	Human	Male	58	Ambulatory	Nasopharyngeal swab
hCoV-19/Mexico/MEX_INER_IMSS_00968/2021	EPI_ISL_2091409	16/04/21	North America / Mexico / State of Mexico	Human	Male	55	Ambulatory	Nasopharyngeal swab
hCoV-19/Mexico/MEX_INER_IMSS_00971/2021	EPI_ISL_2091411	14/04/21	North America / Mexico / State of Mexico	Human	Female	31	Ambulatory	Nasopharyngeal and oropharyngeal swab
hCoV-19/Mexico/CMX_INER_IMSS_00972/2021	EPI_ISL_2091412	15/04/21	North America / Mexico / Mexico City	Human	Male	60	Ambulatory	Nasopharyngeal and oropharyngeal swab
hCoV-19/Mexico/CMX_INER_IMSS_00973/2021	EPI_ISL_2091413	04/04/21	North America / Mexico / Mexico City	Human	Female	74	Hospitalized	Nasopharyngeal and oropharyngeal swab
hCoV-19/Mexico/HID_INER_IMSS_00974/2021	EPI_ISL_2091414	14/04/21	North America / Mexico / Hidalgo	Human	Male	39	Ambulatory	Nasopharyngeal and oropharyngeal swab
hCoV-19/Mexico/CMX_INER_IMSS_00975/2021	EPI_ISL_2091415	16/04/21	North America / Mexico / Mexico City	Human	Male	2	Ambulatory	Nasopharyngeal and oropharyngeal swab
hCoV-19/Mexico/CMX_INER_IMSS_00976/2021	EPI_ISL_2091416	15/04/21	North America / Mexico / Mexico City	Human	Male	52	Hospitalized	Oropharyngeal swab
hCoV-19/Mexico/CMX_INER_IMSS_00977/2021	EPI_ISL_2091417	16/04/21	North America / Mexico / Mexico City	Human	Male	60	Ambulatory	Oropharyngeal swab
hCoV-19/Mexico/CMX_INER_IMSS_00978/2021	EPI_ISL_2091418	16/04/21	North America / Mexico / Mexico City	Human	Male	71	Ambulatory	Oropharyngeal swab
hCoV-19/Mexico/CMX_INER_IMSS_00981/2021	EPI_ISL_2091421	15/04/21	North America / Mexico / Mexico City	Human	Male	51	Hospitalized	Nasopharyngeal and oropharyngeal swab

hCoV-19/Mexico/MEX_INER_IMSS_00983/2021	EPI_ISL_2091422	15/04/21	North America / Mexico / State of Mexico	Human	Male	83	Hospitalized	Nasopharyngeal and oropharyngeal swab
hCoV-19/Mexico/MEX_INER_IMSS_00985/2021	EPI_ISL_2091424	16/04/21	North America / Mexico / State of Mexico	Human	Male	46	Ambulatory	Nasopharyngeal and oropharyngeal swab
hCoV-19/Mexico/PUE_INER_IMSS_00986/2021	EPI_ISL_2091425	11/04/21	North America / Mexico / Puebla	Human	Male	29	Ambulatory	Nasopharyngeal and oropharyngeal swab
hCoV-19/Mexico/OAX_INER_IMSS_00987/2021	EPI_ISL_2091426	15/04/21	North America / Mexico / Oaxaca	Human	Male	67	Hospitalized	Nasopharyngeal and oropharyngeal swab
hCoV-19/Mexico/GRO_INER_IMSS_00988/2021	EPI_ISL_2091427	14/04/21	North America / Mexico / Guerrero	Human	Male	51	Hospitalized	Oropharyngeal swab
hCoV-19/Mexico/MEX_INER_IMSS_00989/2021	EPI_ISL_2091428	12/04/21	North America / Mexico / State of Mexico	Human	Female	59	Hospitalized	Nasopharyngeal and oropharyngeal swab
hCoV-19/Mexico/MEX_INER_IMSS_00990/2021	EPI_ISL_2091429	10/04/21	North America / Mexico / State of Mexico	Human	Female	49	Hospitalized	Nasopharyngeal and oropharyngeal swab
hCoV-19/Mexico/CMX_INER_IMSS_00991/2021	EPI_ISL_2091430	16/04/21	North America / Mexico / Mexico City	Human	Male	62	Hospitalized	Oropharyngeal swab
hCoV-19/Mexico/CMX_INER_IMSS_00992/2021	EPI_ISL_2091431	15/04/21	North America / Mexico / Mexico City	Human	Female	30	Ambulatory	Nasopharyngeal and oropharyngeal swab
hCoV-19/Mexico/OAX_INER_IMSS_00994/2021	EPI_ISL_2091433	14/04/21	North America / Mexico / Oaxaca	Human	Female	26	Ambulatory	Nasopharyngeal and oropharyngeal swab
hCoV-19/Mexico/CMX_INER_IMSS_00996/2021	EPI_ISL_2091435	17/04/21	North America / Mexico / Mexico City	Human	Female	87	Hospitalized	Nasopharyngeal swab
hCoV-19/Mexico/CMX_INER_IMSS_00997/2021	EPI_ISL_2091436	16/04/21	North America / Mexico / Mexico City	Human	Male	59	Hospitalized	Nasopharyngeal swab
hCoV-19/Mexico/MEX_INER_IMSS_00998/2021	EPI_ISL_2091437	17/04/21	North America / Mexico / State of Mexico	Human	Male	54	Hospitalized	Oropharyngeal swab
hCoV-19/Mexico/CMX_INER_IMSS_00999/2021	EPI_ISL_2091438	18/04/21	North America / Mexico / Mexico City	Human	Female	50	Hospitalized	Nasopharyngeal and oropharyngeal swab
hCoV-19/Mexico/CMX_INER_IMSS_01000/2021	EPI_ISL_2091439	17/04/21	North America / Mexico / Mexico City	Human	Male	48	Ambulatory	Oropharyngeal swab
hCoV-19/Mexico/CMX_INER_IMSS_01001/2021	EPI_ISL_2091440	18/04/21	North America / Mexico / Mexico City	Human	Female	67	Hospitalized	Nasopharyngeal swab
hCoV-19/Mexico/MEX_INER_IMSS_01002/2021	EPI_ISL_2091441	27/03/21	North America / Mexico / State of Mexico	Human	Male	49	Hospitalized	Nasopharyngeal and oropharyngeal swab
hCoV-19/Mexico/CMX_INER_IMSS_01003/2021	EPI_ISL_2091442	16/04/21	North America / Mexico / Mexico City	Human	Male	59	Hospitalized	Nasopharyngeal and oropharyngeal swab
hCoV-19/Mexico/MEX_INER_IMSS_01004/2021	EPI_ISL_2091443	17/04/21	North America / Mexico / State of Mexico	Human	Male	95	Hospitalized	Nasopharyngeal and oropharyngeal swab
hCoV-19/Mexico/CMX_INER_IMSS_01005/2021	EPI_ISL_2091444	17/04/21	North America / Mexico / Mexico City	Human	Female	41	Ambulatory	Nasopharyngeal and oropharyngeal swab
hCoV-19/Mexico/MEX_INER_IMSS_01006/2021	EPI_ISL_2091445	16/04/21	North America / Mexico / State of Mexico	Human	Female	39	Hospitalized	Nasopharyngeal and oropharyngeal swab
hCoV-19/Mexico/MEX_INER_IMSS_01007/2021	EPI_ISL_2091446	18/04/21	North America / Mexico / State of Mexico	Human	Female	55	Hospitalized	Nasopharyngeal and oropharyngeal swab
hCoV-19/Mexico/VER_INER_IMSS_01008/2021	EPI_ISL_2091447	15/04/21	North America / Mexico / Veracruz	Human	Male	30	Ambulatory	Nasopharyngeal and oropharyngeal swab
hCoV-19/Mexico/VER_INER_IMSS_01009/2021	EPI_ISL_2091448	15/04/21	North America / Mexico / Veracruz	Human	Female	55	Ambulatory	Nasopharyngeal and oropharyngeal swab
hCoV-19/Mexico/CMX_INER_IMSS_01011/2021	EPI_ISL_2091450	16/04/21	North America / Mexico / Mexico City	Human	Male	56	Ambulatory	Nasopharyngeal and oropharyngeal swab
hCoV-19/Mexico/CMX_INER_IMSS_01012/2021	EPI_ISL_2091451	16/04/21	North America / Mexico / Mexico City	Human	Female	66	Ambulatory	Nasopharyngeal and oropharyngeal swab
hCoV-19/Mexico/CMX_INER_IMSS_01013/2021	EPI_ISL_2091452	16/04/21	North America / Mexico / Mexico City	Human	Female	37	Ambulatory	Nasopharyngeal swab
hCoV-19/Mexico/MEX_INER_IMSS_01014/2021	EPI_ISL_2091453	17/04/21	North America / Mexico / State of Mexico	Human	Male	28	Hospitalized	Nasopharyngeal and oropharyngeal swab
hCoV-19/Mexico/CMX_INER_IMSS_01015/2021	EPI_ISL_2091454	16/04/21	North America / Mexico / Mexico City	Human	Female	44	Ambulatory	Nasopharyngeal and oropharyngeal swab
hCoV-19/Mexico/CMX_INER_IMSS_01016/2021	EPI_ISL_2091455	19/04/21	North America / Mexico / Mexico City	Human	Female	72	Ambulatory	Oropharyngeal swab
hCoV-19/Mexico/CMX_INER_IMSS_01017/2021	EPI_ISL_2091456	18/04/21	North America / Mexico / Mexico City	Human	Male	61	Hospitalized	Nasopharyngeal and oropharyngeal swab
hCoV-19/Mexico/NLE-LESPNL-20210512-402/2021	EPI_ISL_2102600	28/04/21	North America / Mexico / Nuevo Leon	Human	Female	65	ent - Home Mo	ropharyngeal and/or nasopharyngeal exudat
hCoV-19/Mexico/NLE-LESPNL-20210512-416/2021	EPI_ISL_2102611	07/05/21	North America / Mexico / Nuevo Leon	Human	Female	56	zed - Non-Seri	ropharyngeal and/or nasopharyngeal exudat
hCoV-19/Mexico/NLE-LESPNL-20210512-426/2021	EPI_ISL_2102621	04/05/21	North America / Mexico / Nuevo Leon	Human	Female	22	ent - Home Mo	ropharyngeal and/or nasopharyngeal exudat
hCoV-19/Mexico/NLE-LESPNL-20210512-434/2021	EPI_ISL_2102629	06/05/21	North America / Mexico / Nuevo Leon	Human	Male	27	ent - Home Mo	ropharyngeal and/or nasopharyngeal exudat
hCoV-19/Mexico/NLE-LESPNL-20210512-457/2021	EPI_ISL_2102649	03/05/21	North America / Mexico / Nuevo Leon	Human	Female	55	ent - Home Mo	ropharyngeal and/or nasopharyngeal exudat
hCoV-19/Mexico/NLE-LESPNL-20210512-471/2021	EPI_ISL_2102660	29/04/21	North America / Mexico / Nuevo Leon	Human	Female	47	tory - Under Tr	ropharyngeal and/or nasopharyngeal exudat
hCoV-19/Mexico/NLE-LESPNL-20210512-491/2021	EPI_ISL_2102677	30/04/21	North America / Mexico / Nuevo Leon	Human	Female	64	ent - Home Mo	ropharyngeal and/or nasopharyngeal exudat
hCoV-19/Mexico/CMX-INMEGEN-05-01-189/2021	EPI_ISL_2105721	06/05/21	North America / Mexico / Mexico City	Human	Male	58	unknown	Oropharyngeal swab
hCoV-19/Mexico/CMX-INMEGEN-05-01-191/2021	EPI_ISL_2105722	06/05/21	North America / Mexico / Mexico City	Human	Male	59	unknown	Oropharyngeal swab
hCoV-19/Mexico/CMX-INMEGEN-05-01-204/2021	EPI_ISL_2105725	31/03/21	North America / Mexico / Mexico City	Human	Male	29	unknown	Oropharyngeal swab
hCoV-19/Mexico/CMX-INMEGEN-05-01-205/2021	EPI_ISL_2105726	06/04/21	North America / Mexico / Mexico City	Human	Female	29	unknown	Oropharyngeal swab
hCoV-19/Mexico/CMX-INMEGEN-05-01-207/2021	EPI_ISL_2105728	05/04/21	North America / Mexico / Mexico City	Human	Male	29	unknown	Oropharyngeal swab
hCoV-19/Mexico/CMX-INMEGEN-05-01-208/2021	EPI_ISL_2105729	17/04/21	North America / Mexico / Mexico City	Human	Female	29	unknown	Oropharyngeal swab

hCoV-19/Mexico/CMX-INMEGEN-05-01-209/2021	EPI_ISL_2105730	16/03/21	North America / Mexico / Mexico City	Human	Male	60	unknown	Oropharyngeal swab
hCoV-19/Mexico/CMX-INMEGEN-05-01-212/2021	EPI_ISL_2105732	05/04/21	North America / Mexico / Mexico City	Human	Male	36	unknown	Oropharyngeal swab
hCoV-19/Mexico/CMX-INMEGEN-05-01-214/2021	EPI_ISL_2105734	18/03/21	North America / Mexico / Mexico City	Human	Female	74	unknown	Oropharyngeal swab
hCoV-19/Mexico/CMX-INMEGEN-05-01-216/2021	EPI_ISL_2105736	09/04/21	North America / Mexico / Mexico City	Human	Female	28	unknown	Oropharyngeal swab
hCoV-19/Mexico/CMX-INMEGEN-05-01-217/2021	EPI_ISL_2105737	07/04/21	North America / Mexico / Mexico City	Human	Female	88	unknown	Oropharyngeal swab
[truncated: 871,947 more chars]
